# Supplementary material for: Thyroid Hormone Receptor α Controls the Hind Limb Metamorphosis by Regulating Cell Proliferation and Wnt Signaling Pathways in Xenopus tropicalis
Source: Int J Mol Sci. 2022 Jan 22;23(3):1223. doi: 10.3390/ijms23031223 (PMC8835992; doi:10.3390/ijms23031223)
Supplement: Supplementary file 1 [file ijms-23-01223-s001.zip › ijms-1537650-supplementary.pdf]

Figure S1

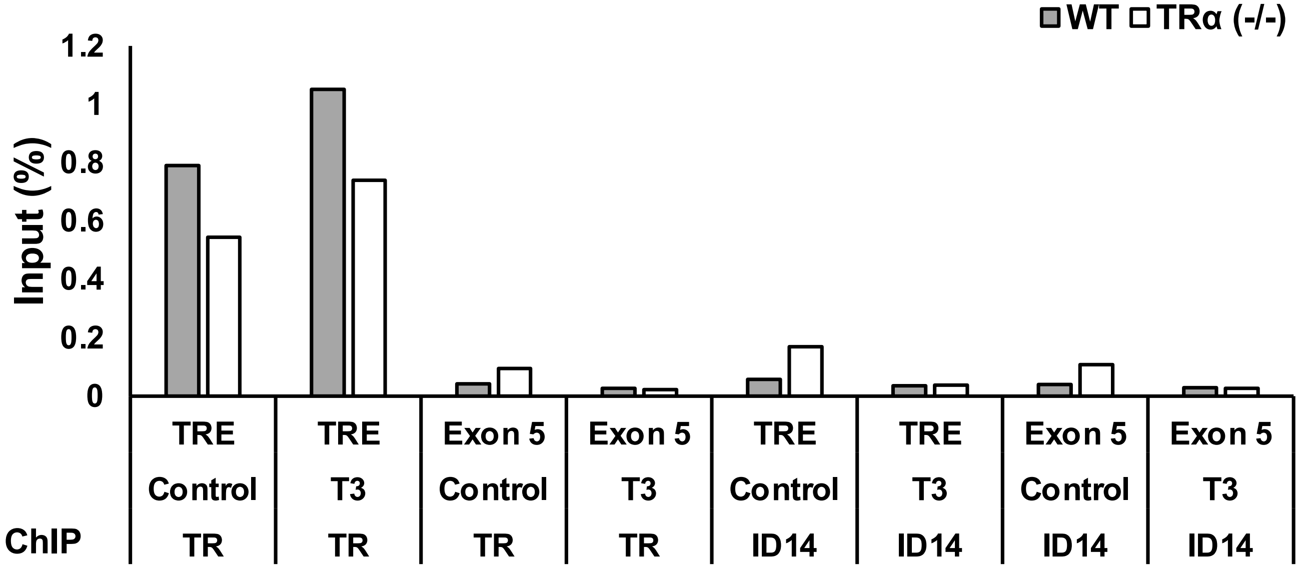

Figure S1. Validation of the ChIP samples by analyzing TR binding to TRβ TRE in the hindlimb of wild type (WT) and TRα (-/-) tadpoles without (Control) or with T3 (T3) treatment. The ChIP samples obtained with anti-TR or ID14 antibody was analyzed by qPCR for the presence of the region containing the TRβ TRE (TRE) or TRβ exon 5 (Exon 5), the negative control region lacking a TRE. Note that as expected, only the TRβ TRE region had positive ChIP signals with anti-TR antibody regardless of T3 treatment, while all the other samples had only background ChIP signals. TRα knockout reduced TR binding to the TRβ TRE region.

Figure S2

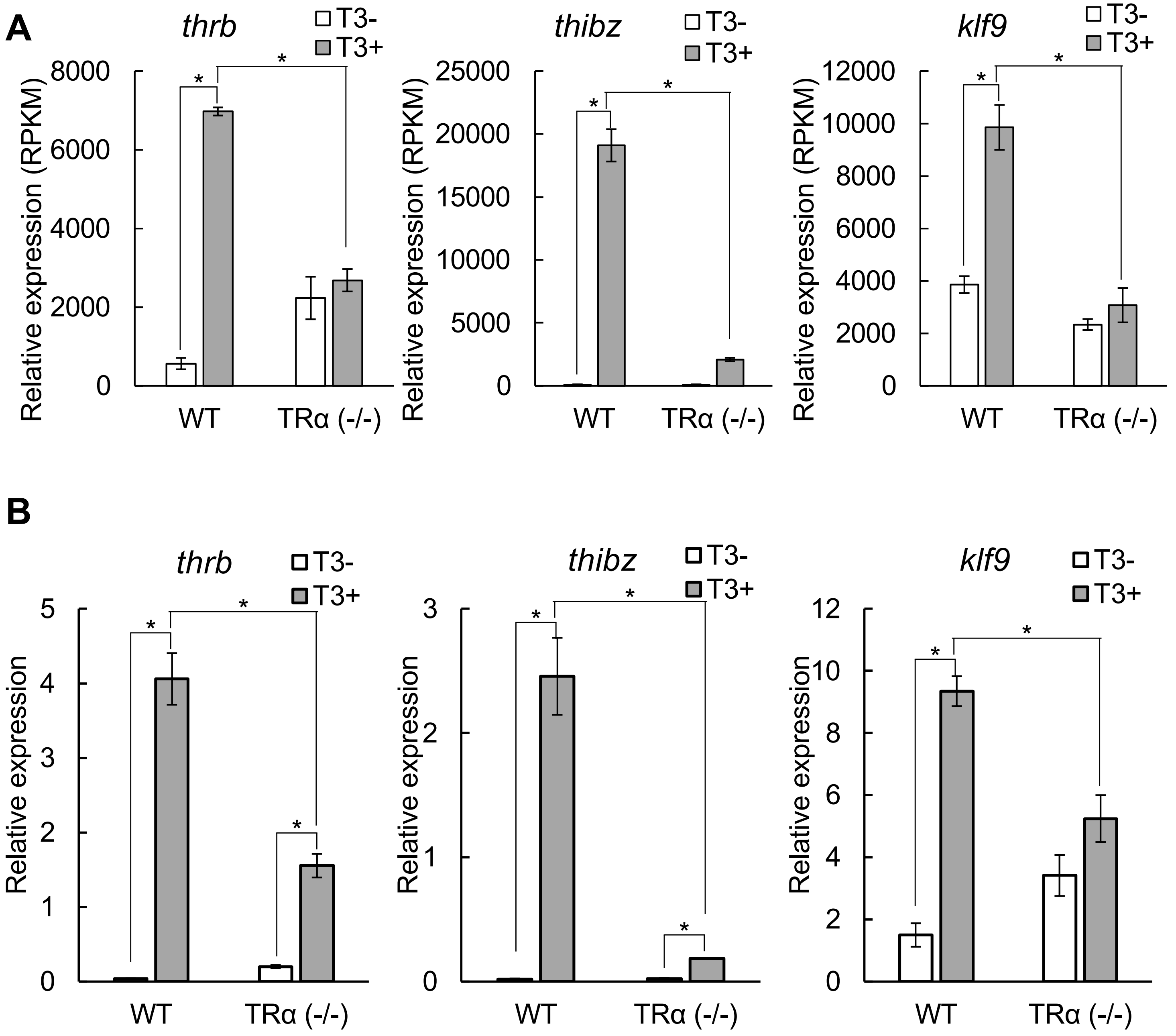

Figure S2. Independent confirmation of RNA-Seq data by qRT-PCR for selected genes in wild type (WT) and TRα (-/-) hindlimb.

(A) Three known T3 responsible genes (thrb, thibz and klf9) are upregulated by T3 in WT hindlimb, and this upregulation is reduced in TRα (-/-) hindlimb. Hindlimbs were harvested from stage-matched *X. tropicalis* tadpoles at stage 54 treated with (T3+) or without (T3-) 10 nM T3 for 18 hours and used for RNA-Seq analyses. The expression was normalized to Reads Per Kilobase of exon model per Million mapped reads (RPKM). Each bar represents the mean plus S.D. and the asterisk (*) indicates a significant difference between T3- and T3+ samples, or WT and TRα (-/-) samples ($P<0.05$).

(B) Relative gene expression of thrb, thibz and klf9 analyzed by qRT-PCR confirms the regulation as found by RNA-Seq analysis. Independent tadpole treatment and RNA isolation were performed as in (A), and qRT-PCR was performed to determine the expression of the genes. Each bar represents the mean plus S.E. and the asterisk (*) indicates a significant difference between the T3- and T3+ samples, or WT and TRα (-/-) samples ($P<0.05$).

Table S1. ChIP-Seq data of wild type hindlimb without T3 treatment (*NA under Gene symbol indicates a peak that could not be assigned to any gene).

Chromosome	Sart position	End position	Absolute peak summit position	Pileup	X.log10.pvalues	Fold enrichments	log10.qval	Sample count	Gene symbol	Distance
GL172637.1	56	913	338, 406, 644	9, 15, 8	9.24298, 15.80067, 10.1465	6.3792, 9.24574, 6.82176	6.53474, 1	3	U5	0
GL172637.1	31319	32091	31850, 31623, 31635	11, 16, 14	9.61965, 12.05974, 15.20637	6.33449, 6.7328, 9.10193	6.89006, 9	3	tmem53	6041
GL172637.1	2356337	2356778	2356570, 2356623, 2356642	9, 20, 6	9.72082, 22.6463, 7.29403	6.64182, 12.00208, 5.30581	6.97155, 1	3	mknk1	145869
GL172637.1	2781591	2782253	2782042, 2782099, 2782135	158, 184, 159	160.07596, 184.22279, 179.93887	22.3486, 22.33224, 28.33628	155.29388	3	NA	10492
GL172637.1	6417217	6417776	6417554, 6417356, 6417553	9, 13, 16	9.72082, 13.14514, 22.37427	6.64182, 8.09002, 12.56044	6.97155, 1	3	acot11	5618
GL172637.1	6915865	6916309	6916067, 6916164, 6916036	19, 26, 8	19.56679, 23.91458, 7.46678	10.46249, 10.80262, 5.43686	16.35231,	3	mrps14	15892
GL172637.1	7193673	7194781	7193970, 7194102, 7194171	8, 12, 7	7.15567, 9.13354, 8.49722	5.25151, 5.83804, 5.97582	4.61046, 6	3	tnr	0
GL172637.1	7587137	7587515	7587328, 7587174, 7587315	7, 9, 9	5.75798, 6.46255, 10.21256	4.48785, 4.68809, 6.90044	3.36626, 4	3	astn1	0
GL172638.1	40	593	254, 307, 251	22, 35, 21	24.27679, 33.66325, 23.4003	12.36565, 13.1486, 12.15468	20.91531,	3	NA	2706
GL172638.1	1945508	1946754	1945916, 1945811, 1945917	18, 24, 17	17.12119, 20.32059, 19.46435	9.27196, 9.36407, 10.92232	13.99654,	3	ankar	0
GL172638.1	2641420	2642195	2641749, 2641832, 2641838	13, 18, 7	13.20994, 17.25928, 8.69489	8.12823, 9.35577, 6.06379	10.26285,	3	gulp1	79610
GL172638.1	2654873	2655826	2655290, 2655175, 2655317	22, 22, 12	18.6395, 16.7916, 11.81848	8.99638, 7.93158, 7.4869	15.45774,	3	gulp1	65979
GL172638.1	2767006	2767960	2767778, 2767785, 2767778	8, 15, 4	8.42257, 14.03607, 4.26291	5.97763, 8.18766, 3.59641	5.76336, 1	3	gulp1	1787
GL172638.1	3304102	3304435	3304125, 3304135, 3304156	9, 17, 6	9.63662, 16.43386, 7.29403	6.59633, 9.1315, 5.30581	6.90556, 1	3	pacrg	141783
GL172638.1	4496575	4496889	4496666, 4496605, 4496605	10, 10, 13	9.83337, 8.20397, 16.0859	6.61776, 5.62902, 9.70995	7.07841, 5	3	serac1	6677
GL172638.1	4661998	4662428	4662161, 4662258, 4662271	8, 17, 5	7.67464, 14.51225, 5.59755	5.55832, 7.96159, 4.38538	5.08299, 1	3	synj2	0
GL172638.1	6313801	6314864	6314362, 6314198, 6314489	8, 9, 7	8.10827, 7.17446, 7.10639	5.80497, 5.1397, 5.27894	5.48761, 4	3	c6orf120	23787
GL172638.1	6540788	6541453	6540906, 6541212, 6541226	51, 84, 30	44.98414, 73.57249, 30.04994	13.28099, 14.99497, 12.96132	41.20549,	3	fam120b	17676
GL172638.1	7245436	7246160	7245639, 7245889, 7245805	7, 6, 4	7.1696, 4.93312, 4.67325	5.31345, 4.04501, 3.78987	4.61046, 2	3	NA	3048
GL172638.1	7493055	7493755	7493466, 7493434, 7493449	12, 11, 9	13.8483, 10.60895, 11.21577	8.63436, 6.9343, 7.38849	10.86694,	3	shprh	0
GL172639.1	18761	19503	19034, 19252, 19208	9, 17, 11	7.71701, 15.93163, 14.45778	5.47767, 8.82633, 8.96373	5.12244, 1	3	plekhf2	128127
GL172639.1	585573	586272	585986, 586074, 585996	21, 24, 17	16.06857, 20.06483, 14.73879	7.79262, 9.21701, 8.09947	12.99028,	3	ywhaz	56495
GL172639.1	1216051	1218423	1217851, 1217105, 1216699	21, 15, 16	16.23632, 11.4891, 15.26551	7.88875, 6.62745, 8.68554	13.15022,	3	azin1	10962
GL172639.1	1278884	1279381	1279213, 1278970, 1279257	9, 16, 7	6.36774, 11.44899, 6.71158	4.62751, 6.36757, 5.05776	3.8942, 8.7	3	azin1	33895
GL172639.1	1283206	1284323	1283694, 1283722, 1283750	35, 49, 19	37.25596, 47.9187, 21.69099	15.20664, 15.42938, 11.71707	33.59962,	3	azin1	38217
GL172639.1	2121413	2121978	2121774, 2121594, 2121580	8, 9, 8	6.8227, 6.18102, 8.35103	5.04883, 4.50804, 5.93881	4.31393, 3	3	lrp12	154199
GL172639.1	2143460	2143953	2143915, 2143901, 2143899	9, 24, 11	8.35434, 25.29108, 12.48556	5.86444, 12.31022, 7.99611	5.71584, 2	3	lrp12	176246
GL172639.1	2153805	2154262	2154215, 2154227, 2154210	5, 10, 9	4.655, 8.4053, 10.49552	3.89157, 5.75445, 7.0436	2.42652, 5	3	lrp12	186591
GL172639.1	2286144	2286485	2286440, 2286439, 2286386	7, 15, 13	7.1696, 15.26186, 16.0859	5.31345, 8.92711, 9.70995	4.61046, 1	3	zfpm2	83143
GL172639.1	2517683	2518278	2517863, 2517770, 2517960	14, 12, 10	14.23954, 9.66318, 10.1418	8.53829, 6.16843, 6.79622	11.24094,	3	OXR1	85890
GL172639.1	3383998	3384491	3384458, 3384438, 3384442	16, 25, 12	14.56946, 22.90359, 15.26371	8.26129, 10.54631, 9.35067	11.55649,	3	NA	0
GL172639.1	4488375	4489290	4488792, 4488632, 4488905	7, 13, 6	6.69449, 12.67661, 6.76586	5.04797, 7.81122, 5.06179	4.1953, 9.9	3	slc2a2	1327
GL172639.1	5324061	5324552	5324290, 5324389, 5324340	6, 9, 5	5.96715, 8.21099, 5.9506	4.64927, 5.77858, 4.54784	3.52158, 5	3	NA	71187
GL172639.1	5983925	5984510	5984278, 5984138, 5984206	7, 15, 6	6.69449, 13.31811, 5.95321	5.04797, 7.74852, 4.64162	4.1953, 10	3	NLGN1	127246
GL172639.1	6725056	6726081	6725642, 6725667, 6725513	6, 7, 7	5.16999, 4.53822, 8.35502	4.18963, 3.70607, 5.9108	2.83325, 2	3	tbl1xr1	153185
GL172639.1	7507510	7508458	7507871, 7508076, 7507845	9, 7, 4	8.35434, 5.97725, 4.67325	5.86444, 4.62287, 3.78987	5.71584, 3	3	NA	9256
GL172640.1	1409188	1411118	1410026, 1410553, 1410530	18, 35, 19	14.82855, 29.57249, 18.90983	7.88255, 10.93664, 10.06679	11.80513,	3	NA	337880
GL172640.1	2155458	2156049	2155716, 2155695, 2155705	10, 8, 5	7.78798, 6.17826, 4.64121	5.36798, 4.64607, 3.88372	5.18872, 3	3	lrrc19	0
GL172640.1	4789476	4790279	4789583, 4789731, 4790106	9, 11, 5	9.24298, 10.00475, 5.9506	6.3792, 6.57041, 4.54784	6.53474, 7	3	PATJ	0
GL172641.1	887301	887979	887637, 887703, 887917	10, 7, 5	9.49535, 5.02593, 5.9506	6.41851, 4.02367, 4.54784	6.77344, 2	3	slc25a3	4184
GL172641.1	2275022	2275385	2275261, 2275106, 2275079	8, 14, 6	6.63031, 9.82448, 6.08001	4.92991, 5.84215, 4.71063	4.13604, 7	3	ccdc59	64307
GL172641.1	3368463	3369363	3368772, 3368697, 3368597, 3368991	14, 11, 4, 5	16.7649, 9.26343, 4.67325, 5.9506	9.96273, 6.11409, 3.78987, 4.54784	13.6543, 6	3	cstf3	70687
GL172641.1	5378510	5380243	5379097, 5379929, 5379914	7, 8, 8	5.39768, 5.43464, 7.63508	4.26169, 4.16933, 5.53536	3.04019, 3	3	NA	8140
GL172641.1	5378854	5380243	5379442, 5379929, 5379914	6, 8, 8	4.48574, 5.43464, 7.63508	3.76346, 4.16933, 5.53536	2.28228, 3	3	NA	8140
GL172641.1	5610007	5610341	5610182, 5610061, 5610122	15, 20, 9	16.11735, 20.00323, 11.64359	9.43066, 10.42807, 7.57973	13.03694,	3	NA	123553
GL172641.1	6280346	6280657	6280401, 6280356, 6280384	13, 21, 9	13.27928, 20.32736, 10.49552	8.16901, 10.3138, 7.0436	10.32936,	3	ube3a	26090
GL172642.1	2220645	2221609	2220920, 2221188, 2220849	18, 26, 10	16.5065, 22.78014, 9.93124	8.89851, 10.14557, 6.67475	13.41011,	3	c1orf52	36653
GL172642.1	2231045	2232238	2231441, 2231688, 2231411	15, 16, 14	13.57755, 13.03911, 13.07989	7.90752, 7.32604, 7.8407	10.61316,	3	c1orf52	26024
GL172642.1	2534473	2535154	2534763, 2534566, 2534555	5, 9, 3	4.69353, 6.52169, 3.17999	3.9134, 4.72584, 2.89245	2.45909, 4	3	col24a1	0
GL172642.1	2867387	2868543	2868056, 2867740, 2867919	145, 141, 100	127.98701, 120.1502, 94.79211	16.78754, 15.65988, 17.95754	123.38561	3	lcat	1657
GL172642.1	4249220	4249609	4249315, 4249511, 4249306	4, 6, 4	3.57366, 4.93312, 4.38152	3.22499, 4.04501, 3.65448	1.59706, 2	3	mtss1l	0
GL172642.1	4716458	4717121	4716697, 4716680, 4716627	85, 93, 56	91.77859, 94.69772, 63.26543	22.01306, 20.19433, 20.59785	87.46411,	3	fa2h	14578
GL172642.1	5322124	5322505	5322420, 5322183, 5322426	8, 10, 5	6.34412, 6.60892, 5.59755	4.75087, 4.62161, 4.38538	3.871, 4.24	3	siah1	2646
GL172642.1	6047475	6047962	6047768, 6047782, 6047704	23, 27, 13	26.23988, 26.2886, 14.46982	13.20864, 11.84892, 8.85107	22.8232, 2	3	neto2	0
GL172643.1	252791	253183	252913, 252864, 252959	5, 7, 4	4.77263, 5.97725, 4.64325	3.9578, 4.62287, 3.77643	2.51418, 3	3	xbp1	19876
GL172643.1	344417	345105	344584, 344902, 344607	8, 9, 5	6.34412, 5.65581, 4.17451	4.75087, 4.1716, 3.60852	3.871, 3.38	3	tcn2	11763
GL172643.1	964479	965453	965271, 965115, 965063	11, 12, 4	10.84329, 9.82596, 4.08034	7.073, 6.26981, 3.50363	8.0324, 7.2	3	CHSY3	11043
GL172643.1	1073955	1074455	1074177, 1074334, 1074401	6, 15, 5	5.20598, 13.97777, 5.45388	4.2113, 8.15212, 4.3157	2.86702, 1	3	CHSY3	0
GL172643.1	2588697	2589016	2588854, 2588854, 2588854	14, 9, 11	15.15267, 8.21099, 13.1143	9.07115, 5.77858, 8.32282	12.11465,	3	znf608	103774
GL172643.1	3102233	3102713	3102266, 3102314, 3102286	14, 15, 6	14.16942, 14.7819, 6.5517	8.49669, 8.63964, 4.95623	11.17387,	3	cep120	9599
GL172643.1	3673816	3674119	3673976, 3673983, 3674081	16, 14, 4	16.83862, 12.49074, 4.59709	9.62959, 7.48027, 3.75554	13.72526,	3	lox	0
GL172643.1	3738386	3739050	3738742, 3738751, 3738810	9, 18, 8	8.77314, 17.32425, 9.05167	6.111, 9.39518, 6.30653	6.09355, 1	3	srfbp1	15076
GL172643.1	4642293	4643447	4642896, 4642835, 4642624, 4643306	16, 28, 8, 5	16.60441, 29.84854, 9.1831, 5.23257	9.49088, 13.59546, 6.37229, 4.20435	13.50404,	3	dtwd2	70988
GL172643.1	5013807	5014766	5013862, 5014256, 5014242	10, 13, 7	10.6018, 12.09006, 7.90186	7.05583, 7.45678, 5.69394	7.80484, 9	3	sema6a	172761
GL172643.1	5084304	5084638	5084319, 5084434, 5084331	11, 17, 8	9.71398, 15.46309, 8.04677	6.39253, 8.5409, 5.77057	6.97155, 1	3	sema6a	102889
GL172643.1	5832303	5833957	5833082, 5833551, 5833120	12, 13, 9	10.3005, 10.11663, 7.86759	6.56436, 6.23937, 5.57018	7.51999, 7	3	fem1c	39898
GL172643.1	5835700	5836365	5836034, 5835775, 5836188	14, 14, 9	14.38232, 12.54273, 8.69919	8.62271, 7.51218, 6.06795	11.37721,	3	fem1c	43295
GL172644.1	1149	2082	2053, 2049, 2053	107, 124, 79	139.88599, 158.38229, 109.63388	34.6645, 34.6558, 34.40181	135.21654	3	NA	7469
GL172644.1	63767	64858	64171, 64657, 64517	28, 32, 18	29.46427, 31.45735, 18.14836	13.36659, 13.02561, 9.89347	25.96977,	3	plcb4	362
GL172644.1	265405	266432	266246, 265930, 265883	41, 48, 31	31.56674, 34.43076, 24.90991	10.12641, 9.58002, 9.74797	28.02397,	3	NA	0
GL172644.1	277739	278126	277910, 277892, 277925	8, 14, 5	8.42257, 13.63145, 5.9506	5.97763, 8.17488, 4.54784	5.76336, 1	3	NA	11744
GL172644.1	302397	303250	302643, 302666, 302667	341, 446, 207	300.91656, 383.85464, 186.23293	18.32382, 17.70586, 18.22608	295.64087	3	lamp5	0
GL172644.1	315622	316581	316093, 316085, 316109	25, 19, 6	26.95645, 14.1866, 5.14244	12.95989, 7.23722, 4.17298	23.5223, 1	3	lamp5	2382
GL172644.1	316990	317651	317384, 317401, 317160	12, 25, 11	10.20921, 21.46009, 11.08949	6.50784, 9.70765, 7.21705	7.4341, 18	3	pak5	3404
GL172644.1	592210	592649	592238, 592231, 592243	19, 18, 11	20.83882, 19.24809, 12.48556	11.21961, 10.55079, 7.99611	17.58277,	3	slx4ip	0
GL172644.1	796011	796690	796294, 796294, 796294	45, 41, 24	50.7744, 41.97565, 28.20642	18.6841, 15.4361, 14.04062	46.91096,	3	jag1	88543
GL172644.1	841607	841982	841826, 841821, 841813	17, 19, 6	17.48974, 18.82616, 7.12188	9.76779, 10.01625, 5.22884	14.35103,	3	jag1	134139
GL172644.1	886685	886963	886938, 886894, 886830	21, 21, 5	21.17819, 19.73353, 5.40789	10.82682, 9.95593, 4.29296	17.91116,	3	jag1	179217
GL172644.1	1234681	1236076	1235636, 1235651, 1235559	15, 19, 10	16.03559, 19.04123, 11.03772	9.3831, 10.14611, 7.29398	12.95858,	3	btbd3	32467
GL172644.1	1315696	1316480	1315894, 1315930, 1315891	18, 17, 7	16.44776, 12.234, 6.2897	8.86281, 6.59482, 4.81137	13.35395,	3	NA	1159
GL172644.1	1619555	1621539	1620733, 1620702, 1620009, 1620879	18, 22, 11, 10	14.82855, 14.8489, 9.8855, 8.39789	7.88255, 6.86296, 6.49765, 5.74985	11.80513,	3	ism1	13797
GL172644.1	1817354	1819869	1818166, 1818409, 1818133	34, 40, 22	29.51673, 31.193, 21.14647	11.23387, 10.23131, 10.48833	26.02047,	3	esf1	3363
GL172644.1	1857593	1858228	1858041, 1858040, 1858041	32, 27, 26	34.68701, 28.00888, 32.40004	14.91466, 12.86965, 15.81804	31.08035,	3	ndufaf5	0
GL172644.1	2149541	2150180	2149958, 2150042, 2149944	5, 9, 3	4.43696, 7.67202, 3.47371	3.76555, 5.44992, 3.03189	2.24017, 5	3	flrt3	66270
GL172644.1	2580066	2580300	2580143, 2580094, 2580109	4, 13, 5	3.39747, 12.09006, 5.9506	3.12111, 7.45678, 4.54784	1.46102, 9	3	NA	183445
GL172644.1	2882511	2882997	2882801, 2882797, 2882775	18, 11, 8	21.52103, 9.17879, 8.6841	11.86022, 6.06147, 6.11715	18.2418, 6	3	7SK	252481
GL172644.1	2987517	2987981	2987721, 2987797, 2987788	12, 17, 14	11.55224, 15.24016, 15.42335	7.3281, 8.40499, 9.2256	8.69537, 1	3	7SK	147497
GL172644.1	3798764	3799170	3798981, 3798863, 3798926	7, 8, 8	7.1696, 7.07159, 9.31951	5.31345, 5.20073, 6.43944	4.61046, 4	3	bfsp1	0
GL172644.1	3912967	3913588	3913144, 3913202, 3913328	8, 14, 3	8.42257, 13.50676, 3.47371	5.97763, 8.09967, 3.03189	5.76336, 1	3	prokr2	49952
GL172644.1	4481085	4482068	4481402, 4481388, 4481336	9, 17, 11	8.88537, 15.35073, 12.41242	6.17591, 8.4724, 7.95707	6.19961, 1	3	etaa1	64807
GL172644.1	4501851	4502580	4502340, 4502242, 4502079	19, 27, 11	19.96873, 26.80764, 12.48556	10.70325, 12.15568, 7.99611	16.74065,	3	etaa1	44295
GL172644.1	4696285	4697260	4696305, 4696607, 4697219	5, 8, 13	4.47183, 6.70176, 15.0131	3.78598, 4.97421, 9.14923	2.26935, 4	3	etaa1	139962
GL172644.1	4730227	4730929	4730699, 4730429, 4730727	16, 22, 9	15.60786, 23.62712, 9.52439	8.89312, 11.97865, 6.53516	12.5472, 2	3	meis1	157666
GL172644.1	4777222	4777901	4777863, 4777849, 4777854	51, 66, 14	62.55934, 75.70485, 16.55387	22.62259, 22.57447, 9.85015	58.5434, 7	3	meis1	110694
GL172644.1	4976932	4977433	4977302, 4977223, 4977226	8, 6, 5	6.90289, 4.4843, 5.75061	5.09802, 3.76254, 4.45735	4.38737, 2	3	meis1	0
GL172644.1	5131660	5132258	5131730, 5131738, 5131866	13, 23, 8	11.98086, 21.90191, 8.35103	7.39024, 10.61546, 5.93881	9.10256, 1	3	NA	53909
GL172644.1	5176007	5176439	5176289, 5176343, 5176409	7, 18, 11	7.1696, 17.65791, 12.34038	5.31345, 9.59733, 7.91841	4.61046, 1	3	NA	9728
GL172644.1	5375526	5375957	5375806, 5375901, 5375881	7, 7, 10	7.1696, 5.97725, 12.25126	5.31345, 4.62287, 7.91211	4.61046, 3	3	spred2	53082
GL172644.1	5657243	5658035	5657864, 5657881, 5657865	10, 12, 7	10.53228, 10.2136, 8.69489	7.01712, 6.51056, 6.06379	7.73927, 7	3	slc1a4	7142
GL172645.1	1065248	1065731	1065706, 1065698, 1065719	42, 34, 15	50.35197, 35.5202, 17.94632	19.81943, 14.6006, 10.45626	46.49512,	3	robo1	0
GL172645.1	1265167	1266326	1265545, 1265593, 1265593	22, 41, 20	22.52709, 41.63242, 23.54048	11.31895, 15.24516, 12.51895	19.2175, 3	3	robo1	0
GL172645.1	1281550	1281980	1281745, 1281781, 1281900	6, 9, 7	5.72604, 7.75488, 7.84208	4.51498, 5.501, 5.66426	3.33547, 5	3	robo1	0
GL172645.1	1607961	1608626	1608012, 1608046, 1608036	7, 8, 9	7.04884, 6.59014, 11.39481	5.2473, 4.90493, 7.46977	4.52221, 4	3	U4	92505
GL172645.1	1728545	1729332	1728714, 1729114, 1729195	5, 13, 10	4.73273, 11.66277, 12.52085	3.93547, 7.19557, 8.03986	2.49377, 8	3	gbe1	198174
GL172645.1	1728545	1729332	1729127, 1729114, 1729195	9, 13, 10	9.30591, 11.66277, 12.52085	6.41439, 7.19557, 8.03986	6.59443, 8	3	gbe1	198174
GL172645.1	1858090	1858762	1858493, 1858443, 1858439	9, 8, 4	8.66407, 5.84201, 3.97874	6.04743, 4.43168, 3.45022	5.99101, 3	3	gbe1	68744
GL172645.1	1899026	1899219	1899126, 1899130, 1899115	11, 12, 4	10.66406, 10.58612, 4.67325	6.96705, 6.74063, 3.78987	7.86396, 7	3	gbe1	28287
GL172645.1	2003894	2004535	2004420, 2004352, 2004222	5, 10, 8	4.69353, 9.028, 10.1465	3.9134, 6.13739, 6.82176	2.45909, 6	3	NA	37754
GL172645.1	2055124	2055853	2055254, 2055554, 2055336	11, 11, 11	9.61965, 8.89461, 11.79996	6.33449, 5.88422, 7.62212	6.89006, 6	3	NA	10966
GL172645.1	2078289	2078734	2078680, 2078451, 2078681	10, 14, 4	9.661, 13.8891, 4.38152	6.51661, 8.32957, 3.65448	6.9283, 11	3	NA	34131
GL172645.1	2129290	2129855	2129612, 2129592, 2129805	13, 19, 9	12.68462, 16.62257, 9.52439	7.81602, 8.68263, 6.53516	9.76298, 1	3	NA	85132
GL172645.1	2197572	2198032	2197834, 2197822, 2197840	11, 6, 9	11.71035, 4.379, 11.39481	7.57196, 3.69486, 7.46977	8.8442, 2.2	3	NA	153414
GL172645.1	2786775	2787237	2787168, 2787179, 2787200	10, 7, 6	11.06011, 5.97725, 6.4014	7.306, 4.62287, 4.8799	8.22712, 3	3	NA	182542
GL172645.1	3007516	3008044	3007695, 3007932, 3007697	6, 10, 8	5.96715, 9.32635, 10.00633	4.64927, 6.31756, 6.75998	3.52158, 6	3	cadm2	20955
GL172645.1	3379245	3379590	3379399, 3379405, 3379289	4, 6, 3	3.74195, 4.93312, 3.47371	3.32091, 4.04501, 3.03189	1.6222, 2.7	3	cadm2	138683
GL172645.1	3386216	3387117	3386396, 3386456, 3386515	5, 9, 4	4.82177, 7.63127, 4.34116	3.98509, 5.42473, 3.63492	2.51418, 5	3	cadm2	145654
GL172645.1	3559730	3560440	3560081, 3559986, 3559960	7, 12, 5	6.37715, 11.86108, 5.40789	4.86324, 7.51216, 4.29296	3.90257, 9	3	cfap70	3909
GL172645.1	3571826	3572150	3572124, 3572132, 3572140	7, 12, 11	7.1696, 8.9579, 10.92742	5.31345, 5.72845, 7.12242	4.61046, 6	3	cfap70	4938
GL172645.1	3756866	3757490	3756932, 3756991, 3756912	14, 31, 14	12.23001, 30.27201, 17.2091	7.31997, 12.71642, 10.19524	9.33844, 2	3	pou1f1	56526
GL172645.1	3917778	3918622	3918473, 3918424, 3918428	10, 14, 9	9.95227, 13.26509, 9.64095	6.68696, 7.95332, 6.59867	7.19087, 1	3	htr1f	4581
GL172645.1	3925392	3926162	3925655, 3926051, 3925783	7, 6, 10	6.99564, 4.62266, 12.71002	5.21786, 3.8507, 8.12734	4.47307, 2	3	htr1f	1114
GL172645.1	3977808	3978747	3978295, 3978617, 3978298	20, 34, 12	18.27108, 31.58761, 13.29687	9.38175, 12.3603, 8.33527	15.10173,	3	znf654	524
GL172645.1	4195910	4197138	4196926, 4196385, 4197101	4, 11, 7	3.48339, 9.22091, 7.96278	3.1722, 6.08767, 5.72394	1.52925, 6	3	NA	94957
GL172645.1	4350584	4351461	4350842, 4350842, 4350842	22, 30, 16	25.84654, 30.39874, 20.29858	13.28717, 13.16591, 11.55461	22.44153,	3	epha3	0
GL172645.1	4362405	4362886	4362733, 4362706, 4362764	8, 15, 4	7.1122, 12.6757, 4.46488	5.22529, 7.35409, 3.69425	4.57946, 9	3	epha3	0
GL172645.1	4474967	4475280	4475253, 4475251, 4475252	37, 55, 51	45.74491, 65.24003, 67.97697	19.35625, 22.17864, 25.79128	41.95496,	3	epha3	27573
GL172645.1	4481699	4482183	4481953, 4481787, 4481770	13, 7, 5	13.86771, 5.78791, 5.45388	8.51067, 4.5064, 4.3157	10.88502,	3	epha3	34305
GL172645.1	4742245	4743238	4743095, 4742484, 4742646	5, 11, 7	4.82177, 9.43766, 8.35502	3.98509, 6.22212, 5.9108	2.51418, 6	3	nsun3	23578
GL172645.1	4902566	4903368	4902772, 4902772, 4902773	32, 33, 18	36.02624, 34.12088, 21.89732	15.70838, 14.18344, 12.06835	32.39277,	3	nsun3	183899
GL172645.1	4908936	4909430	4908968, 4908963, 4908968	14, 20, 6	15.15267, 19.78674, 7.29403	9.07115, 10.29739, 5.30581	12.11465,	3	nsun3	190269
GL172645.1	4949789	4950041	4949816, 4949820, 4949817	37, 57, 28	43.3508, 67.45239, 37.85844	17.94155, 22.44073, 18.2494	39.59576,	3	nsun3	231122
GL172645.1	4993223	4993839	4993627, 4993618, 4993672	8, 11, 8	7.38183, 8.85546, 9.11678	5.38666, 5.85974, 6.33924	4.80856, 6	3	epha6	260386
GL172645.1	5047613	5048542	5048369, 5048176, 5048240	5, 10, 3	4.30277, 7.97488, 3.37781	3.68597, 5.48553, 2.98791	2.12436, 5	3	epha6	205683
GL172645.1	5104170	5104514	5104488, 5104487, 5104432	22, 23, 12	25.96118, 24.50192, 13.44151	13.35351, 12.17534, 8.41472	22.55288,	3	epha6	149711
GL172645.1	5204219	5204671	5204222, 5204380, 5204244	18, 15, 6	19.69649, 13.8628, 6.25896	10.81516, 8.08196, 4.80589	16.47841,	3	epha6	49554
GL172645.1	5213401	5214520	5213789, 5213754, 5213956	6, 8, 5	4.22823, 6.30984, 5.40789	3.59714, 4.72927, 4.29296	2.0602, 3.9	3	epha6	39705
GL172645.1	5242376	5242929	5242574, 5242429, 5242460	10, 14, 6	9.38842, 12.70158, 6.168	6.35473, 7.60958, 4.75778	6.67167, 9	3	epha6	11296
GL172645.1	5323397	5323823	5323548, 5323512, 5323540	35, 39, 15	37.54288, 34.90856, 16.01939	15.37419, 12.37604, 9.37366	33.88042,	3	epha6	0
GL172645.1	5326732	5327507	5326927, 5326957, 5327242	13, 33, 10	9.73159, 29.42446, 10.03522	6.00089, 11.52185, 6.73493	6.98212, 2	3	epha6	0
GL172645.1	5406874	5408188	5407864, 5407551, 5407430	14, 29, 11	11.31081, 23.22735, 9.92808	6.75314, 9.45651, 6.52366	8.46526, 2	3	epha6	0
GL172645.1	5486374	5486681	5486581, 5486462, 5486581	12, 15, 5	13.3031, 15.80067, 5.9506	8.33871, 9.24574, 4.54784	10.35242,	3	epha6	0
GL172645.1	5627377	5628522	5627783, 5627435, 5627412	13, 8, 14	13.5657, 6.44699, 14.1066	8.33634, 4.8155, 8.45936	10.60274,	3	arl6	16263
GL172646.1	388524	388916	388594, 388619, 388799	6, 10, 6	5.3939, 7.61995, 5.95321	4.32314, 5.26198, 4.64162	3.0374, 5.1	3	col4a6	0
GL172646.1	536792	537213	537032, 536957, 537076	24, 26, 14	26.57606, 26.46975, 16.64329	13.07811, 12.31359, 9.89801	23.15164,	3	picalm.2	0
GL172646.1	555946	556525	556413, 556356, 556345	6, 7, 7	5.24248, 5.10722, 8.69489	4.2332, 4.07606, 6.06379	2.89962, 2	3	picalm.2	4182
GL172646.1	1148602	1149649	1148893, 1149066, 1149213	7, 7, 4	5.21879, 4.03287, 4.46488	4.14768, 3.37324, 3.69425	2.87895, 1	3	NA	277875
GL172646.1	1151366	1152202	1151607, 1151458, 1151965	6, 8, 5	4.37941, 5.00801, 5.9506	3.69512, 3.89278, 4.54784	2.19155, 2	3	NA	275322
GL172646.1	1151629	1152761	1152495, 1152649, 1151965	8, 10, 5	6.41335, 7.1111, 5.9506	4.7944, 4.93988, 4.54784	3.93678, 4	3	NA	274763
GL172646.1	1159091	1159910	1159481, 1159382, 1159472	14, 28, 12	14.67861, 28.88701, 13.98181	8.79666, 13.02331, 8.70517	11.66087,	3	NA	267614
GL172646.1	2530230	2531331	2530698, 2530732, 2530632	16, 22, 10	15.47689, 18.72754, 10.3632	8.81381, 9.04803, 6.92219	12.4218, 1	3	gria3	0
GL172646.1	2894968	2896813	2895806, 2895798, 2895732	21, 23, 11	17.71848, 19.04539, 11.92945	8.75244, 8.9325, 7.69409	14.5719, 1	3	NA	133810
GL172646.1	3349757	3350367	3349942, 3349941, 3349832	6, 8, 5	5.96715, 7.07159, 5.59755	4.64927, 5.20073, 4.38538	3.52158, 4	3	abcb7	110389
GL172646.1	3426762	3427715	3427278, 3427144, 3427137	7, 16, 13	6.42049, 15.94397, 14.32377	4.8888, 9.09593, 8.76942	3.94161, 1	3	abcb7	33041
GL172646.1	3620528	3621562	3620946, 3621196, 3620850	8, 14, 6	7.52511, 11.71767, 7.12188	5.47115, 7.00416, 5.22884	4.94319, 9	3	slc16a2	8647
GL172646.1	4145401	4146149	4145886, 4146012, 4145401, 4145974	13, 11, 4, 7	15.29169, 10.60895, 4.67325, 8.69489	9.29854, 6.9343, 3.78987, 6.06379	12.24396,	3	NA	90641
GL172646.1	4751854	4752488	4752254, 4751908, 4752280	10, 11, 7	9.77513, 9.13706, 8.646	6.5837, 6.0355, 6.04229	7.02302, 6	3	NA	8287
GL172646.1	4763341	4763593	4763567, 4763553, 4763566	31, 27, 23	33.7354, 25.18667, 27.70251	14.74934, 11.20272, 14.06048	30.14729,	3	zc4h2	17387
GL172646.1	5011706	5012612	5012434, 5012577, 5012467	9, 15, 9	8.88537, 13.37045, 11.21577	6.17591, 7.78062, 7.38849	6.19961, 1	3	atp1b4	5788
GL172646.1	5585309	5585644	5585545, 5585530, 5585547	32, 38, 33	15.47026, 15.85155, 20.13186	5.31268, 4.73711, 6.98737	12.41604,	3	fgf16	168001
GL172646.1	5589969	5590448	5590329, 5590003, 5590177	17, 18, 11	6.25991, 4.61864, 3.83057	3.44092, 2.66017, 2.82527	3.79333, 2	3	fgf16	172661
GL172646.1	5600280	5600622	5600529, 5600402, 5600512	22, 22, 22	9.33592, 7.25195, 12.75742	4.2165, 3.38154, 5.78628	6.62299, 4	3	fgf16	182972
GL172647.1	131252	133061	131760, 131501, 131413, 132561	10, 21, 7, 11	7.9709, 16.79972, 5.85759, 10.66921	5.48303, 8.21417, 4.54944, 6.9701	5.36088, 1	3	xtr-mir-129-2	18174
GL172647.1	833289	834039	833547, 833623, 833767	9, 9, 9	8.88537, 6.34719, 9.82249	6.17591, 4.61437, 6.69629	6.19961, 3	3	ahcyl2	0
GL172647.1	1166632	1168078	1167068, 1166756, 1167441	11, 14, 8	11.22377, 12.18837, 9.1831	7.29486, 7.29433, 6.37229	8.38272, 9	3	klhdc10	0
GL172647.1	3055192	3056020	3055614, 3055619, 3055631	10, 18, 8	5.37749, 11.26696, 5.39841	3.84941, 5.81728, 4.14589	3.02404, 8	3	mkln1	15197
GL172647.1	3058365	3059061	3058813, 3058760, 3058790	20, 26, 21	6.9874, 8.96523, 10.90366	3.45739, 3.65807, 5.0655	4.46684, 6	3	mkln1	18370
GL172647.1	3060572	3063428	3062753, 3062742, 3062296	58, 61, 44	43.08618, 40.35012, 34.98159	10.56637, 9.02503, 10.83907	39.33566,	3	mkln1	20577
GL172647.1	3954825	3955823	3955305, 3955298, 3955341	10, 22, 7	9.71769, 20.6214, 6.36213	6.54998, 10.17315, 4.85436	6.97155, 1	3	sfmbt2	55416
GL172647.1	5357834	5358422	5358282, 5358197, 5357956	9, 8, 5	8.40425, 6.81727, 4.99237	5.89416, 5.04548, 4.0781	5.76336, 4	3	USP6NL	0
GL172647.1	5498655	5499412	5499144, 5499126, 5499158	9, 22, 19	8.61068, 20.75023, 22.51865	6.01615, 10.2504, 12.19038	5.94135, 1	3	UPF2	10852
GL172648.1	198461	200872	200331, 200361, 199446	13, 14, 6	11.45737, 10.91946, 5.8719	7.06931, 6.51197, 4.59674	8.60415, 8	3	adgrd1	53196
GL172648.1	293402	293783	293732, 293717, 293740	19, 13, 12	20.30552, 11.66277, 14.67601	10.90398, 7.19557, 9.06267	17.06569,	3	NA	18556
GL172648.1	2863217	2864413	2863644, 2863629, 2863971	16, 36, 9	14.79948, 32.70133, 9.41119	8.40174, 12.25543, 6.47287	11.77649,	3	tbx1	12005
GL172648.1	4084751	4085220	4084923, 4084904, 4084895	10, 22, 17	9.18236, 18.47303, 21.19882	6.23091, 8.89891, 11.87761	6.47811, 1	3	NA	10496
GL172648.1	4094515	4094948	4094867, 4094872, 4094712	9, 17, 5	8.55802, 15.99248, 5.03069	5.98518, 8.86336, 4.09861	5.8909, 13	3	NA	6862
GL172648.1	4202310	4202951	4202442, 4202703, 4202670	7, 11, 5	6.42049, 10.05535, 5.54867	4.8888, 6.6012, 4.3619	3.94161, 7	3	NA	0
GL172648.1	4218078	4218783	4218394, 4218246, 4218194	9, 19, 11	8.66407, 18.08435, 12.63525	6.04743, 9.56723, 8.07536	5.99101, 1	3	NA	0
GL172648.1	4403511	4403813	4403784, 4403789, 4403762	17, 29, 12	17.80138, 29.11719, 12.09328	9.95359, 12.78747, 7.64909	14.65165,	3	NA	13028
GL172649.1	145169	146019	145679, 145690, 145625	6, 15, 8	5.96715, 12.49102, 8.9249	4.64927, 7.24068, 6.24211	3.52158, 9	3	slitrk1	62523
GL172649.1	147533	148194	147942, 147729, 147856	9, 16, 7	9.72082, 13.83763, 7.10639	6.64182, 7.81372, 5.27894	6.97155, 1	3	slitrk1	60348
GL172649.1	639491	640964	640263, 640121, 640158	14, 27, 12	15.84929, 29.84144, 13.29687	9.46495, 13.96317, 8.33527	12.77923,	3	spry2	131642
GL172649.1	677331	678057	677520, 677794, 677793	12, 13, 8	13.3031, 10.68145, 9.1831	8.33871, 6.58965, 6.37229	10.35242,	3	spry2	94549
GL172649.1	1132882	1133562	1132971, 1133488, 1133532	5, 10, 4	4.33555, 7.86505, 4.55201	3.70555, 5.4165, 3.73489	2.15195, 5	3	rbm26	95142
GL172649.1	2053689	2054094	2054053, 2054025, 2054057	7, 8, 5	6.99564, 5.75617, 5.9506	5.21786, 4.3766, 4.54784	4.47307, 3	3	kctd12	52820
GL172649.1	2502359	2502713	2502541, 2502430, 2502505	5, 7, 4	4.61714, 5.97725, 4.67325	3.86998, 4.62287, 3.78987	2.39372, 3	3	tbc1d4	13448
GL172649.1	2692879	2693304	2693195, 2693249, 2693249	6, 9, 6	5.16999, 7.28344, 5.75442	4.18963, 5.20811, 4.53101	2.83325, 4	3	klf12	5549
GL172649.1	3138637	3138930	3138770, 3138870, 3138896	6, 8, 5	5.63898, 7.06103, 4.64121	4.46544, 5.19433, 3.88372	3.25772, 4	3	dach1	20652
GL172649.1	4908622	4909349	4908858, 4909164, 4909084	14, 22, 15	16.43009, 22.61717, 17.94632	9.78349, 11.37311, 10.45626	13.33671,	3	mettl18	265909
GL172650.1	37954	38409	38033, 38350, 38160	7, 8, 5	7.1696, 7.01915, 5.9506	5.31345, 5.16892, 4.54784	4.61046, 4	3	prkar1a	179682
GL172650.1	445869	446594	446222, 446369, 446182	12, 17, 4	13.8483, 17.79269, 4.59709	8.63436, 9.94842, 3.75554	10.86694,	3	NA	13714
GL172650.1	730691	731773	731157, 731356, 731299	5, 28, 11	3.73498, 26.47393, 11.08949	3.33372, 11.60281, 7.21705	1.6222, 23	3	fam20c	118832
GL172650.1	980252	980668	980265, 980283, 980302	13, 16, 11	13.49269, 14.69174, 12.13051	8.29387, 8.33598, 7.80464	10.53271,	3	fam20c	97728
GL172650.1	1258552	1258850	1258796, 1258816, 1258814	34, 32, 32	38.23524, 34.62255, 40.95115	16.19629, 14.87654, 18.61058	34.56211,	3	NA	168344
GL172650.1	1462221	1463003	1462308, 1462310, 1462877	7, 9, 5	6.42049, 8.21099, 5.31857	4.8888, 5.77858, 4.24819	3.94161, 5	3	NA	34929
GL172650.1	1590707	1591561	1591367, 1591266, 1590745, 1591284	11, 13, 4, 3	11.85906, 10.59688, 4.67325, 3.47371	7.65504, 6.53722, 3.78987, 3.03189	8.98595, 7	3	NA	58567
GL172650.1	3245604	3246812	3246283, 3246349, 3246318	24, 29, 15	25.45469, 31.85193, 17.07266	12.40824, 14.40958, 9.97618	22.06058,	3	zp2	9452
GL172650.1	3524718	3525866	3524877, 3525513, 3525516	8, 8, 5	7.67464, 7.07159, 5.9506	5.55832, 5.20073, 4.54784	5.08299, 4	3	lyrm1	420
GL172650.1	3525014	3525957	3525845, 3525513, 3525516	7, 8, 5	6.16939, 7.07159, 5.9506	4.73936, 5.20073, 4.54784	3.70916, 4	3	lyrm1	716
GL172650.1	3953842	3954787	3954186, 3954407, 3954088	8, 10, 6	7.83097, 8.36423, 7.29403	5.64832, 5.72892, 5.30581	5.22917, 5	3	umod.1	14552
GL172650.1	4072300	4072707	4072658, 4072505, 4072507	20, 18, 9	19.84853, 17.06771, 10.28145	10.3347, 9.23949, 6.93568	16.62382,	3	NA	74984
GL172650.1	4351842	4352193	4352157, 4352145, 4352167	24, 25, 18	28.65257, 27.83016, 23.48722	14.30048, 13.48127, 12.91397	25.17715,	3	NA	354526
GL172650.1	4408841	4409469	4409141, 4409181, 4409050	7, 9, 10	7.1696, 7.92635, 12.52085	5.31345, 5.60609, 8.03986	4.61046, 5	3	hs3st4	382904
GL172650.1	4443224	4444166	4443485, 4443694, 4443494	14, 31, 14	15.66744, 31.36029, 16.29325	9.36333, 13.34912, 9.7093	12.60415,	3	hs3st4	348207
GL172650.1	4913784	4914094	4914056, 4913816, 4914021	14, 10, 9	12.91867, 7.8291, 9.19414	7.74237, 5.39387, 6.35176	9.98624, 5	3	hs3st2	115631
GL172650.1	5025541	5025978	5025589, 5025569, 5025573	8, 14, 12	6.70593, 11.80872, 13.7434	4.9768, 7.06033, 8.57827	4.20487, 9	3	hs3st2	3747
GL172651.1	1565293	1566732	1566019, 1565821, 1565733	16, 29, 9	15.67432, 29.43697, 10.87975	8.9333, 12.97595, 7.23113	12.61068,	3	trpm8	0
GL172651.1	3332525	3332753	3332628, 3332635, 3332628	40, 39, 44	38.51525, 34.23376, 45.08379	13.92861, 12.02731, 15.92251	34.83764,	3	col6a2	9816
GL172651.1	3475923	3476545	3476206, 3476073, 3476218	5, 10, 3	4.23867, 8.44678, 3.47371	3.64743, 5.78021, 3.03189	2.0685, 5.9	3	col6a1	12352
GL172651.1	3665088	3665983	3665143, 3665135, 3665167	66, 87, 54	77.86633, 101.5396, 71.19955	23.73208, 25.73494, 26.13386	73.69058,	3	col18a1	0
GL172651.1	5305179	5306155	5305623, 5305644, 5305848	8, 17, 9	6.37855, 14.86656, 9.46739	4.77253, 8.17729, 6.50386	3.90357, 1	3	cwc22	155265
GL172652.1	1069978	1070836	1070297, 1070306, 1070237	14, 24, 10	12.08222, 19.91463, 10.71812	7.22895, 9.13097, 7.12015	9.19818, 1	3	mgat4a	11551
GL172652.1	2739971	2740598	2740141, 2740069, 2740099	13, 7, 9	13.34951, 5.58561, 11.21577	8.21021, 4.38027, 7.38849	10.39596,	3	pcdh18	109772
GL172652.1	2841067	2841974	2841719, 2841439, 2841435	26, 30, 23	24.26079, 23.75985, 26.32652	11.0052, 9.4421, 13.25965	20.90031,	3	NA	19756
GL172652.1	3037329	3039720	3037587, 3038738, 3038239	10, 20, 16	8.28204, 16.68318, 16.83466	5.67774, 8.43077, 9.62725	5.64968, 1	3	NA	68647
GL172652.1	3313863	3314116	3314008, 3314035, 3314030	9, 18, 6	9.63662, 18.00717, 7.25145	6.59633, 9.80837, 5.287	6.90556, 1	3	pcdh10	188307
GL172652.1	3785394	3786277	3785855, 3785965, 3785661	15, 12, 8	15.41679, 9.99443, 8.14528	9.0192, 6.37457, 5.82558	12.36344,	3	NA	41158
GL172652.1	4825337	4826449	4825921, 4826016, 4825982, 4826408	14, 14, 7, 4	13.70063, 10.76513, 6.92462, 3.59344	8.21651, 6.41704, 5.17829, 3.23643	10.73143,	3	NA	23986
GL172652.1	5181911	5182772	5182237, 5182262, 5182332	121, 142, 55	113.44639, 123.60917, 44.63151	18.27457, 16.30636, 11.92363	108.94656	3	fat4	141549
GL172652.1	5225474	5226990	5226553, 5226387, 5226180	13, 16, 9	11.50747, 12.44939, 8.98852	7.10015, 6.9679, 6.23511	8.65224, 9	3	fat4	97331
GL172653.1	450871	452117	451419, 451474, 451424	23, 34, 22	23.56695, 31.92732, 25.9713	11.61446, 12.54908, 13.35936	20.22607,	3	aldh3b1	6059

GL172653.1	1322065	1323106	1322351, 1322823, 1322717	8, 16, 4	6.48409, 12.90802, 3.76128	4.83873, 7.24623, 3.33171	4.00135, 1	3	kiaa1671	0
GL172653.1	1594590	1595224	1594773, 1594904, 1595098	14, 10, 5	13.8308, 7.61995, 5.9506	8.29466, 5.26198, 4.54784	10.85567,	3	GRK3	2294
GL172653.1	2542008	2542265	2542225, 2542214, 2542230	15, 21, 7	14.71973, 19.60696, 7.55909	8.60219, 9.87975, 5.52035	11.70031,	3	NA	150078
GL172653.1	2614242	2617976	2614977, 2614556, 2617367, 2614671, 2617363	16, 17, 20, 14, 13	12.18769, 11.29126, 13.46685, 12.55907, 11.42074	6.80985, 6.04742, 6.5842, 7.52221, 7.04675	9.2978, 8.6	3	NA	222312
GL172653.1	2615640	2617976	2617726, 2617367, 2617363	11, 20, 13	6.74933, 13.46685, 11.42074	4.54101, 6.5842, 7.04675	4.24602, 1	3	NA	223710
GL172653.1	3889102	3889435	3889319, 3889240, 3889282	7, 10, 4	5.4599, 7.72315, 4.67325	4.30109, 5.32711, 3.78987	3.09813, 5	3	chek2	1683
GL172653.1	5382236	5382831	5382420, 5382551, 5382595	8, 9, 5	7.77807, 7.83963, 5.9506	5.618, 5.55305, 4.54784	5.17956, 5	3	hnf1a	3359
GL172654.1	49	809	630, 296, 314, 707	16, 34, 20, 9	12.97466, 30.86867, 20.9157, 7.18747	7.2868, 11.96435, 10.97698, 5.14788	10.03988,	3	traf3ip2	7346
GL172654.1	286866	287575	287094, 287126, 287166	9, 12, 8	9.30591, 9.0625, 10.00633	6.41439, 5.7937, 6.75998	6.59443, 6	3	slc35f1	0
GL172654.1	551385	551886	551666, 551671, 551475	20, 18, 3	19.49401, 16.81975, 3.47371	10.12053, 9.08887, 3.03189	16.28237,	3	mcm9	27269
GL172654.1	2547852	2549059	2548217, 2548603, 2548685	26, 29, 15	27.19703, 26.50813, 17.67167	12.74794, 11.27285, 10.30745	23.75642,	3	rhoc	1824
GL172654.1	2941078	2941898	2941679, 2941487, 2941680	4, 11, 8	3.39747, 9.4823, 9.1831	3.12111, 6.24973, 6.37229	1.46102, 6	3	slc16a1	265981
GL172654.1	3732092	3733342	3732545, 3732949, 3732510	8, 12, 4	6.70593, 8.16747, 3.21039	4.9768, 5.23696, 3.0071	4.20487, 5	3	lrig2	24651
GL172654.1	3818141	3818728	3818579, 3818576, 3818494	14, 15, 11	11.84416, 13.37045, 12.9493	7.08219, 7.78062, 8.23866	8.97236, 1	3	magi3	73243
GL172654.1	4228368	4229599	4229329, 4229390, 4228493	15, 11, 11	14.98937, 10.3713, 11.31465	8.76428, 6.79219, 7.3472	11.95813,	3	ap4b1	0
GL172655.1	1162264	1165512	1163452, 1163470, 1163447	31, 36, 23	21.2285, 20.71536, 16.41929	7.91223, 6.66751, 7.46161	17.95971,	3	usp45	46245
GL172655.1	1171300	1172170	1171732, 1171549, 1171718	16, 22, 14	13.38469, 16.87452, 13.98966	7.53684, 7.97846, 8.38968	10.43027,	3	usp45	55281
GL172655.1	1183863	1186434	1184715, 1184512, 1185260, 1186321	32, 39, 24, 12	24.17955, 27.35566, 20.91024, 8.07571	9.08697, 8.76989, 9.70563, 5.18021	20.8214, 2	3	usp45	67844
GL172655.1	1214804	1216806	1215480, 1216020, 1215977	22, 31, 20	18.79573, 23.24262, 17.73515	9.08809, 8.89289, 9.05916	15.60788,	3	usp45	98785
GL172655.1	1260453	1263157	1261119, 1262767, 1261768	26, 31, 14	19.53639, 22.83006, 11.71881	8.34369, 8.68721, 7.00487	16.32346,	3	usp45	144434
GL172655.1	1477440	1477688	1477653, 1477646, 1477623	9, 15, 7	8.16099, 15.26186, 7.20145	5.74847, 8.92711, 5.33074	5.53692, 1	3	GRIN2B	0
GL172655.1	1508969	1509218	1509132, 1509106, 1509079	10, 19, 9	11.06011, 20.47061, 11.64359	7.306, 11.002, 7.57973	8.22712, 1	3	GRIN2B	0
GL172655.1	1837569	1838173	1837797, 1837799, 1837829	12, 18, 10	11.92103, 15.79712, 9.93124	7.54764, 8.46772, 6.67475	9.04508, 1	3	mei1	0
GL172655.1	1959126	1960267	1960079, 1960136, 1960051	17, 23, 14	15.39375, 20.1396, 14.92875	8.49863, 9.56971, 8.94211	12.34203,	3	b3galt2	8943
GL172655.1	2109797	2110683	2110220, 2110280, 2110223	34, 32, 30	29.81074, 26.37138, 27.701	11.39108, 10.21023, 11.60375	26.30802,	3	cdc73	12327
GL172655.1	2550927	2551499	2551238, 2551202, 2551237	11, 8, 5	11.15818, 6.34359, 4.84526	7.25692, 4.75053, 3.99806	8.32008, 3	3	U4	193509
GL172655.1	2688742	2689873	2689059, 2689063, 2689062	21, 25, 19	19.77667, 22.53775, 19.82575	9.9819, 10.33209, 10.61774	16.55569,	3	U4	55135
GL172655.1	3367509	3368045	3367744, 3367746, 3367744	11, 14, 9	11.09349, 13.26509, 10.56949	7.21938, 7.95332, 7.08032	8.25916, 1	3	F13B	6640
GL172655.1	4020003	4020432	4020311, 4020293, 4020232	5, 8, 7	4.82177, 7.07159, 8.08824	3.98509, 5.20073, 5.7849	2.51418, 4	3	nr5a2	66951
GL172655.1	4531126	4531790	4531360, 4531409, 4531318	17, 26, 13	16.4953, 26.46975, 14.93222	9.16875, 12.31359, 9.10541	13.39926,	3	DENND1B	13618
GL172655.1	4752874	4753435	4752896, 4753301, 4753229	7, 7, 4	7.10297, 5.8586, 4.67325	5.27706, 4.55007, 3.78987	4.57191, 3	3	fam102b	0
GL172656.1	680834	681889	681446, 681425, 681746	11, 20, 7	9.05039, 18.05851, 7.45266	5.98149, 9.25366, 5.46481	6.35485, 1	3	znf800	0
GL172656.1	968031	968722	968593, 968174, 968574	7, 12, 3	6.37715, 9.42893, 3.47371	4.86324, 6.02236, 3.03189	3.90257, 6	3	mapk11	9396
GL172656.1	1962607	1963598	1963566, 1963551, 1963546	13, 25, 16	13.71465, 25.91543, 19.86305	8.42261, 12.33685, 11.32783	10.74495,	3	brd1	0
GL172656.1	4671943	4673134	4672975, 4672525, 4672496	12, 20, 6	13.55951, 18.23344, 6.5517	8.47903, 9.35906, 4.95623	10.59689,	3	lrrk2	15436
GL172657.1	84026	85111	84775, 85035, 84549	10, 9, 5	9.49535, 8.0603, 5.31857	6.41851, 5.68758, 4.24819	6.77344, 5	3	abcc1	3409
GL172657.1	517623	519359	518119, 518027, 517994, 518685	21, 29, 10, 10	18.79976, 24.27206, 9.44624, 9.53891	9.39534, 10.02167, 6.38926, 6.44439	15.6117, 2	3	NA	110635
GL172657.1	1653288	1654084	1653620, 1653661, 1653517	6, 14, 7	4.51306, 11.15936, 7.72582	3.78094, 6.65976, 5.6058	2.30616, 8	3	NA	67514
GL172657.1	2120854	2121407	2121026, 2121022, 2121018	10, 16, 14	9.77513, 13.5443, 15.20637	6.5837, 7.63435, 9.10193	7.02302, 1	3	c16orf62	357
GL172657.1	2631257	2631833	2631441, 2631395, 2631518	7, 11, 4	5.55587, 9.90518, 4.46488	4.36159, 6.50968, 3.69425	3.18254, 7	3	chst12	133639
GL172657.1	2677898	2678229	2678155, 2678165, 2678087	16, 15, 6	18.34375, 13.16372, 5.64193	10.49905, 7.65379, 4.46713	15.17198,	3	chst12	87243
GL172657.1	2888655	2889231	2889068, 2889048, 2888963	14, 21, 4	16.32935, 22.31381, 4.67325	9.72892, 11.50891, 3.78987	13.23998,	3	NA	12165
GL172657.1	2907779	2908382	2908278, 2908013, 2908014	14, 21, 18	13.57329, 21.26419, 20.07449	8.13982, 10.87863, 11.036	10.60942,	3	NA	31289
GL172657.1	2939703	2940361	2940246, 2940311, 2940273	42, 51, 32	47.63214, 59.27342, 42.50305	18.22971, 20.73437, 19.50142	43.81326,	3	NA	63213
GL172657.1	3047348	3047903	3047861, 3047595, 3047836	7, 10, 10	7.1696, 8.74956, 10.42037	5.31345, 5.96721, 6.95441	4.61046, 6	3	zfat	84701
GL172657.1	3182673	3183119	3183061, 3183069, 3183078	29, 35, 9	31.93309, 40.9161, 10.96118	14.4579, 17.36254, 7.26984	28.38297,	3	zfat	2473
GL172657.1	3212907	3213415	3213095, 3212943, 3213232	9, 13, 6	9.63662, 11.81904, 6.30561	6.59633, 7.29135, 4.83031	6.90556, 9	3	xtr-mir-30d	13043
GL172657.1	3240910	3241720	3241169, 3241184, 3241281	4, 13, 4	3.26298, 12.09006, 4.67325	3.03953, 7.45678, 3.78987	1.35546, 9	3	xtr-mir-30d	41046
GL172657.1	3260547	3261398	3261118, 3261177, 3261280	7, 9, 3	7.1696, 8.15232, 3.34298	5.31345, 5.74324, 2.97156	4.61046, 5	3	xtr-mir-30d	60683
GL172657.1	3267261	3267524	3267487, 3267482, 3267362	12, 21, 9	13.8483, 24.35402, 11.64359	8.63436, 12.71289, 7.57973	10.86694,	3	xtr-mir-30d	67397
GL172657.1	3275702	3276257	3275896, 3275876, 3275954	8, 11, 8	8.42257, 10.26356, 10.1465	5.97763, 6.72731, 6.82176	5.76336, 7	3	xtr-mir-30d	75838
GL172657.1	3359865	3360657	3360493, 3360367, 3360407	16, 34, 11	14.91759, 34.24377, 12.71187	8.47377, 13.86165, 8.11558	11.89002,	3	xtr-mir-30d	160001
GL172657.1	3402713	3403351	3403334, 3403287, 3403116	25, 37, 6	28.66822, 43.50184, 6.71074	13.97856, 18.03078, 5.03498	25.19216,	3	xtr-mir-30d	202849
GL172657.1	3413866	3414267	3413906, 3413977, 3414008	10, 10, 5	11.06011, 9.12511, 5.9506	7.306, 6.19629, 4.54784	8.22712, 6	3	xtr-mir-30d	214002
GL172657.1	3479253	3479934	3479895, 3479742, 3479906	20, 24, 8	21.18126, 23.92411, 9.05167	11.13591, 11.49076, 6.30653	17.91394,	3	khdrbs3	170766
GL172657.1	3673006	3673624	3673032, 3673032, 3673038	88, 107, 77	83.26651, 96.43219, 76.10773	17.38498, 16.58483, 18.17522	79.03815,	3	khdrbs3	0
GL172657.1	3676400	3676812	3676769, 3676774, 3676785	127, 184, 120	138.88805, 206.70218, 139.15154	25.08741, 28.67176, 27.95506	134.22475	3	khdrbs3	0
GL172657.1	3785079	3785901	3785773, 3785854, 3785862	5, 10, 14	4.82177, 9.39128, 18.40474	3.98509, 6.35644, 10.78924	2.51418, 6	3	khdrbs3	58268
GL172657.1	3859315	3859922	3859543, 3859408, 3859396	20, 15, 14	18.76393, 12.01071, 14.66346	9.67925, 6.9461, 8.7878	15.57675,	3	khdrbs3	132504
GL172657.1	3861810	3863040	3862707, 3862707, 3862708	24, 29, 17	24.13149, 29.11719, 18.50132	11.615, 12.78747, 10.36648	20.77422,	3	khdrbs3	134999
GL172657.1	3883526	3884116	3884090, 3884088, 3884082	25, 39, 26	27.92938, 43.42602, 32.2816	13.54032, 17.1121, 15.7501	24.4711, 3	3	khdrbs3	156715
GL172657.1	3972294	3972873	3972353, 3972353, 3972351	41, 42, 23	49.86291, 44.1924, 29.17927	19.99249, 16.26188, 14.89588	46.01223,	3	NA	88274
GL172657.1	3979664	3980071	3979861, 3979728, 3979672	9, 14, 7	8.40425, 12.04301, 7.40077	5.89416, 7.20479, 5.43746	5.76336, 9	3	NA	81076
GL172657.1	4129581	4130473	4130185, 4130120, 4129879	8, 18, 12	7.28955, 15.79712, 13.36868	5.33177, 8.46772, 8.37481	4.72176, 1	3	NA	7681
GL172657.1	4144654	4145222	4144893, 4144700, 4145005	8, 10, 5	7.99469, 7.68845, 5.91431	5.74128, 5.30522, 4.53172	5.38155, 5	3	NA	22754
GL172657.1	4181605	4182295	4181635, 4181649, 4181897	11, 12, 10	10.1122, 10.58612, 12.61444	6.63573, 6.74063, 8.08336	7.34229, 7	3	NA	59705
GL172657.1	4199156	4199455	4199418, 4199375, 4199413	22, 16, 13	25.96118, 17.16856, 15.70541	13.35351, 9.82359, 9.51556	22.55288,	3	NA	77256
GL172657.1	4311441	4311882	4311635, 4311650, 4311647	10, 28, 15	8.93828, 28.15561, 16.37605	6.08275, 12.58981, 9.5803	6.24946, 2	3	NA	189541
GL172657.1	4327469	4327841	4327674, 4327706, 4327622	15, 22, 8	18.26603, 22.94165, 9.68495	10.62691, 11.56804, 6.61366	15.09729,	3	NA	205569
GL172657.1	4353938	4354428	4354153, 4354153, 4354153	13, 22, 22	14.43911, 24.08291, 26.07069	8.83395, 12.25042, 13.41675	11.43182,	3	NA	232038
GL172657.1	4387650	4388010	4387789, 4387809, 4387775	13, 16, 8	13.20994, 15.67391, 9.68495	8.12823, 8.93306, 6.61366	10.26285,	3	FAM135B	257486
GL172657.1	4396680	4396965	4396746, 4396834, 4396769	6, 8, 6	5.95712, 7.07159, 6.5517	4.64377, 5.20073, 4.95623	3.52158, 4	3	FAM135B	248531
GL172657.1	4459511	4460277	4459862, 4459823, 4460112	26, 25, 20	24.46003, 24.6617, 20.9157	11.12216, 11.58741, 10.97698	21.09356,	3	FAM135B	185219
GL172657.1	4547148	4547565	4547167, 4547238, 4547201	5, 6, 4	4.43696, 4.45754, 4.50795	3.76555, 3.74538, 3.71446	2.24017, 2	3	FAM135B	97931
GL172657.1	4582341	4583150	4582533, 4582636, 4583018	7, 6, 3	6.33443, 4.93312, 3.17999	4.83795, 4.04501, 2.89245	3.86259, 2	3	FAM135B	62346
GL172657.1	4608779	4609273	4609058, 4609128, 4609042	6, 5, 4	5.96715, 3.63593, 4.67325	4.64927, 3.26995, 3.78987	3.52158, 1	3	FAM135B	36223
GL172657.1	4614349	4614996	4614787, 4614776, 4614794	13, 10, 9	13.20994, 8.36423, 8.88966	8.12823, 5.72892, 6.17838	10.26285,	3	FAM135B	30500
GL172657.1	4865980	4866868	4866590, 4866621, 4866529	12, 11, 8	13.05874, 9.8562, 8.9249	8.20296, 6.47973, 6.24211	10.11961,	3	COL22A1	23202
GL172657.1	4948712	4950175	4949013, 4949901, 4949899	9, 10, 8	8.30507, 8.12616, 8.4586	5.83501, 5.58036, 5.99709	5.67102, 5	3	COL22A1	105934
GL172657.1	4948805	4950175	4949893, 4949901, 4949899	9, 10, 8	8.61068, 8.12616, 8.4586	6.01615, 5.58036, 5.99709	5.94135, 5	3	COL22A1	106027
GL172658.1	9381	10085	10000, 10046, 10031	15, 22, 12	15.71867, 21.01356, 13.7434	9.19755, 10.40847, 8.57827	12.65377,	3	gdf5	79293
GL172658.1	426373	426631	426402, 426401, 426404	7, 10, 15	6.60038, 8.24345, 19.89124	4.99378, 5.65367, 11.44789	4.10761, 5	3	NA	0
GL172658.1	1386106	1386583	1386531, 1386558, 1386510	14, 18, 8	14.38232, 17.32425, 9.92329	8.62271, 9.39518, 6.7228	11.37721,	3	acan	71715
GL172658.1	1611730	1612051	1611787, 1611798, 1611860	7, 12, 6	7.1696, 11.86108, 7.12188	5.31345, 7.51216, 5.22884	4.61046, 9	3	mfge8	11352
GL172658.1	2671843	2672955	2672159, 2672273, 2672336	11, 9, 8	11.71035, 8.21099, 9.1831	7.57196, 5.77858, 6.37229	8.8442, 5.7	3	ap3s2	11973
GL172658.1	3807445	3808337	3808267, 3807514, 3807775	8, 11, 7	6.86256, 10.15827, 8.69489	5.0733, 6.66366, 6.06379	4.3506, 7.5	3	slc39a1	9342
GL172658.1	3937058	3937452	3937197, 3937156, 3937166	13, 13, 4	12.94099, 10.98869, 4.67325	7.96907, 6.77996, 3.78987	10.00729,	3	ppip5k1	0
GL172658.1	4139241	4139618	4139307, 4139434, 4139387	8, 13, 5	7.06928, 11.17268, 4.64121	5.19933, 6.89372, 3.88372	4.5414, 8.5	3	TP53BP1	0
GL172658.1	4766504	4766973	4766779, 4766825, 4766695	5, 8, 5	4.47183, 6.44699, 5.80393	3.78598, 4.8155, 4.48186	2.26935, 4	3	NA	76809
GL172659.1	53332	53813	53399, 53624, 53483	6, 11, 4	4.62553, 7.91281, 3.56693	3.85252, 5.26803, 3.22108	2.40124, 5	3	lmf1	0
GL172659.1	55078	55721	55483, 55484, 55318	13, 10, 4	12.09198, 6.8797, 4.15126	7.45795, 4.79317, 3.54016	9.20698, 4	3	lmf1	0
GL172659.1	133546	134364	133874, 133704, 133657	11, 15, 9	11.29029, 13.69427, 10.01275	7.3332, 7.97895, 6.79683	8.44541, 1	3	lmf1	65444
GL172659.1	194527	195069	195031, 195029, 195022	14, 10, 6	14.23954, 8.70492, 6.08001	8.53829, 5.93975, 4.71063	11.24094,	3	sox8	10894
GL172659.1	196029	196914	196703, 196777, 196590	9, 10, 4	7.97705, 8.04981, 3.97874	5.637, 5.53254, 3.45022	5.36577, 5	3	sox8	9049
GL172659.1	395215	396066	395722, 395713, 395568	9, 9, 7	8.0679, 6.49199, 8.35502	5.69219, 4.70689, 5.9108	5.45, 4.133	3	sstr5	50103
GL172659.1	415669	416283	415995, 415959, 415896	5, 6, 6	4.47183, 4.62266, 7.29403	3.78598, 3.8507, 5.30581	2.26935, 2	3	sstr5	29886
GL172659.1	511500	512095	512047, 512009, 512060	7, 14, 11	6.69449, 13.26509, 13.1143	5.04797, 7.95332, 8.32282	4.1953, 10	3	NA	24678
GL172659.1	601996	602885	602421, 602473, 602209	10, 11, 3	10.13706, 10.26356, 3.17999	6.7935, 6.72731, 2.89245	7.36489, 7	3	c1qtnf1	3255
GL172659.1	651035	651538	651489, 651520, 651509	44, 47, 43	53.23508, 55.99157, 56.54957	20.57812, 20.76152, 22.99939	49.33706,	3	c1qtnf1	52294
GL172659.1	2108674	2109698	2109435, 2108973, 2108929	12, 15, 9	10.99193, 11.45083, 9.8849	6.98928, 6.60415, 6.72947	8.17226, 8	3	alcam	244425
GL172659.1	2119973	2122017	2120670, 2120643, 2120255, 2121329	10, 18, 11, 7	8.16254, 16.57986, 11.31465, 6.43637	5.60312, 8.94309, 7.3472, 4.89813	5.53763, 1	3	alcam	255724
GL172659.1	2119979	2122017	2121336, 2120643, 2120255, 2121329	13, 18, 11, 7	11.55805, 16.57986, 11.31465, 6.43637	7.13125, 8.94309, 7.3472, 4.89813	8.70034, 1	3	alcam	255730
GL172659.1	2119979	2122017	2121943, 2120643, 2120255, 2121329	11, 18, 11, 7	7.25747, 16.57986, 11.31465, 6.43637	4.85715, 8.94309, 7.3472, 4.89813	4.69225, 1	3	alcam	255730
GL172659.1	2126485	2127442	2127260, 2126624, 2126618, 2127209	22, 30, 23, 10	11.78278, 16.84369, 17.28136, 5.13389	5.31403, 6.16349, 7.93387, 3.69939	8.91409, 1	3	alcam	262236
GL172659.1	2128723	2131175	2129603, 2129652, 2130258	26, 31, 20	15.62684, 17.71967, 14.64139	6.3776, 6.35155, 7.24182	12.56527,	3	alcam	264474
GL172659.1	2137327	2139577	2138756, 2137220, 2139050, 2138660	16, 10, 19, 20	10.92563, 4.64703, 12.04257, 17.13531	6.05848, 3.40341, 6.0291, 8.69989	8.11049, 2	3	alcam	273078
GL172659.1	2141788	2142706	2141922, 2142552, 2141978	8, 12, 12	4.4894, 6.88989, 9.31002	3.55621, 4.45688, 5.94816	2.28575, 4	3	alcam	277539
GL172659.1	4034182	4035240	4034772, 4034802, 4035012	4, 8, 11	3.57366, 7.07159, 11.86426	3.22499, 5.20073, 7.65794	1.59706, 4	3	gtf2i	2661
GL172660.1	167159	167950	167556, 167458, 167830	12, 20, 8	12.60209, 18.1164, 9.05167	7.9443, 9.28853, 6.30653	9.68421, 1	3	NA	41777
GL172660.1	249372	249712	249708, 249576, 249674	14, 9, 10	15.49091, 6.9998, 11.24131	9.26387, 5.02958, 7.40241	12.43497,	3	NA	1085
GL172660.1	355693	356184	355882, 355978, 356060	11, 15, 11	10.60582, 13.42325, 11.79996	6.93244, 7.81299, 7.62212	7.80831, 1	3	NA	0
GL172660.1	373074	373556	373499, 373499, 373496	22, 27, 10	24.27679, 28.17926, 12.42918	12.36565, 12.97124, 7.99682	20.91531,	3	NA	10665
GL172660.1	421112	421387	421336, 421327, 421354	14, 17, 7	16.7649, 18.56115, 8.646	9.96273, 10.40145, 6.04229	13.6543, 1	3	NA	7952
GL172660.1	554063	554849	554624, 554187, 554181	36, 39, 35	39.90606, 42.49015, 44.13445	16.3414, 16.56863, 19.26254	36.20503,	3	NA	1042
GL172660.1	578059	579001	578108, 578627, 578610	19, 34, 16	21.21506, 38.81665, 18.52308	11.44039, 16.54044, 10.59969	17.94655,	3	NA	21831
GL172660.1	636375	636653	636604, 636466, 636568	8, 6, 5	6.48409, 4.93312, 5.50079	4.83873, 4.04501, 4.33868	4.00135, 2	3	NA	197
GL172660.1	649485	651257	650111, 650132, 649986, 651006	20, 36, 15, 9	17.47467, 33.15791, 14.76444, 7.7212	8.90288, 12.50239, 8.62913, 5.48026	14.33719,	3	NA	1719
GL172660.1	649485	651257	650957, 650132, 649986, 651006	21, 36, 15, 9	18.91592, 33.15791, 14.76444, 7.7212	9.46486, 12.50239, 8.62913, 5.48026	15.72399,	3	NA	1719
GL172660.1	1099444	1100065	1099471, 1099497, 1099462	91, 144, 59	120.19728, 180.63525, 78.31078	33.29735, 34.77908, 27.6393	115.64767	3	NA	14292
GL172660.1	1202599	1203016	1202982, 1202990, 1202982	25, 27, 21	30.84439, 32.14959, 27.07785	15.24754, 15.33095, 14.2403	27.31799,	3	NA	1649
GL172660.1	1427614	1428678	1428083, 1428124, 1428092	10, 17, 11	9.18236, 13.5057, 11.99553	6.23091, 7.35211, 7.73059	6.47811, 1	3	or10g4	20322
GL172660.1	1434548	1435486	1434903, 1435122, 1435098	34, 43, 18	34.27021, 37.39883, 16.51463	13.87685, 12.2859, 8.90345	30.67141,	3	NA	13616
GL172660.1	1455535	1456018	1455880, 1455880, 1455884	14, 14, 6	15.57853, 11.11865, 7.29403	9.31333, 6.63466, 5.30581	12.519, 8.4	3	NA	5472
GL172660.1	1498117	1499348	1498414, 1498725, 1498429	11, 19, 10	10.16451, 17.89367, 11.31133	6.66744, 9.45168, 7.43928	7.39114, 1	3	NA	18222
GL172660.1	1503448	1504823	1504602, 1503847, 1503647	7, 7, 4	6.64707, 5.33631, 4.46488	5.02073, 4.22269, 3.69425	4.15135, 3	3	NA	12747
GL172660.1	1527764	1528580	1527955, 1527953, 1528113	8, 9, 6	8.42257, 7.43399, 7.29403	5.97763, 5.30221, 5.30581	5.76336, 4	3	NA	83
GL172660.1	1543830	1544123	1544019, 1544066, 1544047	9, 17, 9	8.82885, 14.31774, 8.43009	6.14328, 7.84337, 5.90952	6.14693, 1	3	NA	16149
GL172660.1	1709474	1709875	1709620, 1709765, 1709537	5, 9, 3	4.14595, 6.58184, 3.17999	3.59111, 4.7642, 2.89245	1.98639, 4	3	XB-GENE-1010646	11317
GL172660.1	1764458	1764804	1764719, 1764761, 1764799	8, 5, 6	7.99469, 3.85883, 7.29403	5.74128, 3.41255, 5.30581	5.38155, 1	3	slc36a1	0
GL172660.1	1913162	1914618	1914122, 1914506, 1913468	9, 11, 5	9.00096, 9.57292, 5.69847	6.24222, 6.30569, 4.4331	6.30808, 6	3	NA	15455
GL172660.1	2465917	2466350	2466075, 2466032, 2466152	7, 7, 3	5.52352, 4.30364, 3.44998	4.34124, 3.55187, 3.02115	3.15454, 2	3	havcr1	61223
GL172660.1	2885682	2886301	2886075, 2886064, 2885832	8, 10, 6	8.22568, 8.36423, 7.29403	5.8701, 5.72892, 5.30581	5.59681, 5	3	tnni1.2	39908
GL172660.1	2990606	2991266	2991113, 2991134, 2991144	8, 11, 5	8.42257, 9.43766, 5.9506	5.97763, 6.22212, 4.54784	5.76336, 6	3	cd9	9233
GL172660.1	3628026	3629284	3628393, 3628497, 3628498	10, 15, 7	9.44155, 12.58265, 7.40077	6.38646, 7.29694, 5.43746	6.72156, 9	3	kcna6	29409
GL172660.1	3690261	3690727	3690698, 3690676, 3690584	22, 23, 8	25.5108, 22.38211, 9.1831	13.09204, 10.90305, 6.37229	22.11518,	3	ndufa9	11118
GL172660.1	3708232	3708760	3708395, 3708452, 3708493	7, 7, 6	5.65516, 5.65148, 6.76586	4.42382, 4.42152, 5.06179	3.27228, 3	3	ndufa9	0
GL172660.1	3999244	3999748	3999576, 3999546, 3999588	7, 9, 4	6.42049, 8.21099, 4.67325	4.8888, 5.77858, 3.78987	3.94161, 5	3	hand1	5521
GL172660.1	4095407	4096556	4096248, 4095599, 4096071	11, 13, 7	10.84329, 11.2197, 7.34973	7.073, 6.92276, 5.41038	8.0324, 8.5	3	larp1	30726
GL172660.1	4231453	4231911	4231909, 4231741, 4231784	25, 43, 20	25.73051, 43.38306, 23.54048	12.22615, 15.39967, 12.51895	22.32921,	3	canx	4953
GL172661.1	72246	72908	72873, 72881, 72842	50, 69, 38	59.17498, 79.58215, 45.88055	21.15843, 23.18595, 18.99656	55.19719,	3	calm2	86054
GL172661.1	364656	364977	364699, 364682, 364680	30, 32, 20	29.87852, 29.09961, 19.49316	12.86101, 11.68917, 10.12002	26.37381,	3	ttc7a	5851
GL172661.1	367452	367798	367770, 367760, 367764	19, 35, 36	17.0405, 32.67583, 42.42966	8.935, 12.59973, 17.83051	13.92003,	3	ttc7a	3030
GL172661.1	383348	383781	383422, 383412, 383428	12, 20, 9	12.74986, 21.01248, 10.72164	8.02869, 11.03495, 7.15493	9.82533, 1	3	ttc7a	0
GL172661.1	799510	800712	799909, 799828, 799637, 799986	16, 23, 5, 4	15.03785, 18.33737, 5.40789, 4.22496	8.54705, 8.52695, 4.29296, 3.57747	12.00487,	3	epas1	0
GL172661.1	878591	878804	878777, 878757, 878762	21, 24, 15	25.38534, 27.39167, 19.40672	13.30436, 13.56183, 11.21161	21.99339,	3	prkce	34677
GL172661.1	995515	996142	995761, 995742, 995888	12, 14, 7	12.3187, 14.45897, 8.69489	7.78073, 8.66788, 6.06379	9.4231, 11	3	prkce	0
GL172661.1	1029724	1030277	1029847, 1029855, 1030252	7, 11, 13	6.50909, 9.71235, 16.0859	4.94073, 6.39153, 9.70995	4.02371, 7	3	prkce	0
GL172661.1	1101975	1102706	1102552, 1102237, 1102670	14, 14, 5	13.14827, 10.43306, 4.64121	7.88233, 6.21324, 3.88372	10.20482,	3	prkce	0
GL172661.1	1147698	1148401	1148322, 1148391, 1148233	26, 26, 17	30.99137, 31.59995, 21.64669	15.00173, 15.35647, 12.11304	27.46178,	3	srbd1	0
GL172661.1	1610950	1611333	1611305, 1611302, 1611288	7, 15, 8	7.1696, 15.80067, 8.09568	5.31345, 9.24574, 5.79794	4.61046, 1	3	camkmt	45444
GL172661.1	1770053	1770371	1770283, 1770317, 1770302	8, 17, 10	7.67464, 16.63185, 11.6791	5.55832, 9.25145, 7.62925	5.08299, 1	3	camkmt	0
GL172661.1	1940289	1941272	1941082, 1940658, 1940558	6, 11, 6	5.96715, 10.60895, 7.29403	4.64927, 6.9343, 5.30581	3.52158, 7	3	ppm1b	5926
GL172661.1	2286252	2286471	2286291, 2286277, 2286283	9, 19, 10	9.36986, 19.04123, 11.45478	6.44997, 10.14611, 7.51412	6.65425, 1	3	thada	22193
GL172661.1	2305489	2305959	2305693, 2305583, 2305621	17, 33, 21	18.46847, 35.96944, 28.28935	10.34725, 15.26871, 14.87856	15.29249,	3	thada	2705
GL172661.1	2659070	2660046	2659365, 2659501, 2659430	8, 15, 8	7.28955, 12.13791, 9.46129	5.33177, 7.02404, 6.50801	4.72176, 9	3	NA	25220
GL172661.1	2868221	2868574	2868321, 2868312, 2868322	6, 12, 4	5.95712, 9.86753, 4.67325	4.64377, 6.29567, 3.78987	3.52158, 7	3	NA	181835
GL172661.1	2873104	2873825	2873285, 2873160, 2873322	6, 6, 3	5.96715, 4.13436, 3.47371	4.64927, 3.53586, 3.03189	3.52158, 2	3	NA	186718
GL172661.1	2890355	2891061	2890618, 2890582, 2890519	26, 24, 18	24.73166, 19.33661, 16.87729	11.28203, 8.80231, 9.12383	21.3576, 1	3	NA	203969
GL172661.1	2929906	2930762	2930603, 2930603, 2930327	18, 16, 8	18.45086, 13.83763, 8.74279	10.07535, 7.81372, 6.14792	15.27601,	3	NA	243520
GL172661.1	3055044	3055357	3055322, 3055324, 3055353	25, 46, 23	30.06855, 56.32277, 33.57074	14.7997, 21.43477, 17.17183	26.55945,	3	haao	237467
GL172661.1	3073407	3073713	3073463, 3073462, 3073444	5, 19, 5	4.69353, 19.04123, 5.59755	3.9134, 10.14611, 4.38538	2.45909, 1	3	haao	219111
GL172661.1	3255255	3255473	3255355, 3255345, 3255406	10, 9, 5	11.06011, 8.21099, 5.9506	7.306, 5.77858, 4.54784	8.22712, 5	3	haao	37351
GL172661.1	3381799	3382413	3381829, 3381829, 3381835	76, 118, 66	94.01394, 147.36848, 89.58881	27.35752, 32.53129, 30.34914	89.6763, 1	3	haao	22810
GL172661.1	3530653	3530987	3530913, 3530960, 3530868	24, 30, 22	25.10709, 31.58953, 26.37531	12.19994, 13.86841, 13.5919	21.72339,	3	KCNG3	17170
GL172661.1	3610394	3611229	3611029, 3610431, 3610450	13, 23, 12	12.32133, 22.88804, 13.44151	7.59717, 11.20666, 8.41472	9.42482, 1	3	NA	18776
GL172661.1	3627921	3628147	3628118, 3628124, 3628111	15, 12, 8	17.48779, 10.98912, 9.8421	10.20672, 6.98757, 6.68602	14.34959,	3	NA	1858
GL172661.1	3637806	3637995	3637993, 3637880, 3637965	8, 13, 9	6.34412, 10.04078, 9.41119	4.75087, 6.19235, 6.47287	3.871, 7.43	3	eml4	2021
GL172661.1	3735952	3736611	3736236, 3736235, 3736258	9, 17, 7	7.43495, 15.29522, 7.05993	5.30281, 8.43856, 5.25341	4.85899, 1	3	eml4	0
GL172661.1	3839988	3840639	3840479, 3840381, 3840484	11, 8, 9	9.90875, 7.07159, 10.01275	6.51186, 5.20073, 6.79683	7.14919, 4	3	eml4	85491
GL172661.1	3902698	3903108	3903048, 3902913, 3903052	18, 34, 12	19.43049, 34.40738, 13.7434	10.65863, 13.95583, 8.57827	16.22032,	3	eml4	148201
GL172661.1	3996074	3996524	3996084, 3996078, 3996085	50, 72, 52	63.86559, 89.48556, 66.35869	23.88547, 26.99847, 24.33217	59.83467,	3	pkdcc.2	66996
GL172661.1	4117910	4118778	4118453, 4118240, 4118354	12, 7, 7	11.79509, 5.97725, 7.72582	7.47301, 4.62287, 5.6058	8.92495, 3	3	pkdcc.2	11188
GL172661.1	4236825	4237582	4237078, 4237284, 4237277	5, 10, 5	4.69353, 8.74956, 5.80393	3.9134, 5.96721, 4.48186	2.45909, 6	3	pkdcc.2	130103
GL172661.1	4277120	4278499	4277673, 4277878, 4278019	6, 12, 8	5.24248, 9.0625, 7.76694	4.2332, 5.7937, 5.6116	2.89962, 6	3	NA	127968
GL172662.1	492920	493392	493029, 493031, 493026	855, 931, 714	966.38495, 1054.68726, 838.17157	33.97963, 34.31174, 37.13414	959.68481	3	cmc1	58933
GL172662.1	2178654	2179098	2178814, 2178915, 2179053	11, 14, 11	9.43668, 11.28333, 10.01445	6.22151, 6.73619, 6.57631	6.7178, 8.6	3	nkiras1	0
GL172662.1	2181072	2181690	2181450, 2181208, 2181366	9, 11, 9	7.24594, 8.55421, 8.43009	5.18459, 5.67101, 5.90952	4.68113, 6	3	nkiras1	0
GL172662.1	3213649	3213942	3213911, 3213787, 3213836	13, 17, 10	13.20994, 13.72161, 12.33934	8.12823, 7.48229, 7.95424	10.26285,	3	NA	4421
GL172662.1	3866347	3866804	3866627, 3866753, 3866535	8, 7, 3	7.57423, 5.52121, 3.41347	5.4999, 4.33979, 3.00444	4.98865, 3	3	satb1	147579
GL172662.1	4215435	4216059	4215964, 4216026, 4216007	15, 19, 18	17.18944, 18.54823, 23.23937	10.04146, 9.84817, 12.78599	14.06311,	3	tbc1d5	2864
GL172663.1	76393	77344	76588, 76863, 76925	15, 19, 12	15.49092, 18.02027, 14.23156	9.06314, 9.5284, 8.83588	12.43497,	3	NA	3217
GL172663.1	161104	161684	161387, 161430, 161568	16, 12, 8	16.68158, 9.62333, 9.05167	9.53667, 6.14359, 6.30653	13.57813,	3	NA	87928
GL172663.1	190188	190646	190348, 190451, 190247	7, 8, 4	6.33443, 6.51779, 4.46488	4.83795, 4.8598, 3.69425	3.86259, 4	3	ankfn1	61717
GL172663.1	285491	286285	286051, 285831, 285819	10, 11, 6	8.70772, 9.57292, 6.35308	5.94148, 6.30569, 4.85498	6.03286, 6	3	ankfn1	7183
GL172663.1	299651	300012	299800, 299671, 299904	4, 7, 6	3.42565, 4.97304, 5.793	3.13796, 3.98948, 4.55271	1.48489, 2	3	ankfn1	21343
GL172663.1	1082172	1083101	1082825, 1082859, 1082697	26, 20, 9	29.61147, 16.73193, 10.28145	14.18819, 8.45971, 6.93568	26.11284,	3	plk1	2914
GL172663.1	1680359	1680888	1680766, 1680716, 1680735	10, 18, 6	9.54985, 14.06285, 6.50067	6.45088, 7.42469, 4.93052	6.82341, 1	3	TRAP1	1440
GL172663.1	1934920	1935915	1935653, 1934923, 1935087	13, 13, 5	13.00701, 11.31501, 4.84526	8.00827, 6.98159, 3.99806	10.0703, 8	3	glis2	2446
GL172663.1	2085331	2085855	2085457, 2085795, 2085811	7, 25, 11	6.79162, 24.88547, 11.6739	5.10336, 11.72091, 7.55148	4.28406, 2	3	c7orf26	2006
GL172663.1	2094405	2095097	2094477, 2094790, 2094948	8, 13, 7	8.42257, 11.71436, 8.15288	5.97763, 7.22722, 5.81587	5.76336, 9	3	NA	4443
GL172663.1	2436552	2436862	2436626, 2436731, 2436738	10, 16, 3	9.38842, 16.51912, 3.47371	6.35473, 9.44018, 3.03189	6.67167, 1	3	aqp8	11509
GL172663.1	2573791	2574563	2574266, 2574194, 2574167	13, 7, 5	12.62236, 5.75319, 5.9506	7.77868, 4.48487, 4.54784	9.70369, 3	3	prkcb	0
GL172663.1	2642724	2643171	2642952, 2642867, 2642831	11, 12, 5	11.29029, 11.25867, 4.84526	7.3332, 7.1513, 3.99806	8.44541, 8	3	cacng3	2311
GL172663.1	2681718	2682576	2681941, 2681794, 2681987	7, 7, 5	5.97522, 5.16276, 5.50079	4.62162, 4.11176, 4.33868	3.52869, 2	3	cacng3	0
GL172663.1	2777827	2778417	2777944, 2778155, 2777952	8, 17, 8	8.42257, 16.17826, 9.1831	5.97763, 8.97632, 6.37229	5.76336, 1	3	caskin1	24928
GL172663.1	2787638	2788034	2787761, 2787887, 2787834	20, 44, 14	19.42489, 47.05788, 16.2088	10.07876, 17.01824, 9.66324	16.21503,	3	caskin1	34739
GL172663.1	2803703	2804476	2804084, 2804085, 2804083	14, 17, 7	14.38232, 17.11659, 6.55126	8.62271, 9.54398, 4.96529	11.37721,	3	NA	46592
GL172663.1	2838768	2839509	2839101, 2839112, 2839182	15, 24, 9	13.57755, 24.22927, 10.96118	7.90752, 11.6736, 7.26984	10.61316,	3	NA	11559
GL172663.1	2866720	2867117	2867035, 2867000, 2866991	6, 16, 7	5.72604, 16.51912, 7.84208	4.51498, 9.44018, 5.66426	3.33547, 1	3	NA	0
GL172663.1	2947401	2947795	2947557, 2947589, 2947503	6, 11, 4	5.55471, 9.8562, 4.67325	4.41698, 6.47973, 3.78987	3.18192, 7	3	grid2ip	3874
GL172663.1	3244086	3244818	3244720, 3244772, 3244180	7, 6, 4	6.99564, 4.4843, 4.30163	5.21786, 3.76254, 3.61556	4.47307, 2	3	rab26	0
GL172663.1	3859122	3860257	3859355, 3859752, 3859722	15, 21, 8	14.33586, 20.39593, 8.6841	8.36999, 10.35516, 6.11715	11.33286,	3	epn2	23953
GL172663.1	3904550	3905553	3904760, 3905065, 3905232	10, 7, 3	7.61309, 4.08852, 3.44998	5.25765, 3.40999, 3.02115	5.02606, 2	3	b9d1	0
GL172663.1	4067847	4068326	4067957, 4067907, 4067883	9, 10, 10	9.06007, 8.9331, 12.52085	6.27591, 6.07959, 8.03986	6.36394, 6	3	cacna1h	0
GL172663.1	4235924	4236641	4236043, 4236474, 4236223	9, 13, 8	7.93244, 11.76644, 9.46129	5.60981, 7.25915, 6.50801	5.32435, 9	3	cacna1h	137180
GL172663.1	4251205	4251830	4251484, 4251584, 4251572	5, 9, 8	4.82177, 8.21099, 9.05167	3.98509, 5.77858, 6.30653	2.51418, 5	3	cacna1h	152461
GL172663.1	4285762	4287173	4286736, 4286734, 4286735	29, 48, 29	34.75011, 55.51857, 37.78184	16.1291, 20.01407, 17.88422	31.14222,	3	NA	126352
GL172664.1	21096	21394	21388, 21289, 21339	13, 11, 5	14.26982, 10.05535, 5.45388	8.73911, 6.6012, 4.3157	11.26966,	3	map4	0
GL172664.1	191687	192118	191747, 191714, 191766	32, 29, 28	37.15584, 30.62869, 32.97765	16.37888, 13.68177, 15.45615	33.5013, 2	3	NA	14952
GL172664.1	298733	299217	299054, 299057, 298896	4, 10, 4	3.74195, 9.39128, 4.67325	3.32091, 6.35644, 3.78987	1.6222, 6.8	3	NA	8506
GL172664.1	344432	344760	344641, 344546, 344499	9, 10, 6	7.80169, 6.96485, 6.45058	5.52977, 4.84715, 4.90508	5.20141, 4	3	NA	54205
GL172664.1	465966	466801	466392, 466352, 466391	34, 48, 29	33.16388, 47.68304, 33.78949	13.24461, 15.69858, 15.56161	29.58718,	3	csrnp1	57463
GL172664.1	478593	479118	478842, 478868, 478788	6, 6, 6	4.62553, 4.56637, 5.75442	3.85252, 3.81494, 4.53101	2.40124, 2	3	csrnp1	45146
GL172664.1	622895	623345	623317, 623318, 623294	32, 49, 37	33.11712, 53.32421, 44.42432	13.99024, 18.31895, 18.57609	29.54119,	3	wdr48	7759
GL172664.1	627856	628463	627960, 627964, 628097	11, 16, 8	7.86853, 10.56042, 6.1856	5.2402, 5.8453, 4.65072	5.26511, 7	3	wdr48	12720
GL172664.1	635150	635438	635181, 635192, 635254	9, 5, 6	9.00096, 3.19541, 6.12365	6.24222, 2.97945, 4.73409	6.30808, 1	3	wdr48	20014
GL172664.1	720759	721155	720879, 720879, 720903	18, 29, 10	18.52782, 29.11719, 10.30677	10.1215, 12.78747, 6.89026	15.34982,	3	scn5a	0
GL172664.1	827099	827521	827249, 827403, 827120	6, 9, 9	5.47306, 7.24675, 10.87975	4.36955, 5.1851, 7.23113	3.10811, 4	3	scn5a	0
GL172664.1	836479	837835	837314, 837316, 837279	65, 87, 39	64.72508, 85.54505, 37.82658	17.51047, 18.59897, 13.93549	60.68536,	3	scn5a	0
GL172664.1	913595	914394	913919, 913951, 914220	18, 21, 7	20.25794, 20.05836, 7.96278	11.14243, 10.15162, 5.72394	17.01944,	3	exog	14201
GL172664.1	930457	931140	930893, 930892, 930886	28, 50, 12	31.18742, 53.36781, 16.0427	14.39356, 17.903, 9.71071	27.6528, 4	3	exog	0
GL172664.1	964725	965134	964983, 964995, 965004	8, 12, 9	7.28955, 10.30411, 10.28145	5.33177, 6.56659, 6.93568	4.72176, 7	3	acvr2b	0

GL172664.1	1019410	1020366	1019789, 1019772, 1019786	45, 78, 35	48.22384, 88.8684, 43.50117	17.2389, 23.69325, 18.8904	44.39704,	3	acvr2b	41892
GL172664.1	1029222	1029863	1029650, 1029421, 1029657	9, 10, 4	8.77314, 8.4887, 4.67325	6.111, 5.80621, 3.78987	6.09355, 5	3	acvr2b	51704
GL172664.1	1102978	1103847	1103571, 1103518, 1103158	19, 16, 6	17.56914, 14.14493, 6.25896	9.25502, 8.00171, 4.80589	14.42794,	3	acvr2b	125460
GL172664.1	1281409	1281970	1281680, 1281665, 1281422	4, 7, 3	3.57366, 5.16276, 3.47371	3.22499, 4.11176, 3.03189	1.59706, 2	3	NA	18592
GL172664.1	1300632	1301148	1300875, 1300848, 1300793	7, 10, 3	5.901, 8.20397, 3.47371	4.57615, 5.62902, 3.03189	3.49615, 5	3	NA	0
GL172664.1	1538149	1538666	1538564, 1538432, 1538432	14, 15, 9	11.14378, 13.69427, 8.60738	6.65015, 7.97895, 6.01421	8.30717, 1	3	rheb	7527
GL172664.1	1555716	1556080	1555825, 1555995, 1555939	5, 8, 5	4.20732, 6.44699, 4.60894	3.62846, 4.8155, 3.8653	2.04024, 4	3	prkag1	2811
GL172664.1	1622209	1622815	1622448, 1622450, 1622454	36, 40, 23	38.20934, 40.41951, 26.79607	15.35024, 14.9748, 13.53497	34.53662,	3	prkag1	0
GL172664.1	1784796	1785926	1784963, 1784988, 1785021	15, 13, 11	16.36908, 12.03475, 14.13736	9.57629, 7.4231, 8.81822	13.27778,	3	kmt2c	18302
GL172664.1	1934183	1935071	1934607, 1934297, 1934876	8, 8, 8	6.41335, 5.21379, 8.74279	4.7944, 4.02631, 6.14792	3.93678, 2	3	xrcc2	49256
GL172664.1	1950648	1951295	1950677, 1950694, 1950675	9, 14, 9	9.72082, 14.45897, 11.58206	6.64182, 8.66788, 7.55286	6.97155, 1	3	xrcc2	33032
GL172664.1	1990886	1992026	1991613, 1991711, 1991229	12, 16, 5	11.79509, 13.83763, 4.64121	7.47301, 7.81372, 3.88372	8.92495, 1	3	xrcc2	1013
GL172664.1	2002639	2003000	2002795, 2002795, 2002795	14, 25, 18	12.33072, 22.18474, 18.60267	7.38193, 10.12639, 10.16634	9.43363, 1	3	xrcc2	12766
GL172664.1	2032157	2033122	2032265, 2032576, 2032555	15, 14, 4	15.41679, 14.29802, 4.64325	9.0192, 8.57291, 3.77643	12.36344,	3	xrcc2	42284
GL172664.1	2081845	2082391	2082188, 2081996, 2082155	6, 10, 9	5.55471, 8.36423, 10.49552	4.41698, 5.72892, 7.0436	3.18192, 5	3	xrcc2	91972
GL172664.1	2096030	2096386	2096039, 2096063, 2096066	14, 23, 7	16.13274, 23.18973, 8.35502	9.62159, 11.38786, 5.9108	13.05194,	3	xrcc2	106157
GL172664.1	2169571	2169827	2169600, 2169609, 2169588	37, 64, 42	40.43646, 71.82964, 54.42937	16.22831, 21.46923, 22.2201	36.72728,	3	xrcc2	179698
GL172664.1	2363874	2364261	2363904, 2363945, 2363933	16, 16, 8	16.91852, 14.09269, 8.9249	9.67673, 7.96975, 6.24211	13.80145,	3	xrcc2	374001
GL172664.1	2680334	2681071	2680341, 2680903, 2680832	8, 18, 10	6.8227, 15.28549, 10.30677	5.04883, 8.15798, 6.89026	4.31393, 1	3	dpp6	325087
GL172664.1	2684497	2685537	2684581, 2685043, 2684641	15, 15, 11	13.30906, 11.80459, 10.82241	7.74297, 6.81998, 7.0607	10.35786,	3	dpp6	320621
GL172664.1	2725976	2726552	2725988, 2725985, 2726026	16, 37, 18	16.37806, 39.98539, 23.23937	9.35612, 15.96556, 12.78599	13.28648,	3	dpp6	279606
GL172664.1	2885553	2885974	2885583, 2885834, 2885831	9, 6, 4	8.0679, 4.93312, 4.22496	5.69219, 4.04501, 3.57747	5.45, 2.71€	3	dpp6	120184
GL172664.1	2909413	2910102	2910049, 2910054, 2910062	12, 19, 14	12.74986, 18.96889, 16.82614	8.02869, 10.10245, 9.99514	9.82533, 1	3	dpp6	96056
GL172664.1	2990024	2990617	2990575, 2990589, 2990533	8, 15, 6	6.94372, 12.7702, 5.8719	5.12297, 7.41214, 4.59674	4.42528, 1	3	dpp6	15541
GL172664.1	3041761	3042633	3041885, 3041945, 3041805	29, 28, 27	37.27069, 31.72212, 36.77732	17.59332, 14.71186, 17.95288	33.61411,	3	dpp6	0
GL172664.1	3171341	3171817	3171780, 3171776, 3171782	19, 28, 8	22.80552, 31.93789, 9.60885	12.35182, 14.84014, 6.57807	19.4875, 2	3	dpp6	0
GL172664.1	3291352	3291735	3291666, 3291691, 3291701	26, 38, 20	28.09964, 37.29169, 23.16452	13.28747, 14.0261, 12.30292	24.63753,	3	paxip1	0
GL172664.1	3295238	3295899	3295333, 3295335, 3295616	9, 26, 7	6.63709, 22.06964, 5.42746	4.79941, 9.74022, 4.28057	4.14229, 1	3	paxip1	2469
GL172664.1	3371928	3372394	3371987, 3372175, 3372299	5, 6, 3	4.20732, 4.93312, 3.47371	3.62846, 4.04501, 3.03189	2.04024, 2	3	htr5a	63368
GL172664.1	3402818	3403352	3403070, 3403244, 3403304	12, 11, 5	13.05874, 10.60895, 5.80393	8.20296, 6.9343, 4.48186	10.11961,	3	htr5a	32410
GL172664.1	3510017	3510447	3510047, 3510077, 3510167	9, 13, 6	9.50098, 13.1283, 7.29403	6.52233, 8.08007, 5.30581	6.77745, 1	3	htr5a	71902
GL172664.1	3521796	3522573	3521950, 3521970, 3522095	12, 19, 9	13.73771, 20.82621, 11.64359	8.57523, 11.21218, 7.57973	10.76705,	3	htr5a	83681
GL172664.1	3619556	3619842	3619697, 3619563, 3619584	8, 9, 3	7.77807, 7.92635, 3.37781	5.618, 5.60609, 2.98791	5.17956, 5	3	insig1	84863
GL172664.1	3627555	3628178	3627655, 3627685, 3628004	11, 10, 4	10.3251, 8.57382, 3.76128	6.7644, 5.8589, 3.33171	7.54325, 6	3	insig1	76527
GL172664.1	3636062	3636529	3636081, 3636082, 3636161	6, 8, 6	5.3939, 7.07159, 7.29403	4.32314, 5.20073, 5.30581	3.0374, 4.€	3	insig1	68176
GL172664.1	3683202	3683715	3683685, 3683689, 3683467	8, 15, 13	8.42257, 15.80067, 14.39632	5.97763, 9.24574, 8.81006	5.76336, 1	3	insig1	20990
GL172664.1	3712608	3713086	3713043, 3713044, 3713048	42, 68, 37	43.83785, 71.40109, 47.40503	16.06285, 19.49843, 20.33268	40.07568,	3	insig1	0
GL172664.1	3754506	3754986	3754937, 3754959, 3754936	34, 58, 34	38.34463, 63.95937, 42.6431	16.261, 20.05168, 18.80155	34.6694, 6	3	insig1	39378
GL172664.1	3779988	3780747	3780703, 3780659, 3780557	17, 23, 11	18.46847, 27.34036, 12.86886	10.34725, 13.85155, 8.19722	15.29249,	3	insig1	64860
GL172664.1	3837026	3837429	3837329, 3837355, 3837190	6, 10, 4	5.16999, 8.70492, 4.34116	4.18963, 5.93975, 3.63492	2.83325, 6	3	en2	18446
GL172664.1	4162885	4163183	4163136, 4163148, 4163129	72, 96, 62	88.73284, 112.33949, 81.68712	26.58622, 26.70368, 27.97734	84.45057,	3	shh	87040
GL172664.1	4309406	4309904	4309524, 4309680, 4309680	23, 28, 14	21.41593, 28.72043, 17.83243	10.3253, 12.92441, 10.51088	18.14036,	3	rnf32	7157
GL172665.1	387835	388989	388447, 388548, 388238	14, 30, 8	12.13106, 28.52218, 8.56955	7.25904, 12.07378, 6.05652	9.24462, 2	3	ca10	0
GL172665.1	1033535	1034470	1033924, 1033936, 1033927	22, 47, 23	21.76329, 43.91459, 25.2841	10.85936, 14.14294, 12.64245	18.47656,	3	rbfox3	233698
GL172665.1	1526368	1527434	1526633, 1526917, 1526468	6, 12, 4	5.3552, 10.16897, 4.46488	4.3003, 6.4829, 3.69425	3.00253, 7	3	ezh1	0
GL172665.1	1526368	1527539	1527461, 1526917, 1526468	5, 12, 4	4.30277, 10.16897, 4.46488	3.68597, 6.4829, 3.69425	2.12436, 7	3	ezh1	0
GL172665.1	1566515	1567622	1566819, 1566915, 1566949	15, 14, 8	13.20489, 10.80331, 7.67847	7.67906, 6.44051, 5.56054	10.25934,	3	ezh1	24530
GL172665.1	1756103	1757172	1756849, 1756556, 1756165	8, 13, 7	6.44853, 8.84052, 6.83761	4.81646, 5.45267, 5.12939	3.96884, 6	3	vps25	52339
GL172665.1	1757642	1758182	1758097, 1757782, 1758047	6, 12, 6	4.5407, 8.46754, 5.75442	3.79858, 5.42308, 4.53101	2.33, 5.952	3	vps25	51329
GL172665.1	2193334	2194515	2193502, 2194043, 2194137	5, 17, 13	4.47183, 15.13134, 15.34872	3.78598, 8.33865, 9.3288	2.26935, 1	3	becn1	0
GL172665.1	2193536	2194515	2194202, 2194043, 2194137	5, 17, 13	4.54329, 15.13134, 15.34872	3.82752, 8.33865, 9.3288	2.33, 12.27	3	becn1	0
GL172665.1	2608209	2609988	2609115, 2609476, 2608910, 2609803	9, 14, 8, 4	9.72082, 11.76301, 10.1465, 3.56693	6.64182, 7.03214, 6.82176, 3.22108	6.97155, 9	3	NA	46753
GL172665.1	2614118	2615544	2614329, 2614974, 2614347	7, 34, 15	4.62343, 32.56081, 15.55135	3.76189, 12.90382, 9.09889	2.39977, 2	3	NA	52662
GL172665.1	2614118	2615544	2614928, 2614974, 2614347	22, 34, 15	22.52709, 32.56081, 15.55135	11.31895, 12.90382, 9.09889	19.2175, 2	3	NA	52662
GL172665.1	2622179	2622611	2622462, 2622455, 2622486	4, 9, 6	3.74195, 8.21099, 7.29403	3.32091, 5.77858, 5.30581	1.6222, 5.7	3	NA	60723
GL172665.1	2628957	2630557	2629427, 2629105, 2629219	11, 20, 14	12.43693, 22.84892, 18.52583	7.97018, 12.1201, 10.84669	9.52692, 1	3	NA	67501
GL172665.1	2628957	2630557	2630378, 2629105, 2629219	8, 20, 14	8.42257, 22.84892, 18.52583	5.97763, 12.1201, 10.84669	5.76336, 1	3	NA	67501
GL172665.1	2731661	2733511	2732396, 2732387, 2732322	48, 53, 34	44.25835, 47.2677, 36.78746	13.95544, 13.67895, 15.34228	40.49011,	3	tmc6	57512
GL172665.1	2755927	2758090	2757484, 2757539, 2757544	27, 28, 21	25.89358, 25.00917, 21.58792	11.61639, 10.75932, 11.07347	22.48761,	3	tmc6	32933
GL172665.1	2770373	2771107	2770723, 2770748, 2770762	22, 43, 16	16.78782, 34.06539, 13.95354	7.92944, 10.7068, 7.88462	13.67641,	3	tmc6	19916
GL172665.1	2772731	2774362	2773082, 2774258, 2773871	14, 24, 8	8.04702, 12.8171, 4.55211	4.78808, 5.45064, 3.59685	5.43132, 1	3	tmc6	16661
GL172665.1	2772936	2774362	2774250, 2774258, 2773871	12, 24, 8	5.76445, 12.8171, 4.55211	3.79542, 5.45064, 3.59685	3.37252, 1	3	tmc6	16661
GL172665.1	2778251	2779216	2778877, 2778546, 2778482	16, 9, 12	14.45741, 4.91347, 10.50755	8.19281, 3.69842, 6.69224	11.44906,	3	tmc6	11807
GL172665.1	3143002	3143337	3143114, 3143190, 3143107	17, 13, 14	14.5428, 7.82921, 14.04784	7.98018, 4.84214, 8.42437	11.53067,	3	NA	0
GL172665.1	3291631	3292422	3292378, 3291979, 3292027	7, 11, 4	5.33678, 7.61035, 3.73221	4.22299, 5.07811, 3.31545	2.98597, 5	3	dhx8	16855
GL172665.1	3318068	3318865	3318620, 3318394, 3318445	9, 12, 9	8.55802, 10.16897, 8.26067	5.98518, 6.4829, 5.80842	5.8909, 7.5	3	dhx8	43292
GL172665.1	3542511	3543467	3543215, 3542340, 3543197, 3543117	10, 7, 15, 4	9.23294, 4.97304, 13.21475, 3.43992	6.26141, 3.98948, 7.68511, 3.14645	6.52626, 2	3	post	45234
GL172666.1	126513	126944	126764, 126656, 126642	11, 14, 9	10.9046, 12.38816, 9.19414	7.10903, 7.41725, 6.35176	8.08987, 9	3	NA	38919
GL172666.1	453011	454494	453730, 453165, 453138	15, 32, 9	13.97429, 31.84316, 11.21577	8.15, 13.24836, 7.38849	10.98644,	3	LRRTM4	130759
GL172666.1	582982	583770	583493, 583230, 583335	16, 20, 11	15.09881, 18.00103, 13.64572	8.58417, 9.21905, 8.58592	12.06341,	3	LRRTM4	260730
GL172666.1	671143	671700	671457, 671295, 671433	6, 10, 5	5.16999, 7.72315, 4.99237	4.18963, 5.32711, 4.0781	2.83325, 5	3	prom2	226179
GL172666.1	714641	715050	714852, 714851, 714950	31, 31, 9	35.75796, 31.85677, 10.21256	15.95063, 13.63997, 6.90044	32.12963,	3	prom2	182829
GL172666.1	763249	763771	763337, 763467, 763645	5, 10, 5	4.69353, 8.4053, 5.9506	3.9134, 5.75445, 4.54784	2.45909, 5	3	prom2	134108
GL172666.1	1501857	1502792	1502366, 1502564, 1502531	15, 16, 10	13.97429, 15.41438, 11.03772	8.15, 8.77591, 7.29398	10.98644,	3	cox5b.1	31897
GL172666.1	1773257	1774653	1774175, 1773763, 1773342, 1774471	10, 16, 5, 6	7.78798, 12.02209, 5.23257, 6.4014	5.36798, 6.71016, 4.20435, 4.8799	5.18872, 9	3	tmem150a	0
GL172666.1	1905156	1905785	1905373, 1905485, 1905374	11, 18, 7	9.61965, 14.90085, 7.40077	6.33449, 7.92604, 5.43746	6.89006, 1	3	psd4	28968
GL172666.1	2163940	2164974	2163998, 2164239, 2164047	11, 9, 7	10.16451, 6.93223, 7.90186	6.66744, 4.98685, 5.69394	7.39114, 4	3	camk2b	0
GL172666.1	2252028	2252956	2252144, 2252071, 2252645	26, 41, 15	26.68953, 43.3289, 15.55135	12.44476, 16.19679, 9.09889	23.26214,	3	pgam2	0
GL172666.1	2265231	2265808	2265505, 2265699, 2265477	8, 8, 7	7.38183, 5.64525, 8.69489	5.38666, 4.30526, 6.06379	4.80856, 3	3	pgam2	8165
GL172666.1	2359758	2360364	2360104, 2359971, 2360078	8, 9, 7	7.42892, 7.3205, 8.21885	5.41453, 5.23132, 5.84717	4.85345, 4	3	ogdh	4971
GL172666.1	2605631	2605957	2605703, 2605667, 2605672	13, 23, 15	15.27101, 24.15665, 17.85348	9.28755, 11.96843, 10.40618	12.22851,	3	AEBP1	0
GL172666.1	2807474	2808001	2807592, 2807931, 2807799	8, 14, 4	8.42257, 11.67268, 4.38152	5.97763, 6.97641, 3.65448	5.76336, 8	3	XB-GENE-5812048	14285
GL172666.1	3282672	3283611	3283326, 3283003, 3283347, 3282860	13, 7, 10, 12	11.50747, 5.68499, 8.98028, 13.90113	7.10015, 4.44244, 6.10835, 8.66246	8.65224, 3	3	slc25a37	0
GL172666.1	3348987	3349233	3349206, 3349196, 3349211	22, 37, 18	29.43504, 50.20938, 26.51834	15.27618, 21.95862, 14.35044	25.94112,	3	nkx2-6	9497
GL172666.1	3506108	3506922	3506612, 3506377, 3506344	57, 69, 50	51.49289, 61.25236, 50.14982	14.28019, 14.63651, 16.1895	47.62032,	3	adam28	0
GL172666.1	3556664	3557327	3557288, 3557094, 3557290	12, 5, 7	12.60209, 3.47798, 6.59055	7.9443, 3.16702, 4.98809	9.68421, 1	3	adam7	0
GL172666.1	3684409	3684861	3684670, 3684665, 3684819	11, 29, 10	10.60582, 27.02533, 10.97191	6.93244, 11.56914, 7.25854	7.80831, 2	3	DOCK5	0
GL172666.1	3689183	3690209	3689806, 3689766, 3689842	9, 10, 5	8.0222, 6.30783, 4.70718	5.66446, 4.43128, 3.9211	5.40727, 3	3	DOCK5	0
GL172666.1	3904875	3905767	3905699, 3905294, 3905519	5, 10, 3	4.27048, 8.04981, 3.47371	3.6666, 5.53254, 3.03189	2.09564, 5	3	ebf2	1529
GL172666.1	3975114	3975896	3975385, 3975346, 3975610	7, 14, 4	6.16939, 12.28739, 3.59344	4.73936, 7.35528, 3.23643	3.70916, 9	3	ppp2r2a	1252
GL172666.1	4029823	4031943	4030661, 4029959, 4030200, 4030924	9, 19, 4, 10	7.84477, 14.62228, 3.70359, 11.24131	5.5562, 7.49126, 3.29934, 7.40241	5.24227, 1	3	NA	0
GL172666.1	4079091	4079594	4079243, 4079515, 4079261	6, 11, 8	4.37941, 8.97396, 9.3897	3.69512, 5.9338, 6.47354	2.19155, 6	3	DPYSL2	24103
GL172667.1	550132	550812	550262, 550517, 550264	9, 11, 7	9.72082, 10.60895, 7.90186	6.64182, 6.9343, 5.69394	6.97155, 7	3	gpc4	100066
GL172667.1	3939174	3939886	3939640, 3939724, 3939669	11, 12, 9	11.02968, 11.1491, 10.01275	7.18222, 7.08489, 6.79683	8.20839, 8	3	NA	0
GL172667.1	4228847	4229408	4229092, 4228972, 4228935	10, 11, 10	8.84451, 8.89461, 10.84317	6.02544, 5.88422, 7.18868	6.16137, 6	3	NA	440
GL172668.1	35368	35725	35375, 35470, 35411	7, 15, 9	6.37715, 14.46023, 9.41119	4.86324, 8.44539, 6.47287	3.90257, 1	3	NA	114273
GL172668.1	333599	333920	333749, 333701, 333862	9, 17, 10	8.25643, 18.56115, 10.97191	5.80588, 10.40145, 7.25854	5.62516, 1	3	bnip3	16476
GL172668.1	518288	518784	518700, 518730, 518758	9, 13, 6	9.56822, 13.14514, 7.29403	6.55912, 8.09002, 5.30581	6.84121, 1	3	NA	30123
GL172668.1	968444	968922	968541, 968471, 968502	20, 16, 14	18.89193, 12.25152, 14.72868	9.7566, 6.84834, 8.82588	15.7007, 9	3	NA	0
GL172668.1	1159155	1159592	1159435, 1159437, 1159394	25, 52, 23	28.77811, 59.35321, 28.47019	14.04349, 20.30539, 14.4983	25.29939,	3	glrx3	52380
GL172668.1	1189576	1190041	1189956, 1190017, 1189979	10, 16, 7	10.39648, 17.16856, 8.21885	6.94096, 9.82359, 5.84717	7.61003, 1	3	glrx3	21931
GL172668.1	1268620	1269050	1268874, 1268897, 1268994	11, 24, 13	10.3251, 25.29108, 16.38883	6.7644, 12.31022, 9.86104	7.54325, 2	3	glrx3	36163
GL172668.1	1287051	1287649	1287351, 1287328, 1287371	25, 40, 21	28.03181, 44.16141, 24.4673	13.60123, 17.10356, 12.77852	24.57135,	3	ebf3	23915
GL172668.1	1652481	1652964	1652713, 1652619, 1652826	11, 21, 4	11.35776, 20.53474, 4.38152	7.37194, 10.43888, 3.65448	8.51017, 1	3	mgmt	0
GL172668.1	1784802	1785412	1784977, 1784985, 1784916	11, 17, 10	12.43693, 16.69909, 11.38247	7.97018, 9.29214, 7.47651	9.52692, 1	3	mgmt	0
GL172668.1	1929572	1930064	1929933, 1929616, 1929837	6, 10, 9	5.63898, 7.68845, 10.14484	4.46544, 5.30522, 6.86555	3.25772, 5	3	mgmt	88248
GL172668.1	2235770	2236400	2235888, 2235937, 2235919	5, 7, 6	4.82177, 5.97725, 7.29403	3.98509, 4.62287, 5.30581	2.51418, 3	3	mgmt	394446
GL172668.1	2324872	2325636	2325264, 2325320, 2325107	14, 15, 4	15.84929, 15.70444, 4.67325	9.46495, 9.18918, 3.78987	12.77923,	3	mki67	451898
GL172668.1	2817044	2817821	2817281, 2817407, 2817384	6, 22, 4	5.31708, 19.65261, 4.67325	4.2777, 9.59468, 3.78987	2.96727, 1	3	ptpre	0
GL172668.1	3338183	3338445	3338419, 3338415, 3338416	10, 23, 12	10.53228, 24.86003, 14.06375	7.01712, 12.38952, 8.74831	7.73927, 2	3	dock1	45963
GL172668.1	3634628	3635087	3635030, 3635057, 3635053	47, 72, 43	58.93346, 90.58953, 60.02965	22.48045, 27.60737, 25.02193	54.95819,	3	adam12	0
GL172668.1	3875696	3876626	3876215, 3876249, 3875993	8, 13, 5	7.83097, 10.04078, 4.77515	5.64832, 6.19235, 3.9592	5.22917, 7	3	adam12	0
GL172668.1	3940513	3940810	3940667, 3940732, 3940761	8, 8, 6	8.42257, 6.30984, 7.29403	5.97763, 4.72927, 5.30581	5.76336, 3	3	fank1	5103
GL172668.1	3950986	3951562	3951210, 3951425, 3951071	9, 10, 4	9.72082, 8.12616, 4.67325	6.64182, 5.58036, 3.78987	6.97155, 5	3	fank1	15576
GL172668.1	4060524	4061226	4060921, 4060997, 4060854	9, 16, 8	9.72082, 13.6893, 8.56955	6.64182, 7.72299, 6.05652	6.97155, 1	3	uros	0
GL172668.1	4094231	4095812	4094426, 4094463, 4095282, 4094437, 4095049	10, 9, 7, 10, 11	10.67246, 8.21099, 5.97725, 11.24131, 12.63525	7.09497, 5.77858, 4.62287, 7.40241, 8.07536	7.87096, 5	3	uros	10104
GL172668.1	4094925	4096313	4095685, 4095282, 4095049	7, 7, 11	6.89193, 5.97725, 12.63525	5.15998, 4.62287, 8.07536	4.37772, 3	3	uros	10798
GL172668.1	4228264	4228518	4228458, 4228465, 4228485	11, 31, 21	12.25244, 35.77021, 26.46843	7.87094, 15.95791, 13.90883	9.35958, 3	3	aldh18a1	28661
GL172669.1	25756	26416	25982, 26145, 26276	5, 9, 4	4.47183, 7.92635, 4.38152	3.78598, 5.60609, 3.65448	2.26935, 5	3	NA	130491
GL172669.1	2521392	2522391	2522109, 2522006, 2521537	6, 8, 7	4.51306, 6.30984, 6.59055	3.78094, 4.72927, 4.98809	2.30616, 3	3	NA	53517
GL172669.1	2556257	2557106	2556647, 2556853, 2556874	17, 35, 21	9.08366, 21.1039, 14.22285	4.83069, 7.00339, 6.7624	6.38684, 1	3	NA	43425
GL172669.1	2610952	2612093	2611640, 2611300, 2611196	25, 39, 15	11.43079, 16.99108, 6.89794	4.6998, 4.97703, 3.99563	8.57959, 1	3	NA	9145
GL172669.1	2615544	2615979	2615695, 2615762, 2615887	19, 25, 10	9.44702, 10.37419, 4.87533	4.68607, 4.27808, 3.54152	6.72699, 7	3	NA	13737
GL172669.1	2622233	2622936	2622500, 2622539, 2622581	18, 34, 9	10.37348, 18.58446, 4.63075	5.33292, 6.18369, 3.51999	7.59019, 1	3	NA	20426
GL172669.1	2644652	2645497	2645110, 2645151, 2645067	11, 16, 12	4.39694, 6.06431, 6.97149	3.14073, 3.45608, 4.50591	2.20888, 3	3	NA	42845
GL172669.1	2649523	2650831	2649979, 2649974, 2649945	28, 30, 18	18.29736, 16.19266, 12.42823	7.2165, 5.89394, 6.46997	15.12714,	3	NA	47716
GL172669.1	2657156	2657903	2657533, 2657543, 2657624	50, 75, 24	45.87999, 63.57201, 21.00605	14.0435, 13.82275, 9.76145	42.08831,	3	NA	55349
GL172669.1	2702635	2703124	2702909, 2702904, 2702924	12, 8, 6	11.85767, 7.07159, 7.29403	7.51014, 5.20073, 5.30581	8.98544, 4	3	NA	100828
GL172669.1	3164754	3166232	3165713, 3165415, 3165302	13, 29, 16	10.379, 23.61407, 16.18697	6.40208, 9.66413, 9.24186	7.59527, 2	3	mylip	26550
GL172669.1	3402885	3403475	3403275, 3403404, 3403359	7, 14, 3	7.10297, 12.75552, 3.47371	5.27706, 7.64262, 3.03189	4.57191, 1	3	NA	10803
GL172669.1	3786813	3787916	3787264, 3787304, 3787547	20, 25, 6	19.92129, 23.41295, 6.4014	10.37862, 10.84616, 4.8799	16.69414,	3	STMND1	75463
GL172669.1	4090776	4091187	4091074, 4091108, 4091064	28, 16, 19	31.3955, 14.04087, 21.51797	14.51749, 7.93805, 11.61684	27.85612,	3	rnf144b	3007
GL172670.1	440253	440687	440389, 440389, 440434	30, 53, 23	35.66858, 59.25397, 27.28928	16.29014, 19.78446, 13.82197	32.04179,	3	NA	1761
GL172670.1	1322595	1323744	1323612, 1323245, 1322846	12, 11, 9	11.26426, 8.89461, 10.72164	7.15468, 5.88422, 7.15493	8.4212, 6.3	3	NA	2052
GL172670.1	1366518	1367345	1366923, 1366677, 1367030	14, 10, 9	12.64394, 7.38914, 10.07825	7.57426, 5.11603, 6.83102	9.72408, 4	3	bin3	16592
GL172670.1	1392246	1392836	1392414, 1392399, 1392422	11, 11, 13	10.60582, 8.41104, 13.84064	6.93244, 5.58113, 8.49513	7.80831, 5	3	EGR3	8308
GL172670.1	1476620	1477144	1477103, 1476871, 1476893	17, 13, 3	19.49949, 12.03475, 3.47371	10.94229, 7.4231, 3.03189	16.28759,	3	pebp4	13330
GL172670.1	1799384	1799575	1799574, 1799478, 1799498	7, 10, 3	6.69449, 7.65405, 3.37781	5.04797, 5.28351, 2.98791	4.1953, 5.2	3	ank1	49244
GL172670.1	1887646	1888116	1887908, 1887892, 1887719	7, 10, 6	5.52352, 9.39128, 5.83216	4.34124, 6.35644, 4.57461	3.15454, 6	3	plat	0
GL172670.1	2006670	2006962	2006832, 2006877, 2006815	23, 44, 23	23.09591, 50.01992, 28.3568	11.3315, 18.70572, 14.4341	19.76844,	3	NA	14856
GL172670.1	2256666	2257085	2256822, 2256834, 2256852	6, 7, 4	5.51356, 4.8451, 4.67325	4.39314, 3.90649, 3.78987	3.14574, 2	3	fgfr1	0
GL172670.1	2348303	2348724	2348556, 2348682, 2348548	14, 18, 5	13.44866, 16.06714, 5.9506	8.06455, 8.63158, 4.54784	10.49073,	3	letm2	24446
GL172670.1	2393449	2393913	2393728, 2393573, 2393611	6, 7, 4	5.96715, 5.97725, 4.67325	4.64927, 4.62287, 3.78987	3.52158, 3	3	letm2	6305
GL172670.1	2549068	2549754	2549605, 2549490, 2549192	4, 6, 5	3.42565, 4.30294, 5.50079	3.13796, 3.64568, 4.33868	1.48489, 2	3	NA	17645
GL172670.1	2816097	2816544	2816348, 2816380, 2816178	6, 10, 6	5.96715, 9.39128, 7.29403	4.64927, 6.35644, 5.30581	3.52158, 6	3	NA	3006
GL172670.1	2904708	2905474	2905128, 2904972, 2905141	5, 10, 4	4.82177, 9.39128, 4.46488	3.98509, 6.35644, 3.69425	2.51418, 6	3	NA	1199
GL172670.1	3098807	3099289	3098990, 3099034, 3099036	6, 8, 5	5.06496, 6.44699, 5.9506	4.12592, 4.8155, 4.54784	2.73812, 4	3	adam9	0
GL172670.1	3198044	3198514	3198483, 3198316, 3198173	8, 10, 7	8.42257, 9.39128, 8.69489	5.97763, 6.35644, 6.06379	5.76336, 6	3	plekha2	7304
GL172670.1	3407145	3407534	3407502, 3407308, 3407308	8, 9, 4	7.62407, 7.67202, 4.67325	5.52896, 5.44992, 3.78987	5.03638, 5	3	grk5	7235
GL172670.1	3535520	3536285	3535891, 3535901, 3535754	16, 9, 18	14.29285, 6.73685, 20.40623	8.0922, 4.86289, 11.22809	11.29159,	3	XB-GENE-949899	2737
GL172670.1	3546632	3547585	3546921, 3546669, 3547498	8, 16, 7	5.50545, 11.48328, 6.08239	4.21508, 6.38795, 4.68683	3.13928, 8	3	XB-GENE-949899	5129
GL172670.1	4110990	4111705	4111327, 4111372, 4111313	27, 44, 14	30.13583, 49.45457, 14.92875	14.13863, 18.38027, 8.94211	26.6256, 4	3	RFTN1	0
GL172671.1	474035	475169	474516, 474619, 474688	15, 30, 15	15.64184, 29.16514, 17.94632	9.15231, 12.44543, 10.45626	12.57992,	3	maoa	47858
GL172671.1	1246402	1246703	1246570, 1246570, 1246570	24, 34, 22	28.53208, 34.99558, 25.9713	14.23048, 14.29576, 13.35936	25.05891,	3	atp6ap2	35881
GL172671.1	2066718	2067375	2066868, 2067065, 2067105	11, 25, 6	10.60582, 26.51553, 6.6567	6.93244, 12.69611, 5.00845	7.80831, 2	3	NA	23116
GL172671.1	2138360	2138980	2138716, 2138811, 2138764	9, 19, 10	8.77314, 16.8936, 10.59656	6.111, 8.84622, 7.05292	6.09355, 1	3	mid1ip1	5810
GL172672.1	689949	690496	690229, 690197, 690302	17, 20, 4	21.19088, 22.255, 4.67325	11.87339, 11.77279, 3.78987	17.92307,	3	ANO6	19253
GL172672.1	3157053	3157953	3157605, 3157332, 3157420	33, 41, 25	28.52491, 31.36053, 22.84969	11.03473, 10.03294, 10.51468	25.052, 28	3	dock4	16517
GL172672.1	3167571	3169035	3167884, 3167757, 3168148, 3168794	25, 42, 24, 17	16.77853, 29.38709, 18.74584, 11.30195	7.15943, 8.92739, 8.47064, 6.05355	13.66725,	3	dock4	27035
GL172672.1	3961986	3962531	3962251, 3962065, 3962444	4, 7, 4	3.74195, 5.97725, 4.67325	3.32091, 4.62287, 3.78987	1.6222, 3.€	3	foxp2	22895
GL172673.1	22593	23091	22844, 22829, 22784	17, 20, 18	16.23509, 18.71921, 21.49679	9.01086, 9.65224, 11.84671	13.14931,	3	pak6	0
GL172673.1	105124	105676	105481, 105354, 105163	7, 19, 12	5.13345, 16.72984, 13.15613	4.09293, 8.74733, 8.25729	2.80161, 1	3	bub1b	4983
GL172673.1	658976	659723	659123, 659106, 659084	22, 15, 14	25.29347, 13.16372, 16.29325	12.96511, 7.65379, 9.7093	21.90464,	3	npas3	0
GL172673.1	707467	708312	707707, 707795, 707911	4, 8, 12	3.74195, 6.73981, 14.3175	3.32091, 4.99774, 8.88032	1.6222, 4.3	3	npas3	0
GL172673.1	1217968	1219173	1218930, 1218337, 1218245	9, 13, 9	7.59348, 10.81092, 9.35579	5.40134, 6.66988, 6.44216	5.0069, 8.1	3	NA	21409
GL172673.1	2512988	2513730	2513467, 2513478, 2513453	8, 8, 8	8.28589, 6.37769, 8.80246	5.90321, 4.77199, 6.179	5.65299, 4	3	gjd2	13688
GL172673.1	2578833	2579609	2578914, 2579000, 2578914	5, 13, 6	4.05709, 11.03409, 6.6567	3.5365, 6.80804, 5.00845	1.90867, 8	3	aqr	17174
GL172673.1	2623924	2624364	2624243, 2624123, 2624308	7, 7, 5	7.1696, 5.97725, 5.40789	5.31345, 4.62287, 4.29296	4.61046, 3	3	aqr	0
GL172673.1	2681889	2682807	2682404, 2682669, 2682244	11, 15, 7	10.66406, 13.21475, 7.84208	6.96705, 7.68511, 5.66426	7.86396, 1	3	dph6	0
GL172673.1	3285709	3286347	3285880, 3286228, 3285853	9, 11, 9	8.88537, 8.27243, 10.01275	6.17591, 5.49405, 6.79683	6.19961, 5	3	meis2	153543
GL172673.1	3302442	3303072	3302534, 3302509, 3302562	25, 25, 12	26.86394, 24.51518, 12.82015	12.90457, 11.50008, 8.0686	23.43188,	3	spred1	139757
GL172673.1	3831001	3831904	3831500, 3831828, 3831717	14, 6, 7	14.3105, 4.51135, 8.69489	8.58029, 3.77984, 6.06379	11.30855,	3	fmn1	38285
GL172674.1	22786	23057	22998, 23006, 23023	13, 11, 9	13.42064, 9.61892, 11.04428	8.25183, 6.33404, 7.30896	10.46414,	3	bpi	5940
GL172674.1	182917	183562	183132, 183186, 183151	6, 8, 6	5.68215, 6.59014, 6.5517	4.49007, 4.90493, 4.95623	3.29732, 4	3	bpi	0
GL172674.1	327186	327895	327668, 327804, 327476	5, 15, 4	4.30277, 13.11311, 4.67325	3.68597, 7.62272, 3.78987	2.12436, 1	3	rprd1a	0
GL172674.1	363924	364387	364230, 364210, 364234	14, 15, 7	12.33072, 11.88618, 7.10639	7.38193, 6.86988, 5.27894	9.43363, 9	3	vstm2l	3463
GL172674.1	599804	600166	600101, 600076, 600135	14, 12, 16	14.67861, 9.66318, 18.43661	8.79666, 6.16843, 10.55125	11.66087,	3	tgif2	0
GL172674.1	620269	620669	620467, 620547, 620520	9, 12, 6	9.24298, 11.86108, 7.29403	6.3792, 7.51216, 5.30581	6.53474, 9	3	dlgap4	0
GL172674.1	1198802	1199084	1198944, 1199080, 1198946	8, 11, 6	7.33538, 9.13706, 6.6567	5.35908, 6.0355, 5.00845	4.76518, 6	3	arhgap40	9119
GL172674.1	1200559	1200862	1200800, 1200693, 1200857	9, 7, 6	8.66407, 5.13485, 6.4014	6.04743, 4.09383, 4.8799	5.99101, 2	3	arhgap40	7341
GL172674.1	1331829	1332376	1332128, 1332128, 1332150	27, 36, 25	29.63186, 37.26357, 30.2597	13.83816, 14.80359, 14.91057	26.13264,	3	dhx35	3264
GL172674.1	1345666	1345882	1345854, 1345857, 1345847	22, 32, 29	23.3605, 35.0148, 36.22158	11.81917, 15.10864, 16.98927	20.02603,	3	dhx35	17101
GL172674.1	1588045	1588420	1588282, 1588233, 1588256	8, 12, 5	8.42257, 11.86108, 5.50079	5.97763, 7.51216, 4.33868	5.76336, 9	3	MAFB	140277
GL172674.1	1857063	1857409	1857066, 1857064, 1857066	14, 13, 6	14.99044, 10.04078, 7.29403	8.97777, 6.19235, 5.30581	11.95856,	3	plcg1	17384
GL172674.1	2053341	2054034	2053391, 2053353, 2053490	9, 13, 6	9.72082, 12.80191, 7.29403	6.64182, 7.88619, 5.30581	6.97155, 1	3	pigu	7860
GL172674.1	2180853	2181173	2180961, 2181148, 2181001	5, 9, 5	4.82177, 7.06866, 5.9506	3.98509, 5.07306, 4.54784	2.51418, 4	3	ahcy	31513
GL172674.1	2297397	2297722	2297663, 2297692, 2297652	9, 13, 10	9.24298, 12.31712, 10.71812	6.3792, 7.59462, 7.12015	6.53474, 9	3	raly	20549
GL172674.1	2328852	2329789	2329283, 2329574, 2329311	10, 18, 7	8.89113, 14.2312, 6.3257	6.05396, 7.5249, 4.83277	6.20456, 1	3	U5	4923
GL172674.1	2334245	2335294	2334577, 2334726, 2334649	14, 21, 8	14.03163, 18.71315, 7.85773	8.41471, 9.34355, 5.66361	11.04222,	3	U5	0
GL172674.1	2341815	2342397	2342348, 2342364, 2342368	12, 21, 12	11.43506, 19.54437, 13.15613	7.25774, 9.8421, 8.25729	8.58315, 1	3	U5	6987
GL172674.1	2507967	2508517	2508308, 2508381, 2508406	11, 26, 5	10.3251, 26.87838, 5.59755	6.7644, 12.55754, 4.38538	7.54325, 2	3	NA	19860
GL172674.1	2516804	2517420	2517166, 2517353, 2517158	19, 20, 12	18.00678, 17.39434, 14.23156	9.52023, 8.85476, 8.83588	14.84918,	3	chmp4b	13550
GL172674.1	2599671	2600174	2600143, 2600162, 2600009	16, 20, 8	15.94701, 20.68875, 7.76694	9.09776, 10.84083, 5.6116	12.87323,	3	e2f1	5733
GL172674.1	2628754	2629270	2628877, 2629046, 2628918	8, 16, 11	7.47668, 15.41438, 13.46208	5.44269, 8.77591, 8.49639	4.89713, 1	3	NECAB3	0
GL172674.1	2658561	2658929	2658893, 2658899, 2658897	23, 25, 13	24.76553, 25.99907, 14.5443	12.33305, 12.38692, 8.89247	21.39099,	3	NECAB3	0
GL172674.1	2685771	2686105	2685850, 2685850, 2685993	11, 7, 4	10.1122, 5.61836, 4.30163	6.63573, 4.4008, 3.61556	7.34229, 3	3	cbfa2t2	10650
GL172674.1	2711930	2712412	2712324, 2712385, 2712292	14, 25, 16	14.91094, 26.60415, 17.93939	8.9318, 12.74915, 10.26963	11.88377,	3	cbfa2t2	0
GL172674.1	2781686	2782049	2781998, 2782035, 2782044	42, 80, 41	51.31326, 95.49467, 52.35009	20.38505, 26.09315, 21.4584	47.44354,	3	tigd3	299
GL172674.1	2787225	2787701	2787349, 2787348, 2787384	20, 22, 14	22.56332, 22.45914, 15.35015	11.9536, 11.27809, 9.184	19.25287,	3	tigd3	5838
GL172674.1	2825879	2826070	2826042, 2826038, 2825993	6, 6, 4	5.09949, 4.4049, 4.15126	4.14694, 3.71155, 3.54016	2.76939, 2	3	tox2	0
GL172674.1	2841767	2842355	2842239, 2842284, 2842265	24, 28, 23	27.50714, 29.31424, 30.07615	13.62998, 13.27729, 15.38818	24.05846,	3	tox2	0
GL172674.1	2896136	2896413	2896223, 2896385, 2896192	8, 19, 6	7.57423, 18.6168, 5.53406	5.4999, 9.88966, 4.40503	4.98865, 1	3	tox2	32829
GL172674.1	2916038	2916811	2916783, 2916736, 2916404	19, 22, 12	20.21999, 19.48215, 12.62857	10.8531, 9.49346, 7.95947	16.98276,	3	tox2	52731
GL172674.1	2925705	2926097	2925740, 2925740, 2925734	22, 31, 18	26.5563, 31.77267, 21.49679	13.69542, 13.59061, 11.84671	23.13262,	3	tox2	62398
GL172674.1	3004280	3004820	3004283, 3004319, 3004335	27, 38, 28	31.91719, 41.03822, 34.39449	15.19434, 16.15442, 16.28712	28.36766,	3	ralgapb	48113
GL172674.1	3179206	3179431	3179285, 3179329, 3179307	20, 30, 8	21.26668, 29.99979, 8.56955	11.18692, 12.93196, 6.05652	17.9967, 2	3	rbl1	564
GL172674.1	3465464	3465888	3465493, 3465499, 3465532	20, 20, 12	20.37178, 19.71581, 12.56626	10.65022, 10.25455, 7.92374	17.13035,	3	NA	0
GL172674.1	3575538	3576926	3575864, 3576318, 3575662	7, 10, 9	6.25077, 8.0878, 9.70054	4.78814, 5.55635, 6.63089	3.78441, 5	3	cpne1	764
GL172674.1	3839171	3840083	3839547, 3839450, 3839528	9, 26, 11	8.77314, 28.39602, 14.24187	6.111, 13.46449, 8.86619	6.09355, 2	3	trpc4ap	9895
GL172674.1	3887016	3887450	3887062, 3887023, 3887071	11, 8, 5	10.16451, 6.4822, 5.69847	6.66744, 4.83755, 4.4331	7.39114, 4	3	xtr-mir-499	27743
GL172675.1	516674	517714	517096, 517440, 517335	29, 33, 15	30.87393, 29.24304, 15.05559	13.82751, 11.42291, 8.80395	27.34665,	3	mpzl2	0
GL172675.1	1697812	1698442	1698153, 1698128, 1697982	20, 19, 9	20.37178, 16.83862, 9.03892	10.65022, 8.81301, 6.26387	17.13035,	3	bud13	225101
GL172675.1	2047674	2048959	2047975, 2048571, 2048284	6, 10, 5	5.96715, 9.27513, 5.14967	4.64927, 6.2868, 4.16141	3.52158, 6	3	NA	47625
GL172675.1	2981654	2982079	2982030, 2981742, 2981879	8, 10, 5	7.24433, 8.66076, 5.27517	5.30475, 5.91256, 4.22616	4.68023, 6	3	pou2af1	29331
GL172676.1	90683	91738	91044, 91378, 91196	9, 18, 5	7.71701, 16.2906, 5.54867	5.47767, 8.76731, 4.3619	5.12244, 1	3	iqch	0
GL172676.1	681092	681408	681374, 681377, 681399	17, 16, 18	18.82666, 14.04087, 21.59501	10.55599, 7.93805, 11.90136	15.63723,	3	morf4l1	1055
GL172676.1	740932	742133	741880, 741861, 741861	26, 39, 16	25.59096, 39.45936, 17.11217	11.79046, 14.84044, 9.79056	22.19337,	3	adamts7	4176
GL172676.1	1010385	1010785	1010648, 1010632, 1010589	4, 10, 5	3.28906, 8.9331, 4.99237	3.0555, 6.07959, 4.0781	1.37521, 6	3	xtr-mir-184	7121
GL172676.1	1092551	1093219	1092974, 1092810, 1092702	8, 10, 5	6.20993, 9.12511, 5.59755	4.66614, 6.19629, 4.38538	3.74679, 6	3	zfand6	8682
GL172676.1	1181876	1182267	1182013, 1182105, 1182088	13, 18, 11	11.71272, 16.46278, 12.71187	7.22621, 8.87194, 8.11558	8.84616, 1	3	fah	3246

GL172676.1	2175473	2176446	2176075, 2175675, 2176082	21, 25, 15	22.40053, 25.26956, 17.76194	11.56075, 11.95039, 10.35658	19.0946, 2	3	5S_rRNA	120022
GL172676.1	2296557	2296948	2296719, 2296783, 2296711	7, 11, 6	7.1696, 8.77819, 6.168	5.31345, 5.81139, 4.75778	4.61046, 6	3	5S_rRNA	241106
GL172676.1	2609859	2610920	2610570, 2610260, 2610114, 2610639	16, 24, 7, 6	13.10843, 19.01517, 6.88081, 5.64193	7.36828, 8.62128, 5.15373, 4.46713	10.16616,	3	unc5d	111898
GL172676.1	2945282	2945779	2945525, 2945462, 2945465	11, 17, 6	10.9046, 15.29522, 5.64193	7.10903, 8.43856, 4.46713	8.08987, 1	3	unc5d	115872
GL172676.1	2961394	2961763	2961663, 2961669, 2961615	5, 6, 5	3.9168, 4.68027, 5.23257	3.44908, 3.88713, 4.20435	1.78158, 2	3	unc5d	131984
GL172676.1	2962760	2962969	2962819, 2962819, 2962812	8, 7, 4	6.94372, 5.82304, 4.15126	5.12297, 4.52813, 3.54016	4.42528, 3	3	unc5d	133350
GL172676.1	3010722	3011163	3010973, 3010942, 3010856	12, 22, 5	13.05874, 21.28482, 5.91431	8.20296, 10.5715, 4.53172	10.11961,	3	unc5d	181312
GL172676.1	3198287	3198751	3198603, 3198563, 3198706	7, 10, 16	6.84137, 9.17452, 19.65507	5.13151, 6.22617, 11.21774	4.33107, 6	3	dusp26	340629
GL172676.1	3429885	3430420	3429929, 3429892, 3429913	33, 43, 23	35.06587, 48.85407, 26.60554	14.73656, 18.48643, 13.42348	31.45152,	3	dusp26	108960
GL172676.1	3451790	3452493	3452006, 3451946, 3452000	11, 15, 6	11.35776, 11.88618, 5.91225	7.37194, 6.86988, 4.61907	8.51017, 9	3	dusp26	86887
GL172676.1	3454688	3455255	3455107, 3454991, 3454802	5, 19, 4	4.30277, 16.46445, 4.46488	3.68597, 8.58735, 3.69425	2.12436, 1	3	dusp26	84125
GL172676.1	3737417	3737978	3737850, 3737922, 3737919	17, 24, 31	17.4139, 22.6569, 40.97003	9.72241, 10.73418, 18.99312	14.27855,	3	mthfd2	1494
GL172677.1	659232	659854	659626, 659722, 659710	8, 20, 7	8.42257, 22.84892, 8.69489	5.97763, 12.1201, 6.06379	5.76336, 1	3	atp2b2	32089
GL172677.1	2188606	2189332	2189235, 2189202, 2188918	11, 24, 8	9.5732, 23.84928, 9.05167	6.30586, 11.44594, 6.30653	6.84587, 2	3	gstm1	6471
GL172677.1	3004776	3005578	3005264, 3005426, 3005360	11, 13, 6	12.43693, 12.25946, 7.29403	7.97018, 7.55969, 5.30581	9.52692, 9	3	arl8a	0
GL172677.1	3417821	3418560	3418465, 3418358, 3418407	4, 11, 4	3.73494, 9.7598, 4.34116	3.31698, 6.42066, 3.63492	1.6222, 7.1	3	nuak2	516
GL172677.1	3600069	3601035	3600619, 3600699, 3600411	13, 27, 13	12.14841, 27.43215, 13.0991	7.49227, 12.52631, 8.06282	9.26135, 2	3	cdk18	0
GL172678.1	79531	80293	80106, 79705, 80176	12, 12, 8	11.09909, 9.74388, 7.63508	7.05451, 6.2187, 5.53536	8.26391, 7	3	cul4a	8515
GL172678.1	1187711	1188483	1188233, 1188166, 1188314	4, 14, 5	3.42565, 13.8891, 4.84526	3.13796, 8.32957, 3.99806	1.48489, 1	3	cars2	0
GL172678.1	1191026	1191982	1191886, 1191902, 1191825	15, 11, 12	13.8583, 8.59076, 11.60888	8.07921, 5.69393, 7.36202	10.87599,	3	cars2	0
GL172678.1	2232400	2233263	2233198, 2232898, 2232845	8, 7, 4	8.34715, 5.8946, 4.01199	5.9367, 4.57222, 3.46784	5.71011, 3	3	fam155a	7796
GL172678.1	2797118	2797764	2797421, 2797665, 2797589	16, 17, 9	15.09881, 14.97125, 9.24728	8.58417, 8.24108, 6.38161	12.06341,	3	efnb2	224373
GL172679.1	167971	168413	168248, 168167, 168014	8, 8, 8	7.24433, 5.75617, 6.69878	5.30475, 4.3766, 4.97237	4.68023, 3	3	ASIC2	45535
GL172679.1	168806	169230	169072, 169072, 168974	16, 19, 20	17.95866, 18.68597, 22.20869	10.28064, 9.9315, 11.74555	14.80292,	3	ASIC2	44718
GL172679.1	242280	242768	242491, 242480, 242379	8, 6, 6	8.42257, 4.27812, 6.4014	5.97763, 3.62957, 4.8799	5.76336, 2	3	ASIC2	0
GL172679.1	505412	505989	505694, 505861, 505662	11, 11, 12	12.33515, 9.09572, 13.7434	7.91559, 6.00975, 8.57827	9.43699, 6	3	smarcd2	44328
GL172679.1	676434	677339	677046, 677029, 677019	62, 72, 53	68.35293, 75.99268, 58.0183	20.56149, 20.04956, 19.10701	64.27331,	3	scn4a	1204
GL172679.1	684309	685281	684658, 684777, 684785	10, 20, 4	10.53228, 17.55532, 4.64325	7.01712, 8.95123, 3.77643	7.73927, 1	3	scn4a	9079
GL172679.1	827811	828305	827829, 827860, 828233	5, 9, 8	4.77263, 7.75488, 8.80246	3.9578, 5.501, 6.179	2.51418, 5	3	gosr2	0
GL172679.1	854298	855301	854745, 854899, 854546	18, 15, 11	18.60558, 14.7819, 11.4314	10.16809, 8.63964, 7.41405	15.42473,	3	gosr2	16198
GL172679.1	854298	855507	855274, 854899, 854546	6, 15, 11	4.48574, 14.7819, 11.4314	3.76346, 8.63964, 7.41405	2.28228, 1	3	gosr2	16198
GL172679.1	885467	886113	885739, 885775, 885514	24, 33, 15	25.99978, 32.96156, 17.58264	12.73437, 13.51119, 10.25879	22.5903, 2	3	gosr2	47367
GL172679.1	958362	959149	958617, 958645, 958848	13, 44, 14	13.20994, 47.75456, 16.46576	8.12823, 17.41091, 9.80275	10.26285,	3	mocs2	5515
GL172679.1	1116510	1116871	1116831, 1116727, 1116685	20, 32, 18	21.0967, 33.24676, 19.60235	11.08535, 14.06622, 10.75987	17.83215,	3	itga1	20353
GL172679.1	1407899	1408374	1408348, 1408320, 1408313	24, 31, 8	26.38029, 33.66599, 9.68495	12.96149, 14.70817, 6.61366	22.96032,	3	itga1	311742
GL172679.1	1524695	1525082	1524902, 1524869, 1524962	9, 10, 5	9.72082, 7.86505, 5.85847	6.64182, 5.4165, 4.50665	6.97155, 5	3	isl1	198245
GL172679.1	1736733	1737052	1736853, 1736854, 1736983	4, 8, 5	3.70152, 5.93025, 5.59755	3.29816, 4.48816, 4.38538	1.6222, 3.6	3	isl1	5478
GL172679.1	1887407	1888868	1888198, 1888222, 1888319	15, 35, 9	14.71973, 35.9563, 11.12912	8.60219, 14.45276, 7.34851	11.70031,	3	parp8	120434
GL172679.1	2014173	2015250	2014910, 2014910, 2014910	53, 70, 47	58.59706, 77.33985, 57.30606	19.42318, 21.56949, 21.52726	54.62638,	3	parp8	0
GL172679.1	2114351	2114747	2114557, 2114489, 2114519	9, 8, 5	8.66407, 7.01915, 5.69847	6.04743, 5.16892, 4.4331	5.99101, 4	3	parp8	27388
GL172679.1	2208838	2209463	2209415, 2209438, 2209391	11, 11, 9	10.96672, 9.80774, 10.35155	7.14544, 6.45006, 6.97128	8.14942, 7	3	emb	0
GL172679.1	2374071	2374438	2374374, 2374351, 2374374	32, 57, 28	35.18635, 64.25118, 36.96613	15.21026, 20.66934, 17.75574	31.56988,	3	hcn1	0
GL172679.1	2436277	2437226	2436730, 2436674, 2436547	7, 12, 6	7.1696, 11.04188, 7.29403	5.31345, 7.01971, 5.30581	4.61046, 8	3	hcn1	0
GL172679.1	2613306	2614329	2614031, 2613889, 2614002	18, 24, 12	20.55486, 23.99953, 12.88563	11.31361, 11.53593, 8.10565	17.30766,	3	mrps30	7194
GL172679.1	2763139	2763792	2763552, 2763530, 2763671	9, 9, 10	7.39636, 6.55163, 10.3632	5.27874, 4.74494, 6.92219	4.82212, 4	3	fgf10	15739
GL172679.1	2860040	2860421	2860145, 2860198, 2860167	13, 18, 12	12.43975, 17.19487, 13.90113	7.66875, 9.31668, 8.66246	9.5289, 14	3	fgf10	10305
GL172679.1	2925921	2926381	2926019, 2926147, 2926181	6, 15, 5	5.81608, 13.06292, 5.9506	4.56563, 7.59191, 4.54784	3.4176, 10	3	fgf10	76186
GL172679.1	2939081	2939456	2939416, 2939338, 2939115	9, 10, 3	8.55802, 8.84031, 3.41347	5.98518, 6.02287, 3.00444	5.8909, 6.3	3	fgf10	89346
GL172679.1	3192885	3193165	3192917, 3192913, 3192887	6, 13, 8	5.96715, 13.14514, 10.1465	4.64927, 8.09002, 6.82176	3.52158, 1	3	tmem267	7387
GL172679.1	3201134	3201696	3201182, 3201163, 3201185	39, 50, 14	47.80175, 62.33674, 18.28596	19.68513, 22.99102, 10.7324	43.98058,	3	hmgcs1	14041
GL172679.1	3353134	3353478	3353338, 3353239, 3353423	8, 10, 4	7.38183, 9.37821, 4.67325	5.38666, 6.34863, 3.78987	4.80856, 6	3	CCDC152	0
GL172679.1	3539476	3540050	3539549, 3539632, 3539512	9, 18, 10	9.00096, 17.45593, 12.33934	6.24222, 9.47501, 7.95424	6.30808, 1	3	ghr	110609
GL172679.1	3795266	3795864	3795480, 3795480, 3795660	22, 30, 16	22.1372, 28.66215, 17.78134	11.08439, 12.15443, 10.17907	18.83941,	3	oxct1	0
GL172679.1	3802797	3803343	3803291, 3803301, 3803298	7, 27, 12	6.94335, 28.09375, 13.66628	5.18876, 12.92024, 8.53679	4.42528, 2	3	oxct1	0
GL172680.1	36393	38057	37227, 36526, 36834, 37924	11, 11, 12, 8	9.13339, 9.3497, 11.60888, 7.04274	6.03321, 6.16764, 7.36202, 5.18324	6.43178, 6	3	NA	0
GL172680.1	54538	54814	54737, 54571, 54583	5, 7, 4	4.40263, 5.97725, 3.46461	3.74533, 4.62287, 3.1611	2.21004, 3	3	NA	7942
GL172680.1	70921	71306	71038, 71013, 71038	25, 22, 25	23.71142, 17.34562, 27.66765	11.02262, 8.24654, 13.38448	20.3668, 1	3	creld1	0
GL172680.1	85807	86623	86406, 86064, 86328	9, 15, 9	6.76373, 11.72411, 8.13839	4.87998, 6.77081, 5.73483	4.25982, 9	3	rbm46	12692
GL172680.1	333917	334925	334196, 334164, 334153, 334609	11, 9, 5, 3	12.01255, 8.21099, 5.9506, 3.47371	7.73996, 5.77858, 4.54784, 3.03189	9.13199, 5	3	klhdc8b	0
GL172680.1	429922	431035	430700, 430688, 430696	22, 35, 10	23.89932, 34.88273, 10.59656	12.1411, 13.83751, 7.05292	20.54867,	3	klhdc8b	38595
GL172680.1	559897	560308	560073, 560058, 560135	16, 21, 12	16.83862, 22.93552, 15.80834	9.62959, 11.87951, 9.60504	13.72526,	3	NA	0
GL172680.1	578485	579158	578662, 578622, 578655	25, 48, 23	26.23835, 49.93568, 26.70034	12.53018, 16.89772, 13.479	22.82175,	3	NA	0
GL172680.1	615600	616067	615619, 615645, 615771	5, 12, 7	4.47183, 9.66318, 6.83761	3.78598, 6.16843, 5.12939	2.26935, 7	3	NA	7248
GL172680.1	756472	756780	756739, 756737, 756733	23, 41, 27	25.52391, 41.63242, 33.99821	12.78506, 15.24516, 16.4039	22.12768,	3	slc6a8	0
GL172680.1	775273	775543	775328, 775311, 775278	18, 42, 10	21.99239, 48.03333, 11.31133	12.12048, 18.4628, 7.43928	18.69793,	3	slc6a8	0
GL172680.1	810145	810560	810390, 810336, 810456	7, 10, 6	6.16939, 9.17452, 6.99807	4.73936, 6.22617, 5.17195	3.70916, 6	3	slc6a8	5544
GL172680.1	937906	938158	937935, 937946, 937914	28, 58, 32	32.49485, 65.30111, 39.67838	15.17061, 20.77344, 17.86854	28.93256,	3	NA	41491
GL172680.1	994522	995108	994903, 995020, 994749	8, 17, 12	8.4095, 12.89704, 11.60888	5.97056, 6.98739, 7.36202	5.76336, 1	3	NA	13612
GL172680.1	1050095	1051168	1050287, 1050992, 1050997	16, 17, 9	15.54205, 13.76564, 10.07825	8.85329, 7.50888, 6.83102	12.48418,	3	NA	2863
GL172680.1	1078592	1079316	1079094, 1079097, 1079079	15, 23, 7	15.41679, 23.03769, 7.96278	9.0192, 11.29653, 5.72394	12.36344,	3	NA	31360
GL172680.1	1136481	1136673	1136632, 1136562, 1136592	7, 13, 6	6.16939, 13.14514, 6.6567	4.73936, 8.09002, 5.00845	3.70916, 1	3	pim1	71126
GL172680.1	1194261	1194787	1194589, 1194599, 1194678	7, 7, 6	6.55439, 5.21943, 6.45058	4.96711, 4.14808, 4.90508	4.06616, 2	3	pim1	13012
GL172680.1	1199862	1200145	1200119, 1200126, 1200105	48, 65, 48	57.77656, 75.53102, 61.41742	21.31744, 22.96257, 23.44698	53.81638,	3	pim1	7654
GL172680.1	1223519	1224412	1223683, 1224013, 1223765	8, 11, 6	7.88469, 9.3497, 6.5517	5.67897, 6.16764, 4.95623	5.27935, 6	3	pim1	5463
GL172680.1	1380995	1381186	1381011, 1381022, 1381019	21, 20, 11	20.44908, 20.68875, 11.79996	10.38721, 10.84083, 7.62212	17.20509,	3	ndufaf3	2266
GL172680.1	1900710	1901201	1900877, 1900875, 1900878	42, 61, 48	48.81667, 65.84339, 61.70068	18.9195, 19.70218, 23.61338	44.98067,	3	nckipsd	36597
GL172680.1	1950383	1950677	1950429, 1950402, 1950413	13, 20, 14	13.63968, 22.06591, 17.61756	8.37925, 11.6614, 10.40352	10.67258,	3	nckipsd	0
GL172680.1	2125668	2126284	2125918, 2125927, 2125927	11, 18, 8	9.5732, 15.04268, 8.4044	6.30586, 8.01145, 5.9678	6.84587, 1	3	slc26a6	0
GL172680.1	2726678	2727470	2727154, 2727010, 2726909, 2727354	7, 13, 6, 5	6.33443, 10.35117, 5.75442, 4.80993	4.83795, 6.38482, 4.53101, 3.97854	3.86259, 7	3	gata2	92660
GL172680.1	2823357	2824032	2823717, 2823583, 2823627	16, 29, 10	16.01695, 28.65304, 11.24131	9.13983, 12.5148, 7.40241	12.9408, 2	3	eefsec	111764
GL172680.1	3060742	3061809	3061263, 3061355, 3061343	8, 8, 7	8.42257, 5.75617, 7.01416	5.97763, 4.3766, 5.22813	5.76336, 3	3	U4	827
GL172680.1	3146287	3146709	3146665, 3146656, 3146667	35, 44, 25	35.98742, 45.73992, 31.28588	14.4707, 16.28375, 15.49965	32.35472,	3	mgll	0
GL172680.1	3152849	3153366	3153284, 3153251, 3153187	18, 26, 8	17.79149, 24.36556, 9.05167	9.67812, 11.06667, 6.30653	14.64268,	3	mgll	0
GL172680.1	3233651	3234217	3234143, 3233934, 3233949	8, 12, 9	7.15567, 10.73343, 9.64095	5.25151, 6.83116, 6.59867	4.61046, 8	3	mcm2	3723
GL172680.1	3313165	3313670	3313286, 3313346, 3313321	7, 16, 6	6.50909, 17.16856, 7.29403	4.94073, 9.82359, 5.30581	4.02371, 1	3	tpra1	37104
GL172680.1	3329639	3330814	3330169, 3330319, 3330173, 3330769	5, 9, 5, 5	4.0863, 6.34719, 4.51488, 4.77515	3.55452, 4.61437, 3.81106, 3.9592	1.93351, 3	3	tpra1	53578
GL172680.1	3337128	3338113	3337766, 3337537, 3338041	14, 16, 8	13.03233, 12.82211, 8.24665	7.81172, 7.19398, 5.88165	10.09457,	3	tpra1	61067
GL172680.1	3352001	3352665	3352411, 3352454, 3352207	11, 17, 6	11.22377, 14.91871, 5.95321	7.29486, 8.20906, 4.64162	8.38272, 1	3	tmprss15	51676
GL172680.1	3416662	3416971	3416788, 3416879, 3416816	5, 9, 3	4.50727, 7.92635, 3.47371	3.80664, 5.60609, 3.03189	2.30125, 5	3	tmprss15	0
GL172680.1	3460869	3461048	3460932, 3460939, 3460941	6, 6, 6	5.96715, 4.59436, 7.29403	4.64927, 3.83274, 5.30581	3.52158, 2	3	tmprss15	0
GL172680.1	3625213	3625676	3625505, 3625515, 3625518	52, 84, 47	62.18077, 102.77526, 62.88861	21.91, 27.84013, 24.80383	58.16932,	3	chodl	55655
GL172680.1	3785061	3785726	3785673, 3785384, 3785521	18, 15, 9	19.25819, 12.58265, 8.34444	10.55677, 7.29694, 5.85853	16.05481,	3	cpox	6382
GL172681.1	3512320	3512971	3512884, 3512410, 3512433	10, 17, 8	7.86015, 14.22254, 8.4586	5.41341, 7.78557, 5.99709	5.25709, 1	3	NA	0
GL172682.1	917617	918374	918033, 917999, 918195	37, 40, 12	41.06192, 45.47111, 14.23156	16.59398, 17.86546, 8.83588	37.34232,	3	fbxl7	151446
GL172682.1	1176889	1177578	1177220, 1177088, 1176993	7, 17, 7	4.21503, 10.49807, 5.59189	3.49347, 5.59873, 4.38421	2.0478, 7.8	3	znf622	50445
GL172682.1	1269128	1269989	1269623, 1269471, 1269622	17, 19, 11	20.2407, 18.41287, 13.19894	11.35794, 9.76624, 8.36554	17.00287,	3	fam134b	2020
GL172682.1	3562780	3563638	3563166, 3563192, 3563023	55, 87, 34	56.96612, 91.24966, 37.21511	17.68135, 20.9787, 15.59396	53.01654,	3	NA	138921
GL172682.1	3673721	3674471	3673968, 3674051, 3674398	13, 25, 11	12.32133, 22.24272, 11.31465	7.59717, 10.1601, 7.3472	9.42482, 1	3	NA	28088
GL172683.1	297097	297900	297318, 297350, 297510	14, 13, 13	13.44866, 11.98001, 14.39632	8.06455, 7.38972, 8.81006	10.49073,	3	mpp7	0
GL172683.1	536136	536522	536373, 536344, 536465	10, 7, 6	10.39648, 5.97725, 7.05929	6.94096, 4.62287, 5.20024	7.61003, 3	3	armc4	0
GL172683.1	609818	610974	610521, 610520, 610368	18, 41, 19	17.65252, 43.14208, 21.69099	9.59407, 16.09106, 11.71707	14.50784,	3	armc4	1102
GL172683.1	690943	691524	691056, 691200, 691368	8, 7, 4	7.42892, 5.27726, 4.46488	5.41453, 4.18506, 3.69425	4.85345, 3	3	mkx	0
GL172683.1	714116	714683	714315, 714340, 714333	15, 21, 13	14.78606, 19.00078, 14.39632	8.64215, 9.5157, 8.81006	11.76448,	3	mkx	12529
GL172683.1	846875	847784	847375, 847471, 847285	15, 20, 15	16.36908, 21.78996, 20.414	9.57629, 11.49821, 11.69434	13.27778,	3	mastl	7260
GL172683.1	1131365	1131603	1131437, 1131394, 1131404	13, 12, 7	13.79066, 9.66318, 7.84208	8.46641, 6.16843, 5.66426	10.8171, 7	3	gad2	15664
GL172683.1	1312151	1312593	1312558, 1312534, 1312563	19, 38, 24	21.60968, 45.43351, 31.18195	11.67002, 18.73245, 15.73225	18.32833,	3	myo3a	10973
GL172683.1	1352542	1353715	1353472, 1352774, 1353303	11, 12, 5	11.6377, 9.99443, 5.50079	7.53109, 6.37457, 4.33868	8.77589, 7	3	gpr158	17519
GL172683.1	1496619	1497221	1496915, 1496914, 1496914	28, 50, 24	27.57931, 49.35705, 25.66978	12.24979, 15.77945, 12.53702	24.12932,	3	gpr158	4512
GL172683.1	1586402	1586664	1586410, 1586457, 1586433	22, 44, 24	24.67026, 48.16496, 30.6645	12.59865, 17.64353, 15.44595	21.29787,	3	prtfdc1	5674
GL172683.1	1610479	1610916	1610783, 1610591, 1610497	7, 10, 3	6.25077, 7.79347, 3.41347	4.78814, 5.37143, 3.00444	3.78441, 5	3	prtfdc1	0
GL172683.1	1621350	1621728	1621440, 1621378, 1621390	9, 12, 5	9.72082, 11.86108, 5.9506	6.64182, 7.51216, 4.54784	6.97155, 9	3	prtfdc1	8788
GL172683.1	1899199	1899659	1899632, 1899625, 1899633	37, 52, 32	41.49343, 59.00161, 39.67838	16.84705, 20.10849, 17.86854	37.76714,	3	kiaa1217	10452
GL172683.1	2290198	2291713	2290864, 2290862, 2290887	10, 16, 9	9.13239, 11.6224, 8.98852	6.2007, 6.47081, 6.23511	6.43114, 8	3	ptf1a	12794
GL172683.1	2313105	2313916	2313533, 2313797, 2313287	8, 15, 8	7.88469, 12.91479, 8.35103	5.67897, 7.50094, 5.93881	5.27935, 1	3	ptf1a	7308
GL172683.1	2574422	2575388	2574804, 2574781, 2574819	17, 19, 20	18.04435, 18.48026, 26.09407	10.09765, 9.80703, 13.92487	14.88575,	3	spag6	0
GL172683.1	2751293	2751591	2751577, 2751558, 2751341	7, 11, 3	6.42049, 10.10652, 3.47371	4.8888, 6.63228, 3.03189	3.94161, 7	3	dnajc1	34595
GL172683.1	3241641	3242015	3241969, 3241987, 3241987	22, 21, 22	22.76927, 20.96503, 25.9713	11.46452, 10.69835, 13.35936	19.45236,	3	nebl	15660
GL172683.1	3329431	3330015	3329565, 3329640, 3329879	12, 13, 11	12.05017, 12.7389, 13.83651	7.62377, 7.84853, 8.67735	9.16715, 9	3	plxdc2	4690
GL172683.1	3335238	3335613	3335569, 3335479, 3335558	7, 5, 3	6.79162, 3.94561, 3.47371	5.10336, 3.46715, 3.03189	4.28406, 1	3	plxdc2	0
GL172683.1	3372465	3372989	3372777, 3372641, 3372615	6, 8, 5	5.24248, 5.99047, 5.14967	4.2332, 4.52662, 4.16141	2.89962, 3	3	plxdc2	0
GL172683.1	3493267	3493538	3493382, 3493316, 3493318	8, 13, 6	7.99469, 12.80191, 6.50067	5.74128, 7.88619, 4.93052	5.38155, 1	3	plxdc2	33427
GL172683.1	3584883	3585317	3584912, 3584927, 3584941	18, 41, 15	20.35565, 45.53249, 18.95532	11.19891, 17.45867, 10.98488	17.11452,	3	plxdc2	125043
GL172683.1	3631867	3632657	3632629, 3632582, 3632653	60, 87, 28	70.72563, 100.00184, 34.16525	22.78684, 24.98788, 16.15351	66.62228,	3	U2	130566
GL172683.1	3798923	3799864	3799546, 3799519, 3799421	15, 20, 17	14.46121, 19.43804, 22.12333	8.44598, 10.0867, 12.35799	11.45219,	3	U2	35552
GL172684.1	13457	13724	13607, 13684, 13596	4, 8, 7	3.74195, 7.07159, 8.69489	3.32091, 5.20073, 6.06379	1.6222, 4.6	3	zfp64	4557
GL172684.1	125463	125923	125834, 125848, 125707	8, 7, 4	7.1122, 5.97725, 4.67325	5.22529, 4.62287, 3.78987	4.57946, 3	3	sall4	24050
GL172684.1	239690	240411	239807, 239801, 239995	7, 9, 3	6.12952, 6.67403, 3.47371	4.71533, 4.82293, 3.03189	3.67231, 4	3	nfatc2	11587
GL172684.1	308098	308564	308504, 308500, 308559	30, 39, 24	32.20277, 40.67612, 29.81695	14.23201, 15.52754, 14.96922	28.647, 37	3	nfatc2	28801
GL172684.1	482814	483134	483089, 483091, 483083	26, 22, 10	29.82978, 22.85945, 11.997	14.3176, 11.51868, 7.78835	26.32584,	3	kcng1	302
GL172684.1	718990	719628	719354, 719262, 719268	9, 12, 4	8.66407, 10.93692, 4.67325	6.04743, 6.95571, 3.78987	5.99101, 8	3	fam65c	0
GL172684.1	806456	806865	806503, 806609, 806481	15, 24, 8	15.34351, 23.70127, 8.29845	8.97569, 11.35734, 5.91009	12.29352,	3	ptpn1	22571
GL172684.1	905041	905819	905561, 905535, 905433	8, 6, 5	6.48409, 4.4843, 5.9506	4.83873, 3.76254, 4.54784	4.00135, 2	3	cebpb	23582
GL172684.1	918184	918809	918536, 918562, 918573	7, 7, 3	6.60038, 4.72289, 3.2112	4.99378, 3.82689, 2.90793	4.10761, 2	3	cebpb	10592
GL172684.1	932494	932836	932725, 932661, 932734	10, 15, 8	8.98596, 12.49102, 7.5499	6.11182, 7.24068, 5.48567	6.29482, 9	3	cebpb	2225
GL172684.1	945596	945985	945873, 945849, 945831	34, 44, 22	38.23524, 47.75456, 25.03136	16.19629, 17.41091, 12.8114	34.56211,	3	MGC147314	0
GL172684.1	1003526	1004021	1003605, 1003548, 1003715	8, 9, 5	6.08111, 5.9459, 4.67395	4.58437, 4.35743, 3.90232	3.62785, 3	3	spata2	12170
GL172684.1	1029302	1029741	1029638, 1029621, 1029569	20, 25, 14	22.36548, 23.67755, 15.35015	11.8377, 11.00257, 9.184	19.06081,	3	rnf114	1812
GL172684.1	1088296	1089288	1088393, 1088337, 1088500	17, 22, 14	16.76548, 20.18456, 16.12551	9.33227, 9.9117, 9.61761	13.6543, 1	3	snai1	31980
GL172684.1	1098158	1098501	1098206, 1098211, 1098169	7, 10, 9	7.1696, 8.4887, 9.46739	5.31345, 5.80621, 6.50386	4.61046, 5	3	snai1	41842
GL172684.1	1162879	1163196	1162926, 1163043, 1162928	7, 14, 6	6.25077, 12.18837, 6.45058	4.78814, 7.29433, 4.90508	3.78441, 9	3	snai1	106563
GL172684.1	1169079	1169709	1169644, 1169626, 1169645	22, 39, 20	27.19152, 47.9613, 26.76487	14.0553, 19.77932, 14.27334	23.75121,	3	snai1	112763
GL172684.1	1176654	1177159	1176917, 1176925, 1176773	6, 7, 5	5.90927, 5.75319, 5.9506	4.61743, 4.48487, 4.54784	3.50164, 3	3	snai1	120338
GL172684.1	1333101	1333403	1333201, 1333211, 1333225	13, 24, 12	13.34951, 24.5441, 13.82167	8.21021, 11.86236, 8.62016	10.39596,	3	tmem189	0
GL172684.1	1345174	1345763	1345438, 1345400, 1345426	18, 20, 5	16.86914, 15.55335, 4.64121	9.11888, 7.76668, 3.88372	13.75452,	3	tmem189	0
GL172684.1	1483577	1484118	1483906, 1483906, 1483852	19, 24, 13	21.31193, 24.46445, 15.0131	11.49694, 11.8146, 9.14923	18.04063,	3	b4galt6	0
GL172684.1	1542048	1542436	1542233, 1542350, 1542084	5, 7, 4	4.82177, 5.97725, 4.67325	3.98509, 4.62287, 3.78987	2.51418, 3	3	prex1	25221
GL172684.1	1574401	1575125	1574773, 1574581, 1574915, 1574958	13, 7, 8, 5	13.07382, 4.8451, 5.84201, 5.91431	8.04786, 3.90649, 4.43168, 4.53172	10.13353,	3	prex1	0
GL172684.1	1615544	1616013	1615678, 1615779, 1615647	4, 10, 5	3.74195, 9.07627, 5.50079	3.32091, 6.1667, 4.33868	1.6222, 6.5	3	prex1	0
GL172684.1	1759152	1759529	1759206, 1759327, 1759206	10, 17, 9	11.06011, 18.56115, 11.04428	7.306, 10.40145, 7.30896	8.22712, 1	3	prex1	102945
GL172684.1	1821740	1822148	1822066, 1822066, 1822066	22, 47, 14	20.92422, 50.81061, 16.12551	10.35482, 17.80853, 9.61761	17.66578,	3	sulf2	99558
GL172684.1	1837313	1837656	1837331, 1837362, 1837356	23, 24, 18	23.56695, 22.92472, 20.93229	11.61446, 10.89357, 11.52911	20.22607,	3	sulf2	84050
GL172684.1	1976637	1977223	1977136, 1977194, 1977156	21, 41, 21	22.66679, 41.63242, 24.0973	11.71964, 15.24516, 12.56358	19.35329,	3	ncoa3	0
GL172684.1	2203191	2203587	2203384, 2203341, 2203360	5, 7, 5	4.69353, 5.8946, 5.06966	3.9134, 4.57222, 4.11933	2.45909, 3	3	eya2	0
GL172684.1	2291977	2293109	2292075, 2292863, 2292556	6, 6, 5	5.5965, 4.86158, 5.69847	4.44108, 4.00069, 4.4331	3.21976, 2	3	eya2	77119
GL172684.1	2468117	2468914	2468844, 2468743, 2468394	6, 8, 6	5.96715, 6.89656, 7.29403	4.64927, 5.09414, 5.30581	3.52158, 4	3	NA	2649
GL172684.1	2592731	2593189	2593108, 2592931, 2592980	6, 7, 7	4.89903, 4.79556, 6.6707	4.02393, 3.87426, 5.03432	2.58474, 2	3	arhgap27	0
GL172684.1	2671209	2671505	2671344, 2671380, 2671340	6, 8, 3	4.93136, 6.89656, 3.47371	4.04392, 5.09414, 3.03189	2.61555, 4	3	ada.2	848
GL172684.1	2703178	2703416	2703241, 2703240, 2703250	22, 25, 16	24.77128, 25.75015, 18.69936	12.65828, 12.23791, 10.69793	21.39609,	3	ada	14705
GL172684.1	2852887	2853375	2853317, 2853319, 2853307	9, 14, 9	7.35817, 12.1395, 10.35155	5.25488, 7.26424, 6.97128	4.78711, 9	3	cse1l	0
GL172684.1	2913030	2913467	2913430, 2913438, 2913315	8, 16, 8	8.42257, 17.06606, 10.1465	5.97763, 9.76351, 6.82176	5.76336, 1	3	NA	2163
GL172684.1	2932102	2932822	2932470, 2932580, 2932202	5, 8, 4	4.73273, 7.07159, 4.30163	3.93547, 5.20073, 3.61556	2.49377, 4	3	SNORD12	9604
GL172684.1	2938850	2939423	2939293, 2939230, 2939087	6, 8, 3	4.62553, 7.07159, 3.47371	3.85252, 5.20073, 3.03189	2.40124, 4	3	kcnb1	15206
GL172684.1	2965163	2965525	2965219, 2965328, 2965385	7, 7, 3	5.30682, 5.82304, 3.17999	4.20391, 4.52813, 2.89245	2.95991, 3	3	kcnb1	0
GL172684.1	2978415	2978760	2978456, 2978417, 2978468	13, 33, 18	13.79066, 36.37105, 20.15609	8.46641, 15.506, 11.0834	10.8171, 3	3	kcnb1	0
GL172684.1	3231872	3232294	3232250, 3232236, 3232198	11, 7, 9	12.43693, 5.82304, 10.72164	7.97018, 4.52813, 7.15493	9.52692, 3	3	r3hdml	43379
GL172684.1	3419714	3420256	3419922, 3420049, 3420015	6, 10, 4	5.63898, 8.36423, 4.50795	4.46544, 5.72892, 3.71446	3.25772, 5	3	jph2	0
GL172684.1	3469183	3470121	3469571, 3469535, 3469577	13, 13, 8	13.63968, 10.47257, 9.46129	8.37925, 6.46012, 6.50801	10.67258,	3	jph2	40353
GL172684.1	3491563	3491927	3491803, 3491723, 3491676	5, 7, 4	4.82177, 5.97725, 4.67325	3.98509, 4.62287, 3.78987	2.51418, 3	3	trhr3	18997
GL172684.1	3556253	3556836	3556691, 3556744, 3556743	21, 30, 29	24.68074, 32.42001, 38.64196	12.90176, 14.36101, 18.368	21.30801,	3	trhr3	20357
GL172684.1	3586891	3587234	3586894, 3586896, 3586929	10, 13, 7	9.49535, 11.07988, 8.02489	6.41851, 6.83637, 5.75426	6.77344, 8	3	ocstamp	30494
GL172685.1	2019582	2020426	2019931, 2020009, 2020179	13, 20, 5	12.74759, 20.22553, 4.20091	7.85373, 10.56212, 3.62458	9.82421, 1	3	foxn3	7506
GL172685.1	3659909	3660403	3659974, 3660349, 3660135	7, 8, 8	6.46447, 5.96021, 9.31951	4.91463, 4.50731, 6.43944	3.98312, 3	3	ppp4r4	1817
GL172686.1	67944	68474	68016, 68379, 68373	6, 9, 4	5.95712, 8.21099, 4.67325	4.64377, 5.77858, 3.78987	3.52158, 5	3	slitrk3	70840
GL172686.1	352068	352363	352109, 352091, 352071	10, 20, 10	9.44155, 20.22553, 11.10454	6.38646, 10.56212, 7.32977	6.72156, 1	3	mgam	63584
GL172686.1	667796	668313	668153, 668005, 667886	5, 13, 11	4.47183, 12.93015, 11.92945	3.78598, 7.96262, 7.69409	2.26935, 1	3	OTOL1	192421
GL172686.1	1198395	1199003	1198725, 1198474, 1198599	8, 16, 14	6.14488, 13.6893, 15.20637	4.62489, 7.72299, 9.10193	3.6868, 10	3	TRIM59	2533
GL172686.1	1390806	1390983	1390844, 1390846, 1390842	71, 115, 53	88.49621, 140.87363, 70.44074	26.98262, 30.98945, 26.20892	84.21545,	3	schip1	7388
GL172686.1	2034117	2034335	2034333, 2034333, 2034307	19, 22, 17	21.60968, 24.36754, 20.37708	11.67002, 12.41949, 11.43318	18.32833,	3	pqlc2l	46443
GL172686.1	2696923	2697246	2696999, 2696999, 2697114	7, 10, 5	6.01305, 7.8291, 4.64121	4.6447, 5.39387, 3.88372	3.56347, 5	3	kcnab1	0
GL172686.1	3076165	3076573	3076323, 3076277, 3076420	8, 15, 4	7.06928, 13.21475, 4.67325	5.19933, 7.68511, 3.78987	4.5414, 10	3	plch1	8075
GL172687.1	330902	332117	330950, 331038, 331181	4, 12, 7	3.21195, 9.70336, 7.40077	3.00807, 6.19346, 5.43746	1.31375, 7	3	fign	156152
GL172687.1	330902	332117	331715, 331038, 331181	9, 12, 7	8.55802, 9.70336, 7.40077	5.98518, 6.19346, 5.43746	5.8909, 7.1	3	fign	156152
GL172687.1	399724	400006	399888, 399911, 399959	6, 14, 6	4.9641, 11.94813, 6.08001	4.06411, 7.1463, 4.71063	2.64509, 9	3	kcnh7	165303
GL172687.1	600034	600318	600291, 600260, 600272	10, 25, 4	11.06011, 27.7306, 4.67325	7.306, 13.42198, 3.78987	8.22712, 2	3	kcnh7	0
GL172687.1	640241	641000	640945, 640370, 640647	82, 71, 49	85.18822, 66.90886, 52.18307	20.26382, 16.335, 17.69168	80.93923,	3	kcnh7	0
GL172687.1	770982	771424	771347, 771370, 771381	13, 19, 9	12.68462, 19.18794, 10.72164	7.81602, 10.23458, 7.15493	9.76298, 1	3	fap	0
GL172687.1	787209	787633	787552, 787517, 787592	8, 11, 21	6.94372, 7.9598, 26.58709	5.12297, 6.42066, 13.97388	4.42528, 7	3	fap	0
GL172687.1	891277	891711	891341, 891325, 891303	7, 12, 7	5.86458, 10.53798, 7.72582	4.55375, 6.71099, 5.6058	3.46146, 7	3	dpp4	0
GL172687.1	1014041	1014926	1014695, 1014560, 1014638	12, 13, 6	12.74986, 13.14514, 7.29403	8.02869, 8.09002, 5.30581	9.82533, 1	3	tbr1	28850
GL172687.1	1019689	1020509	1019926, 1019881, 1020380	11, 9, 11	11.71035, 8.21099, 12.9493	7.57196, 5.77858, 8.23866	8.8442, 5.7	3	tbr1	23267
GL172687.1	1144603	1144791	1144685, 1144698, 1144698	118, 163, 152	145.61365, 202.46124, 206.21291	31.71664, 35.10416, 42.16238	140.91216	3	tank	14009
GL172687.1	1433711	1434059	1433822, 1433822, 1433822	77, 115, 66	95.33519, 144.68781, 88.39466	27.54449, 32.77997, 29.6567	90.98476,	3	rbms1	55367
GL172687.1	1672513	1673591	1673096, 1672657, 1673125	13, 17, 13	14.97989, 17.04527, 16.70862	9.13126, 9.50106, 10.0169	11.94968,	3	pla2r1	0
GL172687.1	1748006	1748722	1748690, 1748519, 1748672	31, 33, 25	25.22782, 23.02867, 20.69641	9.91517, 8.27344, 9.27215	21.84101,	3	NA	11367
GL172687.1	1752840	1753140	1753052, 1752872, 1753056	12, 24, 13	6.84395, 13.93606, 8.37412	4.42933, 5.97199, 5.16916	4.33359, 1	3	NA	10961
GL172687.1	2087453	2088294	2087745, 2087511, 2087657	28, 50, 20	31.291, 55.6557, 22.54397	14.45526, 19.16385, 11.94229	27.75414,	3	tanc1	0
GL172687.1	2495790	2496735	2496154, 2496078, 2496124	11, 14, 6	10.1122, 13.14791, 6.168	6.63573, 7.88211, 4.75778	7.34229, 1	3	upp2	5668
GL172687.1	2560134	2560492	2560388, 2560465, 2560457	17, 18, 26	17.64386, 16.94269, 31.59632	9.85981, 9.16356, 15.35437	14.49984,	3	acvr1	14322
GL172687.1	2608148	2608487	2608436, 2608383, 2608323	10, 16, 6	10.33015, 16.22539, 6.50067	6.9035, 9.26486, 4.93052	7.54792, 1	3	acvr1	0
GL172687.1	2636772	2637307	2636991, 2637132, 2637169	9, 12, 9	9.72082, 10.53798, 9.19414	6.64182, 6.71099, 6.35176	6.97155, 7	3	acvr1	7285
GL172687.1	2673145	2673927	2673556, 2673446, 2673431	7, 12, 4	6.99564, 10.35, 4.55201	5.21786, 6.59497, 3.73489	4.47307, 7	3	acvr1c	17634
GL172687.1	2685171	2685890	2685676, 2685707, 2685409	7, 8, 6	6.74267, 7.07159, 6.12365	5.07552, 5.20073, 4.73409	4.23998, 4	3	acvr1c	5671
GL172687.1	3060624	3060855	3060747, 3060669, 3060660	6, 10, 4	5.06496, 7.38914, 4.18775	4.12592, 5.11603, 3.55872	2.73812, 4	3	gpd2	0
GL172687.1	3078702	3079097	3079047, 3079050, 3079071	31, 36, 22	37.87839, 36.08404, 25.77562	17.20438, 14.12934, 13.24605	34.21082,	3	gpd2	267
GL172687.1	3367598	3368807	3367917, 3368474, 3368150	7, 7, 3	6.64707, 5.71889, 3.24307	5.02073, 4.46356, 2.92359	4.15135, 3	3	nr4a2	210759
GL172687.1	3658824	3659384	3659281, 3659045, 3659000	8, 10, 6	8.42257, 7.93794, 7.29403	5.97763, 5.46232, 5.30581	5.76336, 5	3	nr4a2	501985
GL172687.1	3668812	3669368	3669341, 3669323, 3669339	30, 36, 20	36.04799, 42.5783, 23.73483	16.51403, 17.91845, 12.62984	32.41366,	3	nr4a2	511973
GL172688.1	514105	514559	514265, 514253, 514282	7, 17, 11	6.16939, 16.17826, 13.1143	4.73936, 8.97632, 8.32282	3.70916, 1	3	sp4	39450
GL172688.1	1420803	1421449	1421133, 1421161, 1421126	12, 24, 17	10.20921, 21.30261, 19.12895	6.50784, 9.9347, 10.73053	7.4341, 18	3	ferd3l	4631
GL172688.1	1928825	1929475	1929140, 1929173, 1929232	12, 18, 7	11.09909, 16.6391, 7.34973	7.05451, 8.9791, 5.41038	8.26391, 1	3	snx13	80364
GL172688.1	2294518	2295289	2294883, 2294883, 2294875	17, 29, 14	17.4139, 29.43697, 16.29325	9.72241, 12.97595, 9.7093	14.27855,	3	agr3	75070
GL172689.1	591369	591831	591563, 591555, 591507	6, 9, 5	5.96715, 8.01512, 5.9506	4.64927, 5.66016, 4.54784	3.52158, 5	3	arrdc4	216407
GL172689.1	1547450	1547897	1547559, 1547625, 1547655	6, 8, 4	5.68215, 6.93693, 4.15126	4.49007, 5.11882, 3.54016	3.29732, 4	3	mctp2	450711
GL172689.1	1563297	1563576	1563524, 1563536, 1563549	17, 22, 22	16.76548, 19.88511, 27.46822	9.33227, 9.73304, 14.21027	13.6543, 1	3	mctp2	435032
GL172689.1	2179995	2180481	2180319, 2180354, 2180344	6, 12, 6	5.51356, 9.99443, 6.45058	4.39314, 6.37457, 4.90508	3.14574, 7	3	NA	49321
GL172689.1	2579998	2580365	2580037, 2580037, 2580057	25, 19, 14	30.32096, 18.54823, 16.82614	14.94603, 9.84817, 9.99514	26.80639,	3	chd2	80501
GL172689.1	3009946	3010519	3010222, 3010094, 3009949	8, 11, 5	6.01859, 8.13814, 5.06966	4.54456, 5.40965, 4.11933	3.56839, 5	3	slco3a1	0
GL172689.1	3456720	3457207	3457057, 3456901, 3457033	10, 8, 7	8.66311, 7.06103, 6.96907	5.914, 5.19433, 5.20309	5.99101, 4	3	klhl25	18417
GL172690.1	10463	10951	10772, 10678, 10825	9, 12, 11	6.39666, 7.51974, 10.87462	4.646, 4.83844, 7.09143	3.92119, 5	3	vegfc	272247
GL172690.1	692665	693360	693135, 692851, 692877	15, 19, 7	15.56593, 18.08435, 6.83761	9.10751, 9.56723, 5.12939	12.50679,	3	NA	11600
GL172690.1	1200543	1201034	1200798, 1200736, 1200735	20, 16, 10	20.60651, 13.5443, 11.6791	10.79142, 7.63435, 7.62925	17.35705,	3	NA	39839
GL172690.1	1209662	1210155	1210124, 1210116, 1210119	12, 24, 8	12.18267, 26.67478, 9.60885	7.70145, 13.13685, 6.57807	9.29304, 2	3	NA	30718

GL172690.1	1506886	1507356	1507296, 1507295, 1507320	13, 11, 9	12.62236, 8.77819, 8.79325	7.77868, 5.81139, 6.12267	9.70369, 6	3	NA	13485
GL172690.1	2089657	2090876	2090044, 2090066, 2090076	26, 48, 20	25.74117, 47.43906, 20.43618	11.87968, 15.57108, 10.68898	22.33901,	3	aadat	370279
GL172690.1	3098802	3099040	3098879, 3098862, 3098828	9, 10, 8	8.55802, 7.55261, 8.74279	5.98518, 5.21944, 6.14792	5.8909, 5.1	3	NA	0
GL172690.1	3454464	3455461	3454938, 3454954, 3454866	26, 40, 26	28.77924, 39.3664, 29.30377	13.69314, 14.39261, 14.00545	25.30043,	3	spock3	0
GL172691.1	155508	155900	155661, 155779, 155669	9, 16, 8	8.35434, 14.92393, 8.9249	5.86444, 8.47764, 6.24211	5.71584, 1	3	grk7	29123
GL172691.1	240941	241335	241159, 241152, 241170	9, 11, 8	9.63662, 8.48203, 9.31951	6.59633, 5.62571, 6.43944	6.90556, 5	3	rasa2	1069
GL172691.1	389315	389632	389500, 389505, 389434	11, 14, 4	9.34791, 10.65218, 4.38152	6.16652, 6.34763, 3.65448	6.63394, 8	3	fbxo36	5244
GL172691.1	393117	393751	393289, 393284, 393510	13, 14, 7	11.55805, 10.80331, 7.72582	7.13125, 6.44051, 5.6058	8.70034, 8	3	fbxo36	1125
GL172691.1	524836	525517	525146, 524972, 525375	5, 12, 5	4.43696, 10.2136, 5.50079	3.76555, 6.51056, 4.33868	2.24017, 7	3	dner	0
GL172691.1	588455	588848	588598, 588787, 588787	8, 7, 5	7.19971, 5.61836, 5.06966	5.27799, 4.4008, 4.11933	4.63758, 3	3	dner	0
GL172691.1	728123	728766	728589, 728582, 728499	7, 9, 12	6.99564, 7.3205, 12.62857	5.21786, 5.23132, 7.95947	4.47307, 4	3	agfg1	0
GL172691.1	1242819	1243781	1243502, 1243475, 1243477	12, 22, 11	11.79509, 22.53781, 11.49087	7.47301, 11.3254, 7.44794	8.92495, 1	3	foxl2	35336
GL172691.1	1777659	1778128	1777759, 1778059, 1777988	7, 8, 8	7.1696, 7.07159, 10.1465	5.31345, 5.20073, 6.82176	4.61046, 4	3	tmtops.2	0
GL172691.1	1827010	1827554	1827525, 1827523, 1827418	19, 36, 18	21.11935, 40.26535, 21.11592	11.38438, 16.55262, 11.63307	17.85404,	3	tmtops.2	42496
GL172691.1	1936222	1938830	1937595, 1938072, 1937425	12, 28, 13	10.93921, 24.14746, 12.34408	6.95711, 10.2723, 7.61094	8.12344, 2	3	NA	11393
GL172691.1	1996582	1998690	1997338, 1997304, 1996740, 1998106	30, 50, 22, 15	21.25158, 37.92168, 17.73317, 9.95558	8.1687, 10.49548, 8.46917, 5.70594	17.98258,	3	st6gal1	34274
GL172691.1	1996633	1999281	1998837, 1997304, 1996740, 1998106	15, 50, 22, 15	7.05394, 37.92168, 17.73317, 9.95558	4.0775, 10.49548, 8.46917, 5.70594	4.52725, 3	3	st6gal1	33683
GL172691.1	1999544	2001493	1999574, 2000510, 2000456, 2001339	12, 23, 15, 9	4.51964, 9.57854, 9.08884, 4.14937	3.1022, 4.19604, 5.20063, 3.21957	2.31269, 6	3	st6gal1	31471
GL172691.1	1999916	2001750	2001062, 2000510, 2000456, 2001339	20, 23, 15, 9	10.13684, 9.57854, 9.08884, 4.14937	4.8616, 4.19604, 5.20063, 3.21957	7.36489, 6	3	st6gal1	31214
GL172691.1	2003643	2004882	2004392, 2004398, 2004099	14, 30, 11	9.16649, 23.3024, 8.03767	5.44587, 9.20329, 5.3465	6.46402, 2	3	st6gal1	28082
GL172691.1	2014959	2015892	2015628, 2015104, 2015488	15, 7, 4	14.78606, 5.97725, 4.67325	8.64215, 4.62287, 3.78987	11.76448,	3	st6gal1	17072
GL172691.1	2105807	2106696	2106058, 2106090, 2106099	8, 9, 4	7.52511, 7.21043, 4.67325	5.47115, 5.1623, 3.78987	4.94319, 4	3	cops9	0
GL172692.1	14716	15262	14787, 14999, 14849	11, 10, 8	9.71398, 6.93626, 9.31951	6.39253, 4.82903, 6.43944	6.97155, 4	3	NA	4944
GL172692.1	62184	63686	62836, 62580, 62804	12, 17, 14	10.34679, 14.4631, 16.37889	6.59299, 7.93171, 9.7558	7.56382, 1	3	scrn1	14471
GL172692.1	2749493	2753436	2751379, 2749629, 2751410, 2749599, 2752060	31, 14, 17, 13, 16	21.70152, 8.2158, 11.142, 8.08112, 11.29605	8.13717, 4.8857, 5.9621, 4.99269, 6.27684	18.41675,	3	npy	76128
GL172692.1	2753664	2754639	2754283, 2754119, 2754277	15, 9, 11	7.44225, 4.73471, 6.96055	4.28406, 3.58545, 4.67206	4.86624, 2	3	npy	74925
GL172692.1	2806605	2808632	2807676, 2807723, 2808249	19, 22, 17	15.14105, 18.27464, 14.82751	7.79671, 8.78311, 8.1535	12.1041, 1	3	npy	20932
GL172692.1	2808820	2809709	2809060, 2809056, 2809343	15, 21, 12	10.70853, 16.75487, 9.21464	6.15483, 8.18812, 5.88864	7.90562, 1	3	npy	19855
GL172693.1	1241192	1242011	1241593, 1241499, 1241744	5, 9, 3	4.82177, 7.79702, 3.47371	3.98509, 5.5269, 3.03189	2.51418, 5	3	sugct	32351
GL172693.1	1954023	1954577	1954363, 1954317, 1954256	4, 7, 4	3.42565, 5.48955, 4.67325	3.13796, 4.31982, 3.78987	1.48489, 3	3	mcm6	0
GL172693.1	2481416	2481619	2481571, 2481581, 2481597	322, 400, 230	422.64706, 518.47656, 320.4834	46.01178, 45.9046, 50.28881	417.17944	3	ikzf1	73907
GL172694.1	318056	318547	318345, 318202, 318345	18, 22, 9	14.55327, 16.31045, 7.94299	7.71735, 7.66148, 5.61625	11.54072,	3	nr2f1	201826
GL172694.1	322388	322934	322455, 322392, 322415	31, 37, 20	30.75727, 35.43217, 22.6299	12.99764, 13.3795, 11.99251	27.23282,	3	nr2f1	197439
GL172694.1	418467	419198	419146, 418727, 418832	7, 15, 6	7.1696, 15.80067, 7.29403	5.31345, 9.24574, 5.30581	4.61046, 1	3	nr2f1	101175
GL172694.1	450567	451081	450986, 450985, 450986	28, 22, 24	30.98295, 23.27801, 30.41517	14.27172, 11.76976, 15.30667	27.45364,	3	nr2f1	69292
GL172694.1	1177337	1177684	1177510, 1177523, 1177568	19, 27, 12	20.13535, 25.5193, 13.15613	10.80268, 11.39695, 8.25729	16.90088,	3	mctp1	0
GL172694.1	1407410	1408202	1407556, 1407519, 1407793	11, 5, 7	11.42621, 3.65953, 7.72582	7.41109, 3.2852, 5.6058	8.57514, 1	3	fam81b	0
GL172694.1	2031180	2031442	2031376, 2031311, 2031367	13, 27, 6	11.45737, 28.61693, 7.29403	7.06931, 13.23235, 5.30581	8.60415, 2	3	lnpep	15482
GL172694.1	2106365	2106971	2106889, 2106773, 2106811	6, 27, 11	4.9641, 26.58221, 11.31465	4.06411, 12.02229, 7.3472	2.64509, 2	3	lix1	0
GL172694.1	2209572	2209849	2209613, 2209611, 2209658	13, 11, 4	12.74759, 9.4823, 4.67325	7.85373, 6.24973, 3.78987	9.82421, 6	3	riok2	32640
GL172694.1	2495127	2496679	2495545, 2495308, 2496203, 2495362, 2496201	17, 8, 13, 17, 9	14.593, 5.38416, 10.7674, 18.35257, 7.86759	8.01072, 4.13667, 6.64292, 10.27929, 5.57018	11.57904,	3	NA	0
GL172694.1	2501382	2504470	2502012, 2502043, 2502041	13, 28, 15	8.81889, 21.37673, 12.50222	5.43946, 8.7647, 7.24756	6.13764, 1	3	NA	0
GL172694.1	2501383	2504578	2503604, 2502043, 2502041	20, 28, 15	15.90637, 21.37673, 12.50222	7.97271, 8.7647, 7.24756	12.83372,	3	NA	0
GL172694.1	2511763	2513278	2512640, 2511967, 2511841	21, 15, 11	15.0193, 8.49108, 7.3919	7.20041, 4.86073, 4.94122	11.9867, 5	3	NA	0
GL172694.1	2513808	2516068	2514961, 2515653, 2514613	16, 23, 19	9.96373, 17.72344, 16.90441	5.50201, 8.1803, 8.85275	7.20175, 1	3	NA	0
GL172694.1	2849841	2850420	2850075, 2850091, 2850023	10, 12, 10	10.33015, 9.09789, 12.71002	6.9035, 5.81578, 8.12734	7.54792, 6	3	st8sia4	50888
GL172695.1	380106	380480	380262, 380282, 380241	9, 9, 6	7.71701, 6.6123, 6.93817	5.47767, 4.78362, 5.14396	5.12244, 4	3	cnga1	6978
GL172695.1	787502	787971	787816, 787742, 787700	14, 21, 13	14.16942, 19.60696, 14.5443	8.49669, 9.87975, 8.89247	11.17387,	3	NA	86120
GL172695.1	1043193	1043527	1043450, 1043437, 1043478	6, 8, 4	5.96715, 7.07159, 4.67325	4.64927, 5.20073, 3.78987	3.52158, 4	3	gabra2	37189
GL172695.1	1115191	1115535	1115490, 1115302, 1115318	6, 11, 5	5.81608, 8.59076, 5.23257	4.56563, 5.69393, 4.20435	3.4176, 6.0	3	gabra2	0
GL172695.1	1375128	1375894	1375307, 1375478, 1375610	7, 14, 12	7.10297, 12.1395, 13.44151	5.27706, 7.26424, 8.41472	4.57191, 9	3	guf1	3338
GL172695.1	2460109	2460496	2460353, 2460359, 2460213	13, 16, 7	12.56081, 15.41438, 7.50542	7.74168, 8.77591, 5.49244	9.64511, 1	3	apbb2	0
GL172695.1	2496971	2497222	2497176, 2497159, 2497062	8, 11, 5	6.41335, 9.01418, 5.3628	4.7944, 5.9589, 4.27046	3.93678, 6	3	nsun7	1635
GL172695.1	2528942	2529407	2529226, 2529261, 2529220	22, 14, 8	23.44815, 11.49628, 9.1831	11.87164, 6.86757, 6.37229	20.11079,	3	nsun7	33606
GL172695.1	2952865	2953388	2952922, 2953202, 2953092	6, 6, 6	5.96715, 4.83043, 6.82211	4.64927, 3.98131, 5.08889	3.52158, 2	3	NA	0
GL172696.1	455141	455590	455541, 455578, 455564	25, 22, 16	27.82794, 20.6214, 18.9725	13.47995, 10.17315, 10.84874	24.37199,	3	NPY2R	58156
GL172696.1	954435	954812	954784, 954778, 954777	8, 17, 9	8.42257, 18.56115, 9.8849	5.97763, 10.40145, 6.72947	5.76336, 1	3	dchs2	0
GL172696.1	1209558	1210139	1209624, 1209898, 1209953	12, 25, 15	5.71497, 12.88097, 12.23277	3.76705, 5.31337, 7.0822	3.32872, 1	3	kiaa0922	1424
GL172696.1	1210423	1210964	1210934, 1210685, 1210719	15, 37, 10	8.2371, 23.96425, 6.85201	4.71868, 7.77924, 4.77562	5.60786, 2	3	kiaa0922	599
GL172696.1	1224420	1224949	1224514, 1224569, 1224493	18, 33, 15	11.92425, 23.36506, 11.2311	6.18373, 8.43073, 6.47057	9.04824, 2	3	kiaa0922	0
GL172696.1	1992322	1992920	1992516, 1992170, 1992662, 1992719	5, 10, 8, 5	4.43696, 7.65405, 5.40929, 5.23257	3.76555, 5.28351, 4.15294, 4.20435	2.24017, 5	3	NA	149005
GL172696.1	2029043	2030243	2029868, 2030028, 2030027	19, 20, 24	18.47044, 17.88727, 25.90456	9.80109, 9.1506, 12.67746	15.29425,	3	NA	111682
GL172696.1	2360780	2361185	2360996, 2360912, 2361012	19, 25, 15	8.03887, 9.06534, 8.39935	4.02159, 3.78469, 4.80926	5.42355, 6	3	sh3d19	51616
GL172696.1	2380465	2381384	2381229, 2381242, 2380542	21, 19, 12	13.83803, 8.71834, 7.69854	6.55469, 4.33635, 4.94791	10.86283,	3	sh3d19	31417
GL172696.1	2389601	2390049	2389712, 2389686, 2389679	20, 26, 15	11.48964, 13.42041, 8.75315	5.53364, 5.38645, 5.00881	8.63562, 1	3	sh3d19	22752
GL172696.1	2391778	2392141	2391933, 2391982, 2391959	27, 31, 18	17.95467, 17.31547, 11.38084	7.27921, 6.18423, 5.88019	14.79952,	3	sh3d19	20660
GL172696.1	2392785	2393105	2392984, 2392947, 2392918	21, 32, 20	12.05981, 17.86154, 13.3935	5.63125, 6.2338, 6.54389	9.17669, 1	3	sh3d19	19696
GL172696.1	2994466	2995200	2994931, 2994895, 2994814	11, 19, 6	12.43693, 21.39089, 7.29403	7.97018, 11.54296, 5.30581	9.52692, 1	3	dclk1	118859
GL172697.1	664757	665702	665298, 665259, 665277	39, 58, 33	29.635, 48.38133, 26.72123	9.78624, 12.62893, 10.08573	26.13572,	3	NA	17036
GL172697.1	666651	667250	666761, 666758, 666728	21, 27, 19	11.65132, 15.37787, 11.81744	5.42812, 6.07089, 5.90698	8.78944, 1	3	NA	18930
GL172697.1	2479072	2479440	2479304, 2479148, 2479238	7, 6, 4	7.1696, 4.93312, 4.26291	5.31345, 4.04501, 3.59641	4.61046, 2	3	NA	190251
GL172697.1	2584029	2585833	2584499, 2585169, 2585137	11, 21, 13	7.47983, 17.12071, 13.33541	4.99628, 8.40123, 8.20195	4.89998, 1	3	NA	83858
GL172697.1	2584029	2585833	2585689, 2585169, 2585137	13, 21, 13	9.48728, 17.12071, 13.33541	5.84994, 8.40123, 8.20195	6.76599, 1	3	NA	83858
GL172697.1	2709014	2709619	2709439, 2709382, 2709338	9, 12, 9	9.06007, 10.73343, 9.64095	6.27591, 6.83116, 6.59867	6.36394, 8	3	aipl1	18334
GL172698.1	1139977	1141857	1141445, 1140091, 1141475, 1140434, 1141750	14, 17, 19, 8, 7	12.43323, 12.26912, 13.88531, 7.76694, 6.55126	7.44494, 6.61547, 7.06303, 5.6116, 4.96529	9.52692, 9	3	sox3	162158
GL172698.1	3089614	3090179	3089848, 3089926, 3089830	15, 21, 5	15.41679, 19.48223, 5.9506	9.0192, 9.80473, 4.54784	12.36344,	3	NA	125827
GL172698.1	3105962	3106227	3106105, 3106161, 3106145	5, 11, 4	4.82177, 10.60895, 4.67325	3.98509, 6.9343, 3.78987	2.51418, 7	3	NA	109779
GL172698.1	3261813	3262311	3262213, 3262124, 3262202	5, 17, 7	4.82177, 17.33491, 8.646	3.98509, 9.67509, 6.04229	2.51418, 1	3	NA	24079
GL172698.1	3271528	3272512	3272190, 3271936, 3271926	9, 18, 8	8.35434, 18.22482, 8.6841	5.86444, 9.93951, 6.11715	5.71584, 1	3	NA	13878
GL172698.1	3280995	3282271	3281573, 3281537, 3281802	9, 20, 11	5.88466, 13.5634, 8.93374	4.31819, 6.63738, 5.90867	3.48105, 1	3	NA	4119
GL172699.1	112970	113249	113191, 113137, 113112	7, 8, 6	6.99564, 6.70176, 6.5517	5.21786, 4.97421, 4.95623	4.47307, 4	3	cdc5l	15456
GL172699.1	170411	170908	170518, 170525, 170519	9, 9, 4	8.40425, 7.79702, 4.67325	5.89416, 5.5269, 3.78987	5.76336, 5	3	cdc5l	22061
GL172699.1	196734	198479	197252, 196849, 197995, 198274	6, 5, 10, 3	5.24248, 3.65953, 8.44678, 3.47371	4.2332, 3.2852, 5.78021, 3.03189	2.89962, 1	3	NA	12500
GL172699.1	249862	250147	250119, 250038, 249921	8, 12, 6	7.15567, 8.75549, 6.03707	5.25151, 5.60227, 4.6874	4.61046, 6	3	MUT	0
GL172699.1	261040	261488	261445, 261195, 261446	18, 17, 8	17.38211, 16.97468, 7.76694	9.43026, 9.45853, 5.6116	14.24778,	3	MUT	0
GL172699.1	262789	263127	263032, 263023, 262924	7, 9, 8	4.74226, 6.37567, 6.83213	3.83953, 4.63258, 5.05462	2.50288, 4	3	MUT	0
GL172699.1	284047	284528	284188, 284304, 284229	8, 16, 14	7.57423, 14.52284, 14.16595	5.4999, 8.2328, 8.49463	4.98865, 1	3	MUT	0
GL172699.1	441791	442363	441808, 441811, 441821	65, 97, 46	84.88689, 122.87919, 62.14717	28.18601, 31.39716, 24.85453	80.6408, 1	3	NA	5720
GL172699.1	467401	468071	467687, 467624, 467961	7, 11, 5	6.33443, 10.21061, 5.23257	4.83795, 6.69533, 4.20435	3.86259, 7	3	NA	2068
GL172699.1	532112	532470	532171, 532159, 532173	16, 12, 9	17.59404, 10.12474, 10.72164	10.07113, 6.45547, 7.15493	14.45148,	3	XB-GENE-5870754	3882
GL172699.1	625074	625947	625650, 625662, 625257	6, 9, 3	5.77068, 6.96586, 3.30894	4.54016, 5.00812, 2.9554	3.37745, 4	3	NA	687
GL172699.1	672800	673237	672988, 673092, 673009	8, 17, 6	7.57423, 14.4143, 6.50067	5.4999, 7.90204, 4.93052	4.98865, 1	3	NA	0
GL172699.1	825813	826328	826096, 826208, 826175	7, 9, 4	7.1696, 8.21099, 4.67325	5.31345, 5.77858, 3.78987	4.61046, 5	3	NA	0
GL172699.1	906744	907288	907005, 907056, 907033	20, 37, 40	22.97335, 44.0032, 53.6198	12.19234, 18.32712, 22.6519	19.64963,	3	NA	6464
GL172699.1	1168528	1168823	1168604, 1168790, 1168776	5, 14, 6	4.54329, 13.63145, 7.29403	3.82752, 8.17488, 5.30581	2.33, 10.84	3	crisp2	17358
GL172699.1	1174382	1174684	1174651, 1174581, 1174651	12, 9, 12	11.73328, 8.21099, 14.96175	7.43625, 5.77858, 9.20442	8.86615, 5	3	crisp2	11497
GL172699.1	1261626	1262452	1261716, 1261857, 1262112	12, 16, 16	9.45689, 11.55244, 15.15882	6.0398, 6.42911, 8.62068	6.73641, 8	3	XB-GENE-5768874	0
GL172699.1	1264996	1265566	1265539, 1265556, 1265562	15, 27, 19	11.16655, 20.95947, 17.92404	6.43142, 8.82608, 9.47009	8.32779, 1	3	XB-GENE-5768874	0
GL172699.1	1267780	1268922	1267953, 1268025, 1268698	16, 22, 10	13.92435, 18.08067, 8.92941	7.86677, 8.67028, 6.07734	10.93921,	3	XB-GENE-5768874	0
GL172699.1	1285719	1286008	1285759, 1285905, 1285879	6, 6, 4	4.77375, 4.83043, 3.88239	3.9459, 3.98131, 3.39841	2.51418, 2	3	crisp2-like	254
GL172699.1	1294704	1295267	1294988, 1295016, 1294980	20, 30, 20	22.97335, 30.562, 24.55945	12.19234, 13.26188, 13.09374	19.64963,	3	crisp2-like	0
GL172699.1	1428094	1428379	1428343, 1428142, 1428142	23, 18, 12	23.09591, 15.53678, 14.40488	11.3315, 8.30996, 8.92522	19.76844,	3	NA	110312
GL172699.1	1434547	1435135	1434693, 1435090, 1434915	17, 15, 11	17.04639, 11.60543, 12.78973	9.50173, 6.69836, 8.15619	13.92543,	3	TFAP2D	115239
GL172699.1	1447847	1448276	1447927, 1447855, 1447885	24, 31, 13	28.41298, 35.54381, 16.49347	14.16116, 15.82346, 9.91245	24.94293,	3	TFAP2D	102098
GL172699.1	1475225	1476976	1476793, 1476035, 1476291	7, 11, 5	5.21879, 8.37597, 5.03069	4.14768, 5.5591, 4.09861	2.87895, 5	3	TFAP2D	73398
GL172699.1	1560076	1560661	1560263, 1560313, 1560288	14, 34, 18	13.09001, 35.34314, 21.39981	7.84687, 14.49755, 11.79257	10.14895,	3	TFAP2D	0
GL172699.1	1605917	1606918	1606658, 1606858, 1606863	6, 5, 8	5.63898, 3.78182, 9.31951	4.46544, 3.36365, 6.43944	3.25772, 1	3	tfap2b	0
GL172699.1	1636745	1637215	1637181, 1637170, 1637171	23, 37, 31	23.40734, 38.48862, 36.63922	11.51858, 15.10074, 16.4732	20.07125,	3	tfap2b	16286
GL172699.1	1639828	1640206	1639906, 1639885, 1639855	10, 16, 10	7.51175, 12.40931, 8.12251	5.19361, 6.94366, 5.57808	4.93097, 9	3	tfap2b	19369
GL172699.1	1649019	1649443	1649038, 1649097, 1649378	8, 6, 4	8.05102, 4.83043, 4.67325	5.77295, 3.98131, 3.78987	5.43473, 2	3	tfap2b	28560
GL172699.1	1731282	1731746	1731682, 1731714, 1731687	20, 38, 24	22.56332, 43.367, 30.6645	11.9536, 17.5141, 15.44595	19.25287,	3	tfap2b	110823
GL172699.1	1806703	1807060	1806965, 1806984, 1807020	7, 20, 14	7.1696, 22.87459, 19.03529	5.31345, 12.13503, 11.08274	4.61046, 1	3	pkhd1	139463
GL172699.1	1817780	1818442	1818253, 1818290, 1818276	16, 24, 18	14.23891, 22.6569, 20.32194	8.05921, 10.73418, 11.17944	11.24094,	3	pkhd1	128081
GL172699.1	1892015	1893146	1892395, 1892471, 1892444	9, 19, 4	8.77314, 20.1301, 4.67325	6.111, 10.79955, 3.78987	6.09355, 1	3	pkhd1	53377
GL172699.1	2176172	2176489	2176356, 2176356, 2176400	8, 11, 6	8.42257, 10.60895, 7.29403	5.97763, 6.9343, 5.30581	5.76336, 7	3	pkhd1	0
GL172699.1	2195324	2195563	2195480, 2195533, 2195518	11, 25, 11	9.05039, 22.53775, 10.5698	5.98149, 10.33209, 6.91098	6.35485, 1	3	pkhd1	0
GL172699.1	2198012	2198251	2198224, 2198210, 2198238	18, 17, 13	17.86195, 13.9447, 12.76267	9.72071, 7.61716, 7.86275	14.71061,	3	pkhd1	0
GL172699.1	2213888	2214282	2214210, 2214218, 2214252	12, 22, 11	12.97977, 22.22711, 12.48556	8.15868, 11.1385, 7.99611	10.04411,	3	pkhd1	0
GL172699.1	2384334	2385031	2384989, 2384998, 2384736	12, 15, 6	13.13892, 15.80067, 7.29403	8.24771, 9.24574, 5.30581	10.1962, 1	3	XB-GENE-971646	2264
GL172699.1	2405830	2406226	2406108, 2406140, 2406041	11, 13, 6	10.49148, 10.7674, 6.6567	6.86423, 6.64292, 5.00845	7.70044, 8	3	paqr8	10837
GL172699.1	2428213	2428757	2428542, 2428692, 2428286	13, 12, 5	15.29169, 11.86108, 5.9506	9.29854, 7.51216, 4.54784	12.24396,	3	paqr8	5005
GL172699.1	2478142	2478652	2478601, 2478619, 2478619	10, 14, 15	9.38842, 11.20037, 18.33148	6.35473, 6.68504, 10.66149	6.67167, 8	3	tram2	0
GL172699.1	2505774	2506222	2505868, 2505869, 2505910	8, 10, 7	5.89711, 6.60892, 5.34867	4.46697, 4.62161, 4.23056	3.49291, 4	3	xkr5	3679
GL172699.1	2507720	2508110	2508086, 2508049, 2508055	15, 25, 18	14.27412, 24.22828, 18.60267	8.3325, 11.32932, 10.16634	11.27364,	3	xkr5	5625
GL172699.1	2621319	2621824	2621462, 2621454, 2621460	74, 72, 34	86.41317, 81.86355, 42.39167	24.34133, 22.94922, 18.65397	82.15224,	3	gata4	25305
GL172699.1	2679688	2680338	2680197, 2679857, 2680014	6, 9, 7	5.43318, 7.83963, 8.08824	4.34622, 5.55305, 5.7849	3.07284, 5	3	BLK	48232
GL172699.1	2734760	2735327	2734786, 2734825, 2734772	25, 27, 8	30.32096, 28.88794, 9.1831	14.94603, 13.39413, 6.37229	26.80639,	3	BLK	0
GL172699.1	2767591	2768080	2767630, 2767617, 2767616	10, 23, 13	10.01299, 23.98856, 16.28606	6.7221, 11.86759, 9.81016	7.24763, 2	3	FAM167A	14583
GL172699.1	2785747	2786220	2785917, 2785887, 2785846	7, 6, 5	6.89193, 4.379, 4.91762	5.15998, 3.69486, 4.03768	4.37772, 2	3	FAM167A	0
GL172699.1	2810150	2811031	2810641, 2810442, 2810249	11, 6, 4	10.60582, 4.93312, 4.67325	6.93244, 4.04501, 3.78987	7.80831, 2	3	tdh	9002
GL172699.1	2821318	2822218	2821401, 2821366, 2821344	8, 11, 11	6.48409, 8.93411, 11.5511	4.83873, 5.9089, 7.48213	4.00135, 6	3	tdh	0
GL172699.1	2830324	2830734	2830621, 2830614, 2830588	18, 25, 19	18.76354, 24.22828, 20.10806	10.26255, 11.32932, 10.78641	15.57675,	3	tdh	5447
GL172699.1	2863926	2864309	2864024, 2864161, 2864255	5, 6, 6	4.33555, 4.45754, 5.83216	3.70555, 3.74538, 4.57461	2.15195, 2	3	xkr6	11716
GL172699.1	2875479	2875819	2875759, 2875762, 2875699	7, 6, 6	5.49154, 4.93312, 7.29403	4.32107, 4.04501, 5.30581	3.12578, 2	3	xkr6	206
GL172699.1	3011883	3012520	3011903, 3011900, 3011909	40, 52, 23	37.00959, 42.54112, 21.68625	13.12345, 11.88653, 10.48657	33.35794,	3	pinx1	0
GL172699.1	3014510	3015480	3014766, 3014884, 3014706	31, 40, 22	25.37889, 30.37232, 20.04483	9.99507, 9.85422, 9.82828	21.98739,	3	pinx1	0
GL172699.1	3053079	3054117	3053318, 3053203, 3053227	17, 24, 13	16.69696, 23.06143, 14.93222	9.29085, 10.97506, 9.10541	13.59308,	3	pinx1	0
GL172699.1	3072088	3072595	3072345, 3072212, 3072385	19, 12, 9	21.21506, 10.03748, 10.42289	11.44039, 6.40131, 7.00726	17.94655,	3	pinx1	3230
GL172699.1	3124964	3125255	3125068, 3125066, 3125036	7, 8, 8	7.15806, 6.27644, 8.51364	5.30717, 4.70819, 6.02666	4.61046, 3	3	sox7	29662
GL172699.1	3184928	3185449	3184975, 3185447, 3184962	7, 6, 4	7.1696, 4.93312, 4.67325	5.31345, 4.04501, 3.78987	4.61046, 2	3	msra.1	18543
GL172699.1	3348285	3349792	3348772, 3349279, 3349383	11, 13, 7	10.21742, 12.99541, 7.10639	6.69945, 8.00139, 5.27894	7.44132, 1	3	msra.1	3454
GL172699.1	3348285	3349808	3349404, 3349279, 3349383	8, 13, 7	7.06928, 12.99541, 7.10639	5.19933, 8.00139, 5.27894	4.5414, 10	3	msra.1	3454
GL172700.1	127699	128198	128111, 127763, 128013	8, 9, 4	6.34412, 6.9998, 4.26291	4.75087, 5.02958, 3.59641	3.871, 4.60	3	limk1	0
GL172700.1	1013138	1014662	1014165, 1013356, 1014133	16, 22, 12	11.66549, 15.85537, 10.80438	6.49652, 7.40917, 6.87466	8.80217, 1	3	NA	32066
GL172700.1	1019548	1020341	1019685, 1019756, 1019698	23, 26, 8	23.81148, 23.18389, 7.59222	11.76131, 10.37813, 5.5104	20.46387,	3	NA	26387
GL172700.1	1019642	1020673	1020146, 1020270, 1019698	8, 18, 8	6.14488, 14.10456, 7.59222	4.62489, 7.44949, 5.5104	3.6868, 11	3	NA	26055
GL172700.1	1027818	1028209	1027965, 1027917, 1028033	10, 11, 5	11.06011, 10.60895, 5.9506	7.306, 6.9343, 4.54784	8.22712, 7	3	NA	18519
GL172700.1	1037029	1038213	1037780, 1037892, 1037191, 1037890	11, 13, 5, 7	9.71398, 10.11663, 4.64121, 6.92462	6.39253, 6.23937, 3.88372, 5.17829	6.97155, 7	3	NA	8515
GL172700.1	1365561	1366270	1365667, 1365667, 1365959	11, 12, 8	9.52723, 11.66217, 8.6841	6.27749, 7.39386, 6.11715	6.80251, 8	3	pola1	4391
GL172700.1	1868027	1868581	1868300, 1868258, 1868231	21, 35, 11	21.10247, 36.30342, 12.2694	10.7812, 14.65322, 7.88012	17.83755,	3	NA	109360
GL172700.1	2000032	2001948	2000916, 2001016, 2001031	10, 17, 14	7.04101, 12.5204, 13.07989	4.89544, 6.76371, 7.8407	4.51536, 9	3	NA	158787
GL172700.1	2000277	2002723	2001546, 2001016, 2001031	13, 17, 14	10.10242, 12.5204, 13.07989	6.23056, 6.76371, 7.8407	7.3336, 9.7	3	NA	158012
GL172700.1	2744096	2745474	2744638, 2744582, 2744298	4, 8, 4	3.51298, 5.93025, 4.55201	3.1896, 4.48816, 3.73489	1.55088, 3	3	nr0b1	34145
GL172700.1	3223954	3224924	3224405, 3224503, 3223818, 3224577	7, 8, 4, 6	6.60038, 6.05185, 4.18775, 7.25145	4.99378, 4.56575, 3.55872, 5.287	4.10761, 3	3	dhrsx	0
GL172701.1	311265	311776	311713, 311568, 311750	29, 32, 13	32.03412, 33.07366, 14.5443	14.51808, 13.96479, 8.89247	28.48199,	3	hnrnpa0	4975
GL172701.1	531820	532037	532002, 531988, 532009	28, 44, 30	32.61062, 45.73992, 37.82916	15.23917, 16.28375, 17.55558	29.04561,	3	matr3	120864
GL172701.1	629381	629607	629578, 629581, 629565	6, 13, 7	5.47306, 11.2197, 8.42533	4.36955, 6.92276, 5.94313	3.10811, 8	3	matr3	218425
GL172701.1	705647	706043	705803, 705960, 705794	8, 18, 9	8.42257, 19.97713, 10.72164	5.97763, 10.97931, 7.15493	5.76336, 1	3	matr3	294691
GL172701.1	827421	828366	827804, 827770, 827554	13, 14, 6	13.34951, 13.0902, 6.45058	8.21021, 7.84698, 4.90508	10.39596,	3	NA	273807
GL172701.1	1066409	1067287	1066778, 1066821, 1066855	16, 23, 13	16.99938, 22.6679, 15.89249	9.72433, 11.0745, 9.61177	13.87966,	3	NA	34886
GL172701.1	1671048	1671433	1671096, 1671135, 1671092	7, 17, 9	6.79162, 16.49924, 10.56949	5.10336, 9.17114, 7.08032	4.28406, 1	3	pcdh1	274112
GL172701.1	1861380	1861966	1861794, 1861480, 1861573	9, 7, 5	8.55802, 5.61836, 5.9506	5.98518, 4.4008, 4.54784	5.8909, 3.3	3	pcdh1	464444
GL172701.1	2361445	2362454	2361682, 2361651, 2361652	27, 37, 29	29.0553, 38.94082, 35.86034	13.49404, 15.36076, 16.77935	25.57039,	3	XB-GENE-942501	466756
GL172701.1	2698350	2700142	2699147, 2699191, 2698817	10, 11, 4	8.93828, 9.05476, 4.38152	6.08275, 5.98422, 3.65448	6.24946, 6	3	XB-GENE-942501	129068
GL172701.1	2724863	2725445	2725249, 2725077, 2725260	5, 7, 5	4.47183, 5.61836, 5.85847	3.78598, 4.4008, 4.50665	2.26935, 3	3	XB-GENE-942501	103765
GL172702.1	1008876	1009590	1009224, 1009102, 1009188	15, 17, 9	18.26603, 18.56115, 11.64359	10.62691, 10.40145, 7.57973	15.09729,	3	NA	182236
GL172702.1	1188580	1189144	1188923, 1188962, 1188962	583, 625, 528	642.97491, 689.06152, 617.39929	31.27539, 31.38335, 35.99518	637.03528	3	NA	2682
GL172702.1	1713499	1714617	1714071, 1713898, 1713898, 1714286	11, 13, 3, 4	11.42621, 13.1283, 3.47371, 4.67325	7.41109, 8.08007, 3.03189, 3.78987	8.57514, 1	3	tk2	0
GL172703.1	694267	695153	694660, 694641, 694667	10, 20, 10	8.84451, 18.23344, 12.08015	6.02544, 9.35906, 7.82917	6.16137, 1	3	os9	10127
GL172703.1	781974	782960	782827, 782888, 782601	8, 9, 7	7.57423, 7.39577, 6.92462	5.4999, 5.27837, 5.17829	4.98865, 4	3	NA	9159
GL172703.1	1711980	1712957	1712092, 1712295, 1712012, 1712584	4, 12, 4, 3	3.74195, 11.86108, 4.15126, 3.17999	3.32091, 7.51216, 3.54016, 2.89245	1.6222, 9.1	3	pou6f1	13490
GL172703.1	2433139	2434521	2434395, 2433983, 2434164	15, 36, 10	14.98937, 34.76818, 10.71812	8.76428, 13.38886, 7.12015	11.95813,	3	scn8a	48764
GL172703.1	2781552	2782729	2781979, 2782627, 2782320	14, 9, 5	13.96392, 7.35794, 5.23257	8.37431, 5.25474, 4.20435	10.9769, 4	3	dip2b	11470
GL172704.1	173515	174108	173716, 173766, 173715	15, 11, 7	16.36908, 9.05476, 7.72582	9.57629, 5.98422, 5.6058	13.27778,	3	NA	0
GL172704.1	471941	472438	472106, 472114, 472145	12, 22, 8	11.61187, 20.947, 9.05167	7.3638, 10.3685, 6.30653	8.75096, 1	3	tyro3	0
GL172704.1	511516	513012	512044, 512544, 511902	9, 16, 11	9.00096, 13.49669, 10.47247	6.24222, 7.60526, 6.85286	6.30808, 1	3	mga	0
GL172704.1	1349183	1349627	1349592, 1349596, 1349574	36, 44, 16	42.81064, 53.1405, 20.763	18.05592, 20.52265, 11.79066	39.06365,	3	tmem260	37159
GL172704.1	1462662	1463560	1463439, 1463251, 1463370	6, 11, 3	5.96715, 9.71235, 3.47371	4.64927, 6.39153, 3.03189	3.52158, 7	3	tmem260	37777
GL172704.1	1828481	1829109	1828827, 1828701, 1828701	12, 20, 8	12.3187, 19.37003, 9.31951	7.78073, 10.0456, 6.43944	9.4231, 16	3	slc35f4	4161
GL172704.1	2412840	2413273	2412865, 2413082, 2413007	5, 9, 7	4.82177, 7.39577, 8.69489	3.98509, 5.27837, 6.06379	2.51418, 4	3	NA	54732
GL172704.1	2442439	2443022	2442640, 2442561, 2442791	8, 13, 4	8.42257, 11.07988, 4.67325	5.97763, 6.83637, 3.78987	5.76336, 8	3	NA	24983
GL172704.1	2488098	2488785	2488481, 2488491, 2488217	13, 14, 7	12.87577, 11.58379, 7.90186	7.93025, 6.92156, 5.69394	9.9455, 8.8	3	daam1	11970
GL172704.1	2753994	2755215	2754703, 2753859, 2754467, 2754464, 2754998	12, 17, 11, 4, 5	10.3005, 13.67787, 7.22061, 3.56693, 4.64121	6.56436, 7.45588, 4.83412, 3.22108, 3.88372	7.51999, 1	3	rtn1	0
GL172704.1	2879644	2879960	2879902, 2879915, 2879902	117, 79, 59	141.68561, 89.91457, 74.30872	30.40475, 23.75043, 25.32029	137.00615	3	pcnx4	414
GL172705.1	549331	549845	549716, 549524, 549582	8, 15, 7	6.59314, 11.45083, 6.59055	4.90679, 6.60415, 4.98809	4.10249, 8	3	rps19bp1	2746
GL172705.1	964095	964965	964460, 964593, 964498	8, 15, 9	6.86256, 15.26186, 9.94831	5.0733, 8.92711, 6.76298	4.3506, 12	3	ddx17	0
GL172705.1	1058858	1059562	1058933, 1058887, 1058891	19, 35, 8	20.21999, 36.21585, 9.1831	10.8531, 14.60259, 6.37229	16.98276,	3	ddx17	0
GL172705.1	1902569	1903432	1902765, 1902780, 1902965	8, 10, 9	7.28955, 7.32562, 10.35155	5.33177, 5.07581, 6.97128	4.72176, 4	3	pdc	1963
GL172705.1	2107502	2107952	2107763, 2107637, 2107636	6, 7, 7	5.5965, 4.94697, 8.21885	4.44108, 3.9726, 5.84717	3.21976, 2	3	fmo5	0
GL172705.1	2785961	2786430	2786000, 2786112, 2786003	5, 22, 3	4.40263, 23.36405, 3.47371	3.74533, 11.8213, 3.03189	2.21004, 2	3	atf6	2450
GL172705.1	3302340	3302711	3302489, 3302632, 3302604	11, 19, 7	10.1122, 16.20799, 6.59055	6.63573, 8.43311, 4.98809	7.34229, 1	3	prrx1	11015
GL172706.1	704838	705548	705309, 704900, 705399	8, 7, 5	8.28589, 5.93105, 5.9506	5.90321, 4.59459, 4.54784	5.65299, 3	3	DPYD	0
GL172706.1	1317869	1318763	1318378, 1318331, 1318208	8, 13, 6	7.06928, 12.25946, 6.08001	5.19933, 7.55969, 4.71063	4.5414, 9.5	3	xtr-mir-137-3	50766
GL172706.1	1373735	1374497	1374428, 1374402, 1374121	10, 9, 10	8.98596, 7.10358, 11.5283	6.11182, 5.09508, 7.55211	6.29482, 4	3	snx7	92948
GL172706.1	1562817	1563797	1563653, 1562989, 1563684	13, 20, 11	13.27928, 18.23344, 10.82241	8.16901, 9.35906, 7.0607	10.32936,	3	plppr5	0
GL172706.1	1740619	1741299	1740832, 1740825, 1740827	21, 34, 15	22.84886, 35.34314, 16.15968	11.82801, 14.49755, 9.45524	19.52948,	3	plppr4	12503
GL172706.1	1951363	1952104	1951780, 1951744, 1952058	8, 14, 4	7.1122, 12.97647, 4.30163	5.22529, 7.77766, 3.61556	4.57946, 1	3	arhgap29	11046
GL172706.1	2539185	2540634	2540136, 2540113, 2539889	26, 33, 27	24.6631, 30.50244, 27.00699	11.24163, 12.11651, 12.27383	21.29104,	3	tmed5	0
GL172707.1	1832289	1833238	1832820, 1832781, 1832579	11, 16, 11	12.01255, 15.04317, 11.79996	7.73996, 8.55029, 7.62212	9.13199, 1	3	tmem47	100787
GL172707.1	2233730	2234451	2234082, 2234004, 2234034	14, 21, 9	15.84929, 22.93552, 11.04428	9.46495, 11.87951, 7.30896	12.77923,	3	DMD	119032
GL172708.1	403294	404244	403623, 403634, 403675	21, 31, 23	22.84886, 31.60612, 27.39097	11.82801, 13.49298, 13.88084	19.52948,	3	hspb1	9332
GL172709.1	1141209	1141583	1141478, 1141476, 1141255	5, 7, 3	4.5799, 5.71889, 3.37781	3.84863, 4.46356, 2.98791	2.3629, 3.4	3	dnajc11	0
GL172709.1	1351816	1352720	1352164, 1352274, 1352177	14, 24, 6	11.06211, 19.76677, 6.03707	6.59982, 9.04653, 4.6874	8.22887, 1	3	dnajc11	125030
GL172709.1	1352907	1353824	1353398, 1353392, 1353345	17, 22, 8	14.01374, 17.52358, 8.4044	7.65897, 8.34854, 5.9678	11.0252, 1	3	dnajc11	126121
GL172709.1	2860326	2861798	2861048, 2860909, 2861113	9, 23, 7	7.84477, 19.70718, 6.92462	5.5562, 9.31653, 5.17829	5.24227, 1	3	tprg1l	11611
GL172709.1	3077372	3077738	3077559, 3077694, 3077620	10, 11, 6	11.06011, 10.60895, 7.05929	7.306, 6.9343, 5.20024	8.22712, 7	3	MEGF6	21391
GL172709.1	3218278	3219909	3219699, 3218971, 3219701	10, 11, 8	10.13706, 10.21061, 9.60885	6.7935, 6.69533, 6.57807	7.36489, 7	3	arhgef16	28754
GL172710.1	864214	864584	864485, 864315, 864504	11, 12, 6	10.06049, 10.58612, 6.168	6.60432, 6.74063, 4.75778	7.29334, 7	3	rnf141	53229
GL172710.1	913494	915874	914360, 914293, 914267	43, 43, 16	39.51486, 35.91962, 14.08695	13.34879, 11.57029, 7.96625	35.82062,	3	rnf141	1939
GL172710.1	927372	927904	927406, 927383, 927405	21, 23, 18	21.4094, 24.41444, 22.10559	10.96606, 12.12294, 12.18231	18.13434,	3	rnf141	1471
GL172710.1	934610	935250	934923, 934915, 934933	4, 11, 4	3.31554, 9.61892, 4.46488	3.07164, 6.33404, 3.69425	1.39756, 7	3	ampd3	0
GL172710.1	943838	944334	943849, 943883, 943970	27, 33, 16	27.00599, 34.64969, 17.2553	12.27323, 14.49247, 9.87431	23.57025,	3	ampd3	1736
GL172710.1	989902	990545	989930, 989918, 989940	11, 14, 11	11.49566, 14.22787, 12.48556	7.45067, 8.53137, 7.99611	8.64106, 1	3	ano5	14202
GL172710.1	1046817	1047501	1047237, 1047149, 1047148	9, 13, 10	8.88537, 12.37541, 12.08015	6.17591, 7.62989, 7.82917	6.19961, 9	3	slc17a6	0
GL172710.1	1209238	1209656	1209580, 1209529, 1209304	7, 5, 5	5.21879, 3.37233, 4.67395	4.14768, 3.09738, 3.90232	2.87895, 1	3	slc17a6	148725
GL172710.1	1420483	1421115	1420673, 1420797, 1420538	6, 8, 5	5.96715, 7.01915, 5.50079	4.64927, 5.16892, 4.33868	3.52158, 4	3	sos1	9101
GL172710.1	1909815	1910848	1910477, 1910514, 1910390	12, 19, 6	11.15354, 16.25859, 6.4014	7.08759, 8.46351, 4.8799	8.31649, 1	3	tmem178a	0
GL172710.1	2111547	2111935	2111655, 2111810, 2111882	6, 6, 5	5.63898, 4.93312, 5.27517	4.46544, 4.04501, 4.22616	3.25772, 2	3	NA	2904
GL172711.1	230831	231495	231401, 231228, 231286	26, 34, 13	25.01044, 32.13526, 13.58227	11.44656, 12.66514, 8.34597	21.62959,	3	lmx1b.2	39860
GL172711.1	481454	482507	482423, 482423, 481543, 482050	14, 12, 4, 4	14.52862, 11.60251, 3.82084, 3.82084	8.70881, 7.3582, 3.36473, 3.36473	11.51702,	3	wdr1	7376
GL172711.1	511163	511909	511363, 511592, 511233, 511664	4, 8, 6, 3	3.54306, 5.84201, 6.93817, 3.24307	3.2072, 4.43168, 5.14396, 2.92359	1.57602, 3	3	NA	2683
GL172711.1	511163	512011	511841, 511592, 511233, 511664	5, 8, 6, 3	4.40263, 5.84201, 6.93817, 3.24307	3.74533, 4.43168, 5.14396, 2.92359	2.21004, 3	3	NA	2683

GL172711.1	1298427	1299525	1298923, 1299430, 1298514	8, 13, 6	5.95727, 8.22922, 6.35308	4.50543, 5.08172, 4.85498	3.52158, 5	3	tspan14	49006
GL172711.1	1667455	1668212	1667921, 1668054, 1667945	18, 17, 8	19.8791, 14.71246, 8.6841	10.9221, 8.08343, 6.11715	16.65356,	3	nrg3	343992
GL172711.1	1731834	1732748	1732321, 1732213, 1732269	7, 6, 4	7.10297, 4.93312, 4.67325	5.27706, 4.04501, 3.78987	4.57191, 2	3	nrg3	279456
GL172711.1	1824508	1825241	1824839, 1824753, 1824558	22, 21, 10	24.67026, 23.50551, 13.18184	12.59865, 12.21671, 8.33771	21.29787,	3	nrg3	186963
GL172711.1	2064000	2064696	2064499, 2064608, 2064118	8, 10, 8	8.42257, 9.39128, 9.60885	5.97763, 6.35644, 6.57807	5.76336, 6	3	nrg3	0
GL172711.1	2943909	2944485	2944184, 2944346, 2944328	12, 11, 16	12.3187, 10.26356, 22.07892	7.78073, 6.72731, 12.42524	9.4231, 7.6	3	rgr	655
GL172712.1	1438544	1439383	1439336, 1439340, 1439301	10, 12, 5	9.44155, 10.53798, 5.9506	6.38646, 6.71099, 4.54784	6.72156, 7	3	edil3	48570
GL172712.1	2018345	2019151	2018707, 2018708, 2018950	18, 25, 11	18.15064, 23.88061, 13.64572	9.89485, 11.12286, 8.58592	14.98708,	3	NA	26678
GL172712.1	2070420	2071371	2070694, 2070935, 2071029	24, 30, 15	23.53058, 27.08037, 17.07266	11.25524, 11.25238, 9.97618	20.19159,	3	cox7c	35271
GL172712.1	2142512	2143048	2142697, 2142655, 2142774	5, 9, 6	4.33555, 7.92635, 6.08001	3.70555, 5.60609, 4.71063	2.15195, 5	3	cox7c	33266
GL172712.1	2159795	2160312	2159979, 2159897, 2159978	31, 47, 22	35.87363, 51.21941, 28.19009	16.0193, 18.03657, 14.60906	32.24278,	3	cox7c	50549
GL172712.1	2520096	2520389	2520366, 2520335, 2520361	19, 18, 14	18.81924, 15.90393, 15.06595	10.01206, 8.53251, 9.02131	15.63044,	3	tmem161b	96773
GL172713.1	14586	15069	14613, 14611, 14613	92, 127, 86	119.66629, 159.17163, 119.07999	32.44735, 33.50888, 35.6079	115.12064	3	nxph2	521838
GL172713.1	76690	76961	76924, 76930, 76908	21, 23, 15	23.92304, 24.15665, 17.40815	12.46182, 11.96843, 10.16282	20.57183,	3	nxph2	459946
GL172713.1	198135	198713	198592, 198702, 198553	12, 17, 14	13.8483, 18.56115, 19.68329	8.63436, 10.40145, 11.3696	10.86694,	3	nxph2	338194
GL172713.1	240884	241892	241475, 241473, 241044, 241460	9, 13, 6, 5	8.0679, 13.14514, 6.45058, 5.31857	5.69219, 8.09002, 4.90508, 4.24819	5.45, 10.37	3	nxph2	295015
GL172713.1	247837	248334	248254, 247911, 247930	7, 8, 9	6.2098, 6.73981, 10.14484	4.76362, 4.99774, 6.86555	3.74679, 4	3	nxph2	288573
GL172713.1	285711	286073	285823, 285927, 285927	11, 17, 6	11.56615, 15.63512, 7.29403	7.49066, 8.64575, 5.30581	8.70729, 1	3	nxph2	250834
GL172713.1	325456	326006	325536, 325487, 325457	10, 18, 6	11.06011, 18.15157, 7.29403	7.306, 9.89541, 5.30581	8.22712, 1	3	nxph2	210901
GL172713.1	410803	411701	411245, 411290, 411335	15, 27, 16	15.79643, 28.35229, 17.4758	9.24325, 13.07444, 10.00266	12.7283, 2	3	nxph2	125206
GL172713.1	472609	472950	472898, 472891, 472910	61, 57, 39	76.52258, 67.85107, 48.81665	25.53977, 22.66485, 20.28394	72.35973,	3	nxph2	63957
GL172713.1	488289	488894	488446, 488447, 488447	19, 33, 14	19.41153, 34.29488, 16.04334	10.36919, 14.28497, 9.57242	16.20286,	3	nxph2	48013
GL172713.1	527895	528517	527959, 527902, 527962	12, 20, 6	12.90199, 21.52275, 7.29403	8.11489, 11.33953, 5.30581	9.97033, 1	3	nxph2	8390
GL172713.1	541569	542580	541692, 542515, 541654	8, 7, 5	7.57423, 5.61836, 5.75061	5.4999, 4.4008, 4.45735	4.98865, 3	3	nxph2	3890
GL172713.1	579745	580158	579878, 579938, 580153	10, 10, 6	11.06011, 9.22453, 6.35308	7.306, 6.25634, 4.85498	8.22712, 6	3	spopl	12202
GL172713.1	636081	636445	636227, 636305, 636332	6, 8, 7	4.43199, 7.07159, 7.96278	3.72897, 5.20073, 5.72394	2.23764, 4	3	hnmt	48644
GL172713.1	678620	679501	679101, 678983, 679458	11, 22, 8	11.09349, 23.80675, 9.76268	7.21938, 12.0859, 6.64964	8.25916, 2	3	hnmt	5588
GL172713.1	778332	778666	778453, 778379, 778616	8, 8, 5	6.90289, 6.85668, 5.27517	5.09802, 5.06969, 4.22616	4.38737, 4	3	thsd7b	0
GL172713.1	858668	859146	858711, 858784, 858878	19, 21, 8	19.56679, 20.74722, 9.92329	10.46249, 10.56702, 6.7228	16.35231,	3	thsd7b	0
GL172713.1	870772	871144	871088, 871004, 870973	5, 8, 4	4.61714, 7.07159, 4.67325	3.86998, 5.20073, 3.78987	2.39372, 4	3	thsd7b	0
GL172713.1	957652	958117	957808, 957824, 957902	12, 8, 10	12.74986, 5.64525, 10.3632	8.02869, 4.30526, 6.92219	9.82533, 3	3	thsd7b	80472
GL172713.1	989970	990520	990241, 990142, 990162	8, 16, 13	7.67464, 16.0832, 16.49347	5.55832, 9.17962, 9.91245	5.08299, 1	3	thsd7b	112790
GL172713.1	1031118	1031577	1031441, 1031234, 1031275	10, 16, 4	10.39648, 13.98946, 4.67325	6.94096, 7.9066, 3.78987	7.61003, 1	3	NA	90595
GL172713.1	1085772	1086359	1085811, 1085776, 1085810	13, 16, 29	14.10549, 13.40251, 36.46839	8.64628, 7.54772, 17.13215	11.11201,	3	NA	35813
GL172713.1	1152403	1152735	1152532, 1152532, 1152565	15, 24, 13	17.09296, 26.09829, 16.70862	9.98755, 12.79322, 10.0169	13.96978,	3	cxcr4	12464
GL172713.1	1166603	1167194	1166878, 1166632, 1166638	9, 18, 5	8.61068, 14.90085, 5.06966	6.01615, 7.92604, 4.11933	5.94135, 1	3	dars	25942
GL172713.1	1183692	1183937	1183920, 1183910, 1183884	10, 8, 6	9.38842, 7.07159, 7.29403	6.35473, 5.20073, 5.30581	6.67167, 4	3	dars	9199
GL172713.1	1197820	1198408	1198240, 1198103, 1198162	5, 5, 7	4.73273, 3.94561, 7.55909	3.93547, 3.46715, 5.52035	2.49377, 1	3	dars	0
GL172713.1	1221434	1221952	1221716, 1221716, 1221749	11, 10, 5	10.49148, 7.79347, 5.50079	6.86423, 5.37143, 4.33868	7.70044, 5	3	dars	0
GL172713.1	1238170	1238578	1238511, 1238475, 1238501	6, 7, 3	5.96715, 5.97725, 3.47371	4.64927, 4.62287, 3.03189	3.52158, 3	3	dars	0
GL172713.1	1247355	1247942	1247914, 1247896, 1247914	68, 99, 38	82.57252, 123.25655, 50.22151	25.28785, 30.53763, 21.54693	78.35162,	3	mcm6.2	1564
GL172713.1	1369934	1370636	1369960, 1369982, 1369968	23, 35, 32	25.13729, 39.86979, 38.62642	12.55499, 16.74338, 17.24954	21.75229,	3	NA	2308
GL172713.1	1406442	1407120	1407066, 1406600, 1407096	14, 12, 11	12.59053, 9.66318, 10.05825	7.54151, 6.16843, 6.60296	9.67379, 7	3	slc11a1	3934
GL172713.1	1442780	1443157	1442925, 1442859, 1442870	10, 8, 9	11.06011, 7.07159, 11.64359	7.306, 5.20073, 7.57973	8.22712, 4	3	ctdsp1	0
GL172713.1	1449429	1450279	1449546, 1449572, 1449554	10, 11, 7	9.54985, 9.4823, 8.49722	6.45088, 6.24973, 5.97582	6.82341, 6	3	ctdsp1	4018
GL172713.1	1512107	1513786	1513446, 1513435, 1512256, 1513447	38, 53, 13, 28	36.31049, 53.48467, 11.25379, 31.9923	13.48556, 16.70765, 6.94381, 14.87246	32.67115,	3	cnot9	11946
GL172713.1	1544184	1544373	1544224, 1544199, 1544210	20, 35, 24	21.35298, 38.68467, 30.41517	11.23841, 16.0441, 15.30667	18.07874,	3	plcd4	0
GL172713.1	1581735	1582750	1582329, 1582122, 1582415	5, 6, 6	4.82177, 4.93312, 7.29403	3.98509, 4.04501, 5.30581	2.51418, 2	3	cyp27a1	0
GL172713.1	1629470	1630023	1629681, 1629681, 1629698	31, 34, 18	31.98677, 32.20527, 18.66986	13.71632, 12.7043, 10.20656	28.43586,	3	grsf1	4269
GL172713.1	1640654	1641257	1640791, 1640866, 1641114	13, 10, 6	10.58604, 7.4213, 6.4014	6.53049, 5.13639, 4.8799	7.78974, 4	3	NA	624
GL172713.1	1644983	1645420	1645193, 1645088, 1645177	5, 8, 4	3.23149, 5.2615, 3.82084	3.00362, 4.05724, 3.36473	1.33251, 3	3	NA	0
GL172713.1	1671671	1672117	1671906, 1672066, 1671901	29, 48, 18	28.61193, 48.0966, 19.75651	12.4907, 15.91578, 10.85036	25.13697,	3	NA	23524
GL172713.1	1673294	1674065	1673357, 1673658, 1673789	9, 17, 9	5.57413, 11.05412, 7.79368	4.11932, 5.91205, 5.52485	3.20013, 8	3	NA	25147
GL172713.1	1730941	1731165	1730942, 1730969, 1730944	16, 29, 17	15.87778, 27.98915, 18.6532	9.05608, 12.12692, 10.45515	12.8057, 2	3	wnt6	11051
GL172713.1	1741349	1741876	1741378, 1741434, 1741398	11, 20, 10	11.15818, 20.07667, 10.84317	7.25692, 10.47238, 7.18868	8.32008, 1	3	WNT10A	15159
GL172713.1	1758962	1759397	1759283, 1759204, 1759254	11, 9, 15	11.71035, 8.0603, 19.40672	7.57196, 5.68758, 11.21161	8.8442, 5.5	3	WNT10A	0
GL172713.1	1866071	1866917	1866308, 1866166, 1866260	9, 16, 8	7.59348, 13.44943, 7.85773	5.40134, 7.57638, 5.66361	5.0069, 10	3	cryba2	1620
GL172713.1	1876726	1877300	1877036, 1877255, 1876770	10, 13, 6	9.77513, 11.56107, 6.168	6.5837, 7.13311, 4.75778	7.02302, 8	3	cryba2	5924
GL172713.1	1891758	1892143	1892104, 1892116, 1892110	23, 36, 11	26.13432, 45.76297, 14.75772	13.14642, 19.79703, 9.09568	22.72107,	3	xtr-mir-375	10219
GL172713.1	1940203	1940614	1940364, 1940352, 1940284	24, 34, 16	20.59468, 30.86867, 16.972	9.52242, 11.96435, 9.70822	17.34584,	3	NA	2944
GL172713.1	2002424	2002846	2002767, 2002783, 2002815	9, 24, 8	6.89504, 19.8158, 7.00671	4.9633, 9.0745, 5.16135	4.38035, 1	3	ihh	0
GL172713.1	2039397	2039680	2039421, 2039464, 2039467	11, 7, 6	10.43535, 4.97304, 6.82211	6.83063, 3.98948, 5.08889	7.6474, 2.7	3	nhej1	0
GL172713.1	2044360	2044771	2044523, 2044470, 2044535	12, 16, 11	9.94519, 11.76475, 11.4314	6.34397, 6.55585, 7.41405	7.18444, 9	3	nhej1	0
GL172713.1	2051668	2052558	2052192, 2052220, 2052402	41, 45, 26	43.35231, 44.32098, 27.95127	16.21005, 15.10858, 13.1988	39.59722,	3	nhej1	0
GL172713.1	2080520	2081125	2080767, 2080780, 2080785	33, 22, 17	33.31306, 19.9442, 19.81591	13.71418, 9.76825, 11.12108	29.7331, 1	3	nhej1	0
GL172713.1	2091596	2092178	2091702, 2091898, 2091601	10, 18, 6	8.79841, 17.13102, 6.6037	5.99719, 9.27793, 4.9822	6.11782, 1	3	slc23a3	0
GL172713.1	2160549	2161129	2160866, 2160814, 2160647	11, 13, 5	11.85906, 11.41208, 5.69847	7.65504, 7.04141, 4.4331	8.98595, 8	3	cnppd1	0
GL172713.1	2222345	2222778	2222355, 2222349, 2222515	11, 23, 4	10.3799, 22.88804, 4.04585	6.79736, 11.20666, 3.48564	7.59574, 1	3	asic4	1134
GL172713.1	2231336	2231691	2231414, 2231357, 2231357	11, 20, 14	10.72304, 18.59503, 15.96227	7.00201, 9.57724, 9.52765	7.91902, 1	3	asic4	1653
GL172713.1	2240447	2240716	2240475, 2240461, 2240476	8, 9, 8	8.42257, 7.47261, 9.31951	5.97763, 5.32627, 6.43944	5.76336, 5	3	asic4	10764
GL172713.1	2289128	2289770	2289543, 2289381, 2289715	17, 15, 13	14.95512, 11.26316, 12.81733	8.23125, 6.49004, 7.8954	11.92636,	3	asic4	59445
GL172713.1	2290589	2291199	2291051, 2291076, 2291103	10, 13, 9	6.36591, 8.81099, 7.03548	4.46794, 5.43464, 5.05212	3.8925, 6.2	3	asic4	60906
GL172713.1	2312983	2313294	2313050, 2313011, 2313019	7, 15, 6	6.89193, 15.80067, 6.76586	5.15998, 9.24574, 5.06179	4.37772, 1	3	asic4	83300
GL172713.1	2319517	2319885	2319851, 2319808, 2319756	16, 20, 11	16.75964, 19.93044, 12.2694	9.5829, 10.38414, 7.88012	13.65293,	3	asic4	89834
GL172713.1	2345707	2346414	2345808, 2346233, 2345752, 2346289	6, 14, 4, 5	4.56865, 11.80872, 3.70359, 4.77515	3.81639, 7.06033, 3.29934, 3.9592	2.35429, 9	3	asic4	116024
GL172713.1	2356694	2357282	2357227, 2357194, 2357190	21, 32, 11	22.14207, 30.86283, 13.19894	11.40611, 12.68438, 8.36554	18.84387,	3	asic4	127011
GL172713.1	2378468	2378898	2378787, 2378588, 2378699	4, 7, 6	3.74195, 5.71889, 7.29403	3.32091, 4.46356, 5.30581	1.6222, 3.4	3	NA	139449
GL172713.1	2385785	2386284	2386047, 2386023, 2385938	11, 10, 7	9.90875, 8.44678, 8.08824	6.51186, 5.78021, 5.7849	7.14919, 5	3	NA	132063
GL172713.1	2406688	2407065	2407039, 2406979, 2407027	8, 17, 11	7.47668, 14.36585, 13.83651	5.44269, 7.8726, 8.67735	4.89713, 1	3	NA	111282
GL172713.1	2411211	2411432	2411309, 2411300, 2411365	15, 21, 5	17.6946, 21.26419, 5.9506	10.31995, 10.87863, 4.54784	14.54864,	3	NA	106915
GL172713.1	2442562	2442824	2442592, 2442590, 2442663	6, 15, 5	5.96715, 15.80067, 5.80393	4.64927, 9.24574, 4.48186	3.52158, 1	3	NA	75523
GL172713.1	2493971	2494398	2494245, 2494246, 2494216	17, 25, 11	15.39375, 19.82856, 10.10247	8.49863, 8.78546, 6.62982	12.34203,	3	NA	23949
GL172713.1	2497476	2498071	2497826, 2497706, 2497821	15, 20, 12	12.95198, 15.30488, 11.87227	7.52379, 7.62258, 7.51879	10.01762,	3	NA	20276
GL172713.1	2530406	2530674	2530490, 2530520, 2530569	8, 17, 9	8.34715, 17.87191, 11.64359	5.9367, 9.99549, 7.57973	5.71011, 1	3	NA	0
GL172713.1	2536561	2536850	2536813, 2536693, 2536813	5, 7, 5	4.82177, 5.97725, 5.91431	3.98509, 4.62287, 4.53172	2.51418, 3	3	NA	0
GL172713.1	2557971	2558703	2558432, 2558476, 2558446	10, 10, 5	8.48936, 7.29424, 5.23257	5.80661, 5.05593, 4.20435	5.82603, 4	3	NA	3100
GL172713.1	2565117	2565642	2565606, 2565575, 2565584	12, 14, 6	9.77685, 11.07823, 5.8719	6.23923, 6.60976, 4.59674	7.0243, 8.4	3	NA	112
GL172713.1	2746610	2747250	2746958, 2746909, 2746916	12, 17, 11	11.55224, 15.13134, 12.78973	7.3281, 8.33865, 8.15619	8.69537, 1	3	des.1	2817
GL172713.1	2976303	2976828	2976775, 2976720, 2976779	27, 24, 14	27.16312, 23.41175, 16.64329	12.36647, 11.1842, 9.89801	23.72391,	3	lct.2	0
GL172713.1	3057740	3058390	3058090, 3058090, 3058111	17, 13, 15	16.76548, 10.51368, 16.75372	9.33227, 6.48562, 9.79627	13.6543, 7	3	r3hdm1	16556
GL172713.1	3059010	3059305	3059245, 3059168, 3059045	6, 9, 6	4.27757, 6.64303, 5.56953	3.62922, 4.80319, 4.42554	2.10191, 4	3	r3hdm1	17826
GL172713.1	3082405	3083473	3083314, 3082663, 3082719	10, 10, 9	8.40516, 7.65405, 9.08997	5.75437, 5.28351, 6.2929	5.76336, 5	3	r3hdm1	41221
GL172713.1	3130855	3132282	3132027, 3132258, 3132182	13, 20, 12	11.98086, 16.44386, 10.84828	7.39024, 8.28902, 6.90153	9.10256, 1	3	NA	36867
GL172713.1	3155785	3156069	3155815, 3155913, 3155816	7, 12, 8	6.50909, 10.78353, 8.4586	4.94073, 6.86188, 5.99709	4.02371, 8	3	NA	13080
GL172713.1	3156950	3157841	3157406, 3157261, 3157365	8, 13, 5	7.99469, 12.31712, 5.40789	5.74128, 7.59462, 4.29296	5.38155, 9	3	NA	11308
GL172714.1	1392433	1393114	1392811, 1392791, 1392501	5, 8, 6	4.82177, 6.05185, 6.76586	3.98509, 4.56575, 5.06179	2.51418, 3	3	elavl2	29703
GL172715.1	608981	610066	609382, 609623, 609314	12, 14, 7	13.13892, 13.32457, 8.15288	8.24771, 7.98941, 5.81587	10.1962, 1	3	sh2d4a	89639
GL172715.1	1619360	1623708	1621621, 1620003, 1621776	57, 57, 44	41.80228, 40.63873, 34.56146	10.32367, 9.90439, 10.65017	38.07155,	3	antxr2	16662
GL172715.1	1802203	1802859	1802290, 1802492, 1802410	13, 12, 4	12.81131, 8.75549, 3.64768	7.8918, 5.60227, 3.26758	9.88323, 6	3	antxr2	57600
GL172715.1	1806207	1807052	1806849, 1806666, 1806861	8, 10, 6	6.94372, 7.97488, 5.99481	5.12297, 5.48553, 4.6644	4.42528, 5	3	antxr2	61604
GL172715.1	1993524	1995312	1993926, 1993553, 1994713	6, 7, 5	5.3939, 5.30663, 5.9506	4.32314, 4.20379, 4.54784	3.0374, 3.0	3	paqr3	55549
GL172715.1	1994416	1995426	1994941, 1995263, 1994713	4, 7, 5	3.57366, 5.55323, 5.9506	3.22499, 4.35994, 4.54784	1.59706, 3	3	paqr3	55435
GL172716.1	606916	607372	607147, 607181, 607166	8, 6, 5	7.83097, 4.76927, 5.9506	5.64832, 3.9431, 4.54784	5.22917, 2	3	NA	54529
GL172716.1	775812	777511	776863, 776473, 776657	12, 15, 20	12.45843, 14.39783, 24.55945	7.86167, 8.40758, 13.09374	9.54648, 1	3	rfc3	62906
GL172716.1	785673	786229	785675, 785737, 785844	29, 38, 16	30.87393, 38.24997, 16.70005	13.82751, 14.56148, 9.54762	27.34665,	3	rfc3	72767
GL172716.1	788078	788569	788322, 788333, 788115	8, 13, 6	5.69515, 8.75247, 5.20372	4.33737, 5.39893, 4.20995	3.30924, 6	3	rfc3	75172
GL172716.1	952163	952814	952613, 952531, 952587	20, 32, 10	19.35636, 31.45735, 10.97191	10.03733, 13.02561, 7.25854	16.14934,	3	pds5b	9023
GL172716.1	1234083	1234689	1234331, 1234529, 1234544	17, 27, 12	14.64358, 25.45196, 13.15613	8.0415, 11.35757, 8.25729	11.62752,	3	FRY	25312
GL172716.1	1441232	1443422	1441460, 1441966, 1441480, 1442232	5, 9, 4, 7	4.82177, 7.28344, 4.30163, 8.08824	3.98509, 5.20811, 3.61556, 5.7849	2.51418, 4	3	rxfp2	97935
GL172716.1	1529890	1530361	1530326, 1530298, 1530181	7, 12, 4	6.33443, 9.31599, 4.01199	4.83795, 5.95189, 3.46784	3.86259, 6	3	rxfp2	10996
GL172716.1	1741546	1742132	1742069, 1742073, 1742107	20, 42, 13	22.66399, 43.64236, 16.70862	12.01241, 15.95348, 10.0169	19.35097,	3	b3glct	0
GL172716.1	1823558	1824442	1823928, 1824010, 1823799	8, 21, 4	7.99469, 19.73353, 4.67325	5.74128, 9.95593, 3.78987	5.38155, 1	3	hsph1	621
GL172716.1	1823558	1824442	1824352, 1824010, 1823799	5, 21, 4	4.61714, 19.73353, 4.67325	3.86998, 9.95593, 3.78987	2.39372, 1	3	hsph1	621
GL172716.1	2019037	2019686	2019314, 2019304, 2019166	20, 50, 16	18.95669, 51.85011, 16.0646	9.79574, 17.08547, 9.16845	15.76377,	3	hmgb1	9250
GL172716.1	2189305	2190032	2189936, 2189937, 2189765	25, 30, 19	27.62793, 30.562, 23.01756	13.3608, 13.26188, 12.47022	24.17701,	3	ubl3	29493
GL172716.1	2293891	2294848	2294400, 2294382, 2294390	6, 11, 10	4.93136, 9.26343, 11.24131	4.04392, 6.11409, 7.40241	2.61555, 6	3	NA	9067
GL172716.1	2458079	2458518	2458455, 2458446, 2458459	48, 78, 39	52.18176, 83.8156, 44.1486	18.12946, 21.24998, 17.53404	48.29979,	3	mtus2	109224
GL172716.1	2459687	2459992	2459741, 2459700, 2459718	24, 33, 23	19.52596, 25.11168, 21.19111	8.90954, 9.27306, 10.19146	16.31324,	3	mtus2	110832
GL172716.1	3009344	3009724	3009557, 3009575, 3009602	21, 26, 13	21.48784, 26.07608, 16.28606	11.01327, 12.07894, 9.81016	18.20998,	3	gsx1	6846
GL172717.1	400554	401301	401184, 401260, 401202	15, 20, 6	14.39822, 18.2926, 6.4014	8.40782, 9.39473, 4.8799	11.39247,	3	fgf12	0
GL172717.1	1792424	1792692	1792555, 1792538, 1792492	4, 8, 9	3.74195, 6.11443, 10.14484	3.32091, 4.60556, 6.86555	1.6222, 3.7	3	lpp	0
GL172717.1	2509233	2510363	2509817, 2509893, 2509871	9, 16, 4	9.36986, 14.92393, 4.46488	6.44997, 8.47764, 3.69425	6.65425, 1	3	rtp3	1523
GL172717.1	2545569	2546870	2546537, 2546308, 2546220	9, 16, 9	9.72082, 15.54288, 11.58206	6.64182, 8.85379, 7.55286	6.97155, 1	3	NA	13712
GL172717.1	3121312	3121989	3121751, 3121751, 3121711	36, 39, 23	38.02184, 39.37589, 26.79607	15.24148, 14.79368, 13.53497	34.35174,	3	NA	8123
GL172718.1	26	756	229, 258, 279	4, 9, 6	3.54306, 8.21099, 6.99807	3.2072, 5.77858, 5.17195	1.57602, 5	3	NA	6313
GL172718.1	251763	252465	252005, 252059, 252045	6, 15, 7	4.48574, 14.58692, 8.15288	3.76346, 8.52203, 5.81587	2.28228, 1	3	ddb2	0
GL172718.1	453341	453797	453404, 453385, 453373	15, 16, 11	14.98937, 14.69174, 12.13051	8.76428, 8.33598, 7.80464	11.95813,	3	nat10	0
GL172718.1	668143	668385	668327, 668335, 668206	7, 12, 5	6.37715, 10.08092, 5.64747	4.86324, 6.42828, 4.40911	3.90257, 7	3	guca1b	786
GL172718.1	680422	680669	680629, 680636, 680617	24, 20, 15	26.57606, 19.50661, 16.91122	13.07811, 10.12815, 9.88541	23.15164,	3	guca1b	7328
GL172718.1	798082	799076	798467, 798575, 798632	9, 6, 7	9.70623, 4.93312, 8.69489	6.63396, 4.04501, 6.06379	6.97147, 2	3	NA	63541
GL172718.1	883625	884089	883653, 883651, 883633	15, 25, 14	15.87516, 26.42765, 16.73403	9.2894, 12.64351, 9.94634	12.80377,	3	NA	4090
GL172718.1	936196	937011	936459, 936430, 936433	8, 12, 8	8.34715, 11.37069, 9.92329	5.9367, 7.21896, 6.7228	5.71011, 8	3	shank2	4448
GL172718.1	947425	947876	947836, 947842, 947824	23, 34, 17	24.14755, 33.53045, 21.19882	11.96297, 13.45313, 11.87761	20.78974,	3	shank2	0
GL172718.1	962450	962898	962663, 962581, 962663	21, 21, 12	21.0274, 18.32622, 12.03712	10.73595, 9.11275, 7.61609	17.7649, 1	3	shank2	0
GL172718.1	1018323	1018716	1018426, 1018514, 1018656	6, 14, 5	5.96715, 14.45897, 5.9506	4.64927, 8.66788, 4.54784	3.52158, 1	3	shank2	0
GL172718.1	1038393	1039063	1038852, 1038745, 1038554	7, 11, 8	5.65516, 7.88157, 7.59222	4.42382, 5.2484, 5.5104	3.27228, 5	3	shank2	0
GL172718.1	1050934	1051362	1051273, 1051270, 1051292	26, 28, 19	27.54911, 25.18978, 20.4007	12.95841, 10.86232, 10.96052	24.09967,	3	shank2	0
GL172718.1	1058616	1059194	1059048, 1059029, 1058990	28, 46, 24	28.60673, 46.01146, 25.98406	12.85696, 15.61176, 12.72498	25.132, 42	3	shank2	0
GL172718.1	1209202	1209517	1209345, 1209335, 1209350	7, 8, 8	6.42049, 6.30984, 7.26744	4.8888, 4.72927, 5.31857	3.94161, 3	3	fadd	2858
GL172718.1	1271814	1272377	1272065, 1272064, 1272103	18, 21, 6	18.52782, 23.31142, 7.29403	10.1215, 12.1022, 5.30581	15.34982,	3	ccnd1	37623
GL172718.1	1285214	1286335	1285537, 1285671, 1285690	10, 16, 3	11.06011, 16.74813, 3.47371	7.306, 9.57609, 3.03189	8.22712, 1	3	ccnd1	51023
GL172718.1	1342903	1343727	1343158, 1343562, 1343413	10, 9, 5	9.33595, 7.10358, 4.64121	6.32332, 5.09508, 3.88372	6.62299, 4	3	ccnd1	108712
GL172718.1	1380421	1381027	1380688, 1380718, 1380724	53, 58, 27	58.70696, 57.685, 29.13465	19.48345, 16.83468, 13.54141	54.73514,	3	NA	137833
GL172718.1	1414534	1415473	1415361, 1415332, 1415339	8, 20, 11	7.72597, 18.97338, 13.37278	5.588, 9.80583, 8.45232	5.13061, 1	3	NA	103387
GL172718.1	1471167	1471634	1471593, 1471585, 1471469	15, 23, 9	14.46121, 22.81409, 10.35155	8.44598, 11.16226, 6.97128	11.45219,	3	NA	47226
GL172718.1	1489564	1490031	1489777, 1489804, 1489874	10, 13, 8	10.07458, 11.17268, 8.86315	6.75761, 6.89372, 6.21039	7.30652, 8	3	NA	28829
GL172718.1	1512586	1513148	1512751, 1512858, 1512877	11, 20, 10	9.90875, 19.10348, 11.60305	6.51186, 9.88447, 7.59048	7.14919, 1	3	NA	5712
GL172718.1	1633663	1634299	1634035, 1633830, 1634084	5, 6, 5	4.17642, 4.93312, 5.91431	3.60969, 4.04501, 4.53172	2.01422, 2	3	NA	35282
GL172718.1	1683814	1684629	1684109, 1684246, 1684455	15, 14, 10	11.13, 7.98747, 7.80277	6.40927, 4.75379, 5.37729	8.29395, 5	3	NA	0
GL172718.1	1781505	1782059	1781516, 1781596, 1781599	15, 16, 5	14.98937, 12.82211, 4.51488	8.76428, 7.19398, 3.81106	11.95813,	3	astl	8798
GL172718.1	1788022	1788611	1788557, 1788506, 1788142	10, 9, 4	8.89113, 6.76868, 3.73221	6.05396, 4.88312, 3.31545	6.20456, 4	3	astl	15315
GL172718.1	1844447	1844779	1844637, 1844529, 1844556	7, 7, 4	6.2098, 5.68499, 4.59709	4.76362, 4.44244, 3.75554	3.74679, 3	3	chst1	38969
GL172718.1	1852365	1852765	1852683, 1852444, 1852480	12, 9, 9	11.49331, 8.21099, 9.35579	7.29275, 5.77858, 6.44216	8.63898, 5	3	chst1	30983
GL172718.1	1860734	1861610	1860958, 1860947, 1861326	7, 19, 6	5.52352, 16.72984, 5.53406	4.34124, 8.74733, 4.40503	3.15454, 1	3	chst1	22138
GL172718.1	1878993	1879430	1879064, 1879218, 1879265	11, 17, 6	11.56615, 16.97468, 6.25896	7.49066, 9.45853, 4.80589	8.70729, 1	3	chst1	4318
GL172718.1	1962098	1962611	1962447, 1962507, 1962440	6, 7, 5	5.96715, 5.97725, 5.9506	4.64927, 4.62287, 4.54784	3.52158, 3	3	slc35c1	23308
GL172718.1	1981739	1982433	1981749, 1981767, 1981797	11, 30, 10	12.43693, 31.32456, 11.83521	7.97018, 13.71163, 7.70798	9.52692, 2	3	slc35c1	3486
GL172718.1	1988588	1988961	1988799, 1988781, 1988824	12, 19, 9	12.74986, 18.68597, 10.14484	8.02869, 9.9315, 6.86555	9.82533, 1	3	slc35c1	0
GL172718.1	2073849	2074303	2074222, 2074222, 2074207	23, 24, 13	23.72927, 25.20527, 15.52427	11.71195, 12.25879, 9.42125	20.38336,	3	pex16	419
GL172718.1	2204939	2205543	2205272, 2205251, 2205263	20, 18, 10	19.49401, 14.31692, 10.71812	10.12053, 7.57602, 7.12015	16.28237,	3	CREB3L1	64934
GL172718.1	2239984	2240840	2240545, 2240566, 2240612	9, 8, 9	8.40425, 6.97779, 9.82249	5.89416, 5.14375, 6.69629	5.76336, 4	3	CREB3L1	29637
GL172718.1	2258126	2258754	2258536, 2258522, 2258509	8, 11, 8	8.42257, 9.26343, 9.60885	5.97763, 6.11409, 6.57807	5.76336, 6	3	CREB3L1	11723
GL172718.1	2267688	2268550	2267949, 2267949, 2267949	18, 28, 11	18.00491, 29.57832, 12.34038	9.807, 13.43449, 7.91841	14.84768,	3	CREB3L1	1927
GL172718.1	2277697	2277996	2277763, 2277911, 2277811	4, 6, 3	3.74195, 4.93312, 3.2112	3.32091, 4.04501, 2.90793	1.6222, 2.7	3	CREB3L1	0
GL172718.1	2285988	2286406	2286307, 2286291, 2286296	16, 9, 11	15.09881, 7.75488, 13.46208	8.58417, 5.501, 8.49639	12.06341,	3	CREB3L1	5464
GL172718.1	2326951	2327175	2327115, 2327132, 2327115	8, 16, 8	6.55639, 13.78781, 8.74279	4.88389, 7.78324, 6.14792	4.06705, 1	3	dgkz	18395
GL172718.1	2355040	2355453	2355169, 2355223, 2355168	15, 18, 12	14.98937, 17.00494, 12.7555	8.76428, 9.20137, 8.03189	11.95813,	3	dgkz	0
GL172718.1	2403967	2404518	2404472, 2404350, 2404443	27, 51, 15	29.14937, 58.66246, 18.74072	13.5502, 20.38808, 10.87493	25.66204,	3	mdk	0
GL172718.1	2422115	2422630	2422502, 2422191, 2422337	11, 10, 4	10.49148, 7.75815, 4.64325	6.86423, 5.34918, 3.77643	7.70044, 5	3	NA	371
GL172718.1	2439926	2440228	2439953, 2439953, 2440025	9, 13, 9	8.88537, 11.92581, 9.76105	6.17591, 7.35664, 6.66343	6.19961, 9	3	NA	18182
GL172718.1	2458871	2459319	2459207, 2459177, 2459269	6, 12, 7	5.72604, 9.66318, 7.40077	4.51498, 6.16843, 5.43746	3.33547, 7	3	NA	37127
GL172718.1	2469089	2470832	2470380, 2470198, 2470177	8, 17, 8	7.15567, 14.86656, 7.50809	5.25151, 8.17729, 5.46116	4.61046, 1	3	NA	26469
GL172718.1	2521892	2522179	2521965, 2522028, 2522012	8, 10, 7	7.47668, 8.84031, 8.08824	5.44269, 6.02287, 5.7849	4.89713, 6	3	ambra1	4361
GL172718.1	2547878	2548933	2548119, 2548121, 2548198	10, 11, 7	11.06011, 10.60895, 8.69489	7.306, 6.9343, 6.06379	8.22712, 7	3	ambra1	0
GL172718.1	2625994	2626669	2626399, 2626451, 2626390	16, 21, 10	14.85827, 17.65171, 9.93124	8.4376, 8.71304, 6.67475	11.83222,	3	atg13	3346
GL172718.1	2636025	2636642	2636449, 2636444, 2636429	33, 59, 19	34.24909, 63.94751, 24.60107	14.25824, 19.59717, 13.32686	30.65114,	3	arhgap1	1536
GL172718.1	2644355	2645087	2644481, 2644567, 2644906	8, 13, 9	6.94372, 9.89237, 9.82249	5.12297, 6.1004, 6.69629	4.42528, 7	3	arhgap1	0
GL172718.1	2675835	2676340	2676079, 2675961, 2676175	5, 6, 7	4.82177, 4.93312, 8.69489	3.98509, 4.04501, 6.06379	2.51418, 2	3	f2	0
GL172718.1	3079656	3080007	3079702, 3079717, 3079703	11, 8, 7	8.69501, 4.79495, 6.2897	5.75929, 3.75444, 4.81137	6.02078, 2	3	ano1	22935
GL172718.1	3083716	3084130	3083782, 3083845, 3083935	8, 11, 7	6.27633, 7.46692, 6.83761	4.70812, 4.98819, 5.12939	3.80887, 5	3	ano1	26995
GL172718.1	3090909	3091495	3091242, 3091260, 3091288	21, 13, 8	19.03369, 8.17733, 7.81204	9.53542, 5.05049, 5.63749	15.83735,	3	ano1	34188
GL172718.1	3103660	3104000	3103760, 3103727, 3103763	14, 11, 11	13.44866, 8.37597, 13.28509	8.06455, 5.5591, 8.40871	10.49073,	3	ano1	46939
GL172719.1	79599	80111	79698, 79651, 79728	5, 8, 4	4.27048, 6.21066, 3.48964	3.6666, 4.66659, 3.17588	2.09564, 3	3	spidr	0
GL172719.1	414782	415332	415290, 415319, 415297	18, 27, 11	20.96944, 28.09375, 12.2694	11.5502, 12.92024, 7.88012	17.70889,	3	U4	10030
GL172719.1	1036216	1037070	1036755, 1036637, 1036457	5, 12, 6	4.20732, 10.25865, 7.18591	3.62846, 6.53846, 5.25776	2.04024, 7	3	sntg1	7554
GL172719.1	1594487	1595057	1594901, 1594894, 1594599	8, 17, 6	7.38183, 15.99248, 7.18591	5.38666, 8.86336, 5.25776	4.80856, 1	3	npbwr1	89629
GL172719.1	1876248	1877154	1877084, 1876812, 1876289, 1877027	16, 7, 6, 4	19.7934, 5.97725, 7.29403, 4.67325	11.29109, 4.62287, 5.30581, 3.78987	16.57067,	3	rgs20	26899
GL172719.1	1934051	1934407	1934270, 1934135, 1934096	5, 9, 5	4.82177, 8.21099, 5.9506	3.98509, 5.77858, 4.54784	2.51418, 5	3	rgs20	0
GL172719.1	2241045	2241456	2241243, 2241289, 2241289	7, 14, 6	6.84137, 13.20621, 6.87953	5.13151, 7.91755, 5.11627	4.33107, 1	3	NA	5176
GL172719.1	2533096	2533615	2533410, 2533324, 2533150	4, 5, 3	3.51298, 3.61259, 3.34298	3.1896, 3.25484, 2.97156	1.55088, 1	3	xkr4	7626
GL172719.1	2963620	2964213	2964182, 2963697, 2963907	7, 9, 3	6.09019, 6.64303, 3.47371	4.69155, 4.80319, 3.03189	3.63504, 4	3	impad1	66021
GL172720.1	107397	107701	107496, 107545, 107522	7, 13, 5	6.33443, 11.92581, 5.14967	4.83795, 7.35664, 4.16141	3.86259, 9	3	nol4	0
GL172720.1	1402362	1402657	1402411, 1402389, 1402391	10, 20, 14	10.39648, 19.93044, 18.64934	6.94096, 10.38414, 10.90476	7.61003, 1	3	cdh2	216165
GL172720.1	1495469	1496393	1495873, 1495911, 1496188	19, 26, 16	16.59925, 22.06964, 17.32802	8.66856, 9.74022, 9.91673	13.49961,	3	cdh2	122429
GL172720.1	1703520	1705573	1704452, 1704436, 1704014	25, 28, 16	23.05436, 23.29645, 14.60098	10.63488, 9.79917, 8.28055	19.72807,	3	enosf1	38745
GL172720.1	1706141	1707602	1707576, 1706216, 1706549	7, 10, 8	4.07942, 5.43447, 5.99585	3.40398, 3.88467, 4.53006	1.93039, 3	3	enosf1	36716
GL172720.1	1712783	1713573	1713253, 1713292, 1712977	16, 21, 15	19.76814, 22.31381, 18.95532	11.27773, 11.50891, 10.98488	16.54793,	3	enosf1	30745
GL172720.1	1720565	1722262	1720890, 1720896, 1720744	27, 43, 19	20.29562, 31.33255, 16.48621	8.47258, 9.50485, 8.60045	17.05596,	3	enosf1	22056
GL172720.1	1722283	1724114	1723522, 1723677, 1723523	21, 33, 15	14.59392, 21.90244, 12.27053	6.96516, 7.75874, 7.10536	11.57992,	3	enosf1	20204
GL172720.1	1729938	1731220	1730423, 1730577, 1730599	17, 23, 19	14.20139, 19.34487, 21.60402	7.77273, 9.10573, 11.66674	11.20483,	3	enosf1	13098
GL172720.1	1733095	1733686	1733430, 1733505, 1733149	14, 19, 7	9.22372, 13.14951, 5.42746	5.48009, 6.6433, 4.28057	6.5183, 10	3	enosf1	10632
GL172720.1	1734723	1736029	1735066, 1735470, 1735068, 1735952	10, 13, 6, 4	5.60999, 7.61839, 4.5515, 3.34447	3.99363, 4.71705, 3.80547, 3.08919	3.23297, 5	3	enosf1	8289
GL172720.1	1735446	1736746	1735989, 1735470, 1736316, 1735952, 1736703	12, 13, 7, 4, 5	8.2673, 7.61839, 3.50629, 3.34447, 4.88115	5.2988, 4.71705, 3.02547, 3.08919, 4.01777	5.63542, 5	3	enosf1	7572
GL172720.1	2750116	2750564	2750382, 2750207, 2750249	5, 11, 4	4.82177, 10.60895, 4.67325	3.98509, 6.9343, 3.78987	2.51418, 7	3	npca1	0
GL172721.1	166543	167644	167077, 167375, 167271	18, 18, 17	20.25794, 19.33283, 19.99824	11.14243, 10.60094, 11.2232	17.01944,	3	olfm3	33941
GL172721.1	179267	180707	180144, 180100, 180100	23, 56, 32	21.54503, 62.97164, 38.5144	10.40228, 20.43707, 17.1834	18.26496,	3	olfm3	46665
GL172721.1	670056	670523	670108, 670158, 670122	15, 23, 19	16.11735, 24.15665, 21.95763	9.43066, 11.96843, 11.8707	13.03694,	3	NA	58188
GL172721.1	2251742	2252767	2252095, 2252288, 2251961	10, 10, 8	7.9709, 8.66076, 7.85773	5.48303, 5.91256, 5.66361	5.36088, 6	3	tada1	2806
GL172721.1	2742360	2743023	2742525, 2742426, 2742918	7, 12, 3	6.01305, 10.73343, 3.47371	4.6447, 6.83116, 3.03189	3.56347, 8	3	hs2st1	47295
GL172722.1	282684	284134	283768, 283804, 283561	23, 30, 18	21.87577, 25.84858, 17.96175	10.59983, 10.56622, 9.78096	18.58593,	3	NA	96579
GL172722.1	939983	940515	940227, 940130, 940319	8, 11, 4	7.19971, 10.21061, 4.67325	5.27799, 6.69533, 3.78987	4.63758, 7	3	ptprd	0
GL172722.1	1552427	1552918	1552478, 1552502, 1552470	12, 14, 14	11.79509, 11.28333, 16.64329	7.47301, 6.73619, 9.89801	8.92495, 8	3	kdm4c	52728
GL172722.1	1590432	1591068	1590534, 1590660, 1590562	6, 11, 7	5.96715, 9.8562, 8.646	4.64927, 6.47973, 6.04229	3.52158, 7	3	kdm4c	14578
GL172722.1	2848604	2848967	2848631, 2848642, 2848636	14, 26, 13	15.31941, 27.47779, 15.34872	9.1665, 12.91577, 9.3288	12.27033,	3	rfx3	10956
GL172723.1	2000900	2001950	2001366, 2001267, 2001450	7, 15, 8	5.86458, 12.96377, 9.3897	4.55375, 7.53102, 6.47354	3.46146, 1	3	rnf180	0
GL172723.1	2029046	2029562	2029191, 2029474, 2029435	10, 18, 7	10.46385, 19.97713, 8.69489	6.97883, 10.97931, 6.06379	7.67431, 1	3	rnf180	15005
GL172723.1	2152799	2153519	2153047, 2153126, 2153091	5, 11, 5	4.36883, 8.66477, 4.84526	3.72533, 5.74034, 3.99806	2.18201, 6	3	htr1a	62162
GL172723.1	2253020	2254069	2253726, 2253701, 2253505	17, 15, 12	19.60089, 15.12096, 16.36823	10.9998, 8.84304, 9.85365	16.38573,	3	NA	4860
GL172723.1	2702512	2703622	2703086, 2703153, 2703347	14, 22, 8	12.8072, 18.08067, 8.14528	7.67424, 8.67028, 5.82558	9.88012, 1	3	zswim6	0
GL172723.1	2798141	2798495	2798420, 2798444, 2798440	10, 13, 7	8.44704, 10.55511, 7.45266	5.78037, 6.51132, 5.46481	5.7869, 7.9	3	zswim6	84584
GL172723.1	2846674	2846977	2846950, 2846943, 2846922	14, 9, 6	15.75768, 8.19918, 6.93817	9.41387, 5.77148, 5.14396	12.69046,	3	smim15	44606
GL172723.1	2983574	2984197	2983741, 2983658, 2983634	5, 7, 4	4.69353, 5.2482, 4.30163	3.9134, 4.16649, 3.61556	2.45909, 3	3	hps5	10334

GL172724.1	582413	583281	582976, 582696, 582837	8, 12, 6	7.67464, 10.12474, 6.6037	5.55832, 6.45547, 4.9822	5.08299, 7	3	baiap2l2	1883
GL172724.1	1137940	1138515	1138229, 1138229, 1138229	14, 24, 11	14.03163, 22.72317, 12.63525	8.41471, 10.77359, 8.07536	11.04222,	3	lgals2	0
GL172724.1	1205395	1205865	1205690, 1205621, 1205724	6, 12, 6	5.96715, 11.86108, 7.29403	4.64927, 7.51216, 5.30581	3.52158, 9	3	elfn2	27979
GL172724.1	1298356	1299049	1298881, 1298430, 1298445	9, 16, 5	8.16099, 12.82211, 5.06966	5.74847, 7.19398, 4.11933	5.53692, 1	3	NA	0
GL172724.1	1387948	1388870	1388115, 1388615, 1388604	9, 10, 5	8.77314, 7.79347, 5.31857	6.111, 5.37143, 4.24819	6.09355, 5	3	rac2	5067
GL172724.1	1543269	1543816	1543612, 1543472, 1543274	8, 22, 6	6.78332, 20.36917, 6.99807	5.02459, 10.02209, 5.17195	4.27811, 1	3	tst	12581
GL172724.1	3019721	3020128	3019885, 3019889, 3019887	193, 225, 98	237.81453, 269.56467, 129.85327	35.791, 34.47975, 34.49033	232.67651	3	NA	4961
GL172725.1	272611	273318	272912, 272979, 272887	14, 11, 11	13.96392, 9.05476, 12.9493	8.37431, 5.98422, 8.23866	10.9769, 6	3	NA	94203
GL172725.1	465432	466329	465812, 465809, 465871	23, 22, 11	24.49582, 20.36917, 10.87462	12.17168, 10.02209, 7.09143	21.12838,	3	enpep	11294
GL172725.1	2200805	2201992	2201033, 2201006, 2200995, 2201700	26, 35, 14, 14	20.58586, 24.1999, 10.81929, 10.8526	8.91213, 8.32847, 6.45034, 6.47083	17.33744,	3	ndst3	163643
GL172726.1	115192	115619	115367, 115368, 115537	10, 14, 6	10.67246, 13.38467, 5.75442	7.09497, 8.02582, 4.53101	7.87096, 1	3	aga	86686
GL172726.1	132141	132736	132545, 132546, 132628	10, 20, 7	8.66311, 21.43553, 7.29952	5.914, 11.28761, 5.38357	5.99101, 1	3	aga	103635
GL172726.1	292963	293613	293089, 293206, 293142	5, 14, 9	4.82177, 14.45897, 10.87975	3.98509, 8.66788, 7.23113	2.51418, 1	3	aga	264457
GL172726.1	784375	785041	784605, 784670, 784660	14, 21, 11	14.03163, 23.70392, 12.48556	8.41471, 12.3334, 7.99611	11.04222,	3	tenm2	205657
GL172726.1	1398037	1398867	1398786, 1398794, 1398828	28, 25, 22	32.04276, 23.54439, 29.52994	14.90243, 10.9238, 15.32589	28.49042,	3	NA	67479
GL172726.1	1661247	1661611	1661455, 1661364, 1661385	6, 6, 5	5.96715, 4.93312, 5.59755	4.64927, 4.04501, 4.38538	3.52158, 2	3	dctd	96044
GL172726.1	1708970	1710023	1709280, 1708997, 1709605	6, 8, 9	5.81608, 6.89656, 10.14484	4.56563, 5.09414, 6.86555	3.4176, 4.5	3	dctd	47632
GL172726.1	2626334	2627452	2627032, 2627178, 2627044	10, 15, 10	9.95227, 13.92002, 10.53703	6.68696, 8.11689, 7.01977	7.19087, 1	3	SNX25	8602
GL172726.1	2685832	2686052	2685883, 2685943, 2686006	6, 13, 8	5.86227, 11.51094, 9.92329	4.59138, 7.10228, 6.7228	3.46055, 8	3	SNX25	0
GL172727.1	47013	47576	47179, 47456, 47101	15, 25, 22	16.81189, 26.34052, 26.90598	9.82925, 12.59134, 13.89421	13.69977,	3	iqcd	7149
GL172727.1	175365	176561	176236, 175881, 176214	51, 42, 38	59.69558, 44.66452, 46.4678	20.97463, 16.52813, 19.34346	55.7112, 4	3	rita1	19203
GL172727.1	184969	185484	185200, 185189, 185143	19, 32, 29	20.05161, 34.91565, 36.72021	10.75274, 15.04994, 17.27746	16.81949,	3	rita1	10280
GL172727.1	2138214	2138653	2138366, 2138367, 2138381	17, 24, 14	18.21101, 23.06143, 16.12551	10.19603, 10.97506, 9.61761	15.04611,	3	OR11L1	13856
GL172727.1	2627818	2628237	2627958, 2628032, 2627875	8, 11, 6	6.86256, 9.05476, 5.99481	5.0733, 5.98422, 4.6644	4.3506, 6.5	3	adcy4	20838
GL172727.1	2649533	2649911	2649842, 2649863, 2649858	9, 9, 5	8.25643, 7.79702, 5.75061	5.80588, 5.5269, 4.45735	5.62516, 5	3	nfatc4	29565
GL172728.1	19929	20525	20369, 20074, 20077	12, 12, 14	13.8483, 11.60251, 16.29325	8.63436, 7.3582, 9.7093	10.86694,	3	NA	131204
GL172728.1	26044	26686	26366, 26617, 26600	7, 9, 8	5.33678, 6.86591, 6.50832	4.22299, 4.94483, 4.85389	2.98597, 4	3	NA	125043
GL172728.1	48501	49344	48903, 48712, 48631	9, 6, 8	9.72082, 4.93312, 9.46129	6.64182, 4.04501, 6.50801	6.97155, 2	3	NA	102385
GL172728.1	103634	104552	104467, 104464, 104467	6, 13, 10	5.3939, 11.71436, 11.24131	4.32314, 7.22722, 7.40241	3.0374, 9.0	3	NA	47177
GL172728.1	149737	150115	149819, 150055, 149833	9, 11, 5	9.72082, 10.53778, 5.9506	6.64182, 6.89189, 4.54784	6.97155, 7	3	NA	1614
GL172728.1	174071	174834	174284, 174305, 174592	10, 10, 4	10.53228, 9.39128, 4.42274	7.01712, 6.35644, 3.67426	7.73927, 6	3	NA	12299
GL172728.1	181623	182250	181798, 181936, 181680	7, 8, 6	7.1696, 7.07159, 6.6567	5.31345, 5.20073, 5.00845	4.61046, 4	3	NA	19851
GL172728.1	193290	194371	193629, 193629, 193884	19, 19, 24	21.50921, 18.6168, 29.47401	11.61175, 9.88966, 14.77381	18.23049,	3	mrpl4	30007
GL172728.1	328965	330557	329820, 329519, 329155, 329609	13, 12, 4, 9	11.76528, 10.93692, 3.88239, 10.01275	7.25843, 6.95571, 3.39841, 6.79683	8.8968, 8.2	3	s1pr2	11891
GL172728.1	367306	367876	367863, 367843, 367848	68, 75, 66	84.52832, 89.08515, 86.14899	26.36824, 25.24455, 28.36246	80.28592,	3	dnmt1	1439
GL172728.1	510494	511370	510743, 511141, 510979	7, 12, 11	6.25077, 11.72254, 13.1143	4.78814, 7.42986, 8.32282	3.78441, 9	3	COL5A3	17781
GL172728.1	536274	537427	537014, 536427, 537091	7, 12, 10	5.901, 8.82199, 10.47831	4.57615, 5.64371, 6.98694	3.49615, 6	3	COL5A3	43561
GL172728.1	561007	561518	561223, 561237, 561185	13, 22, 6	11.81837, 19.82642, 6.93817	7.29094, 9.69808, 5.14396	8.94738, 1	3	rdh8	28729
GL172728.1	631249	632577	631795, 631819, 631830	13, 22, 11	11.26158, 19.82642, 13.0311	6.94861, 9.69808, 8.28053	8.41898, 1	3	c19orf66	14761
GL172728.1	652236	652710	652428, 652443, 652413	7, 9, 5	6.25077, 7.24675, 5.50079	4.78814, 5.1851, 4.33868	3.78441, 4	3	c19orf66	0
GL172728.1	681242	681758	681442, 681447, 681448	12, 21, 6	12.05017, 22.75339, 7.29403	7.62377, 11.77121, 5.30581	9.16715, 1	3	ANGPTL6	17055
GL172728.1	686774	686984	686876, 686957, 686931	8, 18, 5	8.42257, 19.97713, 5.9506	5.97763, 10.97931, 4.54784	5.76336, 1	3	ANGPTL6	22587
GL172728.1	1298196	1299397	1298434, 1298724, 1299187, 1298437	7, 9, 6, 4	6.09019, 7.13885, 4.20515, 4.67325	4.69155, 5.11729, 3.5821, 3.78987	3.63504, 4	3	NA	7369
GL172728.1	2955336	2956497	2955571, 2955585, 2955584	104, 159, 101	104.79361, 155.0929, 116.26542	20.33954, 20.69795, 26.24432	100.36537	3	polr2j	13496
GL172729.1	741766	742056	742028, 742030, 742024	19, 26, 17	21.50921, 27.83582, 21.41937	11.61175, 13.1298, 11.99417	18.23049,	3	TENM2	257577
GL172729.1	834381	835015	834622, 834597, 834631	10, 20, 9	10.07458, 19.43804, 10.28145	6.75761, 10.0867, 6.93568	7.30652, 1	3	NA	233029
GL172729.1	1268868	1269310	1269037, 1269021, 1269183	11, 11, 6	11.02968, 9.95469, 6.99807	7.18222, 6.5399, 5.17195	8.20839, 7	3	NA	172982
GL172729.1	1701675	1702853	1702296, 1702251, 1702232	15, 22, 11	12.43022, 19.04305, 11.6739	7.20335, 9.23371, 7.55148	9.52692, 1	3	mat2b	386966
GL172729.1	1946148	1946733	1946702, 1946723, 1946648	12, 19, 15	11.98519, 18.8972, 17.58264	7.58551, 10.05916, 10.25879	9.10644, 1	3	mat2b	143086
GL172729.1	2896447	2896755	2896620, 2896602, 2896621	25, 32, 15	27.82794, 33.15993, 16.99143	13.47995, 14.01532, 9.93059	24.37199,	3	pdlim4	46875
GL172730.1	1684618	1685409	1684722, 1684683, 1685259	12, 15, 10	8.17829, 10.53542, 9.35556	5.24366, 6.05093, 6.33507	5.55261, 7	3	tshz3	109277
GL172730.1	1687469	1688529	1687928, 1687936, 1687934	16, 23, 8	12.5071, 19.4984, 7.19087	7.00284, 9.19489, 5.27268	9.59366, 1	3	tshz3	106157
GL172731.1	390595	392654	391209, 390806, 391843	7, 15, 12	5.52352, 10.12746, 12.82015	4.34124, 5.80768, 8.0686	3.15454, 7	3	gad1.2	21864
GL172731.1	390595	392654	391790, 390806, 391843	10, 15, 12	8.66311, 10.12746, 12.82015	5.914, 5.80768, 8.0686	5.99101, 7	3	gad1.2	21864
GL172731.1	398177	399884	398995, 399001, 398974	60, 97, 42	40.24226, 67.92493, 32.80255	9.17273, 10.64746, 10.41274	36.53614,	3	gad1.2	29446
GL172731.1	404063	404535	404369, 404231, 404201	45, 60, 19	25.78695, 32.50518, 10.14279	6.9986, 6.90222, 5.03155	22.38388,	3	gad1.2	35332
GL172731.1	406790	407401	406997, 407030, 406943	22, 34, 13	8.59279, 13.8668, 6.67904	3.90841, 4.52768, 4.17117	5.92419, 1	3	gad1.2	38059
GL172731.1	412237	413468	412959, 413000, 413008	37, 67, 23	26.39902, 46.75674, 17.85719	8.83235, 9.93538, 8.2554	22.97868,	3	gad1.2	43506
GL172731.1	466900	467443	467216, 467153, 467336	8, 12, 4	7.15567, 9.74388, 4.22496	5.25151, 6.2187, 3.57747	4.61046, 7	3	gad1.2	98169
GL172731.1	472075	472401	472292, 472250, 472143	6, 9, 6	5.43318, 7.10358, 7.18591	4.34622, 5.09508, 5.25776	3.07284, 4	3	gad1.2	103344
GL172731.1	1102759	1103489	1103097, 1103108, 1103148	10, 17, 5	9.54985, 16.24129, 5.3628	6.45088, 9.01462, 4.27046	6.82341, 1	3	PIGN	0
GL172731.1	1326175	1328185	1327430, 1327693, 1327427	18, 28, 15	15.61903, 23.14394, 14.9375	8.35978, 9.71527, 8.73318	12.55806,	3	NA	6660
GL172731.1	2591484	2592021	2591718, 2591849, 2591605	14, 21, 6	12.86267, 18.48988, 5.91225	7.70816, 9.21025, 4.61907	9.93295, 1	3	foxf2	9369
GL172732.1	175120	175500	175348, 175255, 175300	10, 22, 10	9.661, 21.85303, 11.5283	6.51661, 10.91337, 7.55211	6.9283, 18	3	osr2	1221
GL172732.1	290703	291062	290727, 290730, 290732	24, 26, 13	25.54351, 25.25783, 14.85247	12.46143, 11.59292, 9.06201	22.14693,	3	vps13b	0
GL172732.1	1446441	1446623	1446481, 1446485, 1446448	126, 139, 97	162.3154, 172.68352, 131.20084	35.47511, 33.71759, 35.79485	157.52225	3	rbm12b	108115
GL172732.1	1593587	1594055	1593828, 1593724, 1593790	4, 7, 7	3.57366, 5.8946, 8.08824	3.22499, 4.57222, 5.7849	1.59706, 3	3	triqk	0
GL172732.1	1678289	1678980	1678496, 1678517, 1678523	8, 12, 8	7.99469, 11.84549, 10.1465	5.74128, 7.50292, 6.82176	5.38155, 9	3	triqk	83154
GL172732.1	1706732	1707190	1706819, 1706892, 1706818	19, 13, 12	17.87921, 8.66599, 12.03712	9.44292, 5.34624, 7.61609	14.72728,	3	NA	90229
GL172732.1	1837875	1838095	1837933, 1837968, 1837918	11, 14, 6	10.3799, 11.99537, 6.4014	6.79736, 7.17543, 4.8799	7.59574, 9	3	NA	39942
GL172732.1	2390734	2391218	2391106, 2391072, 2390962	10, 11, 14	10.13706, 10.15827, 16.55387	6.7935, 6.66366, 9.85015	7.36489, 7	3	necab1	0
GL172732.1	2787912	2788542	2788363, 2788289, 2788185	12, 9, 8	11.43506, 7.47261, 9.92329	7.25774, 5.32627, 6.7228	8.58315, 5	3	NA	0
GL172733.1	3827	4225	4158, 4164, 4194	19, 20, 10	19.8867, 17.7751, 10.71812	10.65421, 9.08317, 7.12015	16.66031,	3	atp8b1	0
GL172733.1	98602	99358	98930, 99111, 99090	15, 23, 7	14.78606, 22.2424, 7.96278	8.64215, 10.8193, 5.72394	11.76448,	3	fech	38477
GL172733.1	139237	139828	139408, 139408, 139406	20, 27, 5	21.35298, 25.72383, 5.59755	11.23841, 11.51676, 4.38538	18.07874,	3	NA	16481
GL172733.1	170930	171437	171161, 171249, 170960	19, 15, 8	21.71142, 14.84826, 9.05167	11.72888, 8.67957, 6.30653	18.42619,	3	NA	13621
GL172733.1	265684	266328	265712, 265689, 265712	60, 74, 57	75.83787, 93.41071, 79.68977	25.67355, 28.09221, 29.52195	71.68307,	3	st8sia3	53361
GL172733.1	385804	386374	386092, 386006, 385986	6, 18, 7	5.72604, 14.71602, 7.78342	4.51498, 7.81494, 5.63488	3.33547, 1	3	wdr7	0
GL172733.1	392196	392460	392344, 392354, 392344	12, 15, 11	12.05017, 12.58265, 11.4314	7.62377, 7.29694, 7.41405	9.16715, 9	3	wdr7	0
GL172733.1	481798	482392	482355, 482299, 482357	16, 19, 10	18.5447, 19.41322, 10.59656	10.61177, 10.37021, 7.05292	15.36536,	3	wdr7	0
GL172733.1	739065	739572	739167, 739079, 739069	10, 32, 22	8.89113, 35.62566, 27.58478	6.05396, 15.4707, 14.27522	6.20456, 3	3	txnl1	189405
GL172733.1	932033	932663	932357, 932209, 932238	7, 7, 9	6.64707, 5.97725, 9.82249	5.02073, 4.62287, 6.69629	4.15135, 3	3	tcf4	92431
GL172733.1	955780	956347	955900, 955918, 955928	6, 9, 7	5.96715, 8.10603, 7.61369	4.64927, 5.71528, 5.54854	3.52158, 5	3	tcf4	68747
GL172733.1	1095890	1096129	1095919, 1095915, 1095925	16, 22, 15	17.2479, 23.19276, 17.58264	9.86999, 11.71868, 10.25879	14.1196, 2	3	tcf4	39370
GL172733.1	1107652	1107980	1107852, 1107878, 1107891	6, 10, 9	5.96715, 9.39128, 10.42289	4.64927, 6.35644, 7.00726	3.52158, 6	3	ccdc68	41964
GL172733.1	1196889	1197451	1197370, 1197364, 1197341	12, 14, 4	13.8483, 13.26509, 4.55201	8.63436, 7.95332, 3.73489	10.86694,	3	rab27b	0
GL172733.1	1350464	1351562	1351064, 1351189, 1351196	15, 14, 18	16.36908, 13.44539, 24.00763	9.57629, 8.06258, 13.17779	13.27778,	3	NA	37558
GL172733.1	1360347	1365374	1361626, 1361676, 1363381, 1365082, 1361059, 1364254	31, 37, 19, 18, 18, 16	21.92656, 25.12079, 8.33979, 7.26667, 13.14526, 10.24178	8.24536, 8.26961, 4.15963, 3.79046, 6.88427, 5.66116	18.63506,	3	NA	23746
GL172733.1	1379260	1379626	1379614, 1379503, 1379481	5, 6, 5	4.61714, 4.6513, 5.19074	3.86998, 3.86883, 4.18277	2.39372, 2	3	NA	9494
GL172733.1	1418257	1419134	1418485, 1418332, 1418489	13, 8, 7	14.10549, 6.66413, 8.08824	8.64628, 4.9509, 5.7849	11.11201,	3	c18orf54	934
GL172733.1	1672051	1672302	1672276, 1672275, 1672272	23, 45, 24	28.1049, 52.87357, 33.55553	14.29087, 19.89646, 16.99207	24.64267,	3	DCC	57498
GL172733.1	1830697	1831270	1830784, 1830733, 1830735	5, 9, 7	4.61714, 7.71322, 8.28621	3.86998, 5.47534, 5.87881	2.39372, 5	3	DCC	0
GL172733.1	1977846	1978293	1977874, 1977891, 1977847	64, 70, 47	77.18053, 82.95485, 60.7525	24.35931, 24.48445, 23.54931	73.01245,	3	DCC	95568
GL172733.1	2048764	2049444	2049400, 2049425, 2049395	35, 43, 28	43.98968, 51.83128, 37.55363	19.17759, 20.22374, 18.08183	40.22524,	3	DCC	166486
GL172733.1	2179359	2179818	2179789, 2179776, 2179787	31, 54, 31	33.34702, 60.87101, 38.6896	14.51913, 20.21394, 17.67953	29.76641,	3	mex3c	215378
GL172733.1	2200036	2200403	2200226, 2200170, 2200118	9, 12, 8	8.66407, 10.2136, 8.1956	6.04743, 6.51056, 5.85348	5.99101, 7	3	mex3c	194793
GL172733.1	2249865	2250613	2250572, 2250547, 2250564	43, 51, 27	53.64901, 58.4229, 32.60297	21.29318, 20.25278, 15.59649	49.7454, 5	3	mex3c	144583
GL172733.1	2255585	2255932	2255743, 2255692, 2255790	7, 5, 5	6.69449, 3.58952, 4.74091	5.04797, 3.23987, 3.94006	4.1953, 1.6	3	mex3c	139264
GL172733.1	2299559	2299807	2299589, 2299631, 2299625	23, 17, 16	23.8944, 14.31774, 19.16077	11.81108, 7.84337, 10.95167	20.54405,	3	mex3c	95389
GL172733.1	2468540	2468839	2468678, 2468678, 2468645	12, 19, 12	9.08501, 14.1101, 10.84828	5.80775, 7.19287, 6.90153	6.38792, 1	3	baat	0
GL172733.1	2471569	2472365	2472040, 2472171, 2472028	20, 25, 12	17.86307, 20.24731, 11.21415	9.13605, 9.01913, 7.12434	14.71167,	3	NA	0
GL172733.1	2480312	2481543	2481291, 2481140, 2480874	13, 23, 11	7.87586, 16.76084, 9.36553	4.86993, 7.64736, 6.17745	5.27233, 1	3	NA	6317
GL172733.1	2485001	2486508	2485708, 2485695, 2485663	25, 28, 22	15.77008, 16.20641, 17.00819	6.65511, 6.25305, 8.05423	12.70271,	3	NA	11006
GL172733.1	2488350	2491042	2488543, 2488727, 2490391	22, 27, 18	12.14762, 14.49202, 12.77731	5.48851, 5.68252, 6.67069	9.26087, 1	3	NA	14355
GL172733.1	2488350	2491711	2490380, 2490715, 2490391	23, 29, 18	13.27571, 17.8215, 12.77731	5.84714, 6.78004, 6.67069	10.32627,	3	NA	14355
GL172733.1	2498579	2499676	2499117, 2499021, 2499248	5, 10, 7	4.43696, 9.39128, 6.79502	3.76555, 6.35644, 5.10529	2.24017, 6	3	NA	24584
GL172733.1	2506128	2506812	2506393, 2506779, 2506241	7, 9, 7	4.99677, 6.31893, 5.85759	4.00483, 4.5963, 4.54944	2.67584, 3	3	NA	32133
GL172733.1	2508294	2508890	2508516, 2508523, 2508774	18, 18, 16	17.93309, 16.34754, 17.70367	9.76366, 8.80191, 10.13439	14.77832,	3	NA	34299
GL172733.1	2520454	2521703	2521046, 2521333, 2521312	21, 40, 21	16.62691, 31.193, 18.42256	8.11394, 10.23131, 9.17012	13.52596,	3	NA	46459
GL172733.1	2527636	2528359	2528040, 2528071, 2527983	11, 19, 7	7.12436, 13.45566, 4.76637	4.77406, 6.81692, 3.85525	4.59141, 1	3	NA	42683
GL172733.1	2547935	2548465	2548261, 2548270, 2548073	16, 18, 10	15.87778, 17.52265, 11.24131	9.05608, 9.51543, 7.40241	12.8057, 1	3	NA	22577
GL172733.1	2690994	2691582	2691207, 2691215, 2691316	34, 45, 18	31.9991, 43.6181, 19.30306	12.5891, 14.73744, 10.58333	28.44799,	3	me2	12805
GL172733.1	2786032	2786389	2786364, 2786359, 2786357	33, 44, 45	37.97073, 50.36766, 57.71053	16.45382, 18.90658, 22.73234	34.30122,	3	lipg	14311
GL172733.1	2835052	2835299	2835272, 2835258, 2835269	8, 14, 5	8.42257, 14.45897, 5.80393	5.97763, 8.66788, 4.48186	5.76336, 1	3	lipg	63331
GL172733.1	2854618	2855102	2855036, 2854868, 2854900	6, 12, 7	5.20598, 9.16947, 6.43637	4.2113, 5.86046, 4.89813	2.86702, 6	3	lipg	82897
GL172733.1	2861446	2861961	2861642, 2861667, 2861605	9, 10, 5	9.72082, 7.86505, 5.31857	6.64182, 5.4165, 4.24819	6.97155, 5	3	lipg	89725
GL172734.1	678931	680094	679304, 679256, 679057, 680069	8, 10, 5, 6	7.38183, 7.90132, 3.88081, 4.38967	5.38666, 5.43931, 3.42643, 3.70174	4.80856, 5	3	FAM98A	8845
GL172734.1	682738	683374	683113, 683238, 683318	16, 24, 22	15.28516, 22.33212, 22.95626	8.69747, 10.54139, 11.57681	12.24188,	3	FAM98A	5565
GL172734.1	1575946	1576876	1576666, 1576678, 1576636	20, 26, 11	19.99469, 23.24287, 11.4314	10.42292, 10.41222, 7.41405	16.76522,	3	epm2a	11143
GL172734.1	2064552	2065358	2065113, 2065241, 2065231	18, 36, 26	17.93309, 34.99198, 30.24261	9.76366, 13.51384, 14.56166	14.77832,	3	utrn	12993
GL172735.1	496	955	862, 890, 911	20, 13, 14	19.49401, 12.03475, 15.80333	10.12053, 7.4231, 9.43935	16.28237,	3	NA	14344
GL172735.1	2942	3451	3423, 3414, 3421	24, 19, 19	22.42871, 18.34606, 22.23331	10.59867, 9.72578, 12.02841	19.12211,	3	NA	11848
GL172735.1	54808	55319	55079, 55182, 55033	10, 29, 9	6.34128, 21.51243, 7.41152	4.45239, 8.56001, 5.2882	3.86892, 1	3	NA	2074
GL172735.1	58316	58979	58686, 58687, 58740	31, 39, 11	30.83565, 34.29391, 10.10247	13.0432, 12.0582, 6.62982	27.30938,	3	NA	5582
GL172735.1	130167	130983	130228, 130283, 130312	12, 12, 11	10.1642, 8.05278, 10.52088	6.47994, 5.16604, 6.8818	7.39114, 5	3	NA	15823
GL172735.1	134395	135133	134931, 134997, 135044	15, 24, 14	12.07737, 19.71799, 13.23047	6.98694, 9.01872, 7.9323	9.19364, 1	3	NA	20051
GL172735.1	186694	187426	187366, 187239, 186984	5, 6, 5	4.82177, 4.93312, 5.59755	3.98509, 4.04501, 4.38538	2.51418, 2	3	XB-GENE-5830570	246
GL172735.1	224355	224839	224787, 224811, 224786	13, 23, 14	14.88608, 23.74183, 17.94273	9.08033, 11.71949, 10.5654	11.85945,	3	U4	6741
GL172735.1	308392	308941	308906, 308881, 308879	16, 21, 5	17.95866, 19.92695, 5.91431	10.28064, 10.07243, 4.53172	14.80292,	3	NA	16547
GL172735.1	406541	407055	406925, 406832, 407001	8, 8, 7	8.42257, 7.07159, 8.69489	5.97763, 5.20073, 6.06379	5.76336, 4	3	5S_rRNA	26759
GL172735.1	460550	461172	460949, 460929, 460949	5, 7, 8	4.82177, 5.97725, 10.1465	3.98509, 4.62287, 6.82176	2.51418, 3	3	arc	12237
GL172735.1	466590	467208	466933, 466989, 466662	7, 11, 6	7.10297, 9.95469, 7.29403	5.27706, 6.5399, 5.30581	4.57191, 7	3	arc	18277
GL172735.1	477116	477536	477480, 477183, 477483	7, 8, 5	7.1696, 7.07159, 5.9506	5.31345, 5.20073, 4.54784	4.61046, 4	3	arc	28803
GL172735.1	510289	510725	510401, 510670, 510506	5, 8, 4	4.82177, 5.96021, 4.64325	3.98509, 4.50731, 3.77643	2.51418, 3	3	adgrb1	60106
GL172735.1	532645	533121	532673, 532653, 532697	8, 17, 8	7.52511, 17.48427, 9.1831	5.47115, 9.76452, 6.37229	4.94319, 1	3	adgrb1	37710
GL172735.1	542479	543146	542658, 543013, 542756	10, 11, 10	9.54985, 10.48162, 11.6791	6.45088, 6.85833, 7.62925	6.82341, 7	3	adgrb1	27685
GL172735.1	555194	555435	555407, 555407, 555369	12, 27, 14	12.97977, 30.3501, 16.55387	8.15868, 14.26622, 9.85015	10.04411,	3	adgrb1	15396
GL172735.1	639321	639771	639348, 639352, 639351	48, 74, 38	55.32394, 88.0497, 46.83072	19.90263, 25.1976, 19.55775	51.39656,	3	adgrb1	0
GL172735.1	660130	660970	660329, 660228, 660928	6, 15, 5	5.96715, 15.40598, 5.9506	4.64927, 9.01279, 4.54784	3.52158, 1	3	adgrb1	0
GL172735.1	677042	678437	678085, 677503, 677536, 677792	11, 9, 6, 5	10.66406, 7.43399, 6.6567, 5.31857	6.96705, 5.30221, 5.00845, 4.24819	7.86396, 4	3	adgrb1	0
GL172735.1	758294	758826	758736, 758531, 758493	11, 20, 9	10.66406, 18.53364, 8.60738	6.96705, 9.54017, 6.01421	7.86396, 1	3	adgrb1	0
GL172735.1	853621	854396	854232, 853979, 853867	10, 6, 5	9.08301, 3.67227, 5.23257	6.17079, 3.23006, 4.20435	6.3862, 1.7	3	adgrb1	42627
GL172735.1	869355	869768	869466, 869447, 869382	12, 22, 10	12.74986, 21.56447, 10.03522	8.02869, 10.73971, 6.73493	9.82533, 1	3	adgrb1	58361
GL172735.1	877233	877799	877535, 877566, 877534	9, 15, 8	7.59348, 12.72276, 7.34578	5.40134, 7.383, 5.36526	5.0069, 9.9	3	adgrb1	66239
GL172735.1	880666	880882	880862, 880859, 880862	22, 17, 15	20.03498, 12.81996, 14.37829	9.8224, 6.94148, 8.39573	16.80398,	3	adgrb1	69672
GL172735.1	885393	885861	885747, 885748, 885699	15, 18, 8	12.8537, 16.46278, 7.42597	7.46342, 8.87194, 5.41279	9.92465, 1	3	adgrb1	74399
GL172735.1	985306	986229	985761, 986079, 985635	6, 10, 7	5.63898, 9.39128, 8.69489	4.46544, 6.35644, 6.06379	3.25772, 6	3	adgrb1	174312
GL172735.1	994058	995177	994751, 994898, 994675	5, 20, 10	4.82177, 20.84905, 10.84317	3.98509, 10.93703, 7.18868	2.51418, 1	3	adgrb1	183064
GL172735.1	1081793	1082056	1082004, 1081975, 1081916	11, 24, 8	10.21742, 23.92411, 8.4044	6.69945, 11.49076, 5.9678	7.44132, 2	3	XB-GENE-5872721	232568
GL172735.1	1092954	1093253	1093169, 1093151, 1093054	6, 10, 3	5.96715, 9.39128, 3.47371	4.64927, 6.35644, 3.03189	3.52158, 6	3	XB-GENE-5872721	221371
GL172735.1	1105191	1105675	1105221, 1105249, 1105197	8, 9, 7	7.88469, 8.21099, 7.66926	5.67897, 5.77858, 5.57703	5.27935, 5	3	XB-GENE-5872721	208949
GL172735.1	1210891	1211582	1211153, 1211458, 1211430	5, 9, 4	4.61714, 6.93223, 4.67325	3.86998, 4.98685, 3.78987	2.39372, 4	3	XB-GENE-5872721	103042
GL172735.1	1277954	1278624	1278305, 1278267, 1278303	8, 14, 11	7.19971, 11.36754, 11.92945	5.27799, 6.78813, 7.69409	4.63758, 8	3	XB-GENE-5872721	36000
GL172735.1	1297706	1298521	1298151, 1298271, 1297932	8, 12, 7	6.01859, 8.16747, 6.43637	4.54456, 5.23696, 4.89813	3.56839, 5	3	XB-GENE-5872721	16103
GL172735.1	1299647	1300344	1299975, 1299948, 1299989	8, 14, 5	6.20993, 10.95872, 4.70718	4.66614, 6.53614, 3.9211	3.74679, 8	3	XB-GENE-5872721	14280
GL172735.1	1384861	1385441	1384887, 1384886, 1384887	59, 83, 46	69.95697, 99.8535, 62.31344	22.8538, 26.8386, 24.9517	65.86046,	3	tsnare1	7850
GL172735.1	1402666	1403112	1403054, 1403099, 1403105	30, 41, 26	31.64738, 46.18646, 33.39568	13.90267, 17.83768, 16.38347	28.10302,	3	tsnare1	0
GL172735.1	1483059	1484138	1483531, 1483527, 1483640	14, 11, 7	13.89699, 10.60895, 7.66926	8.3343, 6.9343, 5.57703	10.91278,	3	tsnare1	34093
GL172735.1	1493801	1495788	1495048, 1495057, 1495040	36, 73, 32	36.10191, 70.91075, 35.97895	14.13949, 17.35913, 15.68032	32.46703,	3	tsnare1	44835
GL172735.1	1531408	1532379	1531991, 1532020, 1531798	5, 12, 8	4.82177, 11.86108, 10.0913	3.98509, 7.51216, 6.79758	2.51418, 9	3	tsnare1	82442
GL172735.1	1547787	1548446	1548392, 1548430, 1548418	19, 47, 24	21.81444, 54.59318, 30.91999	11.78833, 19.95276, 15.58778	18.52575,	3	tsnare1	98821
GL172735.1	1623098	1623507	1623351, 1623382, 1623376	15, 13, 13	17.28737, 12.93015, 15.0131	10.09595, 7.96262, 9.14923	14.15669,	3	tsnare1	174132
GL172735.1	1659125	1659389	1659312, 1659330, 1659287	7, 17, 4	7.10297, 18.45238, 4.67325	5.27706, 10.33783, 3.78987	4.57191, 1	3	cox11	166443
GL172735.1	1690609	1691156	1690637, 1690761, 1690859	4, 11, 7	3.74195, 8.93411, 7.20145	3.32091, 5.9089, 5.33074	1.6222, 6.3	3	cox11	134676
GL172735.1	1697170	1697973	1697429, 1697876, 1697439	5, 10, 6	4.54329, 7.86505, 6.71074	3.82752, 5.4165, 5.03498	2.33, 5.400	3	cox11	127859
GL172735.1	1737141	1737515	1737338, 1737460, 1737481	8, 10, 6	8.42257, 7.0814, 6.87953	5.97763, 4.92105, 5.11627	5.76336, 4	3	cox11	88317
GL172735.1	1755291	1755710	1755423, 1755474, 1755481	10, 15, 10	10.46385, 13.74993, 11.24131	6.97883, 8.013, 7.40241	7.67431, 1	3	cox11	70122
GL172735.1	1832265	1832696	1832631, 1832660, 1832653	17, 28, 16	16.76548, 29.4016, 18.88025	9.33227, 13.32928, 10.798	13.6543, 2	3	cox11	2266
GL172735.1	1858166	1858551	1858517, 1858534, 1858485	14, 20, 12	11.06211, 15.14356, 10.59056	6.59982, 7.52945, 6.74336	8.22887, 1	3	cox11	28167
GL172735.1	1861328	1861744	1861605, 1861581, 1861574	27, 25, 21	27.08428, 22.12709, 22.75397	12.31967, 10.0929, 11.77155	23.64654,	3	cox11	31329
GL172735.1	1867877	1868442	1868326, 1868065, 1868097	10, 7, 7	9.54985, 5.48955, 7.66926	6.45088, 4.31982, 5.57703	6.82341, 3	3	ptp4a3	32149
GL172735.1	1942399	1942786	1942649, 1942620, 1942572	10, 18, 7	10.81731, 17.19487, 7.90186	7.17456, 9.31668, 5.69394	8.00764, 1	3	NA	0
GL172735.1	1979960	1980684	1980396, 1980607, 1980329	10, 7, 9	10.39648, 5.75319, 10.87975	6.94096, 4.48487, 7.23113	7.61003, 3	3	NA	9146
GL172735.1	1994003	1994424	1994324, 1994374, 1994379	10, 35, 8	9.23294, 39.86979, 9.11678	6.26141, 16.74338, 6.33924	6.52626, 3	3	gpr20	22838
GL172735.1	2004215	2004790	2004352, 2004354, 2004253	16, 24, 11	14.51319, 20.63686, 10.19222	8.22691, 9.54685, 6.68421	11.50268,	3	gpr20	12472
GL172735.1	2006385	2007033	2006582, 2006450, 2006672	15, 13, 12	13.20489, 8.81099, 12.03712	7.67906, 5.43464, 7.61609	10.25934,	3	gpr20	10229
GL172735.1	2110269	2110798	2110442, 2110504, 2110516	7, 14, 8	5.42862, 12.75552, 6.97106	4.2813, 7.64262, 5.13965	3.06982, 1	3	dennd3	17288
GL172735.1	2150823	2151305	2150892, 2150825, 2150887	11, 12, 4	12.43693, 11.72254, 4.67325	7.97018, 7.42986, 3.78987	9.52692, 9	3	dennd3	0
GL172735.1	2209293	2209727	2209501, 2209335, 2209323	6, 12, 6	5.90927, 10.93692, 5.64193	4.61743, 6.95571, 4.46713	3.50164, 8	3	rplp1	2853
GL172735.1	2327159	2327706	2327252, 2327251, 2327252	18, 21, 11	19.69649, 20.89182, 12.06254	10.81516, 10.65421, 7.76744	16.47841,	3	ptk2	28318
GL172735.1	2388963	2389561	2388970, 2388993, 2388999	44, 71, 38	54.66867, 84.39739, 49.19839	21.42052, 24.75657, 20.9503	50.75022,	3	ptk2	0
GL172735.1	2405223	2405840	2405784, 2405804, 2405686	24, 44, 21	25.63312, 45.55924, 24.0973	12.51508, 16.18397, 12.56358	22.23394,	3	ptk2	0
GL172735.1	2447217	2447526	2447319, 2447360, 2447355	8, 7, 13	8.42257, 5.97725, 15.09516	5.97763, 4.62287, 9.19347	5.76336, 3	3	trappc9	0
GL172735.1	2522036	2523148	2522761, 2522761, 2522761	14, 18, 12	15.15267, 16.40493, 13.7434	9.07115, 8.83679, 8.57827	12.11465,	3	trappc9	0
GL172735.1	2575929	2576655	2576094, 2575954, 2575958	8, 14, 13	6.14488, 11.28333, 14.0426	4.62489, 6.73619, 8.61056	3.6868, 8.6	3	trappc9	0
GL172735.1	2625751	2626846	2626804, 2626807, 2626782	17, 14, 7	20.70049, 14.45897, 8.35502	11.60995, 8.66788, 5.9108	17.44812,	3	trappc9	29637
GL172735.1	2668992	2669498	2669002, 2669002, 2669029	33, 60, 28	35.34935, 69.43006, 35.3581	14.90321, 22.07352, 16.84442	31.72944,	3	trappc9	72878
GL172735.1	2679207	2679544	2679332, 2679378, 2679387	7, 8, 8	6.33443, 5.96021, 8.80246	4.83795, 4.50731, 6.179	3.86259, 3	3	trappc9	83093
GL172735.1	2727264	2728038	2727676, 2727570, 2727821	8, 16, 4	6.66791, 12.77959, 4.67325	4.95324, 7.16814, 3.78987	4.17132, 1	3	KCNK9	49489
GL172735.1	2748890	2749377	2749164, 2749310, 2749286	7, 16, 5	7.1696, 16.74813, 5.9506	5.31345, 9.57609, 4.54784	4.61046, 1	3	KCNK9	28150
GL172735.1	2788195	2788591	2788369, 2788255, 2788425	8, 10, 6	6.55639, 7.17119, 5.2665	4.88389, 4.97797, 4.24757	4.06705, 4	3	KCNK9	0
GL172735.1	2837173	2838010	2837691, 2837691, 2837691	13, 12, 11	12.43975, 10.25865, 11.6739	7.66875, 6.53846, 7.55148	9.5289, 7.6	3	KCNK9	0
GL172736.1	36653	37148	36961, 36912, 36713	15, 16, 10	14.52484, 13.21822, 10.3632	8.4845, 7.43523, 6.92219	11.51408,	3	vsnl1	39999
GL172736.1	288428	289093	288912, 288831, 288658	9, 11, 4	8.11416, 9.95469, 4.01199	5.72019, 6.5399, 3.46784	5.49285, 7	3	smc6	46177
GL172736.1	395110	396286	395837, 395662, 395952	13, 18, 16	12.81131, 15.6398, 16.83466	7.8918, 8.37236, 9.62725	9.88323, 1	3	msgn1	4721
GL172736.1	403670	404408	404218, 404102, 403778	14, 15, 9	12.97522, 14.46023, 8.98852	7.77689, 8.44539, 6.23511	10.03988,	3	msgn1	1728
GL172736.1	551042	551458	551358, 551250, 551281	13, 18, 9	14.35383, 17.52265, 11.64359	8.78627, 9.51543, 7.57973	11.34993,	3	kcns3	43629
GL172736.1	683530	683746	683729, 683697, 683716	15, 15, 7	14.8531, 13.69427, 8.69489	8.68248, 7.97895, 6.06379	11.82783,	3	nt5c1a	41175
GL172736.1	703119	703864	703253, 703259, 703487	9, 12, 11	8.35434, 11.86108, 12.9493	5.86444, 7.51216, 8.23866	5.71584, 9	3	nt5c1a	60764
GL172736.1	827087	827484	827125, 827222, 827177	5, 12, 3	4.82177, 10.25865, 3.47371	3.98509, 6.53846, 3.03189	2.51418, 7	3	osr1	176776
GL172736.1	904160	904398	904186, 904211, 904188	31, 40, 33	37.3478, 44.37267, 42.18798	16.89197, 17.226, 18.9386	33.6894, 4	3	osr1	99862
GL172736.1	961282	961802	961734, 961785, 961736	31, 47, 31	34.86773, 54.47084, 38.44539	15.42176, 19.88236, 17.53684	31.257, 50	3	osr1	42458
GL172736.1	1083859	1084343	1083892, 1084068, 1083867	11, 5, 7	12.33515, 3.94561, 7.01416	7.91559, 3.46715, 5.22813	9.43699, 1	3	osr1	68350
GL172736.1	1166256	1166878	1166469, 1166476, 1166607	16, 22, 18	14.85827, 20.6214, 20.32194	8.4376, 10.17315, 11.17944	11.83222,	3	osr1	150747
GL172736.1	1194656	1195138	1194977, 1194965, 1194979	7, 14, 6	7.1696, 12.97647, 6.5517	5.31345, 7.77766, 4.95623	4.61046, 1	3	osr1	179147
GL172736.1	1220600	1221048	1220814, 1220813, 1220815	12, 18, 14	11.55224, 18.67996, 17.01463	7.3281, 10.2126, 10.0942	8.69537, 1	3	ttc32	187962
GL172736.1	1230904	1231548	1231122, 1231122, 1231123	23, 30, 22	24.58488, 29.23872, 25.9713	12.225, 12.48814, 13.35936	21.21468,	3	ttc32	177462
GL172736.1	1253289	1253779	1253507, 1253445, 1253551	8, 9, 6	8.05102, 7.67202, 7.29403	5.77295, 5.44992, 5.30581	5.43473, 5	3	ttc32	155231
GL172736.1	1331672	1332262	1332224, 1332236, 1332261	14, 12, 6	14.45502, 10.12474, 6.82211	8.66555, 6.45547, 5.08889	11.4469, 7	3	ttc32	76748
GL172736.1	1353360	1354654	1354076, 1354385, 1354075	35, 49, 19	35.64456, 52.81398, 20.70442	14.27334, 18.03745, 11.14035	32.01824,	3	ttc32	54356
GL172736.1	1371611	1372433	1372141, 1372132, 1372161	14, 20, 11	15.49091, 21.26374, 14.75772	9.26387, 11.18517, 9.09568	12.43497,	3	ttc32	36577
GL172736.1	1395806	1396329	1395918, 1396145, 1395950	6, 6, 5	5.96715, 4.93312, 5.9506	4.64927, 4.04501, 4.54784	3.52158, 2	3	ttc32	12681
GL172736.1	1480575	1481127	1481060, 1481068, 1481073	24, 16, 11	28.29526, 15.94397, 13.64572	14.09251, 9.09593, 8.58592	24.82836,	3	laptm4a	1926
GL172736.1	1702520	1703304	1703284, 1703262, 1703274	67, 100, 72	82.44147, 122.25879, 96.60118	25.73239, 29.53669, 30.99901	78.22231,	3	slc7a15	10310
GL172736.1	1755949	1756674	1756251, 1756300, 1756310	9, 6, 7	8.20841, 4.11123, 8.57075	5.77703, 3.52071, 6.00887	5.58112, 2	3	rhob	18586
GL172736.1	1774034	1774534	1774179, 1774298, 1774318	19, 17, 7	18.26836, 14.56175, 7.29952	9.67872, 7.99171, 5.38357	15.09918,	3	rhob	36671
GL172736.1	1962940	1963222	1963102, 1963116, 1963094	8, 14, 4	7.57423, 12.09105, 4.67325	5.4999, 7.23439, 3.78987	4.98865, 9	3	ldah	4911
GL172736.1	2066213	2066570	2066531, 2066545, 2066542	22, 35, 18	25.29347, 37.8794, 20.32194	12.96511, 15.57114, 11.17944	21.90464,	3	ldah	0
GL172736.1	2333971	2334442	2333998, 2333986, 2333984	25, 46, 26	28.03181, 52.82087, 33.79316	13.60123, 19.39968, 16.60607	24.57135,	3	tdrd15	26373
GL172736.1	2586596	2587268	2586782, 2587008, 2586779	11, 11, 14	12.43693, 10.60895, 16.91966	7.97018, 6.9343, 10.04443	9.52692, 7	3	tdrd15	220275
GL172736.1	2726460	2726988	2726487, 2726513, 2726523	89, 103, 69	112.86351, 131.73195, 91.42464	30.48594, 32.69477, 29.72508	108.37022	3	tdrd15	360139
GL172737.1	228239	228912	228762, 228591, 228480	9, 21, 15	9.63662, 20.53474, 18.13606	6.59633, 10.43888, 10.55788	6.90556, 1	3	TMEM182	158254

GL172737.1	1231598	1232105	1232063, 1232031, 1232063	24, 47, 43	24.77116, 52.49995, 54.2805	11.99852, 18.75712, 21.66514	21.39609,	3	spryd4	0
GL172737.1	1388001	1389295	1388911, 1388902, 1389011	12, 21, 10	12.38809, 18.32622, 9.63365	7.82099, 9.11275, 6.50047	9.48755, 1	3	stat2	22210
GL172737.1	2012698	2013089	2012730, 2012709, 2012791	21, 21, 9	23.51727, 20.46506, 9.35579	12.22363, 10.39685, 6.44216	20.17856,	3	rps26	18708
GL172737.1	2071812	2072420	2072057, 2072063, 2072124	8, 9, 5	7.93925, 7.43399, 4.77515	5.70996, 5.30221, 3.9592	5.33002, 4	3	erbb3	3965
GL172737.1	2275183	2276610	2276039, 2276014, 2276040	16, 40, 16	15.34845, 41.10298, 18.61066	8.73591, 15.35707, 10.64858	12.2978, 3	3	smarcc2	1189
GL172737.1	2822876	2823176	2822953, 2822959, 2822967	23, 21, 9	25.32867, 22.48702, 11.21577	12.66898, 11.61241, 7.38849	21.93845,	3	NA	14497
GL172738.1	915439	916129	915949, 915994, 915700	17, 18, 6	19.80801, 15.79712, 7.25145	11.11664, 8.46772, 5.287	16.58454,	3	NA	30608
GL172739.1	442677	443086	442839, 442860, 443019	7, 8, 5	7.1696, 5.96021, 5.59755	5.31345, 4.50731, 4.38538	4.61046, 3	3	neo1	12129
GL172739.1	670429	670925	670796, 670805, 670779	17, 21, 13	16.4953, 19.86199, 14.5443	9.16875, 10.03329, 8.89247	13.39926,	3	myo9a	0
GL172739.1	1930409	1931332	1930934, 1930732, 1930753	14, 23, 6	13.14827, 21.96903, 6.35308	7.88233, 10.65561, 4.85498	10.20482,	3	kti12	0
GL172739.1	1954138	1954848	1954668, 1954563, 1954376	7, 11, 7	5.10557, 7.16781, 8.49722	4.075, 4.80116, 5.97582	2.77482, 4	3	kti12	8225
GL172740.1	244516	245690	244552, 244555, 245501, 244846	12, 8, 6, 4	12.25023, 7.07159, 4.93312, 4.67325	7.74089, 5.20073, 4.04501, 3.78987	9.35789, 4	3	znf804b	193231
GL172740.1	421472	421851	421664, 421692, 421619	17, 15, 7	18.64535, 15.80067, 8.69489	10.45058, 9.24574, 6.06379	15.46286,	3	znf804b	17070
GL172740.1	996124	996884	996450, 996443, 996624	8, 20, 7	7.28955, 17.88727, 7.50542	5.33177, 9.1506, 5.49244	4.72176, 1	3	cdk14	16754
GL172740.1	1329989	1330690	1330429, 1330437, 1330579	8, 8, 9	7.67464, 7.07159, 11.21577	5.55832, 5.20073, 7.38849	5.08299, 4	3	fzd1	60066
GL172740.1	1369376	1370412	1370217, 1370217, 1370251	21, 38, 14	20.95297, 37.36932, 16.29325	10.69108, 14.06921, 9.7093	17.69275,	3	NA	98186
GL172740.1	1803619	1804592	1804067, 1804031, 1804211	9, 15, 9	7.71701, 12.40077, 9.76105	5.47767, 7.18527, 6.66343	5.12244, 9	3	pex1	0
GL172740.1	1979360	1980073	1979576, 1979550, 1979591	13, 25, 19	14.70302, 26.51553, 23.22595	8.98015, 12.69611, 12.5858	11.68444,	3	cdk6	0
GL172740.1	2122801	2123364	2123133, 2123148, 2123163	35, 36, 24	41.26296, 41.26263, 29.47401	17.56792, 17.14056, 14.77381	37.54033,	3	samd9l	14013
GL172741.1	333773	335209	334295, 334299, 334536	18, 20, 15	18.76354, 17.66446, 17.15492	10.26255, 9.01671, 10.0222	15.57675,	3	pde11a	0
GL172741.1	355689	356547	355991, 355956, 356396	7, 10, 6	7.1696, 9.37821, 6.5517	5.31345, 6.34863, 4.95623	4.61046, 6	3	pde11a	0
GL172741.1	371491	371827	371696, 371502, 371541	7, 9, 9	5.4599, 6.73685, 10.01275	4.30109, 4.86289, 6.79683	3.09813, 4	3	pde11a	0
GL172741.1	465568	466139	465695, 465933, 465718	8, 12, 6	7.47668, 10.49031, 6.25896	5.44269, 6.6816, 4.80589	4.89713, 7	3	pde11a	0
GL172741.1	513622	514063	513826, 513975, 513971	8, 14, 4	8.42257, 14.45897, 4.67325	5.97763, 8.66788, 3.78987	5.76336, 1	3	cyct	13678
GL172741.1	578877	579462	578996, 579028, 579095	21, 37, 17	26.4195, 43.62568, 23.46806	13.88193, 18.10397, 13.01603	22.9988, 4	3	chrna1.2	7483
GL172741.1	904378	904626	904412, 904431, 904437	4, 7, 6	3.45429, 5.39662, 7.29403	3.15498, 4.26102, 5.30581	1.5055, 3.1	3	sp3	0
GL172741.1	920514	921636	921394, 920886, 920616	5, 8, 5	4.14595, 5.64525, 5.54867	3.59111, 4.30526, 4.3619	1.98639, 3	3	sp3	0
GL172741.1	1017095	1017407	1017381, 1017379, 1017361	19, 26, 21	19.56679, 26.87838, 27.07785	10.46249, 12.55754, 14.2403	16.35231,	3	NA	0
GL172741.1	1105348	1105860	1105563, 1105738, 1105814	15, 26, 7	14.27412, 26.23182, 8.02489	8.3325, 12.17172, 5.75426	11.27364,	3	NA	0
GL172741.1	1200422	1200837	1200743, 1200804, 1200609	23, 22, 11	24.32011, 19.82642, 12.13051	12.06642, 9.69808, 7.80464	20.95794,	3	map3k20	0
GL172741.1	1266491	1267060	1266836, 1266834, 1266814	9, 15, 3	9.24298, 13.53022, 3.47371	6.3792, 7.87854, 3.03189	6.53474, 1	3	rapgef4	0
GL172741.1	1686454	1686749	1686690, 1686680, 1686681	12, 11, 6	11.67221, 9.80774, 6.87953	7.39985, 6.45006, 5.11627	8.80793, 7	3	dlx2	7107
GL172741.1	2611674	2612685	2612318, 2612303, 2611848	18, 28, 9	16.10489, 24.71449, 8.93877	8.65451, 10.59193, 6.20662	13.02564,	3	NA	449
GL172742.1	94	925	605, 545, 318	15, 19, 10	17.90819, 21.4153, 11.10454	10.43573, 11.55717, 7.32977	14.75478,	3	NA	32029
GL172742.1	214762	215407	215112, 214976, 214965	16, 21, 9	15.09881, 20.32736, 10.28145	8.58417, 10.3138, 6.93568	12.06341,	3	ap2a1	5555
GL172742.1	275175	275405	275208, 275278, 275198	12, 19, 12	10.83541, 17.23199, 12.44386	6.89366, 9.05085, 7.85325	8.02495, 1	3	spib	0
GL172742.1	474029	474523	474280, 474297, 474279	8, 9, 6	6.14488, 5.56445, 5.29847	4.62489, 4.11313, 4.26663	3.6868, 3.3	3	XB-GENE-5872932	9528
GL172742.1	475568	476052	475664, 475750, 475750	8, 10, 8	6.14488, 6.11993, 7.50809	4.62489, 4.31286, 5.46116	3.6868, 3.7	3	XB-GENE-5872932	7999
GL172742.1	477958	478470	478002, 478076, 478033	11, 17, 11	8.81032, 12.26912, 11.73651	5.8315, 6.61547, 7.58663	6.12919, 9	3	XB-GENE-5872932	5581
GL172742.1	597590	598049	597829, 597764, 597829	7, 13, 4	6.25077, 11.26714, 4.67325	4.78814, 6.95205, 3.78987	3.78441, 8	3	pnkp	5911
GL172742.1	1059036	1060315	1059998, 1059648, 1060056	47, 58, 30	42.69857, 49.82159, 29.45148	13.538, 13.23217, 12.61185	38.95348,	3	LIN7B	1214
GL172742.1	1090168	1091636	1090965, 1091047, 1090465, 1090996	39, 51, 10, 21	37.3377, 47.09966, 7.33926, 20.20265	13.66876, 14.28188, 5.08445, 10.2386	33.67971,	3	LIN7B	15806
GL172742.1	1116151	1116830	1116681, 1116682, 1116457	119, 140, 126	139.05609, 159.79947, 155.5712	28.34488, 28.08149, 32.3701	134.39226	3	NA	4613
GL172742.1	1147791	1149490	1148156, 1148357, 1148179	9, 12, 9	7.75911, 9.13354, 9.8849	5.5036, 5.83804, 6.72947	5.16246, 6	3	NA	2604
GL172742.1	1203509	1204520	1203920, 1204092, 1203917	35, 45, 26	37.35097, 48.70588, 30.44038	15.26208, 17.50925, 14.67824	33.69253,	3	gbp6	8698
GL172742.1	1273940	1274577	1274487, 1274136, 1274162	5, 8, 10	3.71026, 4.92092, 9.0947	3.31786, 3.83624, 6.17787	1.6222, 2.7	3	NA	4747
GL172742.1	1287331	1287878	1287616, 1287669, 1287518	9, 14, 4	8.71822, 12.09105, 4.34116	6.07905, 7.23439, 3.63492	6.04275, 9	3	NA	2944
GL172742.1	1913896	1914575	1914331, 1914334, 1914354	20, 22, 20	20.14348, 20.947, 21.65208	10.51267, 10.3685, 11.41641	16.90841,	3	rps6ka1	0
GL172743.1	83701	84922	84499, 84635, 84431	8, 9, 9	6.59314, 7.92635, 9.58224	4.90679, 5.60609, 6.56676	4.10249, 5	3	sgcb	0
GL172743.1	112519	113058	112632, 112712, 112622	7, 13, 8	6.74267, 11.36332, 10.1465	5.07552, 7.01137, 6.82176	4.23998, 8	3	sgcb	21088
GL172743.1	746550	746908	746735, 746719, 746744	9, 25, 13	7.35817, 22.78017, 14.61978	5.25488, 10.47392, 8.93426	4.78711, 1	3	chic2	26191
GL172743.1	748300	748957	748440, 748827, 748684	8, 14, 4	6.59314, 10.18813, 3.27614	4.90679, 6.06346, 3.04759	4.10249, 7	3	chic2	24142
GL172743.1	1324862	1327139	1326286, 1325501, 1325454, 1326264	23, 23, 8, 16	16.90181, 12.94225, 5.01122, 13.61068	7.72454, 5.68681, 3.89487, 7.67493	13.78674,	3	nmu	46755
GL172743.1	1328597	1329780	1329343, 1329459, 1329469	14, 17, 14	9.22372, 9.31887, 11.52691	5.48009, 4.95532, 6.88646	6.5183, 6.7	3	nmu	50490
GL172743.1	1333486	1335654	1335042, 1334790, 1333394, 1335240	10, 22, 8, 8	7.25363, 16.91629, 5.84174, 6.60217	5.03021, 8.00211, 4.43151, 4.91241	4.68848, 1	3	nmu	55379
GL172743.1	1468734	1469271	1468870, 1468960, 1468831	7, 9, 6	7.1696, 8.21099, 7.29403	5.31345, 5.77858, 5.30581	4.61046, 5	3	cep135	13907
GL172743.1	1961947	1962545	1962429, 1962315, 1962344	24, 17, 23	27.61606, 15.75222, 29.81154	13.69419, 8.71708, 15.24424	24.16528,	3	sgcz	128280
GL172743.1	2142665	2143285	2142883, 2142852, 2142977	8, 7, 8	8.42257, 5.97725, 10.1465	5.97763, 4.62287, 6.82176	5.76336, 3	3	sgcz	22315
GL172743.1	2278639	2279071	2278952, 2278912, 2278901	8, 19, 16	7.67464, 20.47061, 22.37427	5.55832, 11.002, 12.56044	5.08299, 1	3	xtr-mir-383	22585
GL172743.1	2376415	2376871	2376735, 2376719, 2376747	8, 11, 8	8.42257, 10.60895, 9.60885	5.97763, 6.9343, 6.57807	5.76336, 7	3	xtr-mir-383	74685
GL172743.1	2675133	2676607	2676147, 2675708, 2675615	9, 12, 9	8.0679, 8.99251, 9.08997	5.69219, 5.75004, 6.2929	5.45, 6.45(	3	tusc3	0
GL172744.1	626807	627140	626809, 626840, 626835	18, 19, 21	17.79149, 16.46445, 24.28039	9.67812, 8.58735, 12.67014	14.64268,	3	diexf	12321
GL172744.1	656032	656818	656548, 656289, 656371	5, 5, 5	3.41365, 3.85883, 4.91762	3.12469, 3.41255, 4.03768	1.47674, 1	3	prph2	879
GL172744.1	881915	882237	881978, 882023, 881922	5, 6, 3	4.54329, 4.93312, 3.47371	3.82752, 4.04501, 3.03189	2.33, 2.71(	3	BICRAL	3604
GL172744.1	994871	995278	995212, 995094, 995213	12, 9, 9	11.37748, 5.99677, 10.35155	7.22306, 4.39002, 6.97128	8.52862, 3	3	slc30a1	17868
GL172744.1	1370055	1370734	1370420, 1370347, 1370376	12, 12, 7	12.90199, 11.48527, 8.49722	8.11489, 7.28792, 5.97582	9.97033, 8	3	nenf	44521
GL172744.1	1434910	1435417	1435243, 1435001, 1435282	8, 6, 4	8.22568, 4.93312, 4.64325	5.8701, 4.04501, 3.77643	5.59681, 2	3	atf3	22354
GL172744.1	1478043	1478433	1478176, 1478089, 1478112	8, 16, 9	7.1122, 14.57865, 9.52439	5.22529, 8.26691, 6.53516	4.57946, 1	3	atf3	17225
GL172744.1	1505949	1506225	1506005, 1506008, 1505997	8, 14, 10	6.27633, 10.12015, 9.53891	4.70812, 6.02198, 6.44439	3.80887, 7	3	NSL1	10724
GL172744.1	1510259	1510635	1510333, 1510262, 1510262	5, 12, 10	3.9718, 9.09789, 11.24131	3.48353, 5.81578, 7.40241	1.83149, 6	3	NSL1	6314
GL172744.1	1542766	1543063	1542792, 1542794, 1542793	51, 69, 43	57.74545, 82.87985, 55.95539	19.87172, 24.94576, 22.65065	53.7857, 7	3	NA	0
GL172744.1	1625439	1625762	1625485, 1625516, 1625589	4, 9, 5	3.63646, 8.0603, 5.19074	3.26116, 5.68758, 4.18277	1.6222, 5.5	3	vash2	0
GL172744.1	1936248	1936510	1936471, 1936443, 1936454	9, 18, 6	9.72082, 19.97713, 7.29403	6.64182, 10.97931, 5.30581	6.97155, 1	3	prox1	158930
GL172744.1	2035116	2035520	2035300, 2035268, 2035320	19, 17, 12	20.39198, 14.97125, 12.44386	10.95535, 8.24108, 7.85325	17.14961,	3	prox1	59920
GL172744.1	2081063	2081429	2081204, 2081398, 2081325	9, 24, 6	7.97705, 23.06143, 6.25896	5.637, 10.97506, 4.80589	5.36577, 1	3	prox1	14011
GL172744.1	2127792	2128051	2127965, 2127968, 2127968	12, 23, 12	11.61187, 22.59564, 13.36868	7.3638, 11.03113, 8.37481	8.75096, 1	3	prox1	0
GL172744.1	2163788	2164353	2164307, 2164318, 2164313	11, 21, 4	10.96672, 21.57397, 4.55201	7.14544, 11.06508, 3.73489	8.14942, 1	3	prox1	25757
GL172744.1	2167014	2167363	2167324, 2167350, 2167319	10, 16, 9	9.38842, 14.57865, 10.56949	6.35473, 8.26691, 7.08032	6.67167, 1	3	prox1	28983
GL172744.1	2292386	2292973	2292919, 2292934, 2292906	12, 28, 6	11.2086, 31.30207, 6.4014	7.12098, 14.46185, 4.8799	8.36821, 2	3	ptpn14	0
GL172744.1	2314131	2314588	2314429, 2314379, 2314369	8, 8, 4	8.42257, 6.55376, 4.67325	5.97763, 4.88226, 3.78987	5.76336, 4	3	ptpn14	17647
GL172744.1	2402510	2403074	2402728, 2402764, 2402850	8, 7, 7	8.42257, 5.97725, 8.69489	5.97763, 4.62287, 6.06379	5.76336, 3	3	cenpf	31683
GL172744.1	2615485	2615973	2615846, 2615839, 2615729	8, 12, 7	7.02691, 10.35, 7.29952	5.17362, 6.59497, 5.38357	4.50222, 7	3	uqcrh	6378
GL172744.1	2664263	2664972	2664359, 2664831, 2664343	13, 8, 9	14.35383, 7.07159, 9.94831	8.78627, 5.20073, 6.76298	11.34993,	3	SPEN	0
GL172745.1	32537	33675	33288, 33140, 33241	9, 14, 4	9.12009, 12.75552, 4.30163	6.30997, 7.64262, 3.61556	6.42028, 1	3	NA	11029
GL172745.1	1218428	1219654	1218859, 1218902, 1218957	15, 25, 19	13.74446, 23.02855, 21.26518	8.00965, 10.61971, 11.46967	10.77314,	3	3-Sep	13733
GL172745.1	1635227	1635738	1635688, 1635696, 1635694	24, 25, 17	26.57606, 23.67755, 21.41937	13.07811, 11.00257, 11.99417	23.15164,	3	apold1	2933
GL172746.1	302112	302733	302258, 302223, 302364	5, 7, 4	4.33555, 5.58561, 4.67325	3.70555, 4.38027, 3.78987	2.15195, 3	3	gria2	18960
GL172746.1	426976	427620	427139, 427330, 427267	10, 16, 5	10.07458, 13.21822, 4.57714	6.75761, 7.43523, 3.84705	7.30652, 1	3	gria2	31311
GL172746.1	1832954	1833432	1833153, 1833152, 1833153	14, 19, 13	14.91094, 19.04123, 15.34872	8.9318, 10.14611, 9.3288	11.88377,	3	NPY1R	57082
GL172746.1	1983865	1984859	1984513, 1984357, 1984382	22, 28, 12	24.08614, 32.38163, 13.44151	12.25235, 15.10352, 8.41472	20.7301, 2	3	npy5r	4702
GL172746.1	2004035	2004993	2004530, 2004570, 2004451	8, 13, 7	5.78012, 9.43802, 6.11595	4.39198, 5.81956, 4.70713	3.38637, 6	3	NA	2131
GL172746.1	2007137	2007547	2007495, 2007449, 2007454	19, 12, 14	19.96873, 9.16947, 14.66346	10.70325, 5.86046, 8.7878	16.74065,	3	NA	5233
GL172746.1	2534537	2535189	2534971, 2534971, 2534971	40, 53, 20	48.2811, 59.03131, 25.00335	19.51647, 19.66173, 13.33871	44.45338,	3	cpe	22043
GL172747.1	921568	922102	922034, 921805, 921882	6, 7, 4	5.55471, 4.82021, 4.22496	4.41698, 3.89031, 3.57747	3.18192, 2	3	tacr3	0
GL172747.1	1292507	1293114	1292788, 1292785, 1292571	23, 30, 8	24.76553, 30.23761, 7.76694	12.33305, 13.07132, 5.6116	21.39099,	3	manba	3090
GL172747.1	2054406	2055343	2054910, 2054802, 2055134	16, 24, 12	13.72284, 21.89488, 13.44151	7.74351, 10.28282, 8.41472	10.75297,	3	adh1c	10767
GL172747.1	2138354	2138941	2138553, 2138613, 2138706	11, 15, 8	10.78278, 13.53022, 7.81204	7.03733, 7.87854, 5.63749	7.97565, 1	3	dnajb14	13689
GL172747.1	2273387	2274039	2273637, 2273855, 2273859	6, 7, 7	5.3939, 5.10722, 7.78342	4.32314, 4.07606, 5.63488	3.0374, 2.8	3	dapp1	7117
GL172748.1	22844	27165	23724, 25583, 26626	30, 27, 21	19.76682, 17.20062, 15.57205	7.45879, 6.91375, 7.51038	16.54671,	3	NA	6233
GL172748.1	1358622	1361689	1358657, 1360213, 1359452, 1360871	8, 20, 10, 10	5.45331, 15.18357, 8.50615, 8.46974	4.1814, 7.55252, 5.81702, 5.79445	3.09249, 1	3	pde4c	310681
GL172748.1	1358623	1361689	1359684, 1360213, 1359452, 1360871	7, 20, 10, 10	4.64678, 15.18357, 8.50615, 8.46974	3.77717, 7.55252, 5.81702, 5.79445	2.42196, 1	3	pde4c	310682
GL172748.1	1358623	1361689	1361073, 1360213, 1359452, 1360871	12, 20, 10, 10	9.77685, 15.18357, 8.50615, 8.46974	6.23923, 7.55252, 5.81702, 5.79445	7.0243, 12	3	pde4c	310682
GL172748.1	1397383	1399996	1397956, 1397962, 1399533	19, 30, 15	13.27796, 22.64852, 14.11641	6.71596, 8.8669, 8.2366	10.32845,	3	pde4c	349442
GL172749.1	585936	586414	586270, 586062, 586268	21, 13, 10	20.37943, 11.26714, 10.65691	10.34521, 6.95205, 7.08637	17.13786,	3	CARTPT	32067
GL172749.1	1274157	1274863	1274274, 1274489, 1274476	18, 23, 18	17.93309, 22.81409, 21.79524	9.76366, 11.16226, 12.01217	14.77832,	3	arhgef28	0
GL172749.1	1678637	1679200	1678920, 1678921, 1679096	19, 17, 7	22.57464, 17.71433, 8.69489	12.222, 9.9018, 6.06379	19.26377,	3	enc1	78231
GL172749.1	1684299	1685203	1684950, 1684634, 1685075	10, 16, 8	10.6018, 16.74813, 9.76268	7.05583, 9.57609, 6.64964	7.80484, 1	3	enc1	72228
GL172749.1	1930043	1931654	1930549, 1930687, 1930693	17, 18, 8	16.42934, 15.69186, 9.31951	9.12876, 8.4039, 6.43944	13.33656,	3	NA	7078
GL172749.1	2243512	2243830	2243742, 2243790, 2243800	6, 7, 5	5.96715, 5.97725, 5.91431	4.64927, 4.62287, 4.53172	3.52158, 3	3	poc5	27119
GL172749.1	2483696	2484302	2484165, 2484123, 2483769	5, 5, 5	4.69353, 3.85883, 5.9506	3.9134, 3.41255, 4.54784	2.45909, 1	3	iqgap2	0
GL172749.1	2592302	2592541	2592474, 2592501, 2592477	25, 29, 16	31.11602, 30.18937, 20.07743	15.4029, 13.42104, 11.44009	27.58298,	3	iqgap2	3503
GL172750.1	557630	558101	557772, 557667, 557663	9, 16, 6	7.97705, 15.47832, 5.83216	5.637, 8.81468, 4.57461	5.36577, 1	3	col21a1	26822
GL172750.1	916348	917013	916851, 916741, 916843	82, 104, 45	81.7112, 96.41704, 44.99851	18.76653, 17.37428, 15.47019	77.49879,	3	fam83b	44123
GL172750.1	1353925	1354321	1354112, 1354118, 1354124	19, 25, 13	11.42422, 13.58731, 7.41044	5.6961, 5.62686, 4.59454	8.57418, 1	3	klhl31	28393
GL172750.1	1354768	1355642	1355398, 1355301, 1355365	11, 23, 17	5.34396, 13.03901, 12.47634	3.6887, 5.73311, 6.73766	2.99301, 1	3	gclc	29080
GL172750.1	1965514	1966063	1965775, 1965668, 1965698	6, 7, 5	5.96715, 5.97725, 5.9506	4.64927, 4.62287, 4.54784	3.52158, 3	3	mcph1	126006
GL172750.1	1993105	1994104	1993815, 1993676, 1993498	19, 26, 12	18.9632, 26.31054, 13.82167	10.09902, 12.21864, 8.62016	15.76968,	3	mcph1	153597
GL172750.1	2024412	2025620	2024687, 2024891, 2025214	11, 20, 4	12.09119, 22.4484, 4.67325	7.78314, 11.88633, 3.78987	9.20684, 1	3	mcph1	184904
GL172750.1	2406877	2408446	2407814, 2407317, 2407372, 2407744	7, 12, 4, 5	7.1696, 9.46718, 4.30163, 5.75061	5.31345, 6.04622, 3.61556, 4.45735	4.61046, 6	3	mcph1	567369
GL172751.1	570267	571123	570328, 570464, 570387	7, 11, 6	7.1696, 9.71235, 7.29403	5.31345, 6.39153, 5.30581	4.61046, 7	3	mms22l	157533
GL172751.1	2356334	2356987	2356563, 2356822, 2356486	8, 13, 5	6.34412, 10.94367, 5.06966	4.75087, 6.7521, 4.11933	3.871, 8.28	3	map3k7	163495
GL172751.1	2438396	2439885	2439364, 2439451, 2439389	13, 29, 9	14.61367, 28.65304, 9.64095	8.93088, 12.5148, 6.59867	11.59864,	3	map3k7	80597
GL172751.1	2485514	2486439	2485963, 2485951, 2485848	8, 20, 8	7.02691, 18.17471, 8.6841	5.17362, 9.32366, 6.11715	4.50222, 1	3	map3k7	34043
GL172752.1	772794	773477	773283, 773262, 773214	53, 77, 32	51.30519, 78.58716, 30.9576	15.60885, 19.25972, 12.7386	47.43558,	3	NA	139056
GL172752.1	2553861	2554388	2553999, 2554060, 2554131	7, 11, 4	7.1696, 10.60895, 4.67325	5.31345, 6.9343, 3.78987	4.61046, 7	3	prdm10	15920
GL172753.1	5075	5858	5493, 5351, 5346	10, 11, 6	10.89158, 10.26356, 6.87953	7.21503, 6.72731, 5.11627	8.0782, 7.6	3	kif4a	121289
GL172753.1	36063	36957	36388, 36482, 36690	15, 21, 11	15.49092, 19.42055, 12.9493	9.06314, 9.76764, 8.23866	12.43497,	3	kif4a	90190
GL172753.1	217892	218529	218284, 218272, 218281	7, 6, 5	7.1696, 4.68027, 5.85847	5.31345, 3.88713, 4.50665	4.61046, 2	3	GDPD2	0
GL172753.1	927937	928242	928194, 928178, 928190	11, 18, 10	11.02968, 17.65791, 11.24131	7.18222, 9.59733, 7.40241	8.20839, 1	3	NA	31473
GL172753.1	1142747	1143875	1142886, 1143304, 1143494	7, 17, 8	6.50909, 17.11659, 9.1831	4.94073, 9.54398, 6.37229	4.02371, 1	3	igbp1	35818
GL172753.1	1142874	1143875	1143395, 1143304, 1143494	7, 17, 8	6.33443, 17.11659, 9.1831	4.83795, 9.54398, 6.37229	3.86259, 1	3	igbp1	35818
GL172753.1	1242598	1243995	1243224, 1243165, 1242638, 1243930	15, 15, 13, 9	12.38496, 9.63527, 11.09194, 6.72209	7.17557, 5.51762, 6.84382, 4.8535	9.48485, 7	3	arr3	65
GL172753.1	1251064	1251843	1251534, 1251523, 1251468	13, 11, 12	11.0266, 9.52738, 11.76526	6.80342, 6.27759, 7.45528	8.20634, 6	3	arr3	0
GL172753.1	1910377	1911208	1910949, 1910829, 1910485, 1911145	7, 10, 4, 4	6.12952, 8.9331, 4.67325, 4.67325	4.71533, 6.07959, 3.78987, 3.78987	3.67231, 6	3	efnb1	117869
GL172753.1	2345147	2345815	2345473, 2345563, 2345513	6, 14, 6	4.48574, 11.58379, 6.4014	3.76346, 6.92156, 4.8799	2.28228, 8	3	efnb1	552639
GL172754.1	292280	293102	292536, 292791, 292666	14, 12, 7	10.23592, 8.02456, 5.00764	6.09264, 5.14861, 4.01185	7.45918, 5	3	tarsl2	2641
GL172754.1	295673	296682	296342, 296374, 296260	21, 27, 14	13.44136, 15.81502, 8.75118	6.34339, 6.2677, 5.19913	10.48422,	3	tarsl2	6034
GL172754.1	298363	300811	299166, 300183, 298753, 300218	13, 33, 16, 19	6.92974, 19.86802, 11.18186, 13.03635	4.31489, 6.87643, 6.20931, 6.57952	4.41351, 1	3	tarsl2	8724
GL172754.1	298363	301280	300570, 300183, 298753, 300218	20, 33, 16, 19	10.72453, 19.86802, 11.18186, 13.03635	5.14861, 6.87643, 6.20931, 6.57952	7.92048, 1	3	tarsl2	8724
GL172754.1	304185	306624	304696, 305236, 305405	20, 41, 11	13.16924, 31.72607, 8.73264	6.42128, 10.19898, 5.78287	10.22507,	3	tarsl2	14546
GL172754.1	310074	311371	310355, 310231, 310445	18, 16, 8	16.33168, 10.24468, 7.42597	8.79227, 5.66283, 5.41279	13.24212,	3	tarsl2	20435
GL172754.1	318829	321956	320010, 319930, 319723	34, 35, 26	29.75142, 27.59588, 22.47193	11.35929, 9.93394, 9.9691	26.24934,	3	tarsl2	29190
GL172754.1	328240	330012	329001, 329323, 329138	16, 25, 9	18.5447, 27.93067, 10.72164	10.61177, 13.54108, 7.15493	15.36536,	3	tarsl2	38601
GL172754.1	335971	338180	337064, 336141, 337149	26, 27, 13	25.29674, 24.92765, 13.0991	11.61597, 11.05203, 8.06282	21.90767,	3	tarsl2	46332
GL172754.1	435340	436313	435860, 435850, 435711	10, 14, 6	9.54985, 13.38467, 6.5517	6.45088, 8.02582, 4.95623	6.82341, 1	3	tarsl2	145701
GL172754.1	901404	901985	901583, 901586, 901542	8, 15, 11	7.42892, 13.26621, 13.0311	5.41453, 7.71668, 8.28053	4.85345, 1	3	fam189a1	0
GL172755.1	269223	269502	269251, 269249, 269225	7, 15, 13	7.04884, 14.27489, 14.0426	5.2473, 8.33298, 8.61056	4.52221, 1	3	mmp3	774
GL172755.1	317930	318841	318323, 318385, 318192	9, 10, 6	6.99678, 7.32562, 5.53406	5.02768, 5.07581, 4.40503	4.47395, 4	3	NA	0
GL172755.1	383360	384880	384406, 384157, 383924	9, 10, 5	9.72082, 9.39128, 5.9506	6.64182, 6.35644, 4.54784	6.97155, 6	3	mmp13	20955
GL172755.1	515062	515959	515287, 515670, 514988, 515525	5, 17, 4, 4	4.82177, 18.53926, 4.64325, 4.64325	3.98509, 10.38866, 3.77643, 3.77643	2.51418, 1	3	NA	959
GL172755.1	515062	515959	515681, 515670, 514988, 515525	5, 17, 4, 4	4.82177, 18.53926, 4.64325, 4.64325	3.98509, 10.38866, 3.77643, 3.77643	2.51418, 1	3	NA	959
GL172755.1	714302	714866	714510, 714462, 714681	5, 7, 4	4.82177, 5.97725, 4.67325	3.98509, 4.62287, 3.78987	2.51418, 3	3	dcun1d5	0
GL172755.1	728460	729065	728943, 728688, 728837	4, 8, 9	3.74195, 6.93693, 10.28145	3.32091, 5.11882, 6.93568	1.6222, 4.5	3	DYNC2H1	0
GL172755.1	955024	955569	955237, 955493, 955051	15, 13, 7	15.19946, 12.09006, 7.66926	8.88991, 7.45678, 5.57703	12.15964,	3	pdgfd	59157
GL172755.1	1277481	1278040	1277854, 1277646, 1277961	13, 11, 11	12.09198, 9.13706, 11.25734	7.45795, 6.0355, 7.31423	9.20698, 6	3	gria4	85788
GL172755.1	1829048	1829400	1829274, 1829285, 1829290	28, 38, 20	31.18742, 43.59668, 26.76487	14.39356, 17.64915, 14.27334	27.6528, 4	3	gucy1a2	0
GL172755.1	1969431	1970011	1969461, 1969457, 1969465	17, 32, 15	18.64535, 34.43067, 19.89124	10.45058, 14.76315, 11.44789	15.46286,	3	gucy1a2	34583
GL172755.1	1998606	1998937	1998846, 1998850, 1998896	17, 23, 13	18.1272, 23.34424, 15.52427	10.1466, 11.48068, 9.42125	14.96605,	3	cwf19l2	27487
GL172755.1	2097091	2097665	2097436, 2097346, 2097441	13, 21, 12	14.10549, 20.39593, 14.14699	8.64628, 10.35516, 8.79187	11.11201,	3	cwf19l2	955
GL172755.1	2436900	2437243	2437178, 2437112, 2437143	9, 8, 4	8.71822, 5.38416, 4.34116	6.07905, 4.13667, 3.63492	6.04275, 3	3	atm	0
GL172755.1	2549651	2550246	2549761, 2550019, 2549940	10, 17, 6	10.53228, 15.99248, 7.29403	7.01712, 8.86336, 5.30581	7.73927, 1	3	ddx10	0
GL172756.1	884763	885808	885117, 885354, 885318	23, 37, 8	24.06243, 38.94082, 8.09568	11.91191, 15.36076, 5.79794	20.70741,	3	mphosph6	79478
GL172757.1	906718	907587	906925, 906923, 906841	20, 16, 12	21.79824, 13.98946, 12.50469	11.50312, 7.9066, 7.88834	18.51069,	3	asz1	0
GL172757.1	961147	961915	961416, 961507, 961522	12, 12, 5	13.22036, 11.86108, 5.9506	8.29296, 7.51216, 4.54784	10.27237,	3	cftr	0
GL172757.1	1043796	1045145	1044634, 1044558, 1044579	12, 43, 17	11.79509, 41.47402, 19.63801	7.47301, 14.37126, 11.0208	8.92495, 3	3	cttnbp2	0
GL172757.1	2022632	2023851	2023331, 2023154, 2023080	10, 15, 5	8.84451, 11.88618, 5.50079	6.02544, 6.86988, 4.33868	6.16137, 9	3	fam3c	0
GL172758.1	197799	198702	197952, 198146, 198054	7, 13, 12	6.55439, 11.2197, 13.22602	4.96711, 6.92276, 8.2961	4.06616, 8	3	XB-GENE-1002559	35598
GL172758.1	202540	204534	202612, 202877, 204377	9, 10, 20	7.10147, 6.66155, 20.70681	5.09375, 4.65493, 10.85168	4.57191, 4	3	XB-GENE-1002559	29766
GL172758.1	202636	204534	204360, 204443, 204377	19, 16, 20	19.41153, 13.03911, 20.70681	10.36919, 7.32604, 10.85168	16.20286,	3	XB-GENE-1002559	29766
GL172758.1	208525	210159	209458, 209893, 209918	17, 11, 13	17.88146, 9.57292, 14.5443	10.00115, 6.30569, 8.89247	14.72916,	3	XB-GENE-1002559	24141
GL172758.1	311007	312122	311356, 311456, 311928	19, 20, 8	14.03522, 12.45317, 5.46433	7.14954, 6.03602, 4.18852	11.04531,	3	XB-GENE-1002559	29276
GL172758.1	315349	316941	315499, 315445, 315481	23, 25, 14	17.93431, 17.93324, 12.16551	8.29881, 7.75813, 7.28026	14.77945,	3	XB-GENE-1002559	33618
GL172758.1	322713	324355	323461, 323550, 323828	18, 26, 13	15.61903, 20.1495, 11.17224	8.35978, 8.67391, 6.89345	12.55806,	3	XB-GENE-1002559	40982
GL172758.1	326117	327125	326422, 326400, 326442	14, 35, 19	11.06211, 30.76785, 18.5476	6.59982, 11.56522, 9.84779	8.22887, 2	3	XB-GENE-1002559	44386
GL172758.1	413246	415733	414409, 415229, 413907, 415260	18, 26, 10, 12	17.65252, 21.35565, 10.71812, 11.26182	9.59407, 9.33842, 7.12015, 7.15321	14.50784,	3	mrps18c	38579
GL172758.1	419626	420465	419784, 420223, 420055	15, 24, 13	11.70397, 20.79901, 12.19507	6.75851, 9.64095, 7.52062	8.83923, 1	3	mrps18c	33847
GL172758.1	422918	423621	423310, 423235, 423267	20, 16, 9	19.99469, 13.44943, 8.34444	10.42292, 7.57638, 5.85853	16.76522,	3	mrps18c	30691
GL172758.1	428971	429736	429410, 429391, 429584	17, 20, 13	19.60089, 20.22553, 15.34872	10.9998, 10.56212, 9.3288	16.38573,	3	mrps18c	24576
GL172758.1	2159967	2161143	2160317, 2160387, 2160719	13, 18, 11	13.27928, 16.40493, 11.49087	8.16901, 8.83679, 7.44794	10.32936,	3	ptch1	15279
GL172759.1	27403	28016	27614, 27784, 27853	9, 15, 13	9.72082, 15.26186, 13.97446	6.64182, 8.92711, 8.57174	6.97155, 1	3	tmem150b	21910
GL172759.1	50330	50608	50355, 50385, 50394	11, 17, 6	12.17114, 17.71433, 7.29403	7.82679, 9.9018, 5.30581	9.28263, 1	3	tmem150b	0
GL172759.1	82310	83128	82390, 82523, 82361	10, 17, 8	8.75281, 14.97125, 10.0913	5.9692, 8.24108, 6.79758	6.07523, 1	3	NA	4773
GL172759.1	388452	388912	388656, 388642, 388684	7, 13, 7	6.69449, 10.89905, 7.72582	5.04797, 6.72447, 5.6058	4.1953, 8.2	3	kcnj14	22327
GL172759.1	397481	398655	398094, 398101, 398218	16, 28, 12	17.08121, 26.61073, 15.80834	9.7724, 11.68245, 9.60504	13.95894,	3	kcnj14	12584
GL172759.1	463970	464299	464117, 464029, 464074	7, 11, 6	5.82861, 9.8562, 6.71074	4.53157, 6.47973, 5.03498	3.42929, 7	3	cyth1	0
GL172759.1	638561	639051	638621, 638625, 638668	6, 13, 5	5.86227, 12.7389, 5.91431	4.59138, 7.84853, 4.53172	3.46055, 9	3	lmtk3	13995
GL172759.1	1021350	1022212	1021720, 1021733, 1021707	83, 99, 40	88.4267, 98.73758, 42.58205	21.31346, 19.71524, 16.19503	84.14669,	3	grin2d	213473
GL172759.1	1112386	1114320	1113514, 1113495, 1113473	79, 118, 61	66.48065, 103.7124, 61.40394	13.81656, 16.1226, 17.47548	62.42073,	3	grin2d	121365
GL172759.1	1562024	1562352	1562323, 1562326, 1562317	28, 36, 36	32.49485, 38.35513, 47.74979	15.17061, 15.43492, 20.95343	28.93256,	3	kdelr2	49385
GL172759.1	1616869	1617565	1617270, 1617271, 1617270	17, 31, 14	16.29925, 31.11912, 15.80333	9.04982, 13.20829, 9.43935	13.21124,	3	kdelr2	0
GL172759.1	1813002	1814092	1813444, 1813787, 1813787	8, 15, 7	6.94372, 14.33606, 8.646	5.12297, 8.37011, 6.04229	4.42528, 1	3	myadm	7545
GL172759.1	1983445	1985350	1983851, 1983970, 1983794, 1985218	10, 19, 7, 3	7.54524, 16.30954, 8.21885, 3.27564	5.21478, 8.49413, 5.84717, 2.93941	4.96292, 1	3	prkcg	7129
GL172759.1	1983445	1985350	1984573, 1983970, 1983794, 1985218	7, 19, 7, 3	4.53203, 16.30954, 8.21885, 3.27564	3.70201, 8.49413, 5.84717, 2.93941	2.3247, 13	3	prkcg	7129
GL172759.1	2242877	2243283	2243079, 2243147, 2242988	5, 9, 6	4.27048, 7.17446, 7.18591	3.6666, 5.1397, 5.25776	2.09564, 4	3	cacng8	83206
GL172759.1	2314840	2315438	2315320, 2315320, 2315159	13, 15, 8	12.87577, 14.21432, 8.62637	7.93025, 8.29617, 6.08668	9.9455, 11	3	pitpnc1	18714
GL172759.1	2324568	2325324	2324910, 2325010, 2325244	6, 16, 7	4.93136, 15.80758, 7.40077	4.04392, 9.01376, 5.43746	2.61555, 1	3	pitpnc1	8828
GL172759.1	2405933	2406359	2406311, 2406311, 2406333	22, 32, 14	23.7162, 34.91565, 19.68329	12.03186, 15.04994, 11.3696	20.37115,	3	pitpnc1	0
GL172759.1	2562717	2563790	2563214, 2562867, 2563524	8, 12, 8	7.99469, 10.16897, 8.9249	5.74128, 6.4829, 6.24211	5.38155, 7	3	FAM83E	0
GL172760.1	495574	496352	495899, 495923, 495850	9, 18, 6	8.0222, 14.80783, 6.2131	5.66446, 7.8701, 4.78171	5.40727, 1	3	cbl	0
GL172760.1	1752477	1753274	1752971, 1752964, 1752983	10, 9, 5	9.18236, 6.55163, 5.27517	6.23091, 4.74494, 4.22616	6.47811, 4	3	oaf	43208
GL172760.1	2225701	2226229	2225988, 2225744, 2225935	6, 5, 5	5.96715, 3.9388, 5.85847	4.64927, 3.46289, 4.50665	3.52158, 1	3	grik4	0
GL172760.1	2365859	2367257	2366624, 2366418, 2366878	24, 36, 15	23.17278, 34.76818, 16.75372	11.04149, 13.38886, 9.79627	19.84345,	3	tmem136	13672
GL172761.1	14338	15346	14765, 15123, 14758	14, 13, 5	15.31941, 10.68145, 5.9506	9.1665, 6.58965, 4.54784	12.27033,	3	adam17	0
GL172761.1	672634	673098	673033, 672963, 673030	29, 46, 25	31.53713, 51.67, 31.16689	14.22213, 18.74068, 15.43191	27.99519,	3	id2	250693
GL172761.1	949987	950181	950091, 950003, 950015	9, 11, 10	9.43487, 10.60895, 12.71002	6.48595, 6.9343, 8.12734	6.71665, 7	3	rnf144a	115852
GL172761.1	955198	955798	955520, 955552, 955756	15, 20, 8	16.28407, 21.26374, 10.1465	9.52725, 11.18517, 6.82176	13.19656,	3	rnf144a	110235
GL172761.1	1003580	1004171	1003586, 1003599, 1003646	26, 32, 15	30.99137, 35.52189, 20.14748	15.00173, 15.40916, 11.5698	27.46178,	3	rnf144a	61862
GL172761.1	1182525	1182817	1182626, 1182625, 1182626	15, 25, 15	15.56593, 27.15225, 19.64455	9.10751, 13.07691, 11.32852	12.50679,	3	cmpk2	49530
GL172761.1	1200451	1200924	1200577, 1200653, 1200849	11, 8, 6	11.71035, 7.07159, 6.71074	7.57196, 5.20073, 5.03498	8.8442, 4.6	3	cmpk2	67456
GL172761.1	1336933	1337324	1337275, 1337147, 1337212	15, 10, 5	14.52484, 7.61995, 5.45388	8.4845, 5.26198, 4.3157	11.51408,	3	NA	1696
GL172761.1	1570725	1571285	1571257, 1571257, 1571258	46, 59, 48	56.13528, 67.64761, 63.48825	21.32498, 21.57862, 24.66364	52.1975, 6	3	sox11	118812
GL172761.1	1610116	1610642	1610257, 1610476, 1610390	10, 13, 7	10.39648, 12.93015, 7.50542	6.94096, 7.96262, 5.49244	7.61003, 1	3	sox11	79455
GL172761.1	1685224	1685603	1685271, 1685238, 1685318	10, 10, 9	9.23294, 9.07627, 11.58206	6.26141, 6.1667, 7.55286	6.52626, 6	3	sox11	4494
GL172761.1	1845990	1849029	1848051, 1848346, 1847033, 1848315	24, 25, 10, 11	20.48755, 19.29692, 9.05276, 10.01445	9.46044, 8.49211, 6.15243, 6.57631	17.24176,	3	sox11	154740
GL172761.1	2143102	2143697	2143382, 2143225, 2143234	23, 26, 21	24.49582, 25.69636, 24.28039	12.17168, 11.85306, 12.67014	21.12838,	3	allc	216033
GL172761.1	2233006	2233577	2233014, 2233118, 2233032	35, 21, 20	42.42841, 21.73308, 26.76487	18.25752, 11.16072, 14.27334	38.68701,	3	allc	126153
GL172761.1	2269329	2269841	2269615, 2269556, 2269427	8, 19, 15	8.42257, 19.64509, 19.06531	5.97763, 10.50948, 11.0407	5.76336, 1	3	allc	89889
GL172761.1	2355855	2356286	2355922, 2355896, 2355923	13, 16, 9	11.87199, 14.69174, 11.64359	7.32374, 8.33598, 7.57973	8.9979, 11	3	allc	3444
GL172761.1	2420838	2422211	2421233, 2422052, 2421153	7, 15, 11	6.12952, 11.01021, 12.34038	4.71533, 6.33676, 7.91841	3.67231, 8	3	NA	1658
GL172762.1	33650	34148	34120, 34122, 34121	45, 71, 43	53.0727, 86.65632, 59.13947	20.01229, 25.97392, 24.50877	49.17743,	3	NA	8814
GL172762.1	74895	75700	75423, 75162, 75224	7, 8, 4	6.01305, 6.37769, 4.59709	4.6447, 4.77199, 3.75554	3.56347, 4	3	NA	0
GL172762.1	92316	92738	92566, 92618, 92531	10, 10, 6	9.18236, 7.68845, 6.71074	6.23091, 5.30522, 5.03498	6.47811, 5	3	NA	0

GL172762.1	132546	132867	132821, 132840, 132816	9, 8, 5	9.56822, 6.34359, 4.99237	6.55912, 4.75053, 4.0781	6.84121, 3	3	NA	0
GL172762.1	193477	193873	193537, 193504, 193526	43, 72, 32	53.05693, 84.89689, 41.91822	20.94451, 24.52567, 19.16778	49.16193,	3	NA	53468
GL172762.1	237192	237555	237517, 237483, 237524	22, 21, 10	25.29347, 19.00078, 10.84317	12.96511, 9.5157, 7.18868	21.90464,	3	NA	97183
GL172762.1	351944	352841	352443, 352363, 352263	8, 15, 8	8.42257, 15.05167, 8.80246	5.97763, 8.8016, 6.179	5.76336, 1	3	NA	211935
GL172762.1	366623	367305	366657, 366924, 366668	7, 9, 5	6.01305, 7.59096, 5.75061	4.6447, 5.39978, 4.45735	3.56347, 5	3	NA	226614
GL172762.1	424875	425235	425163, 425216, 425136	8, 10, 4	7.28955, 8.4053, 4.67325	5.33177, 5.75445, 3.78987	4.72176, 5	3	NA	284866
GL172762.1	435070	435947	435407, 435489, 435214	19, 17, 17	18.07136, 14.56175, 18.96688	9.55936, 7.99171, 10.63714	14.91224,	3	NA	295061
GL172762.1	458812	459459	459205, 459218, 459111	8, 6, 6	7.47668, 3.54303, 5.99481	5.44269, 3.14356, 4.6644	4.89713, 1	3	NA	318803
GL172762.1	516086	516664	516474, 516461, 516589	10, 17, 12	11.06011, 18.56115, 16.36823	7.306, 10.40145, 9.85365	8.22712, 1	3	NA	376077
GL172762.1	541776	542185	542136, 541976, 541989	8, 9, 6	8.28589, 8.21099, 6.03707	5.90321, 5.77858, 4.6874	5.65299, 5	3	NA	401767
GL172762.1	605150	605860	605180, 605223, 605224	11, 20, 14	12.01255, 22.87459, 19.68329	7.73996, 12.13503, 11.3696	9.13199, 1	3	NA	465141
GL172762.1	862507	862928	862898, 862881, 862877	18, 36, 25	19.17347, 39.43118, 33.83951	10.50657, 16.06286, 16.91005	15.97256,	3	c3orf67	685620
GL172762.1	890302	890577	890510, 890551, 890521	7, 17, 6	6.33443, 14.4143, 5.53406	4.83795, 7.90204, 4.40503	3.86259, 1	3	c3orf67	657971
GL172762.1	907688	909055	908976, 908348, 908791	19, 34, 16	16.92778, 30.24646, 14.74849	8.86687, 11.62578, 8.37062	13.8105, 2	3	c3orf67	639493
GL172762.1	1124572	1125231	1125022, 1125126, 1124945	12, 9, 7	13.8483, 8.21099, 8.69489	8.63436, 5.77858, 6.06379	10.86694,	3	c3orf67	423317
GL172762.1	1184233	1184763	1184556, 1184566, 1184549	13, 18, 13	13.20994, 16.01233, 15.17843	8.12823, 8.5983, 9.23814	10.26285,	3	c3orf67	363785
GL172762.1	1187997	1188590	1188034, 1188179, 1188287	10, 9, 6	8.28204, 4.80498, 5.0826	5.67774, 3.6298, 4.13667	5.64968, 2	3	c3orf67	359958
GL172762.1	1191977	1192511	1192229, 1192219, 1192283	8, 18, 11	7.15567, 14.90085, 11.92945	5.25151, 7.92604, 7.69409	4.61046, 1	3	c3orf67	356037
GL172762.1	1276328	1276901	1276676, 1276830, 1276869	11, 18, 13	12.43693, 18.83799, 16.28606	7.97018, 10.30699, 9.81016	9.52692, 1	3	c3orf67	271647
GL172762.1	1402996	1403517	1403204, 1403487, 1403429	6, 17, 7	5.55471, 15.0242, 7.40077	4.41698, 8.27335, 5.43746	3.18192, 1	3	c3orf67	145031
GL172762.1	1418716	1418968	1418877, 1418966, 1418917	11, 22, 8	10.27095, 20.12386, 9.11678	6.73177, 9.87545, 6.33924	7.4917, 17	3	c3orf67	129580
GL172762.1	1492577	1493036	1492627, 1492785, 1492726	5, 9, 3	4.82177, 8.21099, 3.47371	3.98509, 5.77858, 3.03189	2.51418, 5	3	c3orf67	55512
GL172762.1	1533471	1534917	1534440, 1534062, 1533572	8, 14, 3	7.77807, 13.14791, 3.47371	5.618, 7.88211, 3.03189	5.17956, 1	3	c3orf67	13631
GL172762.1	1545567	1546127	1546071, 1546046, 1546095	54, 55, 35	69.18291, 68.34948, 46.97587	24.95476, 23.95694, 20.91031	65.09489,	3	c3orf67	2421
GL172762.1	1552636	1553243	1552958, 1552808, 1553065	6, 16, 4	5.03089, 14.46749, 4.50795	4.10511, 8.19898, 3.71446	2.70624, 1	3	c3orf67	0
GL172762.1	1584827	1585247	1585043, 1585015, 1585069	8, 7, 3	7.38183, 5.58561, 3.17999	5.38666, 4.38027, 2.89245	4.80856, 3	3	c3orf67	0
GL172762.1	1635359	1635783	1635617, 1635779, 1635779	22, 32, 15	24.67026, 33.8711, 17.85348	12.59865, 14.4331, 10.40618	21.29787,	3	c3orf67	0
GL172762.1	1783338	1784230	1783376, 1783389, 1783631	24, 29, 12	27.29272, 28.65304, 12.95194	13.50336, 12.5148, 8.14303	23.84909,	3	MGC107780	7442
GL172762.1	1799019	1799608	1799286, 1799318, 1799224	24, 50, 19	25.27938, 53.2696, 21.69099	12.30321, 17.84962, 11.71707	21.89126,	3	MGC107780	23123
GL172762.1	1860989	1861738	1861491, 1861496, 1861155	13, 23, 11	10.62846, 20.1396, 11.92945	6.5568, 9.56971, 7.69409	7.83043, 1	3	fam107a	6638
GL172762.1	1863505	1863798	1863710, 1863760, 1863738	9, 13, 7	6.96255, 8.22922, 6.36213	5.00603, 5.08172, 4.85436	4.44307, 5	3	fam107a	9154
GL172762.1	1874160	1875017	1874726, 1874777, 1874700	8, 13, 9	7.28955, 10.35117, 10.28145	5.33177, 6.38482, 6.93568	4.72176, 7	3	pde12	3088
GL172762.1	1889880	1890481	1890414, 1890304, 1890163	14, 19, 13	13.26657, 15.76714, 13.70995	7.95422, 8.16901, 8.41989	10.31735,	3	arf4	0
GL172762.1	1903550	1904459	1904114, 1903802, 1903819	6, 9, 4	4.51306, 8.21099, 3.64768	3.78094, 5.77858, 3.26758	2.30616, 5	3	arf4	4426
GL172762.1	1915038	1915318	1915048, 1915040, 1915070	33, 37, 24	35.34935, 39.98539, 29.93386	14.90321, 15.96556, 15.03552	31.72944,	3	dennd6a	0
GL172762.1	1941536	1941890	1941856, 1941853, 1941853	22, 18, 20	24.77128, 16.46278, 22.4589	12.65828, 8.87194, 11.89249	21.39609,	3	dennd6a	0
GL172762.1	1962146	1962818	1962391, 1962455, 1962519	5, 11, 5	3.81096, 9.26343, 5.23257	3.3822, 6.11409, 4.20435	1.68502, 6	3	slmap	3465
GL172762.1	2053134	2053526	2053393, 2053370, 2053349	7, 9, 6	6.64707, 7.51164, 5.793	5.02073, 5.35055, 4.55271	4.15135, 5	3	slmap	0
GL172762.1	2073358	2073695	2073596, 2073541, 2073589	27, 31, 14	20.85352, 23.54024, 11.02224	8.76924, 9.04275, 6.57526	17.59719,	3	flnb	0
GL172762.1	2076124	2076511	2076429, 2076450, 2076408	16, 17, 8	10.12729, 9.27539, 5.33388	5.59546, 4.93219, 4.10413	7.35658, 6	3	flnb	0
GL172762.1	2077213	2078569	2078054, 2078055, 2078054	25, 48, 26	19.02791, 42.72782, 25.66676	8.34516, 13.21064, 11.83547	15.8319, 3	3	flnb	0
GL172762.1	2095224	2095847	2095536, 2095534, 2095576	11, 9, 6	10.16451, 6.20816, 6.35308	6.66744, 4.52542, 4.85498	7.39114, 3	3	flnb	0
GL172762.1	2141336	2141822	2141703, 2141688, 2141649	14, 9, 6	12.8072, 6.55163, 5.83216	7.67424, 4.74494, 4.57461	9.88012, 4	3	flnb	0
GL172762.1	2233430	2234204	2233865, 2233874, 2233890	9, 8, 7	9.36986, 7.07159, 8.69489	6.44997, 5.20073, 6.06379	6.65425, 4	3	dnase1l3	1125
GL172762.1	2263100	2263876	2263286, 2263600, 2263596	5, 13, 5	4.40263, 10.55511, 5.50079	3.74533, 6.51132, 4.33868	2.21004, 7	3	abhd6	4128
GL172762.1	2269710	2270335	2269729, 2269754, 2269956	19, 24, 10	19.33502, 20.11544, 10.59656	10.32316, 9.24605, 7.05292	16.12917,	3	abhd6	0
GL172762.1	2297299	2298391	2297528, 2297802, 2298040	5, 9, 5	4.82177, 7.10358, 4.80993	3.98509, 5.09508, 3.97854	2.51418, 4	3	abhd6	18494
GL172762.1	2358259	2358597	2358278, 2358262, 2358270	33, 59, 22	36.54568, 65.22912, 25.77562	15.6093, 20.27414, 13.24605	32.90263,	3	pxk	0
GL172762.1	2367659	2368118	2368080, 2368043, 2368052	12, 21, 14	9.15708, 19.29849, 13.98966	5.85272, 9.69431, 8.38968	6.45494, 1	3	pxk	0
GL172762.1	2402236	2403287	2403101, 2402743, 2403104	8, 13, 6	8.28589, 10.11663, 6.5517	5.90321, 6.23937, 4.95623	5.65299, 7	3	NA	1125
GL172762.1	2427163	2427950	2427929, 2427916, 2427913	23, 37, 19	22.28866, 38.75833, 21.69099	10.84702, 15.25569, 11.71707	18.98693,	3	NA	0
GL172762.1	2442178	2442765	2442725, 2442715, 2442732	70, 99, 53	85.05456, 122.62537, 68.30876	25.61713, 30.22312, 24.96003	80.80694,	3	NA	0
GL172762.1	2523695	2524091	2524060, 2524053, 2524033	24, 22, 29	28.77451, 21.70761, 36.97725	14.37118, 10.82585, 17.42526	25.29621,	3	hesx1	0
GL172762.1	2535399	2535895	2535705, 2535672, 2535735	8, 9, 6	7.67464, 8.21099, 6.5517	5.55832, 5.77858, 4.95623	5.08299, 5	3	hesx1	10674
GL172762.1	2557783	2558508	2558109, 2558402, 2558353	7, 12, 8	7.1696, 11.86108, 9.8421	5.31345, 7.51216, 6.68602	4.61046, 9	3	hesx1	33058
GL172763.1	163596	164566	164033, 164033, 164025	4, 10, 4	3.28906, 7.75815, 4.34116	3.0555, 5.34918, 3.63492	1.37521, 5	3	celf4	53300
GL172763.1	180721	180941	180723, 180747, 180789	6, 28, 8	5.51356, 29.57832, 9.1831	4.39314, 13.43449, 6.37229	3.14574, 2	3	celf4	70425
GL172763.1	276707	277081	276867, 276838, 276996	20, 17, 16	19.99469, 14.66185, 17.78134	10.42292, 8.05262, 10.17907	16.76522,	3	celf4	166411
GL172763.1	293591	293939	293893, 293898, 293862	9, 14, 8	8.77314, 13.63145, 8.14528	6.111, 8.17488, 5.82558	6.09355, 1	3	celf4	183295
GL172763.1	297432	297728	297620, 297678, 297698	7, 16, 8	7.1696, 17.16856, 9.31951	5.31345, 9.82359, 6.43944	4.61046, 1	3	celf4	187136
GL172763.1	353989	354452	354394, 354282, 354361	12, 25, 15	9.6142, 20.15269, 14.11641	6.1379, 8.96614, 8.2366	6.88539, 1	3	celf4	243693
GL172763.1	358180	358829	358668, 358641, 358527	8, 16, 7	5.47927, 11.4149, 6.01639	4.19817, 6.34731, 4.64674	3.11408, 8	3	celf4	247884
GL172763.1	365244	366171	365685, 365424, 365672, 366130	7, 11, 8, 5	5.30682, 8.59076, 8.35103, 4.84526	4.20391, 5.69393, 5.93881, 3.99806	2.95991, 6	3	celf4	254948
GL172763.1	367404	367813	367703, 367635, 367780	9, 13, 7	9.00096, 11.71436, 7.61369	6.24222, 7.22722, 5.54854	6.30808, 9	3	celf4	257108
GL172763.1	393303	394505	393683, 393822, 393817	115, 155, 60	120.68037, 160.22816, 66.15967	22.54546, 23.22659, 20.31359	116.12573	3	celf4	283007
GL172763.1	417931	418539	418354, 418507, 418043	18, 26, 15	18.52782, 25.69636, 16.91122	10.1215, 11.85306, 9.88541	15.34982,	3	celf4	307635
GL172763.1	474338	474570	474383, 474371, 474535	6, 7, 9	4.86712, 5.2482, 10.72164	4.00414, 4.16649, 7.15493	2.55558, 3	3	celf4	364042
GL172763.1	484158	484940	484772, 484760, 484368	9, 21, 6	7.32038, 20.46506, 6.76586	5.23124, 10.39685, 5.06179	4.75116, 1	3	celf4	373862
GL172763.1	492245	492837	492579, 492293, 492298	7, 11, 5	6.12952, 9.05476, 5.91431	4.71533, 5.98422, 4.53172	3.67231, 6	3	celf4	381949
GL172763.1	520797	521420	520820, 520856, 520965	8, 13, 4	8.42257, 13.14514, 4.67325	5.97763, 8.09002, 3.78987	5.76336, 1	3	celf4	410501
GL172763.1	556911	557355	556957, 556975, 556931	19, 20, 7	18.53897, 17.08177, 7.61369	9.84257, 8.66794, 5.54854	15.36013,	3	celf4	446615
GL172763.1	600612	600968	600910, 600935, 600936	14, 19, 19	16.7649, 19.96513, 25.95226	9.96273, 10.7011, 14.01451	13.6543, 1	3	celf4	490316
GL172763.1	671924	672648	672251, 672362, 672127	8, 14, 9	7.47668, 12.43922, 9.70054	5.44269, 7.44862, 6.63089	4.89713, 9	3	celf4	561628
GL172763.1	717085	717527	717192, 717120, 717150	4, 8, 6	3.74195, 7.07159, 7.29403	3.32091, 5.20073, 5.30581	1.6222, 4.6	3	celf4	606789
GL172763.1	731079	731381	731318, 731329, 731330	7, 10, 5	7.1696, 9.39128, 5.9506	5.31345, 6.35644, 4.54784	4.61046, 6	3	celf4	620783
GL172763.1	747737	748014	747886, 747897, 747948	13, 22, 4	14.70302, 22.85945, 4.67325	8.98015, 11.51868, 3.78987	11.68444,	3	celf4	637441
GL172763.1	805511	806298	805963, 805901, 805929	24, 33, 10	23.67742, 33.77941, 10.84317	11.34307, 13.98464, 7.18868	20.33353,	3	celf4	695215
GL172763.1	865015	865759	865337, 865122, 865511	5, 6, 3	4.36883, 4.93312, 3.47371	3.72533, 4.04501, 3.03189	2.18201, 2	3	celf4	754719
GL172763.1	928637	929537	928942, 929030, 928885	7, 19, 13	6.33443, 16.78404, 13.58227	4.83795, 8.78005, 8.34597	3.86259, 1	3	celf4	818341
GL172763.1	930705	931765	931554, 931296, 930796	11, 15, 5	11.02968, 12.49102, 4.07202	7.18222, 7.24068, 3.54571	8.20839, 9	3	celf4	820409
GL172763.1	938201	938525	938461, 938490, 938494	10, 19, 11	10.6018, 20.82621, 14.75772	7.05583, 11.21218, 9.09568	7.80484, 1	3	celf4	827905
GL172763.1	1010795	1011572	1010967, 1011015, 1011031	12, 11, 12	13.8483, 10.60895, 16.28773	8.63436, 6.9343, 9.81872	10.86694,	3	celf4	900499
GL172763.1	1072939	1073553	1073336, 1073167, 1073198	7, 18, 5	6.69449, 16.94269, 5.3628	5.04797, 9.16356, 4.27046	4.1953, 14	3	celf4	962643
GL172763.1	1109031	1109602	1109403, 1109261, 1109443	7, 6, 4	6.05136, 4.06564, 4.38152	4.66801, 3.49079, 3.65448	3.59939, 1	3	celf4	998735
GL172763.1	1283286	1284023	1283648, 1283669, 1283655	7, 6, 6	6.42049, 4.93312, 6.99807	4.8888, 4.04501, 5.17195	3.94161, 2	3	pik3c3	991656
GL172763.1	1290905	1291307	1291101, 1291038, 1291026	5, 8, 5	4.61714, 7.07159, 5.64747	3.86998, 5.20073, 4.40911	2.39372, 4	3	pik3c3	984372
GL172763.1	1304005	1304479	1304348, 1304342, 1304204	9, 9, 5	8.0679, 6.8008, 5.3628	5.69219, 4.90352, 4.27046	5.45, 4.421	3	pik3c3	971200
GL172763.1	1343473	1344154	1343602, 1343591, 1343580	19, 36, 12	18.13647, 36.83142, 14.96175	9.59882, 14.55552, 9.20442	14.97405,	3	pik3c3	931525
GL172763.1	1384432	1384736	1384637, 1384663, 1384711	7, 9, 6	7.1696, 8.21099, 7.29403	5.31345, 5.77858, 5.30581	4.61046, 5	3	pik3c3	890943
GL172763.1	1435141	1435501	1435474, 1435467, 1435471	16, 22, 11	18.14851, 22.38114, 13.46208	10.3887, 11.23117, 8.49639	14.98517,	3	pik3c3	840178
GL172763.1	1485875	1486120	1486059, 1486044, 1485956	12, 13, 6	13.3031, 13.14514, 6.25896	8.33871, 8.09002, 4.80589	10.35242,	3	pik3c3	789559
GL172763.1	1525148	1525742	1525649, 1525639, 1525672	8, 15, 8	6.27633, 13.4765, 7.76694	4.70812, 7.84563, 5.6116	3.80887, 1	3	pik3c3	749937
GL172763.1	1527662	1528251	1527854, 1527730, 1528054	6, 14, 6	4.89903, 12.04301, 5.33084	4.02393, 7.20479, 4.28587	2.58474, 9	3	pik3c3	747428
GL172763.1	1544434	1544972	1544465, 1544440, 1544467	57, 81, 47	69.4285, 97.93935, 61.79207	23.55822, 26.85753, 24.1603	65.33811,	3	pik3c3	730707
GL172763.1	1617037	1617705	1617599, 1617672, 1617287	5, 10, 5	4.27048, 8.53104, 5.9506	3.6666, 5.83243, 4.54784	2.09564, 6	3	pik3c3	657974
GL172763.1	1700340	1701268	1700444, 1700975, 1700971	5, 13, 5	4.43696, 12.99541, 5.59755	3.76555, 8.00139, 4.38538	2.24017, 1	3	pik3c3	574411
GL172763.1	1712317	1713160	1712398, 1712788, 1712466	10, 13, 8	8.75281, 11.03409, 8.80246	5.9692, 6.80804, 6.179	6.07523, 8	3	pik3c3	562519
GL172763.1	1733910	1734361	1734136, 1734023, 1734172	5, 7, 5	4.82177, 5.97725, 5.80393	3.98509, 4.62287, 4.48186	2.51418, 3	3	pik3c3	541318
GL172763.1	1743895	1744770	1744634, 1744690, 1744158	12, 15, 7	13.8483, 15.40598, 8.69489	8.63436, 9.01279, 6.06379	10.86694,	3	pik3c3	530909
GL172763.1	1749677	1750057	1749686, 1749747, 1749745	8, 13, 5	8.42257, 12.67661, 5.9506	5.97763, 7.81122, 4.54784	5.76336, 9	3	pik3c3	525622
GL172763.1	1811128	1811775	1811637, 1811266, 1811737	5, 10, 5	4.655, 9.37821, 5.9506	3.89157, 6.34863, 4.54784	2.42652, 6	3	pik3c3	463904
GL172763.1	1826520	1826820	1826525, 1826568, 1826577	5, 15, 8	4.82177, 14.15433, 9.1831	3.98509, 8.25968, 6.37229	2.51418, 1	3	pik3c3	448859
GL172763.1	1919245	1919880	1919414, 1919399, 1919713	8, 14, 8	8.05102, 12.75552, 8.4586	5.77295, 7.64262, 5.99709	5.43473, 1	3	pik3c3	355799
GL172763.1	1995988	1996685	1996031, 1996006, 1996094	12, 20, 14	11.98519, 19.64549, 17.61756	7.58551, 10.21207, 10.40352	9.10644, 1	3	pik3c3	278994
GL172763.1	2029038	2029471	2029066, 2029047, 2029041	10, 12, 7	10.96713, 11.20359, 8.49722	7.25596, 7.11794, 5.97582	8.14942, 8	3	pik3c3	246208
GL172763.1	2055359	2055923	2055402, 2055479, 2055392	12, 23, 13	11.73328, 24.41444, 14.85247	7.43625, 12.12294, 9.06201	8.86615, 2	3	pik3c3	219756
GL172763.1	2088153	2088818	2088479, 2088432, 2088190	12, 15, 12	11.43506, 15.19102, 13.59027	7.25774, 8.88488, 8.49571	8.58315, 1	3	pik3c3	186861
GL172763.1	2135955	2136385	2135992, 2135991, 2135961	35, 37, 31	41.26296, 41.10621, 39.58168	17.56792, 16.61993, 18.19777	37.54033,	3	pik3c3	139294
GL172763.1	2161372	2162058	2161573, 2161583, 2161888	19, 13, 10	19.48878, 11.56107, 11.24131	10.41563, 7.13311, 7.40241	16.2775, 8	3	pik3c3	113621
GL172763.1	2173675	2174200	2173806, 2173738, 2173857	7, 16, 3	6.84137, 15.41438, 3.47371	5.13151, 8.77591, 3.03189	4.33107, 1	3	pik3c3	101479
GL172763.1	2181040	2181997	2181251, 2181678, 2181386	9, 10, 6	8.66407, 8.74956, 7.29403	6.04743, 5.96721, 5.30581	5.99101, 6	3	pik3c3	93682
GL172763.1	2194398	2194843	2194408, 2194496, 2194507	5, 11, 6	4.82177, 10.60895, 7.18591	3.98509, 6.9343, 5.25776	2.51418, 7	3	pik3c3	80836
GL172763.1	2220954	2221508	2221084, 2220990, 2220999	16, 21, 16	15.34845, 20.67581, 18.26684	8.73591, 10.52396, 10.45568	12.2978, 1	3	pik3c3	54171
GL172763.1	2230056	2231319	2230565, 2230469, 2231088	11, 6, 12	11.56615, 4.43108, 11.81848	7.49066, 3.72839, 7.4869	8.70729, 2	3	pik3c3	44360
GL172763.1	2446536	2447151	2446730, 2446905, 2446830	6, 6, 5	5.96715, 4.92507, 4.77515	4.64927, 4.04003, 3.9592	3.52158, 2	3	pik3c3	56277
GL172763.1	2454686	2455187	2455159, 2455160, 2455119	64, 84, 53	76.89835, 102.03551, 70.44074	24.20331, 27.45706, 26.20892	72.73295,	3	pik3c3	64427
GL172763.1	2480104	2480501	2480339, 2480187, 2480214	6, 9, 4	5.96715, 7.67202, 4.67325	4.64927, 5.44992, 3.78987	3.52158, 5	3	pik3c3	89845
GL172763.1	2491292	2491876	2491449, 2491591, 2491658	5, 7, 4	4.73273, 5.97725, 4.30163	3.93547, 4.62287, 3.61556	2.49377, 3	3	pik3c3	101033
GL172763.1	2504469	2504788	2504702, 2504702, 2504668	23, 31, 13	27.72716, 37.47519, 17.16464	14.07465, 16.96708, 10.23255	24.27442,	3	pik3c3	114210
GL172764.1	963506	964153	963796, 963864, 963836	9, 9, 6	9.12009, 7.10358, 6.25896	6.30997, 5.09508, 4.80589	6.42028, 4	3	cdh8	322259
GL172764.1	1189610	1189801	1189734, 1189782, 1189782	24, 37, 18	27.08274, 37.28955, 22.99911	13.37906, 14.41741, 12.66051	23.64539,	3	adamts18	481633
GL172764.1	1459024	1459744	1459405, 1459250, 1459601	8, 10, 10	7.47668, 7.97488, 9.63365	5.44269, 5.48553, 6.50047	4.89713, 5	3	adamts18	211690
GL172764.1	1464747	1465735	1465108, 1465255, 1465255	19, 40, 17	17.15489, 40.41951, 17.26186	9.00419, 14.9748, 9.63127	14.02972,	3	adamts18	205699
GL172764.1	1933328	1933891	1933484, 1933787, 1933797	8, 15, 9	7.88469, 15.70444, 11.64359	5.67897, 9.18918, 7.57973	5.27935, 1	3	NA	9817
GL172765.1	88952	89388	89261, 89246, 89128	18, 18, 5	14.97029, 11.49098, 3.46822	7.96784, 5.94126, 3.16061	11.94123,	3	NA	7442
GL172765.1	118447	119198	118792, 118787, 118793	19, 51, 30	24.06881, 61.35504, 39.73233	13.04466, 21.92608, 18.64404	20.71337,	3	NA	17641
GL172765.1	124260	125683	125111, 124844, 124474	19, 22, 17	23.04311, 25.60195, 23.46806	12.48444, 13.14513, 13.01603	19.71723,	3	NA	18930
GL172765.1	166566	167338	167044, 167027, 167032	19, 24, 11	19.10984, 24.46445, 11.08949	10.1875, 11.8146, 7.21705	15.91125,	3	NA	20273
GL172765.1	210663	211291	211046, 211029, 211059	15, 20, 10	15.64184, 19.78674, 12.16489	9.15231, 10.29739, 7.87042	12.57992,	3	NA	64370
GL172765.1	375560	376237	375930, 375994, 375752	4, 9, 7	3.51298, 7.63127, 7.66926	3.1896, 5.42473, 5.57703	1.55088, 5	3	NA	229267
GL172765.1	406762	407083	407049, 407033, 407037	11, 6, 11	12.43693, 4.68027, 13.0311	7.97018, 3.88713, 8.28053	9.52692, 2	3	NA	260469
GL172765.1	419891	421084	420536, 420576, 420132	14, 15, 14	13.44866, 14.52325, 16.46576	8.06455, 8.48354, 9.80275	10.49073,	3	NA	273598
GL172765.1	1045628	1046335	1046007, 1046024, 1045880	63, 120, 43	62.14256, 118.30644, 42.27215	17.05769, 20.10682, 14.79757	58.13147,	3	ephb1	2544
GL172765.1	1119130	1119737	1119327, 1119396, 1119422	8, 10, 5	7.72597, 7.48641, 5.91431	5.588, 5.17758, 4.53172	5.13061, 5	3	IRAK1BP1	11224
GL172765.1	1745873	1746156	1746011, 1746004, 1746044	4, 7, 5	3.74195, 5.97725, 5.9506	3.32091, 4.62287, 4.54784	1.6222, 3.6	3	NA	12341
GL172765.1	1754102	1754691	1754643, 1754646, 1754651	13, 15, 4	12.81131, 13.92002, 4.67325	7.8918, 8.11689, 3.78987	9.88323, 1	3	NA	20570
GL172765.1	1979254	1979804	1979764, 1979795, 1979602	6, 14, 7	5.81608, 14.45897, 8.69489	4.56563, 8.66788, 6.06379	3.4176, 11	3	NA	0
GL172765.1	2122784	2123172	2122788, 2122813, 2122784	16, 23, 14	19.76814, 25.61868, 16.73403	11.27773, 12.84132, 9.94634	16.54793,	3	NA	11012
GL172765.1	2311397	2311853	2311674, 2311621, 2311557	8, 11, 7	8.42257, 10.60895, 8.69489	5.97763, 6.9343, 6.06379	5.76336, 7	3	NA	3972
GL172765.1	2410844	2411241	2410967, 2410871, 2410901	16, 34, 11	18.75167, 36.82624, 13.93476	10.72694, 15.36508, 8.7238	15.56543,	3	nelfb	548
GL172766.1	186	1285	918, 339, 1141	11, 14, 8	9.26086, 12.18837, 7.67847	6.1125, 7.29433, 5.56054	6.5517, 9.4	3	lrch1	51876
GL172766.1	662217	662761	662586, 662560, 662587	16, 21, 5	17.2479, 19.86199, 5.45388	9.86999, 10.03329, 4.3157	14.1196, 1	3	acvr1b	153
GL172766.1	1669115	1670221	1669566, 1669362, 1669709	27, 42, 22	26.25209, 39.92506, 25.3961	11.82739, 13.92837, 13.0251	22.83518,	3	fkbp11	17938
GL172766.1	1760356	1761656	1760828, 1760700, 1760703	49, 77, 24	48.20234, 76.05447, 23.27435	15.57546, 18.15236, 11.10212	44.37595,	3	wnt10b	0
GL172766.1	2151169	2152912	2152155, 2152152, 2152155	12, 24, 14	10.25464, 19.38345, 15.13575	6.53598, 8.82879, 9.06144	7.47735, 1	3	tuba1c	5667
GL172766.1	2359744	2360355	2360092, 2359995, 2359940	6, 17, 10	5.63898, 15.13134, 11.75646	4.46544, 8.33865, 7.66841	3.25772, 1	3	dnajc22	6258
GL172767.1	87412	88369	87735, 87600, 87481	14, 49, 17	12.53762, 46.10937, 18.35257	7.50905, 14.51365, 10.27929	9.62265, 4	3	cyp2j2	0
GL172767.1	1500946	1501495	1501156, 1501072, 1501170	7, 9, 8	6.2098, 6.64303, 8.9249	4.76362, 4.80319, 6.24211	3.74679, 4	3	adamts16	284765
GL172767.1	1638731	1639354	1639250, 1639009, 1638836	8, 10, 4	8.42257, 9.39128, 4.67325	5.97763, 6.35644, 3.78987	5.76336, 6	3	adamts16	146906
GL172767.1	2248980	2249667	2249304, 2249236, 2249343	7, 11, 4	7.1696, 10.15827, 4.67325	5.31345, 6.66366, 3.78987	4.61046, 7	3	ahrr	9472
GL172768.1	2062447	2063326	2062960, 2062966, 2063106	16, 14, 16	16.01695, 11.45303, 21.00727	9.13983, 6.84088, 11.91233	12.9408, 8	3	myom2	32048
GL172769.1	15747	16110	16075, 16028, 16028	6, 8, 4	5.20598, 6.97779, 4.15126	4.2113, 5.14375, 3.54016	2.86702, 4	3	pfkp	297199
GL172769.1	167453	167898	167509, 167510, 167477	12, 20, 10	12.05017, 19.64549, 10.90706	7.62377, 10.21207, 7.22344	9.16715, 1	3	pfkp	145411
GL172769.1	200903	201398	201063, 201094, 201041	8, 19, 7	7.88469, 17.52577, 8.08824	5.67897, 9.22875, 5.7849	5.27935, 1	3	pfkp	111911
GL172769.1	411917	412326	412170, 412266, 412290	13, 20, 10	14.10549, 20.22553, 11.75646	8.64628, 10.56212, 7.66841	11.11201,	3	pitrm1	17460
GL172769.1	548345	548780	548535, 548609, 548456	5, 11, 6	3.45653, 8.44639, 7.29403	3.15293, 5.60333, 5.30581	1.50736, 5	3	pitrm1	153888
GL172769.1	734426	734885	734823, 734752, 734830	7, 6, 6	7.1696, 4.93312, 7.29403	5.31345, 4.04501, 5.30581	4.61046, 2	3	pitrm1	339969
GL172769.1	744931	746044	744975, 745580, 745229	6, 18, 5	5.72604, 17.19487, 5.50079	4.51498, 9.31668, 4.33868	3.33547, 1	3	pitrm1	350474
GL172769.1	969901	970175	969904, 969957, 969912	10, 27, 7	10.6018, 28.43981, 8.69489	7.05583, 13.12665, 6.06379	7.80484, 2	3	fzd8	572091
GL172769.1	1019845	1020356	1020143, 1020222, 1020283	7, 13, 5	6.94335, 9.47155, 5.50079	5.18876, 5.84024, 4.33868	4.42528, 6	3	fzd8	521910
GL172769.1	1038554	1038873	1038843, 1038841, 1038847	14, 25, 19	14.16942, 30.45643, 24.35537	8.49669, 15.02432, 13.19734	11.17387,	3	fzd8	503393
GL172769.1	1201623	1202156	1202088, 1201858, 1201970	9, 13, 11	8.88537, 10.68145, 13.28509	6.17591, 6.58965, 8.40871	6.19961, 8	3	fzd8	340110
GL172769.1	1263042	1263661	1263434, 1263433, 1263434	10, 7, 8	8.66311, 5.97725, 9.53432	5.914, 4.62287, 6.54285	5.99101, 3	3	fzd8	278605
GL172769.1	1747070	1747553	1747475, 1747079, 1747151	10, 10, 7	8.24182, 6.85174, 5.70698	5.65265, 4.77545, 4.45614	5.61182, 4	3	cul2	20278
GL172769.1	1750220	1751565	1750587, 1751202, 1751206	9, 14, 6	7.06624, 12.28739, 5.20372	5.07153, 7.35528, 4.20995	4.53919, 9	3	cul2	23428
GL172769.1	1838219	1838809	1838674, 1838668, 1838694	26, 58, 20	27.19703, 61.28687, 23.25697	12.74794, 18.64788, 12.35623	23.75642,	3	pard3	3606
GL172769.1	2014673	2015378	2015046, 2014815, 2015028	8, 10, 5	7.42892, 8.4887, 5.91431	5.41453, 5.80621, 4.53172	4.85345, 5	3	pard3	0
GL172769.1	2136802	2137047	2137019, 2137021, 2137019	19, 39, 21	21.31193, 44.64822, 27.33352	11.49694, 17.82678, 14.37736	18.04063,	3	pard3	46451
GL172770.1	132122	132868	132698, 132515, 132590	20, 18, 7	21.0967, 19.7713, 7.61369	11.08535, 10.85902, 5.54854	17.83215,	3	adarb2	207682
GL172770.1	247236	247796	247629, 247492, 247599	9, 15, 11	8.35434, 15.26186, 10.77078	5.86444, 8.92711, 7.03024	5.71584, 1	3	adarb2	92754
GL172770.1	560257	560499	560289, 560292, 560279	12, 20, 11	11.55224, 20.22553, 11.08949	7.3281, 10.56212, 7.21705	8.69537, 1	3	wdr37	0
GL172770.1	643481	643920	643569, 643546, 643628	8, 10, 9	7.1122, 7.93794, 10.28145	5.22529, 5.46232, 6.93568	4.57946, 5	3	larp4b	0
GL172770.1	965944	966624	966115, 966391, 966532	11, 12, 3	9.90875, 9.42893, 3.47371	6.51186, 6.02236, 3.03189	7.14919, 6	3	DIP2C	0
GL172770.1	1247758	1248269	1248126, 1247955, 1247793	6, 12, 5	5.24248, 10.16897, 4.99237	4.2332, 6.4829, 4.0781	2.89962, 7	3	esyt2	0
GL172770.1	1698063	1698310	1698091, 1698106, 1698077	16, 22, 8	18.34375, 20.947, 10.1465	10.49905, 10.3685, 6.82176	15.17198,	3	xtr-mir-153-2	250476
GL172770.1	1773024	1773525	1773498, 1773394, 1773494	11, 18, 11	10.78278, 16.88096, 13.83651	7.03733, 9.12607, 8.67735	7.97565, 1	3	xtr-mir-153-2	175261
GL172770.1	1815397	1815766	1815695, 1815711, 1815739	13, 23, 15	13.71465, 23.34424, 19.17716	8.42261, 11.48068, 11.09709	10.74495,	3	xtr-mir-153-2	133020
GL172770.1	1975444	1975935	1975705, 1975873, 1975810	9, 11, 6	8.50607, 9.52738, 6.76586	5.95453, 6.27759, 5.06179	5.84194, 6	3	xtr-mir-153-2	26571
GL172770.1	2015541	2016285	2015931, 2016106, 2015843	5, 6, 6	4.27048, 4.93312, 7.29403	3.6666, 4.04501, 5.30581	2.09564, 2	3	xtr-mir-153-2	66668
GL172770.1	2185009	2185892	2185691, 2185415, 2185452	13, 26, 12	12.87577, 23.6039, 13.29687	7.93025, 10.6216, 8.33527	9.9455, 20	3	ube3c	0
GL172770.1	2257073	2257582	2257295, 2257480, 2257296	25, 20, 17	28.23973, 18.00103, 19.29472	13.72472, 9.21905, 10.82557	24.77449,	3	ube3c	11649
GL172771.1	1342	1999	1838, 1652, 1482, 1822	6, 11, 5, 6	5.96715, 10.05535, 5.80393, 7.12188	4.64927, 6.6012, 4.48186, 5.22884	3.52158, 7	3	NA	44087
GL172771.1	7668	8748	8091, 8539, 8582	16, 18, 11	10.58726, 11.72293, 7.44006	5.86089, 6.07064, 4.97137	7.79092, 9	3	NA	37338
GL172771.1	9914	10663	10358, 10494, 10451	16, 22, 15	9.18584, 13.66632, 9.54024	5.06499, 6.24394, 5.46209	6.48152, 1	3	NA	35423
GL172771.1	15452	16634	16033, 15620, 16270	24, 18, 22	13.09246, 7.7207, 13.46039	5.57666, 4.00128, 6.13882	10.15136,	3	NA	29452
GL172771.1	18109	19301	18652, 18665, 18167	18, 23, 17	8.76658, 11.55794, 8.96217	4.50625, 5.04519, 4.76682	6.08885, 8	3	NA	26785
GL172771.1	20104	20578	20547, 20334, 20342	13, 16, 11	5.18287, 5.7245, 4.34454	3.34771, 3.29623, 3.11115	2.84591, 3	3	NA	25508
GL172771.1	44365	45031	44916, 44953, 44873	10, 18, 5	7.41295, 14.06285, 4.67395	5.1311, 7.42469, 3.90232	4.8384, 11	3	NA	1055
GL172771.1	60631	61670	61270, 61313, 61305	12, 21, 10	6.53104, 12.5456, 7.02444	4.24285, 5.87731, 4.88493	4.04435, 9	3	NA	7153
GL172771.1	69972	70998	70335, 70342, 70762	9, 10, 6	9.72082, 9.27513, 7.29403	6.64182, 6.2868, 5.30581	6.97155, 6	3	eif1ax	5792
GL172771.1	452915	453632	453438, 453224, 453285	8, 13, 5	7.72597, 12.2024, 5.9506	5.588, 7.52507, 4.54784	5.13061, 9	3	map3k15	0
GL172772.1	26452	26938	26678, 26483, 26567	7, 15, 4	6.46447, 13.8628, 4.46488	4.91463, 8.08196, 3.69425	3.98312, 1	3	tnfsf15	10056
GL172772.1	52960	53390	53216, 53216, 53216	22, 22, 13	26.67998, 23.10829, 16.0859	13.76591, 11.66803, 9.70995	23.25346,	3	NA	14016
GL172772.1	341340	341875	341413, 341854, 341481	5, 13, 6	3.68584, 9.78383, 6.12365	3.30216, 6.03321, 4.73409	1.6222, 7.1	3	tnc	139047
GL172772.1	352620	353189	353095, 353101, 353053	46, 66, 53	53.60707, 74.74334, 69.65594	19.85311, 22.06772, 25.74879	49.70391,	3	tnc	150327
GL172772.1	357984	358497	358050, 358171, 358079	17, 13, 10	15.92284, 12.2024, 9.2231	8.82098, 7.52507, 6.25548	12.84985,	3	tnc	155691
GL172772.1	974480	975016	974548, 974598, 974559	44, 62, 27	52.03323, 69.27326, 33.99821	19.87468, 21.04551, 16.4039	48.15272,	3	NA	33768
GL172772.1	1056760	1057426	1056984, 1056896, 1057169	11, 13, 5	9.95877, 11.36332, 4.91762	6.54239, 7.01137, 4.03768	7.19682, 8	3	NA	116048
GL172772.1	1150770	1151127	1150987, 1150838, 1150976	6, 9, 5	5.72604, 6.8008, 5.31857	4.51498, 4.90352, 4.24819	3.33547, 4	3	pappa	35422
GL172772.1	1220305	1221005	1220454, 1220457, 1220513	9, 12, 7	8.50607, 9.13354, 8.15288	5.95453, 5.83804, 5.81587	5.84194, 6	3	pappa	0
GL172772.1	1280526	1280971	1280782, 1280781, 1280736	11, 16, 16	11.71035, 15.28831, 19.65507	7.57196, 8.69939, 11.21774	8.8442, 12	3	pappa	0
GL172772.1	1416796	1417171	1416922, 1416900, 1416969	22, 24, 8	23.10245, 22.26844, 8.62637	11.66453, 10.50366, 6.08668	19.77471,	3	astn2	0
GL172772.1	1613230	1614521	1613976, 1613579, 1613942	18, 28, 12	12.14277, 17.80793, 8.99931	6.3073, 6.98436, 5.75428	9.25603, 1	3	astn2	0
GL172772.1	1633436	1633791	1633498, 1633466, 1633501	5, 7, 5	4.82177, 5.97725, 5.75061	3.98509, 4.62287, 4.45735	2.51418, 3	3	astn2	0
GL172772.1	1809074	1809618	1809360, 1809286, 1809405	12, 20, 3	10.1196, 19.37003, 3.44998	6.45229, 10.0456, 3.02115	7.349, 16.3	3	astn2	94030
GL172772.1	2066681	2067449	2066903, 2067189, 2067223	9, 16, 10	6.82879, 12.25152, 9.26679	4.92128, 6.84834, 6.28179	4.31954, 9	3	5S_rRNA	27521
GL172772.1	2071759	2072090	2071784, 2071802, 2071804	25, 17, 11	25.64823, 14.61162, 11.6121	12.17691, 8.02205, 7.51665	22.24852,	3	5S_rRNA	22880
GL172772.1	2088897	2090081	2089549, 2089447, 2089700	19, 19, 8	21.11935, 16.5695, 8.09568	11.38438, 8.65063, 5.79794	17.85404,	3	5S_rRNA	4889
GL172772.1	2146002	2146630	2146168, 2146356, 2146448	8, 18, 5	6.70593, 16.94269, 5.10931	4.9768, 9.16356, 4.14026	4.20487, 1	3	brinp1	33396
GL172772.1	2185959	2186785	2186255, 2186267, 2186409	11, 23, 14	12.01255, 22.59564, 15.57248	7.73996, 11.03113, 9.30992	9.13199, 1	3	brinp1	0
GL172772.1	2298541	2298755	2298641, 2298591, 2298655	6, 10, 4	5.96715, 7.97488, 4.67325	4.64927, 5.48553, 3.78987	3.52158, 5	3	brinp1	100045
GL172772.1	2343362	2344146	2343747, 2343767, 2343800	6, 14, 5	5.63898, 14.44087, 5.9506	4.46544, 8.65722, 4.54784	3.25772, 1	3	brinp1	144866
GL172773.1	15337	15729	15483, 15469, 15629	13, 25, 9	12.68462, 22.84169, 9.14171	7.81602, 10.50999, 6.32219	9.76298, 1	3	garnl3	0
GL172773.1	36575	37388	37103, 37234, 37250	9, 10, 5	8.55802, 8.01217, 5.40789	5.98518, 5.50894, 4.29296	5.8909, 5.5	3	slc2a8	2642
GL172773.1	51615	52438	52038, 52373, 51723	15, 17, 6	17.48779, 18.53926, 7.29403	10.20672, 10.38866, 5.30581	14.34959,	3	slc2a8	134
GL172773.1	69030	69951	69131, 69344, 69198	7, 14, 7	6.2098, 11.62806, 8.15288	4.76362, 6.94888, 5.81587	3.74679, 8	3	fbxw2	0
GL172773.1	120115	120421	120250, 120302, 120335	5, 8, 6	4.82177, 7.07159, 7.29403	3.98509, 5.20073, 5.30581	2.51418, 4	3	c5	1186
GL172773.1	153562	154156	153577, 153705, 153608	12, 15, 4	12.82537, 14.39783, 4.50795	8.07156, 8.40758, 3.71446	9.89694, 1	3	TRAF1	9420
GL172773.1	165190	165542	165330, 165357, 165367	7, 23, 11	5.79309, 20.88864, 12.86886	4.5096, 10.01184, 8.19722	3.39779, 1	3	phf19	3525
GL172773.1	175659	176125	175760, 175775, 175778	29, 42, 27	27.04179, 37.30737, 27.20923	11.57861, 12.57503, 12.39384	23.60537,	3	phf19	0
GL172773.1	176706	177031	176901, 176917, 176929	17, 15, 14	12.7525, 8.31506, 11.02224	6.90135, 4.76213, 6.57526	9.82709, 5	3	phf19	0
GL172773.1	283996	285153	284646, 284583, 284490	6, 7, 7	5.47306, 5.97725, 6.83761	4.36955, 4.62287, 5.12939	3.10811, 3	3	vav2	0

GL172773.1	294341	295223	294754, 294967, 294959	12, 26, 7	11.73328, 23.97783, 7.96278	7.43625, 10.83957, 5.72394	8.86615, 2	3	vav2	0
GL172773.1	305802	306988	306109, 305880, 306405	7, 14, 8	6.69449, 11.32527, 8.1956	5.04797, 6.76206, 5.85348	4.1953, 8.6	3	vav2	0
GL172773.1	372181	372579	372407, 372416, 372410	47, 64, 35	50.92674, 70.26711, 40.68607	17.87321, 20.65552, 17.22635	47.06179,	3	vav2	0
GL172773.1	378933	379397	379184, 378963, 378946	10, 9, 6	7.01158, 4.96916, 4.99541	4.87678, 3.73371, 4.08336	4.48836, 2	3	vav2	0
GL172773.1	470041	470757	470567, 470512, 470510	18, 29, 13	21.07666, 30.99206, 17.28449	11.6109, 13.89775, 10.28792	17.81304,	3	wdr5	0
GL172773.1	497441	498140	497776, 497785, 497820	10, 18, 13	7.64746, 14.40368, 14.61978	5.27935, 7.62784, 8.93426	5.05779, 1	3	U6atac	8267
GL172773.1	525986	527016	526776, 526704, 526788	9, 16, 6	9.72082, 14.86511, 7.29403	6.64182, 8.44177, 5.30581	6.97155, 1	3	U6atac	36812
GL172773.1	546652	547229	546671, 546737, 546664	12, 12, 9	10.93921, 9.35334, 10.49552	6.95711, 5.9752, 7.0436	8.12344, 6	3	U6atac	57478
GL172773.1	579049	579523	579235, 579329, 579378	17, 40, 24	14.4435, 38.3773, 24.50282	7.91979, 13.854, 11.83761	11.43583,	3	U6atac	89875
GL172773.1	583931	584373	584239, 584268, 584222	12, 19, 6	10.34679, 15.91125, 5.83216	6.59299, 8.25518, 4.57461	7.56382, 1	3	U6atac	94757
GL172773.1	597025	597616	597359, 597290, 597583	5, 6, 6	4.61714, 4.93312, 7.29403	3.86998, 4.04501, 5.30581	2.39372, 2	3	U6atac	107851
GL172773.1	607621	607941	607825, 607781, 607881	5, 8, 8	4.82177, 7.07159, 10.1465	3.98509, 5.20073, 6.82176	2.51418, 4	3	U6atac	118447
GL172773.1	679886	680631	680153, 680461, 680358	15, 8, 9	16.28407, 7.07159, 11.58206	9.52725, 5.20073, 7.55286	13.19656,	3	rxra	49620
GL172773.1	691561	692005	691883, 691893, 691912	21, 36, 19	13.38212, 21.93914, 14.43973	6.31209, 7.15052, 7.38452	10.42795,	3	rxra	38246
GL172773.1	694279	694837	694469, 694508, 694452	50, 62, 34	47.15689, 51.15997, 32.16461	14.66876, 12.63278, 12.68155	43.34619,	3	rxra	35414
GL172773.1	775884	776396	775941, 775886, 776024	22, 25, 4	23.53664, 22.78017, 4.08034	11.92457, 10.47392, 3.50363	20.19714,	3	rxra	9678
GL172773.1	832169	832537	832241, 832184, 832195	5, 11, 7	4.77263, 10.42613, 8.15288	3.9578, 6.8251, 5.81587	2.51418, 7	3	rxra	65963
GL172773.1	870953	871212	871135, 871119, 871130	10, 13, 10	9.33595, 12.67661, 11.5283	6.32332, 7.81122, 7.55211	6.62299, 9	3	col5a1	75113
GL172773.1	881759	882498	882045, 882151, 882179	6, 10, 5	5.90927, 9.39128, 5.40789	4.61743, 6.35644, 4.29296	3.50164, 6	3	col5a1	63827
GL172773.1	914065	914775	914098, 914081, 914096	22, 31, 17	25.40151, 33.37786, 22.90084	13.02826, 14.5374, 12.74458	22.00933,	3	col5a1	31550
GL172773.1	948872	949366	948916, 948900, 949121	7, 18, 15	6.89193, 17.58997, 18.95532	5.15998, 9.55621, 10.98488	4.37772, 1	3	col5a1	0
GL172773.1	962783	963137	963104, 963108, 963093	32, 34, 23	37.88202, 33.22479, 28.93723	16.80937, 13.27919, 14.76095	34.2142, 2	3	col5a1	0
GL172773.1	989415	990105	989467, 989491, 989478	17, 28, 16	18.91905, 29.6677, 19.96942	10.6095, 13.48773, 11.38369	15.72695,	3	col5a1	0
GL172773.1	1077432	1077924	1077898, 1077923, 1077870	18, 27, 10	18.07742, 26.80764, 9.82971	9.85073, 12.15568, 6.61563	14.9177, 2	3	col5a1	0
GL172773.1	1106051	1107131	1106718, 1106718, 1106733	16, 26, 14	15.74146, 23.18389, 13.93205	8.97386, 10.37813, 8.35526	12.67506,	3	col5a1	17858
GL172773.1	1299895	1300825	1300387, 1300417, 1300108	11, 11, 9	9.26086, 8.13814, 10.64485	6.1125, 5.40965, 7.11743	6.5517, 5.6	3	olfm1	10972
GL172773.1	1305026	1305372	1305231, 1305286, 1305207	11, 13, 7	10.3799, 10.43179, 6.6707	6.79736, 6.43483, 5.03432	7.59574, 7	3	olfm1	16103
GL172773.1	1329715	1330295	1330068, 1330020, 1329978	6, 11, 4	5.96715, 9.57292, 4.67325	4.64927, 6.30569, 3.78987	3.52158, 6	3	olfm1	40792
GL172773.1	1421595	1422258	1421893, 1421895, 1421930	10, 27, 6	10.53228, 24.42739, 5.83216	7.01712, 10.7625, 4.57461	7.73927, 2	3	ppp1r26	118864
GL172773.1	1453715	1454171	1453821, 1453762, 1453900	4, 7, 5	3.34243, 5.97725, 5.75061	3.08796, 4.62287, 4.45735	1.41807, 3	3	ppp1r26	86951
GL172773.1	1491905	1492222	1492109, 1492095, 1492106	18, 31, 14	17.2505, 32.82022, 16.46576	9.35044, 14.20748, 9.80275	14.12166,	3	ppp1r26	48900
GL172773.1	1515449	1515930	1515869, 1515845, 1515868	19, 23, 11	23.80016, 27.36982, 14.24187	12.89994, 13.8686, 8.86619	20.45294,	3	ppp1r26	25192
GL172773.1	1561695	1562081	1561949, 1561997, 1562003	5, 10, 8	4.27048, 8.61706, 8.9249	3.6666, 5.8856, 6.24211	2.09564, 6	3	mrps2	1046
GL172773.1	1572120	1572943	1572761, 1572500, 1572493	20, 28, 15	23.18601, 28.3943, 16.52447	12.31532, 12.73107, 9.66554	19.85599,	3	fam69b	0
GL172773.1	1580942	1581397	1581203, 1581095, 1580987	10, 14, 4	10.39648, 13.8891, 4.46488	6.94096, 8.32957, 3.69425	7.61003, 1	3	fam69b	0
GL172773.1	1611045	1611735	1611423, 1611683, 1611288	11, 11, 7	11.93519, 9.8562, 8.21885	7.69727, 6.47973, 5.84717	9.0586, 7.2	3	fam69b	0
GL172773.1	1638787	1639558	1639472, 1639515, 1639525	25, 25, 11	24.26941, 22.84169, 11.6739	11.35377, 10.50999, 7.55148	20.90825,	3	fam69b	13857
GL172773.1	1640615	1641242	1641042, 1641059, 1640804	10, 18, 10	7.44561, 14.99509, 10.42037	5.15177, 7.98278, 6.95441	4.86925, 1	3	agpat2	14674
GL172773.1	1725618	1725877	1725651, 1725625, 1725670	7, 13, 8	6.46447, 10.81092, 9.31951	4.91463, 6.66988, 6.43944	3.98312, 8	3	egfl7	22120
GL172773.1	1728195	1729118	1728544, 1728546, 1728802	7, 16, 4	5.39768, 13.17297, 3.70359	4.26169, 7.40763, 3.29934	3.04019, 1	3	egfl7	24697
GL172773.1	1730423	1730978	1730512, 1730718, 1730704	8, 12, 6	6.14488, 8.68993, 5.8719	4.62489, 5.56143, 4.59674	3.6868, 6.1	3	egfl7	26925
GL172773.1	1767714	1768299	1767938, 1768171, 1767889	5, 8, 7	3.86321, 6.81727, 8.21885	3.41531, 5.04548, 5.84717	1.73293, 4	3	egfl7	64216
GL172773.1	1786403	1786627	1786614, 1786564, 1786583	11, 10, 6	10.16451, 8.88645, 7.29403	6.66744, 6.0511, 5.30581	7.39114, 6	3	egfl7	82905
GL172773.1	1803312	1803668	1803490, 1803495, 1803512	11, 13, 13	11.56615, 12.61502, 15.70541	7.49066, 7.77427, 9.51556	8.70729, 9	3	notch1	67303
GL172773.1	1842023	1842562	1842079, 1842146, 1842083	20, 19, 15	21.26668, 16.25859, 18.53291	11.18692, 8.46351, 10.76715	17.9967, 1	3	notch1	28409
GL172773.1	1848385	1848961	1848461, 1848402, 1848433	7, 11, 6	7.1696, 9.39347, 7.18591	5.31345, 6.19476, 5.25776	4.61046, 6	3	notch1	22010
GL172773.1	1861453	1861842	1861479, 1861480, 1861457	27, 31, 31	30.23936, 35.32126, 41.11717	14.20029, 15.69126, 19.0765	26.7262, 3	3	notch1	9129
GL172773.1	1961170	1962304	1961672, 1961514, 1961754	8, 14, 8	6.70593, 11.80872, 7.5499	4.9768, 7.06033, 5.48567	4.20487, 9	3	sec16a	0
GL172773.1	1985792	1986877	1986342, 1986203, 1986522	8, 8, 3	6.70593, 6.24338, 3.44998	4.9768, 4.6873, 3.02115	4.20487, 3	3	snapc4	925
GL172773.1	2015223	2016073	2015569, 2015562, 2015722	33, 59, 24	34.51596, 59.93009, 26.72921	14.41419, 17.54655, 13.16922	30.91209,	3	card9	6403
GL172773.1	2025805	2026419	2025846, 2026342, 2026142	4, 7, 6	3.51298, 5.97725, 6.35308	3.1896, 4.62287, 4.85498	1.55088, 3	3	card9	0
GL172773.1	2055554	2056030	2055598, 2055609, 2055597	28, 46, 31	24.83772, 43.97227, 33.4708	10.66183, 14.54185, 14.59246	21.46092,	3	gpsm1	0
GL172773.1	2056641	2057148	2056683, 2056770, 2056693	23, 27, 15	18.75967, 20.44304, 12.19525	8.76814, 8.55054, 7.05919	15.57324,	3	gpsm1	0
GL172773.1	2118574	2119287	2118918, 2118696, 2118897	5, 8, 4	4.50727, 6.11443, 4.01199	3.80664, 4.60556, 3.46784	2.30125, 3	3	qsox2	36276
GL172773.1	2156126	2156754	2156143, 2156130, 2156188	13, 21, 14	12.81131, 20.32736, 15.80333	7.8918, 10.3138, 9.43935	9.88323, 1	3	qsox2	0
GL172773.1	2169885	2170297	2170201, 2170166, 2170042	10, 12, 8	9.661, 10.83413, 9.60885	6.51661, 6.89288, 6.57807	6.9283, 8.1	3	qsox2	0
GL172773.1	2176630	2177027	2176642, 2176657, 2176666	11, 32, 23	11.22377, 35.41893, 28.13384	7.29486, 15.3481, 14.30737	8.38272, 3	3	qsox2	0
GL172773.1	2208914	2209492	2209256, 2209294, 2209273	14, 15, 12	16.7649, 12.86621, 15.26371	9.96273, 7.47111, 9.35067	13.6543, 1	3	lhx3	0
GL172773.1	2231387	2232554	2231698, 2231457, 2232257	13, 14, 14	13.34951, 12.92044, 16.55387	8.21021, 7.74345, 9.85015	10.39596,	3	lhx3	14327
GL172773.1	2310478	2311272	2310718, 2310739, 2310709	15, 25, 12	15.71867, 24.81034, 14.76955	9.19755, 11.67607, 9.10943	12.65377,	3	TMEM250	9442
GL172773.1	2321689	2322491	2322029, 2322124, 2322124	6, 13, 5	5.68215, 13.14514, 5.9506	4.49007, 8.09002, 4.54784	3.29732, 1	3	TMEM250	560
GL172773.1	2325433	2325900	2325635, 2325840, 2325819	11, 10, 5	12.09119, 8.04981, 5.85847	7.78314, 5.53254, 4.50665	9.20684, 5	3	TMEM250	4304
GL172773.1	2344541	2345030	2344676, 2344730, 2344764	7, 6, 4	6.42049, 4.6513, 4.30163	4.8888, 3.86883, 3.61556	3.94161, 2	3	TMEM250	23412
GL172773.1	2428897	2429369	2429159, 2429147, 2428921	8, 16, 6	7.1122, 14.52284, 5.83216	5.22529, 8.2328, 4.57461	4.57946, 1	3	nacc2	27856
GL172774.1	681317	682626	681798, 681783, 681852	15, 22, 16	13.8583, 19.65261, 16.972	8.07921, 9.59468, 9.70822	10.87599,	3	frmd4a	16832
GL172774.1	1031129	1032031	1031577, 1031561, 1031386	13, 21, 10	12.38021, 19.73353, 9.93124	7.63279, 9.95593, 6.67475	9.48056, 1	3	dmtf1	0
GL172775.1	4249	5046	4835, 4744, 4931	6, 13, 10	5.27951, 10.68145, 11.5283	4.25534, 6.58965, 7.55211	2.9335, 8.0	3	NA	9642
GL172775.1	32643	33791	33426, 33389, 33381	16, 20, 18	16.30427, 19.10348, 22.31958	9.31205, 9.88447, 12.29844	13.21554,	3	NA	0
GL172775.1	675542	675954	675649, 675650, 675657	9, 15, 5	9.43487, 13.11311, 5.40789	6.48595, 7.62272, 4.29296	6.71665, 1	3	arhgap26	0
GL172775.1	1472798	1473279	1473077, 1473083, 1473003	4, 6, 4	3.74195, 4.20515, 4.67325	3.32091, 3.5821, 3.78987	1.6222, 2.1	3	KCTD16	189806
GL172775.1	1555959	1556446	1556378, 1556417, 1556388	17, 26, 15	18.29581, 26.71306, 19.17716	10.24594, 12.45881, 11.09709	15.12565,	3	NA	222826
GL172775.1	1737627	1738307	1737876, 1737940, 1737952	33, 59, 26	36.33892, 65.01077, 30.9509	15.48701, 20.15808, 14.97804	32.69931,	3	NA	40965
GL172775.1	1838206	1838789	1838353, 1838353, 1838633	10, 10, 6	9.77513, 9.27513, 7.18591	6.5837, 6.2868, 5.25776	7.02302, 6	3	NA	47645
GL172775.1	1892731	1893150	1892759, 1892735, 1892779	36, 51, 43	42.55662, 60.4193, 54.68505	17.90563, 21.38827, 21.90339	38.81344,	3	prelid2	44510
GL172776.1	688446	688761	688636, 688707, 688654	13, 12, 6	12.62236, 9.86753, 6.08001	7.77868, 6.29567, 4.71063	9.70369, 7	3	ctnnal1	39654
GL172776.1	753151	753727	753348, 753426, 753204	8, 17, 8	6.94372, 13.42125, 8.04677	5.12297, 7.30129, 5.77057	4.42528, 1	3	ctnnal1	0
GL172776.1	1624870	1625839	1625445, 1625198, 1625668	8, 16, 10	6.86256, 12.36948, 11.60305	5.0733, 6.91958, 7.59048	4.3506, 9.6	3	nfx1	0
GL172776.1	2322105	2323074	2322453, 2322480, 2322491	20, 23, 20	19.22096, 20.82882, 23.93369	9.95549, 9.97638, 12.7427	16.01831,	3	nfx1	593803
GL172777.1	966917	967347	967156, 967132, 967046	4, 7, 3	3.74195, 5.8586, 3.47371	3.32091, 4.55007, 3.03189	1.6222, 3.5	3	NA	83876
GL172777.1	1819984	1820450	1820378, 1820091, 1820098	13, 20, 12	11.45737, 17.7751, 12.88563	7.06931, 9.08317, 8.10565	8.60415, 1	3	glt1d1	0
GL172778.1	351926	352684	352657, 352656, 352616	54, 71, 35	62.44098, 83.85852, 44.26441	21.08747, 24.46985, 19.33874	58.42654,	3	kif5c	23869
GL172778.1	368785	369158	368855, 368804, 368821	16, 9, 7	15.87778, 6.86591, 6.59055	9.05608, 4.94483, 4.98809	12.8057, 4	3	LYPD6B	7414
GL172778.1	402513	403219	403053, 402613, 403096	13, 8, 14	11.92615, 5.67261, 15.72539	7.35684, 4.32287, 9.39581	9.04988, 3	3	LYPD6B	22157
GL172778.1	425482	425912	425760, 425719, 425812	23, 19, 8	25.23251, 19.48976, 10.00633	12.61172, 10.41622, 6.75998	21.84553,	3	NA	17106
GL172778.1	458735	459931	459100, 458958, 459434	9, 10, 6	8.55802, 6.60892, 5.83216	5.98518, 4.62161, 4.57461	5.8909, 4.2	3	NA	0
GL172778.1	670624	670929	670806, 670702, 670744	7, 6, 6	6.33443, 4.83043, 7.29403	4.83795, 3.98131, 5.30581	3.86259, 2	3	ift22	47440
GL172778.1	1103895	1104606	1104254, 1104471, 1104215	10, 9, 10	10.26482, 7.71322, 10.71812	6.86644, 5.47534, 7.12015	7.48624, 5	3	tnfaip1	0
GL172779.1	240288	240854	240821, 240820, 240780	15, 12, 5	16.20017, 10.93692, 5.59755	9.47871, 6.95571, 4.38538	13.11636,	3	tacr1	0
GL172779.1	1022571	1024253	1023206, 1023122, 1023529	6, 13, 7	5.81608, 12.67661, 8.69489	4.56563, 7.81122, 6.06379	3.4176, 9.9	3	aak1	1495
GL172779.1	1430877	1431938	1431430, 1431472, 1431451	12, 14, 8	12.38809, 14.45897, 9.05167	7.82099, 8.66788, 6.30653	9.48755, 1	3	arhgap25	0
GL172779.1	1526587	1528029	1526886, 1527163, 1526979, 1527584	10, 20, 6, 6	7.41295, 16.30367, 6.30561, 6.03707	5.1311, 8.20624, 4.83031, 4.6874	4.8384, 13	3	slc23a2	0
GL172779.1	1713570	1714034	1713721, 1713850, 1713725	8, 11, 4	7.15567, 9.09572, 4.34116	5.25151, 6.00975, 3.63492	4.61046, 6	3	prnp	80428
GL172779.1	2196920	2197651	2197332, 2197367, 2197356	60, 85, 36	62.19184, 88.85722, 38.09132	18.26797, 20.71224, 15.28176	58.18019,	3	NA	259660
GL172779.1	2261461	2261858	2261831, 2261856, 2261809	16, 32, 17	17.68339, 35.41893, 20.18522	10.1227, 15.3481, 11.32722	14.5382, 3	3	NA	324201
GL172779.1	2263955	2264365	2264074, 2264117, 2264070	5, 8, 4	4.61714, 5.78452, 3.59344	3.86998, 4.39481, 3.23643	2.39372, 3	3	NA	326695
GL172780.1	10738	11647	11040, 11158, 11179	8, 9, 5	8.42257, 8.21099, 5.9506	5.97763, 5.77858, 4.54784	5.76336, 5	3	NA	55873
GL172780.1	16118	18175	16839, 16951, 16916, 17888	12, 20, 15, 7	9.65435, 17.13302, 15.11547, 5.50849	6.16292, 8.69853, 8.83977, 4.33177	6.92291, 1	3	NA	49345
GL172780.1	35648	36808	36176, 36179, 36296	33, 44, 17	33.23109, 41.62011, 18.35257	13.66677, 14.0714, 10.27929	29.6531, 3	3	NA	30712
GL172780.1	620721	621148	620823, 620808, 620958	11, 9, 6	11.15818, 7.06866, 6.71074	7.25692, 5.07306, 5.03498	8.32008, 4	3	PREX2	0
GL172780.1	853328	853920	853684, 853500, 853684	10, 10, 9	9.49535, 7.05193, 9.14171	6.41851, 4.90236, 6.32219	6.77344, 4	3	c8orf34	5855
GL172780.1	1043662	1044042	1043874, 1043664, 1043977	10, 8, 7	11.06011, 6.89656, 7.50542	7.306, 5.09414, 5.49244	8.22712, 4	3	sulf1	58271
GL172780.1	1600924	1601259	1601120, 1601173, 1600995	9, 6, 4	9.56822, 4.93312, 4.55201	6.55912, 4.04501, 3.73489	6.84121, 2	3	nudt4	16607
GL172780.1	1787388	1788441	1788118, 1787820, 1787981	43, 85, 39	41.91548, 86.14491, 44.3432	14.60639, 19.54904, 17.64797	38.18264,	3	socs2	0
GL172781.1	6364	7006	6722, 6963, 6956	7, 9, 7	4.46548, 5.70245, 5.62023	3.65833, 4.20146, 4.40197	2.26751, 3	3	aven	84639
GL172781.1	304124	304659	304236, 304220, 304226	12, 29, 13	12.67544, 27.91762, 16.0859	7.98627, 12.0853, 9.70995	9.75458, 2	3	gpr176	0
GL172781.1	674981	675736	675120, 675593, 675005	7, 8, 5	6.33443, 5.87115, 5.50079	4.83795, 4.45035, 4.33868	3.86259, 3	3	XB-GENE-5954653	4361
GL172781.1	1772675	1773742	1772700, 1772723, 1772702	33, 41, 14	39.95265, 48.43915, 19.68329	17.62762, 19.15447, 11.3696	36.25077,	3	nkx2-1	32084
GL172781.1	2263989	2264607	2264082, 2264044, 2264129	16, 13, 8	16.60441, 10.7674, 7.85773	9.49088, 6.64292, 5.66361	13.50404,	3	chst9	23946
GL172782.1	123015	123245	123061, 123049, 123059	9, 10, 5	9.12009, 8.79469, 5.64747	6.30997, 5.99491, 4.40911	6.42028, 6	3	pc.1	0
GL172782.1	411906	412485	411990, 411917, 411933	16, 20, 17	18.05293, 22.54677, 23.32218	10.33439, 11.94392, 12.94709	14.8942, 1	3	pacs1	10475
GL172782.1	2303346	2303900	2303705, 2303605, 2303641	26, 31, 33	28.48278, 34.06043, 40.08222	13.51628, 14.94221, 17.70411	25.01069,	3	irx3	112680
GL172783.1	174882	175517	175314, 175263, 175093	9, 16, 4	7.28297, 14.98328, 4.67325	5.20782, 8.51381, 3.78987	4.7171, 12	3	NA	20906
GL172783.1	494737	495468	495335, 495136, 494947	9, 16, 9	9.36986, 15.60807, 9.08997	6.44997, 8.89325, 6.2929	6.65425, 1	3	nrxn1	21463
GL172783.1	604083	604464	604378, 604437, 604439	15, 27, 16	16.45523, 30.66659, 20.763	9.62583, 14.45444, 11.79066	13.36057,	3	nrxn1	0
GL172783.1	1226031	1226948	1226852, 1226725, 1226472	9, 9, 6	8.16099, 7.24675, 6.87953	5.74847, 5.1851, 5.11627	5.53692, 4	3	fshr	161092
GL172783.1	1427763	1428221	1428163, 1428146, 1428181	7, 11, 7	7.1696, 10.60895, 8.69489	5.31345, 6.9343, 6.06379	4.61046, 7	3	fshr	13269
GL172783.1	1676997	1677965	1677494, 1677540, 1677618	16, 23, 16	16.83862, 22.88804, 20.763	9.62959, 11.20666, 11.79066	13.72526,	3	gtf2a1l	0
GL172783.1	1811305	1811605	1811548, 1811557, 1811553	22, 29, 13	21.69032, 29.93329, 14.69628	10.81544, 13.26932, 8.97644	18.40561,	3	ppp1r21	0
GL172783.1	1977712	1978998	1978503, 1977744, 1978463, 1978211, 1978806	12, 8, 8, 6, 6	11.09909, 6.21066, 6.14619, 5.60548, 5.67889	7.05451, 4.66659, 4.62573, 4.44624, 4.48822	8.26391, 3	3	NA	12072
GL172783.1	2131853	2132292	2132259, 2132239, 2132265	36, 63, 36	41.58106, 75.2922, 48.36757	17.32868, 23.82, 21.30887	37.85339,	3	kcnk12	0
GL172783.1	2284440	2285437	2284469, 2284465, 2284489	30, 31, 27	30.44049, 31.77267, 32.93628	13.19044, 13.59061, 15.7908	26.92349,	3	epcam	9924
GL172784.1	144	902	373, 398, 401	13, 16, 11	14.79381, 17.16856, 14.03502	9.02996, 9.82359, 8.77076	11.77132,	3	NA	65839
GL172784.1	6698	8031	7887, 7695, 7554	14, 13, 10	14.38232, 12.14594, 11.38247	8.62271, 7.49077, 7.47651	11.37721,	3	NA	58710
GL172784.1	13987	15216	14273, 14283, 14923	7, 10, 7	4.35818, 4.76848, 5.37469	3.58779, 3.47672, 4.2471	2.17369, 2	3	NA	51525
GL172784.1	18196	18731	18292, 18274, 18259	13, 22, 13	7.66098, 11.99891, 10.41976	4.74226, 5.41706, 6.42737	5.0711, 9.2	3	NA	48010
GL172784.1	39495	40253	39837, 40102, 39828	9, 9, 9	8.40425, 6.73685, 10.96118	5.89416, 4.86289, 7.26984	5.76336, 4	3	NA	26488
GL172784.1	50353	51311	51019, 51028, 50772	78, 128, 64	78.96114, 135.80458, 70.60155	19.0473, 23.60863, 20.82843	74.77457,	3	NA	15430
GL172784.1	489343	489808	489691, 489700, 489677	18, 13, 5	19.69649, 11.81904, 5.3628	10.81516, 7.29135, 4.27046	16.47841,	3	abca10	0
GL172784.1	512614	512989	512979, 512971, 512947	33, 46, 28	37.97073, 51.01127, 33.82944	16.45382, 18.36634, 15.95716	34.30122,	3	abca10	0
GL172784.1	517826	518330	517988, 518127, 518114	6, 11, 12	5.96715, 9.17879, 13.59027	4.64927, 6.06147, 8.49571	3.52158, 6	3	abca10	0
GL172784.1	713060	713556	713070, 713300, 713250	4, 10, 3	3.74195, 9.39128, 3.47371	3.32091, 6.35644, 3.03189	1.6222, 6.8	3	mc3r	28919
GL172784.1	846378	847327	846650, 847034, 846944	17, 15, 9	17.80138, 14.7819, 11.64359	9.95359, 8.63964, 7.57973	14.65165,	3	cbln4	21391
GL172784.1	1015397	1016462	1015465, 1015454, 1016129	6, 5, 6	5.96715, 3.78182, 6.87953	4.64927, 3.36365, 5.11627	3.52158, 1	3	cbln4	136549
GL172784.1	1148853	1149986	1149172, 1149170, 1149066	8, 20, 6	7.99469, 21.34919, 7.25145	5.74128, 11.23615, 5.287	5.38155, 1	3	dok5	162182
GL172784.1	1298855	1299369	1298885, 1298862, 1298881	44, 72, 45	54.66867, 86.39674, 58.42674	21.42052, 25.32294, 23.15362	50.75022,	3	dok5	12799
GL172784.1	1548745	1549199	1548927, 1548889, 1548854	8, 8, 4	8.42257, 7.07159, 4.67325	5.97763, 5.20073, 3.78987	5.76336, 4	3	pfdn4	86318
GL172784.1	1587013	1587425	1587065, 1587050, 1587022	35, 24, 34	39.7271, 22.6569, 45.74091	16.65904, 10.73418, 20.59371	36.02906,	3	pfdn4	48092
GL172784.1	1859920	1860719	1860177, 1860195, 1860196	32, 39, 24	37.39359, 42.49015, 28.61177	16.5199, 16.56863, 14.27679	33.73399,	3	znf217	6422
GL172784.1	1904070	1904515	1904212, 1904213, 1904085	6, 6, 6	4.9641, 4.35338, 6.45058	4.06411, 3.67832, 4.90508	2.64509, 2	3	tshz2	22171
GL172784.1	1956275	1956933	1956704, 1956704, 1956704	39, 60, 33	46.97602, 73.03439, 42.73283	19.19764, 24.07764, 19.2545	43.16784,	3	tshz2	25633
GL172784.1	2060571	2061130	2060696, 2060695, 2060907	11, 16, 6	11.42621, 15.28831, 6.08001	7.41109, 8.69939, 4.71063	8.57514, 1	3	tshz2	129929
GL172784.1	2093969	2094374	2094277, 2094070, 2094094	9, 15, 11	7.39636, 12.44573, 11.14477	5.27874, 7.21287, 7.24915	4.82212, 9	3	tshz2	163327
GL172785.1	131485	131826	131768, 131771, 131768	10, 22, 17	9.95227, 21.42357, 18.06396	6.68696, 10.65494, 10.10925	7.19087, 1	3	rasal3	16495
GL172785.1	371015	372302	371858, 371433, 371396	20, 27, 14	22.97335, 29.25974, 17.01463	12.19234, 13.61609, 10.0942	19.64963,	3	NA	17114
GL172785.1	520222	520554	520495, 520493, 520500	18, 23, 17	16.62541, 22.38211, 20.98465	8.97077, 10.90305, 11.76329	13.52454,	3	NA	5914
GL172785.1	695349	695997	695960, 695948, 695902	24, 43, 20	29.5382, 55.23209, 25.96589	14.81049, 22.22539, 13.85721	26.04115,	3	fcer2	10106
GL172785.1	729775	730223	729919, 729956, 729882	36, 35, 18	46.89847, 45.71076, 26.51834	20.46016, 20.18193, 14.35044	43.09162,	3	fcer2	44532
GL172785.1	907262	907703	907396, 907444, 907510	7, 10, 4	6.94335, 9.39128, 4.67325	5.18876, 6.35644, 3.78987	4.42528, 6	3	or10a7	48536
GL172785.1	1085934	1086332	1086164, 1086164, 1086138	34, 48, 27	38.23524, 54.56898, 33.99821	16.19629, 19.47201, 16.4039	34.56211,	3	U4	34006
GL172785.1	1252628	1253160	1252871, 1252675, 1253118, 1252755	7, 7, 9, 4	7.1696, 4.89556, 6.76868, 4.67325	5.31345, 3.93927, 4.88312, 3.78987	4.61046, 2	3	lrrc8e	888
GL172785.1	1260960	1261464	1260987, 1261012, 1261010	34, 54, 27	41.25747, 63.03499, 32.60297	17.9857, 21.42122, 15.59649	37.5352, 5	3	lrrc8e	0
GL172785.1	1306030	1306847	1306377, 1306258, 1306249	8, 14, 5	8.42257, 13.8891, 5.9506	5.97763, 8.32957, 4.54784	5.76336, 1	3	NA	8169
GL172785.1	1554890	1555140	1555112, 1555114, 1555113	95, 139, 91	116.78669, 171.72752, 120.10188	29.35441, 33.30756, 33.24566	112.2622,	3	adgrl1	112384
GL172785.1	1591946	1592730	1592434, 1592411, 1592480	12, 26, 7	10.25464, 25.25783, 6.25413	6.53598, 11.59292, 4.79015	7.47735, 2	3	adgre5	100614
GL172785.1	1959338	1959770	1959550, 1959489, 1959436	5, 8, 6	4.81325, 6.37769, 5.83216	3.98038, 4.77199, 4.57461	2.51418, 4	3	NA	32836
GL172785.1	2025452	2026070	2025615, 2025558, 2025690	9, 16, 10	6.76373, 14.80681, 9.98291	4.87998, 8.40621, 6.70471	4.25982, 1	3	adgre1	6257
GL172785.1	2205534	2205885	2205841, 2205835, 2205560	15, 15, 10	14.65409, 12.49102, 9.26679	8.5626, 7.24068, 6.28179	11.63756,	3	colgalt1	0
GL172785.1	2212368	2212730	2212499, 2212508, 2212499	8, 15, 6	8.22568, 14.03607, 6.35308	5.8701, 8.18766, 4.85498	5.59681, 1	3	colgalt1	0
GL172785.1	2278868	2279125	2279095, 2279087, 2279083	26, 28, 10	25.01044, 25.68606, 9.05276	11.44656, 11.14688, 6.15243	21.62959,	3	gmip	0
GL172785.1	2279588	2280058	2279636, 2279709, 2279694	13, 10, 11	9.55588, 5.89794, 10.14713	5.89229, 4.17346, 6.65691	6.82923, 3	3	gmip	0
GL172785.1	2301663	2302199	2302172, 2302155, 2302117	19, 30, 15	22.46161, 30.72745, 15.95038	12.1581, 13.35925, 9.33339	19.15431,	3	gmip	0
GL172786.1	183362	183586	183425, 183414, 183421	11, 18, 8	11.15818, 16.17802, 8.62637	7.25692, 8.69892, 6.08668	8.32008, 1	3	pcdh15	57077
GL172786.1	193684	193945	193721, 193718, 193724	18, 27, 8	18.22458, 26.88381, 9.1831	9.93936, 12.2008, 6.37229	15.0589, 2	3	pcdh15	46718
GL172786.1	206565	206935	206828, 206755, 206624	6, 11, 7	5.55471, 10.59463, 8.69489	4.41698, 6.92577, 6.06379	3.18192, 7	3	pcdh15	33728
GL172786.1	239183	239623	239498, 239435, 239538	6, 9, 8	5.68215, 8.10603, 9.25066	4.49007, 5.71528, 6.40569	3.29732, 5	3	pcdh15	1040
GL172786.1	322932	323429	323011, 322959, 323025	22, 17, 14	23.7162, 18.36654, 17.01463	12.03186, 10.2875, 10.0942	20.37115,	3	pcdh15	0
GL172786.1	567301	567754	567408, 567351, 567579	6, 7, 6	5.3552, 5.97725, 7.29403	4.3003, 4.62287, 5.30581	3.00253, 3	3	pcdh15	0
GL172786.1	586735	587176	587120, 587126, 587147	6, 10, 9	5.96715, 9.39128, 10.49552	4.64927, 6.35644, 7.0436	3.52158, 6	3	pcdh15	0
GL172786.1	772826	773409	773197, 773105, 773218	5, 7, 4	4.47183, 5.97725, 4.67325	3.78598, 4.62287, 3.78987	2.26935, 3	3	dkk1	73221
GL172786.1	821168	821551	821308, 821308, 821308	13, 17, 13	12.38021, 14.56175, 14.18142	7.63279, 7.99171, 8.68926	9.48056, 1	3	dkk1	25079
GL172786.1	860184	860777	860519, 860775, 860687	11, 18, 8	10.21742, 19.59288, 8.4586	6.69945, 10.7543, 5.99709	7.44132, 1	3	dkk1	8832
GL172786.1	979793	980009	979822, 979834, 979825	41, 47, 32	47.16847, 49.35003, 41.35728	18.40985, 17.00243, 18.84537	43.35748,	3	NA	56310
GL172786.1	1104863	1105217	1104893, 1104992, 1105031	6, 9, 3	5.16999, 8.01512, 3.44998	4.18963, 5.66016, 3.02115	2.83325, 5	3	NA	119149
GL172786.1	1115831	1116456	1116192, 1116200, 1116211	34, 51, 24	34.85817, 52.89878, 26.99032	14.21617, 17.22385, 13.32427	31.2476, 4	3	NA	107910
GL172786.1	1158097	1158410	1158123, 1158137, 1158128	21, 27, 8	22.66679, 25.72383, 8.6841	11.71964, 11.51676, 6.11715	19.35329,	3	NA	65956
GL172786.1	1240026	1241179	1240506, 1240482, 1240552	25, 40, 14	25.40524, 42.37203, 15.96227	12.03153, 16.07522, 9.52765	22.01287,	3	NA	0
GL172786.1	1261501	1261977	1261942, 1261974, 1261935	37, 50, 31	42.62799, 55.00623, 38.2056	17.51484, 18.80271, 17.39643	38.884, 51	3	NA	3806
GL172786.1	1414720	1415062	1414935, 1414745, 1414959	14, 23, 12	13.70063, 20.42056, 14.40488	8.21651, 9.73506, 8.92522	10.73143,	3	sgms1	5518
GL172786.1	1457722	1458472	1457887, 1458006, 1458205	12, 8, 10	11.79509, 6.44699, 11.5283	7.47301, 4.8155, 7.55211	8.92495, 4	3	sgms1	22495
GL172786.1	1463575	1464229	1464006, 1463864, 1463961	22, 18, 11	14.31967, 8.64909, 6.77118	6.58276, 4.44819, 4.55454	11.31755,	3	sgms1	28348
GL172786.1	1466086	1467451	1467108, 1467033, 1466554	31, 47, 29	23.68041, 36.98438, 26.43198	9.11376, 10.89, 11.22943	20.3364, 3	3	sgms1	30859
GL172786.1	1519916	1520514	1519981, 1520035, 1520277	5, 10, 9	3.68584, 8.20397, 8.56228	3.30216, 5.62902, 5.98769	1.6222, 5.7	3	c8orf76	5485
GL172786.1	1573228	1573462	1573295, 1573313, 1573312	16, 18, 15	16.23128, 17.79566, 17.07266	9.26839, 9.68065, 9.97618	13.14614,	3	minpp1	3631
GL172786.1	1589406	1589639	1589515, 1589529, 1589529	31, 36, 11	36.83778, 38.641, 12.19945	16.5907, 15.60125, 7.8422	33.18924,	3	minpp1	19809
GL172786.1	1649904	1650097	1650052, 1650049, 1650070	24, 47, 29	27.83747, 53.40281, 36.84806	13.82444, 19.27034, 17.35105	24.38123,	3	pten	1656
GL172786.1	1710300	1711063	1710648, 1710650, 1710651	56, 89, 66	60.05299, 98.74787, 81.87062	18.86805, 23.5085, 25.93695	56.06395,	3	pten	15950
GL172786.1	1749279	1749687	1749514, 1749514, 1749419	10, 10, 4	8.16254, 6.55702, 3.59344	5.60312, 4.58876, 3.23643	5.53763, 4	3	rnls	25262
GL172786.1	1793468	1794254	1793841, 1793849, 1793849	20, 38, 14	20.44929, 41.13695, 16.37889	10.69687, 16.21162, 9.7558	17.20509,	3	rnls	0
GL172786.1	1854425	1854815	1854621, 1854586, 1854625	7, 10, 9	5.27717, 8.44678, 8.56228	4.185, 5.78021, 5.98769	2.93268, 5	3	ankrd22	3190
GL172786.1	1928646	1929275	1928698, 1928712, 1928726	15, 13, 10	16.90423, 12.86565, 13.18184	9.88146, 7.92422, 8.33771	13.78827,	3	fas	88
GL172786.1	1965757	1966139	1965958, 1965872, 1966106	8, 9, 6	8.42257, 7.21043, 5.91225	5.97763, 5.1623, 4.61907	5.76336, 4	3	CH25H	32495
GL172786.1	1984446	1984990	1984815, 1984851, 1984846	21, 32, 14	20.66146, 34.33575, 13.76252	10.5153, 14.70709, 8.2537	17.41031,	3	CH25H	13644
GL172786.1	2034724	2035764	2035376, 2035099, 2035595	14, 16, 14	14.67861, 13.5443, 15.49745	8.79666, 7.63435, 9.26757	11.66087,	3	NA	0
GL172786.1	2196715	2197673	2197443, 2197433, 2197351	13, 16, 11	12.56081, 15.22617, 12.71187	7.74168, 8.66163, 8.11558	9.64511, 1	3	NA	2669
GL172787.1	693315	694251	694070, 693435, 693595	21, 31, 14	17.51801, 23.67012, 12.42485	8.63427, 9.10853, 7.43979	14.37772,	3	fras1	0
GL172787.1	1316136	1316696	1316523, 1316520, 1316631	12, 19, 7	12.3187, 17.52577, 7.34973	7.78073, 9.22875, 5.41038	9.4231, 14	3	prc1.2	32597
GL172787.1	1330715	1331365	1331205, 1331107, 1330960	13, 31, 10	11.87199, 31.03973, 10.71812	7.32374, 13.16201, 7.12015	8.9979, 27	3	prc1.2	47176
GL172787.1	1491302	1491852	1491649, 1491472, 1491650	19, 24, 16	19.25925, 21.53518, 19.45311	10.27754, 10.07108, 11.10978	16.05534,	3	adamtsl4	45265
GL172787.1	1595579	1596523	1595786, 1596209, 1595761	13, 14, 10	12.62236, 11.2417, 10.1418	7.77868, 6.71052, 6.79622	9.70369, 8	3	mcl1	3751
GL172787.1	1900749	1901520	1901121, 1901371, 1901007	7, 12, 5	7.1696, 11.60251, 5.06966	5.31345, 7.3582, 4.11933	4.61046, 8	3	NA	54685
GL172787.1	1918932	1919930	1919362, 1919406, 1919269	18, 18, 10	17.4488, 15.04268, 10.59656	9.47069, 8.01145, 7.05292	14.31181,	3	NA	36275
GL172787.1	1947166	1948812	1948710, 1948372, 1948620	14, 9, 9	12.38175, 6.20816, 8.74593	7.4133, 4.52542, 6.09519	9.48175, 3	3	NA	7393
GL172787.1	2031696	2032114	2031959, 2032068, 2032080	11, 9, 8	11.93519, 6.9998, 9.3897	7.69727, 5.02958, 6.47354	9.0586, 4.6	3	ANXA9	0
GL172787.1	2066689	2067093	2066768, 2066809, 2066753	14, 19, 11	13.89699, 17.11753, 11.31465	8.3343, 8.98159, 7.3472	10.91278,	3	cers2	14395
GL172788.1	314938	315646	315342, 315292, 315366	22, 33, 10	21.91101, 31.67567, 9.82971	10.94827, 12.7759, 6.61563	18.62037,	3	xtr-mir-365-1	7234
GL172788.1	1766051	1766459	1766174, 1766262, 1766262	12, 18, 14	12.90199, 16.81975, 15.64845	8.11489, 9.08887, 9.35267	9.97033, 1	3	efna5	104770
GL172788.1	2108208	2109037	2108973, 2108963, 2109003	33, 27, 13	38.4404, 25.25239, 14.18142	16.73236, 11.24103, 8.68926	34.76382,	3	efna5	0
GL172789.1	7016	8194	7243, 7417, 7909	7, 7, 9	6.25077, 5.42726, 8.69919	4.78814, 4.28044, 6.06795	3.78441, 3	3	NA	5033
GL172789.1	23889	24388	24366, 24366, 24356	16, 27, 15	14.13236, 25.31851, 15.81457	7.99402, 11.27961, 9.25389	11.1383, 2	3	XB-GENE-5877002	14592
GL172789.1	28991	29926	29051, 29067, 29054, 29688	20, 29, 21, 7	17.52902, 23.41906, 22.44767, 6.01639	8.93546, 9.55919, 11.58891, 4.64674	14.38852,	3	XB-GENE-5877002	9054
GL172789.1	231287	232083	231845, 231989, 231538	9, 8, 4	8.55802, 6.81727, 4.38152	5.98518, 5.04548, 3.65448	5.8909, 4.4	3	NA	488
GL172789.1	242302	242984	242452, 242399, 242383	38, 48, 20	40.30475, 48.69067, 21.20256	15.73097, 16.23016, 11.14863	36.59751,	3	NA	9304
GL172789.1	302180	302813	302611, 302520, 302286	8, 16, 3	8.42257, 17.16856, 3.47371	5.97763, 9.82359, 3.03189	5.76336, 1	3	NA	69182
GL172789.1	409220	409741	409707, 409704, 409704	22, 54, 23	25.73329, 67.02639, 29.81154	13.22149, 23.7019, 15.24424	22.33146,	3	NA	176222
GL172789.1	474489	474755	474572, 474606, 474547	15, 8, 7	17.90819, 6.66413, 8.69489	10.43573, 4.9509, 6.06379	14.75478,	3	NA	111949
GL172789.1	491590	492387	492173, 492098, 492270	13, 24, 18	12.32133, 21.41819, 19.45123	7.59717, 10.00243, 10.67087	9.42482, 1	3	NA	94317
GL172789.1	494354	494915	494470, 494471, 494414	10, 23, 10	9.33595, 21.90191, 9.93124	6.32332, 10.61546, 6.67475	6.62299, 1	3	NA	91789
GL172789.1	506989	507551	507031, 506998, 507038	32, 45, 32	36.8069, 54.04403, 40.95115	16.17179, 20.57893, 18.61058	33.15895,	3	NA	79153
GL172789.1	789236	790091	789676, 789568, 789845	8, 8, 5	8.28589, 7.07159, 5.59755	5.90321, 5.20073, 4.38538	5.65299, 4	3	NA	74113
GL172789.1	868362	868697	868660, 868660, 868570	14, 26, 9	15.49091, 28.39602, 9.82249	9.26387, 13.46449, 6.69629	12.43497,	3	NA	0
GL172789.1	902913	903255	903168, 903220, 903225	7, 13, 7	6.55439, 13.14514, 8.69489	4.96711, 8.09002, 6.06379	4.06616, 1	3	NA	0
GL172789.1	921672	922383	922345, 922348, 922311	23, 33, 11	26.3466, 35.01397, 13.19894	13.27146, 14.70608, 8.36554	22.92763,	3	NA	0

GL172789.1	923338	923693	923611, 923584, 923591	11, 9, 5	9.90875, 6.04841, 5.23257	6.51186, 4.42311, 4.20435	7.14919, 3	3	NA	0
GL172789.1	980052	980491	980431, 980465, 980422	17, 22, 16	19.60089, 25.85271, 23.00461	10.9998, 13.29074, 12.83987	16.38573,	3	NA	4786
GL172789.1	1012859	1013506	1013079, 1013362, 1013408	5, 12, 8	4.40263, 10.68384, 8.56955	3.74533, 6.80072, 6.05652	2.21004, 8	3	NA	37593
GL172789.1	1237310	1238477	1238098, 1237343, 1237484, 1238326	17, 19, 8, 5	15.74197, 16.83862, 8.14528, 4.64121	8.71084, 8.81301, 5.82558, 3.88372	12.67506,	3	lrrtm1	18262
GL172789.1	1239285	1239844	1239800, 1239418, 1239434	15, 15, 13	13.74446, 12.72276, 15.17843	8.00965, 7.383, 9.23814	10.77314,	3	lrrtm1	16895
GL172789.1	1416467	1416746	1416521, 1416499, 1416506	8, 11, 6	6.86256, 10.60895, 6.71074	5.0733, 6.9343, 5.03498	4.3506, 7.9	3	lrrtm1	158161
GL172789.1	1480134	1480891	1480655, 1480178, 1480578	6, 6, 3	5.72604, 4.53871, 3.2112	4.51498, 3.79731, 2.90793	3.33547, 2	3	NA	153938
GL172789.1	1530650	1530960	1530906, 1530939, 1530910	4, 16, 14	3.42565, 15.22617, 15.80333	3.13796, 8.66163, 9.43935	1.48489, 1	3	NA	103869
GL172789.1	1556558	1556971	1556742, 1556907, 1556934	13, 22, 14	12.62236, 23.10829, 16.64329	7.77868, 11.66803, 9.89801	9.70369, 1	3	NA	77858
GL172789.1	1637235	1637807	1637386, 1637587, 1637264	9, 13, 5	9.18105, 12.99541, 5.85847	6.3444, 8.00139, 4.50665	6.47733, 1	3	NA	0
GL172789.1	1740160	1741123	1740436, 1740436, 1740436	20, 15, 17	20.44929, 13.69427, 20.09113	10.69687, 7.97895, 11.27497	17.20509,	3	NA	1786
GL172789.1	1763340	1763889	1763836, 1763881, 1763812	15, 15, 15	15.56593, 13.97777, 18.63599	9.10751, 8.15212, 10.82077	12.50679,	3	NA	24966
GL172789.1	1821486	1822028	1821678, 1821927, 1821744	6, 8, 16	5.1345, 7.01915, 16.63374	4.16817, 5.16892, 9.5083	2.80161, 4	3	NA	83112
GL172789.1	1984920	1985298	1985223, 1985219, 1985236	11, 30, 14	12.43693, 35.79184, 18.52583	7.97018, 16.36294, 10.84669	9.52692, 3	3	NA	39779
GL172789.1	2049455	2050107	2049954, 2049984, 2049984	16, 36, 16	15.94701, 37.00277, 17.85991	9.09776, 14.65375, 10.22415	12.87323,	3	adra1d	80754
GL172789.1	2063174	2063832	2063189, 2063184, 2063219	63, 100, 55	78.23007, 115.07413, 69.64532	25.46164, 26.14901, 24.70716	74.05036,	3	adra1d	67029
GL172789.1	2135562	2135890	2135866, 2135823, 2135837	7, 9, 7	7.1696, 8.21099, 6.71158	5.31345, 5.77858, 5.05776	4.61046, 5	3	adra1d	2655
GL172789.1	2148808	2149468	2149072, 2149068, 2148988	14, 9, 10	14.3105, 8.01512, 11.60305	8.58029, 5.66016, 7.59048	11.30855,	3	adra1d	15901
GL172789.1	2229992	2230493	2230380, 2230450, 2230464	18, 25, 16	19.8791, 28.13462, 19.066	10.9221, 13.66232, 10.89996	16.65356,	3	NA	1299
GL172790.1	1066410	1066694	1066563, 1066523, 1066560	10, 22, 11	8.93828, 22.45914, 12.13051	6.08275, 11.27809, 7.80464	6.24946, 1	3	dusp9	15716
GL172790.1	1464639	1465054	1465010, 1465027, 1464987	26, 16, 17	28.48278, 14.92393, 18.42656	13.51628, 8.47764, 10.3227	25.01069,	3	wnt7c	4451
GL172790.1	1499222	1499768	1499646, 1499327, 1499403	4, 8, 6	3.42565, 7.07159, 6.99807	3.13796, 5.20073, 5.17195	1.48489, 4	3	syp	3557
GL172790.1	1533911	1534610	1533945, 1534399, 1534233	5, 10, 6	3.83692, 7.32562, 6.71074	3.39868, 5.07581, 5.03498	1.70915, 4	3	PRICKLE3	0
GL172790.1	1533911	1534610	1534418, 1534399, 1534233	9, 10, 6	8.16099, 7.32562, 6.71074	5.74847, 5.07581, 5.03498	5.53692, 4	3	PRICKLE3	0
GL172790.1	1696508	1697967	1697670, 1697388, 1697694	21, 27, 19	18.62846, 24.80042, 18.6665	9.29295, 10.9782, 9.91972	15.44705,	3	col27a1	0
GL172790.1	2210511	2211558	2211154, 2211175, 2211155	217, 352, 130	219.25816, 362.18127, 129.31285	23.29279, 25.53988, 20.78759	214.17776	3	apex2	1548
GL172790.1	2223696	2225289	2224182, 2224630, 2224384	9, 27, 7	8.88537, 26.14468, 8.49722	6.17591, 11.7641, 5.97582	6.19961, 2	3	apex2	14733
GL172790.1	2242166	2242783	2242528, 2242568, 2242314	8, 12, 5	5.80896, 6.86826, 4.84526	4.41049, 4.4439, 3.99806	3.41336, 4	3	apex2	33203
GL172790.1	2259172	2261339	2260943, 2259520, 2259343, 2260667	22, 32, 14, 9	16.34185, 22.38781, 10.43465, 5.61083	7.679, 8.21749, 6.21421, 4.14281	13.25202,	3	apex2	50209
GL172791.1	3522	4388	4196, 4194, 4191	34, 68, 19	32.06908, 71.79147, 20.8607	12.62816, 19.68802, 11.2325	28.51613,	3	NA	32534
GL172791.1	828379	828860	828733, 828736, 828445	7, 6, 5	6.74267, 4.76927, 5.3628	5.07552, 3.9431, 4.27046	4.23998, 2	3	HDAC5	13732
GL172791.1	1201185	1201675	1201596, 1201383, 1201557	12, 8, 7	13.8483, 7.07159, 8.69489	8.63436, 5.20073, 6.06379	10.86694,	3	pyy	15132
GL172791.1	1210170	1210704	1210678, 1210633, 1210605	90, 127, 60	113.68957, 162.73825, 80.85848	30.38214, 35.15539, 28.58476	109.18781	3	pyy	24117
GL172791.1	1289130	1289659	1289391, 1289428, 1289205	8, 6, 6	7.38183, 4.89312, 6.45058	5.38666, 4.02027, 4.90508	4.80856, 2	3	adam11	818
GL172791.1	1885858	1886266	1886221, 1885979, 1885948	7, 7, 8	7.1696, 5.97725, 10.1465	5.31345, 4.62287, 6.82176	4.61046, 3	3	plcd3	608
GL172792.1	178860	179266	179028, 179049, 179076	17, 6, 12	15.5079, 4.20515, 13.44151	8.56821, 3.5821, 8.41472	12.45101,	3	trpc6	5949
GL172792.1	192638	192974	192855, 192912, 192893	9, 11, 5	8.50607, 10.00475, 4.95468	5.95453, 6.57041, 4.05779	5.84194, 7	3	pgr	0
GL172792.1	686540	686996	686587, 686579, 686570	6, 11, 10	5.31708, 9.13706, 12.90749	4.2777, 6.0355, 8.21675	2.96727, 6	3	cntn5	0
GL172792.1	1072581	1073230	1073022, 1073065, 1073059	22, 30, 9	23.3605, 29.84375, 11.64359	11.81917, 12.84069, 7.57973	20.02603,	3	cntn5	216056
GL172792.1	1145883	1146544	1146031, 1146126, 1146014	13, 21, 8	14.79381, 21.89512, 8.6841	9.02996, 11.25804, 6.11715	11.77132,	3	cntn5	289358
GL172792.1	1220969	1221418	1221366, 1221367, 1221211	17, 24, 10	18.82666, 28.90463, 11.83521	10.55599, 14.44646, 7.70798	15.63723,	3	cntn5	364444
GL172792.1	1364818	1365420	1364830, 1364839, 1364854	48, 53, 35	55.32394, 64.2554, 42.65756	19.90263, 22.60698, 18.39291	51.39656,	3	ccdc82	481449
GL172792.1	1542645	1543882	1542890, 1542889, 1542889	43, 76, 39	44.97636, 84.76675, 44.1486	16.27951, 22.57431, 17.53404	41.19774,	3	ccdc82	302987
GL172792.1	1580138	1580477	1580415, 1580380, 1580420	8, 7, 9	7.99469, 5.42726, 11.64359	5.74128, 4.28044, 7.57973	5.38155, 3	3	ccdc82	266392
GL172792.1	1687720	1688066	1687978, 1687996, 1687978	15, 23, 10	14.98937, 24.86003, 11.83521	8.76428, 12.38952, 7.70798	11.95813,	3	ccdc82	158803
GL172792.1	1864671	1865186	1864701, 1864698, 1864675	9, 19, 10	7.39636, 16.62257, 10.3632	5.27874, 8.68263, 6.92219	4.82212, 1	3	maml2	0
GL172792.1	1888835	1889619	1889429, 1889097, 1888967	6, 6, 6	5.24248, 4.76927, 7.05929	4.2332, 3.9431, 5.20024	2.89962, 2	3	maml2	0
GL172792.1	1910322	1910650	1910542, 1910519, 1910501	6, 8, 4	5.81608, 6.97779, 4.38152	4.56563, 5.14375, 3.65448	3.4176, 4.5	3	maml2	0
GL172792.1	2090102	2090623	2090489, 2090486, 2090519	9, 14, 6	6.48491, 9.10849, 5.14244	4.70237, 5.41123, 4.17298	4.002, 6.56	3	sesn3	23175
GL172793.1	591744	592816	592186, 592089, 592758	12, 17, 6	11.85767, 15.5773, 6.12365	7.51014, 8.61051, 4.73409	8.98544, 1	3	cdh6	287111
GL172793.1	1193184	1193812	1193433, 1193608, 1193268	9, 8, 5	7.80169, 5.48596, 5.31857	5.52977, 4.2025, 4.24819	5.20141, 3	3	cdh9	95414
GL172793.1	1423988	1425051	1424691, 1424802, 1424775	12, 23, 5	12.97977, 24.67931, 5.9506	8.15868, 12.28149, 4.54784	10.04411,	3	cdh9	55006
GL172793.1	2185157	2186032	2185555, 2185577, 2185539	20, 32, 17	20.9301, 30.57544, 20.47493	10.98561, 12.52038, 11.4869	17.67118,	3	cdh10	109769
GL172794.1	401074	401966	401534, 401898, 401779	11, 14, 6	12.43693, 14.44087, 7.29403	7.97018, 8.65722, 5.30581	9.52692, 1	3	zfand5	17387
GL172794.1	1078157	1078432	1078319, 1078330, 1078312	14, 19, 9	12.97522, 17.76909, 11.64359	7.77689, 9.37618, 7.57973	10.03988,	3	klf9	23654
GL172794.1	1096036	1096338	1096182, 1096190, 1096211	31, 40, 24	33.93406, 43.34221, 27.81654	14.8672, 16.63072, 13.81213	30.34204,	3	klf9	5748
GL172794.1	1220584	1221145	1220866, 1220880, 1220858	21, 21, 19	22.22738, 17.35787, 22.32732	11.45719, 8.54015, 12.08192	18.92683,	3	mamdc2	0
GL172794.1	1848702	1849489	1849013, 1849014, 1848936	44, 47, 17	51.14963, 48.61391, 19.63801	19.35994, 16.60164, 11.0208	47.28226,	3	foxd4l1.1	6411
GL172794.1	2093985	2094801	2094137, 2094142, 2094143	10, 14, 9	9.44155, 12.92044, 10.56949	6.38646, 7.74345, 7.08032	6.72156, 1	3	kank1	0
GL172795.1	1640	1970	1765, 1944, 1789	5, 9, 4	4.655, 7.79702, 4.67325	3.89157, 5.5269, 3.78987	2.42652, 5	3	NA	28645
GL172795.1	421522	422357	422047, 422068, 421818	24, 27, 11	27.39936, 27.51274, 13.64572	13.56637, 12.57423, 8.58592	23.95304,	3	NA	4450
GL172795.1	764237	764814	764767, 764739, 764787	17, 18, 12	15.62395, 15.58811, 15.16114	8.63894, 8.34104, 9.30141	12.56245,	3	NA	5729
GL172795.1	767964	768883	768606, 768498, 768372	10, 15, 5	7.51175, 11.52762, 5.14967	5.19361, 6.65092, 4.16141	4.93097, 8	3	NA	1660
GL172795.1	843277	843853	843572, 843495, 843797	13, 10, 4	13.07382, 9.39128, 4.67325	8.04786, 6.35644, 3.78987	10.13353,	3	NA	72644
GL172795.1	1710491	1711090	1710943, 1710712, 1710859	8, 9, 6	7.57423, 7.03406, 6.35308	5.4999, 5.05123, 4.85498	4.98865, 4	3	sema3f	8835
GL172795.1	2191979	2194022	2193569, 2193233, 2193941	12, 11, 5	13.38718, 9.52738, 5.40789	8.38496, 6.27759, 4.29296	10.43197,	3	rbm5	15129
GL172796.1	64861	65977	65257, 65225, 65231	7, 13, 5	5.58859, 9.74816, 4.77515	4.38214, 6.01114, 3.9592	3.21321, 7	3	csrp2	18641
GL172796.1	67276	68151	67965, 67674, 67566	11, 14, 7	10.21742, 11.20037, 7.66926	6.69945, 6.68504, 5.57703	7.44132, 8	3	csrp2	21056
GL172796.1	1067669	1068441	1068401, 1068394, 1068351	23, 32, 15	26.67393, 31.08261, 17.32264	13.46354, 12.81022, 10.11551	23.24761,	3	pawr	28449
GL172796.1	1417858	1418601	1418050, 1418034, 1418163	20, 26, 14	10.87983, 11.95371, 8.3829	5.22574, 4.77747, 4.98293	8.06801, 9	3	hs6st3	33481
GL172796.1	1419680	1420169	1419980, 1420110, 1420093	55, 97, 35	50.97569, 92.37454, 32.68832	14.723, 17.99134, 12.60662	47.11012,	3	hs6st3	35303
GL172796.1	1421376	1422392	1421773, 1421719, 1421857	24, 50, 21	14.78803, 34.68545, 15.27678	6.38509, 9.23288, 7.34422	11.76639,	3	hs6st3	36999
GL172797.1	882257	883792	883183, 883141, 882570	14, 7, 6	14.67861, 5.07986, 7.12188	8.79666, 4.05845, 5.22884	11.66087,	3	XB-GENE-987190	0
GL172797.1	934274	935141	935003, 934601, 934900	17, 26, 16	11.85963, 20.64235, 14.3623	6.37578, 8.94315, 8.13466	8.98644, 1	3	ppp1r1a	18368
GL172797.1	1173586	1174156	1173820, 1173945, 1173817	9, 19, 10	7.03134, 15.17274, 9.82971	5.04951, 7.81547, 6.61563	4.50586, 1	3	pde1b	0
GL172798.1	9258	10171	9527, 9838, 9820	11, 14, 5	12.43693, 14.45897, 5.9506	7.97018, 8.66788, 4.54784	9.52692, 1	3	NA	27318
GL172798.1	644224	644995	644461, 644490, 644475	47, 52, 26	47.20149, 48.4026, 27.63785	15.84378, 14.55653, 13.01145	43.38986,	3	adamtsl1	0
GL172798.1	2049381	2050460	2049971, 2049882, 2050149	17, 23, 12	16.36399, 21.44504, 14.58408	9.08912, 10.34265, 9.01638	13.27324,	3	aco1	0
GL172798.1	2143855	2144415	2144137, 2144291, 2144141	9, 16, 9	9.72082, 17.16856, 11.64359	6.64182, 9.82359, 7.57973	6.97155, 1	3	ddx58	53572
GL172799.1	22215	23147	22262, 22285, 22290	26, 21, 13	30.62988, 21.65316, 16.49347	14.78972, 11.1127, 9.91245	27.10803,	3	nfe2l2	1122
GL172799.1	613611	614280	613738, 613919, 613864	9, 12, 8	9.12009, 10.93692, 10.1465	6.30997, 6.95571, 6.82176	6.42028, 8	3	hoxd8	3823
GL172799.1	681086	682317	681496, 681525, 681492	86, 123, 58	57.34502, 81.51438, 38.96116	9.70601, 10.09111, 9.12771	53.39062,	3	evx2	17047
GL172799.1	1481854	1482826	1482568, 1482510, 1482501	32, 29, 13	29.89085, 23.81251, 10.08084	12.13243, 9.7714, 6.21719	26.38588,	3	Telomerase-vert	3606
GL172799.1	1518209	1518873	1518336, 1518263, 1518630	5, 9, 4	4.50727, 7.35794, 4.38152	3.80664, 5.25474, 3.65448	2.30125, 4	3	plekha3	1349
GL172800.1	1966	3716	3131, 2756, 2993	31, 38, 16	28.09103, 30.18335, 14.64978	11.47454, 10.32487, 8.31036	24.62929,	3	xtr-mir-155	659776
GL172800.1	64806	65310	64862, 65037, 65144	8, 14, 8	7.02691, 13.26509, 9.05167	5.17362, 7.95332, 6.30653	4.50222, 1	3	xtr-mir-155	598182
GL172800.1	196484	197442	197075, 196811, 196932	14, 26, 16	15.94231, 28.58933, 19.65507	9.51659, 13.57987, 11.21774	12.86893,	3	xtr-mir-155	466050
GL172800.1	211522	212103	211528, 211554, 211601	13, 28, 23	13.79066, 28.80343, 29.05751	8.46641, 12.97367, 14.82811	10.8171, 2	3	xtr-mir-155	451389
GL172800.1	344159	344614	344411, 344452, 344386	5, 8, 5	4.33555, 6.27644, 5.9506	3.70555, 4.70819, 4.54784	2.15195, 3	3	xtr-mir-155	318878
GL172800.1	363856	364874	364216, 364314, 364314	22, 35, 16	23.62598, 36.93173, 18.0198	11.97797, 15.01776, 10.31552	20.28347,	3	xtr-mir-155	298618
GL172800.1	373883	374568	374112, 373918, 374154	7, 14, 3	6.99564, 12.33756, 3.47371	5.21786, 7.38613, 3.03189	4.47307, 9	3	xtr-mir-155	288924
GL172800.1	563601	564200	563984, 563858, 564117	8, 9, 7	8.42257, 8.21099, 8.57075	5.97763, 5.77858, 6.00887	5.76336, 5	3	xtr-mir-155	99292
GL172800.1	582887	583269	583129, 583065, 583093	11, 10, 6	10.43535, 7.75815, 6.25896	6.83063, 5.34918, 4.80589	7.6474, 5.2	3	xtr-mir-155	80223
GL172800.1	613045	613283	613279, 613252, 613255	9, 17, 11	8.82885, 15.81151, 13.28509	6.14328, 8.7532, 8.40871	6.14693, 1	3	xtr-mir-155	50209
GL172800.1	633371	633753	633481, 633481, 633481	18, 24, 11	18.07742, 22.20517, 11.49087	9.85073, 10.46619, 7.44794	14.9177, 1	3	xtr-mir-155	29739
GL172800.1	657757	658160	657985, 657995, 658021	15, 23, 16	16.54256, 22.03663, 17.55092	9.6759, 10.69607, 10.04619	13.44484,	3	xtr-mir-155	5332
GL172800.1	820965	821567	821044, 820996, 820998	13, 24, 20	13.00701, 26.67478, 23.35044	8.00827, 13.13685, 12.41	10.0703, 2	3	jam2	0
GL172800.1	1173610	1174906	1174164, 1174851, 1174467	17, 28, 10	15.5079, 25.81363, 10.1418	8.56821, 11.22036, 6.79622	12.45101,	3	cyyr1	66822
GL172800.1	1210909	1211191	1211101, 1210973, 1210988	12, 11, 7	13.8483, 9.13706, 7.61369	8.63436, 6.0355, 5.54854	10.86694,	3	adamts1	35765
GL172800.1	1293970	1295311	1294805, 1294672, 1294225, 1295016	6, 20, 5, 6	5.95712, 18.17471, 5.23257, 6.45058	4.64377, 9.32366, 4.20435, 4.90508	3.52158, 1	3	NA	6814
GL172800.1	1295923	1296319	1296020, 1296016, 1295988	6, 11, 9	5.47306, 8.00793, 10.64485	4.36955, 5.32781, 7.11743	3.10811, 5	3	NA	5806
GL172800.1	1382368	1382691	1382413, 1382397, 1382376	10, 15, 11	9.38842, 14.15433, 12.63525	6.35473, 8.25968, 8.07536	6.67167, 1	3	adamts5	0
GL172800.1	1626630	1626940	1626728, 1626766, 1626815	9, 9, 6	8.66407, 7.97047, 6.82211	6.04743, 5.63299, 5.08889	5.99101, 5	3	adamts5	189950
GL172800.1	2029430	2029900	2029778, 2029872, 2029782	12, 7, 5	11.98519, 5.48955, 5.40789	7.58551, 4.31982, 4.29296	9.10644, 3	3	adamts5	592750
GL172800.1	2049243	2050123	2049627, 2049441, 2049420	25, 42, 26	27.23898, 47.01839, 29.48523	13.12872, 17.87435, 14.11326	23.79707,	3	adamts5	612563
GL172800.1	2096000	2096624	2096482, 2096472, 2096429	29, 24, 17	27.65909, 20.37258, 16.89228	11.93514, 9.39405, 9.40884	24.20726,	3	adamts5	659320
GL172800.1	2119532	2120269	2119868, 2119999, 2120164	27, 33, 17	27.16312, 30.17618, 18.88718	12.36647, 11.93533, 10.59105	23.72391,	3	adamts5	682852
GL172801.1	511602	512454	511880, 511985, 512296	14, 15, 12	12.97522, 13.16372, 12.03712	7.77689, 7.65379, 7.61609	10.03988,	3	ATP6AP1L	27623
GL172801.1	823237	824057	823897, 823317, 823776	9, 8, 5	7.03134, 6.34359, 4.80993	5.04951, 4.75053, 3.97854	4.50586, 3	3	ssbp2	0
GL172801.1	828176	828808	828603, 828688, 828617	11, 19, 11	7.33946, 13.73923, 8.41671	4.9084, 6.97904, 5.58469	4.7691, 10	3	ssbp2	0
GL172801.1	832570	832998	832802, 832912, 832875	11, 10, 7	6.74933, 4.36227, 4.20114	4.54101, 3.23298, 3.48431	4.24602, 2	3	ssbp2	0
GL172801.1	921873	922620	922107, 922087, 922085	7, 20, 10	7.1696, 22.6463, 11.83521	5.31345, 12.00208, 7.70798	4.61046, 1	3	sctr	0
GL172801.1	1124051	1125469	1124223, 1125098, 1124779	18, 23, 13	13.39411, 15.39345, 11.59311	7.02979, 6.91525, 7.1528	10.43859,	3	znf281	43182
GL172801.1	1221385	1222291	1221746, 1222087, 1221477	11, 7, 5	12.17114, 5.21943, 5.23257	7.82679, 4.14808, 4.20435	9.28263, 2	3	tmem98	0
GL172801.1	1318784	1319451	1318969, 1318891, 1319387	11, 12, 12	10.1122, 9.82596, 13.59027	6.63573, 6.26981, 8.49571	7.34229, 7	3	NA	8040
GL172801.1	1414443	1415224	1414786, 1414893, 1414893	5, 8, 10	4.82177, 7.07159, 13.11399	3.98509, 5.20073, 8.30815	2.51418, 4	3	gngt2	18438
GL172801.1	1458338	1458878	1458359, 1458421, 1458356	13, 22, 8	13.71465, 20.6214, 8.56955	8.42261, 10.17315, 6.05652	10.74495,	3	NA	0
GL172801.1	1628756	1629643	1629305, 1628879, 1629390, 1629357	11, 6, 8, 5	9.43668, 4.92507, 7.06103, 5.9506	6.22151, 4.04003, 5.19433, 4.54784	6.7178, 2.7	3	NA	34523
GL172801.1	1735926	1736592	1736161, 1736182, 1736198	20, 26, 16	22.76587, 26.31054, 19.45311	12.07179, 12.21864, 11.10978	19.44912,	3	XB-GENE-977685	18150
GL172801.1	1845460	1846266	1845968, 1845968, 1845968	16, 14, 10	17.68339, 13.56877, 13.18184	10.1227, 8.1371, 8.33771	14.5382, 1	3	XB-GENE-977685	67769
GL172801.1	2040530	2041129	2040595, 2040582, 2040592	8, 17, 8	6.14488, 13.80996, 7.81204	4.62489, 7.53566, 5.63749	3.6868, 11	3	kat7	0
GL172801.1	2057558	2058031	2057862, 2057936, 2057897	8, 21, 12	7.33538, 20.19181, 13.01912	5.35908, 10.23207, 8.18077	4.76518, 1	3	kat7	2184
GL172801.1	2126333	2126660	2126616, 2126596, 2126597	15, 22, 16	16.90423, 22.85945, 20.763	9.88146, 11.51868, 11.79066	13.78827,	3	dlx4	16343
GL172801.1	2156112	2156344	2156149, 2156142, 2156151	10, 35, 11	9.23294, 36.39152, 11.92945	6.26141, 14.70421, 7.69409	6.52626, 3	3	dlx4	0
GL172801.1	2192733	2193341	2192766, 2192874, 2193038	8, 17, 7	7.83097, 16.76699, 7.34973	5.64832, 9.33319, 5.41038	5.22917, 1	3	dlx3	7654
GL172802.1	520149	520789	520624, 520431, 520291	8, 6, 4	6.86256, 4.30294, 4.59709	5.0733, 3.64568, 3.75554	4.3506, 2.2	3	NA	215417
GL172802.1	703508	703968	703933, 703900, 703936	11, 27, 14	9.95877, 27.59392, 17.40994	6.54239, 12.62252, 10.29833	7.19682, 2	3	TMEM121	84068
GL172802.1	1821879	1822566	1822430, 1822428, 1822426	59, 83, 46	57.83097, 78.98711, 48.37743	16.52176, 17.31703, 16.89428	53.8702, 7	3	U2	0
GL172802.1	1945889	1946321	1946003, 1945991, 1946009	26, 22, 24	32.15248, 23.53859, 31.72657	15.67586, 11.92574, 16.02936	28.59768,	3	U2	0
GL172802.1	1974224	1974640	1974500, 1974498, 1974489	46, 80, 34	43.86337, 78.09924, 35.04605	14.4857, 17.96031, 14.32502	40.10075,	3	U2	0
GL172802.1	1981039	1981613	1981326, 1981291, 1981292	100, 156, 59	99.55985, 154.54297, 56.17187	19.69594, 21.29558, 15.73598	95.17352,	3	U2	6351
GL172802.1	2017517	2017933	2017838, 2017557, 2017846	10, 10, 7	10.96713, 9.27513, 8.646	7.25596, 6.2868, 6.04229	8.14942, 6	3	U2	0
GL172802.1	2039348	2039645	2039456, 2039454, 2039457	60, 89, 45	65.28365, 103.10238, 52.14322	19.85302, 25.55578, 19.47273	61.23718,	3	U2	0
GL172802.1	2060197	2060626	2060330, 2060352, 2060336	30, 55, 31	32.88357, 63.58216, 39.98318	14.63653, 21.24671, 18.42929	29.31253,	3	U2	0
GL172802.1	2083777	2084177	2083948, 2083935, 2084091	15, 15, 8	15.05862, 12.35615, 7.72242	8.80576, 7.15789, 5.58595	12.0251, 9	3	U2	0
GL172802.1	2089201	2089597	2089343, 2089521, 2089500	13, 13, 8	12.56081, 11.03409, 8.9249	7.74168, 6.80804, 6.24211	9.64511, 8	3	U2	0
GL172802.1	2095904	2096229	2096061, 2096082, 2095966	16, 18, 9	12.5071, 14.02138, 7.06537	7.00284, 7.40006, 5.07099	9.59366, 1	3	U2	3353
GL172802.1	2099139	2099644	2099492, 2099476, 2099215	10, 23, 13	6.41567, 19.65454, 11.50623	4.49937, 9.28582, 7.09938	3.93897, 1	3	U2	0
GL172802.1	2111984	2112310	2112134, 2112115, 2112145	8, 8, 7	5.55847, 4.3574, 5.27202	4.24931, 3.47074, 4.18171	3.18478, 2	3	U2	0
GL172802.1	2117592	2118035	2117802, 2117812, 2117804	77, 102, 47	82.35289, 99.56876, 48.06312	20.98143, 19.0583, 16.30431	78.13491,	3	U2	68
GL172803.1	57070	57486	57340, 57290, 57437	10, 13, 6	10.67246, 11.46128, 6.93817	7.09497, 7.07172, 5.14396	7.87096, 8	3	krt8	14178
GL172803.1	326839	327409	327295, 327211, 327294	6, 22, 7	5.63898, 19.7681, 7.78342	4.46544, 9.66336, 5.63488	3.25772, 1	3	krt5.2	9081
GL172803.1	876721	877437	876928, 877049, 876950	18, 12, 5	18.22458, 10.39632, 5.59755	9.93936, 6.6236, 4.38538	15.0589, 7	3	asic1	38637
GL172803.1	981323	981663	981540, 981574, 981532	7, 16, 10	6.33443, 15.87543, 11.997	4.83795, 9.05466, 7.78835	3.86259, 1	3	asic1	0
GL172803.1	1754345	1755415	1755110, 1755075, 1754902	9, 21, 5	7.97705, 18.88456, 5.59755	5.637, 9.44608, 4.38538	5.36577, 1	3	NA	24079
GL172803.1	1773164	1773959	1773599, 1773546, 1773306	7, 8, 5	5.86458, 5.53815, 5.3628	4.55375, 4.2362, 4.27046	3.46146, 3	3	NA	5535
GL172803.1	1822769	1823402	1823278, 1823263, 1823317	32, 36, 14	36.92221, 38.83466, 16.82614	16.24024, 15.71415, 9.99514	33.27195,	3	NA	30016
GL172803.1	1972061	1972595	1972307, 1972323, 1972197	6, 8, 4	5.96715, 7.07159, 4.18775	4.64927, 5.20073, 3.55872	3.52158, 4	3	NA	12200
GL172803.1	1998091	1998959	1998664, 1998641, 1998664	15, 11, 6	14.27412, 10.15827, 5.99481	8.3325, 6.66366, 4.6644	11.27364,	3	NA	0
GL172803.1	2013997	2014609	2014315, 2014341, 2014410	7, 9, 5	5.65516, 7.39577, 5.80393	4.42382, 5.27837, 4.48186	3.27228, 4	3	U2	5984
GL172803.1	2027316	2028043	2027875, 2027758, 2027834	31, 33, 21	30.60197, 31.89384, 21.3191	12.90746, 12.89979, 10.9117	27.08083,	3	U2	6575
GL172803.1	2079742	2080387	2080201, 2079953, 2080097	6, 12, 6	5.51356, 10.83413, 5.67889	4.39314, 6.89288, 4.48822	3.14574, 8	3	NA	3548
GL172803.1	2097308	2097942	2097682, 2097309, 2097757	5, 7, 4	4.82177, 5.13485, 4.55201	3.98509, 4.09383, 3.73489	2.51418, 2	3	NA	0
GL172803.1	2108414	2109721	2108842, 2108649, 2109047	6, 10, 3	5.96715, 9.22453, 3.47371	4.64927, 6.25634, 3.03189	3.52158, 6	3	NA	798
GL172804.1	223682	224426	223753, 223896, 223871, 224234	19, 25, 15, 10	16.7072, 18.79307, 13.96443, 7.83362	8.73367, 8.21771, 8.14399, 5.39672	13.60294,	3	gbf1	0
GL172804.1	294928	295298	295096, 295199, 295084	12, 9, 7	12.45843, 7.83963, 7.29952	7.86167, 5.55305, 5.38357	9.54648, 5	3	elovl3	8752
GL172804.1	1066032	1066243	1066161, 1066153, 1066146	13, 18, 7	15.29169, 19.7713, 8.69489	9.29854, 10.85902, 6.06379	12.24396,	3	camk2g	69127
GL172804.1	1728792	1729484	1729114, 1729227, 1729282	13, 18, 8	13.07382, 16.6391, 9.05167	8.04786, 8.9791, 6.30653	10.13353,	3	col13a1	149368
GL172804.1	2009358	2010586	2010127, 2009730, 2010301	12, 10, 5	12.67544, 8.04981, 5.9506	7.98627, 5.53254, 4.54784	9.75458, 5	3	h2afy2	0
GL172804.1	2016979	2017942	2017487, 2017337, 2017654	8, 6, 15	8.42257, 4.93312, 19.64455	5.97763, 4.04501, 11.32852	5.76336, 2	3	h2afy2	0
GL172805.1	1223474	1224491	1223965, 1224291, 1223716	24, 32, 11	23.90185, 28.24161, 10.77078	11.47743, 11.21535, 7.03024	20.55107,	3	adora1	31729
GL172805.1	1225260	1225957	1225716, 1225431, 1225420	12, 18, 9	9.30462, 11.9328, 8.51771	5.94479, 6.18855, 5.96141	6.59363, 9	3	adora1	33515
GL172806.1	1006392	1006863	1006746, 1006569, 1006735	9, 16, 7	7.47395, 14.19759, 7.72582	5.32711, 8.03393, 5.6058	4.89626, 1	3	chst8	0
GL172806.1	1133128	1133405	1133287, 1133378, 1133345	5, 10, 4	4.50727, 9.22453, 4.67325	3.80664, 6.25634, 3.78987	2.30125, 6	3	chst8	83935
GL172806.1	1777720	1778928	1778382, 1778302, 1778057	26, 28, 14	22.90491, 21.94949, 13.81849	10.21727, 9.06824, 8.28728	19.58421,	3	LRP3	7656
GL172807.1	1849	3091	2202, 2319, 2913	8, 9, 3	8.42257, 8.21099, 3.47371	5.97763, 5.77858, 3.03189	5.76336, 5	3	kctd7	127672
GL172807.1	9943	11082	10597, 10709, 10727	25, 17, 7	23.44347, 11.7941, 5.27202	10.86418, 6.33766, 4.18171	20.10629,	3	kctd7	119681
GL172807.1	14035	15004	14317, 14341, 14720	12, 15, 9	6.80085, 7.93971, 5.9866	4.40352, 4.55427, 4.38351	4.29293, 5	3	kctd7	115759
GL172807.1	15234	15818	15533, 15533, 15307	13, 17, 7	7.99965, 8.85771, 4.30486	4.94388, 4.71219, 3.55268	5.38625, 6	3	kctd7	114945
GL172807.1	26064	26756	26159, 26277, 26504	6, 8, 4	5.96715, 7.07159, 4.67325	4.64927, 5.20073, 3.78987	3.52158, 4	3	kctd7	104007
GL172807.1	31755	32845	32245, 32323, 32143	14, 12, 12	14.10012, 10.44309, 13.44151	8.4555, 6.65248, 8.41472	11.10747,	3	kctd7	97918
GL172807.1	33683	34911	33968, 34789, 34770	6, 10, 6	5.27951, 9.07627, 5.75442	4.25534, 6.1667, 4.53101	2.9335, 6.5	3	kctd7	95852
GL172807.1	33805	35059	34840, 34789, 34770	7, 10, 6	5.65516, 9.07627, 5.75442	4.42382, 6.1667, 4.53101	3.27228, 6	3	kctd7	95704
GL172807.1	44648	46252	45084, 45821, 45841	9, 12, 9	8.16099, 9.95176, 10.42289	5.74847, 6.34805, 7.00726	5.53692, 7	3	kctd7	84511
GL172807.1	249113	250116	249551, 249611, 249427	16, 17, 17	17.2479, 17.63682, 20.77652	9.86999, 9.85561, 11.65116	14.1196, 1	3	U4	0
GL172807.1	263429	264389	263909, 263839, 263941	19, 10, 11	19.56679, 9.39128, 10.98082	10.46249, 6.35644, 7.15369	16.35231,	3	U4	0
GL172807.1	267608	268530	267769, 268307, 267868	10, 15, 8	9.13239, 15.80067, 7.59222	6.2007, 9.24574, 5.5104	6.43114, 1	3	U4	0
GL172807.1	338244	339086	338679, 338665, 338670	9, 14, 7	9.72082, 14.45897, 8.69489	6.64182, 8.66788, 6.06379	6.97155, 1	3	U4	0
GL172807.1	347110	348900	348256, 348274, 348340	23, 15, 18	25.23251, 15.26186, 20.23857	12.61172, 8.92711, 11.13122	21.84553,	3	U4	0
GL172807.1	363735	367540	366037, 364799, 366533	23, 29, 20	16.94087, 19.44072, 15.9025	7.74598, 7.53438, 7.97045	13.82308,	3	U4	0
GL172807.1	728124	728436	728374, 728390, 728405	13, 12, 16	12.62236, 10.08092, 16.83466	7.77868, 6.42828, 9.62725	9.70369, 7	3	tyw1	0
GL172807.1	1344736	1345440	1345098, 1344823, 1345226	9, 15, 6	8.16099, 12.49102, 6.87953	5.74847, 7.24068, 5.11627	5.53692, 9	3	auts2	18152
GL172807.1	1984915	1985881	1985629, 1985776, 1985556	12, 21, 8	12.38809, 20.81922, 9.76268	7.82099, 10.61044, 6.64964	9.48755, 1	3	NA	291102
GL172808.1	68443	69904	68943, 69417, 68947, 69592	16, 19, 11, 10	14.79948, 19.33741, 11.79996, 10.47831	8.40174, 10.3246, 7.62212, 6.98694	11.77649,	3	gja1	136242
GL172808.1	2089750	2091560	2090439, 2090052, 2090438, 2091340	7, 8, 10, 8	5.39768, 6.41216, 9.26679, 6.97106	4.26169, 4.79364, 6.28179, 5.13965	3.04019, 4	3	vnn1	51889
GL172809.1	594619	595103	594835, 594856, 594813	5, 16, 9	4.82177, 15.60807, 9.08997	3.98509, 8.89325, 6.2929	2.51418, 1	3	zbtb18	81542
GL172809.1	903748	903927	903813, 903837, 903825	79, 123, 89	96.60005, 148.73457, 120.36184	27.17778, 30.74738, 34.54525	92.23875,	3	rnaset2	0
GL172810.1	55516	56113	55543, 55585, 55559	12, 29, 7	10.68367, 29.43697, 7.15355	6.80062, 12.97595, 5.30471	7.88119, 2	3	tmem8a	0
GL172810.1	104307	105499	105068, 104677, 105050	18, 18, 10	17.72168, 16.6391, 12.25126	9.63591, 8.9791, 7.91211	14.57495,	3	RGS11	1878
GL172810.1	314934	315407	315339, 315358, 315396	13, 16, 11	12.14841, 14.41261, 12.48556	7.49227, 8.16543, 7.99611	9.26135, 1	3	mprip	36754
GL172810.1	339592	340529	339612, 339648, 340337	7, 13, 4	6.37715, 10.2718, 3.97874	4.86324, 6.33559, 3.45022	3.90257, 7	3	mprip	11632
GL172810.1	581896	582470	582371, 582085, 582206	9, 15, 4	7.75911, 12.86621, 3.32139	5.5036, 7.47111, 3.0752	5.16246, 1	3	rai1	23557
GL172810.1	635748	636363	636140, 636050, 635952	9, 16, 9	8.88537, 13.98946, 11.64359	6.17591, 7.9066, 7.57973	6.19961, 1	3	rai1	77409
GL172810.1	745764	746276	746125, 746145, 745986	27, 31, 15	31.21621, 31.11912, 16.59998	14.78047, 13.20829, 9.70872	27.68078,	3	pemt	0
GL172810.1	764443	764818	764785, 764759, 764670	12, 9, 4	13.05874, 7.75488, 3.79082	8.20296, 5.501, 3.34814	10.11961,	3	pemt	0
GL172810.1	770072	770469	770196, 770153, 770196	18, 20, 16	17.93309, 18.97338, 16.18697	9.76366, 9.80583, 9.24186	14.77832,	3	pemt	0
GL172810.1	814907	815343	815309, 815155, 815303	10, 17, 14	9.89241, 17.56014, 17.40994	6.65218, 9.80985, 10.29833	7.13431, 1	3	pemt	7957
GL172810.1	884444	885100	885045, 885057, 884836	7, 13, 6	6.12952, 11.07988, 5.793	4.71533, 6.83637, 4.55271	3.67231, 8	3	nt5m	8190
GL172810.1	956233	956878	956787, 956853, 956824	31, 47, 38	37.3478, 57.35493, 49.05736	16.89197, 21.55582, 20.86776	33.6894, 5	3	pld6	23990
GL172810.1	1039593	1039859	1039733, 1039765, 1039753	9, 12, 5	7.93244, 11.60251, 5.85847	5.60981, 7.3582, 4.50665	5.32435, 8	3	shmt1	10163
GL172810.1	1101985	1102539	1102306, 1102489, 1102536	8, 17, 10	6.70593, 14.31774, 11.75646	4.9768, 7.84337, 7.66841	4.20487, 1	3	flii	301
GL172810.1	1163520	1164289	1163776, 1163983, 1163854	14, 13, 19	10.30605, 8.33474, 14.10351	6.13551, 5.14537, 7.18906	7.52543, 5	3	llgl1	5953
GL172810.1	1166889	1168165	1167507, 1167291, 1167693	26, 28, 20	24.00086, 24.31455, 15.64067	10.85303, 10.36614, 7.81751	20.64756,	3	alkbh5	6861
GL172810.1	1379772	1380916	1380025, 1380131, 1380174	26, 32, 9	30.3953, 37.16949, 11.58206	14.65168, 16.38697, 7.55286	26.87883,	3	NA	6389
GL172810.1	1470697	1471016	1470987, 1470990, 1470978	14, 19, 12	13.14827, 18.75575, 16.36823	7.88233, 9.97369, 9.85365	10.20482,	3	NA	2396
GL172810.1	1536898	1537199	1536913, 1536993, 1537015	18, 21, 10	19.17347, 20.25932, 10.59656	10.50657, 10.27277, 7.05292	15.97256,	3	NA	4360
GL172810.1	1737026	1737937	1737619, 1737621, 1737660	12, 21, 9	11.26426, 20.12483, 8.47365	7.15468, 10.19169, 5.93535	8.4212, 17	3	NA	2187
GL172810.1	1828160	1828946	1828719, 1828718, 1828702	11, 13, 6	9.71398, 10.51368, 6.6567	6.39253, 6.48562, 5.00845	6.97155, 7	3	MEIOB	23241
GL172810.1	1865938	1866529	1866181, 1866168, 1866331	5, 12, 4	4.73273, 9.82596, 4.46488	3.93547, 6.26981, 3.69425	2.49377, 7	3	hs3st6	0
GL172810.1	1928711	1929116	1928979, 1928991, 1928976	27, 18, 13	28.0693, 17.32425, 13.58227	12.90567, 9.39518, 8.34597	24.60796,	3	hs3st6	24765
GL172810.1	1935434	1936215	1935649, 1935781, 1935921	7, 8, 5	6.60038, 7.07159, 5.45388	4.99378, 5.20073, 4.3157	4.10761, 4	3	hs3st6	31488
GL172810.1	1942783	1943332	1943144, 1943174, 1943144	17, 22, 13	14.15399, 19.37031, 13.45746	7.74398, 9.42716, 8.27333	11.15907,	3	hs3st6	38837
GL172810.1	1944435	1944884	1944819, 1944871, 1944844	32, 34, 18	32.11143, 33.84297, 19.45123	13.40381, 13.63168, 10.67087	28.55731,	3	hs3st6	40489
GL172810.1	2064892	2065393	2065071, 2065212, 2065204	7, 11, 5	7.1696, 10.60895, 5.85847	5.31345, 6.9343, 4.50665	4.61046, 7	3	tex2.1	2585
GL172810.1	2078352	2079377	2078862, 2079044, 2079060	15, 7, 8	17.09296, 5.97725, 9.1831	9.98755, 4.62287, 6.37229	13.96978,	3	tex2.1	16045
GL172811.1	364204	364979	364464, 364404, 364553	16, 16, 9	17.86567, 17.14793, 10.79992	10.22745, 9.81151, 7.19283	14.71418,	3	mfsd7	7887
GL172811.1	1436817	1437277	1437199, 1437204, 1437237	14, 13, 4	14.03163, 10.63899, 4.55201	8.41471, 6.56333, 3.73489	11.04222,	3	melk	92605
GL172811.1	1654625	1655336	1654841, 1655002, 1654951	7, 11, 6	7.1696, 10.15827, 6.76586	5.31345, 6.66366, 5.06179	4.61046, 7	3	pax5	0
GL172811.1	2092677	2093585	2093136, 2093030, 2092972	24, 40, 12	26.97936, 44.80341, 15.47496	13.31777, 17.47621, 9.45079	23.5443, 4	3	fbxo10	53439
GL172812.1	40672	41079	40785, 40679, 40696	9, 8, 3	9.36986, 6.70176, 3.47371	6.44997, 4.97421, 3.03189	6.65425, 4	3	map2k6	186
GL172812.1	55712	56148	55965, 55808, 55963	6, 11, 4	5.47306, 10.60895, 4.67325	4.36955, 6.9343, 3.78987	3.10811, 7	3	map2k6	15226
GL172812.1	186509	186745	186685, 186696, 186691	12, 15, 4	12.90199, 15.80067, 4.38152	8.11489, 9.24574, 3.65448	9.97033, 1	3	kcnj16	47501

GL172812.1	346777	347875	347468, 347531, 346880	6, 11, 6	5.77068, 9.66539, 7.29403	4.54016, 6.36265, 5.30581	3.37745, 7	3	kcnj2	34172
GL172812.1	374982	375465	375062, 375067, 375150	7, 9, 8	7.1696, 8.0603, 9.31951	5.31345, 5.68758, 6.43944	4.61046, 5	3	kcnj2	62377
GL172812.1	998592	999004	998741, 998922, 998797	6, 10, 4	5.31708, 8.84031, 4.11547	4.2777, 6.02287, 3.5218	2.96727, 6	3	sox9	306659
GL172812.1	1076863	1077218	1076904, 1076920, 1076869	16, 22, 18	16.01695, 23.19276, 21.49679	9.13983, 11.71868, 11.84671	12.9408, 2	3	sox9	228445
GL172812.1	1485864	1486609	1486194, 1486326, 1486205	14, 37, 14	13.20712, 36.0262, 14.79463	7.91811, 13.70846, 8.86428	10.26077,	3	slc39a11	74037
GL172812.1	1680875	1681269	1681154, 1681195, 1681128	16, 27, 12	15.60786, 27.35215, 13.15613	8.89312, 12.47874, 8.25729	12.5472, 2	3	slc39a11	75473
GL172812.1	1779927	1781097	1780506, 1780608, 1780505	18, 24, 9	19.43049, 23.27012, 9.76105	10.65863, 11.09959, 6.66343	16.22032,	3	sstr2	11571
GL172813.1	145179	145730	145432, 145504, 145472	4, 9, 5	3.74195, 8.21099, 5.9506	3.32091, 5.77858, 4.54784	1.6222, 5.7	3	thg1l	18429
GL172813.1	208023	208720	208487, 208516, 208516	15, 16, 9	13.80112, 15.47832, 10.07825	8.04428, 8.81468, 6.83102	10.82722,	3	il9	11118
GL172813.1	297658	298216	298037, 297872, 298005	16, 20, 11	14.68346, 21.01248, 12.9493	8.33092, 11.03495, 8.23866	11.66544,	3	slc25a48	56038
GL172813.1	373066	373622	373596, 373587, 373586	19, 33, 10	21.31193, 33.94907, 11.997	11.49694, 14.08334, 7.78835	18.04063,	3	cxcl14	15255
GL172813.1	555676	556678	556307, 556149, 556150	18, 36, 10	17.65252, 37.52903, 11.5283	9.59407, 14.95653, 7.55211	14.50784,	3	h2afy	20605
GL172813.1	644308	644652	644355, 644581, 644533	11, 6, 7	10.72304, 4.93312, 8.69489	7.00201, 4.04501, 6.06379	7.91902, 2	3	h2afy	31700
GL172813.1	702888	703422	703376, 703381, 703390	25, 21, 11	28.13526, 23.12148, 14.68355	13.6627, 11.98982, 9.06344	24.67218,	3	h2afy	90280
GL172813.1	956304	956715	956431, 956429, 956411	26, 31, 17	29.08397, 30.1239, 22.63257	13.87468, 12.63089, 12.61306	25.59847,	3	pitx1	15439
GL172813.1	2029180	2029650	2029555, 2029258, 2029273	9, 7, 5	9.72082, 5.97725, 5.9506	6.64182, 4.62287, 4.54784	6.97155, 3	3	pcdha13	25334
GL172814.1	23858	24278	24052, 24124, 24228	12, 9, 13	10.20921, 6.86591, 14.77384	6.50784, 4.94483, 9.01902	7.4341, 4.4	3	NA	113668
GL172814.1	24670	25133	24993, 24872, 24811	10, 9, 8	8.08479, 6.58184, 7.85773	5.55446, 4.7642, 5.66361	5.46601, 4	3	NA	112813
GL172814.1	49630	50144	49652, 49686, 49898	10, 27, 11	9.89241, 26.96051, 10.92742	6.65218, 12.24626, 7.12242	7.13431, 2	3	NA	87802
GL172814.1	94492	94984	94935, 94873, 94930	6, 8, 13	5.72604, 6.70176, 15.70541	4.51498, 4.97421, 9.51556	3.33547, 4	3	NA	42962
GL172814.1	204056	204502	204467, 204470, 204455	15, 17, 7	15.41679, 14.81481, 6.83761	9.0192, 8.14576, 5.12939	12.36344,	3	myocd	0
GL172814.1	224271	224736	224585, 224626, 224521	8, 9, 6	7.28955, 6.93223, 5.20372	5.33177, 4.98685, 4.20995	4.72176, 4	3	myocd	0
GL172814.1	250300	250744	250522, 250483, 250601	13, 10, 5	15.29169, 9.39128, 5.9506	9.29854, 6.35644, 4.54784	12.24396,	3	map2k4	0
GL172814.1	264500	265241	265043, 264998, 264844	14, 18, 8	15.49091, 16.69883, 8.74279	9.26387, 9.01539, 6.14792	12.43497,	3	map2k4	0
GL172814.1	338025	338409	338100, 338179, 338396	8, 17, 6	6.86256, 15.35073, 6.87953	5.0733, 8.4724, 5.11627	4.3506, 12	3	NA	0
GL172814.1	391300	391650	391449, 391443, 391341	11, 14, 9	10.16451, 12.1395, 10.87975	6.66744, 7.26424, 7.23113	7.39114, 9	3	NA	20344
GL172814.1	420129	420492	420319, 420160, 420253	10, 18, 10	9.60506, 17.3898, 13.18184	6.48358, 9.43492, 8.33771	6.87658, 1	3	NA	4487
GL172814.1	470970	471934	471285, 471283, 471307	8, 19, 7	7.72597, 17.70754, 7.01416	5.588, 9.33888, 5.22813	5.13061, 1	3	znf684	13602
GL172814.1	664253	664754	664648, 664646, 664563	7, 8, 8	7.1696, 7.07159, 9.8421	5.31345, 5.20073, 6.68602	4.61046, 4	3	znf850	43421
GL172814.1	733735	734285	734072, 733810, 733816	17, 19, 10	19.91381, 19.88391, 13.18184	11.176, 10.65254, 8.33771	16.68708,	3	uts2r	92864
GL172814.1	770339	771205	770932, 770909, 770984	11, 16, 11	10.1122, 11.24723, 12.06254	6.63573, 6.24794, 7.76744	7.34229, 8	3	uts2r	55944
GL172814.1	774927	776168	775505, 775504, 775558	12, 13, 4	11.09909, 9.08335, 3.48964	7.05451, 5.60136, 3.17588	8.26391, 6	3	uts2r	50981
GL172814.1	787781	788512	787850, 787889, 788353	7, 12, 4	7.04884, 11.1491, 4.64325	5.2473, 7.08489, 3.77643	4.52221, 8	3	uts2r	38637
GL172814.1	841207	841567	841523, 841516, 841517	28, 29, 23	33.20707, 33.66345, 27.08901	15.59147, 15.4869, 13.70572	29.62933,	3	uts2r	12926
GL172814.1	859429	860103	859920, 859594, 859714	6, 9, 9	5.96715, 8.21099, 11.64359	4.64927, 5.77858, 7.57973	3.52158, 5	3	uts2r	31148
GL172814.1	869494	869986	869748, 869748, 869748	11, 14, 6	12.43693, 14.45897, 7.29403	7.97018, 8.66788, 5.30581	9.52692, 1	3	uts2r	41213
GL172814.1	957145	957885	957591, 957792, 957637	5, 6, 4	4.17642, 4.53871, 3.67542	3.60969, 3.79731, 3.28338	2.01422, 2	3	ogfod3	876
GL172814.1	970618	971116	970756, 970688, 970668	21, 18, 14	20.87917, 16.52108, 17.61756	10.64659, 8.90737, 10.40352	17.62177,	3	ogfod3	0
GL172814.1	1018673	1019009	1018828, 1018816, 1018859	32, 30, 25	35.59971, 26.24524, 29.42358	15.45531, 10.78545, 14.4233	31.9745, 2	3	hexdc	0
GL172814.1	1020186	1020684	1020658, 1020656, 1020647	9, 25, 8	6.545, 20.78471, 7.00671	4.74071, 9.32217, 5.16135	4.05782, 1	3	hexdc	0
GL172814.1	1037815	1038202	1037943, 1037980, 1037888	9, 9, 6	8.55802, 7.35794, 5.99481	5.98518, 5.25474, 4.6644	5.8909, 4.9	3	narf	0
GL172814.1	1125291	1125548	1125337, 1125347, 1125346	17, 15, 10	14.95512, 11.60543, 10.42037	8.23125, 6.69836, 6.95441	11.92636,	3	abcc3	11940
GL172814.1	1185480	1185984	1185953, 1185975, 1185932	21, 24, 11	26.14967, 25.29108, 14.24187	13.73288, 12.31022, 8.86619	22.7357, 2	3	dhrs7c	0
GL172814.1	1197486	1198528	1197790, 1197917, 1197789	12, 17, 14	10.44071, 16.63185, 17.2091	6.65101, 9.25145, 10.19524	7.65237, 1	3	dhrs7c	11090
GL172814.1	1268302	1268533	1268492, 1268496, 1268491	13, 7, 7	12.94099, 5.97725, 7.72582	7.96907, 4.62287, 5.6058	10.00729,	3	9-Sep	48264
GL172814.1	1296003	1296480	1296078, 1296174, 1296235	5, 7, 8	4.40263, 4.94697, 7.81204	3.74533, 3.9726, 5.63749	2.21004, 2	3	9-Sep	20317
GL172814.1	1391942	1392793	1392328, 1392474, 1392672	17, 29, 8	14.74586, 26.95941, 8.6841	8.10377, 11.53125, 6.11715	11.72534,	3	9-Sep	19193
GL172814.1	1407395	1408061	1407566, 1407605, 1407588	24, 28, 25	26.2838, 29.57832, 31.16689	12.90395, 13.43449, 15.43191	22.86648,	3	9-Sep	34646
GL172814.1	1489136	1490281	1490077, 1489888, 1489612	10, 13, 11	8.89113, 11.61168, 11.4314	6.05396, 7.16421, 7.41405	6.20456, 8	3	XB-GENE-5831307	530
GL172814.1	1500069	1500692	1500249, 1500229, 1500235	13, 26, 14	11.81837, 25.54817, 16.29325	7.29094, 11.76506, 9.7093	8.94738, 2	3	sec14l5	2983
GL172814.1	1545659	1546261	1545957, 1546063, 1545748	9, 13, 5	9.63662, 12.86565, 5.50079	6.59633, 7.92422, 4.33868	6.90556, 1	3	mgat5b	0
GL172814.1	1551246	1551792	1551466, 1551617, 1551510	6, 25, 10	5.96715, 25.42729, 11.5283	4.64927, 12.04472, 7.55211	3.52158, 2	3	mgat5b	0
GL172814.1	1571979	1572621	1572250, 1572565, 1572584	10, 23, 10	7.61309, 17.63909, 9.82971	5.25765, 8.13307, 6.61563	5.02606, 1	3	mgat5b	5547
GL172814.1	1636291	1636510	1636469, 1636451, 1636466	22, 15, 13	20.21823, 14.15433, 13.33541	9.93183, 8.25968, 8.20195	16.98149,	3	mxra7	0
GL172814.1	1650787	1651457	1651033, 1651015, 1651188	7, 8, 5	7.1696, 6.17826, 5.91431	5.31345, 4.64607, 4.53172	4.61046, 3	3	mxra7	0
GL172814.1	1709110	1709483	1709433, 1709184, 1709184	23, 36, 11	23.25035, 34.54777, 10.5698	11.42428, 13.26617, 6.91098	19.91801,	3	st6galnac2	5289
GL172814.1	2045989	2046670	2046022, 2046018, 2046021	24, 33, 18	24.13149, 36.37105, 21.89732	11.615, 15.506, 12.06835	20.77422,	3	foxj1	4878
GL172814.1	2066874	2067665	2067317, 2067317, 2067005, 2067394	15, 31, 5, 6	14.58913, 30.65007, 5.19074, 6.71074	8.52337, 12.93538, 4.18277, 5.03498	11.57535,	3	exoc7	4011
GL172815.1	701494	702161	702102, 702037, 702121	10, 19, 9	8.98596, 16.51679, 10.56949	6.11182, 8.61887, 7.08032	6.29482, 1	3	dnajb12	5507
GL172815.1	1692029	1692467	1692113, 1692176, 1692174	9, 10, 12	9.72082, 9.39128, 15.36829	6.64182, 6.35644, 9.40047	6.97155, 6	3	tll2	47190
GL172816.1	7589	8014	7788, 7789, 7788	30, 51, 32	31.20275, 59.64859, 41.35728	13.63964, 20.94785, 18.84537	27.66769,	3	NA	547557
GL172816.1	36170	36552	36532, 36431, 36416	8, 6, 6	7.57423, 4.93312, 6.168	5.4999, 4.04501, 4.75778	4.98865, 2	3	NA	519019
GL172816.1	82313	82765	82631, 82631, 82612	5, 12, 5	4.82177, 11.86108, 5.75061	3.98509, 7.51216, 4.45735	2.51418, 9	3	NA	472806
GL172816.1	206266	206490	206444, 206480, 206421	14, 17, 11	14.75506, 16.97468, 11.79996	8.84125, 9.45853, 7.62212	11.73423,	3	NA	349081
GL172816.1	347291	347613	347502, 347574, 347579	18, 41, 16	19.8791, 43.51781, 20.52695	10.9221, 16.30392, 11.67144	16.65356,	3	NA	207958
GL172816.1	375221	375957	375891, 375818, 375888	20, 35, 15	19.02197, 32.67583, 14.65165	9.83519, 12.59973, 8.56113	15.82604,	3	NA	179614
GL172816.1	474232	474654	474561, 474345, 474326	15, 15, 8	15.56593, 14.98313, 8.6841	9.10751, 8.76054, 6.11715	12.50679,	3	NA	80917
GL172816.1	479945	480343	480017, 479947, 479986	14, 22, 16	15.84929, 24.17682, 19.55336	9.46495, 12.30626, 11.1635	12.77923,	3	NA	75228
GL172816.1	942305	942690	942536, 942543, 942536	7, 12, 5	6.69449, 10.93692, 5.80393	5.04797, 6.95571, 4.48186	4.1953, 8.2	3	atad2b	0
GL172816.1	954658	954944	954763, 954780, 954768	18, 17, 9	16.86914, 13.09378, 7.79368	9.11888, 7.10488, 5.52485	13.75452,	3	ubxn2a	3573
GL172816.1	1102369	1102675	1102640, 1102648, 1102618	14, 18, 18	12.48519, 16.46278, 21.30401	7.47686, 8.87194, 11.73892	9.57251, 1	3	fkbp1b	7258
GL172816.1	1390270	1390795	1390682, 1390758, 1390737	7, 14, 6	6.74267, 12.28739, 6.93817	5.07552, 7.35528, 5.14396	4.23998, 9	3	sbk1	18830
GL172816.1	1514380	1515207	1515003, 1514753, 1514909	12, 19, 5	10.88704, 16.5695, 5.31857	6.92524, 8.65063, 4.24819	8.0744, 13	3	eva1a	0
GL172816.1	1573290	1573776	1573752, 1573750, 1573737	23, 42, 31	27.48315, 48.26707, 42.03584	13.9341, 18.59886, 19.59254	24.03497,	3	eva1a	45812
GL172816.1	1583664	1584109	1584001, 1584021, 1584018	31, 29, 23	30.99386, 28.0611, 26.89275	13.13529, 12.16882, 13.59141	27.46391,	3	eva1a	56186
GL172816.1	1741883	1742570	1741972, 1741915, 1741932	58, 65, 40	66.60995, 71.53575, 51.25164	21.4875, 20.86035, 21.26753	62.54853,	3	ptchd4	0
GL172816.1	1764053	1764691	1764657, 1764427, 1764355	13, 21, 7	13.63968, 17.21476, 6.83761	8.37925, 8.45625, 5.12939	10.67258,	3	ptchd4	0
GL172816.1	1772733	1774355	1773615, 1774265, 1773942	9, 25, 9	9.72082, 30.29745, 11.21577	6.64182, 14.93242, 7.38849	6.97155, 2	3	ptchd4	0
GL172816.1	1779205	1780169	1779512, 1779500, 1779641	10, 12, 6	10.81731, 11.86108, 7.29403	7.17456, 7.51216, 5.30581	8.00764, 9	3	ptchd4	0
GL172816.1	1804438	1805552	1805252, 1805067, 1804836	20, 21, 18	19.56371, 18.38042, 19.45123	10.16265, 9.14502, 10.67087	16.34986,	3	opn5	11157
GL172816.1	1828728	1829277	1829164, 1829224, 1829248	17, 24, 14	18.29581, 25.03583, 15.64845	10.24594, 12.15722, 9.35267	15.12565,	3	opn5	0
GL172816.1	2032433	2032870	2032868, 2032821, 2032815	17, 23, 12	16.97511, 20.53538, 13.01912	9.45879, 9.80281, 8.18077	13.85654,	3	tnfrsf21	0
GL172817.1	1083631	1084397	1083862, 1084201, 1083904	12, 9, 11	11.37748, 8.0603, 12.78973	7.22306, 5.68758, 8.15619	8.52862, 5	3	hddc2	0
GL172817.1	1482565	1483294	1482766, 1482853, 1482889	19, 29, 25	19.25925, 26.95941, 28.55542	10.27754, 11.53125, 13.91184	16.05534,	3	cdh1	6614
GL172818.1	89811	90358	90029, 90036, 90036	26, 35, 19	26.52554, 35.78582, 22.23331	12.34687, 14.35456, 12.02841	23.10248,	3	hlx	238959
GL172818.1	118110	119039	118330, 118313, 118332	336, 300, 242	360.05682, 319.41364, 287.36414	27.92488, 27.19112, 34.16838	354.68808	3	hlx	210278
GL172818.1	219566	220363	220130, 219880, 219647	7, 11, 3	6.55439, 8.66477, 3.17999	4.96711, 5.74034, 2.89245	4.06616, 6	3	hlx	108954
GL172818.1	285709	286458	285761, 286212, 285748	5, 13, 4	4.82177, 12.4939, 4.08034	3.98509, 7.7014, 3.50363	2.51418, 9	3	hlx	42859
GL172818.1	348647	349130	348920, 349033, 348941	7, 7, 8	6.42049, 5.19095, 7.95096	4.8888, 4.12984, 5.71659	3.94161, 2	3	hlx	11573
GL172818.1	689639	689886	689856, 689832, 689672	5, 12, 5	4.69353, 11.42765, 5.9506	3.9134, 7.25328, 4.54784	2.45909, 8	3	slc30a10	21768
GL172818.1	798569	799690	799168, 799168, 799168	69, 104, 57	75.57145, 115.16446, 66.68901	21.12648, 24.48441, 22.01362	71.41886,	3	slc30a10	82401
GL172818.1	817214	818488	817255, 817535, 818314	6, 7, 7	4.93136, 5.97725, 8.08824	4.04392, 4.62287, 5.7849	2.61555, 3	3	slc30a10	101046
GL172818.1	833229	833610	833570, 833559, 833495	12, 12, 5	11.79509, 11.1491, 5.59755	7.47301, 7.08489, 4.38538	8.92495, 8	3	slc30a10	117061
GL172818.1	935391	935866	935837, 935839, 935818	105, 129, 80	137.02765, 163.33684, 110.1001	34.30665, 34.41624, 34.0765	132.37453	3	lyplal1	22074
GL172818.1	967263	967474	967306, 967288, 967278	20, 36, 25	21.61731, 41.03524, 32.82711	11.39575, 17.00632, 16.36153	18.33537,	3	lyplal1	0
GL172818.1	1014287	1014662	1014288, 1014301, 1014295	24, 30, 11	25.81478, 30.4801, 13.64572	12.62377, 13.21372, 8.58592	22.41065,	3	lyplal1	24758
GL172818.1	1153856	1154395	1153921, 1153980, 1153926	14, 30, 12	14.3105, 31.58953, 12.7555	8.58029, 13.86841, 8.03189	11.30855,	3	tgfb2	102624
GL172818.1	1159693	1160791	1160481, 1160488, 1160510	10, 20, 4	8.98596, 17.28879, 4.34116	6.11182, 8.7916, 3.63492	6.29482, 1	3	tgfb2	96228
GL172818.1	1184580	1184923	1184761, 1184746, 1184720	6, 5, 9	4.51306, 3.65953, 10.28145	3.78094, 3.2852, 6.93568	2.30616, 1	3	tgfb2	72096
GL172818.1	1326293	1326771	1326304, 1326399, 1326354	5, 8, 10	4.27048, 6.37769, 10.3632	3.6666, 4.77199, 6.92219	2.09564, 4	3	tgfb2	0
GL172818.1	1371755	1372672	1372516, 1372501, 1372538	13, 26, 24	12.94099, 24.10549, 29.36219	7.96907, 10.91422, 14.70979	10.00729,	3	NA	9357
GL172818.1	1395684	1396976	1396626, 1395899, 1396013	11, 35, 15	10.49148, 36.39152, 16.01939	6.86423, 14.70421, 9.37366	7.70044, 3	3	NA	1221
GL172818.1	1465295	1465844	1465507, 1465376, 1465661	6, 8, 7	5.96715, 6.89656, 8.42533	4.64927, 5.09414, 5.94313	3.52158, 4	3	NA	70832
GL172818.1	1597476	1597975	1597867, 1597665, 1597810	8, 14, 5	7.57423, 13.38467, 4.64121	5.4999, 8.02582, 3.88372	4.98865, 1	3	gpatch2	97590
GL172818.1	1663148	1663462	1663190, 1663208, 1663219	5, 6, 5	4.82177, 4.93312, 5.9506	3.98509, 4.04501, 4.54784	2.51418, 2	3	gpatch2	32103
GL172818.1	1704146	1704641	1704253, 1704436, 1704269	12, 12, 6	11.92103, 9.62333, 6.35308	7.54764, 6.14359, 4.85498	9.04508, 7	3	gpatch2	0
GL172818.1	1777080	1777744	1777200, 1777132, 1777155	7, 16, 3	6.94335, 14.30421, 3.30894	5.18876, 8.09914, 2.9554	4.42528, 1	3	gpatch2	13825
GL172818.1	1807075	1807594	1807507, 1807515, 1807536	8, 9, 10	7.72597, 7.75488, 12.61444	5.588, 5.501, 8.08336	5.13061, 5	3	gpatch2	43820
GL172818.1	1906819	1907681	1907053, 1906885, 1907414	6, 8, 6	5.96715, 7.06103, 6.87953	4.64927, 5.19433, 5.11627	3.52158, 4	3	gpatch2	143564
GL172818.1	1936690	1937169	1937112, 1937120, 1937115	19, 38, 10	21.50921, 40.45844, 11.38247	11.61175, 15.81947, 7.47651	18.23049,	3	gpatch2	173435
GL172818.1	2014716	2015148	2015060, 2015059, 2015059	8, 16, 8	7.67464, 16.51912, 8.4586	5.55832, 9.44018, 5.99709	5.08299, 1	3	gpatch2	251461
GL172818.1	2027511	2028018	2027687, 2027687, 2027593	8, 8, 5	7.72597, 6.41216, 5.06966	5.588, 4.79364, 4.11933	5.13061, 4	3	gpatch2	264256
GL172819.1	217736	218693	218015, 218262, 217937	11, 20, 11	9.95877, 17.5013, 11.73651	6.54239, 8.91884, 7.58663	7.19682, 1	3	NA	56057
GL172819.1	734603	735475	734992, 735211, 735193	14, 8, 5	13.76536, 5.93025, 5.91431	8.2554, 4.48816, 4.53172	10.79347,	3	ap2a2	10357
GL172819.1	889181	889902	889640, 889662, 889457	116, 175, 79	102.28764, 152.71681, 71.45931	16.17152, 16.84552, 15.63634	97.8777, 1	3	NA	4094
GL172819.1	1650417	1651673	1650968, 1651130, 1651094	20, 36, 21	21.89018, 37.61859, 26.12149	11.55756, 15.00821, 13.71725	18.60009,	3	NA	0
GL172820.1	131	1449	584, 1034, 904	13, 20, 8	14.26982, 16.83032, 8.80246	8.73911, 8.51818, 6.179	11.26966,	3	rps6ka3	31491
GL172820.1	8307	8814	8570, 8590, 8763	10, 10, 7	4.5807, 3.88475, 4.10146	3.36353, 2.95209, 3.41853	2.36367, 1	3	rps6ka3	24126
GL172820.1	12506	13105	12710, 12599, 12841	14, 17, 11	9.89837, 12.66847, 8.73264	5.887, 6.85145, 5.78287	7.14011, 9	3	rps6ka3	19835
GL172820.1	19087	19857	19568, 19639, 19314	10, 10, 12	10.07458, 8.70492, 13.66628	6.75761, 5.93975, 8.53679	7.30652, 6	3	rps6ka3	13083
GL172820.1	43223	43566	43436, 43458, 43340	11, 10, 9	5.80019, 5.02586, 5.71106	3.96094, 3.63325, 4.20698	3.40481, 2	3	rps6ka3	0
GL172820.1	45556	47741	46417, 46670, 47163, 46475	22, 20, 9, 13	16.54164, 14.75447, 4.59883, 10.2815	7.79085, 7.30627, 3.49993, 6.34161	13.44447,	3	rps6ka3	0
GL172820.1	53400	54250	54021, 54157, 53881	10, 6, 10	11.06011, 4.93312, 13.18184	7.306, 4.04501, 8.33771	8.22712, 2	3	rps6ka3	0
GL172820.1	1917405	1918313	1918061, 1918104, 1918053	48, 67, 40	42.03876, 56.43657, 40.42059	12.88262, 13.3169, 14.9754	38.30383,	3	slc43a2	0
GL172821.1	211202	211548	211278, 211352, 211409	7, 14, 7	6.60038, 13.14791, 8.21885	4.99378, 7.88211, 5.84717	4.10761, 1	3	vrk1	717942
GL172821.1	277074	277623	277153, 277232, 277236	4, 13, 5	3.74195, 11.31501, 5.91431	3.32091, 6.98159, 4.53172	1.6222, 8.6	3	vrk1	651867
GL172821.1	384752	385179	384871, 385050, 384918	12, 15, 7	11.32055, 13.53022, 7.2501	7.18871, 7.87854, 5.35703	8.47391, 1	3	vrk1	544311
GL172821.1	530994	531235	531113, 531179, 531130	19, 28, 15	24.51879, 31.82951, 19.17716	13.28363, 14.77572, 11.09709	21.15031,	3	vrk1	398255
GL172821.1	975818	976379	976354, 976368, 976351	13, 33, 14	14.43911, 33.52883, 17.01463	8.83395, 13.83916, 10.0942	11.43182,	3	vrk1	30939
GL172821.1	1466686	1468229	1467389, 1467395, 1467490	19, 38, 22	19.25925, 38.66739, 22.81439	10.27754, 14.79682, 11.49162	16.05534,	3	glrx5	17769
GL172822.1	1558461	1558698	1558624, 1558692, 1558516	9, 12, 4	7.59348, 11.1491, 4.67325	5.40134, 7.08489, 3.78987	5.0069, 8.4	3	cidea	19270
GL172822.1	1591109	1591909	1591509, 1591478, 1591203	6, 12, 4	4.56865, 9.39099, 3.73221	3.81639, 5.99868, 3.31545	2.35429, 6	3	impa2	13438
GL172822.1	1592390	1592742	1592645, 1592653, 1592637	17, 23, 8	17.04639, 22.45275, 8.29845	9.50173, 10.94541, 5.91009	13.92543,	3	impa2	12605
GL172823.1	367024	367840	367308, 367552, 367546	11, 19, 6	6.5175, 11.3922, 4.15551	4.39789, 5.67906, 3.54969	4.03139, 8	3	st6galnac3	19927
GL172823.1	371414	372101	371682, 371823, 371540	14, 24, 13	9.0539, 16.16624, 11.09194	5.37869, 7.0817, 6.84382	6.35823, 1	3	st6galnac3	24317
GL172823.1	474976	476058	475623, 475618, 475351	11, 16, 5	10.78278, 14.46749, 4.84526	7.03733, 8.19898, 3.99806	7.97565, 1	3	msh4	0
GL172824.1	80798	81552	81275, 81243, 81212	8, 15, 7	7.19971, 15.19102, 8.69489	5.27799, 8.88488, 6.06379	4.63758, 1	3	trhr2	0
GL172824.1	499132	499387	499357, 499278, 499292	4, 6, 3	3.74195, 4.86158, 3.47371	3.32091, 4.00069, 3.03189	1.6222, 2.6	3	banp	204669
GL172824.1	567617	568012	567677, 567834, 567829	17, 26, 11	10.30167, 15.11015, 7.74126	5.48952, 6.13749, 5.16027	7.52113, 1	3	banp	136044
GL172824.1	574872	576080	575742, 575656, 575732	57, 91, 37	45.67545, 72.62214, 32.46902	11.80551, 13.00492, 11.78724	41.88656,	3	banp	127976
GL172824.1	1316388	1316679	1316456, 1316468, 1316424	11, 17, 13	11.49566, 16.3049, 16.38883	7.45067, 9.05325, 9.86104	8.64106, 1	3	fbxo31	0
GL172824.1	1489569	1490488	1490038, 1490055, 1489976	10, 19, 11	9.08301, 16.62257, 11.79996	6.17079, 8.68263, 7.62212	6.3862, 13	3	fam92b	7610
GL172825.1	871524	872785	871862, 871854, 871881	17, 29, 11	18.29581, 26.25787, 11.31465	10.24594, 11.13033, 7.3472	15.12565,	3	chd8	68282
GL172825.1	1253335	1253611	1253544, 1253575, 1253472	7, 9, 4	6.64707, 8.19918, 4.08034	5.02073, 5.77148, 3.50363	4.15135, 5	3	ASPH	0
GL172825.1	1488066	1488523	1488093, 1488108, 1488185	19, 42, 28	22.35011, 42.59225, 35.87059	12.09487, 15.37049, 17.13761	19.0463, 3	3	nkain3	0
GL172825.1	1735325	1735958	1735716, 1735822, 1735814	8, 12, 5	7.83097, 10.53798, 5.45388	5.64832, 6.71099, 4.3157	5.22917, 7	3	ythdf3	88913
GL172826.1	16111	17259	16473, 16812, 17106	5, 10, 8	4.33555, 7.35725, 9.31951	3.70555, 5.09584, 6.43944	2.15195, 4	3	NA	830
GL172826.1	47544	48206	47871, 47943, 47880	9, 36, 13	5.5514, 29.49433, 10.93492	4.10478, 10.58307, 6.74668	3.18174, 2	3	NA	1903
GL172826.1	130026	130547	130164, 130288, 130303	9, 18, 8	9.50098, 17.3898, 9.68495	6.52233, 9.43492, 6.61366	6.77745, 1	3	NA	21988
GL172826.1	246789	247388	247167, 247136, 247176	7, 7, 9	6.37715, 5.68499, 11.64359	4.86324, 4.44244, 7.57973	3.90257, 3	3	NA	1320
GL172826.1	376879	377185	376903, 376882, 376911	19, 27, 12	21.50921, 26.58221, 15.16114	11.61175, 12.02229, 9.30141	18.23049,	3	cd163	17307
GL172826.1	378965	379539	379017, 379381, 379386	7, 7, 3	5.7233, 4.6991, 3.2112	4.4663, 3.81136, 2.90793	3.33547, 2	3	cd163	14953
GL172826.1	886356	886847	886477, 886521, 886478	13, 22, 14	10.29845, 19.88511, 14.92875	6.35212, 9.73304, 8.94211	7.51866, 1	3	NA	83320
GL172826.1	958069	958778	958548, 958605, 958511	9, 9, 5	7.39636, 7.35794, 5.45388	5.27874, 5.25474, 4.3157	4.82212, 4	3	NA	75886
GL172826.1	1022685	1023115	1022916, 1022910, 1022863	9, 10, 4	8.88537, 9.22453, 4.11547	6.17591, 6.25634, 3.5218	6.19961, 6	3	NA	11549
GL172826.1	1083638	1084071	1083891, 1083796, 1083959	13, 9, 5	12.14841, 7.47261, 5.69847	7.49227, 5.32627, 4.4331	9.26135, 5	3	NA	0
GL172826.1	1107917	1108570	1108533, 1108563, 1108543	47, 62, 38	60.04374, 72.14668, 50.52606	23.13265, 22.58834, 21.72369	56.05474,	3	dtx3	4274
GL172826.1	1134104	1134710	1134143, 1134184, 1134164	6, 13, 8	5.16999, 11.31501, 8.80246	4.18963, 6.98159, 6.179	2.83325, 8	3	dtx3	30461
GL172826.1	1150036	1150998	1150960, 1150944, 1150834	17, 28, 17	16.23509, 27.76847, 19.29472	9.01086, 12.36122, 10.82557	13.14931,	3	dtx3	46393
GL172826.1	1483759	1484672	1483837, 1484379, 1483830, 1484410	9, 14, 6, 3	7.75911, 12.38816, 7.29403, 3.47371	5.5036, 7.41725, 5.30581, 3.03189	5.16246, 9	3	NA	9013
GL172826.1	1533941	1535886	1535763, 1535763, 1535763	21, 31, 12	20.24179, 29.20136, 12.15007	10.26221, 12.10186, 7.68238	17.00384,	3	NA	24151
GL172826.1	1867830	1869254	1869045, 1868379, 1868505	14, 21, 13	15.84929, 21.26419, 15.17843	9.46495, 10.87863, 9.23814	12.77923,	3	NA	1249
GL172827.1	20729	21692	21030, 21192, 21035	11, 14, 6	9.90875, 11.03811, 6.25896	6.51186, 6.58503, 4.80589	7.14919, 8	3	ralgds	93430
GL172827.1	87835	88251	88225, 88226, 88204	22, 34, 22	22.68783, 39.62623, 27.7028	11.41558, 17.02011, 14.34076	19.37324,	3	ralgds	26871
GL172827.1	95171	95611	95531, 95516, 95560	7, 13, 9	6.79162, 13.14514, 11.64359	5.10336, 8.09002, 7.57973	4.28406, 1	3	ralgds	19511
GL172827.1	113976	114722	114686, 114693, 114660	19, 39, 25	19.10984, 43.75036, 32.28849	10.1875, 17.30127, 16.06379	15.91125,	3	ralgds	400
GL172827.1	138435	138884	138823, 138844, 138759	13, 19, 13	13.34951, 18.82616, 14.77384	8.21021, 10.01625, 9.01902	10.39596,	3	ralgds	0
GL172827.1	161120	161622	161198, 161570, 161538	6, 6, 7	4.74339, 4.43108, 7.72582	3.92687, 3.72839, 5.6058	2.50328, 2	3	cel	0
GL172827.1	188149	188656	188594, 188588, 188606	23, 30, 14	20.38534, 26.13055, 13.12965	9.7143, 10.72189, 7.871	17.14354,	3	gtf3c5	2183
GL172827.1	210975	211334	211161, 211301, 211305	7, 13, 6	6.42049, 12.25946, 6.99807	4.8888, 7.55969, 5.17195	3.94161, 9	3	gfi1b	1851
GL172827.1	220402	221204	221105, 221073, 220471	9, 12, 4	7.88835, 10.93692, 4.67325	5.58287, 6.95571, 3.78987	5.28191, 8	3	gfi1b	11278
GL172827.1	226480	226990	226742, 226606, 226601	18, 20, 10	19.17347, 18.3522, 11.10454	10.50657, 9.43067, 7.32977	15.97256,	3	tsc1	11433
GL172827.1	240663	240991	240757, 240752, 240862	6, 7, 4	5.63898, 5.97725, 4.67325	4.46544, 4.62287, 3.78987	3.25772, 3	3	tsc1	0
GL172827.1	255527	255889	255800, 255779, 255757	10, 26, 9	9.18236, 27.92721, 10.42289	6.23091, 13.18442, 7.00726	6.47811, 2	3	tsc1	104
GL172827.1	340051	340700	340386, 340406, 340389	9, 11, 5	7.32038, 8.70225, 4.70718	5.23124, 5.76383, 3.9211	4.75116, 6	3	ak8	0
GL172827.1	354742	355559	355453, 354920, 354769, 355445	18, 17, 6, 9	14.92273, 12.44761, 5.20372, 8.26067	7.93921, 6.72068, 4.20995, 5.80842	11.89467,	3	gtf3c4	615
GL172827.1	356882	357434	356981, 356912, 357279	7, 14, 8	4.04197, 9.76095, 6.76479	3.37925, 5.80364, 5.01316	1.89531, 7	3	ddx31	0
GL172827.1	379241	379932	379734, 379873, 379902	6, 14, 11	5.55471, 10.76513, 10.66921	4.41698, 6.41704, 6.9701	3.18192, 8	3	ddx31	0
GL172827.1	398814	399250	399065, 399064, 399109	25, 34, 17	28.88915, 37.83902, 20.98465	14.10903, 15.96206, 11.76329	25.40799,	3	barhl1	8824
GL172827.1	407106	407677	407455, 407481, 407318	9, 12, 6	7.97705, 10.35, 5.46454	5.637, 6.59497, 4.36458	5.36577, 7	3	barhl1	397
GL172827.1	409323	410335	409874, 409898, 409953	16, 22, 26	16.01695, 21.85303, 32.88676	9.13983, 10.91337, 16.09579	12.9408, 1	3	barhl1	0
GL172827.1	429283	430312	429461, 429673, 429746	4, 12, 12	3.57366, 10.08092, 14.58408	3.22499, 6.42828, 9.01638	1.59706, 7	3	barhl1	11133
GL172827.1	457206	457742	457527, 457492, 457499	20, 29, 32	19.92129, 28.88285, 37.13674	10.37862, 12.64966, 16.36754	16.69414,	3	barhl1	39056
GL172827.1	472719	472937	472741, 472747, 472748	17, 20, 16	16.83467, 15.51138, 16.90298	9.37407, 7.74229, 9.66757	13.72157,	3	barhl1	54569
GL172827.1	545495	546184	545745, 545930, 545265, 545757	6, 8, 4, 5	5.90927, 7.07159, 4.01199, 5.14967	4.61743, 5.20073, 3.46784, 4.16141	3.50164, 4	3	setx	1637
GL172827.1	664893	665453	664927, 664932, 664923	21, 25, 7	24.45764, 24.44271, 8.35502	12.77293, 11.45691, 5.9108	21.09125,	3	NA	7905
GL172827.1	679426	679927	679561, 679874, 679738	5, 12, 6	4.54329, 10.93692, 6.99807	3.82752, 6.95571, 5.17195	2.33, 8.27	3	NA	22438
GL172827.1	715477	716030	715719, 715812, 715890	14, 19, 9	14.23954, 16.30954, 8.88966	8.53829, 8.49413, 6.17838	11.24094,	3	NA	58489
GL172827.1	789734	790805	789981, 789950, 790664	15, 19, 60	9.12594, 10.82275, 70.3591	5.22197, 5.37974, 22.58418	6.42585, 8	3	med27	30478
GL172827.1	789828	790818	790664, 790672, 790664	52, 75, 60	53.10556, 78.36253, 70.3591	16.91788, 19.94037, 22.58418	49.20938,	3	med27	30465
GL172827.1	794412	795001	794475, 794440, 794440	24, 39, 14	17.77616, 31.64366, 9.22157	7.93656, 10.73488, 5.4788	14.62794,	3	med27	26282
GL172827.1	824988	825555	825446, 825117, 825116	7, 11, 8	6.64707, 8.89461, 9.1831	5.02073, 5.88422, 6.37229	4.15135, 6	3	med27	0
GL172827.1	840153	840802	840392, 840396, 840309	28, 35, 14	31.18742, 36.56936, 15.06595	14.39356, 14.80726, 9.02131	27.6528, 3	3	med27	0
GL172827.1	846257	846803	846582, 846554, 846489	16, 41, 12	9.11594, 28.48351, 7.28881	5.02635, 8.78191, 4.69776	6.41768, 2	3	med27	0
GL172827.1	850538	851139	851123, 851090, 851111	87, 131, 78	99.27773, 147.44023, 97.89394	24.64037, 26.83543, 28.39677	94.89366,	3	med27	0
GL172827.1	885763	886526	886498, 886501, 886496	70, 93, 48	82.80698, 114.83063, 58.29453	24.40549, 29.3936, 21.61876	78.5834, 1	3	med27	0
GL172827.1	913736	914296	914239, 914226, 914203	34, 50, 30	36.98435, 51.85011, 33.57177	15.45808, 17.08547, 15.04586	33.33308,	3	med27	7026
GL172827.1	936925	937215	937065, 937059, 936979	12, 17, 8	10.07541, 14.91871, 8.4044	6.42486, 8.20906, 5.9678	7.30708, 1	3	rapgef1	34
GL172827.1	957071	957529	957467, 957503, 957477	29, 38, 27	35.13613, 42.05453, 34.37503	16.35598, 16.74533, 16.61898	31.52082,	3	rapgef1	0
GL172827.1	1021252	1021537	1021376, 1021412, 1021343	23, 12, 13	23.56695, 11.86108, 17.924	11.61446, 7.51216, 10.57401	20.22607,	3	uck1	0
GL172827.1	1033974	1034392	1034178, 1034153, 1034142	10, 7, 11	11.06011, 5.97725, 13.0311	7.306, 4.62287, 8.28053	8.22712, 3	3	uck1	0
GL172827.1	1047182	1047566	1047356, 1047327, 1047331	10, 15, 13	7.9709, 11.68429, 16.70862	5.48303, 6.74649, 10.0169	5.36088, 8	3	pomt1	137
GL172827.1	1108525	1108920	1108821, 1108644, 1108528	6, 5, 7	5.09949, 3.4564, 8.69489	4.14694, 3.15284, 6.06379	2.76939, 1	3	plpp7	1366
GL172827.1	1119086	1119857	1119392, 1119393, 1119366	66, 76, 41	77.33714, 84.312, 48.99221	23.4464, 22.35101, 19.47966	73.16787,	3	fam78a	1278
GL172827.1	1140791	1141670	1141255, 1141139, 1140865	10, 11, 8	8.57531, 8.85546, 8.35103	5.85982, 5.85974, 5.93881	5.90712, 6	3	fam78a	1567
GL172827.1	1148442	1148646	1148584, 1148635, 1148614	13, 18, 7	11.45737, 17.86551, 8.08824	7.06931, 9.72285, 5.7849	8.60415, 1	3	nup214	1604
GL172827.1	1230166	1230751	1230410, 1230440, 1230403	14, 28, 19	11.70599, 24.03743, 19.96565	6.99696, 10.21067, 10.70141	8.84105, 2	3	aif1l	479
GL172827.1	1234846	1235299	1235111, 1235199, 1235173	13, 28, 8	10.21914, 22.79611, 6.53931	6.30293, 9.52497, 4.87324	7.4427, 19	3	aif1l	0
GL172827.1	1244444	1244803	1244700, 1244701, 1244701	14, 25, 15	14.38232, 25.42729, 17.40815	8.62271, 12.04472, 10.16282	11.37721,	3	aif1l	0
GL172827.1	1260409	1260864	1260801, 1260636, 1260816	7, 15, 13	6.50909, 14.15433, 16.18511	4.94073, 8.25968, 9.7598	4.02371, 1	3	aif1l	0
GL172827.1	1360595	1361254	1361094, 1360829, 1360652	7, 11, 4	7.1696, 10.60895, 4.67325	5.31345, 6.9343, 3.78987	4.61046, 7	3	fibcd1	80798
GL172827.1	1393246	1394039	1393883, 1393392, 1393755	41, 77, 32	32.22717, 57.93756, 26.15748	10.42916, 11.45349, 10.0979	28.67087,	3	fibcd1	48013
GL172827.1	1403865	1404490	1404261, 1404316, 1404063	15, 11, 8	12.70921, 9.66539, 8.4044	7.37467, 6.36265, 5.9678	9.7865, 7.0	3	fibcd1	37562
GL172827.1	1465168	1465509	1465290, 1465375, 1465305	19, 37, 20	18.26836, 35.21534, 21.80741	9.67872, 13.26018, 11.50855	15.09918,	3	fibcd1	0
GL172827.1	1478046	1478945	1478433, 1478344, 1478617	12, 12, 7	13.38718, 11.84549, 8.08824	8.38496, 7.50292, 5.7849	10.43197,	3	fibcd1	2931
GL172827.1	1518515	1519189	1519040, 1518921, 1518994	13, 12, 5	12.03613, 10.12474, 4.84526	7.42394, 6.45547, 3.99806	9.15434, 7	3	qrfp	806
GL172827.1	1557717	1558537	1558378, 1558384, 1558400	54, 50, 44	56.96442, 48.87729, 50.71724	18.10294, 15.53378, 19.10898	53.01503,	3	abl1	0
GL172827.1	1564087	1565217	1564167, 1564213, 1564111	11, 17, 10	9.81032, 15.99248, 10.84317	6.45164, 8.86336, 7.18868	7.05644, 1	3	abl1	0
GL172827.1	1652623	1653204	1653093, 1653171, 1653169	9, 27, 18	7.97705, 27.11551, 20.32194	5.637, 12.33821, 11.17944	5.36577, 2	3	exosc2	0
GL172827.1	1657763	1658203	1657958, 1657955, 1657955	14, 21, 9	13.8308, 18.88456, 8.38703	8.29466, 9.44608, 5.88392	10.85567,	3	exosc2	0
GL172827.1	1665286	1665893	1665750, 1665533, 1665559	15, 17, 13	14.71973, 12.63113, 12.87254	8.60219, 6.8293, 7.92832	11.70031,	3	exosc2	441
GL172827.1	1676236	1676788	1676491, 1676411, 1676485	14, 24, 13	15.15267, 24.95217, 15.43582	9.07115, 12.10706, 9.3748	12.11465,	3	prdm12	0

GL172827.1	1758776	1759434	1758971, 1759260, 1759041	9, 19, 7	8.66407, 19.41322, 8.21885	6.04743, 10.37021, 5.84717	5.99101, 1	3	ass1	15964
GL172827.1	1815001	1815535	1815302, 1815214, 1815272	8, 9, 4	8.42257, 8.21099, 4.67325	5.97763, 5.77858, 3.78987	5.76336, 5	3	ass1	0
GL172827.1	1840448	1840787	1840600, 1840595, 1840616	18, 30, 12	18.92486, 30.81101, 13.90113	10.35878, 13.40848, 8.66246	15.73261,	3	hmcn2	0
GL172827.1	1897881	1898375	1898175, 1898098, 1898200	6, 13, 5	5.24248, 9.1776, 4.12265	4.2332, 5.65923, 3.57684	2.89962, 6	3	hmcn2	0
GL172827.1	1939834	1940549	1940434, 1940432, 1940361	9, 11, 4	8.94274, 9.30635, 4.38152	6.20889, 6.14075, 3.65448	6.2534, 6.7	3	ncs1	0
GL172827.1	1968616	1969603	1969172, 1969130, 1968876	8, 13, 7	7.1122, 12.14594, 6.83761	5.22529, 7.49077, 5.12939	4.57946, 9	3	ncs1	6831
GL172828.1	373112	374047	373440, 373433, 373507	11, 27, 12	10.96672, 25.38503, 11.50726	7.14544, 11.31845, 7.30112	8.14942, 2	3	pcdh11x	97117
GL172828.1	584151	584919	584451, 584462, 584533	6, 20, 10	5.95712, 21.17915, 11.83521	4.64377, 11.13464, 7.70798	3.52158, 1	3	NA	142003
GL172828.1	647717	648351	647910, 647989, 647982	13, 14, 12	12.87577, 13.26509, 12.62857	7.93025, 7.95332, 7.95947	9.9455, 10	3	NA	78571
GL172828.1	746757	747496	746911, 746920, 747403	17, 21, 7	16.90454, 19.00078, 6.79502	9.41624, 9.5157, 5.10529	13.78827,	3	NA	18583
GL172828.1	756385	756806	756668, 756659, 756666	13, 21, 12	11.55805, 18.82704, 12.56626	7.13125, 9.41165, 7.92374	8.70034, 1	3	NA	28211
GL172828.1	1131945	1132252	1132161, 1132158, 1132067	11, 14, 7	5.27859, 6.08626, 4.0371	3.6501, 3.70379, 3.37604	2.9335, 3.7	3	klhl4	291280
GL172828.1	1935921	1936356	1936301, 1936301, 1936302	15, 26, 13	16.36908, 29.08816, 15.17843	9.57629, 13.87718, 9.23814	13.27778,	3	dach2	91176
GL172829.1	822751	823521	823178, 823284, 823267	11, 13, 4	11.35776, 12.55412, 3.76128	7.37194, 7.73766, 3.33171	8.51017, 9	3	NA	12502
GL172829.1	875094	875614	875157, 875446, 875544	5, 10, 4	4.54329, 8.44678, 4.67325	3.82752, 5.78021, 3.78987	2.33, 5.932	3	NA	64845
GL172830.1	366357	367884	366689, 367628, 367187	10, 12, 5	10.89158, 11.48527, 5.9506	7.21503, 7.28792, 4.54784	8.0782, 8.8	3	trappc3	10227
GL172830.1	388678	389262	389004, 388939, 388903	9, 13, 5	9.24298, 11.51094, 5.85847	6.3792, 7.10228, 4.50665	6.53474, 8	3	trappc3	0
GL172830.1	1074574	1075159	1075103, 1075061, 1075024	10, 12, 5	8.84451, 8.6575, 5.03069	6.02544, 5.54124, 4.09861	6.16137, 6	3	cntn2	0
GL172830.1	1184818	1185460	1184938, 1185042, 1185326	6, 5, 8	5.24248, 3.58952, 8.56955	4.2332, 3.23987, 6.05652	2.89962, 1	3	mapk13	0
GL172831.1	2128	2450	2194, 2234, 2245	20, 16, 11	22.17215, 13.21822, 13.37278	11.72403, 7.43523, 8.45232	18.87307,	3	NA	37559
GL172831.1	9084	9723	9145, 9290, 9118	7, 10, 8	7.1696, 9.39128, 10.1465	5.31345, 6.35644, 6.82176	4.61046, 6	3	NA	30286
GL172831.1	14905	15872	15308, 15426, 15223	15, 11, 5	18.26603, 10.60895, 5.9506	10.62691, 6.9343, 4.54784	15.09729,	3	NA	24137
GL172831.1	53395	53893	53469, 53454, 53433	9, 14, 5	9.24298, 11.94813, 5.59755	6.3792, 7.1463, 4.38538	6.53474, 9	3	NA	8548
GL172831.1	73946	74612	74335, 74338, 74293	17, 36, 10	15.86203, 38.16755, 12.33934	8.78396, 15.32598, 7.95424	12.79136,	3	NA	29099
GL172831.1	100406	100833	100740, 100463, 100635	9, 12, 8	7.28297, 9.54462, 7.99854	5.20782, 6.09452, 5.74345	4.7171, 6.9	3	NA	55559
GL172831.1	111366	112442	111958, 111739, 112072	9, 11, 11	7.13706, 7.22061, 10.92742	5.11616, 4.83412, 7.12242	4.60345, 4	3	NA	66519
GL172831.1	113631	114085	113818, 113987, 113791	8, 7, 5	6.27633, 4.26316, 4.74091	4.70812, 3.5252, 3.94006	3.80887, 2	3	NA	68784
GL172831.1	129074	129340	129316, 129292, 129313	21, 31, 6	22.94132, 34.571, 5.75442	11.88295, 15.24545, 4.53101	19.61877,	3	NA	84227
GL172831.1	276936	277650	277125, 277563, 277329	10, 7, 7	10.13706, 5.07986, 6.6707	6.7935, 4.05845, 5.03432	7.36489, 2	3	NA	33681
GL172831.1	352089	352666	352109, 352097, 352128	17, 21, 7	17.4139, 19.00078, 8.08824	9.72241, 9.5157, 5.7849	14.27855,	3	NA	26903
GL172831.1	376375	377159	376905, 376525, 376559	13, 15, 7	15.29169, 14.98313, 8.69489	9.29854, 8.76054, 6.06379	12.24396,	3	NA	51189
GL172831.1	416890	417182	417151, 417144, 417127	46, 32, 32	55.85306, 32.1602, 39.43748	21.15985, 13.43212, 17.72718	51.91873,	3	NA	91704
GL172831.1	463074	463469	463206, 463205, 463205	19, 30, 13	20.05161, 31.41226, 15.09516	10.75274, 13.7635, 9.19347	16.81949,	3	NA	137888
GL172831.1	544367	545109	544779, 544744, 544908	6, 8, 4	5.3939, 6.4822, 4.67325	4.32314, 4.83755, 3.78987	3.0374, 4.1	3	NA	219181
GL172831.1	599456	600056	600041, 600014, 600026	41, 39, 33	49.86291, 45.59832, 43.44485	19.99249, 18.38522, 19.66452	46.01223,	3	5S_rRNA	190374
GL172831.1	624106	624763	624297, 624360, 624315	14, 15, 13	14.99044, 13.69427, 14.11158	8.97777, 7.97895, 8.64973	11.95856,	3	5S_rRNA	165667
GL172831.1	660270	661241	660885, 660570, 660629	14, 23, 11	12.8072, 23.11341, 12.06254	7.67424, 11.34202, 7.76744	9.88012, 1	3	5S_rRNA	129189
GL172831.1	763611	764050	763677, 763715, 763998	8, 7, 6	7.47668, 5.97725, 6.87953	5.44269, 4.62287, 5.11627	4.89713, 3	3	5S_rRNA	26380
GL172831.1	773875	774581	774522, 774486, 774374	15, 19, 11	14.09249, 16.25859, 11.6739	8.22203, 8.46351, 7.55148	11.1007, 1	3	5S_rRNA	15849
GL172831.1	780115	780740	780277, 780331, 780270	15, 16, 12	14.71973, 13.08342, 12.95194	8.60219, 7.35304, 8.14303	11.70031,	3	5S_rRNA	9690
GL172831.1	785053	785831	785318, 785249, 785268	17, 20, 13	17.19112, 17.88727, 14.32377	9.58879, 9.1506, 8.76942	14.0641, 1	3	5S_rRNA	4599
GL172831.1	826818	827268	827124, 827129, 826919	9, 10, 5	8.55802, 8.98028, 5.40789	5.98518, 6.10835, 4.29296	5.8909, 6.4	3	5S_rRNA	36271
GL172831.1	923413	923807	923745, 923640, 923741	8, 10, 7	8.42257, 8.16488, 7.20145	5.97763, 5.60458, 5.33074	5.76336, 5	3	U4	37988
GL172831.1	979961	981035	980691, 980756, 980677	14, 19, 13	14.16942, 17.46611, 14.0426	8.49669, 9.19261, 8.61056	11.17387,	3	U4	18046
GL172831.1	1088658	1089181	1088664, 1088675, 1088766	11, 21, 6	10.60582, 20.67581, 7.29403	6.93244, 10.52396, 5.30581	7.80831, 1	3	5S_rRNA	60239
GL172831.1	1134572	1135484	1135100, 1134787, 1135100	9, 10, 8	8.77314, 8.61706, 9.1831	6.111, 5.8856, 6.37229	6.09355, 6	3	igsf11	50938
GL172831.1	1169395	1169886	1169845, 1169857, 1169813	19, 38, 16	18.9632, 40.26984, 18.3512	10.09902, 15.71089, 10.50324	15.76968,	3	igsf11	16536
GL172831.1	1170289	1170645	1170363, 1170348, 1170336	12, 14, 10	10.07541, 10.15403, 9.93124	6.42486, 6.04265, 6.67475	7.30708, 7	3	igsf11	15777
GL172831.1	1203603	1204093	1203738, 1203993, 1203719	8, 11, 3	7.28955, 8.44639, 3.47371	5.33177, 5.60333, 3.03189	4.72176, 5	3	igsf11	10215
GL172831.1	1211054	1211723	1211593, 1211352, 1211268	19, 18, 11	19.25925, 15.09059, 11.6739	10.27754, 8.04034, 7.55148	16.05534,	3	igsf11	17666
GL172831.1	1226753	1227288	1226819, 1226789, 1226773	26, 45, 25	27.63895, 47.72259, 30.93278	13.01211, 16.9593, 15.29819	24.18761,	3	igsf11	33365
GL172831.1	1235601	1236158	1235619, 1235628, 1235628	20, 29, 17	19.56371, 28.20633, 20.09113	10.16265, 12.25351, 11.27497	16.34986,	3	igsf11	42213
GL172831.1	1266301	1266801	1266370, 1266425, 1266744	5, 9, 6	4.82177, 8.21099, 6.87953	3.98509, 5.77858, 5.11627	2.51418, 5	3	igsf11	72913
GL172831.1	1302866	1303482	1303431, 1303331, 1303434	11, 15, 15	10.00935, 11.96891, 17.07266	6.57321, 6.92051, 9.97618	7.24529, 9	3	igsf11	109478
GL172831.1	1367095	1367519	1367097, 1367147, 1367175	11, 18, 5	10.96672, 16.88096, 4.77515	7.14544, 9.12607, 3.9592	8.14942, 1	3	upk1b	72023
GL172831.1	1408211	1408706	1408665, 1408674, 1408676	92, 119, 99	103.63046, 132.03824, 123.79625	24.4507, 25.45389, 30.80794	99.20965,	3	upk1b	30836
GL172831.1	1409669	1410028	1409999, 1410000, 1409997	38, 40, 31	30.57629, 27.36424, 25.46505	10.51504, 8.54238, 10.04077	27.05598,	3	upk1b	29514
GL172831.1	1431509	1431968	1431610, 1431634, 1431658	15, 12, 11	16.9979, 11.86108, 13.83651	9.93422, 7.51216, 8.67735	13.87839,	3	upk1b	7574
GL172831.1	1487481	1488046	1487523, 1487507, 1487531	9, 19, 19	8.35434, 16.8936, 23.43977	5.86444, 8.84622, 12.70353	5.71584, 1	3	NA	1185
GL172831.1	1552886	1553454	1553428, 1553413, 1553367	22, 21, 18	23.53664, 19.11862, 19.60235	11.92457, 9.58634, 10.75987	20.19714,	3	b3gat1	27397
GL172831.1	1586570	1587080	1586943, 1586826, 1586860	8, 11, 5	7.24433, 8.74006, 4.02257	5.30475, 5.78751, 3.51512	4.68023, 6	3	b3gat1	0
GL172831.1	1607270	1607720	1607359, 1607488, 1607459	10, 25, 13	8.66311, 22.30103, 12.44575	5.914, 10.19404, 7.67237	5.99101, 1	3	b3gat1	0
GL172831.1	1608856	1610158	1609101, 1609691, 1609058	7, 14, 6	5.55587, 10.01976, 5.0532	4.36159, 5.96082, 4.11874	3.18254, 7	3	b3gat1	0
GL172831.1	1620823	1621160	1620964, 1620883, 1620932	7, 9, 12	7.1696, 8.21099, 16.28773	5.31345, 5.77858, 9.81872	4.61046, 5	3	b3gat1	0
GL172831.1	1666323	1666929	1666556, 1666707, 1666800	8, 6, 11	7.67464, 4.93312, 12.19945	5.55832, 4.04501, 7.8422	5.08299, 2	3	XB-GENE-988039	21792
GL172831.1	1722880	1723554	1723241, 1723238, 1723305	17, 27, 14	16.10852, 26.43443, 14.40949	8.93393, 11.93497, 8.63873	13.02838,	3	b4galt4	7010
GL172831.1	1734117	1734542	1734485, 1734443, 1734477	10, 12, 9	9.89241, 10.16897, 9.64095	6.65218, 6.4829, 6.59867	7.13431, 7	3	b4galt4	18247
GL172831.1	1735090	1735578	1735521, 1735548, 1735550	12, 21, 10	12.90199, 22.06021, 11.83521	8.11489, 11.35706, 7.70798	9.97033, 1	3	b4galt4	19220
GL172831.1	1823946	1824541	1824215, 1824237, 1824432	29, 33, 12	29.98561, 34.2076, 12.44386	13.3003, 14.23402, 7.85325	26.47844,	3	creg1	12396
GL172831.1	1857698	1858345	1857825, 1857702, 1858186	8, 10, 7	6.48409, 7.26311, 7.50542	4.83873, 5.03621, 5.49244	4.00135, 4	3	creg1	16472
GL172831.1	1911284	1911812	1911386, 1911590, 1911634	36, 42, 28	34.84755, 42.33944, 29.52789	13.43314, 15.23134, 13.40446	31.23749,	3	CD247	1056
GL172831.1	1922202	1922862	1922641, 1922640, 1922641	14, 26, 9	13.96392, 25.77124, 10.42289	8.37431, 11.89756, 7.00726	10.9769, 2	3	CD247	579
GL172832.1	28016	28230	28022, 28023, 28019	18, 27, 15	16.62541, 25.5193, 15.29861	8.97077, 11.39695, 8.94899	13.52454,	3	grm7	261595
GL172832.1	115543	116599	115895, 115894, 115918	27, 46, 28	26.92825, 48.39372, 33.71957	12.22714, 16.90325, 15.89276	23.4948, 4	3	grm7	173226
GL172832.1	462160	462578	462214, 462188, 462186	21, 30, 21	23.22463, 30.81101, 25.67865	12.05089, 13.40848, 13.46987	19.89343,	3	grm7	0
GL172832.1	471079	471406	471379, 471304, 471390	13, 22, 18	13.07382, 21.78003, 23.87427	8.04786, 10.86943, 13.11083	10.13353,	3	grm7	0
GL172832.1	574038	574595	574449, 574482, 574498	11, 12, 14	9.05039, 8.52999, 14.40949	5.98149, 5.4619, 8.63873	6.35485, 6	3	NA	2939
GL172832.1	604484	604896	604786, 604693, 604756	11, 13, 8	12.43693, 13.14514, 9.53432	7.97018, 8.09002, 6.54285	9.52692, 1	3	NA	26096
GL172832.1	629889	630986	630222, 630349, 630001	9, 10, 6	7.75911, 7.29424, 5.91225	5.5036, 5.05593, 4.61907	5.16246, 4	3	U2	18363
GL172832.1	695387	695628	695584, 695577, 695594	14, 24, 5	15.66744, 25.91323, 5.80393	9.36333, 12.68264, 4.48186	12.60415,	3	U2	45839
GL172832.1	979000	979405	979048, 979153, 979079	10, 12, 11	8.84451, 9.54462, 12.06254	6.02544, 6.09452, 7.76744	6.16137, 6	3	NA	37716
GL172832.1	1435558	1435975	1435799, 1435800, 1435822	9, 14, 6	9.24298, 11.94813, 7.12188	6.3792, 7.1463, 5.22884	6.53474, 9	3	thumpd3	23470
GL172832.1	1746972	1747232	1747160, 1747038, 1747195	6, 7, 8	5.24248, 5.36631, 9.31951	4.2332, 4.24177, 6.43944	2.89962, 3	3	tmcc1	2143
GL172833.1	255752	256429	256175, 255974, 255779	19, 15, 6	18.81924, 12.6757, 6.50067	10.01206, 7.35409, 4.93052	15.63044,	3	NA	0
GL172833.1	464650	465028	464828, 464869, 465004	6, 12, 6	4.32795, 8.56154, 6.5517	3.66187, 5.48152, 4.95623	2.1485, 6.0	3	mrps36	0
GL172833.1	1093670	1094410	1094338, 1093938, 1093897	10, 24, 10	7.7171, 20.47742, 9.93124	5.32329, 9.45458, 6.67475	5.12244, 1	3	map3k5	10736
GL172833.1	1824210	1824887	1824529, 1824497, 1824555	9, 9, 6	7.80169, 7.55109, 5.75442	5.52977, 5.37505, 4.53101	5.20141, 5	3	crot	1924
GL172834.1	34477	35377	35093, 35093, 34885	10, 7, 7	8.89113, 4.82021, 6.96907	6.05396, 3.89031, 5.20309	6.20456, 2	3	znf638	71245
GL172834.1	39454	40031	39666, 39711, 39744	6, 15, 9	4.80449, 12.09521, 7.98127	3.96512, 6.99786, 5.63957	2.51418, 9	3	znf638	66591
GL172834.1	61188	61800	61439, 61444, 61422	8, 12, 8	7.72597, 11.37069, 9.92329	5.588, 7.21896, 6.7228	5.13061, 8	3	znf638	44822
GL172834.1	140118	141103	140735, 140732, 140868	18, 17, 12	19.17347, 15.5773, 14.40488	10.50657, 8.61051, 8.92522	15.97256,	3	znf638	7122
GL172834.1	240801	241441	241123, 241097, 241097	8, 15, 15	7.67464, 14.7819, 18.33148	5.55832, 8.63964, 10.66149	5.08299, 1	3	cdc25b	23338
GL172834.1	256250	256698	256442, 256490, 256476	8, 7, 4	8.42257, 5.97725, 4.67325	5.97763, 4.62287, 3.78987	5.76336, 3	3	NA	16784
GL172834.1	290419	290683	290540, 290420, 290582	5, 6, 4	4.82177, 4.73925, 4.67325	3.98509, 3.92426, 3.78987	2.51418, 2	3	SPEF1	4071
GL172834.1	298631	299287	298975, 299068, 298994	10, 22, 14	8.20198, 20.6214, 14.53512	5.62778, 10.17315, 8.71263	5.57541, 1	3	SPEF1	0
GL172834.1	343189	343793	343746, 343766, 343738	54, 71, 35	62.5654, 78.61288, 41.74875	21.15723, 21.76064, 17.8555	58.54935,	3	hspa12b	2251
GL172834.1	418747	419111	418879, 418757, 418810	36, 44, 18	40.44396, 45.92243, 19.83478	16.65775, 16.38477, 10.89618	36.73464,	3	NA	18531
GL172834.1	475572	475988	475921, 475927, 475918	18, 25, 9	20.45461, 26.16839, 10.49552	11.25597, 12.4883, 7.0436	17.20992,	3	XB-GENE-5921670	10822
GL172834.1	510326	511011	510853, 510874, 510618	14, 11, 5	13.26657, 9.17879, 4.99237	7.95422, 6.06147, 4.0781	10.31735,	3	XB-GENE-5921670	11574
GL172834.1	608108	608444	608237, 608299, 608259	20, 24, 13	21.44017, 28.27809, 15.0131	11.29037, 14.08249, 9.14923	18.16397,	3	EBF4	5955
GL172834.1	616757	617415	617308, 616863, 616788, 617381	6, 8, 5, 5	5.47306, 7.07159, 5.03069, 5.23257	4.36955, 5.20073, 4.09861, 4.20435	3.10811, 4	3	EBF4	14604
GL172834.1	784752	785299	784893, 784913, 784860	6, 9, 4	5.96715, 8.19918, 4.26291	4.64927, 5.77148, 3.59641	3.52158, 5	3	pced1a	14706
GL172834.1	792673	793250	793212, 793223, 793180	58, 78, 64	66.98044, 91.92579, 81.74525	21.69131, 25.24106, 26.92824	62.91546,	3	pced1a	6755
GL172834.1	795045	795624	795451, 795574, 795561	16, 18, 12	11.52409, 11.63499, 8.62398	6.41223, 6.02146, 5.52037	8.66865, 8	3	pced1a	4381
GL172834.1	879678	880424	880235, 879967, 879925	16, 21, 14	14.34724, 18.94247, 14.86131	8.12546, 9.48076, 8.90303	11.34358,	3	adam33	0
GL172834.1	895480	896189	895960, 895595, 895672	17, 18, 15	18.1272, 17.19487, 18.13606	10.1466, 9.31668, 10.55788	14.96605,	3	adam33	7008
GL172834.1	908509	908772	908741, 908751, 908736	6, 12, 11	5.24248, 10.12474, 12.78973	4.2332, 6.45547, 8.15619	2.89962, 7	3	dctn1	3849
GL172834.1	1058246	1059119	1058620, 1058648, 1058493	9, 11, 8	8.35434, 10.60895, 9.8421	5.86444, 6.9343, 6.68602	5.71584, 7	3	MGC108497	5418
GL172834.1	1148763	1149481	1149124, 1149266, 1149246	10, 14, 7	9.95227, 11.99537, 8.69489	6.68696, 7.17543, 6.06379	7.19087, 9	3	fbxo41	0
GL172834.1	1207505	1207918	1207690, 1207542, 1207797	10, 8, 7	11.06011, 6.73981, 8.21885	7.306, 4.99774, 5.84717	8.22712, 4	3	NA	927
GL172834.1	1250334	1250858	1250499, 1250506, 1250531	14, 12, 9	16.23025, 11.1491, 10.56949	9.67496, 7.08489, 7.08032	13.14538,	3	not	21504
GL172834.1	1285394	1286469	1286001, 1285516, 1286166, 1286252	12, 8, 11, 14	8.23743, 3.77274, 5.8983, 12.83731	5.28029, 3.09457, 4.02011, 7.69265	5.6081, 1.8	3	rab11fip5	18615
GL172834.1	1286639	1287207	1287062, 1287065, 1287028	20, 30, 9	16.79976, 25.04076, 7.18747	8.50001, 10.12521, 5.14788	13.68819,	3	rab11fip5	17877
GL172834.1	1393524	1394229	1393808, 1393672, 1393792	19, 22, 13	19.56679, 21.92663, 13.21599	10.46249, 10.95766, 8.13179	16.35231,	3	sfxn5	0
GL172834.1	1401191	1401397	1401194, 1401215, 1401238	11, 23, 10	10.66406, 22.96257, 9.93124	6.96705, 11.25142, 6.67475	7.86396, 1	3	sfxn5	603
GL172834.1	1469419	1470065	1469727, 1469486, 1469687	5, 10, 5	4.82177, 8.98028, 5.9506	3.98509, 6.10835, 4.54784	2.51418, 6	3	emx1.2	15844
GL172834.1	1644229	1644610	1644395, 1644408, 1644369	7, 14, 7	7.1696, 14.45897, 8.69489	5.31345, 8.66788, 6.06379	4.61046, 1	3	NA	20495
GL172834.1	1685969	1686809	1686511, 1686427, 1686560	23, 36, 16	23.40734, 34.47503, 17.2553	11.51858, 13.22577, 9.87431	20.07125,	3	NA	9321
GL172834.1	1792136	1792611	1792156, 1792197, 1792195	10, 14, 4	9.13239, 12.33756, 4.67325	6.2007, 7.38613, 3.78987	6.43114, 9	3	NA	0
GL172834.1	1893415	1895329	1895114, 1894109, 1894096	8, 14, 8	7.02691, 13.69481, 9.3897	5.17362, 8.21301, 6.47354	4.50222, 1	3	NA	16853
GL172835.1	379311	380114	379772, 379710, 379720	15, 15, 6	15.19946, 14.71623, 7.05929	8.88991, 8.60008, 5.20024	12.15964,	3	sh3yl1	12841
GL172835.1	397968	398597	398296, 398283, 398112	8, 13, 12	7.67464, 13.14514, 14.58408	5.55832, 8.09002, 9.01638	5.08299, 1	3	sh3yl1	0
GL172835.1	766161	766913	766413, 766367, 766554	6, 9, 8	5.96715, 7.17446, 9.31951	4.64927, 5.1397, 6.43944	3.52158, 4	3	cep19	126289
GL172835.1	924754	925550	925194, 924971, 924953	9, 31, 13	9.70623, 35.32126, 15.70541	6.63396, 15.69126, 9.51556	6.97147, 3	3	SNTG2	0
GL172835.1	1139651	1139943	1139686, 1139675, 1139866	5, 9, 3	4.82177, 7.83963, 3.47371	3.98509, 5.55305, 3.03189	2.51418, 5	3	pxdn	22494
GL172835.1	1157049	1157409	1157074, 1157051, 1157116	13, 20, 10	12.43975, 20.00323, 11.38247	7.66875, 10.42807, 7.47651	9.5289, 16	3	pxdn	5028
GL172835.1	1304762	1306136	1305446, 1305189, 1305330	14, 9, 11	14.10012, 6.31893, 11.25734	8.4555, 4.5963, 7.31423	11.10747,	3	myt1l	0
GL172835.1	1311604	1312858	1312497, 1311738, 1311838	6, 12, 5	5.47306, 9.42893, 5.27517	4.36955, 6.02236, 4.22616	3.10811, 6	3	myt1l	0
GL172835.1	1468777	1469134	1469075, 1469069, 1469084	11, 13, 10	11.56615, 13.14514, 12.90749	7.49066, 8.09002, 8.21675	8.70729, 1	3	myt1l	115193
GL172835.1	1561176	1562196	1562027, 1562027, 1562011	42, 66, 24	46.95799, 73.92887, 27.34926	17.83946, 21.64262, 13.53677	43.14999,	3	tssc1	160384
GL172835.1	1603118	1604057	1603407, 1603145, 1603157	18, 26, 14	15.31233, 21.8089, 14.16595	8.1742, 9.59281, 8.49463	12.26404,	3	tssc1	118523
GL172835.1	1607834	1608288	1607861, 1607838, 1607867	22, 40, 25	21.1268, 40.00639, 28.64844	10.47651, 14.74539, 13.96687	17.86131,	3	tssc1	114292
GL172836.1	784487	785043	784569, 784593, 784639	8, 9, 7	8.10827, 8.21099, 8.69489	5.80497, 5.77858, 6.06379	5.48761, 5	3	NA	27493
GL172836.1	1781809	1782256	1782102, 1782102, 1782102	24, 18, 8	26.2838, 16.69883, 9.92329	12.90395, 9.01539, 6.7228	22.86648,	3	stx7	18605
GL172836.1	1795570	1798102	1795765, 1796225, 1795842, 1797723	13, 13, 6, 9	11.76528, 9.14598, 5.14244, 8.84115	7.25843, 5.63981, 4.17298, 6.1504	8.8968, 6.5	3	stx7	32366
GL172836.1	1795570	1798102	1797142, 1796225, 1795842, 1797723	9, 13, 6, 9	7.32038, 9.14598, 5.14244, 8.84115	5.23124, 5.63981, 4.17298, 6.1504	4.75116, 6	3	stx7	32366
GL172836.1	1812310	1814238	1812679, 1813764, 1812687	6, 15, 7	5.96715, 13.58442, 8.35502	4.64927, 7.91173, 5.9108	3.52158, 1	3	stx7	49106
GL172836.1	1812310	1814238	1813714, 1813764, 1812687	5, 15, 7	4.40263, 13.58442, 8.35502	3.74533, 7.91173, 5.9108	2.21004, 1	3	stx7	49106
GL172836.1	1818314	1819281	1819062, 1818893, 1818447, 1819108	10, 13, 7, 7	6.2925, 7.46083, 5.27202, 5.17296	4.4216, 4.62414, 4.18171, 4.1183	3.82365, 5	3	stx7	55110
GL172836.1	1820588	1821367	1820754, 1821123, 1820665	13, 16, 10	8.70126, 10.74003, 8.43366	5.36772, 5.94987, 5.77207	6.02696, 8	3	stx7	57384
GL172836.1	1831661	1833587	1833308, 1832343, 1832352, 1833206	14, 26, 11, 11	10.20119, 17.99636, 8.00985, 8.35618	6.07143, 7.53907, 5.32902, 5.54667	7.42678, 1	3	stx7	68457
GL172836.1	1834567	1834877	1834698, 1834763, 1834764	9, 16, 8	5.71387, 9.06166, 5.74302	4.20878, 4.99642, 4.36815	3.32765, 6	3	stx7	71363
GL172836.1	1841871	1844659	1842508, 1842059, 1843386, 1844498, 1842181, 1843272, 1844447	15, 11, 16, 14, 8, 10, 13	11.13, 6.96322, 13.21822, 11.03811, 5.94364, 8.32728, 11.86258	6.40927, 4.67372, 7.43523, 6.58503, 4.49672, 5.70592, 7.31799	8.29395, 4	3	stx7	78667
GL172836.1	1850610	1852169	1851554, 1850937, 1851999, 1851103	13, 15, 8, 12	10.46082, 12.13791, 4.75414, 11.50726	6.45284, 7.02404, 3.72794, 7.30112	7.67165, 9	3	stx7	87406
GL172836.1	1858687	1859537	1858958, 1859054, 1858978	7, 11, 5	6.09019, 9.8562, 4.64121	4.69155, 6.47973, 3.88372	3.63504, 7	3	stx7	95483
GL172836.1	1858687	1859537	1859390, 1859054, 1858978	8, 11, 5	7.19971, 9.8562, 4.64121	5.27799, 6.47973, 3.88372	4.63758, 7	3	stx7	95483
GL172836.1	1865308	1866216	1865675, 1865646, 1865960	17, 13, 8	15.28145, 11.71436, 9.1831	8.43017, 7.22722, 6.37229	12.23845,	3	stx7	102104
GL172836.1	1867178	1868237	1867876, 1868002, 1867988	15, 18, 9	11.74431, 15.69186, 9.24728	6.78315, 8.4039, 6.38161	8.87678, 1	3	stx7	103974
GL172836.1	1872527	1873622	1872650, 1873332, 1873329	12, 16, 15	12.3187, 17.16856, 18.95532	7.78073, 9.82359, 10.98488	9.4231, 14	3	stx7	109323
GL172838.1	152921	154335	153647, 153827, 153818	45, 65, 40	44.95473, 60.16244, 43.42818	15.44671, 15.45159, 16.68019	41.17651,	3	shb	55093
GL172838.1	899162	900599	899550, 900270, 899558	15, 21, 9	14.58913, 18.71315, 8.51771	8.52337, 9.34355, 5.96141	11.57535,	3	NA	143
GL172838.1	1435149	1435979	1435161, 1435288, 1435706	8, 13, 4	5.27754, 8.72347, 3.91395	4.06763, 5.38125, 3.41551	2.93292, 6	3	cilp2	31217
GL172838.1	1435149	1436013	1435910, 1435288, 1435706	11, 13, 4	7.83732, 8.72347, 3.91395	5.2206, 5.38125, 3.41551	5.23542, 6	3	cilp2	31217
GL172838.1	1436851	1437603	1437429, 1437089, 1437204	10, 13, 5	6.92459, 8.90011, 5.06966	4.82163, 5.4891, 4.11933	4.40864, 6	3	cilp2	32919
GL172838.1	1733240	1735104	1733722, 1733841, 1733815	18, 14, 9	16.80745, 11.45303, 9.64095	9.0814, 6.84088, 6.59867	13.69549,	3	trabd2a	33789
GL172839.1	297071	297695	297543, 297192, 297563	9, 8, 6	9.12009, 6.34359, 7.25145	6.30997, 4.75053, 5.287	6.42028, 3	3	ap4e1	1899
GL172839.1	918682	919081	919009, 919054, 918991	17, 14, 11	16.10852, 10.32673, 11.6739	8.93393, 6.14815, 7.55148	13.02838,	3	fam214a	26374
GL172839.1	922101	922595	922169, 922134, 922134	15, 27, 19	13.8583, 26.0026, 22.81433	8.07921, 11.68048, 12.35676	10.87599,	3	fam214a	29793
GL172839.1	1099505	1099965	1099863, 1099921, 1099921	7, 16, 13	6.2923, 16.74813, 16.93213	4.81292, 9.57609, 10.12358	3.8235, 13	3	onecut1	118316
GL172840.1	307749	308224	307966, 307972, 307997	7, 7, 5	7.1696, 5.97725, 5.9506	5.31345, 4.62287, 4.54784	4.61046, 3	3	lrrc20	48674
GL172841.1	52483	52857	52685, 52714, 52580	10, 9, 5	8.93828, 6.49199, 5.06966	6.08275, 4.70689, 4.11933	6.24946, 4	3	slc4a11	0
GL172841.1	291733	292583	292069, 292389, 291804	13, 13, 6	15.29169, 13.14514, 7.05929	9.29854, 8.09002, 5.20024	12.24396,	3	c20orf194	34197
GL172841.1	445564	446145	446018, 446091, 446081	4, 17, 12	3.51298, 16.17826, 12.69164	3.1896, 8.97632, 7.99552	1.55088, 1	3	gfra4	3641
GL172841.1	496710	496963	496919, 496784, 496917	9, 7, 7	9.63662, 5.97725, 8.21885	6.59633, 4.62287, 5.84717	6.90556, 3	3	gfra4	22486
GL172841.1	497673	498072	497707, 497722, 497731	9, 11, 10	9.70623, 10.59463, 11.83521	6.63396, 6.92577, 7.70798	6.97147, 7	3	gfra4	23449
GL172841.1	562382	562793	562498, 562421, 562518	10, 15, 3	9.44155, 13.92002, 3.47371	6.38646, 8.11689, 3.03189	6.72156, 1	3	gfra4	88158
GL172841.1	637624	638290	638256, 638264, 638264	51, 59, 61	60.87634, 70.60195, 81.58612	21.65055, 23.21454, 28.46405	56.87875,	3	gfra4	163400
GL172841.1	643749	645573	644637, 644888, 644638	9, 16, 7	7.75911, 14.74902, 8.69489	5.5036, 8.37095, 6.06379	5.16246, 1	3	gfra4	169525
GL172841.1	643749	645573	645250, 644888, 644638	6, 16, 7	4.37941, 14.74902, 8.69489	3.69512, 8.37095, 6.06379	2.19155, 1	3	gfra4	169525
GL172841.1	664799	665106	665016, 664956, 664997	6, 7, 5	5.3939, 5.93105, 5.91431	4.32314, 4.59459, 4.53172	3.0374, 3.6	3	gfra4	190575
GL172841.1	724206	724980	724220, 724872, 724600	6, 10, 3	5.43318, 9.39128, 3.47371	4.34622, 6.35644, 3.03189	3.07284, 6	3	gfra4	249982
GL172841.1	777251	777650	777459, 777460, 777429	10, 12, 9	9.08301, 8.88945, 8.47365	6.17079, 5.68576, 5.93535	6.3862, 6.3	3	gfra4	303027
GL172841.1	797277	797553	797450, 797385, 797383	7, 10, 7	5.82861, 6.82398, 5.88867	4.53157, 4.75785, 4.56857	3.42929, 4	3	gfra4	323053
GL172841.1	811456	811848	811697, 811697, 811697	29, 32, 19	35.67035, 37.40523, 25.38515	16.66859, 16.52681, 13.7311	32.04342,	3	gfra4	337232
GL172841.1	952001	952647	952095, 952085, 952226	55, 69, 31	61.5852, 71.3102, 37.39912	20.14256, 19.04733, 16.92224	57.58014,	3	NA	241873
GL172841.1	968112	968435	968136, 968114, 968216	7, 14, 6	6.60038, 14.02228, 6.50067	4.99378, 8.40914, 4.93052	4.10761, 1	3	NA	226085
GL172841.1	1064822	1066170	1064981, 1064906, 1065639	7, 19, 11	5.24783, 16.10778, 10.66921	4.16625, 8.37295, 6.9701	2.90454, 1	3	NA	128350
GL172841.1	1064822	1066170	1065296, 1064906, 1065639	6, 19, 11	4.74339, 16.10778, 10.66921	3.92687, 8.37295, 6.9701	2.50328, 1	3	NA	128350
GL172841.1	1117319	1117851	1117591, 1117802, 1117689	7, 12, 8	7.1696, 11.31437, 8.56955	5.31345, 7.18497, 6.05652	4.61046, 8	3	NA	76669
GL172841.1	1125270	1126308	1125509, 1125645, 1125426	11, 12, 10	10.1122, 10.68384, 11.75646	6.63573, 6.80072, 7.66841	7.34229, 8	3	NA	68212
GL172841.1	1165107	1165684	1165519, 1165321, 1165342	8, 11, 6	6.59314, 9.39347, 5.8719	4.90679, 6.19476, 4.59674	4.10249, 6	3	NA	28836
GL172841.1	1190329	1191066	1190637, 1190735, 1190415	6, 21, 5	5.96715, 18.32622, 5.06966	4.64927, 9.11275, 4.11933	3.52158, 1	3	NA	3454
GL172841.1	1218706	1219920	1219673, 1219672, 1219671	7, 9, 8	5.10557, 5.79773, 7.11599	4.075, 4.26249, 5.22758	2.77482, 3	3	NA	24095
GL172841.1	1220673	1221643	1221150, 1221185, 1221195	16, 20, 11	15.67432, 18.05851, 10.52088	8.9333, 9.25366, 6.8818	12.61068,	3	NA	26062
GL172841.1	1263730	1264220	1264129, 1264192, 1264193	22, 34, 34	26.19473, 36.34578, 44.68198	13.48821, 15.08303, 19.98751	22.77902,	3	NA	69119
GL172841.1	1286461	1287047	1287018, 1286954, 1286938	12, 11, 7	13.22036, 10.60895, 8.646	8.29296, 6.9343, 6.04229	10.27237,	3	NA	91850
GL172841.1	1334477	1335211	1334805, 1334771, 1335036	5, 12, 7	4.40263, 9.31599, 7.40077	3.74533, 5.95189, 5.43746	2.21004, 6	3	NA	67805
GL172841.1	1389925	1390406	1390370, 1390356, 1390194	7, 7, 6	7.1696, 5.97725, 7.29403	5.31345, 4.62287, 5.30581	4.61046, 3	3	NA	12610
GL172841.1	1396077	1396615	1396118, 1396520, 1396479	13, 20, 7	15.29169, 22.06591, 8.57075	9.29854, 11.6614, 6.00887	12.24396,	3	NA	6401
GL172841.1	1509676	1510349	1509686, 1509706, 1509716	20, 25, 11	19.84853, 25.50707, 12.9493	10.3347, 12.09244, 8.23866	16.62382,	3	avp	11214
GL172841.1	1520465	1520856	1520828, 1520702, 1520737	14, 22, 7	15.84929, 22.77797, 7.96278	9.46495, 11.46975, 5.72394	12.77923,	3	avp	707
GL172841.1	1553193	1553683	1553654, 1553646, 1553643	9, 27, 11	9.36986, 28.70654, 13.28509	6.44997, 13.28584, 8.40871	6.65425, 2	3	gnrh2	1301
GL172841.1	1560838	1561313	1561152, 1561152, 1561146	16, 17, 17	17.41877, 18.56115, 21.30828	9.96955, 10.40145, 11.93561	14.28292,	3	gnrh2	8946
GL172841.1	1574558	1575234	1575181, 1575179, 1575108	13, 21, 21	13.34951, 19.3593, 24.6582	8.21021, 9.73084, 12.88878	10.39596,	3	ptpra	7985
GL172841.1	1615507	1616019	1615834, 1615650, 1615715	10, 16, 7	7.41295, 13.35594, 5.88867	5.1311, 7.51928, 4.56857	4.8384, 10	3	ptpra	0
GL172841.1	1620834	1621642	1621275, 1621236, 1621152	23, 24, 12	21.54503, 24.46445, 11.60888	10.40228, 11.8146, 7.36202	18.26496,	3	ptpra	0
GL172841.1	1625900	1626267	1625919, 1625929, 1625934	11, 9, 10	7.33946, 6.26311, 9.31094	4.9084, 4.56059, 6.30832	4.7691, 3.9	3	ptpra	0
GL172841.1	1646416	1646880	1646609, 1646445, 1646564	12, 13, 4	11.09909, 10.43179, 4.59709	7.05451, 6.43483, 3.75554	8.26391, 7	3	ptpra	762
GL172841.1	1663595	1664053	1664026, 1664027, 1664027	63, 80, 62	79.17209, 94.40261, 83.65222	25.99528, 25.53258, 29.12004	74.98294,	3	NA	6332
GL172841.1	1768239	1768980	1768418, 1768327, 1768360	8, 14, 8	8.4095, 13.69481, 8.62637	5.97056, 8.21301, 6.08668	5.76336, 1	3	rnf24	0
GL172842.1	357841	358382	358028, 357994, 358030	13, 23, 6	12.94099, 21.44504, 7.29403	7.96907, 10.34265, 5.30581	10.00729,	3	zbtb2	11038
GL172842.1	861970	863659	862946, 862932, 862947	6, 9, 6	4.43199, 5.99677, 5.36363	3.72897, 4.39002, 4.30528	2.23764, 3	3	myct1	3273
GL172842.1	866479	867135	866967, 866903, 866818	9, 13, 7	8.25643, 11.61168, 7.05993	5.80588, 7.16421, 5.25341	5.62516, 8	3	myct1	7782
GL172842.1	880191	880801	880315, 880379, 880301	11, 8, 6	12.43693, 7.07159, 7.29403	7.97018, 5.20073, 5.30581	9.52692, 4	3	myct1	21494
GL172842.1	905209	906769	905603, 905752, 906077	7, 8, 10	6.42049, 5.14361, 9.31094	4.8888, 3.9808, 6.30832	3.94161, 2	3	myct1	46512
GL172842.1	905583	906769	906638, 906464, 906077	7, 10, 10	6.01305, 5.9851, 9.31094	4.6447, 4.22813, 6.30832	3.56347, 3	3	myct1	46886
GL172842.1	915688	916451	916373, 916004, 915910	10, 20, 9	8.12348, 18.2926, 8.17871	5.57869, 9.39473, 5.75915	5.50152, 1	3	myct1	56991
GL172842.1	920313	921854	920969, 921039, 920507	9, 13, 7	7.67537, 12.67661, 6.08239	5.45199, 7.81122, 4.68683	5.08317, 9	3	myct1	61616
GL172842.1	920313	921854	921481, 921039, 920507	8, 13, 7	7.28955, 12.67661, 6.08239	5.33177, 7.81122, 4.68683	4.72176, 9	3	myct1	61616
GL172842.1	926318	928451	926987, 927804, 927474, 928189	8, 15, 7, 4	8.28589, 15.33351, 8.08824, 4.30163	5.90321, 8.96975, 5.7849, 3.61556	5.65299, 1	3	myct1	67621
GL172842.1	935871	937478	936620, 937004, 937058	9, 16, 5	6.51482, 11.87373, 4.22765	4.72146, 6.6211, 3.64077	4.02888, 9	3	vip	69192
GL172842.1	939808	940573	940418, 940121, 940133	13, 13, 7	9.09436, 7.07909, 5.48123	5.60811, 4.40122, 4.31456	6.39638, 4	3	vip	66097
GL172842.1	942558	943898	943436, 942680, 943557	10, 15, 10	8.0085, 11.76421, 10.1418	5.50663, 6.79531, 6.79622	5.39412, 9	3	vip	62772
GL172842.1	953943	955166	954656, 954697, 954606	14, 25, 10	9.83335, 19.29692, 7.20496	5.84753, 8.49211, 4.99937	7.07841, 1	3	vip	51504
GL172842.1	957205	959048	957873, 957872, 957997, 958867	11, 16, 8, 8	7.9957, 11.24723, 6.73162, 7.30639	5.32012, 6.24794, 4.99268, 5.34182	5.38235, 8	3	vip	47622
GL172842.1	957374	959048	958350, 957872, 957997, 958867	8, 16, 8, 8	5.55847, 11.24723, 6.73162, 7.30639	4.24931, 6.24794, 4.99268, 5.34182	3.18478, 8	3	vip	47622
GL172842.1	1182446	1183345	1182854, 1183010, 1182976	7, 10, 6	5.97522, 8.0878, 6.93817	4.62162, 5.55635, 5.14396	3.52869, 5	3	gpatch11	3947
GL172842.1	1278449	1279148	1278639, 1278698, 1278717	13, 15, 9	12.94099, 13.74993, 11.04428	7.96907, 8.013, 7.30896	10.00729,	3	crim1	38977
GL172842.1	1820898	1821433	1821288, 1821287, 1821279	15, 21, 7	18.12902, 22.22847, 8.69489	10.55412, 11.45784, 6.06379	14.96737,	3	crim1	281002
GL172844.1	1061	2447	1470, 1981, 1783	25, 34, 25	8.47528, 9.51204, 12.04202	3.57244, 3.25959, 4.95348	5.81307, 6	3	adam23	60002
GL172844.1	3147	4918	3207, 4553, 4137	22, 38, 19	6.63926, 11.59195, 7.48536	3.15278, 3.56147, 3.77332	4.14446, 8	3	adam23	57531
GL172844.1	3325	4923	4415, 4553, 4137	26, 38, 19	8.96495, 11.59195, 7.48536	3.65797, 3.56147, 3.77332	6.27507, 8	3	adam23	57526

GL172844.1	5151	5829	5313, 5370, 5356	24, 40, 20	8.01375, 13.27035, 8.52904	3.49282, 3.85817, 4.11639	5.39933, 1	3	adam23	56620
GL172844.1	907164	907847	907415, 907402, 907370	17, 9, 7	14.06017, 5.72602, 6.59055	7.68709, 4.21655, 4.98809	11.06983,	3	pard3b	0
GL172844.1	1288441	1289094	1288869, 1288792, 1288627	11, 16, 5	9.43668, 12.73735, 4.39541	6.22151, 7.14249, 3.74107	6.7178, 9.9	3	pard3b	78109
GL172845.1	252125	252411	252358, 252381, 252361	16, 16, 12	18.05293, 17.16856, 14.23156	10.33439, 9.82359, 8.83588	14.8942, 1	3	NA	51007
GL172845.1	375160	375771	375417, 375390, 375384	18, 11, 5	20.16146, 10.60895, 5.9506	11.08652, 6.9343, 4.54784	16.9259, 7	3	NA	174042
GL172845.1	461669	462982	462273, 462117, 462275	15, 22, 12	17.18944, 24.56226, 13.59027	10.04146, 12.53481, 8.49571	14.06311,	3	NA	186864
GL172845.1	735956	737089	736524, 736463, 736481	13, 18, 12	11.16633, 14.80783, 13.15613	6.8898, 7.8701, 8.25729	8.32764, 1	3	NA	54619
GL172845.1	1323489	1323825	1323659, 1323658, 1323678	24, 34, 10	22.69279, 33.30058, 11.5283	10.75552, 13.32225, 7.55211	19.37782,	3	NA	0
GL172845.1	1364129	1364607	1364356, 1364320, 1364326	6, 12, 4	4.45871, 8.72259, 4.67325	3.74614, 5.58178, 3.78987	2.26108, 6	3	NA	16883
GL172845.1	1409134	1410600	1409441, 1410298, 1409813	6, 9, 3	4.7134, 7.59096, 3.24307	3.90801, 5.39978, 2.92359	2.47723, 5	3	erf	10924
GL172845.1	1409396	1410676	1410316, 1410298, 1409813	12, 9, 3	11.37748, 7.59096, 3.24307	7.22306, 5.39978, 2.92359	8.52862, 5	3	erf	10848
GL172846.1	471120	471555	471510, 471497, 471524	18, 33, 28	20.55486, 37.31986, 35.87059	11.31361, 16.06786, 17.13761	17.30766,	3	wscd2	18643
GL172846.1	552284	553038	552448, 552448, 552440	194, 286, 163	175.32063, 249.66647, 151.75987	18.25244, 17.69918, 18.92547	170.46242	3	cmklr1	20722
GL172846.1	889381	889804	889656, 889700, 889701	14, 17, 10	12.97522, 12.93592, 11.24131	7.77689, 7.01058, 7.40241	10.03988,	3	epb41l4a	92629
GL172846.1	915221	918266	916099, 917364, 916373	25, 26, 28	21.52286, 21.55462, 25.55005	9.74373, 9.4498, 11.06868	18.24348,	3	epb41l4a	64167
GL172846.1	925506	926366	926030, 925944, 925783	15, 16, 12	12.43022, 11.24723, 10.42593	7.20335, 6.24794, 6.64188	9.52692, 8	3	epb41l4a	56067
GL172846.1	927744	929321	928752, 927915, 928972	12, 18, 10	9.19354, 13.31424, 8.39789	5.87547, 6.983, 5.74985	6.48895, 1	3	epb41l4a	53112
GL172846.1	947939	949750	948447, 948631, 948628	10, 12, 12	8.40516, 9.74388, 12.38374	5.75437, 6.2187, 7.81847	5.76336, 7	3	epb41l4a	32683
GL172846.1	947939	949753	949405, 948631, 948628	9, 12, 12	7.32038, 9.74388, 12.38374	5.23124, 6.2187, 7.81847	4.75116, 7	3	epb41l4a	32680
GL172846.1	1008013	1008456	1008053, 1008065, 1008188	14, 15, 8	13.51064, 13.74993, 8.56955	8.10201, 8.013, 6.05652	10.55025,	3	epb41l4a	0
GL172846.1	1505651	1506081	1505705, 1505959, 1505943	13, 9, 5	11.26158, 6.73685, 5.50079	6.94861, 4.86289, 4.33868	8.41898, 4	3	ythdc2	40292
GL172847.1	74866	76262	75775, 75403, 75612	23, 25, 13	21.87577, 21.514, 14.5443	10.59983, 9.73863, 8.89247	18.58593,	3	exoc2	88378
GL172847.1	1032194	1032645	1032474, 1032416, 1032446	7, 14, 5	6.42049, 12.80997, 5.69847	4.8888, 7.67593, 4.4331	3.94161, 1	3	NA	24499
GL172847.1	1258985	1259580	1259159, 1259158, 1259153	11, 22, 9	11.93519, 22.38114, 9.82249	7.69727, 11.23117, 6.69629	9.0586, 19	3	NA	46528
GL172847.1	1487555	1488349	1487743, 1487849, 1488249	11, 14, 9	9.90875, 10.95872, 10.14484	6.51186, 6.53614, 6.86555	7.14919, 8	3	NA	128377
GL172847.1	1819621	1820182	1820031, 1819995, 1820021	11, 21, 7	10.78278, 19.60696, 6.75301	7.03733, 9.87975, 5.08141	7.97565, 1	3	F13A1	113672
GL172848.1	453467	453941	453649, 453692, 453596	9, 10, 5	7.32038, 8.0878, 4.99237	5.23124, 5.55635, 4.0781	4.75116, 5	3	zfyve27	81580
GL172848.1	455715	456526	455853, 456195, 456413	6, 10, 5	4.43199, 8.12616, 4.84526	3.72897, 5.58036, 3.99806	2.23764, 5	3	zfyve27	78995
GL172848.1	462048	462729	462416, 462432, 462383	7, 8, 6	7.1696, 7.07159, 6.99807	5.31345, 5.20073, 5.17195	4.61046, 4	3	zfyve27	72792
GL172848.1	563207	563961	563708, 563709, 563708	27, 44, 14	26.10728, 46.76728, 15.57248	11.74208, 16.85533, 9.30992	22.69489,	3	zfyve27	0
GL172848.1	908265	908814	908456, 908547, 908607	15, 20, 7	10.50889, 14.03125, 5.12471	6.03504, 6.89739, 4.08731	7.71745, 1	3	crtac1	0
GL172848.1	923483	924292	923970, 923859, 923803	10, 22, 8	10.39648, 19.65261, 8.1956	6.94096, 9.59468, 5.85348	7.61003, 1	3	crtac1	0
GL172848.1	959312	959995	959934, 959699, 959343	11, 17, 6	10.06049, 15.18554, 6.45058	6.60432, 8.37169, 4.90508	7.29334, 1	3	crtac1	0
GL172848.1	1499761	1500225	1500006, 1499936, 1500049	10, 8, 7	10.39648, 7.07159, 8.69489	6.94096, 5.20073, 6.06379	7.61003, 4	3	loxl4	0
GL172849.1	53056	54578	53957, 54053, 54287	15, 19, 8	13.57755, 15.12892, 8.09568	7.90752, 7.78953, 5.79794	10.61316,	3	NA	29992
GL172849.1	161221	162335	162030, 161916, 161793	24, 25, 20	25.36666, 23.54439, 23.83369	12.3555, 10.9238, 12.68602	21.97541,	3	sall3	70000
GL172849.1	992920	993216	992988, 992966, 992944	7, 9, 5	7.1696, 7.92635, 5.3628	5.31345, 5.60609, 4.27046	4.61046, 5	3	pqlc1.1	8657
GL172849.1	1773246	1775720	1774130, 1774033, 1774052	19, 28, 19	14.38566, 20.12319, 17.34669	7.35299, 8.11683, 9.12029	11.38042,	3	kiaa0319	1336
GL172849.1	1778740	1780103	1779011, 1779427, 1779427	21, 34, 18	16.49463, 28.16555, 16.56536	8.03746, 10.5239, 8.93428	13.39914,	3	kiaa0319	6830
GL172850.1	161265	162228	161614, 161660, 161484	16, 31, 15	17.95866, 33.28323, 18.53291	10.28064, 14.48135, 10.76715	14.80292,	3	NA	28951
GL172850.1	1786112	1787170	1786260, 1786556, 1786152	7, 24, 9	5.05064, 24.86921, 9.30116	4.03961, 12.05732, 6.41174	2.72521, 2	3	fam47e	15794
GL172850.1	1786114	1787267	1787116, 1786556, 1786152	15, 24, 9	14.27412, 24.86921, 9.30116	8.3325, 12.05732, 6.41174	11.27364,	3	fam47e	15796
GL172851.1	314967	315933	315139, 315420, 315191	8, 16, 7	8.42257, 15.35105, 7.61369	5.97763, 8.73748, 5.54854	5.76336, 1	3	bsn	19123
GL172851.1	535736	535947	535823, 535825, 535823	149, 157, 123	175.15192, 188.53117, 157.30403	30.37199, 32.23285, 34.66319	170.29442	3	bsn	82331
GL172851.1	1424252	1424816	1424604, 1424602, 1424382	15, 17, 13	17.90819, 18.56115, 16.70862	10.43573, 10.40145, 10.0169	14.75478,	3	NA	6257
GL172851.1	1518801	1519439	1519125, 1519047, 1519069	16, 19, 12	15.80927, 19.33741, 13.01912	9.01478, 10.3246, 8.18077	12.74061,	3	NA	1907
GL172851.1	1611673	1612174	1611905, 1611764, 1611841	6, 10, 3	5.96715, 9.39128, 3.2112	4.64927, 6.35644, 2.90793	3.52158, 6	3	tmprss2	0
GL172851.1	1672865	1673407	1673006, 1672986, 1672968	6, 18, 11	4.51306, 14.49153, 10.52088	3.78094, 7.68038, 6.8818	2.30616, 1	3	tmprss2	0
GL172852.1	118429	118798	118460, 118438, 118455	20, 40, 22	18.5756, 42.09175, 25.21195	9.56551, 15.91573, 12.91737	15.39539,	3	slc5a12	11235
GL172852.1	262769	263117	262819, 262791, 262858	14, 31, 16	13.03233, 31.199, 18.78921	7.81172, 13.2549, 10.74773	10.09457,	3	ano3	0
GL172852.1	274812	275690	275600, 275138, 275138, 275600	29, 20, 14, 14	21.52244, 10.86478, 10.07548, 10.07548	8.56513, 5.21824, 5.99476, 5.99476	18.24321,	3	NA	5338
GL172852.1	279005	279572	279535, 279502, 279506	49, 52, 19	46.278, 45.69223, 16.21962	14.59779, 13.28232, 8.4401	42.48023,	3	NA	9531
GL172852.1	315516	315903	315752, 315787, 315698	5, 8, 3	4.82177, 6.51779, 3.47371	3.98509, 4.8598, 3.03189	2.51418, 4	3	NA	46042
GL172852.1	613697	614201	614000, 614040, 613736	12, 32, 7	12.60209, 32.24067, 7.84208	7.9443, 13.47886, 5.66426	9.68421, 2	3	NA	232621
GL172852.1	749793	750103	749885, 749917, 749878	10, 13, 6	9.661, 11.41208, 6.08001	6.51661, 7.04141, 4.71063	6.9283, 8.7	3	NA	368717
GL172852.1	1120619	1120950	1120718, 1120690, 1120653	12, 24, 10	11.79509, 25.29108, 11.5283	7.47301, 12.31022, 7.55211	8.92495, 2	3	U4	203096
GL172852.1	1619004	1619415	1619354, 1619276, 1619163	9, 11, 3	9.12009, 8.97396, 3.47371	6.30997, 5.9338, 3.03189	6.42028, 6	3	SYT12	3758
GL172853.1	63986	64547	64450, 64166, 64407	21, 31, 19	20.66146, 30.72705, 21.51797	10.5153, 12.98008, 11.61684	17.41031,	3	NA	4044
GL172853.1	79875	80233	79877, 79876, 79904	76, 92, 66	92.35505, 109.69472, 84.50299	26.46792, 27.33334, 27.42218	88.03345,	3	NA	3107
GL172853.1	92662	93514	92861, 92854, 93450	11, 10, 6	7.17708, 7.1111, 5.36363	4.80695, 4.93988, 4.30528	4.61705, 4	3	NA	7952
GL172853.1	95120	95674	95360, 95336, 95360	13, 15, 9	9.31977, 12.49102, 8.60738	5.74669, 7.24068, 6.01421	6.60814, 9	3	NA	5792
GL172853.1	110011	110488	110154, 110208, 110111	5, 13, 5	4.82177, 13.06145, 5.9506	3.98509, 8.04054, 4.54784	2.51418, 1	3	NA	7227
GL172853.1	125602	126355	125861, 125629, 126241	7, 9, 8	6.12952, 7.06866, 9.92329	4.71533, 5.07306, 6.7228	3.67231, 4	3	st8sia5	0
GL172853.1	132401	132822	132496, 132621, 132620	38, 51, 25	37.77121, 53.45611, 28.64844	14.29313, 17.52049, 13.96687	34.10544,	3	st8sia5	0
GL172853.1	134644	135523	135148, 135093, 135068	14, 13, 8	8.32845, 7.11995, 6.21364	4.95118, 4.42492, 4.66848	5.69338, 4	3	st8sia5	0
GL172853.1	142342	142864	142790, 142803, 142794	23, 22, 17	23.09591, 18.08067, 19.46435	11.3315, 8.67028, 10.92232	19.76844,	3	st8sia5	0
GL172853.1	177188	178033	177682, 177682, 177682	40, 40, 26	47.48727, 43.84946, 35.23	19.0486, 16.92313, 17.39383	43.6706, 4	3	st8sia5	18563
GL172853.1	199559	200046	199660, 199772, 199850	8, 9, 9	7.28955, 6.76868, 9.52439	5.33177, 4.88312, 6.53516	4.72176, 4	3	NA	16085
GL172853.1	216503	216772	216528, 216536, 216546	11, 13, 5	10.06049, 12.67661, 5.64747	6.60432, 7.81122, 4.40911	7.29334, 9	3	NA	0
GL172853.1	222855	223271	222913, 222945, 223027	23, 22, 16	22.94393, 20.88093, 18.3512	11.24022, 10.32883, 10.50324	19.62118,	3	NA	0
GL172853.1	286101	286422	286391, 286399, 286322	24, 34, 12	22.96419, 34.65644, 13.29687	10.91709, 14.09951, 8.33527	19.64074,	3	RNF165	6846
GL172853.1	292530	293189	292766, 293072, 292768	5, 10, 8	4.14595, 8.4053, 7.95096	3.59111, 5.75445, 5.71659	1.98639, 5	3	RNF165	13275
GL172853.1	449563	450146	450046, 449808, 449808	8, 8, 8	6.20993, 6.11443, 8.04677	4.66614, 4.60556, 5.77057	3.74679, 3	3	PSTPIP2	0
GL172853.1	640633	640997	640966, 640949, 640906	20, 43, 26	19.99469, 46.40031, 27.95127	10.42292, 17.0803, 13.1988	16.76522,	3	ier3ip1	6691
GL172853.1	665127	666143	665470, 665358, 665471, 665919	8, 15, 7, 4	6.98506, 13.37045, 8.35502, 4.42274	5.14817, 7.78062, 5.9108, 3.67426	4.46457, 1	3	NA	0
GL172853.1	671749	672055	672007, 672016, 671984	14, 16, 13	15.23546, 15.41438, 16.0859	9.11858, 8.77591, 9.70995	12.19387,	3	NA	0
GL172853.1	706802	707235	706979, 706894, 706898	6, 12, 5	5.1345, 9.16947, 5.19074	4.16817, 5.86046, 4.18277	2.80161, 6	3	NA	3930
GL172853.1	708592	709023	708940, 708963, 708947	8, 15, 4	7.67464, 12.09521, 4.18775	5.55832, 6.99786, 3.55872	5.08299, 9	3	NA	2142
GL172853.1	769146	769909	769340, 769297, 769577	5, 8, 6	4.77263, 6.59014, 6.76586	3.9578, 4.90493, 5.06179	2.51418, 4	3	NA	57146
GL172853.1	792451	793039	792905, 792910, 793005	16, 25, 11	16.08763, 25.26956, 11.79996	9.18228, 11.95039, 7.62212	13.00852,	3	NA	80451
GL172853.1	803035	803410	803175, 803204, 803131	6, 14, 4	5.24248, 13.69481, 4.67325	4.2332, 8.21301, 3.78987	2.89962, 1	3	NA	91035
GL172853.1	829660	830235	829761, 830000, 830002	6, 11, 6	5.96715, 10.60895, 7.12188	4.64927, 6.9343, 5.22884	3.52158, 7	3	NA	117660
GL172853.1	936960	937533	937335, 937273, 937206	5, 9, 6	4.82177, 8.21099, 7.29403	3.98509, 5.77858, 5.30581	2.51418, 5	3	smad2	56849
GL172853.1	990730	991137	990927, 990903, 990937	10, 10, 5	10.96713, 8.9331, 5.59755	7.25596, 6.07959, 4.38538	8.14942, 6	3	smad2	3245
GL172853.1	1007162	1007633	1007557, 1007597, 1007435	12, 9, 5	11.2086, 6.8332, 5.80393	7.12098, 4.92409, 4.48186	8.36821, 4	3	smad2	0
GL172853.1	1049423	1049729	1049494, 1049571, 1049550	8, 11, 11	6.27633, 9.30635, 11.20072	4.70812, 6.14075, 7.28154	3.80887, 6	3	zbtb7c	1630
GL172853.1	1076566	1078052	1077648, 1076935, 1077253	8, 16, 4	6.63031, 14.92393, 4.46488	4.92991, 8.47764, 3.69425	4.13604, 1	3	zbtb7c	28773
GL172853.1	1108486	1108931	1108568, 1108567, 1108673	8, 7, 3	7.93925, 5.36631, 3.27564	5.70996, 4.24177, 2.93941	5.33002, 3	3	rpl17	38994
GL172853.1	1117290	1117714	1117402, 1117494, 1117646	7, 10, 8	6.33443, 8.24345, 8.35103	4.83795, 5.65367, 5.93881	3.86259, 5	3	rpl17	30211
GL172853.1	1133183	1133621	1133201, 1133192, 1133213	15, 18, 10	15.34351, 15.38496, 9.82971	8.97569, 8.2181, 6.61563	12.29352,	3	rpl17	14304
GL172853.1	1145682	1146589	1145898, 1146251, 1145802, 1146312	8, 5, 5, 4	7.28955, 3.47798, 4.45433, 3.43992	5.33177, 3.16702, 3.77574, 3.14645	4.72176, 1	3	rpl17	1336
GL172853.1	1169415	1170056	1169810, 1169766, 1169699	12, 17, 6	9.19354, 11.50636, 4.45771	5.87547, 6.17106, 3.74549	6.48895, 8	3	rpl17	15790
GL172853.1	1176410	1177644	1176938, 1176957, 1176972	19, 29, 27	19.96873, 30.18937, 31.35877	10.70325, 13.42104, 14.86484	16.74065,	3	c18orf32	12429
GL172853.1	1195240	1195731	1195403, 1195382, 1195405	6, 5, 3	4.93136, 3.73203, 3.37781	4.04392, 3.33183, 2.98791	2.61555, 1	3	c18orf32	0
GL172853.1	1203617	1203871	1203814, 1203832, 1203824	9, 18, 6	8.88537, 18.07902, 5.793	6.17591, 9.8517, 4.55271	6.19961, 1	3	dym	5195
GL172853.1	1208576	1208972	1208940, 1208925, 1208870	10, 13, 6	9.60506, 11.56107, 6.4014	6.48358, 7.13311, 4.8799	6.87658, 8	3	dym	94
GL172853.1	1244375	1244983	1244644, 1244603, 1244634	9, 8, 6	7.97705, 6.02101, 5.46454	5.637, 4.5461, 4.36458	5.36577, 3	3	dym	0
GL172853.1	1268516	1269165	1268595, 1268923, 1268759	23, 34, 18	22.64709, 33.07446, 19.30306	11.06201, 13.1939, 10.58333	19.33432,	3	dym	0
GL172853.1	1359423	1359931	1359806, 1359843, 1359615	12, 14, 13	9.6142, 12.04301, 12.98465	6.1379, 7.20479, 7.995	6.88539, 9	3	dym	0
GL172853.1	1370211	1370757	1370231, 1370402, 1370303	6, 6, 4	5.72604, 3.5975, 3.76128	4.51498, 3.18006, 3.33171	3.33547, 1	3	dym	0
GL172853.1	1401538	1402155	1401798, 1401797, 1401795	70, 99, 44	82.40413, 108.0126, 53.8559	24.19095, 23.45829, 20.94259	78.18543,	3	dym	22892
GL172853.1	1504139	1505032	1504605, 1504451, 1504785	16, 25, 5	16.99938, 25.42729, 5.45388	9.72433, 12.04472, 4.3157	13.87966,	3	smad7	15712
GL172853.1	1510433	1511042	1510711, 1510554, 1510754	8, 11, 11	7.67464, 10.3713, 14.24187	5.55832, 6.79219, 8.86619	5.08299, 7	3	smad7	22006
GL172853.1	1648119	1648581	1648297, 1648451, 1648400	8, 8, 6	6.78332, 6.11443, 6.168	5.02459, 4.60556, 4.75778	4.27811, 3	3	cfap53	2290
GL172853.1	1677402	1678254	1677865, 1677704, 1678093	11, 12, 4	12.43693, 11.86108, 4.67325	7.97018, 7.51216, 3.78987	9.52692, 9	3	cfap53	15279
GL172853.1	1698985	1699625	1699584, 1699601, 1699601	21, 26, 24	22.75736, 27.83582, 29.14213	11.77357, 13.1298, 14.58342	19.44135,	3	myo5b	8426
GL172853.1	1707156	1707917	1707701, 1707775, 1707314	10, 14, 5	8.93828, 13.44539, 5.9506	6.08275, 8.06258, 4.54784	6.24946, 1	3	myo5b	134
GL172853.1	1726432	1726870	1726843, 1726846, 1726830	15, 23, 7	13.5229, 21.70337, 8.69489	7.87405, 10.4968, 6.06379	10.56184,	3	myo5b	0
GL172853.1	1752172	1752623	1752466, 1752376, 1752451	5, 5, 5	4.82177, 3.94561, 5.9506	3.98509, 3.46715, 4.54784	2.51418, 1	3	myo5b	0
GL172853.1	1761067	1761447	1761260, 1761274, 1761259	16, 18, 11	16.45267, 17.00494, 13.0311	9.40062, 9.20137, 8.28053	13.35812,	3	myo5b	0
GL172854.1	110	1642	1264, 1436, 861	25, 36, 17	20.88821, 29.04054, 15.00847	9.38092, 10.35596, 8.26376	17.63061,	3	MPP4	28797
GL172854.1	7572	8593	7755, 7822, 8232	22, 25, 18	16.38143, 16.87462, 15.93182	7.70111, 7.20841, 8.54944	13.28981,	3	MPP4	21846
GL172854.1	1744451	1746377	1744531, 1744529, 1744530	208, 281, 159	163.67833, 224.24362, 125.35646	14.16002, 14.88117, 13.82383	158.87788	3	pofut2	28854
GL172854.1	1746649	1747001	1746894, 1746849, 1746910	126, 138, 89	75.20325, 73.69565, 50.69724	8.67009, 7.50717, 7.81069	71.0539, 6	3	pofut2	31052
GL172854.1	1755075	1756292	1755378, 1755383, 1755376	116, 146, 112	17.02278, 17.31636, 25.90628	2.42714, 2.20275, 3.22875	13.90267,	3	pofut2	39478
GL172854.1	1757569	1758740	1758501, 1758501, 1758501	1045, 1498, 813	685.70575, 1039.448, 554.31732	11.0831, 12.23653, 11.75762	679.68579	3	pofut2	41972
GL172854.1	1761746	1762775	1762492, 1762484, 1762492	1125, 1340, 782	796.08331, 898.50049, 539.95618	12.60863, 11.52884, 12.00173	789.71045	3	pofut2	46149
GL172855.1	57156	57391	57312, 57318, 57295	17, 24, 14	17.19112, 22.46073, 15.72539	9.58879, 10.61767, 9.39581	14.0641, 1	3	nars2	0
GL172855.1	201853	202589	202254, 202201, 202156	9, 11, 10	8.94274, 10.00475, 11.17239	6.20889, 6.57041, 7.36591	6.2534, 7.3	3	alg8	0
GL172855.1	244194	244794	244613, 244656, 244524	22, 24, 26	23.7162, 25.37763, 29.39413	12.03186, 12.36208, 14.05915	20.37115,	3	ints4	10325
GL172855.1	274314	274642	274617, 274612, 274579	8, 13, 10	7.57423, 13.14514, 13.18184	5.4999, 8.09002, 8.33771	4.98865, 1	3	rsf1	3737
GL172855.1	325592	326759	325877, 326359, 326370	13, 23, 9	12.09198, 19.76013, 10.14484	7.45795, 9.34744, 6.86555	9.20698, 1	3	hikeshi	0
GL172855.1	346642	347517	347140, 347126, 347133	40, 50, 42	43.28053, 51.85011, 53.72387	16.59525, 17.08547, 21.80524	39.52664,	3	ccdc81	0
GL172855.1	554140	554518	554276, 554282, 554326	11, 15, 4	11.56615, 15.33351, 4.55201	7.49066, 8.96975, 3.73489	8.70729, 1	3	prss23	0
GL172855.1	564549	565516	565252, 565112, 564562	13, 20, 15	15.29169, 22.87459, 19.40672	9.29854, 12.13503, 11.21161	12.24396,	3	prss23	0
GL172855.1	602524	603645	602804, 602801, 602741	13, 13, 10	12.32133, 12.37541, 11.83521	7.59717, 7.62989, 7.70798	9.42482, 9	3	prss23	28900
GL172855.1	619908	620491	620361, 619980, 620414	10, 19, 15	9.03421, 15.48693, 17.32264	6.14116, 8.00194, 10.11551	6.33915, 1	3	prss23	46284
GL172855.1	693888	694379	694061, 694115, 694155	7, 11, 5	6.12952, 9.66539, 5.14967	4.71533, 6.36265, 4.16141	3.67231, 7	3	fzd4	11461
GL172855.1	732043	732660	732195, 732450, 732145	7, 7, 4	5.49154, 4.89556, 4.42274	4.32107, 3.93927, 3.67426	3.12578, 2	3	NA	0
GL172855.1	765472	766147	765698, 765807, 765831	12, 30, 7	9.12091, 29.31274, 6.51247	5.83015, 12.53115, 4.9427	6.42097, 2	3	NA	9364
GL172855.1	775338	775933	775780, 775670, 775852	7, 17, 4	6.2923, 13.33786, 4.30163	4.81292, 7.25118, 3.61556	3.8235, 10	3	NA	19230
GL172855.1	814637	815337	814952, 814708, 815230	7, 5, 5	6.60038, 3.21429, 5.03069	4.99378, 2.99211, 4.09861	4.10761, 1	3	NA	58529
GL172855.1	870087	870622	870520, 870616, 870570	8, 12, 10	7.28955, 11.60251, 11.17239	5.33177, 7.3582, 7.36591	4.72176, 8	3	tmem135	17599
GL172855.1	1170525	1170927	1170697, 1170698, 1170697	12, 20, 7	11.85767, 21.17915, 8.35502	7.51014, 11.13464, 5.9108	8.98544, 1	3	grm5	0
GL172855.1	1256215	1257180	1256913, 1256678, 1256744	8, 19, 12	7.99469, 16.5695, 14.96175	5.74128, 8.65063, 9.20442	5.38155, 1	3	nox4	8552
GL172855.1	1519126	1519468	1519330, 1519225, 1519291	10, 11, 6	11.06011, 10.60895, 7.29403	7.306, 6.9343, 5.30581	8.22712, 7	3	chordc1	4353
GL172855.1	1537841	1538468	1538080, 1538252, 1538058	9, 13, 11	7.55321, 9.1776, 10.87462	5.37637, 5.65923, 7.09143	4.96981, 6	3	chordc1	23068
GL172855.1	1561823	1562102	1562058, 1562060, 1561988	7, 11, 4	7.10297, 9.7598, 4.67325	5.27706, 6.42066, 3.78987	4.57191, 7	3	chordc1	47050
GL172855.1	1602856	1603215	1602882, 1602979, 1602913	11, 9, 7	10.00935, 8.19918, 7.66926	6.57321, 5.77148, 5.57703	7.24529, 5	3	chordc1	88083
GL172855.1	1710685	1711501	1711003, 1711003, 1711011	11, 9, 9	10.9046, 7.55109, 9.19414	7.10903, 5.37505, 6.35176	8.08987, 5	3	chordc1	195912
GL172855.1	1747373	1747957	1747764, 1747623, 1747726	40, 44, 14	45.30856, 45.38035, 14.53512	17.77056, 16.0854, 8.71263	41.52534,	3	chordc1	232600
GL172856.1	35244	36212	35683, 35862, 35627	8, 15, 6	7.24433, 12.91479, 5.75442	5.30475, 7.50094, 4.53101	4.68023, 1	3	h6pd	20547
GL172856.1	620772	621595	621144, 621159, 621166	7, 6, 4	6.50909, 4.30294, 4.67325	4.94073, 3.64568, 3.78987	4.02371, 2	3	pex10	123758
GL172856.1	621122	621764	621607, 621607, 621166	5, 6, 4	4.43696, 4.45754, 4.67325	3.76555, 3.74538, 3.78987	2.24017, 2	3	pex10	123589
GL172856.1	869846	870960	869885, 870088, 869875, 870493	13, 17, 4, 5	13.94585, 18.56115, 4.67325, 5.9506	8.5554, 10.40145, 3.78987, 4.54784	10.95967,	3	morn1	27914
GL172856.1	1561459	1561795	1561502, 1561491, 1561569	10, 8, 4	11.06011, 5.43464, 4.38152	7.306, 4.16933, 3.65448	8.22712, 3	3	prkcz	40825
GL172856.1	1753382	1754872	1754307, 1754404, 1754403	21, 31, 25	22.84886, 31.60612, 28.01385	11.82801, 13.49298, 13.59055	19.52948,	3	gabrd	2184
GL172857.1	516	2220	1393, 1767, 1377	9, 10, 6	8.77314, 8.79469, 7.29403	6.111, 5.99491, 5.30581	6.09355, 6	3	NA	38521
GL172857.1	879	2220	1726, 1767, 1377	4, 10, 6	3.28906, 8.79469, 7.29403	3.0555, 5.99491, 5.30581	1.37521, 6	3	NA	38521
GL172857.1	130648	131357	130709, 130817, 131206	9, 12, 9	8.77314, 11.86108, 10.56949	6.111, 7.51216, 7.08032	6.09355, 9	3	NA	89393
GL172857.1	146718	147866	147226, 147114, 147148	8, 12, 10	8.10827, 11.86108, 11.6791	5.80497, 7.51216, 7.62925	5.48761, 9	3	NA	105463
GL172859.1	122515	123574	122766, 122348, 123435, 122725	5, 9, 9, 4	4.30277, 6.34719, 6.20816, 4.55201	3.68597, 4.61437, 4.52542, 3.73489	2.12436, 3	3	runx3	41988
GL172860.1	323448	323767	323572, 323626, 323683	8, 9, 4	7.57423, 7.21043, 4.08034	5.4999, 5.1623, 3.50363	4.98865, 4	3	NA	133942
GL172860.1	1454314	1454569	1454354, 1454358, 1454339	11, 15, 5	10.16451, 13.42325, 5.9506	6.66744, 7.81299, 4.54784	7.39114, 1	3	iws1	912
GL172861.1	523129	523496	523412, 523433, 523470	7, 9, 5	6.37715, 7.79702, 5.9506	4.86324, 5.5269, 4.54784	3.90257, 5	3	pkia	58009
GL172861.1	619259	620541	620204, 619583, 619796	7, 8, 10	6.64707, 6.97779, 11.10454	5.02073, 5.14375, 7.32977	4.15135, 4	3	pkia	0
GL172861.1	1574765	1576708	1575187, 1575967, 1575929	11, 22, 11	6.97015, 11.86398, 8.63544	4.67803, 5.35262, 5.72195	4.4505, 9.1	3	snx16	145852
GL172861.1	1574765	1576708	1576560, 1575967, 1575929	9, 22, 11	4.71777, 11.86398, 8.63544	3.57477, 5.35262, 5.72195	2.48153, 9	3	snx16	145852
GL172861.1	1581477	1582537	1581906, 1581896, 1581862	34, 26, 16	21.85431, 11.60274, 10.77196	7.51885, 4.63765, 5.96852	18.56514,	3	snx16	152564
GL172861.1	1583043	1584535	1583680, 1583266, 1582777, 1584367	15, 14, 8, 14	6.17489, 4.35617, 3.92617, 9.32102	3.62469, 2.83355, 3.19282, 5.53839	3.7142, 2.2	3	snx16	154130
GL172861.1	1583733	1585337	1584250, 1584168, 1584367	18, 19, 14	8.65636, 7.89497, 9.32102	4.45177, 3.95634, 5.53839	5.98572, 5	3	snx16	154820
GL172861.1	1591912	1593775	1593486, 1593462, 1592715	18, 17, 12	14.82855, 12.5571, 11.7126	7.88255, 6.78543, 7.42393	11.80513,	3	snx16	162999
GL172861.1	1656765	1657271	1657240, 1657233, 1657240	24, 38, 13	27.29272, 41.23631, 15.89249	13.50336, 16.26924, 9.61177	23.84909,	3	snx16	227852
GL172862.1	589492	589949	589588, 589588, 589588	23, 19, 19	23.48681, 19.41322, 22.04849	11.56632, 10.37021, 11.92281	20.14878,	3	hoxc3	31079
GL172862.1	956105	956682	956147, 956112, 956131	14, 17, 10	13.32664, 16.76699, 11.997	7.99066, 9.33319, 7.78835	10.37457,	3	NA	3711
GL172862.1	1021031	1021504	1021203, 1021189, 1021189	44, 30, 27	55.12122, 31.86026, 33.99821	21.68686, 14.02881, 16.4039	51.1964, 2	3	NA	3678
GL172862.1	1069840	1070991	1069996, 1069852, 1070271	13, 24, 9	13.14146, 21.95615, 9.41119	8.08784, 10.31898, 6.47287	10.19842,	3	sp7	8009
GL172862.1	1293210	1294193	1293625, 1293260, 1293620	9, 10, 6	8.77314, 7.26311, 5.2665	6.111, 5.03621, 4.24757	6.09355, 4	3	rarg	34293
GL172862.1	1680595	1681694	1680996, 1680866, 1680817	8, 17, 9	6.86256, 14.08219, 10.01275	5.0733, 7.70044, 6.79683	4.3506, 11	3	eif4b	27503
GL172863.1	9993	10664	10446, 10272, 10035	11, 10, 5	9.90875, 9.07627, 5.45388	6.51186, 6.1667, 4.3157	7.14919, 6	3	kcnj3	10779
GL172863.1	58564	59539	58949, 58892, 59389	9, 7, 5	9.12009, 5.97725, 5.9506	6.30997, 4.62287, 4.54784	6.42028, 3	3	kcnj3	0
GL172863.1	97781	98292	98052, 97979, 98249	6, 11, 4	5.95712, 10.3713, 4.67325	4.64377, 6.79219, 3.78987	3.52158, 7	3	kcnj3	0
GL172863.1	208272	208723	208372, 208349, 208376	19, 33, 24	20.93125, 35.19981, 28.82063	11.274, 14.81526, 14.39789	17.67176,	3	galnt13	14614
GL172863.1	215078	215571	215492, 215508, 215545	10, 7, 7	9.33595, 5.71889, 8.69489	6.32332, 4.46356, 6.06379	6.62299, 3	3	galnt13	7766
GL172863.1	254049	254771	254361, 254390, 254190	21, 26, 7	21.64688, 26.55025, 7.61369	11.10892, 12.36162, 5.54854	18.3637, 2	3	galnt13	0
GL172863.1	284885	285092	285051, 285038, 285051	15, 19, 13	15.19946, 17.83113, 15.70541	8.88991, 9.41378, 9.51556	12.15964,	3	galnt13	0
GL172863.1	392495	392973	392659, 392901, 392834	5, 7, 5	3.75999, 5.96795, 5.27517	3.34972, 4.61718, 4.22616	1.63806, 3	3	galnt13	0
GL172863.1	403670	404117	403926, 404039, 403937	9, 18, 7	8.61068, 18.91825, 7.61369	6.01615, 10.35485, 5.54854	5.94135, 1	3	rprm	4874
GL172863.1	571867	572284	571931, 571900, 571900	12, 22, 10	12.45843, 20.55767, 12.16489	7.86167, 10.13496, 7.87042	9.54648, 1	3	ARL6IP6	0
GL172863.1	733545	734382	734334, 734335, 734325	63, 82, 38	77.32037, 100.58306, 48.64148	24.94947, 27.72663, 20.62397	73.15121,	3	stam2	11641
GL172863.1	798424	799056	798655, 798621, 798897	9, 7, 6	8.66407, 5.97725, 7.25145	6.04743, 4.62287, 5.287	5.99101, 3	3	cacnb4	17176
GL172863.1	977788	978561	978291, 978129, 978379	6, 12, 5	5.47306, 10.53798, 5.91431	4.36955, 6.71099, 4.53172	3.10811, 7	3	neb	0
GL172863.1	1154829	1156607	1155274, 1155283, 1155766	20, 27, 20	18.39153, 23.77514, 20.50302	9.4544, 10.38831, 10.7292	15.21806,	3	NA	5825
GL172863.1	1172338	1172624	1172340, 1172387, 1172340	34, 59, 26	40.34511, 66.23651, 33.6591	17.446, 20.81338, 16.5312	36.63731,	3	NA	0
GL172863.1	1189121	1189423	1189356, 1189357, 1189356	17, 36, 14	15.06219, 36.00328, 15.06595	8.2965, 14.08353, 9.02131	12.02822,	3	nmi	0
GL172863.1	1324786	1325133	1324867, 1324828, 1325044	14, 11, 12	13.96392, 8.48203, 13.44151	8.37431, 5.62571, 8.41472	10.9769, 5	3	rnd3	55867
GL172863.1	1346630	1347043	1346835, 1346936, 1346999	5, 9, 6	3.88983, 7.55109, 6.87953	3.43212, 5.37505, 5.11627	1.75752, 5	3	rnd3	33957
GL172863.1	1465323	1465896	1465647, 1465498, 1465680	8, 6, 5	7.15567, 4.30294, 4.70718	5.25151, 3.64568, 3.9211	4.61046, 2	3	rnd3	61688
GL172863.1	1627098	1627445	1627416, 1627408, 1627415	53, 42, 29	63.34983, 46.69275, 36.72021	22.08782, 17.68645, 17.27746	59.32579,	3	mmadhc	8160
GL172863.1	1683838	1684390	1684344, 1684341, 1684344	8, 20, 15	7.52511, 18.17471, 16.91122	5.47115, 9.32366, 9.88541	4.94319, 1	3	lypd6	61
GL172863.1	1692673	1693119	1693091, 1693100, 1692781	6, 11, 5	5.63898, 8.41104, 5.14967	4.46544, 5.58113, 4.16141	3.25772, 5	3	lypd6	0
GL172864.1	41844	43144	42700, 42712, 42814	25, 31, 11	20.05382, 25.86137, 7.61312	8.91088, 10.25213, 5.07984	16.82168,	3	nedd4l	44052
GL172864.1	72002	72269	72224, 72233, 72244	29, 31, 18	31.44014, 32.72958, 22.76598	14.16438, 14.15394, 12.53748	27.8998, 2	3	nedd4l	14927
GL172864.1	193288	193565	193539, 193562, 193529	23, 40, 22	24.40756, 44.37267, 23.70037	12.11882, 17.226, 12.02241	21.04272,	3	nedd4l	0
GL172864.1	344723	345221	345183, 345195, 345117	19, 37, 10	19.33502, 39.31247, 10.97191	10.32316, 15.57531, 7.25854	16.12917,	3	malt1	1865
GL172864.1	390562	390942	390879, 390915, 390901	15, 22, 16	14.98937, 22.3038, 19.86305	8.76428, 11.18464, 11.32783	11.95813,	3	malt1	2749
GL172864.1	563118	564025	563702, 563590, 563338	12, 13, 7	10.93921, 11.2197, 7.50542	6.95711, 6.92276, 5.49244	8.12344, 8	3	rax	2676
GL172864.1	594939	595551	594967, 594964, 595005	18, 14, 17	19.51813, 13.32457, 19.99824	10.7103, 7.98941, 11.2232	16.30567,	3	cplx4	0
GL172864.1	605300	605823	605630, 605397, 605499	8, 9, 7	8.42257, 8.19918, 7.55909	5.97763, 5.77148, 5.52035	5.76336, 5	3	lman1	5314
GL172864.1	809017	809711	809044, 809041, 809043	117, 127, 90	154.78465, 163.98218, 122.47332	36.65987, 35.74078, 35.12766	150.03456	3	ccbe1	106423
GL172864.1	854246	854620	854451, 854514, 854361	7, 18, 7	5.27717, 19.68157, 8.35502	4.185, 10.80641, 5.9108	2.93268, 1	3	ccbe1	151652
GL172864.1	903584	904142	904115, 904104, 904109	62, 79, 62	75.85566, 95.02385, 79.56593	24.64225, 26.34952, 26.75257	71.70061,	3	NA	109078
GL172864.1	913799	914278	914233, 914223, 914139	5, 17, 6	4.23867, 15.29522, 6.30561	3.64743, 8.43856, 4.83031	2.0685, 12	3	NA	98942
GL172864.1	947337	947638	947491, 947579, 947583	8, 11, 7	6.08111, 8.41104, 6.55126	4.58437, 5.58113, 4.96529	3.62785, 5	3	NA	65582
GL172864.1	1007169	1007529	1007184, 1007236, 1007242	5, 13, 7	4.17642, 11.07988, 7.20145	3.60969, 6.83637, 5.33074	2.01422, 8	3	NA	5691
GL172864.1	1083547	1084092	1084047, 1084018, 1083932	11, 10, 7	8.96891, 7.86505, 7.20145	5.93064, 5.4165, 5.33074	6.27876, 5	3	pdzd2	0
GL172864.1	1130520	1131031	1131003, 1131006, 1131004	9, 10, 9	8.25643, 8.32357, 8.88966	5.80588, 5.70361, 6.17838	5.62516, 5	3	pdzd2	0
GL172864.1	1329363	1329773	1329490, 1329709, 1329726	6, 12, 5	5.90927, 9.78474, 4.99237	4.61743, 6.24415, 4.0781	3.50164, 7	3	npr3	8914
GL172864.1	1402966	1403360	1402981, 1403000, 1402980	30, 28, 16	32.78402, 26.47393, 17.4758	14.57733, 11.60281, 10.00266	29.21525,	3	npr3	14752
GL172864.1	1435947	1436445	1436266, 1436111, 1436244	8, 17, 6	6.14488, 14.08219, 6.25896	4.62489, 7.70044, 4.80589	3.6868, 11	3	npr3	47733
GL172864.1	1594085	1594540	1594302, 1594328, 1594204	16, 16, 6	17.16405, 13.98946, 6.45058	9.82095, 7.9066, 4.90508	14.0384, 1	3	NA	28284
GL172864.1	1630172	1630919	1630447, 1630361, 1630443	18, 27, 14	19.00683, 30.99214, 17.51288	10.40758, 14.6477, 10.35066	15.81175,	3	sub1	28304
GL172864.1	1654048	1654826	1654337, 1654700, 1654191	9, 8, 4	8.16099, 7.07159, 4.50795	5.74847, 5.20073, 3.71446	5.53692, 4	3	sub1	4397
GL172865.1	620460	620912	620727, 620734, 620806	6, 7, 6	5.96715, 5.8946, 7.29403	4.64927, 4.57222, 5.30581	3.52158, 3	3	kiaa0368	1089
GL172865.1	1712342	1714416	1713716, 1713315, 1713703	17, 23, 13	16.17152, 20.59333, 13.97446	8.97223, 9.83704, 8.57174	13.0891, 1	3	frmpd1	71976
GL172866.1	201901	202502	202018, 201945, 202001	5, 6, 5	4.27048, 4.56637, 4.99237	3.6666, 3.81494, 4.0781	2.09564, 2	3	arhgef37	0
GL172866.1	374423	375516	374729, 374954, 374682	6, 24, 3	5.96715, 24.46445, 3.47371	4.64927, 11.8146, 3.03189	3.52158, 2	3	dpysl3	10374
GL172866.1	1358351	1358976	1358771, 1358521, 1358823	8, 16, 7	7.15567, 15.10361, 7.2501	5.25151, 8.58708, 5.35703	4.61046, 1	3	msx2	74006
GL172866.1	1445325	1446133	1445597, 1445610, 1445570	7, 11, 13	6.94335, 10.15827, 14.32377	5.18876, 6.66366, 8.76942	4.42528, 7	3	msx2	5309
GL172866.1	1502865	1503552	1503155, 1503001, 1503290	11, 13, 13	9.90875, 10.19363, 14.46982	6.51186, 6.28711, 8.85107	7.14919, 7	3	msx2	62849
GL172867.1	12167	12511	12349, 12266, 12202	5, 9, 4	4.43696, 7.51164, 4.67325	3.76555, 5.35055, 3.78987	2.24017, 5	3	slit2	329875
GL172867.1	31407	32462	32179, 31964, 31632	7, 11, 5	6.37715, 9.52738, 5.27517	4.86324, 6.27759, 4.22616	3.90257, 6	3	slit2	309924

GL172867.1	52802	53085	52826, 52869, 52902	13, 13, 7	15.1723, 12.93015, 8.646	9.23486, 7.96262, 6.04229	12.13305,	3	slit2	289301
GL172867.1	494913	495406	495339, 495261, 495280	8, 14, 6	7.28955, 12.33756, 6.87953	5.33177, 7.38613, 5.11627	4.72176, 9	3	kcnip4	12347
GL172867.1	599277	599750	599712, 599726, 599719	18, 20, 14	22.23921, 22.87459, 18.40474	12.25494, 12.13503, 10.78924	18.93827,	3	adgra3	88470
GL172867.1	686907	687541	686936, 687102, 686934	36, 41, 20	40.22649, 43.61307, 25.00335	16.52975, 16.35802, 13.33871	36.52053,	3	adgra3	679
GL172867.1	880631	880924	880871, 880866, 880897	12, 18, 12	13.55951, 18.60217, 16.0427	8.47903, 10.16604, 9.71071	10.59689,	3	gba3	122057
GL172867.1	909314	910168	910026, 910027, 909962	30, 50, 38	35.54458, 53.76538, 47.202	16.21686, 18.11976, 19.77684	31.92042,	3	ppargc1a	125348
GL172867.1	1020105	1020607	1020180, 1020375, 1020399	8, 9, 5	8.34715, 7.10358, 5.9506	5.9367, 5.09508, 4.54784	5.71011, 4	3	ppargc1a	14909
GL172867.1	1156353	1156824	1156452, 1156449, 1156701	16, 25, 8	11.73743, 17.60286, 6.27048	6.53951, 7.58461, 4.70442	8.87017, 1	3	ppargc1a	67843
GL172867.1	1158338	1159176	1158787, 1158495, 1158782	34, 34, 19	33.39364, 27.16268, 17.86975	13.37519, 10.01101, 9.43718	29.81235,	3	ppargc1a	69828
GL172867.1	1198036	1198389	1198216, 1198274, 1198260	14, 10, 9	14.83249, 8.66076, 9.70054	8.88629, 5.91256, 6.63089	11.80835,	3	ppargc1a	109526
GL172867.1	1349725	1350654	1350021, 1350107, 1350101	11, 21, 4	10.49148, 19.60696, 4.67325	6.86423, 9.87975, 3.78987	7.70044, 1	3	ppargc1a	261215
GL172867.1	1424272	1425080	1424608, 1424814, 1424704	17, 18, 3	20.58282, 16.34754, 3.37781	11.5459, 8.80191, 2.98791	17.33453,	3	ppargc1a	335762
GL172867.1	1512598	1513110	1512881, 1512761, 1512879	7, 11, 5	7.1696, 10.60895, 5.19074	5.31345, 6.9343, 4.18277	4.61046, 7	3	ppargc1a	424088
GL172867.1	1573178	1574230	1573445, 1573619, 1573501	7, 11, 6	5.62168, 9.66539, 7.18591	4.40288, 6.36265, 5.25776	3.24345, 7	3	ppargc1a	484668
GL172867.1	1588604	1589054	1588882, 1588875, 1588859	13, 13, 15	14.43911, 11.12608, 17.76194	8.83395, 6.86492, 10.35658	11.43182,	3	ppargc1a	500094
GL172867.1	1622673	1622970	1622945, 1622923, 1622943	51, 77, 31	66.20698, 96.75334, 43.02231	24.7526, 28.31244, 20.13727	62.15012,	3	ppargc1a	534163
GL172867.1	1630953	1631266	1631100, 1631190, 1631162	5, 5, 5	4.82177, 3.94561, 5.9506	3.98509, 3.46715, 4.54784	2.51418, 1	3	ppargc1a	542443
GL172868.1	1028	1383	1191, 1274, 1084	6, 6, 4	5.86227, 4.93312, 4.67325	4.59138, 4.04501, 3.78987	3.46055, 2	3	NA	2023
GL172868.1	2145	2420	2367, 2362, 2271	5, 7, 5	4.61714, 5.97725, 5.75061	3.86998, 4.62287, 4.45735	2.39372, 3	3	NA	986
GL172868.1	57266	58309	57465, 57725, 57509	11, 16, 6	11.15818, 14.98328, 5.99481	7.25692, 8.51381, 4.6644	8.32008, 1	3	NA	13676
GL172868.1	258532	259921	259207, 259072, 258664, 259121, 259891	12, 13, 4, 6, 7	11.32055, 10.72425, 3.64768, 5.793, 7.10639	7.18871, 6.61618, 3.26758, 4.55271, 5.27894	8.47391, 8	3	acvr2a	115261
GL172868.1	417292	417855	417315, 417332, 417385	24, 29, 12	27.29272, 31.17793, 14.96175	13.50336, 14.00832, 9.20442	23.84909,	3	NA	26476
GL172868.1	622098	622681	622345, 622163, 622315	5, 9, 7	4.69353, 7.83963, 8.646	3.9134, 5.55305, 6.04229	2.45909, 5	3	NA	175657
GL172868.1	767278	767637	767600, 767441, 767592	22, 38, 17	25.73329, 41.94991, 21.64669	13.22149, 16.6843, 12.11304	22.33146,	3	NA	320837
GL172868.1	969542	970641	969905, 970456, 970251	11, 20, 5	10.60582, 17.23653, 5.14967	6.93244, 8.76036, 4.16141	7.80831, 1	3	zeb2	375685
GL172868.1	1079501	1079831	1079698, 1079806, 1079661	8, 11, 4	7.77807, 8.23846, 4.38152	5.618, 5.47271, 3.65448	5.17956, 5	3	zeb2	266495
GL172868.1	1093819	1094314	1094103, 1094148, 1094191	12, 8, 6	13.05874, 7.07159, 6.08001	8.20296, 5.20073, 4.71063	10.11961,	3	zeb2	252012
GL172868.1	1117577	1118148	1118110, 1118116, 1118104	65, 95, 84	82.6404, 116.10483, 116.27303	26.9016, 29.01419, 35.21385	78.41881,	3	zeb2	228178
GL172868.1	1213357	1213991	1213426, 1213395, 1213395	49, 47, 38	55.07697, 51.42707, 52.14061	19.29768, 18.15279, 22.65283	51.1529, 4	3	zeb2	132335
GL172868.1	1577493	1577957	1577917, 1577918, 1577911	69, 80, 65	86.48269, 101.23244, 86.1795	26.92738, 29.13114, 28.93116	82.22159,	3	gtdc1	12149
GL172868.1	1602933	1603444	1603133, 1602970, 1602934	6, 18, 7	5.20598, 17.45593, 7.61369	4.2113, 9.47501, 5.54854	2.86702, 1	3	gtdc1	37589
GL172868.1	1618514	1619045	1618562, 1618590, 1618544	45, 55, 35	57.38791, 65.50603, 45.48785	22.5425, 22.32932, 20.05263	53.43325,	3	gtdc1	53170
GL172869.1	687151	687531	687240, 687226, 687160	11, 15, 8	9.71398, 15.7813, 8.56955	6.39253, 9.23437, 6.05652	6.97155, 1	3	U2	14056
GL172869.1	1052309	1053813	1052836, 1052547, 1053501	9, 16, 8	9.00096, 12.6954, 8.4586	6.24222, 7.11701, 5.99709	6.30808, 9	3	NA	4266
GL172871.1	97898	98966	98097, 98576, 98533	8, 9, 6	7.72597, 7.39577, 6.71074	5.588, 5.27837, 5.03498	5.13061, 4	3	ipo7	0
GL172871.1	106368	107605	106750, 106532, 106779	9, 14, 7	8.77314, 10.88047, 6.96907	6.111, 6.48797, 5.20309	6.09355, 8	3	ipo7	0
GL172871.1	131388	131894	131573, 131795, 131690	19, 31, 14	19.8867, 30.57355, 16.04334	10.65421, 12.89098, 9.57242	16.66031,	3	tmem41b	2614
GL172871.1	136568	137281	136678, 136760, 136750	12, 11, 9	8.45074, 6.174, 8.34444	5.41264, 4.18743, 5.85853	5.79047, 3	3	tmem41b	0
GL172871.1	147443	147980	147806, 147737, 147738	6, 14, 6	5.51356, 10.54152, 5.83216	4.39314, 6.27972, 4.57461	3.14574, 7	3	znf143	0
GL172871.1	155436	156004	155485, 155722, 155499	27, 28, 19	27.3225, 24.65647, 20.03654	12.46113, 10.55907, 10.74374	23.87824,	3	znf143	0
GL172871.1	188547	189208	188831, 188845, 188627	6, 13, 6	4.93136, 8.6375, 5.0826	4.04392, 5.3289, 4.13667	2.61555, 6	3	znf143	14504
GL172871.1	241433	242023	241504, 241497, 241503	15, 30, 16	17.59037, 30.89514, 19.86305	10.26303, 13.45807, 11.32783	14.44848,	3	swap70	811
GL172871.1	311171	311768	311204, 311581, 311273	8, 8, 5	6.31005, 4.69397, 4.45433	4.7294, 3.68889, 3.77574	3.84065, 2	3	sbf2	0
GL172871.1	312888	313552	313518, 313524, 313498	13, 27, 24	10.21914, 23.10589, 30.05209	6.30293, 10.00869, 15.1024	7.4427, 19	3	sbf2	0
GL172871.1	346403	347356	346938, 347290, 347133	23, 32, 29	22.86884, 30.86283, 33.0122	11.19513, 12.68438, 15.10019	19.54862,	3	sbf2	0
GL172871.1	425495	426144	425965, 425881, 425961	16, 25, 10	16.83862, 27.15225, 11.10454	9.62959, 13.07691, 7.32977	13.72526,	3	sbf2	42028
GL172871.1	441305	442558	441981, 441999, 441978	30, 39, 20	23.92527, 26.87043, 17.17989	9.52914, 8.56199, 8.72651	20.57401,	3	adm	42957
GL172871.1	461774	462306	461778, 461812, 461818	13, 26, 14	15.29169, 28.20606, 16.04334	9.29854, 13.35104, 9.57242	12.24396,	3	adm	23209
GL172871.1	475452	476498	476137, 476137, 476239	8, 8, 4	6.78332, 7.07159, 4.26291	5.02459, 5.20073, 3.59641	4.27811, 4	3	adm	9017
GL172871.1	509182	510140	510021, 509580, 509433	6, 14, 10	5.96715, 14.45897, 10.90706	4.64927, 8.66788, 7.22344	3.52158, 1	3	adm	19593
GL172871.1	546482	546792	546595, 546758, 546678	8, 10, 5	8.4095, 9.17452, 5.06966	5.97056, 6.22617, 4.11933	5.76336, 6	3	adm	56893
GL172871.1	592462	593311	593169, 592626, 592626	4, 9, 9	3.31554, 7.59096, 10.79992	3.07164, 5.39978, 7.19283	1.39756, 5	3	adm	102873
GL172871.1	599735	600281	599843, 599879, 599957	17, 12, 12	19.20348, 10.83413, 15.47496	10.77332, 6.89288, 9.45079	16.00121,	3	adm	110146
GL172871.1	612345	612832	612753, 612698, 612425	9, 6, 7	9.72082, 4.93312, 8.21885	6.64182, 4.04501, 5.84717	6.97155, 2	3	adm	122756
GL172871.1	688611	689544	689041, 689044, 688949	10, 14, 10	9.77513, 12.92044, 12.90749	6.5837, 7.74345, 8.21675	7.02302, 1	3	adm	199022
GL172871.1	708516	709174	708538, 708591, 708628	11, 25, 15	11.15818, 26.42765, 17.15492	7.25692, 12.64351, 10.0222	8.32008, 2	3	adm	218927
GL172871.1	730704	730922	730778, 730827, 730817	36, 43, 36	40.66463, 48.62682, 47.44992	16.78775, 18.35503, 20.78012	36.95168,	3	NA	224112
GL172871.1	740166	741099	740711, 740609, 740558	11, 12, 8	11.29029, 10.08092, 9.8421	7.3332, 6.42828, 6.68602	8.44541, 7	3	NA	213935
GL172871.1	807536	807910	807562, 807563, 807562	64, 81, 67	82.84255, 105.855, 91.69556	27.55654, 31.11125, 31.00898	78.61861,	3	NA	147124
GL172871.1	864768	865446	864933, 864919, 864926	13, 15, 12	11.66066, 13.80611, 14.3175	7.19428, 8.04733, 8.88032	8.79778, 1	3	NA	89588
GL172871.1	897052	898675	898642, 898637, 898648	31, 50, 30	32.60369, 52.78546, 33.84593	14.07963, 17.58742, 15.20893	29.03896,	3	NA	56359
GL172871.1	931742	932205	932062, 932078, 931960	15, 19, 8	14.65409, 16.15772, 8.1956	8.5626, 8.40292, 5.85348	11.63756,	3	NA	22829
GL172871.1	944417	945362	944795, 944958, 944570	9, 14, 9	8.77314, 12.1395, 10.14484	6.111, 7.26424, 6.86555	6.09355, 9	3	NA	9672
GL172871.1	984474	985055	985004, 984949, 985021	14, 14, 8	15.84929, 14.45897, 10.1465	9.46495, 8.66788, 6.82176	12.77923,	3	NA	28251
GL172871.1	1039160	1039489	1039464, 1039416, 1039397	12, 6, 10	13.38718, 4.93312, 13.11399	8.38496, 4.04501, 8.30815	10.43197,	3	NA	82937
GL172871.1	1080278	1080832	1080775, 1080596, 1080392	7, 21, 6	7.1696, 20.81922, 6.87953	5.31345, 10.61044, 5.11627	4.61046, 1	3	NA	124055
GL172871.1	1104159	1104409	1104225, 1104216, 1104192	21, 19, 13	22.14207, 15.9599, 13.84064	11.40611, 8.28432, 8.49513	18.84387,	3	NA	147936
GL172871.1	1240593	1241434	1240850, 1241115, 1241090	17, 23, 8	17.48974, 20.94885, 7.99854	9.76779, 10.04755, 5.74345	14.35103,	3	NA	284370
GL172871.1	1246970	1247940	1247673, 1247690, 1247605	14, 14, 5	13.32664, 12.33756, 4.77515	7.99066, 7.38613, 3.9592	10.37457,	3	NA	290747
GL172871.1	1266993	1267768	1267346, 1267573, 1267132	11, 8, 11	11.85906, 7.07159, 13.83651	7.65504, 5.20073, 8.67735	8.98595, 4	3	NA	310770
GL172871.1	1370345	1371492	1371011, 1371250, 1370905	8, 8, 4	8.42257, 7.07159, 4.55201	5.97763, 5.20073, 3.73489	5.76336, 4	3	NA	414122
GL172871.1	1384152	1385031	1384500, 1384526, 1384784	10, 6, 5	9.49535, 4.51135, 5.27517	6.41851, 3.77984, 4.22616	6.77344, 2	3	NA	427929
GL172871.1	1439019	1439714	1439374, 1439435, 1439313	12, 16, 14	9.81839, 12.90802, 15.35015	6.26509, 7.24623, 9.184	7.06426, 1	3	NA	482796
GL172871.1	1489742	1490359	1490310, 1490049, 1490017	5, 8, 4	4.82177, 6.59014, 4.67325	3.98509, 4.90493, 3.78987	2.51418, 4	3	NA	533519
GL172871.1	1515761	1516366	1515768, 1515788, 1515797	29, 41, 28	34.13249, 46.29793, 35.3581	15.76465, 17.90246, 16.84442	30.53689,	3	NA	559538
GL172871.1	1521449	1522067	1522039, 1522012, 1522007	26, 44, 13	28.87987, 50.36766, 14.46982	13.75312, 18.90658, 8.85107	25.39905,	3	NA	565226
GL172871.1	1658551	1659476	1658860, 1658902, 1658726	17, 24, 6	18.21101, 24.1521, 6.45058	10.19603, 11.62735, 4.90508	15.04611,	3	NA	702328
GL172871.1	1696302	1696743	1696382, 1696521, 1696405	8, 14, 8	7.47668, 13.8891, 8.6841	5.44269, 8.32957, 6.11715	4.89713, 1	3	NA	740079
GL172872.1	175573	175937	175646, 175768, 175593	6, 7, 6	5.96715, 5.97725, 6.99807	4.64927, 4.62287, 5.17195	3.52158, 3	3	NA	9908
GL172872.1	181881	182126	181916, 181919, 181969	6, 9, 5	5.96715, 7.43399, 5.9506	4.64927, 5.30221, 4.54784	3.52158, 4	3	ttc21b	12237
GL172872.1	461985	462734	462286, 462006, 462100	21, 25, 17	22.14207, 24.81034, 20.18522	11.40611, 11.67607, 11.32722	18.84387,	3	U2	57936
GL172872.1	839510	840483	839593, 839618, 839606	10, 21, 14	8.40516, 14.88558, 14.79463	5.75437, 7.12614, 8.86428	5.76336, 1	3	b3galt1	8166
GL172872.1	1308906	1309895	1309633, 1309087, 1309530	12, 16, 8	11.26426, 15.60807, 9.76268	7.15468, 8.89325, 6.64964	8.4212, 12	3	abcb11	0
GL172872.1	1387258	1388110	1387764, 1387763, 1387711	33, 33, 15	35.44515, 34.92198, 16.75372	14.9596, 14.65208, 9.79627	31.82294,	3	dhrs9	808
GL172873.1	3493	4161	3779, 3909, 3594	5, 9, 4	4.655, 7.17446, 4.67325	3.89157, 5.1397, 3.78987	2.42652, 4	3	poln	11296
GL172873.1	130816	131524	131127, 131118, 131229	7, 8, 4	6.50909, 6.81727, 4.38152	4.94073, 5.04548, 3.65448	4.02371, 4	3	nat8l	18524
GL172873.1	417299	417870	417521, 417634, 417403	10, 20, 7	9.661, 17.66446, 7.72582	6.51661, 9.01671, 5.6058	6.9283, 14	3	NA	12513
GL172873.1	561718	562524	562411, 562047, 561970	9, 8, 5	8.0222, 5.53815, 4.91762	5.66446, 4.2362, 4.03768	5.40727, 3	3	fgfr3	2707
GL172873.1	563812	564904	563939, 564527, 564335	5, 11, 4	3.52262, 8.23846, 3.85136	3.19626, 5.47271, 3.38149	1.55963, 5	3	fgfr3	4801
GL172873.1	640318	640551	640524, 640498, 640494	8, 9, 8	7.06928, 8.21099, 9.25066	5.19933, 5.77858, 6.40569	4.5414, 5.7	3	NA	15669
GL172873.1	688382	688758	688413, 688422, 688410	9, 14, 10	9.72082, 10.80331, 10.30677	6.64182, 6.44051, 6.89026	6.97155, 8	3	NA	31027
GL172873.1	719597	720066	719783, 719803, 719690	23, 31, 6	25.52391, 30.96083, 6.168	12.78506, 13.11605, 4.75778	22.12768,	3	NA	3159
GL172873.1	949861	950464	949992, 950026, 950155	11, 7, 5	11.15818, 5.97725, 5.9506	7.25692, 4.62287, 4.54784	8.32008, 3	3	tacc3	45160
GL172873.1	1025461	1026001	1025654, 1025655, 1025617	11, 18, 7	12.43693, 19.97713, 7.50542	7.97018, 10.97931, 5.49244	9.52692, 1	3	tmem129	3818
GL172873.1	1038114	1038367	1038253, 1038182, 1038159	12, 16, 5	11.67221, 12.53032, 4.64121	7.39985, 7.01691, 3.88372	8.80793, 9	3	tmem129	1464
GL172873.1	1074042	1075124	1074738, 1074460, 1074938	13, 15, 7	11.87199, 11.56639, 7.29952	7.32374, 6.67456, 5.38357	8.9979, 8.8	3	slbp	17290
GL172873.1	1102676	1104072	1103532, 1103689, 1103797	18, 26, 12	16.62541, 23.91458, 12.03712	8.97077, 10.80262, 7.61609	13.52454,	3	slbp	45924
GL172873.1	1217413	1217749	1217418, 1217430, 1217570	8, 17, 6	6.70593, 14.81481, 6.25896	4.9768, 8.14576, 4.80589	4.20487, 1	3	fam53a	2469
GL172873.1	1273377	1273811	1273765, 1273766, 1273777	26, 30, 22	31.23909, 31.50057, 29.24652	15.14648, 13.81575, 15.17695	27.70337,	3	fam53a	58433
GL172873.1	1429407	1429739	1429609, 1429711, 1429719	15, 20, 6	10.57466, 15.02475, 4.67403	6.07445, 7.46108, 3.8832	7.77923, 1	3	fam53a	214463
GL172873.1	1431096	1431348	1431122, 1431110, 1431119	55, 53, 37	64.23724, 57.22467, 48.83529	21.61341, 18.67685, 21.16667	60.20211,	3	fam53a	216152
GL172873.1	1622355	1623110	1623073, 1623082, 1622864	17, 26, 19	21.34551, 31.2219, 26.56077	11.95527, 15.13645, 14.30986	18.07159,	3	nkx1-1	37946
GL172873.1	1672352	1673085	1672410, 1672978, 1672696	7, 15, 7	7.1696, 14.03607, 8.69489	5.31345, 8.18766, 6.06379	4.61046, 1	3	nkx1-1	4084
GL172873.1	1683481	1683972	1683810, 1683817, 1683691	17, 30, 10	16.56188, 29.23872, 11.38247	9.20909, 12.48814, 7.47651	13.46342,	3	NA	0
GL172874.1	475554	476523	475936, 475927, 475927	31, 31, 13	28.21807, 25.96629, 11.90884	11.54575, 10.30841, 7.34626	24.75379,	3	npm3	12580
GL172874.1	689835	690628	690231, 690234, 690233	17, 17, 16	18.64535, 15.63512, 18.88025	10.45058, 8.64575, 10.798	15.46286,	3	fgf8	45522
GL172875.1	553	1345	771, 1128, 1223	5, 7, 5	4.82177, 5.97725, 5.9506	3.98509, 4.62287, 4.54784	2.51418, 3	3	NA	1187
GL172875.1	527378	528193	527892, 527722, 527715	5, 14, 5	4.36883, 13.0902, 4.99237	3.72533, 7.84698, 4.0781	2.18201, 1	3	NA	4234
GL172875.1	752241	752473	752313, 752352, 752400	8, 9, 5	8.42257, 8.01512, 5.91431	5.97763, 5.66016, 4.53172	5.76336, 5	3	NA	125929
GL172875.1	1026742	1027206	1027162, 1027023, 1027017	9, 14, 7	9.72082, 14.45897, 8.69489	6.64182, 8.66788, 6.06379	6.97155, 1	3	xtr-mir-223	53101
GL172875.1	1319117	1319917	1319739, 1319734, 1319740	7, 18, 5	5.93788, 16.52108, 5.91431	4.59878, 8.90737, 4.53172	3.52158, 1	3	hsf3	521
GL172875.1	1341950	1342396	1342289, 1342290, 1342076	5, 12, 4	4.82177, 11.48527, 4.67325	3.98509, 7.28792, 3.78987	2.51418, 8	3	ngrn	3904
GL172875.1	1420953	1421509	1421481, 1421439, 1421269	7, 15, 7	7.1696, 15.80067, 8.69489	5.31345, 9.24574, 6.06379	4.61046, 1	3	NA	46062
GL172875.1	1583031	1583979	1583676, 1583683, 1583706	14, 24, 13	14.75506, 25.46494, 15.43582	8.84125, 12.41437, 9.3748	11.73423,	3	gpr83	52012
GL172876.1	1040465	1041398	1040750, 1040751, 1040960	12, 22, 7	12.05017, 21.56447, 8.42533	7.62377, 10.73971, 5.94313	9.16715, 1	3	gsto1	0
GL172876.1	1136946	1137387	1137160, 1137124, 1137082	9, 14, 5	8.88537, 12.97647, 5.31857	6.17591, 7.77766, 4.24819	6.19961, 1	3	NA	12102
GL172876.1	1169656	1170272	1170047, 1170074, 1170187	5, 6, 5	4.82177, 4.93312, 5.9506	3.98509, 4.04501, 4.54784	2.51418, 2	3	NA	0
GL172876.1	1304587	1304959	1304883, 1304907, 1304857	18, 32, 14	20.96944, 38.01322, 18.52583	11.5502, 16.88705, 10.84669	17.70889,	3	NA	18260
GL172876.1	1399337	1399809	1399576, 1399656, 1399457	5, 11, 7	4.82177, 10.26356, 8.69489	3.98509, 6.72731, 6.06379	2.51418, 7	3	NA	0
GL172876.1	1612858	1613728	1613382, 1613318, 1613596	14, 18, 7	12.13106, 14.31692, 6.2897	7.25904, 7.57602, 4.81137	9.24462, 1	3	NA	197712
GL172876.1	1615393	1616269	1615745, 1616176, 1616113	15, 19, 8	12.8537, 14.54112, 6.83213	7.46342, 7.44375, 5.05462	9.92465, 1	3	NA	200247
GL172877.1	40323	42126	41853, 41655, 41306	10, 9, 7	8.20198, 6.67403, 7.84208	5.62778, 4.82293, 5.66426	5.57541, 4	3	nf1	1115064
GL172877.1	337418	338191	337473, 338002, 337866	5, 7, 6	4.27048, 5.82304, 5.99481	3.6666, 4.52813, 4.6644	2.09564, 3	3	nf1	818999
GL172877.1	1358296	1358812	1358695, 1358446, 1358476	9, 10, 5	9.63662, 8.84031, 5.23257	6.59633, 6.02287, 4.20435	6.90556, 6	3	wsb1	27530
GL172878.1	201328	201659	201455, 201439, 201475	7, 6, 8	6.46447, 4.86158, 9.60885	4.91463, 4.00069, 6.57807	3.98312, 2	3	NA	420306
GL172878.1	931282	931844	931817, 931797, 931771	34, 42, 29	38.90452, 49.11052, 35.74224	16.59248, 19.0913, 16.71053	35.21949,	3	vrk2	153742
GL172878.1	957631	958548	957844, 958029, 958090	6, 11, 9	5.3552, 10.26356, 10.28145	4.3003, 6.72731, 6.93568	3.00253, 7	3	ccdc85a	147951
GL172878.1	1118452	1119499	1119132, 1118757, 1118423, 1118983	10, 12, 3, 9	9.33595, 10.63474, 3.24307, 10.72164	6.32332, 6.77054, 2.92359, 7.15493	6.62299, 7	3	ccdc85a	0
GL172878.1	1645526	1646614	1646078, 1646143, 1646072	10, 17, 7	9.23294, 12.89704, 6.2897	6.26141, 6.98739, 4.81137	6.52626, 1	3	foxa2	191176
GL172879.1	726372	727445	726864, 726718, 727023	8, 20, 11	5.72321, 15.46964, 12.13051	4.35542, 7.71805, 7.80464	3.33547, 1	3	GAS2L3	0
GL172879.1	1501648	1502299	1502055, 1502292, 1502243	12, 35, 8	10.44071, 36.6591, 9.05167	6.65101, 14.85933, 6.30653	7.65237, 3	3	n4bp3	49932
GL172880.1	22413	22916	22558, 22662, 22783	9, 9, 6	8.0679, 7.06866, 5.53406	5.69219, 5.07306, 4.40503	5.45, 4.651	3	LRRTM3	151722
GL172880.1	193211	193819	193450, 193478, 193742	7, 9, 5	7.1696, 8.21099, 5.9506	5.31345, 5.77858, 4.54784	4.61046, 5	3	LRRTM3	17027
GL172880.1	377991	380877	378932, 379176, 378283, 380381	10, 19, 12, 11	7.54524, 14.70435, 10.84828, 10.10247	5.21478, 7.53937, 6.90153, 6.62982	4.96292, 1	3	sirt1	14024
GL172880.1	380154	380963	380385, 379176, 380794, 380381	12, 19, 10, 11	10.20921, 14.70435, 6.48049, 10.10247	6.50784, 7.53937, 4.54035, 6.62982	7.4341, 11	3	sirt1	16187
GL172880.1	386833	387910	387434, 387574, 387492	13, 29, 18	15.29169, 36.20642, 24.71044	9.29854, 16.98047, 13.52312	12.24396,	3	sirt1	22866
GL172880.1	392664	394250	393057, 393603, 393160	15, 31, 11	14.09249, 32.3733, 12.06254	8.22203, 13.94378, 7.76744	11.1007, 2	3	sirt1	28697
GL172880.1	407348	408704	408130, 407955, 408205	12, 21, 13	9.77685, 17.7017, 13.21599	6.23923, 8.74254, 8.13179	7.0243, 14	3	sirt1	43381
GL172880.1	412293	416037	413014, 413014, 412324, 414165, 415393	19, 29, 10, 6, 13	13.88472, 21.03405, 7.36669, 4.80295, 12.87254	7.06269, 8.31739, 5.10182, 3.96416, 7.92832	10.90098,	3	ecd	40125
GL172880.1	412313	416037	415305, 413014, 412324, 414165, 415393	19, 29, 10, 6, 13	15.14105, 21.03405, 7.36669, 4.80295, 12.87254	7.79671, 8.31739, 5.10182, 3.96416, 7.92832	12.1041, 1	3	ecd	40125
GL172880.1	422082	422803	422357, 422181, 422518	6, 9, 9	5.81608, 8.21099, 11.64359	4.56563, 5.77858, 7.57973	3.4176, 5.7	3	ecd	33359
GL172880.1	427930	428832	428553, 428360, 428265	10, 15, 12	9.661, 15.05167, 16.28773	6.51661, 8.8016, 9.81872	6.9283, 12	3	ecd	27330
GL172880.1	556461	556946	556684, 556688, 556626	9, 10, 5	8.66407, 8.53104, 5.9506	6.04743, 5.83243, 4.54784	5.99101, 6	3	PKD2L1	13057
GL172880.1	973779	974323	974055, 974106, 974237	7, 9, 6	7.1696, 8.21099, 7.29403	5.31345, 5.77858, 5.30581	4.61046, 5	3	NA	11270
GL172882.1	86849	87519	87489, 87032, 87259	5, 7, 3	4.82177, 5.65148, 3.47371	3.98509, 4.42152, 3.03189	2.51418, 3	3	dcaf4	15651
GL172882.1	315327	315998	315606, 315788, 315952	15, 16, 5	12.80515, 13.21822, 4.54579	7.4336, 7.43523, 3.82897	9.87861, 1	3	rgs6	0
GL172882.1	458339	459645	458976, 458995, 458927	7, 15, 3	6.16939, 12.13791, 3.37781	4.73936, 7.02404, 2.98791	3.70916, 9	3	sipa1l1	56588
GL172882.1	458493	459645	459600, 458995, 458927	9, 15, 3	8.30507, 12.13791, 3.37781	5.83501, 7.02404, 2.98791	5.67102, 9	3	sipa1l1	56588
GL172882.1	855819	856685	856037, 856037, 856064	10, 15, 7	10.74428, 13.11311, 7.01416	7.13454, 7.62272, 5.22813	7.93927, 1	3	TTC9	1222
GL172883.1	309718	310047	310004, 309993, 310015	25, 23, 15	31.25446, 25.04419, 18.95532	15.48178, 12.49946, 10.98488	27.71824,	3	arf6.2	54552
GL172883.1	1368469	1369063	1368997, 1369003, 1369006	61, 61, 38	73.67445, 62.37888, 47.45436	23.92913, 17.95252, 19.92565	69.54084,	3	XB-GENE-5804925	4993
GL172884.1	247262	247999	247584, 247584, 247664	12, 13, 7	10.83541, 11.92581, 7.01416	6.89366, 7.35664, 5.22813	8.02495, 9	3	gzf1	6789
GL172884.1	373796	374874	374451, 374363, 374452	23, 14, 14	23.01962, 10.01976, 15.80333	11.28568, 5.96082, 9.43935	19.69475,	3	CD93	5912
GL172884.1	475423	475861	475493, 475578, 475604	7, 10, 10	6.60038, 8.16488, 11.38247	4.99378, 5.60458, 7.47651	4.10761, 5	3	NA	11600
GL172884.1	805425	806273	806085, 805663, 805784	7, 13, 5	6.79162, 13.14514, 5.9506	5.10336, 8.09002, 4.54784	4.28406, 1	3	clhc1	8812
GL172884.1	872102	872693	872427, 872579, 872570	16, 13, 10	15.47689, 10.7674, 10.30677	8.81381, 6.64292, 6.89026	12.4218, 8	3	rtn4	5512
GL172884.1	1184980	1187386	1186536, 1186562, 1185175, 1186571	12, 23, 13, 12	9.69483, 18.4746, 12.81733, 11.50726	6.18815, 8.60508, 7.8954, 7.30112	6.96086, 1	3	c2orf73	18862
GL172884.1	1215522	1215980	1215757, 1215689, 1215831	14, 14, 10	15.15267, 11.99537, 10.59656	9.07115, 7.17543, 7.05292	12.11465,	3	c2orf73	0
GL172884.1	1377292	1378137	1377550, 1377511, 1377524	6, 14, 5	4.59693, 11.32527, 5.50079	3.83437, 6.76206, 4.33868	2.3787, 8.6	3	psme4	0
GL172885.1	127571	128320	127957, 127902, 127996	12, 12, 6	12.11599, 11.04188, 6.76586	7.66241, 7.01971, 5.06179	9.23027, 8	3	NA	119155
GL172885.1	1029635	1030174	1030026, 1030024, 1030022	32, 36, 33	35.18635, 38.5451, 40.66947	15.21026, 15.54541, 18.05017	31.56988,	3	NA	42443
GL172886.1	807670	807952	807839, 807782, 807752	14, 31, 9	14.99044, 35.77021, 10.56949	8.97777, 15.95791, 7.08032	11.95856,	3	NA	5713
GL172887.1	237239	237622	237393, 237361, 237355	24, 33, 21	9.24945, 12.85021, 12.31269	3.9536, 4.30346, 5.75874	6.54121, 1	3	tspan17	42490
GL172887.1	242398	242978	242645, 242648, 242647	107, 111, 50	75.74618, 65.75233, 33.82777	10.98441, 8.46325, 8.91592	71.59229,	3	tspan17	47649
GL172887.1	251230	251561	251351, 251309, 251435	32, 30, 21	12.10342, 7.72828, 7.72885	4.16061, 2.99396, 3.66222	9.21834, 5	3	tspan17	56481
GL172887.1	257362	257750	257451, 257475, 257401	29, 27, 25	8.70066, 4.87768, 8.5631	3.34049, 2.32704, 3.60364	6.02638, 2	3	tspan17	62613
GL172887.1	258060	259447	258782, 258825, 258897	36, 51, 32	13.7491, 18.71393, 14.39219	4.30116, 4.39634, 4.92576	10.7777, 1	3	tspan17	63311
GL172887.1	351975	352657	352177, 352076, 352548	18, 19, 8	14.3755, 13.8119, 6.04892	7.611, 7.02078, 4.56389	11.37132,	3	NA	125938
GL172887.1	353440	353751	353639, 353536, 353651	11, 18, 7	6.97015, 12.34104, 4.78744	4.67803, 6.42014, 3.86898	4.4505, 9.6	3	NA	124844
GL172888.1	498624	499242	498754, 498968, 498992	6, 9, 7	4.83561, 6.26311, 7.72582	3.98453, 4.56059, 5.6058	2.52606, 3	3	qsox1	11662
GL172888.1	505297	506094	505696, 505602, 505668	5, 10, 8	4.82177, 9.39128, 10.1465	3.98509, 6.35644, 6.82176	2.51418, 6	3	qsox1	18335
GL172888.1	524797	526630	525031, 525907, 525869	6, 28, 13	4.9641, 26.27177, 12.65497	4.06411, 11.48537, 7.79825	2.64509, 2	3	NA	19666
GL172888.1	524797	526630	526177, 525907, 525869	12, 28, 13	11.73328, 26.27177, 12.65497	7.43625, 11.48537, 7.79825	8.86615, 2	3	NA	19666
GL172888.1	530419	532229	531413, 530831, 531193	10, 14, 7	8.57531, 9.51418, 5.59189	5.85982, 5.65453, 4.38421	5.90712, 6	3	NA	14067
GL172888.1	1118069	1118771	1118314, 1118400, 1118368	7, 18, 12	5.10557, 14.02138, 11.76526	4.075, 7.40006, 7.45528	2.77482, 1	3	heca	113658
GL172888.1	1462514	1463832	1463239, 1463282, 1463054	28, 51, 32	24.9571, 45.69557, 35.43546	10.72968, 13.61298, 15.3579	21.57686,	3	NA	103809
GL172888.1	1484735	1485971	1485663, 1485533, 1485657	16, 22, 10	17.41877, 22.15105, 12.42918	9.96955, 11.09273, 7.99682	14.28292,	3	NA	126030
GL172889.1	1567662	1568944	1568447, 1568101, 1568008	10, 15, 8	10.6018, 13.37045, 8.80246	7.05583, 7.78062, 6.179	7.80484, 1	3	oca2	55804
GL172890.1	565194	566068	565666, 565567, 565638	114, 136, 61	115.76904, 126.65369, 61.85609	21.08252, 18.41255, 17.69586	111.25166	3	bbs9	0
GL172890.1	1510886	1511484	1511119, 1511378, 1511389	13, 8, 7	12.20544, 4.96414, 6.71158	7.52692, 3.86431, 5.05776	9.31472, 2	3	pou6f2	316061
GL172890.1	1539871	1540827	1540509, 1540182, 1540506	19, 24, 14	10.18445, 10.94367, 7.55781	5.05258, 4.6331, 4.50868	7.41061, 8	3	pou6f2	345046
GL172890.1	1546661	1548157	1547171, 1547139, 1546886	14, 24, 15	8.35264, 15.22774, 10.54355	4.96528, 6.60361, 6.0558	5.71557, 1	3	pou6f2	351836
GL172891.1	111027	111507	111226, 111140, 111157	6, 9, 4	5.27951, 6.55163, 4.50795	4.25534, 4.74494, 3.71446	2.9335, 4.1	3	NA	0
GL172891.1	121908	124090	122910, 122909, 122910	36, 22, 18	39.18397, 19.88511, 19.30306	15.91817, 9.73304, 10.58333	35.49477,	3	NA	0
GL172891.1	152878	153489	153387, 153154, 153133	14, 8, 4	13.8308, 5.81313, 4.50795	8.29466, 4.41317, 3.71446	10.85567,	3	NA	0
GL172891.1	163833	164683	164394, 164591, 164383	4, 6, 8	3.74195, 4.30294, 10.1465	3.32091, 3.64568, 6.82176	1.6222, 2.2	3	NA	0
GL172891.1	180153	181595	181125, 181101, 181144	17, 24, 8	17.04639, 21.77349, 8.80246	9.50173, 10.21126, 6.179	13.92543,	3	NA	67
GL172891.1	186525	186907	186729, 186711, 186593	7, 12, 4	6.89193, 11.66217, 4.67325	5.15998, 7.39386, 3.78987	4.37772, 8	3	NA	6439
GL172891.1	252512	253047	252727, 252713, 252739	11, 9, 7	8.61979, 7.47261, 7.72582	5.71214, 5.32627, 5.6058	5.94958, 5	3	efcab8	5250
GL172891.1	278491	279555	278644, 279015, 278628	15, 9, 16	16.28407, 7.21043, 17.55092	9.52725, 5.1623, 10.04619	13.19656,	3	MYH3	0
GL172891.1	303383	304538	303458, 303918, 303904	32, 52, 46	22.83109, 39.54042, 44.56712	8.42832, 10.64577, 14.85031	19.51252,	3	myh4	7051
GL172891.1	307192	307823	307477, 307474, 307458	42, 68, 34	33.67954, 56.62925, 28.80386	10.81606, 13.11877, 10.85667	30.09283,	3	myh4	3766
GL172891.1	325361	325899	325738, 325539, 325540	11, 11, 5	10.78278, 10.60895, 5.9506	7.03733, 6.9343, 4.54784	7.97565, 7	3	myh4	0
GL172891.1	466252	466811	466528, 466530, 466557	18, 15, 6	20.45461, 15.80067, 7.29403	11.25597, 9.24574, 5.30581	17.20992,	3	myh13	24000
GL172891.1	474107	474447	474137, 474139, 474132	13, 24, 22	12.43975, 26.67478, 29.24652	7.66875, 13.13685, 15.17695	9.5289, 23	3	myh13	31855
GL172891.1	513925	514295	514020, 514025, 514189	6, 11, 3	5.81608, 10.42613, 3.47371	4.56563, 6.8251, 3.03189	3.4176, 7.8	3	NA	22938
GL172891.1	543695	544270	543953, 543955, 543972	6, 9, 10	4.99728, 6.49199, 10.71812	4.08451, 4.70689, 7.12015	2.67584, 4	3	NA	564
GL172891.1	546045	546547	546350, 546356, 546274	10, 12, 7	9.95227, 9.95176, 7.10639	6.68696, 6.34805, 5.27894	7.19087, 7	3	NA	2914
GL172891.1	724871	725530	725130, 724950, 724970	9, 10, 4	9.72082, 9.39128, 4.30163	6.64182, 6.35644, 3.61556	6.97155, 6	3	GSG1L2	6666
GL172891.1	738376	739089	738857, 738489, 738476	10, 7, 6	10.26482, 5.65148, 6.25896	6.86644, 4.42152, 4.80589	7.48624, 3	3	GSG1L2	0
GL172891.1	781684	782616	782028, 782185, 781879	8, 12, 5	8.42257, 11.86108, 5.9506	5.97763, 7.51216, 4.54784	5.76336, 9	3	NA	9846
GL172891.1	862172	862675	862526, 862215, 862210	7, 12, 6	4.94394, 8.08118, 5.53406	3.97064, 5.18359, 4.40503	2.62735, 5	3	NA	3508
GL172891.1	866295	866639	866478, 866461, 866592	9, 14, 7	6.96255, 10.08648, 6.2897	5.00603, 6.00146, 4.81137	4.44307, 7	3	NA	7631
GL172891.1	889710	890345	889826, 889780, 889804	25, 30, 26	26.86394, 28.66215, 30.24261	12.90457, 12.15443, 14.56166	23.43188,	3	ndel1	2059
GL172891.1	890560	890803	890644, 890657, 890729	11, 8, 9	9.43668, 4.79495, 7.79368	6.22151, 3.75444, 5.52485	6.7178, 2.6	3	ndel1	2909
GL172891.1	946839	947784	947371, 947495, 947029, 947692	14, 21, 6, 6	11.75167, 14.60677, 5.14244, 4.96699	7.02514, 6.97222, 4.17298, 4.06589	8.88383, 1	3	myh10	0
GL172891.1	951782	952155	952107, 952115, 952126	25, 21, 7	25.48561, 16.89016, 6.11595	12.0796, 8.26676, 4.70713	22.0906, 1	3	myh10	1172
GL172891.1	981442	982170	981783, 981578, 981826	12, 7, 11	11.2086, 4.5832, 12.78973	7.12098, 3.73555, 8.15619	8.36821, 2	3	mfsd6l	103
GL172891.1	1008930	1009196	1008977, 1009118, 1008979	6, 8, 3	4.80449, 6.55376, 3.47371	3.96512, 4.88226, 3.03189	2.51418, 4	3	pik3r6	0
GL172891.1	1017837	1018213	1018057, 1018065, 1018055	19, 35, 23	19.03618, 37.7818, 27.91582	10.14307, 15.51397, 14.18285	15.83964,	3	pik3r6	4265
GL172891.1	1048456	1048952	1048506, 1048491, 1048553	6, 7, 4	5.96715, 5.2482, 4.67325	4.64927, 4.16649, 3.78987	3.52158, 3	3	pik3r5	0
GL172891.1	1061778	1062335	1061831, 1061806, 1061800	9, 12, 16	8.88537, 11.1491, 19.16077	6.17591, 7.08489, 10.95167	6.19961, 8	3	pik3r5	4110
GL172891.1	1070652	1070958	1070861, 1070818, 1070920	6, 12, 7	5.24248, 10.78353, 8.42533	4.2332, 6.86188, 5.94313	2.89962, 8	3	ntn1	6742
GL172891.1	1097859	1098719	1098278, 1098159, 1098141	11, 24, 12	11.71035, 24.5441, 13.7434	7.57196, 11.86236, 8.57827	8.8442, 21	3	ntn1	0
GL172891.1	1240955	1242037	1241369, 1241368, 1241269	17, 25, 11	17.33887, 25.42729, 13.19894	9.67746, 12.04472, 8.36554	14.20638,	3	NA	5022
GL172891.1	1265032	1265537	1265147, 1265092, 1265129	14, 14, 8	14.38232, 11.58379, 7.63508	8.62271, 6.92156, 5.53536	11.37721,	3	arhgap44	0
GL172891.1	1283563	1284004	1283613, 1283650, 1283869	5, 6, 5	4.50727, 4.30294, 5.9506	3.80664, 3.64568, 4.54784	2.30125, 2	3	arhgap44	0
GL172891.1	1297601	1297890	1297705, 1297771, 1297687	39, 39, 17	43.81178, 40.85774, 18.88718	17.33713, 15.63093, 10.59105	40.0499, 3	3	arhgap44	8007
GL172891.1	1324944	1325633	1325000, 1325039, 1325264	6, 7, 8	5.16999, 5.2482, 8.4586	4.18963, 4.16649, 5.99709	2.83325, 3	3	NA	8254
GL172891.1	1330898	1331446	1330911, 1330965, 1330977	16, 35, 14	16.99938, 37.58854, 17.2091	9.72433, 15.40089, 10.19524	13.87966,	3	NA	2441
GL172891.1	1432503	1433115	1432533, 1432698, 1432986	7, 6, 3	6.94335, 4.93312, 3.47371	5.18876, 4.04501, 3.03189	4.42528, 2	3	tekt3	2438
GL172891.1	1448151	1448502	1448343, 1448334, 1448403	8, 7, 8	7.1122, 5.2482, 9.25066	5.22529, 4.16649, 6.40569	4.57946, 3	3	tekt3	2007

GL172891.1	1470015	1470554	1470070, 1470093, 1470393	6, 13, 7	5.03089, 10.51368, 6.83761	4.10511, 6.48562, 5.12939	2.70624, 7	3	pmp22	1731
GL172891.1	1490214	1490643	1490240, 1490233, 1490259	33, 25, 11	38.20358, 26.2541, 10.28376	16.59192, 12.53961, 6.7395	34.53099,	3	pmp22	21930
GL172891.1	1562159	1562616	1562589, 1562590, 1562608	9, 20, 11	7.97705, 20.53147, 12.41242	5.637, 10.74631, 7.95707	5.36577, 1	3	hs3st3a1	6267
GL172891.1	1630693	1631753	1631032, 1630872, 1630986	15, 11, 21	16.63109, 9.52738, 24.37336	9.72648, 6.27759, 12.7241	13.52931,	3	NA	22271
GL172891.1	1636195	1636914	1636667, 1636737, 1636601	12, 29, 16	13.8483, 36.82147, 20.18713	8.63436, 17.33575, 11.49707	10.86694,	3	NA	27773
GL172892.1	202161	202917	202299, 202462, 202551	5, 7, 5	4.27048, 5.82304, 5.69847	3.6666, 4.52813, 4.4331	2.09564, 3	3	S1PR3	49085
GL172893.1	761416	761808	761502, 761724, 761466	10, 9, 8	8.48936, 6.76868, 7.76694	5.80661, 4.88312, 5.6116	5.82603, 4	3	ccdc30	0
GL172893.1	1401962	1402367	1402276, 1402111, 1402011	5, 7, 11	4.82177, 5.97725, 14.75772	3.98509, 4.62287, 9.09568	2.51418, 3	3	zbtb40	138851
GL172894.1	1133192	1134152	1133351, 1133648, 1133931	21, 17, 10	21.48784, 15.13134, 12.61444	11.01327, 8.33865, 8.08336	18.20998,	3	NA	99114
GL172894.1	1416798	1417407	1417060, 1417175, 1417243	25, 60, 25	25.89704, 63.9799, 30.2597	12.32584, 19.17674, 14.91057	22.49081,	3	il13ra2	29771
GL172895.1	582722	584100	583222, 583458, 583367	12, 13, 9	8.94411, 9.33876, 7.79368	5.71985, 5.75838, 5.52485	6.25442, 6	3	kitlg	59695
GL172895.1	584131	584749	584582, 584556, 584475	10, 11, 11	6.95336, 7.38317, 10.10247	4.83987, 4.93575, 6.62982	4.43454, 4	3	kitlg	59046
GL172896.1	25	892	387, 511, 369	8, 12, 4	8.42257, 11.72254, 4.67325	5.97763, 7.42986, 3.78987	5.76336, 9	3	prdm14	84917
GL172896.1	571857	572915	572522, 572366, 572343	23, 23, 15	24.58488, 21.50896, 15.88211	12.225, 10.38076, 9.29347	21.21468,	3	msc	37228
GL172896.1	934365	934951	934747, 934769, 934751	9, 8, 4	9.72082, 7.07159, 4.67325	6.64182, 5.20073, 3.78987	6.97155, 4	3	SBSPON	0
GL172896.1	1492929	1494106	1493737, 1493580, 1493636	6, 12, 5	5.31708, 10.44309, 5.40789	4.2777, 6.65248, 4.29296	2.96727, 7	3	PI15	21371
GL172896.1	1541137	1541631	1541257, 1541443, 1541374	11, 10, 7	8.4016, 7.90132, 6.59055	5.5752, 5.43931, 4.98809	5.76158, 5	3	PI15	8444
GL172897.1	306714	307797	307238, 307315, 307262	12, 23, 15	10.78431, 20.30715, 16.30311	6.86236, 9.66824, 9.53825	7.9765, 17	3	itgbl1	0
GL172898.1	862284	862840	862550, 862547, 862540	17, 24, 9	18.91905, 25.7315, 10.01275	10.6095, 12.57396, 6.79683	15.72695,	3	NA	43251
GL172899.1	93334	93837	93811, 93805, 93794	19, 21, 14	19.64557, 22.22847, 16.46576	10.50977, 11.45784, 9.80275	16.42918,	3	ipmk	8585
GL172899.1	290940	291978	291369, 291371, 291279	12, 21, 10	9.86028, 15.98701, 10.1418	6.29117, 7.74603, 6.79622	7.1041, 13	3	tfam	75632
GL172899.1	391224	392227	391589, 391589, 391488	21, 34, 15	21.64688, 31.92732, 16.23099	11.10892, 12.54908, 9.49656	18.3637, 2	3	bicc1	54975
GL172899.1	452466	453152	452764, 452776, 452756	12, 8, 13	13.73771, 5.84201, 14.93222	8.57523, 4.43168, 9.10541	10.76705,	3	bicc1	0
GL172899.1	522819	523360	523021, 523271, 523330	4, 7, 5	3.74195, 5.97725, 5.9506	3.32091, 4.62287, 4.54784	1.6222, 3.6	3	bicc1	51565
GL172899.1	683022	683982	683329, 683329, 683402	15, 13, 8	18.26603, 13.14514, 9.92329	10.62691, 8.09002, 6.7228	15.09729,	3	fam13c	15242
GL172899.1	703506	703938	703595, 703588, 703589	18, 23, 13	19.34385, 23.42245, 14.39632	10.60746, 11.52766, 8.81006	16.13713,	3	fam13c	35726
GL172899.1	729040	729625	729312, 729168, 729289	6, 13, 9	5.24248, 10.89905, 9.14171	4.2332, 6.72447, 6.32219	2.89962, 8	3	fam13c	61260
GL172899.1	748329	748898	748836, 748816, 748803	10, 15, 4	11.06011, 14.33606, 4.67325	7.306, 8.37011, 3.78987	8.22712, 1	3	NA	65668
GL172899.1	798047	799073	798594, 798594, 798593	41, 56, 30	43.83385, 55.76789, 33.30346	16.48361, 16.66922, 14.88625	40.07173,	3	NA	15493
GL172899.1	885012	885688	885313, 885257, 885290	5, 11, 4	4.50727, 10.42613, 4.67325	3.80664, 6.8251, 3.78987	2.30125, 7	3	NA	1112
GL172899.1	910214	910668	910642, 910660, 910544	8, 12, 6	7.62407, 10.49031, 6.6567	5.52896, 6.6816, 5.00845	5.03638, 7	3	ccdc6	0
GL172899.1	1105591	1106119	1106050, 1106064, 1106086	11, 31, 17	9.48173, 34.36436, 22.90084	6.24938, 15.12269, 12.74458	6.76069, 3	3	ank3	49742
GL172899.1	1116552	1117167	1116640, 1117093, 1116918	12, 16, 4	11.2086, 15.94397, 4.46488	7.12098, 9.09593, 3.69425	8.36821, 1	3	ank3	60703
GL172899.1	1136697	1136995	1136728, 1136718, 1136740	6, 34, 10	5.96715, 40.99871, 10.47831	4.64927, 17.83277, 6.98694	3.52158, 3	3	ank3	80848
GL172899.1	1398537	1399134	1398907, 1398907, 1398908	29, 39, 17	30.1581, 41.41521, 19.46435	13.4025, 15.94952, 10.92232	26.64715,	3	rhobtb1	30062
GL172899.1	1412944	1413558	1413243, 1413244, 1413244	33, 45, 13	38.32148, 49.0113, 15.17843	16.66185, 17.68126, 9.23814	34.64691,	3	rhobtb1	44469
GL172899.1	1466643	1467532	1466687, 1466695, 1466700	24, 58, 19	25.81478, 59.50755, 21.18265	12.62377, 17.74028, 11.42143	22.41065,	3	tmem26	7196
GL172900.1	170	579	361, 370, 357	15, 29, 17	5.74716, 13.05852, 9.05152	3.41198, 4.81476, 4.81376	3.3562, 10	3	NA	2405
GL172900.1	782	2608	1368, 915, 1580	25, 38, 21	13.1793, 20.21169, 12.13951	5.44461, 6.15815, 5.67128	10.23498,	3	NA	376
GL172900.1	26515	27485	26972, 27112, 26973	6, 22, 10	5.47306, 19.65261, 11.6791	4.36955, 9.59468, 7.62925	3.10811, 1	3	NA	22933
GL172900.1	29370	30063	29752, 29832, 29484	5, 9, 6	4.54329, 6.34719, 6.5517	3.82752, 4.61437, 4.95623	2.33, 3.998	3	NA	25788
GL172900.1	212868	213959	213488, 213248, 213321	16, 22, 10	14.97744, 20.00368, 10.1418	8.51025, 9.80373, 6.79622	11.94792,	3	pth2r	39460
GL172900.1	615928	616610	616307, 616355, 616146	15, 11, 7	16.72085, 10.60895, 8.35502	9.7776, 6.9343, 5.9108	13.61562,	3	NA	13584
GL172900.1	771706	772754	772600, 772640, 771735, 772597	31, 42, 11, 27	25.84374, 35.04517, 7.56293, 26.04768	10.24269, 11.46138, 5.04837, 11.707	22.43881,	3	crygd	4063
GL172900.1	1580387	1581942	1581234, 1580976, 1580990	18, 30, 21	8.91687, 16.52505, 14.80775	4.581, 6.03069, 7.08305	6.22985, 1	3	MDH1B	29772
GL172900.1	1580387	1581947	1581807, 1580976, 1580990	20, 30, 21	9.32565, 16.52505, 14.80775	4.47822, 6.03069, 7.08305	6.61397, 1	3	MDH1B	29772
GL172900.1	1585550	1586708	1586500, 1586452, 1586026	34, 35, 21	29.11562, 26.46553, 15.57205	11.02092, 9.38252, 7.51038	25.62925,	3	MDH1B	34935
GL172902.1	34	638	239, 234, 250	6, 16, 7	5.96715, 16.59459, 7.61369	4.64927, 9.48505, 5.54854	3.52158, 1	3	ccdc122	233767
GL172902.1	303186	305045	304469, 304938, 304446	26, 19, 31	20.67847, 11.3922, 25.95551	8.96301, 5.67906, 10.30262	17.42682,	3	lacc1	38120
GL172902.1	306109	306866	306493, 306414, 306353	12, 17, 13	6.511, 9.796, 7.52536	4.23097, 5.21202, 4.66214	4.02556, 7	3	lacc1	41043
GL172902.1	308690	309385	309145, 309152, 308981	20, 22, 20	13.2003, 12.34725, 13.58756	6.43821, 5.58516, 6.65072	10.25537,	3	lacc1	43624
GL172902.1	313257	316679	314087, 314042, 313368, 314030	26, 38, 11, 27	17.1207, 28.15527, 5.54706, 22.43193	7.09859, 9.37353, 3.80929, 9.6313	13.99654,	3	lacc1	48191
GL172902.1	338211	338804	338774, 338755, 338244	9, 17, 6	9.12009, 18.19786, 7.29403	6.30997, 10.18828, 5.30581	6.42028, 1	3	serp2	27291
GL172902.1	662237	663141	662582, 663000, 662894	9, 9, 5	9.72082, 7.35794, 5.06966	6.64182, 5.25474, 4.11933	6.97155, 4	3	adgra1	14088
GL172902.1	745339	745869	745793, 745790, 745784	14, 15, 9	12.53762, 13.97777, 10.14484	7.50905, 8.15212, 6.86555	9.62265, 1	3	adgra1	0
GL172902.1	771369	771931	771510, 771510, 771510	17, 34, 14	19.91381, 41.26295, 19.30655	11.176, 17.98894, 11.20466	16.68708,	3	adgra1	20445
GL172902.1	1426807	1427391	1427120, 1427007, 1427236	4, 13, 7	3.74195, 11.36332, 8.02489	3.32091, 7.01137, 5.75426	1.6222, 8.6	3	XB-GENE-5817456	360807
GL172902.1	1521335	1522046	1521626, 1521488, 1521600	15, 27, 7	15.87516, 24.42739, 7.10639	9.2894, 10.7625, 5.27894	12.80377,	3	XB-GENE-5817456	455335
GL172902.1	1575749	1576224	1576012, 1576009, 1576082	10, 11, 7	9.18236, 10.26356, 7.50542	6.23091, 6.72731, 5.49244	6.47811, 7	3	XB-GENE-5817456	509749
GL172903.1	31669	32442	31744, 32142, 32145	6, 18, 12	5.16999, 16.23409, 13.51536	4.18963, 8.73298, 8.45502	2.83325, 1	3	wdr81	16726
GL172903.1	316440	317371	316501, 316626, 317027	6, 7, 7	5.31708, 5.36631, 7.01416	4.2777, 4.24177, 5.22813	2.96727, 3	3	rpa1	93209
GL172903.1	843528	844074	844037, 843623, 843637	7, 12, 9	6.16939, 9.54462, 10.07825	4.73936, 6.09452, 6.83102	3.70916, 6	3	DPH1	0
GL172903.1	1395339	1396259	1395681, 1396080, 1396067	9, 11, 14	8.20841, 9.13706, 17.40994	5.77703, 6.0355, 10.29833	5.58112, 6	3	XB-GENE-5723327	630
GL172905.1	460945	463890	462517, 462583, 461819, 462552, 463775	18, 35, 9, 13, 5	15.46406, 35.12344, 8.43009, 13.70995, 4.33804	8.26595, 13.97479, 5.90952, 8.41989, 3.70703	12.40994,	3	atg5	2846
GL172905.1	1072214	1073198	1072772, 1072826, 1072395	11, 11, 7	11.85906, 9.8562, 7.55909	7.65504, 6.47973, 5.52035	8.98595, 7	3	sobp	0
GL172905.1	1397723	1398591	1398358, 1398011, 1397977	8, 7, 4	7.67464, 5.42726, 4.46488	5.55832, 4.28044, 3.69425	5.08299, 3	3	AFG1L	0
GL172906.1	1148838	1149245	1149020, 1148856, 1148960	13, 12, 10	14.43911, 10.35, 10.84317	8.83395, 6.59497, 7.18868	11.43182,	3	PLCE1	38638
GL172907.1	517226	517703	517390, 517493, 517435	5, 7, 4	4.69353, 5.02593, 4.30163	3.9134, 4.02367, 3.61556	2.45909, 2	3	ffar4	0
GL172907.1	780253	781047	780945, 780944, 780945	19, 35, 22	19.56679, 38.89313, 27.2393	10.46249, 16.16687, 14.08214	16.35231,	3	entpd1	21980
GL172907.1	883805	884430	884167, 884325, 884021	6, 8, 4	5.20598, 6.08299, 4.55201	4.2113, 4.58557, 3.73489	2.86702, 3	3	cpn1	7045
GL172907.1	1064552	1065036	1064705, 1064899, 1064720	7, 11, 9	6.50909, 10.60895, 10.56949	4.94073, 6.9343, 7.08032	4.02371, 7	3	abcc2	3396
GL172909.1	8	882	318, 342, 308	131, 204, 103	102.30094, 167.04103, 86.57287	13.27445, 15.19727, 14.53322	97.89091,	3	U2	0
GL172909.1	1873	2313	2056, 2055, 2061	143, 163, 105	116.07539, 118.98477, 88.98942	14.36938, 12.14248, 14.79747	111.55695	3	U2	0
GL172909.1	82569	83044	82896, 82895, 82896	22, 12, 10	24.87341, 10.16897, 10.3632	12.71848, 6.4829, 6.92219	21.49537,	3	katnbl1	1788
GL172909.1	342288	343468	342817, 342838, 342769	9, 8, 9	8.35434, 5.96021, 9.41119	5.86444, 4.50731, 6.47287	5.71584, 3	3	thbs1	188042
GL172909.1	553548	553896	553596, 553819, 553816	24, 23, 13	26.87702, 24.50192, 15.0131	13.25703, 12.17534, 9.14923	23.44461,	3	thbs1	8371
GL172909.1	574566	575511	574630, 574647, 574602	33, 47, 16	36.65024, 50.91201, 19.96942	15.67117, 17.865, 11.38369	33.00496,	3	thbs1	29389
GL172909.1	617843	618349	618306, 618075, 618139	6, 8, 7	5.51356, 7.07159, 8.69489	4.39314, 5.20073, 6.06379	3.14574, 4	3	NA	5581
GL172909.1	650919	651565	651219, 651249, 651079	9, 6, 4	9.00096, 4.51135, 4.46488	6.24222, 3.77984, 3.69425	6.30808, 2	3	NA	0
GL172909.1	727855	728557	728091, 728169, 728284	12, 12, 7	11.09909, 9.24217, 6.59055	7.05451, 5.90582, 4.98809	8.26391, 6	3	atp6v1d	0
GL172909.1	1305823	1306204	1306038, 1305986, 1306005	16, 22, 14	18.5447, 24.08291, 17.40994	10.61177, 12.25042, 10.29833	15.36536,	3	fut8	35769
GL172909.1	1405946	1406419	1406172, 1406359, 1406371	17, 25, 9	17.56639, 22.90359, 10.72164	9.81359, 10.54631, 7.15493	14.4254, 1	3	max	0
GL172909.1	1537903	1538772	1538194, 1538131, 1538148	14, 10, 4	15.84929, 9.39128, 4.67325	9.46495, 6.35644, 3.78987	12.77923,	3	zfyve26	51431
GL172909.1	1546138	1546877	1546343, 1546788, 1546594	4, 8, 4	3.74195, 7.07159, 4.67325	3.32091, 5.20073, 3.78987	1.6222, 4.6	3	zfyve26	59666
GL172910.1	248564	249295	249077, 248830, 248983	10, 10, 5	10.13706, 7.68845, 5.9506	6.7935, 5.30522, 4.54784	7.36489, 5	3	tagap	1513
GL172910.1	526309	528340	527771, 527938, 527129, 528053	14, 27, 5, 9	9.70564, 19.83181, 3.74808, 7.75727	5.77015, 8.22939, 3.34211, 5.50247	6.97127, 1	3	fuca2	0
GL172910.1	530572	531621	531022, 531146, 531390	19, 23, 12	12.6872, 12.89431, 9.88984	6.38411, 5.66395, 6.30956	9.76546, 1	3	fuca2	0
GL172910.1	532973	533852	533117, 533114, 533494	16, 21, 10	12.18769, 14.53859, 9.53891	6.80985, 6.93478, 6.44439	9.2978, 11	3	fuca2	0
GL172910.1	1108983	1109933	1109433, 1109147, 1109048	8, 17, 5	6.90289, 13.63441, 4.42467	5.09802, 7.42967, 3.75832	4.38737, 1	3	NA	6338
GL172910.1	1302816	1303928	1302929, 1303709, 1303403	15, 15, 9	14.33586, 13.21475, 9.76105	8.36999, 7.68511, 6.66343	11.33286,	3	PKIB	0
GL172910.1	1535562	1536404	1536207, 1536217, 1536214	95, 99, 68	107.7272, 108.34313, 79.8152	25.01321, 23.59978, 23.79454	103.27438	3	clvs2	58615
GL172910.1	1542346	1542801	1542528, 1542510, 1542512	90, 109, 120	101.78767, 117.64412, 145.2811	24.47833, 23.54547, 30.61099	97.38234,	3	clvs2	65399
GL172911.1	9947	10622	10486, 10332, 10146	7, 7, 7	5.33678, 3.53604, 6.6707	4.22299, 3.04509, 5.03432	2.98597, 1	3	avpr2	165872
GL172911.1	22645	23922	23459, 23242, 23243	7, 21, 9	6.05136, 19.00078, 9.76105	4.66801, 9.5157, 6.66343	3.59939, 1	3	avpr2	152572
GL172911.1	353870	354580	354212, 354169, 354398	5, 10, 5	4.81325, 6.8797, 5.9506	3.98038, 4.79317, 4.54784	2.51418, 4	3	irak1	0
GL172911.1	780913	781857	781258, 781060, 781082	8, 11, 4	6.27633, 8.13814, 3.70359	4.70812, 5.40965, 3.29934	3.80887, 5	3	flna	25456
GL172911.1	945572	946485	946000, 945925, 945972	7, 14, 3	5.65516, 11.11865, 3.44998	4.42382, 6.63466, 3.02115	3.27228, 8	3	RABGAP1	4324
GL172911.1	1166929	1167347	1167153, 1167164, 1166986	7, 10, 4	6.89193, 9.39128, 4.67325	5.15998, 6.35644, 3.78987	4.37772, 6	3	strbp	28576
GL172911.1	1176008	1176578	1176255, 1176349, 1176165	12, 16, 6	13.8483, 15.22617, 7.29403	8.63436, 8.66163, 5.30581	10.86694,	3	strbp	37655
GL172911.1	1237299	1238434	1238135, 1237684, 1237529	14, 18, 11	12.18032, 13.7776, 10.23777	7.28937, 7.25561, 6.71174	9.29157, 1	3	NA	2426
GL172911.1	1245482	1246050	1245886, 1245897, 1245898	34, 40, 36	40.99084, 44.05677, 44.00592	17.82812, 17.04299, 18.76265	37.27239,	3	NA	10609
GL172911.1	1263229	1263817	1263512, 1263451, 1263504	7, 18, 9	5.97522, 17.32425, 8.60738	4.62162, 9.39518, 6.01421	3.52869, 1	3	NA	28356
GL172911.1	1343890	1344338	1344139, 1344066, 1344218	6, 15, 9	5.90927, 14.71623, 9.8849	4.61743, 8.60008, 6.72947	3.50164, 1	3	crb2	85898
GL172911.1	1368148	1368737	1368179, 1368311, 1368284	11, 11, 7	12.17114, 10.10652, 8.69489	7.82679, 6.63228, 6.06379	9.28263, 7	3	crb2	61499
GL172912.1	647715	648716	648206, 648153, 648175	11, 18, 12	9.34791, 16.01233, 13.82167	6.16652, 8.5983, 8.62016	6.63394, 1	3	NA	46211
GL172912.1	1088737	1090169	1089608, 1089226, 1089283	16, 29, 13	16.45267, 26.95941, 14.46982	9.40062, 11.53125, 8.85107	13.35812,	3	NA	487233
GL172913.1	152278	152938	152313, 152310, 152318	87, 118, 66	106.84344, 146.40607, 88.90022	28.40194, 32.08298, 29.94955	102.39778	3	dlg2	1992
GL172913.1	157759	158116	158081, 158089, 158072	26, 44, 14	28.87987, 50.48505, 17.01463	13.75312, 18.9745, 10.0942	25.39905,	3	dlg2	0
GL172913.1	223755	224539	224354, 224346, 224430	9, 16, 7	7.6342, 16.22539, 7.72582	5.42655, 9.26486, 5.6058	5.04509, 1	3	dlg2	3215
GL172913.1	282670	283167	283122, 283088, 283141	10, 20, 9	11.06011, 21.17915, 10.87975	7.306, 11.13464, 7.23113	8.22712, 1	3	dlg2	62130
GL172913.1	289419	289807	289649, 289552, 289516	5, 12, 7	4.82177, 11.86108, 8.69489	3.98509, 7.51216, 6.06379	2.51418, 9	3	dlg2	68879
GL172913.1	409011	410256	409151, 409012, 409578	5, 6, 11	4.27048, 4.93312, 11.92945	3.6666, 4.04501, 7.69409	2.09564, 2	3	dlg2	188471
GL172913.1	537319	537771	537444, 537412, 537370	7, 10, 5	6.37715, 7.4213, 5.31857	4.86324, 5.13639, 4.24819	3.90257, 4	3	tmem126a	86003
GL172913.1	568530	569156	568572, 568634, 568681	11, 6, 4	10.72304, 4.4843, 4.04585	7.00201, 3.76254, 3.48564	7.91902, 2	3	tmem126a	54618
GL172913.1	621057	621305	621172, 621159, 621165	9, 10, 11	8.55802, 8.0878, 11.79996	5.98518, 5.55635, 7.62212	5.8909, 5.6	3	tmem126a	2469
GL172913.1	635985	637325	636504, 636648, 636497	30, 37, 22	33.70974, 39.7901, 23.02802	15.12793, 15.85208, 11.61988	30.12225,	3	tmem126a	7591
GL172913.1	643073	643760	643089, 643257, 643143	6, 7, 5	5.96715, 5.97725, 5.85847	4.64927, 4.62287, 4.50665	3.52158, 3	3	tmem126a	14679
GL172913.1	663931	664528	664308, 664364, 664469	7, 9, 5	7.1696, 8.01512, 5.9506	5.31345, 5.66016, 4.54784	4.61046, 5	3	tmem126a	35537
GL172913.1	710665	711545	711287, 711167, 710852	13, 14, 8	13.14146, 11.90127, 8.09568	8.08784, 7.11741, 5.79794	10.19842,	3	sytl2	1102
GL172913.1	746462	747230	746735, 746778, 746968	41, 62, 42	44.03036, 67.62791, 53.17887	16.59564, 20.18389, 21.48434	40.2654, 6	3	sytl2	0
GL172913.1	799809	800369	799842, 799841, 799934	13, 18, 4	15.29169, 18.67996, 4.59709	9.29854, 10.2126, 3.75554	12.24396,	3	NA	3737
GL172913.1	854227	854487	854254, 854254, 854256	62, 87, 54	69.71223, 99.75179, 69.39892	21.27817, 24.86757, 25.08079	65.61897,	3	picalm.2	0
GL172913.1	866782	867344	866922, 866788, 866830	16, 19, 11	18.05293, 20.29855, 12.9493	10.33439, 10.89984, 8.23866	14.8942, 1	3	picalm.2	0
GL172913.1	940244	940632	940550, 940384, 940517	7, 9, 4	7.1696, 8.21099, 4.67325	5.31345, 5.77858, 3.78987	4.61046, 5	3	pak1	0
GL172913.1	967538	968253	967974, 967963, 968129	26, 35, 14	25.51664, 31.12918, 13.59776	11.74635, 11.7583, 8.15458	22.12086,	3	pak1	0
GL172913.1	1233724	1234082	1234041, 1234034, 1234055	57, 92, 58	69.71925, 108.86633, 75.67071	23.72396, 26.92879, 26.63652	65.62593,	3	p2ry6	46993
GL172913.1	1259774	1260654	1260525, 1260377, 1260352	22, 39, 16	23.27367, 42.28931, 19.45311	11.76717, 16.45251, 11.10978	19.94098,	3	p2ry6	20421
GL172913.1	1267392	1267900	1267567, 1267577, 1267443	6, 17, 6	4.86712, 14.4631, 5.793	4.00414, 7.93171, 4.55271	2.55558, 1	3	p2ry6	13175
GL172913.1	1271838	1272618	1272090, 1272458, 1271953	6, 12, 7	5.27951, 8.99251, 7.29952	4.25534, 5.75004, 5.38357	2.9335, 6.4	3	p2ry6	8457
GL172913.1	1327764	1328376	1328076, 1328114, 1328102	9, 9, 4	8.40425, 8.21099, 4.67325	5.89416, 5.77858, 3.78987	5.76336, 5	3	NA	0
GL172913.1	1346740	1347277	1346976, 1346977, 1346978	15, 35, 12	15.12865, 37.3034, 16.0427	8.84764, 15.23432, 9.71071	12.09208,	3	fchsd2	3594
GL172913.1	1372544	1372993	1372644, 1372645, 1372644	18, 26, 29	19.78725, 27.92721, 36.84806	10.86837, 13.18442, 17.35105	16.56612,	3	fchsd2	0
GL172913.1	1394051	1394229	1394192, 1394163, 1394192	9, 8, 10	8.0679, 5.14361, 10.84317	5.69219, 3.9808, 7.18868	5.45, 2.913	3	fchsd2	0
GL172913.1	1400134	1400886	1400397, 1400705, 1400485	12, 23, 12	9.98821, 19.29427, 12.7555	6.37071, 9.0764, 8.03189	7.22551, 1	3	fchsd2	0
GL172913.1	1406789	1407406	1407056, 1407325, 1407131	6, 7, 4	5.06496, 5.13485, 4.67325	4.12592, 4.09383, 3.78987	2.73812, 2	3	fchsd2	0
GL172913.1	1424224	1424442	1424405, 1424412, 1424380	20, 28, 10	19.99469, 26.20518, 10.97191	10.42292, 11.44675, 7.25854	16.76522,	3	fchsd2	5673
GL172914.1	28450	28949	28753, 28587, 28583	10, 20, 16	9.28413, 17.13302, 18.3512	6.29221, 8.69853, 10.50324	6.574, 14.1	3	hmgxb3	6853
GL172914.1	101772	102453	102169, 102159, 102190	8, 8, 9	7.67464, 5.75617, 10.01275	5.55832, 4.3766, 6.79683	5.08299, 3	3	npy2r	5555
GL172914.1	666619	667386	667056, 667092, 667141	16, 26, 24	17.08121, 23.66531, 28.30626	9.7724, 10.65732, 14.09893	13.95894,	3	tnip1	0
GL172914.1	853599	854451	854103, 854245, 854393	13, 20, 10	13.00701, 19.10348, 9.2231	8.00827, 9.88447, 6.25548	10.0703, 1	3	tbc1d9b	3168
GL172914.1	1033043	1033263	1033235, 1033237, 1033234	40, 61, 52	48.01232, 71.10303, 69.53137	19.35798, 22.50476, 26.19161	44.18792,	3	rnf130	44233
GL172914.1	1046984	1047719	1047414, 1047589, 1047274	11, 6, 7	12.43693, 4.93312, 7.84208	7.97018, 4.04501, 5.66426	9.52692, 2	3	rnf130	58174
GL172914.1	1072511	1074399	1073965, 1073727, 1072826, 1073759	19, 29, 8, 20	16.92778, 25.77295, 5.67097, 19.73433	8.86687, 10.85583, 4.32182, 10.26574	13.8105, 2	3	rnf130	83701
GL172914.1	1176138	1176559	1176250, 1176263, 1176375	11, 32, 16	10.84329, 34.43067, 19.86305	7.073, 14.76315, 11.32783	8.0324, 31	3	NA	3064
GL172914.1	1254828	1255495	1254930, 1254885, 1255118	12, 32, 9	12.05017, 34.71953, 10.87975	7.62377, 14.9339, 7.23113	9.16715, 3	3	fstl4	55494
GL172914.1	1318246	1319248	1318987, 1318546, 1318920	13, 19, 8	8.87878, 11.41809, 6.15779	5.47606, 5.69283, 4.63309	6.19443, 8	3	fstl4	0
GL172914.1	1320840	1321753	1321027, 1321040, 1321023	26, 41, 17	23.37787, 35.34546, 16.3162	10.49039, 11.92603, 9.06011	20.04309,	3	fstl4	0
GL172914.1	1351236	1351695	1351670, 1351647, 1351666	15, 26, 17	16.03559, 29.08816, 19.37904	9.3831, 13.87718, 10.87373	12.95858,	3	fstl4	0
GL172914.1	1480972	1481659	1481056, 1481405, 1481199	10, 15, 9	8.79841, 12.72276, 10.72164	5.99719, 7.383, 7.15493	6.11782, 9	3	vdac1	10741
GL172915.1	41217	42456	42159, 42001, 41952	12, 18, 7	9.49573, 13.89843, 6.79502	6.06403, 7.32712, 5.10529	6.77365, 1	3	vangl2	193645
GL172915.1	56505	57449	56895, 57032, 56920	13, 16, 8	14.26982, 13.5443, 9.76268	8.73911, 7.63435, 6.64964	11.26966,	3	vangl2	178652
GL172915.1	111204	111507	111295, 111294, 111277	56, 74, 48	60.88105, 82.78331, 58.29453	19.30776, 22.49359, 21.61876	56.88344,	3	vangl2	124594
GL172915.1	127137	127826	127426, 127427, 127450	10, 9, 5	11.06011, 8.21099, 5.9506	7.306, 5.77858, 4.54784	8.22712, 5	3	vangl2	108275
GL172915.1	166365	167272	167154, 167191, 167226	42, 50, 34	26.90036, 26.82399, 23.79761	7.93306, 6.59747, 8.38577	23.46768,	3	vangl2	68829
GL172915.1	168268	168647	168380, 168381, 168380	107, 161, 89	105.61964, 155.75337, 94.13182	19.67516, 20.39476, 21.44987	101.18391	3	vangl2	67454
GL172915.1	303511	304027	303636, 303831, 303622	7, 15, 9	5.58859, 13.31811, 9.64095	4.38214, 7.74852, 6.59867	3.21321, 1	3	vangl2	15808
GL172915.1	367371	367874	367378, 367381, 367379	20, 33, 19	18.45243, 31.18003, 19.96565	9.49115, 12.49589, 10.70141	15.27752,	3	ncstn	2704
GL172915.1	368609	368946	368636, 368627, 368695	9, 13, 9	6.28237, 8.38839, 7.12591	4.57292, 5.17779, 5.10914	3.81457, 5	3	ncstn	1632
GL172915.1	443757	444203	444117, 444159, 444106	9, 16, 13	7.88835, 14.35819, 15.98838	5.58287, 8.13215, 9.66061	5.28191, 1	3	gabpb2	536
GL172915.1	459536	460729	460554, 460041, 459836	8, 21, 8	5.37674, 16.71027, 7.46678	4.13187, 8.16224, 5.43686	3.02338, 1	3	mllt11	0
GL172915.1	463159	464722	464122, 464109, 463710	17, 16, 10	13.83117, 10.50173, 9.82971	7.54848, 5.81125, 6.61563	10.856, 7.8	3	mllt11	0
GL172915.1	470563	471492	470960, 471053, 471108	13, 24, 8	11.0266, 21.01958, 8.4586	6.80342, 9.76933, 5.99709	8.20634, 1	3	mllt11	0
GL172915.1	495063	495668	495510, 495600, 495329	5, 11, 6	4.0863, 8.30667, 5.99481	3.55452, 5.51556, 4.6644	1.93351, 5	3	sema6c	10693
GL172915.1	573111	573516	573475, 573477, 573462	18, 27, 16	19.00683, 28.88794, 19.86305	10.40758, 13.39413, 11.32783	15.81175,	3	sema6c	19331
GL172915.1	621338	621877	621656, 621692, 621872	11, 15, 4	10.9046, 13.8628, 4.64325	7.10903, 8.08196, 3.77643	8.08987, 1	3	sema6c	67558
GL172915.1	688462	688884	688834, 688748, 688840	9, 8, 5	7.55321, 5.38416, 4.33804	5.37637, 4.13667, 3.70703	4.96981, 3	3	sema6c	134682
GL172915.1	689177	689508	689484, 689474, 689478	14, 20, 15	13.8308, 18.97338, 16.99143	8.29466, 9.80583, 9.93059	10.85567,	3	sema6c	135397
GL172915.1	696659	697451	696841, 697021, 697007	6, 6, 4	5.96715, 4.45754, 4.67325	4.64927, 3.74538, 3.78987	3.52158, 2	3	sema6c	142879
GL172915.1	719184	719875	719463, 719366, 719252	12, 16, 5	10.03161, 13.73836, 4.17451	6.39767, 7.753, 3.60852	7.26522, 1	3	sema6c	165404
GL172915.1	720589	721390	720700, 720700, 721030	10, 15, 8	8.0085, 12.58265, 7.46678	5.50663, 7.29694, 5.43686	5.39412, 9	3	sema6c	166809
GL172915.1	831788	832583	831818, 832020, 832088	15, 14, 16	14.33586, 12.28739, 20.88407	8.36999, 7.35528, 11.85118	11.33286,	3	tnfaip8l2	87671
GL172915.1	864385	865202	864871, 864891, 864866	19, 39, 33	18.67786, 42.28931, 41.66339	9.92659, 16.45251, 18.63289	15.49416,	3	tnfaip8l2	55052
GL172915.1	881684	882193	881996, 882073, 882149	7, 7, 9	6.46447, 5.61836, 9.8849	4.91463, 4.4008, 6.72947	3.98312, 3	3	tnfaip8l2	38061
GL172915.1	904554	905207	905132, 904841, 905067	10, 10, 6	9.661, 8.79469, 6.08001	6.51661, 5.99491, 4.71063	6.9283, 6.2	3	tnfaip8l2	15047
GL172915.1	948678	949080	949053, 949037, 949039	8, 10, 7	6.20993, 9.39128, 7.29952	4.66614, 6.35644, 5.38357	3.74679, 6	3	scnm1	0
GL172915.1	1010483	1011138	1010910, 1010877, 1010942	8, 12, 7	7.83097, 11.04188, 8.57075	5.64832, 7.01971, 6.00887	5.22917, 8	3	SCARNA4	16603
GL172915.1	1016039	1016752	1016113, 1016143, 1016102	12, 15, 6	11.15354, 12.05281, 6.82211	7.08759, 6.97189, 5.08889	8.31649, 9	3	atp1a2	13367
GL172915.1	1043178	1044682	1043953, 1044164, 1044506	6, 12, 7	5.27951, 11.1491, 6.92462	4.25534, 7.08489, 5.17829	2.9335, 8.4	3	atp1a2	0
GL172915.1	1076045	1076703	1076297, 1076185, 1076670	9, 10, 5	9.50098, 9.17452, 4.91762	6.52233, 6.22617, 4.03768	6.77745, 6	3	casq1	5526
GL172915.1	1138760	1139035	1138977, 1139008, 1139004	16, 31, 18	17.41877, 33.28323, 22.31958	9.96955, 14.48135, 12.29844	14.28292,	3	casq1	3466
GL172915.1	1284237	1284753	1284572, 1284539, 1284600	7, 19, 7	5.901, 16.15772, 6.08239	4.57615, 8.40292, 4.68683	3.49615, 1	3	bnipl	1567
GL172915.1	1287725	1288166	1288021, 1287760, 1288018	11, 16, 12	9.09169, 11.98466, 11.21415	6.00724, 6.68767, 7.12434	6.39391, 9	3	bnipl	0
GL172915.1	1306634	1307205	1306891, 1306860, 1306718	10, 19, 5	10.20047, 16.41246, 5.10931	6.82978, 8.55605, 4.14026	7.42612, 1	3	prune1	0
GL172915.1	1336256	1336808	1336773, 1336786, 1336804	12, 21, 8	10.93921, 17.60201, 8.29845	6.95711, 8.68374, 5.91009	8.12344, 1	3	fam63a	2639
GL172915.1	1338297	1339505	1338760, 1338808, 1338732	9, 18, 10	6.99678, 14.14652, 10.84317	5.02768, 7.47446, 7.18868	4.47395, 1	3	dusp23	1147
GL172915.1	1347282	1347791	1347321, 1347330, 1347322	17, 26, 14	16.90454, 26.46975, 15.80333	9.41624, 12.31359, 9.43935	13.78827,	3	igsf9	1590
GL172915.1	1482036	1482722	1482277, 1482104, 1482401	21, 21, 14	22.22738, 21.03886, 16.82614	11.45719, 10.74286, 9.99514	18.92683,	3	NA	3096
GL172915.1	1495555	1496487	1496393, 1496279, 1496267	8, 11, 7	6.7444, 10.60895, 8.49722	5.00058, 6.9343, 5.97582	4.24113, 7	3	NA	16615
GL172916.1	55196	55910	55711, 55620, 55498	6, 12, 6	5.47306, 10.35, 6.08001	4.36955, 6.59497, 4.71063	3.10811, 7	3	REEP1	371
GL172916.1	279230	279462	279269, 279250, 279281	15, 32, 15	13.15346, 31.45735, 15.74774	7.64749, 13.02561, 9.21465	10.20968,	3	eif2ak3	5822
GL172916.1	308850	309313	309260, 309262, 309286	51, 61, 25	62.41394, 69.98438, 31.651	22.53827, 21.8954, 15.7065	58.4001, 6	3	eif2ak3	3646
GL172916.1	425597	426288	425612, 425664, 425610	23, 20, 14	24.23344, 20.53147, 16.82614	12.01448, 10.74631, 9.99514	20.87361,	3	foxi4.2	68582
GL172916.1	500324	500670	500460, 500533, 500482	11, 9, 6	8.50933, 6.52169, 5.8719	5.64284, 4.72584, 4.59674	5.845, 4.16	3	foxi4.2	3486
GL172916.1	502223	502436	502380, 502360, 502344	7, 6, 5	4.94394, 4.04318, 4.91762	3.97064, 3.47602, 4.03768	2.62735, 1	3	foxi4.2	5385
GL172916.1	553921	554331	554144, 554184, 554168	18, 13, 10	18.22458, 11.87216, 11.10454	9.93936, 7.32385, 7.32977	15.0589, 9	3	thnsl2	26290
GL172916.1	786115	786642	786302, 786532, 786173	7, 11, 8	6.25077, 8.44639, 8.86315	4.78814, 5.60333, 6.21039	3.78441, 5	3	st3gal5	5664
GL172916.1	789271	789678	789315, 789338, 789300	8, 18, 8	6.59314, 15.23627, 8.04677	4.90679, 8.12825, 5.77057	4.10249, 1	3	st3gal5	8820
GL172916.1	791174	792015	791516, 791210, 791620	14, 18, 6	14.10012, 15.53678, 6.2131	8.4555, 8.30996, 4.78171	11.10747,	3	st3gal5	10723
GL172916.1	1011578	1012236	1011879, 1011990, 1012080	10, 13, 11	9.95227, 12.37541, 11.49087	6.68696, 7.62989, 7.44794	7.19087, 9	3	fgfrl1	0
GL172916.1	1125194	1125666	1125645, 1125631, 1125539	27, 50, 9	29.0553, 56.10026, 10.07825	13.49404, 19.41243, 6.83102	25.57039,	3	fgfrl1	57900
GL172916.1	1148843	1149226	1149027, 1149027, 1149028	11, 17, 13	11.09349, 15.40668, 14.85247	7.21938, 8.50651, 9.06201	8.25916, 1	3	fgfrl1	81549
GL172916.1	1300575	1301092	1300908, 1300984, 1301036	13, 14, 12	13.07382, 12.38816, 13.90113	8.04786, 7.41725, 8.66246	10.13353,	3	spon2	5986
GL172916.1	1333427	1333730	1333660, 1333660, 1333660	10, 10, 6	10.6018, 8.28331, 7.29403	7.05583, 5.67853, 5.30581	7.80484, 5	3	ctbp1	28939
GL172916.1	1411117	1411797	1411744, 1411639, 1411138	5, 7, 5	4.5799, 5.2482, 5.91431	3.84863, 4.16649, 4.53172	2.3629, 3.0	3	ctbp1	6776
GL172916.1	1447463	1447969	1447780, 1447816, 1447833	12, 12, 5	12.11599, 10.30411, 4.67395	7.66241, 6.56659, 3.90232	9.23027, 7	3	ctbp1	43122
GL172917.1	33	655	400, 144, 385	9, 10, 16	9.30591, 9.39128, 20.29858	6.41439, 6.35644, 11.55461	6.59443, 6	3	api5	38743
GL172917.1	24542	24991	24874, 24875, 24875	10, 12, 7	8.89113, 10.83413, 8.08824	6.05396, 6.89288, 5.7849	6.20456, 8	3	api5	14407
GL172917.1	67974	68728	68681, 68650, 68451	7, 15, 6	6.99564, 15.33351, 6.5517	5.21786, 8.96975, 4.95623	4.47307, 1	3	ttc17	0
GL172917.1	176551	177265	176677, 176665, 176637	18, 26, 9	20.06618, 25.04516, 10.72164	11.03116, 11.46708, 7.15493	16.83343,	3	hsd17b12	1499
GL172917.1	265805	266268	265949, 266057, 266058	9, 6, 14	9.43487, 4.93312, 18.28596	6.48595, 4.04501, 10.7324	6.71665, 2	3	alkbh3	31712
GL172917.1	282251	282714	282386, 282387, 282387	58, 75, 37	65.29818, 81.39888, 46.22003	20.77185, 21.37362, 19.63637	61.25137,	3	NA	18874
GL172917.1	326186	326754	326566, 326686, 326292	7, 7, 4	6.01305, 5.05277, 4.08034	4.6447, 4.04098, 3.50363	3.56347, 2	3	accs	0
GL172917.1	413568	414799	413613, 414388, 414348	5, 10, 10	3.9168, 8.0878, 10.59656	3.44908, 5.55635, 7.05292	1.78158, 5	3	NA	32682
GL172917.1	413674	414799	414316, 414388, 414348	7, 10, 10	6.2923, 8.0878, 10.59656	4.81292, 5.55635, 7.05292	3.8235, 5.6	3	NA	32788
GL172917.1	443452	443710	443661, 443664, 443656	11, 10, 12	11.29029, 9.39128, 15.16114	7.3332, 6.35644, 9.30141	8.44541, 6	3	NA	62566
GL172917.1	449629	450105	449854, 449881, 449819	11, 17, 4	12.43693, 18.56115, 4.67325	7.97018, 10.40145, 3.78987	9.52692, 1	3	NA	68743
GL172917.1	488248	488841	488339, 488334, 488483	13, 12, 6	13.42064, 9.86753, 5.75442	8.25183, 6.29567, 4.53101	10.46414,	3	tp53i11	64329
GL172917.1	508648	509815	509100, 509525, 508833, 509131, 509486	4, 9, 6, 5, 6	3.45429, 8.21099, 5.39683, 4.39541, 5.46454	3.15498, 5.77858, 4.32487, 3.74107, 4.36458	1.5055, 5.7	3	tp53i11	43355
GL172917.1	530540	530812	530637, 530554, 530764	4, 8, 3	3.54306, 6.73981, 3.34298	3.2072, 4.99774, 2.97156	1.57602, 4	3	tp53i11	22358
GL172917.1	566759	567533	567118, 567118, 567118	13, 29, 9	12.26307, 30.36318, 10.14484	7.56188, 13.52413, 6.86555	9.36972, 2	3	tp53i11	0
GL172917.1	582550	582858	582714, 582626, 582659	7, 7, 6	5.86458, 5.78791, 5.99481	4.55375, 4.5064, 4.6644	3.46146, 3	3	tspan18	0
GL172917.1	716532	717133	716768, 716839, 716603	8, 10, 4	6.63031, 8.53104, 3.85136	4.92991, 5.83243, 3.38149	4.13604, 6	3	cd82	19273

GL172917.1	732858	733630	733504, 733550, 733472	7, 11, 4	7.1696, 10.3713, 4.64325	5.31345, 6.79219, 3.77643	4.61046, 7	3	cd82	35599
GL172917.1	883714	884249	884127, 883843, 883985	7, 10, 4	5.86458, 6.60892, 4.18775	4.55375, 4.62161, 3.55872	3.46146, 4	3	c11orf74	0
GL172917.1	915406	915834	915673, 915677, 915615	9, 8, 7	9.63662, 7.07159, 8.69489	6.59633, 5.20073, 6.06379	6.90556, 4	3	traf6	0
GL172917.1	919667	920390	920191, 919955, 919955	5, 9, 5	4.82177, 8.21099, 5.9506	3.98509, 5.77858, 4.54784	2.51418, 5	3	traf6	0
GL172917.1	927248	927743	927461, 927500, 927404	6, 11, 5	5.96715, 10.48162, 5.9506	4.64927, 6.85833, 4.54784	3.52158, 7	3	traf6	0
GL172917.1	939312	940191	939574, 939341, 939423	13, 17, 10	13.07382, 15.51996, 10.97191	8.04786, 8.57556, 7.25854	10.13353,	3	traf6	2421
GL172917.1	958031	958512	958037, 958153, 958069	19, 15, 12	21.11935, 15.7813, 14.40488	11.38438, 9.23437, 8.92522	17.85404,	3	PRR5L	1158
GL172917.1	978996	979288	979067, 979051, 979066	15, 23, 9	14.33586, 19.65454, 9.64095	8.36999, 9.28582, 6.59867	11.33286,	3	commd9	16024
GL172917.1	1006134	1006869	1006803, 1006842, 1006851	24, 31, 19	26.87702, 33.4732, 23.65931	13.25703, 14.59388, 12.82349	23.44461,	3	commd9	5179
GL172917.1	1018685	1019553	1019015, 1019018, 1019012	40, 55, 27	41.11728, 55.74756, 30.23679	15.36511, 17.0567, 14.19876	37.39678,	3	ldlrad3	0
GL172917.1	1069637	1070373	1069951, 1069970, 1069952	17, 20, 18	17.33887, 18.3522, 21.30401	9.67746, 9.43067, 11.73892	14.20638,	3	ldlrad3	306
GL172917.1	1092308	1092808	1092646, 1092497, 1092566	14, 14, 4	14.75506, 12.92044, 4.34116	8.84125, 7.74345, 3.63492	11.73423,	3	trim44	5997
GL172917.1	1122839	1123315	1123268, 1123281, 1123254	13, 28, 24	14.18705, 31.82951, 31.18195	8.69244, 14.77572, 15.73225	11.19086,	3	trim44	0
GL172917.1	1141136	1141754	1141521, 1141381, 1141381	15, 21, 15	14.33586, 21.49549, 16.08914	8.36999, 11.01787, 9.41427	11.33286,	3	trim44	9691
GL172917.1	1164977	1165441	1165402, 1165122, 1165381	22, 27, 25	22.44777, 23.77514, 28.28116	11.27125, 10.38831, 13.74932	19.1406, 2	3	fjx1	3681
GL172917.1	1180116	1180429	1180403, 1180402, 1180403	18, 41, 14	20.55486, 43.61307, 17.01463	11.31361, 16.35802, 10.0942	17.30766,	3	pamr1	0
GL172917.1	1225561	1226182	1226128, 1226088, 1226088	9, 23, 7	8.82885, 25.42337, 8.08824	6.14328, 12.72531, 5.7849	6.14693, 2	3	slc1a2	1212
GL172917.1	1335890	1336198	1336118, 1335988, 1336161	10, 8, 6	8.66311, 7.07159, 6.168	5.914, 5.20073, 4.75778	5.99101, 4	3	pdhx	0
GL172917.1	1424355	1424749	1424464, 1424628, 1424463	6, 16, 5	5.96715, 13.88785, 5.80393	4.64927, 7.84443, 4.48186	3.52158, 1	3	ehf	1869
GL172917.1	1486683	1486979	1486814, 1486855, 1486877	6, 14, 8	5.96715, 14.45897, 10.1465	4.64927, 8.66788, 6.82176	3.52158, 1	3	NA	4208
GL172918.1	1952	2270	2102, 2113, 2134	70, 100, 35	26.9565, 43.34338, 11.41568	4.80754, 5.6606, 3.71269	23.52234,	3	NA	13548
GL172918.1	2431	3373	3048, 3118, 3221	69, 84, 40	25.90853, 31.02298, 14.58084	4.67987, 4.70324, 4.19963	22.50202,	3	NA	12445
GL172918.1	3739	5196	5042, 5042, 4251, 5044	127, 103, 32, 104	74.00569, 44.9092, 9.41996, 72.26646	8.38868, 5.71415, 3.35489, 10.62691	69.86832,	3	NA	10622
GL172918.1	37407	38091	37658, 37660, 37901	13, 10, 12	9.66057, 5.45339, 10.15051	5.95697, 3.89639, 6.47146	6.9283, 3.2	3	NA	20651
GL172918.1	39646	40325	39952, 40143, 40231	7, 15, 9	5.24783, 12.44573, 8.38703	4.16625, 7.21287, 5.88392	2.90454, 9	3	NA	22890
GL172918.1	54073	56166	55648, 55013, 55517	34, 27, 27	31.51897, 21.54945, 25.27885	12.32229, 9.14535, 11.25646	27.97718,	3	NA	37317
GL172918.1	61950	63349	62270, 62367, 62936, 62384	21, 25, 10, 23	17.61764, 15.74645, 3.64012, 23.08445	8.69296, 6.64351, 2.81072, 11.32462	14.4747, 1	3	NA	45194
GL172918.1	66622	67072	66817, 66780, 66953	10, 24, 6	6.17333, 14.79391, 3.86963	4.34648, 6.38799, 3.36141	3.71266, 1	3	NA	49866
GL172918.1	72452	75359	72663, 74253, 75181	15, 25, 21	10.0086, 19.47091, 20.55907	5.73727, 8.58769, 10.45354	7.24529, 1	3	aatk	54362
GL172918.1	72691	75390	74178, 74253, 75181	22, 25, 21	17.347, 19.47091, 20.55907	8.24733, 8.58769, 10.45354	14.21403,	3	aatk	54331
GL172918.1	83549	83916	83888, 83894, 83871	7, 11, 8	4.48748, 5.69499, 5.67097	3.67278, 3.89774, 4.32182	2.28392, 3	3	aatk	45805
GL172918.1	88478	89364	88513, 88829, 88826	10, 16, 13	6.31681, 9.3411, 9.85628	4.43694, 5.15119, 6.07806	3.84718, 6	3	aatk	40357
GL172918.1	89095	90750	89299, 89965, 88826, 89764, 90447	10, 20, 13, 14, 7	7.22253, 13.99684, 9.85628, 12.33709, 5.05388	5.0105, 6.87815, 6.07806, 7.38584, 4.0417	4.65954, 1	3	aatk	38971
GL172918.1	167723	168389	168011, 168010, 168011	17, 33, 11	15.28145, 36.16878, 11.79996	8.43017, 15.38644, 7.62212	12.23845,	3	aatk	0
GL172918.1	371708	372043	372005, 372000, 372003	15, 10, 22	15.71867, 7.35725, 28.44344	9.19755, 5.09584, 14.74701	12.65377,	3	rptor	6663
GL172918.1	584264	584596	584361, 584437, 584299	6, 6, 5	4.89903, 4.92507, 5.9506	4.02393, 4.04003, 4.54784	2.58474, 2	3	nptx1	6494
GL172918.1	602361	602747	602689, 602631, 602557	10, 15, 6	11.06011, 15.33351, 7.29403	7.306, 8.96975, 5.30581	8.22712, 1	3	nptx1	4455
GL172918.1	610033	610446	610182, 610186, 610203	4, 9, 9	3.74195, 6.89892, 11.04428	3.32091, 4.96575, 7.30896	1.6222, 4.5	3	nptx1	12127
GL172918.1	816422	816987	816845, 816755, 816780	6, 8, 3	5.43318, 5.40929, 3.47371	4.34622, 4.15294, 3.03189	3.07284, 3	3	card14	4117
GL172918.1	1055261	1055508	1055318, 1055331, 1055339	46, 36, 31	50.29331, 33.4931, 34.41393	17.96098, 12.68497, 15.15214	46.43765,	3	slc16a3	4661
GL172918.1	1224886	1225419	1225288, 1225328, 1225287	9, 19, 8	8.35434, 17.23199, 8.9249	5.86444, 9.05085, 6.24211	5.71584, 1	3	mcrip1	24920
GL172918.1	1343798	1344049	1343976, 1343992, 1343933	5, 8, 5	4.82177, 6.4822, 5.91431	3.98509, 4.83755, 4.53172	2.51418, 4	3	arhgdia	10055
GL172918.1	1385650	1386373	1386241, 1386248, 1386341	13, 21, 13	12.32133, 20.25932, 15.17843	7.59717, 10.27277, 9.23814	9.42482, 1	3	NPB	2548
GL172918.1	1498683	1499639	1498901, 1498923, 1498929	15, 18, 9	9.83195, 10.1114, 5.69077	5.63304, 5.19407, 4.19398	7.07757, 7	3	myadml2	15656
GL172919.1	1151173	1151689	1151410, 1151436, 1151404	20, 30, 13	6.23177, 9.94647, 4.34132	3.15375, 3.65202, 2.91241	3.76804, 7	3	5S_rRNA	1073
GL172919.1	1152705	1153593	1153108, 1153118, 1153141	119, 151, 87	75.00996, 95.66364, 56.98718	9.32554, 9.62667, 9.47104	70.86215,	3	5S_rRNA	0
GL172919.1	1156201	1157446	1156616, 1156800, 1156798	200, 266, 163	168.92082, 233.7979, 149.51581	16.0714, 17.83374, 18.37885	164.097, 2	3	5S_rRNA	0
GL172919.1	1194819	1195664	1195370, 1195258, 1195312	13, 27, 14	13.94585, 28.17926, 15.96227	8.5554, 12.97124, 9.52765	10.95967,	3	5S_rRNA	37451
GL172919.1	1248164	1249052	1248641, 1248569, 1248738	9, 13, 7	8.35434, 11.92581, 7.34973	5.86444, 7.35664, 5.41038	5.71584, 9	3	5S_rRNA	90796
GL172920.1	81465	82358	81683, 81587, 82175	9, 10, 6	8.61068, 8.01217, 6.25896	6.01615, 5.50894, 4.80589	5.94135, 5	3	commd2	35052
GL172920.1	164027	165198	164611, 164436, 164559	7, 18, 9	6.2098, 17.19487, 9.64095	4.76362, 9.31668, 6.59867	3.74679, 1	3	wwtr1	0
GL172920.1	1451860	1452617	1452302, 1451958, 1452273	8, 17, 12	6.98506, 14.31774, 12.15007	5.14817, 7.84337, 7.68238	4.46457, 1	3	plscr1	6662
GL172921.1	51569	52231	51829, 51868, 52023	11, 13, 11	11.71035, 9.81975, 12.78973	7.57196, 6.05544, 8.15619	8.8442, 7.2	3	RUBCN	0
GL172921.1	303835	304311	304111, 304024, 304137	6, 17, 9	4.93136, 15.69342, 9.08997	4.04392, 8.68127, 6.2929	2.61555, 1	3	NA	0
GL172922.1	23769	24815	24609, 24718, 23963	6, 8, 5	5.96715, 6.93693, 5.9506	4.64927, 5.11882, 4.54784	3.52158, 4	3	KYNU	309143
GL172922.1	188894	189532	189502, 189493, 189506	23, 41, 35	24.06243, 41.80316, 42.65756	11.91191, 15.34004, 18.39291	20.70741,	3	KYNU	144426
GL172922.1	190786	191288	191172, 191183, 191179	11, 17, 11	8.69501, 12.19907, 9.07307	5.75929, 6.5743, 5.99563	6.02078, 9	3	KYNU	142670
GL172922.1	436870	437401	437294, 437300, 437261	5, 7, 5	3.61427, 5.71889, 5.85847	3.25593, 4.46356, 4.50665	1.6222, 3.4	3	KYNU	37553
GL172922.1	442742	443132	443034, 443034, 443033	21, 14, 20	21.0274, 14.45897, 25.47128	10.73595, 8.66788, 13.59302	17.7649, 1	3	KYNU	43425
GL172922.1	557530	557868	557778, 557785, 557587	9, 14, 3	9.72082, 13.26509, 3.47371	6.64182, 7.95332, 3.03189	6.97155, 1	3	KYNU	158213
GL172922.1	592169	592539	592508, 592487, 592507	17, 15, 14	19.49949, 15.05167, 18.05504	10.94229, 8.8016, 10.62048	16.28759,	3	KYNU	192852
GL172922.1	860505	860922	860867, 860872, 860850	11, 21, 14	10.3251, 20.53474, 17.61756	6.7644, 10.43888, 10.40352	7.54325, 1	3	NA	250643
GL172922.1	937740	937981	937935, 937954, 937954	18, 21, 18	20.86369, 21.49549, 24.2813	11.49013, 11.01787, 13.31378	17.60698,	3	NA	173584
GL172922.1	966322	966625	966480, 966445, 966579	8, 14, 8	8.42257, 14.45897, 10.0913	5.97763, 8.66788, 6.79758	5.76336, 1	3	NA	144940
GL172922.1	974914	975335	975032, 975210, 975174	17, 26, 9	18.55637, 28.01936, 11.39481	10.39866, 13.2395, 7.46977	15.37671,	3	NA	136230
GL172922.1	984178	984851	984497, 984511, 984470	12, 15, 7	11.37748, 12.6757, 6.88081	7.22306, 7.35409, 5.15373	8.52862, 9	3	NA	126714
GL172922.1	1096464	1096883	1096489, 1096577, 1096633	6, 11, 4	5.72604, 9.66539, 4.67325	4.51498, 6.36265, 3.78987	3.33547, 7	3	NA	14682
GL172922.1	1147214	1147694	1147285, 1147506, 1147483	6, 10, 4	5.3939, 8.98028, 4.22496	4.32314, 6.10835, 3.57747	3.0374, 6.4	3	NA	0
GL172922.1	1243306	1244186	1243662, 1243663, 1243899	11, 12, 11	11.42621, 10.16897, 11.79996	7.41109, 6.4829, 7.62212	8.57514, 7	3	NA	11173
GL172922.1	1269137	1269682	1269617, 1269669, 1269239	7, 10, 9	7.1696, 9.07627, 9.76105	5.31345, 6.1667, 6.66343	4.61046, 6	3	NA	37004
GL172922.1	1278000	1278361	1278183, 1278275, 1278248	6, 10, 8	5.3939, 8.04981, 9.92329	4.32314, 5.53254, 6.7228	3.0374, 5.5	3	NA	45867
GL172922.1	1324359	1324908	1324458, 1324680, 1324412	7, 11, 4	6.79162, 10.60895, 4.38152	5.10336, 6.9343, 3.65448	4.28406, 7	3	NA	33787
GL172922.1	1349528	1350076	1349748, 1349600, 1349933	10, 8, 3	9.60506, 6.93693, 3.47371	6.48358, 5.11882, 3.03189	6.87658, 4	3	NA	8619
GL172923.1	35492	36035	35758, 35682, 35568	5, 17, 7	4.69353, 16.69909, 8.646	3.9134, 9.29214, 6.04229	2.45909, 1	3	NA	45576
GL172924.1	138205	139626	138928, 138870, 138958	13, 16, 10	11.45737, 12.02209, 10.65691	7.06931, 6.71016, 7.08637	8.60415, 9	3	NA	18578
GL172924.1	248859	250758	249848, 249847, 249850	180, 233, 146	160.37833, 208.57407, 136.36102	17.59347, 18.27022, 18.7304	155.59521	3	5S_rRNA	0
GL172924.1	656408	657346	657025, 657025, 657025	27, 54, 27	26.77439, 56.79604, 33.75365	12.13599, 18.0142, 16.26357	23.34518,	3	NA	30282
GL172924.1	732354	732964	732672, 732789, 732682	9, 10, 3	8.20841, 7.90132, 3.24307	5.77703, 5.43931, 2.92359	5.58112, 5	3	NA	43306
GL172924.1	1047211	1047566	1047422, 1047316, 1047312	12, 15, 5	10.34679, 11.4891, 4.51488	6.59299, 6.62745, 3.81106	7.56382, 8	3	NA	29318
GL172925.1	468201	468470	468334, 468304, 468306	11, 20, 16	9.81032, 20.00323, 17.32802	6.45164, 10.42807, 9.91673	7.05644, 1	3	midn	6563
GL172925.1	1204143	1206802	1205295, 1205428, 1205730	33, 48, 20	28.35385, 37.79891, 17.4066	10.9431, 10.96848, 8.8621	24.8854, 3	3	cfd	6407
GL172925.1	1434953	1435236	1435141, 1435060, 1435081	17, 15, 14	20.70049, 14.65124, 17.83243	11.60995, 8.56088, 10.51088	17.44812,	3	timm10	48
GL172926.1	127	1152	603, 602, 262	9, 20, 9	5.02934, 12.18015, 7.75727	3.7719, 5.89175, 5.50247	2.70624, 9	3	NA	73601
GL172926.1	3249	5408	3706, 3913, 4206	24, 41, 10	16.79498, 29.89605, 6.6185	7.41053, 9.38366, 4.62767	13.68351,	3	NA	69345
GL172926.1	5034	7309	5589, 6530, 4206, 5833, 6509	11, 20, 10, 8, 9	5.44429, 11.15942, 6.6185, 5.12819, 6.0761	3.74815, 5.36591, 4.62767, 3.97079, 4.44085	3.08378, 8	3	NA	67444
GL172926.1	5539	7309	6918, 6530, 5833, 6509	13, 20, 8, 9	7.54578, 11.15942, 5.12819, 6.0761	4.67418, 5.36591, 3.97079, 4.44085	4.96336, 8	3	NA	67444
GL172926.1	224565	225880	225524, 225359, 225132	14, 26, 12	11.89097, 20.10587, 11.50726	7.11106, 8.65024, 7.30112	9.0164, 17	3	NA	17098
GL172926.1	227067	228067	227350, 227395, 227277	8, 18, 7	5.47927, 11.84204, 5.76629	4.19817, 6.13746, 4.493	3.11408, 9	3	NA	14911
GL172926.1	414719	417686	415570, 415452, 415430	20, 24, 20	14.37852, 16.46845, 17.9783	7.09272, 7.23892, 9.20537	11.37421,	3	NA	11857
GL172926.1	457240	457827	457413, 457703, 457574	19, 14, 16	19.33502, 13.38467, 18.69936	10.32316, 8.02582, 10.69793	16.12917,	3	NA	54378
GL172926.1	542152	542644	542253, 542556, 542281	26, 20, 12	27.46001, 18.00103, 13.15613	12.90514, 9.21905, 8.25729	24.01256,	3	NA	12463
GL172926.1	608848	609301	608875, 608853, 608916	25, 37, 23	29.11479, 43.62568, 31.21093	14.24195, 18.10397, 15.99223	25.62844,	3	NA	15542
GL172926.1	971758	972278	972049, 971900, 971804	6, 9, 5	5.96715, 8.21099, 5.9506	4.64927, 5.77858, 4.54784	3.52158, 5	3	NA	230
GL172926.1	1245429	1246162	1246104, 1245542, 1245588	9, 14, 13	7.24594, 11.90127, 13.27537	5.18459, 7.11741, 8.16672	4.68113, 9	3	t2r4	4308
GL172927.1	377244	378024	377272, 377881, 377486	7, 8, 4	6.69449, 6.70176, 4.46488	5.04797, 4.97421, 3.69425	4.1953, 4.3	3	dnajb11	31768
GL172927.1	499181	500067	499240, 499552, 499733	15, 11, 12	12.70921, 6.86467, 11.60888	7.37467, 4.6125, 7.36202	9.7865, 4.4	3	dnajb11	2386
GL172927.1	508731	509234	509008, 508986, 509036	9, 11, 9	7.32038, 8.17132, 8.88966	5.23124, 5.43051, 6.17838	4.75116, 5	3	dnajb11	11936
GL172927.1	643481	644481	644181, 644229, 644047	11, 21, 4	9.21796, 19.00078, 4.59709	6.08584, 9.5157, 3.75554	6.51275, 1	3	stat4	0
GL172927.1	676938	678730	677313, 678028, 677317, 678313	13, 16, 9, 12	10.98084, 13.5443, 8.98852, 12.7555	6.7751, 7.63435, 6.23511, 8.03189	8.16259, 1	3	stat4	0
GL172927.1	776766	777721	777691, 777479, 777694	11, 9, 7	10.3251, 6.6123, 8.02489	6.7644, 4.78362, 5.75426	7.54325, 4	3	stat4	59406
GL172927.1	1191541	1192503	1192002, 1192011, 1192295	11, 17, 4	11.02968, 15.35073, 4.38152	7.18222, 8.4724, 3.65448	8.20839, 1	3	tmeff2	0
GL172927.1	1237551	1239238	1238355, 1238572, 1238020	14, 28, 9	11.31081, 24.42736, 8.88966	6.75314, 10.42967, 6.17838	8.46526, 2	3	tmeff2	0
GL172928.1	97254	98166	97731, 97796, 97491	7, 9, 6	7.1696, 7.51164, 7.29403	5.31345, 5.35055, 5.30581	4.61046, 5	3	acoxl	12152
GL172928.1	279607	280168	279954, 279942, 279629	18, 22, 12	20.45461, 21.70761, 16.0427	11.25597, 10.82585, 9.71071	17.20992,	3	bcl2l11	128455
GL172928.1	792450	793190	792617, 792878, 792903	9, 6, 10	7.39636, 4.92507, 11.17239	5.27874, 4.04003, 7.36591	4.82212, 2	3	SYNDIG1	71174
GL172928.1	1412915	1413589	1413057, 1413400, 1412924, 1413413	9, 14, 8, 12	6.82879, 10.57816, 6.57059, 11.16692	4.92128, 6.30219, 4.89275, 7.09571	4.31954, 7	3	abhd12	0
GL172928.1	1412915	1413598	1413470, 1413400, 1412924, 1413413	11, 14, 8, 12	8.96891, 10.57816, 6.57059, 11.16692	5.93064, 6.30219, 4.89275, 7.09571	6.27876, 7	3	abhd12	0
GL172929.1	964189	964672	964385, 964372, 964239	10, 8, 5	10.81731, 7.07159, 5.80393	7.17456, 5.20073, 4.48186	8.00764, 4	3	NA	0
GL172930.1	46692	47583	46973, 47002, 46946	9, 9, 4	8.50607, 6.20816, 4.38152	5.95453, 4.52542, 3.65448	5.84194, 3	3	ptprm	136187
GL172930.1	506125	506542	506488, 506251, 506435	5, 7, 5	4.82177, 5.75319, 5.9506	3.98509, 4.48487, 4.54784	2.51418, 3	3	ptprm	0
GL172930.1	1434108	1434900	1434720, 1434342, 1434344	22, 16, 19	15.80649, 9.19963, 16.39631	7.38225, 5.07263, 8.54633	12.73801,	3	PIEZO2	35016
GL172931.1	350401	350718	350648, 350463, 350533	8, 7, 8	7.67464, 4.79556, 9.1831	5.55832, 3.87426, 6.37229	5.08299, 2	3	lingo1	0
GL172931.1	971262	971805	971602, 971723, 971278	5, 12, 5	4.40263, 10.78353, 5.03069	3.74533, 6.86188, 4.09861	2.21004, 8	3	rpp25	149254
GL172932.1	519546	520257	519690, 519700, 519692	27, 34, 27	27.89898, 30.36865, 32.17306	12.80416, 11.69195, 15.34473	24.44182,	3	DRD4	24318
GL172932.1	1362616	1363444	1362992, 1362726, 1362782	20, 27, 16	16.45874, 20.40114, 13.65256	8.29782, 8.52835, 7.70053	13.36386,	3	ATG2A	0
GL172932.1	1520102	1521095	1520728, 1520731, 1520490	15, 27, 8	14.09249, 25.93224, 9.1831	8.22203, 11.63911, 6.37229	11.1007, 2	3	NA	5505
GL172933.1	500661	501203	500932, 500883, 500916	7, 13, 9	7.1696, 13.06145, 11.64359	5.31345, 8.04054, 7.57973	4.61046, 1	3	pknox2	0
GL172935.1	1098123	1098364	1098328, 1098317, 1098332	11, 8, 8	11.09349, 6.17826, 8.6841	7.21938, 4.64607, 6.11715	8.25916, 3	3	NA	0
GL172935.1	1126758	1127179	1126784, 1126779, 1126760	9, 14, 11	9.72082, 12.80997, 13.1143	6.64182, 7.67593, 8.32282	6.97155, 1	3	NA	27727
GL172935.1	1247286	1249884	1247952, 1247863, 1249113	31, 49, 26	22.31161, 35.39254, 21.9175	8.43221, 9.72692, 9.65412	19.00919,	3	NA	148255
GL172935.1	1260764	1262381	1260899, 1261744, 1260911, 1261870	14, 18, 14, 12	9.89837, 11.72293, 13.23047, 10.76087	5.887, 6.07064, 7.9323, 6.84799	7.14011, 9	3	NA	161733
GL172935.1	1260772	1262381	1261552, 1261744, 1260911, 1261870	10, 18, 14, 12	6.12672, 11.72293, 13.23047, 10.76087	4.31714, 6.07064, 7.9323, 6.84799	3.67045, 9	3	NA	161741
GL172935.1	1367954	1368238	1368189, 1368106, 1368085	5, 9, 10	4.82177, 6.40439, 11.24131	3.98509, 4.65094, 7.40241	2.51418, 4	3	NA	268923
GL172936.1	41189	41792	41375, 41243, 41386	8, 11, 7	6.59314, 10.3713, 8.49722	4.90679, 6.79219, 5.97582	4.10249, 7	3	ajap1	190430
GL172937.1	713823	714829	714214, 714053, 714509, 714433	8, 7, 6, 5	7.06928, 4.28332, 3.45514, 5.54867	5.19933, 3.53849, 3.08455, 4.3619	4.5414, 2.1	3	ptprs	0
GL172937.1	1401358	1401689	1401430, 1401428, 1401558	7, 13, 10	7.1696, 13.14514, 12.71002	5.31345, 8.09002, 8.12734	4.61046, 1	3	NA	60501
GL172938.1	413693	414174	413744, 414097, 413797	12, 9, 7	10.68367, 6.15408, 6.51247	6.80062, 4.4908, 4.9427	7.88119, 3	3	NA	63061
GL172939.1	1084807	1085706	1085447, 1085294, 1085434	20, 16, 22	22.66399, 16.29763, 27.94332	12.01241, 9.30808, 14.47367	19.35097,	3	ist1	43092
GL172939.1	1409065	1409938	1409901, 1409862, 1409826	6, 11, 4	5.96715, 10.60895, 4.67325	4.64927, 6.9343, 3.78987	3.52158, 7	3	def8	14052
GL172940.1	44749	45368	44912, 44959, 44935	12, 20, 11	11.92103, 17.6097, 12.34038	7.54764, 8.98385, 7.91841	9.04508, 1	3	abca9	0
GL172940.1	202811	203080	203028, 202930, 203029	22, 25, 12	23.7162, 25.83246, 13.15613	12.03186, 12.28718, 8.25729	20.37115,	3	abca5	0
GL172940.1	253211	253792	253474, 253625, 253486	23, 35, 18	21.74207, 31.31343, 18.73766	10.51991, 11.85728, 10.24709	18.45613,	3	GPR142	25748
GL172940.1	538902	539474	539357, 539121, 539168	8, 14, 6	8.42257, 14.45897, 7.29403	5.97763, 8.66788, 5.30581	5.76336, 1	3	NA	17202
GL172940.1	565604	566171	565869, 566086, 565971	18, 11, 23	19.97207, 9.7598, 28.93723	10.97636, 6.42066, 14.76095	16.74381,	3	cpsf4l	9610
GL172940.1	621083	622042	621415, 621394, 621381	25, 33, 21	26.23835, 32.72552, 23.31688	12.53018, 13.37535, 12.10543	22.82175,	3	cdc42ep4	10712
GL172941.1	127	1192	460, 394, 427	13, 13, 8	15.29169, 13.14514, 10.1465	9.29854, 8.09002, 6.82176	12.24396,	3	NA	21232
GL172941.1	44760	47136	46666, 46846, 45340, 47077	29, 39, 15, 21	24.63578, 30.14907, 12.99426, 20.43862	10.22153, 10.02439, 7.54975, 10.3809	21.26401,	3	NA	21825
GL172941.1	122785	123899	123676, 123722, 123569	5, 8, 5	4.50727, 6.30984, 5.9506	3.80664, 4.72927, 4.54784	2.30125, 3	3	NA	42860
GL172941.1	187334	188101	188019, 187661, 187864	6, 13, 5	4.93136, 11.26714, 4.64121	4.04392, 6.95205, 3.88372	2.61555, 8	3	NA	60299
GL172941.1	198336	199066	198706, 198950, 198470	9, 5, 3	8.35434, 3.80717, 3.47371	5.86444, 3.3798, 3.03189	5.71584, 1	3	NA	49334
GL172941.1	241953	242692	242058, 242405, 242567	10, 7, 7	8.98596, 5.30663, 7.01416	6.11182, 4.20379, 5.22813	6.29482, 3	3	NA	5708
GL172941.1	378200	379231	378702, 378853, 378809	7, 10, 6	7.1696, 9.39128, 7.29403	5.31345, 6.35644, 5.30581	4.61046, 6	3	NA	5107
GL172941.1	426018	426857	426611, 426363, 426334	10, 12, 5	10.39648, 11.20359, 5.45388	6.94096, 7.11794, 4.3157	7.61003, 8	3	NA	5806
GL172941.1	438429	438944	438871, 438774, 438807	6, 19, 9	5.96715, 21.4153, 11.64359	4.64927, 11.55717, 7.57973	3.52158, 1	3	NA	4693
GL172941.1	506428	507600	506503, 507008, 506659	4, 17, 6	3.74195, 18.56115, 7.29403	3.32091, 10.40145, 5.30581	1.6222, 15	3	NA	13749
GL172941.1	506428	507600	507259, 507008, 506659	6, 17, 6	5.96715, 18.56115, 7.29403	4.64927, 10.40145, 5.30581	3.52158, 1	3	NA	13749
GL172941.1	520699	521369	520985, 520971, 521122	6, 10, 4	5.96715, 8.84031, 4.64325	4.64927, 6.02287, 3.77643	3.52158, 6	3	NA	0
GL172941.1	1035579	1036217	1035659, 1035596, 1035613	21, 26, 16	23.61699, 27.83582, 19.86305	12.28232, 13.1298, 11.32783	20.27492,	3	traf4	20746
GL172942.1	4031	4546	4464, 4278, 4149	6, 8, 4	5.31708, 5.93025, 3.82084	4.2777, 4.48816, 3.36473	2.96727, 3	3	NA	55775
GL172942.1	284217	286279	285129, 284697, 284804, 286083	7, 12, 7, 8	6.09019, 10.63474, 6.96907, 7.95096	4.69155, 6.77054, 5.20309, 5.71659	3.63504, 7	3	ifitm1	26831
GL172942.1	483211	483501	483407, 483366, 483302	7, 8, 7	5.49154, 5.93025, 7.84208	4.32107, 4.48816, 5.66426	3.12578, 3	3	tmem258	0
GL172942.1	682931	684538	683738, 684103, 683343	24, 27, 16	19.33542, 18.92405, 12.97551	8.80163, 7.7629, 7.28731	16.1293, 1	3	NA	17164
GL172942.1	760913	761660	761144, 761009, 761577	6, 9, 6	5.1345, 6.76868, 5.49907	4.16817, 4.88312, 4.38471	2.80161, 4	3	syt7	26831
GL172942.1	919566	920401	920369, 920360, 920310	42, 66, 18	50.48615, 76.19855, 21.89732	19.8983, 22.83668, 12.06835	46.62676,	3	syt7	0
GL172943.1	750993	751804	751465, 751298, 751435	33, 40, 19	32.9881, 36.17324, 20.25304	13.52649, 12.68544, 10.87277	29.41492,	3	ccdc138	764
GL172943.1	1297830	1298792	1298727, 1298586, 1298663	9, 15, 5	7.59348, 13.31811, 5.23257	5.40134, 7.74852, 4.20435	5.0069, 10	3	prrg1	0
GL172944.1	389182	390193	389658, 389562, 389704	21, 19, 10	21.80886, 18.75575, 10.65691	11.20625, 9.97369, 7.08637	18.52047,	3	bmp7.2	72280
GL172945.1	44943	45738	45443, 45519, 45125, 45520	8, 21, 4, 6	8.34715, 18.60078, 4.18775, 7.05929	5.9367, 9.27642, 3.55872, 5.20024	5.71011, 1	3	NA	34745
GL172945.1	55280	56289	55716, 56064, 55759	21, 42, 20	20.87917, 45.75071, 25.00335	10.64659, 17.14573, 13.33871	17.62177,	3	NA	24194
GL172945.1	124665	125280	124770, 125094, 125217	6, 11, 6	5.47306, 8.13814, 5.71639	4.36955, 5.40965, 4.50951	3.10811, 5	3	lgr4	4927
GL172945.1	895135	896186	895376, 895617, 895183	19, 21, 11	21.50921, 24.32709, 14.45778	11.61175, 12.69725, 8.96373	18.23049,	3	mettl15	234545
GL172945.1	1197298	1197977	1197375, 1197437, 1197376	72, 75, 59	91.11128, 90.90784, 79.12764	27.89684, 26.2087, 28.11616	86.80296,	3	kcna4	58198
GL172945.1	1338605	1339791	1338921, 1339294, 1339078	20, 19, 15	24.58631, 21.4153, 20.14748	13.10866, 11.55717, 11.5698	21.21573,	3	fshb	33927
GL172945.1	1380394	1381043	1380580, 1380635, 1380763	9, 17, 11	9.72082, 18.56115, 13.1143	6.64182, 10.40145, 8.32282	6.97155, 1	3	NA	14299
GL172945.1	1407125	1408300	1408006, 1407566, 1407491	10, 22, 12	8.36371, 19.53861, 11.87227	5.7286, 9.52696, 7.51879	5.72471, 1	3	NA	9264
GL172945.1	1414505	1415189	1415036, 1414730, 1414732	8, 12, 3	8.42257, 11.86108, 3.47371	5.97763, 7.51216, 3.03189	5.76336, 9	3	NA	16644
GL172945.1	1442071	1442970	1442559, 1442499, 1442382	14, 16, 10	16.7649, 17.16856, 13.18184	9.96273, 9.82359, 8.33771	13.6543, 1	3	NA	44210
GL172946.1	7	907	363, 355, 368	31, 52, 28	34.34075, 55.66893, 33.18495	15.10867, 18.27712, 15.57843	30.7404, 5	3	nde1	9129
GL172946.1	324008	324982	324367, 324456, 324824	6, 6, 5	5.68215, 4.93312, 5.9506	4.49007, 4.04501, 4.54784	3.29732, 2	3	thumpd1	3854
GL172946.1	759140	760492	759560, 760088, 759438, 760099	11, 16, 5, 5	9.90875, 13.93845, 5.64747, 5.31857	6.51186, 7.87539, 4.40911, 4.24819	7.14919, 1	3	NA	1239
GL172946.1	1312369	1312984	1312919, 1312795, 1312903	7, 10, 6	6.33443, 8.61706, 6.6037	4.83795, 5.8856, 4.9822	3.86259, 6	3	cpped1	452173
GL172946.1	1357107	1357458	1357184, 1357255, 1357296	7, 13, 6	5.65516, 8.90011, 4.85645	4.42382, 5.4891, 3.9975	3.27228, 6	3	cpped1	496911
GL172946.1	1365723	1367859	1367142, 1367311, 1367267	15, 26, 17	10.88041, 18.35433, 15.1943	6.25835, 7.72278, 8.37704	8.06854, 1	3	cpped1	505527
GL172946.1	1371296	1373239	1371585, 1371423, 1371826, 1373067	18, 17, 11, 10	14.3755, 10.96746, 8.93374, 10.25108	7.611, 5.86284, 5.90867, 6.85862	11.37132,	3	cpped1	511100
GL172947.1	283844	284958	284479, 284805, 284701	9, 7, 6	9.12009, 5.97725, 6.76586	6.30997, 4.62287, 5.06179	6.42028, 3	3	tctn1	0
GL172948.1	972499	972775	972743, 972727, 972744	12, 15, 8	11.73328, 14.7819, 9.46129	7.43625, 8.63964, 6.50801	8.86615, 1	3	XB-GENE-5818975	234686
GL172948.1	1171989	1173186	1172656, 1172580, 1172669	52, 76, 44	53.46518, 77.54026, 50.71724	17.10571, 19.17414, 19.10898	49.56414,	3	PLCL1	34933
GL172948.1	1371392	1372980	1372225, 1371853, 1372846, 1371585, 1372219	39, 34, 16, 14, 26	31.64364, 22.7121, 7.01071, 10.19256, 24.22551	10.73487, 7.89485, 3.91745, 6.06616, 10.98452	28.09938,	3	PLCL1	121275
GL172949.1	319506	320558	320402, 320314, 319694	5, 7, 4	4.54329, 5.21943, 3.70359	3.82752, 4.14808, 3.29934	2.33, 2.983	3	dock2	0
GL172949.1	431571	431874	431833, 431845, 431868	15, 11, 6	15.87516, 10.60895, 6.87953	9.2894, 6.9343, 5.11627	12.80377,	3	foxi1	15231
GL172949.1	444646	445671	445076, 445125, 445126	9, 28, 9	7.71701, 26.88936, 11.21577	5.47767, 11.84506, 7.38849	5.12244, 2	3	foxi1	28306
GL172949.1	604457	605045	604604, 604588, 604620	6, 13, 6	5.96715, 13.14514, 6.76586	4.64927, 8.09002, 5.06179	3.52158, 1	3	lcp2	29135
GL172949.1	768018	768426	768296, 768168, 768139	8, 9, 5	7.15567, 6.8332, 5.50079	5.25151, 4.92409, 4.33868	4.61046, 4	3	kcnip1	53838
GL172949.1	1155828	1156371	1156064, 1156063, 1156081	14, 15, 15	15.66744, 14.03607, 17.15492	9.36333, 8.18766, 10.0222	12.60415,	3	hnrnph1	504
GL172950.1	964248	965199	964373, 964346, 965047, 964371, 965099	15, 26, 11, 14, 5	11.78493, 22.89391, 6.74479, 12.12342, 3.12644	6.80797, 10.21095, 4.5382, 7.25433, 2.93307	8.9159, 19	3	ttc30a	12915
GL172951.1	124370	125234	124499, 124821, 124799	11, 20, 16	10.3251, 17.44765, 19.45311	6.7644, 8.88669, 11.10978	7.54325, 1	3	XB-GENE-5914997	0
GL172951.1	124370	125456	125242, 124821, 124799	7, 20, 16	5.58859, 17.44765, 19.45311	4.38214, 8.88669, 11.10978	3.21321, 1	3	XB-GENE-5914997	0
GL172951.1	173741	174313	174259, 174219, 174164	9, 9, 11	7.51337, 7.35794, 12.19945	5.35162, 5.25474, 7.8422	4.93225, 4	3	XB-GENE-5742361	32461
GL172951.1	248939	249683	249412, 249423, 249024	11, 6, 4	12.43693, 4.93312, 4.67325	7.97018, 4.04501, 3.78987	9.52692, 2	3	cadm3	66834
GL172951.1	331478	332029	331863, 331633, 331938	6, 8, 4	4.65447, 6.14619, 4.59709	3.87084, 4.62573, 3.75554	2.42652, 3	3	cadm3	0
GL172951.1	489864	490185	490135, 490152, 490141	9, 18, 11	8.88537, 17.19487, 12.34038	6.17591, 9.31668, 7.91841	6.19961, 1	3	cadm3	112665
GL172951.1	514302	515047	514387, 514673, 514681	6, 18, 6	5.06496, 16.46278, 5.33084	4.12592, 8.87194, 4.28587	2.73812, 1	3	cadm3	137103
GL172951.1	659533	660116	659723, 659786, 659725	8, 9, 5	7.57423, 8.10603, 5.9506	5.4999, 5.71528, 4.54784	4.98865, 5	3	ripk3	148750
GL172951.1	1009040	1009456	1009221, 1009249, 1009294	7, 11, 4	5.79309, 8.59076, 4.30163	4.5096, 5.69393, 3.61556	3.39779, 6	3	NA	3761
GL172951.1	1335789	1336665	1336367, 1336366, 1335896	7, 11, 6	5.10557, 9.71235, 5.39683	4.075, 6.39153, 4.32487	2.77482, 7	3	NA	21267
GL172952.1	102424	103190	102662, 102632, 102792	8, 7, 7	7.99469, 5.78791, 7.72582	5.74128, 4.5064, 5.6058	5.38155, 3	3	PWWP2B	96422
GL172952.1	296416	296939	296586, 296621, 296590	12, 12, 9	11.55224, 9.46718, 11.58206	7.3281, 6.04622, 7.55286	8.69537, 6	3	inpp5a.1	0
GL172952.1	676054	676440	676186, 676285, 676356	7, 7, 8	5.65516, 4.79556, 9.60885	4.42382, 3.87426, 6.57807	3.27228, 2	3	NA	7611
GL172952.1	841881	842232	842095, 841946, 842149	5, 8, 8	4.27048, 6.34359, 8.35103	3.6666, 4.75053, 5.93881	2.09564, 3	3	bub3	32171
GL172952.1	853313	853764	853423, 853657, 853577	8, 12, 7	8.4095, 11.86108, 8.646	5.97056, 7.51216, 6.04229	5.76336, 9	3	bub3	43603
GL172952.1	969059	969890	969387, 969241, 969647	25, 35, 13	25.48561, 32.33926, 13.21599	12.0796, 12.41465, 8.13179	22.0906, 2	3	gpr26	5463
GL172952.1	1214769	1215103	1214994, 1214969, 1214904	7, 22, 6	6.42049, 21.21624, 6.6037	4.8888, 10.53027, 4.9822	3.94161, 1	3	NA	4707
GL172952.1	1259270	1260220	1259519, 1259719, 1259780	34, 45, 23	35.11793, 44.64228, 25.12075	14.36672, 15.2796, 12.54512	31.5028, 4	3	plpp4	30106
GL172953.1	441321	442026	441775, 441650, 441907	8, 9, 4	7.88469, 8.01512, 4.67325	5.67897, 5.66016, 3.78987	5.27935, 5	3	ANKFN1	4893
GL172953.1	474776	475520	475393, 474835, 475397	10, 13, 9	8.12348, 10.23256, 8.88966	5.57869, 6.31126, 6.17838	5.50152, 7	3	ANKFN1	0
GL172953.1	588604	589220	588717, 588617, 588987	10, 13, 7	9.33595, 11.41208, 7.96278	6.32332, 7.04141, 5.72394	6.62299, 8	3	ANKFN1	11810
GL172953.1	1064492	1064984	1064550, 1064799, 1064823	5, 12, 5	4.82177, 10.88526, 5.50079	3.98509, 6.92415, 4.33868	2.51418, 8	3	mmd	10227
GL172953.1	1073386	1073660	1073634, 1073631, 1073635	23, 26, 12	26.5636, 27.83582, 14.96175	13.3989, 13.1298, 9.20442	23.13966,	3	mmd	1551
GL172953.1	1334236	1334843	1334744, 1334736, 1334754	75, 124, 89	39.18537, 67.08999, 62.37376	6.82796, 7.56561, 10.52002	35.49615,	3	tom1l1	4835
GL172953.1	1335313	1336547	1336418, 1336412, 1336379	60, 81, 33	26.64635, 31.73421, 11.70757	5.47188, 4.9733, 3.95043	23.22052,	3	tom1l1	5912
GL172953.1	1337471	1337663	1337564, 1337537, 1337565	43, 66, 29	14.16787, 21.40972, 9.16093	3.88106, 4.05726, 3.48269	11.17262,	3	tom1l1	8070
GL172954.1	802061	803743	803467, 802608, 802794	21, 24, 10	18.35044, 18.03099, 9.26679	9.12717, 8.0756, 6.28179	15.17856,	3	drd2	117261
GL172955.1	966088	967060	966686, 966512, 966558	9, 10, 8	7.10147, 7.20159, 8.35103	5.09375, 4.99723, 5.93881	4.57191, 4	3	xxylt1	22980
GL172955.1	1345845	1346474	1345996, 1346009, 1346130	30, 41, 25	32.98392, 42.32603, 31.651	14.6962, 15.63187, 15.7065	29.41083,	3	NA	3150
GL172956.1	91175	91965	91600, 91419, 91681	7, 8, 6	6.42049, 6.44699, 6.82211	4.8888, 4.8155, 5.08889	3.94161, 4	3	cnot10	0
GL172956.1	898097	898267	898128, 898116, 898128	17, 29, 13	19.49949, 31.55847, 14.85247	10.94229, 14.23483, 9.06201	16.28759,	3	snrk	0
GL172957.1	284400	284836	284601, 284699, 284725	10, 9, 6	8.93828, 6.7053, 5.91225	6.08275, 4.84282, 4.61907	6.24946, 4	3	rasa3	0
GL172957.1	902130	902764	902304, 902421, 902354	9, 22, 8	6.79611, 16.19434, 7.26744	4.90054, 7.5968, 5.31857	4.28827, 1	3	tm2d3	26584
GL172957.1	903937	905610	904374, 905228, 904098, 905255	8, 22, 10, 6	6.27633, 17.08543, 10.03522, 5.2665	4.70812, 8.09812, 6.73493, 4.24757	3.80887, 1	3	txndc9	26964
GL172957.1	903937	905635	904949, 905228, 904098, 905255	10, 22, 10, 6	8.40516, 17.08543, 10.03522, 5.2665	5.75437, 8.09812, 6.73493, 4.24757	5.76336, 1	3	txndc9	26939
GL172957.1	915111	916550	915706, 915720, 915539, 916032	17, 26, 8, 4	14.84969, 21.70642, 8.4586, 3.67542	8.16701, 9.53509, 5.99709, 3.28338	11.82512,	3	txndc9	16024
GL172957.1	1135976	1137295	1136845, 1136450, 1137146	21, 32, 7	21.80886, 32.56757, 8.49722	11.20625, 13.66912, 5.97582	18.52047,	3	mgat4b	20220

GL172958.1	110651	110951	110713, 110678, 110725	9, 16, 12	9.24298, 15.67391, 14.23156	6.3792, 8.93306, 8.83588	6.53474, 1	3	mapkap1	0
GL172958.1	276489	276788	276729, 276564, 276708	11, 8, 5	11.09349, 5.96021, 5.69847	7.21938, 4.50731, 4.4331	8.25916, 3	3	pbx3	52413
GL172958.1	458633	459137	459073, 458990, 458991	9, 21, 11	7.17299, 19.60696, 13.1143	5.13877, 9.87975, 8.32282	4.61317, 1	3	pbx3	108108
GL172958.1	485778	486514	486026, 485949, 485998	15, 18, 10	18.26603, 19.97713, 13.18184	10.62691, 10.97931, 8.33771	15.09729,	3	pbx3	135253
GL172958.1	508485	508932	508733, 508647, 508603	9, 5, 7	9.72082, 3.94561, 8.69489	6.64182, 3.46715, 6.06379	6.97155, 1	3	mvb12b	139316
GL172958.1	540590	541078	540876, 540848, 540823	6, 6, 5	5.96715, 4.93312, 5.9506	4.64927, 4.04501, 4.54784	3.52158, 2	3	mvb12b	107170
GL172958.1	982754	983398	983161, 983174, 983163	21, 23, 11	22.94132, 24.15665, 12.71187	11.88295, 11.96843, 8.11558	19.61877,	3	lmx1b.1	49290
GL172958.1	1031086	1031567	1031290, 1031294, 1031418	13, 16, 5	12.26307, 16.0832, 5.27517	7.56188, 9.17962, 4.22616	9.36972, 1	3	srpk2	73724
GL172958.1	1155877	1156323	1155993, 1156009, 1156119	8, 8, 6	7.47668, 5.99047, 6.03707	5.44269, 4.52662, 4.6874	4.89713, 3	3	pdzd4	1075
GL172958.1	1242304	1242947	1242741, 1242643, 1242661	18, 20, 15	16.27432, 17.88727, 16.01939	8.75742, 9.1506, 9.37366	13.18709,	3	l1cam	3702
GL172959.1	26125	26592	26326, 26429, 26453	11, 17, 9	9.90875, 15.07756, 11.21577	6.51186, 8.30587, 7.38849	7.14919, 1	3	CCDC169	0
GL172959.1	139601	140181	140179, 140138, 140179	14, 18, 16	12.91867, 17.65791, 19.75829	7.74237, 9.59733, 11.27252	9.98624, 1	3	ccna1	1583
GL172959.1	1109216	1109937	1109496, 1109467, 1109491	9, 11, 5	8.16099, 9.8562, 5.75061	5.74847, 6.47973, 4.45735	5.53692, 7	3	NA	69735
GL172960.1	333322	334223	333901, 333973, 333931	37, 57, 24	40.64214, 57.06301, 25.90456	16.3484, 16.91951, 12.67746	36.92964,	3	duox1	0
GL172960.1	758414	760178	759916, 758749, 759974, 758798	13, 14, 12, 9	12.09198, 10.76513, 8.34513, 8.88966	7.45795, 6.41704, 5.34707, 6.17838	9.20698, 8	3	NA	36627
GL172960.1	1266060	1266779	1266306, 1266682, 1266443	6, 10, 3	5.96715, 8.74956, 3.47371	4.64927, 5.96721, 3.03189	3.52158, 6	3	otud7a	0
GL172961.1	38394	38720	38644, 38695, 38664	9, 21, 10	9.72082, 23.12148, 13.18184	6.64182, 11.98982, 8.33771	6.97155, 1	3	NA	76063
GL172961.1	261253	261623	261291, 261467, 261472	10, 7, 6	11.06011, 5.97725, 6.82211	7.306, 4.62287, 5.08889	8.22712, 3	3	ppp2r1a	0
GL172961.1	343801	344163	344096, 344058, 344124	7, 10, 8	7.1696, 9.39128, 10.1465	5.31345, 6.35644, 6.82176	4.61046, 6	3	NA	7437
GL172961.1	442690	442967	442931, 442893, 442937	8, 11, 5	8.42257, 10.60895, 5.9506	5.97763, 6.9343, 4.54784	5.76336, 7	3	NA	90087
GL172961.1	576529	577357	576877, 576848, 576855	13, 9, 7	13.94585, 8.21099, 8.69489	8.5554, 5.77858, 6.06379	10.95967,	3	NA	128086
GL172961.1	606206	606844	606354, 606708, 606662	7, 13, 7	7.1696, 12.99541, 7.20145	5.31345, 8.00139, 5.33074	4.61046, 1	3	NA	98599
GL172961.1	641388	642052	641416, 641400, 641489	25, 29, 18	27.92938, 28.20633, 19.91385	13.54032, 12.25351, 10.94239	24.4711, 2	3	NA	63391
GL172961.1	754465	754816	754583, 754554, 754564	14, 22, 14	12.8072, 17.89095, 14.47197	7.67424, 8.56031, 8.67552	9.88012, 1	3	NA	22894
GL172961.1	759463	759925	759698, 759718, 759691	6, 16, 4	4.86712, 12.77959, 3.82084	4.00414, 7.16814, 3.36473	2.55558, 1	3	NA	27892
GL172961.1	928542	928805	928563, 928572, 928575	40, 70, 46	47.48727, 84.90305, 60.10484	19.0486, 25.5347, 23.65696	43.6706, 8	3	xtr-mir-100	17987
GL172961.1	934649	934938	934712, 934701, 934691	8, 18, 10	7.15567, 18.91825, 10.08818	5.25151, 10.35485, 6.76544	4.61046, 1	3	xtr-mir-100	11854
GL172961.1	942073	942565	942366, 942351, 942462	12, 13, 10	11.67221, 12.93015, 11.10454	7.39985, 7.96262, 7.32977	8.80793, 1	3	xtr-mir-100	4227
GL172961.1	957493	958114	958088, 958081, 958088	45, 67, 40	54.40503, 78.12678, 49.29565	20.79013, 23.3812, 20.11504	50.48956,	3	xtr-mir-125a	3562
GL172961.1	977784	978541	977829, 977834, 977834	12, 22, 10	11.79509, 22.77797, 12.90749	7.47301, 11.46975, 8.21675	8.92495, 1	3	XB-GENE-5727466	3407
GL172961.1	1002100	1002348	1002123, 1002127, 1002127	24, 30, 28	27.72617, 32.70957, 36.0022	13.759, 14.53308, 17.21251	24.2736, 2	3	btg5	3738
GL172961.1	1080293	1080791	1080739, 1080725, 1080749	12, 14, 8	12.82537, 14.45897, 10.1465	8.07156, 8.66788, 6.82176	9.89694, 1	3	kcnn4	17285
GL172961.1	1154872	1155342	1154925, 1155087, 1154908	16, 18, 13	15.60786, 15.09059, 14.61978	8.89312, 8.04034, 8.93426	12.5472, 1	3	hspa1a	1165
GL172961.1	1192605	1193137	1192906, 1192921, 1192643	6, 15, 5	5.47306, 15.33351, 5.9506	4.36955, 8.96975, 4.54784	3.10811, 1	3	smg9	15908
GL172961.1	1206717	1207442	1207412, 1207391, 1207407	38, 67, 34	45.40854, 81.38612, 44.97678	18.7177, 25.14926, 20.15704	41.62352,	3	smg9	30020
GL172961.1	1267175	1267755	1267343, 1267333, 1267324	8, 9, 7	8.42257, 8.21099, 8.69489	5.97763, 5.77858, 6.06379	5.76336, 5	3	NA	10733
GL172961.1	1304626	1305139	1305045, 1304865, 1305034	10, 16, 12	11.06011, 17.16856, 16.36823	7.306, 9.82359, 9.85365	8.22712, 1	3	NA	24049
GL172961.1	1316999	1317527	1317329, 1317343, 1317291	8, 7, 6	8.42257, 5.97725, 7.29403	5.97763, 4.62287, 5.30581	5.76336, 3	3	NA	36422
GL172962.1	105	2730	372, 880, 885	28, 32, 24	22.68557, 24.65519, 20.49057	9.46481, 9.32534, 9.46218	19.37165,	3	aldh5a1	42192
GL172962.1	105	2730	1707, 880, 885	28, 32, 24	20.40284, 24.65519, 20.49057	8.25934, 9.32534, 9.46218	17.16039,	3	aldh5a1	42192
GL172962.1	105	2760	2493, 880, 885	13, 32, 24	6.35419, 24.65519, 20.49057	3.98727, 9.32534, 9.46218	3.881, 21.4	3	aldh5a1	42162
GL172962.1	5601	6134	5892, 5849, 5862	15, 24, 12	8.87716, 13.44123, 7.37193	5.0794, 5.73842, 4.7483	6.19288, 1	3	aldh5a1	38788
GL172962.1	9337	10450	10326, 10208, 9915	12, 24, 13	8.20776, 16.46845, 9.95131	5.26191, 7.23892, 6.13691	5.58106, 1	3	aldh5a1	34472
GL172962.1	13911	14714	14021, 14515, 14346	9, 11, 12	5.76179, 5.36821, 9.24622	4.23947, 3.70305, 5.90835	3.36995, 3	3	aldh5a1	30208
GL172962.1	21982	22972	22586, 22154, 22329	28, 40, 29	24.37258, 33.78757, 28.37675	10.3988, 11.47552, 12.35305	21.00877,	3	aldh5a1	21950
GL172962.1	25349	26513	25952, 26212, 26145	17, 14, 13	12.13217, 7.66381, 9.64122	6.53504, 4.56876, 5.94502	9.24555, 5	3	aldh5a1	18409
GL172962.1	33787	34831	34619, 34461, 34346	40, 42, 32	31.17835, 28.17886, 26.07091	10.22451, 8.43505, 10.05259	27.64458,	3	aldh5a1	10091
GL172962.1	36457	38753	36901, 37481, 36779	19, 36, 21	9.7642, 21.70764, 13.97329	4.84218, 7.05756, 6.6274	7.01379, 1	3	aldh5a1	6169
GL172962.1	585336	586550	585751, 585681, 586075	15, 18, 16	13.5229, 14.02138, 16.90298	7.87405, 7.40006, 9.66757	10.56184,	3	prl.1	93629
GL172962.1	1121847	1122739	1121974, 1122453, 1122129	11, 16, 9	10.06049, 14.19759, 10.35155	6.60432, 8.03393, 6.97128	7.29334, 1	3	sox4	67501
GL172963.1	42	1584	1192, 147, 1069	6, 11, 7	5.90927, 10.59463, 7.45266	4.61743, 6.92577, 5.46481	3.50164, 7	3	NA	85806
GL172963.1	365718	366038	365822, 365845, 365832	15, 12, 8	15.56593, 9.42893, 7.76694	9.10751, 6.02236, 5.6116	12.50679,	3	dot1l	174
GL172963.1	436468	436871	436673, 436486, 436482	6, 9, 8	5.03089, 7.75488, 8.80246	4.10511, 5.501, 6.179	2.70624, 5	3	ap3d1	0
GL172964.1	7512	7923	7873, 7774, 7861	7, 17, 6	5.42862, 15.51996, 5.99481	4.2813, 8.57556, 4.6644	3.06982, 1	3	NA	4517
GL172964.1	126694	127017	126860, 126860, 126860	12, 11, 5	12.82537, 9.30635, 5.64747	8.07156, 6.14075, 4.40911	9.89694, 6	3	adgrf1	19937
GL172964.1	317178	317849	317292, 317611, 317287	11, 17, 6	10.66406, 15.24016, 6.35308	6.96705, 8.40499, 4.85498	7.86396, 1	3	pla2g7	374
GL172964.1	338153	338898	338502, 338281, 338495	9, 11, 8	9.72082, 10.60895, 10.1465	6.64182, 6.9343, 6.82176	6.97155, 7	3	pla2g7	0
GL172964.1	436235	436888	436599, 436582, 436795	5, 7, 5	4.69353, 5.27726, 5.40789	3.9134, 4.18506, 4.29296	2.45909, 3	3	cyp39a1	5016
GL172964.1	440819	442216	441234, 441769, 441246	7, 10, 8	6.60038, 8.0878, 9.53432	4.99378, 5.55635, 6.54285	4.10761, 5	3	cyp39a1	9600
GL172964.1	462852	463327	463164, 463209, 463236	6, 11, 5	4.35354, 8.10521, 4.91762	3.67842, 5.38896, 4.03768	2.16915, 5	3	rcan2	0
GL172964.1	478958	479715	479410, 479115, 479239	18, 28, 17	16.44776, 25.94264, 17.65167	8.86281, 11.29482, 9.86447	13.35395,	3	rcan2	0
GL172964.1	657954	658511	657984, 658028, 657982	57, 70, 29	67.75444, 77.00881, 34.61941	22.61046, 21.40331, 16.05212	63.68127,	3	clic4	4212
GL172964.1	695263	695985	695925, 695376, 695471, 695753	9, 12, 5, 4	9.36986, 10.39632, 5.59755, 4.67325	6.44997, 6.6236, 4.38538, 3.78987	6.65425, 7	3	clic4	41521
GL172964.1	790502	791092	790560, 790577, 790898	9, 15, 10	7.88835, 12.26789, 10.3632	5.58287, 7.10374, 6.92219	5.28191, 9	3	runx2	85764
GL172964.1	790502	791131	791007, 790577, 790898	12, 15, 10	11.43506, 12.26789, 10.3632	7.25774, 7.10374, 6.92219	8.58315, 9	3	runx2	85725
GL172964.1	814272	814776	814595, 814556, 814650	950, 1163, 730	976.38074, 1178.96509, 758.81946	26.96949, 26.26826, 27.41693	969.66272	3	runx2	62080
GL172964.1	990668	991121	991094, 991098, 991090	75, 84, 73	92.53921, 103.2267, 101.02418	27.08499, 28.07514, 32.97009	88.21526,	3	runx2	0
GL172964.1	1122356	1122735	1122548, 1122492, 1122622	16, 20, 5	15.47689, 17.03084, 5.50079	8.81381, 8.63757, 4.33868	12.4218, 1	3	supt3h	63510
GL172964.1	1306165	1306737	1306318, 1306297, 1306206	17, 34, 20	16.42934, 35.5202, 25.00335	9.12876, 14.6006, 13.33871	13.33656,	3	supt3h	79017
GL172964.1	1315067	1315526	1315295, 1315295, 1315329	14, 22, 9	14.10012, 23.27801, 9.64095	8.4555, 11.76976, 6.59867	11.10747,	3	supt3h	87919
GL172964.1	1322271	1323381	1322774, 1322859, 1322867	18, 21, 16	19.8791, 20.32736, 19.65507	10.9221, 10.3138, 11.21774	16.65356,	3	supt3h	95123
GL172965.1	470360	470703	470536, 470533, 470566	8, 6, 4	7.28955, 4.83043, 4.67325	5.33177, 3.98131, 3.78987	4.72176, 2	3	NA	18448
GL172965.1	702585	702935	702853, 702646, 702839	9, 7, 4	9.72082, 5.97725, 4.67325	6.64182, 4.62287, 3.78987	6.97155, 3	3	NA	14330
GL172965.1	904238	904700	904375, 904491, 904664	8, 8, 6	7.1122, 6.77831, 7.29403	5.22529, 5.0215, 5.30581	4.57946, 4	3	gja3	0
GL172965.1	1190981	1191736	1191195, 1191171, 1191073	8, 15, 5	4.91051, 9.2091, 3.46822	3.82948, 5.2699, 3.16061	2.59605, 6	3	ZC3H12C	37325
GL172966.1	51672	52135	51776, 51720, 51678	24, 25, 17	25.27938, 23.81247, 18.73036	12.30321, 11.08247, 10.50006	21.89126,	3	sntb1	312466
GL172966.1	263571	264751	264233, 264233, 264323	14, 19, 7	14.3105, 17.58589, 6.6707	8.58029, 9.26517, 5.03432	11.30855,	3	sntb1	99850
GL172966.1	336667	337110	337083, 337083, 337065	21, 31, 19	22.31353, 33.09602, 23.22595	11.50874, 14.37055, 12.5858	19.0106, 2	3	sntb1	27491
GL172966.1	351678	352383	351793, 352226, 352156	11, 12, 6	9.09169, 10.93692, 6.08001	6.00724, 6.95571, 4.71063	6.39391, 8	3	sntb1	12218
GL172966.1	365365	366035	365690, 365716, 365653	33, 64, 21	29.53885, 65.70161, 21.05831	11.5844, 18.36809, 10.75458	26.04177,	3	sntb1	0
GL172966.1	390864	391395	391376, 391355, 391352	16, 15, 12	16.91852, 14.84826, 14.76955	9.67673, 8.67957, 9.10943	13.80145,	3	sntb1	0
GL172966.1	429274	429657	429579, 429598, 429602	8, 11, 7	7.15567, 9.61892, 7.84208	5.25151, 6.33404, 5.66426	4.61046, 7	3	sntb1	0
GL172966.1	432680	433060	432708, 432993, 432763	8, 14, 6	7.1122, 12.92044, 6.168	5.22529, 7.74345, 4.75778	4.57946, 1	3	sntb1	0
GL172966.1	445640	446350	446198, 446070, 445998	17, 19, 19	15.28145, 15.53291, 21.86777	8.43017, 8.02931, 11.81904	12.23845,	3	sntb1	0
GL172966.1	658677	659213	658873, 658768, 658876	13, 10, 5	12.68462, 9.028, 5.9506	7.81602, 6.13739, 4.54784	9.76298, 6	3	XB-GENE-963065	3901
GL172966.1	732117	732639	732567, 732555, 732344	7, 19, 7	6.99564, 20.1301, 8.69489	5.21786, 10.79955, 6.06379	4.47307, 1	3	mtbp	0
GL172966.1	759934	760495	760126, 760182, 760193	6, 12, 5	5.77068, 10.73343, 5.9506	4.54016, 6.83116, 4.54784	3.37745, 8	3	taf2	0
GL172966.1	809396	809878	809540, 809632, 809596	458, 475, 443	484.98022, 494.39877, 496.80536	27.88129, 26.84432, 31.93984	479.31613	3	taf2	10671
GL172966.1	919682	920731	920563, 920546, 920556	21, 31, 16	19.3971, 30.05049, 17.85991	9.75356, 12.58856, 10.22415	16.1894, 2	3	NA	2817
GL172966.1	1092240	1092827	1092757, 1092599, 1092295	13, 20, 8	13.86771, 21.43553, 9.60885	8.51067, 11.28761, 6.57807	10.88502,	3	NA	6651
GL172966.1	1130021	1130817	1130473, 1130263, 1130202	8, 17, 8	8.42257, 15.99248, 8.80246	5.97763, 8.86336, 6.179	5.76336, 1	3	mal2	25354
GL172967.1	321664	322617	322155, 322158, 322066	9, 11, 10	6.92864, 9.95469, 10.71812	4.98457, 6.5399, 7.12015	4.41243, 7	3	NA	51617
GL172967.1	330291	330775	330492, 330467, 330489	8, 18, 6	6.90289, 18.15157, 6.87953	5.09802, 9.89541, 5.11627	4.38737, 1	3	NA	43459
GL172967.1	363022	364320	363751, 363503, 363844	16, 30, 11	15.87778, 27.45685, 11.37266	9.05608, 11.46511, 7.38048	12.8057, 2	3	NA	9914
GL172967.1	466735	468146	467722, 467483, 466870	18, 34, 14	16.80745, 30.24646, 13.49044	9.0814, 11.62578, 8.08981	13.69549,	3	NA	91255
GL172967.1	674959	675809	675192, 675334, 675136	10, 14, 7	8.84451, 10.76513, 6.88081	6.02544, 6.41704, 5.15373	6.16137, 8	3	igsf9b	36543
GL172968.1	151553	151958	151899, 151707, 151751	6, 9, 5	5.27951, 7.06866, 5.31857	4.25534, 5.07306, 4.24819	2.9335, 4.6	3	kcnh6	0
GL172968.1	404602	405649	405470, 405317, 405127	15, 25, 16	12.0347, 19.55909, 19.45311	6.9608, 8.63629, 11.10978	9.15318, 1	3	tanc2	44188
GL172968.1	426874	427537	427427, 426929, 427062	8, 7, 4	7.47668, 5.30663, 4.67325	5.44269, 4.20379, 3.78987	4.89713, 3	3	tanc2	66460
GL172968.1	449761	450657	450394, 450583, 450504	13, 12, 6	13.20994, 10.39632, 6.99807	8.12823, 6.6236, 5.17195	10.26285,	3	tanc2	89347
GL172968.1	942226	942884	942770, 942805, 942764	8, 13, 8	7.99469, 13.14514, 8.62637	5.74128, 8.09002, 6.08668	5.38155, 1	3	amn	0
GL172968.1	1250891	1251452	1250999, 1251046, 1250996	7, 11, 6	5.30682, 8.17132, 5.99481	4.20391, 5.43051, 4.6644	2.95991, 5	3	NA	11136
GL172968.1	1251620	1251957	1251857, 1251847, 1251792	7, 11, 7	5.39768, 8.41104, 7.10639	4.26169, 5.58113, 5.27894	3.04019, 5	3	NA	10631
GL172969.1	58550	58766	58740, 58739, 58740	11, 18, 7	11.78412, 19.16427, 8.69489	7.61327, 10.50111, 6.06379	8.91516, 1	3	myd88	33286
GL172969.1	60720	61395	61092, 60928, 61238	8, 9, 4	7.93925, 6.6123, 4.67325	5.70996, 4.78362, 3.78987	5.33002, 4	3	myd88	35456
GL172969.1	93646	94496	93715, 94027, 93695	7, 7, 5	6.50909, 5.97725, 5.85847	4.94073, 4.62287, 4.50665	4.02371, 3	3	map3k3	27571
GL172969.1	140004	141500	141032, 140642, 141428	19, 8, 13	17.50859, 5.35923, 13.21599	9.21834, 4.12054, 8.13179	14.36919,	3	acaa1	0
GL172969.1	153144	153637	153412, 153578, 153285	9, 10, 9	6.69985, 7.4213, 7.94299	4.83936, 5.13639, 5.61625	4.20005, 4	3	dlec1	0
GL172969.1	200855	201056	200995, 200964, 200988	9, 13, 6	7.71701, 11.03409, 5.60548	5.47767, 6.80804, 4.44624	5.12244, 8	3	dlec1	18346
GL172969.1	232773	233223	233194, 233194, 233134	14, 16, 15	12.53762, 13.6406, 14.99627	7.50905, 7.69322, 8.76842	9.62265, 1	3	plcd1	0
GL172969.1	255252	255961	255649, 255667, 255265	6, 11, 7	4.7134, 8.62761, 6.1499	3.90801, 5.71704, 4.72762	2.47723, 6	3	vill	0
GL172969.1	258886	259343	259251, 259258, 259304	23, 20, 21	27.0126, 20.3771, 26.35131	13.66126, 10.65342, 13.84438	23.57664,	3	vill	0
GL172969.1	371647	372062	371973, 372029, 372029	15, 22, 16	18.01767, 25.17209, 19.45311	10.49459, 12.89401, 11.10978	14.85992,	3	gars	39076
GL172969.1	393447	394340	393948, 393609, 393484, 394173	7, 9, 8, 4	6.42049, 7.43399, 8.6841, 4.01199	4.8888, 5.30221, 6.11715, 3.46784	3.94161, 4	3	gars	16798
GL172969.1	417510	417763	417719, 417726, 417706	34, 31, 8	38.01896, 35.10245, 9.46129	16.0684, 15.56124, 6.50801	34.34908,	3	gars	0
GL172969.1	440057	440381	440323, 440339, 440344	13, 10, 13	14.43911, 8.12616, 17.28449	8.83395, 5.58036, 10.28792	11.43182,	3	gars	9944
GL172969.1	469629	470099	469716, 469644, 469677	11, 24, 6	10.06049, 25.03583, 5.49907	6.60432, 12.15722, 4.38471	7.29334, 2	3	crhr2	0
GL172969.1	503709	504577	503828, 504199, 504114	7, 10, 8	5.86458, 9.07627, 9.76268	4.55375, 6.1667, 6.64964	3.46146, 6	3	crhr2	23223
GL172969.1	517851	518458	518121, 518151, 517962	26, 24, 6	34.91557, 28.90463, 7.29403	17.22384, 14.44646, 5.30581	31.30381,	3	crhr2	37365
GL172969.1	596544	597078	596606, 596581, 596577	22, 22, 28	27.45805, 25.85271, 38.4921	14.2046, 13.29074, 18.59406	24.01072,	3	crhr2	116058
GL172969.1	676791	677100	677002, 677015, 676931	8, 10, 6	7.77807, 9.39128, 6.6567	5.618, 6.35644, 5.00845	5.17956, 6	3	pomp	132165
GL172969.1	684545	684833	684714, 684728, 684652	11, 13, 6	12.43693, 13.14514, 7.29403	7.97018, 8.09002, 5.30581	9.52692, 1	3	pomp	124432
GL172969.1	690718	691178	691144, 691112, 691128	15, 18, 11	18.26603, 19.24809, 14.75772	10.62691, 10.55079, 9.09568	15.09729,	3	pomp	118087
GL172969.1	747860	748362	748127, 748183, 748277	5, 9, 7	4.82177, 8.21099, 8.69489	3.98509, 5.77858, 6.06379	2.51418, 5	3	pomp	60903
GL172969.1	761655	764053	761864, 762824, 762462	10, 14, 9	7.44561, 11.53986, 10.01275	5.15177, 6.89446, 6.79683	4.86925, 8	3	pomp	45212
GL172969.1	761655	764053	762826, 762824, 762462	9, 14, 9	6.45525, 11.53986, 10.01275	4.68343, 6.89446, 6.79683	3.97502, 8	3	pomp	45212
GL172969.1	779139	779535	779506, 779484, 779503	12, 20, 19	12.11599, 20.68875, 23.88486	7.66241, 10.84083, 12.94573	9.23027, 1	3	pomp	29730
GL172969.1	794330	795322	794977, 794829, 795011	6, 12, 10	5.27951, 10.68384, 11.6791	4.25534, 6.80072, 7.62925	2.9335, 8.0	3	pomp	13943
GL172969.1	838056	838623	838461, 838418, 838257	10, 20, 5	10.01299, 17.71959, 5.31857	6.7221, 9.04982, 4.24819	7.24763, 1	3	fam188b	2713
GL172969.1	847749	848080	848039, 848069, 848044	11, 16, 8	12.01255, 17.16856, 9.60885	7.73996, 9.82359, 6.57807	9.13199, 1	3	fam188b	0
GL172969.1	854597	854903	854878, 854899, 854857	36, 49, 22	43.33179, 55.04894, 27.46822	18.3642, 19.2819, 14.21027	39.57723,	3	fam188b	0
GL172969.1	949222	950007	949870, 949926, 949508	17, 27, 8	17.96244, 27.92464, 10.1465	10.04917, 12.81946, 6.82176	14.80626,	3	fam188b	50255
GL172969.1	1046168	1046478	1046439, 1046419, 1046436	13, 19, 13	13.5657, 19.11425, 17.16464	8.33634, 10.19015, 10.23255	10.60274,	3	adcyap1r1	81368
GL172969.1	1089743	1090226	1089962, 1089965, 1089946	10, 11, 7	8.12348, 8.30667, 6.96907	5.57869, 5.51556, 5.20309	5.50152, 5	3	adcyap1r1	37620
GL172969.1	1122998	1123672	1123145, 1123175, 1123424	13, 20, 10	12.26307, 16.49115, 10.25108	7.56188, 8.31699, 6.85862	9.36972, 1	3	adcyap1r1	4174
GL172969.1	1212596	1213308	1212674, 1212729, 1212660	14, 10, 8	15.66744, 9.39128, 10.1465	9.36333, 6.35644, 6.82176	12.60415,	3	adcyap1r1	0
GL172970.1	171320	172096	171687, 171737, 171692	18, 30, 13	18.22458, 29.99979, 14.32377	9.93936, 12.93196, 8.76942	15.0589, 2	3	copz2	6015
GL172970.1	219920	221080	220355, 220293, 220326	83, 99, 61	75.15429, 81.04806, 58.45118	15.84918, 13.78711, 16.07425	71.00532,	3	snx11	0
GL172970.1	416494	417122	416990, 416815, 416760	8, 11, 6	6.98506, 9.7598, 7.29403	5.14817, 6.42066, 5.30581	4.46457, 7	3	skap1	0
GL172970.1	459798	460183	460142, 459908, 459888	7, 9, 12	7.1696, 8.21099, 15.36829	5.31345, 5.77858, 9.40047	4.61046, 5	3	hoxb1	26780
GL172970.1	472212	472735	472398, 472355, 472335	11, 13, 7	11.56615, 13.14514, 7.90186	7.49066, 8.09002, 5.69394	8.70729, 1	3	hoxb1	14228
GL172970.1	571518	571702	571563, 571564, 571552	82, 37, 39	99.36111, 37.6226, 49.33875	27.08895, 14.60626, 20.5915	94.97629,	3	hoxb8	1164
GL172970.1	597738	598380	598052, 598041, 597802	14, 25, 8	13.09001, 21.89977, 8.86315	7.84687, 9.96113, 6.21039	10.14895,	3	hoxb9	8275
GL172970.1	656721	659005	657980, 658225, 657245, 658476	15, 20, 11, 17	14.58913, 19.78674, 10.5698, 18.80835	8.52337, 10.29739, 6.91098, 10.54536	11.57535,	3	NA	8729
GL172970.1	710136	710844	710215, 710332, 710799	9, 18, 7	8.35434, 16.12237, 8.08824	5.86444, 8.66512, 5.7849	5.71584, 1	3	ube2z	0
GL172970.1	721543	721814	721783, 721783, 721773	38, 62, 33	42.11156, 70.66994, 43.29955	16.77862, 21.78965, 19.58112	38.37566,	3	snf8	0
GL172970.1	907555	907791	907583, 907597, 907588	14, 18, 12	15.49091, 16.88096, 12.62857	9.26387, 9.12607, 7.95947	12.43497,	3	ywhab	61347
GL172970.1	1173016	1173695	1173075, 1173046, 1173064	28, 29, 14	29.73439, 28.42759, 15.80333	13.52745, 12.38278, 9.43935	26.23241,	3	sdc4	82053
GL172970.1	1235876	1236788	1235995, 1236080, 1236598	7, 10, 10	7.1696, 9.37821, 11.997	5.31345, 6.34863, 7.78835	4.61046, 6	3	sdc4	18960
GL172970.1	1243605	1244315	1243990, 1244249, 1243981	8, 10, 5	8.42257, 8.70492, 5.14967	5.97763, 5.93975, 4.16141	5.76336, 6	3	sdc4	11433
GL172971.1	268375	269211	269071, 268969, 268999	10, 11, 3	8.32267, 10.59463, 3.47371	5.70305, 6.92577, 3.03189	5.6878, 7.9	3	ppfibp2	0
GL172971.1	327587	327951	327671, 327840, 327684	9, 12, 5	7.59348, 9.35334, 5.40789	5.40134, 5.9752, 4.29296	5.0069, 6.7	3	ppfibp2	0
GL172971.1	338571	339295	338818, 338877, 338880	11, 22, 10	10.43535, 23.7165, 10.3632	6.83063, 12.03204, 6.92219	7.6474, 20	3	ppfibp2	0
GL172971.1	500807	501094	501046, 501046, 501036	16, 11, 16	16.15907, 10.26356, 18.26684	9.22513, 6.72731, 10.45568	13.07749,	3	U4	45381
GL172971.1	549574	549975	549601, 549627, 549632	26, 37, 28	27.91302, 42.08796, 36.68277	13.17594, 17.19661, 17.59706	24.4551, 3	3	spon1	57496
GL172971.1	562322	562975	562747, 562655, 562726	11, 10, 6	9.52723, 7.97488, 7.12188	6.27749, 5.48553, 5.22884	6.80251, 5	3	spon1	44496
GL172971.1	751285	751638	751578, 751471, 751634	11, 14, 6	11.42621, 14.45897, 6.87953	7.41109, 8.66788, 5.11627	8.57514, 1	3	spon1	0
GL172971.1	1059120	1059557	1059531, 1059455, 1059466	18, 14, 4	19.00683, 11.58379, 4.67325	10.40758, 6.92156, 3.78987	15.81175,	3	kiaa1549l	0
GL172971.1	1144201	1144762	1144328, 1144285, 1144328	8, 7, 10	8.10827, 5.97725, 11.38247	5.80497, 4.62287, 7.47651	5.48761, 3	3	kiaa1549l	58405
GL172971.1	1197764	1198916	1198411, 1197894, 1197918, 1198601	8, 17, 8, 6	8.4095, 16.24129, 8.51364, 6.35308	5.97056, 9.01462, 6.02666, 4.85498	5.76336, 1	3	kiaa1549l	111968
GL172971.1	1249803	1250574	1250292, 1250284, 1249842, 1250518	15, 11, 7, 4	14.8531, 10.60895, 8.28621, 4.30163	8.68248, 6.9343, 5.87881, 3.61556	11.82783,	3	kiaa1549l	164007
GL172971.1	1274161	1274868	1274578, 1274440, 1274558	5, 9, 9	4.82177, 8.21099, 11.64359	3.98509, 5.77858, 7.57973	2.51418, 5	3	kiaa1549l	188365
GL172972.1	797973	799080	798649, 798583, 798953	13, 26, 11	11.07277, 19.47331, 10.14713	6.83197, 8.31002, 6.65691	8.23905, 1	3	dgkh	14718
GL172974.1	116336	116976	116471, 116810, 116601	6, 9, 7	5.72604, 7.63127, 7.40077	4.51498, 5.42473, 5.43746	3.33547, 5	3	NA	65497
GL172974.1	717945	718280	718011, 718179, 718145	10, 10, 6	11.06011, 9.12511, 7.29403	7.306, 6.19629, 5.30581	8.22712, 6	3	5S_rRNA	45759
GL172975.1	502220	502984	502449, 502670, 502645	12, 17, 9	11.43506, 16.17826, 9.82249	7.25774, 8.97632, 6.69629	8.58315, 1	3	chst11	0
GL172976.1	87927	89019	88154, 88300, 88377	7, 8, 5	6.84137, 5.87115, 5.9506	5.13151, 4.45035, 4.54784	4.33107, 3	3	fbxo21	0
GL172976.1	254771	255289	255208, 255252, 255243	13, 17, 10	12.09198, 15.29522, 11.91537	7.45795, 8.43856, 7.74796	9.20698, 1	3	KSR2	31119
GL172976.1	975921	976342	976146, 976126, 976146	19, 7, 6	21.31193, 5.52121, 6.93817	11.49694, 4.33979, 5.14396	18.04063,	3	SRRM4	0
GL172977.1	34145	34720	34564, 34577, 34577	16, 37, 10	16.15907, 36.48766, 10.84317	9.22513, 13.96599, 7.18868	13.07749,	3	NA	3957
GL172977.1	48496	49103	48898, 48879, 48661	6, 12, 6	5.96715, 11.48527, 6.82211	4.64927, 7.28792, 5.08889	3.52158, 8	3	U3	6550
GL172977.1	64166	65075	64669, 64640, 64732	10, 28, 15	8.32267, 25.87795, 16.59998	5.70305, 11.25747, 9.70872	5.6878, 22	3	bid	1243
GL172977.1	132797	133178	133093, 133007, 132964	10, 8, 4	9.03421, 6.55376, 4.55201	6.14116, 4.88226, 3.73489	6.33915, 4	3	slc25a18	0
GL172977.1	178475	178873	178661, 178655, 178693	9, 13, 3	9.72082, 13.14514, 3.47371	6.64182, 8.09002, 3.03189	6.97155, 1	3	cecr2	0
GL172977.1	189449	189809	189572, 189608, 189609	6, 10, 9	5.96715, 9.39128, 11.30431	4.64927, 6.35644, 7.42891	3.52158, 6	3	cecr1	5067
GL172977.1	203927	204649	204329, 204475, 204261	13, 11, 7	13.14146, 10.21061, 7.34973	8.08784, 6.69533, 5.41038	10.19842,	3	cecr1	0
GL172977.1	218057	218388	218192, 218136, 218135	15, 17, 6	14.0331, 16.63185, 6.6567	8.18586, 9.25145, 5.00845	11.04322,	3	cecr1	0
GL172977.1	224379	225044	224973, 224493, 224794	8, 8, 3	6.14488, 7.07159, 3.47371	4.62489, 5.20073, 3.03189	3.6868, 4.6	3	cecr5	0
GL172977.1	252407	252928	252508, 252708, 252627	5, 10, 3	4.82177, 9.39128, 3.47371	3.98509, 6.35644, 3.03189	2.51418, 6	3	NA	5236
GL172977.1	273196	273681	273308, 273303, 273242	8, 10, 7	7.88469, 9.39128, 8.69489	5.67897, 6.35644, 6.06379	5.27935, 6	3	CACNA1C	0
GL172977.1	299755	300143	299879, 299978, 299898	5, 9, 4	4.47183, 6.86591, 4.30163	3.78598, 4.94483, 3.61556	2.26935, 4	3	CACNA1C	0
GL172977.1	325024	325431	325331, 325373, 325369	6, 10, 3	5.1345, 8.70492, 3.37781	4.16817, 5.93975, 2.98791	2.80161, 6	3	CACNA1C	0
GL172977.1	352921	353437	353373, 353335, 353383	24, 15, 21	26.18824, 12.81802, 25.89737	12.84693, 7.4415, 13.59243	22.77286,	3	CACNA1C	0
GL172977.1	371034	371505	371176, 371476, 371274	6, 11, 6	5.96715, 9.13706, 6.87953	4.64927, 6.0355, 5.11627	3.52158, 6	3	CACNA1C	0
GL172977.1	420414	420653	420617, 420625, 420625	20, 20, 17	24.33651, 20.53147, 21.64669	12.96942, 10.74631, 12.11304	20.9737, 1	3	CACNA1C	0
GL172977.1	458764	459700	459392, 459266, 459391	7, 11, 15	6.37715, 9.13706, 18.63599	4.86324, 6.0355, 10.82077	3.90257, 6	3	CACNA1C	22035
GL172977.1	484950	485537	485213, 485213, 485213	28, 90, 34	29.11362, 106.79748, 43.02861	13.15796, 26.88014, 19.02735	25.62753,	3	dcp1b	41548
GL172977.1	524096	524535	524170, 524251, 524375	13, 26, 6	11.16633, 28.39602, 5.83216	6.8898, 13.46449, 4.57461	8.32764, 2	3	dcp1b	2550
GL172977.1	577935	578998	578291, 578464, 578602	15, 24, 8	13.63268, 20.63686, 7.76694	7.94127, 9.54685, 5.6116	10.66672,	3	cacna2d4	0
GL172977.1	585316	585595	585522, 585548, 585565	16, 15, 9	16.23128, 14.58692, 10.64485	9.26839, 8.52203, 7.11743	13.14614,	3	cacna2d4	0
GL172977.1	624970	625446	625010, 625012, 625016	14, 31, 11	15.15267, 35.32126, 12.78973	9.07115, 15.69126, 8.15619	12.11465,	3	cacna2d4	0
GL172977.1	647737	648181	648123, 648160, 648019	5, 15, 4	4.82177, 12.72276, 4.67325	3.98509, 7.383, 3.78987	2.51418, 9	3	cacna2d4	0
GL172977.1	653318	653919	653334, 653345, 653370	20, 17, 10	23.74199, 17.26139, 10.3632	12.63391, 9.63099, 6.92219	20.39591,	3	cacna2d4	0
GL172977.1	680508	680959	680863, 680889, 680779	13, 15, 10	7.59152, 6.56306, 5.91906	4.70117, 3.82207, 4.1867	5.0054, 4.2	3	adipor2	0
GL172977.1	690440	691072	690885, 690753, 690871	20, 28, 19	15.47409, 20.95602, 19.09738	7.72064, 8.5447, 10.17998	12.41969,	3	adipor2	0
GL172977.1	700484	701121	701068, 701116, 701076	43, 44, 29	47.04217, 50.01992, 37.2397	17.44519, 18.70572, 17.5756	43.23296,	3	adipor2	0
GL172977.1	717989	718857	718186, 718288, 718746, 718196	10, 6, 7, 6	10.13706, 4.51135, 5.65148, 7.29403	6.7935, 3.77984, 4.42152, 5.30581	7.36489, 2	3	adipor2	8172
GL172977.1	762117	762883	762211, 762318, 762367	10, 24, 6	10.39648, 22.33212, 5.75442	6.94096, 10.54139, 4.53101	7.61003, 1	3	wnt5b	552
GL172977.1	803838	804500	804017, 804016, 804016	27, 28, 18	25.89358, 25.31181, 18.87511	11.61639, 10.93209, 10.32913	22.48761,	3	fbxl14	29243
GL172977.1	806031	806413	806045, 806100, 806227	23, 12, 7	21.54503, 8.46754, 5.82684	10.40228, 5.42308, 4.53047	18.26496,	3	fbxl14	31436
GL172977.1	824388	824747	824692, 824694, 824680	13, 42, 15	12.87577, 45.5481, 17.94632	7.93025, 17.03003, 10.45626	9.9455, 41	3	erc1	19459
GL172977.1	866979	867509	867103, 867103, 867103	16, 26, 26	15.67432, 24.49803, 31.82009	8.9333, 11.1445, 15.48405	12.61068,	3	erc1	0
GL172977.1	899351	900025	899569, 899872, 899587	17, 29, 20	11.40899, 20.28057, 17.73515	6.115, 7.94221, 9.05916	8.55928, 1	3	erc1	0
GL172977.1	901182	901633	901441, 901469, 901422	40, 43, 19	39.92754, 36.08382, 16.1328	14.70176, 11.64857, 8.38797	36.22596,	3	erc1	0
GL172977.1	920250	920723	920712, 920669, 920686	14, 22, 12	14.10012, 23.27801, 13.59027	8.4555, 11.76976, 8.49571	11.10747,	3	erc1	0
GL172977.1	938941	939178	939125, 939108, 939115	12, 9, 7	12.25023, 6.73685, 7.50542	7.74089, 4.86289, 5.49244	9.35789, 4	3	erc1	0
GL172977.1	968348	968668	968478, 968418, 968430	11, 9, 10	11.78412, 8.21099, 11.83521	7.61327, 5.77858, 7.70798	8.91516, 5	3	erc1	0
GL172977.1	973686	974127	973849, 973972, 973864	9, 6, 7	8.61068, 4.93312, 7.29952	6.01615, 4.04501, 5.38357	5.94135, 2	3	erc1	0
GL172977.1	989113	989742	989167, 989130, 989177	12, 27, 10	10.83541, 27.35215, 11.75646	6.89366, 12.47874, 7.66841	8.02495, 2	3	erc1	0
GL172977.1	1052656	1053059	1052813, 1052805, 1052838	6, 10, 6	5.24248, 8.66076, 6.45058	4.2332, 5.91256, 4.90508	2.89962, 6	3	wnk1	0
GL172977.1	1130542	1130810	1130571, 1130592, 1130644	7, 10, 7	7.1696, 8.44678, 8.35502	5.31345, 5.78021, 5.9108	4.61046, 5	3	wnk1	0
GL172977.1	1179011	1179534	1179387, 1179162, 1179105	12, 22, 13	13.05874, 23.45091, 15.0131	8.20296, 11.87329, 9.14923	10.11961,	3	wnk1	0
GL172977.1	1192410	1192987	1192743, 1192678, 1192447	12, 13, 14	13.05874, 11.76644, 17.61756	8.20296, 7.25915, 10.40352	10.11961,	3	wnk1	4656
GL172977.1	1222623	1223135	1223097, 1223093, 1223090	23, 27, 18	23.01962, 26.50807, 19.45123	11.28568, 11.97847, 10.67087	19.69475,	3	b4galnt3	23008
GL172977.1	1228031	1228347	1228052, 1228046, 1228054	14, 21, 24	12.08222, 18.06033, 24.57189	7.22895, 8.95475, 11.87903	9.19818, 1	3	b4galnt3	17796
GL172977.1	1243423	1243670	1243554, 1243506, 1243534	6, 16, 5	5.96715, 14.46749, 5.50079	4.64927, 8.19898, 4.33868	3.52158, 1	3	b4galnt3	2473
GL172977.1	1262529	1262799	1262572, 1262639, 1262663	8, 13, 7	6.41335, 11.2197, 7.90186	4.7944, 6.92276, 5.69394	3.93678, 8	3	b4galnt3	0
GL172977.1	1290910	1291483	1291143, 1291144, 1291184	7, 8, 10	6.94335, 5.93025, 11.24131	5.18876, 4.48816, 7.40241	4.42528, 3	3	b4galnt3	19567
GL172977.1	1298694	1298987	1298767, 1298975, 1298942	10, 12, 8	10.07458, 11.42765, 9.31951	6.75761, 7.25328, 6.43944	7.30652, 8	3	b4galnt3	27351
GL172978.1	212030	213159	212660, 212459, 212327	15, 34, 13	12.80515, 30.12537, 12.76267	7.4336, 11.56035, 7.86275	9.87861, 2	3	NA	655666
GL172979.1	20194	22119	21479, 21449, 21363	24, 24, 9	23.45797, 18.66087, 8.34444	11.21183, 8.42331, 5.85853	20.12034,	3	fzr1	65895
GL172979.1	298944	299703	299320, 299485, 299418	11, 10, 15	12.43693, 9.39128, 21.28424	7.97018, 6.35644, 12.08458	9.52692, 6	3	ccng2	98419

GL172980.1	21737	22172	22142, 22115, 22138	38, 54, 32	46.49048, 64.20019, 42.20753	19.35686, 22.0806, 19.33316	42.68918,	3	NA	61787
GL172980.1	887714	888491	887812, 887794, 888106	15, 18, 9	14.21298, 15.38496, 7.58036	8.29535, 8.2181, 5.39321	11.21566,	3	sult2b1	33441
GL172980.1	1194538	1195713	1195288, 1195326, 1194773, 1195347	50, 55, 7, 29	59.84246, 61.02379, 5.62023, 36.22158	21.54274, 19.83616, 4.40197, 16.98927	55.85569,	3	ca11	21628
GL172980.1	1228414	1229879	1228556, 1229618, 1228403, 1229381	16, 18, 9, 14	8.22055, 9.25556, 4.77942, 9.07588	4.54136, 4.75144, 3.61366, 5.39179	5.59317, 6	3	ca11	55504
GL172980.1	1228526	1230271	1229832, 1229618, 1228403, 1229381	15, 18, 9, 14	7.84389, 9.25556, 4.77942, 9.07588	4.50175, 4.75144, 3.61366, 5.39179	5.24187, 6	3	ca11	55616
GL172980.1	1252291	1253034	1252735, 1252660, 1252842	20, 14, 10	14.30597, 6.7553, 6.19112	7.05176, 4.06274, 4.35768	11.30445,	3	ca11	79381
GL172980.1	1255312	1256551	1256529, 1256144, 1256136	9, 22, 10	4.49914, 15.459, 7.02444	3.43739, 7.19205, 4.88493	2.29537, 1	3	ca11	82402
GL172980.1	1266936	1267503	1267182, 1267270, 1267110	26, 18, 12	19.41264, 12.70696, 9.99985	8.27769, 6.63009, 6.37794	16.20334,	3	ca11	94026
GL172980.1	1268946	1269686	1269487, 1269362, 1269509	19, 16, 7	12.78229, 10.92524, 4.70411	6.43712, 6.05826, 3.81464	9.85634, 8	3	ca11	96036
GL172981.1	15823	16357	16072, 16090, 16085	18, 14, 11	21.29566, 14.45897, 14.75772	11.73423, 8.66788, 9.09568	18.02512,	3	NA	30160
GL172981.1	39217	39677	39339, 39302, 39330	10, 8, 12	9.89241, 7.07159, 15.47496	6.65218, 5.20073, 9.45079	7.13431, 4	3	NA	6840
GL172981.1	44272	45057	44605, 44680, 44700	43, 38, 38	42.96029, 36.68429, 42.55988	15.16937, 13.69053, 17.04068	39.21146,	3	NA	1460
GL172981.1	549497	549853	549825, 549794, 549694	12, 17, 10	12.67544, 16.11578, 9.88017	7.98627, 8.93835, 6.64506	9.75458, 1	3	rps29	1302
GL172981.1	935524	935927	935693, 935714, 935604	8, 8, 8	8.42257, 7.07159, 8.56955	5.97763, 5.20073, 6.05652	5.76336, 4	3	NA	0
GL172981.1	972690	973336	972906, 973220, 973042	5, 8, 5	4.82177, 7.07159, 5.9506	3.98509, 5.20073, 4.54784	2.51418, 4	3	NA	6978
GL172981.1	1273322	1273949	1273528, 1273696, 1273574	8, 11, 8	8.42257, 10.60895, 10.1465	5.97763, 6.9343, 6.82176	5.76336, 7	3	NA	307610
GL172982.1	675483	676731	675904, 676332, 676119	16, 37, 20	13.43192, 34.2431, 20.77586	7.56568, 12.73035, 10.89313	10.47512,	3	dynlrb2	2902
GL172982.1	777688	778189	777863, 777856, 777864	22, 24, 15	23.01801, 25.7315, 18.13606	11.61387, 12.57396, 10.55788	19.69328,	3	dynlrb2	105107
GL172983.1	220979	221293	221178, 221246, 221112	10, 6, 9	10.74428, 4.73925, 10.49552	7.13454, 3.92426, 7.0436	7.93927, 2	3	NA	3221
GL172983.1	358048	358443	358172, 358154, 358186	13, 9, 7	13.27928, 7.51164, 7.40077	8.16901, 5.35055, 5.43746	10.32936,	3	NA	14838
GL172983.1	368424	368955	368495, 368448, 368453	7, 10, 8	6.74267, 8.36423, 9.31951	5.07552, 5.72892, 6.43944	4.23998, 5	3	NA	25214
GL172983.1	374039	374578	374204, 374245, 374279	6, 9, 9	5.96715, 7.39577, 10.28145	4.64927, 5.27837, 6.93568	3.52158, 4	3	NA	30829
GL172983.1	433227	433788	433607, 433632, 433655	6, 16, 4	5.55471, 16.15392, 4.67325	4.41698, 9.22204, 3.78987	3.18192, 1	3	NA	67755
GL172983.1	448486	448945	448721, 448638, 448518	6, 11, 5	5.55471, 10.26356, 5.9506	4.41698, 6.72731, 4.54784	3.18192, 7	3	NA	52598
GL172983.1	530299	531111	530847, 530683, 530738	5, 11, 7	4.82177, 10.60895, 8.646	3.98509, 6.9343, 6.04229	2.51418, 7	3	NA	13826
GL172983.1	558349	558996	558705, 558690, 558648	8, 8, 9	8.42257, 7.07159, 11.64359	5.97763, 5.20073, 7.57973	5.76336, 4	3	NA	41876
GL172983.1	610995	611823	611230, 611086, 611620	12, 15, 8	11.67221, 12.18092, 9.25066	7.39985, 7.05041, 6.40569	8.80793, 9	3	NA	12777
GL172983.1	629692	630386	630056, 630134, 629866	20, 19, 15	18.6379, 16.10778, 15.74774	9.60312, 8.37295, 9.21465	15.4562, 1	3	NA	0
GL172983.1	638229	640340	639473, 639472, 639473	36, 35, 22	34.55005, 33.23161, 23.85667	13.26744, 12.90762, 12.11568	30.94527,	3	NA	559
GL172983.1	643421	644395	644029, 643565, 644064	15, 15, 8	11.86702, 9.28651, 7.15322	6.85816, 5.31464, 5.25003	8.99365, 6	3	NA	5751
GL172983.1	833799	834402	834186, 834223, 834224	12, 14, 13	12.97977, 11.49628, 13.77491	8.15868, 6.86757, 8.45734	10.04411,	3	NA	4318
GL172983.1	853818	854837	854136, 854348, 854226	13, 16, 5	14.79381, 16.22539, 5.9506	9.02996, 9.26486, 4.54784	11.77132,	3	NA	0
GL172983.1	877704	878186	877890, 877914, 878034	12, 14, 5	12.90199, 14.45897, 5.69847	8.11489, 8.66788, 4.4331	9.97033, 1	3	selenoi	0
GL172983.1	946451	946821	946788, 946727, 946772	13, 12, 11	14.52571, 11.86108, 12.2694	8.88215, 7.51216, 7.88012	11.51483,	3	otof	0
GL172983.1	1010145	1010527	1010325, 1010413, 1010416	9, 13, 7	9.72082, 13.14514, 8.69489	6.64182, 8.09002, 6.06379	6.97155, 1	3	otof	0
GL172983.1	1041330	1041772	1041497, 1041681, 1041493	8, 8, 11	8.42257, 7.07159, 11.6121	5.97763, 5.20073, 7.51665	5.76336, 4	3	c2orf70	0
GL172983.1	1055589	1056059	1055861, 1055840, 1055837	10, 13, 6	10.67246, 13.14514, 6.99807	7.09497, 8.09002, 5.17195	7.87096, 1	3	c2orf70	407
GL172983.1	1089014	1089445	1089244, 1089406, 1089309	8, 19, 5	6.86256, 20.21388, 5.03069	5.0733, 10.84946, 4.09861	4.3506, 17	3	NA	9372
GL172983.1	1169733	1170364	1169912, 1170026, 1169922	6, 9, 5	4.9641, 7.97047, 3.99827	4.06411, 5.63299, 3.50002	2.64509, 5	3	NA	12980
GL172983.1	1185369	1186221	1185559, 1185878, 1185763	9, 15, 10	8.88537, 13.53022, 12.42918	6.17591, 7.87854, 7.99682	6.19961, 1	3	NA	21
GL172983.1	1202763	1203575	1203483, 1203420, 1202712, 1203548	11, 11, 3, 7	12.43693, 10.26356, 3.47371, 8.69489	7.97018, 6.72731, 3.03189, 6.06379	9.52692, 7	3	NA	17415
GL172983.1	1214058	1214862	1214410, 1214453, 1214653	9, 10, 16	6.86176, 7.72315, 17.62687	4.9422, 5.32711, 10.0901	4.3506, 5.2	3	scara3	10011
GL172983.1	1247386	1247781	1247417, 1247648, 1247605	6, 6, 5	5.81608, 4.18132, 5.9506	4.56563, 3.56655, 4.54784	3.4176, 2.0	3	scara3	15948
GL172983.1	1314154	1314387	1314324, 1314211, 1314228	9, 12, 12	7.47395, 9.74388, 14.14699	5.32711, 6.2187, 8.79187	4.89626, 7	3	t2r5	1052
GL172984.1	2847	4155	3546, 3457, 3504	80, 111, 37	71.3634, 94.95564, 29.58031	15.30798, 15.19175, 10.32504	67.25301,	3	plxna1	649231
GL172984.1	6995	7667	7355, 7348, 7400	49, 66, 37	32.08419, 38.94478, 27.14182	8.48414, 7.83021, 9.16926	28.53091,	3	plxna1	645719
GL172984.1	8444	9577	9225, 9169, 9141	24, 43, 21	14.96047, 28.74808, 15.40667	6.47036, 8.44769, 7.41715	11.93161,	3	plxna1	643809
GL172984.1	44670	45897	45226, 45206, 45415	26, 23, 12	27.72954, 21.25586, 12.82015	13.06627, 10.22998, 8.0686	24.27641,	3	plxna1	607489
GL172984.1	120237	120669	120522, 120572, 120297	12, 12, 4	10.39353, 9.24217, 3.85136	6.62187, 5.90582, 3.38149	7.60856, 6	3	plxna1	532717
GL172984.1	148455	148955	148638, 148921, 148637	5, 8, 4	4.47183, 5.93025, 4.46488	3.78598, 4.48816, 3.69425	2.26935, 3	3	plxna1	504431
GL172984.1	265176	266059	265695, 265695, 265438	22, 26, 11	22.06117, 25.04516, 11.25734	11.03864, 11.46708, 7.31423	18.76525,	3	plxna1	387327
GL172984.1	477367	477603	477559, 477550, 477573	8, 14, 9	8.42257, 13.63145, 11.21577	5.97763, 8.17488, 7.38849	5.76336, 1	3	plxna1	175783
GL172984.1	679758	680273	680171, 679856, 680157	5, 6, 3	4.30277, 4.30294, 3.47371	3.68597, 3.64568, 3.03189	2.12436, 2	3	plxna1	0
GL172984.1	742148	742834	742468, 742277, 742738, 742433	7, 9, 9, 4	6.2923, 7.55109, 7.75488, 3.91395	4.81292, 5.37505, 5.501, 3.41551	3.8235, 5.1	3	plxna1	0
GL172984.1	870229	870773	870434, 870306, 870440	7, 8, 7	6.99564, 6.4822, 7.10639	5.21786, 4.83755, 5.27894	4.47307, 4	3	plxna1	28861
GL172984.1	917464	917820	917610, 917668, 917599	7, 11, 9	6.89193, 9.13706, 8.98852	5.15998, 6.0355, 6.23511	4.37772, 6	3	plxna1	76096
GL172984.1	940285	940824	940412, 940377, 940390	19, 16, 16	20.56775, 16.01323, 19.65507	11.05955, 9.13759, 11.21774	17.31957,	3	plxna1	98917
GL172984.1	1070598	1070929	1070824, 1070817, 1070866	5, 12, 4	4.655, 11.0952, 4.64325	3.89157, 7.05215, 3.77643	2.42652, 8	3	chchd6	0
GL172985.1	200927	201220	201146, 201140, 201098	10, 18, 5	9.44155, 17.86551, 5.54867	6.38646, 9.72285, 4.3619	6.72156, 1	3	plk2	114562
GL172985.1	259646	260171	260163, 259949, 259674	6, 20, 6	5.03089, 17.18461, 6.6567	4.10511, 8.72933, 5.00845	2.70624, 1	3	plk2	55611
GL172985.1	351494	351730	351682, 351699, 351703	44, 32, 37	53.09763, 34.71953, 47.13388	20.49751, 14.9339, 20.17371	49.20176,	3	plk2	28164
GL172985.1	376645	377523	376974, 376892, 376883	9, 11, 7	8.30507, 8.55421, 7.50542	5.83501, 5.67101, 5.49244	5.67102, 6	3	dazl	3243
GL172985.1	686221	686598	686397, 686248, 686246	13, 15, 9	13.79066, 14.27489, 9.70054	8.46641, 8.33298, 6.63089	10.8171, 1	3	pde4d	46748
GL172985.1	718455	718912	718722, 718728, 718766	10, 13, 5	8.61898, 10.63899, 4.51488	5.88679, 6.56333, 3.81106	5.94907, 7	3	pde4d	78982
GL172985.1	1028597	1029701	1029032, 1029031, 1028643	7, 21, 5	5.55587, 19.48223, 5.27517	4.36159, 9.80473, 4.22616	3.18254, 1	3	depdc1b	185970
GL172985.1	1129485	1130496	1129826, 1130416, 1129937	6, 6, 7	5.27951, 4.93312, 8.08824	4.25534, 4.04501, 5.7849	2.9335, 2.7	3	depdc1b	85175
GL172986.1	133011	133747	133230, 133358, 133326	8, 15, 8	7.33538, 12.7702, 8.35103	5.35908, 7.41214, 5.93881	4.76518, 1	3	skp2	247
GL172986.1	183486	184217	183760, 183752, 183562	7, 13, 7	5.13345, 10.59688, 6.92462	4.09293, 6.53722, 5.17829	2.80161, 7	3	nipbl	10221
GL172986.1	611770	612647	611960, 611999, 611895	22, 27, 15	21.47487, 25.86233, 16.99143	10.6858, 11.59804, 9.93059	18.19793,	3	NA	10314
GL172986.1	760753	761252	761066, 760906, 761080	7, 17, 3	7.1696, 18.56115, 3.47371	5.31345, 10.40145, 3.03189	4.61046, 1	3	egflam	30453
GL172986.1	767769	768888	768035, 768207, 768477	12, 13, 9	12.38809, 11.81904, 11.64359	7.82099, 7.29135, 7.57973	9.48755, 9	3	egflam	22817
GL172986.1	888097	888680	888600, 888627, 888644	33, 31, 26	35.54165, 32.63958, 32.40004	15.01642, 14.10081, 15.81804	31.9176, 2	3	lifr	13916
GL172986.1	1004706	1005154	1004917, 1004797, 1005061	6, 6, 6	5.81608, 4.93312, 6.6567	4.56563, 4.04501, 5.00845	3.4176, 2.7	3	osmr	2645
GL172986.1	1147770	1148250	1148222, 1148201, 1148199	50, 53, 32	64.03035, 66.46493, 42.3545	23.98207, 23.88478, 19.41693	59.9973, 6	3	rictor	61878
GL172986.1	1154592	1155046	1154794, 1154836, 1154649	7, 9, 4	7.1696, 8.21099, 4.67325	5.31345, 5.77858, 3.78987	4.61046, 5	3	rictor	68700
GL172986.1	1187821	1188548	1187993, 1187981, 1187979	8, 8, 3	8.22568, 7.06103, 3.27564	5.8701, 5.19433, 2.93941	5.59681, 4	3	rictor	101929
GL172986.1	1231826	1232580	1231927, 1232298, 1232119	10, 7, 4	7.78798, 3.90763, 3.12644	5.36798, 3.2905, 2.95476	5.18872, 1	3	rictor	145934
GL172986.1	1233265	1233791	1233678, 1233691, 1233674	32, 50, 24	37.15584, 60.59549, 30.17171	16.37888, 21.97813, 15.16988	33.5013, 5	3	rictor	147373
GL172987.1	56465	56960	56907, 56902, 56764	68, 81, 37	82.57252, 96.9453, 48.24757	25.28785, 26.34299, 20.825	78.35162,	3	NA	59008
GL172987.1	92694	93120	92955, 92945, 92906	12, 14, 5	13.22036, 14.29802, 5.9506	8.29296, 8.57291, 4.54784	10.27237,	3	NA	22848
GL172987.1	98383	98711	98410, 98412, 98410	52, 78, 50	65.87104, 94.42375, 67.56542	24.04731, 26.54148, 26.05662	61.81747,	3	NA	17257
GL172987.1	152053	153096	152381, 152342, 152326	17, 22, 15	17.64386, 22.77797, 17.07266	9.85981, 11.46975, 9.97618	14.49984,	3	NA	35271
GL172987.1	288445	288941	288607, 288761, 288666	6, 10, 6	5.96715, 8.79469, 6.76586	4.64927, 5.99491, 5.06179	3.52158, 6	3	NA	61514
GL172987.1	328428	329312	328767, 329032, 328590	9, 13, 9	9.50098, 11.76644, 10.42289	6.52233, 7.25915, 7.00726	6.77745, 9	3	NA	33981
GL172987.1	353250	353615	353289, 353381, 353368	4, 6, 7	3.74195, 4.93312, 8.69489	3.32091, 4.04501, 6.06379	1.6222, 2.7	3	NA	9678
GL172987.1	373916	374589	373988, 373958, 373942	9, 17, 11	9.72082, 17.79269, 13.83651	6.64182, 9.94842, 8.67735	6.97155, 1	3	NA	0
GL172987.1	465173	465641	465503, 465489, 465371	20, 37, 10	19.70489, 40.48476, 10.84317	10.24795, 16.25649, 7.18868	16.48628,	3	NA	9546
GL172987.1	813288	814246	813971, 813989, 813450	6, 8, 5	5.43318, 7.07159, 5.69847	4.34622, 5.20073, 4.4331	3.07284, 4	3	etfb	15404
GL172987.1	960770	961516	961329, 961329, 961329	21, 43, 13	20.80599, 41.54953, 12.34408	10.60246, 14.41136, 7.61094	17.55058,	3	iglon5	23798
GL172987.1	971635	972015	971906, 971874, 971742	8, 10, 7	7.06928, 7.35725, 6.6707	5.19933, 5.09584, 5.03432	4.5414, 4.9	3	iglon5	34663
GL172987.1	982061	983322	982904, 982725, 982686	12, 21, 14	10.63412, 18.82704, 14.72868	6.77016, 9.41165, 8.82588	7.83559, 1	3	iglon5	45089
GL172987.1	1086127	1086787	1086596, 1086409, 1086453	15, 40, 13	15.27108, 40.92942, 14.39632	8.9326, 15.25969, 8.81006	12.22851,	3	iglon5	149155
GL172987.1	1093433	1093872	1093710, 1093709, 1093709	10, 7, 11	8.66311, 5.8946, 12.78973	5.914, 4.57222, 8.15619	5.99101, 3	3	iglon5	156461
GL172987.1	1112367	1113090	1112957, 1112933, 1112538	13, 12, 7	14.26982, 10.53798, 7.72582	8.73911, 6.71099, 5.6058	11.26966,	3	iglon5	175395
GL172987.1	1162756	1163464	1163050, 1163305, 1163204	9, 10, 4	8.77314, 8.70492, 4.30163	6.111, 5.93975, 3.61556	6.09355, 6	3	iglon5	225784
GL172987.1	1196544	1197242	1196571, 1196570, 1196570	61, 124, 63	82.56877, 163.1073, 88.31002	29.03724, 36.92327, 31.28605	78.34831,	3	iglon5	259572
GL172988.1	258921	259527	259078, 259131, 259143	19, 13, 8	20.74745, 10.35117, 8.51364	11.16575, 6.38482, 6.02666	17.49384,	3	NA	2276
GL172988.1	639356	639944	639799, 639799, 639799	29, 48, 19	32.03412, 52.68947, 21.69099	14.51808, 18.41233, 11.71707	28.48199,	3	pipox	67396
GL172988.1	795173	795938	795528, 795596, 795321	11, 12, 7	12.43693, 11.86108, 8.69489	7.97018, 7.51216, 6.06379	9.52692, 9	3	NA	71529
GL172988.1	1049626	1050079	1050046, 1049897, 1050078	6, 10, 7	5.51356, 7.20159, 7.84208	4.39314, 4.99723, 5.66426	3.14574, 4	3	sez6	0
GL172989.1	64889	65116	65033, 64905, 64959	12, 25, 11	9.08501, 23.61075, 11.6739	5.80775, 10.96304, 7.55148	6.38792, 2	3	txnrd3	1838
GL172989.1	297911	300054	298223, 298599, 298598	49, 57, 34	34.24566, 36.32782, 25.26184	9.2834, 8.45325, 9.07417	30.64787,	3	fgd5	0
GL172989.1	583134	583702	583337, 583311, 583284	13, 19, 15	15.29169, 21.4153, 20.2794	9.29854, 11.55717, 11.63174	12.24396,	3	ccdc174	74991
GL172989.1	1111926	1112461	1112129, 1112039, 1112152	9, 6, 4	8.55802, 4.379, 4.67325	5.98518, 3.69486, 3.78987	5.8909, 2.2	3	aldh1l1	64883
GL172990.1	43415	43715	43460, 43632, 43502	7, 7, 3	6.50909, 5.97725, 3.41347	4.94073, 4.62287, 3.00444	4.02371, 3	3	ace2	0
GL172990.1	966990	967755	967368, 967355, 967347	8, 11, 5	8.42257, 9.66539, 5.69847	5.97763, 6.36265, 4.4331	5.76336, 7	3	NA	0
GL172991.1	9346	10323	10113, 9617, 9748	14, 16, 8	14.10012, 14.92393, 9.76268	8.4555, 8.47764, 6.64964	11.10747,	3	jakmip1	0
GL172991.1	249761	250667	250050, 250050, 250051	11, 19, 14	10.5483, 16.5695, 13.875	6.89817, 8.65063, 8.32113	7.75329, 1	3	man2b2	24123
GL172991.1	353450	354061	354029, 354035, 354032	15, 34, 17	16.03559, 37.12254, 19.81591	9.3831, 15.53943, 11.12108	12.95858,	3	ablim2	0
GL172991.1	484780	485308	485279, 485230, 485164	22, 17, 15	23.62598, 15.5773, 17.07266	11.97797, 8.61051, 9.97618	20.28347,	3	sorcs2	6890
GL172991.1	1036125	1036735	1036416, 1036408, 1036416	15, 24, 7	13.8583, 23.62809, 6.25413	8.07921, 11.31356, 4.79015	10.87599,	3	tbc1d14	0
GL172991.1	1169604	1169844	1169816, 1169796, 1169839	19, 14, 11	22.13159, 12.80997, 12.86886	11.97036, 7.67593, 8.19722	18.83405,	3	S100P	4227
GL172992.1	45782	46290	45985, 45940, 45947	4, 9, 6	3.54306, 7.83963, 6.12365	3.2072, 5.55305, 4.73409	1.57602, 5	3	dbp	0
GL172992.1	196011	196294	196127, 196163, 196105	18, 23, 7	17.93309, 21.25586, 6.83761	9.76366, 10.22998, 5.12939	14.77832,	3	leng1	10
GL172992.1	212889	213095	212986, 212986, 212986	217, 248, 174	262.93539, 301.11023, 229.76598	35.19336, 36.20078, 41.13061	257.72968	3	tmc4	3021
GL172992.1	247460	248119	247505, 247515, 247523	23, 27, 17	24.32011, 29.84144, 19.63801	12.06642, 13.96317, 11.0208	20.95794,	3	ndufa3	2959
GL172992.1	627826	628444	628411, 628410, 628357	23, 37, 16	24.58488, 40.18319, 17.62687	12.225, 16.08067, 10.0901	21.21468,	3	syt5	0
GL172993.1	309058	309958	309774, 309444, 309446	26, 30, 14	20.96151, 22.77635, 13.17984	9.11922, 8.93219, 7.90153	17.70112,	3	enpp7-l	191440
GL172993.1	1085127	1085512	1085206, 1085207, 1085183	11, 14, 4	10.5483, 11.49628, 4.30163	6.89817, 6.86757, 3.61556	7.75329, 8	3	NA	10908
GL172994.1	781888	782662	782119, 782110, 782064	10, 11, 4	8.53211, 8.17132, 4.42274	5.83309, 5.43051, 3.67426	5.86712, 5	3	nt5dc1	3290
GL172995.1	1170973	1172139	1171786, 1171428, 1171440	11, 10, 8	8.84943, 6.55702, 8.56955	5.85597, 4.58876, 6.05652	6.16564, 4	3	NA	4011
GL172996.1	83428	83856	83764, 83769, 83821	6, 7, 7	5.43318, 5.42726, 8.69489	4.34622, 4.28044, 6.06379	3.07284, 3	3	NA	64617
GL172996.1	213878	214133	214022, 214022, 214023	32, 42, 17	36.24469, 45.85291, 20.18522	15.83805, 17.20417, 11.32722	32.60691,	3	NA	22499
GL172996.1	472256	472567	472538, 472391, 472429	10, 11, 7	9.83337, 7.85056, 6.75301	6.61776, 5.22892, 5.08141	7.07841, 5	3	U3	52678
GL172996.1	732089	732842	732476, 732481, 732475	10, 12, 8	9.77513, 9.54462, 8.35103	6.5837, 6.09452, 5.93881	7.02302, 6	3	cntn4	37533
GL172996.1	830150	830525	830348, 830338, 830356	9, 9, 11	7.80169, 6.8008, 12.06254	5.52977, 4.90352, 7.76744	5.20141, 4	3	il5ra	0
GL172996.1	844886	845779	845098, 845465, 845007	6, 15, 3	5.31708, 13.37045, 3.17999	4.2777, 7.78062, 2.89245	2.96727, 1	3	il5ra	0
GL172997.1	588835	589432	589272, 589089, 589188	7, 6, 4	6.50909, 4.18132, 4.38152	4.94073, 3.56655, 3.65448	4.02371, 2	3	ctnnd2	3947
GL172997.1	857327	858346	858034, 858058, 858198	18, 23, 8	17.05737, 20.08442, 8.4044	9.23321, 9.53731, 5.9678	13.93581,	3	ropn1l	1284
GL172998.1	129649	130134	129933, 129780, 129726	10, 13, 9	7.78798, 8.49752, 6.72209	5.36798, 5.24388, 4.8535	5.18872, 5	3	NA	11943
GL172998.1	130419	131149	130638, 130660, 130543	19, 31, 25	18.33514, 28.9306, 26.53867	9.71917, 11.94788, 12.70996	15.164, 25	3	NA	10928
GL172998.1	255875	256844	256400, 256451, 256431	10, 13, 8	10.74428, 12.09006, 9.11678	7.13454, 7.45678, 6.33924	7.93927, 9	3	NA	5086
GL172998.1	402409	403144	402670, 402804, 402768	6, 7, 9	5.96715, 5.97725, 11.04428	4.64927, 4.62287, 7.30896	3.52158, 3	3	NA	0
GL172998.1	523414	525340	523985, 524736, 524738	11, 27, 10	12.43693, 26.73198, 11.38247	7.97018, 12.11089, 7.47651	9.52692, 2	3	NA	55032
GL172998.1	528502	529350	528953, 529056, 528858	5, 15, 8	4.82177, 13.16372, 8.74279	3.98509, 7.65379, 6.14792	2.51418, 1	3	NA	51022
GL172998.1	572020	573032	572316, 572630, 572597	14, 27, 14	15.84929, 31.55624, 19.30655	9.46495, 14.98154, 11.20466	12.77923,	3	NA	7340
GL172998.1	634471	634991	634764, 634720, 634934	15, 18, 7	18.26603, 19.97713, 8.69489	10.62691, 10.97931, 6.06379	15.09729,	3	NA	48975
GL172998.1	792108	792872	792605, 792576, 792743	11, 11, 7	11.71035, 9.52738, 8.646	7.57196, 6.27759, 6.04229	8.8442, 6.9	3	NA	30759
GL172998.1	805602	806526	806281, 805911, 805996	13, 7, 21	13.86771, 5.65148, 23.5695	8.51067, 4.42152, 12.25439	10.88502,	3	NA	17105
GL172998.1	887487	888157	887852, 887623, 887911	9, 7, 5	6.96255, 4.79556, 5.10931	5.00603, 3.87426, 4.14026	4.44307, 2	3	NA	0
GL172998.1	1053024	1054000	1053096, 1053433, 1053471	4, 5, 5	3.74195, 3.94561, 5.9506	3.32091, 3.46715, 4.54784	1.6222, 1.8	3	f13b	7162
GL172998.1	1063602	1064253	1063991, 1063923, 1064008	22, 19, 8	24.77128, 19.8035, 8.51364	12.65828, 10.60442, 6.02666	21.39609,	3	f13b	17740
GL172998.1	1088890	1089335	1089143, 1089168, 1089144	18, 21, 15	19.8791, 20.39593, 19.17716	10.9221, 10.35516, 11.09709	16.65356,	3	f13b	43028
GL172998.1	1172540	1173182	1172960, 1172957, 1172956	24, 25, 9	24.85409, 25.58747, 9.19414	12.04825, 12.14055, 6.35176	21.47699,	3	f13b	126678
GL172998.1	1187631	1188084	1188044, 1187668, 1187987	10, 14, 9	10.6018, 11.67268, 10.79992	7.05583, 6.97641, 7.19283	7.80484, 8	3	f13b	141769
GL172999.1	305553	306451	306080, 305731, 305950	6, 13, 7	5.81608, 10.59688, 8.35502	4.56563, 6.53722, 5.9108	3.4176, 7.9	3	NA	17802
GL173000.1	15008	15809	15565, 15613, 15466	7, 9, 5	7.1696, 8.21099, 5.9506	5.31345, 5.77858, 4.54784	4.61046, 5	3	slc25a26	10709
GL173000.1	68853	69515	69372, 69189, 69167	6, 15, 5	5.63898, 14.09492, 5.14967	4.46544, 8.22351, 4.16141	3.25772, 1	3	slc25a26	0
GL173000.1	119529	119976	119534, 119590, 119594	11, 26, 9	11.6377, 31.2219, 10.79992	7.53109, 15.13645, 7.19283	8.77589, 2	3	lrig1	7585
GL173000.1	183162	183427	183286, 183252, 183273	22, 20, 23	23.44815, 19.37003, 28.81839	11.87164, 10.0456, 14.6944	20.11079,	3	lrig1	71218
GL173000.1	188623	188981	188854, 188750, 188792	13, 17, 14	11.55805, 14.76344, 16.55387	7.13125, 8.11448, 9.85015	8.70034, 1	3	lrig1	76679
GL173000.1	218962	219409	219063, 219148, 219303	19, 23, 11	18.60811, 22.81409, 12.48556	9.8844, 11.16226, 7.99611	15.42706,	3	lrig1	107018
GL173000.1	309177	309806	309207, 309191, 309221	13, 19, 11	12.14841, 17.52577, 12.34038	7.49227, 9.22875, 7.91841	9.26135, 1	3	kbtbd8	17045
GL173000.1	373978	374746	374391, 374118, 374348	8, 11, 3	7.72597, 10.60895, 3.47371	5.588, 6.9343, 3.03189	5.13061, 7	3	NA	0
GL173000.1	588570	589079	589036, 588795, 589043	10, 14, 9	9.38842, 11.58379, 9.52439	6.35473, 6.92156, 6.53516	6.67167, 8	3	suclg2	0
GL173000.1	652511	653172	652552, 652885, 652562	11, 17, 4	12.17114, 17.26139, 4.34116	7.82679, 9.63099, 3.63492	9.28263, 1	3	suclg2	58385
GL173000.1	673035	673710	673526, 673617, 673624	16, 31, 13	17.41877, 36.47396, 16.28606	9.96955, 16.37533, 9.81016	14.28292,	3	suclg2	78909
GL173000.1	681906	682448	682299, 682299, 682299	16, 36, 17	17.77392, 42.20801, 22.12333	10.17481, 17.69938, 12.35799	14.62581,	3	suclg2	87780
GL173000.1	747001	747364	747193, 747193, 747147	9, 10, 5	7.93244, 9.39128, 5.9506	5.60981, 6.35644, 4.54784	5.32435, 6	3	suclg2	152875
GL173000.1	819421	819888	819651, 819625, 819619	13, 8, 15	14.10549, 7.06103, 19.64455	8.64628, 5.19433, 11.32852	11.11201,	3	suclg2	225295
GL173000.1	935317	935932	935473, 935484, 935749	5, 5, 4	4.69353, 3.31156, 4.50795	3.9134, 3.05705, 3.71446	2.45909, 1	3	suclg2	341191
GL173000.1	946048	946791	946448, 946748, 946692	6, 10, 11	5.68215, 8.36423, 12.06254	4.49007, 5.72892, 7.76744	3.29732, 5	3	suclg2	351922
GL173000.1	992819	993464	993092, 992900, 993260	9, 12, 8	9.12009, 9.70336, 8.35103	6.30997, 6.19346, 5.93881	6.42028, 7	3	suclg2	398693
GL173000.1	1073446	1073854	1073737, 1073713, 1073713	6, 10, 5	5.27951, 9.028, 5.54867	4.25534, 6.13739, 4.3619	2.9335, 6.4	3	suclg2	479320
GL173000.1	1081214	1081596	1081369, 1081333, 1081328	7, 10, 5	7.1696, 9.37821, 4.88115	5.31345, 6.34863, 4.01777	4.61046, 6	3	suclg2	487088
GL173001.1	380978	381335	381234, 381215, 381173	8, 10, 7	6.66791, 6.66155, 8.02489	4.95324, 4.65493, 5.75426	4.17132, 4	3	sel1l	615532
GL173001.1	493358	493655	493585, 493547, 493584	7, 18, 13	7.1696, 19.97713, 17.65969	5.31345, 10.97931, 10.45768	4.61046, 1	3	sel1l	503212
GL173001.1	807276	808229	807927, 807608, 807848	5, 14, 8	4.82177, 14.45897, 10.1465	3.98509, 8.66788, 6.82176	2.51418, 1	3	sel1l	188638
GL173002.1	976416	976974	976704, 976838, 976705	13, 30, 12	11.81837, 29.84375, 11.7126	7.29094, 12.84069, 7.42393	8.94738, 2	3	ndor1	7397
GL173002.1	1039124	1039580	1039397, 1039203, 1039525	9, 8, 4	9.24298, 7.01915, 4.55201	6.3792, 5.16892, 3.73489	6.53474, 4	3	slc47a2	0
GL173002.1	1051750	1052414	1052257, 1052269, 1052115	63, 48, 40	49.58273, 31.63052, 29.16413	11.76999, 8.51617, 9.31392	45.73577,	3	slc47a2	11464
GL173002.1	1053145	1054798	1053965, 1053975, 1053567	53, 55, 54	38.5606, 39.40886, 45.50365	10.02607, 9.89391, 12.57845	34.88203,	3	slc47a2	12859
GL173002.1	1092412	1093285	1093030, 1092482, 1092844	4, 10, 9	3.39747, 9.07627, 9.46739	3.12111, 6.1667, 6.50386	1.46102, 6	3	grin1	0
GL173002.1	1107643	1108428	1108178, 1107823, 1107704	7, 6, 5	5.07797, 4.02092, 3.9505	4.05723, 3.46138, 3.47021	2.75052, 1	3	grin1	0
GL173002.1	1111247	1112204	1111957, 1112010, 1111651	12, 11, 16	10.25464, 7.78921, 18.69936	6.53598, 5.19038, 10.69793	7.47735, 5	3	grin1	0
GL173002.1	1130978	1131539	1131445, 1131432, 1131146	17, 17, 15	14.69453, 12.93592, 14.99627	8.07252, 7.01058, 8.76842	11.67625,	3	XB-GENE-5992704	831
GL173002.1	1180355	1180918	1180662, 1180693, 1180523	6, 8, 3	4.83561, 7.07159, 3.47371	3.98453, 5.20073, 3.03189	2.52606, 4	3	znf618	11070
GL173002.1	1197217	1197945	1197638, 1197399, 1197570	11, 7, 11	9.81032, 5.97725, 13.1143	6.45164, 4.62287, 8.32282	7.05644, 3	3	araf	17530
GL173002.1	1202296	1202791	1202542, 1202678, 1202670	10, 9, 9	8.93828, 8.21099, 10.49552	6.08275, 5.77858, 7.0436	6.24946, 5	3	araf	12684
GL173003.1	145140	145547	145481, 145526, 145460	9, 18, 4	7.84477, 17.86551, 4.11547	5.5562, 9.72285, 3.5218	5.24227, 1	3	pdzrn3	0
GL173003.1	592479	592830	592641, 592515, 592676	7, 7, 5	6.46447, 5.13485, 5.45388	4.91463, 4.09383, 4.3157	3.98312, 2	3	plxnb1	30414
GL173003.1	698636	699028	698865, 698892, 698875	10, 23, 8	8.66311, 19.81339, 7.85773	5.914, 9.37856, 5.66361	5.99101, 1	3	XB-GENE-5772074	0
GL173003.1	1098201	1098941	1098490, 1098504, 1098506	50, 87, 23	56.23783, 98.04842, 28.13384	19.48957, 24.05676, 14.30737	52.29848,	3	cpne5	25101
GL173004.1	70751	71464	70876, 70922, 70903	15, 22, 8	17.28737, 22.45914, 9.05167	10.09595, 11.27809, 6.30653	14.15669,	3	ddrgk1	12475
GL173004.1	140315	140516	140469, 140415, 140473	10, 6, 6	10.53228, 4.93312, 7.29403	7.01712, 4.04501, 5.30581	7.73927, 2	3	paip2b	0
GL173004.1	148135	148488	148295, 148288, 148340	9, 19, 6	8.45482, 17.58589, 6.30561	5.92419, 9.26517, 4.83031	5.79307, 1	3	paip2b	4211
GL173004.1	246055	246445	246370, 246363, 246369	9, 14, 5	9.36986, 13.75886, 5.19074	6.44997, 8.2515, 4.18277	6.65425, 1	3	tex261	0
GL173004.1	253098	254230	253382, 253571, 253550	7, 12, 9	5.93788, 11.04188, 8.47365	4.59878, 7.01971, 5.93535	3.52158, 8	3	tex261	4954
GL173004.1	273272	273693	273400, 273476, 273355	4, 9, 4	3.74195, 8.21099, 4.67325	3.32091, 5.77858, 3.78987	1.6222, 5.7	3	atp6v1b1	8279
GL173004.1	359712	361167	359784, 359952, 360475	5, 8, 12	4.33555, 4.81557, 13.66628	3.70555, 3.76783, 8.53679	2.15195, 2	3	atp6v1b1	31865
GL173004.1	570365	571090	571009, 571009, 570503	5, 10, 8	4.50727, 8.32357, 7.85773	3.80664, 5.70361, 5.66361	2.30125, 5	3	exoc6b	19485
GL173004.1	586603	587045	586970, 586722, 586927	11, 14, 9	6.15234, 8.19249, 6.56209	4.17423, 4.87218, 4.75161	3.69408, 5	3	exoc6b	35723
GL173004.1	588202	588782	588428, 588359, 588417	15, 19, 8	9.3336, 12.09967, 5.2088	5.34192, 6.06023, 4.02307	6.62173, 9	3	exoc6b	37322
GL173004.1	613107	613496	613362, 613494, 613276	11, 13, 8	10.43535, 9.43802, 8.09568	6.83063, 5.81956, 5.79794	7.6474, 6.8	3	NA	16428
GL173004.1	633574	634067	633667, 633912, 633775	10, 17, 8	9.23294, 17.11659, 9.46129	6.26141, 9.54398, 6.50801	6.52626, 1	3	cyp26b1	991
GL173004.1	699622	700084	699844, 699814, 699687	10, 11, 7	9.13239, 8.44639, 6.79502	6.2007, 5.60333, 5.10529	6.43114, 5	3	cyp26b1	45899
GL173004.1	744193	744809	744619, 744669, 744631	5, 15, 8	4.82177, 14.71623, 9.46129	3.98509, 8.60008, 6.50801	2.51418, 1	3	cyp26b1	90470
GL173004.1	784897	785807	785549, 785305, 785623	4, 13, 6	3.74195, 12.14594, 7.12188	3.32091, 7.49077, 5.22884	1.6222, 9.4	3	cyp26b1	131174
GL173004.1	811799	812064	811981, 811882, 812028	6, 7, 9	4.65447, 5.97725, 9.41119	3.87084, 4.62287, 6.47287	2.42652, 3	3	cyp26b1	158076
GL173004.1	822039	822411	822058, 822271, 822161	5, 8, 5	4.40263, 6.73981, 5.80393	3.74533, 4.99774, 4.48186	2.21004, 4	3	cyp26b1	168316
GL173004.1	872195	872831	872512, 872676, 872512	16, 18, 5	17.08121, 16.23409, 5.69847	9.7724, 8.73298, 4.4331	13.95894,	3	cyp26b1	218472
GL173004.1	898853	899348	898887, 899095, 898997	5, 9, 4	4.82177, 8.21099, 4.67325	3.98509, 5.77858, 3.78987	2.51418, 5	3	NA	214184
GL173004.1	970358	970757	970459, 970459, 970459	11, 11, 9	9.81032, 7.78921, 8.38703	6.45164, 5.19038, 5.88392	7.05644, 5	3	NA	142775
GL173004.1	971668	972232	971771, 971872, 971921	13, 12, 15	12.81131, 9.62333, 16.44983	7.8918, 6.14359, 9.62273	9.88323, 7	3	NA	141300
GL173004.1	1003173	1004343	1003872, 1003750, 1003448	6, 18, 8	5.63898, 16.52108, 8.35103	4.46544, 8.90737, 5.93881	3.25772, 1	3	NA	109189
GL173004.1	1065874	1066959	1066402, 1066422, 1066056	9, 6, 9	7.43495, 4.68027, 11.58206	5.30281, 3.88713, 7.55286	4.85899, 2	3	NA	46573
GL173004.1	1074565	1074930	1074904, 1074903, 1074899	23, 26, 15	26.3466, 26.31054, 19.17716	13.27146, 12.21864, 11.09709	22.92763,	3	NA	38602
GL173005.1	18731	19984	19334, 19932, 19856	7, 15, 13	4.81615, 10.97496, 13.15724	3.88767, 6.31545, 8.09715	2.51418, 8	3	prickle2	96915
GL173005.1	72905	73410	73380, 73180, 73309	16, 20, 9	15.34845, 19.43804, 9.94831	8.73591, 10.0867, 6.76298	12.2978, 1	3	prickle2	43489
GL173005.1	105635	106158	105973, 106118, 105906	12, 12, 6	12.97977, 10.2136, 6.08001	8.15868, 6.51056, 4.71063	10.04411,	3	prickle2	10741
GL173005.1	168859	169311	168875, 168902, 168880	10, 7, 7	10.07458, 5.97725, 7.2501	6.75761, 4.62287, 5.35703	7.30652, 3	3	prickle2	0
GL173005.1	207832	208289	208264, 208261, 208250	50, 79, 61	59.43943, 98.45537, 81.58612	21.3105, 28.16738, 28.46405	55.45837,	3	prickle2	4602
GL173005.1	236587	237234	237092, 237029, 236939	8, 10, 8	4.565, 6.16601, 5.94364	3.60521, 4.34187, 4.49672	2.3512, 3.8	3	psmd6	25602
GL173005.1	239092	240125	239320, 239468, 239278	33, 25, 21	33.64602, 22.65823, 21.86526	13.90715, 10.40252, 11.24011	30.06009,	3	psmd6	22711
GL173005.1	247986	249761	249218, 248640, 249081	45, 54, 52	26.71084, 32.18614, 38.58849	7.31442, 7.64577, 10.27038	23.28279,	3	psmd6	13075
GL173005.1	251266	252810	251784, 252246, 252338	62, 60, 51	41.5108, 33.51105, 33.80993	9.21141, 7.17184, 8.70953	37.78449,	3	psmd6	10026
GL173005.1	256371	257386	256885, 256832, 256788	29, 47, 39	14.6769, 26.58779, 27.97905	5.43975, 6.95263, 9.04138	11.66006,	3	psmd6	5450
GL173005.1	257745	258923	257808, 257812, 258469	20, 35, 18	10.15696, 19.75224, 11.28471	4.8713, 6.46745, 5.82707	7.38444, 1	3	psmd6	3913
GL173005.1	257745	258951	258748, 257812, 258469	19, 35, 18	9.6036, 19.75224, 11.28471	4.76285, 6.46745, 5.82707	6.87565, 1	3	psmd6	3885
GL173005.1	277673	279141	277863, 278330, 277865	28, 31, 18	24.89725, 24.95584, 17.25868	10.69565, 9.77204, 9.3554	21.51861,	3	atxn7	0
GL173005.1	348120	348731	348691, 348687, 348662	27, 47, 29	27.89898, 52.28061, 33.29793	12.80416, 18.63305, 15.26998	24.44182,	3	atxn7	9028
GL173005.1	491974	492701	492434, 492090, 492451	4, 6, 7	3.21195, 4.56637, 6.75301	3.00807, 3.81494, 5.08141	1.31375, 2	3	synpr	7664

GL173005.1	556859	557345	557300, 557307, 557329	20, 25, 17	21.18126, 27.7306, 21.41937	11.13591, 13.42198, 11.99417	17.91394,	3	synpr	72549
GL173005.1	568963	570161	569368, 569356, 569372	10, 15, 9	8.16254, 14.58692, 8.93877	5.60312, 8.52203, 6.20662	5.53763, 1	3	synpr	84653
GL173005.1	571505	572039	571865, 571794, 571809	6, 10, 5	4.83561, 9.17452, 4.57714	3.98453, 6.22617, 3.84705	2.52606, 6	3	synpr	87195
GL173005.1	577322	577919	577463, 577718, 577443	13, 6, 9	15.07528, 4.68027, 11.58206	9.18277, 3.88713, 7.55286	12.04083,	3	synpr	93012
GL173005.1	653875	654314	654122, 654120, 654013	17, 13, 4	16.83467, 12.14594, 4.67325	9.37407, 7.49077, 3.78987	13.72157,	3	cadps	95709
GL173005.1	746806	747188	746870, 746876, 746910	12, 17, 9	13.38718, 17.56014, 10.79992	8.38496, 9.80985, 7.19283	10.43197,	3	cadps	2835
GL173005.1	759110	759687	759122, 759182, 759143	8, 16, 6	8.42257, 14.98328, 5.60548	5.97763, 8.51381, 4.44624	5.76336, 1	3	cadps	0
GL173005.1	768529	769161	768990, 769160, 768545, 769095	9, 8, 4, 3	8.82885, 5.96021, 4.34116, 3.27564	6.14328, 4.50731, 3.63492, 2.93941	6.14693, 3	3	cadps	0
GL173005.1	936326	936690	936549, 936528, 936492	11, 18, 7	10.43535, 18.91825, 8.42533	6.83063, 10.35485, 5.94313	7.6474, 15	3	cadps	0
GL173005.1	952919	953732	953415, 952998, 953110	10, 18, 8	9.83337, 14.99509, 8.29845	6.61776, 7.98278, 5.91009	7.07841, 1	3	cadps	0
GL173005.1	961890	962400	962213, 962044, 962210	28, 34, 21	28.36137, 34.91002, 24.28039	12.71156, 14.24619, 12.67014	24.89271,	3	cadps	0
GL173005.1	1024184	1024549	1024464, 1024308, 1024386	7, 7, 5	6.55439, 5.58561, 4.67395	4.96711, 4.38027, 3.90232	4.06616, 3	3	cadps	0
GL173005.1	1048534	1048917	1048659, 1048581, 1048668	11, 9, 7	12.43693, 7.75488, 7.96278	7.97018, 5.501, 5.72394	9.52692, 5	3	fezf2	12787
GL173005.1	1068713	1069578	1069021, 1069026, 1068989	11, 18, 6	10.27095, 18.29879, 6.5517	6.73177, 9.984, 4.95623	7.4917, 15	3	fezf2	2687
GL173005.1	1110184	1111038	1110459, 1110459, 1110459	15, 12, 11	16.54256, 10.53798, 12.63525	9.6759, 6.71099, 8.07536	13.44484,	3	NA	31202
GL173005.1	1115640	1116270	1116178, 1115957, 1116002	13, 13, 11	15.29169, 12.80191, 14.75772	9.29854, 7.88619, 9.09568	12.24396,	3	NA	25970
GL173005.1	1173492	1174104	1173582, 1173575, 1173514	21, 21, 18	18.46055, 16.53423, 16.71968	9.19276, 8.06034, 9.02806	15.2852, 1	3	NA	0
GL173006.1	320901	321664	321341, 321454, 321246	23, 49, 17	25.52391, 50.08484, 20.09113	12.78506, 16.56139, 11.27497	22.12768,	3	plod2	188402
GL173006.1	417588	418406	418125, 417756, 417809	16, 25, 8	14.51319, 25.26956, 8.24665	8.22691, 11.95039, 5.88165	11.50268,	3	c3orf58	184283
GL173006.1	433773	434823	434358, 434205, 434288	14, 26, 18	10.90232, 20.32594, 16.93059	6.50142, 8.76992, 9.15621	8.08848, 1	3	c3orf58	167866
GL173006.1	918713	919358	919102, 918942, 918768	6, 10, 5	5.96715, 9.39128, 5.9506	4.64927, 6.35644, 4.54784	3.52158, 6	3	slc9a9	63195
GL173007.1	240506	240920	240738, 240626, 240677	9, 6, 4	9.63662, 4.93312, 4.46488	6.59633, 4.04501, 3.69425	6.90556, 2	3	uncx	78392
GL173007.1	1003442	1004694	1003962, 1003835, 1003964	6, 14, 8	4.27757, 10.18813, 8.35103	3.62922, 6.06346, 5.93881	2.10191, 7	3	gnptg	4988
GL173008.1	39	966	318, 492, 173	36, 34, 17	40.33483, 35.5202, 20.57409	16.59351, 14.6006, 11.54114	36.62727,	3	NA	74921
GL173008.1	37066	37849	37491, 37520, 37394	13, 41, 14	11.55805, 42.14993, 14.66346	7.13125, 15.53337, 8.7878	8.70034, 3	3	NA	38038
GL173008.1	58209	58771	58251, 58239, 58235	26, 36, 22	27.72954, 39.73859, 27.8223	13.06627, 16.24309, 14.40691	24.27641,	3	NA	17116
GL173008.1	70976	71345	71200, 71178, 71199	9, 12, 8	9.30591, 11.66217, 9.05167	6.41439, 7.39386, 6.30653	6.59443, 8	3	NA	4542
GL173008.1	116294	116869	116621, 116682, 116642	8, 8, 6	8.34715, 6.27644, 6.5517	5.9367, 4.70819, 4.95623	5.71011, 3	3	NA	38071
GL173008.1	127916	128207	128035, 127987, 127944	4, 9, 4	3.21195, 6.43335, 4.38152	3.00807, 4.66944, 3.65448	1.31375, 4	3	NA	49693
GL173008.1	238674	239183	239156, 239157, 239139	21, 33, 25	19.90701, 33.12122, 28.46321	10.06042, 13.6033, 13.85724	16.68036,	3	ALK	0
GL173008.1	324440	325338	324799, 324774, 325290	8, 15, 5	6.52004, 13.74993, 5.85847	4.86121, 8.013, 4.50665	4.03345, 1	3	NA	1880
GL173008.1	336466	337079	336786, 336749, 336768	16, 20, 4	16.52811, 16.49115, 4.34116	9.44553, 8.31699, 3.63492	13.43105,	3	NA	0
GL173008.1	348382	349602	349153, 348970, 349013	23, 40, 22	23.8944, 40.75769, 25.21195	11.81108, 15.16353, 12.91737	20.54405,	3	NA	9630
GL173008.1	431459	431961	431621, 431512, 431581	22, 24, 16	22.52709, 21.77349, 19.066	11.31895, 10.21126, 10.89996	19.2175, 1	3	ppp1cb	0
GL173008.1	461613	462076	462046, 461926, 462044	18, 21, 7	18.22458, 21.41771, 8.69489	9.93936, 10.97106, 6.06379	15.0589, 1	3	plb1	20215
GL173008.1	492170	492914	492217, 492194, 492208	10, 15, 16	10.6018, 14.91533, 20.18713	7.05583, 8.71987, 11.49707	7.80484, 1	3	plb1	0
GL173008.1	518975	519614	519248, 519172, 519335	10, 11, 5	10.01299, 10.60895, 5.9506	6.7221, 6.9343, 4.54784	7.24763, 7	3	plb1	0
GL173008.1	534391	535160	534704, 534723, 534679	7, 9, 4	7.1696, 8.21099, 4.67325	5.31345, 5.77858, 3.78987	4.61046, 5	3	plb1	0
GL173008.1	542507	542888	542671, 542662, 542687	10, 10, 13	9.03421, 9.39128, 15.52427	6.14116, 6.35644, 9.42125	6.33915, 6	3	plb1	0
GL173008.1	602975	603481	603453, 603296, 603446	14, 16, 8	13.96392, 13.83763, 9.1831	8.37431, 7.81372, 6.37229	10.9769, 1	3	fosl2	0
GL173008.1	628204	628597	628555, 628543, 628556	11, 25, 21	10.27095, 23.09161, 25.2565	6.73177, 10.65679, 13.23126	7.4917, 19	3	fosl2	20310
GL173008.1	633749	634515	634240, 634148, 634485	5, 16, 9	4.82177, 15.41438, 11.64359	3.98509, 8.77591, 7.57973	2.51418, 1	3	bre	19838
GL173008.1	646626	647307	646894, 647129, 646895	7, 9, 6	7.1696, 8.21099, 7.29403	5.31345, 5.77858, 5.30581	4.61046, 5	3	bre	7046
GL173008.1	695926	696881	695955, 695970, 695980	38, 37, 21	41.48602, 39.69337, 24.95238	16.41423, 15.79594, 13.05777	37.75999,	3	bre	0
GL173008.1	718692	719159	719028, 719057, 719111	8, 17, 8	7.24433, 16.3049, 8.6841	5.30475, 9.05325, 6.11715	4.68023, 1	3	bre	0
GL173008.1	755747	756408	756308, 756228, 756255	7, 36, 9	4.89211, 37.44002, 9.82249	3.93703, 14.9052, 6.69629	2.57963, 3	3	bre	0
GL173008.1	760626	761486	761143, 761146, 760759	11, 13, 7	8.92872, 9.08335, 6.43637	5.90554, 5.60136, 4.89813	6.24102, 6	3	bre	0
GL173008.1	788842	789261	789033, 788881, 789009	6, 10, 6	5.09949, 8.0878, 6.30561	4.14694, 5.55635, 4.83031	2.76939, 5	3	rbks	0
GL173008.1	832882	833139	833038, 833012, 833038	13, 17, 15	13.42064, 16.97468, 17.23823	8.25183, 9.45853, 10.06864	10.46414,	3	rbks	27455
GL173008.1	888127	888584	888385, 888171, 888432	7, 10, 4	6.55439, 8.9331, 4.11547	4.96711, 6.07959, 3.5218	4.06616, 6	3	slc4a1ap	25744
GL173008.1	895166	895562	895196, 895224, 895270	5, 11, 4	4.82177, 10.60895, 4.67325	3.98509, 6.9343, 3.78987	2.51418, 7	3	slc4a1ap	18766
GL173008.1	917439	918024	917826, 917712, 917625	9, 10, 9	8.40425, 7.35725, 11.64359	5.89416, 5.09584, 7.57973	5.76336, 4	3	slc4a1ap	0
GL173008.1	926799	927310	926826, 926817, 926808	33, 52, 34	35.83524, 58.77035, 44.39305	15.1895, 19.97934, 19.82081	32.20474,	3	supt7l	0
GL173008.1	939391	940050	939908, 939585, 939474	6, 12, 5	5.96715, 11.48527, 5.9506	4.64927, 7.28792, 4.54784	3.52158, 8	3	supt7l	3872
GL173008.1	947806	948151	948102, 948061, 948087	7, 18, 13	7.1696, 19.97713, 16.18511	5.31345, 10.97931, 9.7598	4.61046, 1	3	supt7l	12287
GL173008.1	1007238	1007671	1007282, 1007281, 1007453	7, 8, 4	6.2923, 7.06103, 3.97874	4.81292, 5.19433, 3.45022	3.8235, 4.6	3	supt7l	71719
GL173008.1	1035184	1035802	1035800, 1035760, 1035754	15, 23, 15	15.12865, 21.57332, 17.67167	8.84764, 10.41915, 10.30745	12.09208,	3	supt7l	99665
GL173008.1	1080675	1081180	1080877, 1081028, 1080813	5, 9, 8	4.82177, 7.88274, 9.1831	3.98509, 5.57944, 6.37229	2.51418, 5	3	supt7l	145156
GL173008.1	1101154	1101618	1101527, 1101486, 1101591	9, 9, 5	8.55802, 7.79702, 5.85847	5.98518, 5.5269, 4.50665	5.8909, 5.3	3	supt7l	165635
GL173009.1	771065	771608	771395, 771252, 771323	11, 14, 4	9.95877, 8.38214, 3.14704	6.54239, 4.98248, 2.96768	7.19682, 5	3	ngf	21796
GL173009.1	772035	773510	772558, 773157, 773211	10, 22, 11	8.66311, 16.27156, 11.6121	5.914, 7.6398, 7.51665	5.99101, 1	3	ngf	19894
GL173010.1	113702	114154	113845, 114115, 113929	5, 11, 17	4.82177, 9.39347, 19.72642	3.98509, 6.19476, 11.07071	2.51418, 6	3	cldn8.3	25106
GL173010.1	152019	152607	152305, 152129, 152085	10, 9, 5	10.67246, 7.06866, 4.70718	7.09497, 5.07306, 3.9211	7.87096, 4	3	NA	6777
GL173010.1	379402	379947	379431, 379422, 379427	16, 23, 9	16.37806, 21.50896, 9.41119	9.35612, 10.38076, 6.47287	13.28648,	3	tiam1	0
GL173010.1	577909	578372	578115, 578151, 578288	7, 11, 5	5.93788, 9.17879, 5.9506	4.59878, 6.06147, 4.54784	3.52158, 6	3	hunk	0
GL173010.1	765218	765756	765408, 765631, 765666	6, 11, 13	4.40555, 9.30635, 13.97446	3.71197, 6.14075, 8.57174	2.21247, 6	3	urb1	709
GL173010.1	780303	781042	780715, 780868, 780895	14, 18, 12	15.23546, 17.86551, 12.20749	9.11858, 9.72285, 7.71596	12.19387,	3	urb1	15794
GL173010.1	865719	866514	865935, 865942, 865948	50, 56, 31	57.29142, 60.0756, 38.6896	20.0836, 18.87999, 17.67953	53.33783,	3	NA	2169
GL173010.1	1043742	1045205	1044595, 1044592, 1044683	40, 72, 32	44.86061, 79.77496, 38.29311	17.50949, 21.89376, 17.05263	41.08385,	3	c21orf62	44563
GL173010.1	1073842	1074202	1074067, 1074074, 1074081	17, 19, 15	16.56188, 17.58589, 15.36085	9.20909, 9.26517, 8.986	13.46342,	3	olig2	23612
GL173010.1	1075939	1076210	1076182, 1076117, 1076170	8, 14, 10	6.01859, 11.45303, 9.13706	4.54456, 6.84088, 6.20353	3.56839, 8	3	olig2	21604
GL173010.1	1107625	1108066	1107683, 1107897, 1107892	7, 30, 14	6.60038, 33.00599, 16.46576	4.99378, 14.70932, 9.80275	4.10761, 2	3	olig2	8943
GL173010.1	1126416	1126854	1126502, 1126526, 1126637	17, 24, 13	16.97511, 21.1884, 15.98838	9.45879, 9.86789, 9.66061	13.85654,	3	NA	17565
GL173010.1	1156489	1157186	1157040, 1156776, 1157133	15, 21, 9	16.36908, 22.39999, 9.76105	9.57629, 11.56043, 6.66343	13.27778,	3	NA	11553
GL173012.1	319433	319806	319547, 319546, 319546	11, 9, 7	10.96672, 7.39577, 7.61369	7.14544, 5.27837, 5.54854	8.14942, 4	3	NA	293
GL173012.1	332817	333378	332846, 332845, 332844	112, 142, 78	143.65126, 179.64822, 104.98189	33.84067, 35.30949, 32.33361	138.96034	3	NA	8804
GL173012.1	374928	375171	375169, 375170, 375041	6, 13, 4	5.55471, 11.12608, 3.48964	4.41698, 6.86492, 3.17588	3.18192, 8	3	anp32e	13530
GL173012.1	501962	502570	502262, 502291, 502015	6, 12, 5	4.45871, 9.16947, 4.25472	3.74614, 5.86046, 3.65711	2.26108, 6	3	vps45	0
GL173012.1	529919	530108	530076, 529947, 529946	9, 20, 8	8.45482, 19.85827, 9.46129	5.92419, 10.34058, 6.50801	5.79307, 1	3	vps45	10577
GL173012.1	604988	605567	605155, 605156, 605147	10, 14, 9	8.57531, 12.18837, 9.30116	5.85982, 7.29433, 6.41174	5.90712, 9	3	otud7b	0
GL173012.1	633228	633772	633738, 633738, 633700	30, 25, 21	33.18705, 25.34813, 23.8295	14.81701, 11.99737, 12.40706	29.60997,	3	mtmr11	17385
GL173012.1	692752	693680	693178, 693178, 693179	27, 49, 14	26.32521, 49.64961, 12.16551	11.87051, 16.33097, 7.28026	22.90704,	3	sf3b4	16607
GL173012.1	766557	767339	766562, 766658, 766616	27, 36, 17	30.99178, 39.22969, 19.37904	14.64748, 15.94492, 10.87373	27.46196,	3	BOLA1	11583
GL173012.1	817556	818181	818004, 818005, 818004	16, 17, 14	15.60786, 16.11578, 17.61756	8.89312, 8.93835, 10.40352	12.5472, 1	3	nr2f5	21020
GL173012.1	828077	828729	828267, 828548, 828673	7, 13, 3	7.10297, 11.81904, 3.34298	5.27706, 7.29135, 2.97156	4.57191, 9	3	nr2f5	10472
GL173012.1	938789	939845	939405, 939545, 939145, 939578	8, 17, 5, 3	8.10827, 18.19786, 5.9506, 3.47371	5.80497, 10.18828, 4.54784, 3.03189	5.48761, 1	3	hfe2	39344
GL173012.1	1110117	1110693	1110418, 1110460, 1110465	11, 24, 15	9.81032, 22.20517, 18.33148	6.45164, 10.46619, 10.66149	7.05644, 1	3	polr3gl	17488
GL173012.1	1151378	1152560	1152047, 1152065, 1151829	21, 37, 14	21.33165, 40.38358, 16.12551	10.91925, 16.19746, 9.61761	18.05947,	3	ankrd34a	16133
GL173013.1	46770	47368	46831, 46819, 46811	8, 11, 5	8.10827, 10.60895, 5.64747	5.80497, 6.9343, 4.40911	5.48761, 7	3	mast1	2056
GL173013.1	64960	65735	64964, 64969, 64987	29, 45, 37	32.65865, 49.32181, 44.31162	14.88989, 17.85668, 18.50946	29.09305,	3	rtbdn	11922
GL173013.1	137424	138175	137967, 137953, 138004	8, 10, 7	6.78332, 8.4887, 7.78342	5.02459, 5.80621, 5.63488	4.27811, 5	3	junb	4298
GL173013.1	199854	201009	200544, 200623, 200558	15, 16, 9	13.97429, 15.35105, 9.58224	8.15, 8.73748, 6.56676	10.98644,	3	asna1	0
GL173013.1	216825	217617	216839, 216834, 216840	16, 21, 15	15.94701, 19.99241, 16.99143	9.09776, 10.11187, 9.93059	12.87323,	3	XB-GENE-5888819	1477
GL173013.1	555208	555912	555781, 555661, 555758	11, 13, 9	10.16451, 9.20941, 10.14484	6.66744, 5.67878, 6.86555	7.39114, 6	3	nfyc	7077
GL173013.1	1142834	1143699	1143211, 1143011, 1143239	17, 14, 9	17.48974, 13.0902, 10.42289	9.76779, 7.84698, 7.00726	14.35103,	3	NA	148251
GL173015.1	3815	4962	4264, 4201, 4400	5, 10, 9	4.77263, 8.04981, 10.42289	3.9578, 5.53254, 7.00726	2.51418, 5	3	ndufv1	71233
GL173015.1	42780	43440	43137, 43108, 43207	21, 29, 10	22.05757, 29.60014, 9.63365	11.35548, 13.07229, 6.50047	18.76195,	3	ndufv1	32755
GL173015.1	70620	71181	70789, 70865, 70808	5, 10, 4	3.9168, 7.51937, 4.67325	3.44908, 5.19842, 3.78987	1.78158, 5	3	ndufv1	5014
GL173015.1	473328	474518	473622, 473930, 474110	14, 15, 7	12.48519, 11.41281, 6.55126	7.47686, 6.58101, 4.96529	9.57251, 8	3	foxh1	0
GL173015.1	477344	478132	477374, 477494, 477383	19, 21, 13	17.87921, 18.11284, 14.25214	9.44292, 8.98591, 8.72916	14.72728,	3	foxh1	0
GL173015.1	483687	483985	483732, 483906, 483821	15, 13, 9	14.71973, 11.07988, 9.64095	8.60219, 6.83637, 6.59867	11.70031,	3	foxh1	732
GL173015.1	711633	712772	712287, 712303, 712367	18, 14, 12	16.21741, 10.72722, 11.92663	8.72284, 6.39373, 7.55094	13.13331,	3	NA	36943
GL173015.1	784478	785030	784606, 784607, 784610	16, 40, 15	15.09881, 45.02302, 18.13606	8.58417, 17.60406, 10.55788	12.06341,	3	NA	17110
GL173015.1	839117	840439	839380, 839286, 839986	4, 8, 15	3.74195, 5.96021, 17.58264	3.32091, 4.50731, 10.25879	1.6222, 3.6	3	NA	0
GL173015.1	894016	895057	894795, 894504, 894155	8, 13, 5	7.19971, 11.71436, 4.84526	5.27799, 7.22722, 3.99806	4.63758, 9	3	NA	27225
GL173015.1	1143480	1143967	1143736, 1143736, 1143764	15, 19, 10	13.46874, 16.83862, 10.1418	7.84087, 8.81301, 6.79622	10.51051,	3	a2m	17238
GL173015.1	1168170	1168631	1168240, 1168248, 1168250	17, 39, 31	14.95512, 34.35428, 33.72127	8.23125, 12.08925, 14.74096	11.92636,	3	a2m	41928
GL173016.1	77201	77529	77328, 77249, 77232	5, 7, 10	4.82177, 5.07986, 12.33934	3.98509, 4.05845, 7.95424	2.51418, 2	3	tspan8	62403
GL173017.1	5083	5687	5284, 5290, 5160	10, 12, 10	11.06011, 11.86108, 11.5283	7.306, 7.51216, 7.55211	8.22712, 9	3	nucb1	0
GL173017.1	49137	50003	49694, 49799, 49548	8, 11, 8	7.93925, 8.59076, 8.4586	5.70996, 5.69393, 5.99709	5.33002, 6	3	dhdh	745
GL173017.1	96446	97457	96819, 96561, 96542	13, 12, 10	12.94099, 11.86108, 11.83521	7.96907, 7.51216, 7.70798	10.00729,	3	XB-GENE-968486	36116
GL173017.1	156499	156754	156717, 156674, 156699	21, 18, 13	23.41865, 17.86551, 14.5443	12.1655, 9.72285, 8.89247	20.08217,	3	NA	0
GL173017.1	224639	224920	224733, 224664, 224667	6, 13, 5	5.90927, 11.26714, 4.51488	4.61743, 6.95205, 3.81106	3.50164, 8	3	NA	0
GL173017.1	248786	249454	248960, 248929, 248926	5, 11, 12	4.82177, 10.60895, 14.96175	3.98509, 6.9343, 9.20442	2.51418, 7	3	NA	13429
GL173017.1	358157	358511	358311, 358294, 358342	4, 13, 11	3.23728, 10.7674, 11.79996	3.02372, 6.64292, 7.62212	1.33324, 8	3	aldh16a1	9267
GL173017.1	460157	460615	460395, 460321, 460229	7, 15, 3	5.86458, 14.09492, 3.34298	4.55375, 8.22351, 2.97156	3.46146, 1	3	slc17a7	6260
GL173017.1	612708	613489	612775, 613030, 612895	15, 22, 12	15.87516, 22.00083, 13.7434	9.2894, 11.00232, 8.57827	12.80377,	3	NA	1023
GL173017.1	846428	847022	846450, 846619, 846598	6, 11, 7	5.96715, 9.57292, 8.69489	4.64927, 6.30569, 6.06379	3.52158, 6	3	MYBPC2	0
GL173018.1	90093	90811	90807, 90782, 90711	45, 66, 21	53.46264, 79.22325, 26.58709	20.23946, 24.47101, 13.97388	49.56168,	3	cxcr3	7056
GL173018.1	111296	111803	111480, 111600, 111595	23, 14, 7	23.56695, 11.07823, 7.20145	11.61446, 6.60976, 5.33074	20.22607,	3	cxcr3	28259
GL173018.1	381927	382448	382297, 382438, 382090	12, 11, 3	13.3031, 10.60895, 3.47371	8.33871, 6.9343, 3.03189	10.35242,	3	syt3	77922
GL173018.1	775696	776118	775936, 775727, 775942	8, 5, 5	7.57423, 3.21429, 5.31857	5.4999, 2.99211, 4.24819	4.98865, 1	3	shank1	55481
GL173018.1	873787	875060	874008, 874020, 874004	25, 52, 27	26.32546, 55.66893, 32.8241	12.58233, 18.27712, 15.7255	22.90704,	3	NA	29439
GL173019.1	40	462	240, 168, 162	15, 20, 12	14.58913, 19.93044, 13.29687	8.52337, 10.38414, 8.33527	11.57535,	3	plppr1	104515
GL173019.1	933	1291	1012, 1145, 1080	13, 12, 8	12.09198, 10.08092, 7.99854	7.45795, 6.42828, 5.74345	9.20698, 7	3	plppr1	103686
GL173019.1	6270	7236	6971, 7040, 6555, 6990	9, 15, 6, 10	9.12009, 14.15433, 5.91225, 10.97191	6.30997, 8.25968, 4.61907, 7.25854	6.42028, 1	3	plppr1	97741
GL173019.1	19303	19825	19676, 19641, 19674	9, 14, 11	9.24298, 14.45897, 14.75772	6.3792, 8.66788, 9.09568	6.53474, 1	3	plppr1	85152
GL173019.1	29022	29568	29243, 29150, 29341	6, 13, 5	5.96715, 12.03475, 5.91431	4.64927, 7.4231, 4.53172	3.52158, 9	3	plppr1	75409
GL173019.1	765752	766813	766294, 766309, 766207	10, 14, 7	8.32267, 9.95387, 6.6707	5.70305, 5.92073, 5.03432	5.6878, 7.3	3	NA	334582
GL173019.1	901176	903761	903293, 903293, 903280	14, 20, 21	14.23954, 19.78674, 23.23425	8.53829, 10.29739, 12.05658	11.24094,	3	NA	197634
GL173019.1	909707	910713	909982, 910313, 910447	27, 19, 17	31.91719, 21.4153, 22.12333	15.19434, 11.55717, 12.35799	28.36766,	3	NA	190682
GL173019.1	917261	918054	917659, 917651, 917669	23, 44, 25	29.93233, 53.41446, 32.82711	15.31008, 20.68337, 16.36153	26.42616,	3	NA	183341
GL173019.1	922839	924450	923208, 923212, 923216	51, 61, 38	48.70491, 56.99084, 40.05313	15.0682, 15.40623, 15.58636	44.87049,	3	NA	176945
GL173019.1	1117884	1118425	1118036, 1118226, 1117943	14, 13, 4	14.45502, 9.85593, 3.76128	8.66555, 6.07784, 3.33171	11.4469, 7	3	NA	5325
GL173022.1	425504	426106	425595, 425963, 425960	6, 9, 7	5.96715, 8.21099, 8.69489	4.64927, 5.77858, 6.06379	3.52158, 5	3	XB-GENE-5806148	11639
GL173023.1	52774	53985	53584, 53766, 52886	12, 11, 6	13.47263, 9.95469, 6.99807	8.43173, 6.5399, 5.17195	10.51396,	3	naaa	5505
GL173023.1	103537	104047	103735, 103753, 103752	24, 36, 22	25.36666, 37.79934, 25.03136	12.3555, 15.11266, 12.8114	21.97541,	3	NA	858
GL173024.1	514836	515324	515279, 515296, 515276	23, 23, 24	28.76847, 24.15665, 32.3013	14.6664, 11.96843, 16.33791	25.29064,	3	sgcg	0
GL173024.1	753453	753972	753758, 753738, 753740	18, 26, 7	19.00683, 24.56489, 7.96278	10.40758, 11.18383, 5.72394	15.81175,	3	mipep	0
GL173024.1	805563	805835	805808, 805705, 805779	8, 11, 7	6.55639, 8.04011, 6.6707	4.88389, 5.34803, 5.03432	4.06705, 5	3	spata13	0
GL173024.1	977240	977876	977698, 977641, 977674	15, 20, 8	13.36183, 18.78201, 8.4586	7.77533, 9.69018, 5.99709	10.40776,	3	NA	115625
GL173024.1	1051881	1052794	1052096, 1052104, 1051978	12, 13, 7	10.58505, 10.39132, 7.01416	6.73997, 6.40973, 5.22813	7.78888, 7	3	NA	40707
GL173024.1	1127110	1127489	1127283, 1127332, 1127299	13, 7, 7	9.48728, 3.43353, 5.88867	5.84994, 2.9775, 4.56857	6.76599, 1	3	NA	24362
GL173025.1	488475	489108	488804, 488840, 488723	15, 36, 11	14.21298, 37.52903, 11.20072	8.29535, 14.95653, 7.28154	11.21566,	3	mrpl17	0
GL173025.1	687983	688752	688391, 688392, 688405	17, 21, 10	19.01263, 19.54437, 10.97191	10.66355, 9.8421, 7.25854	15.81687,	3	arel1	0
GL173026.1	208010	208930	208608, 208575, 208619	16, 21, 22	18.64741, 21.65316, 27.01579	10.66905, 11.1127, 13.95629	15.46487,	3	b3galt6	12814
GL173026.1	638983	640636	639986, 639909, 639992	27, 38, 24	26.47292, 34.27769, 26.22638	11.9577, 12.39331, 12.86969	23.05104,	3	pusl1	0
GL173026.1	957564	958693	958010, 957965, 957966	68, 73, 45	67.67241, 73.24841, 48.18729	17.73974, 18.38808, 17.21846	63.6004, 6	3	mrpl20	93172
GL173027.1	88145	89046	88797, 88821, 88434	13, 22, 10	13.79066, 18.83145, 11.38247	8.46641, 9.10909, 7.47651	10.8171, 1	3	GRIK3	345154
GL173029.1	2714	3286	3012, 2962, 2740	16, 31, 12	13.19919, 26.39521, 10.30599	7.42362, 10.53981, 6.56776	10.2543, 2	3	NA	7920
GL173029.1	5978	6371	6176, 6142, 6142	20, 31, 31	15.34896, 22.27583, 32.59187	7.64809, 8.41475, 14.07266	12.29825,	3	NA	4835
GL173029.1	10345	10868	10571, 10615, 10690	19, 21, 11	15.64357, 15.36067, 8.57182	8.09525, 7.3913, 5.68205	12.58155,	3	NA	338
GL173029.1	162885	163500	163444, 163265, 163200	7, 8, 5	6.2098, 6.05185, 5.50079	4.76362, 4.56575, 4.33868	3.74679, 3	3	ACOX3	0
GL173029.1	217402	220163	219085, 219196, 218530, 220127	38, 45, 26, 12	29.09911, 34.97483, 24.11382, 8.00253	9.80991, 10.5587, 10.9191, 5.13501	25.61349,	3	SH3TC1	6676
GL173029.1	232628	233254	233158, 232662, 232786	10, 7, 5	9.13239, 5.68499, 5.9506	6.2007, 4.44244, 4.54784	6.43114, 3	3	SH3TC1	0
GL173029.1	279455	281166	279709, 279857, 279797	56, 60, 40	41.13136, 38.91181, 32.56376	10.3034, 8.74926, 10.87898	37.41076,	3	med28	18963
GL173029.1	527449	527731	527476, 527498, 527502	11, 12, 7	11.42621, 11.37069, 8.49722	7.41109, 7.21896, 5.97582	8.57514, 8	3	ldb2	40082
GL173029.1	700498	700972	700711, 700718, 700825	6, 7, 5	4.83561, 5.07986, 4.77515	3.98453, 4.05845, 3.9592	2.52606, 2	3	prom1	8904
GL173029.1	888925	889857	889641, 889425, 889680	17, 22, 15	17.19112, 19.53861, 16.30311	9.58879, 9.52696, 9.53825	14.0641, 1	3	CC2D2A	0
GL173029.1	915707	916054	915719, 915784, 915748	5, 10, 7	4.50727, 7.51937, 8.02489	3.80664, 5.19842, 5.75426	2.30125, 5	3	cc2d2a	1959
GL173030.1	83165	84033	83453, 83453, 83414	7, 10, 7	6.64707, 8.12616, 8.35502	5.02073, 5.58036, 5.9108	4.15135, 5	3	NA	8414
GL173030.1	272975	273630	273548, 273459, 273281	9, 16, 10	7.28297, 15.54288, 11.24131	5.20782, 8.85379, 7.40241	4.7171, 12	3	NA	4408
GL173030.1	279863	280867	280499, 280430, 280318	13, 30, 21	12.49994, 30.89514, 26.35131	7.70504, 13.45807, 13.84438	9.58678, 2	3	NA	11296
GL173030.1	740551	740796	740649, 740620, 740598	9, 7, 10	8.35434, 4.97304, 11.997	5.86444, 3.98948, 7.78835	5.71584, 2	3	tbc1d1	0
GL173031.1	71419	72042	71741, 71612, 71674	8, 19, 15	5.95727, 18.6168, 17.94632	4.50543, 9.88966, 10.45626	3.52158, 1	3	cat2	1739
GL173031.1	135159	135396	135348, 135343, 135365	15, 33, 17	13.20489, 31.31977, 19.63801	7.67906, 12.57463, 11.0208	10.25934,	3	cat2	26236
GL173031.1	142595	142874	142635, 142652, 142718	9, 19, 6	9.72082, 18.41287, 6.25896	6.64182, 9.76624, 4.80589	6.97155, 1	3	cat2	33672
GL173031.1	261789	262738	261909, 261819, 261829	26, 41, 15	28.00593, 39.55822, 18.13606	13.23147, 14.11066, 10.55788	24.54626,	3	tatdn1	0
GL173031.1	470471	471095	470691, 470716, 470493	15, 17, 15	16.03559, 17.95202, 19.40672	9.3831, 10.043, 11.21161	12.95858,	3	trib1	11125
GL173031.1	481865	482428	482119, 481980, 482320	7, 21, 6	5.97522, 16.98161, 6.08001	4.62162, 8.32003, 4.71063	3.52869, 1	3	trib1	22519
GL173031.1	513822	514206	514102, 514133, 514118	6, 13, 7	5.3552, 11.36332, 8.49722	4.3003, 7.01137, 5.97582	3.00253, 8	3	trib1	54476
GL173031.1	885248	885822	885676, 885658, 885735	6, 5, 4	5.55471, 3.94561, 4.67325	4.41698, 3.46715, 3.78987	3.18192, 1	3	myc	149722
GL173031.1	931322	931587	931480, 931567, 931505	9, 9, 8	9.06007, 6.73685, 9.05167	6.27591, 4.86289, 6.30653	6.36394, 4	3	myc	103957
GL173031.1	1001098	1001389	1001169, 1001260, 1001137	15, 11, 14	17.18944, 8.44639, 17.01463	10.04146, 5.60333, 10.0942	14.06311,	3	myc	34155
GL173031.1	1028022	1028314	1028049, 1028059, 1028035	14, 20, 13	14.23954, 19.50661, 14.93222	8.53829, 10.12815, 9.10541	11.24094,	3	myc	7230
GL173031.1	1044613	1045096	1044778, 1044832, 1044661	17, 18, 12	16.90454, 14.99509, 13.7434	9.41624, 7.98278, 8.57827	13.78827,	3	myc	5281
GL173032.1	52542	53218	52875, 53117, 52991	8, 9, 9	6.41335, 6.55163, 9.30116	4.7944, 4.74494, 6.41174	3.93678, 4	3	hla-dqa2	26385
GL173032.1	68043	68904	68310, 68441, 68110, 68757	6, 16, 5, 7	5.96715, 16.98517, 5.9506, 8.69489	4.64927, 9.71597, 4.54784, 6.06379	3.52158, 1	3	hla-dqa2	41886
GL173032.1	161546	161967	161848, 161830, 161749	24, 31, 9	29.94242, 36.23515, 10.72164	15.04037, 16.23378, 7.15493	26.43594,	3	hla-drb1	89942
GL173032.1	588303	589170	588936, 588772, 589040	12, 14, 10	11.43506, 14.29802, 13.18184	7.25774, 8.57291, 8.33771	8.58315, 1	3	NA	12191
GL173033.1	106261	107265	106776, 106766, 106511	7, 8, 4	6.50909, 6.51779, 3.79082	4.94073, 4.8598, 3.34814	4.02371, 4	3	mrpl38	20564
GL173033.1	536562	537530	536897, 537112, 536697	14, 25, 9	13.70063, 22.24272, 10.01275	8.21651, 10.1601, 6.79683	10.73143,	3	tspan9	0
GL173033.1	592893	593754	593623, 593231, 593232	9, 10, 6	8.0679, 8.44678, 6.87953	5.69219, 5.78021, 5.11627	5.45, 5.932	3	tspan9	0
GL173033.1	666839	667572	666998, 667370, 667215	6, 11, 5	5.43318, 9.39347, 4.57714	4.34622, 6.19476, 3.84705	3.07284, 6	3	tspan9	0
GL173033.1	829078	829400	829235, 829278, 829299	6, 20, 12	5.90927, 20.07667, 14.76955	4.61743, 10.47238, 9.10943	3.50164, 1	3	NA	28904
GL173033.1	909914	911016	910788, 910484, 910866	15, 24, 11	15.19946, 22.99283, 12.86886	8.88991, 10.93416, 8.19722	12.15964,	3	prmt8	25772
GL173033.1	949786	950470	950217, 950218, 949900	8, 14, 9	8.22568, 14.15854, 10.49552	5.8701, 8.49024, 7.0436	5.59681, 1	3	prmt8	0
GL173033.1	1016520	1017556	1017172, 1017065, 1016952	11, 13, 6	10.16451, 11.81904, 6.2131	6.66744, 7.29135, 4.78171	7.39114, 9	3	prmt8	41037
GL173033.1	1036336	1037124	1036412, 1036710, 1037059	12, 9, 5	11.43506, 7.47261, 4.88115	7.25774, 5.32627, 4.01777	8.58315, 5	3	prmt8	60853
GL173033.1	1044176	1045181	1044818, 1044620, 1044877	24, 34, 17	26.2838, 34.99558, 21.41937	12.90395, 14.29576, 11.99417	22.86648,	3	prmt8	68693
GL173034.1	11	512	166, 167, 166	32, 32, 17	35.18635, 33.07366, 21.41937	15.21026, 13.96479, 11.99417	31.56988,	3	prdx5	2106
GL173034.1	460617	460919	460744, 460758, 460769	20, 34, 14	21.26668, 34.08207, 14.92875	11.18692, 13.76874, 8.94211	17.9967, 3	3	fut6	6611
GL173034.1	704259	704946	704702, 704691, 704461	14, 14, 7	12.18032, 10.46897, 7.50542	7.28937, 6.23524, 5.49244	9.29157, 7	3	NA	9669
GL173034.1	1132555	1133367	1133235, 1133197, 1133157	10, 19, 8	10.01299, 19.04123, 9.60885	6.7221, 10.14611, 6.57807	7.24763, 1	3	cdca5	24995
GL173035.1	7000	8132	7996, 7125, 7109	7, 15, 7	4.55459, 10.60088, 6.21898	3.7168, 6.09017, 4.76912	2.34094, 7	3	arl14ep	5
GL173035.1	334296	334568	334521, 334519, 334509	22, 40, 10	23.53664, 43.14357, 13.18184	11.92457, 16.51657, 8.33771	20.19714,	3	dnajc24	70759
GL173035.1	743183	743574	743382, 743313, 743401	7, 9, 4	5.86458, 7.28344, 4.38152	4.55375, 5.20811, 3.65448	3.46146, 4	3	rcn1	3076
GL173035.1	811192	811526	811435, 811392, 811354	6, 11, 8	5.72604, 10.60895, 8.9249	4.51498, 6.9343, 6.24211	3.33547, 7	3	wt1	48694
GL173035.1	958916	959602	959361, 959348, 959537	7, 8, 4	6.69449, 6.51779, 4.67325	5.04797, 4.8598, 3.78987	4.1953, 4.1	3	eif3m	4963
GL173036.1	1098621	1099255	1098927, 1098996, 1098989	8, 13, 11	5.75154, 7.57282, 10.92742	4.37362, 4.69013, 7.12242	3.36039, 5	3	ica1	30847
GL173036.1	1104229	1104864	1104595, 1104686, 1104576	17, 17, 12	14.64358, 10.52471, 11.02785	8.0415, 5.61361, 7.01117	11.62752,	3	ica1	36455
GL173036.1	1105326	1106541	1105578, 1105666, 1105589	13, 24, 9	10.21914, 17.55642, 7.79368	6.30293, 7.81745, 5.52485	7.4427, 14	3	ica1	37552
GL173037.1	298085	298647	298274, 298340, 298389	10, 24, 8	10.26482, 22.72317, 8.80246	6.86644, 10.77359, 6.179	7.48624, 1	3	sema4g	0
GL173037.1	643737	644889	644133, 644458, 644254	29, 38, 16	32.03412, 39.00956, 19.86305	14.51808, 14.99063, 11.32783	28.48199,	3	trim8	47549
GL173037.1	982687	983277	982787, 982905, 982892	6, 19, 4	5.96715, 20.21388, 4.67325	4.64927, 10.84946, 3.78987	3.52158, 1	3	cnnm4	1287
GL173037.1	1080107	1080364	1080268, 1080210, 1080205	15, 18, 13	15.41679, 18.91825, 13.84064	9.0192, 10.35485, 8.49513	12.36344,	3	NA	51374
GL173037.1	1081281	1081646	1081532, 1081531, 1081531	9, 11, 7	8.35434, 10.15827, 6.63036	5.86444, 6.66366, 5.0111	5.71584, 7	3	NA	52548
GL173038.1	11986	13157	12576, 12567, 12265	6, 22, 6	5.3552, 19.25987, 5.46454	4.3003, 9.36178, 4.36458	3.00253, 1	3	erg	0
GL173038.1	15524	15838	15668, 15666, 15666	12, 11, 12	12.11599, 7.01341, 12.50469	7.66241, 4.70494, 7.88834	9.23027, 4	3	erg	0
GL173038.1	73023	73834	73082, 73773, 73118	11, 10, 6	10.60582, 8.01217, 6.76586	6.93244, 5.50894, 5.06179	7.80831, 5	3	erg	32456
GL173038.1	104055	104400	104300, 104300, 104365	9, 7, 6	8.94274, 5.33631, 5.91225	6.20889, 4.22269, 4.61907	6.2534, 3.0	3	erg	63488
GL173038.1	135803	136295	136061, 136044, 135812	8, 6, 5	8.16648, 4.92507, 5.10931	5.83735, 4.04003, 4.14026	5.54098, 2	3	erg	95236
GL173038.1	167313	167808	167782, 167781, 167601	14, 18, 7	14.38232, 18.29879, 7.96278	8.62271, 9.984, 5.72394	11.37721,	3	ets2	120047
GL173038.1	228270	228788	228691, 228498, 228493	20, 24, 15	19.99469, 24.30704, 15.48721	10.42292, 11.72023, 9.06094	16.76522,	3	ets2	59067
GL173038.1	232513	233048	232585, 232556, 232604	19, 21, 16	16.59925, 17.80261, 14.55254	8.66856, 8.80213, 8.25096	13.49961,	3	ets2	54807
GL173038.1	235571	235783	235644, 235585, 235596	14, 19, 17	12.43323, 15.04204, 16.89228	7.44494, 7.73818, 9.40884	9.52692, 1	3	ets2	52072
GL173038.1	238148	238798	238554, 238577, 238712	13, 22, 12	13.27928, 19.37031, 12.03712	8.16901, 9.42716, 7.61609	10.32936,	3	ets2	49057
GL173038.1	277483	277968	277671, 277705, 277601	5, 13, 4	4.69353, 11.36332, 4.67325	3.9134, 7.01137, 3.78987	2.45909, 8	3	ets2	9887
GL173038.1	282786	284175	283240, 283231, 282927	11, 12, 8	10.66406, 9.16947, 8.24665	6.96705, 5.86046, 5.88165	7.86396, 6	3	ets2	3680
GL173038.1	347862	348483	348225, 347920, 348111	6, 16, 6	5.96715, 17.16856, 7.29403	4.64927, 9.82359, 5.30581	3.52158, 1	3	psmg1	24981
GL173038.1	379624	380030	379863, 379858, 379712	5, 9, 5	4.82177, 8.21099, 5.9506	3.98509, 5.77858, 4.54784	2.51418, 5	3	psmg1	406
GL173038.1	469236	469983	469662, 469532, 469448	6, 23, 13	5.27951, 23.74183, 15.0131	4.25534, 11.71949, 9.14923	2.9335, 20	3	wrb	0

GL173038.1	565635	566209	565937, 565762, 565752	7, 14, 6	6.84137, 13.03306, 7.12188	5.13151, 7.81216, 5.22884	4.33107, 1	3	sh3bgr	0
GL173038.1	612281	613153	612833, 612936, 612923	5, 8, 13	4.82177, 7.07159, 15.52427	3.98509, 5.20073, 9.42125	2.51418, 4	3	NA	2212
GL173038.1	744442	744759	744504, 744480, 744510	10, 11, 6	8.61898, 8.23846, 6.45058	5.88679, 5.47271, 4.90508	5.94907, 5	3	NA	5973
GL173038.1	750289	750698	750401, 750429, 750596	6, 8, 10	4.5407, 5.40929, 11.10454	3.79858, 4.15294, 7.32977	2.33, 3.15	3	NA	11820
GL173038.1	774562	775191	774749, 774919, 774851	5, 8, 8	4.47183, 5.99047, 8.56955	3.78598, 4.52662, 6.05652	2.26935, 3	3	NA	0
GL173038.1	787574	788242	787735, 787738, 787726	17, 11, 10	17.26461, 8.93411, 9.73055	9.63292, 5.9089, 6.55755	14.13485,	3	NA	12470
GL173038.1	847766	848134	848133, 848129, 848044	16, 11, 11	13.87338, 7.49521, 11.86426	7.83559, 5.00592, 7.65794	10.89011,	3	igsf5	4125
GL173038.1	849782	850668	849829, 849981, 849859	15, 15, 8	12.8537, 11.37503, 8.04677	7.46342, 6.55803, 5.77057	9.92465, 8	3	igsf5	1591
GL173038.1	864982	865428	865273, 865257, 865227	5, 6, 8	4.82177, 4.93312, 10.1465	3.98509, 4.04501, 6.82176	2.51418, 2	3	igsf5	0
GL173038.1	889868	890423	890130, 890166, 890159	7, 9, 12	7.1696, 8.21099, 15.36829	5.31345, 5.77858, 9.40047	4.61046, 5	3	igsf5	20537
GL173038.1	953518	953854	953570, 953546, 953520	26, 63, 32	29.61147, 73.70043, 39.67838	14.18819, 22.94689, 17.86854	26.11284,	3	dscam	52941
GL173038.1	1033498	1033953	1033718, 1033718, 1033718	10, 14, 11	11.06011, 14.45897, 13.28509	7.306, 8.66788, 8.40871	8.22712, 1	3	dscam	0
GL173039.1	591992	592874	592640, 592637, 592590	12, 21, 11	12.67544, 18.88456, 12.06254	7.98627, 9.44608, 7.76744	9.75458, 1	3	ifnlr1	15492
GL173039.1	733882	734580	734100, 734159, 734119	6, 13, 4	5.96715, 13.14514, 4.67325	4.64927, 8.09002, 3.78987	3.52158, 1	3	cyp4f2	41340
GL173040.1	17016	17606	17242, 17342, 17230	8, 18, 6	6.70593, 17.06771, 6.76586	4.9768, 9.23949, 5.06179	4.20487, 1	3	foxp1	69612
GL173040.1	98308	98816	98371, 98412, 98503	8, 19, 8	7.02691, 18.41287, 9.60885	5.17362, 9.76624, 6.57807	4.50222, 1	3	foxp1	0
GL173040.1	308490	308938	308908, 308911, 308906	22, 32, 12	23.53664, 35.83564, 15.06051	11.92457, 15.59527, 9.25266	20.19714,	3	foxp1	124366
GL173040.1	412478	413139	412798, 412799, 412798	32, 53, 25	38.38874, 63.56498, 31.77551	17.10916, 22.21089, 15.77668	34.7132, 5	3	eif4e3	218366
GL173040.1	502078	502642	502245, 502256, 502321	8, 6, 5	8.42257, 4.93312, 5.69847	5.97763, 4.04501, 4.4331	5.76336, 2	3	eif4e3	128863
GL173040.1	654934	655812	655052, 655720, 655183	9, 9, 3	8.25643, 7.03406, 3.44998	5.80588, 5.05123, 3.02115	5.62516, 4	3	GPR27	4729
GL173040.1	747214	747656	747600, 747617, 747623	9, 11, 9	8.0679, 9.7598, 9.30116	5.69219, 6.42066, 6.41174	5.45, 7.16	3	rybp	0
GL173040.1	779357	779907	779881, 779868, 779889	86, 119, 52	100.81613, 137.94075, 66.92889	25.85456, 27.87144, 24.66572	96.4182, 1	3	rybp	0
GL173040.1	813947	814797	814489, 814516, 814508	11, 23, 9	11.02968, 22.81409, 10.01275	7.18222, 11.16226, 6.79683	8.20839, 1	3	rybp	31198
GL173040.1	840367	840580	840386, 840386, 840386	9, 12, 6	9.72082, 11.0952, 7.12188	6.64182, 7.05215, 5.22884	6.97155, 8	3	U4	22893
GL173040.1	909455	910724	910329, 909883, 910355	10, 17, 9	8.84451, 15.81151, 8.01994	6.02544, 8.7532, 5.66309	6.16137, 1	3	shq1	7273
GL173041.1	495259	495870	495403, 495403, 495630	5, 17, 5	4.82177, 15.99248, 4.57714	3.98509, 8.86336, 3.84705	2.51418, 1	3	nek6	87888
GL173041.1	682236	682669	682513, 682569, 682368	7, 7, 3	6.79162, 5.97725, 3.34298	5.10336, 4.62287, 2.97156	4.28406, 3	3	lhx2	10710
GL173041.1	739084	739494	739455, 739491, 739439	13, 24, 11	13.94585, 24.46445, 12.9493	8.5554, 11.8146, 8.23866	10.95967,	3	lhx2	13784
GL173041.1	756417	757166	756916, 756512, 757029	11, 11, 5	9.05039, 9.01418, 5.23257	5.98149, 5.9589, 4.20435	6.35485, 6	3	lhx2	31117
GL173041.1	1006187	1006974	1006259, 1006602, 1006245	12, 13, 5	11.92103, 11.51094, 5.91431	7.54764, 7.10228, 4.53172	9.04508, 8	3	dennd1a	18271
GL173041.1	1023968	1024391	1024098, 1024031, 1024054	6, 9, 4	5.3552, 6.49199, 3.62036	4.3003, 4.70689, 3.25193	3.00253, 4	3	dennd1a	36052
GL173042.1	62	438	423, 413, 426	20, 32, 20	26.13767, 40.45961, 27.34127	13.94781, 18.32506, 14.56493	22.72398,	3	NA	55069
GL173042.1	25279	25564	25357, 25367, 25366	4, 9, 5	3.74195, 7.79702, 5.80393	3.32091, 5.5269, 4.48186	1.6222, 5.3	3	NA	29943
GL173042.1	170724	171858	170935, 171143, 171015	6, 9, 6	5.47306, 6.6123, 6.82211	4.36955, 4.78362, 5.08889	3.10811, 4	3	NA	106054
GL173042.1	295923	296419	296031, 296258, 296278	7, 14, 7	6.25077, 9.92123, 6.59055	4.78814, 5.90089, 4.98809	3.78441, 7	3	NA	209865
GL173042.1	324903	325648	325296, 325276, 325188	11, 9, 10	9.7619, 7.21043, 9.82971	6.42195, 5.1623, 6.61563	7.0115, 4.7	3	NA	180636
GL173042.1	395860	396424	396238, 396293, 396132	11, 12, 10	10.21742, 9.35334, 9.53891	6.69945, 5.9752, 6.44439	7.44132, 6	3	NA	109860
GL173042.1	512096	512969	512431, 512815, 512819	10, 15, 8	8.79841, 13.74993, 9.46129	5.99719, 8.013, 6.50801	6.11782, 1	3	NA	1993
GL173042.1	792736	793453	793229, 793232, 793224	23, 29, 20	24.58488, 25.95243, 22.4589	12.225, 10.95717, 11.89249	21.21468,	3	NA	6119
GL173042.1	855260	855994	855827, 855822, 855367	6, 8, 4	5.96715, 6.85668, 4.67325	4.64927, 5.06969, 3.78987	3.52158, 4	3	NA	18742
GL173042.1	877445	878516	877723, 878353, 878232	8, 14, 13	8.42257, 14.45897, 18.01082	5.97763, 8.66788, 10.61163	5.76336, 1	3	NA	5659
GL173042.1	918644	920181	919128, 919364, 919337	8, 17, 6	8.42257, 16.17826, 6.99807	5.97763, 8.97632, 5.17195	5.76336, 1	3	NA	16317
GL173042.1	1039093	1039569	1039128, 1039136, 1039121	30, 33, 19	34.82499, 35.01397, 25.38515	15.79062, 14.70608, 13.7311	31.21537,	3	NA	13639
GL173043.1	191433	192535	191914, 191919, 191959	8, 13, 5	6.8227, 10.19363, 4.99237	5.04883, 6.28711, 4.0781	4.31393, 7	3	chrdl1	29937
GL173043.1	883428	884001	883536, 883711, 883814	12, 13, 4	10.20921, 8.44264, 3.39155	6.50784, 5.21062, 3.11756	7.4341, 5.9	3	lhfpl1	17168
GL173044.1	127686	128310	128190, 128219, 128238	11, 20, 13	9.95877, 18.23344, 14.5443	6.54239, 9.35906, 8.89247	7.19682, 1	3	gpr132	0
GL173044.1	879600	879829	879639, 879645, 879668	8, 16, 5	6.63031, 14.63495, 5.3628	4.92991, 8.3013, 4.27046	4.13604, 1	3	brf1	0
GL173044.1	964559	965256	964753, 964989, 964745	10, 16, 8	8.57531, 14.92393, 8.35103	5.85982, 8.47764, 5.93881	5.90712, 1	3	hspa2	11240
GL173044.1	966097	967012	966248, 966238, 966236	9, 7, 5	7.6342, 5.07986, 5.23257	5.42655, 4.05845, 4.20435	5.04509, 2	3	hspa2	12778
GL173044.1	971572	972199	971806, 972017, 971747	6, 16, 6	5.1345, 13.5443, 5.793	4.16817, 7.63435, 4.55271	2.80161, 1	3	hspa2	18253
GL173044.1	974000	974896	974060, 974070, 974632	11, 11, 7	11.71035, 8.59076, 7.29952	7.57196, 5.69393, 5.38357	8.8442, 6.0	3	hspa2	20681
GL173044.1	976138	976971	976539, 976756, 976490	6, 12, 6	5.63898, 9.95176, 6.2131	4.46544, 6.34805, 4.78171	3.25772, 7	3	hspa2	22819
GL173044.1	981881	983060	982894, 982482, 981944	11, 13, 5	12.43693, 13.14514, 5.9506	7.97018, 8.09002, 4.54784	9.52692, 1	3	hspa2	28562
GL173044.1	989897	990766	990551, 990530, 990284	12, 14, 7	13.8483, 14.45897, 8.69489	8.63436, 8.66788, 6.06379	10.86694,	3	hspa2	36578
GL173044.1	1052417	1053431	1052843, 1051634, 1052843, 1053179	11, 17, 8, 10	12.43693, 18.56115, 6.21066, 10.1418	7.97018, 10.40145, 4.66659, 6.79622	9.52692, 1	3	zbtb1	14647
GL173045.1	1065312	1066239	1065756, 1065849, 1065948	40, 51, 20	37.78072, 43.78619, 17.6399	13.53323, 12.73268, 9.00197	34.11478,	3	XB-GENE-956774	0
GL173046.1	729655	730626	729894, 730194, 730063	12, 17, 7	11.73328, 14.56175, 8.69489	7.43625, 7.99171, 6.06379	8.86615, 1	3	abhd17a	0
GL173048.1	668309	668636	668476, 668538, 668610	8, 10, 5	7.93925, 8.24345, 5.9506	5.70996, 5.65367, 4.54784	5.33002, 5	3	mad1l1	89454
GL173049.1	25921	26186	26122, 25992, 26166	5, 8, 7	4.82177, 6.51779, 8.69489	3.98509, 4.8598, 6.06379	2.51418, 4	3	itpkb	2921
GL173049.1	193337	194445	194267, 194391, 193561, 194208	8, 14, 4, 8	6.86256, 10.91946, 4.26291, 7.11599	5.0733, 6.51197, 3.59641, 5.22758	4.3506, 8.2	3	cdc42bpa	0
GL173049.1	198673	199647	198699, 198731, 198706	14, 27, 28	11.5269, 23.26933, 31.25921	6.88646, 10.10097, 14.43633	8.67131, 2	3	cdc42bpa	0
GL173049.1	201967	202579	202189, 202374, 202194	16, 20, 13	13.57573, 15.26423, 11.37851	7.65356, 7.59908, 7.02073	10.61158,	3	cdc42bpa	0
GL173049.1	216904	217396	217016, 217069, 216915	10, 5, 10	9.13239, 3.4998, 10.42037	6.2007, 3.18133, 6.95441	6.43114, 1	3	cdc42bpa	0
GL173049.1	227651	228168	227791, 227859, 228045	7, 14, 4	6.60038, 10.43306, 4.55201	4.99378, 6.21324, 3.73489	4.10761, 7	3	cdc42bpa	0
GL173049.1	253602	253959	253689, 253706, 253703	13, 17, 9	12.62236, 15.13134, 10.07825	7.77868, 8.33865, 6.83102	9.70369, 1	3	cdc42bpa	20361
GL173049.1	399761	400601	400166, 400157, 399903	8, 8, 7	7.28955, 5.72807, 7.34973	5.33177, 4.35854, 5.41038	4.72176, 3	3	ehd3	524
GL173049.1	406348	406818	406365, 406353, 406368	24, 33, 24	26.87702, 36.8901, 29.25157	13.25703, 15.8132, 14.64634	23.44461,	3	ehd3	0
GL173049.1	507044	507368	507093, 507078, 507078	7, 9, 6	7.1696, 8.21099, 6.87953	5.31345, 5.77858, 5.11627	4.61046, 5	3	CAPN14	19899
GL173049.1	542294	542669	542478, 542389, 542563	4, 7, 4	3.28906, 5.68499, 4.67325	3.0555, 4.44244, 3.78987	1.37521, 3	3	galnt14	0
GL173049.1	543438	543933	543629, 543451, 543604	4, 8, 3	3.23728, 6.34359, 3.47371	3.02372, 4.75053, 3.03189	1.33324, 3	3	galnt14	0
GL173049.1	757974	758523	758004, 758091, 758281	5, 5, 3	4.40263, 3.83284, 3.47371	3.74533, 3.39609, 3.03189	2.21004, 1	3	galnt14	0
GL173049.1	783105	783697	783304, 783359, 783387	11, 13, 6	10.1122, 12.4939, 7.29403	6.63573, 7.7014, 5.30581	7.34229, 9	3	NA	3352
GL173049.1	856089	856623	856550, 856419, 856571	8, 13, 10	6.63031, 11.07988, 10.7802	4.92991, 6.83637, 7.15425	4.13604, 8	3	capn13	0
GL173049.1	869986	870553	870522, 870516, 870523	121, 184, 93	147.40521, 227.28737, 117.52551	31.09095, 35.63526, 30.77465	142.69409	3	capn13	7901
GL173049.1	939589	940029	939609, 939593, 939632	15, 16, 7	17.48779, 17.16856, 8.69489	10.20672, 9.82359, 6.06379	14.34959,	3	lclat1	0
GL173049.1	950234	950796	950760, 950691, 950729	15, 22, 14	15.05862, 21.49375, 18.77534	8.80576, 10.69716, 10.96344	12.0251, 1	3	lclat1	0
GL173049.1	998754	999655	998900, 999327, 998864	24, 17, 7	23.82647, 16.97468, 7.2501	11.43229, 9.45853, 5.35703	20.47802,	3	lclat1	24043
GL173049.1	1013148	1013818	1013312, 1013519, 1013275	8, 8, 7	7.88469, 6.70176, 8.02489	5.67897, 4.97421, 5.75426	5.27935, 4	3	lclat1	38437
GL173049.1	1022894	1023847	1023530, 1023529, 1023530	85, 113, 55	98.08076, 130.27515, 67.28123	24.98274, 27.17695, 23.34213	93.70672,	3	lclat1	48183
GL173049.1	1034211	1034569	1034473, 1034446, 1034500	15, 25, 9	16.90423, 25.99907, 11.64359	9.88146, 12.38692, 7.57973	13.78827,	3	lclat1	59500
GL173049.1	1070976	1071300	1071111, 1071049, 1071099	7, 17, 8	6.64707, 15.5773, 6.90087	5.02073, 8.61051, 5.09678	4.15135, 1	3	lclat1	96265
GL173050.1	331614	332253	332124, 331951, 331944	7, 7, 6	5.901, 4.79556, 7.25145	4.57615, 3.87426, 5.287	3.49615, 2	3	ssu72	30999
GL173050.1	1067269	1070286	1067547, 1068940, 1068295	33, 37, 25	23.31399, 29.32006, 20.48365	8.40675, 10.19814, 9.15198	19.98066,	3	NA	21637
GL173051.1	832149	832887	832387, 832367, 832328	11, 21, 12	8.19386, 15.70697, 12.62857	5.44467, 7.58673, 7.95947	5.56766, 1	3	amy2a	419962
GL173053.1	824745	825354	825059, 825093, 824970	6, 9, 8	5.47306, 8.21099, 10.1465	4.36955, 5.77858, 6.82176	3.10811, 5	3	commd4	55844
GL173054.1	327965	328977	328261, 328763, 328270	11, 15, 7	9.39207, 10.60088, 6.59055	6.1939, 6.09017, 4.98809	6.67495, 7	3	ttc33	0
GL173054.1	357986	358450	358153, 358116, 358130	6, 8, 7	5.31708, 6.44699, 8.08824	4.2777, 4.8155, 5.7849	2.96727, 4	3	ttc33	0
GL173054.1	404954	405604	405141, 405133, 405255	10, 13, 13	10.33015, 11.41208, 13.51952	6.9035, 7.04141, 8.30949	7.54792, 8	3	ttc33	0
GL173054.1	561775	562209	561863, 561786, 561797	17, 35, 16	16.69696, 37.02376, 17.85991	9.29085, 15.07132, 10.22415	13.59308,	3	ptger4	80747
GL173054.1	651217	651661	651568, 651363, 651590	13, 9, 15	11.81837, 6.52169, 17.49481	7.29094, 4.72584, 10.21058	8.94738, 4	3	NA	133635
GL173054.1	666432	667740	666612, 666920, 667521	21, 20, 7	22.14207, 17.66446, 7.10639	11.40611, 9.01671, 5.27894	18.84387,	3	NA	117556
GL173054.1	690789	691196	690824, 690816, 690815	8, 21, 7	7.57423, 21.11333, 8.69489	5.4999, 10.78774, 6.06379	4.98865, 1	3	NA	94100
GL173054.1	772153	772726	772305, 772534, 772435	11, 14, 6	11.42621, 11.80872, 7.29403	7.41109, 7.06033, 5.30581	8.57514, 9	3	NA	12570
GL173054.1	824305	824848	824424, 824334, 824315	20, 18, 9	23.62787, 17.19487, 9.76105	12.56888, 9.31668, 6.66343	20.28502,	3	NA	33219
GL173054.1	941618	942050	942033, 942029, 942029	10, 18, 5	10.39648, 18.29879, 5.9506	6.94096, 9.984, 4.54784	7.61003, 1	3	c9	0
GL173054.1	973308	974207	973784, 973797, 973789	25, 35, 17	27.23898, 37.20959, 19.29472	13.12872, 15.1796, 10.82557	23.79707,	3	c9	20789
GL173054.1	1014868	1015746	1015487, 1015176, 1015455	9, 10, 6	8.50607, 9.39128, 6.5517	5.95453, 6.35644, 4.95623	5.84194, 6	3	c9	62349
GL173055.1	315447	315974	315944, 315916, 315526	16, 12, 6	16.91852, 10.93692, 5.8719	9.67673, 6.95571, 4.59674	13.80145,	3	brsk2	0
GL173055.1	492745	493353	493083, 493127, 493093	15, 11, 15	13.63268, 10.26356, 16.52447	7.94127, 6.72731, 9.66554	10.66672,	3	tnni2	11681
GL173055.1	582050	582925	582558, 582442, 582323	12, 7, 10	11.92103, 5.65148, 11.10454	7.54764, 4.42152, 7.32977	9.04508, 3	3	mrpl23	0
GL173056.1	69	637	428, 356, 386	32, 36, 18	24.26629, 25.24775, 12.28794	9.13021, 8.55982, 6.38986	20.90549,	3	NA	21822
GL173056.1	3148	4275	4126, 4111, 4108	30, 51, 37	22.07512, 42.20519, 34.33438	8.57686, 12.03002, 12.77972	18.77889,	3	NA	18184
GL173056.1	21666	22329	21785, 22056, 21803	9, 9, 10	6.66833, 6.31893, 10.7802	4.8193, 4.5963, 7.15425	4.17154, 3	3	NA	130
GL173056.1	457487	458173	457882, 457755, 457716	16, 19, 11	18.24544, 18.96889, 13.46208	10.44358, 10.10245, 8.49639	15.07927,	3	adcyap1	253502
GL173057.1	24617	25509	25291, 25203, 25204	22, 29, 12	26.93229, 30.27596, 15.5838	13.9091, 13.47239, 9.50165	23.49868,	3	angpt1	126476
GL173057.1	256804	257217	257067, 256842, 257114	12, 6, 12	12.74986, 4.93312, 15.16114	8.02869, 4.04501, 9.30141	9.82533, 2	3	angpt1	9949
GL173057.1	654980	655413	655142, 655128, 655080	9, 13, 6	9.00096, 11.46128, 6.6567	6.24222, 7.07172, 5.00845	6.30808, 8	3	eif3e	15498
GL173057.1	661188	661455	661315, 661315, 661316	30, 33, 17	33.70974, 33.12122, 19.63801	15.12793, 13.6033, 11.0208	30.12225,	3	eif3e	21706
GL173057.1	748022	748429	748214, 748336, 748199	8, 9, 5	8.34715, 7.79702, 5.9506	5.9367, 5.5269, 4.54784	5.71011, 5	3	emc2	11507
GL173057.1	815220	815786	815607, 815606, 815607	23, 36, 22	26.5636, 36.49457, 28.06591	13.3989, 14.36298, 14.54105	23.13966,	3	tmem74	14304
GL173057.1	856856	857647	857130, 857136, 857404	6, 13, 5	5.96715, 13.14514, 5.9506	4.64927, 8.09002, 4.54784	3.52158, 1	3	trhr	21195
GL173057.1	1020046	1021283	1020768, 1020918, 1020117, 1021174	17, 19, 8, 12	15.17095, 13.21641, 6.97106, 11.21415	8.3628, 6.68112, 5.13965, 7.12434	12.13225,	3	nudcd1	0
GL173058.1	395164	395875	395570, 395467, 395434	12, 17, 15	10.68367, 18.56115, 17.76194	6.80062, 10.40145, 10.35658	7.88119, 1	3	NA	31603
GL173059.1	246161	246581	246220, 246421, 246219	8, 10, 6	7.42892, 8.32357, 6.76586	5.41453, 5.70361, 5.06179	4.85345, 5	3	NA	21359
GL173059.1	341005	341492	341327, 341360, 341367	18, 29, 17	21.75314, 32.56557, 21.88119	11.98894, 14.83449, 12.23429	18.46685,	3	adora2b	22601
GL173059.1	695568	696126	695640, 695780, 695725	9, 7, 10	8.0222, 5.2482, 10.65691	5.66446, 4.16649, 7.08637	5.40727, 3	3	abr	0
GL173059.1	721790	722425	722122, 722119, 722198	11, 24, 7	9.71398, 21.59421, 7.61369	6.39253, 10.10576, 5.54854	6.97155, 1	3	abr	0
GL173059.1	782375	782580	782456, 782439, 782426	48, 61, 31	57.10162, 69.38355, 38.56693	20.92598, 21.5709, 17.6079	53.1505, 6	3	abr	0
GL173060.1	318700	319104	319002, 319103, 318931	10, 13, 5	10.74428, 9.96603, 5.80393	7.13454, 6.14603, 4.48186	7.93927, 7	3	ttc29	35638
GL173060.1	330622	330939	330909, 330914, 330910	13, 18, 7	12.32133, 17.06771, 7.50542	7.59717, 9.23949, 5.49244	9.42482, 1	3	ttc29	23803
GL173060.1	456619	457466	457021, 456767, 456997	8, 6, 6	8.42257, 4.93312, 7.29403	5.97763, 4.04501, 5.30581	5.76336, 2	3	ttc29	12523
GL173060.1	624151	624986	624289, 624274, 624274	14, 22, 17	14.3105, 22.00083, 19.55066	8.58029, 11.00232, 10.97134	11.30855,	3	slc10a7	0
GL173060.1	887811	888709	888021, 888068, 887955	21, 21, 14	23.41865, 19.42055, 14.92875	12.1655, 9.76764, 8.94211	20.08217,	3	znf827	0
GL173061.1	441153	442231	441309, 441493, 441268	8, 11, 7	6.90289, 7.85056, 6.36213	5.09802, 5.22892, 4.85436	4.38737, 5	3	NA	94913
GL173061.1	539839	541606	540539, 540846, 540273, 541493	21, 44, 20, 10	14.02824, 33.42838, 16.05616, 5.64879	6.65703, 10.15052, 8.06056, 4.01779	11.03913,	3	NA	1887
GL173061.1	543668	545602	544443, 543885, 545272, 545185	28, 21, 18, 14	18.9643, 9.50305, 7.81313, 8.21854	7.53918, 4.419, 4.04482, 4.88728	15.77076,	3	NA	5716
GL173061.1	547210	548671	547658, 547850, 547706	14, 14, 17	7.9791, 7.38019, 13.75562	4.74897, 4.4086, 7.50283	5.3677, 4.9	3	NA	9258
GL173061.1	1026309	1028558	1026545, 1027048, 1026779	34, 32, 21	29.63357, 27.32695, 19.91648	11.29623, 10.71879, 10.06612	26.13433,	3	ccdc127	135243
GL173061.1	1031888	1032796	1032233, 1032184, 1031931, 1032692	13, 11, 8, 6	8.07552, 5.69499, 5.37675, 4.01771	4.98934, 3.89774, 4.13188, 3.45927	5.45748, 3	3	ccdc127	140822
GL173061.1	1036783	1037624	1036999, 1037216, 1036926, 1037470	11, 13, 6, 8	9.85927, 11.81904, 5.8719, 9.11678	6.48161, 7.29135, 4.59674, 6.33924	7.10352, 9	3	ccdc127	145717
GL173061.1	1036783	1037627	1037447, 1037216, 1036926, 1037470	8, 13, 6, 8	7.06928, 11.81904, 5.8719, 9.11678	5.19933, 7.29135, 4.59674, 6.33924	4.5414, 9.1	3	ccdc127	145717
GL173062.1	79	1607	857, 440, 1039	22, 13, 19	21.33403, 11.12608, 18.90983	10.60109, 6.86492, 10.06679	18.06131,	3	sycp3	133199
GL173062.1	3276	4275	3547, 3365, 3736	11, 8, 17	6.92002, 4.46521, 14.82751	4.64687, 3.54053, 8.1535	4.40423, 2	3	sycp3	130531
GL173062.1	7096	7764	7304, 7542, 7543	15, 17, 10	14.27412, 15.40668, 9.26679	8.3325, 8.50651, 6.28179	11.27364,	3	sycp3	127042
GL173062.1	40405	41112	41077, 41069, 41087	29, 51, 39	34.49933, 63.50921, 53.53982	15.98132, 23.17481, 23.03662	30.89597,	3	sycp3	93694
GL173062.1	179027	179339	179305, 179213, 179156	11, 10, 4	11.49566, 8.98028, 3.82084	7.45067, 6.10835, 3.36473	8.64106, 6	3	sycp3	37010
GL173062.1	183788	184155	184124, 184101, 183995	11, 15, 11	12.17114, 15.26186, 13.46208	7.82679, 8.92711, 8.49639	9.28263, 1	3	sycp3	41771
GL173062.1	439991	440881	440393, 440484, 440212	13, 17, 13	11.45737, 17.33491, 15.17843	7.06931, 9.67509, 9.23814	8.60415, 1	3	NA	118327
GL173062.1	473245	473660	473460, 473470, 473414	12, 8, 8	12.52976, 6.08299, 8.4044	7.90277, 4.58557, 5.9678	9.6155, 3.7	3	NA	85548
GL173062.1	480683	481455	481413, 481315, 481367	13, 18, 15	13.20994, 19.97713, 16.52447	8.12823, 10.97931, 9.66554	10.26285,	3	NA	77753
GL173062.1	505673	506081	506031, 506027, 506055	65, 113, 75	72.38902, 135.56902, 97.55038	21.30146, 29.51472, 29.84642	68.26989,	3	NA	53127
GL173062.1	529536	529811	529672, 529746, 529781	6, 15, 7	5.72604, 13.58442, 6.6707	4.51498, 7.91173, 5.03432	3.33547, 1	3	NA	29397
GL173062.1	601225	601805	601277, 601516, 601295	11, 31, 14	12.25244, 33.4732, 16.29325	7.87094, 14.59388, 9.7093	9.35958, 3	3	NA	22636
GL173062.1	692358	692825	692725, 692757, 692554	19, 16, 8	23.93339, 17.16856, 10.1465	12.9719, 9.82359, 6.82176	20.58207,	3	NA	67151
GL173062.1	756377	756858	756661, 756757, 756751	13, 36, 16	12.03613, 35.84309, 19.066	7.42394, 13.99278, 10.89996	9.15434, 3	3	NA	131170
GL173062.1	760154	760499	760312, 760277, 760277	7, 15, 6	5.93788, 11.3375, 5.36363	4.59878, 6.53521, 4.30528	3.52158, 8	3	NA	134947
GL173062.1	792928	793272	793233, 793230, 793229	10, 13, 8	9.83337, 12.4939, 9.31951	6.61776, 7.7014, 6.43944	7.07841, 9	3	lrrc4c	138094
GL173062.1	960953	961240	961014, 961018, 961136	8, 15, 8	8.42257, 14.91533, 10.1465	5.97763, 8.71987, 6.82176	5.76336, 1	3	lrrc4c	27539
GL173062.1	1016317	1016893	1016638, 1016666, 1016642	13, 21, 14	14.61367, 24.35402, 18.05504	8.93088, 12.71289, 10.62048	11.59864,	3	lrrc4c	82903
GL173063.1	34908	35177	35073, 34952, 35068	7, 14, 6	5.901, 12.28739, 7.25145	4.57615, 7.35528, 5.287	3.49615, 9	3	xtr-mir-9a-1	22788
GL173063.1	116659	117051	117021, 116962, 117007	22, 25, 23	23.18766, 26.34052, 29.05751	11.71562, 12.59134, 14.82811	19.85731,	3	mef2d	95382
GL173063.1	194444	195117	195085, 194450, 194483	10, 17, 10	8.44704, 14.66185, 10.7802	5.78037, 8.05262, 7.15425	5.7869, 11	3	mef2d	17316
GL173063.1	243029	243915	243862, 243889, 243852	26, 45, 19	28.48278, 46.25035, 21.10095	13.51628, 16.14781, 11.37361	25.01069,	3	mef2d	0
GL173063.1	280189	280757	280719, 280717, 280679	28, 32, 17	31.18742, 33.59991, 22.24742	14.39356, 14.27354, 12.42079	27.6528, 3	3	mef2d	32056
GL173063.1	304251	304891	304436, 304760, 304458	12, 19, 10	10.3005, 15.62573, 8.77043	6.56436, 8.08461, 5.98002	7.51999, 1	3	mef2d	56118
GL173063.1	433251	434160	433288, 433783, 433926	11, 9, 3	10.00935, 6.46255, 3.47371	6.57321, 4.68809, 3.03189	7.24529, 4	3	gpatch4	12683
GL173063.1	537284	537792	537548, 537547, 537527	31, 71, 21	26.21798, 66.6123, 19.37125	10.44393, 16.20968, 9.73802	22.80168,	3	ca14	19877
GL173063.1	554305	554827	554645, 554700, 554723	20, 17, 10	21.61731, 15.40668, 10.53703	11.39575, 8.50651, 7.01977	18.33537,	3	ca14	2842
GL173063.1	623808	624023	623985, 624000, 623992	15, 14, 14	16.9979, 14.45897, 16.2088	9.93422, 8.66788, 9.66324	13.87839,	3	KIRREL1	0
GL173063.1	710627	711519	710830, 710813, 710822	20, 18, 9	20.06876, 17.72647, 8.43009	10.4676, 9.63881, 5.90952	16.8357, 1	3	KIRREL1	62402
GL173063.1	745813	746372	746312, 746321, 746346	19, 25, 15	19.8867, 25.58747, 19.40672	10.65421, 12.14055, 11.21161	16.66031,	3	KIRREL1	97588
GL173063.1	761303	761929	761789, 761718, 761744	17, 25, 10	16.97511, 25.19159, 10.3632	9.45879, 11.90378, 6.92219	13.85654,	3	KIRREL1	113078
GL173063.1	778391	778810	778425, 778594, 778617	7, 7, 10	7.1696, 5.97725, 13.18184	5.31345, 4.62287, 8.33771	4.61046, 3	3	KIRREL1	130166
GL173063.1	830401	831303	830509, 830783, 830599	4, 7, 6	3.74195, 4.62896, 7.18591	3.32091, 3.76551, 5.25776	1.6222, 2.4	3	5S_rRNA	121367
GL173063.1	839391	840835	840504, 840504, 839520, 840503	21, 39, 5, 15	21.567, 37.41993, 3.9505, 15.61614	11.06089, 13.71348, 3.47021, 9.13715	18.2862, 3	3	5S_rRNA	111835
GL173063.1	922408	922913	922572, 922802, 922752	6, 13, 4	5.96715, 13.14514, 4.67325	4.64927, 8.09002, 3.78987	3.52158, 1	3	5S_rRNA	29757
GL173063.1	933386	933904	933403, 933417, 933409	32, 56, 35	36.46676, 64.26354, 46.66623	15.96988, 21.14763, 20.73295	32.8252, 6	3	5S_rRNA	18766
GL173063.1	989206	989668	989414, 989535, 989425	9, 8, 6	8.88537, 7.07159, 6.71074	6.17591, 5.20073, 5.03498	6.19961, 4	3	NA	1121
GL173063.1	1036387	1036749	1036648, 1036517, 1036712	5, 9, 5	4.82177, 8.21099, 5.3628	3.98509, 5.77858, 4.27046	2.51418, 5	3	NA	38351
GL173064.1	730604	731536	731172, 731102, 731276	9, 18, 10	7.97705, 17.19487, 10.71812	5.637, 9.31668, 7.12015	5.36577, 1	3	extl3	13712
GL173064.1	778902	779317	779295, 779226, 779279	9, 10, 7	8.35434, 8.9331, 7.66926	5.86444, 6.07959, 5.57703	5.71584, 6	3	extl3	0
GL173064.1	820987	821628	821159, 821115, 821168	11, 8, 9	10.21742, 7.07159, 10.01275	6.69945, 5.20073, 6.79683	7.44132, 4	3	extl3	19633
GL173064.1	823474	824726	823943, 823983, 824235	15, 11, 6	15.12865, 9.8562, 6.08001	8.84764, 6.47973, 4.71063	12.09208,	3	extl3	22120
GL173064.1	929475	930461	930417, 930206, 929894	43, 50, 17	47.04217, 53.07457, 19.37904	17.44519, 17.74381, 10.87373	43.23296,	3	NA	0
GL173064.1	965675	966066	965707, 965702, 965697	48, 61, 48	58.19263, 70.10627, 59.79596	21.55943, 21.96147, 22.49578	54.22716,	3	kif13b	0
GL173064.1	999057	999658	999615, 999631, 999616	24, 26, 16	28.17887, 28.01936, 18.3512	14.02452, 13.2395, 10.50324	24.71516,	3	kif13b	20713
GL173065.1	132	1290	855, 588, 404	22, 44, 27	8.50909, 24.45787, 19.47087	3.87443, 6.70849, 8.04239	5.84494, 2	3	sox13	13611
GL173065.1	132	1303	1233, 588, 404	17, 44, 27	5.18144, 24.45787, 19.47087	2.96347, 6.70849, 8.04239	2.84456, 2	3	sox13	13598
GL173065.1	4365	5855	5343, 4965, 5342	108, 100, 76	100.7765, 75.86361, 72.38857	17.71969, 12.1547, 16.95318	96.37927,	3	sox13	9046
GL173066.1	66478	66768	66620, 66611, 66665	8, 7, 10	7.1122, 5.33631, 11.10454	5.22529, 4.22269, 7.32977	4.57946, 3	3	anos1	0
GL173066.1	172050	172960	172163, 172086, 172095	18, 37, 12	19.8791, 41.00088, 14.06375	10.9221, 16.55823, 8.74831	16.65356,	3	anos1	14522
GL173068.1	26123	27586	26348, 26517, 27253	5, 8, 5	4.81325, 6.02101, 5.91431	3.98038, 4.5461, 4.53172	2.51418, 3	3	NA	0
GL173068.1	130693	131504	130950, 130982, 130929	26, 26, 18	23.81005, 21.06429, 19.67904	10.74163, 9.17619, 10.80493	20.46254,	3	sft2d1	48565
GL173068.1	133995	134547	134366, 134400, 134400	22, 34, 14	17.89775, 29.42076, 13.70709	8.56425, 11.18275, 8.22039	14.74507,	3	sft2d1	45522
GL173068.1	356991	357389	357180, 357171, 357018	11, 25, 5	9.43668, 24.01826, 4.17451	6.22151, 11.20454, 3.60852	6.7178, 20	3	t2	19699
GL173069.1	21921	22519	22322, 22153, 22181	31, 44, 26	32.69429, 47.55277, 30.54062	14.1331, 17.29688, 14.73724	29.12759,	3	CSMD3	319257
GL173069.1	89580	89915	89663, 89778, 89780	9, 12, 6	8.88537, 11.48527, 6.5517	6.17591, 7.28792, 4.95623	6.19961, 8	3	CSMD3	251861
GL173069.1	121291	121956	121319, 121379, 121296	17, 21, 9	17.26461, 21.03886, 11.04428	9.63292, 10.74286, 7.30896	14.13485,	3	CSMD3	219820
GL173069.1	178223	178614	178530, 178524, 178406	8, 13, 5	7.88469, 10.8548, 5.9506	5.67897, 6.69706, 4.54784	5.27935, 8	3	CSMD3	163162
GL173069.1	257676	258144	257923, 258106, 258068	5, 10, 4	4.20732, 8.88645, 4.64325	3.62846, 6.0511, 3.77643	2.04024, 6	3	CSMD3	83632
GL173069.1	276021	276297	276057, 276086, 276048	5, 12, 8	4.82177, 10.49031, 9.92329	3.98509, 6.6816, 6.7228	2.51418, 7	3	CSMD3	65479
GL173069.1	543431	543674	543465, 543572, 543446	18, 14, 10	18.68415, 13.0902, 12.42918	10.2151, 7.84698, 7.99682	15.49996,	3	CSMD3	0
GL173069.1	588023	588765	588545, 588625, 588410	9, 11, 4	9.70623, 8.74006, 4.67325	6.63396, 5.78751, 3.78987	6.97147, 6	3	CSMD3	0
GL173069.1	645799	646223	646109, 645965, 645980	10, 14, 5	10.01299, 13.63145, 5.75061	6.7221, 8.17488, 4.45735	7.24763, 1	3	CSMD3	42233
GL173069.1	750692	751753	751345, 751194, 751335	12, 15, 12	11.26426, 13.11311, 13.59027	7.15468, 7.62272, 8.49571	8.4212, 10	3	CSMD3	147126
GL173069.1	808003	808799	808228, 808318, 808266	7, 14, 10	7.1696, 13.14791, 11.6791	5.31345, 7.88211, 7.62925	4.61046, 1	3	kcnv1	155792
GL173069.1	834670	834972	834820, 834863, 834872	6, 9, 4	4.99728, 8.21099, 4.30163	4.08451, 5.77858, 3.61556	2.67584, 5	3	kcnv1	129619
GL173069.1	874655	875372	874987, 874868, 874932	10, 17, 10	9.89241, 18.11497, 11.997	6.65218, 10.13939, 7.78835	7.13431, 1	3	kcnv1	89219
GL173069.1	906671	907593	906717, 906742, 906698	16, 14, 16	17.08121, 12.28739, 18.3512	9.7724, 7.35528, 10.50324	13.95894,	3	kcnv1	56998
GL173070.1	52	1601	370, 372, 470	62, 71, 33	65.43662, 74.94239, 37.46766	19.06331, 19.96174, 16.15548	61.38792,	3	NA	109917
GL173070.1	137497	138430	138037, 138046, 137880	10, 15, 9	7.86015, 10.7341, 7.86759	5.41341, 6.17021, 5.57018	5.25709, 8	3	nhsl1	10175
GL173070.1	513545	514922	514318, 513881, 514309	16, 20, 13	14.18542, 14.83072, 12.70856	8.02648, 7.34984, 7.83036	11.18941,	3	reps1	0
GL173070.1	923002	923645	923427, 923429, 923588	18, 22, 18	18.07742, 22.00083, 20.15609	9.85073, 11.00232, 11.0834	14.9177, 1	3	NA	203682
GL173071.1	75604	76060	76020, 76003, 76001	14, 29, 14	11.98579, 24.42946, 14.86131	7.16952, 10.10797, 8.90303	9.10677, 2	3	NA	120621
GL173071.1	198606	198951	198775, 198674, 198664	9, 12, 5	8.0222, 10.83413, 5.27517	5.66446, 6.89288, 4.22616	5.40727, 8	3	NA	0
GL173071.1	223139	223516	223240, 223296, 223249	10, 11, 14	9.54985, 8.30667, 16.46576	6.45088, 5.51556, 9.80275	6.82341, 5	3	NA	9430
GL173071.1	278343	279145	278519, 278793, 278723	7, 8, 7	6.64707, 6.89656, 7.05993	5.02073, 5.09414, 5.25341	4.15135, 4	3	NA	14125
GL173071.1	472544	472854	472609, 472659, 472751	7, 13, 5	6.16939, 11.61168, 4.91762	4.73936, 7.16421, 4.03768	3.70916, 8	3	hdgf	1524
GL173071.1	546919	547572	547394, 547432, 547460	5, 13, 11	4.81325, 12.09006, 10.5698	3.98038, 7.45678, 6.91098	2.51418, 9	3	NA	31998
GL173071.1	688080	689509	688823, 688896, 688809	12, 16, 5	11.73328, 13.35594, 5.14967	7.43625, 7.51928, 4.16141	8.86615, 1	3	ntrk1	30960
GL173071.1	845981	847140	846242, 846594, 846723	7, 12, 13	6.2098, 10.25865, 13.77491	4.76362, 6.53846, 8.45734	3.74679, 7	3	pear1	3493
GL173071.1	926997	927642	927179, 927434, 927241	8, 9, 4	8.42257, 8.01512, 3.88239	5.97763, 5.66016, 3.39841	5.76336, 5	3	NA	35089
GL173071.1	936944	937723	937190, 937330, 937423	10, 17, 7	7.93364, 12.41151, 5.76629	5.45963, 6.69937, 4.493	5.325, 9.67	3	NA	25008
GL173072.1	71662	72716	72158, 72242, 72189	15, 24, 8	17.6946, 25.37763, 10.1465	10.31995, 12.36208, 6.82176	14.54864,	3	NA	NA
GL173072.1	135803	136923	136495, 136209, 136017	11, 13, 8	10.16451, 12.03475, 9.76268	6.66744, 7.4231, 6.64964	7.39114, 9	3	NA	NA
GL173072.1	729874	730213	729946, 730057, 729953	7, 9, 7	6.94335, 7.63127, 8.35502	5.18876, 5.42473, 5.9108	4.42528, 5	3	NA	NA
GL173073.1	363787	364316	364121, 364100, 364252	9, 14, 6	7.97705, 12.23767, 7.25145	5.637, 7.32468, 5.287	5.36577, 9	3	vim	0
GL173073.1	472579	472987	472754, 472790, 472864	16, 26, 19	15.60786, 24.56489, 22.14038	8.89312, 11.18383, 11.97538	12.5472, 2	3	cubn	0
GL173073.1	757888	758615	758583, 758573, 758567	19, 29, 13	18.53897, 31.08464, 14.93222	9.84257, 13.95282, 9.10541	15.36013,	3	U4	35024
GL173074.1	1045	1370	1144, 1085, 1061	9, 6, 8	9.72082, 4.93312, 10.1465	6.64182, 4.04501, 6.82176	6.97155, 2	3	arl4a	139875
GL173074.1	102126	102536	102289, 102359, 102213	13, 10, 8	13.07382, 9.17452, 9.11678	8.04786, 6.22617, 6.33924	10.13353,	3	arl4a	38709
GL173074.1	190632	190932	190842, 190868, 190864	16, 19, 12	16.45267, 15.21683, 12.50469	9.40062, 7.84157, 7.88834	13.35812,	3	scin	0

GL173074.1	279668	280100	279941, 279910, 279855	9, 9, 8	8.11416, 6.89892, 8.29845	5.72019, 4.96575, 5.91009	5.49285, 4	3	NA	15574
GL173074.1	291252	292175	291281, 291270, 291290	124, 146, 104	144.21822, 166.05476, 128.86131	28.37319, 28.13094, 30.74417	139.52409	3	NA	27158
GL173075.1	921872	922319	921996, 921943, 921893	17, 13, 18	17.11839, 12.31712, 22.99911	9.54506, 7.59462, 12.66051	13.99434,	3	itga8	8970
GL173076.1	9	762	326, 323, 317	14, 23, 6	15.49091, 25.81799, 7.29403	9.26387, 12.95947, 5.30581	12.43497,	3	atp1b2	59747
GL173076.1	38159	39303	38840, 38563, 39010	11, 13, 7	10.66406, 11.61168, 7.61369	6.96705, 7.16421, 5.54854	7.86396, 8	3	atp1b2	21206
GL173076.1	39805	40791	39939, 40484, 40625	6, 11, 4	4.9641, 9.09572, 3.97874	4.06411, 6.00975, 3.45022	2.64509, 6	3	atp1b2	19718
GL173076.1	55472	56534	55976, 55870, 56172	5, 7, 5	4.27048, 5.07986, 3.97425	3.6666, 4.05845, 3.48505	2.09564, 2	3	atp1b2	3975
GL173076.1	75323	75795	75617, 75634, 75676	11, 12, 10	8.84943, 9.66318, 9.49232	5.85597, 6.16843, 6.41671	6.16564, 7	3	tp53	0
GL173076.1	79540	80261	80104, 80105, 80155	20, 16, 16	19.56371, 15.41438, 17.2553	10.16265, 8.77591, 9.87431	16.34986,	3	tp53	0
GL173076.1	192003	192255	192040, 192043, 192174	5, 10, 6	4.20732, 8.12616, 7.12188	3.62846, 5.58036, 5.22884	2.04024, 5	3	krt15	19103
GL173076.1	213786	214338	214260, 214245, 214219	32, 39, 15	39.04999, 44.64822, 18.13606	17.49928, 17.82678, 10.55788	35.36284,	3	krt15	0
GL173076.1	302975	303266	303211, 303204, 303202	23, 54, 20	24.76553, 59.64441, 24.77855	12.33305, 19.54091, 13.21509	21.39099,	3	krt17	22015
GL173076.1	350819	351412	350859, 350853, 350847	91, 160, 87	113.06406, 199.77663, 114.21288	29.52902, 35.36487, 32.31615	108.56856	3	terf2ip	7844
GL173076.1	386686	387350	386987, 387215, 387338	8, 19, 7	5.83807, 13.70317, 6.75301	4.42916, 6.95836, 5.08141	3.43803, 1	3	krt	7817
GL173076.1	412505	412789	412764, 412742, 412758	48, 39, 36	56.44855, 41.41521, 45.50011	20.54864, 15.94952, 19.64283	52.5065, 3	3	gast	671
GL173076.1	456864	457683	457245, 457312, 457280	6, 26, 8	5.81608, 30.7371, 9.46129	4.56563, 14.8527, 6.50801	3.4176, 27	3	hap1	0
GL173076.1	473507	474056	473600, 473528, 473653	12, 18, 8	12.97977, 19.97713, 10.1465	8.15868, 10.97931, 6.82176	10.04411,	3	hap1	0
GL173076.1	494695	495263	495179, 495164, 495185	21, 31, 18	23.03475, 32.63958, 21.11592	11.93841, 14.10081, 11.63307	19.70924,	3	hap1	19816
GL173076.1	507267	507765	507268, 507385, 507509	8, 7, 5	8.42257, 5.82304, 5.45388	5.97763, 4.52813, 4.3157	5.76336, 3	3	jup	11001
GL173076.1	575775	576168	576005, 576138, 576117	7, 15, 9	6.89193, 14.52325, 10.56949	5.15998, 8.48354, 7.08032	4.37772, 1	3	jup	3218
GL173076.1	591468	591841	591499, 591614, 591580	15, 18, 11	16.63109, 19.97713, 13.93476	9.72648, 10.97931, 8.7238	13.52931,	3	p3h4	7578
GL173076.1	641672	642097	642067, 642093, 641987	13, 23, 18	11.45737, 23.18973, 19.75651	7.06931, 11.38786, 10.85036	8.60415, 2	3	nt5c3b	3916
GL173076.1	689343	690306	689381, 689389, 689346	28, 23, 12	33.20707, 24.15665, 14.49372	15.59147, 11.96843, 8.97057	29.62933,	3	acly	0
GL173076.1	856375	856734	856542, 856542, 856614	7, 9, 3	6.42049, 8.21099, 3.47371	4.8888, 5.77858, 3.03189	3.94161, 5	3	znf385c	0
GL173076.1	973113	973591	973147, 973175, 973138	13, 29, 7	13.94585, 29.43697, 8.646	8.5554, 12.97595, 6.04229	10.95967,	3	dhx58	1276
GL173076.1	1007922	1008322	1008288, 1008266, 1008278	14, 13, 8	14.3105, 12.80191, 9.05167	8.58029, 7.88619, 6.30653	11.30855,	3	kat2a	1229
GL173076.1	1021649	1022144	1021809, 1021811, 1021808	5, 9, 4	4.47183, 8.21099, 4.30163	3.78598, 5.77858, 3.61556	2.26935, 5	3	rab5c	0
GL173077.1	336886	337507	337143, 337132, 337112	16, 26, 16	18.5447, 28.58933, 21.00727	10.61177, 13.57987, 11.91233	15.36536,	3	lyar	12208
GL173077.1	342446	343007	342758, 342824, 342730	8, 7, 5	8.34715, 5.97725, 5.9506	5.9367, 4.62287, 4.54784	5.71011, 3	3	lyar	6708
GL173077.1	547833	548784	548669, 548715, 548667	63, 109, 43	70.64163, 120.52125, 53.24361	21.30648, 24.71738, 21.05443	66.53883,	3	stx18	36137
GL173077.1	888517	889570	889074, 889340, 888790	7, 13, 5	6.42049, 10.04078, 4.77515	4.8888, 6.19235, 3.9592	3.94161, 7	3	EVC2	0
GL173078.1	244	2239	1109, 1088, 1439	16, 24, 9	13.57573, 18.2778, 7.31381	7.65356, 8.21118, 5.22713	10.61158,	3	rgs1	202896
GL173078.1	4362	5588	4368, 4791, 4517	9, 20, 10	3.53038, 9.5592, 5.58008	2.84109, 4.58706, 3.97502	1.56731, 6	3	rgs1	199547
GL173078.1	7030	9462	8318, 7920, 7661	20, 30, 20	14.98529, 23.21304, 17.9783	7.43841, 9.15697, 9.20537	11.9549, 2	3	rgs1	195673
GL173078.1	14669	17786	16969, 15300, 16986, 15791, 17148	17, 18, 25, 12, 13	12.56383, 12.08688, 19.29692, 9.21464, 10.2815	6.78942, 6.27562, 8.49211, 5.88864, 6.34161	9.64791, 9	3	rgs1	187349
GL173078.1	26757	27776	27597, 27390, 27431	11, 16, 9	10.21742, 14.69174, 9.64095	6.69945, 8.33598, 6.59867	7.44132, 1	3	rgs1	177359
GL173078.1	30943	31955	31076, 31197, 31480	11, 16, 8	10.21742, 14.69174, 8.35103	6.69945, 8.33598, 5.93881	7.44132, 1	3	rgs1	173180
GL173078.1	38851	39429	39236, 39306, 38919	14, 24, 13	13.14827, 23.70127, 14.5443	7.88233, 11.35734, 8.89247	10.20482,	3	rgs1	165706
GL173078.1	43392	45292	44047, 44366, 43485, 44639	18, 22, 10, 14	15.41313, 18.22573, 7.50676, 13.93205	8.23514, 8.75462, 5.19045, 8.35526	12.36037,	3	rgs1	159843
GL173078.1	150539	150912	150557, 150566, 150564	23, 24, 22	25.82433, 25.55301, 27.58478	12.96322, 12.46712, 14.27522	22.41977,	3	rgs1	54223
GL173078.1	302505	303297	302892, 303234, 302636	13, 8, 4	13.49269, 6.85668, 4.38152	8.29387, 5.06969, 3.65448	10.53271,	3	rgs2	24244
GL173078.1	447287	448016	447584, 447648, 447453	5, 15, 5	4.36883, 13.53022, 5.9506	3.72533, 7.87854, 4.54784	2.18201, 1	3	uchl5	14469
GL173078.1	994495	995501	995166, 995207, 994676	6, 7, 7	5.96715, 5.97725, 6.92462	4.64927, 4.62287, 5.17829	3.52158, 3	3	NA	1101
GL173078.1	1040470	1040983	1040681, 1040669, 1040745	15, 17, 8	16.45523, 18.56115, 9.68495	9.62583, 10.40145, 6.61366	13.36057,	3	NA	1480
GL173079.1	705949	706373	706057, 706058, 706178	10, 9, 6	9.54985, 8.21099, 7.29403	6.45088, 5.77858, 5.30581	6.82341, 5	3	psip1	443105
GL173079.1	809154	809782	809367, 809369, 809371	37, 49, 24	39.25707, 52.02346, 27.91262	15.54328, 17.60461, 13.86856	35.5666, 4	3	psip1	546310
GL173080.1	16968	17316	16978, 16997, 16996	7, 12, 6	6.94335, 11.48527, 7.29403	5.18876, 7.28792, 5.30581	4.42528, 8	3	cyp26a1	40596
GL173080.1	91767	92050	91770, 91784, 91790	19, 28, 9	20.21999, 27.69258, 10.87975	10.8531, 12.31649, 7.23113	16.98276,	3	exoc6	2908
GL173080.1	217567	219038	218272, 218419, 218277	9, 9, 8	7.80169, 7.35794, 8.4586	5.52977, 5.25474, 5.99709	5.20141, 4	3	exoc6	387
GL173080.1	350115	350939	350255, 350485, 350782	9, 6, 4	8.25643, 4.11123, 4.46488	5.80588, 3.52071, 3.69425	5.62516, 2	3	hhex	744
GL173080.1	599791	601046	599841, 600320, 600865	7, 9, 11	6.94335, 7.79702, 13.28509	5.18876, 5.5269, 8.40871	4.42528, 5	3	btaf1	3954
GL173080.1	735298	735677	735421, 735378, 735415	6, 10, 6	5.43318, 7.23223, 5.91225	4.34622, 5.01665, 4.61907	3.07284, 4	3	ccnj	299
GL173081.1	77400	78376	77545, 77544, 77515	11, 11, 10	12.43693, 10.60895, 12.80767	7.97018, 6.9343, 8.1718	9.52692, 7	3	stim2	445356
GL173081.1	293879	295131	294551, 294847, 294723	16, 11, 19	17.16405, 10.26356, 23.12109	9.82095, 6.72731, 12.52775	14.0384, 7	3	stim2	228601
GL173081.1	573561	574012	573870, 573896, 573785	5, 17, 6	4.47183, 13.9447, 5.91225	3.78598, 7.61716, 4.61907	2.26935, 1	3	stim2	7783
GL173081.1	789563	790049	789929, 789975, 789975	9, 21, 14	8.61068, 19.73353, 15.80333	6.01615, 9.95593, 9.43935	5.94135, 1	3	sel1l3	49172
GL173081.1	1036479	1038182	1037797, 1037936, 1036829, 1037840	19, 34, 24, 14	9.68339, 20.10788, 19.01504, 8.07905	4.80219, 6.78635, 8.62121, 4.80656	6.95005, 1	3	pi4k2b	44950
GL173081.1	1039062	1039750	1039398, 1039331, 1039402	29, 36, 18	16.8826, 19.99465, 10.3741	6.36245, 6.39272, 5.33325	13.76772,	3	pi4k2b	47533
GL173081.1	1043522	1045139	1044493, 1044012, 1044445	15, 18, 8	7.96481, 8.1824, 4.21411	4.56807, 4.22089, 3.37814	5.35503, 5	3	pi4k2b	51993
GL173081.1	1045812	1046266	1046195, 1045897, 1046031	20, 12, 6	13.61651, 5.52025, 3.1682	6.66671, 3.65601, 2.89114	10.65152,	3	pi4k2b	54283
GL173082.1	16010	16364	16336, 16258, 16275	20, 19, 13	23.40409, 17.76909, 16.93213	12.44081, 9.37618, 10.12358	20.06837,	3	arpc2	0
GL173082.1	80211	80430	80253, 80219, 80219	41, 36, 36	49.18219, 38.93244, 49.17624	19.59148, 15.77121, 21.7705	45.34109,	3	arpc2	55928
GL173082.1	87293	87716	87675, 87688, 87700	17, 32, 11	17.72219, 36.1569, 13.64572	9.90648, 15.78594, 8.58592	14.57533,	3	tns1	49572
GL173082.1	99803	100250	99945, 99981, 99882	11, 12, 5	11.85906, 10.88526, 5.14967	7.65504, 6.92415, 4.16141	8.98595, 8	3	tns1	37038
GL173082.1	123662	124384	124219, 124208, 124217	12, 10, 15	13.05874, 9.39128, 17.76194	8.20296, 6.35644, 10.35658	10.11961,	3	tns1	12904
GL173082.1	184625	186002	185352, 185346, 185138	15, 23, 4	11.82584, 17.39075, 3.70359	6.83297, 7.99459, 3.29934	8.95463, 1	3	tns1	0
GL173082.1	189698	190199	190101, 190099, 190149	18, 36, 14	18.29925, 34.917, 16.29325	9.98428, 13.47192, 9.7093	15.12881,	3	tns1	0
GL173082.1	220968	221597	221135, 221020, 221012	8, 8, 10	6.01859, 5.53815, 10.47831	4.54456, 4.2362, 6.98694	3.56839, 3	3	tns1	0
GL173082.1	292155	292603	292449, 292347, 292242	7, 15, 3	7.1696, 13.92002, 3.47371	5.31345, 8.11689, 3.03189	4.61046, 1	3	tns1	9963
GL173082.1	293892	294387	294140, 293983, 294029	5, 9, 5	4.82177, 7.67202, 5.9506	3.98509, 5.44992, 4.54784	2.51418, 5	3	tns1	11700
GL173082.1	304373	304979	304630, 304893, 304429	8, 13, 6	8.42257, 13.14514, 7.29403	5.97763, 8.09002, 5.30581	5.76336, 1	3	tns1	22181
GL173082.1	312234	312670	312639, 312632, 312642	13, 14, 12	14.61367, 14.45897, 15.5838	8.93088, 8.66788, 9.50165	11.59864,	3	tns1	30042
GL173082.1	372098	372797	372476, 372256, 372247	5, 7, 17	4.82177, 5.97725, 22.3736	3.98509, 4.62287, 12.48422	2.51418, 3	3	NA	68758
GL173082.1	425818	426106	426065, 426071, 426062	9, 9, 5	9.36986, 7.79702, 5.50079	6.44997, 5.5269, 4.33868	6.65425, 5	3	NA	15449
GL173082.1	491687	492268	492044, 491703, 491927	5, 15, 6	4.50727, 12.01071, 5.46454	3.80664, 6.9461, 4.36458	2.30125, 9	3	NA	45161
GL173082.1	492629	493206	492825, 492729, 493042	6, 13, 6	5.5965, 9.96603, 5.60548	4.44108, 6.14603, 4.44624	3.21976, 7	3	NA	46103
GL173082.1	532377	532975	532436, 532728, 532571	6, 9, 5	5.24248, 6.7053, 5.59755	4.2332, 4.84282, 4.38538	2.89962, 4	3	NA	85851
GL173082.1	612449	612925	612474, 612544, 612505	12, 8, 15	10.48835, 6.41216, 18.13606	6.6804, 4.79364, 10.55788	7.69782, 4	3	NA	165923
GL173082.1	625865	626324	626137, 626295, 626139	8, 14, 10	5.89711, 8.89018, 9.01123	4.46697, 5.28138, 6.12719	3.49291, 6	3	NA	179339
GL173082.1	735863	736258	736092, 736092, 736196	8, 13, 4	7.15567, 11.12608, 4.50795	5.25151, 6.86492, 3.71446	4.61046, 8	3	igfbp5	196798
GL173082.1	782007	782315	782227, 782235, 782109	7, 15, 6	6.42049, 13.92002, 6.99807	4.8888, 8.11689, 5.17195	3.94161, 1	3	igfbp5	150741
GL173082.1	791541	791787	791557, 791567, 791591	20, 26, 9	17.97756, 26.63135, 8.34444	9.20492, 12.41002, 5.85853	14.82106,	3	igfbp5	141269
GL173082.1	813239	813801	813284, 813266, 813265	22, 39, 12	24.37361, 42.89945, 13.15613	12.42309, 16.80585, 8.25729	21.00961,	3	igfbp5	119255
GL173082.1	837923	838542	837925, 838099, 837981	19, 28, 10	18.89089, 25.12925, 10.3632	10.05535, 10.82777, 6.92219	15.69992,	3	igfbp5	94514
GL173082.1	861493	861892	861781, 861846, 861843	15, 19, 13	13.46874, 17.40691, 13.97446	7.84087, 9.15675, 8.57174	10.51051,	3	igfbp5	71164
GL173082.1	872686	873167	872827, 873004, 873004	6, 14, 7	4.80449, 14.29802, 6.92462	3.96512, 8.57291, 5.17829	2.51418, 1	3	igfbp5	59889
GL173082.1	894637	895366	894878, 895095, 894814	17, 8, 6	18.46847, 7.07159, 7.12188	10.34725, 5.20073, 5.22884	15.29249,	3	igfbp5	37690
GL173082.1	909320	909882	909821, 909811, 909813	13, 21, 12	12.03613, 20.60499, 14.40488	7.42394, 10.48124, 8.92522	9.15434, 1	3	igfbp5	23174
GL173082.1	943489	943876	943569, 943510, 943847	8, 11, 6	6.55639, 8.04011, 5.53406	4.88389, 5.34803, 4.40503	4.06705, 5	3	igfbp5	0
GL173082.1	953629	954873	953814, 954471, 954074	15, 17, 15	13.30906, 14.4143, 16.08914	7.74297, 7.90204, 9.41427	10.35786,	3	igfbp5	6288
GL173082.1	964500	965247	964956, 964935, 965037	11, 25, 20	9.71398, 23.09161, 23.73483	6.39253, 10.65679, 12.62984	6.97155, 1	3	igfbp2	8970
GL173082.1	974394	975207	975031, 975143, 974513	9, 13, 4	8.35434, 10.81092, 3.82084	5.86444, 6.66988, 3.36473	5.71584, 8	3	igfbp2	0
GL173082.1	991171	991883	991605, 991667, 991421	11, 29, 7	9.17548, 25.5962, 7.40077	6.05941, 10.75635, 5.43746	6.47227, 2	3	igfbp2	0
GL173082.1	997365	997952	997906, 997921, 997778	37, 34, 21	44.50208, 35.88116, 25.57125	18.62207, 14.81115, 13.40941	40.73035,	3	igfbp2	0
GL173082.1	1009074	1009750	1009141, 1009107, 1009274	12, 18, 13	11.85767, 16.75904, 15.0131	7.51014, 9.05198, 9.14923	8.98544, 1	3	MGC147242	8606
GL173082.1	1021383	1021866	1021558, 1021546, 1021661	44, 50, 30	51.14963, 53.66526, 35.62895	19.35994, 18.06508, 16.26673	47.28226,	3	MGC147242	106
GL173083.1	463114	463748	463473, 463216, 463692	8, 20, 7	6.66791, 17.44765, 7.40077	4.95324, 8.88669, 5.43746	4.17132, 1	3	ABLIM3	0
GL173083.1	988439	989075	988488, 988448, 988489	33, 38, 38	37.29474, 43.25339, 49.629	16.05296, 17.44735, 21.2019	33.63731,	3	ppp2r2b	19519
GL173084.1	50056	50945	50853, 50812, 50393	6, 13, 5	4.45871, 11.61168, 4.99237	3.74614, 7.16421, 4.0781	2.26108, 8	3	dock6	0
GL173084.1	58773	59925	59070, 58961, 59462, 58910	11, 9, 10, 7	10.1122, 6.67403, 7.72315, 6.92462	6.63573, 4.82293, 5.32711, 5.17829	7.34229, 4	3	dock6	0
GL173084.1	70292	71403	70594, 70892, 70533	14, 17, 11	12.18032, 11.56918, 10.52088	7.28937, 6.20732, 6.8818	9.29157, 8	3	dock6	3005
GL173084.1	174391	174778	174530, 174516, 174498	7, 5, 4	6.37715, 3.25262, 3.94606	4.86324, 3.01775, 3.43278	3.90257, 1	3	elavl3	3636
GL173084.1	231041	232118	231497, 231868, 231528	10, 8, 8	10.81731, 7.07159, 8.4586	7.17456, 5.20073, 5.99709	8.00764, 4	3	znf653	7758
GL173084.1	241529	242413	241774, 242094, 241819	7, 8, 5	6.50909, 6.24338, 4.64121	4.94073, 4.6873, 3.88372	4.02371, 3	3	znf653	0
GL173084.1	284137	284638	284507, 284304, 284462	5, 11, 6	4.54329, 10.21061, 6.6567	3.82752, 6.69533, 5.00845	2.33, 7.59	3	tmem56.2	0
GL173084.1	311924	313042	312472, 312514, 312571	11, 21, 12	9.5732, 16.31939, 12.82015	6.30586, 7.93648, 8.0686	6.84587, 1	3	cnn2	0
GL173084.1	377670	378318	378103, 378100, 378114	15, 13, 10	16.36908, 13.14514, 11.38247	9.57629, 8.09002, 7.47651	13.27778,	3	lyl1	8733
GL173084.1	410788	411170	410974, 410985, 410879	10, 18, 6	10.07458, 18.29879, 7.29403	6.75761, 9.984, 5.30581	7.30652, 1	3	lyl1	17298
GL173084.1	423944	424217	424110, 424115, 424094	14, 28, 20	12.38175, 24.65647, 22.04588	7.4133, 10.55907, 11.64959	9.48175, 2	3	nfix	4832
GL173084.1	450074	450602	450552, 450598, 450575	47, 55, 33	54.52553, 63.09522, 43.8899	19.91382, 20.97552, 19.91902	50.60815,	3	nfix	0
GL173084.1	501809	502379	501820, 501817, 501907	11, 23, 16	11.42621, 18.61395, 17.62687	7.41109, 8.68467, 10.0901	8.57514, 1	3	nfix	27674
GL173084.1	554092	554479	554417, 554435, 554432	11, 20, 12	11.35776, 21.01248, 16.0427	7.37194, 11.03495, 9.71071	8.51017, 1	3	gadd45gip1	46974
GL173084.1	604410	605194	604912, 604799, 604707	4, 8, 7	3.74195, 7.07159, 8.69489	3.32091, 5.20073, 6.06379	1.6222, 4.6	3	gadd45gip1	354
GL173084.1	611493	612078	611855, 611864, 611493	5, 8, 6	4.77263, 7.07159, 7.29403	3.9578, 5.20073, 5.30581	2.51418, 4	3	NA	0
GL173084.1	667611	668055	667795, 667892, 667999	4, 8, 6	3.74195, 7.07159, 7.29403	3.32091, 5.20073, 5.30581	1.6222, 4.6	3	farsa	4798
GL173084.1	696699	697265	696952, 696970, 697077	7, 11, 8	6.60038, 8.85546, 8.4586	4.99378, 5.85974, 5.99709	4.10761, 6	3	gcdh	0
GL173084.1	716954	717429	717398, 717378, 717402	38, 56, 37	44.89375, 64.75166, 46.6052	18.41369, 21.41842, 19.86311	41.11632,	3	lgals9	0
GL173084.1	828051	828511	828164, 828320, 828260	10, 5, 6	11.06011, 3.94561, 7.29403	7.306, 3.46715, 5.30581	8.22712, 1	3	lgals9	77786
GL173084.1	899695	900909	900677, 900770, 900425	8, 7, 9	6.8227, 5.42726, 9.52439	5.04883, 4.28044, 6.53516	4.31393, 3	3	rab3d	9279
GL173084.1	909453	910000	909731, 909748, 909685	11, 10, 6	8.47312, 8.70492, 6.35308	5.62011, 5.93975, 4.85498	5.81094, 6	3	rab3d	188
GL173084.1	928458	929000	928715, 928646, 928935	10, 19, 9	8.70772, 16.5695, 10.28145	5.94148, 8.65063, 6.93568	6.03286, 1	3	tmem205	376
GL173084.1	940692	941318	941246, 941260, 941281	6, 10, 6	5.72604, 8.4887, 6.87953	4.51498, 5.80621, 5.11627	3.33547, 5	3	tmem205	2914
GL173084.1	993817	995163	994114, 994414, 994099	22, 19, 7	23.3605, 17.40691, 7.45266	11.81917, 9.15675, 5.46481	20.02603,	3	dnase2	3078
GL173084.1	1028176	1028759	1028551, 1028229, 1028305	11, 12, 6	8.262, 8.99251, 5.83216	5.4875, 5.75004, 4.57461	5.63022, 6	3	thibz	12557
GL173085.1	139815	140286	140198, 139988, 140193	5, 8, 6	4.82177, 7.07159, 7.29403	3.98509, 5.20073, 5.30581	2.51418, 4	3	rps19	31358
GL173085.1	145347	145905	145873, 145890, 145878	20, 17, 10	21.18126, 16.11578, 13.18184	11.13591, 8.93835, 8.33771	17.91394,	3	rps19	36890
GL173085.1	229522	230436	229615, 229696, 229592	15, 40, 13	14.98937, 47.65043, 15.52427	8.76428, 19.14471, 9.42125	11.95813,	3	cadm4	108463
GL173085.1	287259	287836	287268, 287325, 287266	15, 15, 10	14.92086, 12.13791, 10.3632	8.72319, 7.02404, 6.92219	11.89292,	3	cadm4	51063
GL173085.1	451055	451994	451443, 451442, 451443	21, 21, 22	23.32111, 18.71315, 26.58401	12.10792, 9.34355, 13.71123	19.98747,	3	NA	10300
GL173085.1	487565	488503	487821, 487845, 487826	32, 50, 35	31.48135, 47.42907, 39.13085	13.03943, 14.80392, 16.307	27.94013,	3	NA	0
GL173085.1	514737	515995	515738, 514936, 514902	6, 7, 5	5.1345, 5.97725, 5.45388	4.16817, 4.62287, 4.3157	2.80161, 3	3	NA	24483
GL173085.1	582920	583452	583425, 583392, 583419	30, 38, 14	36.57213, 48.41922, 19.68329	16.8223, 20.49348, 11.3696	32.92888,	3	NA	18252
GL173085.1	598952	599436	599135, 599365, 599224	4, 6, 3	3.74195, 4.93312, 3.47371	3.32091, 4.04501, 3.03189	1.6222, 2.7	3	xrcc1	30798
GL173085.1	611824	612405	612359, 612274, 612194	7, 8, 9	6.60038, 7.07159, 9.41119	4.99378, 5.20073, 6.47287	4.10761, 4	3	xrcc1	17829
GL173085.1	622490	622932	622672, 622673, 622673	29, 46, 23	32.23876, 49.06374, 26.51165	14.63993, 17.27373, 13.36842	28.68208,	3	xrcc1	7302
GL173085.1	754288	755029	754533, 754536, 754664	7, 9, 5	5.52352, 6.04841, 4.84526	4.34124, 4.42311, 3.99806	3.15454, 3	3	phldb3	8390
GL173085.1	804815	805175	804855, 804886, 804833	12, 11, 4	12.45843, 10.60895, 4.38152	7.86167, 6.9343, 3.65448	9.54648, 7	3	NA	1753
GL173085.1	849280	850038	849356, 849304, 849891	16, 14, 9	13.52743, 10.80331, 8.69919	7.62404, 6.44051, 6.06795	10.56607,	3	NA	17762
GL173085.1	959951	960370	960317, 960302, 960179	15, 12, 6	14.8531, 9.50574, 6.25896	8.68248, 6.07027, 4.80589	11.82783,	3	NA	128433
GL173086.1	31	547	232, 279, 161	10, 18, 5	11.06011, 19.97713, 5.9506	7.306, 10.97931, 4.54784	8.22712, 1	3	crls1	8572
GL173086.1	122055	122795	122490, 122507, 122514	17, 24, 17	18.82666, 27.07821, 23.17908	10.55599, 13.37637, 12.87887	15.63723,	3	fermt1	52225
GL173086.1	269962	270510	270345, 270384, 270110	17, 11, 11	16.42934, 8.00793, 12.19945	9.12876, 5.32781, 7.8422	13.33656,	3	bmp2	18099
GL173086.1	765149	765805	765490, 765431, 765498	17, 23, 11	15.5079, 21.25586, 10.66921	8.56821, 10.22998, 6.9701	12.45101,	3	plcb1	0
GL173086.1	774951	775538	775438, 775027, 775009	7, 8, 8	6.42049, 6.4822, 8.86315	4.8888, 4.83755, 6.21039	3.94161, 4	3	plcb1	0
GL173086.1	966750	967711	967523, 967080, 967128	9, 16, 12	8.77314, 15.80758, 13.15613	6.111, 9.01376, 8.25729	6.09355, 1	3	plcb1	45408
GL173086.1	975127	976906	976124, 976121, 976061	45, 61, 48	55.98753, 71.35845, 61.98795	21.71871, 22.64482, 23.78217	52.05133,	3	plcb1	53785
GL173087.1	237091	238120	237762, 237970, 237992	13, 14, 10	14.18705, 14.45897, 11.5283	8.69244, 8.66788, 7.55211	11.19086,	3	ltn1	165992
GL173087.1	519948	520675	520526, 520405, 520556	8, 26, 5	6.70593, 24.16991, 5.03069	4.9768, 10.95194, 4.09861	4.20487, 2	3	map3k7cl	14271
GL173087.1	854905	855774	855488, 855480, 855129	9, 5, 5	8.16099, 3.65953, 4.95468	5.74847, 3.2852, 4.05779	5.53692, 1	3	grik1	63652
GL173088.1	493	2083	676, 1340, 1042	6, 17, 10	4.13253, 10.71452, 9.58601	3.53466, 5.71999, 6.47231	1.97664, 8	3	NA	26907
GL173088.1	493	2083	1132, 1340, 1042	8, 17, 10	5.80896, 10.71452, 9.58601	4.41049, 5.71999, 6.47231	3.41336, 8	3	NA	26907
GL173088.1	4745	7595	4908, 5563, 5526, 6653	10, 27, 9, 13	5.23077, 15.6584, 5.22034, 9.76299	3.75891, 6.1968, 3.89338, 6.02032	2.8906, 12	3	NA	21395
GL173088.1	4745	7595	6639, 5563, 5526, 6653	9, 27, 9, 13	4.91578, 15.6584, 5.22034, 9.76299	3.69989, 6.1968, 3.89338, 6.02032	2.60124, 1	3	NA	21395
GL173088.1	7824	9200	8500, 8796, 8388	10, 16, 9	5.85903, 7.71989, 5.94272	4.14909, 4.27864, 4.35539	3.4588, 5.2	3	NA	19790
GL173088.1	167994	168530	168054, 168358, 168198	8, 6, 7	6.90289, 4.4843, 8.28621	5.09802, 3.76254, 5.87881	4.38737, 2	3	mmp16	30635
GL173088.1	179299	179789	179762, 179759, 179650	27, 34, 7	30.66321, 35.08167, 7.45266	14.45243, 14.34568, 5.46481	27.14087,	3	mmp16	19376
GL173088.1	359105	359447	359221, 359236, 359237	11, 13, 12	11.29029, 11.36332, 15.5838	7.3332, 7.01137, 9.50165	8.44541, 8	3	NA	4964
GL173088.1	615213	615722	615471, 615565, 615397	11, 11, 7	10.43535, 9.57292, 7.40077	6.83063, 6.30569, 5.43746	7.6474, 6.9	3	pskh2	1766
GL173088.1	687357	688152	687518, 687951, 687550	10, 19, 9	9.49535, 17.00475, 9.8849	6.41851, 8.91339, 6.72947	6.77344, 1	3	ca2	61468
GL173088.1	953733	954196	953775, 953815, 953760	11, 11, 9	10.49148, 9.05476, 9.24728	6.86423, 5.98422, 6.38161	7.70044, 6	3	lrrcc1	41179
GL173088.1	964843	965274	965090, 965067, 965190	8, 10, 4	7.42892, 8.84031, 4.59709	5.41453, 6.02287, 3.75554	4.85345, 6	3	lrrcc1	52289
GL173088.1	973826	974975	974496, 974594, 974259	10, 19, 11	9.71769, 19.8035, 13.64572	6.54998, 10.60442, 8.58592	6.97155, 1	3	lrrcc1	61272
GL173089.1	33972	34534	34506, 34506, 34506	56, 61, 70	67.4078, 73.22185, 91.85702	22.91194, 23.6762, 29.41392	63.33852,	3	NA	145449
GL173089.1	51610	52130	51638, 51634, 51692	25, 23, 5	28.23973, 21.70337, 5.9506	13.72472, 10.4968, 4.54784	24.77449,	3	NA	127853
GL173089.1	141559	142036	141914, 141746, 141684	9, 11, 4	9.72082, 10.60895, 4.67325	6.64182, 6.9343, 3.78987	6.97155, 7	3	NA	37947
GL173089.1	189243	189765	189703, 189687, 189642	11, 21, 10	10.49148, 19.00078, 10.97191	6.86423, 9.5157, 7.25854	7.70044, 1	3	NA	7657
GL173089.1	209340	209725	209492, 209510, 209601	12, 6, 6	10.63412, 4.18132, 7.29403	6.77016, 3.56655, 5.30581	7.83559, 2	3	NA	27754
GL173089.1	549348	550394	549580, 549916, 549609	8, 9, 12	5.13458, 5.36772, 11.81848	3.97494, 3.98738, 7.4869	2.80161, 3	3	NA	86075
GL173089.1	573370	574108	573950, 573862, 573903	13, 18, 4	13.94585, 19.59288, 4.67325	8.5554, 10.7543, 3.78987	10.95967,	3	NA	62361
GL173089.1	597043	597672	597277, 597273, 597465	10, 17, 12	11.06011, 18.56115, 16.0427	7.306, 10.40145, 9.71071	8.22712, 1	3	NA	38797
GL173089.1	655747	656179	656080, 656034, 656039	7, 9, 15	6.99564, 8.21099, 19.29094	5.21786, 5.77858, 11.15405	4.47307, 5	3	XB-GENE-5820599	0
GL173089.1	712167	713007	712564, 712724, 712587	18, 18, 13	19.78725, 18.52518, 15.89249	10.86837, 10.11992, 9.61177	16.56612,	3	rraga	2762
GL173089.1	771544	772111	771872, 771850, 771780	25, 46, 25	29.94456, 51.22834, 31.77551	14.7276, 18.48945, 15.77668	26.43808,	3	NA	28378
GL173089.1	920363	920763	920606, 920605, 920606	13, 13, 8	14.97989, 13.14514, 10.1465	9.13126, 8.09002, 6.82176	11.94968,	3	NA	70426
GL173090.1	6291	7553	6389, 6860, 6873	12, 18, 5	12.11599, 18.60217, 5.64747	7.66241, 10.16604, 4.40911	9.23027, 1	3	grin2a	27932
GL173090.1	282587	283335	283196, 283169, 283219	18, 17, 17	15.7774, 13.42125, 17.51936	8.45576, 7.30129, 9.78549	12.70982,	3	grin2a	162626
GL173091.1	647180	648317	647419, 647895, 647982	12, 21, 14	11.92103, 19.00078, 16.64329	7.54764, 9.5157, 9.89801	9.04508, 1	3	rbm19	5683
GL173091.1	905790	906486	905974, 905889, 905968	13, 13, 10	14.10549, 12.09006, 11.6791	8.64628, 7.45678, 7.62925	11.11201,	3	ficd	5469
GL173092.1	8876	9540	9039, 9355, 8963	6, 11, 4	5.55471, 10.3713, 4.67325	4.41698, 6.79219, 3.78987	3.18192, 7	3	ccnb3	42081
GL173092.1	274216	274499	274471, 274483, 274468	18, 23, 13	19.00683, 22.52393, 14.69628	10.40758, 10.9881, 8.97644	15.81175,	3	znf534	16157
GL173092.1	476665	477274	476838, 476787, 476778	6, 8, 4	5.20598, 6.37769, 4.67325	4.2113, 4.77199, 3.78987	2.86702, 4	3	NA	7810
GL173092.1	694760	695577	695357, 695306, 695311	14, 18, 9	13.70063, 16.52108, 9.64095	8.21651, 8.90737, 6.59867	10.73143,	3	NA	17746
GL173093.1	671	2096	1666, 1298, 1292	13, 15, 9	13.94585, 15.80067, 11.21577	8.5554, 9.24574, 7.38849	10.95967,	3	NA	18878
GL173093.1	12333	13841	12578, 13236, 12607, 13181, 13682	10, 16, 6, 5, 8	8.08479, 11.6224, 4.96699, 3.83562, 6.66626	5.55446, 6.47081, 4.06589, 3.39785, 4.95222	5.46601, 8	3	NA	7133
GL173093.1	22727	23116	22985, 22777, 22813	7, 14, 10	5.49154, 12.18837, 10.97191	4.32107, 7.29433, 7.25854	3.12578, 9	3	NA	0
GL173093.1	227858	228343	227905, 227900, 228084	21, 18, 9	20.17379, 14.2312, 8.74593	10.2212, 7.5249, 6.09519	16.93778,	3	grk4	0
GL173093.1	347089	347294	347115, 347120, 347107	13, 12, 7	13.00701, 10.08092, 7.78342	8.00827, 6.42828, 5.63488	10.0703, 7	3	htt	0
GL173093.1	389366	389847	389559, 389594, 389556	11, 6, 11	11.42621, 4.93312, 11.49087	7.41109, 4.04501, 7.44794	8.57514, 2	3	htt	3751
GL173093.1	894566	895735	894765, 894736, 894744	67, 81, 39	65.46481, 76.16441, 38.56139	17.10446, 16.84555, 14.34016	61.41578,	3	lrpap1	211482
GL173095.1	67043	68498	67701, 67886, 68093	10, 19, 11	10.01299, 17.46611, 13.64572	6.7221, 9.19261, 8.58592	7.24763, 1	3	asap1	135500
GL173095.1	120115	120549	120266, 120220, 120184	12, 6, 13	12.05017, 4.25354, 17.40688	7.62377, 3.61361, 10.34389	9.16715, 2	3	asap1	83449
GL173095.1	130574	130958	130733, 130772, 130759	16, 30, 13	14.79948, 30.81101, 14.11158	8.40174, 13.40848, 8.64973	11.77649,	3	asap1	73040
GL173095.1	220603	221233	220742, 220657, 220672	9, 10, 5	7.67537, 8.04981, 4.74091	5.45199, 5.53254, 3.94006	5.08317, 5	3	asap1	0
GL173095.1	273495	273767	273733, 273741, 273757	19, 18, 12	16.59925, 13.93917, 11.21415	8.66856, 7.35127, 7.12434	13.49961,	3	42Sp50	5768
GL173095.1	278254	278956	278282, 278261, 278259	35, 59, 31	36.97461, 65.78364, 36.85188	15.04271, 20.57022, 16.59905	33.32366,	3	42Sp50	10527
GL173095.1	300663	301635	301420, 301395, 301460	10, 9, 8	9.13239, 7.88274, 7.99854	6.2007, 5.57944, 5.74345	6.43114, 5	3	fam49b	32407
GL173095.1	317983	318381	318022, 318235, 318157	6, 5, 5	5.90927, 3.94561, 5.3628	4.61743, 3.46715, 4.27046	3.50164, 1	3	fam49b	15661
GL173095.1	418045	418881	418159, 418159, 418159	20, 22, 15	20.06876, 18.93661, 14.11641	10.4676, 9.17097, 8.2366	16.8357, 1	3	fam49b	73121
GL173095.1	433717	434284	434046, 434068, 434065	11, 12, 12	10.49148, 9.74388, 13.01912	6.86423, 6.2187, 8.18077	7.70044, 7	3	fam49b	88793
GL173095.1	455167	455680	455625, 455676, 455634	53, 92, 61	58.92844, 108.73, 79.98518	19.60512, 26.86252, 27.53325	54.95334,	3	fam49b	110243
GL173095.1	477122	477629	477239, 477272, 477179	7, 8, 7	6.2098, 6.05185, 7.20145	4.76362, 4.56575, 5.33074	3.74679, 3	3	fam49b	132198
GL173095.1	530858	531469	530987, 531164, 530995	8, 10, 3	7.02691, 8.16488, 3.27564	5.17362, 5.60458, 2.93941	4.50222, 5	3	fam49b	185934
GL173095.1	861800	862634	861932, 862196, 862195	15, 17, 17	14.8531, 14.91871, 20.18522	8.68248, 8.20906, 11.32722	11.82783,	3	U2	54235
GL173095.1	878403	878785	878574, 878719, 878649	7, 11, 3	7.1696, 9.95469, 3.47371	5.31345, 6.5399, 3.03189	4.61046, 7	3	U2	70838
GL173095.1	947152	948008	947749, 947646, 947878	6, 11, 9	4.08609, 8.44639, 8.69919	3.50422, 5.60333, 6.06795	1.93351, 5	3	U2	139587
GL173095.1	989498	990000	989544, 989512, 989524	27, 48, 20	29.5337, 51.4621, 22.4589	13.7796, 17.73119, 11.89249	26.03702,	3	U2	181933
GL173096.1	13	667	308, 498, 94	8, 17, 7	8.42257, 16.56523, 8.21885	5.97763, 9.21112, 5.84717	5.76336, 1	3	NA	66126
GL173096.1	242295	242859	242718, 242782, 242774	8, 15, 10	8.42257, 14.71623, 12.42918	5.97763, 8.60008, 7.99682	5.76336, 1	3	gys1	25596
GL173096.1	247491	248257	248053, 248019, 247935	10, 21, 12	11.06011, 22.6637, 15.5838	7.306, 11.7178, 9.50165	8.22712, 1	3	lhb	21504
GL173096.1	488103	488378	488151, 488164, 488176	20, 27, 21	22.46383, 30.04218, 27.33352	11.89537, 14.08283, 14.37736	19.15645,	3	kcna7	10909
GL173098.1	882934	883405	883144, 883147, 883062	8, 15, 7	6.86256, 13.11311, 8.21885	5.0733, 7.62272, 5.84717	4.3506, 10	3	UBXN8	416170
GL173098.1	931919	933177	932338, 932562, 932541	9, 15, 6	8.55802, 13.69427, 6.5517	5.98518, 7.97895, 4.95623	5.8909, 10	3	UBXN8	465155
GL173098.1	939436	939841	939573, 939570, 939675	10, 15, 9	6.12672, 8.18687, 7.2501	4.31714, 4.69077, 5.1872	3.67045, 5	3	UBXN8	472672
GL173098.1	945623	946268	946147, 946141, 945860	14, 20, 7	8.04702, 9.72522, 4.28728	4.78808, 4.66519, 3.5411	5.43132, 7	3	UBXN8	478859
GL173098.1	947265	948254	947875, 947978, 947826	20, 40, 17	15.3077, 32.06002, 15.83523	7.62421, 10.63841, 8.76764	12.25967,	3	UBXN8	480501
GL173100.1	385508	385975	385569, 385595, 385594	7, 18, 6	7.1696, 19.97713, 7.29403	5.31345, 10.97931, 5.30581	4.61046, 1	3	COBLL1	29059
GL173100.1	609121	609843	609240, 609342, 609653	7, 13, 6	7.1696, 13.14514, 7.29403	5.31345, 8.09002, 5.30581	4.61046, 1	3	grb14	52854
GL173101.1	9317	9882	9452, 9400, 9466	7, 12, 5	5.58859, 9.66318, 4.99237	4.38214, 6.16843, 4.0781	3.21321, 7	3	kctd3	0
GL173101.1	358567	359146	359004, 359116, 359096	16, 21, 15	17.95866, 21.41771, 20.14748	10.28064, 10.97106, 11.5698	14.80292,	3	USH2A	0
GL173101.1	503199	504259	503797, 503650, 503622	11, 17, 9	12.43693, 17.26139, 11.64359	7.97018, 9.63099, 7.57973	9.52692, 1	3	USH2A	47090
GL173101.1	556852	557586	557386, 557168, 557172	22, 43, 15	15.19962, 31.66593, 11.69344	7.05137, 9.64688, 6.75208	12.15977,	3	esrrg	35649
GL173101.1	558689	559245	559012, 558760, 558877	12, 23, 10	5.95143, 11.30099, 6.39865	3.90323, 4.93041, 4.48862	3.52158, 8	3	esrrg	33990

GL173101.1	586530	587091	587066, 587065, 587076	123, 193, 118	159.40286, 251.04027, 164.39745	35.66523, 40.70907, 41.03696	154.62607	3	esrrg	6144
GL173101.1	780316	780942	780848, 780914, 780702	6, 8, 6	5.43318, 7.07159, 6.6567	4.34622, 5.20073, 5.00845	3.07284, 4	3	esrrg	35501
GL173102.1	71419	72702	72221, 72629, 71777	13, 22, 12	11.60911, 18.77934, 12.38374	7.16262, 9.07845, 7.81847	8.74856, 1	3	fam234a	0
GL173102.1	173117	173674	173214, 173586, 173218	12, 10, 6	10.68367, 7.29424, 5.60548	6.80062, 5.05593, 4.44624	7.88119, 4	3	mrpl28	31049
GL173102.1	304100	304680	304532, 304220, 304359	10, 14, 8	8.93828, 10.95872, 7.85773	6.08275, 6.53614, 5.66361	6.24946, 8	3	usp22	0
GL173102.1	442350	443090	442881, 442941, 442804	9, 17, 10	7.17299, 14.61162, 10.25108	5.13877, 8.02205, 6.85862	4.61317, 1	3	dhrs7b	17897
GL173102.1	536628	537181	536882, 537065, 536917	20, 22, 13	20.06876, 19.53861, 15.79818	10.4676, 9.52696, 9.56343	16.8357, 1	3	arpc1a	0
GL173102.1	889838	890571	890183, 890235, 889935	7, 6, 6	7.1696, 4.93312, 7.29403	5.31345, 4.04501, 5.30581	4.61046, 2	3	pdap1	81443
GL173102.1	902908	904500	903610, 903570, 904028	23, 23, 21	23.56695, 20.53538, 26.82916	11.61446, 9.80281, 14.10583	20.22607,	3	pdap1	94513
GL173102.1	911252	911856	911492, 911459, 911481	8, 12, 7	8.42257, 11.86108, 8.69489	5.97763, 7.51216, 6.06379	5.76336, 9	3	pdap1	102857
GL173102.1	943017	943557	943221, 943414, 943138	19, 28, 14	18.9632, 26.07315, 15.06595	10.09902, 11.37028, 9.02131	15.76968,	3	pdap1	134622
GL173103.1	760353	760969	760708, 760717, 760757	5, 9, 5	4.54329, 8.21099, 5.40789	3.82752, 5.77858, 4.29296	2.33, 5.708	3	dct	381722
GL173105.1	932184	934201	934047, 933459, 933695	41, 60, 35	33.95618, 45.10352, 30.20987	11.24566, 10.84257, 11.26982	30.36374,	3	stk40	14555
GL173106.1	79588	80313	79742, 79861, 79888	8, 17, 15	8.42257, 17.11659, 19.40672	5.97763, 9.54398, 11.21161	5.76336, 1	3	fzd3	7599
GL173106.1	97602	98132	97664, 97662, 97606	50, 47, 28	58.27484, 50.50956, 37.40405	20.64288, 17.64125, 17.99919	54.30824,	3	fzd3	0
GL173106.1	161764	162222	161794, 161804, 161794	45, 35, 30	52.81714, 36.6591, 36.39614	19.86366, 14.85933, 16.71894	48.92521,	3	elp3	2146
GL173106.1	203059	203737	203243, 203353, 203179	9, 6, 6	9.00096, 4.93312, 6.76586	6.24222, 4.04501, 5.06179	6.30808, 2	3	elp3	0
GL173106.1	258139	259051	258599, 258295, 258622	5, 8, 8	4.61714, 5.99047, 9.46129	3.86998, 4.52662, 6.50801	2.39372, 3	3	stk35	0
GL173106.1	268575	268982	268735, 268735, 268665	17, 29, 15	18.64535, 29.27601, 17.94632	10.45058, 12.88102, 10.45626	15.46286,	3	stk35	8937
GL173106.1	330697	331336	331044, 330984, 331080	8, 8, 7	7.38183, 5.59124, 7.45266	5.38666, 4.27045, 5.46481	4.80856, 3	3	pnoc	0
GL173106.1	380582	380928	380640, 380626, 380790	5, 8, 4	4.47183, 6.4822, 4.38152	3.78598, 4.83755, 3.65448	2.26935, 4	3	pnoc	16058
GL173106.1	386194	386771	386220, 386207, 386226	23, 55, 19	23.81148, 63.09522, 23.01756	11.76131, 20.97552, 12.47022	20.46387,	3	znf395	13171
GL173106.1	478627	478928	478653, 478676, 478678	8, 14, 4	7.93925, 13.38467, 4.22496	5.70996, 8.02582, 3.57747	5.33002, 1	3	NA	25729
GL173106.1	526209	527314	526911, 527177, 526394	14, 14, 8	14.67861, 11.36754, 8.4044	8.79666, 6.78813, 5.9678	11.66087,	3	NA	7970
GL173106.1	537794	538109	537949, 538059, 538064	7, 22, 13	7.1696, 21.49375, 16.49347	5.31345, 10.69716, 9.91245	4.61046, 1	3	NA	1363
GL173106.1	543262	543781	543513, 543385, 543598	9, 12, 9	8.35434, 9.0625, 10.87975	5.86444, 5.7937, 7.23113	5.71584, 6	3	NA	6831
GL173106.1	585023	585369	585025, 585050, 585054	42, 77, 47	50.21881, 95.64694, 59.22097	19.74117, 27.71257, 22.64924	46.36386,	3	NA	48592
GL173106.1	598596	598978	598786, 598612, 598625	4, 22, 9	3.74195, 24.27169, 11.64359	3.32091, 12.36262, 7.57973	1.6222, 21	3	NA	62165
GL173106.1	641988	642448	642019, 642024, 642038	18, 33, 13	21.07666, 39.56544, 16.93213	11.6109, 17.39878, 10.12358	17.81304,	3	ncoa1	97936
GL173106.1	730498	730883	730755, 730746, 730849	9, 7, 5	8.16099, 5.78791, 5.9506	5.74847, 4.5064, 4.54784	5.53692, 3	3	ncoa1	9501
GL173106.1	740619	741034	740635, 740636, 740836	8, 8, 5	7.83097, 5.53815, 4.91762	5.64832, 4.2362, 4.03768	5.22917, 3	3	ncoa1	0
GL173106.1	752135	752994	752775, 752214, 752608	8, 7, 6	6.55639, 4.516, 7.29403	4.88389, 3.6915, 5.30581	4.06705, 2	3	ncoa1	0
GL173106.1	808279	808862	808332, 808362, 808302	23, 23, 20	26.5636, 26.33488, 26.09407	13.3989, 13.26456, 13.92487	23.13966,	3	ncoa1	0
GL173106.1	838936	839289	839254, 839253, 839258	18, 34, 9	17.93309, 34.99558, 9.8849	9.76366, 14.29576, 6.72947	14.77832,	3	ncoa1	1839
GL173106.1	869537	869864	869779, 869817, 869695	11, 11, 17	9.30417, 9.57292, 17.99358	6.13939, 6.30569, 10.06761	6.59351, 6	3	adcy3	0
GL173106.1	906052	906658	906518, 906278, 906228	6, 11, 12	5.16999, 8.51798, 12.95194	4.18963, 5.64827, 8.14303	2.83325, 6	3	adcy3	0
GL173106.1	923826	924957	924236, 924232, 923973	16, 11, 10	16.01695, 10.00475, 11.31133	9.13983, 6.57041, 7.43928	12.9408, 7	3	dnajc27	0
GL173107.1	12136	12852	12241, 12361, 12337	8, 18, 7	7.57423, 17.13102, 7.84208	5.4999, 9.27793, 5.66426	4.98865, 1	3	wnt3a	85116
GL173107.1	83635	83963	83908, 83922, 83907	10, 15, 14	7.93364, 13.42325, 15.80333	5.45963, 7.81299, 9.43935	5.325, 10.6	3	wnt3a	14005
GL173107.1	84549	84873	84610, 84578, 84558	15, 11, 9	14.33586, 9.05476, 9.08997	8.36999, 5.98422, 6.2929	11.33286,	3	wnt3a	13095
GL173107.1	98552	98889	98559, 98561, 98579	23, 35, 21	25.13729, 35.8708, 26.23567	12.55499, 14.40349, 13.78052	21.75229,	3	wnt3a	0
GL173107.1	149243	149890	149674, 149671, 149310	8, 9, 5	6.41335, 6.76868, 4.99237	4.7944, 4.88312, 4.0781	3.93678, 4	3	wnt3a	32783
GL173107.1	220307	220725	220593, 220598, 220640	6, 6, 4	5.96715, 4.93312, 4.67325	4.64927, 4.04501, 3.78987	3.52158, 2	3	arf5	19949
GL173107.1	226507	227491	226982, 226983, 227140	12, 26, 16	11.85767, 24.56489, 18.1835	7.51014, 11.18383, 10.40854	8.98544, 2	3	arf5	13183
GL173107.1	236026	236586	236378, 236269, 236398	11, 15, 11	11.85906, 13.53022, 12.48556	7.65504, 7.87854, 7.99611	8.98595, 1	3	arf5	4088
GL173107.1	325986	326391	326101, 326047, 326084	6, 9, 4	5.96715, 8.21099, 4.67325	4.64927, 5.77858, 3.78987	3.52158, 5	3	obscn	0
GL173107.1	475698	476131	476057, 476111, 476079	15, 29, 16	16.63109, 30.10341, 20.18713	9.72648, 13.37008, 11.49707	13.52931,	3	NA	0
GL173107.1	495588	496161	495606, 495646, 495687	16, 34, 17	16.60441, 35.60957, 21.19882	9.49088, 14.65267, 11.87761	13.50404,	3	NA	12622
GL173107.1	558142	558639	558465, 558484, 558427	11, 23, 17	10.78278, 23.98856, 21.09096	7.03733, 11.86759, 11.82018	7.97565, 2	3	gjc2	0
GL173107.1	620123	620819	620169, 620285, 620264, 620701	13, 19, 6, 5	13.07382, 18.96889, 6.12365, 4.64121	8.04786, 10.10245, 4.73409, 3.88372	10.13353,	3	NA	5356
GL173107.1	646263	646743	646284, 646296, 646303	8, 12, 4	8.42257, 11.86108, 4.67325	5.97763, 7.51216, 3.78987	5.76336, 9	3	cyp8b1	1577
GL173107.1	686127	687210	687060, 686405, 686536	14, 12, 6	10.41299, 7.88606, 4.82956	6.20094, 5.0632, 3.98076	7.62633, 5	3	higd1a	2339
GL173107.1	688140	689084	688580, 688591, 688705	20, 24, 21	17.42069, 21.13179, 22.75397	8.87054, 9.83481, 11.77155	14.2845, 1	3	ccdc13	1070
GL173107.1	773661	773971	773918, 773817, 773852	8, 8, 5	7.77807, 6.73981, 4.4844	5.618, 4.99774, 3.79332	5.17956, 4	3	nktr	3084
GL173107.1	794479	795927	795183, 795379, 795399	14, 21, 10	12.18032, 18.48988, 10.25108	7.28937, 9.21025, 6.85862	9.29157, 1	3	nktr	0
GL173107.1	798806	799559	799068, 799249, 799154	11, 12, 7	8.12678, 7.94095, 6.51247	5.40251, 5.09702, 4.9427	5.50441, 5	3	nktr	0
GL173107.1	814842	815348	815081, 815175, 815256	4, 6, 5	3.57366, 4.79966, 5.31857	3.22499, 3.96211, 4.24819	1.59706, 2	3	sec22c	351
GL173107.1	838090	838621	838182, 838152, 838138	8, 7, 6	8.10827, 5.21943, 6.08001	5.80497, 4.14808, 4.71063	5.48761, 2	3	sec22c	14397
GL173108.1	107590	108605	108074, 108359, 108146	14, 13, 10	9.80114, 7.66441, 8.29242	5.82799, 4.74429, 5.68421	7.04828, 5	3	pcdh7	253544
GL173108.1	628171	628640	628386, 628430, 628391	4, 7, 8	3.74195, 5.65148, 10.1465	3.32091, 4.42152, 6.82176	1.6222, 3.3	3	pcdh7	237705
GL173109.1	754611	755074	754757, 754942, 754747	10, 18, 6	9.83337, 18.00717, 6.6567	6.61776, 9.80837, 5.00845	7.07841, 1	3	ehbp1	0
GL173110.1	307080	307462	307351, 307328, 307403	11, 22, 9	10.3799, 21.56447, 9.08997	6.79736, 10.73971, 6.2929	7.59574, 1	3	slc29a2	10421
GL173111.1	149830	150838	150283, 150055, 150243	28, 36, 19	29.46427, 35.1431, 20.8607	13.36659, 13.59847, 11.2325	25.96977,	3	nrip1	29088
GL173111.1	232362	233899	232641, 233262, 233586	6, 8, 8	5.68215, 6.70176, 8.9249	4.49007, 4.97421, 6.24211	3.29732, 4	3	nrip1	407
GL173111.1	293929	295183	294409, 294435, 294436, 295160	11, 22, 9, 5	10.66406, 21.56447, 8.88966, 4.45433	6.96705, 10.73971, 6.17838, 3.77574	7.86396, 1	3	nrip1	61974
GL173111.1	797768	798144	797935, 797993, 797955	10, 21, 9	8.61898, 20.96503, 9.82249	5.88679, 10.69835, 6.69629	5.94907, 1	3	xtr-mir-125b-2	47842
GL173112.1	23650	23996	23963, 23962, 23954	15, 29, 14	18.26603, 34.37277, 19.03529	10.62691, 15.90664, 11.08274	15.09729,	3	dopey2	55638
GL173112.1	88390	88984	88623, 88521, 88519	7, 8, 6	6.46447, 6.37769, 6.82211	4.91463, 4.77199, 5.08889	3.98312, 4	3	dopey2	0
GL173112.1	282529	283171	282559, 282587, 282562	49, 96, 43	60.07519, 121.38034, 55.37987	22.16717, 31.16183, 22.31234	56.08595,	3	NA	268
GL173112.1	296081	296658	296528, 296423, 296473	16, 21, 7	17.86567, 20.25932, 7.72582	10.22745, 10.27277, 5.6058	14.71418,	3	NA	13820
GL173112.1	348555	349513	348654, 348719, 348607	13, 13, 8	13.79066, 10.81092, 9.31951	8.46641, 6.66988, 6.43944	10.8171, 8	3	NA	66294
GL173112.1	400689	401238	401197, 401209, 401114	14, 23, 8	15.40455, 22.38211, 7.90404	9.21493, 10.90305, 5.68997	12.35264,	3	NA	118428
GL173112.1	515271	516405	515746, 516052, 515425	15, 30, 12	13.25675, 23.5747, 13.01912	7.71088, 9.34511, 8.18077	10.3079, 2	3	runx1	79380
GL173112.1	536210	536584	536537, 536533, 536553	12, 10, 6	12.45843, 7.97488, 5.91225	7.86167, 5.48553, 4.61907	9.54648, 5	3	runx1	59201
GL173112.1	585221	585756	585643, 585645, 585655	31, 43, 29	37.61042, 48.07102, 38.64196	17.04674, 18.03457, 18.368	33.94708,	3	runx1	10029
GL173112.1	652221	652693	652667, 652635, 652597	9, 14, 8	7.71701, 11.36754, 10.1465	5.47767, 6.78813, 6.82176	5.12244, 8	3	runx1	0
GL173112.1	721230	721847	721591, 721614, 721500	61, 73, 45	65.80561, 72.90292, 49.23478	19.68267, 18.23369, 17.80746	61.75326,	3	patl2	0
GL173112.1	800902	801487	801242, 801242, 801239	20, 20, 9	20.06876, 18.84531, 10.87975	10.4676, 9.72843, 7.23113	16.8357, 1	3	donson	0
GL173113.1	6924	7601	6953, 6951, 6946	38, 76, 20	46.91574, 88.27624, 23.83369	19.60794, 24.33707, 12.68602	43.10862,	3	magi1	265627
GL173113.1	58608	59214	58979, 58802, 58835	8, 14, 11	7.24433, 12.92044, 12.9493	5.30475, 7.74345, 8.23866	4.68023, 1	3	magi1	214014
GL173113.1	163444	163858	163566, 163546, 163514	9, 9, 6	6.63709, 5.72602, 5.39683	4.79941, 4.21655, 4.32487	4.14229, 3	3	magi1	109370
GL173113.1	165167	165455	165281, 165372, 165397	14, 19, 10	11.93818, 16.36083, 10.47831	7.14017, 8.52498, 6.98694	9.06106, 1	3	magi1	107773
GL173113.1	244415	244721	244527, 244515, 244448	15, 8, 4	16.36908, 6.70176, 4.50795	9.57629, 4.97421, 3.71446	13.27778,	3	magi1	28507
GL173113.1	250866	251863	251611, 251599, 251067	22, 42, 13	23.89932, 46.90917, 13.21599	12.1411, 17.81128, 8.13179	20.54867,	3	magi1	21365
GL173113.1	258477	258931	258530, 258554, 258485	28, 50, 26	32.26664, 60.31878, 31.93369	15.03533, 21.81793, 15.54972	28.70906,	3	magi1	14297
GL173113.1	294926	295620	295582, 295574, 295573	16, 24, 10	16.08763, 25.20527, 12.16489	9.18228, 12.25879, 7.87042	13.00852,	3	magi1	0
GL173113.1	320279	320828	320338, 320297, 320299	15, 25, 13	15.49092, 25.83246, 14.46982	9.06314, 12.28718, 8.85107	12.43497,	3	magi1	0
GL173113.1	385425	386018	385899, 385796, 385832	31, 41, 22	29.63639, 39.04127, 23.93583	12.35052, 13.8335, 12.16286	26.13704,	3	magi1	10668
GL173113.1	428126	429094	428830, 428919, 428714	10, 24, 7	10.39648, 25.91323, 8.69489	6.94096, 12.68264, 6.06379	7.61003, 2	3	magi1	53369
GL173113.1	466535	467454	467142, 467085, 466909	10, 15, 5	10.20047, 12.18092, 5.40789	6.82978, 7.05041, 4.29296	7.42612, 9	3	NA	64230
GL173113.1	502141	502729	502605, 502605, 502605	10, 17, 8	10.67246, 17.87191, 9.25066	7.09497, 9.99549, 6.40569	7.87096, 1	3	NA	28955
GL173113.1	547027	547763	547717, 547690, 547717	8, 20, 10	7.1122, 17.71959, 10.65691	5.22529, 9.04982, 7.08637	4.57946, 1	3	NA	14708
GL173113.1	556858	557753	557464, 557435, 557450	128, 168, 97	102.12423, 136.88959, 83.19047	13.73287, 14.75575, 14.88987	97.7157, 1	3	NA	24539
GL173113.1	559365	560295	559538, 559901, 559534	115, 126, 72	87.06195, 88.00365, 52.87708	12.37159, 10.98244, 10.88441	82.79468,	3	NA	27046
GL173113.1	567484	568191	567625, 567718, 567627	10, 16, 7	8.57531, 12.82211, 6.71158	5.85982, 7.19398, 5.05776	5.90712, 1	3	NA	31423
GL173113.1	591263	591798	591564, 591408, 591400	21, 36, 15	21.4094, 34.40266, 15.61614	10.96606, 13.18562, 9.13715	18.13434,	3	NA	7816
GL173113.1	608017	609057	608331, 608136, 608424	7, 9, 8	7.1696, 8.21099, 10.1465	5.31345, 5.77858, 6.82176	4.61046, 5	3	NA	7871
GL173113.1	660605	660868	660839, 660841, 660826	22, 36, 29	26.67998, 44.15687, 40.22158	13.76591, 18.85178, 19.23523	23.25346,	3	NA	60459
GL173113.1	689077	689500	689131, 689127, 689120	17, 27, 20	16.90454, 27.35215, 23.63711	9.41624, 12.47874, 12.57415	13.78827,	3	adamts9	66611
GL173113.1	717595	718387	717877, 717992, 717952	22, 23, 12	22.21386, 20.82882, 15.16114	11.13052, 9.97638, 9.30141	18.91372,	3	adamts9	37724
GL173113.1	754358	755407	755012, 755011, 755011	21, 22, 21	21.7275, 18.72754, 23.74199	11.15737, 9.04803, 12.35575	18.44198,	3	adamts9	704
GL173113.1	759463	760206	760170, 760165, 760178	17, 34, 8	16.83467, 35.34314, 8.24665	9.37407, 14.49755, 5.88165	13.72157,	3	adamts9	0
GL173113.1	793399	793948	793732, 793681, 793610	9, 13, 9	9.72082, 10.63899, 9.76105	6.64182, 6.56333, 6.66343	6.97155, 7	3	adamts9	0
GL173113.1	852701	853314	853120, 852879, 853005	6, 16, 11	5.27951, 14.57865, 12.13051	4.25534, 8.26691, 7.80464	2.9335, 11	3	adamts9	0
GL173113.1	864914	865485	865432, 865401, 865423	8, 8, 13	8.05102, 6.51779, 14.5443	5.77295, 4.8598, 8.89247	5.43473, 4	3	adamts9	0
GL173115.1	69934	70513	70302, 70284, 70343	19, 28, 13	18.89089, 30.31328, 15.09516	10.05535, 13.87249, 9.19347	15.69992,	3	myl3	298834
GL173115.1	116858	117785	117602, 117625, 117602	14, 13, 12	14.10012, 13.14514, 15.26371	8.4555, 8.09002, 9.35067	11.10747,	3	myl3	251562
GL173115.1	124593	124939	124684, 124684, 124685	11, 9, 7	10.5483, 8.21099, 8.21885	6.89817, 5.77858, 5.84717	7.75329, 5	3	myl3	244408
GL173115.1	171286	171676	171421, 171455, 171305	7, 12, 15	6.33443, 9.42893, 17.07266	4.83795, 6.02236, 9.97618	3.86259, 6	3	myl3	197671
GL173115.1	223966	224363	224040, 224083, 224188	17, 24, 6	18.73544, 25.82195, 7.05929	10.50302, 12.62806, 5.20024	15.55015,	3	myl3	144984
GL173115.1	280050	280617	280446, 280446, 280408	6, 9, 5	5.96715, 8.21099, 5.9506	4.64927, 5.77858, 4.54784	3.52158, 5	3	myl3	88730
GL173115.1	435845	436202	436005, 436030, 436112	8, 9, 7	8.42257, 8.21099, 8.08824	5.97763, 5.77858, 5.7849	5.76336, 5	3	myl3	58684
GL173115.1	457366	457990	457720, 457661, 457770	12, 24, 16	11.37748, 23.41175, 20.07743	7.22306, 11.1842, 11.44009	8.52862, 2	3	myl3	80205
GL173115.1	569821	570219	569966, 569883, 569849	9, 7, 8	9.00096, 5.97725, 8.24665	6.24222, 4.62287, 5.88165	6.30808, 3	3	pth1r	84527
GL173115.1	813050	813645	813092, 813325, 813502	7, 6, 6	6.2923, 4.27812, 5.95321	4.81292, 3.62957, 4.64162	3.8235, 2.1	3	tmie	4625
GL173115.1	861292	861854	861556, 861504, 861815	5, 8, 5	4.11592, 7.07159, 5.06966	3.57272, 5.20073, 4.11933	1.96078, 4	3	als2cl	0
GL173115.1	881142	881793	881688, 881533, 881280	13, 24, 9	11.21374, 23.55545, 10.28145	6.91908, 11.27011, 6.93568	8.37313, 2	3	als2cl	0
GL173115.1	891016	891889	891602, 891602, 891640	13, 21, 9	13.27928, 20.53474, 10.28145	8.16901, 10.43888, 6.93568	10.32936,	3	als2cl	0
GL173116.1	591691	592014	591721, 591783, 591740	12, 13, 13	12.45843, 11.71436, 15.26294	7.86167, 7.22722, 9.28325	9.54648, 9	3	impdh1	4662
GL173117.1	695118	695788	695581, 695482, 695442	17, 21, 6	21.19088, 22.06021, 7.29403	11.87339, 11.35706, 5.30581	17.92307,	3	b9d2	67040
GL173117.1	801391	802003	801895, 801681, 801574	15, 17, 12	13.91603, 14.51225, 10.98235	8.11445, 7.96159, 6.98343	10.93128,	3	exosc5	44714
GL173117.1	804360	805893	804585, 804537, 804928	14, 23, 15	11.61573, 20.88864, 14.32502	6.94127, 10.01184, 8.36342	8.75458, 1	3	exosc5	40824
GL173118.1	283568	284737	284325, 284090, 284435	173, 307, 130	175.25128, 321.52258, 141.17014	22.67534, 26.24785, 24.85333	170.39316	3	NA	15047
GL173118.1	717431	718125	717734, 717561, 717878	6, 10, 5	5.43318, 8.32357, 5.40789	4.34622, 5.70361, 4.29296	3.07284, 5	3	smap1	0
GL173120.1	3201	3717	3238, 3545, 3592	6, 9, 9	5.90927, 8.21099, 9.8849	4.61743, 5.77858, 6.72947	3.50164, 5	3	herpud2	22098
GL173120.1	880169	881385	880676, 881071, 881017	33, 47, 23	28.07365, 35.40679, 18.75609	10.79372, 10.21849, 8.76608	24.61225,	3	AMPH	46185
GL173120.1	889232	889830	889366, 889382, 889366	43, 74, 29	22.44016, 42.4653, 19.29416	6.192, 7.68614, 7.46436	19.13319,	3	AMPH	55248
GL173120.1	891817	893622	892115, 892089, 892045, 893533	58, 84, 37, 27	28.36979, 40.13059, 21.89628, 12.61865	6.06976, 6.21495, 6.94731, 4.90765	24.90104,	3	AMPH	57833
GL173120.1	891817	893622	892641, 892089, 892045, 893533	27, 84, 37, 27	6.55195, 40.13059, 21.89628, 12.61865	2.81093, 6.21495, 6.94731, 4.90765	4.06472, 3	3	AMPH	57833
GL173120.1	891817	893622	893385, 892089, 892045, 893533	60, 84, 37, 27	29.0052, 40.13059, 21.89628, 12.61865	6.01987, 6.21495, 6.94731, 4.90765	25.52123,	3	AMPH	57833
GL173120.1	894832	895219	894980, 895042, 895057	38, 49, 27	13.16706, 15.28841, 13.50887	3.97354, 3.7758, 5.268	10.22309,	3	AMPH	60848
GL173120.1	895418	897531	895633, 896492, 895672, 896989	25, 50, 21, 16	5.62307, 15.20995, 9.01339, 6.0555	2.6283, 3.70589, 4.2032, 3.45189	3.24482, 1	3	AMPH	61434
GL173120.1	895537	897531	896050, 896492, 895672, 896989	22, 50, 21, 16	4.26993, 15.20995, 9.01339, 6.0555	2.33112, 3.70589, 4.2032, 3.45189	2.09564, 1	3	AMPH	61553
GL173120.1	895537	897547	896842, 896492, 895672, 896989	23, 50, 21, 16	4.84551, 15.20995, 9.01339, 6.0555	2.47563, 3.70589, 4.2032, 3.45189	2.53536, 1	3	AMPH	61553
GL173120.1	899051	901035	900094, 899063, 900096, 900086, 900885	22, 23, 32, 12, 13	10.22595, 6.44387, 13.99003, 6.64075, 9.01977	4.60096, 3.01176, 4.78584, 4.308, 5.56237	7.44942, 4	3	AMPH	65067
GL173120.1	899933	901035	900904, 900096, 900086, 900885	13, 32, 12, 13	5.19589, 13.99003, 6.64075, 9.01977	3.3546, 4.78584, 4.308, 5.56237	2.85838, 1	3	AMPH	65949
GL173121.1	430520	430983	430732, 430693, 430901	7, 7, 7	6.69449, 5.97725, 8.49722	5.04797, 4.62287, 5.97582	4.1953, 3.6	3	stac3	0
GL173121.1	736976	737449	737157, 737184, 737169	8, 9, 4	8.42257, 6.9998, 4.67325	5.97763, 5.02958, 3.78987	5.76336, 4	3	gli1	10717
GL173121.1	774566	774950	774744, 774623, 774655	5, 8, 6	4.73273, 7.07159, 6.82211	3.93547, 5.20073, 5.08889	2.49377, 4	3	gli1	18280
GL173121.1	844316	844762	844625, 844734, 844732	11, 18, 6	10.78278, 17.32425, 7.29403	7.03733, 9.39518, 5.30581	7.97565, 1	3	arhgap9	0
GL173122.1	632496	634920	634611, 634155, 634639	27, 23, 43	23.78293, 18.61395, 43.98995	10.39276, 8.68467, 15.73264	20.43599,	3	ralgapa2	37485
GL173122.1	648895	649426	649072, 649336, 649178	10, 6, 7	11.06011, 4.93312, 7.96278	7.306, 4.04501, 5.72394	8.22712, 2	3	ralgapa2	22979
GL173122.1	656095	656805	656775, 656736, 656659	12, 16, 12	13.8483, 16.59459, 13.7434	8.63436, 9.48505, 8.57827	10.86694,	3	ralgapa2	15600
GL173122.1	884093	884653	884296, 884369, 884355	14, 15, 8	15.40455, 14.65124, 10.1465	9.21493, 8.56088, 6.82176	12.35264,	3	insm1	26449
GL173122.1	889314	890486	889986, 889819, 890331, 889978	12, 6, 6, 9	12.82537, 4.93312, 4.93312, 10.28145	8.07156, 4.04501, 4.04501, 6.93568	9.89694, 2	3	insm1	31670
GL173123.1	227371	229080	227811, 228507, 228971	12, 18, 11	10.39353, 13.81765, 11.20072	6.62187, 7.27929, 7.28154	7.60856, 1	3	mettl22	0
GL173123.1	886705	886980	886875, 886846, 886853	28, 48, 21	23.18557, 37.17689, 18.32862	9.73814, 10.70313, 9.11417	19.85583,	3	c16orf72	273724
GL173123.1	889673	891487	890574, 890576, 890559	30, 51, 28	26.71605, 43.43671, 28.08851	11.04782, 12.57528, 12.55014	23.28785,	3	c16orf72	276692
GL173124.1	112858	113620	113087, 113129, 113128	25, 33, 21	28.34522, 34.56012, 25.2565	13.78731, 14.44003, 13.23126	24.87704,	3	csnk2b	22798
GL173124.1	213721	214295	213926, 213830, 214199	6, 10, 4	5.96715, 9.39128, 4.67325	4.64927, 6.35644, 3.78987	3.52158, 6	3	abhd16a	23453
GL173125.1	71650	72016	71711, 71675, 71757	8, 14, 8	7.33538, 12.43922, 9.31951	5.35908, 7.44862, 6.43944	4.76518, 9	3	lpin1	28773
GL173125.1	195990	196807	196413, 196514, 196660	13, 13, 10	13.94585, 13.14514, 13.11399	8.5554, 8.09002, 8.30815	10.95967,	3	lpin1	66008
GL173125.1	377122	377427	377253, 377262, 377253	330, 378, 304	399.90759, 455.03528, 382.75778	37.57639, 37.56166, 40.93648	394.47562	3	NA	89244
GL173125.1	387094	388069	387533, 387919, 387160	11, 15, 7	10.84329, 13.80611, 7.34973	7.073, 8.04733, 5.41038	8.0324, 11	3	NA	78602
GL173125.1	723345	723788	723438, 723355, 723392	13, 24, 4	14.0251, 26.19212, 4.34116	8.6006, 12.84924, 3.63492	11.03614,	3	U2	19527
GL173125.1	823529	823794	823569, 823591, 823564	10, 8, 6	9.28413, 6.05185, 6.82211	6.29221, 4.56575, 5.08889	6.574, 3.72	3	fam84a	3586
GL173125.1	897583	898022	897963, 897709, 897978	9, 13, 16	6.96255, 8.6375, 17.11217	5.00603, 5.3289, 9.79056	4.44307, 6	3	fam84a	77640
GL173125.1	899874	900242	900178, 900182, 900202	13, 20, 12	11.55805, 15.38682, 11.81848	7.13125, 7.67002, 7.4869	8.70034, 1	3	fam84a	79931
GL173126.1	156333	157223	156464, 156982, 156463	11, 9, 18	11.71035, 6.93223, 22.65197	7.57196, 4.98685, 12.47685	8.8442, 4.5	3	NA	0
GL173126.1	638514	639423	638910, 638936, 639337	9, 10, 9	6.63709, 6.28381, 7.75727	4.79941, 4.41612, 5.50247	4.14229, 3	3	NA	0
GL173126.1	641192	641997	641662, 641275, 641744	11, 14, 9	8.2278, 9.79262, 7.83046	5.466, 5.82283, 5.54742	5.59878, 7	3	NA	0
GL173126.1	676903	677780	677184, 677310, 677395	12, 15, 7	10.68367, 14.15433, 8.69489	6.80062, 8.25968, 6.06379	7.88119, 1	3	NA	25528
GL173127.1	18089	18546	18400, 18384, 18438	8, 11, 6	7.57423, 8.44639, 6.168	5.4999, 5.60333, 4.75778	4.98865, 5	3	wdr89	0
GL173128.1	882203	882492	882263, 882260, 882253	10, 9, 9	11.06011, 8.21099, 11.64359	7.306, 5.77858, 7.57973	8.22712, 5	3	laptm5	164146
GL173130.1	2373	3027	2996, 2996, 2972	22, 39, 21	25.40151, 44.64822, 27.73099	13.02826, 17.82678, 14.58796	22.00933,	3	npr2	0
GL173130.1	13264	13774	13473, 13419, 13437	13, 10, 12	12.68462, 8.16488, 13.51536	7.81602, 5.60458, 8.45502	9.76298, 5	3	npr2	0
GL173130.1	23992	26230	25175, 24218, 25625	7, 9, 7	5.65516, 7.13885, 7.50542	4.42382, 5.11729, 5.49244	3.27228, 4	3	npr2	3308
GL173130.1	51390	52007	51625, 51467, 51660	14, 29, 13	16.32935, 34.13175, 16.70862	9.72892, 15.76422, 10.0169	13.23998,	3	acsl5	0
GL173130.1	107884	108255	108033, 108133, 108022	5, 10, 6	4.82177, 9.39128, 5.8719	3.98509, 6.35644, 4.59674	2.51418, 6	3	NA	0
GL173130.1	133572	133976	133776, 133755, 133668	10, 13, 10	10.6018, 13.14514, 12.90749	7.05583, 8.09002, 8.21675	7.80484, 1	3	NA	0
GL173130.1	150136	150562	150238, 150193, 150263	11, 11, 7	11.15818, 9.80774, 8.42533	7.25692, 6.45006, 5.94313	8.32008, 7	3	NA	0
GL173130.1	164279	164596	164377, 164425, 164424	5, 9, 8	4.82177, 8.21099, 10.1465	3.98509, 5.77858, 6.82176	2.51418, 5	3	NA	0
GL173130.1	227559	227876	227795, 227833, 227782	17, 19, 15	14.64358, 16.78404, 17.23823	8.0415, 8.78005, 10.06864	11.62752,	3	NA	51550
GL173130.1	245566	246070	245600, 245584, 245608	14, 13, 12	13.57329, 10.2718, 14.96175	8.13982, 6.33559, 9.20442	10.60942,	3	tcf7l2	44558
GL173130.1	272717	273511	272970, 273152, 272937	13, 26, 22	10.29845, 21.20893, 23.54667	6.35212, 9.2566, 11.93057	7.51866, 1	3	tcf7l2	17117
GL173130.1	300150	300574	300402, 300414, 300313	21, 23, 10	22.94132, 21.38155, 10.84317	11.88295, 10.30482, 7.18868	19.61877,	3	tcf7l2	0
GL173130.1	308183	308807	308189, 308193, 308248	12, 16, 12	11.61187, 15.16461, 14.23156	7.3638, 8.6242, 8.83588	8.75096, 1	3	tcf7l2	0
GL173130.1	320175	320660	320361, 320383, 320461	7, 15, 9	5.42862, 12.44573, 8.74593	4.2813, 7.21287, 6.09519	3.06982, 9	3	tcf7l2	0
GL173130.1	324058	324833	324318, 324317, 324175	10, 12, 6	7.31658, 8.9579, 4.96699	5.07009, 5.72845, 4.06589	4.74811, 6	3	tcf7l2	0
GL173130.1	351176	351618	351293, 351517, 351489	10, 13, 11	8.84451, 10.00327, 12.63525	6.02544, 6.16911, 8.07536	6.16137, 7	3	tcf7l2	0
GL173130.1	358883	359618	358974, 359264, 359153	10, 8, 11	8.48936, 5.93025, 12.48556	5.80661, 4.48816, 7.99611	5.82603, 3	3	tcf7l2	0
GL173130.1	371766	372771	372588, 372231, 372067	8, 7, 11	6.70593, 4.92115, 11.6739	4.9768, 3.95586, 7.55148	4.20487, 2	3	tcf7l2	0
GL173130.1	443232	444029	443839, 443926, 443943	16, 31, 15	13.19919, 27.59813, 14.54087	7.42362, 11.19976, 8.49419	10.2543, 2	3	tcf7l2	18647
GL173130.1	448099	448583	448537, 448522, 448386	15, 24, 8	13.05189, 19.96444, 6.83213	7.58513, 9.15947, 5.05462	10.11376,	3	tcf7l2	23514
GL173130.1	460026	460498	460178, 460375, 460232	10, 17, 9	7.31658, 12.85839, 9.19414	5.07009, 6.96436, 6.35176	4.74811, 1	3	tcf7l2	35441
GL173130.1	463437	464577	463968, 464039, 464250	19, 31, 9	17.32971, 28.15182, 8.84115	9.11001, 11.5086, 6.1504	14.19806,	3	tcf7l2	38852
GL173130.1	486533	487035	486810, 486883, 486562	8, 13, 6	7.42892, 12.80191, 6.25896	5.41453, 7.88619, 4.80589	4.85345, 1	3	habp2	37296
GL173130.1	495006	495487	495421, 495432, 495447	14, 21, 7	16.23025, 22.57492, 8.69489	9.67496, 11.66487, 6.06379	13.14538,	3	habp2	28844
GL173130.1	508370	508717	508665, 508649, 508692	11, 17, 7	9.26086, 12.63113, 6.08239	6.1125, 6.8293, 4.68683	6.5517, 9.8	3	habp2	15614
GL173130.1	509644	510073	509690, 509675, 509647	8, 21, 9	5.89711, 17.35787, 8.01994	4.46697, 8.54015, 5.66309	3.49291, 1	3	habp2	14258
GL173130.1	540724	541354	540847, 540847, 541038	12, 19, 11	11.32055, 17.06093, 12.48556	7.18871, 8.94736, 7.99611	8.47391, 1	3	habp2	0
GL173130.1	569965	570529	570280, 570280, 570279	17, 16, 15	17.48974, 15.35105, 17.94632	9.76779, 8.73748, 10.45626	14.35103,	3	NRAP	0
GL173130.1	589002	589386	589258, 589274, 589272	44, 68, 30	54.37297, 82.9937, 38.61061	21.24657, 25.51911, 18.0061	50.45813,	3	NRAP	3265
GL173130.1	602769	603683	602907, 602903, 602898	7, 9, 6	6.42049, 6.37567, 5.0826	4.8888, 4.63258, 4.13667	3.94161, 4	3	arhgef5	0
GL173130.1	616419	617061	616638, 616761, 616915	23, 22, 24	20.10283, 16.70948, 22.85773	9.54811, 7.88525, 10.85367	16.86916,	3	NA	1772
GL173130.1	637446	638407	637871, 637840, 638117	23, 45, 14	28.23409, 51.41798, 17.61756	14.36443, 19.05392, 10.40352	24.76954,	3	mxi1	5846
GL173130.1	644514	645204	644543, 645084, 644925	14, 22, 14	16.6367, 25.8245, 19.68329	9.89449, 13.2744, 11.3696	13.53421,	3	mxi1	0
GL173130.1	663180	663697	663526, 663531, 663395	12, 15, 13	10.34679, 13.69427, 15.34872	6.59299, 7.97895, 9.3288	7.56382, 1	3	mxi1	7445
GL173130.1	681717	682175	681976, 681834, 681935	12, 7, 5	12.60209, 5.97725, 5.9506	7.9443, 4.62287, 4.54784	9.68421, 3	3	mxi1	25982
GL173130.1	690798	691543	691177, 691418, 690965	5, 7, 5	4.33555, 5.55323, 5.69847	3.70555, 4.35994, 4.4331	2.15195, 3	3	dusp5	18603
GL173130.1	697027	697635	697369, 697366, 697180	10, 13, 5	11.06011, 13.14514, 5.9506	7.306, 8.09002, 4.54784	8.22712, 1	3	dusp5	12511
GL173130.1	707803	708368	707944, 708037, 708041	6, 12, 6	5.96715, 11.86108, 7.05929	4.64927, 7.51216, 5.20024	3.52158, 9	3	dusp5	1778
GL173130.1	754485	755207	754945, 754527, 754931	10, 6, 8	8.70772, 4.93312, 10.1465	5.94148, 4.04501, 6.82176	6.03286, 2	3	smc3	8978
GL173130.1	759791	760190	759878, 760113, 759923	4, 6, 5	3.74195, 4.93312, 5.9506	3.32091, 4.04501, 4.54784	1.6222, 2.7	3	smc3	14284
GL173130.1	771158	771885	771623, 771492, 771493	8, 20, 8	6.86256, 18.9091, 7.46678	5.0733, 9.76697, 5.43686	4.3506, 15	3	smc3	25651
GL173130.1	776132	776696	776658, 776660, 776628	6, 12, 5	5.86227, 11.66217, 5.54867	4.59138, 7.39386, 4.3619	3.46055, 8	3	smc3	30625
GL173130.1	799412	799907	799704, 799779, 799817	9, 17, 6	7.88835, 13.76564, 6.71074	5.58287, 7.50888, 5.03498	5.28191, 1	3	NA	10208
GL173130.1	813648	814256	814194, 814135, 813896	8, 14, 7	7.33538, 12.54273, 7.55909	5.35908, 7.51218, 5.52035	4.76518, 9	3	NA	0
GL173130.1	832412	834372	833115, 833907, 832967	16, 25, 14	12.71534, 20.68479, 13.875	7.12912, 9.26557, 8.32113	9.79248, 1	3	rnf115	0
GL173130.1	832509	834372	833913, 833907, 832967	13, 25, 14	9.84003, 20.68479, 13.875	6.06799, 9.26557, 8.32113	7.08478, 1	3	rnf115	0
GL173130.1	872184	872915	872484, 872468, 872267	7, 14, 6	6.16939, 13.50676, 7.29403	4.73936, 8.09967, 5.30581	3.70916, 1	3	pias3	0
GL173131.1	735413	735952	735716, 735603, 735857	12, 15, 7	11.61187, 12.72276, 8.35502	7.3638, 7.383, 5.9108	8.75096, 9	3	b3gnt2	7510
GL173132.1	43901	44679	44153, 44115, 43940, 44501	9, 8, 8, 4	9.72082, 6.89656, 8.14528, 3.64768	6.64182, 5.09414, 5.82558, 3.26758	6.97155, 4	3	efr3a	0
GL173132.1	153214	153871	153760, 153818, 153810	5, 12, 9	4.77263, 10.83413, 11.58206	3.9578, 6.89288, 7.55286	2.51418, 8	3	NA	38399
GL173132.1	384918	385701	385143, 384990, 385360	14, 14, 6	13.32664, 10.50512, 5.71639	7.99066, 6.2574, 4.50951	10.37457,	3	lrrc6	12222
GL173132.1	420250	420698	420421, 420501, 420533	5, 11, 5	4.47183, 8.30667, 4.77515	3.78598, 5.51556, 3.9592	2.26935, 5	3	phf20l1	0
GL173132.1	447054	447454	447156, 447365, 447155	9, 14, 22	8.55802, 10.50512, 26.17114	5.98518, 6.2574, 13.47463	5.8909, 7.8	3	phf20l1	0
GL173132.1	681745	682356	681837, 681859, 681905	20, 16, 6	23.85765, 15.80758, 7.18591	12.69961, 9.01376, 5.25776	20.50816,	3	tg	23040
GL173132.1	714128	714493	714438, 714453, 714448	13, 20, 12	13.20994, 19.93044, 15.5838	8.12823, 10.38414, 9.50165	10.26285,	3	wisp1	16031
GL173132.1	765935	766548	765978, 766476, 766032	5, 8, 5	4.82177, 6.11443, 5.9506	3.98509, 4.60556, 4.54784	2.51418, 3	3	ndrg1	6161
GL173132.1	803144	803559	803530, 803467, 803441	9, 11, 8	9.12009, 10.05535, 8.04677	6.30997, 6.6012, 5.77057	6.42028, 7	3	st3gal1	25392
GL173133.1	530534	531340	531003, 530995, 531014	11, 14, 4	12.33515, 14.09002, 4.67325	7.91559, 8.44949, 3.78987	9.43699, 1	3	NA	116896

GL173133.1	591000	591549	591473, 591474, 591515	19, 15, 10	21.91879, 13.16372, 12.52085	11.84839, 7.65379, 8.03986	18.62754,	3	NA	177362
GL173134.1	52098	52558	52283, 52453, 52381	7, 7, 5	7.1696, 5.97725, 5.9506	5.31345, 4.62287, 4.54784	4.61046, 3	3	wdr11	15373
GL173134.1	76696	76997	76891, 76987, 76986	19, 36, 18	19.64557, 37.35154, 22.99911	10.50977, 14.85422, 12.66051	16.42918,	3	wdr11	39971
GL173134.1	151046	151339	151084, 151079, 151071	9, 19, 5	9.36986, 20.1301, 5.50079	6.44997, 10.79955, 4.33868	6.65425, 1	3	fgfr2	54292
GL173134.1	156522	156925	156674, 156741, 156553	11, 27, 11	10.21742, 29.84144, 11.79996	6.69945, 13.96317, 7.62212	7.44132, 2	3	fgfr2	48706
GL173134.1	282083	282594	282119, 282144, 282112	22, 38, 12	22.85143, 42.80689, 14.40488	11.51387, 17.18536, 8.92522	19.53157,	3	fgfr2	10640
GL173134.1	372125	372666	372594, 372576, 372526	5, 10, 15	4.61714, 9.07627, 17.94632	3.86998, 6.1667, 10.45626	2.39372, 6	3	nsmce4a	2959
GL173134.1	585251	585581	585350, 585350, 585350	12, 8, 6	13.55951, 6.89656, 6.87953	8.47903, 5.09414, 5.11627	10.59689,	3	htra1	0
GL173134.1	768521	768985	768953, 768953, 768932	19, 31, 17	23.04311, 33.4732, 23.17908	12.48444, 14.59388, 12.87887	19.71723,	3	styk1	0
GL173135.1	7177	7775	7365, 7457, 7724	14, 9, 7	9.9642, 3.82874, 5.88867	5.92701, 3.02231, 4.56857	7.20203, 1	3	NA	45123
GL173135.1	17614	18451	18265, 18353, 18283	19, 24, 14	14.83805, 15.46834, 11.02224	7.61793, 6.72468, 6.57526	11.81382,	3	NA	34447
GL173135.1	101850	102411	102072, 102355, 102014	7, 8, 4	6.50909, 6.62693, 4.46488	4.94073, 4.92781, 3.69425	4.02371, 4	3	NA	11849
GL173135.1	124638	125244	124841, 125032, 124888	9, 22, 9	7.47395, 18.62484, 8.05901	5.32711, 8.98779, 5.6868	4.89626, 1	3	NA	0
GL173135.1	636078	636854	636568, 636641, 636596	10, 16, 9	8.08479, 12.6954, 10.14484	5.55446, 7.11701, 6.86555	5.46601, 9	3	rad50	54341
GL173135.1	828040	828951	828344, 828589, 828371	14, 14, 17	15.75768, 12.70158, 20.37708	9.41387, 7.60958, 11.43318	12.69046,	3	p4ha2	0
GL173135.1	880819	881037	880872, 880897, 880986	9, 29, 5	9.72082, 31.75332, 5.9506	6.64182, 14.35085, 4.54784	6.97155, 2	3	p4ha2	39713
GL173136.1	105695	106177	105781, 105902, 105836	6, 8, 5	5.06496, 6.73981, 5.9506	4.12592, 4.99774, 4.54784	2.73812, 4	3	ado	63016
GL173136.1	254194	254649	254254, 254220, 254232	10, 24, 24	10.6018, 22.07986, 31.72657	7.05583, 10.39207, 16.02936	7.80484, 1	3	rtkn2	0
GL173136.1	600366	600593	600385, 600390, 600425	6, 17, 7	5.96715, 18.56115, 8.646	4.64927, 10.40145, 6.04229	3.52158, 1	3	arid5b	90631
GL173136.1	606748	607392	607084, 606989, 606940	34, 45, 38	34.10667, 46.88007, 42.46649	13.78286, 16.49307, 16.98603	30.51164,	3	arid5b	97013
GL173136.1	749447	750342	749990, 749947, 750116	18, 35, 11	20.86369, 35.53384, 12.48556	11.49013, 14.20975, 7.99611	17.60698,	3	arid5b	239712
GL173136.1	763376	763858	763411, 763473, 763417	7, 6, 4	7.1696, 4.93312, 4.67325	5.31345, 4.04501, 3.78987	4.61046, 2	3	arid5b	253641
GL173136.1	823842	825403	825142, 825118, 825141	13, 30, 13	11.98086, 29.76648, 13.58227	7.39024, 12.79553, 8.34597	9.10256, 2	3	arid5b	314107
GL173136.1	845181	845730	845595, 845495, 845392	8, 8, 5	6.20993, 5.14361, 4.22765	4.66614, 3.9808, 3.64077	3.74679, 2	3	arid5b	335446
GL173136.1	857274	857820	857485, 857485, 857533	10, 22, 11	11.06011, 25.85271, 14.75772	7.306, 13.29074, 9.09568	8.22712, 2	3	arid5b	347539
GL173137.1	148735	149811	149530, 149095, 149234	40, 46, 23	37.07819, 39.45021, 21.85729	13.15967, 12.30404, 10.58878	33.42529,	3	spint1	22098
GL173137.1	597395	598051	597942, 597623, 597699	10, 11, 7	9.33595, 8.77819, 7.29952	6.32332, 5.81139, 5.38357	6.62299, 6	3	chp1	7138
GL173137.1	755612	756564	756263, 756262, 756178	13, 16, 8	13.20994, 15.67391, 8.6841	8.12823, 8.93306, 6.11715	10.26285,	3	NA	6797
GL173138.1	212498	213216	212836, 212673, 212795	14, 25, 11	14.45502, 23.9492, 11.5511	8.66555, 11.16355, 7.48213	11.4469, 2	3	kif1b	0
GL173139.1	652	879	682, 689, 798	13, 13, 7	15.29169, 13.14514, 8.35502	9.29854, 8.09002, 5.9108	12.24396,	3	nrp1	289893
GL173139.1	108327	109038	108470, 108644, 108768	4, 6, 4	3.74195, 4.93312, 4.30163	3.32091, 4.04501, 3.61556	1.6222, 2.7	3	nrp1	181734
GL173139.1	283466	283861	283834, 283535, 283638	9, 10, 5	9.63662, 8.28331, 5.9506	6.59633, 5.67853, 4.54784	6.90556, 5	3	nrp1	6911
GL173139.1	374497	374699	374649, 374671, 374668	12, 19, 10	13.64785, 18.6168, 12.16489	8.52686, 9.88966, 7.87042	10.68062,	3	nrp1	0
GL173139.1	690260	691188	690716, 690877, 690798	12, 17, 8	10.73374, 13.5057, 7.59222	6.83135, 7.35211, 5.5104	7.92922, 1	3	NA	3408
GL173139.1	741366	741931	741883, 741629, 741631	9, 10, 6	8.20841, 8.66076, 6.25896	5.77703, 5.91256, 4.80589	5.58112, 6	3	zeb1	1563
GL173140.1	114032	114474	114426, 114337, 114449	10, 13, 14	9.54985, 11.81904, 16.46576	6.45088, 7.29135, 9.80275	6.82341, 9	3	grin2c	0
GL173140.1	121002	121706	121219, 121052, 121100	11, 22, 11	10.66406, 21.28482, 12.78973	6.96705, 10.5715, 8.15619	7.86396, 1	3	grin2c	0
GL173140.1	163534	164060	163793, 163923, 163906	13, 11, 7	13.63968, 9.4823, 6.63036	8.37925, 6.24973, 5.0111	10.67258,	3	tmem104	0
GL173140.1	231918	232615	232564, 232565, 232586	34, 23, 19	39.2513, 20.1396, 23.01756	16.79793, 9.56971, 12.47022	35.56108,	3	slc9a3r1	6126
GL173140.1	284552	285308	285144, 285077, 284725	5, 8, 7	3.81096, 4.96414, 7.10639	3.3822, 3.86431, 5.27894	1.68502, 2	3	xylt2	6759
GL173140.1	339670	340564	339737, 339843, 339744	20, 28, 15	19.99469, 25.74967, 14.76444	10.42292, 11.1835, 8.62913	16.76522,	3	acsf2	0
GL173140.1	342279	342565	342306, 342318, 342306	10, 23, 16	7.86015, 19.55015, 15.59631	5.41341, 9.225, 8.88613	5.25709, 1	3	acsf2	0
GL173140.1	386478	386889	386861, 386861, 386782	46, 35, 14	56.27795, 37.39783, 16.91966	21.40851, 15.28944, 10.04443	52.33826,	3	cacna1g	0
GL173140.1	486120	487651	486450, 486680, 486681	11, 24, 15	9.34791, 21.41819, 16.23099	6.16652, 10.00243, 9.49656	6.63394, 1	3	spata20	64596
GL173140.1	491918	492256	492157, 491989, 491935	5, 15, 9	4.33555, 12.44573, 9.14171	3.70555, 7.21287, 6.32219	2.15195, 9	3	spata20	59991
GL173140.1	496919	497349	497146, 496989, 497230	5, 6, 4	4.02828, 3.95542, 4.04585	3.51866, 3.41818, 3.48564	1.88216, 1	3	spata20	54898
GL173140.1	656697	657122	656840, 656839, 656839	13, 19, 9	12.81131, 17.52577, 10.56949	7.8918, 9.22875, 7.08032	9.88323, 1	3	NA	3751
GL173140.1	678389	678810	678567, 678651, 678622	13, 10, 5	13.63968, 9.028, 5.14967	8.37925, 6.13739, 4.16141	10.67258,	3	ndrg4	0
GL173140.1	722353	722993	722690, 722715, 722580	14, 11, 8	15.15267, 10.60895, 10.1465	9.07115, 6.9343, 6.82176	12.11465,	3	sla2	1687
GL173141.1	80343	80760	80364, 80402, 80394	19, 18, 9	20.05161, 18.3735, 10.21256	10.75274, 10.0289, 6.90044	16.81949,	3	trpc4	7734
GL173141.1	292157	292530	292400, 292379, 292394	26, 10, 9	26.12618, 8.79469, 10.72164	12.10877, 5.99491, 7.15493	22.71324,	3	supt20h	22837
GL173142.1	510453	511622	511163, 511167, 511040	19, 29, 11	19.64557, 28.27962, 12.13051	10.50977, 12.2963, 7.80464	16.42918,	3	hnf4g	214491
GL173143.1	411183	411562	411294, 411218, 411514	11, 8, 8	10.00935, 7.07159, 7.95096	6.57321, 5.20073, 5.71659	7.24529, 4	3	wnk3	0
GL173143.1	742981	744534	743554, 743390, 743794	37, 54, 26	32.74773, 44.23142, 23.94847	11.93325, 12.03612, 10.82241	29.18004,	3	pfkfb1	0
GL173143.1	762364	762792	762670, 762481, 762633	5, 9, 12	3.25091, 6.46255, 12.88563	3.01661, 4.68809, 8.10565	1.34649, 4	3	pfkfb1	6728
GL173143.1	793077	793986	793898, 793516, 793554	10, 15, 7	7.25363, 9.8904, 6.36213	5.03021, 5.66748, 4.85436	4.68848, 7	3	pfkfb1	37441
GL173143.1	801776	803116	802158, 802053, 802759	11, 11, 7	5.51277, 6.56017, 5.29734	3.78886, 4.42417, 4.19787	3.14574, 4	3	pfkfb1	46140
GL173143.1	818872	820622	819129, 819187, 819906, 819300, 820549	16, 19, 13, 14, 8	13.01894, 13.77548, 7.73433, 11.60289, 5.37675	7.31376, 6.99985, 4.78574, 6.93334, 4.13188	10.08178,	3	pfkfb1	63236
GL173143.1	835306	836532	835784, 835789, 835784	13, 23, 13	7.68437, 13.36183, 9.12714	4.75611, 5.8889, 5.62823	5.09175, 1	3	pfkfb1	79670
GL173143.1	838924	839929	839509, 839366, 838952, 839727	12, 19, 12, 12	5.83141, 8.308, 7.14709, 7.16705	3.83393, 4.14494, 4.61185, 4.62393	3.43207, 5	3	pfkfb1	83288
GL173143.1	852968	854027	853830, 853874, 853873	67, 98, 54	60.61982, 88.98412, 56.45384	14.99592, 16.5212, 17.83447	56.6253, 8	3	pfkfb1	97332
GL173143.1	865019	866523	865335, 865750, 865166, 866379	84, 107, 49, 49	19.96759, 21.65768, 12.25672, 12.37278	3.22549, 2.9329, 3.16584, 3.18814	16.73974,	3	pfkfb1	109383
GL173143.1	866279	869233	867974, 868033, 866379, 867916, 868997	310, 455, 49, 199, 33	216.5731, 323.13004, 12.37278, 147.87688, 4.94897	11.84015, 12.39197, 3.18814, 12.75255, 2.16895	211.50417	3	pfkfb1	110643
GL173144.1	51395	51898	51732, 51599, 51519	6, 6, 3	5.95712, 4.93312, 3.47371	4.64377, 4.04501, 3.03189	3.52158, 2	3	NA	8101
GL173144.1	74675	75579	75304, 75345, 75314	36, 54, 36	34.9986, 51.47768, 38.91515	13.51755, 15.32092, 15.76111	31.38561,	3	ublcp1	1310
GL173144.1	293659	294246	294025, 293785, 293857	13, 17, 12	11.16633, 12.78176, 12.69164	6.8898, 6.91875, 7.99552	8.32764, 1	3	NA	140516
GL173144.1	318910	319322	319138, 319079, 319160	6, 10, 4	5.96715, 8.24345, 4.67325	4.64927, 5.65367, 3.78987	3.52158, 5	3	NA	115440
GL173144.1	500849	501231	501089, 501059, 501131	9, 25, 8	7.71701, 25.75015, 9.60885	5.47767, 12.23791, 6.57807	5.12244, 2	3	clint1	52173
GL173144.1	620643	620983	620890, 620827, 620833	7, 12, 6	6.79162, 11.84549, 6.87953	5.10336, 7.50292, 5.11627	4.28406, 9	3	adam19	0
GL173144.1	679748	680090	679782, 679822, 679770	8, 10, 7	6.27633, 7.55261, 7.05993	4.70812, 5.21944, 5.25341	3.80887, 5	3	cyfip2	0
GL173144.1	764798	765116	764878, 765017, 765017	11, 14, 5	9.21796, 11.03811, 4.39541	6.08584, 6.58503, 3.74107	6.51275, 8	3	ITK	0
GL173144.1	791283	792696	791798, 791978, 791772	4, 10, 3	3.74195, 9.07627, 3.47371	3.32091, 6.1667, 3.03189	1.6222, 6.5	3	med7	7063
GL173146.1	853095	853813	853412, 853396, 853330	19, 29, 15	20.74745, 28.96045, 14.32502	11.16575, 12.69527, 8.36342	17.49384,	3	cd226	114945
GL173147.1	451	2144	914, 967, 905	9, 12, 8	7.59348, 8.05278, 7.90404	5.40134, 5.16604, 5.68997	5.0069, 5.5	3	btbd1	11806
GL173147.1	5451	7193	6195, 6269, 6941	7, 12, 6	4.55459, 6.71984, 4.96699	3.7168, 4.35512, 4.06589	2.34094, 4	3	btbd1	6757
GL173147.1	133682	134188	133883, 133888, 133896	7, 7, 4	7.10297, 5.97725, 4.38152	5.27706, 4.62287, 3.65448	4.57191, 3	3	homer2	0
GL173147.1	515790	516167	515822, 515847, 515845	9, 8, 5	8.40425, 6.62693, 5.9506	5.89416, 4.92781, 4.54784	5.76336, 4	3	usp8	6380
GL173147.1	755704	756232	755777, 756014, 756013	7, 11, 10	6.50909, 8.04011, 9.73055	4.94073, 5.34803, 6.55755	4.02371, 5	3	nmrk2	801
GL173149.1	153055	154382	154031, 153987, 154010	9, 14, 5	8.66407, 12.28739, 5.9506	6.04743, 7.35528, 4.54784	5.99101, 9	3	NA	85894
GL173149.1	850904	852026	851244, 851701, 851371	29, 30, 17	30.96647, 31.06513, 18.42656	13.88253, 13.55836, 10.3227	27.4375, 2	3	mdm4	110754
GL173151.1	22	635	330, 377, 372	13, 36, 15	15.29169, 44.43662, 18.95532	9.29854, 19.01688, 10.98488	12.24396,	3	mapre1	36537
GL173151.1	91741	93244	92100, 92725, 92083	14, 13, 5	14.23954, 11.98001, 5.9506	8.53829, 7.38972, 4.54784	11.24094,	3	NA	10173
GL173151.1	133304	133702	133541, 133570, 133490	11, 9, 8	10.5483, 6.8008, 8.04677	6.89817, 4.90352, 5.77057	7.75329, 4	3	kif3b	705
GL173151.1	153530	154293	153785, 153858, 153968	7, 20, 10	5.10557, 16.07543, 9.68182	4.075, 8.07188, 6.52889	2.77482, 1	3	TM9SF4	0
GL173151.1	157903	158299	158020, 158020, 158020	13, 16, 8	11.60911, 12.53032, 7.67847	7.16262, 7.01691, 5.56054	8.74856, 9	3	TM9SF4	2393
GL173151.1	211455	212001	211948, 211859, 211836	18, 20, 7	22.92104, 22.87459, 8.69489	12.61945, 12.13503, 6.06379	19.59912,	3	TM9SF4	55945
GL173151.1	244262	245277	245227, 244633, 245013	7, 14, 9	7.1696, 14.45897, 9.76105	5.31345, 8.66788, 6.66343	4.61046, 1	3	TM9SF4	88752
GL173151.1	300944	301536	301121, 301047, 301025	32, 36, 9	28.97187, 31.89001, 7.79368	11.61816, 11.82176, 5.52485	25.48871,	3	ZBTB20	36106
GL173151.1	368531	369052	368871, 368864, 368750	11, 16, 6	9.66657, 13.35594, 5.95321	6.36338, 7.51928, 4.64162	6.93336, 1	3	ZBTB20	0
GL173151.1	373717	374012	373848, 373908, 373820	11, 14, 6	10.66406, 11.85481, 6.2131	6.96705, 7.08876, 4.78171	7.86396, 9	3	ZBTB20	0
GL173151.1	385281	385862	385609, 385641, 385599	19, 10, 11	21.40997, 7.38914, 10.33022	11.55406, 5.11603, 6.76749	18.13483,	3	ZBTB20	0
GL173151.1	394212	394565	394438, 394282, 394266	16, 18, 6	17.50585, 17.86551, 6.6567	10.02008, 9.72285, 5.00845	14.36688,	3	ZBTB20	0
GL173151.1	405338	405896	405359, 405367, 405402	14, 19, 16	13.63662, 18.21411, 17.55092	8.17799, 9.64585, 10.04619	10.67031,	3	ZBTB20	0
GL173151.1	453646	454430	453905, 454176, 454127	6, 7, 5	5.55471, 5.02593, 5.14967	4.41698, 4.02367, 4.16141	3.18192, 2	3	ZBTB20	0
GL173151.1	503545	504054	503570, 503599, 503575	21, 44, 15	19.27424, 38.94788, 14.01468	9.67974, 12.71637, 8.17463	16.06988,	3	ZBTB20	0
GL173151.1	524137	524844	524420, 524558, 524396	11, 16, 10	9.13339, 13.3097, 9.44624	6.03321, 7.49106, 6.38926	6.43178, 1	3	ZBTB20	0
GL173151.1	565812	567785	566662, 566657, 566698	23, 31, 16	23.48681, 29.90493, 17.04172	11.56632, 12.50474, 9.74922	20.14878,	3	ZBTB20	0
GL173151.1	580669	581930	581185, 580853, 581631	17, 20, 13	15.11635, 16.87998, 13.45746	8.32952, 8.54771, 8.27333	12.08029,	3	ZBTB20	0
GL173151.1	617507	618014	617802, 617757, 617774	9, 6, 4	8.20841, 4.20515, 4.67325	5.77703, 3.5821, 3.78987	5.58112, 2	3	ZBTB20	0
GL173151.1	638723	639535	638824, 639206, 639366	9, 13, 7	8.45482, 10.7674, 7.01416	5.92419, 6.64292, 5.22813	5.79307, 8	3	ZBTB20	0
GL173151.1	661778	662097	662035, 661950, 662044	15, 13, 6	10.95062, 7.37317, 5.02414	6.30074, 4.57267, 4.10097	8.13456, 4	3	ZBTB20	0
GL173151.1	666152	667141	666823, 666624, 666548	45, 60, 18	46.24725, 60.10813, 19.30306	16.14612, 17.23774, 10.58333	42.44975,	3	ZBTB20	0
GL173151.1	676246	677153	676512, 676677, 677080	5, 12, 6	4.82177, 11.86108, 7.29403	3.98509, 7.51216, 5.30581	2.51418, 9	3	ZBTB20	0
GL173151.1	687991	688603	688293, 688276, 688184	9, 17, 5	8.77314, 16.17826, 5.9506	6.111, 8.97632, 4.54784	6.09355, 1	3	ZBTB20	0
GL173151.1	711873	712536	712251, 712251, 712279	17, 55, 16	17.64386, 63.58216, 19.35429	9.85981, 21.24671, 11.05657	14.49984,	3	ZBTB20	0
GL173151.1	736398	736798	736792, 736740, 736757	20, 23, 16	19.15409, 22.81409, 16.972	9.91507, 11.16226, 9.70822	15.95382,	3	ZBTB20	0
GL173151.1	751182	752089	751342, 751338, 751322	10, 13, 5	9.03421, 12.80191, 5.40789	6.14116, 7.88619, 4.29296	6.33915, 1	3	ZBTB20	0
GL173151.1	837502	838237	837976, 837688, 837772	11, 6, 5	9.81032, 4.379, 5.06966	6.45164, 3.69486, 4.11933	7.05644, 2	3	spice1	0
GL173153.1	236135	237199	236320, 236297, 236343	6, 14, 6	5.09949, 13.63145, 6.71074	4.14694, 8.17488, 5.03498	2.76939, 1	3	slit1	9819
GL173153.1	316995	317985	317209, 317257, 317851	14, 14, 13	15.31941, 12.59519, 16.0859	9.1665, 7.54437, 9.70995	12.27033,	3	NA	9347
GL173156.1	100839	101577	101047, 101528, 101021	7, 8, 4	5.65516, 6.66413, 4.46488	4.42382, 4.9509, 3.69425	3.27228, 4	3	icam5	0
GL173156.1	178457	179030	178890, 178751, 178749	12, 9, 9	12.82537, 8.21099, 10.87975	8.07156, 5.77858, 7.23113	9.89694, 5	3	tyk2	6186
GL173156.1	225698	226208	226004, 226161, 226159	14, 16, 8	13.09001, 14.09269, 9.60885	7.84687, 7.96975, 6.57807	10.14895,	3	cdc37	21936
GL173156.1	238336	239740	238842, 239469, 238523	6, 10, 6	5.3939, 9.39128, 6.35308	4.32314, 6.35644, 4.85498	3.0374, 6.8	3	cdc37	34574
GL173156.1	256286	257131	256711, 256806, 256921	7, 10, 14	5.4599, 7.90132, 13.93205	4.30109, 5.43931, 8.35526	3.09813, 5	3	cdc37	52524
GL173156.1	357278	357644	357539, 357614, 357616	10, 19, 12	9.49535, 18.8972, 14.67601	6.41851, 10.05916, 9.06267	6.77344, 1	3	pde4a	101388
GL173156.1	387906	388617	388303, 388497, 388191	4, 6, 5	3.74195, 4.92507, 5.9506	3.32091, 4.04003, 4.54784	1.6222, 2.7	3	pde4a	70415
GL173156.1	437941	438315	438111, 438189, 438132	4, 14, 5	3.74195, 13.0902, 5.69847	3.32091, 7.84698, 4.4331	1.6222, 10	3	pde4a	20717
GL173156.1	461599	461788	461657, 461660, 461647	16, 26, 11	12.8006, 21.8089, 10.05825	7.18091, 9.59281, 6.60296	9.87433, 1	3	pde4a	0
GL173156.1	483555	485183	484584, 484040, 484353	6, 15, 8	5.16999, 12.72276, 8.80246	4.18963, 7.383, 6.179	2.83325, 9	3	pde4a	0
GL173156.1	534151	534738	534714, 534634, 534701	75, 98, 59	88.91885, 116.72382, 73.63322	25.1574, 27.84196, 24.9329	84.63412,	3	keap1	0
GL173156.1	541947	542876	542513, 542545, 542559	11, 14, 6	10.1122, 12.1395, 5.91225	6.63573, 7.26424, 4.61907	7.34229, 9	3	keap1	0
GL173156.1	558015	558821	558302, 558134, 558073	18, 20, 30	18.37468, 18.17471, 36.8562	10.02961, 9.32366, 16.98881	15.20182,	3	s1pr5	4639
GL173156.1	593824	594428	594145, 594280, 594141	6, 11, 5	5.96715, 10.60895, 5.9506	4.64927, 6.9343, 4.54784	3.52158, 7	3	s1pr5	29209
GL173156.1	662132	662879	662434, 662362, 662315	9, 10, 5	8.45482, 8.36423, 5.3628	5.92419, 5.72892, 4.27046	5.79307, 5	3	kri1	0
GL173156.1	667963	668820	668249, 668394, 668269	5, 16, 6	4.43696, 14.86511, 6.99807	3.76555, 8.44177, 5.17195	2.24017, 1	3	kri1	0
GL173156.1	789619	790236	789892, 789718, 789863	13, 9, 4	15.29169, 8.21099, 4.67325	9.29854, 5.77858, 3.78987	12.24396,	3	ilf3	9282
GL173156.1	814416	815144	814561, 814560, 814710	14, 34, 8	14.67861, 37.2227, 8.9249	8.79666, 15.59843, 6.24211	11.66087,	3	ilf3	1386
GL173157.1	239077	239549	239514, 239498, 239480	18, 21, 16	22.23921, 24.35402, 23.11042	12.25494, 12.71289, 12.88555	18.93827,	3	NA	8037
GL173157.1	280415	281163	280854, 280954, 281103	15, 14, 9	15.41679, 14.45897, 10.56949	9.0192, 8.66788, 7.08032	12.36344,	3	NA	1805
GL173157.1	327318	327658	327448, 327435, 327465	7, 9, 5	6.09019, 8.21099, 5.9506	4.69155, 5.77858, 4.54784	3.63504, 5	3	tpsg1	13794
GL173157.1	502222	502558	502331, 502317, 502263	8, 7, 7	8.42257, 5.97725, 8.69489	5.97763, 4.62287, 6.06379	5.76336, 3	3	NA	6107
GL173157.1	525790	526962	526669, 526859, 525978, 526902	10, 16, 6, 7	8.12348, 11.69316, 5.56953, 6.63036	5.57869, 6.51305, 4.42554, 5.0111	5.50152, 8	3	NA	0
GL173157.1	547397	548185	547787, 547761, 547954	5, 8, 7	4.82177, 7.07159, 8.69489	3.98509, 5.20073, 6.06379	2.51418, 4	3	NA	11167
GL173157.1	614933	615833	615367, 615753, 615267	6, 9, 4	5.81608, 7.13885, 4.22496	4.56563, 5.11729, 3.57747	3.4176, 4.7	3	prss29	3510
GL173157.1	642098	642854	642293, 642503, 642377	10, 5, 6	9.77513, 3.35187, 5.91225	6.5837, 3.08382, 4.61907	7.02302, 1	3	NA	4209
GL173157.1	665586	666866	665734, 665734, 666009	41, 46, 32	45.90935, 51.01127, 40.29995	17.67687, 18.36634, 18.23202	42.11716,	3	pfas	24031
GL173157.1	688836	689322	689205, 689279, 689283	13, 17, 14	13.34951, 18.19786, 14.66346	8.21021, 10.18828, 8.7878	10.39596,	3	pfas	1575
GL173157.1	721510	722051	721779, 721775, 721847	6, 9, 7	5.72604, 7.83963, 8.49722	4.51498, 5.55305, 5.97582	3.33547, 5	3	ppil3	0
GL173157.1	733176	734365	733524, 734304, 733725	5, 7, 5	4.82177, 5.97725, 5.9506	3.98509, 4.62287, 4.54784	2.51418, 3	3	ppil3	5592
GL173157.1	802928	803335	803302, 803256, 803308	15, 32, 21	18.26603, 41.22502, 30.38164	10.62691, 18.76902, 15.90848	15.09729,	3	NA	2120
GL173157.1	823413	823793	823735, 823734, 823705	47, 67, 35	63.41125, 84.42473, 50.0928	25.10997, 26.84065, 22.65443	59.38653,	3	NA	22605
GL173159.1	28432	29036	28790, 28821, 28795	7, 21, 14	6.37715, 18.06033, 15.20637	4.86324, 8.95475, 9.10193	3.90257, 1	3	amotl1	0
GL173159.1	60184	60852	60449, 60450, 60409	10, 12, 3	10.46385, 10.73343, 3.47371	6.97883, 6.83116, 3.03189	7.67431, 8	3	amotl1	0
GL173159.1	97154	97715	97606, 97610, 97592	13, 13, 11	11.0266, 9.33876, 10.82241	6.80342, 5.75838, 7.0607	8.20634, 6	3	amotl1	30
GL173159.1	215238	215486	215328, 215328, 215334	11, 12, 12	12.43693, 11.86108, 16.36823	7.97018, 7.51216, 9.85365	9.52692, 9	3	XB-GENE-979293	14349
GL173159.1	284996	285420	285299, 285291, 285294	81, 119, 86	97.9016, 145.2491, 114.96034	26.8379, 31.06813, 33.29963	93.52888,	3	ankrd49	48697
GL173159.1	412656	413199	413041, 413131, 413152	11, 15, 9	12.43693, 14.27489, 11.64359	7.97018, 8.33298, 7.57973	9.52692, 1	3	mre11	0
GL173159.1	582343	582731	582554, 582566, 582566	13, 18, 9	11.60911, 16.34754, 9.64095	7.16262, 8.80191, 6.59867	8.74856, 1	3	NA	1093
GL173159.1	641577	641981	641674, 641803, 641646	7, 17, 8	5.49154, 14.86656, 7.11599	4.32107, 8.17729, 5.22758	3.12578, 1	3	NA	2294
GL173159.1	692618	693832	692920, 693112, 693149	12, 14, 11	13.64785, 12.80997, 12.2694	8.52686, 7.67593, 7.88012	10.68062,	3	NA	10794
GL173159.1	708248	708533	708325, 708319, 708328	8, 19, 8	6.48409, 15.76714, 7.81204	4.83873, 8.16901, 5.63749	4.00135, 1	3	NA	26424
GL173159.1	732023	732522	732135, 732252, 732034	11, 23, 10	10.00935, 22.74071, 11.17239	6.57321, 11.1182, 7.36591	7.24529, 1	3	panx1	4070
GL173160.1	425403	426500	425767, 425743, 425980	26, 29, 21	24.06525, 23.66336, 20.55907	10.89067, 9.69073, 10.45354	20.71001,	3	CCSER2	144660
GL173160.1	429339	430186	429644, 429786, 429988	20, 35, 20	16.65181, 29.90361, 18.80932	8.41216, 11.10915, 9.70668	13.54908,	3	CCSER2	148596
GL173160.1	514369	515086	514860, 514612, 514617	17, 22, 14	15.56568, 18.98967, 15.35015	8.60343, 9.20223, 9.184	12.50679,	3	CCSER2	233626
GL173160.1	759530	761240	759923, 760215, 759804, 760256	15, 25, 5, 10	13.74446, 21.24733, 4.54579, 10.47831	8.00965, 9.58569, 3.82897, 6.98694	10.77314,	3	CCSER2	478787
GL173160.1	806963	807786	807272, 807298, 807544	6, 10, 7	5.96715, 9.39128, 7.84208	4.64927, 6.35644, 5.66426	3.52158, 6	3	CCSER2	526220
GL173161.1	542791	543742	543162, 543197, 543283	14, 27, 8	12.75226, 26.58221, 8.86315	7.64062, 12.02229, 6.21039	9.82689, 2	3	aspg	0
GL173162.1	60246	61182	60634, 60942, 60685	11, 18, 9	11.09349, 16.57986, 10.01275	7.21938, 8.94309, 6.79683	8.25916, 1	3	NA	26557
GL173164.1	9659	10259	10232, 10208, 10042	17, 27, 14	15.98419, 25.45196, 13.17984	8.85831, 11.35757, 7.90153	12.90915,	3	NA	10650
GL173164.1	31210	31620	31521, 31567, 31426	13, 22, 8	12.20544, 21.01356, 7.67847	7.52692, 10.40847, 5.56054	9.31472, 1	3	NA	1139
GL173164.1	176862	177384	177192, 177018, 177242	7, 8, 5	6.37715, 7.07159, 5.91431	4.86324, 5.20073, 4.53172	3.90257, 4	3	NA	0
GL173164.1	252720	253009	252756, 252759, 252755	11, 16, 6	10.43535, 14.80681, 6.99807	6.83063, 8.40621, 5.17195	7.6474, 11	3	capn2.2	15879
GL173164.1	262311	263069	262894, 262591, 262867	10, 8, 8	8.79841, 5.81313, 7.42597	5.99719, 4.41317, 5.41279	6.11782, 3	3	capn2.2	5819
GL173164.1	355913	356834	356418, 356410, 356165	16, 21, 6	15.74146, 17.80261, 5.91225	8.97386, 8.80213, 4.61907	12.67506,	3	fbxo28	10710
GL173164.1	357527	358275	357830, 357990, 357708	7, 13, 9	5.52352, 9.74816, 10.14484	4.34124, 6.01114, 6.86555	3.15454, 7	3	fbxo28	9269
GL173164.1	416251	416702	416300, 416532, 416443	23, 20, 20	25.82433, 19.30258, 22.29127	12.96322, 10.00482, 11.79412	22.41977,	3	nvl	0
GL173164.1	473055	473416	473094, 473139, 473092	13, 20, 18	11.35857, 18.53364, 19.83478	7.00844, 9.54017, 10.89618	8.51036, 1	3	wdr26	0
GL173164.1	496823	497710	497387, 497215, 497602	6, 6, 5	5.96715, 4.76927, 5.9506	4.64927, 3.9431, 4.54784	3.52158, 2	3	wdr26	8861
GL173164.1	571246	571717	571439, 571467, 571465	18, 16, 26	16.5065, 14.52284, 27.48447	8.89851, 8.2328, 12.91976	13.41011,	3	dnah14	4770
GL173164.1	642978	643639	643113, 643543, 643583	6, 7, 7	5.51356, 5.97725, 7.84208	4.39314, 4.62287, 5.66426	3.14574, 3	3	dnah14	0
GL173164.1	653717	654149	653883, 653945, 654121	5, 12, 6	4.82177, 10.83413, 6.5517	3.98509, 6.89288, 4.95623	2.51418, 8	3	dnah14	0
GL173164.1	767108	767516	767129, 767321, 767411	4, 8, 9	3.34243, 6.24338, 10.14484	3.08796, 4.6873, 6.86555	1.41807, 3	3	enah	5334
GL173164.1	797604	798070	797651, 797634, 797753	17, 14, 6	17.33887, 13.63145, 6.99807	9.67746, 8.17488, 5.17195	14.20638,	3	enah	35830
GL173164.1	825218	825797	825766, 825492, 825697	17, 13, 6	18.55637, 11.12608, 6.35308	10.39866, 6.86492, 4.85498	15.37671,	3	enah	63444
GL173165.1	220932	221576	221337, 221302, 221313	9, 19, 14	8.35434, 18.02027, 15.88228	5.86444, 9.5284, 9.48329	5.71584, 1	3	NA	6726
GL173166.1	246360	246953	246658, 246625, 246918	15, 19, 11	15.41679, 19.48976, 13.64572	9.0192, 10.41622, 8.58592	12.36344,	3	XB-GENE-5861581	94328
GL173166.1	273219	273829	273571, 273571, 273624	7, 6, 4	7.1696, 4.93312, 4.46488	5.31345, 4.04501, 3.69425	4.61046, 2	3	XB-GENE-5861581	67452
GL173167.1	22012	22669	22600, 22573, 22596	10, 13, 10	8.32267, 10.04078, 9.2231	5.70305, 6.19235, 6.25548	5.6878, 7.4	3	cdh4	232235
GL173167.1	41998	43175	42468, 42294, 42453	6, 13, 13	5.96715, 13.14514, 17.924	4.64927, 8.09002, 10.57401	3.52158, 1	3	cdh4	211729
GL173167.1	48959	49524	49414, 49239, 49096	14, 19, 9	15.40455, 19.48976, 11.64359	9.21493, 10.41622, 7.57973	12.35264,	3	cdh4	205380
GL173167.1	77317	78024	77426, 77994, 77826	9, 15, 7	9.12009, 15.80067, 8.69489	6.30997, 9.24574, 6.06379	6.42028, 1	3	cdh4	176880
GL173167.1	99810	100548	99917, 100054, 100138	9, 9, 5	8.66407, 6.04841, 5.80393	6.04743, 4.42311, 4.48186	5.99101, 3	3	cdh4	154356
GL173167.1	117643	118021	117767, 117857, 117858	6, 12, 5	5.63898, 11.86108, 5.69847	4.46544, 7.51216, 4.4331	3.25772, 9	3	cdh4	136883
GL173167.1	146029	146508	146235, 146074, 146245	8, 18, 10	7.1122, 16.23409, 10.30677	5.22529, 8.73298, 6.89026	4.57946, 1	3	cdh4	108396
GL173167.1	150601	151177	151125, 150845, 150980	11, 6, 8	9.5732, 3.40406, 7.76694	6.30586, 3.0502, 5.6116	6.84587, 1	3	cdh4	103727
GL173167.1	208628	209315	209137, 208878, 208963	7, 11, 9	5.75798, 8.59076, 7.7212	4.48785, 5.69393, 5.48026	3.36626, 6	3	cdh4	45589
GL173167.1	237942	238551	237975, 238001, 237948	29, 29, 12	32.03412, 29.43697, 13.15613	14.51808, 12.97595, 8.25729	28.48199,	3	cdh4	16353
GL173167.1	243204	243658	243574, 243585, 243592	44, 61, 33	54.22687, 74.49574, 45.65273	21.16065, 24.39034, 20.91135	50.3145, 7	3	cdh4	11246
GL173167.1	250750	252085	251570, 251379, 251382	11, 8, 7	11.29029, 7.07159, 8.15288	7.3332, 5.20073, 5.81587	8.44541, 4	3	cdh4	2819
GL173167.1	286418	287103	286758, 287020, 286956	25, 51, 26	27.23898, 59.14983, 32.64075	13.12872, 20.66418, 15.95571	23.79707,	3	cdh4	0
GL173167.1	298953	299270	299229, 299094, 299212	10, 14, 8	11.06011, 12.18837, 8.24665	7.306, 7.29433, 5.88165	8.22712, 9	3	cdh4	0
GL173167.1	382994	383618	383589, 383590, 383555	33, 25, 32	37.85575, 28.66243, 42.20753	16.38562, 13.97513, 19.33316	34.18862,	3	psma7	0
GL173167.1	407906	408477	408419, 408430, 408447	19, 33, 14	20.39198, 34.38272, 16.82614	10.95535, 14.33629, 9.99514	17.14961,	3	mtg2	2448
GL173167.1	435526	435937	435865, 435875, 435864	8, 18, 6	7.67464, 17.52265, 6.82211	5.55832, 9.51543, 5.08889	5.08299, 1	3	hrh3	16528
GL173167.1	441109	441424	441112, 441185, 441174	7, 21, 5	7.1696, 24.35402, 5.9506	5.31345, 12.71289, 4.54784	4.61046, 2	3	hrh3	22111
GL173167.1	488160	489912	488937, 489354, 489336	10, 15, 7	10.39648, 13.31811, 8.21885	6.94096, 7.74852, 5.84717	7.61003, 1	3	osbpl2	9535
GL173167.1	595593	596278	596028, 596029, 596041	32, 42, 14	33.83282, 42.4233, 15.20637	14.41056, 15.27744, 9.10193	30.24308,	3	lama5	0
GL173167.1	741259	742029	741612, 741518, 742003	6, 11, 8	5.31708, 9.66539, 10.00633	4.2777, 6.36265, 6.75998	2.96727, 7	3	rbbp8nl	0
GL173167.1	748652	749708	749358, 749072, 749192	9, 20, 6	7.84477, 16.73193, 6.03707	5.5562, 8.45971, 4.6874	5.24227, 1	3	rbbp8nl	958
GL173168.1	50426	50823	50769, 50674, 50656	10, 12, 6	8.0085, 7.13547, 5.14244	5.50663, 4.60482, 4.17298	5.39412, 4	3	NA	9609
GL173168.1	51776	52287	51874, 51960, 52135	13, 33, 13	11.81837, 30.30572, 13.97446	7.29094, 12.00715, 8.57174	8.94738, 2	3	NA	10959
GL173168.1	94699	95096	94833, 94943, 94858	10, 12, 6	9.54985, 9.78474, 7.29403	6.45088, 6.24415, 5.30581	6.82341, 7	3	NA	620
GL173168.1	354048	354955	354821, 354578, 354310	5, 9, 4	4.82177, 8.21099, 4.67325	3.98509, 5.77858, 3.78987	2.51418, 5	3	NA	7207
GL173168.1	546270	546702	546535, 546371, 546278	8, 8, 3	8.22568, 7.01915, 3.34298	5.8701, 5.16892, 2.97156	5.59681, 4	3	kif13a	71956
GL173168.1	552456	553030	552915, 552676, 552691	5, 8, 4	4.82177, 6.62693, 4.67325	3.98509, 4.92781, 3.78987	2.51418, 4	3	kif13a	78142
GL173168.1	594565	595362	595210, 595158, 595227	7, 6, 7	7.1696, 4.83043, 7.96278	5.31345, 3.98131, 5.72394	4.61046, 2	3	NA	38745
GL173168.1	745246	745558	745452, 745526, 745452	12, 20, 11	10.1196, 17.13302, 9.7601	6.45229, 8.69853, 6.42085	7.349, 14.1	3	NA	98570
GL173169.1	125098	125773	125163, 125120, 125260	20, 25, 14	20.44929, 25.58747, 17.01463	10.69687, 12.14055, 10.0942	17.20509,	3	tmem30cp	430
GL173169.1	324472	325733	325287, 325146, 324874	14, 11, 7	14.23954, 9.22091, 7.10639	8.53829, 6.08767, 5.27894	11.24094,	3	cmss1	7885
GL173169.1	651398	651739	651517, 651495, 651521	9, 23, 12	9.72082, 22.6679, 16.36823	6.64182, 11.0745, 9.85365	6.97155, 1	3	glra2	38254
GL173170.1	381978	382332	382234, 382290, 382169	8, 10, 6	5.55847, 6.25995, 5.60548	4.24931, 4.40107, 4.44624	3.18478, 3	3	prmt7	20556
GL173171.1	15654	16496	15753, 15730, 15784	8, 9, 8	7.33538, 6.40439, 8.09568	5.35908, 4.65094, 5.79794	4.76518, 4	3	NA	4467
GL173171.1	77468	78093	77712, 77650, 77666	14, 20, 5	14.23954, 19.71581, 5.91431	8.53829, 10.25455, 4.53172	11.24094,	3	NA	0
GL173171.1	125448	125878	125827, 125595, 125775	9, 12, 6	9.72082, 11.86108, 7.29403	6.64182, 7.51216, 5.30581	6.97155, 9	3	NA	32634
GL173171.1	141917	142425	142187, 142212, 142285	12, 14, 17	8.48203, 9.98671, 17.26186	5.43209, 5.94071, 9.63127	5.81957, 7	3	NA	39174
GL173171.1	142651	143891	143044, 143426, 143300	10, 15, 8	6.72901, 11.26316, 6.44721	4.69767, 6.49004, 4.81564	4.22745, 8	3	NA	37708
GL173171.1	382048	382897	382402, 382315, 382590	8, 9, 4	8.42257, 8.21099, 4.67325	5.97763, 5.77858, 3.78987	5.76336, 5	3	NA	15476
GL173171.1	555733	556848	556217, 556236, 556542	8, 22, 5	6.70593, 18.423, 5.06966	4.9768, 8.86967, 4.11933	4.20487, 1	3	NA	12170
GL173171.1	787119	787562	787136, 787133, 787176	39, 58, 41	47.11081, 72.01686, 56.68708	19.2772, 24.52527, 23.98501	43.30072,	3	NA	7152
GL173172.1	235742	236939	236122, 236255, 235938	26, 43, 14	27.19703, 41.8549, 13.93205	12.74794, 14.57403, 8.35526	23.75642,	3	vopp1	0

GL173173.1	328337	328931	328781, 328404, 328656	9, 18, 4	9.30591, 17.65791, 4.67325	6.41439, 9.59733, 3.78987	6.59443, 1	3	NA	91022
GL173173.1	389826	390353	389949, 390054, 390099	11, 11, 10	12.33515, 9.17879, 11.03772	7.91559, 6.06147, 7.29398	9.43699, 6	3	NA	46840
GL173173.1	481949	482452	482399, 482223, 482381	5, 14, 7	4.02828, 12.38816, 7.78342	3.51866, 7.41725, 5.63488	1.88216, 9	3	NA	0
GL173174.1	58	4141	1911, 1787, 1567	68, 94, 61	34.98977, 44.80946, 36.35981	6.60748, 6.27747, 7.82646	31.37698,	3	rspo1	168738
GL173174.1	4676	9587	4804, 7549, 5836, 7259	36, 112, 25, 64	6.38105, 54.04001, 4.61699, 34.57329	2.41397, 6.4817, 2.32323, 6.95158	3.90606, 5	3	rspo1	163292
GL173174.1	4676	9587	7291, 7549, 5836, 7259	97, 112, 25, 64	55.54068, 54.04001, 4.61699, 34.57329	7.95062, 6.4817, 2.32323, 6.95158	51.61032,	3	rspo1	163292
GL173174.1	13024	15441	14546, 14547, 14375	54, 81, 46	33.98534, 50.88013, 34.94691	8.21736, 8.81931, 10.28137	30.39245,	3	rspo1	157438
GL173174.1	19151	20466	19535, 19471, 19536	62, 68, 48	57.55584, 53.0559, 47.19936	15.31666, 11.81719, 15.4463	53.59875,	3	rspo1	152413
GL173174.1	570767	571219	571015, 570932, 571041	9, 18, 12	9.50098, 19.97713, 16.36823	6.52233, 10.97931, 9.85365	6.77745, 1	3	epha10	0
GL173175.1	141266	141503	141442, 141443, 141442	12, 12, 11	12.05017, 10.83413, 13.28509	7.62377, 6.89288, 8.40871	9.16715, 8	3	mettl3	524
GL173176.1	215214	215552	215232, 215217, 215347	8, 12, 5	8.34715, 10.93692, 5.50079	5.9367, 6.95571, 4.33868	5.71011, 8	3	mycn	87905
GL173176.1	341686	342339	342173, 341902, 341947	7, 9, 6	6.25077, 8.21099, 6.35308	4.78814, 5.77858, 4.85498	3.78441, 5	3	mycn	32286
GL173176.1	579795	580086	580060, 579845, 579886	5, 10, 5	4.655, 8.36423, 5.06966	3.89157, 5.72892, 4.11933	2.42652, 5	3	fam49a	0
GL173176.1	786240	786792	786619, 786580, 786623	15, 24, 14	17.28737, 25.03583, 15.49745	10.09595, 12.15722, 9.26757	14.15669,	3	fam49a	176938
GL173177.1	544266	544548	544351, 544471, 544351	8, 7, 9	6.55639, 5.55323, 9.8849	4.88389, 4.35994, 6.72947	4.06705, 3	3	krr1	0
GL173177.1	714539	715322	714879, 714665, 714889	11, 14, 9	8.81032, 11.07823, 7.75727	5.8315, 6.60976, 5.50247	6.12919, 8	3	BBS10	6509
GL173177.1	714539	715459	715317, 714665, 714889	9, 14, 9	6.36774, 11.07823, 7.75727	4.62751, 6.60976, 5.50247	3.8942, 8.4	3	BBS10	6509
GL173178.1	14220	15124	14964, 14661, 14590	12, 28, 7	11.32055, 29.75777, 7.96278	7.18871, 13.54138, 5.72394	8.47391, 2	3	rai14	103213
GL173178.1	27191	27907	27367, 27291, 27237	16, 18, 9	15.34845, 16.52108, 9.46739	8.73591, 8.90737, 6.50386	12.2978, 1	3	rai14	90430
GL173178.1	113180	113371	113345, 113333, 113230	13, 18, 10	13.42064, 15.53678, 9.98291	8.25183, 8.30996, 6.70471	10.46414,	3	rai14	4966
GL173178.1	327678	327990	327745, 327710, 327820	10, 11, 4	9.54985, 8.93411, 3.85136	6.45088, 5.9089, 3.38149	6.82341, 6	3	prlr	68139
GL173178.1	454342	455223	454727, 454739, 454736	10, 21, 4	10.26482, 20.81922, 4.22496	6.86644, 10.61044, 3.57747	7.48624, 1	3	prlr	194803
GL173178.1	563777	564575	563995, 564037, 564254	18, 17, 9	17.58399, 15.5773, 9.14171	9.55259, 8.61051, 6.32219	14.44229,	3	spef2	136725
GL173178.1	588065	588695	588467, 588616, 588618	8, 15, 12	6.78332, 11.11727, 10.38562	5.02459, 6.40155, 6.61699	4.27811, 8	3	spef2	112605
GL173178.1	591782	592307	591816, 591820, 591820	25, 34, 39	28.23973, 33.84297, 48.06316	13.72472, 13.63168, 19.83945	24.77449,	3	spef2	108993
GL173178.1	603538	604130	603825, 603857, 603985	15, 21, 10	15.05862, 18.16568, 9.82971	8.80576, 9.01729, 6.61563	12.0251, 1	3	spef2	97170
GL173178.1	609614	610026	609918, 609943, 609928	32, 25, 17	37.27419, 23.34787, 21.19882	16.44909, 10.80775, 11.87761	33.6175, 2	3	spef2	91274
GL173178.1	773941	774812	774226, 774499, 774558	34, 46, 17	27.07779, 35.18337, 13.45736	9.96817, 10.38219, 7.32301	23.64059,	3	spef2	2614
GL173178.1	775997	776956	776641, 776615, 776293	32, 53, 25	25.84314, 44.7902, 23.39814	9.93384, 12.56702, 10.83742	22.43823,	3	spef2	4670
GL173179.1	12848	13417	13370, 13121, 13137	8, 15, 13	8.42257, 15.80067, 18.01082	5.97763, 9.24574, 10.61163	5.76336, 1	3	NA	77479
GL173179.1	51075	51637	51604, 51607, 51591	5, 9, 6	4.82177, 8.21099, 7.29403	3.98509, 5.77858, 5.30581	2.51418, 5	3	NA	39259
GL173179.1	74057	74603	74234, 74237, 74323	7, 10, 5	6.60038, 8.79469, 5.59755	4.99378, 5.99491, 4.38538	4.10761, 6	3	NA	16293
GL173179.1	556540	557056	556797, 556611, 556570	18, 21, 7	17.86195, 20.25932, 8.08824	9.72071, 10.27277, 5.7849	14.71061,	3	shisa7	4487
GL173180.1	5196	5593	5337, 5428, 5391	10, 13, 6	9.49535, 10.2718, 7.18591	6.41851, 6.33559, 5.25776	6.77344, 7	3	nell1	26285
GL173180.1	15266	16709	15484, 15844, 16466	9, 10, 8	9.06007, 9.27513, 8.6841	6.27591, 6.2868, 6.11715	6.36394, 6	3	nell1	15169
GL173180.1	15266	16709	16627, 15844, 16466	6, 10, 8	4.89903, 9.27513, 8.6841	4.02393, 6.2868, 6.11715	2.58474, 6	3	nell1	15169
GL173180.1	43267	43684	43294, 43361, 43392	14, 14, 3	16.7649, 14.45897, 3.47371	9.96273, 8.66788, 3.03189	13.6543, 1	3	nell1	0
GL173180.1	61587	62014	61815, 61863, 61806	23, 36, 14	24.58488, 38.35513, 17.2091	12.225, 15.43492, 10.19524	21.21468,	3	nell1	0
GL173180.1	76842	77462	77247, 76960, 76958	9, 12, 6	7.03134, 8.88945, 5.46454	5.04951, 5.68576, 4.36458	4.50586, 6	3	nell1	0
GL173180.1	79005	79362	79064, 79048, 79079	11, 15, 8	9.66657, 12.7702, 7.90404	6.36338, 7.41214, 5.68997	6.93336, 1	3	nell1	0
GL173180.1	79718	80818	80108, 80566, 80585	10, 11, 6	8.20198, 8.27243, 5.02414	5.62778, 5.49405, 4.10097	5.57541, 5	3	nell1	0
GL173180.1	87709	88182	87896, 87754, 87897	20, 20, 12	18.51378, 19.71581, 12.09328	9.52818, 10.25455, 7.64909	15.33675,	3	nell1	0
GL173180.1	109320	110271	109694, 110201, 109582	26, 26, 25	28.67952, 23.54286, 26.84901	13.63367, 10.58612, 12.89564	25.20328,	3	nell1	0
GL173180.1	129747	130860	130157, 130818, 130782	16, 16, 9	17.41877, 15.28831, 9.94831	9.96955, 8.69939, 6.76298	14.28292,	3	nell1	0
GL173180.1	231507	231933	231727, 231748, 231738	20, 22, 23	20.84802, 22.45914, 27.80858	10.93641, 11.27809, 14.1214	17.59179,	3	nell1	0
GL173180.1	240950	241461	241213, 241217, 241191	6, 9, 7	5.06496, 8.21099, 8.28621	4.12592, 5.77858, 5.87881	2.73812, 5	3	nell1	0
GL173180.1	249483	250032	249513, 249498, 249540	16, 19, 11	14.68346, 16.8936, 10.87462	8.33092, 8.84622, 7.09143	11.66544,	3	nell1	0
GL173180.1	254675	255126	254863, 254916, 254921	10, 10, 6	8.16254, 7.55261, 5.29847	5.60312, 5.21944, 4.26663	5.53763, 5	3	nell1	0
GL173180.1	271961	272549	272194, 272403, 272339	12, 9, 7	11.32055, 7.28344, 8.35502	7.18871, 5.20811, 5.9108	8.47391, 4	3	nell1	0
GL173180.1	288977	289489	289414, 289403, 289152	5, 9, 6	4.82177, 8.21099, 7.29403	3.98509, 5.77858, 5.30581	2.51418, 5	3	nell1	0
GL173180.1	294612	295167	294833, 295084, 294748	4, 10, 6	3.48339, 9.39128, 7.29403	3.1722, 6.35644, 5.30581	1.52925, 6	3	nell1	0
GL173180.1	302364	303407	302480, 302861, 302947	10, 10, 7	11.06011, 8.0878, 6.75301	7.306, 5.55635, 5.08141	8.22712, 5	3	nell1	4403
GL173180.1	357802	358209	357822, 357829, 358154	8, 14, 5	6.78332, 10.88047, 4.64121	5.02459, 6.48797, 3.88372	4.27811, 8	3	slc6a5	35504
GL173180.1	435223	437139	436417, 436554, 436497	9, 11, 8	8.55802, 10.60895, 8.98772	5.98518, 6.9343, 6.27416	5.8909, 7.9	3	slc6a5	0
GL173180.1	480287	480565	480525, 480432, 480387	8, 6, 6	6.94372, 3.95542, 5.60548	5.12297, 3.41818, 4.44624	4.42528, 1	3	prmt3	0
GL173180.1	503064	503462	503128, 503107, 503130	17, 26, 14	16.90454, 24.63218, 16.2088	9.41624, 11.22343, 9.66324	13.78827,	3	prmt3	0
GL173180.1	509089	509440	509354, 509382, 509379	9, 17, 11	8.0679, 15.18554, 13.1143	5.69219, 8.37169, 8.32282	5.45, 12.32	3	prmt3	0
GL173180.1	589611	589919	589631, 589628, 589628	5, 7, 7	4.17642, 5.10722, 7.10639	3.60969, 4.07606, 5.27894	2.01422, 2	3	nav2	5502
GL173180.1	636639	637112	636934, 636725, 636727	9, 13, 6	9.72082, 13.14514, 7.29403	6.64182, 8.09002, 5.30581	6.97155, 1	3	nav2	0
GL173180.1	683040	684070	683317, 683421, 683401	5, 10, 8	4.82177, 8.28331, 10.0913	3.98509, 5.67853, 6.79758	2.51418, 5	3	nav2	0
GL173180.1	756248	756673	756550, 756591, 756573	10, 28, 12	8.32267, 27.03127, 12.7555	5.70305, 11.92807, 8.03189	5.6878, 23	3	nav2	57980
GL173181.1	9373	10260	9561, 9561, 9556	28, 32, 16	31.3955, 32.9028, 19.86305	14.51749, 13.86483, 11.32783	27.85612,	3	otop3	0
GL173181.1	63882	64386	64086, 64092, 64073	9, 19, 8	7.17299, 18.08435, 7.38564	5.13877, 9.56723, 5.38892	4.61317, 1	3	mrpl58	2356
GL173181.1	171590	172003	171918, 171837, 171809	6, 10, 6	5.96715, 9.39128, 6.87953	4.64927, 6.35644, 5.11627	3.52158, 6	3	hn1	2881
GL173181.1	238041	238955	238246, 238820, 238499	6, 12, 4	4.65447, 9.54462, 3.70359	3.87084, 6.09452, 3.29934	2.42652, 6	3	tmem94	1079
GL173181.1	256323	256762	256420, 256339, 256363	7, 13, 9	7.1696, 13.14514, 11.64359	5.31345, 8.09002, 7.57973	4.61046, 1	3	tmem94	0
GL173181.1	266935	267419	267143, 267118, 266949	8, 12, 6	6.78332, 10.73343, 7.25145	5.02459, 6.83116, 5.287	4.27811, 8	3	tmem94	0
GL173181.1	294251	294696	294639, 294666, 294680	9, 10, 6	8.30507, 8.20397, 5.83216	5.83501, 5.62902, 4.57461	5.67102, 5	3	caskin2	1193
GL173181.1	323355	324271	323846, 323846, 323984	7, 6, 3	5.27717, 4.76927, 3.34298	4.185, 3.9431, 2.97156	2.93268, 2	3	tsen54	2476
GL173181.1	539609	540145	539866, 540051, 539883	6, 5, 5	5.96715, 3.73203, 5.91431	4.64927, 3.33183, 4.53172	3.52158, 1	3	galk1	0
GL173181.1	562077	562585	562574, 562402, 562215	6, 8, 9	5.47306, 7.07159, 10.49552	4.36955, 5.20073, 7.0436	3.10811, 4	3	galk1	7352
GL173181.1	634197	634765	634280, 634473, 634276	5, 6, 6	4.61714, 4.89312, 6.87953	3.86998, 4.02027, 5.11627	2.39372, 2	3	wbp2	0
GL173181.1	689699	690918	690344, 690534, 690172	11, 15, 8	6.31992, 8.27198, 6.1856	4.27658, 4.7381, 4.65072	3.85018, 5	3	NA	0
GL173181.1	694276	694721	694302, 694291, 694308	53, 54, 35	58.27062, 56.51143, 44.66119	19.2446, 17.86465, 19.57099	54.30414,	3	NA	123
GL173182.1	378831	379764	379337, 379372, 379346	12, 18, 10	10.1642, 15.79712, 12.16489	6.47994, 8.46772, 7.87042	7.39114, 1	3	NA	21715
GL173183.1	31811	32262	32199, 32209, 32230	37, 64, 32	45.46008, 74.74284, 39.43748	19.18814, 23.02412, 17.72718	41.67442,	3	eif3h	273064
GL173183.1	63611	63981	63954, 63943, 63864	12, 19, 7	12.3187, 17.17455, 8.646	7.78073, 9.01609, 6.04229	9.4231, 14	3	eif3h	241345
GL173183.1	113866	114332	114131, 114208, 114029	5, 9, 5	4.82177, 8.01512, 5.9506	3.98509, 5.66016, 4.54784	2.51418, 5	3	eif3h	190994
GL173183.1	273839	274132	273891, 273904, 273941	5, 9, 5	4.54329, 8.21099, 5.31857	3.82752, 5.77858, 4.24819	2.33, 5.708	3	eif3h	31194
GL173183.1	362090	362356	362327, 362294, 362323	37, 43, 34	40.03318, 44.87308, 47.22582	15.99336, 16.22194, 21.42934	36.33001,	3	eif3h	0
GL173183.1	573116	573672	573425, 573443, 573444	15, 31, 8	14.98937, 32.3733, 9.05167	8.76428, 13.94378, 6.30653	11.95813,	3	slc30a8	27721
GL173183.1	600808	601375	600878, 600813, 600848	52, 60, 48	64.62125, 69.30603, 63.33316	23.31938, 22.00569, 24.57256	60.58236,	3	med30	10555
GL173183.1	637902	638339	638308, 638298, 638310	10, 28, 12	9.49535, 29.22753, 13.44151	6.41851, 13.22571, 8.41472	6.77344, 2	3	med30	22804
GL173183.1	682815	683332	683299, 683176, 683049	14, 11, 8	14.10012, 9.71235, 9.05167	8.4555, 6.39153, 6.30653	11.10747,	3	med30	67717
GL173183.1	741420	742177	741750, 741712, 741677	11, 17, 10	12.43693, 18.56115, 12.71002	7.97018, 10.40145, 8.12734	9.52692, 1	3	med30	126322
GL173185.1	129817	130148	129955, 129824, 130000	7, 10, 3	6.79162, 9.39128, 3.17999	5.10336, 6.35644, 2.89245	4.28406, 6	3	NA	32835
GL173185.1	211265	211825	211661, 211431, 211698	15, 15, 14	16.72085, 13.69427, 16.29325	9.7776, 7.97895, 9.7093	13.61562,	3	NA	47062
GL173185.1	339051	339937	339349, 339205, 339282	9, 12, 11	9.00096, 10.30411, 13.28509	6.24222, 6.56659, 8.40871	6.30808, 7	3	trps1	136480
GL173185.1	459144	459777	459426, 459486, 459444	10, 16, 8	9.18236, 15.47832, 9.31951	6.23091, 8.81468, 6.43944	6.47811, 1	3	trps1	16640
GL173185.1	585864	586219	585895, 585922, 585866	10, 22, 15	8.48936, 18.72754, 17.76194	5.80661, 9.04803, 10.35658	5.82603, 1	3	trps1	0
GL173185.1	611360	611767	611403, 611514, 611518	7, 10, 3	5.79309, 7.8291, 3.34298	4.5096, 5.39387, 2.97156	3.39779, 5	3	trps1	16809
GL173186.1	107322	108184	107746, 107731, 107933	4, 7, 3	3.23728, 5.68499, 3.47371	3.02372, 4.44244, 3.03189	1.33324, 3	3	ppp1r3c.1	2377
GL173186.1	654025	654452	654219, 654219, 654219	27, 28, 17	29.83076, 30.12516, 18.42656	13.9568, 13.76034, 10.3227	26.3266, 2	3	HTR7	77476
GL173187.1	27747	28562	27977, 28056, 28473	10, 20, 4	11.06011, 19.85827, 4.67325	7.306, 10.34058, 3.78987	8.22712, 1	3	NA	NA
GL173187.1	538759	539150	539028, 538992, 539051	38, 80, 33	36.53212, 83.03391, 36.28073	13.60695, 20.08498, 15.4526	32.88935,	3	NA	NA
GL173187.1	786193	787066	786829, 786754, 786782	7, 15, 8	7.15806, 15.80067, 10.00633	5.30717, 9.24574, 6.75998	4.61046, 1	3	NA	NA
GL173188.1	112547	113163	112918, 112918, 112809	13, 29, 12	12.38021, 25.13767, 11.60888	7.63279, 10.49978, 7.36202	9.48056, 2	3	macf1	0
GL173189.1	28096	29331	29125, 28435, 28231	7, 18, 4	5.27717, 14.40368, 4.67325	4.185, 7.62784, 3.78987	2.93268, 1	3	erc2	232460
GL173189.1	117716	118337	117783, 118221, 118219	6, 8, 6	5.63898, 5.35923, 5.71639	4.46544, 4.12054, 4.50951	3.25772, 3	3	erc2	143454
GL173189.1	179560	180351	179981, 180161, 180121	5, 14, 11	4.40263, 11.53986, 13.46208	3.74533, 6.89446, 8.49639	2.21004, 8	3	erc2	81440
GL173189.1	192579	193735	193106, 193298, 193317	8, 14, 8	7.47668, 12.86494, 8.14528	5.44269, 7.70954, 5.82558	4.89713, 1	3	erc2	68056
GL173189.1	221205	221941	221382, 221311, 221538	8, 9, 6	7.1122, 6.67403, 6.71074	5.22529, 4.82293, 5.03498	4.57946, 4	3	erc2	39850
GL173189.1	249882	250724	250252, 250384, 250302	12, 9, 9	13.8483, 8.21099, 10.35155	8.63436, 5.77858, 6.97128	10.86694,	3	erc2	11067
GL173189.1	291464	292313	292143, 292140, 291952	7, 15, 9	7.1696, 14.52325, 11.21577	5.31345, 8.48354, 7.38849	4.61046, 1	3	erc2	0
GL173189.1	308892	309252	309115, 309116, 309119	85, 106, 53	106.07704, 126.51602, 68.30876	29.04639, 28.6503, 24.96003	101.6378,	3	erc2	0
GL173189.1	335083	336242	335585, 335615, 335595	10, 31, 8	9.38842, 31.60612, 9.3897	6.35473, 13.49298, 6.47354	6.67167, 2	3	erc2	0
GL173189.1	335083	336242	336030, 335615, 335595	6, 31, 8	5.24248, 31.60612, 9.3897	4.2332, 13.49298, 6.47354	2.89962, 2	3	erc2	0
GL173189.1	343434	343899	343781, 343697, 343592	5, 6, 5	4.69353, 4.6513, 5.10931	3.9134, 3.86883, 4.14026	2.45909, 2	3	erc2	0
GL173189.1	373665	374692	374343, 374242, 374344	20, 31, 15	19.02197, 30.42189, 16.44983	9.83519, 12.80311, 9.62273	15.82604,	3	erc2	0
GL173189.1	399131	399623	399165, 399160, 399151	12, 34, 5	9.45689, 33.53045, 5.40789	6.0398, 13.45313, 4.29296	6.73641, 3	3	erc2	0
GL173189.1	403151	403537	403166, 403165, 403220	11, 14, 5	8.50933, 9.33643, 4.99237	5.64284, 5.54763, 4.0781	5.845, 6.77	3	erc2	0
GL173189.1	412375	412594	412411, 412423, 412422	14, 23, 11	13.63662, 21.83526, 12.06254	8.17799, 10.57561, 7.76744	10.67031,	3	erc2	0
GL173189.1	432234	432732	432667, 432698, 432669	26, 23, 26	31.36507, 27.36982, 37.42201	15.21991, 13.8686, 18.53699	27.82689,	3	erc2	0
GL173189.1	447332	448387	448123, 448123, 447696, 448124	16, 25, 8, 12	14.34724, 22.96588, 7.95096, 13.15613	8.12546, 10.58288, 5.71659, 8.25729	11.34358,	3	erc2	0
GL173189.1	455345	456475	455798, 455650, 455649	10, 21, 6	10.89158, 21.26419, 7.25145	7.21503, 10.87863, 5.287	8.0782, 18	3	erc2	6228
GL173189.1	492302	492781	492333, 492305, 492332	10, 27, 10	8.79841, 25.58706, 9.49232	5.99719, 11.43661, 6.41671	6.11782, 2	3	ccdc66	7960
GL173189.1	493235	493726	493685, 493607, 493509	12, 21, 11	11.37748, 17.80261, 10.92742	7.22306, 8.80213, 7.12242	8.52862, 1	3	ccdc66	7015
GL173189.1	498475	499454	498874, 499089, 498884	4, 13, 8	3.63646, 11.2197, 9.31951	3.26116, 6.92276, 6.43944	1.6222, 8.5	3	ccdc66	1287
GL173189.1	560945	562123	561417, 561206, 561671, 561291	11, 10, 8, 9	9.21796, 6.30783, 4.59642, 8.26067	6.08584, 4.43128, 3.62559, 5.80842	6.51275, 3	3	FAM208A	0
GL173189.1	649398	649876	649579, 649774, 649596	16, 31, 22	17.95866, 33.86171, 28.19009	10.28064, 14.82427, 14.60906	14.80292,	3	arhgef3.2	26237
GL173189.1	656463	656796	656636, 656558, 656557	7, 9, 6	7.1696, 8.21099, 7.29403	5.31345, 5.77858, 5.30581	4.61046, 5	3	arhgef3.2	33302
GL173189.1	679125	679638	679543, 679562, 679574	20, 25, 12	22.17215, 26.34052, 13.59027	11.72403, 12.59134, 8.49571	18.87307,	3	arhgef3.2	55964
GL173189.1	686727	687602	687230, 687362, 687294	8, 8, 6	7.57423, 7.07159, 7.29403	5.4999, 5.20073, 5.30581	4.98865, 4	3	il17rd	60648
GL173189.1	702787	703250	703089, 703112, 703085	13, 29, 13	14.52571, 30.90019, 13.97446	8.88215, 13.84312, 8.57174	11.51483,	3	il17rd	45000
GL173189.1	711768	712199	711962, 711920, 711935	9, 12, 7	8.88537, 10.68384, 8.28621	6.17591, 6.80072, 5.87881	6.19961, 8	3	il17rd	36051
GL173189.1	727533	727997	727858, 727853, 727826	5, 10, 8	4.54329, 8.4887, 9.05167	3.82752, 5.80621, 6.30653	2.33, 5.972	3	il17rd	20253
GL173189.1	783453	784212	783795, 783765, 783649	10, 7, 8	7.25363, 5.2482, 8.4044	5.03021, 4.16649, 5.9678	4.68848, 3	3	il17rd	0
GL173189.1	820438	821145	820815, 820517, 820629	9, 14, 3	9.72082, 14.45897, 3.47371	6.64182, 8.66788, 3.03189	6.97155, 1	3	il17rd	5866
GL173192.1	13	239	67, 75, 78	13, 24, 11	14.10549, 28.8739, 14.75772	8.64628, 14.4287, 9.09568	11.11201,	3	ache	4150
GL173192.1	86461	87832	87089, 86913, 87192	8, 17, 15	6.37855, 14.91871, 16.52447	4.77253, 8.20906, 9.66554	3.90357, 1	3	pelp1	8893
GL173192.1	86541	87908	87668, 87687, 87192	9, 6, 15	7.55321, 3.80944, 16.52447	5.37637, 3.32146, 9.66554	4.96981, 1	3	pelp1	8973
GL173192.1	98731	99180	98915, 99025, 98942	6, 5, 5	5.96715, 3.94561, 5.31857	4.64927, 3.46715, 4.24819	3.52158, 1	3	arrb2	460
GL173192.1	104485	105326	105234, 105098, 104655	6, 12, 4	5.43318, 11.31437, 4.64325	4.34622, 7.18497, 3.77643	3.07284, 8	3	arrb2	0
GL173192.1	110250	110588	110537, 110523, 110562	7, 14, 4	6.46447, 12.33756, 4.67325	4.91463, 7.38613, 3.78987	3.98312, 9	3	arrb2	0
GL173192.1	167750	168344	168031, 167946, 168069	11, 8, 6	11.78412, 7.07159, 7.29403	7.61327, 5.20073, 5.30581	8.91516, 4	3	ddx27	12226
GL173192.1	238959	240089	238991, 239030, 239903, 239000, 239881	15, 11, 14, 9, 5	17.18944, 10.60895, 14.36901, 10.42289, 5.14967	10.04146, 6.9343, 8.61486, 7.00726, 4.16141	14.06311,	3	NA	0
GL173192.1	276775	277889	277374, 276942, 277012	13, 13, 6	12.62236, 10.8548, 6.5517	7.77868, 6.69706, 4.95623	9.70369, 8	3	NA	601
GL173192.1	345013	345893	345585, 345106, 345444	13, 13, 8	15.1723, 13.14514, 10.1465	9.23486, 8.09002, 6.82176	12.13305,	3	NA	12975
GL173192.1	405512	406337	405994, 406105, 406011	12, 15, 16	11.43506, 14.03607, 18.0198	7.25774, 8.18766, 10.31552	8.58315, 1	3	NA	0
GL173192.1	416507	417520	417304, 417053, 417134	8, 13, 7	7.93925, 13.1283, 8.49722	5.70996, 8.08007, 5.97582	5.33002, 1	3	NA	0
GL173192.1	482428	482902	482851, 482850, 482869	14, 7, 9	14.38232, 5.82304, 10.01275	8.62271, 4.52813, 6.79683	11.37721,	3	NA	18277
GL173192.1	497493	498352	497820, 497679, 497561	11, 19, 8	9.95877, 18.48026, 8.14528	6.54239, 9.80703, 5.82558	7.19682, 1	3	NA	33342
GL173192.1	516859	517576	516984, 517183, 517507	6, 9, 5	5.96715, 8.21099, 5.9506	4.64927, 5.77858, 4.54784	3.52158, 5	3	NA	52708
GL173192.1	548357	549277	548904, 549266, 548446	7, 7, 5	6.89193, 5.58561, 5.64747	5.15998, 4.38027, 4.40911	4.37772, 3	3	XB-GENE-5961370	22931
GL173192.1	567087	567468	567424, 567441, 567387	9, 12, 11	8.30507, 11.86108, 12.86886	5.83501, 7.51216, 8.19722	5.67102, 9	3	XB-GENE-5961370	4740
GL173192.1	586040	586585	586520, 586503, 586558	12, 13, 12	11.67221, 10.07856, 14.76955	7.39985, 6.21577, 9.10943	8.80793, 7	3	XB-GENE-5961370	0
GL173192.1	617009	617991	617216, 617620, 617726	13, 18, 5	12.26307, 18.07902, 5.75061	7.56188, 9.8517, 4.45735	9.36972, 1	3	XB-GENE-5961370	0
GL173192.1	632161	632510	632469, 632477, 632476	33, 61, 22	38.68132, 70.35182, 25.9713	16.87521, 22.09482, 13.35936	34.99999,	3	XB-GENE-5961370	0
GL173193.1	7575	8123	8090, 8062, 8095	8, 7, 10	7.47668, 5.75319, 11.24131	5.44269, 4.48487, 7.40241	4.89713, 3	3	NA	1384
GL173193.1	58854	59284	58963, 59003, 59083	7, 15, 8	7.1696, 15.80067, 10.1465	5.31345, 9.24574, 6.82176	4.61046, 1	3	NA	25423
GL173193.1	89095	89528	89274, 89226, 89191	13, 10, 11	12.62236, 6.38085, 11.92945	7.77868, 4.47737, 7.69409	9.70369, 4	3	NA	0
GL173193.1	112931	114019	113797, 113532, 113797, 113338	18, 6, 12, 4	22.92104, 4.93312, 11.86108, 4.67325	12.61945, 4.04501, 7.51216, 3.78987	19.59912,	3	NA	16416
GL173193.1	154453	154801	154479, 154491, 154555	13, 19, 11	15.29169, 21.4153, 14.68355	9.29854, 11.55717, 9.06344	12.24396,	3	NA	0
GL173193.1	249947	250431	250386, 250382, 250392	25, 27, 15	27.43165, 30.66659, 18.53291	13.24375, 14.45444, 10.76715	23.98467,	3	NA	8036
GL173193.1	262451	263122	262766, 262995, 262806	23, 32, 13	26.3466, 30.01908, 14.0426	13.27146, 12.20479, 8.61056	22.92763,	3	NA	0
GL173193.1	273832	274364	274321, 274241, 274253	10, 12, 12	11.06011, 11.86108, 16.36823	7.306, 7.51216, 9.85365	8.22712, 9	3	NA	575
GL173193.1	307660	308100	308074, 308042, 308054	14, 17, 14	15.66744, 18.56115, 18.05504	9.36333, 10.40145, 10.62048	12.60415,	3	NA	5684
GL173193.1	324064	324570	324464, 324479, 324540	12, 18, 14	13.8483, 19.97713, 17.40994	8.63436, 10.97931, 10.29833	10.86694,	3	NA	22088
GL173193.1	376097	376742	376402, 376573, 376497	6, 10, 5	5.96715, 9.39128, 5.9506	4.64927, 6.35644, 4.54784	3.52158, 6	3	NA	501
GL173193.1	418979	419552	419251, 419325, 419126	5, 6, 3	4.82177, 4.93312, 3.34298	3.98509, 4.04501, 2.97156	2.51418, 2	3	NA	4738
GL173193.1	624299	625476	624765, 624844, 624867	7, 7, 4	6.2923, 5.48955, 4.42274	4.81292, 4.31982, 3.67426	3.8235, 3.2	3	NA	9580
GL173193.1	705152	705605	705405, 705408, 705404	10, 16, 10	9.95227, 15.35105, 9.98291	6.68696, 8.73748, 6.70471	7.19087, 1	3	NA	3730
GL173193.1	743465	743965	743589, 743585, 743775	6, 22, 10	5.09949, 22.45914, 12.61444	4.14694, 11.27809, 8.08336	2.76939, 1	3	XB-GENE-5888675	4135
GL173195.1	136734	137027	136746, 136803, 136783	12, 12, 10	12.90199, 10.93692, 12.25126	8.11489, 6.95571, 7.91211	9.97033, 8	3	tcof1	14818
GL173195.1	194789	195365	195028, 195012, 194997	9, 18, 9	8.0679, 16.81975, 9.70054	5.69219, 9.08887, 6.63089	5.45, 13.89	3	csf1r	72364
GL173195.1	487647	488682	487784, 488180, 487785	18, 18, 17	16.38949, 12.91519, 16.54102	8.8274, 6.7505, 9.19646	13.29737,	3	slc6a7	0
GL173195.1	501794	502603	501829, 501866, 502070	11, 11, 9	10.9046, 10.31711, 10.42289	7.10903, 6.75959, 7.00726	8.08987, 7	3	slc6a7	5768
GL173195.1	566228	566614	566473, 566324, 566505	7, 9, 7	6.55439, 7.88274, 8.69489	4.96711, 5.57944, 6.06379	4.06616, 5	3	camk2a	0
GL173195.1	676743	677229	677002, 677010, 677038	5, 7, 6	4.47183, 5.97725, 7.29403	3.78598, 4.62287, 5.30581	2.26935, 3	3	NA	21789
GL173195.1	696993	698094	697547, 697547, 697762	52, 79, 37	56.06557, 78.30681, 41.07858	18.4915, 18.39894, 16.60374	52.1286, 7	3	arsi	27592
GL173195.1	740182	741388	740843, 740524, 741113	15, 18, 8	14.46121, 15.79712, 9.31951	8.44598, 8.46772, 6.43944	11.45219,	3	arsi	1472
GL173196.1	8	2017	78, 79, 99, 1854	83, 113, 85, 42	59.03089, 80.61558, 72.16093, 24.57909	10.63677, 11.20364, 14.24609, 7.07129	55.05469,	3	NA	11971
GL173196.1	597308	597491	597358, 597360, 597358	398, 324, 312	514.95129, 421.60315, 428.64032	45.65257, 45.01961, 51.66009	509.23706	3	FAAP100	0
GL173197.1	156307	156789	156473, 156484, 156477	17, 13, 15	17.19112, 11.56107, 17.07266	9.58879, 7.13311, 9.97618	14.0641, 8	3	anapc1	2017
GL173197.1	747274	748851	747450, 747683, 747817, 748787	10, 12, 6, 6	8.16254, 8.05278, 4.80295, 5.33084	5.60312, 5.16604, 3.96416, 4.28587	5.53763, 5	3	slc24a3	46754
GL173198.1	365	674	626, 598, 585	13, 17, 9	10.29845, 12.06123, 8.01994	6.35212, 6.49348, 5.66309	7.51866, 9	3	tm6sf1	11021
GL173198.1	1355	4423	1804, 3773, 2445	9, 13, 8	6.06453, 7.50529, 6.57059	4.43343, 4.65031, 4.89275	3.6122, 5.0	3	tm6sf1	7272
GL173199.1	634417	634724	634590, 634570, 634626	8, 13, 7	7.67464, 11.2197, 8.21885	5.55832, 6.92276, 5.84717	5.08299, 8	3	slitrk5	23803
GL173200.1	212628	213325	213161, 213158, 213134	7, 13, 7	6.16939, 12.61502, 7.45266	4.73936, 7.77427, 5.46481	3.70916, 9	3	thdl18	9788
GL173200.1	218826	220674	219581, 219554, 219617	24, 28, 23	26.57606, 26.74919, 26.79607	13.07811, 11.76319, 13.53497	23.15164,	3	thdl18	15986
GL173201.1	19	740	365, 239, 306	13, 24, 13	13.07382, 26.28682, 15.89249	8.04786, 12.90575, 9.61177	10.13353,	3	pgat	26590
GL173201.1	7531	8140	8090, 8074, 8066	30, 44, 22	29.11778, 39.903, 22.53708	12.41795, 13.19278, 11.32496	25.63118,	3	pgat	19190
GL173201.1	550336	551145	550839, 550584, 550526	23, 51, 18	24.49582, 50.39705, 21.69448	12.17168, 15.92099, 11.95651	21.12838,	3	C7	0
GL173204.1	27262	27698	27318, 27317, 27317	23, 28, 17	28.76847, 33.56964, 21.88119	14.6664, 15.80476, 12.23429	25.29064,	3	ATP10B	103484
GL173204.1	429486	429998	429671, 429713, 429682	12, 10, 9	12.67544, 9.39128, 10.21256	7.98627, 6.35644, 6.90044	9.75458, 6	3	C1QTNF2	1662
GL173206.1	13980	14947	14667, 14818, 14278	15, 12, 8	15.71867, 9.82596, 9.92329	9.19755, 6.26981, 6.7228	12.65377,	3	NA	0
GL173206.1	233404	234166	233775, 233777, 233752	34, 36, 34	36.11374, 35.60603, 40.85612	14.94713, 13.85882, 17.74845	32.47832,	3	slc36a4	0
GL173206.1	278050	278396	278314, 278334, 278348	21, 41, 13	21.10247, 38.53997, 13.45746	10.7812, 13.56702, 8.27333	17.83755,	3	slc36a4	0
GL173206.1	280526	280932	280577, 280581, 280554	11, 34, 12	8.96891, 29.65157, 11.92663	5.93064, 11.30585, 7.55094	6.27876, 2	3	slc36a4	0
GL173206.1	320362	320899	320578, 320585, 320576	17, 35, 8	16.76548, 34.25995, 7.95096	9.33227, 13.48426, 5.71659	13.6543, 3	3	slc36a4	7953
GL173206.1	353967	354397	354359, 354338, 354362	21, 32, 26	22.48839, 35.31675, 32.88676	11.61323, 15.28753, 16.09579	19.18044,	3	mtnr1b	34280
GL173206.1	414968	415385	415157, 415113, 415089	6, 13, 5	4.45871, 9.67757, 5.80393	3.74614, 5.96749, 4.48186	2.26108, 7	3	mtnr1b	25420
GL173206.1	418253	418833	418345, 418466, 418290	12, 11, 5	10.73374, 7.88157, 4.77515	6.83135, 5.2484, 3.9592	7.92922, 5	3	mtnr1b	28705
GL173206.1	491000	491518	491126, 491069, 491092	4, 16, 9	3.63646, 16.15392, 10.72164	3.26116, 9.22204, 7.15493	1.6222, 13	3	fat3	98185
GL173206.1	499966	500446	500383, 500405, 500354	20, 19, 11	21.98313, 19.48976, 12.2694	11.61252, 10.41622, 7.88012	18.6897, 1	3	fat3	89257
GL173206.1	599177	599581	599347, 599356, 599339	5, 8, 6	4.50727, 6.62693, 6.25896	3.80664, 4.92781, 4.80589	2.30125, 4	3	fat3	0
GL173207.1	8540	9165	8941, 8957, 8963	31, 60, 25	31.56363, 61.96767, 26.08981	13.46809, 18.15562, 12.44125	28.02095,	3	rpl23	1218
GL173207.1	74056	74549	74223, 74097, 74100	16, 8, 8	13.97573, 5.40929, 8.74279	7.8982, 4.15294, 6.14792	10.98763,	3	pip4k2a	0
GL173207.1	187100	187729	187351, 187352, 187351	20, 32, 18	19.42489, 32.24067, 18.87511	10.07876, 13.47886, 10.32913	16.21503,	3	npepps	1022
GL173207.1	236819	237316	237069, 237092, 237126	7, 12, 10	6.64707, 10.08092, 13.00956	5.02073, 6.42828, 8.2622	4.15135, 7	3	NA	0
GL173207.1	279407	280299	279607, 279804, 279563	11, 15, 12	10.96672, 12.53666, 11.7126	7.14544, 7.2687, 7.42393	8.14942, 9	3	socs7	733
GL173207.1	297111	297414	297233, 297202, 297300	5, 16, 8	4.82177, 15.80758, 7.42597	3.98509, 9.01376, 5.41279	2.51418, 1	3	socs7	18437
GL173207.1	380694	381359	380953, 381333, 381273	11, 13, 6	12.43693, 11.66277, 5.91225	7.97018, 7.19557, 4.61907	9.52692, 8	3	srcin1	0
GL173207.1	441839	442188	442092, 442092, 442055	17, 17, 9	16.4953, 15.35073, 9.30116	9.16875, 8.4724, 6.41174	13.39926,	3	srcin1	0
GL173207.1	561974	562743	562290, 562497, 562497	18, 28, 11	18.07742, 29.57832, 11.03484	9.85073, 13.43449, 7.18523	14.9177, 2	3	srcin1	43137
GL173207.1	582160	583768	582797, 583020, 582739	7, 9, 8	6.60038, 7.43399, 8.80246	4.99378, 5.30221, 6.179	4.10761, 4	3	mllt6	29417
GL173208.1	79246	79715	79540, 79679, 79469	11, 19, 4	11.15818, 18.96889, 4.64325	7.25692, 10.10245, 3.77643	8.32008, 1	3	NA	0
GL173208.1	143827	144760	144067, 143973, 143973	8, 10, 10	7.67464, 7.8291, 10.42037	5.55832, 5.39387, 6.95441	5.08299, 5	3	NA	48067
GL173208.1	148258	149268	148833, 148810, 149140	7, 12, 8	6.69449, 9.24217, 8.56955	5.04797, 5.90582, 6.05652	4.1953, 6.6	3	NA	52498
GL173208.1	172688	173111	172819, 172801, 172802	5, 7, 9	4.02828, 5.97725, 10.28145	3.51866, 4.62287, 6.93568	1.88216, 3	3	5S_rRNA	31071
GL173208.1	264705	265268	264757, 264719, 264824	14, 21, 11	15.57853, 20.19181, 12.86886	9.31333, 10.23207, 8.19722	12.519, 17	3	5S_rRNA	59125
GL173208.1	381609	381977	381891, 381936, 381872	9, 11, 6	9.06007, 9.43766, 6.76586	6.27591, 6.22212, 5.06179	6.36394, 6	3	U4	29166
GL173208.1	397488	398169	397861, 397593, 397765	9, 7, 9	8.77314, 5.58561, 10.96118	6.111, 4.38027, 7.26984	6.09355, 3	3	U4	12974
GL173208.1	418072	418932	418247, 418504, 418456	4, 10, 4	3.74195, 9.17452, 4.34116	3.32091, 6.22617, 3.63492	1.6222, 6.6	3	U4	6768
GL173208.1	497338	497863	497531, 497464, 497591	6, 7, 8	5.81608, 5.45824, 8.35103	4.56563, 4.30004, 5.93881	3.4176, 3.2	3	5S_rRNA	16616
GL173208.1	516610	517868	517664, 517664, 516685, 517664	18, 20, 3, 12	19.51813, 22.87459, 3.47371, 16.36823	10.7103, 12.13503, 3.03189, 9.85365	16.30567,	3	5S_rRNA	35888
GL173208.1	590408	590988	590583, 590963, 590936	10, 15, 11	9.33595, 12.40077, 11.4314	6.32332, 7.18527, 7.41405	6.62299, 9	3	5S_rRNA	109686
GL173208.1	606207	606800	606272, 606282, 606484	5, 13, 6	4.82177, 11.76644, 6.82211	3.98509, 7.25915, 5.08889	2.51418, 9	3	or52d1	110105
GL173208.1	671555	672264	671780, 671944, 671968	8, 12, 6	6.86256, 10.68384, 6.6037	5.0733, 6.80072, 4.9822	4.3506, 8.0	3	or52d1	44641
GL173208.1	680183	680547	680501, 680209, 680477	7, 6, 5	7.1696, 4.93312, 5.9506	5.31345, 4.04501, 4.54784	4.61046, 2	3	or52d1	36358
GL173208.1	730914	731766	731763, 731737, 731736	28, 32, 21	30.98295, 32.56757, 29.35663	14.27172, 13.66912, 15.41596	27.45364,	3	or52d1	13054
GL173209.1	552812	553971	553580, 553323, 553321	11, 14, 7	12.43693, 14.22787, 8.69489	7.97018, 8.53137, 6.06379	9.52692, 1	3	card11	21943

GL173209.1	559476	560456	560000, 560029, 559996	9, 14, 8	9.72082, 14.45897, 10.1465	6.64182, 8.66788, 6.82176	6.97155, 1	3	card11	15458
GL173209.1	593768	596058	594237, 594231, 594315, 595077	14, 18, 14, 6	11.26858, 12.84507, 12.93312, 4.13532	6.7271, 6.70988, 7.7512, 3.53648	8.42534, 1	3	card11	14046
GL173209.1	597251	598299	597859, 597837, 597401	9, 15, 11	5.18738, 7.72326, 8.54035	3.87239, 4.43594, 5.66231	2.85031, 5	3	card11	17529
GL173209.1	604721	606076	604955, 605402, 604961	10, 18, 8	6.17333, 10.36918, 6.02227	4.34648, 5.33063, 4.54691	3.71266, 7	3	card11	24999
GL173209.1	607543	609321	607691, 608318, 609127	9, 19, 9	5.96058, 11.49636, 6.56209	4.36684, 5.73455, 4.75161	3.52158, 8	3	card11	27821
GL173209.1	607543	609321	608846, 608318, 609127	9, 19, 9	5.96058, 11.49636, 6.56209	4.36684, 5.73455, 4.75161	3.52158, 8	3	card11	27821
GL173209.1	615523	618795	617058, 617087, 616829, 618280	12, 16, 6, 5	11.92103, 14.14493, 6.5517, 5.50079	7.54764, 8.00171, 4.95623, 4.33868	9.04508, 1	3	card11	35801
GL173209.1	615523	618795	617795, 617087, 616829, 618280	7, 16, 6, 5	6.01305, 14.14493, 6.5517, 5.50079	4.6447, 8.00171, 4.95623, 4.33868	3.56347, 1	3	card11	35801
GL173209.1	615523	618795	618559, 617087, 616829, 618280	6, 16, 6, 5	4.9641, 14.14493, 6.5517, 5.50079	4.06411, 8.00171, 4.95623, 4.33868	2.64509, 1	3	card11	35801
GL173209.1	623966	627586	627168, 625388, 624011, 626144	21, 24, 10, 13	16.06857, 16.95987, 8.25787, 11.68143	7.79262, 7.49786, 5.66266, 7.20703	12.99028,	3	card11	44244
GL173209.1	632121	633236	632907, 632867, 632287	22, 23, 17	25.08108, 27.36982, 21.19882	12.84061, 13.8686, 11.87761	21.69836,	3	card11	52399
GL173209.1	767977	768491	768250, 768265, 768327	18, 24, 12	15.9393, 20.63686, 12.15007	8.55398, 9.54685, 7.68238	12.86597,	3	NA	60985
GL173210.1	62977	64276	63257, 63201, 63283, 63889	10, 21, 9, 21	4.82548, 10.3901, 5.11691, 17.56988	3.51126, 4.82344, 3.82754, 8.66481	2.5177, 7.7	3	NA	0
GL173210.1	72181	73138	72886, 72346, 72843	15, 17, 8	7.49768, 7.24481, 4.10077	4.31386, 3.90271, 3.30506	4.9177, 4.8	3	NA	0
GL173210.1	80761	82166	81125, 81103, 81978, 81138	14, 19, 19, 15	6.77332, 6.82427, 8.01429, 8.79397	4.07257, 3.48631, 4.01041, 5.03201	4.26931, 4	3	NA	0
GL173210.1	80786	82166	82031, 81978, 81138	14, 19, 15	7.86796, 8.01429, 8.79397	4.68519, 4.01041, 5.03201	5.26471, 5	3	NA	0
GL173210.1	179405	180664	180167, 180033, 179835	9, 18, 9	7.6342, 16.94269, 10.01275	5.42655, 9.16356, 6.79683	5.04509, 1	3	NA	26746
GL173210.1	186596	187217	186957, 186946, 186836	11, 13, 7	11.35776, 12.7389, 7.10639	7.37194, 7.84853, 5.27894	8.51017, 9	3	NA	20193
GL173210.1	365201	367751	366665, 366561, 366392	15, 28, 10	11.50631, 22.99352, 8.39789	6.63794, 9.63279, 5.74985	8.65136, 1	3	NA	8259
GL173210.1	629764	630064	629812, 629775, 629875	8, 10, 4	7.67464, 8.32357, 4.46488	5.55832, 5.70361, 3.69425	5.08299, 5	3	NA	20204
GL173210.1	671852	673198	672460, 672429, 672388	6, 12, 3	5.96715, 11.31437, 3.47371	4.64927, 7.18497, 3.03189	3.52158, 8	3	NA	17012
GL173211.1	30188	31168	30907, 30946, 30679	9, 13, 4	9.72082, 13.14514, 4.67325	6.64182, 8.09002, 3.78987	6.97155, 1	3	NA	79491
GL173211.1	531682	533069	532478, 532639, 531923	12, 11, 5	12.18267, 10.21061, 5.23257	7.70145, 6.69533, 4.20435	9.29304, 7	3	rab21	3865
GL173212.1	151841	152266	152084, 152117, 152207	8, 8, 7	7.33538, 5.64525, 7.10639	5.35908, 4.30526, 5.27894	4.76518, 3	3	pwwp2a	18046
GL173212.1	485225	486385	485681, 485985, 485940	16, 26, 9	14.18542, 23.24287, 8.34444	8.02648, 10.41222, 5.85853	11.18941,	3	uhrf1bp1l	112103
GL173212.1	696119	696338	696155, 696184, 696169	10, 6, 5	9.77513, 4.86158, 4.99237	6.5837, 4.00069, 4.0781	7.02302, 2	3	uhrf1bp1l	0
GL173213.1	699470	699990	699816, 699711, 699815	8, 13, 11	8.42257, 12.55412, 14.68355	5.97763, 7.73766, 9.06344	5.76336, 9	3	NA	48184
GL173214.1	24965	25353	25307, 25327, 25325	45, 75, 71	57.0677, 95.73493, 97.48138	22.35408, 28.83482, 32.07901	53.1168, 9	3	NA	3303
GL173214.1	43883	44191	43912, 43912, 43934	32, 58, 40	33.20449, 60.71044, 48.69077	14.04144, 18.3514, 19.75813	29.62708,	3	NA	0
GL173214.1	44859	45555	45515, 45542, 45510	19, 27, 17	16.03137, 20.8716, 15.58167	8.32714, 8.77893, 8.61317	12.95511,	3	NA	0
GL173214.1	178216	178509	178394, 178359, 178393	6, 9, 8	5.96715, 7.21043, 6.97106	4.64927, 5.1623, 5.13965	3.52158, 4	3	NA	47220
GL173214.1	180423	180807	180775, 180766, 180775	15, 31, 31	17.28737, 36.23515, 39.84799	10.09595, 16.23378, 18.35147	14.15669,	3	NA	49427
GL173214.1	198837	199337	199185, 199005, 199297	8, 11, 7	8.42257, 10.60895, 8.69489	5.97763, 6.9343, 6.06379	5.76336, 7	3	NA	67841
GL173214.1	271565	272095	271633, 271584, 271631	10, 16, 11	11.06011, 17.16856, 14.75772	7.306, 9.82359, 9.09568	8.22712, 1	3	NA	140569
GL173214.1	395130	395729	395314, 395271, 395329	9, 8, 6	8.50607, 7.07159, 7.29403	5.95453, 5.20073, 5.30581	5.84194, 4	3	NA	94780
GL173214.1	531601	531982	531841, 531701, 531817	22, 17, 17	23.80731, 16.17826, 19.99824	12.08624, 8.97632, 11.2232	20.45995,	3	NA	39477
GL173214.1	536848	537457	537231, 537118, 537234	11, 20, 8	10.3251, 19.30258, 8.4044	6.7644, 10.00482, 5.9678	7.54325, 1	3	NA	41697
GL173214.1	584079	584490	584122, 584111, 584343	11, 11, 5	12.43693, 10.60895, 5.9506	7.97018, 6.9343, 4.54784	9.52692, 7	3	NA	2831
GL173215.1	503551	504351	503661, 504126, 504145	21, 19, 11	17.56768, 13.70317, 10.61924	8.66352, 6.95836, 6.94042	14.42654,	3	scamp1	319
GL173215.1	511166	511558	511285, 511291, 511294	6, 6, 3	4.9641, 4.30294, 3.2112	4.06411, 3.64568, 2.90793	2.64509, 2	3	scamp1	0
GL173217.1	30865	31297	31271, 31282, 31271	63, 68, 50	79.49368, 80.61102, 66.26778	26.17816, 24.22171, 25.29529	75.30153,	3	NA	94041
GL173217.1	124561	125013	124979, 124964, 124967	15, 27, 6	15.56593, 31.67248, 6.93817	9.10751, 15.05014, 5.14396	12.50679,	3	NA	325
GL173217.1	163134	163893	163648, 163550, 163551	21, 43, 18	20.73342, 44.96478, 21.20939	10.5587, 16.27305, 11.68575	17.48034,	3	thap12	793
GL173217.1	171325	172013	171373, 171385, 171729	11, 20, 6	10.3251, 18.17471, 6.168	6.7644, 9.32366, 4.75778	7.54325, 1	3	thap12	1630
GL173217.1	190056	190488	190410, 190469, 190462	8, 8, 12	8.42257, 6.55376, 13.90113	5.97763, 4.88226, 8.66246	5.76336, 4	3	thap12	20361
GL173217.1	458044	458533	458245, 458490, 458244	11, 14, 7	10.1122, 11.71767, 7.20145	6.63573, 7.00416, 5.33074	7.34229, 9	3	thap12	288349
GL173217.1	600380	600758	600421, 600412, 600412	10, 28, 15	8.93828, 29.94002, 17.58264	6.08275, 13.64998, 10.25879	6.24946, 2	3	thap12	430685
GL173217.1	626340	626751	626608, 626536, 626529	8, 8, 4	6.70593, 6.11443, 3.48964	4.9768, 4.60556, 3.17588	4.20487, 3	3	thap12	456645
GL173217.1	636913	637614	637558, 637174, 637230	11, 10, 5	12.33515, 8.88645, 5.91431	7.91559, 6.0511, 4.53172	9.43699, 6	3	thap12	467218
GL173218.1	48766	49322	49237, 49192, 49037	7, 11, 3	7.1696, 10.60895, 3.47371	5.31345, 6.9343, 3.03189	4.61046, 7	3	NA	23658
GL173218.1	82282	82866	82722, 82800, 82729	12, 16, 16	10.58505, 14.25068, 18.3512	6.73997, 8.0664, 10.50324	7.78888, 1	3	gjd3	1172
GL173218.1	148872	149472	149114, 149069, 149023	4, 14, 3	3.51298, 13.8891, 3.17999	3.1896, 8.32957, 2.89245	1.55088, 1	3	rara	11827
GL173218.1	179178	179954	179309, 179518, 179616	11, 18, 6	10.9046, 17.58997, 6.76586	7.10903, 9.55621, 5.06179	8.08987, 1	3	rara	42133
GL173218.1	276086	276586	276484, 276471, 276556	14, 19, 18	14.99044, 19.64509, 21.69448	8.97777, 10.50948, 11.95651	11.95856,	3	wipf2	10358
GL173218.1	317038	317760	317434, 317098, 317120	19, 21, 20	17.4485, 18.27236, 22.4589	9.18194, 9.0807, 11.89249	14.31174,	3	rapgefl1	0
GL173218.1	345656	345935	345682, 345696, 345683	38, 58, 31	45.9404, 70.68876, 39.98318	19.03191, 23.76775, 18.42929	42.14754,	3	casc3	9464
GL173218.1	379551	380114	379892, 379777, 379714	5, 12, 7	4.17642, 10.2136, 7.40077	3.60969, 6.51056, 5.43746	2.01422, 7	3	msl1	5204
GL173218.1	491822	492215	492026, 492044, 492038	12, 10, 10	13.55951, 8.84031, 13.18184	8.47903, 6.02287, 8.33771	10.59689,	3	thra	0
GL173218.1	512715	513455	513029, 513133, 512985	32, 33, 22	33.83282, 32.41711, 25.21195	14.41056, 13.19841, 12.91737	30.24308,	3	thra	0
GL173218.1	537129	537664	537468, 537553, 537462	7, 11, 7	4.60029, 6.11322, 5.53602	3.74674, 4.15041, 4.34911	2.38171, 3	3	thra	0
GL173218.1	539398	539977	539929, 539948, 539927	34, 70, 35	38.56595, 85.48432, 44.52776	16.39199, 25.85153, 19.49295	34.88715,	3	thra	0
GL173218.1	569679	570238	570209, 570194, 570207	12, 26, 10	12.60209, 24.83666, 10.47831	7.9443, 11.34395, 6.98694	9.68421, 2	3	thra	13700
GL173218.1	637869	638191	637980, 637980, 637979	12, 22, 11	9.94519, 18.77934, 11.49087	6.34397, 9.07845, 7.44794	7.18444, 1	3	csf3	50652
GL173218.1	642586	643057	642786, 642913, 642795	20, 22, 11	19.15409, 18.32381, 10.52088	9.91507, 8.81177, 6.8818	15.95382,	3	csf3	45786
GL173218.1	649960	650295	650138, 650123, 650143	27, 34, 16	26.25209, 34.24377, 17.78134	11.82739, 13.86165, 10.17907	22.83518,	3	csf3	38548
GL173218.1	702892	703105	702945, 702931, 703000	11, 9, 12	12.43693, 8.21099, 15.80834	7.97018, 5.77858, 9.60504	9.52692, 5	3	csf3	10010
GL173219.1	661	1050	986, 859, 710	5, 10, 6	4.82177, 8.61706, 6.76586	3.98509, 5.8856, 5.06179	2.51418, 6	3	gpr78	92573
GL173219.1	202406	203356	203144, 202728, 202737	5, 17, 7	4.17642, 15.29522, 6.83761	3.60969, 8.43856, 5.12939	2.01422, 1	3	cpz	25826
GL173219.1	246312	246815	246657, 246776, 246670	10, 18, 9	9.77513, 15.53678, 8.98852	6.5837, 8.30996, 6.23511	7.02302, 1	3	cpz	69732
GL173219.1	397653	398822	398422, 397857, 398543	7, 20, 11	4.60029, 17.18461, 10.92742	3.74674, 8.72933, 7.12242	2.38171, 1	3	NA	5925
GL173219.1	425643	426082	425962, 425787, 426012	4, 9, 6	3.74195, 8.0603, 6.12365	3.32091, 5.68758, 4.73409	1.6222, 5.5	3	hmx1	8975
GL173219.1	565760	566289	565994, 566035, 565998	9, 6, 4	8.45482, 4.93312, 4.46488	5.92419, 4.04501, 3.69425	5.79307, 2	3	adra2c	1971
GL173219.1	744991	745489	745077, 745095, 745121	15, 13, 14	9.38678, 5.75783, 10.07548	5.37277, 3.65688, 5.99476	6.67051, 3	3	adra2c	181202
GL173219.1	747847	748300	748093, 748081, 748265	13, 21, 11	8.3376, 13.64182, 8.20847	5.14709, 6.4498, 5.45386	5.70205, 1	3	adra2c	184058
GL173219.1	754195	755299	754406, 754943, 754418, 754999	16, 19, 11, 7	14.97744, 16.10778, 11.20072, 6.2897	8.51025, 8.37295, 7.28154, 4.81137	11.94792,	3	adra2c	190406
GL173219.1	754344	755299	754884, 754943, 754418, 754999	6, 19, 11, 7	4.06322, 16.10778, 11.20072, 6.2897	3.4892, 8.37295, 7.28154, 4.81137	1.9144, 13	3	adra2c	190555
GL173220.1	38907	39564	39346, 39426, 39102	12, 13, 11	11.49331, 10.7674, 12.34038	7.29275, 6.64292, 7.91841	8.63898, 8	3	NA	412835
GL173220.1	52201	53371	52704, 52903, 52975	37, 68, 26	40.8506, 75.68718, 31.82009	16.47027, 21.63379, 15.48405	37.13474,	3	NA	399028
GL173220.1	64546	65335	65304, 65318, 65291	24, 39, 23	27.18719, 46.86901, 29.30255	13.44092, 19.13447, 14.96427	23.74699,	3	NA	387064
GL173220.1	199161	199502	199171, 199270, 199201	5, 10, 3	4.36883, 8.61706, 3.47371	3.72533, 5.8856, 3.03189	2.18201, 6	3	NA	252897
GL173220.1	243509	243896	243767, 243747, 243767	8, 11, 9	8.10827, 9.39347, 10.96118	5.80497, 6.19476, 7.26984	5.48761, 6	3	NA	208503
GL173220.1	273144	273783	273164, 273173, 273173	68, 88, 51	80.87902, 103.37126, 67.97697	24.36628, 26.15772, 25.79128	76.67492,	3	NA	178616
GL173220.1	297574	298176	298042, 298044, 297762	12, 14, 6	13.8483, 14.45897, 7.29403	8.63436, 8.66788, 5.30581	10.86694,	3	NA	154223
GL173220.1	308672	309028	308888, 309016, 309019	9, 17, 7	9.72082, 16.49924, 8.08824	6.64182, 9.17114, 5.7849	6.97155, 1	3	NA	143371
GL173220.1	398382	399319	398831, 398842, 399005	12, 10, 7	12.90199, 8.9331, 7.45266	8.11489, 6.07959, 5.46481	9.97033, 6	3	NA	53080
GL173220.1	443665	444262	443957, 443966, 444143	17, 36, 13	17.88146, 36.57807, 14.77384	10.00115, 14.41064, 9.01902	14.72916,	3	NA	8137
GL173220.1	473159	473543	473482, 473258, 473189	5, 6, 3	4.43696, 4.45754, 3.47371	3.76555, 3.74538, 3.03189	2.24017, 2	3	NA	18540
GL173221.1	332423	333228	332998, 332986, 332310, 333053	6, 11, 4, 4	5.90927, 9.66539, 4.67325, 4.59709	4.61743, 6.36265, 3.78987, 3.75554	3.50164, 7	3	nudt22	13340
GL173221.1	697645	698632	698406, 698138, 697750, 698149	26, 67, 11, 24	22.73294, 58.22506, 8.29649, 23.96688	10.11848, 14.01855, 5.50917, 11.51637	19.41738,	3	trmt112	19347
GL173222.1	27	759	468, 643, 535	9, 20, 13	8.35434, 21.01248, 14.0426	5.86444, 11.03495, 8.61056	5.71584, 1	3	U1	0
GL173222.1	8078	8811	8486, 8389, 8409	11, 16, 10	7.80634, 11.80086, 8.18963	5.20114, 6.57745, 5.62006	5.20581, 9	3	NA	0
GL173222.1	160489	160789	160587, 160612, 160581	5, 9, 8	4.17642, 7.67202, 10.0913	3.60969, 5.44992, 6.79758	2.01422, 5	3	ankrd55	0
GL173222.1	243013	243707	243481, 243522, 243417	5, 13, 6	4.69353, 11.41208, 5.43047	3.9134, 7.04141, 4.34463	2.45909, 8	3	ankrd55	42754
GL173222.1	347253	347572	347524, 347530, 347344	8, 10, 4	8.42257, 9.39128, 4.67325	5.97763, 6.35644, 3.78987	5.76336, 6	3	map3k1	89033
GL173222.1	557091	558836	558139, 558165, 557297	21, 32, 24	18.68517, 27.43773, 27.07887	9.32683, 10.77844, 13.37677	15.50082,	3	gpbp1	28348
GL173222.1	645625	646458	645901, 645905, 645894	13, 22, 5	14.26982, 25.49262, 5.59755	8.73911, 13.08144, 4.38538	11.26966,	3	fbxl16	3513
GL173223.1	230540	231284	231002, 230904, 231087	8, 20, 4	7.28955, 19.16931, 4.46488	5.33177, 9.92426, 3.69425	4.72176, 1	3	ric8a	0
GL173223.1	236665	237564	236962, 237198, 236961	9, 18, 8	8.30507, 17.00494, 9.31951	5.83501, 9.20137, 6.43944	5.67102, 1	3	ric8a	0
GL173223.1	328732	329301	328844, 328737, 328776	9, 22, 13	8.71822, 22.85945, 15.26294	6.07905, 11.51868, 9.28325	6.04275, 1	3	prmt1	19331
GL173223.1	638148	638651	638368, 638361, 638346	21, 27, 18	23.03475, 27.19383, 23.48722	11.93841, 12.3847, 12.91397	19.70924,	3	napsa	822
GL173224.1	279330	280424	280097, 279723, 279683	11, 13, 9	9.71398, 10.68145, 10.14484	6.39253, 6.58965, 6.86555	6.97155, 8	3	itga9	14737
GL173226.1	512634	513505	513270, 512785, 512874	9, 10, 6	6.57543, 8.24345, 5.91225	4.76012, 5.65367, 4.61907	4.08545, 5	3	lpar1	0
GL173227.1	223684	224602	224184, 224108, 224042	7, 9, 10	6.2098, 6.76868, 10.25108	4.76362, 4.88312, 6.85862	3.74679, 4	3	cabp4	55313
GL173227.1	613592	614567	614227, 614112, 614267	11, 11, 14	11.02968, 10.05535, 15.06595	7.18222, 6.6012, 9.02131	8.20839, 7	3	NA	119121
GL173228.1	151101	151684	151312, 151418, 151344	12, 14, 14	12.45843, 13.26509, 17.2091	7.86167, 7.95332, 10.19524	9.54648, 1	3	NA	58867
GL173228.1	172810	173101	172852, 172838, 172896	5, 20, 4	3.9718, 20.45393, 4.67325	3.48353, 10.69966, 3.78987	1.83149, 1	3	NA	37450
GL173228.1	233093	233531	233155, 233128, 233193	12, 12, 7	13.22036, 11.86108, 7.29952	8.29296, 7.51216, 5.38357	10.27237,	3	NA	5406
GL173228.1	260278	260893	260552, 260764, 260368	8, 8, 4	7.83097, 6.37769, 4.55201	5.64832, 4.77199, 3.73489	5.22917, 4	3	NA	14687
GL173228.1	312186	312801	312543, 312579, 312551	13, 23, 12	13.5657, 22.03663, 14.49372	8.33634, 10.69607, 8.97057	10.60274,	3	NA	50963
GL173228.1	345639	346143	345727, 345781, 345749	5, 5, 6	4.27048, 3.56671, 6.99807	3.6666, 3.22503, 5.17195	2.09564, 1	3	NA	17621
GL173228.1	362185	363078	362938, 362701, 362579	15, 20, 5	13.30906, 15.72353, 4.77515	7.74297, 7.86582, 3.9592	10.35786,	3	NA	686
GL173228.1	681117	682465	682217, 682166, 681568	26, 46, 30	23.81005, 44.04671, 32.53128	10.74163, 14.58028, 14.42712	20.46254,	3	NA	158045
GL173229.1	40248	41489	40639, 40694, 40666	18, 20, 15	17.51609, 18.71921, 17.40815	9.51146, 9.65224, 10.16282	14.37616,	3	ube2a	11834
GL173229.1	491488	493487	492043, 492421, 492158	33, 26, 22	32.59258, 22.50106, 22.40151	13.29899, 9.98574, 11.24343	29.02815,	3	utp14a	166662
GL173231.1	44631	45100	44882, 44888, 44885	6, 10, 4	5.81608, 7.97488, 4.59709	4.56563, 5.48553, 3.75554	3.4176, 5.5	3	NA	59615
GL173231.1	54559	54958	54905, 54799, 54873	13, 11, 11	13.20994, 9.17879, 14.03502	8.12823, 6.06147, 8.77076	10.26285,	3	NA	49757
GL173231.1	111796	112516	112204, 112181, 111900	5, 8, 6	4.82177, 6.81727, 7.29403	3.98509, 5.04548, 5.30581	2.51418, 4	3	NA	3875
GL173231.1	255564	256977	256446, 256021, 256098	9, 8, 4	9.18105, 7.07159, 4.67325	6.3444, 5.20073, 3.78987	6.47733, 4	3	NA	47590
GL173231.1	264367	267139	265089, 265043, 265061, 266272, 266964	25, 20, 11, 8, 6	22.25941, 14.38423, 9.51925, 6.38723, 4.50415	10.16982, 7.09595, 6.27256, 4.77798, 3.77524	18.9583, 1	3	NA	37428
GL173231.1	281353	281875	281646, 281393, 281613	5, 7, 5	4.61714, 5.97725, 5.80393	3.86998, 4.62287, 4.48186	2.39372, 3	3	NA	22692
GL173231.1	294324	294666	294424, 294535, 294658	5, 19, 7	4.5799, 20.55804, 7.96278	3.84863, 11.0538, 5.72394	2.3629, 17	3	NA	9901
GL173231.1	303052	303431	303252, 303216, 303230	6, 19, 8	5.55471, 17.89367, 8.24665	4.41698, 9.45168, 5.88165	3.18192, 1	3	NA	1136
GL173231.1	346072	346308	346262, 346270, 346277	18, 17, 10	19.69649, 16.97468, 13.11399	10.81516, 9.45853, 8.30815	16.47841,	3	NA	38888
GL173231.1	542134	543034	542631, 542565, 542536	6, 9, 9	5.96715, 8.21099, 10.87975	4.64927, 5.77858, 7.23113	3.52158, 5	3	NA	234950
GL173231.1	560460	560972	560757, 560706, 560642	7, 14, 8	6.94335, 12.75552, 9.11678	5.18876, 7.64262, 6.33924	4.42528, 1	3	NA	253276
GL173231.1	577723	578290	578243, 578089, 578250	15, 22, 12	16.9979, 23.10829, 14.23156	9.93422, 11.66803, 8.83588	13.87839,	3	NA	270539
GL173231.1	588218	588767	588224, 588245, 588224	79, 120, 89	100.24422, 150.6815, 119.83711	29.13559, 33.07804, 34.2548	95.8515, 1	3	NA	281034
GL173231.1	607939	608237	608102, 608200, 608213	6, 12, 15	5.3939, 11.84549, 18.74072	4.32314, 7.50292, 10.87493	3.0374, 9.1	3	NA	300755
GL173231.1	656880	657261	656881, 657003, 656896	15, 24, 13	14.98937, 24.5441, 16.38883	8.76428, 11.86236, 9.86104	11.95813,	3	NA	349696
GL173232.1	44864	45569	45393, 45379, 45343	18, 26, 10	19.34385, 25.92257, 10.30677	10.60746, 11.98756, 6.89026	16.13713,	3	mboat1	86834
GL173232.1	282690	283260	282833, 282845, 282839	34, 39, 20	36.78619, 40.85774, 23.16452	15.34154, 15.63093, 12.30292	33.13869,	3	e2f3	30904
GL173233.1	123	778	646, 457, 439	39, 51, 34	44.60531, 52.71627, 40.85612	17.80161, 17.12719, 17.74845	40.83193,	3	rad51ap1	7826
GL173233.1	121837	123075	122798, 122850, 122511, 123002	5, 8, 3, 4	4.82177, 6.44699, 3.47371, 4.67325	3.98509, 4.8155, 3.03189, 3.78987	2.51418, 4	3	fgf6	0
GL173233.1	153542	154497	154344, 153766, 154056	7, 13, 7	6.01305, 11.31501, 7.96278	4.6447, 6.98159, 5.72394	3.56347, 8	3	fgf6	23068
GL173233.1	169101	170071	169978, 169980, 169979	26, 57, 40	33.14758, 70.24516, 55.46154	16.2436, 24.02451, 23.71309	29.5713, 6	3	NA	10146
GL173233.1	218119	218900	218209, 218141, 218126	15, 29, 10	15.71867, 31.6555, 11.45478	9.19755, 14.2926, 7.51412	12.65377,	3	tigar	5596
GL173233.1	226677	227266	227123, 227190, 227220	14, 25, 9	15.23546, 26.60415, 11.30431	9.11858, 12.74915, 7.42891	12.19387,	3	ccnd2	11778
GL173233.1	343055	345540	345385, 343487, 345002, 343225	12, 6, 9, 4	10.20921, 4.53871, 8.01512, 4.67325	6.50784, 3.79731, 5.66016, 3.78987	7.4341, 2.4	3	ccnd2	66271
GL173233.1	370000	370602	370302, 370347, 370396	7, 9, 4	6.33443, 8.21099, 4.67325	4.83795, 5.77858, 3.78987	3.86259, 5	3	ccnd2	93216
GL173233.1	444186	444562	444307, 444328, 444408	12, 16, 5	13.82915, 15.94397, 5.50079	8.62415, 9.09593, 4.33868	10.85461,	3	ccnd2	167402
GL173233.1	464563	465060	464798, 464798, 464797	19, 27, 16	21.02475, 28.00888, 19.65507	11.32893, 12.86965, 11.21774	17.76283,	3	ccnd2	187779
GL173233.1	523219	523524	523256, 523291, 523331	10, 6, 6	11.06011, 4.93312, 7.25145	7.306, 4.04501, 5.287	8.22712, 2	3	ccnd2	246435
GL173233.1	547039	547632	547492, 547489, 547493	10, 12, 8	9.89241, 11.20359, 9.92329	6.65218, 7.11794, 6.7228	7.13431, 8	3	ccnd2	270255
GL173233.1	564213	564907	564656, 564431, 564706	12, 19, 8	12.05017, 19.96513, 8.9249	7.62377, 10.7011, 6.24211	9.16715, 1	3	ccnd2	287429
GL173233.1	581084	581768	581113, 581114, 581121	98, 117, 70	128.79736, 149.03912, 94.48956	33.92989, 33.83386, 30.92329	124.19077	3	ccnd2	304300
GL173233.1	603836	604407	604181, 604181, 604181	45, 45, 32	54.13096, 51.41798, 42.50305	20.62976, 19.05392, 19.50142	50.22035,	3	ccnd2	327052
GL173233.1	659336	660043	659598, 659598, 659597	14, 19, 12	15.31941, 21.4153, 14.76955	9.1665, 11.55717, 9.10943	12.27033,	3	ccnd2	382552
GL173233.1	665569	666045	665893, 665938, 665780	4, 7, 3	3.74195, 5.97725, 3.47371	3.32091, 4.62287, 3.03189	1.6222, 3.6	3	ccnd2	388785
GL173233.1	670934	671597	671389, 671503, 671445	15, 16, 7	18.26603, 17.16856, 8.69489	10.62691, 9.82359, 6.06379	15.09729,	3	ccnd2	394150
GL173233.1	677128	677689	677290, 677547, 677557	6, 7, 5	5.96715, 5.97725, 5.9506	4.64927, 4.62287, 4.54784	3.52158, 3	3	ccnd2	400344
GL173234.1	151726	152065	152013, 152019, 152020	82, 108, 57	95.48676, 125.33214, 70.25173	25.11515, 27.20031, 24.02827	91.13552,	3	MED19	0
GL173235.1	60	1256	665, 596, 676	50, 62, 52	50.1519, 61.35206, 63.217	16.19058, 17.06349, 22.50613	46.29769,	3	NA	18360
GL173235.1	473159	473925	473627, 473732, 473650	62, 60, 51	61.27156, 62.62027, 57.3075	17.02531, 18.48369, 19.62664	57.26914,	3	bach2	4665
GL173235.1	663567	663934	663744, 663646, 663743	6, 7, 5	5.96715, 5.93105, 5.9506	4.64927, 4.59459, 4.54784	3.52158, 3	3	bach2	148108
GL173237.1	59944	60266	60236, 60233, 60236	27, 27, 10	31.67873, 29.16563, 10.65691	15.05383, 13.55991, 7.08637	28.13373,	3	timp3	2859
GL173237.1	76677	77318	77053, 77271, 76679	6, 5, 5	5.96715, 3.94561, 5.45388	4.64927, 3.46715, 4.3157	3.52158, 1	3	timp3	0
GL173237.1	87559	87990	87933, 87947, 87932	15, 19, 10	17.28737, 19.48976, 13.18184	10.09595, 10.41622, 8.33771	14.15669,	3	timp3	0
GL173237.1	89383	89631	89473, 89443, 89501	4, 11, 3	3.57366, 9.4823, 3.47371	3.22499, 6.24973, 3.03189	1.59706, 6	3	timp3	0
GL173237.1	95346	96744	96381, 96243, 96395	25, 30, 20	25.81344, 28.94703, 21.49956	12.27579, 12.31903, 11.32573	22.40976,	3	timp3	0
GL173237.1	113731	114590	114131, 114317, 114151	18, 33, 8	15.7774, 26.83658, 7.11599	8.45576, 10.14525, 5.22758	12.70982,	3	SYN3	5242
GL173237.1	117990	118513	118466, 118469, 118497	19, 39, 16	17.4485, 34.29391, 16.76702	9.18194, 12.0582, 9.58727	14.31174,	3	SYN3	1319
GL173237.1	121727	122204	121978, 121938, 122104	6, 10, 9	4.83561, 6.40551, 8.69919	3.98453, 4.49295, 6.06795	2.52606, 4	3	SYN3	0
GL173237.1	130163	130700	130677, 130687, 130645	28, 49, 26	31.93238, 57.81544, 31.59632	14.83687, 20.85799, 15.35437	28.38246,	3	SYN3	0
GL173237.1	151246	151489	151334, 151302, 151359	13, 12, 7	12.26307, 9.66318, 7.55909	7.56188, 6.16843, 5.52035	9.36972, 7	3	rtcb	0
GL173237.1	251451	253038	251930, 251832, 251972	8, 10, 4	7.72597, 9.32635, 4.46488	5.588, 6.31756, 3.69425	5.13061, 6	3	NA	930
GL173237.1	260092	260674	260592, 260597, 260576	21, 17, 5	25.14429, 17.71433, 4.88115	13.1674, 9.9018, 4.01777	21.75899,	3	NA	2415
GL173237.1	284345	284578	284504, 284502, 284543	7, 13, 6	6.42049, 11.31501, 6.45058	4.8888, 6.98159, 4.90508	3.94161, 8	3	akr1c2	1046
GL173237.1	303011	303561	303527, 303531, 303517	30, 37, 22	42.18562, 49.07084, 31.78972	19.99491, 21.30315, 16.45634	38.44819,	3	XB-GENE-5836417	19085
GL173237.1	373141	373520	373373, 373293, 373288	5, 12, 3	4.82177, 11.86108, 3.47371	3.98509, 7.51216, 3.03189	2.51418, 9	3	akr1d1	0
GL173237.1	461127	461700	461131, 461168, 461239	18, 21, 9	20.55486, 22.14394, 10.21256	11.31361, 11.40723, 6.90044	17.30766,	3	trim24	5675
GL173237.1	540697	541592	541124, 541367, 540993	22, 18, 11	25.96118, 18.00717, 13.46208	13.35351, 9.80837, 8.49639	22.55288,	3	atp6v0a4	0
GL173237.1	546627	547263	546716, 546641, 546682	5, 11, 10	4.54329, 9.8562, 11.83521	3.82752, 6.47973, 7.70798	2.33, 7.257	3	atp6v0a4	2974
GL173237.1	554111	554352	554325, 554325, 554321	48, 53, 37	55.81601, 63.42943, 45.96839	20.18466, 22.13334, 19.48806	51.88229,	3	kiaa1549	0
GL173237.1	571065	571369	571208, 571208, 571152	12, 14, 6	13.22036, 12.64814, 7.29403	8.29296, 7.57684, 5.30581	10.27237,	3	kiaa1549	2417
GL173237.1	612977	613536	613512, 613196, 613445	6, 10, 5	5.90927, 8.66076, 5.14967	4.61743, 5.91256, 4.16141	3.50164, 6	3	hipk2	3582
GL173237.1	669142	669766	669708, 669714, 669662	18, 25, 15	16.33168, 21.09071, 17.76194	8.79227, 9.49621, 10.35658	13.24212,	3	tbxas1	24682
GL173237.1	670226	671257	670759, 670561, 670572	19, 20, 4	17.87921, 15.63797, 3.64768	9.44292, 7.81594, 3.26758	14.72728,	3	tbxas1	23191
GL173237.1	680941	681585	681209, 681200, 681298	20, 15, 6	23.85765, 15.80067, 7.29403	12.69961, 9.24574, 5.30581	20.50816,	3	tbxas1	12863
GL173238.1	198923	200007	199083, 199440, 199469	9, 23, 9	7.80169, 19.29427, 10.49552	5.52977, 9.0764, 7.0436	5.20141, 1	3	arhgap22	6859
GL173239.1	364982	365658	365299, 365021, 365262	11, 8, 6	12.01255, 7.07159, 7.29403	7.73996, 5.20073, 5.30581	9.13199, 4	3	NA	37332
GL173240.1	184471	185542	185007, 184490, 185292, 184613	7, 7, 8, 5	6.60038, 5.16276, 5.81313, 5.9506	4.99378, 4.11176, 4.41317, 4.54784	4.10761, 2	3	NA	46940
GL173240.1	193369	193980	193739, 193712, 193565	14, 18, 9	14.67861, 15.43521, 9.82249	8.79666, 8.2485, 6.69629	11.66087,	3	NA	55838
GL173240.1	424787	425432	425210, 425212, 425210	51, 73, 58	54.2141, 82.08238, 74.19445	17.92744, 22.59785, 25.77949	50.30209,	3	mal	1533
GL173241.1	26997	27951	27317, 27845, 27324	11, 8, 6	10.21742, 6.17826, 5.46454	6.69945, 4.64607, 4.36458	7.44132, 3	3	NA	42454
GL173241.1	46776	47226	46962, 47122, 46975	13, 21, 8	13.71465, 24.35402, 9.46129	8.42261, 12.71289, 6.50801	10.74495,	3	NA	23179
GL173241.1	146835	147559	147343, 147429, 147140	9, 19, 5	8.66407, 17.89367, 5.91431	6.04743, 9.45168, 4.53172	5.99101, 1	3	NA	17437
GL173241.1	175484	175885	175756, 175623, 175624	23, 42, 16	23.81148, 45.24856, 18.9725	11.76131, 16.85938, 10.84874	20.46387,	3	prdx3	2649
GL173241.1	190176	190499	190451, 190436, 190455	12, 18, 15	11.43506, 18.22482, 17.23823	7.25774, 9.93951, 10.06864	8.58315, 1	3	prdx3	17341
GL173241.1	197932	199572	198607, 199354, 199383	10, 23, 20	9.54985, 23.18973, 22.80441	6.45088, 11.38786, 12.09422	6.82341, 2	3	grk5	14023
GL173241.1	212546	213164	212992, 212995, 212988	10, 16, 11	11.06011, 16.22539, 12.48556	7.306, 9.26486, 7.99611	8.22712, 1	3	grk5	431
GL173241.1	246298	246701	246412, 246446, 246357	12, 15, 5	11.04522, 15.80067, 5.85847	7.02174, 9.24574, 4.50665	8.22317, 1	3	rgs10	4078
GL173241.1	269147	269587	269419, 269420, 269423	7, 18, 8	7.1696, 19.97713, 10.1465	5.31345, 10.97931, 6.82176	4.61046, 1	3	tial1	0
GL173241.1	278598	278900	278866, 278849, 278851	7, 22, 5	6.89193, 24.17682, 5.9506	5.15998, 12.30626, 4.54784	4.37772, 2	3	bag3	0
GL173241.1	286232	286791	286469, 286505, 286528	6, 5, 6	5.72604, 3.4564, 6.87953	4.51498, 3.15284, 5.11627	3.33547, 1	3	bag3	0
GL173241.1	300143	300682	300215, 300390, 300382	19, 23, 11	19.10984, 20.08442, 11.31465	10.1875, 9.53731, 7.3472	15.91125,	3	inpp5f	3905
GL173241.1	304850	305903	305426, 305327, 305174	4, 12, 8	3.74195, 9.70336, 7.81204	3.32091, 6.19346, 5.63749	1.6222, 7.1	3	inpp5f	0
GL173241.1	320773	321213	321181, 321176, 321188	12, 10, 5	12.3187, 9.39128, 5.9506	7.78073, 6.35644, 4.54784	9.4231, 6.8	3	inpp5f	0
GL173241.1	379974	380509	380287, 380271, 380092	6, 11, 5	5.31708, 9.61892, 4.42467	4.2777, 6.33404, 3.75832	2.96727, 7	3	mcmbp	706
GL173241.1	386983	387800	387417, 387538, 387734	10, 6, 4	10.01299, 4.93312, 4.38152	6.7221, 4.04501, 3.65448	7.24763, 2	3	mcmbp	0
GL173241.1	394567	395162	394924, 394857, 394801	8, 20, 5	8.42257, 20.45393, 5.9506	5.97763, 10.69966, 4.54784	5.76336, 1	3	sec23ip	0
GL173241.1	529883	530186	529985, 529991, 529929	5, 9, 5	4.73273, 7.43399, 5.80393	3.93547, 5.30221, 4.48186	2.49377, 4	3	CACUL1	8375
GL173241.1	547972	548921	548435, 548457, 548578	9, 9, 7	8.71822, 7.35794, 7.72582	6.07905, 5.25474, 5.6058	6.04275, 4	3	prlhr	4838
GL173241.1	569208	569870	569528, 569548, 569528	15, 16, 5	16.28407, 15.87543, 5.9506	9.52725, 9.05466, 4.54784	13.19656,	3	prlhr	14392
GL173241.1	600100	600542	600449, 600438, 600377	7, 17, 11	7.1696, 18.56115, 13.19894	5.31345, 10.40145, 8.36554	4.61046, 1	3	fam204a	39813
GL173241.1	610515	611141	611041, 610934, 611084	10, 14, 15	9.03421, 13.75886, 16.75372	6.14116, 8.2515, 9.79627	6.33915, 1	3	fam204a	29214
GL173241.1	611495	612042	611548, 611579, 611574	12, 10, 9	11.43506, 8.84031, 8.79325	7.25774, 6.02287, 6.12267	8.58315, 6	3	fam204a	28313
GL173241.1	619626	619972	619783, 619845, 619928	11, 16, 7	11.15818, 15.80758, 7.50542	7.25692, 9.01376, 5.49244	8.32008, 1	3	fam204a	20383
GL173241.1	626650	627275	626911, 626860, 627116	10, 20, 11	9.89241, 17.44765, 12.06254	6.65218, 8.88669, 7.76744	7.13431, 1	3	fam204a	13080
GL173241.1	660463	660801	660587, 660773, 660706	7, 10, 6	6.12952, 7.72315, 6.08001	4.71533, 5.32711, 4.71063	3.67231, 5	3	fam204a	1750
GL173241.1	671429	672634	672141, 672143, 672107	11, 18, 9	9.81032, 16.94269, 9.30116	6.45164, 9.16356, 6.41174	7.05644, 1	3	fam204a	12716
GL173242.1	181629	182558	181786, 182120, 181825	11, 27, 11	8.84943, 25.58706, 11.31465	5.85597, 11.43661, 7.3472	6.16564, 2	3	npffr2	29315
GL173242.1	184769	185210	185012, 185008, 184973	11, 10, 5	9.09169, 6.55702, 4.30991	6.00724, 4.58876, 3.69024	6.39391, 4	3	npffr2	32455
GL173242.1	202183	203419	202722, 202734, 203376	8, 21, 7	7.99469, 19.0595, 8.08824	5.74128, 9.55089, 5.7849	5.38155, 1	3	npffr2	49869
GL173242.1	215673	216336	215887, 216284, 216064	11, 15, 5	9.52723, 13.21475, 5.31857	6.27749, 7.68511, 4.24819	6.80251, 1	3	npffr2	63359
GL173242.1	224086	224714	224508, 224334, 224513	19, 24, 12	20.13535, 22.33212, 14.06375	10.80268, 10.54139, 8.74831	16.90088,	3	npffr2	71772
GL173242.1	604535	604929	604680, 604654, 604677	18, 22, 13	18.52782, 18.83145, 14.69628	10.1215, 9.10909, 8.97644	15.34982,	3	ankrd17	32
GL173243.1	492653	493373	492846, 492875, 493100	15, 20, 11	16.72085, 20.93036, 12.06254	9.7776, 10.98577, 7.76744	13.61562,	3	lrfn1	128224
GL173243.1	507633	509031	508315, 507686, 508646, 507774, 508554	19, 11, 23, 7, 12	18.67786, 7.30109, 19.97505, 6.36213, 12.50469	9.92659, 4.88441, 9.47317, 4.85436, 7.88834	15.49416,	3	lrfn1	143204
GL173244.1	98864	99650	99553, 99477, 98976	56, 71, 31	53.16864, 62.43759, 26.46652	15.40385, 14.51184, 10.57849	49.27171,	3	kiss1	39526
GL173245.1	41815	42050	41985, 41972, 41965	12, 20, 13	12.45843, 20.07667, 16.28606	7.86167, 10.47238, 9.81016	9.54648, 1	3	acer3	860
GL173245.1	135734	136321	136274, 136261, 136289	28, 62, 43	32.61062, 76.10716, 57.16365	15.23917, 24.78373, 23.35904	29.04561,	3	tsku	29714
GL173245.1	154812	155390	155079, 155276, 155238	13, 9, 5	15.1723, 7.97047, 5.9506	9.23486, 5.63299, 4.54784	12.13305,	3	NA	16756

GL173245.1	175427	176047	175911, 175911, 175731	12, 17, 5	11.32055, 13.9447, 4.33804	7.18871, 7.61716, 3.70703	8.47391, 1	3	NA	0
GL173245.1	194135	194785	194416, 194269, 194405	7, 9, 5	6.94335, 7.06866, 5.80393	5.18876, 5.07306, 4.48186	4.42528, 4	3	NA	0
GL173245.1	232253	232487	232308, 232386, 232299	7, 5, 5	5.19005, 3.65953, 4.20091	4.12926, 3.2852, 3.62458	2.85262, 1	3	lrrc32.1	11490
GL173245.1	234256	234784	234293, 234280, 234339	15, 12, 8	15.12865, 10.53798, 7.34578	8.84764, 6.71099, 5.36526	12.09208,	3	lrrc32.1	9193
GL173245.1	258428	259316	258447, 258467, 258505	24, 30, 17	24.93771, 31.67912, 19.72642	12.0984, 13.92147, 11.07071	21.55825,	3	lrrc32.1	12554
GL173245.1	280530	281185	280904, 280734, 280744	4, 6, 5	3.74195, 4.93312, 5.9506	3.32091, 4.04501, 4.54784	1.6222, 2.7	3	emsy	22291
GL173245.1	323842	324747	324130, 323944, 324485	8, 7, 8	7.28955, 4.05129, 7.07917	5.33177, 3.3854, 5.20531	4.72176, 1	3	emsy	0
GL173245.1	396279	396611	396604, 396586, 396583	10, 12, 9	9.77513, 10.63474, 9.52439	6.5837, 6.77054, 6.53516	7.02302, 7	3	NA	8382
GL173245.1	415619	416253	415743, 416027, 415899	6, 13, 8	5.96715, 13.14514, 9.76268	4.64927, 8.09002, 6.64964	3.52158, 1	3	NA	27722
GL173245.1	486769	487646	486893, 486995, 486944	29, 66, 15	32.23876, 68.91714, 17.23823	14.63993, 19.11735, 10.06864	28.68208,	3	NA	98872
GL173245.1	600778	601494	601211, 601161, 601141	8, 27, 10	8.42257, 27.11551, 12.61444	5.97763, 12.33821, 8.08336	5.76336, 2	3	wnt11	3493
GL173245.1	623499	624118	623907, 623902, 623944	7, 11, 7	7.1696, 10.60895, 8.21885	5.31345, 6.9343, 5.84717	4.61046, 7	3	wnt11	0
GL173245.1	633737	634692	634465, 634250, 634044	11, 18, 7	12.43693, 19.5052, 7.90186	7.97018, 10.70269, 5.69394	9.52692, 1	3	wnt11	0
GL173245.1	641454	642553	642403, 641884, 641608	11, 18, 8	9.17548, 14.36017, 7.11599	6.05941, 7.60184, 5.22758	6.47227, 1	3	wnt11	137
GL173245.1	645323	645610	645373, 645368, 645375	17, 16, 5	15.28145, 12.6954, 4.33804	8.43017, 7.11701, 3.70703	12.23845,	3	wnt11	4006
GL173245.1	659930	660676	659932, 659981, 660053	12, 13, 9	11.55224, 10.31133, 10.01275	7.3281, 6.36011, 6.79683	8.69537, 7	3	wnt11	18613
GL173246.1	104060	104464	104442, 104136, 104314	9, 11, 8	6.42583, 5.58861, 6.93578	4.66464, 3.83409, 5.11812	3.94676, 3	3	NA	40600
GL173246.1	136935	137260	137200, 137207, 137140	11, 15, 7	8.61979, 11.37503, 5.32289	5.71214, 6.55803, 4.21415	5.94958, 8	3	LRRC3C	33160
GL173246.1	140011	140413	140304, 140388, 140380	11, 15, 11	8.92872, 10.56806, 9.7601	5.90554, 6.07049, 6.42085	6.24102, 7	3	LRRC3C	30007
GL173246.1	206647	207524	207054, 207066, 207052	38, 53, 29	37.5312, 55.36779, 31.26155	14.15925, 17.68667, 14.05808	33.86901,	3	LRRC3C	35443
GL173246.1	338212	338822	338322, 338332, 338330	43, 43, 26	48.79427, 46.20208, 32.16442	18.45184, 16.96807, 15.68273	44.95878,	3	NA	0
GL173246.1	357265	357688	357506, 357519, 357491	14, 20, 9	10.82413, 15.59554, 7.54596	6.45331, 7.79123, 5.37187	8.01418, 1	3	grb7	0
GL173246.1	360897	361183	360972, 360954, 361073	24, 27, 13	21.49774, 22.17456, 12.54936	10.0491, 9.48858, 7.73479	18.2198, 1	3	grb7	2876
GL173246.1	395178	395731	395251, 395458, 395356	6, 12, 5	5.09949, 11.48527, 5.64747	4.14694, 7.28792, 4.40911	2.76939, 8	3	mien1	9138
GL173246.1	409643	410276	410087, 410181, 410073	13, 18, 11	12.26307, 14.67054, 8.89958	7.56188, 7.78766, 5.88733	9.36972, 1	3	mien1	698
GL173246.1	412242	412770	412348, 412457, 412297	10, 12, 9	8.20198, 8.72259, 6.8329	5.62778, 5.58178, 4.92389	5.57541, 6	3	NA	694
GL173246.1	544488	544801	544610, 544542, 544532	15, 20, 10	14.8531, 21.26374, 11.997	8.68248, 11.18517, 7.78835	11.82783,	3	pnmt	3911
GL173246.1	560385	560681	560397, 560488, 560502	6, 10, 4	5.86227, 9.39128, 4.67325	4.59138, 6.35644, 3.78987	3.46055, 6	3	NA	2621
GL173246.1	578990	579249	579018, 579192, 579199	9, 10, 10	8.71822, 8.0878, 11.38247	6.07905, 5.55635, 7.47651	6.04275, 5	3	stard3	0
GL173246.1	649736	650382	650211, 650190, 650240	26, 23, 16	28.19417, 21.83526, 18.69936	13.34394, 10.57561, 10.69793	24.73016,	3	ppp1r1b	21927
GL173246.1	660710	660988	660908, 660842, 660868	15, 21, 13	13.36183, 21.11333, 15.34872	7.77533, 10.78774, 9.3288	10.40776,	3	neurod2	18689
GL173247.1	150122	150369	150234, 150233, 150241	169, 162, 85	214.9938, 200.33121, 110.64519	37.41884, 34.70028, 31.49652	209.93105	3	usp15	0
GL173248.1	96078	96546	96245, 96182, 96172	17, 18, 16	10.59442, 8.10003, 12.36386	5.65259, 4.18132, 6.91618	7.79798, 5	3	NA	0
GL173248.1	97604	98222	97738, 98096, 98097	16, 27, 9	9.57404, 15.32776, 5.57157	5.28151, 6.04854, 4.11768	6.84664, 1	3	NA	0
GL173248.1	97612	98270	98155, 98096, 98097	26, 27, 9	19.41264, 15.32776, 5.57157	8.27769, 6.04854, 4.11768	16.20334,	3	NA	0
GL173248.1	549761	550136	549864, 549837, 549898	7, 10, 4	7.1696, 9.39128, 4.67325	5.31345, 6.35644, 3.78987	4.61046, 6	3	nxpe1	20616
GL173250.1	320794	321916	321648, 321697, 321696	22, 20, 10	23.7162, 21.0954, 12.16489	12.03186, 11.08457, 7.87042	20.37115,	3	fbln7	14381
GL173250.1	415640	416133	415803, 415843, 416050	6, 10, 6	4.56865, 8.01217, 6.87953	3.81639, 5.50894, 5.11627	2.35429, 5	3	mertk	0
GL173250.1	498512	498889	498533, 498529, 498770	14, 21, 8	13.44866, 20.39593, 9.31951	8.06455, 10.35516, 6.43944	10.49073,	3	dzank1	0
GL173250.1	629870	631169	630492, 630336, 630576	6, 11, 8	5.31708, 7.97598, 7.5499	4.2777, 5.30773, 5.48567	2.96727, 5	3	NA	1959
GL173251.1	71691	72086	71795, 71799, 71790	12, 19, 9	10.44071, 17.64648, 9.41119	6.65101, 9.30188, 6.47287	7.65237, 1	3	phka2	18069
GL173251.1	429159	430097	429651, 429415, 429673	9, 15, 11	9.72082, 13.4765, 14.24187	6.64182, 7.84563, 8.86619	6.97155, 1	3	btk	0
GL173251.1	593695	594117	593983, 593978, 594022	13, 9, 6	14.43911, 8.21099, 6.6037	8.83395, 5.77858, 4.9822	11.43182,	3	faah2	4694
GL173252.1	56078	56409	56347, 56379, 56176	14, 19, 11	12.97522, 17.52577, 12.19945	7.77689, 9.22875, 7.8422	10.03988,	3	NA	2779
GL173252.1	139211	139585	139285, 139272, 139352	9, 9, 5	9.12009, 7.92635, 5.9506	6.30997, 5.60609, 4.54784	6.42028, 5	3	NA	22949
GL173252.1	150317	150920	150678, 150734, 150766	15, 11, 11	18.26603, 10.60895, 14.03502	10.62691, 6.9343, 8.77076	15.09729,	3	NA	34055
GL173252.1	270298	270827	270610, 270768, 270546	12, 23, 10	11.15354, 21.19364, 10.7802	7.08759, 10.19296, 7.15425	8.31649, 1	3	NA	4464
GL173252.1	286283	286754	286383, 286384, 286562	23, 26, 13	22.01139, 21.2576, 12.81733	10.68097, 9.28371, 7.8954	18.71671,	3	NA	0
GL173252.1	344694	345234	344937, 344813, 344937	9, 10, 3	9.36986, 8.04981, 3.30894	6.44997, 5.53254, 2.9554	6.65425, 5	3	NA	9118
GL173252.1	482685	483492	482805, 482689, 482724	5, 19, 7	4.54329, 18.48026, 8.69489	3.82752, 9.80703, 6.06379	2.33, 15.49	3	NA	1447
GL173252.1	566627	567173	566862, 566948, 566991	8, 12, 8	6.20993, 9.99443, 8.04677	4.66614, 6.37457, 5.77057	3.74679, 7	3	NA	18541
GL173252.1	614807	615400	615252, 614882, 614975	6, 9, 5	5.68215, 6.26311, 4.36653	4.49007, 4.56059, 3.72397	3.29732, 3	3	NA	5167
GL173253.1	496491	496687	496539, 496537, 496636	9, 13, 6	7.80169, 12.09006, 6.76586	5.52977, 7.45678, 5.06179	5.20141, 9	3	NA	3136
GL173253.1	569599	570013	569809, 569852, 569783	9, 16, 5	8.45482, 12.48972, 5.59755	5.92419, 6.99232, 4.38538	5.79307, 9	3	NA	0
GL173253.1	581097	581783	581615, 581476, 581258	6, 11, 3	5.3939, 8.74006, 3.47371	4.32314, 5.78751, 3.03189	3.0374, 6.2	3	NA	9949
GL173254.1	189628	189953	189882, 189782, 189899	21, 16, 13	15.58521, 8.88286, 9.95131	7.51782, 4.8983, 6.13691	12.5256, 6	3	zmiz1	8594
GL173254.1	191761	193154	192614, 192310, 192655	29, 50, 23	24.47403, 47.80022, 21.40794	10.13246, 14.98925, 10.32054	21.10728,	3	zmiz1	10727
GL173255.1	480149	480603	480382, 480535, 480197	5, 7, 5	4.50727, 5.75319, 4.39541	3.80664, 4.48487, 3.74107	2.30125, 3	3	slc7a2.1	35082
GL173256.1	30676	31063	30845, 30837, 30855	16, 21, 19	19.7934, 23.60416, 24.98385	11.29109, 12.27478, 13.52596	16.57067,	3	NA	35447
GL173257.1	183568	184358	184124, 183951, 183868	9, 19, 10	5.18738, 9.53566, 7.02444	3.87239, 4.72946, 4.88493	2.85031, 6	3	NA	NA
GL173257.1	185387	185987	185610, 185545, 185598	11, 15, 9	6.87051, 6.31264, 5.94272	4.61612, 3.69426, 4.35539	4.35824, 3	3	NA	NA
GL173257.1	187019	187964	187514, 187625, 187241	30, 47, 25	27.3368, 36.88765, 23.62611	11.39713, 10.84807, 10.97213	23.89199,	3	NA	NA
GL173257.1	562030	562957	562562, 562588, 562272	8, 13, 3	7.99469, 12.25946, 3.30894	5.74128, 7.55969, 2.9554	5.38155, 9	3	NA	NA
GL173258.1	69	1407	778, 568, 731	6, 9, 4	5.96715, 7.55109, 4.67325	4.64927, 5.37505, 3.78987	3.52158, 5	3	NA	139368
GL173258.1	11791	12403	11821, 11963, 12131	6, 7, 6	5.20598, 5.19095, 6.25896	4.2113, 4.12984, 4.80589	2.86702, 2	3	NA	128372
GL173258.1	13869	15570	14728, 14431, 14886	6, 9, 5	5.96715, 8.19918, 5.69847	4.64927, 5.77148, 4.4331	3.52158, 5	3	NA	125205
GL173258.1	31413	31872	31530, 31648, 31524	6, 7, 3	5.96715, 5.97725, 3.47371	4.64927, 4.62287, 3.03189	3.52158, 3	3	NA	108903
GL173258.1	39799	40642	40059, 40263, 40313	6, 6, 7	5.16999, 4.4049, 6.75301	4.18963, 3.71155, 5.08141	2.83325, 2	3	NA	100133
GL173258.1	99562	99810	99601, 99654, 99658	5, 7, 4	4.47183, 4.67552, 3.88239	3.78598, 3.79595, 3.39841	2.26935, 2	3	NA	40965
GL173258.1	102381	103762	102609, 102929, 102201, 102993	7, 16, 5, 4	6.79162, 15.10361, 4.99237, 4.15126	5.10336, 8.58708, 4.0781, 3.54016	4.28406, 1	3	NA	37013
GL173258.1	144178	144734	144182, 144183, 144208	23, 28, 11	24.06243, 26.47393, 11.20072	11.91191, 11.60281, 7.28154	20.70741,	3	NA	0
GL173258.1	156137	157201	156906, 156796, 156870	10, 15, 6	8.40516, 12.05281, 5.46454	5.75437, 6.97189, 4.36458	5.76336, 9	3	NA	8668
GL173258.1	263428	264044	263596, 263853, 263522	4, 8, 6	3.51298, 6.37769, 6.30561	3.1896, 4.77199, 4.83031	1.55088, 4	3	tff1	0
GL173258.1	292417	292747	292718, 292722, 292706	19, 20, 9	21.31193, 21.01248, 9.46739	11.49694, 11.03495, 6.50386	18.04063,	3	TMPRSS3	0
GL173258.1	297894	298390	298233, 297993, 297998	6, 7, 7	4.93136, 5.13485, 6.79502	4.04392, 4.09383, 5.10529	2.61555, 2	3	TMPRSS3	4856
GL173258.1	383436	384234	383656, 383775, 383798	7, 19, 10	7.1696, 18.48026, 10.1418	5.31345, 9.80703, 6.79622	4.61046, 1	3	bcl9	56968
GL173258.1	398602	398847	398702, 398769, 398746	11, 10, 4	10.78278, 7.05193, 3.56693	7.03733, 4.90236, 3.22108	7.97565, 4	3	bcl9	42355
GL173258.1	402961	403226	403012, 402970, 402987	6, 13, 7	4.22823, 9.60796, 6.92462	3.59714, 5.92446, 5.17829	2.0602, 7.0	3	bcl9	37976
GL173258.1	404855	405277	404930, 404893, 405053	8, 10, 5	6.48409, 6.50583, 4.99237	4.83873, 4.55637, 4.0781	4.00135, 4	3	bcl9	35925
GL173258.1	435062	435386	435094, 435133, 435139	8, 12, 10	6.24296, 11.54355, 12.16489	4.68703, 7.32289, 7.87042	3.77872, 8	3	bcl9	5816
GL173258.1	456491	456718	456496, 456529, 456551	5, 6, 7	4.73273, 4.56637, 7.10639	3.93547, 3.81494, 5.27894	2.49377, 2	3	bcl9	0
GL173258.1	480796	481102	481080, 481050, 481073	10, 13, 6	9.60506, 11.92581, 6.45058	6.48358, 7.35664, 4.90508	6.87658, 9	3	gja5	3634
GL173258.1	510170	510630	510461, 510431, 510255	13, 14, 3	15.29169, 13.8891, 3.47371	9.29854, 8.32957, 3.03189	12.24396,	3	gja5	24338
GL173258.1	541802	542778	542698, 542501, 542576	12, 19, 11	13.13892, 17.83113, 11.99553	8.24771, 9.41378, 7.73059	10.1962, 1	3	gja8	37005
GL173258.1	575602	576198	576125, 576155, 576087	8, 10, 5	8.22568, 9.39128, 5.80393	5.8701, 6.35644, 4.48186	5.59681, 6	3	gja8	3585
GL173259.1	62983	63521	63381, 63431, 63064	12, 8, 8	12.18267, 6.44699, 9.31951	7.70145, 4.8155, 6.43944	9.29304, 4	3	NA	30163
GL173260.1	199157	199367	199159, 199193, 199158	10, 20, 25	10.39648, 17.83099, 33.68896	6.94096, 9.11676, 16.82945	7.61003, 1	3	cbln2	141131
GL173261.1	1009	1474	1087, 1057, 1072	6, 9, 8	5.96715, 7.59096, 9.31951	4.64927, 5.39978, 6.43944	3.52158, 5	3	mitf	14597
GL173261.1	16614	17140	17016, 17015, 17016	14, 27, 15	13.96392, 28.52801, 15.88211	8.37431, 13.17929, 9.29347	10.9769, 2	3	mitf	0
GL173261.1	198251	198643	198532, 198384, 198388	11, 19, 6	11.85906, 17.06093, 6.25896	7.65504, 8.94736, 4.80589	8.98595, 1	3	XB-GENE-5822220	0
GL173261.1	398787	399224	398935, 398878, 398912	8, 7, 8	8.42257, 5.97725, 10.0913	5.97763, 4.62287, 6.79758	5.76336, 3	3	XB-GENE-5822220	168184
GL173261.1	438052	438534	438064, 438078, 438078	36, 53, 36	43.33179, 61.12123, 46.86716	18.3642, 20.82445, 20.44196	39.57723,	3	XB-GENE-5822220	207449
GL173261.1	468454	468706	468519, 468501, 468508	5, 8, 3	4.82177, 6.17826, 3.17999	3.98509, 4.64607, 2.89245	2.51418, 3	3	XB-GENE-5822220	237851
GL173261.1	534097	535088	534153, 534109, 534168	12, 25, 11	11.15354, 26.2541, 11.99553	7.08759, 12.53961, 7.73059	8.31649, 2	3	XB-GENE-5822220	303494
GL173261.1	534097	535220	534848, 534109, 534168	13, 25, 11	11.98086, 26.2541, 11.99553	7.39024, 12.53961, 7.73059	9.10256, 2	3	XB-GENE-5822220	303494
GL173262.1	108270	109231	108453, 109051, 108566	15, 15, 13	11.58458, 10.37501, 13.33541	6.68565, 5.955, 8.20195	8.72487, 7	3	NA	10020
GL173262.1	120400	120649	120620, 120517, 120464	7, 9, 8	6.01305, 6.67403, 9.05167	4.6447, 4.82293, 6.30653	3.56347, 4	3	NA	20940
GL173262.1	121644	121981	121812, 121841, 121671	10, 14, 6	9.33595, 11.71767, 6.45058	6.32332, 7.00416, 4.90508	6.62299, 9	3	NA	19608
GL173262.1	229007	229990	229634, 229181, 229446	7, 12, 5	7.1696, 9.90946, 5.9506	5.31345, 6.32175, 4.54784	4.61046, 7	3	NA	0
GL173262.1	375048	375999	375154, 375189, 375868, 375129, 375751	18, 22, 19, 10, 8	12.7397, 15.25055, 12.1862, 7.6817, 5.6948	6.64898, 7.0789, 6.10753, 5.30096, 4.33715	9.81664, 1	3	NA	42255
GL173262.1	376655	377086	376893, 376998, 376727	20, 18, 12	14.52575, 11.29454, 9.7119	7.1761, 5.8325, 6.19878	11.51483,	3	NA	41168
GL173262.1	387138	387551	387522, 387516, 387502	10, 24, 16	11.04401, 24.70533, 21.65777	7.29736, 11.95904, 12.22781	8.22236, 2	3	NA	30703
GL173262.1	501328	502053	501766, 501780, 501523	7, 11, 6	7.1696, 10.05535, 7.29403	5.31345, 6.6012, 5.30581	4.61046, 7	3	slamf8	0
GL173262.1	572979	573288	573125, 573063, 573020	8, 13, 6	5.89711, 9.71274, 5.75442	4.46697, 5.98923, 4.53101	3.49291, 7	3	NA	44154
GL173262.1	585901	586296	586205, 586194, 586151	10, 12, 7	10.53228, 11.86108, 8.02489	7.01712, 7.51216, 5.75426	7.73927, 9	3	NA	31146
GL173263.1	161708	162078	161839, 161930, 161870	15, 11, 5	12.70921, 8.27243, 4.60894	7.37467, 5.49405, 3.8653	9.7865, 5.7	3	NA	10611
GL173263.1	384137	384832	384308, 384771, 384541	5, 6, 5	4.82177, 4.76927, 5.31857	3.98509, 3.9431, 4.24819	2.51418, 2	3	ivd	14309
GL173264.1	144	1479	1329, 942, 1011	19, 27, 19	13.62865, 17.95903, 17.5517	6.91567, 7.28135, 9.24446	10.66325,	3	actn3	26802
GL173264.1	3168	4782	3925, 4262, 3902	13, 28, 10	7.82724, 18.96884, 7.33926	4.84097, 7.5414, 5.08445	5.22612, 1	3	actn3	23499
GL173264.1	10422	11017	10513, 10889, 10841	8, 13, 10	8.42257, 13.14514, 13.18184	5.97763, 8.09002, 8.33771	5.76336, 1	3	actn3	17264
GL173264.1	16042	17488	16461, 16708, 16914	24, 47, 18	21.20631, 41.71935, 16.26613	9.87836, 13.06103, 8.75244	17.93797,	3	actn3	10793
GL173264.1	429695	430886	430021, 430098, 430055	14, 22, 11	14.16942, 21.85303, 12.63525	8.49669, 10.91337, 8.07536	11.17387,	3	sart1	4801
GL173264.1	585025	586609	585740, 586041, 585234, 586355	17, 13, 10, 14	11.13538, 7.14051, 8.36243, 10.88612	5.95833, 4.43687, 5.7278, 6.49145	8.29923, 4	3	rce1	130777
GL173264.1	588313	589139	588522, 588539, 588507	27, 34, 14	18.60525, 24.74518, 10.1337	7.60214, 8.82788, 6.03025	15.42458,	3	rce1	134065
GL173264.1	590964	591282	591189, 591058, 591030	13, 20, 9	6.53162, 11.0674, 5.8994	4.08738, 5.31959, 4.32763	4.04487, 8	3	rce1	136716
GL173264.1	599911	600933	599980, 600892, 600169	11, 14, 10	5.27859, 6.93048, 6.01389	3.6501, 4.1587, 4.2462	2.9335, 4.5	3	rce1	145663
GL173264.1	601518	602382	602000, 601984, 601809	49, 51, 33	47.48785, 47.37846, 32.28815	15.20877, 14.41681, 13.12462	43.67101,	3	rce1	147270
GL173264.1	608829	609776	608935, 608945, 608906	18, 25, 8	12.67071, 15.74645, 5.6948	6.6092, 6.64351, 4.33715	9.75044, 1	3	rce1	154581
GL173264.1	613004	613537	613223, 613425, 613217	15, 21, 17	8.57024, 10.08741, 14.39501	4.9053, 4.68347, 7.89032	5.90296, 7	3	rce1	158756
GL173264.1	617464	619185	618540, 618520, 618581	41, 41, 21	45.04856, 39.11414, 24.4673	17.17942, 13.87242, 12.77852	41.26878,	3	rce1	163216
GL173266.1	43530	43887	43633, 43622, 43621	16, 27, 11	16.30427, 29.07231, 11.92945	9.31205, 13.5042, 7.69409	13.21554,	3	eogt	203806
GL173266.1	89835	90246	90131, 90088, 90110	93, 129, 79	113.23794, 154.8815, 100.54393	28.59248, 30.67971, 29.29909	108.74047	3	eogt	157447
GL173266.1	131804	132465	131928, 131913, 131908	26, 31, 19	26.20487, 31.94142, 23.01756	12.15566, 13.68968, 12.47022	22.78872,	3	eogt	115228
GL173266.1	216578	217148	216753, 216754, 216860	13, 23, 9	14.10549, 24.95167, 11.21577	8.64628, 12.44425, 7.38849	11.11201,	3	eogt	30545
GL173266.1	233107	233570	233224, 233174, 233161	11, 21, 13	11.6377, 18.11284, 14.39632	7.53109, 8.98591, 8.81006	8.77589, 1	3	eogt	14123
GL173266.1	257773	258137	258108, 258073, 258087	7, 8, 8	7.1696, 7.07159, 9.05167	5.31345, 5.20073, 6.30653	4.61046, 4	3	eogt	0
GL173266.1	280210	281087	280646, 280686, 280772	12, 31, 9	10.39353, 29.6897, 8.69919	6.62187, 12.38109, 6.06795	7.60856, 2	3	eogt	4434
GL173266.1	292926	293316	293060, 292976, 292985	10, 24, 10	9.28413, 21.07552, 11.10454	6.29221, 9.80196, 7.32977	6.574, 18.0	3	tmf1	0
GL173266.1	318210	318644	318339, 318449, 318488	8, 19, 12	7.33538, 18.75575, 14.23156	5.35908, 9.97369, 8.83588	4.76518, 1	3	tmf1	0
GL173266.1	374909	375315	374938, 374949, 374939	57, 49, 38	68.85737, 56.08739, 49.34066	23.23359, 19.8694, 21.0335	64.77254,	3	uba3	28583
GL173266.1	417646	418418	418241, 418174, 418190	9, 17, 6	8.0679, 18.28171, 6.08001	5.69219, 10.23765, 4.71063	5.45, 15.30	3	arl6ip5	3497
GL173266.1	428084	428599	428157, 428176, 428296	18, 15, 6	16.74626, 13.69427, 5.83216	9.04422, 7.97895, 4.57461	13.64034,	3	lmod3	1057
GL173266.1	445880	446465	446034, 446036, 446033	23, 49, 34	27.72716, 60.567, 43.83181	14.07465, 22.45398, 19.49562	24.27442,	3	lmod3	4342
GL173266.1	541044	541324	541074, 541054, 541092	42, 50, 26	45.37831, 55.98822, 31.59632	16.93323, 19.34968, 15.35437	41.5938, 5	3	frmd4b	0
GL173266.1	551808	552383	552045, 552063, 552118	10, 18, 6	10.81731, 17.86551, 6.6037	7.17456, 9.72285, 4.9822	8.00764, 1	3	frmd4b	0
GL173266.1	557504	557823	557523, 557532, 557517	50, 72, 56	60.53185, 83.59547, 70.19845	21.94127, 23.84322, 24.51129	56.53857,	3	frmd4b	1443
GL173266.1	574767	576142	575880, 575331, 575785	22, 41, 13	19.28187, 37.78176, 12.1463	9.3748, 13.16839, 7.49099	16.07739,	3	frmd4b	18706
GL173266.1	578842	579571	579237, 579260, 579388	19, 23, 10	16.44012, 16.72294, 9.26679	8.5727, 7.62665, 6.28179	13.34655,	3	frmd4b	22781
GL173267.1	962	1627	1473, 1358, 1602	9, 6, 8	9.72082, 4.93312, 10.1465	6.64182, 4.04501, 6.82176	6.97155, 2	3	NA	12005
GL173267.1	38748	39820	39126, 39531, 39578	11, 13, 8	10.84329, 10.43179, 8.35103	7.073, 6.43483, 5.93881	8.0324, 7.8	3	NA	24434
GL173267.1	99195	100227	99709, 99842, 99492	6, 17, 7	4.83561, 14.31774, 6.71158	3.98453, 7.84337, 5.05776	2.52606, 1	3	NA	8322
GL173267.1	187911	188877	188478, 188565, 188478	14, 21, 8	13.09001, 18.38042, 7.59222	7.84687, 9.14502, 5.5104	10.14895,	3	NA	33040
GL173267.1	370574	371962	371257, 371493, 371243	8, 13, 12	8.34715, 11.36332, 13.29687	5.9367, 7.01137, 8.33527	5.71011, 8	3	NA	2303
GL173267.1	396237	396507	396303, 396315, 396458	6, 9, 4	5.96715, 7.88274, 4.67325	4.64927, 5.57944, 3.78987	3.52158, 5	3	NA	7277
GL173267.1	428076	428597	428129, 428431, 428350	7, 10, 4	7.1696, 9.07627, 4.38152	5.31345, 6.1667, 3.65448	4.61046, 6	3	NA	0
GL173267.1	455033	456220	455525, 455184, 455519	16, 16, 8	15.60786, 11.58732, 7.59222	8.89312, 6.44989, 5.5104	12.5472, 8	3	NA	7788
GL173267.1	485371	486888	485831, 486554, 485862	11, 13, 5	7.86853, 8.49752, 4.51488	5.2402, 5.24388, 3.81106	5.26511, 5	3	NA	7607
GL173267.1	485371	486888	486520, 486554, 485862	9, 13, 5	5.85972, 8.49752, 4.51488	4.30221, 5.24388, 3.81106	3.45931, 5	3	NA	7607
GL173268.1	591294	593524	591930, 592249, 591944	9, 12, 8	7.03134, 9.42893, 8.14528	5.04951, 6.02236, 5.82558	4.50586, 6	3	tmem132e	204195
GL173268.1	591758	593524	593340, 592249, 591944	7, 12, 8	5.55587, 9.42893, 8.14528	4.36159, 6.02236, 5.82558	3.18254, 6	3	tmem132e	204659
GL173268.1	599603	601973	601573, 599987, 601794, 600493	12, 17, 8, 19	7.75782, 15.07756, 3.97927, 19.75671	4.98431, 8.30587, 3.22691, 10.5764	5.16173, 1	3	tmem132e	212504
GL173268.1	605196	606784	605979, 605766, 605534, 606263	12, 14, 10, 6	7.37376, 8.35803, 7.45013, 4.38967	4.74941, 4.96842, 5.15463, 3.70174	4.80231, 5	3	tmem132e	218097
GL173268.1	606530	608609	607373, 605766, 607787, 607343	15, 14, 13, 11	11.31512, 8.35803, 9.11457, 10.71972	6.5216, 4.96842, 5.62052, 7.00004	8.46949, 5	3	tmem132e	219431
GL173268.1	613587	615611	614328, 614036, 614897	8, 16, 12	8.42257, 17.16856, 14.23156	5.97763, 9.82359, 8.83588	5.76336, 1	3	tmem132e	226488
GL173269.1	40711	41695	41668, 41612, 41663	15, 10, 8	17.48779, 9.39128, 8.80246	10.20672, 6.35644, 6.179	14.34959,	3	slc35a3.2	21283
GL173269.1	123710	124165	124128, 124118, 124130	53, 63, 30	68.35755, 72.56681, 36.97385	24.98856, 22.33293, 17.05765	64.2778, 6	3	GRAMD1A	11253
GL173269.1	130973	131592	131041, 130991, 130998	14, 28, 14	16.43009, 29.14146, 15.20637	9.78349, 13.17452, 9.10193	13.33671,	3	GRAMD1A	3826
GL173269.1	157515	158431	157608, 158162, 158079	5, 17, 6	4.69353, 16.56523, 6.35308	3.9134, 9.21112, 4.85498	2.45909, 1	3	GRAMD1A	5408
GL173269.1	157515	158431	158394, 158162, 158079	9, 17, 6	9.50098, 16.56523, 6.35308	6.52233, 9.21112, 4.85498	6.77745, 1	3	GRAMD1A	5408
GL173269.1	197004	197503	197143, 197144, 197257	9, 12, 7	8.71822, 10.39632, 7.10639	6.07905, 6.6236, 5.27894	6.04275, 7	3	GRAMD1A	44897
GL173269.1	222909	223189	223142, 223170, 223137	18, 11, 19	17.2505, 10.60895, 22.81433	9.35044, 6.9343, 12.35676	14.12166,	3	GRAMD1A	70802
GL173269.1	289462	289698	289663, 289691, 289647	13, 18, 19	12.20544, 17.58997, 23.22595	7.52692, 9.55621, 12.5858	9.31472, 1	3	NA	132004
GL173269.1	310783	311250	310920, 311059, 310972	8, 14, 9	7.15567, 14.45897, 10.56949	5.25151, 8.66788, 7.08032	4.61046, 1	3	NA	110452
GL173269.1	382750	383496	383436, 383183, 383163	7, 10, 5	5.93788, 8.04981, 4.80993	4.59878, 5.53254, 3.97854	3.52158, 5	3	NA	38206
GL173269.1	406991	407816	407063, 407178, 407065, 407619	7, 17, 8, 5	5.7233, 13.67787, 7.67847, 4.14842	4.4663, 7.45588, 5.56054, 3.59261	3.33547, 1	3	NA	13886
GL173269.1	411342	411827	411579, 411778, 411792	10, 19, 16	9.03421, 15.67257, 17.32802	6.14116, 8.11255, 9.91673	6.33915, 1	3	NA	9875
GL173269.1	458602	459193	459051, 458878, 458760	6, 5, 3	5.51356, 3.61259, 3.47371	4.39314, 3.25484, 3.03189	3.14574, 1	3	NA	0
GL173269.1	477991	478531	478497, 478493, 478467	15, 29, 16	16.54256, 33.43586, 21.39033	9.6759, 15.35187, 12.09963	13.44484,	3	NA	0
GL173269.1	485444	486143	485676, 485668, 485978	7, 11, 6	7.1696, 9.7598, 7.29403	5.31345, 6.42066, 5.30581	4.61046, 7	3	NA	0
GL173269.1	496235	496745	496674, 496513, 496442	26, 37, 20	29.72008, 40.08397, 23.16452	14.2526, 16.02291, 12.30292	26.219, 36	3	NA	0
GL173269.1	521980	522516	522027, 522066, 522009	30, 36, 23	34.36701, 38.35513, 27.70251	15.5187, 15.43492, 14.06048	30.76642,	3	NA	0
GL173269.1	573614	574414	573849, 573957, 573992	6, 9, 10	4.7134, 7.75488, 11.997	3.90801, 5.501, 7.78835	2.47723, 5	3	NA	0
GL173272.1	31385	32251	32057, 32076, 32146	26, 56, 24	23.87327, 55.93557, 25.51625	10.77851, 16.7527, 12.44511	20.52357,	3	atp8a2	70616
GL173272.1	136576	137339	136993, 137004, 136615	5, 7, 7	4.82177, 5.82304, 7.96278	3.98509, 4.52813, 5.72394	2.51418, 3	3	atp8a2	0
GL173273.1	27432	28042	27713, 27614, 27750	18, 9, 9	20.35565, 6.76868, 9.8849	11.19891, 4.88312, 6.72947	17.11452,	3	cyp7b1	0
GL173273.1	196502	197281	196779, 196695, 196861	10, 16, 5	9.49535, 14.30421, 5.14967	6.41851, 8.09914, 4.16141	6.77344, 1	3	armc1	65667
GL173274.1	610	1581	741, 650, 638	15, 25, 21	17.80052, 29.44854, 30.02716	10.37752, 14.43793, 15.74085	14.65143,	3	KIAA1328	12021
GL173274.1	57296	58059	57917, 57400, 57387	6, 8, 9	5.55471, 6.21066, 8.34444	4.41698, 4.66659, 5.85853	3.18192, 3	3	KIAA1328	0
GL173274.1	126171	126676	126550, 126273, 126515	6, 7, 4	5.96715, 5.97725, 4.59709	4.64927, 4.62287, 3.75554	3.52158, 3	3	tpgs2	1929
GL173274.1	198648	199113	198952, 198686, 198701	10, 11, 6	9.661, 10.60895, 6.6037	6.51661, 6.9343, 4.9822	6.9283, 7.9	3	aqp3	1497
GL173274.1	221071	221514	221300, 221187, 221338	12, 7, 6	9.30462, 4.89556, 5.46454	5.94479, 3.93927, 4.36458	6.59363, 2	3	aqp3	2321
GL173274.1	228991	229851	229264, 229438, 229421	5, 10, 11	4.50727, 8.32357, 13.19894	3.80664, 5.70361, 8.36554	2.30125, 5	3	aqp3	10241
GL173274.1	407974	408364	408291, 408259, 408351	16, 20, 15	14.79948, 18.1164, 17.85348	8.40174, 9.28853, 10.40618	11.77649,	3	dcaf12	0
GL173274.1	428187	429610	429254, 429359, 428667, 429261	13, 13, 3, 10	13.5657, 11.56107, 3.17999, 12.16489	8.33634, 7.13311, 2.89245, 7.87042	10.60274,	3	ubap1	9818
GL173274.1	438608	439126	439049, 438909, 438908	10, 9, 6	11.06011, 8.21099, 6.6567	7.306, 5.77858, 5.00845	8.22712, 5	3	ubap1	302
GL173274.1	539915	540420	540133, 540132, 540326	9, 15, 9	6.76373, 11.56639, 7.06537	4.87998, 6.67456, 5.07099	4.25982, 8	3	nudt2	42937
GL173274.1	541990	542342	542023, 542022, 542018	20, 29, 28	21.61731, 30.10341, 32.87535	11.39575, 13.37008, 15.39573	18.33537,	3	nudt2	45012
GL173274.1	552255	552900	552319, 552320, 552320	16, 22, 14	19.7934, 25.85271, 18.05504	11.29109, 13.29074, 10.62048	16.57067,	3	il11ra	47938
GL173274.1	602252	602918	602469, 602469, 602470	10, 18, 11	10.53228, 16.6391, 11.6121	7.01712, 8.9791, 7.51665	7.73927, 1	3	il11ra	0
GL173274.1	627846	628110	627987, 628006, 627996	16, 14, 8	15.87778, 12.33756, 8.14528	9.05608, 7.38613, 5.82558	12.8057, 9	3	il11ra	0
GL173275.1	98444	99330	98956, 98956, 98955	33, 59, 35	31.32862, 57.50198, 39.50447	12.57962, 16.36422, 16.52752	27.79117,	3	dsn1	67763
GL173275.1	103515	103850	103610, 103608, 103685	9, 18, 6	6.82879, 13.23994, 5.11235	4.92128, 6.93954, 4.15475	4.31954, 1	3	dsn1	72834
GL173275.1	135846	136673	135974, 135929, 135850	9, 7, 4	8.35434, 5.45824, 4.67325	5.86444, 4.30004, 3.78987	5.71584, 3	3	olfm2	71721
GL173275.1	191452	191800	191597, 191548, 191725	5, 6, 5	4.82177, 4.76927, 5.9506	3.98509, 3.9431, 4.54784	2.51418, 2	3	olfm2	16594
GL173275.1	289431	289916	289591, 289580, 289469	5, 9, 7	4.54329, 7.47261, 8.49722	3.82752, 5.32627, 5.97582	2.33, 5.031	3	olfm2	34812
GL173275.1	320812	321927	321694, 321620, 321583	26, 62, 16	23.19798, 64.3429, 16.90298	10.38628, 18.51614, 9.66757	19.86744,	3	olfm2	66193
GL173275.1	377482	377985	377887, 377666, 377659	20, 33, 20	22.97335, 38.81641, 26.22416	12.19234, 16.95528, 13.9932	19.64963,	3	olfm2	122863
GL173275.1	438940	439995	439385, 439373, 439355	33, 33, 21	35.06587, 32.72552, 24.00714	14.73656, 13.37535, 12.51097	31.45152,	3	pin1	147156
GL173275.1	453636	454159	453658, 453706, 453651	14, 31, 18	12.28014, 32.55021, 21.39981	7.35082, 14.04807, 11.79257	9.38613, 2	3	pin1	132992
GL173275.1	512210	512851	512463, 512340, 512720	7, 13, 6	6.89193, 11.12608, 6.87953	5.15998, 6.86492, 5.11627	4.37772, 8	3	pin1	74300
GL173275.1	579521	580588	579754, 580274, 580360	9, 7, 6	7.47395, 5.97725, 7.29403	5.32711, 4.62287, 5.30581	4.89626, 3	3	pin1	6563
GL173275.1	592615	593488	593007, 592977, 593038	18, 23, 10	20.16146, 22.6679, 11.38247	11.08652, 11.0745, 7.47651	16.9259, 1	3	pin1	730
GL173276.1	179651	179878	179798, 179821, 179810	9, 11, 7	7.55321, 9.13706, 8.02489	5.37637, 6.0355, 5.75426	4.96981, 6	3	ldb1	20117
GL173276.1	322551	323281	322621, 323058, 322814	13, 25, 11	11.30985, 22.30103, 10.5698	6.9784, 10.19404, 6.91098	8.46461, 1	3	pprc1	67979
GL173277.1	139033	139403	139187, 139354, 139200	9, 15, 4	9.36986, 14.52325, 4.67325	6.44997, 8.48354, 3.78987	6.65425, 1	3	ext1	0
GL173277.1	168151	169093	168430, 168708, 168701	8, 12, 7	7.77807, 10.88526, 7.10639	5.618, 6.92415, 5.27894	5.17956, 8	3	ext1	23650
GL173277.1	180391	181086	181024, 181040, 181060	63, 122, 60	78.69691, 152.36023, 76.23772	25.72569, 32.85951, 25.90328	74.51289,	3	ext1	35890
GL173277.1	215372	215919	215874, 215848, 215532	11, 9, 5	11.15818, 8.21099, 5.80393	7.25692, 5.77858, 4.48186	8.32008, 5	3	ext1	70871
GL173277.1	419681	420066	419914, 419913, 419912	11, 13, 7	9.95877, 12.03475, 8.21885	6.54239, 7.4231, 5.84717	7.19682, 9	3	ext1	275180
GL173277.1	458204	458633	458377, 458520, 458349	7, 14, 9	5.79309, 11.49628, 10.56949	4.5096, 6.86757, 7.08032	3.39779, 8	3	ext1	313703
GL173277.1	472701	473181	473038, 472993, 473027	9, 11, 11	8.40425, 9.43766, 13.46208	5.89416, 6.22212, 8.49639	5.76336, 6	3	ext1	328200
GL173277.1	476496	477121	477068, 476758, 476641	8, 6, 6	6.34412, 4.4049, 6.35308	4.75087, 3.71155, 4.85498	3.871, 2.29	3	ext1	331995
GL173277.1	494322	494858	494831, 494834, 494847	41, 52, 36	48.2721, 54.31823, 41.69891	19.05636, 17.55507, 17.39833	44.4445, 5	3	ext1	349821
GL173277.1	542123	542434	542142, 542187, 542152	45, 46, 24	53.46264, 55.08362, 27.07887	20.23946, 20.71057, 13.37677	49.56168,	3	ext1	397622
GL173277.1	553984	554475	554423, 554113, 554137	5, 9, 6	4.20732, 8.10603, 5.60548	3.62846, 5.71528, 4.44624	2.04024, 5	3	ext1	409483
GL173278.1	257520	258075	257768, 257735, 257644	8, 7, 4	7.02691, 5.78791, 4.30163	5.17362, 4.5064, 3.61556	4.50222, 3	3	slc4a4	0
GL173279.1	52399	52775	52642, 52642, 52642	28, 21, 20	31.93238, 21.57397, 27.64436	14.83687, 11.06508, 14.71524	28.38246,	3	nlgn4x	219679
GL173279.1	261326	261523	261356, 261332, 261342	13, 19, 11	12.49994, 17.89367, 13.0311	7.70504, 9.45168, 8.28053	9.58678, 1	3	nlgn4x	10931
GL173279.1	588525	592553	589245, 588432, 589352, 591598, 588567, 590238	18, 10, 14, 19, 7, 11	13.90855, 4.3083, 7.41969, 13.14951, 4.89532, 9.2906	7.33312, 3.20092, 4.43079, 6.6433, 3.93912, 6.13097	10.92411,	3	U3	0
GL173280.1	3566	3814	3696, 3781, 3766	7, 9, 5	7.1696, 8.21099, 5.9506	5.31345, 5.77858, 4.54784	4.61046, 5	3	NA	284487
GL173280.1	10744	11430	11353, 11394, 11365	5, 14, 8	4.43696, 14.09002, 9.1831	3.76555, 8.44949, 6.37229	2.24017, 1	3	NA	276871
GL173280.1	143330	143733	143694, 143702, 143683	11, 17, 10	11.29029, 16.49924, 11.60305	7.3332, 9.17114, 7.59048	8.44541, 1	3	NA	144568

GL173280.1	236362	236671	236629, 236634, 236551	9, 12, 7	9.06007, 11.25867, 7.55909	6.27591, 7.1513, 5.52035	6.36394, 8	3	NA	51630
GL173280.1	366702	367289	366990, 367187, 366783	7, 5, 6	7.10297, 3.94561, 6.5517	5.27706, 3.46715, 4.95623	4.57191, 1	3	NA	58893
GL173280.1	447904	448366	448024, 447963, 448052	4, 6, 3	3.74195, 4.73925, 3.47371	3.32091, 3.92426, 3.03189	1.6222, 2.5	3	NA	80984
GL173280.1	579171	580020	579526, 579515, 579740	16, 18, 13	19.18518, 19.97713, 15.89249	10.96495, 10.97931, 9.61177	15.98366,	3	NA	47667
GL173281.1	8	1135	380, 549, 551	89, 140, 104	92.4086, 140.40742, 115.2216	20.71171, 21.42815, 24.5087	88.08655,	3	NA	42608
GL173281.1	6652	7386	6777, 6783, 6783	30, 49, 19	30.52291, 47.45062, 17.7091	13.23888, 15.18978, 9.33983	27.00341,	3	NA	36357
GL173281.1	45197	45892	45420, 45411, 45411	11, 20, 8	11.85906, 22.87459, 10.0913	7.65504, 12.13503, 6.79758	8.98595, 1	3	NA	0
GL173281.1	51735	52477	52032, 52084, 52080	35, 64, 43	39.83972, 74.48753, 57.47857	16.7256, 22.88599, 23.54311	36.13961,	3	NA	3113
GL173281.1	70759	71094	71058, 70905, 70932	15, 15, 10	15.41679, 13.92002, 11.997	9.0192, 8.11689, 7.78835	12.36344,	3	NA	0
GL173281.1	97076	97518	97265, 97277, 97299	9, 13, 7	7.39636, 11.51094, 8.69489	5.27874, 7.10228, 6.06379	4.82212, 8	3	tbc1d10b	4250
GL173281.1	122334	122859	122822, 122654, 122818	11, 10, 9	9.95877, 9.028, 9.70054	6.54239, 6.13739, 6.63089	7.19682, 6	3	tbc1d10b	3666
GL173281.1	157120	157836	157780, 157746, 157737	8, 12, 4	7.33538, 9.95176, 4.67325	5.35908, 6.34805, 3.78987	4.76518, 7	3	dctpp1	2543
GL173281.1	311162	311476	311329, 311342, 311227	12, 14, 11	11.92103, 10.57816, 12.34038	7.54764, 6.30219, 7.91841	9.04508, 7	3	fbrs	1514
GL173281.1	380631	381126	380950, 380772, 380939	13, 13, 7	12.09198, 13.14514, 8.69489	7.45795, 8.09002, 6.06379	9.20698, 1	3	pus3	2236
GL173281.1	433757	434790	434334, 434317, 434338	22, 33, 17	20.92422, 30.97311, 18.88718	10.35482, 12.3796, 10.59105	17.66578,	3	rnf25	936
GL173281.1	462493	463302	462798, 462515, 463085	7, 8, 9	7.1696, 7.07159, 11.39481	5.31345, 5.20073, 7.46977	4.61046, 4	3	armc5	8379
GL173281.1	471194	471617	471407, 471467, 471357	10, 12, 6	10.39648, 9.90946, 5.91225	6.94096, 6.32175, 4.61907	7.61003, 7	3	armc5	64
GL173281.1	495804	496651	496162, 496478, 496160	14, 10, 7	12.97522, 7.68845, 8.69489	7.77689, 5.30522, 6.06379	10.03988,	3	tgfb1i1	0
GL173281.1	508961	509327	509139, 509242, 509100	4, 12, 6	3.39747, 10.53798, 5.91225	3.12111, 6.71099, 4.61907	1.46102, 7	3	tgfb1i1	4680
GL173281.1	589939	591900	591090, 591661, 591052	7, 12, 4	6.09019, 9.46718, 4.50795	4.69155, 6.04622, 3.71446	3.63504, 6	3	mss51	0
GL173281.1	590513	591900	591694, 591661, 591052	6, 12, 4	4.99728, 9.46718, 4.50795	4.08451, 6.04622, 3.71446	2.67584, 6	3	mss51	0
GL173282.1	120048	120555	120217, 120250, 120313	9, 11, 3	9.72082, 9.4823, 3.47371	6.64182, 6.24973, 3.03189	6.97155, 6	3	homer3	4150
GL173282.1	586395	587396	587254, 587236, 586895	15, 14, 10	16.54256, 13.82362, 11.6791	9.6759, 8.29035, 7.62925	13.44484,	3	hook3	15297
GL173283.1	529470	529927	529880, 529890, 529883	10, 8, 5	8.66311, 6.27644, 5.9506	5.914, 4.70819, 4.54784	5.99101, 3	3	sass6	30885
GL173283.1	564085	564610	564336, 564357, 564125	18, 18, 10	18.84377, 16.06714, 11.6791	10.31044, 8.63158, 7.62925	15.65357,	3	sass6	65500
GL173283.1	586199	587047	586398, 586258, 586828, 586263, 586870	19, 16, 13, 10, 5	18.9632, 13.44943, 10.23256, 9.82971, 4.28214	10.09902, 7.57638, 6.31126, 6.61563, 3.6736	15.76968,	3	sass6	87614
GL173285.1	57301	57544	57456, 57463, 57346	16, 10, 7	13.52743, 7.1111, 6.55126	7.62404, 4.93988, 4.96529	10.56607,	3	rfx1	0
GL173285.1	106369	106977	106958, 106930, 106933	60, 91, 56	72.79102, 106.75105, 71.15837	23.94049, 26.37894, 25.06692	68.66759,	3	rln3	18542
GL173285.1	187536	188361	187912, 187638, 188060	6, 6, 5	4.9641, 4.379, 5.85847	4.06411, 3.69486, 4.50665	2.64509, 2	3	clpp	14849
GL173285.1	189300	190512	189939, 189619, 190060	9, 9, 4	8.66407, 7.35794, 4.46488	6.04743, 5.25474, 3.69425	5.99101, 4	3	clpp	12698
GL173285.1	307022	307659	307520, 307163, 307299	11, 9, 5	12.01255, 8.21099, 5.9506	7.73996, 5.77858, 4.54784	9.13199, 5	3	cacna1a	2761
GL173285.1	328677	329072	329041, 329041, 328935	7, 12, 4	5.55587, 9.74388, 3.29862	4.36159, 6.2187, 3.06133	3.18254, 7	3	cacna1a	0
GL173285.1	333715	334111	334041, 334041, 334041	23, 32, 35	21.67593, 28.06502, 37.72517	10.48041, 11.11877, 15.48081	18.39184,	3	cacna1a	0
GL173285.1	335339	335757	335755, 335704, 335706	19, 38, 29	16.87204, 35.67579, 30.47277	8.83319, 13.14036, 13.58917	13.75724,	3	cacna1a	0
GL173285.1	503619	503935	503757, 503800, 503854	6, 7, 3	5.3939, 5.71889, 3.47371	4.32314, 4.46356, 3.03189	3.0374, 3.4	3	nacc1	0
GL173285.1	517685	518419	518134, 518000, 517971	7, 9, 6	6.50909, 5.89579, 6.6567	4.94073, 4.32532, 5.00845	4.02371, 3	3	TRMT1	0
GL173285.1	574454	575659	575092, 574731, 575198	8, 10, 12	7.77807, 8.32357, 14.40488	5.618, 5.70361, 8.92522	5.17956, 5	3	dhps	0
GL173286.1	91499	92019	91759, 91671, 91898	20, 20, 8	20.76674, 19.23568, 9.46129	10.88765, 9.96438, 6.50801	17.51241,	3	NFKBID	26583
GL173286.1	137641	138049	137988, 137896, 138008	9, 11, 5	8.16099, 7.78921, 4.67395	5.74847, 5.19038, 3.90232	5.53692, 5	3	nphs1	0
GL173286.1	154823	155414	155331, 155339, 155334	7, 26, 16	6.94335, 28.49224, 19.86305	5.18876, 13.52193, 11.32783	4.42528, 2	3	nphs1	1315
GL173286.1	191760	192142	192142, 192095, 192113	11, 16, 6	9.95877, 12.53032, 5.46454	6.54239, 7.01691, 4.36458	7.19682, 9	3	kirrel2	23588
GL173286.1	192520	193132	192792, 192765, 192945	10, 25, 10	9.03421, 23.41295, 10.3632	6.14116, 10.84616, 6.92219	6.33915, 2	3	kirrel2	22598
GL173286.1	211254	211930	211676, 211297, 211322	7, 20, 9	7.15806, 18.59503, 8.34444	5.30717, 9.57724, 5.85853	4.61046, 1	3	kirrel2	3800
GL173286.1	239196	240180	239609, 239378, 239395	12, 15, 4	11.43506, 14.71623, 4.11547	7.25774, 8.60008, 3.5218	8.58315, 1	3	kirrel2	343
GL173286.1	247867	248735	248255, 248491, 248703	12, 20, 5	10.48835, 17.94395, 5.23257	6.6804, 9.1847, 4.20435	7.69782, 1	3	aplp1	0
GL173286.1	252820	253242	253140, 253208, 252903	8, 8, 5	5.95727, 5.53815, 5.19074	4.50543, 4.2362, 4.18277	3.52158, 3	3	aplp1	0
GL173286.1	258612	259493	258687, 258987, 258662	5, 8, 5	4.61714, 7.07159, 5.9506	3.86998, 5.20073, 4.54784	2.39372, 4	3	aplp1	0
GL173286.1	272811	273362	273339, 273326, 273347	8, 12, 9	7.88469, 10.08092, 11.12912	5.67897, 6.42828, 7.34851	5.27935, 7	3	aplp1	0
GL173286.1	286996	287670	287158, 287136, 287322	301, 240, 209	313.73151, 237.95767, 232.4272	25.91097, 22.64223, 28.63865	308.43341	3	aplp1	4138
GL173286.1	300178	300691	300313, 300406, 300382	12, 19, 9	12.74986, 19.18794, 11.39481	8.02869, 10.23458, 7.46977	9.82533, 1	3	aplp1	17320
GL173286.1	307087	307559	307198, 307197, 307472	5, 10, 5	4.82177, 9.39128, 5.54867	3.98509, 6.35644, 4.3619	2.51418, 6	3	gna13	18633
GL173286.1	316070	316458	316139, 316312, 316165	8, 15, 9	6.44853, 11.26316, 9.03892	4.81646, 6.49004, 6.26387	3.96884, 8	3	gna13	9734
GL173286.1	321804	322433	322274, 322282, 322261	35, 82, 37	35.39268, 84.28413, 42.51077	14.12881, 19.86743, 17.44571	31.77178,	3	gna13	3759
GL173286.1	403092	403441	403396, 403317, 403339	5, 5, 4	4.82177, 3.94561, 4.67325	3.98509, 3.46715, 3.78987	2.51418, 1	3	arhgap33	13629
GL173286.1	418799	419232	418967, 419056, 419016	24, 24, 7	24.44619, 22.72317, 6.59055	11.80365, 10.77359, 4.98809	21.08023,	3	arhgap33	0
GL173286.1	436529	437166	436735, 436996, 436738	9, 19, 10	8.77314, 16.05817, 11.75646	6.111, 8.3432, 7.66841	6.09355, 1	3	arhgap33	0
GL173286.1	448099	448899	448378, 448420, 448357	8, 12, 6	6.37855, 8.46754, 4.75057	4.77253, 5.42308, 3.93137	3.90357, 5	3	arhgap33	10434
GL173286.1	452871	453519	453479, 453473, 453459	55, 85, 49	65.14967, 97.78922, 59.52502	22.12752, 24.84061, 21.84703	61.10432,	3	arhgap33	15206
GL173286.1	465520	465931	465730, 465710, 465602	12, 15, 9	12.25023, 13.31811, 9.76105	7.74089, 7.74852, 6.66343	9.35789, 1	3	lin37	10394
GL173286.1	491298	491945	491650, 491621, 491672	22, 29, 9	26.43423, 31.95135, 11.64359	13.62565, 14.46878, 7.57973	23.01317,	3	hspb6	1707
GL173286.1	571638	572149	571847, 571996, 571842	7, 10, 7	5.42862, 7.61995, 7.90186	4.2813, 5.26198, 5.69394	3.06982, 5	3	rbm42	0
GL173286.1	613291	613912	613645, 613403, 613511	4, 9, 7	3.74195, 8.10603, 7.2501	3.32091, 5.71528, 5.35703	1.6222, 5.6	3	NA	23372
GL173287.1	424067	425006	424951, 424567, 424569	10, 16, 11	9.03421, 13.35594, 12.13051	6.14116, 7.51928, 7.80464	6.33915, 1	3	sgce	0
GL173287.1	577381	578160	578020, 577642, 577989	14, 20, 14	5.84903, 8.04612, 7.7011	3.57947, 3.90414, 4.58995	3.44895, 5	3	col1a2	64733
GL173289.1	191840	193578	193010, 192686, 193015	19, 23, 11	18.20214, 19.92085, 11.73651	9.6386, 9.44142, 7.58663	15.03745,	3	rbp3	50404
GL173289.1	213106	214268	213531, 213501, 213606	8, 18, 9	8.42257, 17.86551, 10.72164	5.97763, 9.72285, 7.15493	5.76336, 1	3	rbp3	29714
GL173289.1	213106	214268	214112, 213501, 213606	6, 18, 9	5.96715, 17.86551, 10.72164	4.64927, 9.72285, 7.15493	3.52158, 1	3	rbp3	29714
GL173289.1	405523	407288	406799, 407175, 405878	6, 14, 5	5.3939, 11.28333, 4.60894	4.32314, 6.73619, 3.8653	3.0374, 8.6	3	gdf10	30580
GL173289.1	499903	501047	500588, 500596, 500463	15, 20, 9	18.26603, 22.87459, 11.64359	10.62691, 12.13503, 7.57973	15.09729,	3	NA	23530
GL173290.1	40573	40812	40602, 40623, 40601	16, 28, 12	19.7934, 34.08019, 16.36823	11.29109, 16.10384, 9.85365	16.57067,	3	NA	32425
GL173290.1	63327	64229	64002, 63710, 63599	10, 27, 6	11.04401, 31.78991, 7.29403	7.29736, 15.11938, 5.30581	8.22236, 2	3	NA	9008
GL173290.1	72285	72813	72288, 72302, 72312	20, 42, 15	25.52229, 53.89993, 21.38372	13.62049, 21.90883, 12.12757	22.12641,	3	NA	424
GL173290.1	79996	80451	80050, 80013, 80009	25, 27, 18	33.23791, 33.60851, 26.51834	16.58592, 16.18004, 14.35044	29.65938,	3	NA	0
GL173290.1	144580	145776	144846, 144861, 144881	13, 27, 12	13.71465, 29.25974, 15.80834	8.42261, 13.61609, 9.60504	10.74495,	3	NA	16766
GL173290.1	163944	164522	164244, 164204, 164357	11, 13, 5	11.15818, 11.61168, 5.14967	7.25692, 7.16421, 4.16141	8.32008, 8	3	NA	0
GL173290.1	179285	179947	179748, 179544, 179544	9, 12, 10	9.72082, 11.86108, 12.71002	6.64182, 7.51216, 8.12734	6.97155, 9	3	xk81a1	931
GL173290.1	297248	298062	297804, 297534, 297784	7, 11, 8	7.1696, 9.66539, 8.80246	5.31345, 6.36265, 6.179	4.61046, 7	3	NA	3822
GL173290.1	380860	382081	381400, 381648, 381428	7, 9, 8	7.1696, 8.21099, 8.98772	5.31345, 5.77858, 6.27416	4.61046, 5	3	NA	12342
GL173290.1	466528	466901	466652, 466792, 466737	16, 15, 6	14.29285, 11.22634, 5.29847	8.0922, 6.46769, 4.26663	11.29159,	3	smarce1	0
GL173290.1	467915	468346	468055, 468230, 468099	7, 19, 7	4.6941, 15.48693, 6.47418	3.80809, 8.00194, 4.92032	2.45935, 1	3	smarce1	0
GL173290.1	520171	520645	520610, 520611, 520603	19, 28, 9	22.68927, 29.22753, 11.21577	12.28657, 13.22571, 7.38849	19.37453,	3	smarce1	35237
GL173290.1	562949	563242	563015, 563006, 563001	14, 16, 12	16.43009, 15.47832, 12.50469	9.78349, 8.81468, 7.88834	13.33671,	3	smarce1	78015
GL173291.1	698	1150	940, 866, 1044	11, 16, 7	8.65724, 11.65768, 5.92008	5.73562, 6.49186, 4.58787	5.98642, 8	3	NA	7038
GL173291.1	2374	3123	2809, 2686, 2586	18, 24, 16	15.99408, 19.8158, 16.83466	8.58723, 9.0745, 9.62725	12.91885,	3	NA	5065
GL173291.1	223619	224030	223846, 223769, 223781	7, 10, 4	7.1696, 9.39128, 4.67325	5.31345, 6.35644, 3.78987	4.61046, 6	3	NA	212499
GL173291.1	234384	234933	234887, 234792, 234895	12, 20, 19	12.11599, 18.2926, 22.23331	7.66241, 9.39473, 12.02841	9.23027, 1	3	NA	223264
GL173291.1	253032	253392	253290, 253257, 253243	6, 5, 5	5.90927, 3.94561, 5.9506	4.61743, 3.46715, 4.54784	3.50164, 1	3	NA	241912
GL173291.1	361735	362378	362264, 362268, 362266	18, 24, 16	17.38211, 23.92411, 18.78921	9.43026, 11.49076, 10.74773	14.24778,	3	NA	350615
GL173291.1	394734	395321	394905, 395166, 395183	8, 13, 5	7.67464, 12.31712, 5.9506	5.55832, 7.59462, 4.54784	5.08299, 9	3	NA	383614
GL173291.1	505226	505684	505394, 505453, 505386	13, 25, 19	13.86771, 28.03215, 25.11547	8.51067, 13.60143, 13.59366	10.88502,	3	NA	494106
GL173291.1	531349	531650	531606, 531577, 531590	8, 11, 5	8.42257, 10.10652, 5.50079	5.97763, 6.63228, 4.33868	5.76336, 7	3	NA	520229
GL173293.1	45669	46188	46030, 46076, 45970	7, 6, 4	7.1696, 4.53871, 4.67325	5.31345, 3.79731, 3.78987	4.61046, 2	3	NA	7738
GL173293.1	143318	143746	143492, 143638, 143725	7, 9, 6	5.93788, 4.71739, 4.30184	4.59878, 3.57453, 3.64496	3.52158, 2	3	NA	3531
GL173293.1	148624	149279	148644, 148664, 148725	61, 93, 69	69.61686, 101.60376, 86.10577	21.69666, 23.11319, 26.7175	65.52451,	3	NA	434
GL173293.1	283679	284141	283847, 283858, 283865	7, 14, 8	5.65516, 11.45303, 8.62637	4.42382, 6.84088, 6.08668	3.27228, 8	3	cldn15.1	2479
GL173293.1	350859	351503	351051, 351080, 351026	15, 25, 7	16.90423, 30.17196, 8.08824	9.88146, 14.85972, 5.7849	13.78827,	3	vmo1	16766
GL173293.1	362968	363855	363524, 363099, 363220	10, 12, 5	8.79841, 10.39632, 5.06966	5.99719, 6.6236, 4.11933	6.11782, 7	3	vmo1	4414
GL173293.1	388600	389348	389107, 388722, 388734	8, 6, 5	6.59314, 4.11123, 5.14967	4.90679, 3.52071, 4.16141	4.10249, 2	3	dnajc18	1206
GL173293.1	489153	489822	489180, 489183, 489181	140, 191, 125	186.35298, 250.25078, 175.83678	39.38575, 41.32362, 42.79882	181.43797	3	NA	985
GL173294.1	30826	31141	30968, 30909, 31021	6, 8, 6	5.96715, 6.21066, 7.29403	4.64927, 4.66659, 5.30581	3.52158, 3	3	NA	52
GL173294.1	208270	209856	208660, 208666, 209433	15, 25, 10	13.97429, 23.34787, 10.3632	8.15, 10.80775, 6.92219	10.98644,	3	NA	17940
GL173294.1	259341	259909	259690, 259756, 259680	28, 36, 20	23.60262, 27.14232, 16.95943	9.96844, 9.43369, 8.59501	20.2611, 2	3	NA	4609
GL173294.1	271724	272055	271960, 272011, 272000	14, 13, 12	15.49091, 13.14514, 13.82167	9.26387, 8.09002, 8.62016	12.43497,	3	NA	16992
GL173294.1	299171	300478	299750, 299750, 299745	18, 15, 12	18.84377, 14.46023, 14.76955	10.31044, 8.44539, 9.10943	15.65357,	3	fcrl3	5419
GL173294.1	328281	329251	328468, 328606, 329012	14, 19, 7	14.16942, 18.34606, 7.2501	8.49669, 9.72578, 5.35703	11.17387,	3	fcrl3	0
GL173294.1	527667	528928	528617, 528427, 528012	8, 8, 6	6.48409, 7.07159, 5.46454	4.83873, 5.20073, 4.36458	4.00135, 4	3	NA	2298
GL173294.1	552038	553206	552488, 552849, 552959	6, 13, 7	4.80449, 11.41208, 6.55126	3.96512, 7.04141, 4.96529	2.51418, 8	3	NA	14562
GL173294.1	557034	557954	557488, 557480, 557625	13, 7, 8	12.56081, 4.38653, 7.22894	7.74168, 3.60643, 5.29553	9.64511, 2	3	NA	19558
GL173294.1	577450	578059	577748, 577610, 577677	9, 7, 5	9.72082, 5.48955, 5.9506	6.64182, 4.31982, 4.54784	6.97155, 3	3	NA	39974
GL173295.1	19359	19834	19765, 19637, 19686	13, 15, 5	12.49994, 15.33351, 5.50079	7.70504, 8.96975, 4.33868	9.58678, 1	3	susd4	0
GL173295.1	108964	109953	109678, 109805, 109803	15, 14, 13	14.0331, 11.80872, 14.69628	8.18586, 7.06033, 8.97644	11.04322,	3	disp1	0
GL173295.1	283869	284760	284406, 284095, 284308	11, 12, 11	10.3251, 11.04188, 12.34038	6.7644, 7.01971, 7.91841	7.54325, 8	3	taf1a	16904
GL173295.1	300080	300647	300438, 300440, 300272	29, 23, 9	32.76592, 24.95167, 10.87975	14.95372, 12.44425, 7.23113	29.1976, 2	3	taf1a	1017
GL173295.1	325204	325655	325561, 325561, 325372	7, 6, 4	6.42049, 4.51135, 3.76128	4.8888, 3.77984, 3.33171	3.94161, 2	3	hhipl2	0
GL173295.1	328453	329342	328852, 328878, 328849	6, 11, 9	5.3552, 10.00475, 11.04428	4.3003, 6.57041, 7.30896	3.00253, 7	3	hhipl2	0
GL173295.1	403296	403859	403658, 403491, 403643	11, 18, 9	11.29029, 19.97713, 10.28145	7.3332, 10.97931, 6.93568	8.44541, 1	3	hhipl2	57580
GL173295.1	462987	463383	463127, 463046, 463170	23, 24, 16	24.85714, 24.30704, 21.00727	12.38779, 11.72023, 11.91233	21.47972,	3	DUSP10	53227
GL173295.1	483435	483776	483696, 483675, 483682	14, 15, 8	11.43945, 12.7702, 8.74279	6.8325, 7.41214, 6.14792	8.5874, 10	3	DUSP10	32834
GL173295.1	507164	507360	507327, 507320, 507247	23, 40, 10	25.13729, 41.27839, 10.47831	12.55499, 15.45571, 6.98694	21.75229,	3	DUSP10	9250
GL173296.1	10232	11264	10969, 10942, 10975	18, 14, 8	19.69649, 13.03306, 10.1465	10.81516, 7.81216, 6.82176	16.47841,	3	NA	496727
GL173296.1	66748	68065	67246, 67650, 67384	7, 13, 6	5.4599, 10.35117, 6.08001	4.30109, 6.38482, 4.71063	3.09813, 7	3	NA	439926
GL173297.1	15891	16545	15972, 16380, 16372	9, 14, 6	9.72082, 14.45897, 7.29403	6.64182, 8.66788, 5.30581	6.97155, 1	3	NA	1879
GL173297.1	34112	34652	34130, 34200, 34289	12, 8, 4	13.8483, 7.07159, 4.15126	8.63436, 5.20073, 3.54016	10.86694,	3	bcl7c	3690
GL173297.1	53794	53987	53896, 53917, 53899	47, 30, 38	54.15861, 31.15101, 44.55311	19.70293, 13.60907, 18.21266	50.24762,	3	NA	0
GL173297.1	78317	78951	78900, 78436, 78615	8, 7, 7	7.47668, 5.42726, 7.50542	5.44269, 4.28044, 5.49244	4.89713, 3	3	znf668	154
GL173297.1	157325	157643	157368, 157330, 157400	12, 17, 11	11.15354, 15.40668, 11.73651	7.08759, 8.50651, 7.58663	8.31649, 1	3	hsd3b7	0
GL173297.1	162085	162683	162430, 162572, 162182	6, 10, 6	5.81608, 9.12511, 6.5517	4.56563, 6.19629, 4.95623	3.4176, 6.5	3	hsd3b7	374
GL173297.1	175304	175813	175769, 175628, 175756	6, 11, 6	4.99728, 8.37597, 6.168	4.08451, 5.5591, 4.75778	2.67584, 5	3	setd1a	7428
GL173297.1	184490	185067	184917, 185015, 185040	6, 12, 10	5.72604, 9.86753, 11.83521	4.51498, 6.29567, 7.70798	3.33547, 7	3	setd1a	0
GL173297.1	303248	303792	303368, 303368, 303368	24, 29, 16	25.63312, 32.77739, 19.75829	12.51508, 14.96054, 11.27252	22.23394,	3	prss8	0
GL173297.1	407284	407931	407813, 407479, 407478	6, 12, 11	5.96715, 11.86108, 13.1143	4.64927, 7.51216, 8.32282	3.52158, 9	3	NA	5645
GL173299.1	537912	538327	538164, 538039, 538144	12, 6, 8	13.64785, 4.56637, 10.1465	8.52686, 3.81494, 6.82176	10.68062,	3	shcbp1	494528
GL173300.1	548190	548843	548583, 548460, 548527	8, 10, 9	7.67464, 8.32357, 10.87975	5.55832, 5.70361, 7.23113	5.08299, 5	3	kansl1l	0
GL173300.1	561809	562849	561940, 562481, 562298	16, 26, 14	10.155, 18.87865, 9.22157	5.61134, 7.99555, 5.4788	7.38249, 1	3	kansl1l	4937
GL173302.1	61048	61396	61284, 61284, 61284	46, 62, 28	54.24099, 71.15286, 34.9876	20.22038, 22.04953, 16.63102	50.32833,	3	ncam2	258357
GL173302.1	182215	182704	182302, 182302, 182302	18, 26, 8	19.00683, 25.62201, 9.1831	10.40758, 11.8089, 6.37229	15.81175,	3	ncam2	137049
GL173302.1	193925	194867	194427, 194268, 194269	13, 10, 11	11.16633, 6.50583, 11.49087	6.8898, 4.55637, 7.44794	8.32764, 4	3	ncam2	124886
GL173302.1	221634	222061	221660, 221646, 221666	24, 25, 22	25.63312, 24.88547, 25.67929	12.51508, 11.72091, 13.19012	22.23394,	3	ncam2	97692
GL173303.1	57558	57883	57856, 57857, 57732	9, 18, 8	9.72082, 16.94269, 8.35103	6.64182, 9.16356, 5.93881	6.97155, 1	3	lrsam1	0
GL173303.1	99387	99871	99682, 99554, 99588	5, 8, 4	4.33555, 5.46019, 4.15126	3.70555, 4.18585, 3.54016	2.15195, 3	3	fkbp15	0
GL173303.1	118759	119189	118857, 118944, 118941	14, 7, 5	12.69785, 4.01459, 4.70718	7.6073, 3.36117, 3.9211	9.77574, 1	3	slc31a1	9926
GL173303.1	127080	127295	127135, 127229, 127243	11, 18, 12	7.80634, 12.63894, 11.30996	5.20114, 6.5909, 7.18231	5.20581, 9	3	slc31a1	1820
GL173303.1	127961	128691	128454, 128367, 128217	20, 27, 8	18.39153, 23.71775, 6.93578	9.4544, 10.35558, 5.11812	15.21806,	3	slc31a1	424
GL173303.1	136804	138849	137191, 138489, 138313	19, 29, 16	18.67786, 24.37675, 16.0646	9.92659, 10.07904, 9.16845	15.49416,	3	slc31a1	5537
GL173303.1	148442	149364	149040, 149087, 148704	23, 28, 13	24.06243, 27.32048, 14.11158	11.91191, 12.09764, 8.64973	20.70741,	3	prpf4	543
GL173303.1	196890	198125	197462, 197325, 197257	6, 19, 10	5.96715, 18.34606, 10.65691	4.64927, 9.72578, 7.08637	3.52158, 1	3	WDR31	4586
GL173303.1	237613	238595	238418, 238409, 237848	15, 30, 13	16.20017, 33.20756, 15.17843	9.47871, 14.82921, 9.23814	13.11636,	3	BSPRY	3749
GL173303.1	296425	296982	296718, 296652, 296648	7, 12, 4	6.16939, 8.99251, 4.18775	4.73936, 5.75004, 3.55872	3.70916, 6	3	tubb4b	18154
GL173303.1	298414	299071	298451, 298434, 298491	14, 24, 8	13.8308, 21.95615, 8.56955	8.29466, 10.31898, 6.05652	10.85567,	3	tubb4b	16065
GL173303.1	306411	306944	306509, 306603, 306531	10, 24, 9	9.23294, 24.46445, 9.19414	6.26141, 11.8146, 6.35176	6.52626, 2	3	tubb4b	8192
GL173303.1	340560	340896	340724, 340742, 340817	12, 15, 9	12.52976, 14.7819, 10.42289	7.90277, 8.63964, 7.00726	9.6155, 11	3	slc34a3	7005
GL173303.1	405531	406594	405830, 406064, 406221	8, 16, 8	8.42257, 17.16856, 10.1465	5.97763, 9.82359, 6.82176	5.76336, 1	3	slc34a3	51174
GL173303.1	422824	423024	422852, 422872, 422871	34, 56, 37	36.11374, 63.55, 44.88342	14.94713, 20.75404, 18.84748	32.47832,	3	lmo4.2	41269
GL173303.1	550919	551679	550947, 550966, 550953	31, 45, 32	30.14713, 43.8494, 35.97895	12.6443, 14.85911, 15.68032	26.6365, 4	3	glipr2	14444
GL173303.1	571653	572189	571915, 571981, 571940	7, 21, 9	6.09019, 20.32736, 10.28145	4.69155, 10.3138, 6.93568	3.63504, 1	3	glipr2	0
GL173303.1	584229	585037	584382, 584378, 584935	9, 12, 5	9.72082, 10.12474, 4.91762	6.64182, 6.45547, 4.03768	6.97155, 7	3	glipr2	5234
GL173303.1	592314	592710	592357, 592410, 592374	7, 11, 11	5.21879, 7.16781, 10.77078	4.14768, 4.80116, 7.03024	2.87895, 4	3	ppara	12598
GL173303.1	608049	609191	608528, 608226, 609109	7, 19, 7	6.74267, 17.06093, 6.43637	5.07552, 8.94736, 4.89813	4.23998, 1	3	ppara	594
GL173304.1	140562	141310	140948, 140953, 140993	15, 12, 11	14.39822, 8.6575, 11.92945	8.40782, 5.54124, 7.69409	11.39247,	3	rab28	150495
GL173304.1	141778	142241	141954, 141954, 141941	10, 14, 11	8.44704, 10.76513, 11.92945	5.78037, 6.41704, 7.69409	5.7869, 8.1	3	rab28	149564
GL173304.1	319892	320150	319897, 319921, 319928	9, 7, 6	9.00096, 5.97725, 6.99807	6.24222, 4.62287, 5.17195	6.30808, 3	3	rab28	0
GL173304.1	436521	436960	436893, 436906, 436868	29, 48, 27	32.76592, 50.9723, 36.3158	14.95372, 17.46203, 17.70214	29.1976, 4	3	nkx3-2	51176
GL173305.1	142	590	546, 589, 554	24, 29, 20	27.72617, 33.2124, 21.72938	13.759, 15.21917, 11.4623	24.2736, 2	3	phox2a	38143
GL173305.1	53093	53408	53299, 53275, 53228	14, 18, 13	15.31941, 16.06714, 16.18511	9.1665, 8.63158, 9.7598	12.27033,	3	clpb	8498
GL173305.1	70306	70936	70598, 70530, 70457	8, 7, 4	8.42257, 5.97725, 4.67325	5.97763, 4.62287, 3.78987	5.76336, 3	3	clpb	0
GL173305.1	158294	158786	158544, 158680, 158638	6, 14, 3	5.96715, 14.45897, 3.47371	4.64927, 8.66788, 3.03189	3.52158, 1	3	NA	7434
GL173305.1	178151	178697	178578, 178214, 178328	5, 11, 5	3.86321, 9.13706, 5.9506	3.41531, 6.0355, 4.54784	1.73293, 6	3	arrb1	0
GL173305.1	196717	197072	196935, 197013, 196962	5, 10, 6	4.82177, 9.39128, 7.29403	3.98509, 6.35644, 5.30581	2.51418, 6	3	arrb1	0
GL173305.1	207500	207966	207815, 207817, 207808	15, 33, 9	16.20017, 35.57923, 9.76105	9.47871, 15.03857, 6.66343	13.11636,	3	arrb1	0
GL173305.1	233706	234821	234183, 234208, 234015	21, 43, 16	21.25459, 45.71592, 18.78921	10.87284, 16.69384, 10.74773	17.98535,	3	arrb1	12948
GL173305.1	239057	239603	239171, 239175, 239174	23, 26, 13	24.76553, 24.23472, 14.5443	12.33305, 10.98992, 8.89247	21.39099,	3	arrb1	18299
GL173305.1	254767	255369	255350, 255317, 255214	21, 17, 9	22.31353, 12.85839, 9.70054	11.50874, 6.96436, 6.63089	19.0106, 1	3	NA	19327
GL173305.1	265030	266203	265297, 265729, 265579	7, 26, 4	7.1696, 27.13121, 4.67325	5.31345, 12.7086, 3.78987	4.61046, 2	3	NA	8493
GL173305.1	272490	273017	272813, 272707, 272823	11, 24, 14	11.02968, 23.41175, 14.53512	7.18222, 11.1842, 8.71263	8.20839, 2	3	NA	1679
GL173305.1	313123	314723	314026, 314041, 314499	8, 13, 7	8.42257, 13.14514, 8.69489	5.97763, 8.09002, 6.06379	5.76336, 1	3	NA	33733
GL173305.1	320858	321870	320910, 321462, 321200	6, 10, 5	5.96715, 8.16488, 5.64747	4.64927, 5.60458, 4.40911	3.52158, 5	3	NA	41468
GL173305.1	325703	327596	327100, 326169, 326798	11, 17, 12	10.9046, 13.99022, 13.08717	7.10903, 7.64472, 8.21885	8.08987, 1	3	NA	46313
GL173305.1	356798	358108	356926, 357178, 357897	8, 10, 8	6.17724, 6.21268, 7.50809	4.64542, 4.37127, 5.46116	3.71619, 3	3	lats2	20499
GL173305.1	357316	359469	359267, 358680, 357897	7, 13, 8	5.02357, 9.02151, 7.50809	4.02214, 5.56343, 5.46116	2.70132, 6	3	lats2	19138
GL173306.1	1208	1889	1243, 1212, 1247	13, 12, 13	14.97989, 10.63474, 15.34872	9.13126, 6.77054, 9.3288	11.94968,	3	NA	106349
GL173306.1	280770	281247	281035, 281142, 281047	7, 14, 5	7.1696, 11.90127, 4.84526	5.31345, 7.11741, 3.99806	4.61046, 9	3	reep3	88814
GL173306.1	475881	476862	476148, 476537, 476370	13, 19, 14	12.68462, 16.30954, 17.40994	7.81602, 8.49413, 10.29833	9.76298, 1	3	nrbf2	1453
GL173306.1	483303	484199	483951, 483484, 483404	13, 9, 6	11.76528, 6.7053, 6.99807	7.25843, 4.84282, 5.17195	8.8968, 4.3	3	nrbf2	8875
GL173306.1	485707	485949	485746, 485914, 485802	7, 8, 4	5.30682, 5.56458, 4.22496	4.20391, 4.25326, 3.57747	2.95991, 3	3	nrbf2	11279
GL173306.1	495409	496217	495769, 495568, 495487	8, 8, 7	6.37855, 5.48596, 7.84208	4.77253, 4.2025, 5.66426	3.90357, 3	3	nrbf2	20981
GL173308.1	86528	87101	86837, 86851, 86851	21, 22, 17	25.14429, 22.94165, 22.63257	13.1674, 11.56804, 12.61306	21.75899,	3	NA	21250
GL173308.1	308326	309276	308919, 309104, 308771	15, 21, 9	16.63109, 21.41771, 10.14484	9.72648, 10.97106, 6.86555	13.52931,	3	t2r20	2736
GL173308.1	443881	444282	443944, 443943, 443898	15, 19, 10	16.72085, 19.96513, 13.18184	9.7776, 10.7011, 8.33771	13.61562,	3	phgdh	3014
GL173309.1	2348	2850	2602, 2764, 2788	13, 14, 11	9.3861, 9.2785, 10.98082	5.78755, 5.51289, 7.15369	6.66988, 6	3	DOC2A	5837
GL173309.1	6081	6549	6525, 6505, 6505	48, 54, 24	53.91633, 61.33422, 27.81654	19.10194, 20.47031, 13.81213	50.00864,	3	DOC2A	2138
GL173309.1	36198	36761	36588, 36472, 36650	19, 11, 11	20.05161, 10.00475, 11.49087	10.75274, 6.57041, 7.44794	16.81949,	3	NA	0
GL173309.1	74960	75421	75372, 75343, 75337	7, 6, 7	6.50909, 4.56637, 7.45266	4.94073, 3.81494, 5.46481	4.02371, 2	3	nme2	2219
GL173309.1	92297	92665	92615, 92615, 92600	8, 18, 13	6.78332, 16.81975, 14.39632	5.02459, 9.08887, 8.81006	4.27811, 1	3	XB-GENE-5854345	2339
GL173309.1	173441	173786	173480, 173490, 173496	21, 33, 11	22.75736, 35.48338, 13.1143	11.77357, 14.98211, 8.32282	19.44135,	3	NA	0
GL173309.1	224965	225833	225080, 225101, 225689	6, 9, 4	5.96715, 8.21099, 4.67325	4.64927, 5.77858, 3.78987	3.52158, 5	3	nlk.2	13000
GL173309.1	260960	261395	261108, 261126, 261131	16, 7, 8	18.34375, 5.97725, 10.1465	10.49905, 4.62287, 6.82176	15.17198,	3	B2M	25513
GL173309.1	266131	267052	266556, 266832, 266824	5, 7, 6	4.82177, 5.97725, 6.99807	3.98509, 4.62287, 5.17195	2.51418, 3	3	B2M	19856
GL173309.1	283777	284385	283801, 283783, 283780	37, 37, 26	43.22798, 37.53865, 32.16442	17.86899, 14.55858, 15.68273	39.47506,	3	B2M	2523
GL173309.1	385764	386294	386164, 386117, 386116	7, 16, 4	7.1696, 17.16856, 4.67325	5.31345, 9.82359, 3.78987	4.61046, 1	3	hist2h2ab	30982
GL173309.1	434638	435166	434845, 434854, 434789	17, 20, 20	13.31149, 16.49115, 19.5528	7.23535, 8.31699, 10.15606	10.36022,	3	hist1h4c	4230
GL173309.1	443881	444494	444396, 444401, 443956	7, 6, 5	5.27717, 3.52517, 4.22765	4.185, 3.13158, 3.64077	2.93268, 1	3	NA	345
GL173309.1	513194	513865	513752, 513725, 513726	17, 22, 12	18.04435, 21.35393, 14.40488	10.09765, 10.61306, 8.92522	14.88575,	3	hist1h4c	1573
GL173310.1	37877	38150	37967, 38005, 37960	9, 7, 8	9.56822, 5.96795, 10.1465	6.55912, 4.61718, 6.82176	6.84121, 3	3	erp44	10100
GL173310.1	495023	495376	495198, 495309, 495316	9, 11, 5	9.36986, 9.66539, 5.54867	6.44997, 6.36265, 4.3619	6.65425, 7	3	tbx20	135585
GL173311.1	325	723	452, 567, 486	8, 15, 8	4.07435, 8.55865, 7.50809	3.28805, 4.89877, 5.46116	1.92544, 6	3	xtr-mir-427-3	5471
GL173311.1	37363	37804	37528, 37494, 37495	14, 26, 8	16.7649, 31.99133, 10.0913	9.96273, 15.58299, 6.79758	13.6543, 2	3	xtr-mir-427-3	0
GL173311.1	193917	194978	194794, 194396, 194347	9, 17, 9	7.47395, 14.86656, 9.14171	5.32711, 8.17729, 6.32219	4.89626, 1	3	NA	248
GL173311.1	311062	311511	311485, 311503, 311492	15, 11, 5	18.26603, 10.60895, 5.9506	10.62691, 6.9343, 4.54784	15.09729,	3	NA	2176
GL173311.1	487827	488115	487857, 487958, 487951	14, 9, 5	16.03678, 8.21099, 4.88115	9.5688, 5.77858, 4.01777	12.95916,	3	chd3	0
GL173311.1	500685	501094	500918, 500856, 500973	6, 10, 8	5.90927, 9.39128, 9.1831	4.61743, 6.35644, 6.37229	3.50164, 6	3	chd3	0
GL173311.1	521032	521494	521259, 521336, 521411	13, 32, 7	11.55805, 32.9028, 7.10639	7.13125, 13.86483, 5.27894	8.70034, 2	3	chd3	0
GL173312.1	165679	165964	165719, 165690, 165705	18, 37, 26	20.35565, 39.03289, 33.79316	11.19891, 15.41384, 16.60607	17.11452,	3	znf568	8280
GL173312.1	250317	250806	250350, 250379, 250367	8, 7, 7	7.42892, 5.42726, 8.08824	5.41453, 4.28044, 5.7849	4.85345, 3	3	MGC145518	896
GL173312.1	346741	347280	346838, 346773, 347126	5, 7, 3	4.54329, 5.33631, 3.34298	3.82752, 4.22269, 2.97156	2.33, 3.091	3	XB-GENE-5822524	0
GL173312.1	516200	516937	516384, 516727, 516458	19, 25, 20	20.74745, 25.42729, 24.77855	11.16575, 12.04472, 13.21509	17.49384,	3	NA	4123
GL173313.1	219390	219907	219829, 219723, 219533	27, 29, 12	30.77168, 31.46221, 14.40488	14.51687, 14.17752, 8.92522	27.24691,	3	znf208	0
GL173313.1	413638	414079	413931, 413931, 413910	22, 28, 11	26.19473, 33.32234, 11.79996	13.48821, 15.65935, 7.62212	22.77902,	3	NA	4419
GL173313.1	454291	454775	454399, 454328, 454311	17, 16, 10	17.26461, 16.22539, 11.60305	9.63292, 9.26486, 7.59048	14.13485,	3	NA	0
GL173313.1	486194	486644	486241, 486443, 486561	8, 13, 7	8.42257, 12.55412, 8.69489	5.97763, 7.73766, 6.06379	5.76336, 9	3	NA	6683
GL173314.1	187475	187885	187671, 187623, 187688	24, 16, 15	25.45469, 15.80758, 18.63599	12.40824, 9.01376, 10.82077	22.06058,	3	fat1	16130
GL173315.1	136637	137370	137050, 137050, 136992	22, 21, 11	23.89932, 21.03886, 12.9493	12.1411, 10.74286, 8.23866	20.54867,	3	tbca	10654
GL173315.1	330301	330612	330451, 330442, 330382	7, 7, 4	6.42049, 5.75319, 4.67325	4.8888, 4.48487, 3.78987	3.94161, 3	3	pde8b	18765

GL173316.1	104189	104650	104442, 104457, 104448	7, 9, 7	7.1696, 8.21099, 8.69489	5.31345, 5.77858, 6.06379	4.61046, 5	3	NA	9392
GL173316.1	148666	149682	149093, 149044, 149318	16, 23, 14	12.18769, 18.56726, 12.98163	6.80985, 8.65798, 7.7808	9.2978, 15	3	NA	2354
GL173316.1	152514	153397	152942, 152551, 152777	24, 27, 13	22.75993, 23.77514, 11.86258	10.79546, 10.38831, 7.31799	19.44364,	3	NA	6202
GL173316.1	227021	227645	227614, 227485, 227624	10, 11, 9	11.06011, 9.61892, 10.35155	7.306, 6.33404, 6.97128	8.22712, 7	3	NA	6970
GL173316.1	263306	263915	263824, 263825, 263825	32, 43, 21	38.13301, 48.85407, 24.6582	16.95794, 18.48643, 12.88878	34.46145,	3	NA	254
GL173316.1	315375	315921	315617, 315537, 315632	7, 10, 6	7.1696, 9.39128, 7.29403	5.31345, 6.35644, 5.30581	4.61046, 6	3	NA	4215
GL173316.1	442431	443332	442564, 442461, 442542	11, 37, 11	11.71035, 41.53486, 13.64572	7.57196, 16.87138, 8.58592	8.8442, 37	3	NA	169
GL173316.1	535925	536771	536053, 536419, 536037	12, 20, 6	7.62604, 12.04745, 4.69928	4.90347, 5.82217, 3.89912	5.03809, 9	3	xtr-mir-427-2	0
GL173316.1	537051	537830	537293, 537119, 537562, 537111	12, 30, 12, 10	6.97595, 20.63489, 4.74337, 8.15593	4.50859, 7.86971, 3.22352, 5.59899	4.45626, 1	3	xtr-mir-427-3	0
GL173316.1	542609	542993	542699, 542780, 542791	5, 15, 7	4.27048, 12.35615, 7.55909	3.6666, 7.15789, 5.52035	2.09564, 9	3	xtr-mir-427-2	472
GL173316.1	547799	548400	547981, 548204, 548208	13, 19, 21	9.62543, 12.42239, 21.93599	5.93525, 6.23734, 11.28256	6.8956, 9.6	3	xtr-mir-427-3	0
GL173316.1	558134	558967	558841, 558343, 558346	9, 17, 11	5.46202, 11.50636, 8.89958	4.04762, 6.17106, 5.88733	3.10014, 8	3	xtr-mir-427-3	0
GL173318.1	94362	95125	94835, 94624, 94676	11, 22, 10	11.02968, 20.68559, 11.5283	7.18222, 10.21163, 7.55211	8.20839, 1	3	nmur2	194995
GL173318.1	185980	186567	186005, 186037, 186283	26, 27, 19	25.97051, 26.43443, 19.82575	12.01609, 11.93497, 10.61774	22.56197,	3	nmur2	103553
GL173318.1	188390	188955	188429, 188421, 188422	16, 27, 19	12.3854, 25.45196, 20.10806	6.9292, 11.35757, 10.78641	9.48521, 2	3	nmur2	101165
GL173318.1	203491	204101	203861, 203644, 203898	7, 10, 7	6.33443, 9.39128, 8.69489	4.83795, 6.35644, 6.06379	3.86259, 6	3	nmur2	86019
GL173318.1	299100	299719	299112, 299122, 299131	36, 59, 37	43.7347, 71.71843, 50.24323	18.6024, 23.84328, 21.97799	39.97363,	3	nmur2	0
GL173318.1	416190	416863	416680, 416693, 416646	5, 13, 11	4.54329, 12.2024, 13.28509	3.82752, 7.52507, 8.40871	2.33, 9.476	3	glra1	95881
GL173318.1	422625	423096	423047, 423069, 423070	27, 32, 8	31.33009, 31.45735, 7.81204	14.84788, 13.02561, 5.63749	27.79259,	3	glra1	89648
GL173318.1	427524	428070	428019, 428031, 428036	23, 36, 17	25.32867, 37.17613, 19.55066	12.66898, 14.7533, 10.97134	21.93845,	3	glra1	84674
GL173318.1	452192	452768	452224, 452648, 452367	13, 9, 6	12.26307, 7.17446, 6.25896	7.56188, 5.1397, 4.80589	9.36972, 4	3	glra1	59976
GL173319.1	115115	115574	115285, 115284, 115288	8, 7, 3	8.42257, 5.52121, 3.44998	5.97763, 4.33979, 3.02115	5.76336, 3	3	NA	3288
GL173319.1	150138	150630	150297, 150405, 150333	8, 9, 6	6.34412, 6.12736, 5.91225	4.75087, 4.47368, 4.61907	3.871, 3.79	3	cd200r1	89
GL173319.1	226541	226828	226812, 226789, 226697	11, 19, 8	10.9046, 17.46611, 8.9249	7.10903, 9.19261, 6.24211	8.08987, 1	3	NA	0
GL173319.1	255507	256419	255574, 255525, 255573	25, 45, 14	22.49739, 45.47107, 14.34766	10.30852, 15.72457, 8.60225	19.18888,	3	NA	15634
GL173319.1	258885	259368	259312, 259329, 259317	28, 26, 16	26.02644, 21.06429, 16.18697	11.34326, 9.17619, 9.24186	22.61629,	3	NA	19012
GL173319.1	307565	308637	308126, 308118, 308194	16, 22, 16	18.05293, 25.38455, 20.07743	10.33439, 13.01836, 11.44009	14.8942, 2	3	NA	11665
GL173319.1	419874	420735	420538, 420586, 420463	8, 15, 15	5.58533, 9.97812, 16.99143	4.26664, 5.71925, 9.93059	3.2111, 7.3	3	ildr1	0
GL173319.1	463190	463484	463313, 463282, 463193	11, 12, 9	10.00935, 10.68384, 10.56949	6.57321, 6.80072, 7.08032	7.24529, 8	3	ILDR2	0
GL173319.1	478443	478640	478587, 478456, 478470	6, 17, 10	5.24248, 17.79269, 12.25126	4.2332, 9.94842, 7.91211	2.89962, 1	3	ILDR2	0
GL173319.1	515386	515807	515619, 515629, 515623	8, 21, 17	7.72597, 20.81922, 19.29472	5.588, 10.61044, 10.82557	5.13061, 1	3	ILDR2	27700
GL173319.1	540297	541270	540530, 540721, 540740	6, 10, 3	5.96715, 9.39128, 3.47371	4.64927, 6.35644, 3.03189	3.52158, 6	3	ILDR2	52611
GL173320.1	404289	404743	404701, 404577, 404593	6, 10, 10	5.31708, 9.39128, 12.71002	4.2777, 6.35644, 8.12734	2.96727, 6	3	AQP4	130521
GL173320.1	501000	502651	501641, 501661, 502203	13, 16, 14	12.68462, 14.74902, 14.16595	7.81602, 8.37095, 8.49463	9.76298, 1	3	AQP4	227232
GL173320.1	535096	536634	535573, 535838, 535710	11, 14, 15	8.8889, 10.99827, 15.17593	5.88065, 6.5605, 8.87588	6.20288, 8	3	AQP4	261328
GL173320.1	539857	540860	540309, 540331, 540086	17, 14, 7	17.48974, 12.18837, 6.59055	9.76779, 7.29433, 4.98809	14.35103,	3	AQP4	266089
GL173320.1	545211	547442	546552, 546228, 546239	22, 34, 12	22.68783, 36.25163, 14.23156	11.41558, 15.02786, 8.83588	19.37324,	3	AQP4	271443
GL173321.1	149651	150338	149931, 149931, 149931	9, 15, 5	8.0679, 12.35615, 5.23257	5.69219, 7.15789, 4.20435	5.45, 9.624	3	ncapg	198406
GL173321.1	201759	203649	201875, 203478, 202257, 203424	8, 17, 6, 6	6.94372, 11.56918, 5.91225, 5.91225	5.12297, 6.20732, 4.61907, 4.61907	4.42528, 8	3	ncapg	145095
GL173321.1	257540	258161	257671, 257747, 257701	13, 15, 8	12.32133, 11.18975, 7.95096	7.59717, 6.44549, 5.71659	9.42482, 8	3	ncapg	90583
GL173321.1	260873	262141	262048, 261578, 261581	10, 17, 13	8.79841, 13.33786, 14.25214	5.99719, 7.25118, 8.72916	6.11782, 1	3	ncapg	86603
GL173321.1	293939	294361	294027, 294048, 294027	73, 90, 68	88.99923, 109.80175, 89.07948	26.21156, 28.37991, 28.93848	84.71327,	3	ncapg	54383
GL173321.1	322630	322859	322783, 322764, 322828	8, 12, 8	8.42257, 11.86108, 10.1465	5.97763, 7.51216, 6.82176	5.76336, 9	3	ncapg	25885
GL173322.1	71937	72515	72399, 72286, 72267	13, 13, 6	14.0251, 11.56107, 5.95321	8.6006, 7.13311, 4.64162	11.03614,	3	cdk12	0
GL173322.1	116174	116599	116565, 116547, 116545	61, 72, 58	75.89615, 83.85128, 77.07962	25.18292, 23.97666, 27.45807	71.7405, 7	3	med1	0
GL173322.1	150086	150662	150514, 150294, 150188	13, 14, 9	11.98086, 12.86494, 10.07825	7.39024, 7.70954, 6.83102	9.10256, 1	3	fbxl20	0
GL173322.1	166667	167265	166709, 166697, 166688	39, 53, 38	38.80831, 53.22855, 43.13209	14.47711, 16.57657, 17.37613	35.12484,	3	ubp1	584
GL173322.1	170849	171320	170948, 170966, 171037	17, 30, 10	11.89307, 23.3024, 7.12656	6.39527, 9.20329, 4.94968	9.01841, 2	3	ubp1	0
GL173322.1	188749	189838	189291, 189038, 188970	10, 32, 16	8.70772, 28.66342, 16.83466	5.94148, 11.44736, 9.62725	6.03286, 2	3	ubp1	5887
GL173322.1	208589	209081	208758, 208757, 208764	19, 35, 13	18.67786, 34.646, 13.84064	9.92659, 13.70289, 8.49513	15.49416,	3	stac2	20746
GL173322.1	210296	210812	210524, 210395, 210778	9, 9, 7	6.57543, 5.06422, 6.1499	4.76012, 3.79406, 4.72762	4.08545, 2	3	stac2	19015
GL173322.1	230899	231522	231010, 231195, 231147	7, 9, 6	7.1696, 7.67202, 6.35308	5.31345, 5.44992, 4.85498	4.61046, 5	3	stac2	0
GL173322.1	369632	369878	369809, 369872, 369850	10, 15, 14	10.26482, 12.31186, 17.61756	6.86644, 7.13071, 10.40352	7.48624, 9	3	arl5c	11264
GL173322.1	374927	375504	375020, 375011, 375009	75, 90, 51	88.91885, 103.00874, 66.88151	25.1574, 25.0519, 25.14834	84.63412,	3	arl5c	16559
GL173322.1	411354	411876	411820, 411845, 411816	10, 15, 10	11.06011, 15.80067, 13.18184	7.306, 9.24574, 8.33771	8.22712, 1	3	fbxo47	49697
GL173322.1	496204	496820	496489, 496472, 496483	28, 48, 30	25.51056, 47.76506, 34.90543	11.046, 15.74155, 15.83834	22.11518,	3	lasp1	0
GL173322.1	531134	531864	531592, 531572, 531560	14, 20, 11	12.86267, 17.94395, 11.5511	7.70816, 9.1847, 7.48213	9.93295, 1	3	lasp1	5852
GL173322.1	538332	538974	538601, 538766, 538409	6, 7, 4	5.63898, 5.97725, 4.64325	4.46544, 4.62287, 3.77643	3.25772, 3	3	lasp1	13050
GL173322.1	545584	546464	546038, 545852, 545626	12, 8, 5	11.2086, 6.30984, 5.23257	7.12098, 4.72927, 4.20435	8.36821, 3	3	lasp1	20302
GL173323.1	121161	122308	121716, 121525, 121726	15, 41, 7	14.78606, 38.61066, 7.29952	8.64215, 13.60445, 5.38357	11.76448,	3	vars2	167006
GL173324.1	150	697	659, 661, 415	9, 12, 5	9.36986, 11.86108, 5.9506	6.44997, 7.51216, 4.54784	6.65425, 9	3	rnf4	120838
GL173324.1	242149	242750	242440, 242421, 242511	6, 9, 4	4.9641, 7.92635, 4.15126	4.06411, 5.60609, 3.54016	2.64509, 5	3	tnip2	0
GL173324.1	330023	330706	330082, 330340, 330426	9, 15, 9	9.72082, 15.47929, 9.64095	6.64182, 9.05625, 6.59867	6.97155, 1	3	tnip2	84695
GL173324.1	381304	381820	381705, 381669, 381733	14, 15, 9	13.03233, 13.4765, 10.28145	7.81172, 7.84563, 6.93568	10.09457,	3	add1	46746
GL173324.1	394949	395680	395557, 395584, 395581	11, 20, 13	11.35776, 19.30258, 14.25214	7.37194, 10.00482, 8.72916	8.51017, 1	3	add1	32886
GL173324.1	489956	490301	490171, 490045, 490067	5, 10, 6	4.82177, 7.38914, 6.4014	3.98509, 5.11603, 4.8799	2.51418, 4	3	mfsd10	0
GL173324.1	543439	544454	544146, 543907, 543858	11, 19, 13	9.00946, 17.11753, 13.90715	5.95596, 8.98159, 8.53326	6.31598, 1	3	NA	6733
GL173325.1	407487	408180	407864, 407777, 407971	6, 14, 7	5.47306, 13.75886, 8.646	4.36955, 8.2515, 6.04229	3.10811, 1	3	cog3	37293
GL173326.1	137094	137789	137512, 137327, 137548	11, 12, 6	12.17114, 9.99443, 6.35308	7.82679, 6.37457, 4.85498	9.28263, 7	3	ubl4a	0
GL173326.1	361967	362966	362256, 362169, 362734	16, 25, 10	16.01695, 21.78802, 10.1418	9.13983, 9.89653, 6.79622	12.9408, 1	3	sema3f	0
GL173326.1	443365	444033	443867, 443762, 443735	10, 16, 4	9.83337, 16.44448, 4.67325	6.61776, 9.39573, 3.78987	7.07841, 1	3	FOXP3	6327
GL173326.1	451368	451957	451688, 451893, 451679	12, 14, 10	10.68367, 11.49628, 10.42037	6.80062, 6.86757, 6.95441	7.88119, 8	3	FOXP3	0
GL173328.1	25509	25997	25929, 25818, 25910	6, 9, 5	5.1345, 8.21099, 5.59755	4.16817, 5.77858, 4.38538	2.80161, 5	3	trappc10	0
GL173328.1	56088	56643	56368, 56377, 56350	51, 84, 32	55.01747, 91.82454, 37.13674	18.36299, 22.45229, 16.36754	51.09443,	3	pwp2	9067
GL173328.1	81933	82437	82085, 82197, 82134	6, 6, 11	4.99728, 4.93312, 12.86886	4.08451, 4.04501, 8.19722	2.67584, 2	3	vtcn1	10911
GL173328.1	105404	106352	106312, 105519, 106112	12, 7, 3	13.8483, 5.97725, 3.47371	8.63436, 4.62287, 3.03189	10.86694,	3	vtcn1	0
GL173328.1	136162	136480	136300, 136304, 136336	12, 12, 13	11.79509, 11.37069, 14.69628	7.47301, 7.21896, 8.97644	8.92495, 8	3	vtcn1	14094
GL173328.1	152070	152608	152410, 152272, 152269	15, 13, 12	15.49092, 13.14514, 14.76955	9.06314, 8.09002, 9.10943	12.43497,	3	fstl1	5048
GL173328.1	187856	188588	188255, 188029, 188003	10, 8, 10	9.661, 6.11443, 11.24131	6.51661, 4.60556, 7.40241	6.9283, 3.7	3	lrrc58	0
GL173328.1	230597	231516	231125, 230994, 231148	11, 9, 5	11.15818, 7.39577, 5.31857	7.25692, 5.27837, 4.24819	8.32008, 4	3	sec22a	2241
GL173328.1	243893	244234	244041, 244182, 244183	6, 11, 8	4.04056, 8.00793, 7.67847	3.47431, 5.32781, 5.56054	1.89399, 5	3	gsk3b	14325
GL173328.1	257733	258270	258259, 258221, 258233	24, 27, 9	25.36666, 27.59392, 10.21256	12.3555, 12.62252, 6.90044	21.97541,	3	gsk3b	289
GL173328.1	265101	265642	265597, 265613, 265606	25, 37, 17	25.98133, 40.38358, 19.81591	12.3763, 16.19746, 11.12108	22.57216,	3	gsk3b	0
GL173328.1	278658	278956	278731, 278723, 278734	10, 17, 6	8.24182, 12.78176, 5.8719	5.65265, 6.91875, 4.59674	5.61182, 1	3	gsk3b	0
GL173328.1	331312	331705	331536, 331622, 331566	6, 8, 9	4.89903, 4.5773, 8.13839	4.02393, 3.61319, 5.73483	2.58474, 2	3	maats1	1661
GL173328.1	338413	339375	338783, 338772, 338678	31, 47, 13	30.75727, 48.34496, 11.54949	12.99764, 16.45617, 7.12599	27.23282,	3	cox17	358
GL173328.1	371617	371913	371762, 371747, 371802	23, 21, 12	23.56695, 18.48988, 11.81848	11.61446, 9.21025, 7.4869	20.22607,	3	popdc2	16342
GL173328.1	428219	428618	428442, 428287, 428322	14, 12, 7	13.57329, 11.86108, 7.72582	8.13982, 7.51216, 5.6058	10.60942,	3	hao2	4542
GL173328.1	429721	430091	430047, 429908, 429929	15, 8, 4	15.64184, 7.07159, 4.55201	9.15231, 5.20073, 3.73489	12.57992,	3	hao2	6044
GL173328.1	438775	439407	439083, 439117, 439002	15, 19, 6	14.39822, 17.70754, 7.29403	8.40782, 9.33888, 5.30581	11.39247,	3	hao2	15098
GL173328.1	439905	440452	439949, 439908, 440044	18, 20, 7	17.65252, 18.1164, 8.49722	9.59407, 9.28853, 5.97582	14.50784,	3	hao2	16228
GL173328.1	458291	458999	458807, 458797, 458727	15, 8, 6	17.48779, 7.07159, 7.29403	10.20672, 5.20073, 5.30581	14.34959,	3	TBX15	5610
GL173328.1	511193	511682	511552, 511556, 511541	11, 23, 16	11.35776, 23.90561, 19.25686	7.37194, 11.81781, 11.00387	8.51017, 2	3	U4	496
GL173329.1	110043	110961	110453, 110689, 110143, 110696	12, 17, 9, 11	10.99193, 13.05396, 7.83046, 10.10247	6.98928, 7.08107, 5.54742, 6.62982	8.17226, 1	3	NA	22928
GL173330.1	481731	482298	481875, 481982, 482048	12, 24, 12	11.2086, 22.39621, 14.06375	7.12098, 10.57939, 8.74831	8.36821, 1	3	NA	14307
GL173330.1	501647	502822	502157, 501914, 501859	9, 24, 15	5.66664, 18.11253, 13.8161	4.17853, 8.1203, 8.05343	3.28356, 1	3	NA	1457
GL173330.1	505457	506332	506132, 506144, 505592	10, 25, 9	7.31658, 20.39098, 7.06537	5.07009, 9.09981, 5.07099	4.74811, 1	3	NA	5267
GL173331.1	127964	128161	127994, 128016, 128007	172, 186, 119	219.69598, 241.29483, 162.74034	37.93555, 40.10043, 39.573	214.61276	3	prkx	52009
GL173333.1	12300	12790	12530, 12541, 12530	11, 8, 4	12.43693, 7.07159, 4.67325	7.97018, 5.20073, 3.78987	9.52692, 4	3	NA	35527
GL173333.1	38343	39003	38868, 38874, 38882	16, 28, 15	17.50585, 28.97121, 17.40815	10.02008, 13.07333, 10.16282	14.36688,	3	NA	9314
GL173333.1	63529	64233	63731, 63972, 63819	11, 23, 21	11.29029, 22.38211, 25.46511	7.3332, 10.90305, 13.3495	8.44541, 1	3	NA	0
GL173333.1	103921	104668	104400, 104279, 104072	9, 7, 5	8.50607, 5.97725, 5.85847	5.95453, 4.62287, 4.50665	5.84194, 3	3	NA	6009
GL173333.1	151363	152166	151705, 151712, 151664	8, 8, 8	8.42257, 7.07159, 10.1465	5.97763, 5.20073, 6.82176	5.76336, 4	3	NA	0
GL173333.1	157833	158501	158382, 158345, 158153	7, 19, 9	7.1696, 21.4153, 11.64359	5.31345, 11.55717, 7.57973	4.61046, 1	3	NA	0
GL173333.1	189630	190413	190106, 190101, 189845	12, 17, 7	12.90199, 16.43386, 7.96278	8.11489, 9.1315, 5.72394	9.97033, 1	3	NA	0
GL173333.1	266260	266789	266745, 266562, 266674	5, 10, 7	4.82177, 9.39128, 8.69489	3.98509, 6.35644, 6.06379	2.51418, 6	3	NA	425
GL173333.1	273996	274946	274517, 274444, 274501	7, 8, 7	7.1696, 7.07159, 8.69489	5.31345, 5.20073, 6.06379	4.61046, 4	3	NA	667
GL173333.1	319370	319768	319374, 319420, 319388	51, 84, 56	62.26951, 103.2267, 74.13675	22.45457, 28.07514, 26.80284	58.25733,	3	NA	0
GL173333.1	441529	441887	441653, 441782, 441730	11, 14, 6	10.43535, 12.09105, 7.12188	6.83063, 7.23439, 5.22884	7.6474, 9.3	3	khsrp	0
GL173333.1	465681	465999	465856, 465899, 465910	14, 8, 16	12.86267, 4.85726, 15.65308	7.70816, 3.7949, 8.92046	9.93295, 2	3	zswim4	189
GL173333.1	490325	491122	490860, 490838, 490991	8, 5, 9	7.47668, 3.94561, 9.24728	5.44269, 3.46715, 6.38161	4.89713, 1	3	xtr-mir-23a-1	6068
GL173334.1	138042	138585	138382, 138120, 138176	11, 16, 9	10.00935, 16.01323, 10.56949	6.57321, 9.13759, 7.08032	7.24529, 1	3	NA	9281
GL173334.1	145430	146137	145481, 145497, 145502	12, 9, 9	12.11599, 7.43399, 10.14484	7.66241, 5.30221, 6.86555	9.23027, 4	3	NA	1729
GL173334.1	223799	224486	224354, 224045, 224409	8, 6, 4	8.42257, 4.93312, 4.34116	5.97763, 4.04501, 3.63492	5.76336, 2	3	tecta.2	8936
GL173334.1	271539	271989	271727, 271727, 271757	9, 12, 5	7.71701, 9.74388, 4.84526	5.47767, 6.2187, 3.99806	5.12244, 7	3	b4galt3	34442
GL173334.1	272988	273476	273430, 273373, 273369	14, 21, 10	13.26657, 20.39593, 9.88017	7.95422, 10.35516, 6.64506	10.31735,	3	b4galt3	32955
GL173334.1	293111	293846	293412, 293436, 293352	12, 9, 8	12.60209, 6.58184, 10.0913	7.9443, 4.7642, 6.79758	9.68421, 4	3	b4galt3	12585
GL173334.1	335979	336442	335998, 336033, 336026	41, 75, 43	54.26364, 96.07326, 59.49041	22.58075, 29.02243, 24.71149	50.35048,	3	b4galt3	9728
GL173334.1	377738	378058	378017, 378054, 378031	11, 25, 8	12.33515, 30.17196, 10.1465	7.91559, 14.85972, 6.82176	9.43699, 2	3	NA	15870
GL173334.1	395514	396434	395541, 395597, 396255	8, 11, 8	8.42257, 9.4823, 8.62637	5.97763, 6.24973, 6.08668	5.76336, 6	3	NA	850
GL173334.1	425975	426656	426441, 426264, 426219	8, 9, 9	7.33538, 6.7053, 9.64095	5.35908, 4.84282, 6.59867	4.76518, 4	3	NA	31311
GL173334.1	431697	432451	431885, 431920, 431837	9, 10, 10	7.75911, 7.68845, 9.98291	5.5036, 5.30522, 6.70471	5.16246, 5	3	NA	37033
GL173335.1	17	1190	343, 517, 812	25, 19, 17	30.06855, 21.4153, 21.64669	14.7997, 11.55717, 12.11304	26.55945,	3	NA	50721
GL173335.1	9055	11379	9370, 9327, 10579	23, 32, 20	16.44627, 22.80082, 15.10764	7.47622, 8.41383, 7.50876	13.35256,	3	NA	40532
GL173335.1	13902	15119	14351, 14355, 14360	28, 31, 18	21.7495, 20.89272, 13.14526	8.96174, 7.75461, 6.88427	18.46345,	3	NA	36792
GL173335.1	20301	21052	20992, 20877, 20652	12, 10, 6	13.8483, 9.39128, 6.08001	8.63436, 6.35644, 4.71063	10.86694,	3	NA	30859
GL173335.1	24205	24825	24305, 24445, 24422	6, 13, 11	5.96715, 13.14514, 12.41242	4.64927, 8.09002, 7.95707	3.52158, 1	3	NA	27086
GL173335.1	29438	29929	29800, 29842, 29894	12, 14, 7	13.47263, 14.45897, 8.21885	8.43173, 8.66788, 5.84717	10.51396,	3	NA	21982
GL173335.1	290602	291006	290836, 290759, 290775	10, 15, 5	9.13239, 13.80611, 5.45388	6.2007, 8.04733, 4.3157	6.43114, 1	3	dhx15	20801
GL173335.1	314446	315014	314854, 314758, 314867	8, 9, 10	6.24296, 6.58184, 9.58601	4.68703, 4.7642, 6.47231	3.77872, 4	3	dhx15	44645
GL173335.1	418211	418973	418512, 418809, 418749	6, 6, 8	5.24248, 4.68027, 7.72242	4.2332, 3.88713, 5.58595	2.89962, 2	3	dhx15	148410
GL173335.1	506681	507242	507160, 507153, 507147	25, 24, 6	25.64823, 24.70533, 7.29403	12.17691, 11.95904, 5.30581	22.24852,	3	dhx15	236880
GL173336.1	514780	515446	514949, 515069, 515240	10, 7, 6	9.54985, 5.97725, 6.82211	6.45088, 4.62287, 5.08889	6.82341, 3	3	fst	36786
GL173337.1	39735	40232	40004, 40169, 40156	8, 11, 6	8.42257, 10.60895, 7.25145	5.97763, 6.9343, 5.287	5.76336, 7	3	NA	9618
GL173337.1	448344	448735	448467, 448375, 448703	5, 8, 4	4.82177, 7.07159, 4.67325	3.98509, 5.20073, 3.78987	2.51418, 4	3	prss1.2	33571
GL173338.1	74250	75236	74534, 74809, 74688	10, 13, 4	11.06011, 13.1283, 4.67325	7.306, 8.08007, 3.78987	8.22712, 1	3	sec14l1	0
GL173338.1	89092	89779	89247, 89246, 89266	7, 10, 3	5.79309, 8.12616, 3.47371	4.5096, 5.58036, 3.03189	3.39779, 5	3	sec14l1	0
GL173338.1	115272	116192	115801, 115448, 115914	7, 13, 5	6.33443, 9.85593, 4.95468	4.83795, 6.07784, 4.05779	3.86259, 7	3	sec14l1	0
GL173338.1	153143	153391	153332, 153332, 153357	10, 9, 8	8.89113, 7.06866, 7.11599	6.05396, 5.07306, 5.22758	6.20456, 4	3	NA	15997
GL173338.1	182013	182329	182042, 182047, 182025	16, 23, 14	16.60441, 20.65165, 15.42335	9.49088, 9.87151, 9.2256	13.50404,	3	NA	44867
GL173338.1	343032	343633	343156, 343149, 343229	24, 11, 9	27.83747, 10.60895, 10.72164	13.82444, 6.9343, 7.15493	24.38123,	3	rogdi	21387
GL173338.1	519964	520707	520461, 520249, 520512	11, 14, 6	9.71398, 11.85481, 6.5517	6.39253, 7.08876, 4.95623	6.97155, 9	3	NA	4441
GL173338.1	527915	528461	528180, 528163, 528163	30, 32, 23	32.49002, 34.71953, 29.30255	14.4026, 14.9339, 14.96427	28.92807,	3	NA	0
GL173341.1	14036	14694	14298, 14135, 14278	9, 12, 9	7.6342, 10.88526, 9.64095	5.42655, 6.92415, 6.59867	5.04509, 8	3	gfra1	9897
GL173341.1	76300	76858	76818, 76518, 76708	8, 7, 7	6.94372, 5.97725, 7.40077	5.12297, 4.62287, 5.43746	4.42528, 3	3	gfra1	0
GL173341.1	103908	104598	103939, 103936, 103937	55, 89, 59	68.2775, 113.90413, 78.47203	23.9154, 31.03662, 27.73337	64.19886,	3	gfra1	5448
GL173341.1	143958	144389	144095, 144145, 144111	6, 8, 5	5.86227, 6.89656, 5.9506	4.59138, 5.09414, 4.54784	3.46055, 4	3	gfra1	45498
GL173341.1	164019	164271	164048, 164047, 164053	18, 24, 11	16.33168, 20.63686, 12.06254	8.79227, 9.54685, 7.76744	13.24212,	3	gfra1	65559
GL173341.1	216045	216300	216298, 216297, 216193	10, 13, 6	11.06011, 13.14514, 6.12365	7.306, 8.09002, 4.73409	8.22712, 1	3	gfra1	117585
GL173341.1	236187	237101	236323, 236274, 236832, 236849	15, 11, 8, 12	14.46121, 7.85056, 5.35923, 11.98157	8.44598, 5.22892, 4.12054, 7.58338	11.45219,	3	gfra1	137727
GL173341.1	280553	280859	280751, 280715, 280818	7, 9, 8	7.1696, 8.15232, 8.74279	5.31345, 5.74324, 6.14792	4.61046, 5	3	atrnl1	106214
GL173341.1	285421	286306	285691, 285602, 285987, 285626, 285994	11, 14, 5, 4, 5	12.17114, 14.22787, 3.94561, 3.79082, 5.40789	7.82679, 8.53137, 3.46715, 3.34814, 4.29296	9.28263, 1	3	atrnl1	100767
GL173341.1	295891	296211	296146, 296193, 296152	13, 32, 15	9.73159, 26.11837, 13.0803	6.00089, 10.07742, 7.60258	6.98212, 2	3	atrnl1	90862
GL173341.1	343897	344762	344654, 344465, 344196	10, 5, 8	10.67246, 3.94561, 10.1465	7.09497, 3.46715, 6.82176	7.87096, 1	3	atrnl1	42311
GL173341.1	425239	425537	425265, 425400, 425311	9, 17, 6	9.72082, 18.56115, 7.29403	6.64182, 10.40145, 5.30581	6.97155, 1	3	atrnl1	0
GL173341.1	434051	435430	434936, 434934, 434457	8, 11, 8	7.67464, 10.60895, 10.1465	5.55832, 6.9343, 6.82176	5.08299, 7	3	atrnl1	0
GL173341.1	485767	485977	485797, 485819, 485797	25, 28, 17	28.66822, 28.31419, 18.13503	13.97856, 12.68363, 10.15123	25.19216,	3	atrnl1	27430
GL173341.1	564287	565148	564465, 564670, 564897	14, 26, 8	11.14378, 21.01653, 6.15779	6.65015, 9.1497, 4.63309	8.30717, 1	3	fam160b1	176
GL173343.1	25118	25708	25263, 25302, 25291	28, 33, 27	21.44011, 23.6332, 25.22187	8.79807, 8.55727, 11.22324	18.16397,	3	NA	6233
GL173343.1	27456	27940	27589, 27603, 27661	32, 47, 25	25.79377, 39.09033, 22.63838	9.90819, 11.82726, 10.39091	22.39052,	3	NA	4001
GL173343.1	28566	29599	29109, 29287, 29441	13, 17, 8	6.79296, 8.39389, 4.55211	4.23629, 4.47278, 3.59685	4.28535, 5	3	NA	2342
GL173343.1	110056	111049	110508, 110447, 110439	10, 34, 7	10.39648, 35.5202, 8.49722	6.94096, 14.6006, 5.97582	7.61003, 3	3	NA	13361
GL173343.1	130654	131054	130943, 130915, 130861	9, 15, 4	7.80169, 13.74993, 4.46488	5.52977, 8.013, 3.69425	5.20141, 1	3	NA	33959
GL173343.1	148419	149066	148465, 148432, 148824	7, 17, 6	7.1696, 18.56115, 7.29403	5.31345, 10.40145, 5.30581	4.61046, 1	3	NA	51724
GL173343.1	174935	175454	175388, 175075, 175278	6, 6, 7	4.83561, 4.35338, 7.66926	3.98453, 3.67832, 5.57703	2.52606, 2	3	NA	78240
GL173343.1	264875	265622	265012, 265506, 265059	4, 9, 11	3.74195, 7.88274, 12.9493	3.32091, 5.57944, 8.23866	1.6222, 5.4	3	NA	101827
GL173343.1	409911	410463	409969, 409978, 410112	8, 16, 7	8.42257, 16.01323, 8.69489	5.97763, 9.13759, 6.06379	5.76336, 1	3	NA	5059
GL173343.1	423711	424530	423784, 423985, 424000	6, 7, 5	5.31708, 5.05277, 5.31857	4.2777, 4.04098, 4.24819	2.96727, 2	3	plekha7	944
GL173343.1	512935	513858	513180, 513182, 513255	12, 6, 11	13.8483, 4.93312, 11.86426	8.63436, 4.04501, 7.65794	10.86694,	3	SNORD14	16339
GL173344.1	45036	45427	45371, 45298, 45344	7, 6, 7	6.12952, 4.32803, 6.25413	4.71533, 3.66192, 4.79015	3.67231, 2	3	DAB2IP	0
GL173344.1	61511	62108	61671, 62082, 61910	6, 14, 5	5.55471, 11.67268, 5.75061	4.41698, 6.97641, 4.45735	3.18192, 8	3	DAB2IP	0
GL173344.1	101336	101880	101498, 101805, 101798	11, 8, 10	11.6377, 7.07159, 11.24131	7.53109, 5.20073, 7.40241	8.77589, 4	3	DAB2IP	27158
GL173344.1	130691	131401	131072, 131069, 130740	12, 37, 13	11.37748, 37.96311, 13.70995	7.22306, 14.80011, 8.41989	8.52862, 3	3	DAB2IP	56513
GL173344.1	146965	147452	147012, 146999, 147095	11, 20, 7	10.49148, 18.41223, 6.6707	6.86423, 9.46689, 5.03432	7.70044, 1	3	DAB2IP	72787
GL173344.1	172111	172651	172158, 172534, 172601	8, 8, 5	8.22568, 7.07159, 5.9506	5.8701, 5.20073, 4.54784	5.59681, 4	3	DAB2IP	97933
GL173344.1	218336	218547	218413, 218364, 218363	8, 11, 4	6.90289, 8.66477, 3.91395	5.09802, 5.74034, 3.41551	4.38737, 6	3	DAB2IP	144158
GL173344.1	228892	229704	229180, 229610, 229204	10, 12, 3	8.75281, 9.99443, 3.17999	5.9692, 6.37457, 2.89245	6.07523, 7	3	DAB2IP	154714
GL173344.1	255872	256271	256176, 256203, 256233	14, 12, 8	16.7649, 11.86108, 10.1465	9.96273, 7.51216, 6.82176	13.6543, 9	3	DAB2IP	181694
GL173344.1	276527	277004	276605, 276531, 276571	15, 30, 21	15.71867, 31.86026, 26.35131	9.19755, 14.02881, 13.84438	12.65377,	3	DAB2IP	202349
GL173344.1	288411	289073	288656, 288916, 288852	9, 10, 7	9.72082, 7.26311, 8.646	6.64182, 5.03621, 6.04229	6.97155, 4	3	DAB2IP	214233
GL173344.1	331641	332063	331850, 331849, 332001	32, 58, 31	29.48894, 59.50755, 34.95884	11.90655, 17.74028, 15.4759	25.99357,	3	DAB2IP	257463
GL173344.1	364582	364833	364702, 364702, 364801	13, 11, 10	13.20994, 10.15827, 11.10454	8.12823, 6.66366, 7.32977	10.26285,	3	DAB2IP	290404
GL173344.1	445170	445575	445574, 445563, 445567	30, 28, 40	35.92026, 31.93789, 54.59982	16.43872, 14.84014, 23.21866	32.28851,	3	DAB2IP	370992
GL173344.1	459582	460125	459602, 459741, 459686	7, 17, 4	5.05064, 13.21469, 3.51503	4.03961, 7.17728, 3.19081	2.72521, 1	3	DAB2IP	385404
GL173344.1	462214	463596	462776, 462775, 462336, 462776	10, 19, 4, 10	8.57531, 15.39581, 3.46461, 10.71812	5.85982, 7.94776, 3.1611, 7.12015	5.90712, 1	3	DAB2IP	388036
GL173344.1	484995	485252	485226, 485218, 485209	13, 18, 10	12.68462, 14.67054, 10.3632	7.81602, 7.78766, 6.92219	9.76298, 1	3	DAB2IP	410817
GL173344.1	491886	492768	492329, 492373, 492506	6, 14, 11	5.96715, 14.29802, 13.83651	4.64927, 8.57291, 8.67735	3.52158, 1	3	DAB2IP	417708
GL173344.1	508724	509499	509422, 509422, 509422	29, 41, 16	32.76592, 42.86565, 18.3512	14.95372, 15.93503, 10.50324	29.1976, 3	3	DAB2IP	434546
GL173344.1	535509	536234	536188, 536185, 536189	19, 23, 10	17.87921, 22.88804, 9.82971	9.44292, 11.20666, 6.61563	14.72728,	3	DAB2IP	461331
GL173345.1	6914	7465	7123, 7145, 6995	7, 6, 5	4.99677, 4.06564, 4.57714	4.00483, 3.49079, 3.84705	2.67584, 1	3	NA	16953
GL173345.1	9139	9865	9416, 9625, 9737	7, 12, 5	4.99677, 10.16897, 4.45433	4.00483, 6.4829, 3.77574	2.67584, 7	3	NA	14553
GL173345.1	16150	17184	16574, 16571, 16573	42, 46, 27	36.00106, 40.28168, 26.87418	11.92532, 12.7006, 12.1951	32.36834,	3	NA	7234
GL173345.1	39341	40696	40378, 39791, 40197	8, 9, 7	8.42257, 8.01512, 8.57075	5.97763, 5.66016, 6.00887	5.76336, 5	3	NA	0
GL173345.1	46451	46986	46530, 46560, 46533	17, 35, 15	13.18767, 32.95099, 14.32502	7.16109, 12.75182, 8.36342	10.24314,	3	NA	0
GL173345.1	89493	90179	89795, 89773, 89783	9, 13, 11	5.93508, 10.55511, 11.49087	4.3505, 6.51132, 7.44794	3.52158, 7	3	NA	3064
GL173345.1	114264	114680	114309, 114295, 114305	30, 21, 22	35.54458, 20.74722, 26.79743	16.21686, 10.56702, 13.83268	31.92042,	3	NA	0
GL173345.1	200783	201259	200854, 200898, 200940	10, 14, 7	10.39648, 13.44539, 8.646	6.94096, 8.06258, 6.04229	7.61003, 1	3	rpl13a	19935
GL173345.1	220637	220954	220836, 220836, 220840	9, 13, 6	7.88835, 9.71274, 6.25896	5.58287, 5.98923, 4.80589	5.28191, 7	3	rpl13a	240
GL173345.1	226378	227467	227168, 227194, 226608, 227069	9, 35, 6, 9	6.28237, 31.75303, 4.64903, 7.79368	4.57292, 12.09485, 3.8674, 5.52485	3.81457, 2	3	rpl13a	483
GL173345.1	230590	231263	230937, 230807, 230930	11, 14, 13	8.19386, 8.0323, 11.77124	5.44467, 4.7796, 7.26208	5.56766, 5	3	rps11	1950
GL173345.1	240168	240815	240682, 240731, 240701	21, 35, 19	18.13445, 30.59064, 20.47556	8.99874, 11.47103, 11.00494	14.9725, 2	3	rps11	1257
GL173345.1	258409	258943	258889, 258803, 258687	9, 10, 4	9.72082, 8.88645, 4.67325	6.64182, 6.0511, 3.78987	6.97155, 6	3	trim25	7496
GL173345.1	287279	287682	287511, 287374, 287446	11, 12, 4	10.78278, 10.93692, 4.55201	7.03733, 6.95571, 3.73489	7.97565, 8	3	trim25	14426
GL173345.1	302551	303172	303057, 303035, 303072	17, 18, 5	17.88146, 17.65791, 5.9506	10.00115, 9.59733, 4.54784	14.72916,	3	trim25	29698
GL173345.1	328671	329519	328967, 329040, 329063	11, 12, 21	11.42621, 11.86108, 25.2565	7.41109, 7.51216, 13.23126	8.57514, 9	3	fam214b	13323
GL173345.1	338245	338516	338485, 338469, 338464	12, 19, 5	12.60209, 21.01008, 5.69847	7.9443, 11.32031, 4.4331	9.68421, 1	3	fam214b	4326
GL173345.1	416291	416901	416610, 416834, 416613	8, 12, 8	7.19971, 10.44309, 8.80246	5.27799, 6.65248, 6.179	4.63758, 7	3	vcp	686
GL173345.1	460550	461124	460783, 460809, 460984	5, 7, 6	4.82177, 5.97725, 7.18591	3.98509, 4.62287, 5.25776	2.51418, 3	3	dnajb5	10395
GL173345.1	510746	511576	511091, 510923, 511098	5, 15, 11	3.9718, 12.01071, 11.49087	3.48353, 6.9461, 7.44794	1.83149, 9	3	vwa5a	6376
GL173345.1	524493	525027	524876, 524977, 524958	10, 16, 8	10.6018, 14.92393, 8.74279	7.05583, 8.47764, 6.14792	7.80484, 1	3	vwa5a	20123
GL173347.1	15719	17401	16810, 16219, 17244, 16726	13, 25, 8, 13	11.26158, 25.34813, 5.51195, 13.0991	6.94861, 11.99737, 4.21928, 8.06282	8.41898, 2	3	NA	0
GL173347.1	66824	67469	67053, 67061, 67142	13, 15, 12	12.03613, 13.16372, 13.15613	7.42394, 7.65379, 8.25729	9.15434, 1	3	metrnl	0
GL173347.1	84063	84576	84187, 84365, 84206	7, 15, 5	6.79162, 12.26789, 5.54867	5.10336, 7.10374, 4.3619	4.28406, 9	3	metrnl	10760
GL173347.1	193884	194663	194303, 194060, 194281	10, 7, 6	11.06011, 5.96795, 6.50067	7.306, 4.61718, 4.93052	8.22712, 3	3	trim16	28167
GL173347.1	253079	253385	253099, 253110, 253110	56, 78, 46	64.27989, 93.01241, 61.17808	21.15669, 25.80294, 24.28708	60.24439,	3	trim16	87362
GL173347.1	267553	267839	267717, 267767, 267795	13, 25, 8	11.66066, 24.37073, 8.80246	7.19428, 11.41406, 6.179	8.79778, 2	3	trim16	101836
GL173347.1	270615	270896	270853, 270845, 270842	15, 14, 12	15.19946, 11.71767, 14.23156	8.88991, 7.00416, 8.83588	12.15964,	3	trim16	104898
GL173347.1	291195	291757	291727, 291725, 291714	37, 45, 26	47.73378, 56.17128, 37.05589	20.5251, 21.82674, 18.35144	43.91362,	3	NA	123360
GL173347.1	302539	302995	302563, 302878, 302605	8, 8, 7	8.16648, 7.07159, 8.42533	5.83735, 5.20073, 5.94313	5.54098, 4	3	NA	112122
GL173347.1	332480	333217	332905, 332947, 332546, 332904	9, 15, 3, 5	8.20841, 13.31811, 3.41347, 5.80393	5.77703, 7.74852, 3.00444, 4.48186	5.58112, 1	3	NA	81900
GL173347.1	351302	351887	351607, 351603, 351465	13, 21, 11	13.5657, 19.11862, 11.92945	8.33634, 9.58634, 7.69409	10.60274,	3	NA	63230
GL173347.1	376047	376906	376260, 376465, 376392	16, 7, 7	16.30427, 4.89556, 7.40077	9.31205, 3.93927, 5.43746	13.21554,	3	NA	38211

GL173347.1	397413	397955	397521, 397435, 397513	20, 24, 17	20.21889, 22.99283, 20.77652	10.55812, 10.93416, 11.65116	16.98202,	3	NA	17162
GL173347.1	432096	432740	432368, 432166, 432232	9, 11, 8	6.42583, 7.49521, 7.85773	4.66464, 5.00592, 5.66361	3.94676, 5	3	NA	0
GL173347.1	447072	447336	447310, 447314, 447129	18, 22, 8	18.22458, 19.15079, 8.80246	9.93936, 9.2973, 6.179	15.0589, 1	3	NA	0
GL173347.1	449044	449508	449095, 449310, 449360	11, 18, 7	9.34791, 13.7776, 7.01416	6.16652, 7.25561, 5.22813	6.63394, 1	3	NA	0
GL173347.1	459981	460311	460116, 460114, 460112	186, 144, 112	215.85121, 161.51337, 134.55461	30.91854, 27.21576, 29.52206	210.7854,	3	NA	227
GL173347.1	472538	473245	472689, 472830, 472766	9, 20, 8	9.72082, 22.87459, 10.1465	6.64182, 12.13503, 6.82176	6.97155, 1	3	NA	12784
GL173347.1	492842	493197	493159, 493158, 493106	11, 11, 7	12.43693, 10.60895, 8.69489	7.97018, 6.9343, 6.06379	9.52692, 7	3	NA	33088
GL173348.1	28271	28561	28383, 28401, 28405	12, 13, 9	13.05874, 11.92581, 10.56949	8.20296, 7.35664, 7.08032	10.11961,	3	XB-GENE-5722383	273165
GL173348.1	29127	29743	29577, 29466, 29614	10, 19, 10	10.39648, 19.48976, 11.997	6.94096, 10.41622, 7.78835	7.61003, 1	3	XB-GENE-5722383	271983
GL173348.1	206395	206644	206417, 206429, 206497	23, 27, 22	28.90671, 32.02844, 27.46822	14.74388, 15.25977, 14.21027	25.42528,	3	XB-GENE-5722383	95082
GL173348.1	224938	225642	225513, 225215, 225022	7, 9, 5	5.24783, 6.37567, 4.07202	4.16625, 4.63258, 3.54571	2.90454, 4	3	XB-GENE-5722383	76084
GL173348.1	229723	230947	230058, 230344, 230055	5, 21, 15	3.54515, 16.93576, 15.36085	3.21097, 8.29331, 8.986	1.57763, 1	3	XB-GENE-5722383	70779
GL173348.1	229723	230947	230593, 230344, 230055	8, 21, 15	6.63031, 16.93576, 15.36085	4.92991, 8.29331, 8.986	4.13604, 1	3	XB-GENE-5722383	70779
GL173348.1	245287	245767	245478, 245536, 245510	20, 25, 13	19.15409, 25.75015, 14.69628	9.91507, 12.23791, 8.97644	15.95382,	3	XB-GENE-5722383	55959
GL173348.1	278590	279461	278904, 278639, 279068	7, 9, 7	7.1696, 8.21099, 7.66926	5.31345, 5.77858, 5.57703	4.61046, 5	3	XB-GENE-5722383	22265
GL173348.1	309715	310117	309962, 309977, 309908	14, 20, 7	14.10012, 19.30258, 7.72582	8.4555, 10.00482, 5.6058	11.10747,	3	XB-GENE-5722383	662
GL173348.1	322664	323058	322953, 322973, 322873	8, 18, 7	7.02691, 14.76178, 7.96278	5.17362, 7.84242, 5.72394	4.50222, 1	3	XB-GENE-5722383	13611
GL173348.1	336600	337997	337063, 337025, 337024	25, 28, 20	27.33487, 29.57832, 23.93369	13.18598, 13.43449, 12.7427	23.89047,	3	NA	7463
GL173348.1	354584	355050	354831, 354818, 354829	23, 25, 18	24.58488, 23.61075, 20.23857	12.225, 10.96304, 11.13122	21.21468,	3	NA	0
GL173348.1	378042	378408	378155, 378155, 378155	6, 18, 13	5.72604, 16.69883, 15.0131	4.51498, 9.01539, 9.14923	3.33547, 1	3	NA	0
GL173348.1	417313	418650	417507, 417919, 417507	10, 20, 14	10.26482, 19.64549, 16.29325	6.86644, 10.21207, 9.7093	7.48624, 1	3	NA	14040
GL173348.1	494887	495126	495093, 495102, 495085	19, 20, 23	21.91879, 18.78201, 30.34798	11.84839, 9.69018, 15.53488	18.62754,	3	NA	4654
GL173349.1	11629	12015	11882, 11887, 11681	9, 9, 5	9.24298, 8.21099, 5.9506	6.3792, 5.77858, 4.54784	6.53474, 5	3	NA	88443
GL173349.1	52934	53204	53039, 53100, 53033	10, 19, 7	10.81731, 17.76909, 8.21885	7.17456, 9.37618, 5.84717	8.00764, 1	3	NA	47254
GL173349.1	305078	305470	305347, 305368, 305380	12, 14, 7	12.38809, 13.50676, 7.96278	7.82099, 8.09967, 5.72394	9.48755, 1	3	p2ry12	5368
GL173349.1	330559	331009	330811, 330675, 330794	5, 7, 7	4.50727, 5.71889, 7.29952	3.80664, 4.46356, 5.38357	2.30125, 3	3	p2ry12	18697
GL173349.1	352997	353483	353433, 353308, 353419	15, 13, 8	14.78606, 12.43433, 8.80246	8.64215, 7.66548, 6.179	11.76448,	3	igsf10	29746
GL173349.1	480182	480633	480398, 480416, 480378	8, 17, 10	8.42257, 15.69342, 11.5283	5.97763, 8.68127, 7.55211	5.76336, 1	3	aadac	34804
GL173349.1	488729	489510	489193, 489267, 489272	10, 26, 9	9.60506, 28.39602, 11.39481	6.48358, 13.46449, 7.46977	6.87658, 2	3	aadac	25927
GL173350.1	30169	31016	30597, 30535, 30632	6, 20, 14	5.3552, 19.37003, 16.29325	4.3003, 10.0456, 9.7093	3.00253, 1	3	vcl	46511
GL173350.1	155500	156449	156248, 155907, 155745	12, 26, 9	10.99193, 27.13121, 10.01275	6.98928, 12.7086, 6.79683	8.17226, 2	3	vcl	0
GL173351.1	109166	110379	109441, 109441, 109441	32, 34, 29	35.08498, 34.57293, 37.78184	15.1502, 14.05129, 17.88422	31.47012,	3	NA	12967
GL173351.1	190932	191290	191233, 190973, 191092	8, 6, 5	7.47668, 4.73925, 5.27517	5.44269, 3.92426, 4.22616	4.89713, 2	3	CDC20B	24319
GL173351.1	270386	270761	270428, 270398, 270415	18, 39, 13	18.84377, 40.58608, 13.90715	10.31044, 15.47636, 8.53326	15.65357,	3	MCIDAS	2146
GL173351.1	498850	499663	499440, 499450, 499430	13, 21, 14	12.62236, 21.57397, 15.20637	7.77868, 11.06508, 9.10193	9.70369, 1	3	U1	0
GL173351.1	505167	506283	505899, 505893, 506010	23, 20, 22	12.90486, 8.67669, 13.06703	5.66898, 4.18235, 5.94035	9.97288, 6	3	U1	0
GL173352.1	8541	9702	9608, 9356, 9123	6, 9, 5	4.25277, 6.52169, 4.17451	3.61311, 4.72584, 3.60852	2.08209, 4	3	NA	143047
GL173352.1	115453	116593	116337, 115811, 116132	20, 19, 10	17.10419, 13.52551, 10.03522	8.68132, 6.85674, 6.73493	13.9806, 1	3	NA	36156
GL173352.1	132216	133155	132926, 132913, 132511	62, 79, 37	63.90886, 78.99213, 37.9539	18.30131, 18.69192, 14.79486	59.87754,	3	NA	19594
GL173352.1	263694	264431	264208, 264205, 264208	135, 133, 87	148.26579, 136.11919, 96.33981	25.66058, 22.11056, 23.25911	143.55074	3	NA	0
GL173352.1	419094	420366	420022, 419643, 419613	6, 14, 7	5.3939, 12.97647, 7.2501	4.32314, 7.77766, 5.35703	3.0374, 10	3	chmp5	15969
GL173353.1	383447	384452	383761, 384207, 383939	8, 13, 5	7.1122, 11.17268, 4.84526	5.22529, 6.89372, 3.99806	4.57946, 8	3	gadd45b	3604
GL173354.1	299532	300003	299718, 299826, 299770	7, 11, 5	6.55439, 9.39347, 4.88115	4.96711, 6.19476, 4.01777	4.06616, 6	3	pkn2	126093
GL173355.1	48735	49161	48841, 48971, 48826	9, 17, 6	7.55321, 15.63512, 7.25145	5.37637, 8.64575, 5.287	4.96981, 1	3	STUM	17217
GL173355.1	85769	87086	86605, 86603, 86612	190, 217, 148	166.23303, 195.68317, 146.30214	17.09371, 18.40432, 21.01626	161.42001	3	parp1	41958
GL173355.1	96945	97480	97426, 97412, 97412	28, 36, 25	30.98295, 37.44002, 29.93842	14.27172, 14.9052, 14.72403	27.45364,	3	parp1	31564
GL173355.1	111435	112560	111749, 111749, 111749	32, 48, 22	37.03852, 56.26148, 26.79743	16.30926, 20.44083, 13.83268	33.3866, 5	3	parp1	16484
GL173355.1	124543	125060	124778, 124787, 124795	32, 67, 28	35.18635, 75.44024, 31.1707	15.21026, 21.96564, 14.3836	31.56988,	3	parp1	3984
GL173355.1	128219	128661	128538, 128522, 128531	9, 11, 15	6.3106, 5.65919, 13.52972	4.59097, 3.87629, 7.87823	3.84106, 3	3	parp1	383
GL173355.1	159647	160628	160024, 159980, 160103	11, 27, 8	10.96672, 26.96051, 8.24665	7.14544, 12.24626, 5.88165	8.14942, 2	3	lin9	0
GL173355.1	269063	269526	269331, 269302, 269330	18, 21, 10	19.43049, 20.67581, 10.53703	10.65863, 10.52396, 7.01977	16.22032,	3	acbd3	10437
GL173355.1	301717	302487	301759, 301996, 302302	5, 8, 6	4.47183, 6.4822, 6.87953	3.78598, 4.83755, 5.11627	2.26935, 4	3	NA	0
GL173355.1	342612	343046	342985, 342995, 342977	13, 8, 7	14.61367, 7.07159, 8.21885	8.93088, 5.20073, 5.84717	11.59864,	3	h3f3b	1338
GL173355.1	367754	368126	367932, 367910, 367980	8, 20, 11	4.84648, 15.76665, 9.48032	3.7879, 7.891, 6.24851	2.5362, 12	3	sde2	3233
GL173355.1	370550	370860	370679, 370661, 370673	31, 34, 17	32.24754, 32.77861, 17.51936	13.8697, 13.02656, 9.78549	28.69044,	3	sde2	499
GL173355.1	404768	405675	404984, 405115, 405115	20, 33, 18	19.08776, 31.04172, 18.66986	9.87497, 12.41812, 10.20656	15.89017,	3	tmem63a	4127
GL173355.1	409959	410553	410400, 410458, 410267	37, 46, 23	41.38441, 44.5763, 25.2841	16.78306, 14.85509, 12.64245	37.65983,	3	tmem63a	0
GL173355.1	437872	438290	438249, 438261, 438259	7, 11, 9	6.25077, 9.4823, 11.64359	4.78814, 6.24973, 7.57973	3.78441, 6	3	ephx1	768
GL173355.1	449653	450194	449715, 449736, 449846	7, 9, 4	6.33443, 7.13885, 3.97874	4.83795, 5.11729, 3.45022	3.86259, 4	3	ephx1	0
GL173356.1	234156	234374	234349, 234328, 234347	39, 70, 27	45.31759, 86.22749, 36.3158	18.22001, 26.25879, 17.70214	41.53417,	3	pdcl	542
GL173356.1	324039	324978	324523, 324523, 324522	34, 33, 14	37.38918, 34.12088, 14.53512	15.69656, 14.18344, 8.71263	33.72985,	3	anapc13.1	8785
GL173356.1	428802	429302	429066, 429037, 429062	9, 7, 6	9.63662, 5.97725, 7.29403	6.59633, 4.62287, 5.30581	6.90556, 3	3	f8	8991
GL173357.1	39111	40269	39552, 39413, 39982	20, 15, 7	18.70067, 11.01021, 6.36213	9.64104, 6.33676, 4.85436	15.51577,	3	TOMM40L	0
GL173357.1	142429	143050	142625, 142838, 142521	13, 13, 6	12.81131, 12.86565, 5.53406	7.8918, 7.92422, 4.40503	9.88323, 1	3	mpz	326
GL173357.1	162937	163336	163229, 163291, 163296	14, 14, 9	16.23025, 14.45897, 11.64359	9.67496, 8.66788, 7.57973	13.14538,	3	sdhc	163
GL173357.1	202321	202916	202627, 202678, 202645	4, 8, 4	3.74195, 7.07159, 4.38152	3.32091, 5.20073, 3.65448	1.6222, 4.6	3	NA	0
GL173357.1	232363	233196	232616, 232632, 233051	19, 19, 15	15.14105, 11.84639, 12.78435	7.79671, 5.92263, 7.42082	12.1041, 9	3	NA	14918
GL173357.1	267688	268570	268188, 268077, 268006	19, 25, 12	15.69097, 17.60286, 8.79345	8.12352, 7.58461, 5.62592	12.62685,	3	NA	44496
GL173357.1	269546	270493	270200, 269694, 269776	11, 21, 13	7.96355, 13.9189, 10.78246	5.29991, 6.59812, 6.65226	5.35378, 1	3	NA	42573
GL173357.1	318582	319232	318849, 318664, 318803	14, 10, 7	14.23954, 8.36423, 8.69489	8.53829, 5.72892, 6.06379	11.24094,	3	NA	0
GL173357.1	489831	490148	489908, 489969, 490093	7, 8, 6	6.33443, 5.78452, 4.80295	4.83795, 4.39481, 3.96416	3.86259, 3	3	NA	29131
GL173357.1	494829	496130	495766, 495127, 495768	24, 38, 31	27.39936, 37.76342, 36.85188	13.56637, 14.28878, 16.59905	23.95304,	3	NA	34129
GL173357.1	511594	512629	512428, 512475, 512477	15, 22, 15	15.64184, 21.70761, 14.87928	9.15231, 10.82585, 8.69822	12.57992,	3	NA	50894
GL173359.1	179321	181116	180290, 180312, 179581	8, 9, 9	6.78332, 8.21099, 9.46739	5.02459, 5.77858, 6.50386	4.27811, 5	3	cntnap4	93945
GL173359.1	186743	189240	187355, 187013, 187621, 188937	10, 12, 4, 12	8.20198, 8.68993, 3.43992, 12.88563	5.62778, 5.56143, 3.14645, 8.10565	5.57541, 6	3	cntnap4	85821
GL173360.1	53410	53996	53646, 53661, 53653	9, 14, 11	9.30591, 11.85481, 12.48556	6.41439, 7.08876, 7.99611	6.59443, 9	3	zfand3	191453
GL173360.1	155466	156111	156022, 155748, 155986	6, 8, 4	5.96715, 6.24338, 4.50795	4.64927, 4.6873, 3.71446	3.52158, 3	3	zfand3	89338
GL173360.1	179821	180203	180166, 180144, 179940	11, 12, 3	12.17114, 10.63474, 3.37781	7.82679, 6.77054, 2.98791	9.28263, 7	3	zfand3	65246
GL173360.1	222689	223080	222864, 222978, 222800	5, 6, 6	4.77263, 4.93312, 7.29403	3.9578, 4.04501, 5.30581	2.51418, 2	3	zfand3	22369
GL173360.1	256073	256561	256527, 256382, 256256	8, 13, 5	7.77807, 10.55511, 4.25472	5.618, 6.51132, 3.65711	5.17956, 7	3	zfand3	0
GL173360.1	283597	284084	283735, 283848, 283903	28, 51, 29	30.68278, 53.93216, 30.93982	14.09279, 17.7756, 13.86669	27.1602, 5	3	zfand3	0
GL173360.1	287810	288181	287937, 287955, 287872	5, 7, 13	3.35103, 3.40505, 11.59311	3.08326, 2.95874, 7.1528	1.42597, 1	3	zfand3	0
GL173360.1	310530	311030	310573, 310769, 310592	13, 17, 11	12.49994, 14.56175, 11.03484	7.70504, 7.99171, 7.18523	9.58678, 1	3	zfand3	0
GL173360.1	362710	363608	363056, 363089, 363115, 363333	20, 12, 9, 4	21.44017, 10.93692, 10.14484, 3.91395	11.29037, 6.95571, 6.86555, 3.41551	18.16397,	3	btbd9	0
GL173360.1	371166	371946	371579, 371439, 371589	15, 17, 6	14.15244, 15.35073, 6.08001	8.25853, 8.4724, 4.71063	11.15777,	3	btbd9	0
GL173360.1	380574	380988	380818, 380845, 380805	29, 36, 20	31.63492, 38.07464, 25.00335	14.28035, 15.27209, 13.33871	28.09085,	3	btbd9	0
GL173360.1	400906	401622	401264, 401282, 401094	18, 26, 11	17.31602, 22.01696, 10.66921	9.39018, 9.71038, 6.9701	14.18471,	3	btbd9	1876
GL173360.1	483472	484191	483905, 483600, 484026	19, 26, 8	16.65303, 21.70642, 8.04677	8.701, 9.53509, 5.77057	13.55018,	3	glo1	74633
GL173360.1	504334	504696	504663, 504641, 504633	25, 34, 20	25.73051, 32.9999, 24.24089	12.22615, 13.15166, 12.91583	22.32921,	3	glo1	95495
GL173361.1	67450	68488	67714, 67739, 67691	9, 21, 10	7.75911, 20.39593, 10.59656	5.5036, 10.35516, 7.05292	5.16246, 1	3	klhl35	0
GL173361.1	109809	110432	110413, 110403, 110364	14, 33, 11	14.10012, 36.8901, 12.34038	8.4555, 15.8132, 7.91841	11.10747,	3	gpr25	8479
GL173361.1	141786	142624	141954, 142037, 142182	13, 11, 9	10.62846, 7.97598, 9.19414	6.5568, 5.30773, 6.35176	7.83043, 5	3	map6	0
GL173361.1	141891	142624	142337, 142037, 142182	9, 11, 9	6.28237, 7.97598, 9.19414	4.57292, 5.30773, 6.35176	3.81457, 5	3	map6	0
GL173361.1	164704	165541	165335, 165334, 165331	46, 55, 40	56.85934, 63.09522, 52.39471	21.7493, 20.97552, 21.9379	52.91185,	3	mogat2.2	0
GL173361.1	221765	223201	222157, 221803, 222822, 222363	8, 13, 11, 10	7.38183, 10.23256, 8.13814, 11.45478	5.38666, 6.31126, 5.40965, 7.51412	4.80856, 7	3	dgat2	5319
GL173361.1	260615	260999	260938, 260900, 260938	20, 16, 15	21.18126, 14.52284, 15.95038	11.13591, 8.2328, 9.33339	17.91394,	3	uvrag	0
GL173361.1	310268	310587	310535, 310538, 310583	37, 44, 55	40.43646, 45.55924, 72.9047	16.22831, 16.18397, 26.60902	36.72728,	3	uvrag	15744
GL173361.1	352135	352899	352182, 352144, 352180	37, 44, 24	36.81489, 46.1997, 25.66978	14.14962, 16.53868, 12.53702	33.16672,	3	uvrag	57611
GL173361.1	355768	356327	356323, 356280, 356267	16, 12, 9	12.63122, 7.77827, 7.15656	7.07806, 4.99688, 5.12844	9.71235, 5	3	uvrag	61244
GL173361.1	463732	464135	464001, 463970, 463814	15, 15, 6	16.03559, 15.19102, 5.99481	9.3831, 8.88488, 4.6644	12.95858,	3	uvrag	169208
GL173361.1	469844	470213	470166, 470140, 470162	11, 14, 11	12.43693, 14.45897, 14.75772	7.97018, 8.66788, 9.09568	9.52692, 1	3	uvrag	175320
GL173362.1	10749	12739	12069, 11036, 11472	11, 23, 10	10.3251, 22.17331, 11.24131	6.7644, 10.77791, 7.40241	7.54325, 1	3	lsamp	82857
GL173362.1	21051	21686	21098, 21095, 21108	16, 15, 14	16.45267, 13.26621, 18.28596	9.40062, 7.71668, 10.7324	13.35812,	3	lsamp	73910
GL173362.1	39473	40091	39704, 39844, 39582	5, 6, 3	4.40263, 4.25354, 3.24307	3.74533, 3.61361, 2.92359	2.21004, 2	3	lsamp	55505
GL173362.1	49304	49701	49622, 49649, 49670	6, 9, 7	4.99728, 8.15232, 7.78342	4.08451, 5.74324, 5.63488	2.67584, 5	3	lsamp	45895
GL173362.1	106851	107355	107063, 107324, 107233	4, 10, 7	3.48339, 8.12616, 7.2501	3.1722, 5.58036, 5.35703	1.52925, 5	3	lsamp	0
GL173362.1	120196	120679	120669, 120677, 120677	9, 19, 7	7.39636, 16.41246, 6.6707	5.27874, 8.55605, 5.03432	4.82212, 1	3	lsamp	0
GL173362.1	141463	142730	142098, 142333, 142072	9, 11, 11	8.20841, 8.30667, 10.66921	5.77703, 5.51556, 6.9701	5.58112, 5	3	lsamp	0
GL173362.1	165696	166436	166065, 166393, 166261	11, 9, 6	10.78278, 7.75488, 6.93817	7.03733, 5.501, 5.14396	7.97565, 5	3	lsamp	0
GL173362.1	196873	197615	197564, 197565, 197587	37, 59, 39	43.3508, 68.76186, 47.34253	17.94155, 22.19053, 19.41402	39.59576,	3	lsamp	0
GL173362.1	203440	203840	203478, 203576, 203517	21, 18, 11	20.95297, 16.69883, 12.06254	10.69108, 9.01539, 7.76744	17.69275,	3	lsamp	0
GL173362.1	210039	210568	210514, 210538, 210538	23, 20, 8	22.72043, 18.3522, 9.31951	11.10603, 9.43067, 6.43944	19.40507,	3	lsamp	0
GL173362.1	244520	244924	244547, 244550, 244573	17, 34, 24	16.6291, 32.70562, 27.07887	9.24979, 12.98539, 13.37677	13.52753,	3	lsamp	0
GL173362.1	291547	292002	291990, 291947, 291938	41, 54, 37	47.52853, 59.21452, 46.0937	18.62042, 19.30716, 19.56193	43.71125,	3	lsamp	0
GL173362.1	302424	302663	302440, 302451, 302453	22, 23, 7	23.80731, 21.44504, 7.01416	12.08624, 10.34265, 5.22813	20.45995,	3	lsamp	0
GL173362.1	333576	333980	333660, 333627, 333577	8, 6, 4	5.83807, 3.22653, 4.42274	4.42916, 2.93053, 3.67426	3.43803, 1	3	lsamp	0
GL173362.1	336967	337911	337164, 337014, 337767, 337761	10, 13, 14, 5	8.20198, 10.00327, 11.41012, 5.59755	5.62778, 6.16911, 6.81441, 4.38538	5.57541, 7	3	lsamp	0
GL173362.1	405373	405786	405759, 405755, 405760	19, 21, 19	23.41291, 22.6637, 21.43283	12.68879, 11.7178, 11.56736	20.07675,	3	lsamp	0
GL173362.1	417048	417660	417212, 417114, 417089	17, 18, 6	17.88146, 17.72647, 6.2131	10.00115, 9.63881, 4.78171	14.72916,	3	lsamp	0
GL173362.1	423922	424603	424004, 424025, 424005	13, 13, 8	11.26158, 11.03409, 8.62637	6.94861, 6.80804, 6.08668	8.41898, 8	3	lsamp	0
GL173362.1	458602	459128	458900, 458926, 459025	15, 14, 8	14.33586, 11.94813, 9.46129	8.36999, 7.1463, 6.50801	11.33286,	3	lsamp	9627
GL173362.1	491434	492088	491505, 491662, 491510	9, 7, 9	7.97705, 4.8451, 9.30116	5.637, 3.90649, 6.41174	5.36577, 2	3	gap43	0
GL173362.1	501114	501484	501423, 501434, 501430	21, 25, 14	22.14207, 27.7306, 17.72406	11.40611, 13.42198, 10.45693	18.84387,	3	gap43	1932
GL173363.1	61169	62046	61507, 61512, 61496	21, 20, 7	23.81985, 20.53147, 7.50542	12.40141, 10.74631, 5.49244	20.47156,	3	MGC147188	80528
GL173363.1	69764	70573	70545, 70201, 70236	8, 10, 12	8.42257, 8.70492, 15.80834	5.97763, 5.93975, 9.60504	5.76336, 6	3	MGC147188	72001
GL173363.1	80223	81093	80887, 80653, 80861	12, 24, 9	12.18267, 25.91323, 11.64359	7.70145, 12.68264, 7.57973	9.29304, 2	3	MGC147188	61481
GL173363.1	219116	219695	219666, 219668, 219648	42, 51, 36	50.48615, 57.49079, 45.50011	19.8983, 19.72909, 19.64283	46.62676,	3	MGC147188	70703
GL173363.1	367306	367993	367497, 367701, 367904	4, 5, 4	3.63646, 3.94561, 4.42274	3.26116, 3.46715, 3.67426	1.6222, 1.8	3	hpn	34885
GL173363.1	475085	475427	475158, 475100, 475100	13, 11, 9	13.00701, 10.60895, 9.76105	8.00827, 6.9343, 6.66343	10.0703, 7	3	lgi4	16973
GL173364.1	99	878	522, 687, 415	14, 19, 10	16.43009, 19.96513, 12.33934	9.78349, 10.7011, 7.95424	13.33671,	3	NA	44868
GL173364.1	40565	41052	40814, 40964, 40747	6, 14, 3	4.65447, 14.09002, 3.27564	3.87084, 8.44949, 2.93941	2.42652, 1	3	NA	4694
GL173364.1	97557	99070	97908, 97952, 98137	10, 16, 23	9.03421, 13.59227, 24.34301	6.14116, 7.66367, 12.08015	6.33915, 1	3	NA	10069
GL173364.1	110049	110276	110081, 110093, 110079	9, 23, 4	9.63662, 25.71782, 4.67325	6.59633, 12.90012, 3.78987	6.90556, 2	3	NA	22561
GL173364.1	122729	123353	122757, 122761, 122778	109, 170, 93	135.61601, 211.27402, 122.39755	31.48276, 35.50194, 33.34724	130.96988	3	NA	35241
GL173364.1	161461	161984	161526, 161529, 161604	8, 19, 10	8.42257, 17.89367, 10.47831	5.97763, 9.45168, 6.98694	5.76336, 1	3	NA	73973
GL173364.1	206427	206951	206461, 206745, 206467	8, 5, 9	8.42257, 3.94561, 11.64359	5.97763, 3.46715, 7.57973	5.76336, 1	3	setbp1	96381
GL173364.1	223068	223555	223111, 223085, 223143	6, 14, 8	5.96715, 13.26509, 9.46129	4.64927, 7.95332, 6.50801	3.52158, 1	3	setbp1	79777
GL173364.1	247051	247697	247412, 247439, 247466	8, 12, 6	6.52004, 9.90946, 7.25145	4.86121, 6.32175, 5.287	4.03345, 7	3	setbp1	55635
GL173364.1	296292	296610	296545, 296529, 296398	6, 9, 5	4.9641, 6.86591, 5.50079	4.06411, 4.94483, 4.33868	2.64509, 4	3	setbp1	6722
GL173364.1	369822	370468	370161, 369853, 369853	15, 21, 19	14.09249, 18.32622, 21.18265	8.22203, 9.11275, 11.42143	11.1007, 1	3	setbp1	0
GL173364.1	493541	494143	493750, 493741, 493746	25, 50, 27	25.48561, 48.71987, 30.06009	12.0796, 15.45358, 14.09351	22.0906, 4	3	setbp1	62555
GL173364.1	498862	499916	499022, 499001, 498984	8, 18, 13	6.7444, 13.09371, 12.98465	5.00058, 6.85423, 7.995	4.24113, 1	3	setbp1	67876
GL173364.1	511837	512310	512215, 512227, 512193	49, 57, 33	60.66377, 64.71645, 44.50592	22.51048, 20.92344, 20.26878	56.66896,	3	setbp1	80851
GL173365.1	22702	24525	23158, 23121, 23158	10, 12, 13	8.32267, 8.78862, 12.76267	5.70305, 5.62291, 7.86275	5.6878, 6.2	3	NA	59
GL173365.1	101930	102669	102243, 102307, 102243	19, 14, 18	21.11935, 14.45897, 23.23937	11.38438, 8.66788, 12.78599	17.85404,	3	NA	66385
GL173365.1	156103	156726	156272, 156171, 156139	16, 13, 8	15.74146, 9.89237, 9.8421	8.97386, 6.1004, 6.68602	12.67506,	3	NA	12328
GL173365.1	167288	167938	167701, 167483, 167704	7, 14, 10	5.82861, 13.38467, 11.997	4.53157, 8.02582, 7.78835	3.42929, 1	3	NA	1116
GL173365.1	446310	446935	446805, 446657, 446550	6, 15, 4	5.63898, 14.21432, 4.67325	4.46544, 8.29617, 3.78987	3.25772, 1	3	NA	10709
GL173366.1	141	1416	681, 1179, 1078	22, 21, 9	25.18667, 20.81922, 9.8849	12.90256, 10.61044, 6.72947	21.80058,	3	trpc2	1303
GL173366.1	219831	221315	220961, 220100, 219855, 221068	10, 9, 4, 5	9.33595, 7.59096, 4.22496, 5.27517	6.32332, 5.39978, 3.57747, 4.22616	6.62299, 5	3	NA	0
GL173366.1	255374	256075	255958, 256067, 255754	5, 9, 6	4.82177, 8.21099, 7.05929	3.98509, 5.77858, 5.20024	2.51418, 5	3	NA	18050
GL173366.1	420341	420973	420478, 420616, 420741	7, 7, 7	5.24783, 4.45047, 7.01416	4.16625, 3.64847, 5.22813	2.90454, 2	3	NA	6145
GL173366.1	421891	422384	422196, 422132, 422223	15, 20, 9	14.8531, 18.78201, 9.08997	8.68248, 9.69018, 6.2929	11.82783,	3	NA	7695
GL173366.1	428681	429899	429690, 429815, 429806	5, 9, 7	4.82177, 8.21099, 8.35502	3.98509, 5.77858, 5.9108	2.51418, 5	3	NA	14485
GL173366.1	449643	450122	449873, 449717, 449729	7, 7, 8	7.1696, 5.97725, 9.92329	5.31345, 4.62287, 6.7228	4.61046, 3	3	NA	35447
GL173366.1	464076	464669	464358, 464584, 464350	10, 8, 6	8.89113, 7.07159, 6.4014	6.05396, 5.20073, 4.8799	6.20456, 4	3	NA	49880
GL173366.1	469539	470004	469658, 469657, 469836	15, 18, 10	17.09296, 19.5052, 13.18184	9.98755, 10.70269, 8.33771	13.96978,	3	NA	55343
GL173366.1	475736	476988	476584, 476104, 476204	21, 25, 23	20.73342, 23.02855, 24.34301	10.5587, 10.61971, 12.08015	17.48034,	3	NA	61540
GL173366.1	493616	494973	494259, 494328, 494409	15, 26, 11	13.74446, 22.17581, 9.14441	8.00965, 9.80046, 6.04007	10.77314,	3	NA	79420
GL173366.1	495354	495932	495787, 495505, 495778	11, 13, 11	9.26086, 8.49752, 9.2906	6.1125, 5.24388, 6.13097	6.5517, 5.9	3	NA	81158
GL173367.1	44746	45356	44887, 44974, 44833	6, 9, 6	5.47306, 6.7053, 6.08001	4.36955, 4.84282, 4.71063	3.10811, 4	3	ubtd2	206886
GL173367.1	52654	53445	53251, 53252, 52985	19, 20, 17	19.33502, 19.30258, 19.81591	10.32316, 10.00482, 11.12108	16.12917,	3	ubtd2	198797
GL173368.1	7587	8494	7943, 7792, 7800	25, 39, 27	14.11499, 20.96817, 19.40508	5.86726, 6.27385, 8.00851	11.12136,	3	slc25a36	7583
GL173368.1	9027	9975	9695, 9803, 9780	21, 38, 15	13.56124, 25.65483, 10.89199	6.40694, 8.27251, 6.26534	10.59862,	3	slc25a36	6102
GL173368.1	51359	51835	51809, 51809, 51762	24, 34, 17	25.45469, 38.05053, 19.46435	12.40824, 16.08706, 10.92232	22.06058,	3	slc25a36	18155
GL173368.1	301272	301887	301560, 301568, 301568	10, 10, 9	7.93364, 6.45533, 9.82249	5.45963, 4.52444, 6.69629	5.325, 4.09	3	clstn2	52550
GL173369.1	251705	252095	251816, 251771, 251853	12, 12, 6	11.73328, 10.03748, 6.168	7.43625, 6.40131, 4.75778	8.86615, 7	3	NA	3471
GL173369.1	441138	442578	441858, 442087, 442515	25, 37, 12	21.41422, 31.05149, 10.59056	9.68132, 11.05771, 6.74336	18.1389, 2	3	NA	68203
GL173370.1	449	1279	781, 769, 774	90, 111, 45	96.48127, 120.39129, 48.96733	22.07789, 23.88646, 17.65646	92.1212, 1	3	muc3a	0
GL173370.1	53126	53579	53554, 53537, 53514	15, 17, 10	15.87516, 15.46309, 11.17239	9.2894, 8.5409, 7.36591	12.80377,	3	serpine1	1409
GL173370.1	110120	110880	110487, 110789, 110392	13, 9, 5	12.32133, 7.79702, 5.64747	7.59717, 5.5269, 4.40911	9.42482, 5	3	XB-GENE-5892958	30884
GL173370.1	184801	185590	185279, 185312, 185399	5, 6, 3	4.82177, 4.93312, 3.47371	3.98509, 4.04501, 3.03189	2.51418, 2	3	NA	31167
GL173370.1	205993	206409	206019, 206044, 206058	23, 33, 20	24.06243, 36.8901, 27.34127	11.91191, 15.8132, 14.56493	20.70741,	3	NA	52359
GL173370.1	268343	268881	268574, 268693, 268710	14, 9, 5	13.70063, 7.88274, 4.91762	8.21651, 5.57944, 4.03768	10.73143,	3	syt4	19612
GL173370.1	292342	293431	292883, 292614, 293001	12, 8, 6	13.8483, 7.07159, 7.12188	8.63436, 5.20073, 5.22884	10.86694,	3	syt4	0
GL173370.1	342544	343228	342577, 342578, 342563	54, 77, 40	65.77729, 89.44645, 47.99237	22.98191, 24.45559, 19.34622	61.72517,	3	syt4	34139
GL173370.1	480160	480498	480466, 480371, 480471	12, 16, 7	13.8483, 17.16856, 8.69489	8.63436, 9.82359, 6.06379	10.86694,	3	syt4	171755
GL173370.1	498739	499507	499264, 499231, 499259	23, 28, 8	27.48315, 30.40849, 10.1465	13.9341, 13.92926, 6.82176	24.03497,	3	syt4	190334
GL173371.1	28145	29417	28724, 28946, 29085	34, 36, 38	29.4009, 30.12315, 40.54008	11.17219, 10.90185, 15.86653	25.90795,	3	aicda	1235
GL173371.1	86566	87299	86685, 86725, 86818	9, 6, 6	8.30507, 4.93312, 6.45058	5.83501, 4.04501, 4.90508	5.67102, 2	3	NA	2199
GL173371.1	93290	93777	93509, 93463, 93370	7, 9, 9	7.1696, 8.21099, 11.64359	5.31345, 5.77858, 7.57973	4.61046, 5	3	NA	8923
GL173371.1	299374	300371	300229, 300145, 300157	18, 36, 14	19.43049, 37.00277, 14.47197	10.65863, 14.65375, 8.67552	16.22032,	3	rbp5	0
GL173371.1	336390	337014	336558, 336918, 336918	7, 18, 9	5.42862, 16.01233, 9.24728	4.2813, 8.5983, 6.38161	3.06982, 1	3	clstn3	0
GL173371.1	370711	371670	371471, 371387, 370845	9, 7, 7	9.06007, 5.97725, 7.96278	6.27591, 4.62287, 5.72394	6.36394, 3	3	ptpn6	0
GL173371.1	452873	453219	453145, 453127, 453128	10, 8, 6	10.26482, 6.11443, 6.45058	6.86644, 4.60556, 4.90508	7.48624, 3	3	atn1	14682
GL173371.1	479335	480324	479878, 479379, 480087	8, 11, 6	6.37855, 8.62761, 5.95321	4.77253, 5.71704, 4.64162	3.90357, 6	3	lrrc23	0
GL173371.1	519464	520016	519657, 519594, 519788	19, 25, 15	19.25925, 23.67755, 16.75372	10.27754, 11.00257, 9.79627	16.05534,	3	usp5	1186
GL173372.1	217180	218115	217640, 217469, 217838	7, 10, 4	6.37715, 8.16488, 4.67325	4.86324, 5.60458, 3.78987	3.90257, 5	3	A2ML1	0
GL173372.1	258406	258704	258636, 258641, 258650	15, 20, 9	14.98937, 19.37003, 10.35155	8.76428, 10.0456, 6.97128	11.95813,	3	A2ML1	29180
GL173372.1	279936	280848	280128, 280148, 280165, 280757	19, 32, 13, 5	20.56775, 32.08022, 14.85247, 4.51488	11.05955, 13.3857, 9.06201, 3.81106	17.31957,	3	pzp	7710
GL173372.1	485417	486560	486019, 485933, 485946	15, 19, 12	13.30906, 15.35066, 11.76526	7.74297, 7.92094, 7.45528	10.35786,	3	NA	9927
GL173372.1	489074	489335	489246, 489283, 489179	17, 11, 6	16.4953, 8.00793, 5.60548	9.16875, 5.32781, 4.44624	13.39926,	3	NA	13584
GL173373.1	28	1470	980, 961, 1145	32, 41, 35	29.89085, 35.34546, 29.77263	12.13243, 11.92603, 11.04076	26.38588,	3	NA	16379
GL173373.1	3217	3908	3657, 3658, 3654	22, 28, 42	17.39157, 19.49408, 37.3919	8.27284, 7.80062, 12.61768	14.25679,	3	NA	13941
GL173373.1	62117	62945	62798, 62636, 62247	4, 14, 4	3.6687, 10.39738, 4.67325	3.27956, 6.19139, 3.78987	1.6222, 7.7	3	NA	3734
GL173373.1	91817	92415	91943, 91954, 91949	17, 22, 19	18.82666, 21.56447, 24	10.55599, 10.73971, 13.00773	15.63723,	3	NA	0
GL173373.1	249579	250653	249753, 249730, 250546	6, 6, 7	5.77068, 4.73925, 7.84208	4.54016, 3.92426, 5.66426	3.37745, 2	3	XB-GENE-5899798	0
GL173373.1	392005	392684	392361, 392439, 392543	13, 22, 18	13.79066, 19.71017, 20.07449	8.46641, 9.6289, 11.036	10.8171, 1	3	NA	477
GL173373.1	450140	450866	450363, 450315, 450413	8, 8, 7	6.78332, 7.07159, 7.34973	5.02459, 5.20073, 5.41038	4.27811, 4	3	NA	4325
GL173374.1	7675	8111	8047, 8063, 8013	6, 7, 4	5.90927, 5.97725, 4.67325	4.61743, 4.62287, 3.78987	3.50164, 3	3	stag3	48
GL173374.1	110064	110834	110582, 110582, 110581	9, 13, 7	7.59348, 12.03475, 7.40077	5.40134, 7.4231, 5.43746	5.0069, 9.3	3	NA	249
GL173374.1	117920	118104	117923, 117965, 117950	31, 53, 22	34.23788, 59.14236, 27.35307	15.04757, 19.7229, 14.14591	30.64031,	3	NA	8105
GL173374.1	166087	166469	166234, 166258, 166278	33, 33, 22	38.20358, 40.35691, 26.07069	16.59192, 17.86611, 13.41675	34.53099,	3	gal3st4	12423
GL173374.1	186273	187349	186618, 186885, 186992	10, 6, 4	8.57531, 4.25354, 4.42274	5.85982, 3.61361, 3.67426	5.90712, 2	3	gal3st4	0
GL173374.1	339055	339961	339253, 339776, 339719	8, 7, 8	7.47668, 5.68499, 8.09568	5.44269, 4.44244, 5.79794	4.89713, 3	3	NA	0
GL173374.1	406666	407404	407132, 407103, 406778	17, 21, 9	16.23509, 18.71315, 7.51189	9.01086, 9.34355, 5.35071	13.14931,	3	taf6	0
GL173374.1	432266	432993	432933, 432656, 432562	9, 9, 7	8.88537, 8.21099, 8.42533	6.17591, 5.77858, 5.94313	6.19961, 5	3	NA	0
GL173374.1	439999	441124	440860, 440865, 440356	15, 14, 10	13.80112, 12.70158, 9.53891	8.04428, 7.60958, 6.44439	10.82722,	3	NA	1846
GL173374.1	452082	452742	452633, 452176, 452597	6, 10, 4	5.16999, 7.14103, 3.59344	4.18963, 4.95885, 3.23643	2.83325, 4	3	mogat2.1	13717
GL173375.1	46977	47499	47432, 47275, 47469	10, 14, 6	7.44561, 10.95872, 4.85645	5.15177, 6.53614, 3.9975	4.86925, 8	3	stmn4	6514
GL173375.1	69530	70261	69692, 69564, 69623	10, 19, 16	10.01299, 19.48976, 20.88407	6.7221, 10.41622, 11.85118	7.24763, 1	3	stmn4	29067
GL173375.1	101201	101590	101317, 101342, 101322	12, 15, 7	13.8483, 15.80067, 8.69489	8.63436, 9.24574, 6.06379	10.86694,	3	ptk2b	9758
GL173375.1	176619	177500	177433, 177383, 177367	13, 25, 9	11.50747, 21.40647, 7.79368	7.10015, 9.67687, 5.52485	8.65224, 1	3	chrna2	17518
GL173375.1	178438	179306	178683, 178929, 178817	8, 12, 6	6.11283, 7.54492, 4.75057	4.60454, 4.85383, 3.93137	3.65697, 5	3	chrna2	19337
GL173375.1	185596	186268	186216, 186165, 186247	24, 17, 14	23.67742, 15.35073, 16.2088	11.34307, 8.4724, 9.66324	20.33353,	3	chrna2	26495
GL173375.1	190996	191477	191251, 191385, 191326	21, 24, 16	19.4592, 23.62809, 18.61066	9.79088, 11.31356, 10.64858	16.24853,	3	chrna2	31895
GL173375.1	201466	202580	201924, 202360, 202024	6, 12, 8	5.96715, 11.86108, 10.1465	4.64927, 7.51216, 6.82176	3.52158, 9	3	chrna2	42365
GL173375.1	313970	314355	313973, 313973, 314013	11, 22, 9	11.35776, 20.88093, 8.69919	7.37194, 10.32883, 6.06795	8.51017, 1	3	ephx2	6949
GL173375.1	387541	388027	387613, 387566, 387642	5, 9, 5	3.78532, 7.10358, 5.59755	3.36588, 5.09508, 4.38538	1.66163, 4	3	ephx2	0
GL173376.1	76400	77262	76775, 76680, 76850	7, 7, 5	6.46447, 5.8586, 5.14967	4.91463, 4.55007, 4.16141	3.98312, 3	3	xtr-mir-92a-1	7892
GL173377.1	160017	161346	160637, 160942, 160321, 161150	8, 9, 3, 3	7.99469, 6.89892, 3.47371, 3.44998	5.74128, 4.96575, 3.03189, 3.02115	5.38155, 4	3	galr1	46030
GL173377.1	160954	161977	161273, 161741, 161150	6, 9, 3	5.55471, 6.26311, 3.44998	4.41698, 4.56059, 3.02115	3.18192, 3	3	galr1	45399

GL173379.1	36896	37758	37164, 37085, 37250	11, 17, 13	11.56615, 15.51996, 14.77384	7.49066, 8.57556, 9.01902	8.70729, 1	3	NA	93909
GL173381.1	9793	10357	10070, 10300, 10229	14, 22, 19	16.23025, 25.8245, 25.66388	9.67496, 13.2744, 13.87136	13.14538,	3	NA	11693
GL173381.1	39691	40863	40607, 40599, 40603	11, 14, 12	11.49566, 13.03306, 13.59027	7.45067, 7.81216, 8.49571	8.64106, 1	3	NA	0
GL173381.1	155784	156049	156010, 156031, 155988	6, 21, 6	5.43318, 21.57397, 6.76586	4.34622, 11.06508, 5.06179	3.07284, 1	3	NA	4625
GL173381.1	215543	216024	215920, 215815, 215647	11, 10, 5	9.26086, 7.1111, 4.64121	6.1125, 4.93988, 3.88372	6.5517, 4.6	3	NA	23657
GL173381.1	362110	362610	362453, 362554, 362256	10, 13, 4	11.06011, 12.31712, 4.67325	7.306, 7.59462, 3.78987	8.22712, 9	3	or13c8	4050
GL173381.1	367863	368442	368142, 368202, 368078	5, 8, 7	4.61714, 6.81727, 8.08824	3.86998, 5.04548, 5.7849	2.39372, 4	3	or13c8	9803
GL173381.1	416322	416884	416719, 416646, 416744	8, 23, 6	7.77807, 22.74071, 7.29403	5.618, 11.1182, 5.30581	5.17956, 1	3	NA	12579
GL173383.1	140870	141567	141125, 141429, 140967, 141518	14, 23, 7, 5	13.44866, 22.59564, 8.08824, 5.31857	8.06455, 11.03113, 5.7849, 4.24819	10.49073,	3	gabrb2	164989
GL173383.1	373182	373515	373388, 373437, 373473	7, 9, 5	6.2923, 8.21099, 5.59755	4.81292, 5.77858, 4.38538	3.8235, 5.7	3	gabrb2	0
GL173384.1	72920	73875	73377, 73692, 73813	8, 20, 7	8.22568, 22.54677, 8.49722	5.8701, 11.94392, 5.97582	5.59681, 1	3	NA	4246
GL173384.1	105638	106181	106134, 106127, 106032	19, 27, 16	20.74745, 26.58221, 19.65507	11.16575, 12.02229, 11.21774	17.49384,	3	NA	0
GL173384.1	114409	115510	115489, 115143, 114697	5, 9, 8	4.43696, 8.21099, 8.24665	3.76555, 5.77858, 5.88165	2.24017, 5	3	NA	0
GL173384.1	122014	122551	122027, 122043, 122045	64, 99, 74	78.93889, 124.71275, 99.73602	25.3393, 31.26979, 31.65036	74.75262,	3	NA	0
GL173384.1	149192	149822	149341, 149222, 149221	7, 7, 4	6.09019, 5.42726, 4.67325	4.69155, 4.28044, 3.78987	3.63504, 3	3	NA	0
GL173384.1	158738	159194	158813, 158851, 159047	8, 18, 7	7.47668, 16.17802, 6.6707	5.44269, 8.69892, 5.03432	4.89713, 1	3	NA	0
GL173384.1	193909	194310	194153, 194026, 194135	6, 16, 5	5.31708, 12.25152, 4.57714	4.2777, 6.84834, 3.84705	2.96727, 9	3	NA	7970
GL173384.1	300203	300739	300713, 300708, 300677	24, 26, 10	24.13149, 23.42183, 10.59656	11.615, 10.51587, 7.05292	20.77422,	3	ripk4	2412
GL173384.1	330541	331598	331458, 331228, 331256	6, 9, 7	5.86227, 8.19918, 7.72582	4.59138, 5.77148, 5.6058	3.46055, 5	3	prdm15	4237
GL173384.1	341961	342558	342331, 342449, 342380	7, 10, 8	7.1696, 9.39128, 10.1465	5.31345, 6.35644, 6.82176	4.61046, 6	3	C2CD2	0
GL173384.1	350623	350969	350826, 350826, 350826	6, 11, 8	5.96715, 10.60895, 10.1465	4.64927, 6.9343, 6.82176	3.52158, 7	3	C2CD2	0
GL173385.1	68047	68768	68428, 68729, 68428	17, 18, 10	17.64386, 15.28549, 11.38247	9.85981, 8.15798, 7.47651	14.49984,	3	NA	5152
GL173385.1	81660	82186	82070, 81915, 82088	14, 10, 5	16.23025, 9.39128, 5.9506	9.67496, 6.35644, 4.54784	13.14538,	3	NA	0
GL173385.1	216459	216900	216691, 216680, 216693	63, 81, 38	72.43607, 95.57406, 47.202	22.26256, 25.64123, 19.77684	68.31667,	3	NA	17546
GL173385.1	226804	227529	226856, 226897, 226846	18, 33, 15	22.92104, 41.78416, 21.28424	12.61945, 18.7034, 12.08458	19.59912,	3	NA	27891
GL173385.1	368089	369155	368441, 368271, 368343	8, 13, 8	7.19971, 11.66277, 9.46129	5.27799, 7.19557, 6.50801	4.63758, 8	3	NA	44652
GL173385.1	368089	369261	368827, 369159, 368343	9, 9, 8	8.25643, 6.6123, 9.46129	5.80588, 4.78362, 6.50801	5.62516, 4	3	NA	44652
GL173385.1	467036	467514	467180, 467191, 467320	7, 10, 7	6.89193, 9.39128, 8.08824	5.15998, 6.35644, 5.7849	4.37772, 6	3	NA	143599
GL173386.1	46050	47170	46658, 46490, 46693	16, 14, 11	16.60441, 12.64814, 13.55304	9.49088, 7.57684, 8.54092	13.50404,	3	NA	1684
GL173386.1	234122	234575	234323, 234349, 234365	10, 12, 9	9.28413, 10.78353, 8.88966	6.29221, 6.86188, 6.17838	6.574, 8.12	3	cul3	0
GL173386.1	379128	379417	379187, 379140, 379251	10, 13, 5	8.61898, 10.72425, 4.99237	5.88679, 6.61618, 4.0781	5.94907, 8	3	pxylp1	84624
GL173386.1	472096	472622	472156, 472260, 472393	10, 10, 4	11.06011, 9.39128, 4.67325	7.306, 6.35644, 3.78987	8.22712, 6	3	pxylp1	177592
GL173387.1	5765	6394	5948, 6112, 5866	4, 7, 7	3.74195, 5.05277, 7.61369	3.32091, 4.04098, 5.54854	1.6222, 2.8	3	RAB11FIP4	2842
GL173387.1	18151	19393	19069, 19298, 19064	11, 11, 9	9.7619, 8.04011, 8.79325	6.42195, 5.34803, 6.12267	7.0115, 5.5	3	RAB11FIP4	0
GL173387.1	119419	120119	119525, 119687, 119689	5, 13, 3	4.30277, 10.51368, 3.27564	3.68597, 6.48562, 2.93941	2.12436, 7	3	RAB11FIP4	65770
GL173387.1	295720	297060	296125, 296576, 296323	23, 22, 17	22.94393, 21.28482, 20.98465	11.24022, 10.5715, 11.76329	19.62118,	3	NA	42824
GL173387.1	421509	423123	421720, 422574, 422754	5, 10, 7	4.82177, 9.12511, 7.29952	3.98509, 6.19629, 5.38357	2.51418, 6	3	NA	18176
GL173387.1	421768	423123	422233, 422574, 422754	9, 10, 7	9.72082, 9.12511, 7.29952	6.64182, 6.19629, 5.38357	6.97155, 6	3	NA	18176
GL173389.1	32043	32386	32192, 32295, 32316	10, 12, 6	7.44561, 8.40592, 5.1729	5.15177, 5.3848, 4.19138	4.86925, 5	3	cntrl	587
GL173389.1	40096	40828	40411, 40301, 40142	9, 17, 6	6.57543, 14.22254, 5.02414	4.76012, 7.78557, 4.10097	4.08545, 1	3	cntrl	0
GL173389.1	73256	74078	73714, 73724, 73692	9, 9, 6	7.39636, 6.8008, 5.75442	5.27874, 4.90352, 4.53101	4.82212, 4	3	cntrl	0
GL173389.1	92983	94070	93312, 93671, 93689	10, 10, 14	9.95227, 8.79469, 16.12551	6.68696, 5.99491, 9.61761	7.19087, 6	3	rab14	5760
GL173389.1	110593	111356	111106, 110919, 110859	31, 47, 31	31.90101, 46.15953, 32.36224	13.66595, 15.29471, 13.93726	28.35191,	3	gsn	20513
GL173389.1	117978	118548	118386, 118286, 118369	10, 15, 6	8.89113, 12.53666, 6.35308	6.05396, 7.2687, 4.85498	6.20456, 9	3	gsn	13321
GL173389.1	122976	123306	123141, 123177, 123185	9, 17, 4	8.20841, 15.40668, 4.67325	5.77703, 8.50651, 3.78987	5.58112, 1	3	gsn	8563
GL173389.1	144366	144958	144811, 144760, 144626	9, 19, 9	5.06812, 11.79136, 6.88962	3.79653, 5.89289, 4.95986	2.74103, 9	3	gsn	0
GL173389.1	148597	149958	149914, 149914, 149909	37, 50, 32	35.80231, 46.92072, 33.76867	13.58413, 14.55202, 14.3728	32.17318,	3	gsn	0
GL173389.1	187109	187892	187497, 187585, 187625	6, 13, 9	5.96715, 13.14514, 11.12912	4.64927, 8.09002, 7.34851	3.52158, 1	3	NA	15180
GL173389.1	235060	235702	235467, 235299, 235669	6, 11, 9	5.47306, 9.39347, 10.35155	4.36955, 6.19476, 6.97128	3.10811, 6	3	NA	21621
GL173389.1	246993	247337	247035, 247019, 247034	81, 107, 54	103.92797, 128.83067, 71.83261	30.05223, 29.2517, 26.50481	99.50475,	3	NA	33554
GL173389.1	278383	278972	278911, 278903, 278875	12, 24, 17	10.68367, 23.62809, 20.18522	6.80062, 11.31356, 11.32722	7.88119, 2	3	ttll11	18900
GL173389.1	286915	287680	287372, 287233, 287316	7, 12, 6	7.1696, 11.48527, 7.29403	5.31345, 7.28792, 5.30581	4.61046, 8	3	ttll11	10192
GL173389.1	302654	303170	302960, 302967, 302924	7, 11, 3	6.84137, 9.17879, 3.47371	5.13151, 6.06147, 3.03189	4.33107, 6	3	ttll11	0
GL173389.1	331599	332227	331666, 331619, 331634	45, 50, 38	55.54271, 59.91041, 51.15282	21.45734, 21.58195, 22.08605	51.61233,	3	ttll11	0
GL173389.1	369528	370363	369945, 369853, 370039	6, 11, 8	4.25277, 8.97396, 8.74279	3.61311, 5.9338, 6.14792	2.08209, 6	3	ttll11	0
GL173389.1	419460	420210	419979, 419497, 419657	7, 7, 4	5.62168, 5.07986, 4.42274	4.40288, 4.05845, 3.67426	3.24345, 2	3	ttll11	16111
GL173389.1	434642	435256	435052, 435069, 435053	30, 33, 14	29.04424, 27.98422, 13.49044	12.37532, 10.74624, 8.08981	25.55974,	3	ttll11	31293
GL173389.1	447862	448113	448106, 448080, 448069	22, 26, 21	24.67026, 27.21682, 25.46511	12.59865, 12.75977, 13.3495	21.29787,	3	ttll11	44513
GL173389.1	453151	453420	453183, 453159, 453171	8, 13, 9	8.42257, 11.46128, 9.46739	5.97763, 7.07172, 6.50386	5.76336, 8	3	ttll11	49802
GL173390.1	32782	33942	33378, 33498, 33780	8, 18, 7	7.24433, 14.27393, 6.92462	5.30475, 7.55037, 5.17829	4.68023, 1	3	NA	13337
GL173390.1	37925	38672	38029, 38545, 38354	6, 7, 6	5.09949, 4.47213, 5.8719	4.14694, 3.6627, 4.59674	2.76939, 2	3	NA	18480
GL173390.1	76397	76756	76541, 76488, 76431	7, 7, 6	4.84123, 4.32411, 5.56953	3.90398, 3.56536, 4.42554	2.5312, 2.2	3	NA	0
GL173390.1	119948	120616	120435, 120428, 120187	13, 27, 10	13.63968, 28.00888, 11.38247	8.37925, 12.86965, 7.47651	10.67258,	3	NA	15627
GL173390.1	161918	162828	162240, 162447, 162020	8, 9, 5	6.70593, 7.92635, 5.14967	4.9768, 5.60609, 4.16141	4.20487, 5	3	NA	10301
GL173390.1	200448	201550	200777, 200905, 201135	16, 23, 14	13.15364, 17.47276, 13.98966	7.39585, 8.04022, 8.38968	10.20978,	3	NA	13726
GL173390.1	202101	202901	202577, 202295, 202610	12, 10, 6	8.2673, 4.86265, 4.36741	5.2988, 3.53382, 3.68738	5.63542, 2	3	NA	12375
GL173390.1	243098	243854	243429, 243485, 243476	34, 50, 21	37.49223, 53.07457, 22.99085	15.75733, 17.74381, 11.91236	33.83086,	3	NA	3188
GL173391.1	213875	214496	214198, 214205, 214145	8, 16, 5	8.42257, 17.16856, 5.9506	5.97763, 9.82359, 4.54784	5.76336, 1	3	NA	78189
GL173391.1	324544	325235	324798, 324764, 324986	14, 23, 7	12.53762, 17.93788, 6.75301	7.50905, 8.30082, 5.08141	9.62265, 1	3	NA	6769
GL173391.1	325368	325715	325518, 325480, 325446	8, 13, 6	5.78012, 7.6876, 5.60548	4.39198, 4.75803, 4.44624	3.38637, 5	3	NA	6289
GL173393.1	60344	60696	60546, 60392, 60531	12, 14, 11	13.8483, 14.15854, 12.9493	8.63436, 8.49024, 8.23866	10.86694,	3	anapc15	8850
GL173393.1	74489	75111	74935, 74516, 74791	4, 11, 19	3.73494, 8.48203, 20.55112	3.31698, 5.62571, 11.04971	1.6222, 5.9	3	anapc15	137
GL173393.1	109110	109592	109352, 109352, 109441	5, 7, 5	4.82177, 5.97725, 5.9506	3.98509, 4.62287, 4.54784	2.51418, 3	3	ngfr	24271
GL173393.1	130435	131022	130529, 130800, 130579	18, 16, 11	17.65252, 14.19759, 12.63525	9.59407, 8.03393, 8.07536	14.50784,	3	ngfr	2841
GL173393.1	267571	267795	267743, 267678, 267731	14, 13, 13	14.38232, 10.72425, 16.70862	8.62271, 6.61618, 10.0169	11.37721,	3	tubz1	14328
GL173393.1	337301	337914	337500, 337644, 337516	9, 13, 3	8.0222, 11.92581, 3.47371	5.66446, 7.35664, 3.03189	5.40727, 9	3	kif9	3989
GL173393.1	448492	449076	449009, 449069, 449013	14, 15, 10	16.7649, 15.80067, 11.83521	9.96273, 9.24574, 7.70798	13.6543, 1	3	kif9	95301
GL173394.1	61673	62294	61969, 62009, 61958	11, 16, 8	12.43693, 15.54288, 10.1465	7.97018, 8.85379, 6.82176	9.52692, 1	3	NA	41112
GL173394.1	124328	124640	124418, 124447, 124370	8, 8, 7	7.1122, 6.4822, 6.96907	5.22529, 4.83755, 5.20309	4.57946, 4	3	esyt3	0
GL173394.1	131739	132108	131886, 131902, 131800	8, 13, 8	8.4095, 12.86565, 9.76268	5.97056, 7.92422, 6.64964	5.76336, 1	3	esyt3	3265
GL173394.1	162736	163450	163147, 162976, 163117	8, 10, 7	8.42257, 9.39128, 8.08824	5.97763, 6.35644, 5.7849	5.76336, 6	3	esyt3	34262
GL173394.1	201962	202629	202008, 201991, 201997	86, 167, 54	112.57162, 215.13823, 74.19649	31.98549, 38.41197, 27.89042	108.08052	3	NA	13863
GL173394.1	347689	348012	347728, 347806, 347912	12, 12, 13	11.67221, 10.08092, 13.70995	7.39985, 6.42828, 8.41989	8.80793, 7	3	NA	6218
GL173394.1	390685	392003	390726, 391821, 391735	7, 36, 17	5.05064, 35.4501, 17.51936	4.03961, 13.77094, 9.78549	2.72521, 3	3	NA	11614
GL173394.1	390685	392003	391790, 391821, 391735	23, 36, 17	24.23344, 35.4501, 17.51936	12.01448, 13.77094, 9.78549	20.87361,	3	NA	11614
GL173396.1	1257	2081	2034, 2042, 2042	20, 23, 11	23.29435, 25.23193, 11.79996	12.37775, 12.61137, 7.62212	19.96127,	3	XB-GENE-5823762	11413
GL173396.1	85965	86763	86318, 86233, 86236	9, 9, 5	9.24298, 7.43399, 5.9506	6.3792, 5.30221, 4.54784	6.53474, 4	3	NA	3835
GL173396.1	180841	181345	181025, 181081, 181080	13, 11, 10	15.1723, 10.42613, 11.83521	9.23486, 6.8251, 7.70798	12.13305,	3	NA	0
GL173396.1	431337	431939	431391, 431538, 431439	5, 7, 5	4.82177, 5.36631, 5.64747	3.98509, 4.24177, 4.40911	2.51418, 3	3	NA	9731
GL173397.1	296724	297153	296838, 296974, 296912	9, 22, 9	8.45482, 22.00083, 10.87975	5.92419, 11.00232, 7.23113	5.79307, 1	3	ranbp9	4001
GL173398.1	120144	120936	120626, 120433, 120882	9, 23, 7	9.72082, 25.71782, 8.69489	6.64182, 12.90012, 6.06379	6.97155, 2	3	nbas	0
GL173398.1	435998	436629	436348, 436363, 436308	63, 72, 55	49.73377, 51.2611, 47.33242	11.8261, 10.38163, 13.07131	45.88481,	3	nbas	7590
GL173398.1	437430	437858	437700, 437695, 437747	54, 83, 49	39.87658, 64.32672, 40.33439	10.29392, 12.18213, 11.79242	36.17607,	3	nbas	9022
GL173398.1	439269	439643	439473, 439482, 439554	30, 26, 23	15.92075, 9.90962, 13.09588	5.78341, 3.99465, 5.7604	12.84796,	3	nbas	10861
GL173398.1	444782	445050	444914, 444952, 444901	40, 43, 30	38.81866, 37.10415, 30.67896	14.09331, 12.14148, 13.3307	35.13505,	3	nbas	16374
GL173398.1	446165	446613	446324, 446448, 446231	31, 45, 22	20.14135, 27.40643, 16.3064	7.40815, 7.55832, 7.65922	16.90681,	3	nbas	17757
GL173398.1	450968	452029	451774, 451778, 451589	169, 182, 109	181.55853, 183.68188, 117.05873	25.59645, 22.67376, 23.31176	176.66827	3	nbas	22560
GL173399.1	64015	64522	64483, 64483, 64482	19, 28, 20	19.18419, 30.69885, 26.49033	10.23232, 14.10238, 14.13188	15.98306,	3	has2	99120
GL173399.1	139920	140323	140256, 140285, 140292	6, 25, 13	5.96715, 26.60415, 15.0131	4.64927, 12.74915, 9.14923	3.52158, 2	3	has2	23319
GL173399.1	231970	232638	232045, 232012, 232424	15, 18, 7	15.41679, 17.65791, 7.55909	9.0192, 9.59733, 5.52035	12.36344,	3	has2	53765
GL173399.1	258909	259310	259250, 258950, 258965	8, 8, 4	7.24433, 5.61813, 4.22496	5.30475, 4.28778, 3.57747	4.68023, 3	3	has2	80704
GL173399.1	300377	300729	300384, 300407, 300410	10, 12, 7	10.39648, 8.99251, 7.61369	6.94096, 5.75004, 5.54854	7.61003, 6	3	has2	122172
GL173399.1	450292	451098	450919, 450914, 450919	25, 44, 22	30.32096, 53.69254, 28.83653	14.94603, 20.84663, 14.95889	26.80639,	3	has2	272087
GL173400.1	14308	15290	14566, 14644, 15025	4, 9, 5	3.23728, 5.79773, 4.20091	3.02372, 4.26249, 3.62458	1.33324, 3	3	NA	131858
GL173400.1	18571	18992	18652, 18606, 18800	10, 34, 14	8.84451, 34.82499, 14.99696	6.02544, 14.19696, 8.98153	6.16137, 3	3	NA	128156
GL173400.1	417253	418040	417687, 417687, 417705	18, 23, 9	17.93309, 22.38211, 10.79992	9.76366, 10.90305, 7.19283	14.77832,	3	creb3	0
GL173401.1	6987	7803	7441, 7432, 7482	13, 8, 11	12.43975, 6.44699, 11.86426	7.66875, 4.8155, 7.65794	9.5289, 4.0	3	fhod3	37692
GL173401.1	130998	131256	131127, 131128, 131158	8, 18, 6	8.42257, 17.25928, 6.12365	5.97763, 9.35577, 4.73409	5.76336, 1	3	fhod3	0
GL173401.1	436837	437075	437010, 437020, 437019	139, 134, 97	160.25652, 149.26619, 119.29397	28.64648, 26.39392, 29.58768	155.47418	3	pdia4	0
GL173401.1	447933	449438	448348, 448521, 448681	15, 15, 8	14.98937, 12.18092, 7.85773	8.76428, 7.05041, 5.66361	11.95813,	3	pdia4	4476
GL173402.1	24969	26020	25359, 25541, 25867	4, 5, 10	3.28906, 3.94561, 11.60305	3.0555, 3.46715, 7.59048	1.37521, 1	3	pthlh	197803
GL173402.1	37051	37936	37239, 37417, 37374	9, 18, 12	7.80169, 13.16644, 11.07378	5.52977, 6.89662, 7.03912	5.20141, 1	3	pthlh	185887
GL173402.1	70349	70623	70586, 70603, 70570	16, 33, 12	14.13236, 31.748, 11.81848	7.99402, 12.81693, 7.4869	11.1383, 2	3	pthlh	153200
GL173402.1	73431	74474	73714, 74164, 74060	13, 18, 5	10.25864, 13.12998, 4.12265	6.32743, 6.87536, 3.57684	7.48119, 1	3	pthlh	149349
GL173402.1	107551	108014	107643, 107708, 107721	5, 6, 5	4.69353, 4.56637, 5.50079	3.9134, 3.81494, 4.33868	2.45909, 2	3	pthlh	115809
GL173402.1	118972	119641	119104, 119172, 119102	8, 11, 5	7.28955, 8.41104, 5.50079	5.33177, 5.58113, 4.33868	4.72176, 5	3	pthlh	104182
GL173402.1	159447	159989	159664, 159883, 159737	8, 12, 4	8.42257, 11.86108, 4.67325	5.97763, 7.51216, 3.78987	5.76336, 9	3	pthlh	63834
GL173402.1	169054	169707	169528, 169497, 169475	11, 13, 9	11.71035, 12.93015, 9.41119	7.57196, 7.96262, 6.47287	8.8442, 10	3	pthlh	54116
GL173402.1	176327	177142	176686, 176666, 176421	8, 9, 6	8.42257, 8.21099, 6.99807	5.97763, 5.77858, 5.17195	5.76336, 5	3	pthlh	46681
GL173402.1	192717	193505	193498, 193440, 193468	4, 7, 5	3.74195, 5.97725, 5.9506	3.32091, 4.62287, 4.54784	1.6222, 3.6	3	pthlh	30318
GL173402.1	240897	241507	241479, 241461, 241201	12, 17, 5	11.92103, 17.4092, 4.77515	7.54764, 9.7196, 3.9592	9.04508, 1	3	KLHL42	0
GL173402.1	313732	314303	314158, 314156, 314168	7, 17, 12	6.16939, 16.43386, 12.88563	4.73936, 9.1315, 8.10565	3.70916, 1	3	NA	426
GL173402.1	339377	339574	339429, 339428, 339427	26, 28, 15	28.87987, 26.40614, 17.40815	13.75312, 11.5634, 10.16282	25.39905,	3	tulp3	397
GL173402.1	358006	358482	358023, 358038, 358040	24, 22, 16	26.57606, 25.85271, 19.45311	13.07811, 13.29074, 11.10978	23.15164,	3	rhno1	0
GL173402.1	375300	376529	375776, 375826, 376022	11, 19, 7	9.21796, 14.70435, 5.98393	6.08584, 7.53937, 4.62695	6.51275, 1	3	foxm1	7195
GL173402.1	411184	411637	411457, 411367, 411463	9, 15, 8	6.60612, 9.05755, 6.63406	4.77968, 5.18265, 4.93224	4.11315, 6	3	NA	3321
GL173402.1	414208	414712	414524, 414532, 414325	11, 18, 12	7.80634, 11.90241, 10.71774	5.20114, 6.17143, 6.82153	5.20581, 9	3	NA	5853
GL173402.1	417897	418536	418504, 418503, 418483	19, 20, 13	16.59925, 15.68063, 11.33661	8.66856, 7.8408, 6.9949	13.49961,	3	NA	2029
GL173402.1	442829	443820	443091, 443549, 443584	13, 23, 14	6.23426, 11.65914, 10.25207	3.92006, 5.09078, 6.10251	3.77051, 8	3	NA	14349
GL173402.1	442829	443864	443547, 443549, 443584	26, 23, 14	17.317, 11.65914, 10.25207	7.19621, 5.09078, 6.10251	14.18565,	3	NA	14349
GL173403.1	52931	54426	54120, 53759, 53373	10, 13, 11	9.661, 10.19363, 11.14477	6.51661, 6.28711, 7.24915	6.9283, 7.5	3	NA	NA
GL173403.1	180203	180800	180716, 180680, 180703	10, 21, 10	10.33015, 20.89182, 12.16489	6.9035, 10.65421, 7.87042	7.54792, 1	3	NA	NA
GL173403.1	240665	241131	241104, 241074, 241083	12, 14, 16	13.05874, 13.8891, 20.52695	8.20296, 8.32957, 11.67144	10.11961,	3	NA	NA
GL173403.1	301091	301442	301182, 301228, 301222	8, 9, 8	8.42257, 7.79702, 9.60885	5.97763, 5.5269, 6.57807	5.76336, 5	3	NA	NA
GL173403.1	412893	413182	413156, 413123, 413145	21, 15, 8	26.14967, 14.91533, 9.8421	13.73288, 8.71987, 6.68602	22.7357, 1	3	NA	NA
GL173405.1	39495	40022	39894, 39614, 39791	8, 12, 9	7.1122, 9.58381, 9.8849	5.22529, 6.11896, 6.72947	4.57946, 6	3	zhx2	25151
GL173405.1	84698	85552	85434, 85413, 84798	11, 9, 5	9.34791, 6.49199, 5.40789	6.16652, 4.70689, 4.29296	6.63394, 4	3	derl1	0
GL173405.1	137163	137440	137382, 137393, 137414	19, 35, 16	19.41153, 36.6591, 20.07743	10.36919, 14.85933, 11.44009	16.20286,	3	fam83a	0
GL173405.1	207177	207585	207429, 207429, 207429	9, 23, 13	8.77314, 23.82337, 15.34872	6.111, 11.76844, 9.3288	6.09355, 2	3	atad2	2820
GL173405.1	256137	256368	256324, 256306, 256321	10, 7, 6	10.89158, 5.75319, 7.29403	7.21503, 4.48487, 5.30581	8.0782, 3.4	3	fbxo32	12999
GL173405.1	340372	340877	340625, 340611, 340533	13, 20, 14	14.0251, 19.23568, 18.77534	8.6006, 9.96438, 10.96344	11.03614,	3	fam91a1	0
GL173405.1	419206	419603	419238, 419217, 419409	7, 12, 5	6.89193, 10.44309, 5.50079	5.15998, 6.65248, 4.33868	4.37772, 7	3	fer1l6	2934
GL173405.1	439467	440356	439637, 439613, 439739	21, 27, 9	22.40053, 25.65523, 10.79992	11.56075, 11.47654, 7.19283	19.0946, 2	3	fer1l6	23195
GL173406.1	2638	3298	2691, 3009, 3003	17, 16, 11	21.19088, 15.41438, 13.64572	11.87339, 8.77591, 8.58592	17.92307,	3	NA	213850
GL173406.1	18961	19260	19221, 19206, 19259	26, 43, 23	28.98144, 46.20208, 29.81154	13.81363, 16.96807, 15.24424	25.49811,	3	NA	197888
GL173406.1	61279	61781	61364, 61331, 61306	7, 14, 13	6.64707, 13.14791, 14.77384	5.02073, 7.88211, 9.01902	4.15135, 1	3	NA	155367
GL173406.1	77090	77524	77376, 77445, 77458	6, 12, 6	5.90927, 9.66318, 6.25896	4.61743, 6.16843, 4.80589	3.50164, 7	3	NA	139624
GL173406.1	80344	80941	80843, 80843, 80739	8, 15, 12	6.55639, 11.30021, 13.90113	4.88389, 6.51254, 8.66246	4.06705, 8	3	NA	136207
GL173406.1	96284	96933	96658, 96658, 96658	11, 12, 8	12.43693, 11.86108, 10.0913	7.97018, 7.51216, 6.79758	9.52692, 9	3	NA	120215
GL173406.1	198547	199308	199198, 199164, 198948	5, 6, 11	4.82177, 4.25354, 12.19945	3.98509, 3.61361, 7.8422	2.51418, 2	3	NA	17840
GL173406.1	314956	315422	315394, 315363, 315372	22, 18, 14	22.29116, 16.40493, 14.99696	11.17704, 8.83679, 8.98153	18.98923,	3	NA	81729
GL173406.1	337323	338138	337730, 337470, 337788	6, 8, 7	4.65447, 5.19022, 7.50542	3.87084, 4.01102, 5.49244	2.42652, 2	3	NA	104096
GL173406.1	350545	351168	351143, 351139, 351138	7, 17, 21	7.1696, 18.56115, 30.38164	5.31345, 10.40145, 15.90848	4.61046, 1	3	NA	117318
GL173406.1	381662	382355	381819, 381949, 381887	10, 15, 9	9.38842, 15.55346, 11.64359	6.35473, 9.10013, 7.57973	6.67167, 1	3	NA	148435
GL173406.1	410776	411738	411013, 410990, 411482	16, 26, 14	18.75167, 25.99905, 16.82614	10.72694, 12.03308, 9.99514	15.56543,	3	NA	177549
GL173407.1	3187	3858	3532, 3548, 3744	8, 8, 13	8.42257, 7.07159, 18.01082	5.97763, 5.20073, 10.61163	5.76336, 4	3	NA	15345
GL173408.1	443454	444556	443684, 443705, 444356, 443646	18, 27, 12, 14	15.11489, 24.55032, 7.83184, 14.2259	8.05499, 10.83345, 5.02982, 8.5302	12.07901,	3	nxph1	65599
GL173409.1	33578	34212	33872, 33830, 33968	7, 11, 5	6.94335, 10.42613, 4.67395	5.18876, 6.8251, 3.90232	4.42528, 7	3	NA	27459
GL173409.1	70401	71002	70749, 70662, 70695	4, 10, 7	3.54306, 8.20397, 8.15288	3.2072, 5.62902, 5.81587	1.57602, 5	3	NA	1212
GL173409.1	85799	86507	85970, 86114, 86173	12, 14, 6	7.89363, 7.64299, 5.14244	5.06786, 4.55693, 4.17298	5.28702, 5	3	NA	12712
GL173409.1	87460	88224	87885, 87548, 87640	14, 12, 6	10.78545, 6.95547, 5.56953	6.42953, 4.49628, 4.42554	7.97757, 4	3	NA	10995
GL173409.1	97830	98722	98450, 98403, 98001	17, 17, 8	13.31149, 13.63441, 8.09568	7.23535, 7.42967, 5.79794	10.36022,	3	NA	497
GL173409.1	175911	176423	176197, 176200, 176239	19, 23, 16	18.33514, 23.26667, 16.0646	9.71917, 11.43408, 9.16845	15.164, 20	3	U4	6776
GL173409.1	190383	190964	190826, 190473, 190698	13, 18, 9	9.45332, 12.60518, 7.2501	5.82899, 6.57148, 5.1872	6.7332, 9.8	3	NA	5510
GL173409.1	246391	246954	246456, 246500, 246569	6, 9, 14	5.3939, 7.88274, 15.88228	4.32314, 5.57944, 9.48329	3.0374, 5.4	3	NA	828
GL173409.1	273218	273918	273446, 273450, 273476	52, 49, 59	60.4828, 51.83057, 73.76686	20.94212, 17.49963, 25.00942	56.48999,	3	U4	598
GL173409.1	292477	293618	292884, 292890, 292881	128, 188, 122	136.97304, 206.3979, 135.51309	24.0279, 27.34224, 25.66625	132.32001	3	5S_rRNA	0
GL173409.1	309316	309646	309462, 309471, 309529	11, 12, 9	9.7619, 10.35, 11.39481	6.42195, 6.59497, 7.46977	7.0115, 7.7	3	U4	971
GL173409.1	328036	328586	328174, 328188, 328187	47, 74, 43	50.72242, 79.92539, 51.332	17.75947, 21.09239, 19.93078	46.85953,	3	5S_rRNA	0
GL173409.1	363342	364198	363729, 363725, 363892	6, 8, 5	5.96715, 7.07159, 5.9506	4.64927, 5.20073, 4.54784	3.52158, 4	3	NA	9960
GL173409.1	386487	387358	386894, 386690, 386701	13, 15, 8	11.26158, 13.31811, 8.14528	6.94861, 7.74852, 5.82558	8.41898, 1	3	U4	2076
GL173410.1	12574	12928	12603, 12602, 12604	27, 54, 39	29.73087, 58.68856, 49.8778	13.89723, 19.02271, 20.90854	26.22908,	3	zranb3	0
GL173410.1	41358	41692	41647, 41480, 41659	11, 9, 6	10.00935, 6.20816, 6.08001	6.57321, 4.52542, 4.71063	7.24529, 3	3	zranb3	3310
GL173410.1	43492	44039	44006, 43998, 44007	10, 29, 9	8.75281, 30.99206, 10.56949	5.9692, 13.89775, 7.08032	6.07523, 2	3	rab3gap1	1066
GL173410.1	54880	55734	54943, 55009, 55462	7, 8, 9	6.42049, 6.59014, 10.35155	4.8888, 4.90493, 6.97128	3.94161, 4	3	rab3gap1	0
GL173410.1	74376	74669	74576, 74620, 74583	10, 21, 9	6.83954, 15.3231, 7.58036	4.76771, 7.3702, 5.39321	4.33005, 1	3	rab3gap1	0
GL173410.1	78454	78885	78585, 78584, 78584	14, 16, 12	11.66068, 11.34727, 11.81848	6.969, 6.30719, 7.4869	8.79778, 8	3	rab3gap1	2438
GL173410.1	122809	123201	122873, 122811, 122886	6, 7, 3	5.5965, 5.8586, 3.47371	4.44108, 4.55007, 3.03189	3.21976, 3	3	ccnt2	5510
GL173410.1	143392	144104	143473, 143429, 143510	27, 25, 17	30.34385, 26.96634, 19.46435	14.2625, 12.9658, 10.92232	26.82879,	3	ccnt2	2034
GL173410.1	154552	155126	154815, 154823, 154781	22, 44, 9	23.62598, 45.73992, 10.07825	11.97797, 16.28375, 6.83102	20.28347,	3	acmsd	0
GL173410.1	174956	175614	175148, 174963, 174961	5, 16, 11	3.37168, 12.61232, 12.48556	3.09695, 7.0666, 7.99611	1.44277, 9	3	acmsd	5524
GL173410.1	179964	180636	179993, 179976, 179990	22, 32, 19	21.40417, 29.88364, 20.78218	10.64328, 12.12836, 11.18623	18.12938,	3	tmem163	3756
GL173410.1	181910	182318	181941, 182121, 182110	10, 9, 7	7.57902, 5.74975, 5.88867	5.23613, 4.23176, 4.56857	4.99303, 3	3	tmem163	2074
GL173410.1	193647	194751	194077, 194096, 194232	8, 11, 6	7.67464, 10.60895, 7.29403	5.55832, 6.9343, 5.30581	5.08299, 7	3	tmem163	0
GL173410.1	255863	256154	256023, 256068, 256056	13, 14, 8	12.49994, 13.95531, 7.38564	7.70504, 8.36917, 5.38892	9.58678, 1	3	mgat5	0
GL173410.1	291619	292004	291800, 291766, 291749	19, 12, 6	19.96873, 11.04188, 5.29847	10.70325, 7.01971, 4.26663	16.74065,	3	mgat5	0
GL173410.1	360365	360879	360562, 360561, 360562	23, 25, 16	24.94963, 25.99907, 19.86305	12.44303, 12.38692, 11.32783	21.56948,	3	mgat5	61911
GL173410.1	377362	377796	377386, 377406, 377411	88, 108, 68	103.0169, 126.65588, 89.55207	25.98406, 27.79114, 29.2091	98.60098,	3	mgat5	78908
GL173411.1	32623	33318	32668, 33243, 33146	10, 23, 7	11.06011, 22.74071, 7.66926	7.306, 11.1182, 5.57703	8.22712, 1	3	U3	7134
GL173411.1	37851	38354	37914, 38104, 38100	9, 12, 6	8.77314, 9.02737, 5.95321	6.111, 5.77179, 4.64162	6.09355, 6	3	U3	12362
GL173411.1	44713	45523	45510, 45042, 45300	5, 10, 10	4.5799, 9.39128, 12.33934	3.84863, 6.35644, 7.95424	2.3629, 6.8	3	U3	19224
GL173411.1	91634	92011	91854, 91935, 91918	6, 8, 7	5.86227, 6.4822, 7.40077	4.59138, 4.83755, 5.43746	3.46055, 4	3	U3	66145
GL173411.1	120089	120529	120477, 120454, 120387	5, 11, 3	4.0863, 8.41104, 3.2112	3.55452, 5.58113, 2.90793	1.93351, 5	3	U3	94600
GL173411.1	142908	143629	143031, 143167, 143161	6, 15, 10	5.96715, 15.80067, 13.18184	4.64927, 9.24574, 8.33771	3.52158, 1	3	U3	117419
GL173411.1	176883	177415	177386, 177391, 177379	28, 41, 29	32.96488, 43.14208, 37.50782	15.44861, 16.09106, 17.72857	29.39246,	3	U3	151394
GL173411.1	182928	183678	183329, 183565, 183364	9, 6, 6	9.63662, 4.93312, 7.29403	6.59633, 4.04501, 5.30581	6.90556, 2	3	U3	157439
GL173411.1	189516	189844	189668, 189671, 189680	28, 33, 24	38.31764, 41.48688, 30.17171	18.49968, 18.52972, 15.16988	34.6431, 3	3	agbl3	155250
GL173411.1	209108	209715	209433, 209355, 209395	6, 12, 7	5.96715, 10.93692, 8.69489	4.64927, 6.95571, 6.06379	3.52158, 8	3	agbl3	135379
GL173411.1	232554	232880	232762, 232697, 232684	10, 12, 5	9.95227, 11.86108, 5.31857	6.68696, 7.51216, 4.24819	7.19087, 9	3	agbl3	112214
GL173411.1	241375	241925	241876, 241884, 241917	30, 36, 11	30.1965, 36.57807, 11.5511	13.04721, 14.41064, 7.48213	26.68441,	3	agbl3	103169
GL173411.1	257442	257884	257846, 257663, 257748	8, 6, 4	8.34715, 4.93312, 4.67325	5.9367, 4.04501, 3.78987	5.71011, 2	3	agbl3	87210
GL173411.1	289877	291357	290105, 289991, 290440	7, 10, 9	6.16939, 6.93626, 9.24728	4.73936, 4.82903, 6.38161	3.70916, 4	3	agbl3	53737
GL173411.1	353740	354327	354186, 354173, 354202	13, 14, 6	14.10549, 12.54273, 5.8719	8.64628, 7.51218, 4.59674	11.11201,	3	agbl3	0
GL173411.1	450199	450657	450235, 450336, 450436	7, 5, 7	7.1696, 3.94561, 8.69489	5.31345, 3.46715, 6.06379	4.61046, 1	3	NA	0
GL173412.1	36854	37384	37120, 37191, 37167	9, 12, 8	8.40425, 11.66217, 10.00633	5.89416, 7.39386, 6.75998	5.76336, 8	3	NA	NA
GL173412.1	43341	43852	43807, 43787, 43807	27, 41, 12	29.0553, 46.29793, 14.76955	13.49404, 17.90246, 9.10943	25.57039,	3	NA	NA
GL173412.1	156352	157675	157307, 157314, 157162	16, 16, 6	15.60786, 14.19759, 7.29403	8.89312, 8.03393, 5.30581	12.5472, 1	3	NA	NA
GL173412.1	309439	310332	309727, 310186, 310104	13, 8, 9	11.60911, 5.67261, 10.64485	7.16262, 4.32287, 7.11743	8.74856, 3	3	NA	NA
GL173413.1	78	1512	242, 207, 239, 1391	26, 30, 13, 13	20.819, 20.52904, 10.41976, 10.01567	9.04044, 7.81898, 6.42737, 6.17679	17.56327,	3	focad	32017
GL173413.1	78	1536	955, 207, 239, 1391	19, 30, 13, 13	13.20984, 20.52904, 10.41976, 10.01567	6.67739, 7.81898, 6.42737, 6.17679	10.26285,	3	focad	31993
GL173413.1	389821	390497	390462, 390159, 390409	7, 9, 5	5.65516, 6.89892, 5.14967	4.42382, 4.96575, 4.16141	3.27228, 4	3	mllt3	77158
GL173413.1	424397	426008	425037, 425339, 425325	8, 16, 5	8.42257, 15.47832, 5.59755	5.97763, 8.81468, 4.38538	5.76336, 1	3	mllt3	111734
GL173414.1	322980	323268	322982, 322987, 323051	9, 14, 4	9.72082, 13.26509, 4.34116	6.64182, 7.95332, 3.63492	6.97155, 1	3	NA	3380
GL173414.1	333452	333858	333803, 333831, 333826	15, 12, 17	17.48779, 11.25867, 20.98465	10.20672, 7.1513, 11.76329	14.34959,	3	NA	0
GL173415.1	127578	128271	128161, 128151, 127801	11, 10, 5	11.09349, 9.39128, 5.9506	7.21938, 6.35644, 4.54784	8.25916, 6	3	NA	9922
GL173415.1	301388	301990	301807, 301474, 301859	6, 12, 12	5.96715, 11.86108, 15.80834	4.64927, 7.51216, 9.60504	3.52158, 9	3	NA	16598
GL173415.1	320104	320758	320385, 320268, 320151	10, 6, 11	9.83337, 4.53871, 14.68355	6.61776, 3.79731, 9.06344	7.07841, 2	3	NA	4334
GL173415.1	401589	402190	402018, 402037, 402072	11, 15, 8	9.90875, 11.76421, 7.72242	6.51186, 6.79531, 5.58595	7.14919, 9	3	NA	8105
GL173415.1	426390	427260	426928, 426942, 427089	15, 13, 7	17.09296, 12.14594, 8.69489	9.98755, 7.49077, 6.06379	13.96978,	3	NA	15584
GL173416.1	35772	36051	36022, 36019, 36017	13, 19, 12	13.34951, 18.14896, 14.96175	8.21021, 9.60638, 9.20442	10.39596,	3	NA	12270
GL173416.1	98404	99378	99347, 99343, 99285	12, 20, 14	13.8483, 22.87459, 19.68329	8.63436, 12.13503, 11.3696	10.86694,	3	NA	1431
GL173416.1	229193	229767	229507, 229507, 229486	16, 13, 8	14.62621, 9.74816, 7.95096	8.29596, 6.01114, 5.71659	11.61068,	3	NA	58653

GL173416.1	232357	233480	232684, 232771, 232925	9, 14, 4	6.69985, 11.90127, 3.59344	4.83936, 7.11741, 3.23643	4.20005, 9	3	SCARA5	55856
GL173416.1	232475	233480	233203, 232771, 232925	8, 14, 4	5.80896, 11.90127, 3.59344	4.41049, 7.11741, 3.23643	3.41336, 9	3	SCARA5	55856
GL173416.1	252418	252667	252450, 252450, 252468	14, 8, 5	14.38232, 5.81313, 5.14967	8.62271, 4.41317, 4.16141	11.37721,	3	SCARA5	36669
GL173417.1	54621	55100	54887, 54927, 54910	8, 10, 5	7.83097, 8.4887, 4.91762	5.64832, 5.80621, 4.03768	5.22917, 5	3	pmepa1	58263
GL173417.1	89919	90533	89986, 89957, 89961	26, 55, 23	30.86956, 63.3374, 26.60554	14.93039, 21.11024, 13.42348	27.34261,	3	pmepa1	22830
GL173417.1	129083	129551	129477, 129501, 129475	12, 17, 20	12.3187, 14.91871, 24.34577	7.78073, 8.20906, 12.9746	9.4231, 12	3	pmepa1	13491
GL173417.1	138055	138514	138194, 138402, 138406	11, 10, 9	10.27095, 8.66076, 11.21577	6.73177, 5.91256, 7.38849	7.4917, 6.1	3	pmepa1	22463
GL173417.1	141251	141497	141438, 141280, 141356	8, 14, 6	5.1114, 11.90127, 5.8719	3.95989, 7.11741, 4.59674	2.78023, 9	3	spire1	23774
GL173417.1	200930	201342	201044, 201107, 201005	7, 8, 9	5.33678, 7.07159, 9.41119	4.22299, 5.20073, 6.47287	2.98597, 4	3	pck1	12018
GL173417.1	221818	222506	222053, 222187, 221920	7, 12, 8	7.1696, 10.44309, 9.60885	5.31345, 6.65248, 6.57807	4.61046, 7	3	pck1	32906
GL173417.1	278140	278567	278411, 278405, 278357	18, 12, 7	19.51813, 11.86108, 8.69489	10.7103, 7.51216, 6.06379	16.30567,	3	rbm38	6546
GL173417.1	293218	293721	293282, 293281, 293263	14, 10, 10	14.16942, 9.32635, 10.97191	8.49669, 6.31756, 7.25854	11.17387,	3	rae1	0
GL173417.1	353159	353559	353200, 353422, 353261	5, 8, 6	4.82177, 5.78452, 5.2665	3.98509, 4.39481, 4.24757	2.51418, 3	3	bmp7.1	0
GL173417.1	403317	403923	403839, 403358, 403564	5, 16, 6	4.82177, 15.22617, 6.08001	3.98509, 8.66163, 4.71063	2.51418, 1	3	bmp7.1	22401
GL173417.1	421405	421869	421843, 421838, 421843	7, 14, 17	7.15806, 14.45897, 19.81591	5.30717, 8.66788, 11.12108	4.61046, 1	3	bmp7.1	40489
GL173417.1	429117	429918	429661, 429152, 429734, 429538	9, 8, 6, 4	9.72082, 7.07159, 4.93312, 4.67325	6.64182, 5.20073, 4.04501, 3.78987	6.97155, 4	3	bmp7.1	48201
GL173418.1	26	1499	424, 643, 859	15, 15, 9	18.26603, 15.80067, 11.64359	10.62691, 9.24574, 7.57973	15.09729,	3	snoU25	29978
GL173418.1	7926	8530	8404, 8347, 8279	17, 21, 11	16.36399, 19.60696, 12.06254	9.08912, 9.87975, 7.76744	13.27324,	3	snoU25	22947
GL173418.1	229247	230191	229547, 229373, 229979	7, 10, 8	6.2923, 7.79347, 8.9249	4.81292, 5.37143, 6.24211	3.8235, 5.3	3	rps6ka4	4738
GL173419.1	3188	3795	3225, 3316, 3224	17, 45, 16	17.48974, 48.60518, 19.35429	9.76779, 17.45266, 11.05657	14.35103,	3	selenbp1	2766
GL173419.1	69470	69949	69706, 69718, 69905	6, 17, 4	5.72604, 15.46309, 4.46488	4.51498, 8.5409, 3.69425	3.33547, 1	3	pogz	0
GL173419.1	93093	93710	93297, 93362, 93631	10, 15, 8	10.01299, 13.8628, 8.56955	6.7221, 8.08196, 6.05652	7.24763, 1	3	cgn	4898
GL173419.1	115400	116354	115939, 115808, 116006	10, 19, 7	8.08479, 16.30954, 7.40077	5.55446, 8.49413, 5.43746	5.46601, 1	3	cgn	1770
GL173419.1	140020	140383	140255, 140306, 140302	6, 8, 6	5.96715, 7.07159, 6.71074	4.64927, 5.20073, 5.03498	3.52158, 4	3	rab13	0
GL173419.1	145477	146167	145698, 145833, 145743	4, 8, 4	3.54306, 6.89656, 4.26291	3.2072, 5.09414, 3.59641	1.57602, 4	3	TUFT1	799
GL173419.1	193105	193526	193499, 193486, 193431	6, 10, 13	5.43318, 9.39128, 16.28606	4.34622, 6.35644, 9.81016	3.07284, 6	3	tpm3	0
GL173419.1	309759	310381	309936, 309804, 309796	7, 30, 9	7.1696, 33.30958, 9.52439	5.31345, 14.8899, 6.53516	4.61046, 2	3	NA	7577
GL173419.1	420263	420853	420318, 420317, 420556	7, 5, 5	7.1696, 3.94561, 5.9506	5.31345, 3.46715, 4.54784	4.61046, 1	3	NA	48175
GL173420.1	59715	60208	59917, 59891, 59961	11, 9, 14	11.29029, 8.21099, 16.46576	7.3332, 5.77858, 9.80275	8.44541, 5	3	NA	8763
GL173420.1	130152	130619	130516, 130503, 130525	5, 6, 9	4.82177, 4.93312, 11.64359	3.98509, 4.04501, 7.57973	2.51418, 2	3	NA	10899
GL173420.1	279809	280752	280405, 280314, 280442	16, 19, 8	17.08121, 18.41287, 8.56955	9.7724, 9.76624, 6.05652	13.95894,	3	NA	77575
GL173420.1	285080	285579	285540, 285542, 285558	11, 15, 7	12.43693, 15.80067, 8.69489	7.97018, 9.24574, 6.06379	9.52692, 1	3	NA	72748
GL173420.1	308093	308700	308301, 308327, 308275	12, 8, 6	13.8483, 7.07159, 7.29403	8.63436, 5.20073, 5.30581	10.86694,	3	NA	49627
GL173420.1	350224	350835	350553, 350742, 350592	5, 9, 4	4.82177, 8.21099, 4.67325	3.98509, 5.77858, 3.78987	2.51418, 5	3	NA	7492
GL173420.1	364098	364683	364325, 364325, 364326	16, 16, 15	17.08121, 15.47832, 18.95532	9.7724, 8.81468, 10.98488	13.95894,	3	NA	1452
GL173420.1	421163	421744	421483, 421494, 421578	13, 25, 11	15.1723, 30.45643, 14.03502	9.23486, 15.02432, 8.77076	12.13305,	3	NA	18368
GL173421.1	122381	122860	122615, 122707, 122753	13, 17, 7	12.81131, 14.97125, 6.2897	7.8918, 8.24108, 4.81137	9.88323, 1	3	camsap3	569
GL173421.1	145723	146265	145969, 146151, 146140	6, 8, 6	4.32795, 4.94245, 5.99481	3.66187, 3.85022, 4.6644	2.1485, 2.7	3	camsap3	0
GL173421.1	149903	151070	150624, 150467, 150911	8, 17, 7	6.63031, 15.13134, 8.02489	4.92991, 8.33865, 5.75426	4.13604, 1	3	camsap3	0
GL173421.1	223545	224150	223938, 224067, 224052	8, 15, 6	7.88469, 14.46023, 6.12365	5.67897, 8.44539, 4.73409	5.27935, 1	3	stxbp2	0
GL173421.1	260995	261325	261025, 261069, 261187	11, 18, 8	12.25244, 18.67996, 9.1831	7.87094, 10.2126, 6.37229	9.35958, 1	3	stxbp2	28367
GL173421.1	293529	294459	293832, 294220, 293727	12, 21, 11	11.79509, 19.00078, 13.83651	7.47301, 9.5157, 8.67735	8.92495, 1	3	stxbp2	60901
GL173421.1	340029	340870	340117, 340509, 340447	15, 17, 9	18.26603, 18.19786, 11.64359	10.62691, 10.18828, 7.57973	15.09729,	3	NA	33445
GL173422.1	64131	64663	64167, 64417, 64568	7, 8, 5	6.16939, 5.72807, 4.99237	4.73936, 4.35854, 4.0781	3.70916, 3	3	FAM114A2	42878
GL173422.1	87888	88227	88206, 88197, 88191	13, 17, 20	13.71465, 15.99248, 26.09407	8.42261, 8.86336, 13.92487	10.74495,	3	gria1	23958
GL173422.1	93447	93900	93522, 93565, 93563	8, 13, 8	8.22568, 11.51094, 9.92329	5.8701, 7.10228, 6.7228	5.59681, 8	3	gria1	18285
GL173422.1	155328	156376	156245, 156245, 155657, 156245	24, 39, 5, 16	29.14926, 44.19312, 4.77515, 19.066	14.58753, 17.56009, 3.9592, 10.89996	25.66204,	3	gria1	0
GL173422.1	216358	216837	216723, 216717, 216638	60, 94, 32	68.33228, 114.33751, 39.5574	21.47598, 28.63822, 17.79758	64.25286,	3	gria1	0
GL173422.1	374395	374862	374431, 374431, 374430	13, 15, 9	12.87577, 12.86621, 11.48736	7.93025, 7.47111, 7.51109	9.9455, 10	3	gria1	100706
GL173422.1	377477	377794	377561, 377729, 377508	7, 14, 4	5.58859, 11.36754, 4.64325	4.38214, 6.78813, 3.77643	3.21321, 8	3	gria1	103788
GL173422.1	384802	385130	384811, 384843, 384828	27, 23, 18	33.46234, 24.41444, 22.5396	16.09572, 12.12294, 12.41681	29.88004,	3	gria1	111113
GL173422.1	401220	401890	401364, 401511, 401248	4, 7, 4	3.74195, 5.8586, 4.67325	3.32091, 4.55007, 3.78987	1.6222, 3.5	3	gria1	127531
GL173423.1	171073	172454	171787, 172352, 171802	10, 12, 10	10.53228, 10.08092, 11.24131	7.01712, 6.42828, 7.40241	7.73927, 7	3	fam83c	6740
GL173425.1	50710	51310	50977, 50952, 50916	4, 12, 7	3.74195, 11.86108, 8.69489	3.32091, 7.51216, 6.06379	1.6222, 9.1	3	NA	12004
GL173425.1	143478	143855	143746, 143723, 143822	9, 7, 6	9.72082, 5.97725, 7.25145	6.64182, 4.62287, 5.287	6.97155, 3	3	NA	7811
GL173425.1	152607	153052	152921, 152756, 152915	10, 8, 5	11.06011, 7.07159, 5.80393	7.306, 5.20073, 4.48186	8.22712, 4	3	NA	16940
GL173425.1	164744	165733	165230, 165557, 165541	7, 8, 9	7.1696, 7.07159, 11.64359	5.31345, 5.20073, 7.57973	4.61046, 4	3	NA	15477
GL173425.1	174692	175359	174796, 174899, 174931	7, 12, 4	7.1696, 11.86108, 4.67325	5.31345, 7.51216, 3.78987	4.61046, 9	3	NA	5851
GL173425.1	198478	199194	198819, 198958, 198618	18, 21, 6	16.74626, 19.99241, 5.64193	9.04422, 10.11187, 4.46713	13.64034,	3	NA	3723
GL173425.1	243226	244038	243362, 243858, 243998	7, 10, 5	6.60038, 9.39128, 5.9506	4.99378, 6.35644, 4.54784	4.10761, 6	3	NA	48471
GL173425.1	257767	258350	258056, 258065, 258268	7, 17, 6	7.1696, 18.56115, 7.29403	5.31345, 10.40145, 5.30581	4.61046, 1	3	zc3h3	46806
GL173425.1	265728	266423	266084, 266130, 266226	8, 10, 5	8.42257, 9.39128, 5.9506	5.97763, 6.35644, 4.54784	5.76336, 6	3	zc3h3	38733
GL173425.1	278204	278726	278300, 278298, 278423	11, 15, 8	12.43693, 15.80067, 10.1465	7.97018, 9.24574, 6.82176	9.52692, 1	3	zc3h3	26430
GL173425.1	326905	327670	327016, 326940, 327246	5, 10, 11	4.27048, 8.74956, 12.9493	3.6666, 5.96721, 8.23866	2.09564, 6	3	zc3h3	0
GL173425.1	343122	343755	343690, 343302, 343179	7, 11, 9	5.62168, 9.8562, 10.14484	4.40288, 6.47973, 6.86555	3.24345, 7	3	zc3h3	0
GL173425.1	373368	373948	373674, 373409, 373796	11, 9, 6	9.52723, 6.8008, 4.96699	6.27749, 4.90352, 4.06589	6.80251, 4	3	zc3h3	0
GL173425.1	383707	384398	383933, 383910, 383923	12, 15, 12	9.45689, 9.44471, 10.76087	6.0398, 5.40644, 6.84799	6.73641, 6	3	zc3h3	0
GL173425.1	386283	386504	386375, 386376, 386387	34, 54, 25	38.12669, 59.64441, 28.93252	16.13209, 19.54091, 14.1346	34.4553, 5	3	zc3h3	0
GL173425.1	423939	424210	424171, 424158, 424173	12, 13, 9	12.38809, 10.51368, 10.01275	7.82099, 6.48562, 6.79683	9.48755, 7	3	MAFA	6103
GL173426.1	57886	58405	58010, 58367, 58270	8, 15, 3	7.47668, 13.92002, 3.17999	5.44269, 8.11689, 2.89245	4.89713, 1	3	rhbg	6244
GL173426.1	93527	94150	94090, 93575, 93616	5, 11, 3	4.73273, 9.8562, 3.37781	3.93547, 6.47973, 2.98791	2.49377, 7	3	hapln2	17033
GL173426.1	116500	116849	116635, 116734, 116773	8, 11, 5	7.99469, 10.60895, 5.9506	5.74128, 6.9343, 4.54784	5.38155, 7	3	hapln2	2283
GL173426.1	127836	128507	128118, 128351, 127946	9, 12, 4	9.72082, 10.73343, 4.67325	6.64182, 6.83116, 3.78987	6.97155, 8	3	NA	4580
GL173426.1	185447	185798	185766, 185757, 185771	11, 18, 13	10.21742, 13.85792, 14.25214	6.69945, 7.30313, 8.72916	7.44132, 1	3	nes	38179
GL173426.1	186082	186398	186167, 186108, 186106	8, 16, 6	6.66791, 11.76475, 5.46454	4.95324, 6.55585, 4.36458	4.17132, 9	3	nes	37579
GL173426.1	247257	247833	247267, 247277, 247283	12, 28, 14	12.45843, 30.60127, 15.80333	7.86167, 14.04419, 9.43935	9.54648, 2	3	dnaja4.2	5677
GL173426.1	254205	254947	254397, 254433, 254788	5, 16, 7	4.82177, 15.67391, 7.01416	3.98509, 8.93306, 5.22813	2.51418, 1	3	dnaja4.2	0
GL173426.1	275810	276362	276047, 275996, 275909	9, 16, 11	6.73165, 14.69174, 11.92945	4.85958, 8.33598, 7.69409	4.22986, 1	3	dnaja4.2	7779
GL173427.1	264904	265414	264979, 265352, 265173	9, 6, 5	8.61068, 4.93312, 5.40789	6.01615, 4.04501, 4.29296	5.94135, 2	3	NA	104705
GL173427.1	365572	366704	366494, 365888, 366161	5, 14, 11	4.82177, 14.45897, 14.75772	3.98509, 8.66788, 9.09568	2.51418, 1	3	NA	205373
GL173429.1	16870	17528	17257, 17041, 16932, 17301	9, 11, 3, 4	9.36986, 10.60895, 3.27564, 4.46488	6.44997, 6.9343, 2.93941, 3.69425	6.65425, 7	3	NA	444
GL173429.1	53634	54296	53711, 53872, 54038	15, 25, 11	18.26603, 28.66243, 13.83651	10.62691, 13.97513, 8.67735	15.09729,	3	NA	7138
GL173429.1	84802	85744	85171, 85499, 85167	16, 12, 7	17.77392, 10.58612, 8.69489	10.17481, 6.74063, 6.06379	14.62581,	3	NA	0
GL173429.1	141325	141702	141564, 141547, 141472	5, 6, 4	4.82177, 4.93312, 4.67325	3.98509, 4.04501, 3.78987	2.51418, 2	3	NA	6062
GL173429.1	295163	295653	295539, 295436, 295284	6, 6, 3	5.27951, 4.25354, 3.17999	4.25534, 3.61361, 2.89245	2.9335, 2.1	3	NA	133
GL173429.1	323831	324437	323988, 324304, 324171	12, 19, 7	11.49331, 18.14896, 8.21885	7.29275, 9.60638, 5.84717	8.63898, 1	3	NA	0
GL173429.1	348375	348940	348881, 348777, 348896	12, 20, 9	13.8483, 21.43553, 11.64359	8.63436, 11.28761, 7.57973	10.86694,	3	NA	20399
GL173429.1	367994	368402	368186, 368186, 368104	40, 46, 24	48.69246, 51.55863, 27.25833	19.75914, 18.67724, 13.48302	44.85831,	3	NA	40018
GL173430.1	312	735	703, 698, 655	8, 20, 15	8.42257, 22.87459, 18.33148	5.97763, 12.13503, 10.66149	5.76336, 1	3	cldn14	2327
GL173430.1	30872	31377	31302, 31344, 31343	11, 23, 9	11.42621, 22.74071, 9.94831	7.41109, 11.1182, 6.76298	8.57514, 1	3	cldn14	27098
GL173430.1	165085	166049	165728, 165729, 165455	30, 37, 13	34.59393, 39.98539, 15.17843	15.65348, 15.96556, 9.23814	30.98841,	3	hlcs	0
GL173430.1	325491	326575	326420, 326155, 326213	14, 8, 8	15.75768, 7.07159, 9.53432	9.41387, 5.20073, 6.54285	12.69046,	3	dscr3	564
GL173430.1	382000	382417	382236, 382175, 382230	8, 8, 3	8.42257, 6.55376, 3.47371	5.97763, 4.88226, 3.03189	5.76336, 4	3	dyrk1a	0
GL173431.1	29828	30306	29833, 29875, 29854	49, 82, 39	58.40389, 95.94815, 48.56166	21.19734, 25.34623, 20.13358	54.43562,	3	cdk8	6679
GL173431.1	181801	182247	181822, 181815, 181830	25, 42, 29	28.13526, 48.86538, 36.97725	13.6627, 18.94797, 17.42526	24.67218,	3	usp12	15714
GL173431.1	238434	238945	238555, 238592, 238574	9, 24, 6	9.43487, 27.28611, 6.5517	6.48595, 13.49944, 4.95623	6.71665, 2	3	mtif3	1226
GL173431.1	376380	376961	376861, 376711, 376722	6, 16, 4	5.95712, 14.57865, 4.01199	4.64377, 8.26691, 3.46784	3.52158, 1	3	LNX2	15698
GL173431.1	387632	388279	388117, 387731, 387796	16, 23, 17	15.67432, 22.59564, 21.88119	8.9333, 11.03113, 12.23429	12.61068,	3	LNX2	4380
GL173432.1	177701	178192	177916, 177775, 177919	20, 30, 11	18.82768, 30.89514, 11.31465	9.71777, 13.45807, 7.3472	15.63815,	3	NA	2890
GL173432.1	206146	206607	206581, 206563, 206565	63, 107, 47	77.62016, 129.12114, 59.49082	25.11789, 29.38732, 22.80773	73.44797,	3	itga2b.1	0
GL173432.1	388209	389010	388660, 388639, 388348	13, 22, 7	12.74759, 21.08061, 6.36213	7.85373, 10.44876, 4.85436	9.82421, 1	3	NA	23339
GL173433.1	30349	31115	30378, 30364, 30377	40, 70, 33	47.61701, 86.22749, 40.43161	19.12502, 26.25879, 17.91013	43.79825,	3	tenm4	18240
GL173433.1	82181	82842	82339, 82377, 82340	23, 44, 19	22.64709, 45.02779, 22.81433	11.06201, 15.89182, 12.35676	19.33432,	3	tenm4	0
GL173433.1	149144	149744	149335, 149331, 149441	10, 18, 4	10.13706, 16.6391, 4.67325	6.7935, 8.9791, 3.78987	7.36489, 1	3	tenm4	0
GL173433.1	161949	162523	162475, 162493, 162498	71, 102, 33	83.43866, 126.28924, 36.999	24.24748, 30.50153, 15.8777	79.20886,	3	tenm4	0
GL173433.1	256961	257322	257293, 257291, 257296	26, 49, 27	30.3953, 52.81398, 32.8241	14.65168, 18.03745, 15.7255	26.87883,	3	tenm4	0
GL173433.1	346564	347349	346655, 347075, 346989	7, 12, 3	6.50909, 10.35, 3.24307	4.94073, 6.59497, 2.92359	4.02371, 7	3	tenm4	0
GL173433.1	378812	379426	379185, 379045, 379219	8, 10, 9	6.63031, 7.61995, 9.41119	4.92991, 5.26198, 6.47287	4.13604, 5	3	tenm4	16904
GL173434.1	9919	11038	10228, 10192, 10117	18, 18, 11	16.93135, 14.06285, 12.55982	9.15667, 7.42469, 8.03554	13.81374,	3	neurl1b	14362
GL173435.1	19609	20139	19990, 19697, 20032	6, 8, 7	5.96715, 7.07159, 8.69489	4.64927, 5.20073, 6.06379	3.52158, 4	3	NA	1192
GL173435.1	124541	125982	125284, 125405, 125406	18, 11, 13	18.84377, 10.05535, 15.09516	10.31044, 6.6012, 9.19347	15.65357,	3	NA	9418
GL173435.1	148185	148736	148357, 148324, 148243	8, 8, 5	5.35164, 5.75617, 4.30991	4.11562, 4.3766, 3.69024	3.0004, 3.4	3	SLCO2B1	19998
GL173435.1	151489	151928	151888, 151878, 151890	26, 13, 12	26.04806, 10.19363, 13.29687	12.06225, 6.28711, 8.33527	22.63704,	3	SLCO2B1	16806
GL173435.1	180111	180698	180549, 180387, 180402	13, 14, 7	14.79381, 12.97647, 8.57075	9.02996, 7.77766, 6.00887	11.77132,	3	SLCO2B1	0
GL173435.1	279512	279841	279825, 279809, 279807	16, 17, 10	16.37806, 14.4631, 11.45478	9.35612, 7.93171, 7.51412	13.28648,	3	NA	5171
GL173435.1	376293	376670	376635, 376627, 376624	30, 40, 31	33.18705, 45.02302, 37.62455	14.81701, 17.60406, 17.05506	29.60997,	3	NA	11230
GL173435.1	385744	386721	386203, 385927, 386137	5, 18, 10	4.54329, 18.3735, 12.08015	3.82752, 10.0289, 7.82917	2.33, 15.38	3	cdk4	6357
GL173435.1	407039	407612	407251, 407243, 407287	15, 24, 8	17.90819, 27.60616, 10.1465	10.43573, 13.68835, 6.82176	14.75478,	3	cdk4	5539
GL173439.1	9893	10234	10151, 10140, 10094	14, 18, 7	15.071, 18.83799, 8.57075	9.02422, 10.30699, 6.00887	12.03664,	3	rxfp3	116870
GL173439.1	209843	210903	210308, 210528, 210308	9, 17, 11	7.35817, 15.18554, 11.14477	5.25488, 8.37169, 7.24915	4.78711, 1	3	slc45a2	1044
GL173439.1	278377	278939	278780, 278680, 278716	5, 9, 3	4.82177, 7.67202, 3.47371	3.98509, 5.44992, 3.03189	2.51418, 5	3	NA	27043
GL173439.1	291407	292127	292093, 292100, 291558	12, 18, 11	11.49331, 18.44896, 12.48556	7.29275, 10.0742, 7.99611	8.63898, 1	3	NA	13855
GL173439.1	351088	351503	351307, 351434, 351392	6, 17, 9	5.06496, 16.97468, 11.64359	4.12592, 9.45853, 7.57973	2.73812, 1	3	NA	4925
GL173439.1	366914	367119	367059, 367067, 367066	25, 23, 13	28.34522, 27.34036, 15.70541	13.78731, 13.85155, 9.51556	24.87704,	3	NA	20751
GL173439.1	402808	403235	403233, 403233, 402903	7, 7, 3	7.1696, 5.97725, 3.37781	5.31345, 4.62287, 2.98791	4.61046, 3	3	NA	56645
GL173441.1	21467	22441	21974, 21918, 21854	7, 9, 9	6.25077, 7.83963, 9.19414	4.78814, 5.55305, 6.35176	3.78441, 5	3	NA	132923
GL173441.1	28010	28443	28149, 28136, 28375	12, 18, 6	13.22036, 16.69883, 6.50067	8.29296, 9.01539, 4.93052	10.27237,	3	NA	126921
GL173441.1	53768	54283	54015, 53951, 54090	6, 7, 4	5.03089, 5.97725, 4.67325	4.10511, 4.62287, 3.78987	2.70624, 3	3	NA	101081
GL173441.1	63334	64221	63571, 63567, 63594	18, 20, 8	15.72423, 15.98596, 7.26744	8.42352, 8.01936, 5.31857	12.65901,	3	NA	91143
GL173441.1	111795	112300	112012, 112025, 112189	5, 7, 5	4.33555, 5.97725, 5.80393	3.70555, 4.62287, 4.48186	2.15195, 3	3	NA	43064
GL173441.1	205068	205683	205304, 205259, 205450	20, 20, 8	15.26665, 11.44306, 5.16822	7.60048, 5.50984, 3.99676	12.22466,	3	c11orf58	23125
GL173441.1	207445	208041	207785, 207558, 207706	12, 13, 12	7.47293, 6.0514, 9.54032	4.80986, 3.81832, 6.09184	4.89611, 3	3	c11orf58	20767
GL173441.1	209166	209759	209207, 209304, 209307	19, 45, 22	14.22783, 38.2846, 22.00648	7.26115, 12.06917, 11.00572	11.23024,	3	c11orf58	19049
GL173441.1	312518	312983	312977, 312932, 312917	20, 17, 13	20.21889, 15.0242, 13.97446	10.55812, 8.27335, 8.57174	16.98202,	3	pik3c2a	0
GL173441.1	391501	392376	391662, 391891, 392114	4, 7, 9	3.74195, 5.97725, 10.64485	3.32091, 4.62287, 7.11743	1.6222, 3.6	3	NA	0
GL173442.1	141	1263	548, 601, 190, 1039	22, 30, 16, 15	16.38143, 21.71333, 13.24565, 12.12091	7.70111, 8.39632, 7.45197, 7.01362	13.28981,	3	hibch	16993
GL173442.1	73157	73974	73328, 73287, 73288	12, 15, 9	11.37748, 13.01314, 10.56949	7.22306, 7.56134, 7.08032	8.52862, 1	3	hibch	0
GL173443.1	363483	364505	363512, 363946, 363923	4, 11, 4	3.23728, 8.59076, 4.26291	3.02372, 5.69393, 3.59641	1.33324, 6	3	SIPA1L2	3610
GL173444.1	11920	12675	12298, 12343, 12322	28, 35, 19	28.12112, 33.0906, 21.51797	12.56942, 12.82925, 11.61684	24.65842,	3	hdac8	0
GL173444.1	209463	209933	209597, 209521, 209693	7, 7, 4	6.50909, 5.27726, 4.55201	4.94073, 4.18506, 3.73489	4.02371, 3	3	shroom4	0
GL173444.1	382855	384445	383946, 384069, 384261	6, 15, 3	4.56865, 11.1534, 3.47371	3.81639, 6.42345, 3.03189	2.35429, 8	3	krt18l	118278
GL173444.1	389606	390269	389919, 390112, 390164	7, 10, 4	5.33678, 7.26311, 4.67325	4.22299, 5.03621, 3.78987	2.98597, 4	3	krt18l	125029
GL173445.1	88623	89110	88985, 88855, 89062	9, 16, 11	8.45482, 13.98946, 12.19945	5.92419, 7.9066, 7.8422	5.79307, 1	3	AMN1	15238
GL173445.1	98280	98488	98455, 98462, 98351	6, 19, 4	5.96715, 20.82621, 4.67325	4.64927, 11.21218, 3.78987	3.52158, 1	3	AMN1	5860
GL173445.1	115786	116218	116006, 115918, 115887	11, 14, 13	10.5483, 11.32527, 14.77384	6.89817, 6.76206, 9.01902	7.75329, 8	3	AMN1	0
GL173445.1	117371	117892	117439, 117540, 117570	5, 9, 7	3.61427, 5.65581, 6.08239	3.25593, 4.1716, 4.68683	1.6222, 3.3	3	AMN1	406
GL173445.1	135470	135856	135496, 135492, 135501	43, 41, 14	49.86065, 47.09922, 15.88228	19.07084, 18.36939, 9.48329	46.01003,	3	dennd5b	0
GL173445.1	148179	148742	148454, 148687, 148683	20, 16, 14	19.35636, 12.82211, 14.47197	10.03733, 7.19398, 8.67552	16.14934,	3	dennd5b	0
GL173445.1	149003	149312	149236, 149267, 149285	19, 12, 11	18.00678, 8.92355, 11.03484	9.52023, 5.70703, 7.18523	14.84918,	3	dennd5b	0
GL173445.1	195839	196910	196221, 196238, 195932	11, 9, 11	12.43693, 8.21099, 14.68355	7.97018, 5.77858, 9.06344	9.52692, 5	3	NA	0
GL173445.1	212130	212376	212351, 212334, 212314	7, 9, 10	7.1696, 8.21099, 12.90749	5.31345, 5.77858, 8.21675	4.61046, 5	3	kdm7a	0
GL173445.1	302346	302780	302718, 302646, 302715	6, 21, 7	5.96715, 22.39999, 8.49722	4.64927, 11.56043, 5.97582	3.52158, 1	3	mkrn1	0
GL173445.1	336320	336972	336479, 336552, 336512	19, 15, 13	20.21999, 13.80611, 13.97446	10.8531, 8.04733, 8.57174	16.98276,	3	DENND2A	0
GL173445.1	341741	342334	341758, 341783, 341939	7, 14, 7	7.1696, 12.49074, 7.66926	5.31345, 7.48027, 5.57703	4.61046, 9	3	DENND2A	1249
GL173445.1	365516	365805	365558, 365551, 365545	8, 7, 7	7.47668, 5.13485, 6.92462	5.44269, 4.09383, 5.17829	4.89713, 2	3	ADCK2	6521
GL173445.1	378824	379457	378833, 379256, 378833	10, 10, 5	9.18236, 8.44678, 5.50079	6.23091, 5.78021, 4.33868	6.47811, 5	3	ADCK2	2623
GL173446.1	21394	21714	21572, 21571, 21598	16, 19, 14	15.47689, 18.96889, 16.82614	8.81381, 10.10245, 9.99514	12.4218, 1	3	RIMKLB	26074
GL173446.1	29427	29733	29571, 29593, 29652	12, 13, 10	11.98519, 13.14514, 10.47831	7.58551, 8.09002, 6.98694	9.10644, 1	3	RIMKLB	18055
GL173446.1	56789	57194	57086, 57099, 57094	11, 17, 9	9.71398, 15.69342, 10.64485	6.39253, 8.68127, 7.11743	6.97155, 1	3	RIMKLB	3775
GL173446.1	96537	96817	96584, 96585, 96578	6, 6, 6	5.24248, 4.13436, 6.5517	4.2332, 3.53586, 4.95623	2.89962, 2	3	NA	7370
GL173446.1	119898	121256	120464, 120486, 120310	15, 28, 12	12.43022, 24.37082, 10.84828	7.20335, 10.39781, 6.90153	9.52692, 2	3	NA	2428
GL173446.1	119898	121256	121033, 120486, 120310	10, 28, 12	6.92459, 24.37082, 10.84828	4.82163, 10.39781, 6.90153	4.40864, 2	3	NA	2428
GL173446.1	147803	148444	147833, 147885, 147920	16, 33, 18	14.13236, 28.71334, 18.73766	7.99402, 11.13605, 10.24709	11.1383, 2	3	NA	3630
GL173446.1	162394	162825	162694, 162703, 162716	28, 50, 20	25.01726, 47.50276, 19.98274	10.76393, 14.84062, 10.41571	21.63609,	3	NA	0
GL173446.1	210230	210587	210403, 210416, 210395	25, 29, 18	28.13526, 28.35337, 20.40623	13.6627, 12.33938, 11.22809	24.67218,	3	NA	29468
GL173446.1	337420	337903	337730, 337631, 337738	20, 30, 8	15.90637, 23.16861, 6.15779	7.97271, 9.13398, 4.63309	12.83372,	3	NA	10329
GL173447.1	390000	390416	390258, 390250, 390104	9, 14, 6	5.71387, 8.19249, 4.91111	4.20878, 4.87218, 4.03141	3.32765, 5	3	NA	79473
GL173447.1	390834	392354	391631, 391631, 392266	19, 31, 9	17.09748, 26.50484, 8.60738	8.96946, 10.59929, 6.01421	13.97411,	3	NA	80307
GL173448.1	367325	368112	367634, 367544, 367645	11, 11, 5	9.17548, 8.70225, 5.54867	6.05941, 5.76383, 4.3619	6.47227, 6	3	uqcrfs1	37025
GL173448.1	387954	388560	388220, 388314, 388314	16, 17, 13	18.5447, 16.97468, 13.84064	10.61177, 9.45853, 8.49513	15.36536,	3	uqcrfs1	57654
GL173449.1	71800	72132	71986, 72079, 72055	8, 7, 10	8.42257, 5.8946, 11.6791	5.97763, 4.57222, 7.62925	5.76336, 3	3	gabrg2	45087
GL173449.1	374365	374706	374685, 374516, 374642	14, 13, 5	14.67861, 13.06145, 5.59755	8.79666, 8.04054, 4.38538	11.66087,	3	GABRA1	77735
GL173450.1	280613	281182	281027, 280999, 280957	5, 16, 6	4.82177, 17.16856, 7.29403	3.98509, 9.82359, 5.30581	2.51418, 1	3	bsx	53681
GL173451.1	388606	390355	388746, 390080, 389701	7, 28, 20	5.7233, 29.4016, 24.34577	4.4663, 13.32928, 12.9746	3.33547, 2	3	chka	30155
GL173451.1	388606	390355	389654, 390080, 389701	18, 28, 20	19.17347, 29.4016, 24.34577	10.50657, 13.32928, 12.9746	15.97256,	3	chka	30155
GL173451.1	406545	407024	406746, 406712, 406644	10, 9, 9	6.41567, 4.71739, 7.06537	4.49937, 3.57453, 5.07099	3.93897, 2	3	chka	48094
GL173452.1	129341	129914	129868, 129910, 129887	52, 59, 56	59.50017, 66.35104, 73.35641	20.38787, 20.87507, 26.3467	55.51827,	3	hspa5	2709
GL173452.1	211841	212427	212179, 212194, 211853, 212180	67, 69, 5, 38	81.40427, 79.08792, 3.32425, 47.97123	25.15925, 22.92716, 3.06549, 20.23009	77.19484,	3	rpl35	2565
GL173452.1	340067	340368	340342, 340327, 340132	10, 12, 8	10.74428, 10.98912, 8.09568	7.13454, 6.98757, 5.79794	7.93927, 8	3	wdr38	114154
GL173454.1	383184	383707	383360, 383424, 383447	22, 28, 14	26.43423, 30.03223, 16.82614	13.62565, 13.70494, 9.99514	23.01317,	3	NA	NA
GL173455.1	30835	31968	31936, 31781, 31328	8, 9, 11	6.90289, 7.10358, 13.28509	5.09802, 5.09508, 8.40871	4.38737, 4	3	NA	41770
GL173455.1	132771	133711	133373, 133479, 133233	6, 9, 4	5.86227, 7.28344, 4.67325	4.59138, 5.20811, 3.78987	3.46055, 4	3	NA	6509
GL173457.1	38	521	329, 351, 262	16, 23, 5	19.7934, 27.36982, 5.9506	11.29109, 13.8686, 4.54784	16.57067,	3	igsf21	153144
GL173457.1	20313	21064	20602, 20620, 20602	17, 12, 14	14.95512, 9.39099, 16.64329	8.23125, 5.99868, 9.89801	11.92636,	3	igsf21	132601
GL173457.1	30765	31139	30835, 30915, 31069	8, 13, 5	7.67464, 11.36332, 5.9506	5.55832, 7.01137, 4.54784	5.08299, 8	3	igsf21	122526
GL173457.1	36853	37430	37403, 37345, 37403	27, 34, 18	34.1727, 42.66207, 26.15761	16.50366, 18.81268, 14.19257	30.57599,	3	igsf21	116235
GL173457.1	117502	117974	117555, 117532, 117775	5, 7, 3	4.82177, 5.97725, 3.47371	3.98509, 4.62287, 3.03189	2.51418, 3	3	igsf21	35691
GL173457.1	156532	156771	156702, 156573, 156561	14, 25, 6	14.60315, 25.91543, 6.08001	8.75252, 12.33685, 4.71063	11.5888, 2	3	igsf21	0
GL173457.1	172156	172972	172355, 172475, 172888	4, 12, 7	3.74195, 11.86108, 7.84208	3.32091, 7.51216, 5.66426	1.6222, 9.1	3	igsf21	0
GL173457.1	211383	212284	211726, 211777, 212004	9, 17, 13	6.66833, 12.59401, 11.29503	4.8193, 6.8073, 6.96926	4.17154, 9	3	igsf21	0
GL173457.1	214054	214573	214069, 214158, 214219	11, 24, 10	8.50933, 19.8158, 7.89605	5.64284, 9.0745, 5.43599	5.845, 16.7	3	igsf21	0
GL173457.1	238405	238600	238448, 238446, 238417	16, 18, 14	15.47689, 16.81975, 13.70709	8.81381, 9.08887, 8.22039	12.4218, 1	3	igsf21	0
GL173457.1	248861	249458	249120, 249277, 249140	5, 7, 3	4.30277, 5.78791, 3.24307	3.68597, 4.5064, 2.92359	2.12436, 3	3	igsf21	0
GL173457.1	266024	266666	266318, 266408, 266305	8, 12, 5	6.31005, 9.78474, 4.45433	4.7294, 6.24415, 3.77574	3.84065, 7	3	igsf21	16380
GL173457.1	276982	277793	277515, 277515, 277211	9, 9, 6	7.84477, 5.32556, 5.56953	5.5562, 3.96047, 4.42554	5.24227, 3	3	igsf21	27338
GL173457.1	280194	280762	280637, 280534, 280515	10, 11, 10	7.9709, 7.91281, 9.82971	5.48303, 5.26803, 6.61563	5.36088, 5	3	igsf21	30550
GL173457.1	289615	290186	290076, 289924, 290115	6, 14, 6	4.17989, 11.53986, 5.46454	3.56562, 6.89446, 4.36458	2.01712, 8	3	klhdc7a	25162
GL173457.1	296152	296566	296234, 296348, 296204	10, 6, 5	10.01299, 4.76927, 5.40789	6.7221, 3.9431, 4.29296	7.24763, 2	3	klhdc7a	18782
GL173457.1	322848	323416	322951, 322946, 322996	13, 17, 12	11.21374, 15.24016, 13.01912	6.91908, 8.40499, 8.18077	8.37313, 1	3	klhdc7a	5730
GL173457.1	325455	326168	325912, 325651, 325976	10, 8, 7	8.0085, 4.69397, 6.43637	5.50663, 3.68889, 4.89813	5.39412, 2	3	klhdc7a	8337
GL173457.1	369705	370292	369776, 370022, 370033	6, 9, 5	5.90927, 8.21099, 5.91431	4.61743, 5.77858, 4.53172	3.50164, 5	3	klhdc7a	52587
GL173457.1	375135	376279	375602, 375609, 375562	22, 21, 16	25.18667, 21.11333, 18.88025	12.90256, 10.78774, 10.798	21.80058,	3	klhdc7a	58017
GL173457.1	380773	382066	381469, 381107, 381004, 381886	10, 7, 6, 5	10.39648, 5.97725, 5.49907, 4.91762	6.94096, 4.62287, 4.38471, 4.03768	7.61003, 3	3	klhdc7a	63655
GL173457.1	389286	390357	389790, 389776, 389380	21, 27, 9	21.33165, 25.58706, 10.28145	10.91925, 11.43661, 6.93568	18.05947,	3	klhdc7a	72168
GL173458.1	11844	12372	11884, 11875, 11908	21, 14, 11	19.84159, 12.92044, 10.66921	10.02101, 7.74345, 6.9701	16.61753,	3	NA	9658
GL173458.1	91686	92334	91905, 91950, 92204	10, 12, 9	9.661, 11.04188, 9.64095	6.51661, 7.01971, 6.59867	6.9283, 8.3	3	NA	9950
GL173458.1	135553	135868	135750, 135621, 135743	8, 6, 3	8.05102, 4.93312, 3.47371	5.77295, 4.04501, 3.03189	5.43473, 2	3	brpf1	0
GL173458.1	139002	139263	139220, 139174, 139212	14, 14, 6	14.99044, 12.38816, 7.25145	8.97777, 7.41725, 5.287	11.95856,	3	brpf1	0
GL173458.1	208171	208616	208538, 208392, 208295	10, 10, 5	11.06011, 8.61706, 5.9506	7.306, 5.8856, 4.54784	8.22712, 6	3	XB-GENE-5901104	1223
GL173459.1	16763	17539	16907, 16999, 17151	13, 15, 11	6.44198, 5.10554, 7.09192	4.0367, 3.10233, 4.75383	3.96273, 2	3	cntnap5	0
GL173459.1	80611	81114	80869, 80869, 80869	22, 20, 19	22.68783, 17.03084, 21.51797	11.41558, 8.63757, 11.61684	19.37324,	3	cntnap5	0
GL173459.1	105198	106067	105533, 105580, 105395	12, 12, 10	10.44071, 11.1491, 12.90749	6.65101, 7.08489, 8.21675	7.65237, 8	3	cntnap5	0
GL173459.1	197554	198748	198571, 198019, 197834	4, 7, 4	3.74195, 5.30663, 4.67325	3.32091, 4.20379, 3.78987	1.6222, 3.0	3	5S_rRNA	6027
GL173459.1	221267	221957	221401, 221366, 221473	6, 13, 7	5.55471, 10.51368, 6.75301	4.41698, 6.48562, 5.08141	3.18192, 7	3	5S_rRNA	16155
GL173459.1	268887	269412	269017, 269163, 269236	11, 12, 7	11.09349, 10.30411, 7.66926	7.21938, 6.56659, 5.57703	8.25916, 7	3	5S_rRNA	63775
GL173459.1	365121	365847	365356, 365375, 365340	10, 14, 5	11.06011, 14.45897, 5.9506	7.306, 8.66788, 4.54784	8.22712, 1	3	5S_rRNA	160009
GL173459.1	393371	393819	393614, 393595, 393600	16, 13, 14	17.3328, 10.98869, 16.73403	9.91952, 6.77996, 9.94634	14.20089,	3	5S_rRNA	188259
GL173459.1	407313	407635	407540, 407546, 407396	8, 14, 5	7.28955, 13.03306, 5.3628	5.33177, 7.81216, 4.27046	4.72176, 1	3	5S_rRNA	202201
GL173460.1	3196	3657	3561, 3385, 3453	10, 23, 7	11.06011, 26.02147, 8.69489	7.306, 13.07981, 6.06379	8.22712, 2	3	ric3	33828
GL173460.1	12109	12867	12561, 12485, 12548	7, 16, 5	6.50909, 16.67093, 5.9506	4.94073, 9.53036, 4.54784	4.02371, 1	3	ric3	24618
GL173460.1	42325	42730	42686, 42696, 42697	33, 39, 20	34.42642, 40.49654, 21.72938	14.36183, 15.42552, 11.4623	30.82458,	3	ric3	0
GL173460.1	86001	86569	86262, 86053, 86037	7, 11, 4	7.1696, 10.60895, 4.67325	5.31345, 6.9343, 3.78987	4.61046, 7	3	tub	23933

GL173460.1	98497	99181	99123, 98838, 99129	40, 61, 37	41.20566, 56.84684, 38.79473	15.41479, 15.34128, 15.27663	37.48402,	3	tub	36429
GL173460.1	114967	115762	115298, 115197, 115264	21, 35, 7	22.31353, 34.88273, 6.75301	11.50874, 13.83751, 5.08141	19.0106, 3	3	NA	22228
GL173460.1	123961	125188	124885, 124167, 124493	14, 10, 7	12.75226, 7.97488, 6.59055	7.64062, 5.48553, 4.98809	9.82689, 5	3	NA	12802
GL173460.1	192334	192899	192862, 192865, 192848	19, 28, 14	18.60811, 28.88701, 14.72868	9.8844, 13.02331, 8.82588	15.42706,	3	psma1	5298
GL173460.1	214121	214940	214601, 214498, 214495	15, 19, 11	13.57755, 14.70435, 11.31465	7.90752, 7.53937, 7.3472	10.61316,	3	pde3b	0
GL173460.1	218734	219337	219052, 219112, 219120	7, 18, 7	4.50966, 14.18874, 6.79502	3.68734, 7.49959, 5.10529	2.30322, 1	3	pde3b	0
GL173460.1	276925	277342	277298, 277326, 277305	24, 29, 15	28.65257, 32.15266, 18.13606	14.30048, 14.58867, 10.55788	25.17715,	3	pde3b	0
GL173460.1	287562	288359	287883, 288058, 287767	12, 14, 14	6.82234, 6.61986, 10.75327	4.41639, 3.9891, 6.40975	4.31393, 4	3	cyp2r1	0
GL173460.1	291433	293059	292373, 292387, 292510	27, 44, 15	21.30403, 34.97878, 11.59124	9.01197, 10.8378, 6.68971	18.03341,	3	cyp2r1	116
GL173460.1	303422	304149	304074, 303823, 304021	13, 13, 5	13.42064, 12.31712, 4.57714	8.25183, 7.59462, 3.84705	10.46414,	3	calca	7814
GL173460.1	333854	334838	334213, 334742, 334229	17, 18, 15	15.86203, 16.23409, 16.01939	8.78396, 8.73298, 9.37366	12.79136,	3	calca	16368
GL173460.1	344394	344882	344816, 344836, 344789	9, 15, 9	8.88537, 13.37045, 9.76105	6.17591, 7.78062, 6.66343	6.19961, 1	3	insc	15968
GL173460.1	365283	365676	365635, 365498, 365595	14, 23, 7	16.43009, 23.11341, 8.57075	9.78349, 11.34202, 6.00887	13.33671,	3	insc	0
GL173460.1	370956	371764	371353, 371334, 371221	13, 13, 11	15.29169, 13.14514, 14.45778	9.29854, 8.09002, 8.96373	12.24396,	3	insc	0
GL173461.1	260587	261072	260811, 260893, 260738	6, 10, 4	5.96715, 8.79469, 4.67325	4.64927, 5.99491, 3.78987	3.52158, 6	3	cxorf56	124045
GL173462.1	315528	316573	315831, 315314, 316347, 315559	8, 7, 9, 5	7.42892, 5.8586, 8.21099, 5.69847	5.41453, 4.55007, 5.77858, 4.4331	4.85345, 3	3	g3bp1	50397
GL173462.1	352672	354043	352916, 353535, 353079, 353797	9, 14, 6, 6	7.43495, 10.25698, 5.39683, 5.56953	5.30281, 6.10551, 4.32487, 4.42554	4.85899, 7	3	g3bp1	87541
GL173462.1	352672	354081	353873, 353535, 353079, 353797	7, 14, 6, 6	5.4599, 10.25698, 5.39683, 5.56953	4.30109, 6.10551, 4.32487, 4.42554	3.09813, 7	3	g3bp1	87541
GL173462.1	374148	375498	374755, 375166, 374632	10, 7, 9	11.06011, 5.97725, 11.64359	7.306, 4.62287, 7.57973	8.22712, 3	3	g3bp1	109017
GL173463.1	4488	5371	4959, 4960, 4958	29, 36, 27	30.24529, 37.9823, 33.63328	13.45419, 15.21857, 16.19431	26.7321, 3	3	p3h3	8694
GL173463.1	47739	48691	48309, 48283, 48578	10, 8, 5	9.44155, 6.93693, 4.54579	6.38646, 5.11882, 3.82897	6.72156, 4	3	GPR162	0
GL173463.1	96265	97255	96886, 96644, 96734	14, 28, 19	14.83249, 27.76847, 23.88486	8.88629, 12.36122, 12.94573	11.80835,	3	GPR162	29228
GL173463.1	203928	204294	204032, 204219, 203985	7, 13, 9	5.13345, 9.33876, 9.94831	4.09293, 5.75838, 6.76298	2.80161, 6	3	pianp	0
GL173463.1	248448	249291	249117, 249204, 249204	19, 18, 14	17.87921, 16.2906, 15.72539	9.44292, 8.76731, 9.39581	14.72728,	3	XB-GENE-1017156	0
GL173463.1	291065	291390	291387, 291388, 291387	14, 18, 12	13.38733, 16.34754, 12.95194	8.02744, 8.80191, 8.14303	10.43197,	3	ncapd2	10399
GL173463.1	371516	372466	371850, 371846, 371841	93, 106, 70	101.5622, 107.38931, 82.72266	23.09486, 20.64454, 24.36052	97.15829,	3	nop2	1941
GL173464.1	141698	141945	141761, 141731, 141795	8, 17, 11	8.42257, 18.56115, 14.75772	5.97763, 10.40145, 9.09568	5.76336, 1	3	elob	70922
GL173464.1	271679	272279	272018, 272019, 272018	7, 11, 6	7.1696, 9.30635, 7.29403	5.31345, 6.14075, 5.30581	4.61046, 6	3	kremen2	2200
GL173464.1	292730	293292	293233, 292807, 292799	7, 22, 16	7.1696, 25.60195, 21.00727	5.31345, 13.14513, 11.91233	4.61046, 2	3	cldn6.2	4597
GL173464.1	319895	320629	320272, 320271, 320125	6, 14, 8	5.3552, 11.90127, 8.4044	4.3003, 7.11741, 5.9678	3.00253, 9	3	eln2	5660
GL173464.1	357053	357525	357332, 357257, 357308	11, 8, 7	8.73311, 5.53815, 6.59055	5.78316, 4.2362, 4.98809	6.05699, 3	3	NA	332
GL173464.1	360122	360712	360561, 360555, 360543	15, 18, 9	13.46874, 16.46278, 9.46739	7.84087, 8.87194, 6.50386	10.51051,	3	NA	3401
GL173465.1	8860	9402	9325, 9043, 8950	6, 11, 6	5.96715, 10.60895, 7.29403	4.64927, 6.9343, 5.30581	3.52158, 7	3	ppp1ca	4818
GL173465.1	35464	36016	35793, 35615, 35858	8, 15, 4	5.47927, 11.3375, 3.70359	4.19817, 6.53521, 3.29934	3.11408, 8	3	NA	2042
GL173465.1	52439	53119	52659, 52824, 52605	12, 14, 8	11.79509, 10.88047, 8.51364	7.47301, 6.48797, 6.02666	8.92495, 8	3	NA	1815
GL173465.1	95729	96567	96013, 96204, 95977	6, 6, 4	5.96715, 4.93312, 4.67325	4.64927, 4.04501, 3.78987	3.52158, 2	3	atp2a1	6389
GL173466.1	60196	61212	60388, 60550, 60486	5, 23, 7	4.33555, 21.38155, 8.08824	3.70555, 10.30482, 5.7849	2.15195, 1	3	NA	10208
GL173466.1	66493	67010	66777, 66598, 66546	21, 17, 12	17.46864, 12.78176, 10.84828	8.60523, 6.91875, 6.90153	14.33148,	3	NA	16505
GL173466.1	128349	128861	128473, 128555, 128497	18, 14, 8	19.97207, 14.45897, 9.60885	10.97636, 8.66788, 6.57807	16.74381,	3	NA	25006
GL173466.1	185223	185919	185692, 185777, 185625	8, 8, 6	6.78332, 5.12058, 5.39683	5.02459, 3.96585, 4.32487	4.27811, 2	3	wnt7a	0
GL173466.1	190137	190962	190644, 190779, 190575	14, 19, 9	12.64394, 16.67602, 8.26067	7.57426, 8.71486, 5.80842	9.72408, 1	3	wnt7a	0
GL173466.1	242868	243558	243193, 243179, 243205	8, 15, 11	6.20993, 13.42325, 12.34038	4.66614, 7.81299, 7.91841	3.74679, 1	3	NA	22594
GL173466.1	258897	259452	259020, 258985, 259065	13, 20, 11	13.20994, 19.85827, 11.6121	8.12823, 10.34058, 7.51665	10.26285,	3	gnas	28832
GL173466.1	304399	305037	304612, 304588, 304566	9, 12, 9	9.72082, 11.72254, 10.87975	6.64182, 7.42986, 7.23113	6.97155, 9	3	gnas	0
GL173466.1	323836	324217	324065, 323862, 324090	9, 9, 7	9.30591, 7.35794, 7.40077	6.41439, 5.25474, 5.43746	6.59443, 4	3	gnas	0
GL173466.1	344896	345538	345118, 345257, 345136	9, 9, 9	8.25643, 8.21099, 10.72164	5.80588, 5.77858, 7.15493	5.62516, 5	3	nelfcd	230
GL173466.1	368853	369220	369170, 369166, 368932	6, 17, 4	5.95712, 17.4092, 4.67325	4.64377, 9.7196, 3.78987	3.52158, 1	3	ctsz	0
GL173467.1	91980	92496	92380, 92389, 92082	23, 26, 13	21.03902, 21.86052, 12.98465	10.10107, 9.62194, 7.995	17.77621,	3	SLC5A6	66457
GL173467.1	98204	98477	98399, 98391, 98398	9, 15, 6	7.51337, 13.58442, 6.76586	5.35162, 7.91173, 5.06179	4.93225, 1	3	SLC5A6	60476
GL173467.1	113809	114547	114045, 114258, 113917	6, 7, 3	5.96715, 5.97725, 3.47371	4.64927, 4.62287, 3.03189	3.52158, 3	3	SLC5A6	44406
GL173467.1	230560	230980	230889, 230899, 230924	6, 10, 5	5.27951, 8.84031, 4.67395	4.25534, 6.02287, 3.90232	2.9335, 6.3	3	hadha	0
GL173467.1	260582	261008	260606, 260615, 260615	9, 13, 13	9.18105, 12.25946, 16.49347	6.3444, 7.55969, 9.91245	6.47733, 9	3	NA	10462
GL173468.1	6855	7435	7020, 6933, 7102	14, 11, 10	16.7649, 10.60895, 13.18184	9.96273, 6.9343, 8.33771	13.6543, 7	3	mical2	35416
GL173468.1	76637	77188	76974, 76870, 76811	9, 11, 7	7.75911, 10.26356, 7.84208	5.5036, 6.72731, 5.66426	5.16246, 7	3	mical2	0
GL173468.1	94324	95081	94674, 94507, 94431	7, 8, 7	7.1696, 7.07159, 8.35502	5.31345, 5.20073, 5.9108	4.61046, 4	3	mical2	0
GL173468.1	132682	133039	132959, 132995, 132987	6, 15, 12	4.45871, 13.92002, 12.09328	3.74614, 8.11689, 7.64909	2.26108, 1	3	mical2	11695
GL173468.1	134514	134819	134812, 134785, 134739	11, 15, 12	9.66657, 12.91479, 12.62857	6.36338, 7.50094, 7.95947	6.93336, 1	3	mical2	13527
GL173468.1	149901	150408	150038, 150037, 150038	10, 15, 9	9.23294, 15.19102, 9.14171	6.26141, 8.88488, 6.32219	6.52626, 1	3	dkk3	13471
GL173468.1	160544	160890	160865, 160851, 160856	16, 23, 11	19.7934, 26.44164, 13.1143	11.29109, 13.32732, 8.32282	16.57067,	3	dkk3	2989
GL173468.1	286240	286764	286327, 286283, 286685	22, 18, 9	22.52709, 19.41853, 10.87975	11.31895, 10.65157, 7.23113	19.2175, 1	3	usp47	59735
GL173468.1	309379	309757	309520, 309617, 309608	4, 6, 10	3.42565, 4.56637, 11.31133	3.13796, 3.81494, 7.43928	1.48489, 2	3	usp47	82874
GL173468.1	332346	332751	332719, 332714, 332525	9, 10, 5	9.72082, 9.39128, 5.9506	6.64182, 6.35644, 4.54784	6.97155, 6	3	usp47	105841
GL173468.1	354121	354678	354122, 354133, 354149	13, 28, 22	13.07382, 28.07714, 26.90598	8.04786, 12.54342, 13.89421	10.13353,	3	usp47	127616
GL173468.1	363931	364480	363996, 363959, 363966	19, 40, 30	15.78667, 35.4806, 33.93866	8.18067, 12.32799, 15.26407	12.71882,	3	usp47	137426
GL173468.1	389533	390104	390075, 390078, 390073	25, 24, 21	28.66822, 23.48334, 24.0973	13.97856, 11.22699, 12.56358	25.19216,	3	usp47	163028
GL173468.1	398052	398446	398293, 398105, 398220	7, 7, 4	7.1696, 5.97725, 4.67325	5.31345, 4.62287, 3.78987	4.61046, 3	3	usp47	171547
GL173469.1	36826	37188	36903, 36931, 37042	14, 14, 7	14.52862, 12.70158, 7.10639	8.70881, 7.60958, 5.27894	11.51702,	3	PIRT	27095
GL173469.1	53610	54374	53640, 53641, 53636	21, 22, 18	20.87917, 19.42606, 22.00076	10.64659, 9.46019, 12.12506	17.62177,	3	PIRT	43879
GL173469.1	56240	56941	56684, 56273, 56351	19, 27, 6	17.4485, 25.18667, 5.20372	9.18194, 11.20272, 4.20995	14.31174,	3	PIRT	46509
GL173469.1	130738	131270	131089, 131040, 130808	6, 10, 4	4.7134, 7.75815, 3.79082	3.90801, 5.34918, 3.34814	2.47723, 5	3	NA	112675
GL173469.1	199876	200941	200508, 200556, 200473	9, 20, 19	9.72082, 21.88095, 24.85428	6.64182, 11.5521, 13.45893	6.97155, 1	3	NA	43004
GL173469.1	218296	218708	218325, 218335, 218375	10, 22, 12	11.06011, 24.08291, 16.28773	7.306, 12.25042, 9.81872	8.22712, 2	3	NA	25237
GL173469.1	251393	251990	251660, 251793, 251909	5, 9, 5	4.23867, 7.03406, 4.33804	3.64743, 5.05123, 3.70703	2.0685, 4.6	3	NA	0
GL173469.1	252196	252715	252518, 252634, 252633	7, 11, 10	6.01305, 9.01418, 9.93124	4.6447, 5.9589, 6.67475	3.56347, 6	3	NA	0
GL173469.1	390805	391502	391082, 391007, 391141	7, 16, 10	6.09019, 12.32991, 10.30677	4.69155, 6.89567, 6.89026	3.63504, 9	3	NA	0
GL173469.1	404635	405198	404688, 405115, 404789	7, 12, 7	5.52352, 7.99652, 6.21898	4.34124, 5.1313, 4.76912	3.15454, 5	3	NA	2484
GL173469.1	407990	408847	408557, 408214, 408675	6, 19, 9	5.81608, 17.64648, 9.64095	4.56563, 9.30188, 6.59867	3.4176, 14	3	NA	5839
GL173470.1	59	1390	1196, 533, 1219	14, 26, 18	15.75768, 27.65533, 20.75291	9.41387, 13.0219, 11.42699	12.69046,	3	NA	15154
GL173470.1	5960	6797	6651, 6013, 6127	10, 11, 11	8.84451, 8.20476, 10.5698	6.02544, 5.45153, 6.91098	6.16137, 5	3	NA	9747
GL173470.1	7014	7915	7315, 7369, 7589	8, 9, 6	6.63031, 7.13885, 5.49907	4.92991, 5.11729, 4.38471	4.13604, 4	3	NA	8629
GL173470.1	17101	17478	17292, 17106, 17137	6, 11, 9	5.16999, 8.10521, 9.64095	4.18963, 5.38896, 6.59867	2.83325, 5	3	NA	0
GL173470.1	18617	19229	18802, 18692, 18681	5, 13, 8	4.47183, 9.89237, 7.72242	3.78598, 6.1004, 5.58595	2.26935, 7	3	NA	0
GL173470.1	64437	65066	64906, 65025, 65037	27, 37, 17	31.44513, 37.28955, 20.18522	14.9159, 14.41741, 11.32722	27.90458,	3	NA	15126
GL173470.1	112258	112643	112463, 112458, 112487	10, 17, 6	9.83337, 15.51996, 7.29403	6.61776, 8.57556, 5.30581	7.07841, 1	3	NA	29643
GL173470.1	277022	278833	277205, 277149, 277012, 278494	10, 10, 5, 8	9.33595, 8.04981, 4.39541, 8.24665	6.32332, 5.53254, 3.74107, 5.88165	6.62299, 5	3	NA	0
GL173472.1	110172	111215	110824, 110802, 110346	11, 24, 8	12.17114, 25.12019, 9.92329	7.82679, 12.2078, 6.7228	9.28263, 2	3	syn2	0
GL173472.1	115622	116730	115913, 116313, 116430	8, 5, 8	7.77807, 3.58952, 9.1831	5.618, 3.23987, 6.37229	5.17956, 1	3	syn2	0
GL173472.1	133684	133935	133727, 133691, 133713	41, 46, 46	45.58145, 48.77369, 56.56651	17.48698, 17.11298, 21.57759	41.79387,	3	syn2	2970
GL173472.1	234718	235496	234823, 234971, 234977	4, 9, 4	3.23728, 7.35794, 4.42274	3.02372, 5.25474, 3.67426	1.33324, 4	3	FGD3	6895
GL173472.1	234746	235496	235438, 234971, 234977	4, 9, 4	3.31554, 7.35794, 4.42274	3.07164, 5.25474, 3.67426	1.39756, 4	3	FGD3	6895
GL173472.1	265016	265288	265090, 265083, 265087	104, 114, 72	116.14404, 131.70216, 90.21541	24.90295, 27.35978, 27.40047	111.62519	3	FGD3	2404
GL173472.1	285144	285887	285265, 285268, 285370	7, 12, 5	6.46447, 10.68384, 5.64747	4.91463, 6.80072, 4.40911	3.98312, 8	3	FGD3	22532
GL173472.1	305497	305859	305645, 305651, 305642	12, 24, 14	13.13892, 23.48334, 16.91966	8.24771, 11.22699, 10.04443	10.1962, 2	3	BICD2	25220
GL173472.1	317235	317536	317442, 317438, 317455	14, 28, 21	15.75768, 33.56964, 26.82916	9.41387, 15.80476, 14.10583	12.69046,	3	BICD2	13543
GL173472.1	323308	324925	323685, 323771, 323763	10, 14, 4	10.07458, 13.38467, 4.64325	6.75761, 8.02582, 3.77643	7.30652, 1	3	BICD2	6154
GL173472.1	333468	334247	333748, 333758, 333962	23, 16, 12	21.67593, 11.76475, 12.50469	10.48041, 6.55585, 7.88834	18.39184,	3	BICD2	0
GL173472.1	342369	342896	342657, 342516, 342545	5, 5, 5	4.23867, 3.73203, 5.10931	3.64743, 3.33183, 4.14026	2.0685, 1.7	3	BICD2	0
GL173472.1	361598	362020	361865, 361896, 361683	4, 7, 3	3.70152, 5.82304, 3.47371	3.29816, 4.52813, 3.03189	1.6222, 3.5	3	BICD2	0
GL173473.1	1036	1968	1327, 1305, 1296, 1574	16, 16, 12, 12	10.155, 9.10727, 9.12115, 9.12115	5.61134, 5.02156, 5.8303, 5.8303	7.38249, 6	3	atl3	13680
GL173473.1	11719	11943	11887, 11837, 11804	24, 33, 28	6.77606, 7.32639, 14.13835	3.0591, 2.74409, 5.37425	4.27202, 4	3	atl3	3705
GL173475.1	72622	73146	72873, 72973, 73114	10, 6, 5	9.23294, 4.62266, 5.9506	6.26141, 3.8507, 4.54784	6.52626, 2	3	NA	9403
GL173475.1	155569	156092	155944, 155944, 155798	14, 11, 11	12.43323, 8.23846, 9.97106	7.44494, 5.47271, 6.54988	9.52692, 5	3	NA	597
GL173475.1	158073	158876	158502, 158223, 158391	12, 15, 11	9.69483, 13.06292, 9.84332	6.18815, 7.59191, 6.47185	6.96086, 1	3	NA	0
GL173475.1	178557	179401	179192, 179108, 178810	16, 28, 7	18.75167, 29.31424, 7.61369	10.72694, 13.27729, 5.54854	15.56543,	3	NA	10126
GL173475.1	261456	261975	261926, 261910, 261861	8, 23, 12	8.42257, 27.36982, 15.80834	5.97763, 13.8686, 9.60504	5.76336, 2	3	NA	18936
GL173475.1	307827	308219	308188, 308168, 308150	12, 16, 15	11.37748, 16.29763, 15.88211	7.22306, 9.30808, 9.29347	8.52862, 1	3	NA	937
GL173476.1	38588	39369	38958, 38935, 38948	8, 22, 9	6.78332, 20.68559, 9.46739	5.02459, 10.21163, 6.50386	4.27811, 1	3	lrfn5	277757
GL173476.1	116850	119433	118586, 118570, 117128, 118880	31, 39, 7, 24	28.47612, 32.26438, 4.21814, 22.91617	11.69085, 11.03771, 3.49552, 10.88848	25.00436,	3	lrfn5	197693
GL173477.1	35466	36011	35554, 35551, 35553	64, 115, 42	74.48429, 136.07944, 52.65003	22.88424, 28.82456, 21.17275	70.34288,	3	ralyl	0
GL173481.1	2345	2754	2560, 2616, 2515	18, 25, 13	10.72119, 14.48465, 10.2815	5.51944, 6.03881, 6.34161	7.91768, 1	3	qtrt1	13432
GL173481.1	7793	8739	8297, 8335, 7936	9, 21, 8	5.18738, 13.58157, 6.13023	3.87239, 6.41774, 4.61559	2.85031, 1	3	qtrt1	7447
GL173481.1	133131	133695	133165, 133188, 133191	10, 12, 9	10.67246, 11.86108, 11.04428	7.09497, 7.51216, 7.30896	7.87096, 9	3	NA	11678
GL173481.1	233580	234603	233786, 233764, 233799	60, 91, 39	67.62178, 102.59374, 47.81944	21.0928, 24.41117, 19.69558	63.55029,	3	tmed1	18883
GL173481.1	240049	240602	240553, 240537, 240552	34, 58, 59	33.47109, 52.87105, 64.21683	13.4193, 14.56825, 19.73857	29.88859,	3	tmed1	12884
GL173481.1	241601	242065	242038, 242017, 242034	35, 60, 54	34.4272, 55.74982, 57.11369	13.57884, 15.1899, 18.18178	30.82509,	3	tmed1	11421
GL173481.1	301313	302212	301640, 301639, 301644	6, 7, 5	5.03089, 5.97725, 5.9506	4.10511, 4.62287, 4.54784	2.70624, 3	3	dnm2	0
GL173481.1	316950	317458	317252, 317266, 317226	10, 12, 7	6.4408, 7.2756, 4.83008	4.51525, 4.68974, 3.89673	3.96156, 4	3	dnm2	0
GL173481.1	320769	321374	320957, 321000, 321028	26, 19, 23	23.07976, 12.95248, 22.82596	10.31801, 6.53239, 11.16938	19.75307,	3	dnm2	0
GL173481.1	339593	340895	339918, 339916, 340735	17, 23, 14	11.10571, 12.36302, 10.81929	5.94142, 5.41361, 6.45034	8.27035, 9	3	dnm2	17802
GL173481.1	360427	360951	360745, 360764, 360735	22, 24, 16	18.23604, 19.1975, 15.00193	8.76062, 8.7238, 8.52517	15.07022,	3	dnm2	38636
GL173481.1	369784	370307	370160, 370172, 370162	15, 41, 20	18.26603, 51.17868, 25.47128	10.62691, 20.76829, 13.59302	15.09729,	3	dnm2	47993
GL173482.1	22870	23438	23248, 23312, 23176	7, 10, 5	6.74267, 8.0878, 5.45388	5.07552, 5.55635, 4.3157	4.23998, 5	3	NA	92407
GL173482.1	63687	64801	64569, 64109, 64163	5, 24, 6	4.82177, 28.04916, 7.29403	3.98509, 13.94861, 5.30581	2.51418, 2	3	NA	51044
GL173482.1	87879	88842	88807, 88756, 88790	13, 14, 10	14.0251, 14.45897, 12.42918	8.6006, 8.66788, 7.99682	11.03614,	3	NA	27003
GL173482.1	128073	128458	128430, 128422, 128429	14, 11, 14	15.84929, 10.26356, 17.40994	9.46495, 6.72731, 10.29833	12.77923,	3	NA	7877
GL173482.1	289450	289786	289479, 289480, 289483	18, 16, 11	19.51813, 16.01323, 12.34038	10.7103, 9.13759, 7.91841	16.30567,	3	NA	21016
GL173482.1	307725	308461	308020, 307987, 307778	9, 11, 7	6.51482, 9.61892, 7.84208	4.72146, 6.33404, 5.66426	4.02888, 7	3	rdd2	32733
GL173482.1	314049	314760	314594, 314462, 314398	30, 35, 25	31.82977, 38.99848, 31.40622	14.01074, 16.22895, 15.56799	28.282, 35	3	rdd2	26434
GL173483.1	101484	102055	101790, 101807, 101810	8, 16, 6	7.67464, 13.49669, 5.20372	5.55832, 7.60526, 4.20995	5.08299, 1	3	cracr2a	0
GL173483.1	103996	104535	104398, 104393, 104344	9, 19, 9	8.88537, 16.78404, 8.60738	6.17591, 8.78005, 6.01421	6.19961, 1	3	cracr2a	0
GL173483.1	177230	178101	177797, 177798, 177650	20, 24, 11	23.40409, 24.1521, 11.92945	12.44081, 11.62735, 7.69409	20.06837,	3	MGAT4B	5340
GL173483.1	207757	208095	207910, 207763, 208017	5, 11, 8	4.5799, 9.66539, 9.1831	3.84863, 6.36265, 6.37229	2.3629, 7.0	3	MGAT4B	0
GL173483.1	230452	231028	230699, 230782, 230798	13, 29, 12	13.34951, 29.51828, 14.40488	8.21021, 13.02394, 8.92522	10.39596,	3	MGAT4B	16114
GL173483.1	286317	286564	286502, 286519, 286539	20, 16, 19	22.76587, 16.37065, 22.04849	12.07179, 9.3517, 11.92281	19.44912,	3	maml1	0
GL173483.1	302527	303036	303003, 302985, 302954	9, 12, 11	8.50607, 11.04188, 14.03502	5.95453, 7.01971, 8.77076	5.84194, 8	3	maml1	2473
GL173484.1	15016	15699	15166, 15372, 15389	7, 10, 6	6.55439, 7.93794, 6.03707	4.96711, 5.46232, 4.6874	4.06616, 5	3	ptpn4	11694
GL173484.1	49064	49546	49099, 49068, 49163	9, 19, 8	6.66833, 14.9136, 7.34578	4.8193, 7.66241, 5.36526	4.17154, 1	3	ptpn4	0
GL173484.1	84775	85452	85043, 84851, 84863	12, 20, 8	10.39353, 16.58658, 8.9249	6.62187, 8.3735, 6.24211	7.60856, 1	3	epb41l5	0
GL173484.1	91570	92333	91754, 91751, 92221	9, 15, 9	6.28237, 11.11727, 10.14484	4.57292, 6.40155, 6.86555	3.81457, 8	3	epb41l5	0
GL173484.1	95558	96031	95953, 95955, 95924	23, 20, 9	21.87577, 16.25749, 9.19414	10.59983, 8.17901, 6.35176	18.58593,	3	epb41l5	0
GL173484.1	98255	99763	99043, 98953, 99161	9, 21, 7	6.39666, 19.29849, 7.20145	4.646, 9.69431, 5.33074	3.92119, 1	3	epb41l5	0
GL173484.1	117654	118269	118219, 118234, 118112	13, 22, 15	12.56081, 25.60195, 16.99143	7.74168, 13.14513, 9.93059	9.64511, 2	3	kalrn	6327
GL173484.1	220193	220676	220559, 220574, 220409	11, 18, 8	8.43721, 15.09059, 8.4586	5.59757, 8.04034, 5.99709	5.77761, 1	3	NA	0
GL173484.1	265087	265515	265312, 265306, 265115	5, 6, 14	3.54515, 3.40406, 15.20637	3.21097, 3.0502, 9.10193	1.57763, 1	3	NA	20343
GL173484.1	267610	268051	267810, 267827, 267809	11, 23, 9	8.61979, 19.65454, 7.7212	5.71214, 9.28582, 5.48026	5.94958, 1	3	NA	22866
GL173484.1	285311	285968	285921, 285754, 285380	8, 6, 10	6.78332, 3.45514, 10.90706	5.02459, 3.08455, 7.22344	4.27811, 1	3	NA	18829
GL173484.1	290585	291038	290802, 290956, 290818	6, 11, 7	5.96715, 10.60895, 8.08824	4.64927, 6.9343, 5.7849	3.52158, 7	3	NA	13759
GL173484.1	297999	298549	298469, 298476, 298463	7, 10, 9	6.79162, 8.79469, 11.04428	5.10336, 5.99491, 7.30896	4.28406, 6	3	NA	6248
GL173484.1	309945	310303	310199, 310024, 310210	7, 7, 11	7.1696, 5.97725, 12.63525	5.31345, 4.62287, 8.07536	4.61046, 3	3	NA	507
GL173484.1	327149	327879	327360, 327507, 327646	5, 7, 5	4.61714, 5.82304, 5.91431	3.86998, 4.52813, 4.53172	2.39372, 3	3	NA	11
GL173484.1	327859	328600	328481, 328458, 327646, 328424	10, 12, 5, 5	10.67246, 11.60251, 5.91431, 5.91431	7.09497, 7.3582, 4.53172, 4.53172	7.87096, 8	3	NA	0
GL173484.1	336292	337041	336605, 336691, 336462	5, 10, 9	4.82177, 9.39128, 11.48736	3.98509, 6.35644, 7.51109	2.51418, 6	3	NA	6861
GL173484.1	359443	359791	359527, 359624, 359524	7, 11, 8	5.30682, 6.96322, 7.76694	4.20391, 4.67372, 5.6116	2.95991, 4	3	NA	30012
GL173484.1	361926	362848	362267, 362454, 362257	23, 30, 13	25.4258, 27.71453, 14.69628	12.72675, 11.61145, 8.97644	22.03293,	3	NA	32495
GL173484.1	372910	373923	373638, 373521, 373707	12, 12, 13	11.37748, 11.31437, 15.09516	7.22306, 7.18497, 9.19347	8.52862, 8	3	NA	43479
GL173487.1	70233	70876	70470, 70613, 70578	19, 23, 10	19.80551, 21.25586, 10.53703	10.60563, 10.22998, 7.01977	16.58234,	3	nup205	0
GL173487.1	100073	100623	100328, 100253, 100484	6, 11, 5	5.96715, 10.60895, 5.69847	4.64927, 6.9343, 4.4331	3.52158, 7	3	nup205	5655
GL173487.1	117603	118025	117684, 117693, 117725	9, 27, 8	9.72082, 30.77408, 10.1465	6.64182, 14.51829, 6.82176	6.97155, 2	3	slc13a4	0
GL173487.1	166133	166739	166525, 166639, 166604	10, 10, 3	11.06011, 9.39128, 3.47371	7.306, 6.35644, 3.03189	8.22712, 6	3	slc13a4	25642
GL173487.1	171970	172836	172175, 172591, 172448	13, 17, 7	15.1723, 18.56115, 8.02489	9.23486, 10.40145, 5.75426	12.13305,	3	mtpn	22668
GL173487.1	212086	212569	212471, 212495, 212448	35, 46, 16	43.84078, 57.79924, 20.07743	19.09013, 22.30123, 11.44009	40.07852,	3	mtpn	0
GL173487.1	258270	258604	258297, 258295, 258307	13, 21, 10	14.79381, 24.22016, 12.16489	9.02996, 12.63513, 7.87042	11.77132,	3	mtpn	33678
GL173487.1	274410	274872	274788, 274794, 274558	7, 14, 10	6.01305, 10.99827, 10.71812	4.6447, 6.5605, 7.12015	3.56347, 8	3	mtpn	49818
GL173487.1	309775	310435	310124, 309945, 309872	8, 12, 5	7.33538, 11.86108, 5.9506	5.35908, 7.51216, 4.54784	4.76518, 9	3	mtpn	85183
GL173487.1	316731	317500	316846, 316845, 317060	10, 13, 4	10.96713, 12.61502, 4.67325	7.25596, 7.77427, 3.78987	8.14942, 9	3	mtpn	92139
GL173487.1	331942	332377	332340, 332294, 332347	15, 16, 10	14.98937, 17.16856, 12.90749	8.76428, 9.82359, 8.21675	11.95813,	3	mtpn	107350
GL173487.1	341064	341587	341549, 341549, 341559	17, 23, 12	16.36399, 23.5013, 13.7434	9.08912, 11.57503, 8.57827	13.27324,	3	mtpn	116472
GL173487.1	354151	354780	354357, 354486, 354748	5, 7, 6	4.655, 5.33631, 7.12188	3.89157, 4.22269, 5.22884	2.42652, 3	3	mtpn	129559
GL173487.1	372969	373759	373554, 373345, 373533	6, 13, 9	5.03089, 11.92581, 10.87975	4.10511, 7.35664, 7.23113	2.70624, 9	3	mtpn	148377
GL173487.1	376230	376667	376529, 376470, 376276	7, 8, 5	6.55439, 6.21066, 5.27517	4.96711, 4.66659, 4.22616	4.06616, 3	3	mtpn	151638
GL173488.1	222517	223383	222874, 222867, 222830	14, 35, 16	13.70063, 35.12344, 18.88025	8.21651, 13.97479, 10.798	10.73143,	3	osbpl10	50125
GL173489.1	42109	42783	42551, 42252, 42331	7, 9, 5	6.09019, 7.03406, 5.50079	4.69155, 5.05123, 4.33868	3.63504, 4	3	MINK1	41297
GL173489.1	57050	58035	57221, 57573, 57099	7, 10, 5	6.79162, 9.39128, 4.77515	5.10336, 6.35644, 3.9592	4.28406, 6	3	MINK1	26045
GL173489.1	66784	67792	67378, 67006, 67468	7, 7, 6	6.99564, 5.52121, 7.29403	5.21786, 4.33979, 5.30581	4.47307, 3	3	MINK1	16288
GL173489.1	87302	88717	87863, 87827, 87678	6, 14, 16	4.48574, 10.54152, 16.43863	3.76346, 6.27972, 9.39225	2.28228, 7	3	MINK1	0
GL173489.1	118653	119134	119079, 119076, 119090	13, 26, 13	14.10549, 30.27268, 16.93213	8.64628, 14.5794, 10.12358	11.11201,	3	MINK1	4333
GL173489.1	200499	201372	201047, 200773, 200866	8, 7, 5	7.15567, 4.7469, 5.59755	5.25151, 3.84255, 4.38538	4.61046, 2	3	rangrf	284
GL173489.1	238350	238952	238624, 238698, 238398	7, 16, 5	5.19005, 12.82211, 4.67395	4.12926, 7.19398, 3.90232	2.85262, 1	3	chrne	0
GL173489.1	242726	243445	243255, 242873, 243066	13, 14, 5	10.98084, 10.22245, 4.84526	6.7751, 6.08441, 3.99806	8.16259, 7	3	chrne	0
GL173489.1	253949	254437	254042, 254211, 254096	6, 8, 5	5.55471, 7.07159, 5.69847	4.41698, 5.20073, 4.4331	3.18192, 4	3	eif4e2	1151
GL173489.1	268402	269416	268481, 268609, 268519	69, 109, 45	51.61457, 84.68585, 37.74145	11.10028, 12.81728, 11.81329	47.7401, 8	3	eif4e2	0
GL173489.1	322724	323275	322865, 323131, 322938	5, 10, 8	4.82177, 8.74956, 10.0913	3.98509, 5.96721, 6.79758	2.51418, 6	3	NA	26327
GL173489.1	355249	355865	355299, 355275, 355252	17, 22, 8	17.88146, 21.78003, 8.4044	10.00115, 10.86943, 5.9678	14.72916,	3	NA	0
GL173489.1	359845	360356	360014, 359913, 360039	8, 9, 6	6.48409, 6.52169, 6.12365	4.83873, 4.72584, 4.73409	4.00135, 4	3	NA	0
GL173491.1	52719	53293	52755, 52855, 53166	13, 15, 7	12.56081, 13.92002, 6.83761	7.74168, 8.11689, 5.12939	9.64511, 1	3	mbnl1	123900
GL173492.1	154	765	669, 366, 350	13, 12, 6	15.29169, 11.86108, 7.29403	9.29854, 7.51216, 5.30581	12.24396,	3	gpr37	25049
GL173494.1	4380	6266	5525, 5526, 5544	28, 31, 12	21.97606, 19.29649, 8.85135	9.08243, 7.02904, 5.66201	18.68307,	3	NA	NA
GL173494.1	47161	48209	47654, 47759, 47594	17, 30, 13	12.34441, 21.10554, 11.33661	6.6598, 8.09737, 6.9949	9.44574, 1	3	NA	NA
GL173494.1	52047	52875	52714, 52715, 52610	47, 62, 23	48.24549, 64.43645, 24.04869	16.40251, 18.56261, 11.90367	44.41845,	3	NA	NA
GL173494.1	58860	59356	59030, 59159, 59113	7, 22, 11	7.1696, 24.96436, 12.9493	5.31345, 12.77201, 8.23866	4.61046, 2	3	NA	NA
GL173494.1	80196	81034	80459, 80798, 80691	9, 28, 9	9.72082, 33.56964, 11.64359	6.64182, 15.80476, 7.57973	6.97155, 3	3	NA	NA
GL173494.1	88457	90121	89793, 89643, 89051	16, 24, 11	11.48924, 17.47967, 9.67839	6.3915, 7.77602, 6.37065	8.63527, 1	3	NA	NA
GL173494.1	94658	95397	95068, 94928, 94933	22, 26, 30	21.69032, 24.83666, 33.57177	10.81544, 11.34395, 15.04586	18.40561,	3	NA	NA
GL173494.1	102505	103320	103107, 103077, 102893	49, 89, 30	55.42102, 104.40909, 36.8562	19.49177, 26.18932, 16.98881	51.49193,	3	NA	NA
GL173494.1	113284	113732	113470, 113567, 113554	25, 27, 13	23.9866, 23.26933, 12.70856	11.18574, 10.10097, 7.83036	20.63364,	3	NA	NA
GL173494.1	144733	145372	145101, 145117, 145003	18, 22, 14	15.83097, 17.79763, 13.59776	8.48825, 8.50637, 8.15458	12.7618, 1	3	NA	NA
GL173494.1	153329	154075	153645, 153727, 153518	8, 9, 8	6.0497, 5.99677, 6.63406	4.56438, 4.39002, 4.93224	3.59874, 3	3	NA	NA
GL173494.1	193450	194421	193826, 193671, 193479	13, 10, 5	12.81131, 8.53104, 4.99237	7.8918, 5.83243, 4.0781	9.88323, 6	3	NA	NA
GL173494.1	202756	203296	203110, 202958, 203128	28, 44, 17	28.60673, 45.83094, 18.35257	12.85696, 16.3341, 10.27929	25.132, 42	3	NA	NA
GL173494.1	207265	208409	207871, 207550, 207863	22, 32, 8	16.26324, 22.64898, 5.76741	7.63516, 8.34136, 4.38382	13.1768, 1	3	NA	NA
GL173494.1	211358	212079	211738, 211578, 211683	18, 11, 6	13.20598, 4.94975, 4.36741	6.9197, 3.45762, 3.68738	10.2604, 2	3	NA	NA
GL173494.1	229874	231014	230558, 230418, 230451	18, 24, 13	12.98682, 15.81036, 11.21286	6.79206, 6.89857, 6.91854	10.05096,	3	NA	NA
GL173494.1	243709	245274	244645, 244750, 244765	20, 32, 11	11.41647, 17.83671, 5.76233	5.49627, 6.22379, 3.93817	8.56664, 1	3	NA	NA
GL173494.1	249126	250146	249817, 249591, 249814	31, 44, 22	19.60375, 27.13369, 16.20599	7.16562, 7.63998, 7.60329	16.38853,	3	NA	NA
GL173494.1	282452	283013	282904, 282770, 282604	6, 24, 6	3.5367, 17.59504, 5.33084	3.13931, 7.83833, 4.28587	1.57353, 1	3	NA	NA
GL173494.1	283817	285179	284389, 284556, 284210	20, 23, 10	18.1524, 15.26482, 10.03522	9.31021, 6.84801, 6.73493	14.98854,	3	NA	NA
GL173495.1	5869	6537	6274, 6149, 6075	11, 7, 8	11.71035, 5.97725, 9.76268	7.57196, 4.62287, 6.64964	8.8442, 3.6	3	pdzd8	0
GL173495.1	74945	75444	75252, 75255, 75172	11, 14, 4	12.43693, 14.45897, 4.67325	7.97018, 8.66788, 3.78987	9.52692, 1	3	PNLIPRP3	11120
GL173495.1	147844	148217	148144, 148146, 148175	6, 10, 19	5.96715, 9.39128, 25.11547	4.64927, 6.35644, 13.59366	3.52158, 6	3	hspa12a	428
GL173495.1	180379	181085	180811, 180772, 180948	9, 15, 12	8.66407, 12.35615, 12.50469	6.04743, 7.15789, 7.88834	5.99101, 9	3	eno4	13609
GL173495.1	251250	252004	251535, 251411, 251727	23, 29, 22	29.33336, 32.77739, 29.24652	14.98133, 14.96054, 15.17695	25.84217,	3	shtn1	0
GL173495.1	266914	267820	267274, 267221, 267399	13, 25, 19	13.49269, 25.26956, 20.70442	8.29387, 11.95039, 11.14035	10.53271,	3	shtn1	0
GL173495.1	283611	284684	283985, 284142, 284142	11, 25, 19	11.6377, 24.29926, 23.01756	7.53109, 11.37153, 12.47022	8.77589, 2	3	shtn1	0
GL173495.1	314014	314498	314302, 314319, 314336	7, 15, 11	5.02357, 10.47071, 10.52088	4.02214, 6.01219, 6.8818	2.70132, 7	3	shtn1	22580
GL173495.1	326481	327304	326892, 327010, 327019	28, 31, 23	28.44258, 30.27201, 24.49362	12.75966, 12.71642, 12.17036	24.97166,	3	shtn1	35047
GL173496.1	46693	47178	46748, 46758, 46747	10, 17, 11	10.6018, 18.56115, 14.68355	7.05583, 10.40145, 9.06344	7.80484, 1	3	NA	NA
GL173497.1	63168	64000	63520, 63540, 63545	7, 8, 6	7.1696, 6.4822, 6.08001	5.31345, 4.83755, 4.71063	4.61046, 4	3	ar	0
GL173497.1	129384	129935	129478, 129711, 129779	8, 27, 6	7.42892, 25.58706, 6.76586	5.41453, 11.43661, 5.06179	4.85345, 2	3	ophn1	0
GL173497.1	206004	206735	206181, 206320, 206226	7, 15, 8	5.65516, 13.37045, 9.92329	4.42382, 7.78062, 6.7228	3.27228, 1	3	ophn1	0
GL173497.1	333269	334444	333920, 333662, 334016	13, 28, 9	13.49269, 28.55622, 10.42289	8.29387, 12.82701, 7.00726	10.53271,	3	NA	62741
GL173498.1	2898	3412	3373, 3363, 3384	120, 205, 133	155.58171, 264.15204, 179.07823	35.41557, 40.34237, 39.74965	150.82523	3	gdap2	0
GL173498.1	100087	100666	100224, 100414, 100274	6, 15, 9	5.72604, 14.39783, 10.49552	4.51498, 8.40758, 7.0436	3.33547, 1	3	STXBP5L	0

GL173498.1	205005	205655	205379, 205435, 205457	6, 7, 10	5.96715, 5.97725, 10.97191	4.64927, 4.62287, 7.25854	3.52158, 3	3	polq	0
GL173498.1	232496	232900	232781, 232501, 232743	5, 15, 7	4.73273, 14.03607, 7.01416	3.93547, 8.18766, 5.22813	2.49377, 1	3	usf3	0
GL173498.1	244038	244800	244358, 244332, 244158	10, 8, 5	9.661, 5.93025, 5.40789	6.51661, 4.48816, 4.29296	6.9283, 3.6	3	usf3	0
GL173498.1	257874	258553	258161, 258208, 258294	9, 9, 6	6.06453, 5.92075, 7.18591	4.43343, 4.34131, 5.25776	3.6122, 3.6	3	usf3	0
GL173500.1	17218	18105	17655, 17713, 17671	11, 31, 20	10.72304, 29.26997, 25.96589	7.00201, 12.14098, 13.85721	7.91902, 2	3	pacsin3	35283
GL173500.1	27191	27508	27424, 27414, 27413	10, 27, 9	11.06011, 27.75806, 11.30431	7.306, 12.72023, 7.42891	8.22712, 2	3	pacsin3	25880
GL173500.1	52919	53190	53088, 53092, 53074	17, 27, 15	14.74586, 25.72383, 15.61614	8.10377, 11.51676, 9.13715	11.72534,	3	pacsin3	198
GL173500.1	103016	103313	103281, 103287, 103200	20, 36, 22	21.44017, 41.72793, 28.83653	11.29037, 17.41549, 14.95889	18.16397,	3	lrp4	11443
GL173500.1	203147	203747	203619, 203617, 203485	10, 22, 9	5.90228, 13.66632, 7.65011	4.17618, 6.24394, 5.43639	3.49736, 1	3	c11orf49	0
GL173500.1	206449	206922	206741, 206741, 206758	27, 54, 15	24.13746, 50.88626, 14.65165	10.59569, 15.03547, 8.56113	20.78004,	3	c11orf49	0
GL173500.1	245141	245595	245163, 245468, 245164	10, 8, 9	10.89158, 6.73981, 10.49552	7.21503, 4.99774, 7.0436	8.0782, 4.3	3	NA	0
GL173500.1	286770	287215	286993, 286900, 287011	8, 12, 8	4.91051, 6.88989, 6.90087	3.82948, 4.45688, 5.09678	2.59605, 4	3	arfgap2	30188
GL173500.1	324031	325333	324922, 324683, 324679	24, 32, 30	21.32183, 31.92171, 33.75388	9.94595, 13.29383, 15.15418	18.0503, 2	3	arfgap2	67449
GL173500.1	326314	326570	326426, 326404, 326532	10, 13, 8	6.22053, 9.14598, 5.79199	4.37622, 5.63981, 4.3996	3.75715, 6	3	arfgap2	69732
GL173500.1	333164	333801	333394, 333507, 333460	11, 22, 10	9.13339, 21.49375, 10.59656	6.03321, 10.69716, 7.05292	6.43178, 1	3	arfgap2	76582
GL173501.1	231635	232370	231866, 232156, 232195	21, 35, 12	19.52176, 33.37403, 11.81848	9.8285, 12.98696, 7.4869	16.3092, 2	3	NA	NA
GL173501.1	311746	312802	312147, 312493, 312351	19, 43, 16	20.74745, 49.31736, 19.65507	11.16575, 18.75496, 11.21774	17.49384,	3	NA	NA
GL173501.1	325915	326673	326050, 326478, 326054	15, 15, 6	15.41679, 14.84826, 7.25145	9.0192, 8.67957, 5.287	12.36344,	3	NA	NA
GL173502.1	232694	233660	233089, 233066, 233055	8, 11, 6	7.67464, 9.39347, 7.25145	5.55832, 6.19476, 5.287	5.08299, 6	3	GRIK2	0
GL173502.1	313133	313452	313372, 313302, 313413	6, 7, 4	5.81608, 5.97725, 4.67325	4.56563, 4.62287, 3.78987	3.4176, 3.6	3	GRIK2	0
GL173503.1	150321	150996	150741, 150572, 150606	7, 14, 8	7.1696, 14.45897, 10.0913	5.31345, 8.66788, 6.79758	4.61046, 1	3	NA	5684
GL173503.1	158346	158831	158637, 158670, 158418	8, 13, 9	8.42257, 13.14514, 11.58206	5.97763, 8.09002, 7.55286	5.76336, 1	3	NA	13709
GL173505.1	54632	55069	54689, 54670, 54735	5, 6, 5	4.82177, 4.68027, 5.80393	3.98509, 3.88713, 4.48186	2.51418, 2	3	eaf2	9784
GL173505.1	86848	87222	87022, 87018, 87110	7, 8, 5	7.1696, 6.51779, 5.03069	5.31345, 4.8598, 4.09861	4.61046, 4	3	eaf2	16317
GL173505.1	141929	142380	142068, 142076, 142048	4, 7, 5	3.63646, 5.13485, 4.70718	3.26116, 4.09383, 3.9211	1.6222, 2.9	3	eaf2	71398
GL173505.1	171110	171494	171445, 171454, 171445	17, 43, 23	19.10743, 46.40031, 31.67201	10.71816, 17.0803, 16.23115	15.90925,	3	vipr1	63840
GL173505.1	228053	228439	228197, 228235, 228209	5, 11, 7	4.5799, 9.3497, 6.25413	3.84863, 6.16764, 4.79015	2.3629, 6.7	3	vipr1	6895
GL173505.1	290554	291211	291158, 291184, 291181	88, 94, 62	108.00981, 112.88451, 81.21793	28.48748, 27.92116, 27.70554	103.5545,	3	vipr1	13841
GL173505.1	336352	336709	336669, 336704, 336673	19, 32, 16	24.51879, 41.74651, 23.11042	13.28363, 19.06933, 12.88555	21.15031,	3	vipr1	59639
GL173507.1	47494	48061	47711, 48000, 47668	6, 14, 9	5.96715, 13.44539, 10.14484	4.64927, 8.06258, 6.86555	3.52158, 1	3	NA	7022
GL173507.1	119662	120133	119875, 119909, 119809	9, 9, 7	9.00096, 7.79702, 7.84208	6.24222, 5.5269, 5.66426	6.30808, 5	3	NA	24537
GL173507.1	134830	135203	134971, 134895, 134996	6, 7, 4	5.72604, 5.68499, 4.67325	4.51498, 4.44244, 3.78987	3.33547, 3	3	NA	39705
GL173507.1	140348	141012	140459, 140462, 140630	14, 11, 10	12.97522, 8.30667, 9.93124	7.77689, 5.51556, 6.67475	10.03988,	3	NA	45223
GL173507.1	160115	160811	160588, 160492, 160436	13, 12, 13	11.55805, 9.74388, 13.04158	7.13125, 6.2187, 8.02877	8.70034, 7	3	U6	41174
GL173508.1	23688	24224	23799, 23820, 23694	9, 13, 5	7.20928, 9.37163, 4.67395	5.16158, 5.77863, 3.90232	4.64652, 6	3	NA	1117
GL173508.1	27625	28083	28027, 27983, 27971	13, 22, 11	13.86771, 23.19276, 14.03502	8.51067, 11.71868, 8.77076	10.88502,	3	NA	819
GL173508.1	80549	80823	80627, 80617, 80628	40, 57, 25	43.38131, 57.75111, 29.52458	16.65322, 17.26272, 14.48246	39.62551,	3	rcan1	2659
GL173508.1	127731	128597	128049, 127928, 128338	10, 15, 7	11.06011, 15.80067, 8.69489	7.306, 9.24574, 6.06379	8.22712, 1	3	kcne1	29699
GL173508.1	192358	192702	192481, 192495, 192361	14, 23, 9	13.20712, 21.38155, 8.88966	7.91811, 10.30482, 6.17838	10.26077,	3	kcne1	32451
GL173508.1	287756	288087	287782, 287772, 287765	10, 6, 3	11.06011, 4.93312, 3.47371	7.306, 4.04501, 3.03189	8.22712, 2	3	mrps6	0
GL173510.1	163679	164587	163984, 164241, 164527	10, 10, 4	8.48936, 8.12616, 4.30163	5.80661, 5.58036, 3.61556	5.82603, 5	3	atf5.2	1468
GL173510.1	300612	301030	300877, 300917, 300957	5, 10, 7	4.82177, 8.57382, 8.69489	3.98509, 5.8589, 6.06379	2.51418, 6	3	NA	9115
GL173512.1	8664	8985	8724, 8708, 8687	8, 7, 6	7.42892, 5.78791, 7.29403	5.41453, 4.5064, 5.30581	4.85345, 3	3	ddx11	0
GL173512.1	13610	14281	13860, 14114, 14015	8, 8, 6	8.05102, 6.70176, 7.05929	5.77295, 4.97421, 5.20024	5.43473, 4	3	ddx11	0
GL173512.1	59372	59775	59507, 59621, 59506	17, 28, 23	17.88146, 27.46787, 27.18864	10.00115, 12.18424, 13.7636	14.72916,	3	washc1	2357
GL173512.1	63018	63564	63537, 63403, 63391	9, 16, 8	7.39636, 12.13574, 6.53931	5.27874, 6.77854, 4.87324	4.82212, 9	3	washc1	0
GL173512.1	75497	76251	76187, 76161, 76191	16, 25, 12	18.14851, 30.45643, 14.96175	10.3887, 15.02432, 9.20442	14.98517,	3	washc1	719
GL173512.1	189935	190245	189963, 189937, 189965	25, 40, 24	25.98133, 42.27808, 26.72921	12.3763, 16.0217, 13.16922	22.57216,	3	slc6a1	2233
GL173512.1	217295	217812	217757, 217770, 217763	11, 9, 5	11.02968, 7.63127, 5.9506	7.18222, 5.42473, 4.54784	8.20839, 5	3	slc6a12	0
GL173512.1	233146	233413	233178, 233181, 233188	15, 17, 7	13.36183, 12.70601, 5.98393	7.77533, 6.87373, 4.62695	10.40776,	3	slc6a12	3795
GL173512.1	264035	264394	264342, 264364, 264326	36, 57, 47	43.19982, 67.19026, 60.04174	18.28614, 22.29376, 23.13147	39.4477, 6	3	slc6a13	9593
GL173512.1	270708	271544	271148, 271353, 271060	7, 11, 9	6.50909, 9.8562, 9.82249	4.94073, 6.47973, 6.69629	4.02371, 7	3	kdm5a	9755
GL173512.1	275484	275731	275689, 275727, 275636	8, 9, 6	8.42257, 8.21099, 6.35308	5.97763, 5.77858, 4.85498	5.76336, 5	3	kdm5a	5568
GL173513.1	6091	6736	6592, 6509, 6619	25, 19, 19	28.4518, 18.34606, 23.01756	13.85048, 9.72578, 12.47022	24.98054,	3	NA	2500
GL173513.1	221676	222210	221702, 221739, 221689	13, 25, 7	13.49269, 27.93067, 7.84208	8.29387, 13.54108, 5.66426	10.53271,	3	scnn1a	0
GL173513.1	308115	309015	308117, 308117, 308192	13, 26, 11	14.43911, 28.11231, 14.75772	8.83395, 13.29504, 9.09568	11.43182,	3	NA	4233
GL173514.1	46599	47033	46658, 46630, 46637	10, 11, 9	8.28204, 8.89461, 10.56949	5.67774, 5.88422, 7.08032	5.64968, 6	3	a4gnt	4367
GL173514.1	156792	157634	157538, 157413, 157224	5, 8, 6	4.82177, 6.97779, 6.30561	3.98509, 5.14375, 4.83031	2.51418, 4	3	XB-GENE-969944	5878
GL173514.1	176225	176615	176451, 176411, 176422	16, 24, 13	18.05293, 24.5441, 15.52427	10.33439, 11.86236, 9.42125	14.8942, 2	3	XB-GENE-969944	817
GL173514.1	197150	197425	197395, 197302, 197306	18, 16, 16	18.68415, 14.35819, 18.1835	10.2151, 8.13215, 10.40854	15.49996,	3	NA	7594
GL173514.1	274992	275266	275228, 275189, 275109	9, 15, 6	9.72082, 14.58692, 7.29403	6.64182, 8.52203, 5.30581	6.97155, 1	3	NA	6595
GL173515.1	20709	22039	21405, 21429, 21531	12, 7, 5	12.45843, 5.55323, 5.85847	7.86167, 4.35994, 4.50665	9.54648, 3	3	NA	40391
GL173515.1	52842	53434	52871, 52861, 52865	29, 38, 28	34.75011, 39.62702, 36.96613	16.1291, 15.34231, 17.75574	31.14222,	3	NA	8996
GL173515.1	203484	204090	203511, 203486, 203531	44, 68, 30	52.69127, 80.8812, 36.6241	20.25943, 24.36745, 16.85279	48.80268,	3	stat3.1	5704
GL173515.1	276523	276852	276790, 276821, 276809	23, 24, 19	30.56965, 28.90463, 26.10028	15.65359, 14.44646, 14.0872	27.04952,	3	stat3.1	16171
GL173515.1	314595	315588	314674, 314691, 314695	11, 30, 10	12.33515, 33.93966, 12.42918	7.91559, 15.26467, 7.99682	9.43699, 3	3	stat3.1	54243
GL173515.1	322084	322448	322335, 322323, 322344	7, 14, 8	7.1696, 13.38467, 10.1465	5.31345, 8.02582, 6.82176	4.61046, 1	3	stat3.1	61732
GL173516.1	8744	9151	8984, 8929, 8955	11, 11, 6	9.34791, 7.94428, 5.71639	6.16652, 5.2878, 4.50951	6.63394, 5	3	CD79A	10114
GL173516.1	206907	207670	207313, 207349, 207223	5, 18, 5	4.54329, 16.40493, 5.06966	3.82752, 8.83679, 4.11933	2.33, 13.49	3	ARHGEF1	0
GL173517.1	50704	51443	50928, 51170, 51049	8, 9, 5	8.05102, 7.10358, 4.45433	5.77295, 5.09508, 3.77574	5.43473, 4	3	fbln2	31369
GL173517.1	125922	127038	126141, 126461, 126537	7, 9, 6	6.89193, 7.13885, 6.5517	5.15998, 5.11729, 4.95623	4.37772, 4	3	rpl29	14127
GL173517.1	134547	135204	134888, 135021, 134650	4, 6, 4	3.74195, 4.93312, 4.34116	3.32091, 4.04501, 3.63492	1.6222, 2.7	3	rpl29	22752
GL173517.1	135950	136871	136505, 136416, 136458	8, 7, 6	8.34715, 5.97725, 7.12188	5.9367, 4.62287, 5.22884	5.71011, 3	3	rpl29	24155
GL173517.1	231368	232986	232274, 232275, 232077	7, 20, 14	5.93788, 16.12055, 13.28155	4.59878, 8.0984, 7.96331	3.52158, 1	3	alas1	4146
GL173518.1	90300	90781	90701, 90622, 90635	15, 14, 11	16.90423, 14.45897, 14.24187	9.88146, 8.66788, 8.86619	13.78827,	3	NA	10387
GL173518.1	110075	110656	110407, 110460, 110434	10, 15, 8	8.84451, 15.12096, 8.14528	6.02544, 8.84304, 5.82558	6.16137, 1	3	pld2	0
GL173518.1	134476	134932	134861, 134904, 134719	11, 19, 6	12.43693, 19.88391, 7.29403	7.97018, 10.65254, 5.30581	9.52692, 1	3	NA	4230
GL173518.1	150181	151268	150886, 150980, 150663	7, 7, 6	5.39768, 5.97725, 5.39683	4.26169, 4.62287, 4.32487	3.04019, 3	3	NA	4592
GL173518.1	256124	256857	256613, 256614, 256641	73, 77, 67	81.44082, 84.14503, 84.18289	22.27589, 21.83147, 26.70469	77.23116,	3	U2	0
GL173518.1	272793	273071	272863, 272798, 272876	7, 16, 4	7.1696, 16.01323, 4.67325	5.31345, 9.13759, 3.78987	4.61046, 1	3	NA	2525
GL173518.1	283316	283932	283742, 283588, 283722	21, 22, 8	20.17379, 20.43157, 8.51364	10.2212, 10.05943, 6.02666	16.93778,	3	NA	3493
GL173518.1	291120	292404	291708, 291836, 291319	7, 9, 8	6.33443, 7.71322, 8.35103	4.83795, 5.47534, 5.93881	3.86259, 5	3	NA	2764
GL173518.1	291120	292478	292363, 291836, 291319	5, 9, 8	4.20732, 7.71322, 8.35103	3.62846, 5.47534, 5.93881	2.04024, 5	3	NA	2764
GL173519.1	3527	4094	3753, 3762, 3745	10, 5, 8	9.44155, 3.9118, 9.11678	6.38646, 3.44594, 6.33924	6.72156, 1	3	ddx19b	3540
GL173519.1	8069	8936	8316, 8336, 8130	12, 19, 6	11.85767, 20.38412, 6.5517	7.51014, 10.95068, 4.95623	8.98544, 1	3	ddx19b	0
GL173519.1	42296	43080	42377, 42349, 42352	12, 9, 8	13.82915, 8.21099, 10.0913	8.62415, 5.77858, 6.79758	10.85461,	3	otud3	2218
GL173519.1	99538	99901	99851, 99839, 99871	14, 13, 15	14.16942, 12.03475, 17.15492	8.49669, 7.4231, 10.0222	11.17387,	3	XB-GENE-5903348	0
GL173519.1	110081	110425	110367, 110348, 110359	12, 23, 13	13.73771, 26.22929, 17.924	8.57523, 13.2024, 10.57401	10.76705,	3	HTR6	0
GL173519.1	147679	148283	147922, 148125, 148125	9, 11, 8	7.80169, 10.05535, 9.60885	5.52977, 6.6012, 6.57807	5.20141, 7	3	NA	0
GL173519.1	154993	155719	155570, 155044, 155684	7, 10, 4	5.65516, 8.98028, 3.91395	4.42382, 6.10835, 3.41551	3.27228, 6	3	NA	0
GL173519.1	166482	167213	166897, 166950, 166575	9, 7, 6	7.80169, 5.71889, 6.25896	5.52977, 4.46356, 4.80589	5.20141, 3	3	nbl1	0
GL173519.1	199061	199725	199340, 199295, 199305	14, 24, 12	12.59053, 22.59107, 12.7555	7.54151, 10.69506, 8.03189	9.67379, 1	3	capzb	0
GL173520.1	35509	35764	35629, 35625, 35628	13, 15, 9	13.27928, 14.15433, 10.28145	8.16901, 8.25968, 6.93568	10.32936,	3	zbtb34	16491
GL173520.1	101476	102219	101689, 101700, 102061	7, 12, 6	6.50909, 10.63474, 7.29403	4.94073, 6.77054, 5.30581	4.02371, 7	3	ralgps1	0
GL173520.1	217785	218152	217827, 217906, 217850	16, 11, 6	17.68339, 9.8562, 6.2131	10.1227, 6.47973, 4.78171	14.5382, 7	3	ralgps1	5804
GL173520.1	286879	287874	287286, 286895, 287267	16, 18, 9	17.95866, 16.12237, 10.28145	10.28064, 8.66512, 6.93568	14.80292,	3	NA	13845
GL173522.1	3099	3327	3125, 3153, 3160	20, 21, 9	25.10871, 24.01007, 11.58206	13.39632, 12.51268, 7.55286	21.72465,	3	pou5f3.1	20060
GL173522.1	10274	11291	10322, 10675, 10665	7, 13, 9	7.1696, 13.06145, 11.64359	5.31345, 8.04054, 7.57973	4.61046, 1	3	pou5f3.1	12096
GL173522.1	27819	28200	27853, 27860, 27857	10, 11, 8	9.03421, 8.74006, 8.6841	6.14116, 5.78751, 6.11715	6.33915, 6	3	pou5f3.1	894
GL173522.1	43742	44167	43958, 44049, 43780	6, 10, 6	4.89903, 8.04981, 5.11235	4.02393, 5.53254, 4.15475	2.58474, 5	3	NA	198
GL173522.1	80376	81237	80535, 80887, 80877	6, 10, 9	5.47306, 7.48641, 10.56949	4.36955, 5.17758, 7.08032	3.10811, 5	3	abca2	0
GL173522.1	107234	107638	107259, 107430, 107509	16, 13, 5	15.67432, 9.57351, 4.84526	8.9333, 5.90318, 3.99806	12.61068,	3	clic3	284
GL173522.1	134501	135210	134831, 135093, 134860	11, 23, 6	12.43693, 23.5013, 6.76586	7.97018, 11.57503, 5.06179	9.52692, 2	3	trub2	15886
GL173522.1	146467	146815	146596, 146596, 146613	13, 18, 10	13.27928, 18.91825, 10.19609	8.16901, 10.35485, 6.82728	10.32936,	3	trub2	4281
GL173522.1	157130	157378	157345, 157328, 157288	28, 36, 16	35.94532, 46.39141, 21.65777	17.18016, 20.1647, 12.22781	32.31287,	3	trub2	0
GL173522.1	174762	175709	174991, 175080, 175113	11, 17, 5	9.81032, 16.43386, 5.10931	6.45164, 9.1315, 4.14026	7.05644, 1	3	edf1	0
GL173522.1	194835	195231	194862, 194851, 194843	16, 26, 12	16.60441, 27.7452, 13.59027	9.49088, 13.07563, 8.49571	13.50404,	3	DPH7	0
GL173522.1	223395	224068	223410, 223427, 223408	23, 43, 17	24.94963, 46.9058, 21.09096	12.44303, 17.36748, 11.82018	21.56948,	3	pnpla6	0
GL173522.1	239099	240220	239758, 239600, 239769	7, 25, 7	7.1696, 25.75015, 6.36213	5.31345, 12.23791, 4.85436	4.61046, 2	3	pnpla6	0
GL173522.1	251704	252184	252045, 252140, 252092	7, 13, 12	7.1696, 13.14514, 16.36823	5.31345, 8.09002, 9.85365	4.61046, 1	3	pnpla6	4657
GL173522.1	281542	282148	281920, 281949, 281866	114, 128, 82	124.18346, 133.97845, 93.00269	24.25667, 22.96415, 23.89046	119.60551	3	nsmf	801
GL173522.1	288708	289206	289103, 289134, 289159	9, 24, 14	8.25643, 26.00533, 15.64845	5.80588, 12.73769, 9.35267	5.62516, 2	3	nsmf	7967
GL173522.1	313197	313478	313408, 313445, 313426	12, 15, 13	13.47263, 15.80067, 15.0131	8.43173, 9.24574, 9.14923	10.51396,	3	entpd8	0
GL173522.1	327444	328336	328044, 327940, 328005	9, 12, 5	9.70623, 11.84549, 5.9506	6.63396, 7.50292, 4.54784	6.97147, 9	3	noxa1	88
GL173523.1	4972	5828	5250, 5449, 5454	6, 15, 5	5.51356, 14.33606, 5.9506	4.39314, 8.37011, 4.54784	3.14574, 1	3	NA	32172
GL173523.1	27832	28642	28233, 28026, 28286	8, 12, 9	6.70593, 9.0625, 9.41119	4.9768, 5.7937, 6.47287	4.20487, 6	3	NA	9358
GL173523.1	48653	50059	49159, 49314, 49847, 49780	13, 7, 6, 13	12.20544, 5.2482, 4.45754, 13.90715	7.52692, 4.16649, 3.74538, 8.53326	9.31472, 3	3	NA	7147
GL173523.1	65038	65697	65153, 65282, 65620	10, 14, 8	8.93828, 9.69817, 8.24665	6.08275, 5.76563, 5.88165	6.24946, 7	3	NA	0
GL173523.1	79455	79812	79633, 79554, 79630	6, 5, 3	5.96715, 3.94561, 3.47371	4.64927, 3.46715, 3.03189	3.52158, 1	3	NA	8703
GL173523.1	88036	88927	88557, 88567, 88564	16, 24, 8	17.41877, 24.5441, 9.92329	9.96955, 11.86236, 6.7228	14.28292,	3	NA	216
GL173523.1	153867	154385	153959, 153920, 153872	19, 9, 4	24.51879, 8.21099, 4.67325	13.28363, 5.77858, 3.78987	21.15031,	3	NA	5768
GL173523.1	242105	242462	242175, 242457, 242215	5, 9, 5	4.82177, 8.21099, 5.9506	3.98509, 5.77858, 4.54784	2.51418, 5	3	NA	1233
GL173523.1	255981	256324	256243, 256049, 256234	8, 12, 4	7.33538, 11.20359, 4.22496	5.35908, 7.11794, 3.57747	4.76518, 8	3	NA	6360
GL173523.1	282916	283291	283079, 283073, 283081	6, 9, 5	5.96715, 8.21099, 5.3628	4.64927, 5.77858, 4.27046	3.52158, 5	3	rnf121	7113
GL173523.1	286655	287016	286908, 286679, 286832	8, 6, 8	7.99469, 4.68027, 8.86315	5.74128, 3.88713, 6.21039	5.38155, 2	3	rnf121	3388
GL173525.1	285966	287276	286888, 286410, 286287	30, 33, 11	28.54149, 27.87558, 9.51925	12.0849, 10.68868, 6.27256	25.068, 24	3	ftsj3	16128
GL173525.1	296430	297407	296945, 296690, 296737	13, 13, 13	15.29169, 13.14514, 18.01082	9.29854, 8.09002, 10.61163	12.24396,	3	ftsj3	26592
GL173525.1	302752	303926	303547, 303578, 303540	20, 30, 17	24.33651, 36.17136, 21.64669	12.96942, 16.5867, 12.11304	20.9737, 3	3	ftsj3	32914
GL173525.1	310789	312512	312177, 311975, 312100	31, 46, 19	32.24754, 43.38729, 18.90983	13.8697, 14.24153, 10.06679	28.69044,	3	ftsj3	40951
GL173526.1	107	506	258, 268, 352	8, 7, 4	8.42257, 5.8946, 4.67325	5.97763, 4.57222, 3.78987	5.76336, 3	3	ankrd50	56591
GL173527.1	22225	22717	22605, 22470, 22430	8, 7, 7	7.02691, 5.05277, 7.96278	5.17362, 4.04098, 5.72394	4.50222, 2	3	PTN	3929
GL173527.1	60182	60721	60377, 60331, 60230	14, 27, 15	14.16942, 28.88794, 16.75372	8.49669, 13.39413, 9.79627	11.17387,	3	NA	16076
GL173527.1	111128	111598	111459, 111223, 111220	6, 7, 5	5.96715, 5.97725, 5.9506	4.64927, 4.62287, 4.54784	3.52158, 3	3	NA	0
GL173527.1	157793	158688	158293, 158326, 158301	17, 14, 7	17.19112, 11.20037, 7.84208	9.58879, 6.68504, 5.66426	14.0641, 8	3	NA	30832
GL173527.1	166731	166985	166890, 166931, 166957	7, 11, 6	7.1696, 10.15827, 7.29403	5.31345, 6.66366, 5.30581	4.61046, 7	3	CREB3L2	23359
GL173527.1	208571	209103	208582, 208877, 208890	14, 23, 12	13.76536, 22.03663, 12.15007	8.2554, 10.69607, 7.68238	10.79347,	3	CREB3L2	11658
GL173527.1	220449	221071	220997, 220935, 221021	19, 30, 25	21.91879, 34.37792, 31.77551	11.84839, 15.52518, 15.77668	18.62754,	3	ssbp1	12581
GL173527.1	252880	253237	253046, 253141, 252918	5, 8, 4	4.82177, 7.07159, 4.67325	3.98509, 5.20073, 3.78987	2.51418, 4	3	wee2	0
GL173527.1	287516	288037	287979, 287709, 287640	7, 6, 6	7.1696, 4.89312, 7.29403	5.31345, 4.02027, 5.30581	4.61046, 2	3	agk	0
GL173527.1	307169	308238	308028, 308033, 307934	9, 9, 9	9.72082, 8.21099, 11.64359	6.64182, 5.77858, 7.57973	6.97155, 5	3	agk	9224
GL173527.1	313776	315233	314508, 314523, 314639	25, 29, 13	26.15197, 28.88285, 12.98465	12.47847, 12.64966, 7.995	22.73773,	3	agk	15831
GL173528.1	29823	30212	30191, 30184, 30182	32, 33, 17	36.24469, 34.47113, 20.18522	15.83805, 14.38797, 11.32722	32.60691,	3	rab11fip2	47600
GL173528.1	71830	72433	72201, 72157, 72325	12, 10, 10	10.99193, 8.88645, 11.45478	6.98928, 6.0511, 7.51412	8.17226, 6	3	rab11fip2	5379
GL173528.1	175058	175566	175275, 175331, 175325	7, 13, 7	6.60038, 11.26714, 8.35502	4.99378, 6.95205, 5.9108	4.10761, 8	3	rab11fip2	68402
GL173528.1	184796	185075	185042, 185024, 185048	24, 35, 33	26.57606, 37.97766, 41.92324	13.07811, 15.62873, 18.7845	23.15164,	3	rab11fip2	78140
GL173528.1	203654	203965	203716, 203749, 203709	16, 17, 6	16.91852, 16.17826, 7.05929	9.67673, 8.97632, 5.20024	13.80145,	3	rab11fip2	96998
GL173528.1	234245	235032	234548, 234471, 234490	14, 18, 13	13.20712, 14.2312, 13.45746	7.91811, 7.5249, 8.27333	10.26077,	3	rab11fip2	127589
GL173528.1	267275	267723	267506, 267503, 267585	19, 22, 7	22.68927, 21.21624, 7.55909	12.28657, 10.53027, 5.52035	19.37453,	3	rab11fip2	160619
GL173529.1	14	430	119, 106, 112	42, 55, 36	46.52257, 58.7238, 43.65414	17.58845, 18.60125, 18.55479	42.72088,	3	smarcal1	3650
GL173529.1	52907	53674	53085, 52971, 52970	10, 15, 6	9.08301, 12.86621, 6.71074	6.17079, 7.47111, 5.03498	6.3862, 10	3	4-Mar	0
GL173529.1	80179	80746	80503, 80520, 80485	22, 33, 13	23.18766, 37.31986, 15.70541	11.71562, 16.06786, 9.51556	19.85731,	3	XRCC5	0
GL173529.1	94617	95137	94823, 94767, 94907	6, 8, 5	4.80449, 6.37769, 4.91762	3.96512, 4.77199, 4.03768	2.51418, 4	3	XRCC5	451
GL173529.1	104347	105019	104737, 104737, 104800	20, 21, 15	19.56371, 19.23811, 16.44983	10.16265, 9.65805, 9.62273	16.34986,	3	tmem169	0
GL173529.1	157595	158355	157596, 157697, 157768	8, 24, 9	8.42257, 25.12019, 10.87975	5.97763, 12.2078, 7.23113	5.76336, 2	3	map3k2	0
GL173529.1	178677	179198	178704, 178793, 178822	25, 42, 16	27.72746, 45.14985, 19.45311	13.42011, 16.80325, 11.10978	24.27465,	3	bin1	1991
GL173529.1	240628	241343	241286, 241262, 241292	33, 27, 12	41.64833, 29.6442, 15.16114	18.6241, 13.84552, 9.30141	37.91969,	3	cyp27c1	20392
GL173530.1	84427	84937	84772, 84674, 84716	20, 26, 12	22.97335, 32.02458, 14.23156	12.19234, 15.60218, 8.83588	19.64963,	3	NA	46661
GL173530.1	114376	114896	114740, 114874, 114754	14, 32, 7	16.7649, 40.45961, 8.69489	9.96273, 18.32506, 6.06379	13.6543, 3	3	NA	16702
GL173530.1	172539	173364	172820, 172831, 172872	16, 25, 18	16.01695, 24.6617, 19.75651	9.13983, 11.58741, 10.85036	12.9408, 2	3	NA	5620
GL173531.1	118648	119094	119046, 118999, 118967	12, 9, 3	13.73771, 8.21099, 3.47371	8.57523, 5.77858, 3.03189	10.76705,	3	mhc2a	13593
GL173532.1	22275	23398	22693, 22733, 22797, 23319	17, 31, 11, 15	11.31646, 19.93951, 6.9177, 10.95218	6.06187, 7.31657, 4.64542, 6.30168	8.47078, 1	3	NA	4025
GL173532.1	283186	283763	283610, 283566, 283641	8, 12, 3	8.42257, 11.86108, 3.47371	5.97763, 7.51216, 3.03189	5.76336, 9	3	NA	125072
GL173533.1	5574	6018	5622, 5613, 5601	7, 9, 6	7.04884, 7.97047, 5.60548	5.2473, 5.63299, 4.44624	4.52221, 5	3	kcnk16	151582
GL173533.1	19852	20677	20161, 20265, 20286	7, 8, 9	5.97522, 5.51195, 9.19414	4.62162, 4.21928, 6.35176	3.52869, 3	3	kcnk16	136923
GL173533.1	30942	31266	31127, 31124, 31083	8, 14, 7	7.38183, 13.63145, 7.29952	5.38666, 8.17488, 5.38357	4.80856, 1	3	kcnk16	126334
GL173533.1	52881	53161	53102, 53097, 53119	21, 17, 11	23.81985, 15.69342, 13.46208	12.40141, 8.68127, 8.49639	20.47156,	3	kcnk16	104439
GL173533.1	101924	102436	102279, 102242, 102096	9, 23, 13	9.18105, 22.17331, 15.70541	6.3444, 10.77791, 9.51556	6.47733, 1	3	kcnk16	55164
GL173533.1	140566	140879	140568, 140664, 140568	10, 14, 6	8.12348, 10.54152, 5.53406	5.57869, 6.27972, 4.40503	5.50152, 7	3	kcnk16	16721
GL173533.1	141474	141974	141923, 141904, 141853	11, 16, 10	9.26086, 12.77959, 10.1418	6.1125, 7.16814, 6.79622	6.5517, 10	3	kcnk16	15626
GL173533.1	192657	193112	192699, 192939, 192841	9, 13, 13	8.0679, 10.23256, 13.45746	5.69219, 6.31126, 8.27333	5.45, 7.61	3	ccdc167	16328
GL173533.1	228033	228405	228059, 228055, 228059	17, 18, 16	19.39948, 17.45593, 17.62687	10.88538, 9.47501, 10.0901	16.19131,	3	ccdc167	51704
GL173533.1	234161	234501	234319, 234295, 234326	9, 12, 8	6.39666, 9.31599, 6.76479	4.646, 5.95189, 5.01316	3.92119, 6	3	ccdc167	57832
GL173533.1	235549	236560	236117, 235720, 236111	20, 16, 18	18.6379, 13.73836, 18.87511	9.60312, 7.753, 10.32913	15.4562, 1	3	ccdc167	59220
GL173533.1	252531	253179	252687, 252787, 252678	6, 13, 4	4.30262, 11.31501, 4.04585	3.64547, 6.98159, 3.48564	2.12436, 8	3	ccdc167	76202
GL173533.1	253690	253946	253896, 253842, 253804	6, 10, 6	4.22823, 8.12616, 6.35308	3.59714, 5.58036, 4.85498	2.0602, 5.6	3	ccdc167	77361
GL173533.1	275680	276069	275853, 275738, 275881	8, 10, 8	7.99469, 9.39128, 8.98772	5.74128, 6.35644, 6.27416	5.38155, 6	3	ccdc167	99351
GL173533.1	283420	283842	283791, 283803, 283788	19, 17, 9	21.71142, 15.99248, 9.46739	11.72888, 8.86336, 6.50386	18.42619,	3	ccdc167	107091
GL173533.1	289766	290358	290130, 290126, 290130	21, 22, 16	25.75943, 23.45091, 18.88025	13.51522, 11.87329, 10.798	22.35693,	3	ccdc167	113437
GL173533.1	297569	297998	297706, 297724, 297726	18, 12, 13	21.75314, 11.86108, 16.0859	11.98894, 7.51216, 9.70995	18.46685,	3	ccdc167	121240
GL173534.1	12032	13042	12547, 12614, 12560	12, 23, 7	11.04522, 22.2424, 7.78342	7.02174, 10.8193, 5.63488	8.22317, 1	3	NA	NA
GL173534.1	123031	123362	123266, 123261, 123289	16, 23, 23	17.2479, 24.50192, 28.5849	9.86999, 12.17534, 14.56309	14.1196, 2	3	NA	NA
GL173534.1	169214	169470	169441, 169415, 169335	9, 11, 7	9.24298, 9.43766, 8.35502	6.3792, 6.22212, 5.9108	6.53474, 6	3	NA	NA
GL173535.1	34548	34946	34897, 34894, 34941	28, 50, 48	30.68278, 57.37417, 65.44716	14.09279, 20.13049, 25.81166	27.1602, 5	3	NA	NA
GL173535.1	168039	168641	168397, 168292, 168133	9, 14, 12	9.12009, 12.54273, 14.3175	6.30997, 7.51218, 8.88032	6.42028, 9	3	NA	NA
GL173535.1	176995	177739	177096, 177091, 177369	17, 22, 3	21.19088, 23.02459, 3.47371	11.87339, 11.61782, 3.03189	17.92307,	3	NA	NA
GL173535.1	303763	304277	304192, 304233, 304157	7, 16, 5	6.55439, 16.90523, 5.9506	4.96711, 9.66889, 4.54784	4.06616, 1	3	NA	NA
GL173536.1	21958	22187	21984, 22014, 22041	8, 7, 7	8.34715, 5.97725, 8.08824	5.9367, 4.62287, 5.7849	5.71011, 3	3	TBCD	11341
GL173536.1	64175	64961	64912, 64934, 64902	19, 41, 15	19.64557, 41.5477, 17.40815	10.50977, 15.19816, 10.16282	16.42918,	3	TBCD	0
GL173536.1	99783	100187	99982, 100144, 99959	10, 6, 3	9.38842, 4.93312, 3.44998	6.35473, 4.04501, 3.02115	6.67167, 2	3	TBCD	0
GL173536.1	117345	119112	117526, 117581, 118763, 118022	11, 9, 22, 15	9.90875, 6.6123, 22.85945, 14.32502	6.51186, 4.78362, 11.51868, 8.36342	7.14919, 4	3	TBCD	198
GL173536.1	117345	119112	118904, 118763, 118022	6, 22, 15	4.77375, 22.85945, 14.32502	3.9459, 11.51868, 8.36342	2.51418, 1	3	TBCD	198
GL173536.1	125165	125636	125584, 125530, 125535	9, 8, 3	9.72082, 7.07159, 3.47371	6.64182, 5.20073, 3.03189	6.97155, 4	3	fn3k	7581
GL173536.1	188271	189111	188726, 188725, 188868	45, 77, 51	38.92263, 66.53319, 55.26075	12.37366, 14.36457, 18.49572	35.23731,	3	NA	737
GL173536.1	203591	203947	203747, 203733, 203694	5, 6, 6	4.36883, 4.15772, 6.5517	3.72533, 3.55114, 4.95623	2.18201, 2	3	kcnk1	1666
GL173539.1	79178	79792	79509, 79351, 79344	12, 16, 9	12.90199, 17.16856, 11.64359	8.11489, 9.82359, 7.57973	9.97033, 1	3	NA	349
GL173539.1	312239	312946	312537, 312658, 312473	16, 36, 8	17.77392, 41.03524, 10.0913	10.17481, 17.00632, 6.79758	14.62581,	3	XB-GENE-5824086	280
GL173540.1	69450	69887	69622, 69699, 69553	9, 6, 5	6.69985, 3.48987, 4.99237	4.83936, 3.10789, 4.0781	4.20005, 1	3	NA	35367
GL173540.1	70450	71089	70637, 70476, 70497	24, 32, 13	25.29738, 34.91565, 15.70541	12.30321, 15.04994, 9.51556	21.89126,	3	NA	34165
GL173540.1	99575	100555	100324, 100280, 100285	26, 27, 22	28.28954, 28.79687, 24.76686	13.4009, 13.33976, 12.65568	24.82295,	3	NA	4699
GL173540.1	121119	121637	121582, 121600, 121589	50, 71, 30	64.87399, 87.54969, 39.15927	24.47699, 26.46188, 18.31952	60.83146,	3	lrfn2	5015
GL173540.1	139221	139803	139368, 139259, 139414	9, 10, 12	8.45482, 8.70492, 13.51536	5.92419, 5.93975, 8.45502	5.79307, 6	3	lrfn2	0
GL173540.1	148022	148255	148182, 148207, 148087	5, 7, 5	4.82177, 5.71889, 5.9506	3.98509, 4.46356, 4.54784	2.51418, 3	3	lrfn2	0
GL173540.1	205859	206214	205863, 205863, 205863	13, 14, 8	11.35857, 10.84175, 7.76694	7.00844, 6.46415, 5.6116	8.51036, 8	3	lrfn2	12613
GL173540.1	206718	207431	207124, 206881, 207382	8, 17, 8	5.40205, 14.17543, 6.63406	4.14825, 7.75699, 4.93224	3.04418, 1	3	lrfn2	13472
GL173540.1	212382	212593	212410, 212430, 212432	43, 64, 31	46.83475, 71.14867, 38.6896	17.32703, 21.11277, 17.67953	43.02902,	3	lrfn2	19136
GL173541.1	50107	51045	50450, 50987, 50902	12, 10, 9	9.6142, 7.29424, 9.30116	6.1379, 5.05593, 6.41174	6.88539, 4	3	NA	28412
GL173541.1	67988	68541	68293, 68337, 68197	23, 28, 6	21.2886, 26.33876, 6.168	10.24946, 11.52425, 4.75778	18.01831,	3	shc1	33841
GL173541.1	86825	89173	87871, 88081, 87791	11, 12, 4	12.17114, 10.68384, 4.67325	7.82679, 6.80072, 3.78987	9.28263, 8	3	shc1	13209
GL173541.1	86825	89173	88783, 88081, 87791	4, 12, 4	3.54306, 10.68384, 4.67325	3.2072, 6.80072, 3.78987	1.57602, 8	3	shc1	13209
GL173541.1	110446	111764	111235, 111263, 110943	13, 21, 11	11.66066, 26.0026, 13.46208	7.19428, 11.68048, 8.49639	8.79778, 2	3	shc1	804
GL173541.1	118049	118460	118203, 118174, 118189	13, 17, 12	12.62236, 17.18862, 12.50469	7.77868, 9.58729, 7.88834	9.70369, 1	3	cks1b	686
GL173541.1	173653	174375	173799, 174184, 174039	11, 9, 7	8.92872, 5.99677, 6.36213	5.90554, 4.39002, 4.85436	6.24102, 3	3	cks1b	51138
GL173541.1	177232	177794	177751, 177630, 177606	29, 38, 20	28.08935, 38.75223, 21.65208	12.18529, 14.8448, 11.41641	24.62775,	3	cks1b	54717
GL173541.1	212310	212592	212551, 212534, 212527	10, 20, 7	11.06011, 21.34919, 8.69489	7.306, 11.23615, 6.06379	8.22712, 1	3	cks1b	89795
GL173541.1	229413	230307	230179, 229879, 229873	16, 21, 11	11.8839, 15.06496, 9.07307	6.6272, 7.22584, 5.99563	9.00954, 1	3	cks1b	106898
GL173541.1	231631	232415	231876, 232050, 232299	17, 22, 13	13.78629, 17.43414, 11.86258	7.52135, 8.29723, 7.31799	10.81362,	3	cks1b	109116
GL173541.1	241341	242457	242322, 242152, 242218, 242218	8, 7, 8, 8	6.20993, 5.48955, 7.99854, 7.99854	4.66614, 4.31982, 5.74345, 5.74345	3.74679, 3	3	cks1b	118826
GL173541.1	253059	253844	253641, 253482, 253281	6, 10, 6	5.24248, 7.68845, 5.793	4.2332, 5.30522, 4.55271	2.89962, 5	3	cks1b	130544
GL173541.1	262783	263667	263194, 263281, 262957	9, 5, 4	8.94274, 3.94561, 4.67325	6.20889, 3.46715, 3.78987	6.2534, 1	3	cks1b	140268
GL173542.1	209	925	548, 434, 487	11, 21, 9	6.23522, 14.2063, 6.88962	4.22478, 6.75342, 4.95986	3.77143, 1	3	NA	115989
GL173542.1	5674	6959	6391, 6367, 6401	19, 19, 16	9.72366, 7.82164, 9.34879	4.8231, 3.92328, 5.15548	6.97434, 5	3	NA	109955
GL173542.1	9367	10360	9602, 9750, 10063	26, 29, 22	19.49497, 19.99326, 18.26691	8.32158, 7.80145, 8.77861	16.2833, 1	3	NA	106554

GL173542.1	16668	17323	17062, 16964, 17077	18, 37, 16	11.22557, 26.07674, 10.98708	5.79449, 8.68842, 6.09457	8.38431, 2	3	NA	99591
GL173542.1	18742	19531	18950, 19382, 19282	17, 23, 12	9.08366, 10.9592, 6.6941	4.83069, 4.77983, 4.33977	6.38684, 8	3	NA	97383
GL173542.1	26607	27497	26784, 26862, 26914	12, 24, 14	7.04352, 16.10046, 11.02224	4.5493, 7.04768, 6.57526	4.51776, 1	3	NA	89417
GL173542.1	34357	35291	35060, 34653, 34653	27, 18, 16	20.08882, 12.27659, 15.42885	8.36376, 6.38339, 8.78469	16.85549,	3	NA	81623
GL173542.1	39063	40096	39515, 39348, 39259	21, 20, 15	13.20712, 12.42539, 12.12091	6.22, 6.02128, 7.01362	10.26077,	3	NA	76818
GL173542.1	46887	49175	48519, 47521, 47233, 48996	13, 26, 16, 10	7.82724, 18.10237, 14.17745, 7.50676	4.84097, 7.59326, 8.02161, 5.19045	5.22612, 1	3	NA	67739
GL173542.1	49383	50883	50292, 49893, 50617, 49770	15, 17, 18, 7	9.10054, 9.51864, 9.88597, 4.64327	5.20735, 5.06217, 5.07586, 3.77487	6.4025, 6.9	3	NA	66031
GL173542.1	55561	57371	56019, 56283, 56159	17, 22, 11	9.10486, 11.06224, 6.83332	4.84187, 4.97761, 4.59305	6.4068, 8.3	3	NA	59543
GL173542.1	55561	57371	57270, 56283, 56159	17, 22, 11	7.2755, 11.06224, 6.83332	3.9175, 4.97761, 4.59305	4.71016, 8	3	NA	59543
GL173542.1	57908	59344	58077, 58926, 58094, 59271	20, 24, 15, 10	9.52366, 11.08507, 8.87657, 4.79347	4.57042, 4.69237, 5.07906, 3.49185	6.79981, 8	3	NA	57570
GL173542.1	60666	61878	61531, 61466, 61661	36, 30, 22	23.5515, 15.42824, 14.77343	7.8185, 5.58629, 6.8227	20.21129,	3	NA	55036
GL173542.1	64701	65717	65136, 64977, 65338	23, 28, 16	11.00759, 14.07639, 7.98294	4.801, 5.34907, 4.41589	8.18782, 1	3	NA	51197
GL173542.1	66867	67642	66925, 67292, 67286	23, 20, 18	11.65734, 9.3443, 10.71419	5.08996, 4.48686, 5.51566	8.79529, 6	3	NA	49272
GL173542.1	70337	71484	71299, 71068, 70814	24, 23, 11	14.50813, 13.9123, 5.9241	6.24786, 6.15929, 4.0357	11.49826,	3	NA	45430
GL173542.1	104212	104673	104244, 104240, 104390	9, 17, 6	8.55802, 15.5773, 6.35308	5.98518, 8.61051, 4.85498	5.8909, 12	3	NA	12241
GL173542.1	179932	180450	180248, 180175, 180073	11, 11, 8	9.34791, 9.8562, 7.76694	6.16652, 6.47973, 5.6116	6.63394, 7	3	NA	12178
GL173543.1	23478	24255	23811, 23990, 23984	59, 96, 24	58.32494, 94.8339, 22.91617	16.75989, 19.22064, 10.88848	54.35769,	3	mybpc3	54214
GL173543.1	49361	49727	49716, 49695, 49667	44, 47, 36	54.0819, 56.66151, 49.85503	21.07542, 21.15122, 22.15445	50.17164,	3	mybpc3	28742
GL173544.1	7518	7990	7535, 7555, 7573	17, 7, 11	18.64535, 5.97725, 14.03502	10.45058, 4.62287, 8.77076	15.46286,	3	xtr-let-7e-2	18206
GL173544.1	54618	55523	55090, 54842, 55100	6, 16, 10	5.96715, 16.37065, 10.71812	4.64927, 9.3517, 7.12015	3.52158, 1	3	mcat	9411
GL173544.1	68515	69124	68578, 68786, 68714	6, 9, 9	5.81608, 7.79702, 8.93877	4.56563, 5.5269, 6.20662	3.4176, 5.3	3	mcat	279
GL173544.1	72216	72648	72383, 72357, 72322	5, 10, 7	4.82177, 8.53104, 6.51247	3.98509, 5.83243, 4.9427	2.51418, 6	3	mcat	3980
GL173544.1	88686	89418	88995, 89111, 89377	23, 29, 10	23.32852, 27.09161, 8.97012	11.47124, 11.60727, 6.10216	19.99469,	3	ttll1	145
GL173544.1	90334	90735	90490, 90415, 90507	7, 10, 10	4.55459, 5.94126, 8.77043	3.7168, 4.20062, 5.98002	2.34094, 3	3	pacsin2	640
GL173544.1	104456	104992	104526, 104566, 104802	9, 7, 5	7.93244, 5.19095, 5.69847	5.60981, 4.12984, 4.4331	5.32435, 2	3	pacsin2	0
GL173544.1	109238	109725	109326, 109301, 109310	9, 12, 5	8.0222, 11.78365, 5.69847	5.66446, 7.46621, 4.4331	5.40727, 9	3	pacsin2	0
GL173544.1	115746	116264	116066, 116001, 116212	9, 6, 6	9.12009, 4.93312, 6.6567	6.30997, 4.04501, 5.00845	6.42028, 2	3	pacsin2	0
GL173544.1	140112	140756	140496, 140299, 140228	11, 7, 5	11.85906, 5.97725, 5.45388	7.65504, 4.62287, 4.3157	8.98595, 3	3	a4galt	8936
GL173544.1	185549	185936	185794, 185792, 185834	35, 39, 10	36.33864, 39.79767, 9.82971	14.6736, 15.03047, 6.61563	32.69907,	3	NA	2947
GL173544.1	187830	188348	187978, 188242, 188268	15, 16, 10	10.84562, 10.27269, 9.68182	6.23737, 5.67895, 6.52889	8.03447, 7	3	NA	535
GL173544.1	266203	266647	266617, 266645, 266590	10, 10, 4	10.39648, 7.79347, 4.64325	6.94096, 5.37143, 3.77643	7.61003, 5	3	phf21b	2579
GL173544.1	285003	285410	285379, 285372, 285362	17, 19, 13	19.20348, 19.33741, 15.79818	10.77332, 10.3246, 9.56343	16.00121,	3	phf21b	7161
GL173544.1	313752	314363	314170, 314062, 314043	5, 10, 6	4.82177, 9.39128, 7.29403	3.98509, 6.35644, 5.30581	2.51418, 6	3	NA	3276
GL173545.1	1067	1543	1293, 1279, 1287	9, 11, 8	9.72082, 9.95469, 10.1465	6.64182, 6.5399, 6.82176	6.97155, 7	3	gbp7	6983
GL173545.1	49408	49923	49628, 49478, 49464	18, 23, 12	18.84377, 23.74183, 14.23156	10.31044, 11.71949, 8.83588	15.65357,	3	gbp7	0
GL173545.1	78945	79797	79085, 79625, 79333	10, 13, 15	10.39648, 10.81092, 16.30311	6.94096, 6.66988, 9.53825	7.61003, 8	3	gbp7	0
GL173545.1	150383	151664	150761, 151326, 151294	10, 37, 11	10.20047, 43.13613, 12.9493	6.82978, 17.81474, 8.23866	7.42612, 3	3	NA	22759
GL173545.1	216717	217157	216727, 216719, 217111	8, 9, 4	8.42257, 8.21099, 4.67325	5.97763, 5.77858, 3.78987	5.76336, 5	3	NA	89093
GL173545.1	271125	271613	271244, 271256, 271265	75, 62, 66	71.34727, 53.94612, 69.93635	16.85605, 13.76117, 19.61771	67.2371, 5	3	NA	143501
GL173545.1	271903	272729	272314, 272138, 272326	27, 33, 12	15.79336, 21.66943, 5.98883	6.25787, 7.65457, 3.9249	12.72547,	3	NA	144279
GL173545.1	293206	293663	293286, 293529, 293427	11, 16, 4	12.43693, 15.41438, 4.67325	7.97018, 8.77591, 3.78987	9.52692, 1	3	NA	165582
GL173546.1	30024	30642	30291, 30253, 30592	5, 8, 4	4.82177, 7.07159, 4.67325	3.98509, 5.20073, 3.78987	2.51418, 4	3	smyd2	60190
GL173546.1	73465	73741	73604, 73686, 73674	15, 25, 15	15.12865, 25.91543, 16.91122	8.84764, 12.33685, 9.88541	12.09208,	3	smyd2	17091
GL173546.1	107264	108021	107733, 107582, 107556	18, 19, 12	18.37468, 17.64648, 12.56626	10.02961, 9.30188, 7.92374	15.20182,	3	smyd2	0
GL173546.1	180801	181432	181153, 181245, 180920	8, 9, 4	7.62407, 6.76868, 4.67325	5.52896, 4.88312, 3.78987	5.03638, 4	3	rab30	8095
GL173546.1	226729	227176	227083, 227059, 227085	31, 45, 29	33.53976, 48.3063, 36.97725	14.63333, 17.28504, 17.42526	29.95568,	3	ANKRD42	0
GL173546.1	238956	239459	239005, 239001, 239080	9, 13, 10	8.35434, 11.12608, 10.59656	5.86444, 6.86492, 7.05292	5.71584, 8	3	ccdc90b	0
GL173547.1	1296	1666	1392, 1338, 1545	9, 10, 6	6.79611, 6.66155, 5.60548	4.90054, 4.65493, 4.44624	4.28827, 4	3	NA	2498
GL173547.1	9680	10383	9709, 9733, 9702	31, 40, 19	32.24754, 38.82634, 22.51865	13.8697, 14.09749, 12.19038	28.69044,	3	NA	0
GL173547.1	15966	16427	16342, 16339, 16363	13, 26, 12	13.00701, 23.54286, 13.59027	8.00827, 10.58612, 8.49571	10.0703, 2	3	NA	5019
GL173547.1	18884	19549	18990, 18974, 19002	5, 9, 6	4.20732, 5.67905, 6.168	3.62846, 4.18648, 4.75778	2.04024, 3	3	NA	7937
GL173547.1	125515	125925	125877, 125881, 125909	26, 29, 15	25.29674, 25.53788, 16.59998	11.61597, 10.7236, 9.70872	21.90767,	3	gpr19	6285
GL173547.1	147758	148144	147997, 148016, 147960	13, 16, 7	11.21374, 13.12803, 6.51247	6.91908, 7.38023, 4.9427	8.37313, 1	3	tuba8	0
GL173547.1	157661	158271	157869, 158001, 157720	6, 12, 4	5.31708, 9.90946, 4.42274	4.2777, 6.32175, 3.67426	2.96727, 7	3	NA	2586
GL173547.1	223636	224005	223958, 223949, 223939	52, 85, 44	63.29543, 99.06791, 56.33466	22.5514, 25.4673, 22.40119	59.2722, 9	3	mical3	18379
GL173547.1	237140	237635	237179, 237165, 237204	17, 37, 11	17.64386, 41.42659, 11.73651	9.85981, 16.80781, 7.58663	14.49984,	3	mical3	4749
GL173548.1	54509	54759	54709, 54736, 54739	12, 6, 7	13.8483, 4.93312, 8.69489	8.63436, 4.04501, 6.06379	10.86694,	3	NA	46189
GL173548.1	180671	182881	182262, 182153, 182258	53, 67, 32	48.31358, 58.34872, 30.34	14.16515, 14.06796, 12.38652	44.48537,	3	NA	52597
GL173548.1	191877	192505	192132, 192145, 192013	14, 17, 12	14.60315, 15.13134, 15.16114	8.75252, 8.33865, 9.30141	11.5888, 1	3	NA	63803
GL173548.1	210516	211293	210949, 210934, 211023	13, 18, 9	14.26982, 16.2906, 10.21256	8.73911, 8.76731, 6.90044	11.26966,	3	NA	82442
GL173549.1	192477	193283	192763, 192786, 192695	175, 264, 98	178.28114, 260.0842, 102.86008	22.9875, 22.56699, 21.69144	173.40764	3	NA	NA
GL173550.1	93	849	688, 629, 545	5, 10, 8	4.82177, 9.39128, 10.1465	3.98509, 6.35644, 6.82176	2.51418, 6	3	asxl2	150464
GL173550.1	17127	17466	17149, 17161, 17163	7, 21, 7	6.25077, 21.41771, 7.50542	4.78814, 10.97106, 5.49244	3.78441, 1	3	asxl2	133847
GL173550.1	39935	40211	40138, 40162, 40175	14, 28, 16	16.23025, 33.82218, 20.52695	9.67496, 15.9529, 11.67144	13.14538,	3	asxl2	111102
GL173550.1	79160	80345	79901, 79756, 79432	25, 50, 12	26.32546, 58.85878, 14.86478	12.58233, 20.97695, 9.15668	22.90704,	3	asxl2	70968
GL173550.1	87207	88159	88076, 88003, 88117	6, 13, 8	5.06496, 11.81904, 9.31951	4.12592, 7.29135, 6.43944	2.73812, 9	3	asxl2	63154
GL173550.1	88473	88953	88704, 88519, 88698	11, 15, 6	11.29029, 14.27489, 6.6567	7.3332, 8.33298, 5.00845	8.44541, 1	3	asxl2	62360
GL173550.1	164752	165550	164918, 164838, 165403	10, 15, 7	9.661, 13.53022, 7.40077	6.51661, 7.87854, 5.43746	6.9283, 10	3	asxl2	0
GL173550.1	276090	276433	276277, 276274, 276165	22, 35, 19	27.19152, 43.26143, 21.69099	14.0553, 18.74919, 11.71707	23.75121,	3	rab10	7506
GL173551.1	137898	138505	138197, 138260, 138169	6, 8, 5	5.03089, 5.00801, 5.23257	4.10511, 3.89278, 4.20435	2.70624, 2	3	pga4	4549
GL173551.1	271719	275118	272487, 273454, 272088, 273154	15, 18, 13, 16	11.27762, 12.50492, 10.45491, 14.55254	6.49882, 6.5139, 6.44917, 8.25096	8.43406, 9	3	NA	40775
GL173551.1	271889	275118	273931, 273454, 272088, 273154	18, 18, 13, 16	15.01817, 12.50492, 10.45491, 14.55254	7.99668, 6.5139, 6.44917, 8.25096	11.98561,	3	NA	40945
GL173551.1	280151	281073	280592, 280600, 280615	20, 22, 13	22.97335, 22.22711, 16.70862	12.19234, 11.1385, 10.0169	19.64963,	3	NA	49207
GL173551.1	290884	292135	291583, 291591, 291381	15, 29, 11	16.03559, 34.37277, 13.46208	9.3831, 15.90664, 8.49639	12.95858,	3	NA	59940
GL173552.1	43070	43735	43433, 43181, 43526	13, 9, 12	13.07382, 7.51164, 12.62857	8.04786, 5.35055, 7.95947	10.13353,	3	NA	0
GL173552.1	70908	71901	71640, 71751, 71779	12, 12, 11	12.74986, 11.60251, 12.19945	8.02869, 7.3582, 7.8422	9.82533, 8	3	znf665	5306
GL173552.1	141450	141710	141496, 141590, 141628	24, 35, 16	28.41298, 40.9161, 18.1835	14.16116, 17.36254, 10.40854	24.94293,	3	NA	34869
GL173552.1	213379	214256	213853, 213866, 213864	14, 13, 7	14.03163, 11.31501, 8.69489	8.41471, 6.98159, 6.06379	11.04222,	3	NA	3353
GL173552.1	241303	241815	241509, 241780, 241778	6, 12, 6	4.65447, 8.6575, 6.5517	3.87084, 5.54124, 4.95623	2.42652, 6	3	NA	7240
GL173553.1	2034	2312	2218, 2202, 2219	18, 16, 11	19.17347, 16.0832, 12.06254	10.50657, 9.17962, 7.76744	15.97256,	3	nfkbie	90804
GL173553.1	26006	26425	26257, 26316, 26334	7, 6, 7	7.1696, 4.93312, 8.28621	5.31345, 4.04501, 5.87881	4.61046, 2	3	nfkbie	66691
GL173553.1	31404	31925	31665, 31700, 31697	11, 13, 7	11.71035, 10.00327, 6.55126	7.57196, 6.16911, 4.96529	8.8442, 7.3	3	nfkbie	61191
GL173553.1	56884	57273	57042, 57239, 57104	13, 15, 6	11.35857, 12.62899, 5.23492	7.00844, 7.3254, 4.22867	8.51036, 9	3	nfkbie	35843
GL173553.1	70813	71292	70877, 70859, 70952	5, 13, 3	4.47183, 11.12608, 3.17999	3.78598, 6.86492, 2.89245	2.26935, 8	3	nfkbie	21824
GL173553.1	88220	88582	88366, 88326, 88294	8, 12, 11	6.90289, 11.20359, 14.13736	5.09802, 7.11794, 8.81822	4.38737, 8	3	nfkbie	4534
GL173553.1	105487	106611	106146, 106208, 105726	13, 16, 8	13.27928, 12.73735, 9.46129	8.16901, 7.14249, 6.50801	10.32936,	3	nfkbie	0
GL173553.1	133569	134042	133712, 133727, 133770	16, 24, 13	19.41253, 26.77414, 15.89249	11.08796, 13.19592, 9.61177	16.20324,	3	nfkbie	15489
GL173553.1	140542	141041	140722, 140984, 140622	5, 7, 4	4.82177, 5.97725, 4.67325	3.98509, 4.62287, 3.78987	2.51418, 3	3	arhgef10l	14987
GL173553.1	148822	149671	149633, 149279, 149126	4, 18, 8	3.28906, 15.95793, 8.74279	3.0555, 8.56528, 6.14792	1.37521, 1	3	arhgef10l	6357
GL173553.1	154531	154841	154788, 154546, 154561	5, 12, 6	4.40263, 10.35, 6.6037	3.74533, 6.59497, 4.9822	2.21004, 7	3	arhgef10l	1187
GL173553.1	164331	164819	164788, 164764, 164788	26, 51, 31	28.77924, 56.92836, 39.71417	13.69314, 19.41531, 18.2743	25.30043,	3	arhgef10l	0
GL173553.1	175050	175545	175463, 175472, 175491	14, 29, 18	15.31941, 33.2124, 23.23937	9.1665, 15.21917, 12.78599	12.27033,	3	arhgef10l	0
GL173553.1	183579	184513	184073, 184028, 183904	19, 31, 20	19.64557, 36.11736, 21.34975	10.50977, 16.16392, 11.23648	16.42918,	3	arhgef10l	0
GL173553.1	205017	205546	205412, 205384, 205404	28, 48, 33	18.86146, 33.56252, 28.85478	7.48895, 9.24138, 11.21235	15.67075,	3	arhgef10l	144
GL173553.1	207297	208324	207833, 207817, 207927	65, 67, 46	63.968, 55.30877, 46.37577	17.15813, 12.88689, 15.8065	59.93566,	3	arhgef10l	2424
GL173553.1	235358	235798	235599, 235622, 235622	8, 16, 7	7.33538, 14.69174, 7.72582	5.35908, 8.33598, 5.6058	4.76518, 1	3	rcc2	28262
GL173554.1	4819	5349	5059, 5066, 5034	8, 24, 4	7.83097, 23.48334, 4.67325	5.64832, 11.22699, 3.78987	5.22917, 2	3	MGC69493	88409
GL173554.1	11201	11949	11495, 11763, 11219	6, 8, 5	5.96715, 5.81313, 5.64747	4.64927, 4.41317, 4.40911	3.52158, 3	3	MGC69493	81809
GL173554.1	271379	272004	271719, 271816, 271684	13, 18, 10	12.56081, 15.6398, 10.97191	7.74168, 8.37236, 7.25854	9.64511, 1	3	NA	28658
GL173554.1	287660	288545	288086, 288179, 288220	17, 38, 10	19.30082, 42.80689, 11.60305	10.82906, 17.18536, 7.59048	16.09547,	3	NA	44939
GL173555.1	7059	10753	7878, 7433, 9744, 8308, 9518, 10297	17, 19, 11, 11, 4, 7	16.90454, 14.46084, 6.40541, 11.5511, 3.43992, 6.6707	9.41624, 7.39684, 4.32899, 7.48213, 3.14645, 5.03432	13.78827,	3	NA	49561
GL173555.1	21076	21609	21560, 21540, 21533	26, 29, 8	27.91302, 29.84913, 7.81204	13.17594, 13.21951, 5.63749	24.4551, 2	3	NA	38705
GL173555.1	182802	183120	183090, 183061, 182937	9, 17, 7	8.88537, 14.86656, 7.34973	6.17591, 8.17729, 5.41038	6.19961, 1	3	ABCG1	0
GL173555.1	203145	203571	203477, 203447, 203521	5, 12, 7	4.73273, 11.86108, 8.49722	3.93547, 7.51216, 5.97582	2.49377, 9	3	ABCG1	0
GL173555.1	211505	211876	211788, 211779, 211756	8, 8, 6	8.4095, 7.07159, 7.29403	5.97056, 5.20073, 5.30581	5.76336, 4	3	ABCG1	5006
GL173555.1	219011	219436	219096, 219095, 219116	10, 9, 7	9.60506, 7.28344, 7.55909	6.48358, 5.20811, 5.52035	6.87658, 4	3	ABCG1	12512
GL173555.1	247167	247526	247210, 247205, 247195	7, 15, 15	7.10297, 15.47929, 18.53291	5.27706, 9.05625, 10.76715	4.57191, 1	3	ABCG1	40668
GL173556.1	24407	25815	25301, 24759, 25025	4, 11, 7	3.74195, 8.97396, 8.49722	3.32091, 5.9338, 5.97582	1.6222, 6.4	3	NA	NA
GL173556.1	167088	167614	167559, 167521, 167552	10, 17, 9	10.67246, 16.76699, 8.88966	7.09497, 9.33319, 6.17838	7.87096, 1	3	NA	NA
GL173556.1	193143	193337	193312, 193309, 193312	22, 22, 17	21.40417, 18.57394, 18.80835	10.64328, 8.95796, 10.54536	18.12938,	3	NA	NA
GL173556.1	195697	196053	196044, 196003, 196028	26, 45, 18	25.01044, 45.30229, 18.21161	11.44656, 15.63352, 9.93156	21.62959,	3	NA	NA
GL173556.1	200103	200624	200117, 200125, 200166	14, 24, 17	13.57329, 21.6536, 20.57409	8.13982, 10.14069, 11.54114	10.60942,	3	NA	NA
GL173556.1	246364	246628	246597, 246597, 246599	34, 49, 32	38.01896, 54.06084, 42.9586	16.0684, 18.72814, 19.75937	34.34908,	3	NA	NA
GL173558.1	33	947	312, 578, 413	24, 38, 24	23.10275, 34.40545, 25.44036	10.99971, 12.46078, 12.39965	19.77471,	3	sorcs3	16090
GL173558.1	4349	5123	4625, 4628, 4615	21, 38, 27	17.41956, 30.97811, 27.27747	8.57638, 10.71141, 12.43437	14.28349,	3	sorcs3	11914
GL173558.1	6617	7151	7101, 6985, 6702	11, 18, 11	10.21742, 14.80783, 10.77078	6.69945, 7.8701, 7.03024	7.44132, 1	3	sorcs3	9886
GL173558.1	32835	34103	33433, 33435, 33434	66, 65, 35	69.62333, 64.56054, 39.88788	19.4633, 17.43354, 16.75407	65.53082,	3	sorcs3	0
GL173558.1	69975	70422	70020, 70003, 69992	18, 17, 12	19.17347, 16.83555, 12.7555	10.50657, 9.3746, 8.03189	15.97256,	3	sorcs3	4344
GL173558.1	77194	77636	77243, 77237, 77362	30, 32, 11	33.92505, 33.59991, 13.1143	15.25598, 14.27354, 8.32282	30.33351,	3	sorcs3	11563
GL173558.1	83080	83590	83239, 83314, 83135	11, 13, 6	10.72304, 9.85593, 6.5517	7.00201, 6.07784, 4.95623	7.91902, 7	3	sorcs3	17449
GL173558.1	107328	108224	108166, 108173, 108177	17, 30, 9	20.35299, 32.90641, 11.64359	11.41991, 14.6501, 7.57973	17.11204,	3	sorcs3	41697
GL173558.1	142262	142702	142534, 142324, 142502	6, 6, 3	4.48574, 4.93312, 3.17999	3.76346, 4.04501, 2.89245	2.28228, 2	3	sorcs3	76631
GL173558.1	160542	161580	160868, 161319, 160861	12, 16, 7	13.05874, 15.04317, 8.49722	8.20296, 8.55029, 5.97582	10.11961,	3	sorcs3	94911
GL173558.1	172663	174510	173417, 173394, 173455	16, 22, 14	15.34845, 21.01356, 14.79463	8.73591, 10.40847, 8.86428	12.2978, 1	3	sorcs3	107032
GL173558.1	181939	184144	183313, 182202, 182705	10, 14, 7	11.06011, 14.45897, 8.69489	7.306, 8.66788, 6.06379	8.22712, 1	3	sorcs3	116308
GL173558.1	196908	197840	197464, 197429, 197406	9, 15, 5	9.36986, 15.19102, 5.9506	6.44997, 8.88488, 4.54784	6.65425, 1	3	sorcs3	131277
GL173558.1	198205	198722	198338, 198373, 198349	5, 12, 3	4.50727, 10.93692, 3.47371	3.80664, 6.95571, 3.03189	2.30125, 8	3	sorcs3	132574
GL173558.1	212737	213311	212995, 212963, 213049	11, 10, 9	9.00946, 6.85174, 8.43009	5.95596, 4.77545, 5.90952	6.31598, 4	3	sorcs3	147106
GL173558.1	224919	225578	225139, 225408, 225287	8, 15, 3	7.99469, 13.26621, 3.24307	5.74128, 7.71668, 2.92359	5.38155, 1	3	sorcs3	159288
GL173558.1	226008	226512	226191, 226026, 226133	7, 20, 7	6.05136, 18.65688, 7.40077	4.66801, 9.61459, 5.43746	3.59939, 1	3	sorcs3	160377
GL173558.1	277068	277556	277204, 277288, 277176	15, 16, 15	18.26603, 15.67391, 16.91122	10.62691, 8.93306, 9.88541	15.09729,	3	sorcs3	211437
GL173559.1	283726	284811	284409, 284476, 283800, 284517	22, 29, 12, 11	18.486, 24.64275, 9.21464, 8.29649	8.9065, 10.22538, 5.88864, 5.50917	15.30952,	3	U6	0
GL173560.1	21082	22172	21316, 21559, 21642	10, 20, 15	8.0085, 14.60421, 15.36085	5.50663, 7.22067, 8.986	5.39412, 1	3	xpo5	0
GL173560.1	51125	52349	52008, 51754, 51918	8, 12, 10	7.24433, 9.16947, 10.71812	5.30475, 5.86046, 7.12015	4.68023, 6	3	NA	0
GL173560.1	70643	71416	71373, 71204, 71002	9, 11, 4	9.72082, 10.42613, 4.55201	6.64182, 6.8251, 3.73489	6.97155, 7	3	exd3	6678
GL173560.1	86246	86920	86873, 86890, 86889	56, 68, 45	69.29388, 79.42607, 58.42674	23.99003, 23.58686, 23.15362	65.20489,	3	exd3	0
GL173560.1	99518	100236	99917, 99972, 100036	9, 23, 7	7.47395, 19.55015, 6.6707	5.32711, 9.225, 5.03432	4.89626, 1	3	exd3	0
GL173560.1	101582	101953	101730, 101872, 101665	8, 16, 8	6.66791, 12.05974, 8.04677	4.95324, 6.7328, 5.77057	4.17132, 9	3	exd3	0
GL173560.1	146503	147492	147010, 147244, 147038	16, 15, 8	16.91852, 14.7819, 10.1465	9.67673, 8.63964, 6.82176	13.80145,	3	tcte1	10175
GL173560.1	188958	189509	189232, 189196, 189264	10, 15, 8	10.53228, 14.39783, 10.1465	7.01712, 8.40758, 6.82176	7.73927, 1	3	NA	17908
GL173560.1	282014	282459	282427, 282409, 282403	15, 10, 6	18.26603, 9.39128, 7.29403	10.62691, 6.35644, 5.30581	15.09729,	3	hsp90ab1	2812
GL173560.1	299035	299555	299375, 299210, 299102	6, 5, 3	5.96715, 3.94561, 3.47371	4.64927, 3.46715, 3.03189	3.52158, 1	3	hist1h4c	8231
GL173561.1	16654	17682	17132, 16736, 17323	15, 6, 9	18.12902, 4.93312, 11.64359	10.55412, 4.04501, 7.57973	14.96737,	3	NA	30660
GL173561.1	80290	81673	81569, 80600, 81419	7, 11, 13	4.74226, 7.72874, 11.42074	3.83953, 5.15241, 7.04675	2.50288, 5	3	per1	0
GL173561.1	83392	83834	83462, 83517, 83572	11, 12, 14	8.54584, 8.13852, 11.45195	5.66575, 5.21905, 6.84022	5.88025, 5	3	per1	0
GL173561.1	99057	99537	99293, 99320, 99094	7, 13, 8	7.1696, 12.55412, 7.76694	5.31345, 7.73766, 5.6116	4.61046, 9	3	vamp2	3662
GL173561.1	173771	173947	173840, 173840, 173840	752, 832, 599	949.37866, 1050.37036, 799.21008	45.36077, 45.66128, 52.038	942.74609	3	U8	5995
GL173561.1	185851	186333	186171, 186221, 186179	12, 12, 7	12.25023, 11.86108, 8.646	7.74089, 7.51216, 6.04229	9.35789, 9	3	NA	1374
GL173561.1	242042	242349	242349, 242307, 242287	24, 30, 21	25.27938, 28.94703, 24.85325	12.30321, 12.31903, 13.00095	21.89126,	3	cops6	13766
GL173561.1	247484	247668	247512, 247508, 247524	28, 50, 19	31.08474, 53.96709, 22.04849	14.33238, 18.23011, 11.92281	27.55281,	3	cops6	8447
GL173561.1	258513	259130	258692, 258693, 258760	15, 38, 12	14.39822, 38.49907, 11.07378	8.40782, 14.70177, 7.03912	11.39247,	3	cops6	0
GL173561.1	261349	261754	261676, 261678, 261724	12, 21, 17	11.09909, 17.5526, 18.96688	7.05451, 8.65464, 10.63714	8.26391, 1	3	cops6	0
GL173562.1	249749	250756	249910, 250096, 250202	14, 20, 5	11.93818, 19.16931, 4.88115	7.14017, 9.92426, 4.01777	9.06106, 1	3	NA	2565
GL173563.1	235	3963	2754, 3025, 775	27, 27, 24	19.1132, 18.4299, 20.26516	7.85904, 7.51442, 9.33214	15.91451,	3	NA	44184
GL173563.1	20858	22029	21164, 21516, 20971	11, 14, 6	11.56615, 14.36901, 6.99807	7.49066, 8.61486, 5.17195	8.70729, 1	3	NA	26118
GL173564.1	199361	200170	199759, 199759, 199729	8, 9, 5	7.57423, 8.21099, 5.80393	5.4999, 5.77858, 4.48186	4.98865, 5	3	ighx	0
GL173565.1	150553	150925	150781, 150776, 150780	11, 7, 6	11.22377, 5.36631, 6.6037	7.29486, 4.24177, 4.9822	8.38272, 3	3	maea	31990
GL173565.1	220926	221625	221245, 220997, 221024	14, 8, 6	14.3105, 6.77831, 6.30561	8.58029, 5.0215, 4.83031	11.30855,	3	maea	0
GL173565.1	266865	268263	267404, 267408, 267429	52, 71, 26	53.92344, 77.30183, 29.21416	17.34645, 21.10845, 13.95216	50.01554,	3	uvssa	0
GL173566.1	41667	42799	41919, 42450, 42402	14, 17, 12	14.10012, 16.36909, 13.90113	8.4555, 9.09221, 8.66246	11.10747,	3	pax7	23093
GL173566.1	74427	74969	74607, 74760, 74578	12, 16, 13	11.98519, 14.74902, 16.49347	7.58551, 8.37095, 9.91245	9.10644, 1	3	pax7	0
GL173566.1	97198	97977	97319, 97275, 97599	8, 8, 4	8.42257, 7.07159, 4.67325	5.97763, 5.20073, 3.78987	5.76336, 4	3	pax7	0
GL173566.1	183996	184625	184356, 184348, 184209	7, 18, 5	6.33443, 18.22482, 5.69847	4.83795, 9.93951, 4.4331	3.86259, 1	3	pax7	23586
GL173566.1	185570	185997	185929, 185964, 185811	8, 10, 6	7.06928, 8.20397, 6.6567	5.19933, 5.62902, 5.00845	4.5414, 5.7	3	aldh4a1	22797
GL173566.1	212115	212503	212341, 212199, 212142	9, 10, 7	8.77314, 8.53104, 7.10639	6.111, 5.83243, 5.27894	6.09355, 6	3	aldh4a1	0
GL173566.1	263645	264898	264344, 264308, 264289	41, 74, 27	42.70038, 78.7926, 29.05416	15.84198, 20.55078, 13.49336	38.95523,	3	iffo2	0
GL173567.1	112637	113358	112920, 112891, 112726	16, 22, 5	16.45267, 22.45914, 5.9506	9.40062, 11.27809, 4.54784	13.35812,	3	hnrnpu	13389
GL173568.1	159335	159655	159537, 159555, 159588	8, 10, 10	7.72597, 9.07627, 11.24131	5.588, 6.1667, 7.40241	5.13061, 6	3	NA	114205
GL173568.1	229119	230136	229756, 229711, 229599	17, 25, 5	17.33887, 24.29926, 5.14967	9.67746, 11.37153, 4.16141	14.20638,	3	NA	43724
GL173568.1	260859	262003	261338, 261581, 261316	24, 30, 18	22.55986, 26.65553, 17.96175	10.67652, 11.01397, 9.78096	19.24961,	3	NA	11857
GL173568.1	262432	262978	262599, 262733, 262695	15, 13, 10	11.99233, 8.38839, 8.39789	6.93485, 5.17779, 5.74985	9.11296, 5	3	NA	10882
GL173568.1	277556	279418	278121, 278637, 278629	17, 29, 19	13.69742, 23.37083, 17.5517	7.46768, 9.53331, 9.24446	10.72893,	3	NA	2615
GL173569.1	28	896	208, 715, 69	7, 13, 6	7.1696, 13.06145, 7.29403	5.31345, 8.04054, 5.30581	4.61046, 1	3	NA	3755
GL173569.1	268765	269601	268945, 269301, 268902, 269223	10, 20, 10, 6	9.18236, 18.9091, 11.03772, 5.95321	6.23091, 9.76697, 7.29398, 4.64162	6.47811, 1	3	olfml2a	24867
GL173570.1	65148	65793	65602, 65681, 65687	38, 36, 22	37.21685, 32.63727, 20.85813	13.9846, 12.22094, 10.31514	33.56121,	3	NA	109992
GL173570.1	181114	181811	181504, 181504, 181473	16, 17, 12	17.41877, 17.87191, 15.36829	9.96955, 9.99549, 9.40047	14.28292,	3	NA	1005
GL173571.1	218153	218781	218591, 218560, 218564	20, 34, 9	19.84853, 36.63209, 10.14484	10.3347, 15.251, 6.86555	16.62382,	3	sirt3.2	0
GL173572.1	93733	94426	93923, 94299, 94043	8, 10, 7	8.22568, 8.4887, 7.2501	5.8701, 5.80621, 5.35703	5.59681, 5	3	dpp9	3776
GL173574.1	273799	274630	274334, 274138, 274200	12, 12, 13	13.38718, 11.86108, 17.65969	8.38496, 7.51216, 10.45768	10.43197,	3	socs6	34219
GL173576.1	83	469	390, 390, 380	391, 485, 281	428.92627, 531.38397, 309.09244	29.94313, 30.38651, 29.10481	423.44879	3	slc35e1	6576
GL173577.1	72583	73557	72697, 73047, 72931	19, 17, 10	19.25925, 15.46309, 10.97191	10.27754, 8.5409, 7.25854	16.05534,	3	5S_rRNA	10987
GL173577.1	100725	101824	101250, 101508, 101359	4, 11, 8	3.74195, 10.60895, 10.1465	3.32091, 6.9343, 6.82176	1.6222, 7.9	3	5S_rRNA	12854
GL173577.1	117459	117904	117860, 117822, 117857	6, 11, 10	4.65447, 9.3497, 10.84317	3.87084, 6.16764, 7.18868	2.42652, 6	3	5S_rRNA	2661
GL173577.1	124194	124573	124252, 124252, 124409	6, 12, 7	4.22823, 7.96865, 6.59055	3.59714, 5.1141, 4.98809	2.0602, 5.4	3	5S_rRNA	9396
GL173577.1	133934	134453	134112, 134150, 134210	16, 20, 9	15.60786, 16.78097, 8.74593	8.89312, 8.48884, 6.09519	12.5472, 1	3	NA	18509
GL173577.1	137803	138676	138056, 138056, 138167	9, 18, 10	7.20928, 13.31424, 9.73055	5.16158, 6.983, 6.55755	4.64652, 1	3	NA	14286
GL173577.1	150350	150780	150626, 150616, 150588	6, 6, 7	5.96715, 4.93312, 8.69489	4.64927, 4.04501, 6.06379	3.52158, 2	3	NA	2182
GL173577.1	180511	181286	181200, 180773, 181045	9, 11, 6	9.63662, 10.26356, 6.99807	6.59633, 6.72731, 5.17195	6.90556, 7	3	XB-GENE-5767170	0
GL173577.1	188742	189397	189014, 189243, 189134	6, 6, 7	5.96715, 4.93312, 8.21885	4.64927, 4.04501, 5.84717	3.52158, 2	3	XB-GENE-5767170	0
GL173577.1	248841	249297	248988, 249115, 249029	5, 7, 5	4.82177, 5.97725, 5.64747	3.98509, 4.62287, 4.40911	2.51418, 3	3	NA	16087
GL173577.1	269653	270213	269789, 269964, 269959	6, 8, 9	5.31708, 5.46019, 10.79992	4.2777, 4.18585, 7.19283	2.96727, 3	3	NA	36899
GL173577.1	270855	271196	270934, 271024, 270965	6, 17, 4	5.51356, 15.69342, 4.46488	4.39314, 8.68127, 3.69425	3.14574, 1	3	NA	38101
GL173578.1	44682	45088	45068, 45056, 45044	61, 98, 55	69.85999, 118.00048, 71.96968	21.82805, 28.45554, 26.06189	65.76476,	3	XB-GENE-5795351	6144
GL173578.1	187951	188551	188093, 188241, 188068	7, 9, 3	7.1696, 8.21099, 3.47371	5.31345, 5.77858, 3.03189	4.61046, 5	3	NA	6438
GL173579.1	257791	258719	258030, 258096, 257925	12, 21, 6	11.61187, 20.12483, 6.08001	7.3638, 10.19169, 4.71063	8.75096, 1	3	pcdh8.2	7872
GL173580.1	82344	82676	82627, 82638, 82643	37, 35, 21	42.16415, 37.3034, 29.35663	17.24147, 15.23432, 15.41596	38.42738,	3	NA	1490
GL173580.1	151531	152528	151843, 152220, 152054	6, 14, 8	4.17989, 10.08648, 7.11599	3.56562, 6.00146, 5.22758	2.01712, 7	3	NA	2013
GL173580.1	181405	182323	182230, 182143, 182064	13, 10, 8	11.98086, 7.72315, 9.46129	7.39024, 5.32711, 6.50801	9.10256, 5	3	NA	212
GL173580.1	269738	270467	269923, 269901, 269993	12, 24, 11	13.8483, 28.90463, 14.75772	8.63436, 14.44646, 9.09568	10.86694,	3	NA	23250
GL173581.1	40953	41455	41063, 41201, 41195	6, 11, 8	5.63898, 9.17879, 8.6841	4.46544, 6.06147, 6.11715	3.25772, 6	3	ipo8	0
GL173581.1	54101	54434	54352, 54353, 54352	11, 15, 8	10.21742, 13.21475, 9.46129	6.69945, 7.68511, 6.50801	7.44132, 1	3	ipo8	188
GL173581.1	107569	108224	108131, 107826, 108099	8, 12, 6	8.34715, 10.44309, 7.29403	5.9367, 6.65248, 5.30581	5.71011, 7	3	TMTC1	18733
GL173581.1	186332	187008	186669, 186737, 186531	10, 8, 4	11.06011, 6.89656, 4.38152	7.306, 5.09414, 3.65448	8.22712, 4	3	ovch1	0
GL173581.1	256300	256975	256627, 256498, 256326	13, 22, 9	13.63968, 23.53859, 9.35579	8.37925, 11.92574, 6.44216	10.67258,	3	ergic2	40168
GL173581.1	270202	270473	270243, 270249, 270249	13, 14, 4	15.29169, 14.45897, 4.67325	9.29854, 8.66788, 3.78987	12.24396,	3	ergic2	54070
GL173582.1	78639	79047	78827, 78753, 78829	7, 12, 6	7.1696, 11.86108, 7.29403	5.31345, 7.51216, 5.30581	4.61046, 9	3	fcrl2	11359
GL173582.1	131198	131716	131536, 131358, 131357	5, 19, 6	4.82177, 21.4153, 7.29403	3.98509, 11.55717, 5.30581	2.51418, 1	3	fcrl2	7105
GL173582.1	152527	152930	152779, 152770, 152818	10, 13, 12	11.06011, 11.98001, 14.40488	7.306, 7.38972, 8.92522	8.22712, 9	3	NA	8400
GL173582.1	245808	247951	246605, 246319, 247734, 246378, 247462	40, 42, 22, 35, 6	40.25873, 36.57784, 14.43505, 37.55862, 3.37308	14.88537, 12.20999, 6.64342, 15.38339, 3.02934	36.55223,	3	NA	0
GL173584.1	40294	41394	40780, 40582, 40891	12, 11, 7	12.60209, 10.05535, 8.69489	7.9443, 6.6012, 6.06379	9.68421, 7	3	NA	136867
GL173585.1	178659	179374	179031, 179326, 178975	14, 13, 8	15.31941, 13.14514, 9.46129	9.1665, 8.09002, 6.50801	12.27033,	3	sh2b2	43931
GL173585.1	284163	284706	284321, 284386, 284334	20, 30, 15	12.40477, 16.31013, 10.40443	6.01034, 5.94206, 5.97257	9.50403, 1	3	U6	13
GL173585.1	285241	286348	286232, 286097, 286097	19, 28, 15	11.69153, 15.36254, 10.57179	5.83912, 5.88547, 6.07272	8.82703, 1	3	U6	0
GL173586.1	31689	32500	31968, 32122, 32050	14, 17, 10	13.51064, 14.31774, 10.71812	8.10201, 7.84337, 7.12015	10.55025,	3	NA	5486
GL173586.1	37596	38899	38074, 38152, 38406	14, 19, 8	10.82413, 14.87127, 6.63406	6.45331, 7.63748, 4.93224	8.01418, 1	3	NA	11393
GL173586.1	59563	59946	59936, 59896, 59828	13, 20, 5	11.81837, 19.03817, 4.39541	7.29094, 9.84499, 3.74107	8.94738, 1	3	NA	33360
GL173586.1	60463	60728	60492, 60544, 60540	11, 13, 17	9.52723, 10.7674, 19.99824	6.27749, 6.64292, 11.2232	6.80251, 8	3	NA	34260
GL173586.1	118938	119548	119163, 119377, 119440	6, 6, 5	4.77375, 3.76939, 4.70718	3.9459, 3.29482, 3.9211	2.51418, 1	3	eif4g2	26372
GL173586.1	122916	123603	123459, 123577, 123481	11, 12, 6	9.39207, 10.25865, 5.8719	6.1939, 6.53846, 4.59674	6.67495, 7	3	eif4g2	22317
GL173586.1	170863	171419	171144, 171120, 171278	6, 11, 7	5.1345, 9.71235, 7.90186	4.16817, 6.39153, 5.69394	2.80161, 7	3	ctr9	0
GL173586.1	190527	191278	190578, 190623, 190739	15, 24, 18	16.03559, 22.26844, 20.40623	9.3831, 10.50366, 11.22809	12.95858,	3	ctr9	9011
GL173586.1	207365	208012	207600, 207449, 207412	7, 17, 6	6.69449, 16.17826, 6.5517	5.04797, 8.97632, 4.95623	4.1953, 13	3	MRVI1	22395

GL173586.1	250358	251073	251002, 251004, 251027	32, 62, 33	32.19237, 68.38261, 40.66947	13.4508, 20.57703, 18.05017	28.63672,	3	MRVI1	0
GL173587.1	51413	51870	51844, 51852, 51840	12, 17, 13	11.32055, 16.69909, 15.61412	7.18871, 9.29214, 9.46817	8.47391, 1	3	mlx	2430
GL173587.1	62212	63951	62973, 63177, 63345	14, 11, 8	10.03085, 6.79231, 7.50809	5.96757, 4.56763, 5.46116	7.26507, 4	3	NA	2041
GL173587.1	148504	148923	148626, 148770, 148562	7, 15, 6	6.01305, 12.72276, 6.45058	4.6447, 7.383, 4.90508	3.56347, 9	3	rtfdc1	0
GL173587.1	155242	156214	155575, 155723, 156104	7, 14, 11	4.44367, 7.56057, 9.18051	3.644, 4.51024, 6.06254	2.24674, 5	3	rtfdc1	0
GL173587.1	158252	159311	158511, 158972, 158484	11, 18, 13	8.92872, 12.02482, 11.72615	5.90554, 6.2405, 7.23445	6.24102, 9	3	rtfdc1	0
GL173587.1	190574	191763	190885, 190823, 190978	9, 26, 11	8.82885, 26.71306, 12.86886	6.14328, 12.45881, 8.19722	6.14693, 2	3	tfap2c	25129
GL173587.1	234495	234813	234694, 234810, 234789	10, 14, 13	9.23294, 11.80872, 14.69628	6.26141, 7.06033, 8.97644	6.52626, 9	3	tfap2c	4679
GL173587.1	243070	244183	243569, 244160, 244152	7, 38, 19	4.46548, 40.5536, 22.23331	3.65833, 15.87432, 12.02841	2.26751, 3	3	tfap2c	13254
GL173587.1	243070	244183	244158, 244160, 244152	25, 38, 19	25.40524, 40.5536, 22.23331	12.03153, 15.87432, 12.02841	22.01287,	3	tfap2c	13254
GL173587.1	267719	268339	267730, 267997, 268047	6, 11, 8	5.96715, 10.60895, 7.90404	4.64927, 6.9343, 5.68997	3.52158, 7	3	tfap2c	37903
GL173588.1	213129	213489	213413, 213398, 213386	9, 12, 13	9.72082, 11.86108, 18.01082	6.64182, 7.51216, 10.61163	6.97155, 9	3	NA	17286
GL173588.1	247181	247534	247480, 247508, 247495	17, 31, 22	20.35299, 34.26225, 30.43427	11.41991, 15.06205, 15.79077	17.11204,	3	NA	51338
GL173589.1	21992	22520	22240, 22395, 22118	6, 9, 6	5.96715, 8.21099, 7.29403	4.64927, 5.77858, 5.30581	3.52158, 5	3	NA	10316
GL173589.1	27117	27723	27291, 27125, 27226	4, 8, 6	3.74195, 7.07159, 7.29403	3.32091, 5.20073, 5.30581	1.6222, 4.6	3	surf4.2	5760
GL173589.1	34305	34842	34772, 34783, 34705	16, 15, 9	19.7934, 15.80067, 11.64359	11.29109, 9.24574, 7.57973	16.57067,	3	surf4.2	190
GL173589.1	56671	56945	56699, 56697, 56686	22, 33, 17	22.36913, 33.20176, 18.6532	11.22395, 13.64983, 10.45515	19.06395,	3	stkld1	0
GL173589.1	57395	57682	57669, 57653, 57634	22, 32, 15	22.52709, 32.00072, 15.36085	11.31895, 13.33961, 8.986	19.2175, 2	3	stkld1	0
GL173589.1	79151	79597	79232, 79243, 79355	9, 12, 10	9.72082, 11.86108, 12.71002	6.64182, 7.51216, 8.12734	6.97155, 9	3	sardh	6672
GL173589.1	97765	98272	97836, 97840, 97908	5, 10, 8	4.82177, 9.12511, 9.76268	3.98509, 6.19629, 6.64964	2.51418, 6	3	sardh	0
GL173589.1	176400	177033	176745, 176731, 176570	8, 10, 8	8.42257, 9.39128, 10.1465	5.97763, 6.35644, 6.82176	5.76336, 6	3	dbh	20585
GL173589.1	201424	201945	201601, 201827, 201601	9, 11, 6	8.25643, 8.66477, 5.46454	5.80588, 5.74034, 4.36458	5.62516, 6	3	fam163b	34345
GL173589.1	208614	209033	208838, 208799, 208800	16, 14, 9	19.64758, 13.20621, 9.82249	11.21376, 7.91755, 6.69629	16.43074,	3	fam163b	27257
GL173589.1	265525	266252	266032, 266150, 265905	5, 14, 3	4.82177, 14.45897, 3.47371	3.98509, 8.66788, 3.03189	2.51418, 1	3	NA	2369
GL173590.1	11264	11934	11503, 11718, 11744	7, 9, 7	5.86458, 6.67403, 7.50542	4.55375, 4.82293, 5.49244	3.46146, 4	3	mmp24	9341
GL173590.1	82843	83578	83520, 83531, 83548	33, 41, 22	37.29474, 44.19611, 24.76686	16.05296, 16.6903, 12.65568	33.63731,	3	mtss1.2	22905
GL173590.1	94954	95500	95083, 95195, 95270	5, 5, 6	4.20732, 3.54416, 5.83216	3.62846, 3.21033, 4.57461	2.04024, 1	3	mtss1.2	10983
GL173590.1	168947	169718	169479, 169479, 169651	12, 16, 10	13.22036, 14.41261, 12.71002	8.29296, 8.16543, 8.12734	10.27237,	3	tldc2	11914
GL173590.1	178375	179086	178439, 178901, 178555	9, 9, 6	9.72082, 8.21099, 7.29403	6.64182, 5.77858, 5.30581	6.97155, 5	3	soga1	17927
GL173590.1	201644	202715	202357, 202352, 202237	15, 21, 12	14.39822, 19.79752, 12.38374	8.40782, 9.99446, 7.81847	11.39247,	3	soga1	0
GL173590.1	234573	235297	235194, 235281, 235259	16, 23, 24	15.34845, 24.41444, 29.70137	8.73591, 12.12294, 14.90351	12.2978, 2	3	soga1	0
GL173591.1	36	1191	358, 382, 354	42, 45, 30	43.20476, 45.98691, 33.04074	15.70958, 16.00422, 14.72999	39.45248,	3	NA	12894
GL173591.1	176503	176904	176561, 176648, 176775	5, 8, 6	4.82177, 7.07159, 7.05929	3.98509, 5.20073, 5.20024	2.51418, 4	3	NA	22567
GL173592.1	1675	2440	2301, 1988, 2169	8, 7, 3	8.42257, 5.97725, 3.47371	5.97763, 4.62287, 3.03189	5.76336, 3	3	scube1	54231
GL173592.1	11887	12377	12022, 12214, 12061	7, 11, 8	7.1696, 10.60895, 10.1465	5.31345, 6.9343, 6.82176	4.61046, 7	3	scube1	44294
GL173592.1	29988	30288	30205, 30244, 30259	7, 8, 12	5.79309, 6.11443, 12.62857	4.5096, 4.60556, 7.95947	3.39779, 3	3	scube1	26383
GL173592.1	33085	33987	33354, 33354, 33354	19, 19, 8	21.71142, 18.6168, 7.59222	11.72888, 9.88966, 5.5104	18.42619,	3	scube1	22684
GL173592.1	46037	46760	46714, 46715, 46663	14, 13, 10	16.32935, 13.14514, 11.17239	9.72892, 8.09002, 7.36591	13.23998,	3	scube1	9911
GL173592.1	56908	57408	57313, 57353, 57353	15, 18, 15	16.20017, 17.936, 19.40672	9.47871, 9.76542, 11.21161	13.11636,	3	scube1	0
GL173592.1	73492	73963	73912, 73911, 73925	24, 34, 13	20.38162, 28.69689, 11.25379	9.39926, 10.80056, 6.94381	17.13986,	3	scube1	0
GL173592.1	75324	75808	75779, 75781, 75778	48, 72, 51	52.39049, 80.68519, 67.03466	18.24557, 22.35062, 25.23822	48.50657,	3	scube1	0
GL173592.1	96322	96713	96652, 96654, 96655	8, 7, 15	7.77807, 4.516, 17.49481	5.618, 3.6915, 10.21058	5.17956, 2	3	scube1	0
GL173592.1	145955	146352	146318, 146304, 146326	20, 26, 23	18.0937, 22.78014, 24.80209	9.27485, 10.14557, 12.3549	14.93353,	3	scube1	0
GL173592.1	238985	239811	239432, 239516, 239542	16, 17, 5	15.74146, 13.72161, 4.70718	8.97386, 7.48229, 3.9211	12.67506,	3	fgd4	0
GL173593.1	23828	24458	24253, 24162, 23905	6, 13, 6	5.77068, 11.26714, 6.25896	4.54016, 6.95205, 4.80589	3.37745, 8	3	hist1h4c	4068
GL173593.1	123549	123946	123676, 123644, 123753	6, 7, 7	5.24248, 4.16467, 6.55126	4.2332, 3.46025, 4.96529	2.89962, 2	3	hist1h2ah	59889
GL173593.1	129251	130482	129660, 130083, 130171	9, 12, 7	7.24594, 9.0625, 6.21898	5.18459, 5.7937, 4.76912	4.68113, 6	3	hist1h2ah	53353
GL173594.1	14456	15097	14953, 14694, 14646	15, 10, 7	12.16365, 7.4213, 6.6707	7.03981, 5.13639, 5.03432	9.27622, 4	3	NA	NA
GL173594.1	170055	170465	170340, 170273, 170161	5, 6, 4	4.30277, 4.62266, 3.82084	3.68597, 3.8507, 3.36473	2.12436, 2	3	NA	NA
GL173594.1	218720	219293	218952, 219039, 218890	11, 23, 9	12.43693, 27.36982, 11.64359	7.97018, 13.8686, 7.57973	9.52692, 2	3	NA	NA
GL173594.1	225159	225634	225268, 225368, 225408	10, 26, 12	9.54985, 27.30311, 16.0427	6.45088, 12.81135, 9.71071	6.82341, 2	3	NA	NA
GL173594.1	251814	252504	252036, 252087, 252006	22, 26, 22	19.39365, 21.35565, 23.24673	9.44099, 9.33842, 11.75103	16.18635,	3	NA	NA
GL173594.1	252748	253846	253100, 253435, 253222	13, 15, 7	9.55588, 9.66304, 5.17296	5.89229, 5.53388, 4.1183	6.82923, 7	3	NA	NA
GL173594.1	255953	256350	255992, 256005, 255982	9, 17, 9	5.02934, 9.796, 5.73147	3.7719, 5.21202, 4.22005	2.70624, 7	3	NA	NA
GL173594.1	261470	262043	261901, 261999, 261976	30, 49, 30	28.54149, 43.48391, 29.51654	12.0849, 13.23702, 12.64975	25.068, 39	3	NA	NA
GL173594.1	262735	263016	262899, 262881, 262952	12, 30, 13	7.89363, 21.55828, 9.29268	5.06786, 8.31953, 5.73001	5.28702, 1	3	NA	NA
GL173594.1	278733	279389	279005, 278993, 278815	8, 9, 3	6.44853, 7.03406, 3.47371	4.81646, 5.05123, 3.03189	3.96884, 4	3	NA	NA
GL173595.1	29901	30861	30491, 30112, 30086	9, 15, 10	9.72082, 15.12096, 12.33934	6.64182, 8.84304, 7.95424	6.97155, 1	3	atp1a1	17404
GL173595.1	65710	66138	65923, 65859, 65849	13, 9, 14	13.34951, 8.21099, 14.16595	8.21021, 5.77858, 8.49463	10.39596,	3	atp1a1	9322
GL173595.1	81164	81431	81214, 81215, 81198	18, 32, 13	19.00683, 32.48508, 12.60191	10.40758, 13.62105, 7.76639	15.81175,	3	atp1a1	24776
GL173595.1	89525	89901	89609, 89661, 89589	9, 8, 10	6.73165, 5.28564, 8.39789	4.85958, 4.07288, 5.74985	4.22986, 3	3	NA	27780
GL173595.1	136797	137535	137123, 137132, 137130	11, 11, 8	8.54584, 9.57292, 8.80246	5.66575, 6.30569, 6.179	5.88025, 6	3	slc22a15.1	0
GL173595.1	152148	152608	152373, 152211, 152414	12, 14, 11	11.92103, 13.50676, 12.9493	7.54764, 8.09967, 8.23866	9.04508, 1	3	ptpn23	0
GL173595.1	161168	162536	162355, 161783, 161577	8, 10, 10	7.06928, 8.70492, 11.24131	5.19933, 5.93975, 7.40241	4.5414, 6.1	3	ptpn23	0
GL173595.1	216011	216414	216093, 216092, 216112	17, 21, 9	16.76548, 21.41771, 10.01275	9.33227, 10.97106, 6.79683	13.6543, 1	3	scap	1130
GL173596.1	52591	53338	53028, 53092, 53112	36, 46, 26	42.06099, 47.74661, 31.05589	17.61243, 16.54833, 15.03947	38.32584,	3	NA	21478
GL173596.1	68001	68557	68499, 68471, 68514	13, 13, 10	12.94099, 13.06145, 12.33934	7.96907, 8.04054, 7.95424	10.00729,	3	NA	33326
GL173596.1	162066	162699	162137, 162214, 162226	7, 18, 9	7.1696, 18.60217, 9.94831	5.31345, 10.16604, 6.76298	4.61046, 1	3	NA	37738
GL173596.1	226498	227244	226808, 226945, 226772	8, 7, 5	6.66791, 4.516, 4.88115	4.95324, 3.6915, 4.01777	4.17132, 2	3	NA	1775
GL173596.1	227978	228455	228429, 228399, 228400	13, 26, 14	12.56081, 27.30311, 17.2091	7.74168, 12.81135, 10.19524	9.64511, 2	3	NA	3255
GL173596.1	245470	245765	245757, 245745, 245732	37, 58, 38	37.7281, 63.1048, 43.42611	14.66624, 19.59774, 17.54885	34.06293,	3	NA	13073
GL173596.1	272525	272750	272721, 272719, 272729	11, 19, 9	10.72304, 17.76909, 10.28145	7.00201, 9.37618, 6.93568	7.91902, 1	3	NA	0
GL173597.1	135239	135615	135356, 135295, 135349	10, 10, 5	9.18236, 9.27513, 5.59755	6.23091, 6.2868, 4.38538	6.47811, 6	3	pkhd1l1	0
GL173599.1	36594	37085	36622, 36612, 36622	10, 23, 11	11.06011, 24.86003, 14.75772	7.306, 12.38952, 9.09568	8.22712, 2	3	GHDC	19994
GL173599.1	77789	78260	77816, 77900, 77856	13, 18, 10	15.29169, 19.97713, 13.18184	9.29854, 10.97931, 8.33771	12.24396,	3	NA	11242
GL173599.1	99306	99925	99337, 99337, 99341	34, 46, 13	41.806, 52.00807, 16.0859	18.30935, 18.93363, 9.70995	38.07516,	3	NA	0
GL173599.1	108878	109915	109736, 109656, 109181	11, 10, 8	12.43693, 9.39128, 10.1465	7.97018, 6.35644, 6.82176	9.52692, 6	3	NA	1778
GL173599.1	154941	155540	155506, 155515, 155513	63, 93, 53	79.3324, 112.28786, 70.60102	26.0864, 28.12012, 26.30292	75.14178,	3	KCNH4	32108
GL173599.1	193883	194321	194248, 194280, 194053	13, 16, 5	13.49269, 14.80681, 5.19074	8.29387, 8.40621, 4.18277	10.53271,	3	KCNH4	0
GL173599.1	211421	212084	211451, 211458, 211513	6, 12, 4	5.31708, 11.60251, 4.38152	4.2777, 7.3582, 3.65448	2.96727, 8	3	KCNH4	340
GL173599.1	217872	218351	218316, 218325, 218325	120, 175, 103	152.73091, 221.25728, 139.95694	34.04447, 37.19793, 36.99145	147.99243	3	KCNH4	6791
GL173600.1	79780	80018	79859, 79938, 79966	13, 21, 6	9.03179, 13.73307, 4.41214	5.56973, 6.4985, 3.71621	6.33776, 1	3	NA	41342
GL173600.1	153694	154336	153919, 153911, 153939, 154276	18, 25, 6, 6	17.31602, 22.12709, 5.2665, 5.29847	9.39018, 10.0929, 4.24757, 4.26663	14.18471,	3	NA	18271
GL173601.1	2055	3139	2480, 2481, 2459	54, 78, 54	65.77729, 95.74834, 73.49598	22.98191, 27.24326, 27.47997	61.72517,	3	morc3	204977
GL173601.1	11403	12046	11874, 11763, 11792	12, 22, 18	13.8483, 25.85271, 24.2813	8.63436, 13.29074, 13.31378	10.86694,	3	morc3	196070
GL173601.1	22144	22564	22147, 22165, 22147	11, 20, 5	12.43693, 22.87459, 5.9506	7.97018, 12.13503, 4.54784	9.52692, 1	3	morc3	185552
GL173601.1	66579	66984	66958, 66927, 66940	17, 35, 15	21.19088, 44.58944, 20.98137	11.87339, 19.52904, 11.95164	17.92307,	3	morc3	141132
GL173601.1	103906	104330	103935, 103939, 103934	66, 55, 62	90.50413, 75.56123, 88.48964	30.88143, 28.16566, 31.94721	86.20287,	3	morc3	103786
GL173601.1	233981	234407	234374, 234186, 234391	6, 10, 6	4.51306, 6.99366, 5.23492	3.78094, 4.86542, 4.22867	2.30616, 4	3	chaf1b	3765
GL173603.1	1697	2485	1819, 2113, 1980	14, 16, 11	16.7649, 17.16856, 13.46208	9.96273, 9.82359, 8.49639	13.6543, 1	3	NA	37258
GL173603.1	28815	29138	28948, 28903, 28939	7, 9, 15	6.37715, 7.28344, 18.53291	4.86324, 5.20811, 10.76715	3.90257, 4	3	NA	10605
GL173603.1	88707	89088	88925, 88907, 89016	11, 17, 5	10.9046, 15.35073, 4.70718	7.10903, 8.4724, 3.9211	8.08987, 1	3	znf184	23017
GL173603.1	89482	90427	90067, 90253, 90125	9, 12, 11	8.66407, 10.03748, 12.34038	6.04743, 6.40131, 7.91841	5.99101, 7	3	znf184	21678
GL173603.1	95544	96195	95832, 95747, 95928	4, 11, 8	3.74195, 10.60895, 10.0913	3.32091, 6.9343, 6.79758	1.6222, 7.9	3	znf184	15910
GL173603.1	110688	111441	111018, 110878, 110939	13, 13, 13	12.43975, 10.89905, 15.34872	7.66875, 6.72447, 9.3288	9.5289, 8.2	3	znf184	664
GL173603.1	132412	133009	132940, 132695, 132895	7, 9, 8	6.12952, 6.15408, 8.14528	4.71533, 4.4908, 5.82558	3.67231, 3	3	znf184	0
GL173603.1	166812	168220	167608, 167370, 167620	14, 18, 6	13.44866, 15.09059, 6.08001	8.06455, 8.04034, 4.71063	10.49073,	3	znf184	0
GL173603.1	170219	170670	170413, 170377, 170389	7, 14, 8	5.62168, 11.62806, 8.98772	4.40288, 6.94888, 6.27416	3.24345, 8	3	znf184	0
GL173607.1	1609	3111	2126, 2061, 2076	24, 23, 18	25.90685, 23.18973, 19.15771	12.67883, 11.38786, 10.49722	22.50036,	3	dtnb	13250
GL173607.1	68691	69224	68746, 68753, 68902	7, 14, 4	7.1696, 13.20621, 4.15126	5.31345, 7.91755, 3.54016	4.61046, 1	3	dtnb	0
GL173607.1	174463	174896	174532, 174476, 174563	6, 18, 4	5.77068, 17.45593, 3.85136	4.54016, 9.47501, 3.38149	3.37745, 1	3	dnmt3a	0
GL173607.1	250961	251999	251287, 251764, 251020	6, 7, 6	5.96715, 5.97725, 7.29403	4.64927, 4.62287, 5.30581	3.52158, 3	3	dpysl5	0
GL173607.1	266437	266911	266783, 266790, 266715	19, 25, 15	22.57464, 29.44854, 21.38372	12.222, 14.43793, 12.12757	19.26377,	3	dpysl5	4047
GL173609.1	43033	43524	43473, 43367, 43309	9, 16, 9	8.40425, 15.41438, 10.56949	5.89416, 8.77591, 7.08032	5.76336, 1	3	crtap	9030
GL173609.1	190419	190892	190726, 190563, 190727	16, 18, 9	19.7934, 19.97713, 11.64359	11.29109, 10.97931, 7.57973	16.57067,	3	itgb3	3245
GL173609.1	227402	227821	227491, 227629, 227480	7, 6, 4	6.16939, 4.93312, 4.67325	4.73936, 4.04501, 3.78987	3.70916, 2	3	rprml	4159
GL173611.1	211879	212952	212347, 212390, 212231	6, 10, 6	5.96715, 9.39128, 7.29403	4.64927, 6.35644, 5.30581	3.52158, 6	3	rasgrf2	0
GL173611.1	211879	213147	213082, 212390, 212231	4, 10, 6	3.74195, 9.39128, 7.29403	3.32091, 6.35644, 5.30581	1.6222, 6.8	3	rasgrf2	0
GL173612.1	5817	6441	6045, 6269, 6121	7, 10, 5	6.99564, 9.39128, 5.06966	5.21786, 6.35644, 4.11933	4.47307, 6	3	NA	2180
GL173612.1	20483	21214	20619, 20679, 20668	10, 15, 6	11.06011, 15.26186, 7.29403	7.306, 8.92711, 5.30581	8.22712, 1	3	NA	111
GL173612.1	75093	76215	75392, 75965, 75843	16, 9, 9	16.68158, 7.55109, 9.35579	9.53667, 5.37505, 6.44216	13.57813,	3	NA	4501
GL173612.1	87880	88270	88132, 88108, 88109	8, 29, 13	7.62407, 28.35337, 16.0859	5.52896, 12.33938, 9.70995	5.03638, 2	3	NA	17288
GL173612.1	146721	147210	147038, 147049, 146731	15, 15, 5	16.36908, 14.09492, 5.9506	9.57629, 8.22351, 4.54784	13.27778,	3	NA	7972
GL173612.1	182101	182602	182331, 182324, 182300	21, 26, 10	23.41865, 29.61081, 11.24131	12.1655, 14.1878, 7.40241	20.08217,	3	rrm1	0
GL173612.1	201670	202056	201957, 201900, 201923	12, 18, 12	12.45843, 16.81975, 13.01912	7.86167, 9.08887, 8.18077	9.54648, 1	3	rrm1	0
GL173612.1	218633	219194	218885, 218894, 218945	9, 25, 14	6.66833, 22.47805, 13.12965	4.8193, 10.29723, 7.871	4.17154, 1	3	ZAR1L	3095
GL173613.1	119852	120479	120099, 120408, 119976	5, 11, 8	4.82177, 9.66539, 10.1465	3.98509, 6.36265, 6.82176	2.51418, 7	3	psd	26199
GL173614.1	81364	82356	81807, 81959, 81917	17, 24, 17	13.43767, 19.76677, 17.32537	7.31117, 9.04653, 9.66937	10.48055,	3	NA	2824
GL173614.1	117281	118123	117731, 117502, 117312	8, 11, 4	7.06928, 8.37597, 4.67325	5.19933, 5.5591, 3.78987	4.5414, 5.8	3	pcmtd2	3504
GL173614.1	146358	146821	146447, 146522, 146422	8, 14, 6	7.67464, 14.45897, 7.29403	5.55832, 8.66788, 5.30581	5.08299, 1	3	NA	3036
GL173614.1	201583	202001	201940, 201954, 201958	5, 9, 5	4.82177, 8.21099, 5.9506	3.98509, 5.77858, 4.54784	2.51418, 5	3	myt1	15271
GL173615.1	139551	140930	139932, 140153, 140277	14, 10, 9	11.61573, 6.99366, 9.24728	6.94127, 4.86542, 6.38161	8.75458, 4	3	NA	NA
GL173615.1	153980	154424	154203, 154247, 154390	8, 9, 8	6.08111, 5.70245, 7.67847	4.58437, 4.20146, 5.56054	3.62785, 3	3	NA	NA
GL173615.1	196512	197045	196737, 196855, 196819	8, 12, 9	6.7444, 9.09789, 8.88966	5.00058, 5.81578, 6.17838	4.24113, 6	3	NA	NA
GL173615.1	208310	209080	208465, 208471, 208522, 208904	13, 18, 5, 4	10.21914, 14.27393, 4.14842, 3.25394	6.30293, 7.55037, 3.59261, 3.03398	7.4427, 11	3	NA	NA
GL173616.1	45	1445	459, 820, 850	16, 30, 14	16.15907, 31.50057, 16.29325	9.22513, 13.81575, 9.7093	13.07749,	3	TEX2	3776
GL173616.1	87502	88123	87945, 88070, 87779	17, 12, 6	20.70049, 11.86108, 7.25145	11.60995, 7.51216, 5.287	17.44812,	3	pecam1	12050
GL173616.1	102116	102907	102563, 102555, 102560	41, 59, 38	44.32947, 62.14349, 44.44731	16.76657, 18.66244, 18.15025	40.56007,	3	NA	9822
GL173616.1	147125	147552	147219, 147197, 147251	9, 11, 8	8.35434, 8.89461, 9.92329	5.86444, 5.88422, 6.7228	5.71584, 6	3	ddx5	3713
GL173616.1	153310	154503	153879, 153704, 153692	9, 17, 15	8.16099, 15.07756, 16.15968	5.74847, 8.30587, 9.45524	5.53692, 1	3	cep95	3462
GL173616.1	153310	155619	154501, 153704, 153692, 154972	8, 17, 15, 10	6.90289, 15.07756, 16.15968, 9.73055	5.09802, 8.30587, 9.45524, 6.55755	4.38737, 1	3	cep95	2346
GL173616.1	242386	243228	242676, 242958, 242959	11, 22, 20	9.48173, 17.52358, 19.031	6.24938, 8.34854, 9.84066	6.76069, 1	3	SMURF2	12280
GL173616.1	249135	249985	249623, 249594, 249601	41, 47, 29	47.16847, 51.84917, 32.28143	18.40985, 18.38979, 14.66534	43.35748,	3	SMURF2	19029
GL173616.1	275129	275851	275438, 275441, 275455	17, 25, 13	17.80138, 28.99223, 17.40688	9.95359, 14.16979, 10.34389	14.65165,	3	kpna2	3467
GL173616.1	288355	288957	288482, 288409, 288454	12, 8, 6	13.8483, 7.07159, 7.29403	8.63436, 5.20073, 5.30581	10.86694,	3	kpna2	2413
GL173617.1	145527	146530	146025, 146020, 146010	923, 1188, 541	763.03613, 1001.84167, 425.57147	16.77673, 17.55681, 14.99443	756.73541	3	NA	19473
GL173618.1	62488	63292	63006, 63136, 63196	9, 9, 4	9.72082, 8.21099, 4.67325	6.64182, 5.77858, 3.78987	6.97155, 5	3	znf300	0
GL173618.1	162183	162729	162635, 162334, 162519	7, 10, 5	5.10557, 7.4213, 4.51488	4.075, 5.13639, 3.81106	2.77482, 4	3	U2	21221
GL173618.1	209726	209975	209847, 209781, 209867	6, 18, 4	5.96715, 19.97713, 4.67325	4.64927, 10.97931, 3.78987	3.52158, 1	3	XB-GENE-996645	15926
GL173618.1	222676	223419	222994, 223407, 223349	7, 18, 8	5.42862, 16.57986, 8.04677	4.2813, 8.94309, 5.77057	3.06982, 1	3	NA	2964
GL173618.1	248179	249023	248735, 248672, 248836	5, 20, 7	4.82177, 21.61088, 8.69489	3.98509, 11.39194, 6.06379	2.51418, 1	3	wdr73	0
GL173620.1	232	1616	1122, 520, 276, 998	14, 22, 4, 5	12.64394, 18.98967, 4.67325, 4.99237	7.57426, 9.20223, 3.78987, 4.0781	9.72408, 1	3	NA	175868
GL173620.1	4781	5918	5474, 5291, 5333	11, 15, 8	7.89996, 9.00797, 7.26744	5.25996, 5.1542, 5.31857	5.29303, 6	3	NA	171566
GL173620.1	7898	8616	8273, 8010, 8041	10, 15, 8	8.84451, 12.49102, 8.04677	6.02544, 7.24068, 5.77057	6.16137, 9	3	NA	168868
GL173620.1	14332	18954	14885, 18138, 14787, 16158, 18009	19, 26, 8, 6, 10	13.0093, 16.17773, 6.83213, 4.38967, 8.18963	6.56431, 6.6389, 5.05462, 3.70174, 5.62006	10.07255,	3	NA	158530
GL173620.1	14332	18954	18682, 18138, 14787, 16158, 18009	15, 26, 8, 6, 10	8.15176, 16.17773, 6.83213, 4.38967, 8.18963	4.67129, 6.6389, 5.05462, 3.70174, 5.62006	5.52906, 1	3	NA	158530
GL173620.1	20628	21436	20952, 20880, 21224	10, 18, 8	6.01276, 12.14952, 7.81204	4.24549, 6.31114, 5.63749	3.56347, 9	3	NA	156048
GL173620.1	26990	27833	27558, 27618, 27624	8, 17, 7	8.42257, 18.56115, 8.69489	5.97763, 10.40145, 6.06379	5.76336, 1	3	NA	149651
GL173620.1	33729	37179	34928, 34770, 34933, 36744	18, 25, 10, 6	15.51535, 19.12602, 10.30677, 5.1729	8.29699, 8.39863, 6.89026, 4.19138	12.45808,	3	NA	140305
GL173620.1	47939	48844	48592, 48404, 48202	9, 20, 17	7.84477, 17.71959, 18.80835	5.5562, 9.04982, 10.54536	5.24227, 1	3	NA	128640
GL173620.1	157405	157982	157674, 157731, 157781	17, 19, 6	17.33887, 16.10778, 5.71639	9.67746, 8.37295, 4.50951	14.20638,	3	NA	19502
GL173622.1	350	631	456, 421, 492	13, 11, 10	9.84003, 7.11569, 8.84916	6.06799, 4.76866, 6.02829	7.08478, 4	3	NA	13859
GL173624.1	167353	167954	167788, 167518, 167586	10, 10, 7	10.01299, 8.61706, 7.72582	6.7221, 5.8856, 5.6058	7.24763, 6	3	NA	NA
GL173625.1	33826	34387	34085, 34010, 34008	46, 44, 18	46.41629, 46.1997, 18.80607	15.82822, 16.53868, 10.28795	42.61673,	3	usp21	196
GL173625.1	62664	64156	63215, 63230, 63028	22, 34, 18	19.45007, 29.42076, 17.71996	9.47443, 11.18275, 9.63487	16.23965,	3	ppox	8232
GL173625.1	79873	80400	79981, 79981, 79982	19, 26, 21	14.62897, 19.51425, 21.3191	7.49517, 8.33186, 10.9117	11.61323,	3	dnz1	0
GL173625.1	83551	83869	83812, 83816, 83807	39, 52, 17	41.0697, 55.27423, 17.78648	15.75185, 18.06483, 9.94474	37.34998,	3	dnz1	0
GL173625.1	119482	119994	119788, 119707, 119762	13, 12, 10	13.49269, 10.39632, 10.97191	8.29387, 6.6236, 7.25854	10.53271,	3	adamts4	4291
GL173625.1	242375	243009	242789, 242796, 242905	8, 12, 12	6.63031, 10.2136, 12.38374	4.92991, 6.51056, 7.81847	4.13604, 7	3	NA	24157
GL173625.1	243709	244802	244149, 243811, 244443	12, 9, 11	11.32055, 7.3205, 11.31465	7.18871, 5.23132, 7.3472	8.47391, 4	3	NA	25491
GL173626.1	14490	14967	14741, 14701, 14658	12, 11, 5	10.48835, 8.13814, 4.28214	6.6804, 5.40965, 3.6736	7.69782, 5	3	mpped1	10890
GL173626.1	15402	16306	15971, 15958, 15971	12, 14, 10	10.53646, 11.62806, 10.30677	6.71006, 6.94888, 6.89026	7.74314, 8	3	mpped1	9551
GL173626.1	46504	47371	46722, 46547, 46772	11, 11, 4	11.42621, 10.00475, 4.34116	7.41109, 6.57041, 3.63492	8.57514, 7	3	efcab6	417
GL173626.1	72956	73389	73128, 73008, 73060	21, 10, 10	20.73342, 8.79469, 11.6791	10.5587, 5.99491, 7.62925	17.48034,	3	efcab6	0
GL173626.1	78512	79896	79253, 78986, 78954	17, 15, 12	16.4953, 13.21475, 13.08717	9.16875, 7.68511, 8.21885	13.39926,	3	efcab6	0
GL173626.1	88068	89756	88632, 89007, 87994, 89096	11, 10, 6, 5	10.9046, 8.57382, 6.35308, 5.31857	7.10903, 5.8589, 4.85498, 4.24819	8.08987, 6	3	efcab6	0
GL173626.1	104862	105281	105170, 104986, 105136	15, 14, 7	13.97429, 10.68957, 6.88081	8.15, 6.3706, 5.15373	10.98644,	3	efcab6	0
GL173626.1	109615	109961	109802, 109789, 109805	13, 12, 8	11.35857, 8.8556, 7.85773	7.00844, 5.66466, 5.66361	8.51036, 6	3	sult4a1	348
GL173626.1	162359	163122	162832, 162780, 162732	10, 23, 12	8.32267, 20.19511, 12.7555	5.70305, 9.60233, 8.03189	5.6878, 17	3	parvb	0
GL173626.1	195116	196153	195583, 195870, 195876	8, 13, 6	5.45331, 9.53929, 5.46454	4.1814, 5.88205, 4.36458	3.09249, 6	3	PARVG	0
GL173626.1	233948	234549	234480, 234491, 234522	11, 21, 17	11.85906, 20.53474, 19.99824	7.65504, 10.43888, 11.2232	8.98595, 1	3	PARVG	34335
GL173627.1	27557	28358	27668, 27651, 28258, 27708	20, 19, 12, 7	20.44929, 14.6632, 7.54492, 6.83761	10.69687, 7.51524, 4.85383, 5.12939	17.20509,	3	adamts12	0
GL173627.1	98325	98823	98443, 98530, 98466	13, 14, 11	12.26307, 11.94813, 14.68355	7.56188, 7.1463, 9.06344	9.36972, 9	3	adamts12	0
GL173627.1	107789	108511	108329, 108181, 108105	9, 13, 5	8.82885, 10.98869, 5.14967	6.14328, 6.77996, 4.16141	6.14693, 8	3	adamts12	0
GL173627.1	141907	142880	142708, 142745, 142775	15, 18, 8	16.90423, 18.60217, 10.00633	9.88146, 10.16604, 6.75998	13.78827,	3	adamts12	15908
GL173627.1	183227	183639	183580, 183587, 183603	15, 27, 13	16.03559, 25.38503, 15.89249	9.3831, 11.31845, 9.61177	12.95858,	3	tars	0
GL173627.1	197168	198136	197589, 197626, 197661	13, 6, 7	13.27928, 4.93312, 8.49722	8.16901, 4.04501, 5.97582	10.32936,	3	tars	0
GL173627.1	225856	226883	226517, 226133, 225981	6, 11, 7	5.24248, 9.09572, 6.01639	4.2332, 6.00975, 4.64674	2.89962, 6	3	tars	11968
GL173628.1	14121	14631	14143, 14152, 14148	39, 59, 64	47.11081, 70.06207, 89.33206	19.2772, 22.91245, 31.32387	43.30072,	3	NA	120050
GL173628.1	24212	24742	24223, 24220, 24292	45, 54, 28	51.82819, 60.08271, 35.61161	19.29056, 19.7804, 16.98975	47.95054,	3	NA	109939
GL173628.1	193146	193499	193353, 193291, 193251	11, 11, 4	12.43693, 10.60895, 4.67325	7.97018, 6.9343, 3.78987	9.52692, 7	3	NA	15059
GL173628.1	210765	211864	211426, 211507, 211728	22, 22, 12	21.61793, 20.18456, 13.01912	10.77188, 9.9117, 8.18077	18.33579,	3	NA	0
GL173629.1	130794	131710	130901, 131344, 131177	8, 12, 8	7.57423, 9.82596, 8.14528	5.4999, 6.26981, 5.82558	4.98865, 7	3	NA	0
GL173629.1	147876	149200	147993, 148903, 148944	6, 14, 9	4.86712, 11.90127, 10.42289	4.00414, 7.11741, 7.00726	2.55558, 9	3	NA	12394
GL173629.1	148089	149200	148604, 148903, 148944	10, 14, 9	9.661, 11.90127, 10.42289	6.51661, 7.11741, 7.00726	6.9283, 9.1	3	NA	12607
GL173629.1	155903	156312	156120, 156023, 156098	7, 11, 7	7.1696, 10.60895, 8.69489	5.31345, 6.9343, 6.06379	4.61046, 7	3	NA	20421
GL173629.1	222406	222773	222442, 222416, 222439	9, 9, 5	9.72082, 8.21099, 5.9506	6.64182, 5.77858, 4.54784	6.97155, 5	3	NA	6341
GL173630.1	39759	40303	40184, 40177, 40118	12, 15, 5	13.73771, 13.16372, 5.40789	8.57523, 7.65379, 4.29296	10.76705,	3	NA	11568
GL173631.1	116399	117079	116455, 116450, 116433	23, 28, 17	23.25035, 28.23463, 18.57687	11.42428, 12.63655, 10.41063	19.91801,	3	xtr-mir-139	16887
GL173631.1	128611	129567	128664, 128865, 129398	8, 10, 4	7.77807, 8.20397, 4.38152	5.618, 5.62902, 3.65448	5.17956, 5	3	xtr-mir-139	4399
GL173631.1	162759	163410	162913, 162936, 163253	6, 5, 13	5.96715, 3.94561, 14.69628	4.64927, 3.46715, 8.97644	3.52158, 1	3	pde2a	0
GL173634.1	12529	13323	13289, 13151, 13286	10, 19, 8	9.77513, 18.8972, 8.9249	6.5837, 10.05916, 6.24211	7.02302, 1	3	NA	30287
GL173635.1	8742	9353	8851, 8850, 8872	9, 8, 6	8.66407, 5.96021, 5.99481	6.04743, 4.50731, 4.6644	5.99101, 3	3	NA	NA
GL173635.1	11912	12814	12507, 12298, 12076	12, 20, 12	12.18267, 19.57575, 12.62857	7.70145, 10.16993, 7.95947	9.29304, 1	3	NA	NA
GL173635.1	224033	224868	224427, 224474, 224589	10, 9, 8	11.06011, 8.21099, 10.1465	7.306, 5.77858, 6.82176	8.22712, 5	3	NA	NA
GL173636.1	204	2083	1284, 1184, 1272	30, 27, 15	33.70974, 25.58706, 17.23823	15.12793, 11.43661, 10.06864	30.12225,	3	NA	NA
GL173636.1	31663	32130	32103, 32089, 32103	78, 86, 61	96.50127, 102.94474, 81.25791	27.64575, 26.91788, 28.27289	92.14066,	3	NA	NA
GL173636.1	40110	40534	40147, 40148, 40129	40, 56, 29	30.3957, 46.52064, 25.6955	9.86485, 12.42746, 10.8122	26.8792, 4	3	NA	NA
GL173636.1	43539	43949	43915, 43920, 43904	123, 120, 85	144.53987, 134.37613, 108.23565	28.93702, 25.99483, 30.19367	139.8439,	3	NA	NA
GL173636.1	127790	128072	127983, 127963, 127913	5, 18, 5	4.47183, 17.00494, 4.99237	3.78598, 9.20137, 4.0781	2.26935, 1	3	NA	NA
GL173636.1	128276	129005	128687, 128687, 128504	14, 22, 11	15.84929, 22.77797, 12.78973	9.46495, 11.46975, 8.15619	12.77923,	3	NA	NA
GL173636.1	147204	147642	147208, 147208, 147232	10, 19, 16	9.23294, 18.02027, 20.29858	6.26141, 9.5284, 11.55461	6.52626, 1	3	NA	NA
GL173637.1	15512	15945	15887, 15617, 15944	7, 9, 3	7.1696, 8.21099, 3.34298	5.31345, 5.77858, 2.97156	4.61046, 5	3	sox6	0
GL173637.1	31065	31579	31491, 31528, 31548	22, 35, 18	23.44815, 36.39152, 21.49679	11.87164, 14.70421, 11.84671	20.11079,	3	sox6	0
GL173637.1	38129	38405	38264, 38227, 38217	9, 15, 9	8.35434, 12.81802, 10.42289	5.86444, 7.4415, 7.00726	5.71584, 1	3	sox6	0
GL173637.1	112398	113149	112948, 112447, 112446	5, 11, 5	4.82177, 9.90518, 5.9506	3.98509, 6.50968, 4.54784	2.51418, 7	3	sox6	17873
GL173637.1	128286	130502	129556, 129288, 129398	17, 20, 9	13.26996, 15.02475, 8.47365	7.21042, 7.46108, 5.93535	10.32054,	3	sox6	33761
GL173637.1	150369	150698	150677, 150668, 150635	25, 27, 18	25.0899, 27.84104, 19.75651	11.84301, 12.76965, 10.85036	21.70687,	3	sox6	55844
GL173637.1	154382	155483	155048, 154486, 155184	15, 9, 8	12.34004, 6.40439, 7.67847	7.148, 4.65094, 5.56054	9.44153, 4	3	sox6	59857
GL173638.1	220448	222205	221001, 220995, 220996, 222099	14, 18, 9, 8	12.13106, 15.28549, 9.76105, 8.56955	7.25904, 8.15798, 6.66343, 6.05652	9.24462, 1	3	NA	132532
GL173638.1	228850	229714	229286, 229285, 229280	37, 45, 27	44.63541, 50.06706, 34.50341	18.70088, 18.27985, 16.69193	40.86146,	3	NA	140934
GL173640.1	130769	131059	130881, 130902, 130995	19, 28, 21	17.87921, 25.74967, 25.2565	9.44292, 11.1835, 13.23126	14.72728,	3	NA	47
GL173640.1	186344	187086	186706, 186708, 186525	8, 16, 10	6.27633, 13.5443, 11.03772	4.70812, 7.63435, 7.29398	3.80887, 1	3	NA	48217
GL173640.1	196046	196655	196262, 196304, 196465	9, 5, 4	7.67537, 3.52186, 4.67325	5.45199, 3.19576, 3.78987	5.08317, 1	3	NA	57919
GL173640.1	242426	243017	242831, 242739, 242877	25, 21, 13	27.82794, 22.22847, 15.70541	13.47995, 11.45784, 9.51556	24.37199,	3	NA	104299
GL173642.1	140100	141029	140228, 140420, 140565	16, 15, 8	16.15907, 12.13791, 9.11678	9.22513, 7.02404, 6.33924	13.07749,	3	NA	47633
GL173643.1	67640	68026	67683, 67696, 67678	18, 24, 7	22.92104, 26.09829, 8.69489	12.61945, 12.79322, 6.06379	19.59912,	3	XB-GENE-5794232	70498
GL173644.1	38454	39066	38850, 38878, 38648	14, 17, 7	14.3105, 14.86656, 8.69489	8.58029, 8.17729, 6.06379	11.30855,	3	NA	5287
GL173645.1	140714	141497	141117, 141299, 141086	8, 16, 7	7.38183, 14.46749, 8.35502	5.38666, 8.19898, 5.9108	4.80856, 1	3	NA	23663
GL173645.1	159738	160709	160046, 159861, 160009	6, 17, 4	5.63898, 14.26997, 4.67325	4.46544, 7.81436, 3.78987	3.25772, 1	3	NA	4451
GL173646.1	84	3667	1497, 1319, 1315	54, 85, 35	38.73137, 58.12905, 27.05001	9.8652, 10.06073, 9.66558	35.04912,	3	miga2	133344
GL173646.1	13251	14177	13464, 13602, 13904	11, 19, 4	10.96672, 19.56704, 4.46488	7.14544, 10.46264, 3.69425	8.14942, 1	3	miga2	122834

GL173646.1	16382	17211	16817, 16868, 16863	9, 8, 6	7.71701, 5.75617, 5.99481	5.47767, 4.3766, 4.6644	5.12244, 3	3	miga2	119800
GL173646.1	43073	44213	43942, 43684, 43925	15, 24, 7	16.20017, 25.37763, 7.61369	9.47871, 12.36208, 5.54854	13.11636,	3	miga2	92798
GL173647.1	26563	27330	26873, 26884, 26739	5, 8, 4	4.82177, 7.07159, 4.67325	3.98509, 5.20073, 3.78987	2.51418, 4	3	5S_rRNA	72060
GL173647.1	99530	100772	100170, 100190, 100032	189, 257, 134	198.85918, 275.61316, 148.94156	24.92805, 27.15148, 26.27288	193.87567	3	5S_rRNA	0
GL173647.1	108301	109917	108729, 108918, 108762	115, 146, 105	116.62686, 146.04898, 117.5385	21.06656, 21.47795, 25.08707	112.10446	3	5S_rRNA	0
GL173647.1	146296	148410	146570, 146569, 147714, 146659	9, 10, 8, 6	7.84477, 7.8291, 5.93025, 5.43047	5.5562, 5.39387, 4.48816, 4.34463	5.24227, 5	3	NA	23951
GL173647.1	171795	172189	172047, 172072, 171985	28, 30, 21	21.09709, 23.90068, 18.85876	8.61819, 9.51618, 9.43063	17.83253,	3	NA	172
GL173647.1	173793	174139	173946, 173934, 173905	34, 29, 17	30.48098, 25.89229, 15.28913	11.75293, 10.92318, 8.43485	26.96282,	3	NA	273
GL173647.1	191077	191889	191204, 191204, 191200	181, 170, 131	198.11438, 172.14415, 150.47078	26.98397, 22.59404, 28.01783	193.13399	3	NA	14952
GL173647.1	198429	199246	198563, 198541, 198562	80, 123, 71	33.76366, 59.82008, 39.34539	5.37204, 6.59987, 7.2979	30.17518,	3	NA	7595
GL173647.1	200866	205006	202585, 202585, 202585	210, 267, 142	127.12533, 161.08635, 92.30315	9.2297, 9.31433, 9.94962	122.52891	3	NA	1835
GL173647.1	219646	220814	220660, 220520, 220679	19, 37, 36	18.4025, 32.29327, 41.00183	9.75995, 11.69558, 16.98661	15.22838,	3	NA	9318
GL173648.1	18125	18596	18431, 18427, 18331	12, 24, 13	13.55951, 27.82525, 17.924	8.47903, 13.81726, 10.57401	10.59689,	3	NA	NA
GL173648.1	58440	58897	58500, 58512, 58594	15, 22, 7	14.71973, 22.45914, 7.84208	8.60219, 11.27809, 5.66426	11.70031,	3	NA	NA
GL173648.1	71558	72489	72378, 71853, 72059	24, 20, 17	25.81478, 18.71921, 19.81591	12.62377, 9.65224, 11.12108	22.41065,	3	NA	NA
GL173648.1	211071	211695	211176, 211218, 211478	12, 8, 10	13.38718, 7.07159, 13.18184	8.38496, 5.20073, 8.33771	10.43197,	3	NA	NA
GL173649.1	93550	94165	93993, 93831, 94101	8, 9, 7	6.55639, 5.97124, 5.82684	4.88389, 4.37367, 4.53047	4.06705, 3	3	NA	NA
GL173649.1	110993	111294	111242, 111271, 111253	12, 26, 11	9.49573, 19.11224, 10.82241	6.06403, 8.11843, 7.0607	6.77365, 1	3	NA	NA
GL173650.1	34729	35001	34977, 34897, 34964	8, 15, 9	6.8227, 12.13791, 9.58224	5.04883, 7.02404, 6.56676	4.31393, 9	3	ucp2	0
GL173650.1	52430	53070	52791, 52753, 52912	11, 9, 10	8.43721, 6.49199, 10.59656	5.59757, 4.70689, 7.05292	5.77761, 4	3	ucp2	13592
GL173650.1	58241	58784	58571, 58508, 58551	31, 32, 16	32.87743, 34.33575, 18.69936	14.24129, 14.70709, 10.69793	29.30683,	3	c2cd3	9353
GL173650.1	128644	128970	128743, 128792, 128745	23, 24, 12	22.64709, 23.92411, 11.40764	11.06201, 11.49076, 7.24123	19.33432,	3	ppme1	717
GL173650.1	135354	136048	135698, 135573, 135469	11, 15, 5	7.59564, 9.08252, 3.27287	5.06888, 5.19699, 3.03127	5.00893, 6	3	p4ha3	1570
GL173650.1	136522	136982	136738, 136649, 136647	21, 37, 16	18.35044, 33.26746, 15.82635	9.12717, 12.20773, 9.02508	15.17856,	3	p4ha3	636
GL173650.1	173331	173902	173779, 173542, 173564	6, 11, 7	5.96715, 10.60895, 8.69489	4.64927, 6.9343, 6.06379	3.52158, 7	3	NA	1946
GL173650.1	188528	188929	188733, 188702, 188709	24, 48, 28	23.67742, 44.72785, 31.43825	11.34307, 14.18828, 14.54294	20.33353,	3	NA	17143
GL173652.1	61038	62020	61853, 61813, 61670	15, 12, 4	14.09249, 10.16897, 3.14704	8.22203, 6.4829, 2.96768	11.1007, 7	3	xtr-mir-1a-2	4541
GL173652.1	143743	144111	144082, 144029, 143991	10, 9, 7	9.44155, 6.9998, 7.40077	6.38646, 5.02958, 5.43746	6.72156, 4	3	NA	821
GL173652.1	160970	161290	161243, 161242, 161243	21, 23, 9	21.97388, 20.94885, 9.94831	11.30529, 10.04755, 6.76298	18.68096,	3	c20orf85	126
GL173652.1	189023	189559	189101, 189330, 189328	7, 12, 11	4.15603, 8.56154, 10.19222	3.45455, 5.48152, 6.68421	1.99613, 6	3	c20orf85	25445
GL173652.1	194434	194811	194607, 194731, 194638	11, 8, 3	9.26086, 6.73981, 3.37781	6.1125, 4.99774, 2.98791	6.5517, 4.3	3	c20orf85	30856
GL173652.1	218440	219375	218994, 219016, 218693	21, 22, 6	25.75943, 23.7165, 7.29403	13.51522, 12.03204, 5.30581	22.35693,	3	c20orf85	54862
GL173653.1	225993	226942	226473, 226399, 226509	6, 11, 4	5.96715, 10.60895, 4.67325	4.64927, 6.9343, 3.78987	3.52158, 7	3	rassf6	152674
GL173655.1	81413	82463	81942, 81966, 81880	20, 24, 11	20.68624, 25.12019, 12.34038	10.83932, 12.2078, 7.91841	17.4343, 2	3	NA	42688
GL173655.1	152133	152745	152317, 152594, 152645	6, 16, 7	5.96715, 17.16856, 8.69489	4.64927, 9.82359, 6.06379	3.52158, 1	3	NA	113408
GL173655.1	163471	163996	163732, 163807, 163757	8, 20, 5	7.02691, 21.69995, 5.9506	5.17362, 11.44483, 4.54784	4.50222, 1	3	NA	124746
GL173655.1	181507	182376	181988, 181764, 181690	9, 12, 7	8.16099, 10.25865, 8.15288	5.74847, 6.53846, 5.81587	5.53692, 7	3	NA	142782
GL173655.1	196554	196922	196723, 196771, 196741	20, 11, 3	20.84802, 9.09572, 3.47371	10.93641, 6.00975, 3.03189	17.59179,	3	NA	157829
GL173656.1	111344	111895	111605, 111605, 111737	7, 15, 9	6.33443, 13.21475, 8.88966	4.83795, 7.68511, 6.17838	3.86259, 1	3	nbeal2	0
GL173656.1	184160	184773	184654, 184663, 184665	55, 88, 54	62.63328, 101.29671, 65.9252	20.71938, 25.15015, 23.06691	58.6162, 9	3	ccdc12	8488
GL173656.1	215823	216094	216035, 216033, 216020	17, 34, 11	21.06519, 41.53215, 14.68355	11.80641, 18.14786, 9.06344	17.80185,	3	ccdc12	18589
GL173658.1	108017	109218	108562, 108491, 108562	16, 28, 9	15.87778, 29.84854, 10.01275	9.05608, 13.59546, 6.79683	12.8057, 2	3	XB-GENE-5760633	8151
GL173658.1	129166	129650	129599, 129623, 129614	57, 80, 41	68.29954, 98.09069, 53.48362	22.91779, 27.44911, 22.12428	64.22055,	3	XB-GENE-5760633	926
GL173658.1	148006	148387	148040, 148040, 148101	11, 22, 11	9.90875, 23.27801, 12.63525	6.51186, 11.76976, 8.07536	7.14919, 2	3	TMEM198	0
GL173658.1	175238	175510	175272, 175251, 175264	30, 52, 28	35.30015, 65.29349, 36.96613	16.07224, 23.71051, 17.75574	31.6812, 6	3	obsl1	0
GL173659.1	66387	66699	66524, 66633, 66554	5, 8, 4	4.40263, 6.89656, 4.67325	3.74533, 5.09414, 3.78987	2.21004, 4	3	XB-GENE-5905274	6084
GL173659.1	118310	118991	118635, 118875, 118874	6, 8, 9	5.96715, 7.07159, 11.48736	4.64927, 5.20073, 7.51109	3.52158, 4	3	XB-GENE-5846549	2140
GL173659.1	230954	231537	231452, 231461, 231199	13, 26, 16	12.56081, 25.40202, 16.0646	7.74168, 11.67835, 9.16845	9.64511, 2	3	NA	7155
GL173660.1	24174	24739	24461, 24284, 24468	11, 11, 4	12.43693, 10.60895, 4.67325	7.97018, 6.9343, 3.78987	9.52692, 7	3	NA	0
GL173660.1	43215	43984	43860, 43532, 43680	6, 11, 5	5.96715, 10.60895, 5.91431	4.64927, 6.9343, 4.53172	3.52158, 7	3	nisch	0
GL173660.1	88608	89277	88975, 88817, 89122	13, 13, 15	9.73159, 10.2718, 12.70238	6.00089, 6.33559, 7.37048	6.98212, 7	3	nisch	0
GL173660.1	93853	94400	93933, 94116, 93972	13, 20, 7	10.17993, 15.72353, 4.58378	6.27862, 7.86582, 3.73593	7.40619, 1	3	uqcrc1	0
GL173660.1	113082	114225	113179, 113765, 113759	18, 28, 18	18.07742, 29.4016, 19.75651	9.85073, 13.32928, 10.85036	14.9177, 2	3	uqcrc1	6688
GL173660.1	127424	128002	127595, 127739, 127568	6, 9, 5	5.95712, 8.21099, 5.50079	4.64377, 5.77858, 4.33868	3.52158, 5	3	NA	3208
GL173660.1	163178	163841	163527, 163378, 163355	10, 19, 9	10.26482, 19.64509, 10.56949	6.86644, 10.50948, 7.08032	7.48624, 1	3	U4	5917
GL173660.1	193802	194645	194549, 194549, 194538	107, 148, 126	103.60004, 142.82895, 140.66539	18.96216, 20.02533, 26.13944	99.18005,	3	U4	21695
GL173660.1	213073	213700	213301, 213406, 213230	7, 10, 7	7.1696, 9.39128, 8.69489	5.31345, 6.35644, 6.06379	4.61046, 6	3	U4	40966
GL173661.1	8397	9153	8984, 8971, 8970	19, 22, 18	19.10984, 20.88093, 21.30401	10.1875, 10.32883, 11.73892	15.91125,	3	NA	6447
GL173663.1	4168	4440	4171, 4198, 4171	34, 51, 34	41.94645, 63.81345, 45.89866	18.3921, 23.35214, 20.68333	38.2133, 5	3	NA	11091
GL173663.1	82815	83542	83340, 82947, 82901	8, 12, 8	7.83097, 10.30411, 8.1956	5.64832, 6.56659, 5.85348	5.22917, 7	3	NA	12138
GL173663.1	203861	204112	204085, 204065, 204081	24, 27, 16	25.27938, 28.00888, 20.29858	12.30321, 12.86965, 11.55461	21.89126,	3	NA	103324
GL173663.1	206428	206860	206697, 206721, 206630	13, 18, 6	10.98084, 15.90393, 6.08001	6.7751, 8.53251, 4.71063	8.16259, 1	3	NA	105891
GL173664.1	8765	9641	9097, 9500, 9276	9, 7, 5	7.93244, 4.89556, 4.91762	5.60981, 3.93927, 4.03768	5.32435, 2	3	NA	NA
GL173664.1	26041	26486	26282, 26434, 26431	7, 13, 7	7.1696, 13.14514, 8.08824	5.31345, 8.09002, 5.7849	4.61046, 1	3	NA	NA
GL173664.1	97142	97534	97501, 97506, 97506	20, 20, 12	20.44929, 21.01248, 14.23156	10.69687, 11.03495, 8.83588	17.20509,	3	NA	NA
GL173664.1	124636	125046	125002, 124983, 124827	5, 13, 5	4.82177, 13.14514, 5.9506	3.98509, 8.09002, 4.54784	2.51418, 1	3	NA	NA
GL173664.1	137323	137940	137397, 137354, 137358	9, 16, 7	9.72082, 17.06606, 8.69489	6.64182, 9.76351, 6.06379	6.97155, 1	3	NA	NA
GL173664.1	175773	176241	175963, 175963, 175963	8, 16, 9	7.1122, 14.57865, 11.64359	5.22529, 8.26691, 7.57973	4.57946, 1	3	NA	NA
GL173664.1	185903	187072	186987, 186987, 186962	14, 12, 7	13.14827, 10.63474, 7.50542	7.88233, 6.77054, 5.49244	10.20482,	3	NA	NA
GL173664.1	205851	207009	205902, 206334, 206120	8, 8, 5	8.42257, 6.37769, 5.54867	5.97763, 4.77199, 4.3619	5.76336, 4	3	NA	NA
GL173665.1	151774	152722	152449, 152456, 152463	28, 53, 12	31.291, 63.16079, 13.29687	14.45526, 21.97984, 8.33527	27.75414,	3	NA	0
GL173665.1	202385	202699	202573, 202548, 202605	20, 23, 13	11.46516, 10.88535, 9.01977	5.52112, 4.74761, 5.56237	8.6119, 8.2	3	NA	4954
GL173665.1	204169	204868	204723, 204429, 204437	16, 28, 14	8.22055, 15.38633, 10.25207	4.54136, 5.89566, 6.10251	5.59317, 1	3	NA	6738
GL173665.1	209715	210566	210034, 210046, 210232	23, 27, 14	22.64709, 24.18563, 14.66346	11.06201, 10.62335, 8.7878	19.33432,	3	NA	12284
GL173666.1	203192	203500	203341, 203228, 203276	4, 8, 3	3.34243, 5.81313, 3.47371	3.08796, 4.41317, 3.03189	1.41807, 3	3	NA	1835
GL173667.1	6645	6961	6852, 6722, 6895	8, 12, 4	8.22568, 11.86108, 4.67325	5.8701, 7.51216, 3.78987	5.59681, 9	3	NA	2219
GL173667.1	133361	133682	133540, 133590, 133548	12, 9, 9	13.8483, 8.21099, 11.64359	8.63436, 5.77858, 7.57973	10.86694,	3	SSPO	18621
GL173667.1	157523	157858	157649, 157754, 157646	15, 36, 11	14.65409, 39.73859, 11.49087	8.5626, 16.24309, 7.44794	11.63756,	3	NA	24647
GL173667.1	169867	170788	170510, 170114, 170220	8, 6, 5	7.62407, 4.62266, 5.9506	5.52896, 3.8507, 4.54784	5.03638, 2	3	NA	11717
GL173668.1	22286	22606	22551, 22510, 22356	6, 10, 5	5.81608, 7.93794, 5.9506	4.56563, 5.46232, 4.54784	3.4176, 5.4	3	NA	NA
GL173668.1	46550	47059	46889, 46714, 46686	9, 13, 5	7.71701, 11.17268, 5.14967	5.47767, 6.89372, 4.16141	5.12244, 8	3	NA	NA
GL173668.1	59288	60187	59808, 59697, 59695	5, 14, 7	4.82177, 11.11865, 6.75301	3.98509, 6.63466, 5.08141	2.51418, 8	3	NA	NA
GL173668.1	142459	142821	142751, 142761, 142757	10, 18, 11	9.33595, 15.04268, 10.66921	6.32332, 8.01145, 6.9701	6.62299, 1	3	NA	NA
GL173668.1	145798	147227	146228, 147181, 146031, 147174	10, 22, 12, 16	8.57531, 18.37326, 11.50726, 15.94441	5.85982, 8.84063, 7.30112, 9.0962	5.90712, 1	3	NA	NA
GL173668.1	145798	147228	147164, 147181, 146031, 147174	10, 22, 12, 16	8.40516, 18.37326, 11.50726, 15.94441	5.75437, 8.84063, 7.30112, 9.0962	5.76336, 1	3	NA	NA
GL173668.1	151825	152402	152221, 152000, 152012, 152350	8, 9, 5, 4	8.34715, 7.13885, 4.91762, 4.59709	5.9367, 5.11729, 4.03768, 3.75554	5.71011, 4	3	NA	NA
GL173668.1	183733	184142	184116, 184116, 184116	51, 97, 61	69.9706, 130.79028, 87.49385	26.95989, 35.57205, 31.91683	65.8739, 1	3	NA	NA
GL173668.1	203202	203656	203392, 203370, 203421	9, 15, 7	9.72082, 15.80067, 8.69489	6.64182, 9.24574, 6.06379	6.97155, 1	3	NA	NA
GL173669.1	190945	191434	191156, 191182, 190962	5, 10, 4	4.81325, 8.24345, 4.15126	3.98038, 5.65367, 3.54016	2.51418, 5	3	NA	8222
GL173670.1	10260	10951	10290, 10320, 10295	26, 27, 23	35.26919, 33.60851, 34.77999	17.41492, 16.18004, 17.73239	31.65079,	3	ccr7	34669
GL173670.1	18110	18568	18442, 18444, 18455	14, 19, 7	16.7649, 21.01008, 8.69489	9.96273, 11.32031, 6.06379	13.6543, 1	3	ccr7	27052
GL173670.1	59557	60049	59598, 59674, 59736	6, 9, 7	5.47306, 8.10603, 7.96278	4.36955, 5.71528, 5.72394	3.10811, 5	3	NA	639
GL173670.1	65337	65657	65483, 65485, 65445	33, 35, 20	36.54568, 36.48016, 24.34577	15.6093, 14.75556, 12.9746	32.90263,	3	NA	6419
GL173670.1	133920	135679	135141, 135345, 134440	11, 25, 8	10.1122, 21.78802, 7.42597	6.63573, 9.89653, 5.41279	7.34229, 1	3	NA	9052
GL173670.1	182569	182953	182897, 182793, 182686	7, 9, 3	6.2098, 7.24675, 3.2112	4.76362, 5.1851, 2.90793	3.74679, 4	3	top2a	1905
GL173671.1	8511	9350	9049, 9014, 8625, 9055	8, 11, 4, 9	7.1122, 10.3713, 4.08034, 10.42289	5.22529, 6.79219, 3.50363, 7.00726	4.57946, 7	3	padi2	0
GL173671.1	68876	69424	69047, 69011, 68939	10, 17, 6	7.25363, 12.70601, 5.0826	5.03021, 6.87373, 4.13667	4.68848, 9	3	atp13a2	9498
GL173671.1	86653	87412	87038, 87076, 86920	15, 14, 11	18.26603, 14.45897, 13.55304	10.62691, 8.66788, 8.54092	15.09729,	3	irs2	3134
GL173671.1	96712	97222	97195, 97164, 97116	19, 21, 24	19.8867, 19.48223, 30.17171	10.65421, 9.80473, 15.16988	16.66031,	3	irs2	13193
GL173671.1	99995	100549	100353, 100383, 100270	10, 10, 4	9.28413, 7.72315, 3.43992	6.29221, 5.32711, 3.14645	6.574, 5.26	3	nyap1	11888
GL173671.1	156449	157640	156999, 157061, 156504	8, 18, 9	7.28955, 16.81975, 9.41119	5.33177, 9.08887, 6.47287	4.72176, 1	3	SMG5	9380
GL173671.1	159390	159883	159471, 159477, 159681	11, 11, 10	10.9046, 8.66477, 10.71812	7.10903, 5.74034, 7.12015	8.08987, 6	3	SMG5	7137
GL173671.1	175873	176497	176304, 176319, 176216	84, 127, 69	87.66757, 128.72072, 77.22511	20.57927, 21.48292, 21.96418	83.39504,	3	SMG5	0
GL173671.1	189524	190048	189601, 189647, 189612	10, 20, 10	8.0085, 17.88727, 10.25108	5.50663, 9.1506, 6.85862	5.39412, 1	3	crocc	0
GL173671.1	195118	195824	195197, 195740, 195153	6, 6, 4	5.96715, 4.92507, 4.38152	4.64927, 4.04003, 3.65448	3.52158, 2	3	crocc	0
GL173671.1	240123	240538	240237, 240158, 240229	7, 9, 6	7.1696, 8.21099, 7.29403	5.31345, 5.77858, 5.30581	4.61046, 5	3	necap1	6824
GL173672.1	38150	39104	38452, 38345, 38551	18, 15, 7	19.17347, 15.80067, 7.96278	10.50657, 9.24574, 5.72394	15.97256,	3	snx4	0
GL173672.1	100966	101652	101190, 101088, 101226	6, 6, 4	4.37941, 4.4843, 4.34116	3.69512, 3.76254, 3.63492	2.19155, 2	3	GPD1	385
GL173672.1	144646	145243	144911, 145121, 144762	4, 9, 8	3.39747, 7.10358, 8.4044	3.12111, 5.09508, 5.9678	1.46102, 4	3	parp14.2	0
GL173672.1	228826	229191	229165, 229161, 229161	48, 59, 42	62.90348, 75.73843, 57.73918	24.32015, 26.14458, 24.14987	58.88377,	3	NA	11555
GL173673.1	9637	10313	9934, 9972, 9967	15, 11, 7	17.09296, 10.48162, 7.61369	9.98755, 6.85833, 5.54854	13.96978,	3	bace2	894
GL173673.1	21315	21553	21525, 21416, 21518	7, 10, 7	6.50909, 9.37821, 8.646	4.94073, 6.34863, 6.04229	4.02371, 6	3	bace2	0
GL173673.1	32597	33836	33464, 33734, 33578	11, 15, 14	9.52723, 14.39783, 15.35015	6.27749, 8.40758, 9.184	6.80251, 1	3	bace2	0
GL173673.1	70289	70635	70609, 70579, 70608	12, 15, 8	12.11599, 14.21432, 9.76268	7.66241, 8.29617, 6.64964	9.23027, 1	3	fam3b	0
GL173673.1	105797	106382	105940, 105921, 106028	13, 16, 12	14.70302, 17.16856, 14.76955	8.98015, 9.82359, 9.10943	11.68444,	3	mx1	1202
GL173673.1	133654	134737	134573, 133823, 133823	17, 20, 18	14.64358, 14.83072, 18.94478	8.0415, 7.34984, 10.37065	11.62752,	3	mx1	0
GL173673.1	139141	139884	139827, 139748, 139321	7, 13, 9	6.16939, 10.11663, 8.98857	4.73936, 6.23937, 6.23511	3.70916, 7	3	mx1	0
GL173673.1	182302	182827	182628, 182694, 182738	5, 7, 5	4.54329, 5.82304, 5.9506	3.82752, 4.52813, 4.54784	2.33, 3.538	3	NA	0
GL173673.1	205277	205896	205364, 205688, 205288, 205812	6, 12, 8, 5	5.96715, 11.86108, 10.1465, 5.9506	4.64927, 7.51216, 6.82176, 4.54784	3.52158, 9	3	NA	15814
GL173674.1	163818	164714	164385, 164380, 164161	4, 10, 6	3.74195, 9.39128, 7.29403	3.32091, 6.35644, 5.30581	1.6222, 6.8	3	tap1	0
GL173675.1	65226	66326	66156, 66236, 66207	12, 17, 7	12.60209, 15.93163, 8.69489	7.9443, 8.82633, 6.06379	9.68421, 1	3	cmtr1	25366
GL173675.1	80424	81520	81328, 81247, 81332	21, 37, 9	21.17819, 37.70701, 9.8849	10.82682, 14.65424, 6.72947	17.91116,	3	kcnk5	22884
GL173675.1	103387	103723	103521, 103516, 103525	10, 14, 8	10.96713, 14.15854, 10.1465	7.25596, 8.49024, 6.82176	8.14942, 1	3	kcnk5	681
GL173675.1	131537	132276	131618, 132007, 132097	11, 19, 10	12.33515, 20.47061, 12.33934	7.91559, 11.002, 7.95424	9.43699, 1	3	kcnk5	12205
GL173675.1	166939	167603	167100, 167270, 167372	15, 26, 11	17.90819, 28.58933, 12.06254	10.43573, 13.57987, 7.76744	14.75478,	3	SAYSD1	0
GL173675.1	217469	217999	217973, 217956, 217982	32, 57, 40	37.88202, 68.80804, 52.39471	16.80937, 23.20563, 21.9379	34.2142, 6	3	GLP1R	0
GL173677.1	198743	199337	199124, 199160, 198991	9, 9, 12	7.67537, 7.51164, 12.26557	5.45199, 5.35055, 7.74983	5.08317, 5	3	mdga2	12823
GL173677.1	201586	202236	202118, 202057, 201988	12, 16, 13	11.32055, 13.5443, 11.81672	7.18871, 7.63435, 7.28993	8.47391, 1	3	mdga2	15666
GL173677.1	204489	205165	204934, 204952, 204735	5, 16, 10	4.77263, 15.94397, 10.59656	3.9578, 9.09593, 7.05292	2.51418, 1	3	mdga2	18569
GL173678.1	31623	32377	31761, 32219, 32205	14, 21, 14	12.8072, 18.48988, 15.80333	7.67424, 9.21025, 9.43935	9.88012, 1	3	NA	16267
GL173678.1	93683	94471	94029, 94190, 93808, 94338	17, 17, 9, 15	13.18767, 12.26912, 7.65011, 14.8216	7.16109, 6.61547, 5.43639, 8.66354	10.24314,	3	NA	1205
GL173679.1	34961	36037	35710, 35753, 35017, 35777	11, 16, 5, 4	10.1122, 13.83763, 4.64121, 3.46461	6.63573, 7.81372, 3.88372, 3.1611	7.34229, 1	3	cth	137575
GL173679.1	38772	39535	39312, 39126, 38880	7, 9, 5	5.65516, 6.37567, 4.64121	4.42382, 4.63258, 3.88372	3.27228, 4	3	cth	134077
GL173679.1	112981	115207	113895, 114089, 114038	26, 30, 19	24.46003, 24.7325, 19.4202	11.12216, 9.95894, 10.3744	21.09356,	3	cth	58405
GL173679.1	195503	196356	195974, 195871, 195943	12, 16, 11	12.05017, 15.28831, 11.5511	7.62377, 8.69939, 7.48213	9.16715, 1	3	cth	12173
GL173679.1	203902	204665	204413, 204295, 204456	27, 32, 23	32.92392, 41.70567, 29.05751	15.78361, 19.04588, 14.82811	29.35243,	3	cth	20572
GL173680.1	194460	195336	194772, 194509, 194503	10, 16, 19	9.54985, 14.35819, 20.93999	6.45088, 8.13215, 11.27914	6.82341, 1	3	NA	NA
GL173681.1	121410	122418	121619, 122087, 121960	11, 9, 14	12.33515, 8.21099, 16.91966	7.91559, 5.77858, 10.04443	9.43699, 5	3	NA	627
GL173681.1	194079	194898	194585, 194497, 194367	5, 13, 5	4.43696, 11.71436, 5.31857	3.76655, 7.22722, 4.24819	2.24017, 9	3	dennd4b	1146
GL173681.1	201054	201722	201352, 201351, 201517	14, 15, 11	14.38232, 14.27489, 14.03502	8.62271, 8.33298, 8.77076	11.37721,	3	dennd4b	8121
GL173682.1	1556	3065	1587, 2593, 2239	5, 14, 9	3.61427, 13.50676, 8.60738	3.25593, 8.09967, 6.01421	1.6222, 10	3	NA	NA
GL173682.1	8571	9614	9118, 9183, 9256	7, 10, 5	7.1696, 9.39128, 5.9506	5.31345, 6.35644, 4.54784	4.61046, 6	3	NA	NA
GL173685.1	21058	22204	21839, 21236, 21595	20, 13, 9	21.98313, 13.14514, 10.28145	11.61252, 8.09002, 6.93568	18.6897, 1	3	hspg2	13421
GL173685.1	27953	29611	29384, 28674, 28634	10, 11, 5	10.96713, 10.60895, 5.9506	7.25596, 6.9343, 4.54784	8.14942, 7	3	hspg2	6014
GL173685.1	62779	63141	62966, 63022, 63021	15, 15, 11	14.21298, 13.16372, 11.92945	8.29535, 7.65379, 7.69409	11.21566,	3	hspg2	0
GL173685.1	87109	87928	87359, 87481, 87473	18, 9, 8	20.16146, 8.21099, 8.86315	11.08652, 5.77858, 6.21039	16.9259, 5	3	hspg2	0
GL173685.1	100347	101078	100954, 100954, 100908	15, 30, 7	12.95198, 31.15101, 6.75301	7.52379, 13.60907, 5.08141	10.01762,	3	hspg2	2067
GL173685.1	127711	128000	127960, 127981, 127944	21, 14, 9	22.94132, 12.18837, 10.64485	11.88295, 7.29433, 7.11743	19.61877,	3	USP48	0
GL173685.1	141713	142197	141814, 141822, 141810	15, 14, 7	15.71867, 12.59519, 8.69489	9.19755, 7.54437, 6.06379	12.65377,	3	cela3b	0
GL173685.1	160461	160935	160831, 160865, 160904	6, 10, 6	5.55471, 9.37821, 6.6567	4.41698, 6.34863, 5.00845	3.18192, 6	3	cela3a	0
GL173685.1	182720	183881	183538, 183539, 183539	13, 20, 12	14.61367, 22.06591, 13.59027	8.93088, 11.6614, 8.49571	11.59864,	3	cela3a	614
GL173687.1	56564	57033	56686, 56987, 56791	7, 14, 16	5.10557, 11.49628, 15.37396	4.075, 6.86757, 8.75139	2.77482, 8	3	hes7.1	28783
GL173687.1	63926	65232	64796, 64816, 64756	31, 27, 17	29.56509, 23.71775, 15.10076	12.30966, 10.35558, 8.32001	26.0673, 2	3	hes7.1	20584
GL173687.1	88445	89890	88483, 88461, 89402, 88507	25, 47, 16, 27	18.94365, 35.81106, 6.36371, 26.87418	8.29934, 10.38805, 3.59946, 12.1951	15.75107,	3	hes7.1	35
GL173687.1	91784	92296	92262, 92260, 92272	51, 109, 43	52.60276, 119.28135, 48.21561	17.0672, 24.20763, 18.11781	48.71577,	3	hes7.1	3374
GL173687.1	119564	119920	119728, 119578, 119613	6, 7, 7	5.43318, 5.97725, 8.08824	4.34622, 4.62287, 5.7849	3.07284, 3	3	gucy2d	0
GL173688.1	32149	32931	32197, 32185, 32536	9, 26, 5	9.72082, 25.32968, 5.23257	6.64182, 11.63548, 4.20435	6.97155, 2	3	NA	18744
GL173688.1	33729	34155	33769, 33775, 33971	5, 9, 4	4.82177, 6.15408, 3.88239	3.98509, 4.4908, 3.39841	2.51418, 3	3	NA	17520
GL173688.1	45117	45437	45405, 45377, 45268	7, 16, 3	7.1696, 16.67093, 3.47371	5.31345, 9.53036, 3.03189	4.61046, 1	3	NA	6238
GL173688.1	71537	72346	72231, 71782, 71887	7, 12, 7	6.55439, 11.37069, 7.84208	4.96711, 7.21896, 5.66426	4.06616, 8	3	NA	15181
GL173688.1	175012	175708	175676, 175680, 175247	21, 18, 13	26.69759, 19.97713, 17.16464	14.03424, 10.97931, 10.23255	23.26976,	3	NA	118656
GL173690.1	1770	3589	2968, 2080, 1971	9, 10, 6	6.545, 7.38914, 5.91225	4.74071, 5.11603, 4.61907	4.05782, 4	3	NA	146659
GL173690.1	7290	8715	8338, 7513, 7751	17, 26, 11	15.22598, 23.12524, 11.31465	8.39635, 10.34426, 7.3472	12.18492,	3	NA	141533
GL173690.1	59988	60510	60358, 60332, 60346	14, 13, 11	15.31941, 13.14514, 14.75772	9.1665, 8.09002, 9.09568	12.27033,	3	NA	89738
GL173692.1	209093	209753	209256, 209484, 209473	23, 30, 19	14.86354, 18.31158, 13.17941	6.64015, 6.79667, 6.66019	11.83739,	3	C2orf88	7005
GL173692.1	210384	211436	210606, 210627, 210566, 210984	21, 23, 15, 11	13.29412, 12.2742, 9.40084, 5.9573	6.26571, 5.37231, 5.38093, 4.05579	10.34371,	3	C2orf88	8296
GL173692.1	210384	211436	211119, 210627, 210566, 210984	18, 23, 15, 11	10.69571, 12.2742, 9.40084, 5.9573	5.50569, 5.37231, 5.38093, 4.05579	7.89316, 9	3	C2orf88	8296
GL173693.1	47	934	595, 506, 344	20, 23, 20	21.61731, 23.98856, 24.34577	11.39575, 11.86759, 12.9746	18.33537,	3	NA	118562
GL173693.1	15324	15683	15455, 15479, 15553	6, 14, 3	5.96715, 13.75886, 3.47371	4.64927, 8.2515, 3.03189	3.52158, 1	3	NA	103813
GL173693.1	39483	40045	39851, 39535, 39813	11, 8, 6	10.66406, 6.24338, 6.12365	6.96705, 4.6873, 4.73409	7.86396, 3	3	NA	79451
GL173693.1	114140	114685	114437, 114220, 114443	14, 9, 9	16.7649, 8.21099, 11.48736	9.96273, 5.77858, 7.51109	13.6543, 5	3	NA	4811
GL173693.1	174939	175570	175392, 175352, 175413	12, 20, 12	11.26426, 18.65688, 12.38374	7.15468, 9.61459, 7.81847	8.4212, 15	3	lbh	3419
GL173694.1	28	1188	241, 1020, 185	10, 19, 4	10.81731, 19.8035, 4.67325	7.17456, 10.60442, 3.78987	8.00764, 1	3	NA	11117
GL173694.1	28	1188	1035, 1020, 185	10, 19, 4	10.81731, 19.8035, 4.67325	7.17456, 10.60442, 3.78987	8.00764, 1	3	NA	11117
GL173694.1	6760	7351	6882, 6894, 6872	46, 57, 31	58.55399, 64.02193, 38.6896	22.74499, 20.5446, 17.67953	54.58403,	3	NA	4954
GL173695.1	445	997	781, 766, 835	7, 8, 4	6.89193, 6.44699, 4.42274	5.15998, 4.8155, 3.67426	4.37772, 4	3	dnah2	104952
GL173695.1	16759	17197	17041, 17012, 17050	17, 26, 10	18.46847, 27.83582, 12.90749	10.34725, 13.1298, 8.21675	15.29249,	3	dnah2	88752
GL173695.1	30823	31376	31120, 31095, 31145	10, 20, 6	9.89241, 19.16931, 5.71639	6.65218, 9.92426, 4.50951	7.13431, 1	3	dnah2	74573
GL173695.1	132055	133215	132491, 132671, 132632	17, 14, 14	13.96763, 10.54152, 13.03055	7.63104, 6.27972, 7.81064	10.98042,	3	dnah2	0
GL173695.1	159747	160490	160054, 160066, 159962	5, 7, 3	4.82177, 5.97725, 3.47371	3.98509, 4.62287, 3.03189	2.51418, 3	3	dnah2	0
GL173695.1	180234	180754	180450, 180432, 180635	12, 25, 14	10.63412, 24.01826, 15.35015	6.77016, 11.20454, 9.184	7.83559, 2	3	dnah2	0
GL173696.1	13629	14075	13907, 13771, 13696	5, 5, 3	4.61714, 3.80717, 3.47371	3.86998, 3.3798, 3.03189	2.39372, 1	3	fxyd1	33739
GL173696.1	73805	74780	73881, 73878, 73872	41, 83, 47	50.42744, 101.15813, 61.79207	20.32531, 27.5126, 24.1603	46.56946,	3	fxyd1	21308
GL173696.1	149452	150480	150252, 150099, 149483	12, 12, 13	13.73771, 10.98912, 15.70541	8.57523, 6.98757, 9.51556	10.76705,	3	psmc4	17386
GL173697.1	367	611	498, 406, 430	6, 15, 5	5.55471, 13.4765, 5.9506	4.41698, 7.84563, 4.54784	3.18192, 1	3	lrp6	13192
GL173697.1	69479	69987	69674, 69760, 69879	10, 17, 7	10.89158, 15.40668, 8.49722	7.21503, 8.50651, 5.97582	8.0782, 12	3	bcl2l14	553
GL173697.1	116891	117232	116924, 116896, 117018	6, 9, 5	5.06496, 7.28344, 5.14967	4.12592, 5.20811, 4.16141	2.73812, 4	3	NA	13243
GL173697.1	148486	149265	149062, 148933, 148934	13, 19, 4	12.26307, 18.54823, 4.67325	7.56188, 9.84817, 3.78987	9.36972, 1	3	parp12	4498
GL173698.1	177208	177601	177378, 177348, 177451	10, 8, 4	10.46385, 6.55376, 4.55201	6.97883, 4.88226, 3.73489	7.67431, 4	3	NA	3947
GL173699.1	83133	84037	83858, 83595, 83469	18, 15, 6	18.84377, 12.49102, 5.99481	10.31044, 7.24068, 4.6644	15.65357,	3	NA	240
GL173699.1	122475	123116	122586, 122708, 122707	16, 24, 12	14.40209, 22.99283, 12.62857	8.159, 10.93416, 7.95947	11.39589,	3	NA	0
GL173699.1	185566	186275	185809, 186093, 186121	13, 18, 7	13.42064, 17.72647, 8.646	8.25183, 9.63881, 6.04229	10.46414,	3	NA	31505
GL173701.1	7993	8339	8152, 8046, 8019	12, 13, 4	10.99193, 12.14594, 4.08034	6.98928, 7.49077, 3.50363	8.17226, 9	3	slc12a4	78886
GL173701.1	72279	73915	73502, 72709, 73042, 72833	11, 7, 8, 11	9.95877, 4.79556, 5.72807, 12.63525	6.54239, 3.87426, 4.35854, 8.07536	7.19682, 2	3	slc12a4	13310
GL173702.1	17468	18198	17857, 17896, 17787	14, 14, 11	13.26657, 11.62806, 14.75772	7.95422, 6.94888, 9.09568	10.31735,	3	NA	1606
GL173702.1	76448	77006	76494, 76469, 76469	35, 41, 21	44.60062, 51.62965, 28.28935	19.53558, 21.03412, 14.87856	40.82737,	3	NA	3549
GL173702.1	102062	102382	102295, 102293, 102312	9, 15, 12	9.30591, 15.80067, 13.36868	6.41439, 9.24574, 8.37481	6.59443, 1	3	NA	0
GL173703.1	43731	44241	43863, 43977, 43856	5, 11, 6	4.33555, 7.63964, 4.75057	3.70555, 5.09648, 3.93137	2.15195, 5	3	stk11ip	16026
GL173703.1	48070	48689	48338, 48303, 48314	5, 16, 13	4.47183, 12.25152, 12.81733	3.78598, 6.84834, 7.8954	2.26935, 9	3	stk11ip	11578
GL173703.1	80432	81070	80883, 80887, 80491	5, 13, 7	4.82177, 11.61168, 8.21885	3.98509, 7.16421, 5.84717	2.51418, 8	3	stk11ip	1101
GL173703.1	137290	137722	137690, 137717, 137672	17, 29, 14	13.56628, 25.5962, 14.40949	7.3886, 10.75635, 8.63873	10.60322,	3	myo10.2	3624
GL173703.1	142111	142464	142169, 142142, 142153	14, 17, 7	11.66068, 13.05396, 5.85759	6.969, 7.08107, 4.54944	8.79778, 1	3	myo10.2	0
GL173703.1	150305	150848	150778, 150648, 150535	9, 10, 7	7.20928, 9.17452, 7.72582	5.16158, 6.22617, 5.6058	4.64652, 6	3	myo10.2	0
GL173703.1	172045	172315	172180, 172180, 172178	12, 18, 7	11.32055, 18.91825, 8.646	7.18871, 10.35485, 6.04229	8.47391, 1	3	myo10.2	0
GL173704.1	131259	131684	131515, 131510, 131468	10, 17, 6	9.23294, 17.11659, 6.50067	6.26141, 9.54398, 4.93052	6.52626, 1	3	arl5b	0
GL173706.1	34456	34943	34495, 34707, 34732	10, 10, 9	11.06011, 8.32357, 10.87975	7.306, 5.70361, 7.23113	8.22712, 5	3	kcnc1	0
GL173706.1	50755	51158	51051, 50831, 50778	11, 17, 9	9.90875, 13.99022, 9.70054	6.51186, 7.64472, 6.63089	7.14919, 1	3	ap5z1	202
GL173706.1	76741	77623	76830, 76825, 77485	13, 24, 12	13.94585, 23.62809, 14.40488	8.5554, 11.31356, 8.92522	10.95967,	3	abcf2	0
GL173706.1	105730	106806	106012, 105995, 106010	46, 55, 28	48.97602, 55.48774, 33.50283	17.22506, 16.92495, 15.76552	45.13744,	3	abcf2	1852
GL173706.1	131710	132048	131944, 131795, 131903	7, 10, 5	6.60038, 9.39128, 5.54867	4.99378, 6.35644, 4.3619	4.10761, 6	3	saal1	0
GL173706.1	153274	153973	153323, 153634, 153681	8, 15, 3	7.72597, 14.98313, 3.47371	5.588, 8.76054, 3.03189	5.13061, 1	3	gtf2h1	576
GL173706.1	166244	166722	166459, 166473, 166542	9, 5, 6	9.72082, 3.94561, 7.29403	6.64182, 3.46715, 5.30581	6.97155, 1	3	gtf2h1	13546
GL173707.1	73807	74125	73817, 73868, 73838	12, 7, 8	10.78431, 4.516, 8.35103	6.86236, 3.6915, 5.93881	7.9765, 2.3	3	oplah	24580
GL173707.1	80894	81154	81070, 81066, 81063	26, 38, 13	24.87014, 39.1835, 13.21599	11.3637, 15.08945, 8.13179	21.4923, 3	3	oplah	17551
GL173707.1	145378	145798	145487, 145503, 145576	10, 15, 9	10.20047, 15.80067, 9.46739	6.82978, 9.24574, 6.50386	7.42612, 1	3	gpt2	0
GL173707.1	176586	177270	176612, 176613, 176619	73, 142, 56	95.20745, 183.14493, 75.60924	29.64286, 36.86025, 27.66497	90.85838,	3	gpt2	6087
GL173707.1	183699	184254	183883, 183727, 183767	38, 53, 17	26.30716, 37.69221, 9.98715	8.55195, 9.70118, 5.31628	22.88948,	3	5S_rRNA	42
GL173707.1	187119	189535	187825, 188117, 188119	38, 42, 25	23.00579, 23.78131, 15.68735	7.1951, 6.78976, 6.61454	19.68166,	3	5S_rRNA	0
GL173708.1	60875	61354	60879, 60879, 61100	6, 10, 7	5.96715, 9.39128, 8.21885	4.64927, 6.35644, 5.84717	3.52158, 6	3	gbp4	39562
GL173708.1	65552	66100	65969, 66020, 65980	7, 9, 8	5.97522, 8.21099, 9.46129	4.62162, 5.77858, 6.50801	3.52869, 5	3	gbp4	34816
GL173709.1	7254	7792	7654, 7670, 7707	11, 23, 10	11.93519, 24.15665, 10.97191	7.69727, 11.96843, 7.25854	9.0586, 20	3	kcnj9	2959
GL173709.1	28102	28672	28623, 28623, 28621	52, 65, 40	65.23727, 78.75459, 50.1805	23.67775, 24.72114, 20.63692	61.19138,	3	cox6b1	6959

GL173709.1	38568	39781	39349, 39345, 38928, 39444	12, 12, 4, 6	13.05874, 11.04188, 4.59709, 7.05929	8.20296, 7.01971, 3.75554, 5.20024	10.11961,	3	cox6b1	17425
GL173709.1	57003	57716	57077, 57128, 57161	7, 7, 4	6.46447, 4.30364, 4.67325	4.91463, 3.55187, 3.78987	3.98312, 2	3	cox6b1	35860
GL173709.1	145904	146286	146063, 146030, 146099	5, 6, 4	4.61714, 4.93312, 4.67325	3.86998, 4.04501, 3.78987	2.39372, 2	3	NA	832
GL173710.1	8419	8827	8749, 8754, 8747	7, 12, 9	7.1696, 11.72254, 10.87975	5.31345, 7.42986, 7.23113	4.61046, 9	3	KCNJ6	982
GL173710.1	24922	25489	25222, 25355, 25123	11, 14, 9	12.43693, 14.45897, 11.39481	7.97018, 8.66788, 7.46977	9.52692, 1	3	KCNJ6	17485
GL173710.1	40394	41746	41242, 41133, 41041	21, 30, 21	21.64688, 30.39874, 24.0973	11.10892, 13.16591, 12.56358	18.3637, 2	3	KCNJ6	32957
GL173710.1	163712	164142	164110, 164108, 164112	13, 15, 12	13.49269, 13.06292, 13.98181	8.29387, 7.59191, 8.70517	10.53271,	3	kcnj15	20427
GL173711.1	99714	101448	101027, 101000, 101005	29, 33, 19	20.92166, 22.95514, 14.57865	8.26088, 8.23928, 7.46571	17.6635, 1	3	NA	66547
GL173711.1	115148	116329	115488, 115792, 115415, 116110	21, 29, 10, 11	17.51801, 23.81251, 6.97437, 8.06567	8.63427, 9.7714, 4.85319, 5.3641	14.37772,	3	NA	51858
GL173712.1	87681	88241	87982, 87875, 87928	10, 22, 16	11.06011, 21.01356, 20.07743	7.306, 10.40847, 11.44009	8.22712, 1	3	NA	4256
GL173712.1	98477	98845	98643, 98722, 98603	6, 14, 9	5.24248, 12.43922, 10.56949	4.2332, 7.44862, 7.08032	2.89962, 9	3	NA	239
GL173712.1	111828	112535	112185, 112088, 112195	15, 21, 21	14.78606, 18.43497, 23.91788	8.64215, 9.17752, 12.4588	11.76448,	3	ccdc172	659
GL173712.1	155541	155787	155610, 155631, 155605	10, 26, 8	8.66311, 24.9752, 7.38564	5.914, 11.42574, 5.38892	5.99101, 2	3	NA	4591
GL173712.1	177909	178220	178012, 178040, 178058	11, 27, 9	9.95877, 27.51274, 9.19414	6.54239, 12.57423, 6.35176	7.19682, 2	3	CCDC172	0
GL173714.1	43505	43932	43663, 43725, 43903	14, 16, 11	13.14827, 15.87543, 13.1143	7.88233, 9.05466, 8.32282	10.20482,	3	stim1	0
GL173714.1	82074	82678	82499, 82506, 82102	10, 16, 9	7.22253, 14.41261, 7.2501	5.0105, 8.16543, 5.1872	4.65954, 1	3	stim1	0
GL173714.1	90619	91034	90746, 90723, 90966	5, 7, 6	4.50727, 5.8946, 6.03707	3.80664, 4.57222, 4.6874	2.30125, 3	3	stim1	1010
GL173714.1	164568	164789	164686, 164744, 164759	6, 10, 10	5.63898, 8.28331, 11.5283	4.46544, 5.67853, 7.55211	3.25772, 5	3	NA	318
GL173715.1	178982	179999	179495, 179809, 179485, 179924	56, 61, 39, 32	24.16765, 22.33271, 15.40858, 10.68403	5.25079, 4.49685, 4.51244, 3.72276	20.80961,	3	NA	57529
GL173715.1	180233	181826	180594, 180592, 180598	80, 67, 54	41.6565, 24.60294, 25.66424	6.85854, 4.5627, 5.8044	37.9277, 2	3	NA	58780
GL173715.1	182721	184296	183313, 183322, 183309	123, 174, 147	64.86962, 100.53347, 100.27262	7.3107, 8.51509, 10.76061	60.82748,	3	NA	61268
GL173715.1	187447	190047	188158, 189057, 188174, 188586, 189047	175, 448, 169, 84, 422	98.32679, 418.05685, 102.06171, 32.81887, 433.83698	8.19176, 20.94435, 9.05417, 4.97808, 25.86644	93.95123,	3	NA	65994
GL173715.1	187447	190047	189072, 189057, 188174, 188586, 189047	422, 448, 169, 84, 422	405.85367, 418.05685, 102.06171, 32.81887, 433.83698	22.27368, 20.94435, 9.05417, 4.97808, 25.86644	400.41333	3	NA	65994
GL173716.1	108126	108534	108488, 108442, 108478	19, 36, 13	19.48878, 37.44002, 14.85247	10.41563, 14.9052, 9.06201	16.2775, 3	3	NA	0
GL173716.1	123053	123277	123227, 123248, 123233	10, 19, 12	8.93828, 16.94897, 11.87227	6.08275, 8.87967, 7.51879	6.24946, 1	3	NA	2368
GL173716.1	142864	143943	143343, 143342, 143269	22, 28, 10	25.29347, 31.72212, 12.52085	12.96511, 14.71186, 8.03986	21.90464,	3	slc22a13	2049
GL173717.1	42513	43668	42917, 42810, 42884	14, 12, 14	13.96392, 10.39632, 13.81849	8.37431, 6.6236, 8.28728	10.9769, 7	3	rerg	0
GL173717.1	59316	61502	60140, 59485, 60234	6, 14, 12	4.77375, 11.15936, 12.15007	3.9459, 6.65976, 7.68238	2.51418, 8	3	rerg	923
GL173717.1	59316	61502	60832, 59485, 60234	7, 14, 12	5.93788, 11.15936, 12.15007	4.59878, 6.65976, 7.68238	3.52158, 8	3	rerg	923
GL173717.1	80507	81129	80643, 80693, 80699	16, 15, 17	18.14851, 15.7813, 22.63257	10.3887, 9.23437, 12.61306	14.98517,	3	ptpro	0
GL173717.1	94346	95839	95090, 94790, 95252	10, 17, 7	10.39648, 17.87191, 8.69489	6.94096, 9.99549, 6.06379	7.61003, 1	3	ptpro	0
GL173717.1	104680	105466	105254, 104898, 104914	8, 10, 6	8.42257, 9.39128, 7.29403	5.97763, 6.35644, 5.30581	5.76336, 6	3	ptpro	0
GL173717.1	127581	127986	127847, 127804, 127591	6, 14, 4	5.09949, 11.71767, 4.15126	4.14694, 7.00416, 3.54016	2.76939, 9	3	ptpro	0
GL173718.1	76891	78143	77588, 77321, 77509	15, 18, 14	15.56593, 16.17802, 14.40949	9.10751, 8.69892, 8.63873	12.50679,	3	C8G	790
GL173718.1	110606	111243	111154, 111182, 111201	10, 12, 13	11.06011, 11.86108, 16.70862	7.306, 7.51216, 10.0169	8.22712, 9	3	ambp	5414
GL173718.1	180595	181153	180679, 180799, 180857	8, 10, 6	6.31005, 7.79347, 6.35308	4.7294, 5.37143, 4.85498	3.84065, 5	3	NA	21820
GL173720.1	49	1083	517, 797, 802	67, 57, 65	64.66017, 55.26995, 66.07884	16.74229, 16.0424, 18.15086	60.62079,	3	spcs1	3397
GL173720.1	59744	60274	60170, 60083, 60122	9, 16, 13	7.55321, 14.25068, 13.58227	5.37637, 8.0664, 8.34597	4.96981, 1	3	pbrm1	0
GL173720.1	61752	62644	62266, 62172, 61897	12, 17, 7	9.04939, 12.16434, 4.98481	5.78552, 6.55391, 3.99709	6.35412, 9	3	pbrm1	0
GL173720.1	65941	66215	66134, 66183, 66076	16, 25, 13	14.34724, 23.81247, 12.65497	8.12546, 11.08247, 7.79825	11.34358,	3	pbrm1	0
GL173720.1	97911	98288	98161, 98175, 98129	16, 22, 19	19.07423, 25.85271, 25.24922	10.90446, 13.29074, 13.66204	15.87684,	3	pbrm1	8491
GL173720.1	159241	159791	159362, 159418, 159425	10, 13, 11	10.39648, 13.14514, 14.68355	6.94096, 8.09002, 9.06344	7.61003, 1	3	rrp1b	549
GL173720.1	183792	184185	184008, 183895, 183890	15, 11, 4	14.21298, 10.15827, 3.25394	8.29535, 6.66366, 3.03398	11.21566,	3	sik1	0
GL173721.1	80502	80941	80819, 80819, 80819	24, 23, 8	28.29526, 24.59021, 9.05167	14.09251, 12.22818, 6.30653	24.82836,	3	NA	11680
GL173721.1	112652	113198	113168, 112789, 113060	5, 6, 7	4.43696, 4.93312, 7.90186	3.76555, 4.04501, 5.69394	2.24017, 2	3	NA	7311
GL173722.1	65203	65951	65658, 65613, 65400	11, 11, 13	11.49566, 10.60895, 16.49347	7.45067, 6.9343, 9.91245	8.64106, 7	3	NA	106079
GL173722.1	93853	94623	94095, 94185, 94426	5, 7, 3	4.82177, 5.97725, 3.47371	3.98509, 4.62287, 3.03189	2.51418, 3	3	NA	77407
GL173722.1	94081	94786	94764, 94185, 94426	5, 7, 3	4.82177, 5.97725, 3.47371	3.98509, 4.62287, 3.03189	2.51418, 3	3	NA	77244
GL173722.1	157888	158466	158034, 158096, 158056	17, 31, 12	16.90454, 30.72705, 13.7434	9.41624, 12.98008, 8.57827	13.78827,	3	NA	13564
GL173722.1	181782	182937	181861, 182548, 182351	12, 14, 7	13.55951, 13.14791, 7.84208	8.47903, 7.88211, 5.66426	10.59689,	3	NA	6926
GL173723.1	128	895	696, 696, 696	15, 19, 10	15.41679, 18.54823, 11.997	9.0192, 9.84817, 7.78835	12.36344,	3	ehmt1	94362
GL173723.1	42758	43767	42817, 43110, 42943	6, 12, 8	5.3552, 9.54462, 7.81204	4.3003, 6.09452, 5.63749	3.00253, 6	3	ehmt1	51490
GL173723.1	126132	126557	126527, 126487, 126510	17, 11, 15	18.64535, 9.13706, 17.76194	10.45058, 6.0355, 10.35658	15.46286,	3	ehmt1	4285
GL173723.1	132208	132680	132427, 132493, 132290	7, 16, 5	6.37715, 14.14493, 5.45388	4.86324, 8.00171, 4.3157	3.90257, 1	3	ehmt1	10361
GL173723.1	154490	155428	155057, 155183, 155263	17, 27, 11	16.36399, 26.07341, 12.78973	9.08912, 11.72214, 8.15619	13.27324,	3	arrdc1	12942
GL173723.1	169864	170152	169885, 169896, 169892	10, 7, 7	9.18236, 5.82304, 7.20145	6.23091, 4.52813, 5.33074	6.47811, 3	3	arrdc1	0
GL173723.1	179480	180068	179649, 179980, 179919	6, 18, 7	5.55471, 15.7443, 7.15355	4.41698, 8.43569, 5.30471	3.18192, 1	3	arrdc1	0
GL173723.1	185641	186006	185800, 185775, 185796	7, 23, 12	7.1696, 24.32774, 15.16114	5.31345, 12.071, 9.30141	4.61046, 2	3	arrdc1	943
GL173724.1	135906	136575	136170, 136014, 136067	13, 20, 11	15.1723, 20.76851, 13.83651	9.23486, 10.88872, 8.67735	12.13305,	3	NA	3347
GL173726.1	16415	17130	16707, 16698, 16493	14, 13, 7	16.23025, 11.41208, 7.72582	9.67496, 7.04141, 5.6058	13.14538,	3	NA	NA
GL173726.1	67131	67680	67354, 67193, 67261	10, 7, 5	8.93828, 4.16467, 5.19074	6.08275, 3.46025, 4.18277	6.24946, 2	3	NA	NA
GL173726.1	93886	94856	94702, 94548, 94482	15, 23, 3	14.8531, 23.5013, 3.44998	8.68248, 11.57503, 3.02115	11.82783,	3	NA	NA
GL173726.1	135079	135534	135148, 135396, 135436	7, 9, 8	6.94335, 8.21099, 9.46129	5.18876, 5.77858, 6.50801	4.42528, 5	3	NA	NA
GL173726.1	158240	159206	158409, 158614, 158791	5, 9, 3	4.69353, 7.75488, 3.47371	3.9134, 5.501, 3.03189	2.45909, 5	3	NA	NA
GL173726.1	158240	159222	158882, 158614, 158791	6, 9, 3	5.68215, 7.75488, 3.47371	4.49007, 5.501, 3.03189	3.29732, 5	3	NA	NA
GL173726.1	167743	168510	168258, 168299, 168355	10, 15, 8	9.77513, 13.26621, 7.95096	6.5837, 7.71668, 5.71659	7.02302, 1	3	NA	NA
GL173726.1	173129	174308	173539, 173359, 173408	13, 16, 5	13.94585, 14.69174, 4.91762	8.5554, 8.33598, 4.03768	10.95967,	3	NA	NA
GL173727.1	38383	39426	38934, 38839, 38603	11, 8, 4	12.33515, 7.07159, 4.67325	7.91559, 5.20073, 3.78987	9.43699, 4	3	slc1a3	0
GL173727.1	45948	47055	46794, 46485, 46100	8, 16, 9	8.42257, 17.16856, 10.87975	5.97763, 9.82359, 7.23113	5.76336, 1	3	slc1a3	0
GL173727.1	103579	104710	104106, 103815, 104032	13, 23, 6	13.00701, 21.50896, 6.35308	8.00827, 10.38076, 4.85498	10.0703, 1	3	NA	4190
GL173727.1	111882	113040	112426, 112281, 112277	9, 15, 12	6.03824, 9.31256, 10.50755	4.41659, 5.32972, 6.69224	3.58764, 6	3	ugt3a2	6537
GL173727.1	113149	115029	113323, 114278, 114651	7, 26, 12	4.25513, 20.78105, 10.18892	3.51991, 9.01951, 6.49527	2.08427, 1	3	ugt3a2	4548
GL173727.1	113149	115029	114218, 114278, 114651	17, 26, 12	14.9022, 20.78105, 10.18892	8.19901, 9.01951, 6.49527	11.87536,	3	ugt3a2	4548
GL173727.1	141652	143145	141921, 141941, 142264, 142809	7, 20, 5, 4	5.33678, 15.38682, 4.45433, 3.46461	4.22299, 7.67002, 3.77574, 3.1611	2.98597, 1	3	NA	9476
GL173727.1	141652	143145	142766, 141941, 142264, 142809	7, 20, 5, 4	5.39768, 15.38682, 4.45433, 3.46461	4.26169, 7.67002, 3.77574, 3.1611	3.04019, 1	3	NA	9476
GL173727.1	150052	150783	150098, 150486, 150425	6, 9, 15	5.43318, 6.6123, 17.07266	4.34622, 4.78362, 9.97618	3.07284, 4	3	NA	1838
GL173727.1	156640	157138	156845, 157021, 156930	5, 8, 5	4.82177, 6.30984, 5.75061	3.98509, 4.72927, 4.45735	2.51418, 3	3	NA	0
GL173727.1	182839	183505	183080, 183030, 183242	10, 12, 5	8.66311, 7.69911, 5.03069	5.914, 4.94827, 4.09861	5.99101, 5	3	NA	21952
GL173728.1	159443	160045	159476, 159479, 159495	24, 38, 23	29.8059, 47.80625, 30.34798	14.96295, 20.13297, 15.53488	26.30321,	3	ostf1	0
GL173728.1	173002	173780	173713, 173503, 173278	14, 13, 4	16.7649, 13.14514, 4.67325	9.96273, 8.09002, 3.78987	13.6543, 1	3	ostf1	11953
GL173729.1	12675	12908	12864, 12867, 12879	26, 62, 39	30.16559, 76.85542, 50.43494	14.51619, 25.20611, 21.23549	26.65425,	3	cdh26	4053
GL173729.1	22630	23945	23338, 22714, 23725, 23688	9, 5, 8, 8	8.25643, 3.78182, 6.66413, 8.62637	5.80588, 3.36365, 4.9509, 6.08668	5.62516, 1	3	cdh26	0
GL173729.1	29771	30093	29781, 29816, 29810	20, 27, 18	22.17215, 27.67569, 21.11592	11.72403, 12.67118, 11.63307	18.87307,	3	cdh26	0
GL173729.1	112236	112731	112391, 112392, 112392	29, 21, 17	31.83288, 21.11333, 21.64669	14.39823, 10.78774, 12.11304	28.28486,	3	NA	0
GL173729.1	166224	166923	166460, 166528, 166666	5, 8, 6	4.82177, 7.06103, 7.29403	3.98509, 5.19433, 5.30581	2.51418, 4	3	phactr3	0
GL173730.1	56	988	832, 216, 795	43, 72, 33	15.45097, 28.26125, 13.6988	4.19619, 4.91787, 4.57698	12.39707,	3	notum	9796
GL173730.1	3067	3805	3542, 3250, 3277, 3566	37, 65, 30, 25	10.36782, 19.28974, 10.34191, 7.29023	3.31163, 3.74913, 3.77633, 3.16441	7.58462, 1	3	notum	6979
GL173730.1	4053	4751	4576, 4427, 4423	55, 83, 33	22.15264, 30.98905, 12.30986	4.873, 4.74864, 4.13437	18.85422,	3	notum	6033
GL173730.1	5485	6126	5779, 6007, 5768	33, 41, 31	10.61806, 10.63028, 13.62691	3.62949, 3.17168, 4.77649	7.82034, 7	3	notum	4658
GL173731.1	22188	22836	22239, 22725, 22554	8, 13, 6	8.22568, 13.14514, 7.29403	5.8701, 8.09002, 5.30581	5.59681, 1	3	barx1	100164
GL173731.1	49523	50920	50033, 50333, 50333	18, 12, 8	14.2021, 7.15851, 6.47762	7.50755, 4.61876, 4.83468	11.20534,	3	barx1	72080
GL173731.1	69533	69825	69621, 69676, 69665	19, 20, 19	20.21999, 19.78674, 21.51797	10.8531, 10.29739, 11.61684	16.98276,	3	barx1	53175
GL173731.1	78053	78558	78487, 78530, 78521	13, 17, 11	13.42064, 15.99248, 14.03502	8.25183, 8.86336, 8.77076	10.46414,	3	barx1	44442
GL173731.1	93962	94486	94136, 94114, 94124	8, 17, 9	6.59314, 15.87131, 8.79325	4.90679, 8.78961, 6.12267	4.10249, 1	3	barx1	28514
GL173733.1	30353	31698	30900, 31275, 30663, 31298	25, 16, 5, 5	27.23898, 15.04317, 5.31857, 5.31857	13.12872, 8.55029, 4.24819, 4.24819	23.79707,	3	NA	106043
GL173733.1	43071	43945	43529, 43600, 43590	13, 33, 10	7.92498, 23.47926, 7.23146	4.89925, 8.4845, 5.01616	5.31763, 2	3	NA	93796
GL173733.1	53671	54988	54485, 54585, 54447	19, 18, 11	15.45691, 11.37801, 9.80152	7.98408, 5.87862, 6.44625	12.40293,	3	NA	82753
GL173733.1	69741	70496	70162, 70247, 70217	27, 35, 16	22.52291, 26.25048, 13.99771	9.68194, 9.2795, 7.91165	19.21369,	3	NA	67245
GL173733.1	70795	71551	71202, 70956, 71277	20, 32, 11	14.67588, 22.99336, 8.32623	7.26146, 8.50621, 5.52786	11.65904,	3	NA	66190
GL173733.1	74248	74713	74582, 74589, 74594	10, 16, 8	4.38095, 6.59801, 4.56848	3.24409, 3.71333, 3.60747	2.193, 4.2:	3	NA	63028
GL173733.1	86791	88974	87739, 86744, 87989, 86857	19, 20, 21, 15	10.22803, 8.78847, 9.4524, 9.70749	5.07463, 4.23262, 4.39643, 5.55993	7.45145, 6	3	NA	48767
GL173733.1	94381	95685	94948, 95202, 94985	50, 66, 35	45.07191, 56.97772, 34.4428	13.65545, 13.8124, 13.58767	41.29201,	3	NA	42056
GL173733.1	104562	105147	105014, 105077, 104748	26, 31, 10	27.37164, 32.72958, 9.63365	12.85231, 14.15394, 6.50047	23.92596,	3	NA	32594
GL173733.1	118140	119027	118738, 118414, 118376	6, 6, 4	5.96715, 4.93312, 4.67325	4.64927, 4.04501, 3.78987	3.52158, 2	3	NA	18714
GL173733.1	158975	160063	159348, 159410, 159392	9, 21, 16	6.69985, 16.40465, 14.69895	4.83936, 7.98556, 8.34038	4.20005, 1	3	NA	17441
GL173734.1	51004	51526	51279, 51265, 51260	16, 19, 18	12.03393, 14.0344, 21.30401	6.71728, 7.14906, 11.73892	9.15254, 1	3	NA	113031
GL173734.1	110367	110807	110562, 110426, 110665	6, 6, 4	5.96715, 4.53871, 3.94606	4.64927, 3.79731, 3.43278	3.52158, 2	3	NA	53750
GL173734.1	140529	141041	140547, 140559, 140555	151, 200, 116	197.93318, 258.19244, 159.6582	38.9068, 40.3046, 39.74176	192.95398	3	NA	23516
GL173734.1	177193	178175	177734, 177980, 177248	6, 18, 5	5.96715, 17.06771, 4.70718	4.64927, 9.23949, 3.9211	3.52158, 1	3	NA	341
GL173735.1	6099	6615	6502, 6320, 6274	7, 10, 11	6.42049, 9.39128, 14.34865	4.8888, 6.35644, 8.91469	3.94161, 6	3	phc2	11415
GL173735.1	19889	20381	20053, 20351, 20315	6, 14, 6	5.68215, 14.02228, 7.29403	4.49007, 8.40914, 5.30581	3.29732, 1	3	phc2	0
GL173735.1	54256	54739	54535, 54534, 54535	10, 13, 8	8.08479, 10.7674, 7.67847	5.55446, 6.64292, 5.56054	5.46601, 8	3	znf362	4855
GL173735.1	65780	66485	66071, 65916, 66387	13, 8, 6	13.20994, 6.37769, 7.29403	8.12823, 4.77199, 5.30581	10.26285,	3	znf362	16379
GL173735.1	81529	82409	81884, 82050, 81865	8, 12, 9	7.06928, 8.56154, 9.35579	5.19933, 5.48152, 6.44216	4.5414, 6.0	3	trim62	8926
GL173735.1	135289	136121	135645, 135415, 135640	19, 28, 10	18.26836, 30.50449, 11.38247	9.67872, 13.98649, 7.47651	15.09918,	3	TMEM269	168
GL173735.1	145147	145964	145679, 145588, 145467	6, 11, 5	5.96715, 10.60895, 5.9506	4.64927, 6.9343, 4.54784	3.52158, 7	3	TMEM269	10026
GL173736.1	19383	19726	19517, 19426, 19528	7, 9, 5	5.86458, 6.15408, 5.23257	4.55375, 4.4908, 4.20435	3.46146, 3	3	NA	0
GL173736.1	72580	73424	73036, 73030, 73176	31, 28, 20	34.54899, 29.31424, 22.1269	15.23237, 13.27729, 11.69737	30.94432,	3	NA	1692
GL173737.1	16351	16805	16644, 16644, 16609	12, 14, 17	12.3187, 12.54273, 20.87985	7.78073, 7.51218, 11.70696	9.4231, 9.8	3	NA	NA
GL173738.1	16401	16669	16437, 16432, 16435	13, 14, 9	13.63968, 13.38467, 10.72164	8.37925, 8.02582, 7.15493	10.67258,	3	ADTRP	164
GL173738.1	48942	49449	49289, 49408, 49303	6, 11, 7	5.5965, 10.60895, 8.69489	4.44108, 6.9343, 6.06379	3.21976, 7	3	ADTRP	32705
GL173738.1	76902	77492	77368, 77201, 77259	8, 24, 6	6.66791, 19.62119, 4.13532	4.95324, 8.96363, 3.53648	4.17132, 1	3	ADTRP	60665
GL173738.1	80015	80384	80064, 80040, 80046	17, 25, 25	17.48974, 22.47805, 29.93842	9.76779, 10.29723, 14.72403	14.35103,	3	ADTRP	63778
GL173738.1	93674	94640	94363, 94381, 94367	25, 33, 9	25.81344, 34.92198, 10.56949	12.27579, 14.65208, 7.08032	22.40976,	3	ADTRP	77437
GL173738.1	129425	130007	129437, 129465, 129461	9, 21, 13	9.72082, 23.90685, 16.93213	6.64182, 12.45235, 10.12358	6.97155, 2	3	ADTRP	113188
GL173738.1	176011	176221	176042, 176045, 176046	18, 27, 16	16.16093, 24.92765, 17.55092	8.68854, 11.05203, 10.04619	13.07895,	3	ADTRP	159774
GL173738.1	176827	177567	177313, 177262, 177272	16, 19, 10	14.29285, 16.5695, 10.03522	8.0922, 8.65063, 6.73493	11.29159,	3	ADTRP	160590
GL173740.1	45793	46319	46080, 46245, 46091	9, 25, 12	7.80169, 22.12709, 12.7555	5.52977, 10.0929, 8.03189	5.20141, 1	3	NA	44808
GL173741.1	44230	44752	44722, 44714, 44716	31, 47, 24	37.09031, 56.25683, 31.18195	16.73998, 20.91566, 15.73225	33.43702,	3	vwa5a.2	613
GL173741.1	120824	121557	120901, 121282, 121211	10, 10, 6	9.661, 7.4213, 5.83216	6.51661, 5.13639, 4.57461	6.9283, 4.9	3	kif27	6642
GL173741.1	129762	130425	129894, 129894, 129894	28, 50, 32	32.26664, 57.13668, 39.80045	15.03533, 19.99601, 17.94007	28.70906,	3	kif27	15580
GL173741.1	152150	152896	152185, 152168, 152184	45, 48, 36	54.82371, 58.67031, 46.03242	21.03541, 21.8378, 19.95486	50.90322,	3	kif27	37968
GL173741.1	164384	164779	164612, 164746, 164573	19, 25, 14	19.8867, 25.42729, 15.72539	10.65421, 12.04472, 9.39581	16.66031,	3	kif27	50202
GL173742.1	5	533	279, 224, 306	14, 14, 6	16.7649, 14.45897, 7.29403	9.96273, 8.66788, 5.30581	13.6543, 1	3	btnl2	12777
GL173742.1	37868	38446	37946, 38301, 37919	13, 16, 10	14.26982, 17.16856, 13.18184	8.73911, 9.82359, 8.33771	11.26966,	3	btnl2	14122
GL173742.1	43886	44878	44200, 44243, 44175	18, 23, 7	17.38211, 22.17331, 7.50542	9.43026, 10.77791, 5.49244	14.24778,	3	btnl2	20140
GL173742.1	45872	46803	46372, 46400, 46008	11, 21, 8	8.36628, 19.3593, 8.4586	5.55301, 9.73084, 5.99709	5.72708, 1	3	btnl2	22126
GL173742.1	66596	67686	67013, 66975, 67228	17, 17, 13	15.5079, 13.29655, 11.90884	8.56821, 7.22638, 7.34626	12.45101,	3	btnl2	42850
GL173742.1	123290	123587	123335, 123542, 123306	10, 7, 4	10.96713, 5.97725, 4.67325	7.25596, 4.62287, 3.78987	8.14942, 3	3	NA	3107
GL173742.1	142723	143004	142913, 142872, 142757	12, 12, 7	13.8483, 11.86108, 8.69489	8.63436, 7.51216, 6.06379	10.86694,	3	NA	22540
GL173743.1	108006	110053	108412, 108424, 108775	16, 25, 10	15.67432, 23.09161, 9.40066	8.9333, 10.65679, 6.36205	12.61068,	3	ifngr2.2	5043
GL173744.1	4518	4934	4739, 4693, 4695	7, 11, 6	6.16939, 8.66477, 6.76586	4.73936, 5.74034, 5.06179	3.70916, 6	3	chrm2	58304
GL173744.1	52559	53149	52596, 52564, 52569	16, 31, 8	19.7934, 39.13602, 10.1465	11.29109, 17.93949, 6.82176	16.57067,	3	chrm2	10089
GL173744.1	76256	77348	76722, 76936, 76851	11, 7, 7	12.43693, 5.97725, 8.49722	7.97018, 4.62287, 5.97582	9.52692, 3	3	chrm2	11604
GL173744.1	109076	110627	109875, 109681, 110181	16, 13, 10	13.97573, 8.22922, 9.2231	7.8982, 5.08172, 6.25548	10.98763,	3	chrm2	44424
GL173744.1	188870	189257	188971, 189013, 189079	4, 13, 5	3.74195, 13.14514, 5.9506	3.32091, 8.09002, 4.54784	1.6222, 10	3	chrm2	124218
GL173745.1	123	719	332, 240, 239	11, 18, 8	12.43693, 19.95398, 10.1465	7.97018, 10.96581, 6.82176	9.52692, 1	3	NA	NA
GL173746.1	7011	7421	7078, 7080, 7148	6, 7, 6	4.74339, 4.94697, 5.91225	3.92687, 3.9726, 4.61907	2.50328, 2	3	iqsec1	107579
GL173746.1	10570	10886	10768, 10660, 10628	4, 8, 5	3.54306, 6.11443, 4.67395	3.2072, 4.60556, 3.90232	1.57602, 3	3	iqsec1	104114
GL173746.1	56468	57301	56958, 57160, 57040	28, 27, 12	29.91801, 23.32437, 11.60888	13.63686, 10.13211, 7.36202	26.41247,	3	iqsec1	57699
GL173746.1	70924	71269	70985, 70927, 70927	5, 18, 7	4.82177, 18.07902, 6.83761	3.98509, 9.8517, 5.12939	2.51418, 1	3	iqsec1	43731
GL173746.1	79218	79989	79405, 79717, 79596	13, 9, 5	14.88608, 7.21043, 5.85847	9.08033, 5.1623, 4.50665	11.85945,	3	iqsec1	35011
GL173746.1	117438	118461	118241, 118237, 118242	15, 23, 18	13.74446, 20.94885, 20.23857	8.00965, 10.04755, 11.13122	10.77314,	3	iqsec1	0
GL173747.1	80286	81055	80713, 80685, 80693	17, 28, 10	16.10852, 24.14746, 9.2231	8.93393, 10.2723, 6.25548	13.02838,	3	mxd4	17457
GL173747.1	91864	92603	92491, 92337, 91776, 92515	5, 10, 4, 3	4.69353, 8.74956, 4.67325, 3.44998	3.9134, 5.96721, 3.78987, 3.02115	2.45909, 6	3	zfyve28	24806
GL173747.1	138673	139409	139257, 139174, 139343	13, 10, 10	12.49994, 8.74956, 10.59656	7.70504, 5.96721, 7.05292	9.58678, 6	3	zfyve28	0
GL173748.1	122641	123087	122915, 122896, 122891	38, 59, 41	42.11156, 67.40672, 47.79134	16.77862, 21.44719, 18.77435	38.37566,	3	cela2a	18521
GL173748.1	125595	126346	125952, 125953, 125681	12, 15, 9	8.48203, 9.74721, 6.21488	5.43209, 5.58323, 4.52972	5.81957, 7	3	cela2a	15262
GL173748.1	136327	136979	136778, 136826, 136825	10, 21, 17	10.13706, 21.11333, 21.53216	6.7935, 10.78774, 12.05331	7.36489, 1	3	cela2a	4629
GL173748.1	164729	165261	165221, 165217, 164911	14, 14, 5	14.99044, 12.38816, 5.64747	8.97777, 7.41725, 4.40911	11.95856,	3	tmem147	1161
GL173748.1	166734	167548	166837, 166803, 167352	7, 7, 8	6.09019, 4.6991, 7.85773	4.69155, 3.81136, 5.66361	3.63504, 2	3	tmem147	0
GL173748.1	169789	170007	169922, 169975, 169957	15, 31, 16	14.33586, 32.9115, 17.4758	8.36999, 14.26142, 10.00266	11.33286,	3	atp4a	1606
GL173749.1	39635	40221	40069, 40179, 40070	70, 60, 59	79.48769, 64.79188, 72.33491	22.66358, 19.59662, 24.19261	75.29589,	3	NA	60892
GL173749.1	53218	54299	53589, 53818, 53689	7, 7, 5	5.86458, 5.16276, 5.31857	4.55375, 4.11176, 4.24819	3.46146, 2	3	NA	46814
GL173749.1	55274	56267	55716, 55603, 56082	7, 12, 6	6.12952, 10.93692, 7.25145	4.71533, 6.95571, 5.287	3.67231, 8	3	NA	44846
GL173750.1	67114	68241	68042, 67216, 68038	6, 9, 6	4.27757, 7.67202, 5.83216	3.62922, 5.44992, 4.57461	2.10191, 5	3	NA	38892
GL173750.1	145431	145730	145463, 145566, 145537	5, 6, 5	4.82177, 4.83043, 5.9506	3.98509, 3.98131, 4.54784	2.51418, 2	3	NA	13434
GL173751.1	12037	12529	12472, 12483, 12488	22, 25, 11	24.67026, 26.42765, 14.13736	12.59865, 12.64351, 8.81822	21.29787,	3	scube2	1028
GL173751.1	23348	23937	23455, 23469, 23494	10, 14, 5	10.53228, 14.45897, 5.9506	7.01712, 8.66788, 4.54784	7.73927, 1	3	scube2	0
GL173751.1	36450	37274	36585, 36727, 36711	10, 13, 14	9.28413, 11.76644, 16.73403	6.29221, 7.25915, 9.94634	6.574, 9.0(	3	scube2	0
GL173751.1	41471	42005	41987, 41959, 41946	17, 24, 12	16.29925, 22.01781, 13.51536	9.04982, 10.35539, 8.45502	13.21124,	3	scube2	0
GL173751.1	48807	49768	49102, 48971, 49009	13, 18, 14	11.50747, 14.31692, 16.04334	7.10015, 7.57602, 9.57242	8.65224, 1	3	scube2	0
GL173751.1	50776	51699	51469, 51226, 51661	9, 13, 5	7.28297, 9.53929, 4.45433	5.20782, 5.88205, 3.77574	4.7171, 6.9	3	scube2	0
GL173751.1	57993	58999	58504, 58471, 58569	15, 25, 9	15.64184, 23.54439, 9.46739	9.15231, 10.9238, 6.50386	12.57992,	3	scube2	3684
GL173751.1	90732	91416	91255, 90953, 90966	13, 12, 8	10.54397, 7.80498, 7.46678	6.5044, 5.01329, 5.43686	7.7502, 5.3	3	dennd5a	0
GL173751.1	112377	113107	112909, 112423, 112728	9, 10, 7	9.72082, 9.27513, 8.08824	6.64182, 6.2868, 5.7849	6.97155, 6	3	zdhhc13	7893
GL173752.1	55	666	388, 501, 458	8, 17, 7	8.42257, 18.53926, 8.49722	5.97763, 10.38866, 5.97582	5.76336, 1	3	NA	NA
GL173752.1	59487	60307	59780, 60092, 59614	8, 9, 7	8.28589, 7.67202, 8.35502	5.90321, 5.44992, 5.9108	5.65299, 5	3	NA	NA
GL173752.1	65752	66686	66464, 66125, 66465	9, 17, 9	7.39636, 14.31774, 9.70054	5.27874, 7.84337, 6.63089	4.82212, 1	3	NA	NA
GL173752.1	69892	70473	70404, 70354, 70206	9, 13, 5	6.99678, 9.64264, 4.70718	5.02768, 5.94589, 3.9211	4.47395, 7	3	NA	NA
GL173752.1	96286	97108	96961, 96968, 96473	4, 6, 7	3.74195, 4.93312, 8.69489	3.32091, 4.04501, 6.06379	1.6222, 2.7	3	NA	NA
GL173752.1	123215	123698	123263, 123230, 123278	22, 47, 35	25.73329, 55.21653, 45.48785	13.22149, 20.31243, 20.05263	22.33146,	3	NA	NA
GL173752.1	166113	166586	166365, 166121, 166314	9, 14, 3	9.72082, 14.02228, 3.47371	6.64182, 8.40914, 3.03189	6.97155, 1	3	NA	NA
GL173754.1	9216	9529	9395, 9315, 9340	24, 29, 12	31.25967, 34.86947, 16.36823	15.77492, 16.19933, 9.85365	27.72325,	3	NA	0
GL173754.1	57095	57819	57307, 57478, 57261	6, 14, 8	5.96715, 12.54273, 9.60885	4.64927, 7.51218, 6.57807	3.52158, 9	3	NA	31882
GL173754.1	142892	144106	143499, 143300, 143499	29, 27, 18	26.64759, 23.37969, 17.48575	11.35252, 10.16344, 9.49308	23.22161,	3	NA	2251
GL173755.1	7798	8529	8236, 8024, 8144	6, 7, 11	5.96715, 5.97725, 14.75772	4.64927, 4.62287, 9.09568	3.52158, 3	3	NA	6397
GL173755.1	29645	30489	30260, 29863, 30192	8, 12, 6	6.78332, 9.99443, 5.56953	5.02459, 6.37457, 4.42554	4.27811, 7	3	kank2	0
GL173755.1	33925	34633	34325, 34024, 34526	6, 8, 5	4.65447, 5.87115, 4.4844	3.87084, 4.45035, 3.79332	2.42652, 3	3	kank2	342
GL173755.1	122102	122573	122293, 122222, 122279	6, 11, 7	5.96715, 10.26356, 8.28621	4.64927, 6.72731, 5.87881	3.52158, 7	3	timm29	4776
GL173755.1	133259	133915	133485, 133621, 133524	5, 11, 10	4.30277, 9.22091, 11.03772	3.68597, 6.08767, 7.29398	2.12436, 6	3	yipf2	0
GL173755.1	146876	147259	147192, 147190, 147204	12, 11, 10	11.49331, 9.01418, 12.71002	7.29275, 5.9589, 8.12734	8.63898, 6	3	carm1	0
GL173758.1	70998	71987	71538, 71731, 71257, 71656	11, 9, 8, 10	7.65477, 4.64907, 6.38723, 8.57994	5.10598, 3.53152, 4.77798, 5.86268	5.06493, 2	3	NA	8920
GL173758.1	74335	75087	74881, 74559, 74916	8, 13, 13	5.78012, 9.02151, 13.15724	4.39198, 5.56343, 8.09715	3.38637, 6	3	NA	12257
GL173758.1	82329	83092	82954, 82379, 82844	13, 10, 11	13.20994, 9.32635, 12.48556	8.12823, 6.31756, 7.99611	10.26285,	3	NA	20251
GL173758.1	95434	97816	95968, 95916, 95916	40, 43, 17	27.65371, 26.58998, 10.89684	8.66375, 7.6246, 5.82283	24.20218,	3	NA	33356
GL173758.1	109406	109920	109497, 109498, 109525	21, 18, 16	14.0604, 8.41964, 10.77196	6.6744, 4.33577, 5.96852	11.07001,	3	NA	47328
GL173758.1	114921	116789	116448, 116207, 115648, 116623	29, 32, 21, 20	18.00453, 17.59145, 13.44603, 12.13946	6.86316, 6.12537, 6.34586, 5.87038	14.84751,	3	NA	52843
GL173758.1	117846	118124	117890, 117911, 117894	13, 21, 18	4.42148, 7.51295, 9.63356	2.95299, 3.57481, 4.94486	2.2282, 5.0	3	NA	55768
GL173758.1	121917	123297	122725, 122778, 122775	29, 55, 33	15.42696, 34.09069, 22.97402	5.74431, 8.08148, 8.24804	12.37357,	3	NA	59839
GL173758.1	124522	126810	125482, 125958, 125480	24, 32, 18	11.86625, 14.37451, 9.74474	5.02698, 4.91956, 5.00238	8.99294, 1	3	NA	62444
GL173758.1	130444	132613	131730, 131701, 132137	34, 39, 19	24.45458, 25.20894, 15.39325	8.69097, 7.87287, 7.94624	21.08853,	3	NA	68366
GL173758.1	133861	134632	134126, 134124, 134156	19, 39, 15	9.52482, 23.87033, 10.40443	4.72415, 7.3434, 5.97257	6.8008, 20	3	NA	71783
GL173758.1	136693	137437	136791, 137059, 137352	13, 23, 11	7.38922, 14.18811, 9.32791	4.58208, 6.29696, 6.15412	4.81586, 1	3	NA	74615
GL173758.1	145210	146558	146101, 146158, 146085	24, 43, 18	13.11573, 22.2961, 12.48517	5.58738, 6.14573, 6.50258	10.17342,	3	NA	83132
GL173758.1	149410	153742	151566, 150479, 149737, 151062, 153280	42, 76, 28, 34, 22	21.76345, 38.07154, 15.72562, 21.56466, 11.02258	6.11044, 6.49594, 6.04211, 7.3943, 4.95941	18.47671,	3	NA	87332
GL173758.1	155615	156268	155866, 155844, 155841	22, 28, 14	10.53758, 9.98662, 6.94629	4.73957, 3.83174, 4.16739	7.74425, 7	3	NA	93537
GL173758.1	164265	165874	164996, 164962, 165225	45, 60, 23	46.33674, 57.21774, 25.45026	16.195, 15.86294, 12.74129	42.53798,	3	NA	102187
GL173759.1	20525	20932	20608, 20618, 20595	6, 21, 6	5.3939, 20.32736, 6.76586	4.32314, 10.3138, 5.06179	3.0374, 17	3	ST5	0
GL173759.1	33020	34163	33816, 33846, 33656	16, 21, 16	14.13236, 16.79972, 16.76702	7.99402, 8.21417, 9.58727	11.1383, 1	3	ST5	0
GL173759.1	45047	45594	45300, 45363, 45351	6, 9, 7	5.96715, 7.88274, 8.69489	4.64927, 5.57944, 6.06379	3.52158, 5	3	ST5	6857
GL173759.1	59638	60104	59640, 59677, 59688	20, 23, 10	21.79824, 25.81799, 13.18184	11.50312, 12.95947, 8.33771	18.51069,	3	TRIM66	0
GL173759.1	101657	102830	102406, 102405, 102363	11, 15, 9	10.72304, 14.39783, 11.64359	7.00201, 8.40758, 7.57973	7.91902, 1	3	stk33	7522
GL173759.1	123646	124230	123832, 123977, 123964	24, 18, 11	30.95212, 19.97713, 14.75772	15.60555, 10.97931, 9.09568	27.42344,	3	stk33	29511
GL173759.1	143503	143816	143694, 143650, 143589	13, 11, 7	11.76528, 7.46692, 6.55126	7.25843, 4.98819, 4.96529	8.8968, 5.0	3	stk33	49368
GL173759.1	147883	148610	148336, 148233, 148230	10, 15, 9	9.77513, 15.26186, 10.35155	6.5837, 8.92711, 6.97128	7.02302, 1	3	stk33	53748
GL173759.1	165278	165828	165286, 165822, 165455	7, 14, 9	4.60029, 8.78441, 7.51189	3.74674, 5.21877, 5.35071	2.38171, 6	3	stk33	71143
GL173759.1	169331	170180	170111, 170140, 170127	21, 39, 17	19.21346, 38.32878, 16.95256	9.64325, 14.21157, 9.4452	16.01093,	3	stk33	75196
GL173759.1	175358	175728	175503, 175587, 175585	9, 18, 6	6.45525, 15.33505, 6.08001	4.68343, 8.18793, 4.71063	3.97502, 1	3	stk33	81223
GL173761.1	204624	205173	204951, 204970, 205002	13, 15, 8	10.93547, 12.40077, 8.1956	6.74702, 7.18527, 5.85348	8.12008, 9	3	sox5	0
GL173761.1	250819	251214	250937, 250953, 251176	5, 8, 7	4.82177, 6.89656, 8.646	3.98509, 5.09414, 6.04229	2.51418, 4	3	sox5	33435
GL173761.1	265738	266817	266054, 266253, 266138	12, 22, 9	13.8483, 25.85271, 11.64359	8.63436, 13.29074, 7.57973	10.86694,	3	sox5	48354

GL173762.1	19879	20994	20579, 20153, 20481	8, 14, 5	8.42257, 12.92044, 5.9506	5.97763, 7.74345, 4.54784	5.76336, 1	3	xtr-mir-34b-2	6167
GL173762.1	98377	99014	98690, 98669, 98492	13, 8, 7	15.29169, 7.07159, 8.69489	9.29854, 5.20073, 6.06379	12.24396,	3	NA	22777
GL173762.1	135230	136069	135595, 135451, 135538	8, 10, 9	7.02691, 9.39128, 9.82249	5.17362, 6.35644, 6.69629	4.50222, 6	3	NA	8820
GL173763.1	24216	24697	24637, 24512, 24615	20, 23, 8	21.26668, 22.312, 9.31951	11.18692, 10.86102, 6.43944	17.9967, 1	3	nup98	0
GL173763.1	60427	61097	60975, 60942, 60937	14, 25, 13	15.31941, 27.63197, 16.28606	9.1665, 13.36321, 9.81016	12.27033,	3	CHRNA10	0
GL173763.1	70467	71418	71033, 71215, 71237	9, 7, 5	8.45482, 5.36631, 5.91431	5.92419, 4.24177, 4.53172	5.79307, 3	3	CHRNA10	5964
GL173763.1	116658	117358	116988, 117222, 116735	10, 7, 5	11.06011, 5.97725, 5.9506	7.306, 4.62287, 4.54784	8.22712, 3	3	znf347	15705
GL173763.1	160111	160525	160504, 160510, 160474	14, 21, 11	13.32664, 21.26419, 12.19945	7.99066, 10.87863, 7.8422	10.37457,	3	stard10	2632
GL173763.1	164562	165739	164644, 165510, 164965	7, 10, 6	5.07797, 6.96485, 4.88363	4.05723, 4.84715, 4.01438	2.75052, 4	3	stard10	7083
GL173763.1	164788	165739	165611, 165510, 164965	7, 10, 6	5.97522, 6.96485, 4.88363	4.62162, 4.84715, 4.01438	3.52869, 4	3	stard10	7309
GL173763.1	167023	167332	167167, 167147, 167166	7, 10, 7	6.16939, 8.16488, 6.83761	4.73936, 5.60458, 5.12939	3.70916, 5	3	stard10	9544
GL173764.1	22011	22432	22183, 22377, 22168	8, 6, 9	7.62407, 4.93312, 11.21577	5.52896, 4.04501, 7.38849	5.03638, 2	3	dlg4	355
GL173764.1	48053	49236	48307, 48139, 48148	12, 13, 7	13.05874, 13.14514, 8.21885	8.20296, 8.09002, 5.84717	10.11961,	3	dlg4	26397
GL173764.1	51567	52033	51997, 51981, 51963	7, 9, 7	6.69449, 8.21099, 8.21885	5.04797, 5.77858, 5.84717	4.1953, 5.7	3	dlg4	29911
GL173764.1	75821	76692	76212, 76144, 76419	18, 22, 20	16.21741, 19.7681, 20.04603	8.72284, 9.66336, 10.45389	13.13331,	3	clec10a	33025
GL173764.1	82385	83577	82903, 83006, 82880	18, 24, 19	12.98682, 17.63382, 17.44852	6.79206, 7.85932, 9.18195	10.05096,	3	clec10a	26140
GL173764.1	88168	89098	88605, 88739, 88879	18, 24, 19	16.04928, 21.89488, 19.82575	8.62074, 10.28282, 10.61774	12.97113,	3	clec10a	20619
GL173764.1	103062	103522	103227, 103115, 103177	13, 29, 11	12.09198, 27.49738, 11.08949	7.45795, 11.84146, 7.21705	9.20698, 2	3	clec10a	6195
GL173764.1	136774	137286	136999, 137065, 137065	11, 23, 11	9.05039, 19.24395, 10.47247	5.98149, 9.04725, 6.85286	6.35485, 1	3	TNK1	328
GL173764.1	180858	181088	181031, 181032, 181048	7, 10, 4	7.1696, 8.24345, 4.67325	5.31345, 5.65367, 3.78987	4.61046, 5	3	NA	16461
GL173765.1	122167	122966	122665, 122574, 122464	10, 15, 5	8.28204, 11.60543, 4.14842	5.67774, 6.69836, 3.59261	5.64968, 8	3	scx	14846
GL173765.1	137289	137719	137670, 137709, 137690	19, 22, 7	20.6571, 19.9442, 6.79502	11.11239, 9.76825, 5.10529	17.40645,	3	hsf1	1902
GL173765.1	141766	142255	142086, 141963, 141944	6, 12, 6	3.725, 7.77827, 5.39683	3.26525, 4.99688, 4.32487	1.6222, 5.3	3	hsf1	0
GL173767.1	41033	41941	41912, 41909, 41909	136, 155, 104	149.76915, 171.48772, 125.21449	25.84926, 26.83458, 28.9938	145.04518	3	NA	2176
GL173767.1	44203	44734	44682, 44684, 44714	73, 77, 50	61.64907, 61.92491, 45.43616	13.65224, 12.74491, 13.82962	57.64343,	3	NA	5346
GL173767.1	49300	49715	49662, 49669, 49666	10, 21, 12	5.63009, 13.40365, 10.07459	4.00614, 6.32345, 6.42436	3.2518, 10	3	NA	10443
GL173767.1	56177	56931	56565, 56584, 56462	8, 7, 7	7.02691, 5.16276, 7.40077	5.17362, 4.11176, 5.43746	4.50222, 2	3	NA	17320
GL173767.1	90954	91303	91060, 91069, 91051	6, 6, 3	5.96715, 4.93312, 3.47371	4.64927, 4.04501, 3.03189	3.52158, 2	3	NA	52097
GL173767.1	107234	107883	107705, 107825, 107715	8, 12, 5	8.42257, 11.86108, 5.9506	5.97763, 7.51216, 4.54784	5.76336, 9	3	NA	68377
GL173767.1	161740	162013	161988, 161987, 161898	12, 16, 4	13.8483, 17.16856, 4.67325	8.63436, 9.82359, 3.78987	10.86694,	3	NA	122883
GL173768.1	160420	161894	161427, 161181, 161041	32, 40, 30	30.88024, 38.08473, 30.75087	12.69434, 13.69629, 13.37305	27.35276,	3	ccdc112	9504
GL173770.1	17061	18775	17992, 18098, 18556	18, 28, 23	16.56571, 25.12925, 23.90484	8.93449, 10.82777, 11.81735	13.46708,	3	atxn10	469
GL173770.1	29529	30351	29934, 30059, 30139	11, 23, 9	9.26086, 21.07044, 8.60738	6.1125, 10.11973, 6.01421	6.5517, 17	3	atxn10	0
GL173770.1	40944	41235	41006, 41168, 41180	5, 9, 6	4.77263, 7.35794, 5.75442	3.9578, 5.25474, 4.53101	2.51418, 4	3	atxn10	0
GL173770.1	66679	67494	67037, 66998, 67041	16, 16, 14	15.16035, 14.74902, 15.35015	8.6216, 8.37095, 9.184	12.12209,	3	atxn10	0
GL173770.1	78676	79505	79274, 79399, 78780	5, 9, 4	4.82177, 7.88274, 4.18775	3.98509, 5.57944, 3.55872	2.51418, 5	3	atxn10	0
GL173770.1	123493	123883	123622, 123747, 123729	6, 6, 4	5.96715, 4.93312, 4.67325	4.64927, 4.04501, 3.78987	3.52158, 2	3	wnt7b	0
GL173770.1	152823	153715	153185, 153167, 153216	17, 17, 10	17.19112, 15.75222, 11.17239	9.58879, 8.71708, 7.36591	14.0641, 1	3	wnt7b	12078
GL173770.1	157971	159447	158406, 158370, 158440	12, 18, 7	11.79509, 18.75856, 8.646	7.47301, 10.25958, 6.04229	8.92495, 1	3	wnt7b	17226
GL173772.1	22	1476	988, 339, 487	19, 22, 14	22.02449, 25.85271, 18.52583	11.90906, 13.29074, 10.84669	18.72942,	3	shoc2	31073
GL173772.1	5511	5959	5830, 5814, 5797	8, 7, 5	7.15567, 5.82304, 5.14967	5.25151, 4.52813, 4.16141	4.61046, 3	3	shoc2	26590
GL173772.1	7815	8233	7971, 8159, 8103	5, 14, 7	4.82177, 14.45897, 8.69489	3.98509, 8.66788, 6.06379	2.51418, 1	3	shoc2	24316
GL173772.1	13737	14111	13949, 13949, 13799	12, 16, 7	13.8483, 17.16856, 8.69489	8.63436, 9.82359, 6.06379	10.86694,	3	shoc2	18438
GL173772.1	44491	45129	44842, 44686, 44785	15, 7, 9	16.90423, 5.42726, 10.01275	9.88146, 4.28044, 6.79683	13.78827,	3	shoc2	1655
GL173772.1	53428	54052	53817, 53846, 53576	7, 14, 5	5.42862, 12.09105, 5.19074	4.2813, 7.23439, 4.18277	3.06982, 9	3	pdcd4	0
GL173772.1	130984	131644	131462, 131583, 131163	16, 17, 5	17.41877, 16.11578, 5.91431	9.96955, 8.93835, 4.53172	14.28292,	3	rbm20	11103
GL173772.1	154443	154884	154454, 154480, 154470	14, 14, 7	15.31941, 14.45897, 7.50542	9.1665, 8.66788, 5.49244	12.27033,	3	rbm20	34562
GL173775.1	22066	23150	22950, 22700, 22675	13, 22, 7	13.49269, 24.36754, 8.69489	8.29387, 12.41949, 6.06379	10.53271,	3	rnf208	8608
GL173775.1	98263	98553	98352, 98353, 98400	14, 27, 8	12.86267, 25.12134, 9.76268	7.70816, 11.16466, 6.64964	9.93295, 2	3	U2	3686
GL173775.1	129338	130831	129613, 130379, 129623	12, 14, 4	12.45843, 14.45897, 4.67325	7.86167, 8.66788, 3.78987	9.54648, 1	3	ddr2	0
GL173775.1	129433	130854	130815, 130379, 129623	7, 14, 4	6.60038, 14.45897, 4.67325	4.99378, 8.66788, 3.78987	4.10761, 1	3	ddr2	0
GL173775.1	137359	137984	137826, 137882, 137560	5, 6, 3	4.82177, 4.93312, 3.47371	3.98509, 4.04501, 3.03189	2.51418, 2	3	ddr2	0
GL173776.1	552	1252	647, 698, 575	11, 7, 5	12.43693, 5.97725, 5.9506	7.97018, 4.62287, 4.54784	9.52692, 3	3	fbxo24	510
GL173776.1	6607	7060	6687, 6767, 6784	11, 8, 4	10.9046, 5.35923, 4.67325	7.10903, 4.12054, 3.78987	8.08987, 3	3	fbxo24	0
GL173776.1	12685	14406	13679, 13629, 13667	34, 40, 25	34.1882, 38.3773, 26.08981	13.8297, 13.854, 12.44125	30.5914, 3	3	fbxo24	544
GL173776.1	24461	25276	25083, 24839, 24620	7, 7, 10	7.1696, 5.97725, 13.18184	5.31345, 4.62287, 8.33771	4.61046, 3	3	zmynd15	0
GL173776.1	75736	77066	76233, 76225, 76130	26, 37, 12	24.26079, 36.25517, 11.92663	11.0052, 13.83603, 7.55094	20.90031,	3	kif1c	1384
GL173776.1	93396	94607	93622, 93704, 93601	14, 11, 10	13.20712, 8.44639, 9.82971	7.91811, 5.60333, 6.61563	10.26077,	3	kif1c	19044
GL173776.1	102036	102310	102282, 102280, 102274	28, 45, 24	33.20707, 50.06706, 30.41517	15.59147, 18.27985, 15.30667	29.62933,	3	camta2	11575
GL173776.1	137883	138712	138144, 138136, 138329	10, 9, 11	11.06011, 8.21099, 12.41242	7.306, 5.77858, 7.95707	8.22712, 5	3	camta2	3453
GL173776.1	148028	148302	148201, 148168, 148179	22, 20, 13	21.33403, 17.66446, 12.54936	10.60109, 9.01671, 7.73479	18.06131,	3	spag7	0
GL173776.1	151925	152428	152184, 152165, 152165	12, 26, 19	8.94411, 24.23472, 21.02007	5.71985, 10.98992, 11.32618	6.25442, 2	3	spag7	0
GL173777.1	21301	21716	21357, 21370, 21654	4, 9, 3	3.74195, 7.67202, 3.47371	3.32091, 5.44992, 3.03189	1.6222, 5.2	3	NA	0
GL173777.1	35003	36187	35518, 35801, 35511	21, 20, 19	21.17819, 19.10348, 21.43283	10.82682, 9.88447, 11.56736	17.91116,	3	NA	0
GL173777.1	54469	55307	54588, 54748, 54562	19, 31, 11	20.13535, 32.72958, 13.46208	10.80268, 14.15394, 8.49639	16.90088,	3	adamts13	1439
GL173777.1	98592	99089	98941, 99037, 99039	5, 19, 17	3.88983, 18.08435, 20.57409	3.43212, 9.56723, 11.54114	1.75752, 1	3	slc2a6	1493
GL173778.1	6570	7072	7046, 7036, 7045	72, 112, 72	86.52798, 132.84331, 92.19866	25.39323, 28.74855, 28.50342	82.26621,	3	iqcb1	12567
GL173778.1	10904	11306	11242, 11262, 11136	14, 39, 12	8.23285, 28.74869, 7.43533	4.89559, 9.38332, 4.78692	5.60367, 2	3	iqcb1	8333
GL173778.1	32109	32907	32775, 32243, 32543	8, 7, 6	8.42257, 5.97725, 7.29403	5.97763, 4.62287, 5.30581	5.76336, 3	3	iqcb1	3644
GL173778.1	43389	44107	44051, 43518, 43502	7, 11, 5	7.1696, 10.60895, 5.9506	5.31345, 6.9343, 4.54784	4.61046, 7	3	cfap58	8599
GL173778.1	56497	57525	57047, 57058, 57372	13, 34, 11	10.10242, 27.75525, 10.66921	6.23056, 10.31256, 6.9701	7.3336, 24	3	cfap58	0
GL173778.1	58227	59128	58675, 58543, 58595	11, 29, 13	8.84943, 22.2713, 13.33541	5.85597, 8.95169, 8.20195	6.16564, 1	3	cfap58	0
GL173778.1	65595	66364	65638, 65702, 65993	23, 18, 6	26.5636, 19.68157, 6.35308	13.3989, 10.80641, 4.85498	23.13966,	3	cfap58	0
GL173778.1	102960	103647	103466, 103498, 103439	8, 15, 14	5.13458, 10.97496, 14.34766	3.97494, 6.31545, 8.60225	2.80161, 8	3	itprip	7807
GL173778.1	132265	132916	132408, 132461, 132291	24, 25, 28	22.82755, 22.90359, 31.43825	10.8357, 10.54631, 14.54294	19.50902,	3	NA	280
GL173779.1	34108	34495	34385, 34439, 34313	11, 14, 3	11.15818, 12.43922, 3.44998	7.25692, 7.44862, 3.02115	8.32008, 9	3	plxna3	719
GL173779.1	74404	75205	74929, 74912, 74748	7, 10, 8	6.42049, 8.88645, 9.25066	4.8888, 6.0511, 6.40569	3.94161, 6	3	plxna3	1021
GL173779.1	119700	120821	120473, 120207, 120214	28, 32, 24	29.28762, 31.00894, 26.55881	13.26146, 12.768, 13.06784	25.7973, 2	3	fam50a	6176
GL173779.1	150233	151252	150685, 151185, 150767	4, 15, 10	3.74195, 12.96377, 11.997	3.32091, 7.53102, 7.78835	1.6222, 10	3	gdi1	0
GL173779.1	156157	156347	156316, 156309, 156305	15, 17, 7	16.11735, 14.56175, 8.49722	9.43066, 7.99171, 5.97582	13.03694,	3	atp6ap1.2	0
GL173780.1	21	728	427, 433, 524	36, 29, 11	38.59159, 27.09161, 12.63525	15.57248, 11.60727, 8.07536	34.9121, 2	3	NA	NA
GL173780.1	18520	20139	19355, 18829, 19518	58, 73, 36	44.65045, 48.14585, 29.58241	11.15026, 9.29619, 10.62744	40.87629,	3	NA	NA
GL173780.1	21743	22899	22618, 22456, 22635	39, 60, 27	25.97136, 37.11064, 20.15328	8.18471, 8.19829, 8.39761	22.56277,	3	NA	NA
GL173780.1	60688	61596	60971, 61300, 61031	22, 33, 16	19.91488, 32.49356, 16.972	9.75078, 13.24221, 9.70822	16.68802,	3	NA	NA
GL173780.1	77987	78416	78161, 78254, 78025	11, 16, 9	7.09825, 9.38905, 6.26241	4.75778, 5.17793, 4.56014	4.5697, 6.8	3	NA	NA
GL173780.1	78570	79112	78804, 78868, 78742	9, 12, 11	5.18738, 5.84199, 8.12222	3.87239, 3.84002, 5.39965	2.85031, 3	3	NA	NA
GL173780.1	79429	80119	79977, 79803, 79878	10, 18, 9	6.03527, 10.89261, 6.168	4.25963, 5.61234, 4.49971	3.58483, 8	3	NA	NA
GL173780.1	82237	82655	82526, 82554, 82475	12, 23, 11	7.57441, 14.61996, 7.53807	4.87186, 6.51541, 5.03278	4.98873, 1	3	NA	NA
GL173780.1	101089	101733	101578, 101463, 101465	14, 16, 10	8.04702, 8.2015, 6.97437	4.78808, 4.53124, 4.85319	5.43132, 5	3	NA	NA
GL173780.1	120764	121272	121108, 120998, 121028	10, 23, 8	8.32267, 18.85109, 7.04274	5.70305, 8.82063, 5.18324	5.6878, 15	3	NA	NA
GL173780.1	130901	131846	131524, 131448, 131518	17, 20, 24	11.53451, 12.37016, 19.61938	6.18731, 5.99201, 8.9626	8.6788, 9.6	3	NA	NA
GL173780.1	135971	136578	136125, 136408, 136430	19, 32, 23	12.05549, 23.11039, 18.39355	6.03614, 8.56262, 8.5589	9.17245, 1	3	NA	NA
GL173780.1	147399	148386	147747, 147868, 148053	12, 8, 15	10.44071, 5.78452, 13.91459	6.65101, 4.39481, 8.11358	7.65237, 3	3	NA	NA
GL173780.1	149619	150281	149860, 150054, 150116	9, 12, 7	6.69985, 9.20568, 4.85166	4.83936, 5.88305, 3.91076	4.20005, 6	3	NA	NA
GL173781.1	122760	123299	123189, 123113, 122833	11, 12, 4	11.71035, 11.86108, 3.64768	7.57196, 7.51216, 3.26758	8.8442, 9.1	3	NA	21823
GL173782.1	35725	36746	36293, 36326, 36120	7, 16, 5	7.1696, 17.16856, 5.91431	5.31345, 9.82359, 4.53172	4.61046, 1	3	NA	82760
GL173782.1	50895	51536	51168, 51089, 51134	11, 11, 7	12.43693, 10.60895, 8.69489	7.97018, 6.9343, 6.06379	9.52692, 7	3	NA	67970
GL173782.1	83528	83956	83884, 83895, 83906	10, 16, 11	8.66311, 12.25152, 11.31465	5.914, 6.84834, 7.3472	5.99101, 9	3	NA	35550
GL173783.1	47514	48068	47928, 47853, 47855	9, 11, 4	9.72082, 10.60895, 4.67325	6.64182, 6.9343, 3.78987	6.97155, 7	3	clasp1	10926
GL173783.1	77283	77662	77633, 77612, 77652	27, 28, 24	35.24656, 34.21132, 31.58771	17.1108, 16.18039, 15.95403	31.62888,	3	clasp1	0
GL173783.1	137341	137972	137837, 137866, 137859	7, 23, 9	5.39768, 20.1396, 9.19414	4.26169, 9.56971, 6.35176	3.04019, 1	3	clasp1	2411
GL173785.1	13915	14437	14025, 13934, 14191	10, 8, 3	8.84451, 7.07159, 3.34298	6.02544, 5.20073, 2.97156	6.16137, 4	3	NA	NA
GL173785.1	65690	66378	65719, 65719, 65728	15, 24, 14	17.48779, 25.91323, 17.61756	10.20672, 12.68264, 10.40352	14.34959,	3	NA	NA
GL173785.1	76955	77443	76977, 77172, 77035	19, 25, 21	18.89089, 22.71902, 24.0973	10.05535, 10.4381, 12.56358	15.69992,	3	NA	NA
GL173785.1	87345	87622	87408, 87454, 87469	9, 14, 9	9.50098, 12.09105, 11.64359	6.52233, 7.23439, 7.57973	6.77745, 9	3	NA	NA
GL173785.1	141479	141925	141896, 141877, 141898	26, 49, 22	29.18746, 55.96937, 28.44344	13.93627, 19.80236, 14.74701	25.69939,	3	NA	NA
GL173786.1	36665	37356	37117, 36903, 37261	23, 18, 9	26.13432, 18.44896, 11.64359	13.14642, 10.0742, 7.57973	22.72107,	3	NA	7328
GL173786.1	59401	59940	59879, 59886, 59878	20, 41, 23	19.42489, 44.395, 25.79135	10.07876, 16.80408, 12.94369	16.21503,	3	NA	4702
GL173786.1	130980	131406	131197, 131174, 131172	20, 29, 13	18.39153, 25.25061, 12.39468	9.4544, 10.56277, 7.64153	15.21806,	3	NA	57180
GL173786.1	148126	148466	148257, 148289, 148370	13, 16, 10	8.50204, 10.2168, 8.25787	5.24662, 5.64681, 5.66266	5.83844, 7	3	NA	74326
GL173786.1	150793	152047	151580, 151994, 151996	33, 37, 20	33.31306, 35.87548, 22.89302	13.71418, 13.62471, 12.14573	29.7331, 3	3	NA	76993
GL173787.1	112	3663	2602, 2189, 2836	48, 60, 45	35.50181, 40.10537, 37.31723	10.00866, 9.12851, 11.6156	31.87859,	3	NA	46703
GL173787.1	77487	78148	77893, 78004, 77810	14, 11, 7	9.55023, 5.97505, 5.27202	5.67626, 4.06653, 4.18171	6.82374, 3	3	NA	0
GL173787.1	79417	80268	79963, 79666, 80013	16, 19, 10	10.43961, 12.04257, 7.62252	5.77528, 6.0291, 5.26361	7.65157, 9	3	NA	0
GL173789.1	97133	97787	97583, 97628, 97703	20, 14, 11	17.05264, 8.83702, 9.18051	8.65057, 5.24989, 6.06254	13.93142,	3	atp5g1	28265
GL173789.1	100603	101061	100791, 100824, 100807	16, 30, 12	13.3378, 27.71453, 10.71774	7.50821, 11.61145, 6.82153	10.38551,	3	atp5g1	24991
GL173789.1	143621	144256	143806, 143752, 143825	11, 14, 4	8.54584, 12.23767, 4.64325	5.66575, 7.32468, 3.77643	5.88025, 9	3	atp5g1	5394
GL173790.1	6762	7262	6848, 6982, 7092	8, 6, 10	8.16648, 4.45754, 10.97191	5.83735, 3.74538, 7.25854	5.54098, 2	3	NA	NA
GL173790.1	24704	25384	24826, 24779, 24922	6, 10, 6	5.96715, 9.32635, 6.87953	4.64927, 6.31756, 5.11627	3.52158, 6	3	NA	NA
GL173790.1	53891	54300	54260, 54279, 54255	26, 28, 17	32.28932, 34.34391, 24.08167	15.75453, 16.25767, 13.29929	28.73106,	3	NA	NA
GL173790.1	81436	81840	81680, 81530, 81823	9, 7, 5	8.94274, 5.97725, 5.40789	6.20889, 4.62287, 4.29296	6.2534, 3.6	3	NA	NA
GL173790.1	103916	104453	104112, 104143, 104083	8, 16, 5	6.86256, 13.5443, 4.99237	5.0733, 7.63435, 4.0781	4.3506, 10	3	NA	NA
GL173791.1	996	1496	1184, 1195, 1278	14, 27, 4	14.23954, 30.88259, 4.67325	8.53829, 14.58271, 3.78987	11.24094,	3	NA	22341
GL173791.1	82601	83142	82876, 82769, 82876	9, 8, 13	8.55802, 6.24338, 15.70541	5.98518, 4.6873, 9.51556	5.8909, 3.9	3	NA	7440
GL173791.1	107361	107799	107426, 107490, 107404	17, 28, 8	15.00845, 23.60795, 8.14528	8.26375, 9.9714, 5.82558	11.97619,	3	NA	32200
GL173791.1	127298	127774	127367, 127520, 127369	9, 17, 12	7.55321, 14.81481, 13.01912	5.37637, 8.14576, 8.18077	4.96981, 1	3	NA	52137
GL173791.1	146142	146777	146425, 146442, 146338	18, 18, 7	18.07742, 18.00717, 8.646	9.85073, 9.80837, 6.04229	14.9177, 1	3	NA	70981
GL173792.1	24555	26120	24867, 25171, 25777	20, 34, 18	18.5756, 30.9966, 18.60267	9.56551, 12.03444, 10.16634	15.39539,	3	NA	3179
GL173792.1	33164	33989	33421, 33408, 33636	29, 55, 27	31.05973, 60.48025, 31.16394	13.938, 19.54132, 14.74952	27.52836,	3	NA	0
GL173792.1	57177	57948	57622, 57349, 57688	7, 7, 8	6.09019, 5.45824, 7.30639	4.69155, 4.30004, 5.34182	3.63504, 3	3	NA	3863
GL173793.1	8216	8887	8660, 8467, 8470	6, 7, 6	5.96715, 5.97725, 7.29403	4.64927, 4.62287, 5.30581	3.52158, 3	3	mocs1	124015
GL173793.1	48679	49633	49097, 49100, 49087	48, 46, 33	56.70724, 48.96656, 38.55811	20.69793, 17.21981, 16.80216	52.76192,	3	mocs1	83269
GL173793.1	113788	114925	114531, 114463, 114656	13, 16, 10	12.74759, 15.60807, 10.53703	7.85373, 8.89325, 7.01977	9.82421, 1	3	mocs1	17977
GL173793.1	154611	155331	155049, 155059, 155046	7, 13, 6	6.69449, 13.14514, 5.99481	5.04797, 8.09002, 4.6644	4.1953, 10	3	mocs1	9581
GL173794.1	132	1022	474, 457, 776	12, 12, 9	13.8483, 11.86108, 10.72164	8.63436, 7.51216, 7.15493	10.86694,	3	c17orf58	0
GL173794.1	4125	4609	4143, 4471, 4428	5, 8, 8	3.86321, 5.81313, 8.24665	3.41531, 4.41317, 5.88165	1.73293, 3	3	c17orf58	0
GL173794.1	36934	37800	37413, 36953, 37488, 37136	16, 11, 15, 9	13.77263, 5.80451, 9.26058, 8.60738	7.77396, 3.96355, 5.29965, 6.01421	10.80034,	3	bptf	0
GL173794.1	39083	39746	39283, 39389, 39350	8, 15, 8	5.89711, 9.97812, 7.50809	4.46697, 5.71925, 5.46116	3.49291, 7	3	bptf	0
GL173794.1	46573	47195	46929, 47038, 46915	24, 16, 18	25.63312, 15.04317, 20.75291	12.51508, 8.55029, 11.42699	22.23394,	3	bptf	99
GL173794.1	56769	57497	57271, 57238, 56912	6, 8, 8	5.96715, 7.07159, 10.1465	4.64927, 5.20073, 6.82176	3.52158, 4	3	NA	2443
GL173794.1	82273	82549	82420, 82399, 82405	12, 17, 8	13.8483, 18.56115, 10.1465	8.63436, 10.40145, 6.82176	10.86694,	3	NA	16686
GL173794.1	88073	88878	88326, 88287, 88530	20, 45, 15	23.40409, 48.60518, 18.13606	12.44081, 17.45266, 10.55788	20.06837,	3	XB-GENE-959703	12710
GL173794.1	95476	96607	95950, 96210, 95986	15, 28, 14	12.25119, 23.82061, 13.70709	7.09349, 10.08962, 8.22039	9.35859, 2	3	XB-GENE-959703	4981
GL173794.1	163558	164344	163632, 163822, 163808	6, 25, 8	5.24248, 27.53425, 8.6841	4.2332, 13.30495, 6.11715	2.89962, 2	3	psmd12	9105
GL173794.1	163558	164369	164119, 163822, 163808	9, 25, 8	8.66407, 27.53425, 8.6841	6.04743, 13.30495, 6.11715	5.99101, 2	3	psmd12	9080
GL173796.1	1118	1790	1314, 1197, 1345	6, 17, 5	5.96715, 18.56115, 5.9506	4.64927, 10.40145, 4.54784	3.52158, 1	3	st8sia1	7784
GL173796.1	16417	16737	16470, 16573, 16629	8, 9, 5	7.88469, 7.88274, 5.54867	5.67897, 5.57944, 4.3619	5.27935, 5	3	st8sia1	4503
GL173796.1	29099	29998	29501, 29462, 29870	14, 17, 9	14.91094, 15.63512, 9.8849	8.9318, 8.64575, 6.72947	11.88377,	3	NA	15785
GL173796.1	65562	65954	65630, 65643, 65626	8, 10, 8	7.47668, 9.39128, 9.68495	5.44269, 6.35644, 6.61366	4.89713, 6	3	tmem63b	12177
GL173796.1	98574	98981	98646, 98702, 98836	6, 7, 4	5.96715, 5.39662, 4.67325	4.64927, 4.26102, 3.78987	3.52158, 3	3	mrpl14	6498
GL173796.1	105948	106906	106181, 106661, 106663	11, 11, 7	9.21796, 6.91365, 6.47418	6.08584, 4.64291, 4.92032	6.51275, 4	3	mrpl14	0
GL173796.1	112253	113374	112873, 113091, 112977	26, 20, 11	23.62264, 15.26423, 10.10247	10.63249, 7.59908, 6.62982	20.28033,	3	mrpl14	2566
GL173797.1	54954	56628	55612, 55584, 55603	174, 241, 133	123.73451, 179.28453, 102.22947	11.69969, 12.97451, 12.94455	119.15984	3	5S_rRNA	0
GL173797.1	58438	59831	59027, 58857, 58920	172, 209, 123	120.86245, 142.58592, 90.69672	11.46055, 11.10168, 11.95303	116.30672	3	5S_rRNA	0
GL173797.1	139418	139890	139499, 139497, 139495	62, 52, 56	68.57453, 53.85582, 67.41307	20.67757, 17.31083, 22.91494	64.49225,	3	U4	15788
GL173797.1	144718	145578	145019, 145023, 145050	49, 54, 46	55.77095, 60.30513, 52.84224	19.68981, 19.90236, 19.41197	51.83777,	3	U4	21088
GL173798.1	49240	49844	49666, 49475, 49610	22, 41, 21	22.60711, 45.42588, 28.14653	11.36706, 17.39706, 14.80483	19.29541,	3	capsl	3045
GL173798.1	63846	65419	64310, 64132, 64336	6, 11, 6	5.63898, 9.17879, 5.46454	4.46544, 6.06147, 4.36458	3.25772, 6	3	capsl	0
GL173798.1	142067	143758	142328, 142638, 142563	10, 21, 16	7.57902, 15.06496, 15.94441	5.23613, 7.22584, 9.0962	4.99303, 1	3	ugt3a2	18960
GL173799.1	6997	7657	7337, 7489, 7040	4, 6, 4	3.74195, 4.93312, 4.67325	3.32091, 4.04501, 3.78987	1.6222, 2.7	3	NA	20189
GL173799.1	13046	13858	13247, 13246, 13406	11, 9, 6	7.59564, 5.89579, 4.50415	5.06888, 4.32532, 3.77524	5.00893, 3	3	NA	13988
GL173799.1	13165	14070	13837, 13660, 13406	11, 10, 6	7.39504, 6.66155, 4.50415	4.94318, 4.65493, 3.77524	4.82154, 4	3	NA	13776
GL173799.1	26775	27433	27287, 27178, 27104	11, 15, 6	10.1122, 13.11311, 6.71074	6.63573, 7.62272, 5.03498	7.34229, 1	3	NA	413
GL173799.1	126896	127275	127258, 127248, 127236	14, 14, 9	14.67861, 12.59519, 8.88966	8.79666, 7.54437, 6.17838	11.66087,	3	NA	0
GL173799.1	137954	138722	138241, 138203, 138233	15, 21, 12	16.36908, 19.11862, 13.08717	9.57629, 9.58634, 8.21885	13.27778,	3	NA	766
GL173800.1	736	2268	1469, 1190, 1132	12, 18, 9	12.05017, 16.94269, 10.14484	7.62377, 9.16356, 6.86555	9.16715, 1	3	U3	0
GL173800.1	12385	15489	13106, 13161, 14655	9, 19, 15	6.51482, 14.38143, 13.52972	4.72146, 7.35052, 7.87823	4.02888, 1	3	U3	0
GL173800.1	13753	16504	14660, 13161, 15935, 14655, 15742, 16086	13, 19, 11, 15, 9, 7	10.0641, 14.38143, 5.8983, 13.52972, 6.61462, 4.68368	6.20681, 7.35052, 4.02011, 7.87823, 4.7851, 3.80129	7.29669, 1	3	U3	0
GL173801.1	3256	3965	3659, 3663, 3719	34, 67, 29	27.67217, 58.41079, 25.74658	10.27002, 14.0928, 10.84098	24.22022,	3	cck	15977
GL173801.1	7773	9254	7868, 7908, 8455, 8955	11, 15, 14, 6	5.12023, 5.71023, 5.5721, 4.03691	3.55706, 3.39385, 3.43633, 3.4719	2.78902, 3	3	cck	10688
GL173802.1	6097	6436	6404, 6421, 6398	6, 7, 7	5.16999, 5.71889, 7.40077	4.18963, 4.46356, 5.43746	2.83325, 3	3	NA	0
GL173802.1	16056	16323	16127, 16180, 16179	10, 11, 10	9.18236, 10.60895, 11.38247	6.23091, 6.9343, 7.47651	6.47811, 7	3	NA	0
GL173803.1	141701	142469	141925, 142338, 141813	7, 7, 6	7.1696, 5.97725, 7.29403	5.31345, 4.62287, 5.30581	4.61046, 3	3	tmem178b	0
GL173805.1	1486	1806	1637, 1637, 1637	10, 23, 7	11.06011, 24.50192, 8.08824	7.306, 12.17534, 5.7849	8.22712, 2	3	RAB3IL1	18606
GL173806.1	36894	37220	37152, 37164, 37146	16, 31, 9	17.08121, 30.42189, 10.87975	9.7724, 12.80311, 7.23113	13.95894,	3	sfxn3	0
GL173807.1	4242	5190	4480, 4648, 4656	43, 57, 30	48.6796, 59.09894, 32.86861	18.38553, 17.94526, 14.62763	44.84555,	3	NA	110496
GL173807.1	18614	19084	18882, 18700, 18959	10, 6, 5	8.44704, 3.85021, 4.91762	5.78037, 3.34853, 4.03768	5.7869, 1.8	3	NA	96602
GL173807.1	42673	44569	43373, 43595, 43594	19, 18, 12	13.92205, 10.1344, 8.43382	7.0842, 5.2062, 5.40213	10.93707,	3	NA	71117
GL173807.1	45010	48657	45805, 47707, 45797, 46450, 48298	9, 26, 13, 11, 8	4.17668, 15.83587, 8.96696, 6.98215, 4.89868	3.23648, 6.4761, 5.53001, 4.68549, 3.8218	2.01446, 1	3	NA	67029
GL173807.1	45010	48657	47780, 47707, 45797, 46450, 48298	14, 26, 13, 11, 8	8.25658, 15.83587, 8.96696, 6.98215, 4.89868	4.90937, 6.4761, 5.53001, 4.68549, 3.8218	5.62529, 1	3	NA	67029
GL173807.1	141219	141777	141429, 141634, 141594	12, 11, 10	13.8483, 10.3713, 12.90749	8.63436, 6.79219, 8.21675	10.86694,	3	NA	18338
GL173809.1	125706	126024	125789, 125793, 125859	15, 16, 8	18.01767, 17.16856, 9.8421	10.49459, 9.82359, 6.68602	14.85992,	3	NA	47021
GL173811.1	76908	77649	77199, 77203, 77199	17, 30, 15	15.86203, 31.58953, 14.76444	8.78396, 13.86841, 8.62913	12.79136,	3	hs3st1	8145
GL173811.1	82856	83513	83336, 83184, 83182	29, 51, 17	28.23632, 52.6256, 16.65618	12.27101, 17.07926, 9.26618	24.77139,	3	hs3st1	2281
GL173812.1	123168	126563	123992, 123721, 123329, 124059	9, 15, 7, 12	6.82879, 10.7005, 5.82684, 11.60888	4.92128, 6.15, 4.53047, 7.36202	4.31954, 8	3	rgs18	59153
GL173812.1	123168	126563	125434, 123721, 123329, 124059	7, 15, 7, 12	4.94394, 10.7005, 5.82684, 11.60888	3.97064, 6.15, 4.53047, 7.36202	2.62735, 8	3	rgs18	59153
GL173812.1	123168	126563	126156, 123721, 123329, 124059	9, 15, 7, 12	6.96255, 10.7005, 5.82684, 11.60888	5.00603, 6.15, 4.53047, 7.36202	4.44307, 8	3	rgs18	59153
GL173812.1	131486	132369	131662, 131928, 131747	13, 13, 10	15.29169, 13.14514, 13.18184	9.29854, 8.09002, 8.33771	12.24396,	3	rgs18	67471
GL173812.1	140018	141159	140652, 140701, 140651	19, 21, 9	23.53998, 24.35402, 11.04428	12.7584, 12.71289, 7.30896	20.2, 21.17	3	rgs18	76003
GL173815.1	14484	15512	14689, 14598, 15321, 14598	12, 18, 9, 12	12.25023, 19.97713, 7.97047, 14.86478	7.74089, 10.97931, 5.63299, 9.15668	9.35789, 1	3	kcnt1	39444
GL173815.1	21268	22040	21722, 21451, 21440	10, 8, 9	11.06011, 7.07159, 11.64359	7.306, 5.20073, 7.57973	8.22712, 4	3	kcnt1	32916
GL173815.1	139152	140003	139330, 139880, 139395	8, 13, 5	8.42257, 13.14514, 5.9506	5.97763, 8.09002, 4.54784	5.76336, 1	3	kcnt1	24754
GL173816.1	24	3004	546, 622, 712	106, 155, 89	90.88738, 138.37758, 82.74879	15.13107, 17.3177, 16.88608	86.58096,	3	5S_rRNA	0
GL173816.1	7038	9105	8548, 8333, 7769	42, 61, 34	39.39846, 59.49788, 33.63124	13.65088, 16.56333, 13.51064	35.70598,	3	5S_rRNA	0
GL173816.1	36204	36653	36322, 36346, 36365	13, 18, 13	8.75972, 11.66417, 11.37851	5.40335, 6.03777, 7.02073	6.08202, 8	3	alox15b	0
GL173816.1	125949	126418	126056, 126081, 126062	19, 24, 26	19.8867, 24.86921, 35.38432	10.65421, 12.05732, 17.47674	16.66031,	3	aloxe3	0
GL173818.1	67757	69301	68924, 68609, 68610	27, 38, 38	25.27632, 39.99112, 46.4678	11.25499, 15.55078, 19.34346	21.8885, 3	3	5S_rRNA	0
GL173818.1	73775	76386	75195, 75203, 75217	55, 86, 38	47.32562, 77.44036, 32.88907	13.06833, 15.82341, 11.6709	43.51138,	3	5S_rRNA	0
GL173818.1	125406	125936	125781, 125787, 125787	27, 23, 18	34.32016, 26.6587, 22.76598	16.58774, 13.45462, 12.53748	30.72034,	3	5S_rRNA	49067
GL173819.1	49062	49761	49332, 49067, 49114	10, 21, 8	8.12348, 19.60696, 7.42597	5.57869, 9.87975, 5.41279	5.50152, 1	3	NA	NA
GL173820.1	306	856	618, 615, 661	10, 8, 8	11.06011, 7.07159, 10.1465	7.306, 5.20073, 6.82176	8.22712, 4	3	NA	2388
GL173820.1	32377	33235	32625, 32726, 32762	8, 10, 5	8.42257, 9.39128, 5.9506	5.97763, 6.35644, 4.54784	5.76336, 6	3	NA	5577
GL173820.1	69989	70664	70466, 70480, 70089	10, 14, 6	10.01299, 11.36754, 5.8719	6.7221, 6.78813, 4.59674	7.24763, 8	3	NA	12525
GL173821.1	82611	83102	82767, 82733, 82758	8, 13, 7	7.19971, 12.55412, 7.96278	5.27799, 7.73766, 5.72394	4.63758, 9	3	vwa5b1	0
GL173821.1	90527	91683	91178, 91611, 91093	11, 12, 5	10.1122, 10.08092, 4.91762	6.63573, 6.42828, 4.03768	7.34229, 7	3	vwa5b1	0
GL173822.1	16102	16614	16576, 16284, 16263	6, 11, 5	5.96715, 10.31711, 5.85847	4.64927, 6.75959, 4.50665	3.52158, 7	3	kiaa0930	0
GL173822.1	36947	37860	36988, 36992, 37732	7, 10, 9	4.64678, 7.45371, 9.58224	3.77717, 5.1569, 6.56676	2.42196, 5	3	kiaa0930	3538
GL173822.1	36980	37881	37700, 37709, 37732	10, 10, 9	7.7171, 7.72315, 9.58224	5.32329, 5.32711, 6.56676	5.12244, 5	3	kiaa0930	3571
GL173822.1	71040	71725	71426, 71446, 71424	20, 31, 14	22.36548, 32.82022, 15.35015	11.8377, 14.20748, 9.184	19.06081,	3	smc1b	0
GL173822.1	83580	84135	83620, 83966, 83980	11, 8, 8	9.52723, 4.96414, 7.63508	6.27749, 3.86431, 5.53536	6.80251, 2	3	smc1b	0
GL173822.1	95571	96322	96074, 96208, 95964	10, 7, 5	11.06011, 5.97725, 5.9506	7.306, 4.62287, 4.54784	8.22712, 3	3	ribc2	0
GL173822.1	100131	100763	100393, 100378, 100266	4, 6, 4	3.60479, 4.93312, 4.30163	3.24297, 4.04501, 3.61556	1.6222, 2.7	3	ribc2	0
GL173822.1	109637	110151	109641, 109650, 109649	18, 14, 14	20.25794, 12.43922, 16.37889	11.14243, 7.44862, 9.7558	17.01944,	3	ribc2	0
GL173822.1	128311	128853	128693, 128813, 128816	10, 12, 8	11.06011, 11.86108, 10.1465	7.306, 7.51216, 6.82176	8.22712, 9	3	ribc2	17924
GL173823.1	14638	14987	14863, 14938, 14875	7, 8, 6	7.1696, 7.07159, 7.29403	5.31345, 5.20073, 5.30581	4.61046, 4	3	NA	30349
GL173823.1	29690	30193	30152, 29797, 30028	6, 15, 5	5.63898, 15.33351, 5.9506	4.46544, 8.96975, 4.54784	3.25772, 1	3	NA	15143
GL173823.1	43772	44759	43929, 43994, 44184	16, 13, 8	14.79948, 12.61502, 8.4586	8.40174, 7.77427, 5.99709	11.77649,	3	NA	577
GL173823.1	65704	66044	66020, 66006, 66042	71, 57, 68	85.50179, 64.13629, 87.70657	25.3487, 20.60678, 28.1559	81.2496, 6	3	NA	0
GL173824.1	79157	79590	79281, 79285, 79476	7, 7, 3	7.1696, 5.97725, 3.47371	5.31345, 4.62287, 3.03189	4.61046, 3	3	NA	NA
GL173824.1	87154	87915	87225, 87490, 87287	8, 8, 3	8.42257, 7.07159, 3.47371	5.97763, 5.20073, 3.03189	5.76336, 4	3	NA	NA
GL173827.1	8719	9339	9328, 9313, 9293	46, 78, 30	54.24099, 92.87459, 38.34481	20.22038, 25.73134, 17.85338	50.32833,	3	NA	NA
GL173827.1	106197	106428	106292, 106291, 106375	10, 14, 11	9.38842, 10.39738, 9.40347	6.35473, 6.19139, 6.20096	6.67167, 7	3	NA	NA

GL173827.1	130470	130958	130537, 130472, 130478	9, 16, 9	9.72082, 17.14793, 11.64359	6.64182, 9.81151, 7.57973	6.97155, 1	3	NA	NA
GL173828.1	16317	16961	16493, 16746, 16330	5, 7, 4	4.82177, 5.97725, 4.67325	3.98509, 4.62287, 3.78987	2.51418, 3	3	nos3	5030
GL173828.1	29674	30202	30116, 30026, 30094	8, 12, 5	8.10827, 10.35, 5.54867	5.80497, 6.59497, 4.3619	5.48761, 7	3	nos3	0
GL173828.1	54267	54694	54571, 54425, 54438	31, 30, 16	34.86773, 30.4801, 17.62687	15.42176, 13.21372, 10.0901	31.257, 27	3	atg9b	2034
GL173828.1	59205	59997	59647, 59788, 59392	6, 7, 8	5.24248, 5.96795, 7.46678	4.2332, 4.61718, 5.43686	2.89962, 3	3	atg9b	6972
GL173828.1	60411	60891	60550, 60575, 60719	15, 15, 6	17.90819, 15.6285, 6.6567	10.43573, 9.14444, 5.00845	14.75478,	3	atg9b	8178
GL173828.1	80610	81152	80986, 80993, 80985	44, 76, 20	50.1891, 81.71398, 23.16452	18.80338, 21.0988, 12.30292	46.33454,	3	cdk5	0
GL173828.1	100786	101409	100908, 101023, 101134	8, 5, 6	6.34412, 3.41392, 6.25896	4.75087, 3.12487, 4.80589	3.871, 1.5	3	NA	1875
GL173828.1	102801	103666	103429, 103042, 103076	10, 11, 7	9.89241, 9.7598, 7.90186	6.65218, 6.42066, 5.69394	7.13431, 7	3	NA	3890
GL173828.1	113752	115019	114148, 114438, 114238	17, 14, 6	15.28145, 11.49628, 5.46454	8.43017, 6.86757, 4.36458	12.23845,	3	NA	0
GL173830.1	55636	56127	55738, 55693, 55665	16, 30, 11	16.23128, 30.23761, 12.78973	9.26839, 13.07132, 8.15619	13.14614,	3	NA	4356
GL173830.1	76514	76861	76682, 76766, 76790	7, 10, 7	6.46447, 8.44678, 8.646	4.91463, 5.78021, 6.04229	3.98312, 5	3	NA	5279
GL173831.1	29725	30463	30298, 30331, 29882	11, 11, 5	12.43693, 10.26356, 5.91431	7.97018, 6.72731, 4.53172	9.52692, 7	3	ddost	7661
GL173834.1	5080	5472	5235, 5305, 5225	4, 11, 8	3.74195, 9.80774, 9.76268	3.32091, 6.45006, 6.64964	1.6222, 7.2	3	NA	0
GL173834.1	51827	52319	51926, 51982, 51983	23, 28, 10	24.76553, 27.46787, 11.5283	12.33305, 12.18424, 7.55211	21.39099,	3	NA	19756
GL173835.1	133126	133909	133323, 133226, 133697	9, 13, 6	7.39636, 12.61502, 6.76586	5.27874, 7.77427, 5.06179	4.82212, 9	3	NA	NA
GL173835.1	133126	133980	133848, 133226, 133697	13, 13, 6	12.09198, 12.61502, 6.76586	7.45795, 7.77427, 5.06179	9.20698, 9	3	NA	NA
GL173836.1	75	758	349, 321, 426	9, 10, 4	9.72082, 9.39128, 4.67325	6.64182, 6.35644, 3.78987	6.97155, 6	3	NA	NA
GL173837.1	19944	20935	20372, 20137, 20521	9, 6, 3	9.72082, 4.93312, 3.47371	6.64182, 4.04501, 3.03189	6.97155, 2	3	ckap4	60818
GL173837.1	27921	29410	28210, 28009, 29215, 29016	11, 7, 5, 3	12.43693, 5.97725, 3.94561, 3.47371	7.97018, 4.62287, 3.46715, 3.03189	9.52692, 3	3	ckap4	52343
GL173837.1	43905	44953	44445, 44266, 44534	7, 13, 6	7.1696, 13.14514, 7.29403	5.31345, 8.09002, 5.30581	4.61046, 1	3	ckap4	36800
GL173838.1	125	829	451, 335, 430	15, 13, 9	13.00173, 9.02151, 9.30116	7.55434, 5.56343, 6.41174	10.06533,	3	NA	NA
GL173838.1	1856	2287	2130, 2127, 2155	15, 15, 8	11.6639, 9.66304, 7.42597	6.73404, 5.53388, 5.41279	8.80068, 7	3	NA	NA
GL173838.1	7004	7506	7176, 7287, 7364	19, 30, 19	12.6872, 20.67039, 16.30745	6.38411, 7.88677, 8.49288	9.76546, 1	3	NA	NA
GL173838.1	8428	8899	8646, 8612, 8657	18, 25, 9	12.67071, 16.35591, 6.0761	6.6092, 6.94591, 4.44085	9.75044, 1	3	NA	NA
GL173838.1	15320	15801	15596, 15416, 15619	7, 7, 7	6.09019, 5.05277, 6.63036	4.69155, 4.04098, 5.0111	3.63504, 2	3	NA	NA
GL173838.1	19019	19895	19426, 19365, 19446	14, 23, 13	13.20712, 22.03663, 13.97446	7.91811, 10.69607, 8.57174	10.26077,	3	NA	NA
GL173839.1	2527	4498	2728, 3569, 2705	9, 15, 7	6.63709, 10.31209, 6.21898	4.79941, 5.91747, 4.76912	4.14229, 7	3	phf2	62680
GL173839.1	2642	4498	3563, 3569, 2705	7, 15, 7	4.74226, 10.31209, 6.21898	3.83953, 5.91747, 4.76912	2.50288, 7	3	phf2	62680
GL173839.1	2642	5014	4482, 3569, 2705	10, 15, 7	7.61309, 10.31209, 6.21898	5.25765, 5.91747, 4.76912	5.02606, 7	3	phf2	62164
GL173839.1	5630	6158	5705, 5817, 6049	10, 17, 7	7.8239, 11.44415, 6.01639	5.3906, 6.13523, 4.64674	5.22304, 8	3	phf2	61020
GL173839.1	43082	43829	43502, 43313, 43313	9, 18, 8	8.50607, 17.79566, 9.53432	5.95453, 9.68065, 6.54285	5.84194, 1	3	phf2	23349
GL173839.1	87074	87838	87764, 87574, 87500	11, 28, 16	9.00946, 23.66073, 15.4842	5.95596, 10.00069, 8.81824	6.31598, 2	3	phf2	0
GL173839.1	89315	89830	89557, 89574, 89576	10, 19, 7	7.8239, 13.31797, 5.32289	5.3906, 6.73865, 4.21415	5.22304, 1	3	phf2	0
GL173839.1	107864	108380	108086, 108039, 108018	28, 41, 30	30.48685, 43.80519, 37.45558	13.97597, 16.4673, 17.33866	26.96846,	3	phf2	5937
GL173839.1	113182	113938	113451, 113461, 113646	24, 36, 25	27.83747, 40.81123, 33.39399	13.82444, 16.87418, 16.67053	24.38123,	3	phf2	11255
GL173841.1	61065	61730	61553, 61254, 61252	4, 8, 5	3.74195, 5.93025, 5.19074	3.32091, 4.48816, 4.18277	1.6222, 3.6	3	ppfibp1	0
GL173841.1	76714	77776	77159, 77243, 77332	14, 26, 11	13.09001, 22.17581, 11.4314	7.84687, 9.80046, 7.41405	10.14895,	3	ppfibp1	4308
GL173841.1	94832	95357	95161, 95166, 95154	24, 39, 18	22.42871, 35.28939, 18.14836	10.59867, 12.57491, 9.89347	19.12211,	3	ppfibp1	22426
GL173841.1	100368	100610	100457, 100527, 100552	18, 14, 18	16.62541, 12.04301, 19.67904	8.97077, 7.20479, 10.80493	13.52454,	3	ppfibp1	27962
GL173841.1	113198	114095	113657, 113654, 113416	11, 17, 11	10.27095, 17.11659, 11.99553	6.73177, 9.54398, 7.73059	7.4917, 14	3	ppfibp1	40792
GL173841.1	125803	126847	126569, 126059, 126372	17, 33, 18	13.96763, 30.11187, 18.33972	7.63104, 11.89975, 10.0086	10.98042,	3	ppfibp1	53397
GL173842.1	30365	31121	30559, 30882, 30707	11, 17, 9	7.83732, 11.60083, 8.05901	5.2206, 6.22562, 5.6868	5.23542, 8	3	NA	NA
GL173842.1	46640	47401	47055, 46997, 47087	27, 25, 18	27.24252, 23.34787, 18.47005	12.41362, 10.80775, 10.08686	23.80018,	3	NA	NA
GL173842.1	66536	67199	67131, 66924, 66629	14, 23, 14	7.17348, 11.8657, 10.31225	4.2931, 5.18446, 6.13929	4.61362, 9	3	NA	NA
GL173842.1	67620	68767	68026, 68329, 68553	32, 41, 14	24.89135, 28.55865, 10.22223	9.44483, 8.8133, 6.08428	21.51276,	3	NA	NA
GL173842.1	81774	83765	82214, 82515, 82208	43, 69, 38	33.04362, 52.95078, 30.87481	10.24741, 11.55029, 10.66074	29.46975,	3	NA	NA
GL173842.1	102171	103616	102897, 102604, 102583	26, 32, 21	23.81005, 29.88364, 20.68121	10.74163, 12.12836, 10.52721	20.46254,	3	NA	NA
GL173842.1	103843	104341	104011, 104013, 104244	16, 15, 16	11.73743, 9.66304, 13.32499	6.53951, 5.53388, 7.50039	8.87017, 7	3	NA	NA
GL173842.1	114597	115876	115581, 115477, 115304	13, 20, 14	9.22191, 14.38423, 13.12965	5.68647, 7.09595, 7.871	6.51656, 1	3	NA	NA
GL173843.1	99651	100443	100218, 100055, 100048	10, 12, 6	8.32267, 9.13354, 4.75057	5.70305, 5.83804, 3.93137	5.6878, 6.5	3	gpa33	5011
GL173843.1	104674	105718	104705, 104677, 104678	10, 17, 11	8.70772, 14.51225, 10.66921	5.94148, 7.96159, 6.9701	6.03286, 1	3	gpa33	10034
GL173843.1	139266	139879	139342, 139270, 139332	5, 27, 8	4.5799, 28.70654, 8.4044	3.84863, 13.28584, 5.9678	2.3629, 25	3	gpa33	44626
GL173844.1	7537	7948	7701, 7762, 7816	7, 6, 5	5.82861, 4.30294, 5.3628	4.53157, 3.64568, 4.27046	3.42929, 2	3	NA	18475
GL173844.1	71627	72305	71919, 71961, 71752	8, 9, 4	6.70593, 6.26311, 3.88239	4.9768, 4.56059, 3.39841	4.20487, 3	3	NA	3062
GL173845.1	871	1505	922, 934, 901	31, 45, 26	32.69429, 49.0113, 32.88676	14.1331, 17.68126, 16.09579	29.12759,	3	NA	4007
GL173845.1	37437	38038	37578, 37855, 37670	14, 22, 14	12.8072, 20.88093, 15.06595	7.67424, 10.32883, 9.02131	9.88012, 1	3	ssr2	14250
GL173845.1	77814	78735	78391, 78408, 78284	12, 15, 6	10.83541, 12.62899, 5.43047	6.89366, 7.3254, 4.34463	8.02495, 9	3	ssr2	54627
GL173845.1	91549	91926	91859, 91868, 91839	11, 17, 7	10.72304, 16.43386, 6.59055	7.00201, 9.1315, 4.98809	7.91902, 1	3	ssr2	68362
GL173845.1	95220	95712	95507, 95347, 95282	6, 8, 9	5.31708, 7.07159, 9.70054	4.2777, 5.20073, 6.63089	2.96727, 4	3	ssr2	72033
GL173845.1	126145	126548	126376, 126405, 126199	11, 14, 14	11.42621, 13.50676, 15.80333	7.41109, 8.09967, 9.43935	8.57514, 1	3	ssr2	102958
GL173846.1	91	702	347, 347, 531	10, 14, 26	11.06011, 14.09002, 32.51974	7.306, 8.44949, 15.88658	8.22712, 1	3	chpf2	2708
GL173846.1	2838	3310	2877, 2874, 3035	8, 11, 10	8.42257, 9.7598, 9.53891	5.97763, 6.42066, 6.44439	5.76336, 7	3	chpf2	100
GL173846.1	11184	11958	11201, 11801, 11507	4, 9, 5	3.74195, 8.21099, 5.9506	3.32091, 5.77858, 4.54784	1.6222, 5.7	3	chpf2	0
GL173846.1	19238	19655	19396, 19455, 19332	7, 19, 5	7.1696, 20.55804, 5.50079	5.31345, 11.0538, 4.33868	4.61046, 1	3	chpf2	5087
GL173846.1	97521	97743	97691, 97686, 97719	8, 22, 18	8.42257, 22.3038, 24.85881	5.97763, 11.18464, 13.59437	5.76336, 1	3	smarcd3	11834
GL173846.1	104676	104970	104766, 104767, 104759	20, 37, 21	19.49401, 38.04945, 23.5695	10.12053, 14.84938, 12.25439	16.28237,	3	smarcd3	18989
GL173847.1	99	1656	807, 1236, 468	55, 63, 48	55.35419, 59.93617, 51.76319	16.85743, 16.03902, 17.89742	51.42649,	3	lsm10	47310
GL173847.1	31905	32442	32071, 32158, 32169	7, 7, 6	6.69449, 5.8586, 7.29403	5.04797, 4.55007, 5.30581	4.1953, 3.5	3	lsm10	16524
GL173848.1	39991	40332	40146, 40151, 40141	22, 26, 17	22.44777, 25.92257, 18.80835	11.27125, 11.98756, 10.54536	19.1406, 2	3	NA	16447
GL173848.1	71240	71602	71332, 71339, 71533	12, 15, 15	12.18267, 13.4765, 16.99143	7.70145, 7.84563, 9.93059	9.29304, 1	3	NA	437
GL173848.1	113020	113629	113540, 113488, 113544	21, 27, 20	21.7275, 27.35215, 22.80441	11.15737, 12.47874, 12.09422	18.44198,	3	NA	444
GL173848.1	121849	122803	121996, 121903, 121953	14, 17, 10	14.83249, 16.56523, 10.59656	8.88629, 9.21112, 7.05292	11.80835,	3	NA	9273
GL173849.1	4875	5473	4915, 4880, 4901	80, 102, 54	94.48528, 111.71344, 66.68111	25.57481, 23.82869, 23.5024	90.14348,	3	XB-GENE-5809894	22904
GL173849.1	23917	24301	23932, 23922, 23953	27, 61, 32	25.61495, 66.25966, 37.23787	11.45294, 19.91787, 16.42754	22.21656,	3	XB-GENE-5809894	4076
GL173849.1	99132	99745	99555, 99604, 99702	18, 29, 10	16.62541, 28.88285, 11.17239	8.97077, 12.64966, 7.36591	13.52454,	3	srrm2	0
GL173850.1	60271	60913	60670, 60776, 60911	9, 6, 5	8.25643, 4.93312, 5.14967	5.80588, 4.04501, 4.16141	5.62516, 2	3	large1	0
GL173851.1	6255	6679	6537, 6443, 6569	5, 7, 6	4.82177, 5.75319, 6.99807	3.98509, 4.48487, 5.17195	2.51418, 3	3	NA	26308
GL173851.1	11268	11932	11317, 11365, 11810	6, 11, 7	5.96715, 10.60895, 8.69489	4.64927, 6.9343, 6.06379	3.52158, 7	3	NA	21055
GL173851.1	36995	38348	37615, 37708, 37276	15, 15, 10	13.36183, 11.72411, 9.68182	7.77533, 6.77081, 6.52889	10.40776,	3	NA	1613
GL173851.1	62494	62796	62669, 62613, 62584	5, 8, 5	4.82177, 7.07159, 5.9506	3.98509, 5.20073, 4.54784	2.51418, 4	3	NA	1271
GL173851.1	69445	70275	69972, 69971, 69786	9, 7, 19	9.36986, 5.97725, 24.60107	6.44997, 4.62287, 13.32686	6.65425, 3	3	NA	2902
GL173851.1	175516	175825	175690, 175637, 175626	6, 9, 6	5.96715, 8.21099, 7.29403	4.64927, 5.77858, 5.30581	3.52158, 5	3	NA	76901
GL173852.1	28175	29445	29096, 29121, 28772	16, 17, 9	14.85827, 15.87131, 11.04428	8.4376, 8.78961, 7.30896	11.83222,	3	NA	24241
GL173852.1	59141	60035	59498, 59697, 59674	11, 13, 5	12.43693, 13.14514, 5.9506	7.97018, 8.09002, 4.54784	9.52692, 1	3	NA	4440
GL173852.1	87061	88129	87923, 87915, 87317	14, 18, 15	13.57329, 15.48582, 16.01939	8.13982, 8.27912, 9.37366	10.60942,	3	tpm2	28785
GL173853.1	40857	41433	41188, 41287, 40954	11, 17, 8	7.74505, 13.09378, 6.02227	5.16265, 7.10488, 4.54691	5.14926, 1	3	cep112	0
GL173853.1	44962	45272	45047, 45132, 45077	9, 13, 8	5.83497, 8.30814, 6.27048	4.28635, 5.12931, 4.70442	3.43552, 5	3	cep112	0
GL173853.1	72440	73544	72514, 72798, 72510	13, 30, 17	9.91363, 26.01696, 16.20643	6.11357, 10.65908, 8.99344	7.15376, 2	3	cep112	0
GL173853.1	98642	99036	99006, 98981, 98795	11, 10, 5	11.15818, 8.9331, 5.9506	7.25692, 6.07959, 4.54784	8.32008, 6	3	cep112	3491
GL173853.1	108822	109573	109056, 109063, 109329	7, 25, 9	7.1696, 27.15225, 9.76105	5.31345, 13.07691, 6.66343	4.61046, 2	3	AXIN2	0
GL173853.1	135100	135701	135612, 135676, 135619	15, 21, 12	14.21298, 18.11284, 12.03712	8.29535, 8.98591, 7.61609	11.21566,	3	AXIN2	7400
GL173853.1	136128	136713	136158, 136193, 136174	23, 39, 25	24.94963, 42.09095, 30.81762	12.44303, 16.33801, 15.23219	21.56948,	3	AXIN2	8428
GL173855.1	32736	33665	33266, 33068, 33058	15, 34, 8	15.56593, 32.56081, 9.05167	9.10751, 12.90382, 6.30653	12.50679,	3	NA	NA
GL173855.1	68277	69091	68875, 68895, 68879	30, 99, 37	24.66485, 92.90774, 39.11235	9.92261, 17.58828, 15.45969	21.29276,	3	NA	NA
GL173855.1	69830	70563	70212, 70306, 70178	30, 58, 17	25.13207, 42.52359, 13.83231	10.17468, 10.36156, 7.54917	21.7476, 3	3	NA	NA
GL173855.1	113498	113997	113944, 113995, 113995	30, 66, 41	32.10847, 75.58281, 52.90765	14.17604, 22.50986, 21.78625	28.55462,	3	NA	NA
GL173857.1	5653	6512	6030, 6214, 6194	11, 10, 9	12.33515, 8.79469, 10.21256	7.91559, 5.99491, 6.90044	9.43699, 6	3	WNK2	10333
GL173857.1	77780	78875	78461, 78460, 78461	22, 28, 15	24.97667, 30.99659, 19.17716	12.77925, 14.27985, 11.09709	21.59612,	3	fam120a	0
GL173857.1	97369	97886	97383, 97383, 97374	13, 18, 8	14.18705, 17.65791, 9.68495	8.69244, 9.59733, 6.61366	11.19086,	3	fam120a	0
GL173858.1	29423	30243	29603, 29827, 29614	10, 15, 11	5.94607, 8.65012, 9.7601	4.20363, 4.95042, 6.42085	3.52158, 6	3	NA	44723
GL173858.1	31414	32427	31905, 31513, 32172	9, 23, 7	6.03824, 18.4746, 5.73648	4.41659, 8.60508, 4.47449	3.58764, 1	3	NA	42539
GL173858.1	69264	70359	69583, 69662, 69569	37, 46, 26	31.6832, 34.77694, 24.56787	11.38005, 10.20928, 11.18558	28.13808,	3	NA	4607
GL173858.1	100465	101160	100961, 100756, 100843	13, 12, 10	9.3861, 5.45901, 7.12656	5.78755, 3.62131, 4.94968	6.66988, 3	3	NA	23690
GL173858.1	119380	120051	119656, 119665, 119421	10, 10, 6	7.01158, 5.45339, 5.43047	4.87678, 3.89639, 4.34463	4.48836, 3	3	NA	42605
GL173859.1	105	703	309, 343, 411	19, 22, 15	22.35011, 23.27801, 17.94632	12.09487, 11.76976, 10.45626	19.0463, 2	3	NA	50939
GL173859.1	5996	6940	6398, 6419, 6470	18, 16, 9	17.51609, 13.08342, 9.52439	9.51146, 7.35304, 6.53516	14.37616,	3	NA	44702
GL173859.1	12294	12610	12461, 12471, 12435	12, 18, 5	8.77375, 11.35007, 3.64364	5.61364, 5.86316, 3.27494	6.09412, 8	3	NA	39032
GL173859.1	38730	39198	38842, 38831, 38818	10, 12, 6	9.03421, 9.90946, 7.29403	6.14116, 6.32175, 5.30581	6.33915, 7	3	NA	12444
GL173859.1	39147	39840	39392, 39486, 38818, 39573	17, 16, 6, 3	17.96244, 14.57865, 7.29403, 3.47371	10.04917, 8.26691, 5.30581, 3.03189	14.80626,	3	NA	11802
GL173859.1	112076	112909	112357, 112356, 112237	6, 8, 6	5.24248, 6.66413, 6.168	4.2332, 4.9509, 4.75778	2.89962, 4	3	adra1b	0
GL173860.1	4838	5104	5018, 5021, 5013	10, 10, 10	10.81731, 8.88645, 10.90706	7.17456, 6.0511, 7.22344	8.00764, 6	3	NA	NA
GL173860.1	25967	26419	26200, 26155, 26221	12, 9, 8	12.60209, 8.21099, 9.05167	7.9443, 5.77858, 6.30653	9.68421, 5	3	NA	NA
GL173860.1	110323	110637	110554, 110469, 110443	10, 10, 5	11.06011, 8.74956, 5.85847	7.306, 5.96721, 4.50665	8.22712, 6	3	NA	NA
GL173860.1	116870	117181	116897, 116885, 116896	39, 51, 32	48.08644, 60.2889, 42.80507	19.85319, 21.31358, 19.67264	44.26125,	3	NA	NA
GL173861.1	48024	49343	48512, 48251, 48654	5, 9, 5	4.20732, 7.21043, 4.91762	3.62846, 5.1623, 4.03768	2.04024, 4	3	XB-GENE-5944458	10242
GL173861.1	61931	62477	62270, 62298, 62221	4, 12, 6	3.74195, 11.86108, 7.29403	3.32091, 7.51216, 5.30581	1.6222, 9.1	3	XB-GENE-5944458	24149
GL173861.1	103338	103713	103674, 103656, 103673	30, 26, 14	35.06034, 28.88589, 15.96227	15.93019, 13.75671, 9.52765	31.44637,	3	XB-GENE-5944458	65556
GL173861.1	113725	114270	114222, 113821, 114150	12, 8, 11	10.58505, 4.85726, 11.25734	6.73997, 3.7949, 7.31423	7.78888, 2	3	XB-GENE-5944458	75943
GL173862.1	45388	45772	45653, 45545, 45556	8, 27, 11	7.77807, 30.45463, 13.28509	5.618, 14.32841, 8.40871	5.17956, 2	3	NA	5689
GL173862.1	79040	80082	79447, 79569, 79592	23, 52, 24	21.80868, 52.61212, 26.55881	10.55972, 16.66176, 13.06784	18.52047,	3	NA	25545
GL173863.1	15897	16637	16584, 16158, 16300	4, 9, 5	3.74195, 8.21099, 5.9506	3.32091, 5.77858, 4.54784	1.6222, 5.7	3	NA	18086
GL173863.1	65541	65996	65897, 65612, 65670	6, 11, 5	4.65447, 7.97598, 4.33804	3.87084, 5.30773, 3.70703	2.42652, 5	3	NA	27846
GL173863.1	106215	106650	106411, 106390, 106287	19, 19, 13	10.09815, 6.75192, 7.01238	5.00905, 3.45552, 4.3626	7.32971, 4	3	NA	10033
GL173863.1	107110	108099	107810, 107752, 107815	50, 88, 32	43.91825, 78.53127, 26.68986	13.11198, 15.65357, 10.37852	40.15508,	3	NA	10928
GL173864.1	48330	48710	48356, 48352, 48358	28, 43, 21	25.83022, 43.55196, 23.07125	11.22993, 15.49206, 11.96005	22.42551,	3	rab22a	0
GL173864.1	69919	70803	70092, 70420, 70331	5, 15, 5	4.69353, 14.84826, 5.69847	3.9134, 8.67957, 4.4331	2.45909, 1	3	rab22a	13629
GL173864.1	87642	87973	87728, 87645, 87701	22, 37, 8	25.96118, 37.70701, 9.31951	13.35351, 14.65424, 6.43944	22.55288,	3	ppp4r1l	0
GL173864.1	104809	105061	104994, 105012, 104984	27, 30, 10	30.55576, 30.31791, 11.91537	14.38856, 13.11844, 7.74796	27.03577,	3	znf41	60
GL173864.1	110136	111077	110625, 110641, 110670	6, 12, 6	5.63898, 10.49031, 5.75442	4.46544, 6.6816, 4.53101	3.25772, 7	3	znf41	1875
GL173865.1	53	791	331, 205, 495	11, 22, 10	12.25244, 25.85271, 11.60305	7.87094, 13.29074, 7.59048	9.35958, 2	3	abcc10	28984
GL173865.1	30261	31399	30886, 30882, 30919	30, 24, 7	31.37869, 23.06143, 7.50542	13.74365, 10.97506, 5.49244	27.83999,	3	abcc10	0
GL173865.1	39471	40003	39729, 39737, 39665	7, 9, 16	7.1696, 8.21099, 16.972	5.31345, 5.77858, 9.70822	4.61046, 5	3	abcc10	0
GL173865.1	83581	84233	83814, 83638, 83651	10, 11, 11	10.6018, 10.15827, 13.1143	7.05583, 6.66366, 8.32282	7.80484, 7	3	dlk2	20631
GL173865.1	118190	119021	118950, 118773, 118917	12, 14, 4	13.8483, 14.45897, 4.67325	8.63436, 8.66788, 3.78987	10.86694,	3	NA	0
GL173866.1	797	1177	1053, 1022, 1146	14, 12, 7	16.03678, 11.86108, 8.69489	9.5688, 7.51216, 6.06379	12.95916,	3	POU2F1	91892
GL173866.1	15260	15747	15329, 15294, 15368	6, 18, 5	5.68215, 17.79566, 5.9506	4.49007, 9.68065, 4.54784	3.29732, 1	3	POU2F1	77322
GL173866.1	29521	29921	29668, 29682, 29563	7, 9, 4	6.89193, 6.86591, 4.38152	5.15998, 4.94483, 3.65448	4.37772, 4	3	POU2F1	63148
GL173866.1	47671	48846	47979, 48576, 47797, 48180	10, 12, 5, 5	7.51175, 7.29941, 4.22765, 4.25472	5.19361, 4.70419, 3.64077, 3.65711	4.93097, 4	3	POU2F1	44223
GL173866.1	50510	51493	51069, 50824, 51116	18, 20, 8	15.56701, 15.18357, 8.04677	8.32827, 7.55252, 5.77057	12.50773,	3	POU2F1	41576
GL173866.1	65417	65644	65466, 65443, 65484	9, 15, 6	7.71701, 14.03607, 5.91225	5.47767, 8.18766, 4.61907	5.12244, 1	3	POU2F1	27425
GL173866.1	66113	66377	66300, 66209, 66264	12, 8, 7	11.67221, 6.08299, 7.55909	7.39985, 4.58557, 5.52035	8.80793, 3	3	POU2F1	26692
GL173866.1	74055	74767	74580, 74523, 74565	16, 24, 10	14.45741, 20.0145, 9.82971	8.19281, 9.18815, 6.61563	11.44906,	3	POU2F1	18302
GL173866.1	120961	121358	121274, 121276, 121260	17, 28, 8	18.64535, 32.60991, 10.1465	10.45058, 15.23874, 6.82176	15.46286,	3	POU2F1	0
GL173867.1	15726	16077	15824, 15858, 15910	7, 7, 6	7.1696, 5.97725, 6.99807	5.31345, 4.62287, 5.17195	4.61046, 3	3	ndufa4	3477
GL173867.1	25710	26269	25965, 25951, 25950	9, 10, 4	9.36986, 9.32635, 4.22496	6.44997, 6.31756, 3.57747	6.65425, 6	3	lsr	1282
GL173869.1	32748	33974	33098, 32901, 32819	6, 9, 7	5.72604, 8.21099, 8.21885	4.51498, 5.77858, 5.84717	3.33547, 5	3	add3	6247
GL173869.1	68260	70510	68684, 69335, 68668, 69473, 70302	13, 14, 11, 6, 7	11.11934, 11.36754, 10.10247, 4.80295, 5.85759	6.86076, 6.78813, 6.62982, 3.96416, 4.54944	8.28353, 8	3	NA	12159
GL173869.1	90742	91402	90889, 91055, 91181	13, 17, 4	12.94099, 14.12865, 3.21039	7.96907, 7.72861, 3.0071	10.00729,	3	NA	0
GL173869.1	96517	97532	97197, 97081, 96737	22, 28, 14	24.67026, 30.99659, 16.12551	12.59865, 14.27985, 9.61761	21.29787,	3	NA	0
GL173869.1	119954	120491	120120, 120008, 120244	5, 10, 5	4.81325, 8.70492, 5.40789	3.98038, 5.93975, 4.29296	2.51418, 6	3	NA	0
GL173869.1	131346	132369	132015, 132053, 132028	22, 31, 29	24.08614, 31.85677, 34.61941	12.25235, 13.63997, 16.05212	20.7301, 2	3	NA	772
GL173870.1	48906	49711	49378, 49354, 49331	8, 16, 10	6.20993, 14.35819, 9.82971	4.66614, 8.13215, 6.61563	3.74679, 1	3	ubl5	0
GL173871.1	30856	31523	31405, 31209, 31264	12, 23, 12	6.03944, 12.34072, 7.49967	3.95428, 5.40323, 4.82618	3.58881, 9	3	NA	NA
GL173871.1	35386	36180	35837, 35828, 35837	13, 20, 11	7.56859, 10.27059, 7.32071	4.68763, 4.92624, 4.89668	4.98492, 7	3	NA	NA
GL173871.1	41622	42241	41806, 41864, 41894	8, 13, 13	6.0497, 9.47155, 11.86258	4.56438, 5.84024, 7.31799	3.59874, 6	3	NA	NA
GL173871.1	55572	56184	55761, 55797, 55860	17, 14, 7	11.47144, 7.62225, 5.03066	6.15094, 4.54517, 4.02672	8.61801, 5	3	NA	NA
GL173871.1	73377	74159	73567, 73406, 73405	7, 11, 8	6.12952, 9.39347, 8.51364	4.71533, 6.19476, 6.02666	3.67231, 6	3	NA	NA
GL173871.1	121396	122251	122072, 122007, 121881	8, 7, 4	7.57423, 5.97725, 4.67325	5.4999, 4.62287, 3.78987	4.98865, 3	3	NA	NA
GL173872.1	45614	46008	45977, 45921, 45800	13, 10, 4	11.30985, 7.20159, 3.54079	6.9784, 4.99723, 3.20587	8.46461, 4	3	sult1b1	0
GL173872.1	110640	111010	110837, 110738, 110835	8, 18, 5	7.99469, 16.69883, 5.69847	5.74128, 9.01539, 4.4331	5.38155, 1	3	cln3	5545
GL173873.1	51	1011	847, 448, 164	6, 9, 5	5.16999, 8.21099, 5.9506	4.18963, 5.77858, 4.54784	2.83325, 5	3	dnhd1	40477
GL173873.1	5146	5490	5413, 5437, 5443	18, 22, 14	18.15064, 22.61717, 16.29325	9.89485, 11.37311, 9.7093	14.98708,	3	dnhd1	35998
GL173873.1	12618	14400	14022, 12630, 13747, 12674, 14205	13, 14, 18, 5, 15	10.54397, 10.36194, 14.94782, 4.33804, 17.07266	6.5044, 6.16969, 7.95431, 3.70703, 9.97618	7.7502, 7.7	3	dnhd1	27088
GL173873.1	76494	77157	77041, 76650, 76761	4, 11, 5	3.51298, 10.60895, 5.50079	3.1896, 6.9343, 4.33868	1.55088, 7	3	dnhd1	0
GL173873.1	116658	117098	116941, 116786, 116711	6, 12, 7	5.96715, 10.83413, 7.05993	4.64927, 6.89288, 5.25341	3.52158, 8	3	tpp1	0
GL173874.1	6017	6937	6089, 6297, 6423	9, 10, 12	9.72082, 8.4887, 15.36829	6.64182, 5.80621, 9.40047	6.97155, 5	3	gas2	2137
GL173874.1	28555	29250	28777, 28742, 28742	9, 22, 5	9.72082, 23.27801, 5.80393	6.64182, 11.76976, 4.48186	6.97155, 2	3	gas2	0
GL173874.1	38203	38864	38591, 38457, 38450	9, 11, 6	8.77314, 9.43766, 5.83216	6.111, 6.22212, 4.57461	6.09355, 6	3	gas2	0
GL173874.1	57084	57922	57439, 57492, 57489	4, 11, 6	3.31554, 8.48203, 5.99481	3.07164, 5.62571, 4.6644	1.39756, 5	3	gas2	0
GL173874.1	77645	78219	78165, 78159, 78189	16, 30, 12	16.30427, 31.06513, 13.15613	9.31205, 13.55836, 8.25729	13.21554,	3	gas2	1532
GL173874.1	101567	102078	102067, 102045, 102047	27, 28, 13	32.16072, 31.30207, 13.84064	15.33749, 14.46185, 8.49513	28.60555,	3	gas2	25454
GL173874.1	108554	109826	109362, 109389, 109380	10, 12, 9	11.06011, 10.98912, 10.87975	7.306, 6.98757, 7.23113	8.22712, 8	3	gas2	32441
GL173874.1	116911	118446	117988, 117160, 117806	13, 17, 8	12.74759, 12.97503, 8.04677	7.85373, 7.03392, 5.77057	9.82421, 1	3	gas2	40798
GL173874.1	130569	130812	130783, 130774, 130732	8, 21, 6	5.27754, 18.00814, 5.43047	4.06763, 8.9238, 4.34463	2.93292, 1	3	gas2	54456
GL173874.1	131144	131810	131630, 131662, 131348	13, 16, 8	10.41975, 12.61232, 7.76694	6.42736, 7.0666, 5.6116	7.63296, 9	3	gas2	55031
GL173875.1	56773	57399	57263, 57152, 57132	5, 7, 3	4.82177, 5.97725, 3.47371	3.98509, 4.62287, 3.03189	2.51418, 3	3	NA	6216
GL173876.1	1682	2419	2122, 2153, 1952	22, 20, 29	22.60711, 20.68875, 37.2397	11.36706, 10.84083, 17.5756	19.29541,	3	fbxo42	0
GL173876.1	12005	12432	12389, 12375, 12420	13, 13, 9	12.20544, 10.07856, 8.84115	7.52692, 6.21577, 6.1504	9.31472, 7	3	fbxo42	0
GL173876.1	69160	69453	69310, 69313, 69285	15, 12, 13	14.27412, 9.74388, 14.39632	8.3325, 6.2187, 8.81006	11.27364,	3	NA	296
GL173876.1	70346	70658	70453, 70405, 70474	13, 14, 6	11.50747, 12.49074, 5.67889	7.10015, 7.48027, 4.48822	8.65224, 9	3	NA	1482
GL173876.1	107646	108347	107812, 107855, 107989	5, 12, 9	4.5799, 11.84549, 10.56949	3.84863, 7.50292, 7.08032	2.3629, 9.1	3	NA	38782
GL173876.1	118369	118811	118566, 118565, 118490	37, 40, 20	37.55792, 38.52561, 20.9157	14.56952, 13.93422, 10.97698	33.89533,	3	NA	49505
GL173877.1	7798	8617	8273, 8532, 8109	7, 9, 4	6.79162, 8.21099, 4.67325	5.10336, 5.77858, 3.78987	4.28406, 5	3	NA	NA
GL173878.1	20469	21079	20959, 20946, 20900	17, 19, 14	19.70371, 20.64644, 17.61756	11.05791, 11.1061, 10.40352	16.48557,	3	klhdc9	0
GL173878.1	46346	47177	46631, 46475, 46588	24, 27, 10	23.75166, 25.86233, 8.88909	11.38751, 11.59804, 6.05271	20.40529,	3	mex3a	15328
GL173878.1	49023	50257	50100, 49934, 49654	7, 9, 16	4.02345, 4.89512, 15.71034	3.36702, 3.68681, 8.95507	1.88011, 2	3	mex3a	12248
GL173878.1	134716	135548	135174, 135136, 135259	21, 37, 22	16.71629, 30.78346, 20.36134	8.16574, 10.92233, 10.0174	13.61174,	3	adam15	0
GL173878.1	136819	137652	137237, 137302, 137208	25, 34, 26	21.52286, 26.73512, 25.53891	9.74373, 9.79617, 11.75956	18.24348,	3	adam15	0
GL173878.1	139576	140129	139896, 139856, 139679	11, 20, 15	6.47272, 11.73876, 11.62511	4.37034, 5.6617, 6.71037	3.99115, 9	3	adam15	0
GL173879.1	8568	9145	8870, 8706, 8884	13, 24, 10	11.21374, 20.21746, 9.40066	6.91908, 9.30469, 6.36205	8.37313, 1	3	NA	5400
GL173879.1	31597	31868	31827, 31651, 31787	14, 9, 12	16.7649, 8.21099, 15.36829	9.96273, 5.77858, 9.40047	13.6543, 5	3	NA	13941
GL173879.1	42923	44028	43500, 43482, 43536	17, 28, 10	17.19112, 27.92177, 10.97191	9.58879, 12.45165, 7.25854	14.0641, 2	3	NA	25267
GL173879.1	57443	58129	57635, 58003, 57528	8, 9, 5	7.77807, 6.37567, 5.91431	5.618, 4.63258, 4.53172	5.17956, 4	3	NA	27988
GL173879.1	66583	67098	66821, 66796, 66796	19, 21, 16	21.31193, 23.12148, 19.25686	11.49694, 11.98982, 11.00387	18.04063,	3	NA	19019
GL173879.1	88589	89864	89655, 89663, 89674	58, 83, 40	57.28736, 76.96922, 45.06913	16.64046, 16.53304, 17.63093	53.33384,	3	NA	0
GL173879.1	117260	117598	117332, 117474, 117318	23, 33, 9	23.8944, 35.01397, 9.41119	11.81108, 14.70608, 6.47287	20.54405,	3	NA	19399
GL173880.1	48810	49588	49492, 49233, 49235	5, 9, 6	4.82177, 8.21099, 7.29403	3.98509, 5.77858, 5.30581	2.51418, 5	3	5S_rRNA	1926
GL173880.1	89110	90034	89732, 89946, 89596	8, 10, 6	8.42257, 9.39128, 7.29403	5.97763, 6.35644, 5.30581	5.76336, 6	3	NA	11537
GL173882.1	139	719	406, 303, 199	10, 6, 4	11.06011, 4.93312, 4.67325	7.306, 4.04501, 3.78987	8.22712, 2	3	NA	NA
GL173882.1	22675	23229	22773, 23122, 22874	8, 9, 7	7.19971, 8.21099, 8.15288	5.27799, 5.77858, 5.81587	4.63758, 5	3	NA	NA
GL173882.1	27278	27915	27674, 27646, 27695	17, 8, 7	17.04639, 6.24338, 7.72582	9.50173, 4.6873, 5.6058	13.92543,	3	NA	NA
GL173882.1	45324	45825	45741, 45655, 45748	7, 9, 15	7.1696, 8.21099, 18.95532	5.31345, 5.77858, 10.98488	4.61046, 5	3	NA	NA
GL173884.1	21982	22703	22508, 22508, 22482	12, 16, 12	11.04522, 13.35594, 11.98157	7.02174, 7.51928, 7.58338	8.22317, 1	3	fam168a	482
GL173884.1	26064	26348	26285, 26325, 26249	7, 10, 8	4.76667, 5.75033, 6.50832	3.85544, 4.08112, 4.85389	2.51418, 3	3	fam168a	0
GL173884.1	30642	31336	30721, 30779, 30685	18, 22, 13	17.18556, 17.70531, 12.49731	9.31103, 8.4531, 7.70345	14.05939,	3	fam168a	0
GL173884.1	33884	34363	34110, 34150, 34304	8, 12, 6	6.34412, 7.75173, 5.60548	4.75087, 4.98057, 4.44624	3.871, 5.2	3	fam168a	0
GL173884.1	115121	115675	115552, 115387, 115487	7, 5, 3	7.1696, 3.94561, 3.47371	5.31345, 3.46715, 3.03189	4.61046, 1	3	fam168a	74475
GL173885.1	3869	4153	4002, 4133, 4147	4, 9, 5	3.39747, 6.10084, 4.91762	3.12111, 4.45669, 4.03768	1.46102, 3	3	NA	0
GL173885.1	15087	15605	15124, 15105, 15092	32, 44, 32	35.59971, 47.65339, 40.8184	15.45531, 17.35371, 18.53362	31.9745, 4	3	kpna1	0
GL173885.1	52221	53058	52759, 52759, 52753	9, 12, 6	8.25643, 11.84549, 6.93817	5.80588, 7.50292, 5.14396	5.62516, 9	3	ndc80	0
GL173885.1	82824	83614	83577, 83530, 83574	8, 22, 6	7.57423, 20.947, 6.76586	5.4999, 10.3685, 5.06179	4.98865, 1	3	gramd1c	0
GL173886.1	21514	22873	22203, 22680, 22650	11, 18, 10	12.43693, 17.00494, 12.90749	7.97018, 9.20137, 8.21675	9.52692, 1	3	NA	NA
GL173886.1	43238	44230	43578, 43689, 43759	5, 7, 9	4.82177, 5.97725, 11.64359	3.98509, 4.62287, 7.57973	2.51418, 3	3	NA	NA
GL173886.1	56567	57374	57349, 57348, 57301	30, 25, 13	34.59393, 25.42729, 13.0991	15.65348, 12.04472, 8.06282	30.98841,	3	NA	NA
GL173886.1	73712	74439	74013, 74182, 74174	18, 26, 16	20.25794, 25.04516, 18.26684	11.14243, 11.46708, 10.45568	17.01944,	3	NA	NA
GL173886.1	90971	91679	91345, 91368, 91535	11, 19, 9	10.5483, 15.48693, 7.58036	6.89817, 8.00194, 5.39321	7.75329, 1	3	NA	NA
GL173886.1	113726	114587	113817, 114334, 113568, 114109	5, 5, 4, 4	4.82177, 3.94561, 4.67325, 4.67325	3.98509, 3.46715, 3.78987, 3.78987	2.51418, 1	3	NA	NA
GL173886.1	122987	123728	123611, 123628, 123611	10, 6, 12	9.38842, 4.89312, 14.58408	6.35473, 4.02027, 9.01638	6.67167, 2	3	NA	NA
GL173887.1	2145	2869	2437, 2316, 2405	17, 32, 13	15.5079, 28.72483, 12.87254	8.56821, 11.48129, 7.92832	12.45101,	3	dcbld1	17109
GL173888.1	82315	83584	82966, 82798, 83001	13, 17, 14	14.43911, 18.56115, 17.94273	8.83395, 10.40145, 10.5654	11.43182,	3	NA	NA
GL173889.1	70196	70853	70541, 70374, 70556	6, 13, 12	5.24248, 11.17268, 15.80834	4.2332, 6.89372, 9.60504	2.89962, 8	3	NA	27874

GL173889.1	80123	80902	80546, 80554, 80282	10, 11, 4	11.06011, 10.3713, 4.64325	7.306, 6.79219, 3.77643	8.22712, 7	3	NA	37801
GL173890.1	99	437	375, 306, 399	13, 12, 8	15.29169, 11.86108, 10.1465	9.29854, 7.51216, 6.82176	12.24396,	3	NA	54721
GL173890.1	9309	9664	9407, 9587, 9591	8, 16, 7	8.34715, 17.16856, 8.69489	5.9367, 9.82359, 6.06379	5.71011, 1	3	NA	45494
GL173890.1	45674	46311	45930, 46124, 46111	5, 7, 6	4.81325, 5.61836, 5.67889	3.98038, 4.4008, 4.48822	2.51418, 3	3	NA	8847
GL173890.1	65109	65495	65313, 65371, 65138	6, 16, 8	5.90927, 15.41438, 8.24665	4.61743, 8.77591, 5.88165	3.50164, 1	3	NA	8433
GL173890.1	79054	79342	79176, 79068, 79165	17, 26, 11	17.4139, 24.16991, 12.55982	9.72241, 10.95194, 8.03554	14.27855,	3	NA	22378
GL173890.1	82807	83806	83411, 83313, 83616	9, 12, 4	9.00096, 9.74388, 4.67325	6.24222, 6.2187, 3.78987	6.30808, 7	3	NA	26131
GL173890.1	98469	99105	98693, 98693, 98691	10, 25, 10	9.13239, 25.58747, 9.88017	6.2007, 12.14055, 6.64506	6.43114, 2	3	NA	41793
GL173891.1	69296	69882	69483, 69439, 69413	41, 69, 36	37.28547, 62.51269, 35.58974	12.91045, 15.14924, 13.84963	33.62869,	3	NA	0
GL173892.1	13991	14249	14132, 14141, 14146	10, 9, 6	11.06011, 7.28344, 7.25145	7.306, 5.20811, 5.287	8.22712, 4	3	sema5b	0
GL173892.1	36607	37297	37139, 37217, 37084	10, 12, 7	8.70772, 9.74388, 6.59055	5.94148, 6.2187, 4.98809	6.03286, 7	3	dirc2	0
GL173892.1	60060	60704	60191, 60239, 60543	11, 16, 8	11.15818, 15.47832, 10.0913	7.25692, 8.81468, 6.79758	8.32008, 1	3	hspbap1	9414
GL173892.1	80683	81419	81126, 80791, 81098	9, 11, 5	6.545, 6.53768, 4.70718	4.74071, 4.41031, 3.9211	4.05782, 4	3	hspbap1	0
GL173892.1	83953	84508	84440, 84412, 84454	12, 20, 7	9.19354, 14.67897, 6.39902	5.87547, 7.26322, 4.87615	6.48895, 1	3	hspbap1	517
GL173892.1	87901	88692	88313, 88313, 88042	11, 12, 7	9.09169, 7.83184, 6.6707	6.00724, 5.02982, 5.03432	6.39391, 5	3	hspbap1	4465
GL173892.1	100595	101596	101154, 101104, 101086	18, 16, 13	15.9393, 12.29059, 14.25214	8.55398, 6.87192, 8.72916	12.86597,	3	slc15a2	0
GL173893.1	40309	41103	40626, 40621, 40617	14, 18, 11	14.03163, 16.12237, 13.64572	8.41471, 8.66512, 8.58592	11.04222,	3	ano9	28480
GL173894.1	37	713	309, 367, 371	8, 10, 17	8.42257, 9.39128, 24.08167	5.97763, 6.35644, 13.29929	5.76336, 6	3	NA	43077
GL173895.1	125	484	250, 204, 211	10, 6, 6	11.06011, 4.93312, 7.29403	7.306, 4.04501, 5.30581	8.22712, 2	3	NA	98722
GL173898.1	92019	92642	92248, 92473, 92604	8, 23, 7	8.42257, 24.32774, 7.50542	5.97763, 12.071, 5.49244	5.76336, 2	3	znf470	4273
GL173899.1	61	408	284, 236, 216	34, 38, 24	45.26209, 51.93702, 33.22758	20.32056, 22.53648, 16.82367	41.47997,	3	NA	NA
GL173899.1	29844	30624	30150, 30178, 30440	8, 10, 10	7.99469, 8.98028, 9.93124	5.74128, 6.10835, 6.67475	5.38155, 6	3	NA	NA
GL173899.1	65302	66304	65422, 65455, 65427	16, 39, 19	9.99067, 29.00137, 15.96207	5.51737, 9.4972, 8.28561	7.22782, 2	3	NA	NA
GL173899.1	94579	95642	95330, 95300, 95447	18, 29, 9	13.2432, 21.1912, 6.86115	6.94144, 8.39672, 4.94181	10.29479,	3	NA	NA
GL173899.1	105077	106186	105357, 105352, 105352	22, 43, 23	19.79638, 40.02891, 24.12143	9.68019, 13.61364, 11.9473	16.57342,	3	NA	NA
GL173900.1	109	600	526, 474, 329	14, 11, 5	15.49091, 10.3713, 5.31857	9.26387, 6.79219, 4.24819	12.43497,	3	NA	38788
GL173900.1	866	1602	1107, 1128, 1333	7, 13, 5	6.50909, 12.86565, 5.31857	4.94073, 7.92422, 4.24819	4.02371, 1	3	NA	37786
GL173900.1	21284	22138	21761, 21675, 21542	15, 21, 17	15.19946, 20.12483, 20.28053	8.88991, 10.19169, 11.37995	12.15964,	3	NA	17250
GL173900.1	46668	47006	46821, 46869, 46866	11, 16, 5	12.33515, 17.16856, 5.9506	7.91559, 9.82359, 4.54784	9.43699, 1	3	NA	0
GL173900.1	98251	98529	98335, 98350, 98341	8, 10, 8	8.42257, 9.39128, 10.1465	5.97763, 6.35644, 6.82176	5.76336, 6	3	U2	0
GL173901.1	30	413	334, 332, 162	13, 10, 8	15.29169, 9.39128, 10.1465	9.29854, 6.35644, 6.82176	12.24396,	3	pex11b	5703
GL173901.1	25740	26404	26272, 26278, 26274	22, 42, 26	22.93435, 42.76287, 28.86285	11.56366, 15.46468, 13.74298	19.61206,	3	XB-GENE-5994092	248
GL173901.1	63802	64607	64130, 64122, 64264	13, 18, 10	12.68462, 15.79712, 13.18184	7.81602, 8.46772, 8.33771	9.76298, 1	3	lmna	0
GL173901.1	87978	89355	88920, 88516, 88917	22, 22, 14	22.29116, 17.79763, 13.65217	11.17704, 8.50637, 8.18735	18.98923,	3	lmna	0
GL173901.1	103439	104238	104164, 103956, 103981	11, 14, 11	10.5483, 13.26509, 11.86426	6.89817, 7.95332, 7.65794	7.75329, 1	3	lmna	2535
GL173902.1	2802	4593	3428, 3491, 4055	19, 38, 18	19.10984, 34.79529, 19.01509	10.1875, 12.66766, 10.4125	15.91125,	3	MRAS	7776
GL173902.1	34163	34570	34298, 34308, 34197	5, 11, 9	4.40263, 9.17879, 9.46739	3.74533, 6.06147, 6.50386	2.21004, 6	3	MRAS	17842
GL173902.1	41132	41932	41508, 41579, 41538	7, 11, 8	4.60029, 9.57292, 7.07917	3.74674, 6.30569, 5.20531	2.38171, 6	3	MRAS	24811
GL173902.1	76880	77374	77017, 77242, 77123	12, 15, 3	12.60209, 13.6391, 3.47371	7.9443, 7.9452, 3.03189	9.68421, 1	3	MRAS	60559
GL173902.1	82894	83950	83148, 83205, 83172	12, 23, 19	9.2673, 18.70806, 19.82575	5.9215, 8.73854, 10.61774	6.55802, 1	3	MRAS	66573
GL173902.1	87897	88686	88553, 88472, 88110	7, 8, 5	5.10557, 4.98599, 4.04715	4.075, 3.87849, 3.53035	2.77482, 2	3	MRAS	71576
GL173902.1	103395	103890	103452, 103438, 103444	9, 13, 12	6.99678, 10.07856, 12.44386	5.02768, 6.21577, 7.85325	4.47395, 7	3	MRAS	87074
GL173902.1	111312	111681	111589, 111603, 111496	7, 27, 4	7.1696, 28.61693, 4.38152	5.31345, 13.23235, 3.65448	4.61046, 2	3	MRAS	94991
GL173903.1	13699	14153	13941, 13891, 13940	18, 24, 23	20.65644, 22.01781, 26.51165	11.37184, 10.35539, 13.36842	17.40599,	3	NA	0
GL173903.1	26743	27222	26808, 26873, 26987	16, 24, 14	17.95866, 27.39167, 17.61756	10.28064, 13.56183, 10.40352	14.80292,	3	zg16	0
GL173903.1	33135	33391	33273, 33197, 33316	11, 14, 9	11.6377, 13.44539, 9.52439	7.53109, 8.06258, 6.53516	8.77589, 1	3	zg16	5169
GL173903.1	110603	111369	111105, 110834, 111025	8, 10, 10	8.42257, 9.39128, 11.75646	5.97763, 6.35644, 7.66841	5.76336, 6	3	hist1h2bk	478
GL173904.1	47	1174	513, 719, 818	26, 36, 23	30.16559, 35.4501, 26.14485	14.51619, 13.77094, 13.15263	26.65425,	3	NA	45151
GL173904.1	7245	8235	7944, 7932, 7491	31, 29, 17	33.34702, 30.27596, 20.77652	14.51913, 13.47239, 11.65116	29.76641,	3	NA	38090
GL173904.1	14059	16631	15614, 15731, 15613	47, 68, 46	35.94987, 51.25377, 38.71023	10.44667, 11.19642, 11.95704	32.3174, 4	3	NA	29694
GL173904.1	19381	20052	19608, 19918, 19457, 19946	20, 26, 11, 18	8.43513, 9.59863, 3.64035, 8.63764	4.0747, 3.88211, 2.72147, 4.44254	5.77574, 7	3	NA	26273
GL173904.1	23376	26509	25482, 24965, 25044	21, 39, 19	13.29412, 24.6643, 13.41446	6.26571, 7.65468, 6.79346	10.34371,	3	NA	19816
GL173904.1	32932	33630	33113, 33078, 33077	10, 14, 8	6.72901, 10.08648, 6.57059	4.69767, 6.00146, 4.89275	4.22745, 7	3	NA	12695
GL173904.1	34764	35951	35404, 35333, 34877	30, 21, 16	26.35845, 13.98185, 14.89938	10.84833, 6.63201, 8.46267	22.93938,	3	NA	10374
GL173904.1	39735	40851	40435, 40201, 40410	20, 41, 25	15.60128, 34.71959, 25.11227	7.79457, 11.61691, 11.85637	12.54127,	3	NA	5474
GL173904.1	59935	61115	60574, 60246, 60222	20, 27, 17	17.42069, 23.10589, 17.13652	8.87054, 10.00869, 9.55597	14.2845, 1	3	NA	0
GL173904.1	71900	72731	72618, 72149, 72623	24, 22, 15	26.7757, 21.42357, 17.23823	13.19685, 10.65494, 10.06864	23.34605,	3	NA	273
GL173904.1	78282	79290	78535, 78927, 78894	26, 26, 24	26.68953, 23.06693, 27.25833	12.44476, 10.3106, 13.48302	23.26214,	3	NA	6655
GL173907.1	57381	57888	57400, 57534, 57514	8, 10, 6	7.62407, 9.39128, 6.99807	5.52896, 6.35644, 5.17195	5.03638, 6	3	top1.1	0
GL173907.1	83115	83609	83514, 83367, 83468	9, 14, 6	8.66407, 12.43922, 5.60548	6.04743, 7.44862, 4.44624	5.99101, 9	3	NA	8525
GL173908.1	49590	50059	50006, 50002, 50023	17, 22, 19	18.64535, 22.94165, 22.81433	10.45058, 11.56804, 12.35676	15.46286,	3	NA	22333
GL173908.1	71061	71623	71275, 71343, 71407	41, 54, 26	43.16346, 51.62808, 29.12527	16.10316, 15.39399, 13.89927	39.41181,	3	NA	769
GL173910.1	108535	109116	108914, 108920, 109054	11, 11, 4	12.43693, 10.60895, 4.67325	7.97018, 6.9343, 3.78987	9.52692, 7	3	NA	7722
GL173913.1	13249	13894	13584, 13461, 13485	11, 12, 7	12.01255, 11.86108, 8.69489	7.73996, 7.51216, 6.06379	9.13199, 9	3	naa38	8091
GL173913.1	29539	30447	30059, 30056, 30230	12, 11, 3	13.22036, 10.60895, 3.47371	8.29296, 6.9343, 3.03189	10.27237,	3	naa38	2453
GL173913.1	95191	95560	95452, 95446, 95467	9, 12, 4	8.66407, 10.35, 4.67325	6.04743, 6.59497, 3.78987	5.99101, 7	3	kdm6b	17447
GL173913.1	108525	109825	109026, 108949, 108525, 109394	5, 9, 3, 5	4.82177, 7.51164, 3.47371, 5.9506	3.98509, 5.35055, 3.03189, 4.54784	2.51418, 5	3	kdm6b	30781
GL173913.1	108525	109825	109640, 108949, 108525, 109394	4, 9, 3, 5	3.74195, 7.51164, 3.47371, 5.9506	3.32091, 5.35055, 3.03189, 4.54784	1.6222, 5.(	3	kdm6b	30781
GL173914.1	28180	28585	28545, 28478, 28298	9, 9, 7	6.39666, 5.34657, 5.73648	4.646, 3.97388, 4.47449	3.92119, 3	3	NA	NA
GL173914.1	32795	33118	32825, 32826, 32822	44, 58, 43	52.42524, 67.46518, 59.49041	20.10375, 21.95906, 24.71149	48.5407, 6	3	NA	NA
GL173915.1	5462	5982	5680, 5557, 5569	7, 10, 6	7.1696, 9.39128, 7.29403	5.31345, 6.35644, 5.30581	4.61046, 6	3	NA	NA
GL173915.1	53516	54414	53933, 53968, 53826	10, 24, 9	8.48936, 23.99953, 10.42289	5.80661, 11.53593, 7.00726	5.82603, 2	3	NA	NA
GL173915.1	70461	70720	70583, 70611, 70633	6, 9, 9	4.15609, 5.63274, 9.30116	3.55007, 4.15683, 6.41174	1.99613, 3	3	NA	NA
GL173915.1	73247	73681	73294, 73265, 73283	19, 16, 5	18.33514, 12.61232, 4.60894	9.71917, 7.0666, 3.8653	15.164, 9.8	3	NA	NA
GL173915.1	89230	89542	89444, 89508, 89516	10, 16, 12	9.54985, 16.98517, 15.5838	6.45088, 9.71597, 9.50165	6.82341, 1	3	NA	NA
GL173915.1	107971	108567	108035, 108184, 108538	5, 10, 6	4.82177, 9.39128, 7.29403	3.98509, 6.35644, 5.30581	2.51418, 6	3	NA	NA
GL173916.1	47950	48625	48129, 47978, 48229	8, 6, 3	8.42257, 4.93312, 3.47371	5.97763, 4.04501, 3.03189	5.76336, 2	3	NA	0
GL173916.1	66913	67729	67005, 67274, 67248	7, 15, 10	7.1696, 15.80067, 13.18184	5.31345, 9.24574, 8.33771	4.61046, 1	3	NA	18922
GL173916.1	66997	67729	67661, 67274, 67248	5, 15, 10	4.82177, 15.80067, 13.18184	3.98509, 9.24574, 8.33771	2.51418, 1	3	NA	19006
GL173916.1	101553	101754	101728, 101728, 101727	77, 90, 53	99.82725, 118.09576, 73.88477	30.00479, 32.72757, 28.22653	95.43803,	3	nat16	17821
GL173916.1	116636	117240	117142, 116968, 116924	16, 15, 13	16.37806, 12.13791, 15.61412	9.35612, 7.02404, 9.46817	13.28648,	3	nat16	2335
GL173916.1	140321	141033	140773, 140690, 140694	8, 14, 6	8.42257, 14.45897, 7.29403	5.97763, 8.66788, 5.30581	5.76336, 1	3	mogat3	0
GL173918.1	71942	72502	72353, 72464, 72455	8, 14, 15	8.34715, 14.45897, 19.64455	5.9367, 8.66788, 11.32852	5.71011, 1	3	NA	0
GL173919.1	82097	82887	82570, 82519, 82355	10, 12, 5	11.06011, 11.86108, 5.9506	7.306, 7.51216, 4.54784	8.22712, 9	3	ogfr	3699
GL173919.1	88954	89781	89455, 89544, 89135	7, 20, 15	6.89193, 18.71921, 17.94632	5.15998, 9.65224, 10.45626	4.37772, 1	3	ogfr	0
GL173919.1	96091	96573	96336, 96307, 96482	6, 10, 6	5.96715, 8.88645, 7.29403	4.64927, 6.0511, 5.30581	3.52158, 6	3	ogfr	6045
GL173920.1	39389	40710	40042, 39732, 39943	11, 22, 11	9.30417, 18.57394, 12.2694	6.13939, 8.95796, 7.88012	6.59351, 1	3	dnttip1	9836
GL173921.1	62	1877	1024, 1271, 1154	31, 41, 19	31.81583, 38.191, 20.8607	13.61594, 13.38287, 11.2325	28.26817,	3	NA	63883
GL173922.1	16105	17289	16975, 17033, 17033	22, 14, 13	28.30573, 14.45897, 17.16464	14.67216, 8.66788, 10.23255	24.83844,	3	mrto4	0
GL173922.1	41523	42328	41563, 41677, 41748	12, 17, 12	13.13892, 15.13134, 13.51536	8.24771, 8.33865, 8.45502	10.1962, 1	3	emc1	7881
GL173923.1	4464	4960	4796, 4809, 4803	10, 18, 11	10.01299, 19.41853, 12.06254	6.7221, 10.65157, 7.76744	7.24763, 1	3	5S_rRNA	15390
GL173923.1	16460	17024	16943, 16936, 16693	21, 32, 25	10.39168, 16.50783, 15.63688	4.82417, 5.70161, 6.58984	7.60739, 1	3	5S_rRNA	3326
GL173923.1	20409	20748	20593, 20580, 20571	126, 154, 117	145.49379, 171.02481, 139.10207	28.06031, 27.01892, 29.25261	140.79297	3	5S_rRNA	0
GL173923.1	42854	43541	43063, 43040, 43202	7, 13, 5	6.84137, 11.36332, 5.9506	5.13151, 7.01137, 4.54784	4.33107, 8	3	cpsf1	0
GL173923.1	89267	89667	89302, 89295, 89301	38, 52, 29	44.89375, 62.52823, 34.94567	18.41369, 22.10947, 16.24413	41.11632,	3	rgs19	166
GL173923.1	105684	106393	105928, 106249, 106127	14, 11, 6	16.23025, 10.60895, 7.29403	9.67496, 6.9343, 5.30581	13.14538,	3	rgs19	8280
GL173925.1	14233	16662	14725, 14683, 15611, 16308, 15643	27, 28, 17, 32, 13	26.92825, 23.39931, 11.41327, 28.24161, 13.33541	12.22714, 9.85591, 6.11746, 11.21535, 8.20195	23.4948, 2	3	casr	70620
GL173925.1	23288	24329	24063, 23523, 24032	19, 32, 14	17.94274, 29.74962, 14.86131	9.48142, 12.05289, 8.90303	14.78775,	3	casr	62953
GL173925.1	38808	39234	38916, 38953, 39077	8, 9, 10	6.86256, 6.93223, 10.30677	5.0733, 4.98685, 6.89026	4.3506, 4.5	3	casr	48048
GL173925.1	85140	85650	85591, 85624, 85619	24, 21, 15	25.45469, 21.03886, 16.67639	12.40824, 10.74286, 9.7523	22.06058,	3	casr	1632
GL173927.1	55	341	90, 249, 279	6, 8, 5	5.96715, 7.07159, 5.9506	4.64927, 5.20073, 4.54784	3.52158, 4	3	NA	NA
GL173927.1	18785	19127	18847, 18926, 18885	21, 21, 21	23.22463, 21.03886, 25.46511	12.05089, 10.74286, 13.3495	19.89343,	3	NA	NA
GL173928.1	71638	72628	71986, 72269, 72254	15, 41, 13	16.03559, 42.14993, 14.85247	9.3831, 15.53337, 9.06201	12.95858,	3	NA	NA
GL173930.1	85933	86708	86533, 86274, 86083	10, 7, 3	7.64746, 5.97725, 3.47371	5.27935, 4.62287, 3.03189	5.05779, 3	3	ccm2l	0
GL173930.1	109200	110752	109419, 109396, 110634, 109400	16, 19, 9, 11	17.95866, 19.8035, 7.43399, 12.48556	10.28064, 10.60442, 5.30221, 7.99611	14.80292,	3	csnk2a1	13669
GL173931.1	89	1011	663, 674, 867	48, 41, 29	49.46946, 40.96635, 30.32102	16.64639, 14.87712, 13.49912	45.62411,	3	xa-1	74527
GL173931.1	7922	9806	8648, 8629, 8656	29, 36, 26	26.97517, 34.40266, 25.79617	11.54031, 13.18562, 11.91238	23.54052,	3	xa-1	65732
GL173931.1	19557	20542	19924, 19815, 19653	22, 38, 20	17.12757, 32.59401, 17.9783	8.12209, 11.52006, 9.20537	14.00286,	3	xa-1	54996
GL173931.1	22528	23803	23481, 23284, 22621, 23520	16, 15, 15, 10	11.05358, 7.76192, 11.16794, 6.37737	6.13368, 4.457, 6.43227, 4.47517	8.22712, 5	3	xa-1	51735
GL173932.1	27775	29618	27857, 27856, 27875, 29471	16, 16, 20, 7	14.97744, 14.57865, 21.96564, 3.8682	8.51025, 8.26691, 11.60219, 3.26445	11.94792,	3	NA	NA
GL173932.1	32959	34471	33795, 33793, 34116	27, 53, 22	21.77481, 48.18554, 19.73919	9.26851, 14.1051, 9.64616	18.48801,	3	NA	NA
GL173932.1	46046	47083	46712, 46717, 46481	17, 23, 16	13.10638, 17.76591, 14.60098	7.11242, 8.20412, 8.28055	10.16442,	3	NA	NA
GL173932.1	63109	64780	64504, 64373, 64526	48, 54, 41	40.96087, 43.03845, 37.62717	12.37883, 11.54123, 13.08779	37.24308,	3	NA	NA
GL173932.1	73567	73966	73790, 73826, 73724	9, 15, 7	5.69017, 8.88599, 5.12471	4.1936, 5.08443, 4.08731	3.30453, 6	3	NA	NA
GL173932.1	103671	104437	104169, 104016, 104245	10, 12, 11	11.06011, 11.86108, 14.68355	7.306, 7.51216, 9.06344	8.22712, 9	3	NA	NA
GL173933.1	4427	4939	4684, 4639, 4809	9, 6, 7	9.72082, 4.93312, 7.50542	6.64182, 4.04501, 5.49244	6.97155, 2	3	hp1bp3	19590
GL173933.1	10070	10630	10389, 10534, 10606	20, 19, 17	20.60651, 18.6168, 19.99824	10.79142, 9.88966, 11.2232	17.35705,	3	hp1bp3	13899
GL173933.1	21449	22329	21943, 22181, 21704	4, 6, 5	3.74195, 4.93312, 5.3628	3.32091, 4.04501, 4.27046	1.6222, 2.7	3	hp1bp3	2200
GL173933.1	35467	36310	36272, 35681, 36248	5, 9, 6	4.82177, 8.21099, 7.29403	3.98509, 5.77858, 5.30581	2.51418, 5	3	hp1bp3	5218
GL173933.1	64484	65079	64645, 64577, 64486	16, 18, 6	16.23128, 18.52518, 7.29403	9.26839, 10.11992, 5.30581	13.14614,	3	eif4g3	0
GL173933.1	99394	99988	99478, 99429, 99554	19, 15, 5	24.34652, 15.80067, 5.9506	13.19266, 9.24574, 4.54784	20.98338,	3	eif4g3	0
GL173933.1	126379	126623	126471, 126453, 126464	25, 22, 12	32.58788, 25.85271, 16.36823	16.22977, 13.29074, 9.85365	29.02357,	3	eif4g3	24557
GL173934.1	70522	71353	70896, 70963, 71063	9, 15, 7	7.88835, 11.96891, 7.61369	5.58287, 6.92051, 5.54854	5.28191, 9	3	nup43	0
GL173935.1	10990	11312	11074, 11108, 11250	6, 6, 4	5.96715, 4.73925, 4.67325	4.64927, 3.92426, 3.78987	3.52158, 2	3	NA	56651
GL173935.1	33969	34752	34356, 34602, 34540	13, 17, 11	7.15359, 8.33852, 8.06567	4.44447, 4.44455, 5.3641	4.61046, 5	3	NA	33211
GL173935.1	40818	41502	41291, 41223, 41301	14, 19, 13	9.51969, 11.87408, 10.56182	5.65785, 5.93761, 6.51547	6.79598, 9	3	NA	26461
GL173937.1	7885	8570	7959, 7912, 7909	18, 31, 23	18.15064, 32.72958, 28.81839	9.89485, 14.15394, 14.6944	14.98708,	3	epha2	11412
GL173937.1	18209	18814	18508, 18510, 18439	14, 12, 7	12.18032, 8.99251, 6.88081	7.28937, 5.75004, 5.15373	9.29157, 6	3	epha2	1168
GL173937.1	81684	82092	82033, 81867, 81798	7, 10, 3	6.50909, 7.35725, 3.17999	4.94073, 5.09584, 2.89245	4.02371, 4	3	NA	25032
GL173937.1	82491	83015	82903, 82966, 82939	8, 22, 7	7.67464, 20.4944, 7.96278	5.55832, 10.09705, 5.72394	5.08299, 1	3	NA	25839
GL173937.1	96182	96441	96267, 96268, 96246	49, 85, 32	52.61655, 91.90572, 37.65014	17.92897, 22.07077, 16.672	48.72944,	3	NA	39530
GL173940.1	49222	49758	49579, 49743, 49385	9, 10, 9	6.82879, 7.05193, 8.26067	4.92128, 4.90236, 5.80842	4.31954, 4	3	NA	45828
GL173940.1	52619	53467	53002, 52915, 53121	11, 17, 5	9.09169, 14.4143, 4.22765	6.00724, 7.90204, 3.64077	6.39391, 1	3	NA	42119
GL173940.1	80308	80904	80663, 80539, 80819	12, 9, 4	10.1642, 7.06866, 4.46488	6.47994, 5.07306, 3.69425	7.39114, 4	3	NA	14682
GL173940.1	81106	81668	81246, 81350, 81553	8, 12, 6	5.83807, 9.86753, 6.6567	4.42916, 6.29567, 5.00845	3.43803, 7	3	NA	13918
GL173940.1	102051	102577	102477, 102229, 102484	10, 10, 5	10.96713, 9.39128, 5.9506	7.25596, 6.35644, 4.54784	8.14942, 6	3	NA	4196
GL173941.1	46660	47281	47060, 47008, 47039	9, 16, 4	9.43487, 15.54288, 4.59709	6.48595, 8.85379, 3.75554	6.71665, 1	3	U4	772
GL173941.1	58955	59849	59349, 59249, 59446	11, 11, 7	11.85906, 10.60895, 8.35502	7.65504, 6.9343, 5.9108	8.98595, 7	3	NA	0
GL173941.1	69791	70278	70053, 70172, 69894	8, 8, 5	8.42257, 7.07159, 5.9506	5.97763, 5.20073, 4.54784	5.76336, 4	3	NA	1837
GL173941.1	93481	93930	93585, 93615, 93750	4, 7, 5	3.70152, 5.97725, 5.9506	3.29816, 4.62287, 4.54784	1.6222, 3.6	3	NA	692
GL173942.1	39805	41425	40136, 40676, 40632, 41334	13, 19, 12, 9	7.99965, 10.65871, 9.12115, 6.21488	4.94388, 5.29481, 5.8303, 4.52972	5.38625, 8	3	U5	0
GL173942.1	42995	44546	43891, 43650, 43779	16, 19, 15	8.8891, 9.19554, 11.2311	4.90171, 4.56396, 6.47057	6.20307, 6	3	U5	0
GL173942.1	47844	49680	48533, 48955, 48372, 49553	23, 22, 13, 5	18.75967, 15.63682, 11.86258, 4.39541	8.76814, 7.28914, 7.31799, 3.74107	15.57324,	3	U5	0
GL173942.1	54709	55956	55153, 55286, 55252	14, 26, 13	12.28014, 26.23182, 14.93222	7.35082, 12.17172, 9.10541	9.38613, 2	3	U5	0
GL173942.1	66060	67091	66634, 66620, 66487	18, 20, 11	16.93135, 17.03084, 11.03484	9.15667, 8.63757, 7.18523	13.81374,	3	U5	0
GL173942.1	78434	81741	79762, 80781, 78923	21, 25, 14	16.32152, 13.75003, 10.3731	7.9377, 5.70043, 6.17652	13.23239,	3	U5	0
GL173942.1	78434	81741	81068, 80781, 78923	15, 25, 14	8.41212, 13.75003, 10.3731	4.81641, 5.70043, 6.17652	5.76336, 1	3	U5	0
GL173942.1	85021	87511	86905, 85621, 85074, 85806, 86170, 86828	24, 28, 9, 9, 10, 12	15.40726, 16.31071, 5.0332, 5.04977, 5.68365, 7.43533	6.69385, 6.29934, 3.77435, 3.78487, 4.03952, 4.78692	12.35531,	3	U5	0
GL173942.1	90094	96356	90922, 93650, 90815, 92301, 93792, 95653	19, 32, 14, 16, 14, 20	8.13128, 14.41022, 7.26692, 8.40116, 6.90006, 10.72646	4.06375, 4.93209, 4.34518, 4.63766, 4.14197, 5.14957	5.50886, 1	3	U5	0
GL173942.1	90094	96356	92207, 93650, 90815, 92301, 93792, 95653	22, 32, 14, 16, 14, 20	11.06254, 14.41022, 7.26692, 8.40116, 6.90006, 10.72646	4.97775, 4.93209, 4.34518, 4.63766, 4.14197, 5.14957	8.2293, 11	3	U5	0
GL173942.1	90094	96356	93756, 93650, 90815, 92301, 93792, 95653	20, 32, 14, 16, 14, 20	9.52366, 14.41022, 7.26692, 8.40116, 6.90006, 10.72646	4.57042, 4.93209, 4.34518, 4.63766, 4.14197, 5.14957	6.79981, 1	3	U5	0
GL173942.1	90094	96356	95960, 93650, 90815, 92301, 93792, 95653	18, 32, 14, 16, 14, 20	6.76133, 14.41022, 7.26692, 8.40116, 6.90006, 10.72646	3.56182, 4.93209, 4.34518, 4.63766, 4.14197, 5.14957	4.25784, 1	3	U5	0
GL173942.1	99411	101320	100356, 100193, 100770	27, 30, 19	20.00725, 20.04805, 14.64912	8.32101, 7.59063, 7.50698	16.77756,	3	U5	0
GL173943.1	41224	42085	42029, 41327, 41806	7, 11, 8	6.69449, 9.8562, 8.4586	5.04797, 6.47973, 5.99709	4.1953, 7.2	3	scarb2	0
GL173944.1	26689	27361	27328, 27331, 27278	13, 21, 9	9.22191, 15.17457, 6.38417	5.68647, 7.28701, 4.63801	6.51656, 1	3	camsap1	0
GL173944.1	39853	40743	40453, 40037, 40024	9, 11, 9	7.47395, 8.30667, 9.52439	5.32711, 5.51556, 6.53516	4.89626, 5	3	camsap1	3305
GL173944.1	59868	60454	60178, 60141, 60140	26, 24, 14	31.88377, 25.20527, 19.68329	15.52087, 12.25879, 11.3696	28.33509,	3	ubac1	11899
GL173945.1	79820	80325	80013, 80013, 79976	26, 24, 15	25.81707, 23.1305, 14.9375	11.92481, 11.01626, 8.73318	22.41278,	3	xtr-mir-428a	789
GL173945.1	83028	83518	83505, 83492, 83481	25, 30, 23	23.44347, 29.99979, 25.2841	10.86418, 12.93196, 12.64245	20.10629,	3	xtr-mir-428a	1842
GL173945.1	137357	138145	137779, 137727, 137779	14, 19, 9	13.51064, 17.28986, 10.42289	8.10201, 9.08588, 7.00726	10.55025,	3	cxxc1	296
GL173946.1	22451	22898	22603, 22702, 22702	14, 17, 17	14.99044, 16.56523, 19.29472	8.97777, 9.21112, 10.82557	11.95856,	3	NA	22851
GL173948.1	71406	72100	71525, 71930, 71932	22, 21, 21	18.58803, 15.9464, 21.38553	8.96622, 7.72286, 10.95169	15.40748,	3	NA	NA
GL173949.1	20030	20891	20864, 20843, 20842	46, 92, 39	53.48275, 109.83453, 50.72068	19.78126, 27.40195, 21.40283	49.58095,	3	NA	NA
GL173949.1	37106	37654	37265, 37292, 37278	12, 13, 10	13.8483, 13.14514, 13.18184	8.63436, 8.09002, 8.33771	10.86694,	3	NA	NA
GL173949.1	47775	48379	47790, 47872, 47848	12, 7, 6	11.55224, 5.97725, 7.29403	7.3281, 4.62287, 5.30581	8.69537, 3	3	NA	NA
GL173949.1	56650	57015	56685, 56655, 56667	39, 62, 43	46.06227, 71.76951, 50.88403	18.65855, 22.38323, 19.66836	42.26746,	3	NA	NA
GL173949.1	72195	72770	72242, 72221, 72198	30, 32, 28	34.14409, 29.74962, 33.28997	15.38622, 12.05289, 15.6403	30.54789,	3	NA	NA
GL173949.1	93003	93252	93214, 93225, 93198	13, 14, 18	13.34951, 14.44087, 23.23937	8.21021, 8.65722, 12.78599	10.39596,	3	NA	NA
GL173950.1	39820	40501	40163, 40398, 40071	9, 17, 5	8.88537, 15.5773, 5.31857	6.17591, 8.61051, 4.24819	6.19961, 1	3	NA	3197
GL173951.1	11274	11682	11492, 11657, 11596	8, 10, 11	8.42257, 9.39128, 14.13736	5.97763, 6.35644, 8.81822	5.76336, 6	3	itpr2	0
GL173951.1	19909	20330	19923, 19960, 19951	14, 17, 7	12.75226, 13.54833, 6.83761	7.64062, 7.37778, 5.12939	9.82689, 1	3	itpr2	0
GL173951.1	25375	25676	25635, 25626, 25623	6, 7, 10	5.81608, 5.16276, 11.83521	4.56563, 4.11176, 7.70798	3.4176, 2.9	3	itpr2	0
GL173951.1	81170	81411	81274, 81271, 81277	30, 38, 24	39.23927, 45.82759, 33.55553	18.365, 18.96527, 16.99207	35.54939,	3	itpr2	0
GL173951.1	93583	94225	93943, 93901, 94073	6, 11, 6	5.16999, 8.62761, 6.25896	4.18963, 5.71704, 4.80589	2.83325, 6	3	itpr2	0
GL173951.1	109596	110521	110481, 110230, 110164	16, 15, 5	12.97466, 10.97496, 3.68478	7.2868, 6.31545, 3.30148	10.03988,	3	itpr2	0
GL173951.1	114515	115051	114972, 114997, 115039	13, 32, 14	9.62543, 34.24151, 13.81849	5.93525, 14.65146, 8.28728	6.8956, 30	3	itpr2	0
GL173952.1	48703	49177	48797, 48927, 48876	5, 7, 6	4.69353, 5.48955, 6.5517	3.9134, 4.31982, 4.95623	2.45909, 3	3	NA	2973
GL173952.1	83876	85569	85237, 84324, 84491, 84831, 85215	9, 8, 6, 3, 3	9.56822, 7.07159, 7.29403, 3.47371, 3.47371	6.55912, 5.20073, 5.30581, 3.03189, 3.03189	6.84121, 4	3	NA	29291
GL173952.1	92020	93177	92320, 93052, 92168	28, 42, 37	26.09262, 38.33247, 36.73957	11.38154, 13.09711, 14.10729	22.68054,	3	NA	37435
GL173952.1	98712	99147	98829, 98835, 98770	42, 55, 49	52.3296, 70.73722, 65.83873	20.9839, 25.34237, 25.54571	48.4462, 6	3	NA	44127
GL173953.1	3652	4216	4082, 4010, 4130	7, 6, 6	7.1696, 4.93312, 7.29403	5.31345, 4.04501, 5.30581	4.61046, 2	3	NA	47039
GL173953.1	33248	33835	33685, 33630, 33332	9, 14, 7	7.88835, 11.36754, 7.61369	5.58287, 6.78813, 5.54854	5.28191, 8	3	NA	17420
GL173955.1	1646	2925	2428, 2456, 2508	32, 32, 26	19.28631, 16.2908, 17.16881	6.82444, 5.61891, 7.12246	16.0816, 1	3	SNTB2	52734
GL173955.1	3621	4361	3842, 3993, 4283	22, 24, 24	10.11919, 9.37325, 14.68904	4.55395, 4.00136, 6.33639	7.349, 6.81	3	SNTB2	51298
GL173956.1	34141	34504	34259, 34305, 34292	9, 7, 8	8.35434, 5.97725, 8.14528	5.86444, 4.62287, 5.82558	5.71584, 3	3	NA	NA
GL173956.1	53392	54484	53518, 53755, 53661, 54396	24, 37, 19, 15	17.6968, 27.64749, 15.16208, 10.68616	7.89346, 9.40272, 7.80916, 6.14138	14.55074,	3	NA	NA
GL173956.1	62263	62804	62562, 62588, 62556	10, 14, 6	8.66311, 10.57816, 4.67403	5.914, 6.30219, 3.8832	5.99101, 7	3	NA	NA
GL173956.1	62999	63479	63408, 63387, 63442	9, 12, 13	7.24594, 8.52999, 12.98465	5.18459, 5.4619, 7.995	4.68113, 6	3	NA	NA
GL173956.1	95027	95445	95246, 95246, 95385	17, 32, 6	20.46701, 35.62566, 6.168	11.48256, 15.4707, 4.75778	17.22179,	3	NA	NA
GL173957.1	17532	17989	17736, 17727, 17723	26, 26, 19	29.08397, 28.49224, 23.77131	13.87468, 13.52193, 12.88432	25.59847,	3	NA	1425
GL173957.1	72876	73233	73008, 73078, 72977	7, 11, 4	6.55439, 9.66539, 3.91395	4.96711, 6.36265, 3.41551	4.06616, 7	3	NA	2720
GL173961.1	73	1055	714, 667, 311	17, 20, 11	18.64535, 20.07667, 12.34038	10.45058, 10.47238, 7.91841	15.46286,	3	NA	1177
GL173961.1	77814	78747	78194, 78226, 77891	6, 15, 7	5.77068, 13.58442, 7.96278	4.54016, 7.91173, 5.72394	3.37745, 1	3	NA	0
GL173961.1	89033	89563	89467, 89161, 89166	7, 17, 8	7.1696, 16.43386, 7.50809	5.31345, 9.1315, 5.46116	4.61046, 1	3	NA	0
GL173962.1	16434	16870	16463, 16485, 16691	7, 10, 6	7.1696, 9.39128, 7.29403	5.31345, 6.35644, 5.30581	4.61046, 6	3	cntfr	16380
GL173962.1	94643	95233	94752, 94897, 94971	11, 12, 3	12.17114, 11.86108, 3.47371	7.82679, 7.51216, 3.03189	9.28263, 9	3	cntfr	1603
GL173963.1	666	2042	1261, 1341, 1253	6, 12, 7	5.96715, 10.83413, 8.69489	4.64927, 6.89288, 6.06379	3.52158, 8	3	tfr2	0
GL173963.1	45349	46639	45602, 45590, 45301, 46195	7, 7, 5, 6	6.37715, 5.82304, 4.20091, 5.39683	4.86324, 4.52813, 3.62458, 4.32487	3.90257, 3	3	NA	4496
GL173963.1	66268	66962	66646, 66367, 66349	7, 8, 6	5.58859, 6.27644, 7.29403	4.38214, 4.70819, 5.30581	3.21321, 3	3	NA	14401
GL173963.1	77303	77556	77479, 77362, 77474	8, 11, 7	7.67464, 8.66477, 7.40077	5.55832, 5.74034, 5.43746	5.08299, 6	3	NA	25436
GL173964.1	51297	52219	52094, 51885, 51461	9, 23, 6	6.82879, 20.53538, 5.91225	4.92128, 9.80281, 4.61907	4.31954, 1	3	NA	NA
GL173964.1	54497	54940	54785, 54760, 54851	10, 12, 7	8.32267, 8.8556, 8.08824	5.70305, 5.66466, 5.7849	5.6878, 6.3	3	NA	NA
GL173964.1	61941	62441	62331, 62040, 62039	6, 12, 9	4.89903, 11.86108, 10.72164	4.02393, 7.51216, 7.15493	2.58474, 9	3	NA	NA
GL173964.1	84383	85005	84755, 84763, 84736	10, 17, 15	9.71769, 15.99248, 18.23304	6.54998, 8.86336, 10.60943	6.97155, 1	3	NA	NA
GL173967.1	34066	35152	34952, 34776, 34723	10, 11, 4	11.06011, 10.60895, 4.67325	7.306, 6.9343, 3.78987	8.22712, 7	3	vegfa	0
GL173969.1	4502	5019	4929, 4746, 4954	10, 17, 4	11.06011, 18.19786, 4.67325	7.306, 10.18828, 3.78987	8.22712, 1	3	gli2	34157
GL173969.1	11512	12225	11732, 11838, 11912	4, 11, 6	3.74195, 10.53778, 7.29403	3.32091, 6.89189, 5.30581	1.6222, 7.9	3	gli2	26951
GL173969.1	34456	35324	34861, 34661, 34620	9, 12, 7	9.70623, 11.60251, 7.90186	6.63396, 7.3582, 5.69394	6.97147, 8	3	gli2	3852
GL173969.1	50616	51218	50902, 50789, 51049	4, 10, 4	3.63646, 9.17452, 3.62036	3.26116, 6.22617, 3.25193	1.6222, 6.6	3	gli2	0
GL173969.1	84567	84826	84578, 84583, 84584	27, 34, 21	31.91719, 37.73442, 28.88396	15.19434, 15.90028, 15.18096	28.36766,	3	gli2	18484
GL173969.1	91793	92568	91985, 92256, 92051	8, 14, 7	7.67464, 12.64814, 7.61369	5.55832, 7.57684, 5.54854	5.08299, 9	3	gli2	25710
GL173971.1	3687	4347	4316, 3794, 3828	10, 10, 7	8.24182, 8.12616, 6.43637	5.65265, 5.58036, 4.89813	5.61182, 5	3	utp6	2323
GL173971.1	5343	6139	5969, 5945, 5634	10, 18, 7	7.93364, 16.6391, 5.98393	5.45963, 8.9791, 4.62695	5.325, 13.7	3	utp6	531
GL173971.1	72075	73410	72830, 73102, 72783	12, 18, 15	11.98519, 17.79566, 15.61614	7.58551, 9.68065, 9.13715	9.10644, 1	3	crlf3	2200
GL173973.1	58	389	289, 286, 94	8, 8, 8	7.77807, 6.59014, 7.50809	5.618, 4.90493, 5.46116	5.17956, 4	3	NA	33
GL173973.1	1301	1901	1516, 1517, 1542	8, 16, 11	6.94372, 15.04317, 10.37715	5.12297, 8.55029, 6.79571	4.42528, 1	3	NA	0
GL173973.1	12626	13321	12881, 13036, 12724	5, 15, 13	4.82177, 15.80067, 16.0859	3.98509, 9.24574, 9.70995	2.51418, 1	3	NA	0
GL173973.1	50581	51454	51079, 51062, 51083	17, 14, 11	19.10743, 13.03306, 11.99553	10.71816, 7.81216, 7.73059	15.90925,	3	NA	0
GL173973.1	85910	87187	86557, 86184, 86624	6, 17, 13	5.68215, 16.49924, 14.77384	4.49007, 9.17114, 9.01902	3.29732, 1	3	NA	32871
GL173973.1	91463	91963	91596, 91617, 91837	8, 10, 8	8.42257, 9.39128, 9.60885	5.97763, 6.35644, 6.57807	5.76336, 6	3	NA	38424
GL173974.1	61758	62079	61829, 61835, 61905	19, 32, 11	16.08128, 28.6023, 11.20072	8.35706, 11.41363, 7.28154	13.00262,	3	NA	NA
GL173974.1	63692	64194	63753, 64079, 63892	12, 9, 4	9.15708, 5.36772, 3.43992	5.85272, 3.98738, 3.14645	6.45494, 3	3	NA	NA

GL173975.1	15169	15699	15549, 15473, 15508	13, 9, 8	11.21374, 6.8008, 7.34578	6.91908, 4.90352, 5.36526	8.37313, 4	3	clcnkb	0
GL173975.1	24043	24502	24207, 24157, 24188	12, 9, 6	13.8483, 8.21099, 6.82211	8.63436, 5.77858, 5.08889	10.86694,	3	clcnkb	7104
GL173975.1	44267	45542	44606, 44630, 45264	82, 87, 39	85.96878, 83.51506, 37.63195	20.61, 17.79781, 13.82905	81.71088,	3	gigyf1	2625
GL173975.1	79637	80452	79931, 79962, 80082	18, 21, 15	15.72423, 19.48223, 17.49481	8.42352, 9.80473, 10.21058	12.65901,	3	NA	6014
GL173975.1	93410	93744	93458, 93459, 93653	28, 29, 13	26.70619, 23.37083, 11.77124	11.73811, 9.53331, 7.26208	23.27819,	3	NA	5305
GL173975.1	98979	99951	99572, 99567, 99518	78, 105, 52	79.41463, 108.20036, 53.85411	19.24684, 21.29153, 17.30994	75.2234, 1	3	NA	10874
GL173979.1	55419	56620	56249, 56278, 56063	18, 17, 14	18.07742, 14.03605, 15.80333	9.85073, 7.67248, 9.43935	14.9177, 1	3	camk4	11734
GL173979.1	64387	64789	64563, 64611, 64604	11, 11, 10	10.78278, 9.66539, 11.997	7.03733, 6.36265, 7.78835	7.97565, 7	3	stard4	13179
GL173979.1	89033	89729	89557, 89524, 89530	5, 13, 9	4.82177, 13.14514, 11.58206	3.98509, 8.09002, 7.55286	2.51418, 1	3	stard4	3164
GL173981.1	15419	15848	15728, 15502, 15509	7, 13, 9	7.1696, 11.03409, 9.64095	5.31345, 6.80804, 6.59867	4.61046, 8	3	NA	18111
GL173981.1	21526	21814	21780, 21795, 21793	14, 17, 13	13.57329, 15.63512, 14.77384	8.13982, 8.64575, 9.01902	10.60942,	3	NA	12145
GL173981.1	43848	44580	43856, 43967, 44410	13, 8, 5	14.52571, 6.97779, 5.80393	8.88215, 5.14375, 4.48186	11.51483,	3	NA	5244
GL173981.1	53389	53994	53665, 53426, 53635	8, 7, 8	6.70593, 4.24316, 8.6841	4.9768, 3.51202, 6.11715	4.20487, 2	3	NA	14785
GL173981.1	71553	72393	72141, 72159, 71783, 72162	31, 61, 12, 23	24.17431, 53.18874, 9.47337, 22.69931	9.3661, 13.7474, 6.05008, 11.09335	20.81623,	3	NA	32949
GL173982.1	16486	16922	16584, 16826, 16820	7, 8, 3	7.1696, 7.07159, 3.47371	5.31345, 5.20073, 3.03189	4.61046, 4	3	NA	23350
GL173983.1	35599	36049	35711, 35894, 35830	11, 10, 5	12.43693, 9.39128, 5.9506	7.97018, 6.35644, 4.54784	9.52692, 6	3	rhot1	12639
GL173983.1	65275	65886	65806, 65582, 65439	6, 9, 3	5.96715, 8.21099, 3.47371	4.64927, 5.77858, 3.03189	3.52158, 5	3	rhot1	0
GL173984.1	33691	34308	33893, 34024, 34129	7, 8, 6	7.1696, 6.66413, 7.12188	5.31345, 4.9509, 5.22884	4.61046, 4	3	NA	16872
GL173985.1	28085	28562	28521, 28531, 28521	19, 26, 18	19.96873, 25.54817, 21.79524	10.70325, 11.76506, 12.01217	16.74065,	3	spag17	0
GL173985.1	45302	45712	45684, 45692, 45681	15, 16, 19	14.21298, 14.46749, 21.69099	8.29535, 8.19898, 11.71707	11.21566,	3	spag17	271
GL173985.1	87738	88675	88403, 88050, 88493	10, 11, 5	11.06011, 10.60895, 5.9506	7.306, 6.9343, 4.54784	8.22712, 7	3	spag17	42707
GL173986.1	27650	28134	27859, 27981, 27883	12, 15, 8	11.92103, 13.69427, 9.68495	7.54764, 7.97895, 6.61366	9.04508, 1	3	c1qtnf4	915
GL173987.1	54	1013	660, 653, 671	28, 42, 24	32.38019, 48.38512, 29.81695	15.10266, 18.66765, 14.96922	28.8203, 4	3	NA	NA
GL173987.1	49452	50968	49704, 50277, 50429	30, 46, 26	25.29203, 38.2706, 25.0423	10.26157, 11.75356, 11.46539	21.90377,	3	NA	NA
GL173987.1	59221	60497	59528, 59821, 60399	23, 25, 15	17.89062, 14.38302, 11.65918	8.2742, 5.99139, 6.73116	14.73817,	3	NA	NA
GL173987.1	83464	84253	83872, 84112, 84124	11, 17, 10	12.43693, 17.71433, 12.16489	7.97018, 9.9018, 7.87042	9.52692, 1	3	NA	NA
GL173988.1	34927	35265	35105, 35226, 35237	12, 20, 13	9.5349, 17.71959, 13.70995	6.08845, 9.04982, 8.41989	6.80991, 1	3	NA	NA
GL173988.1	68229	68847	68547, 68682, 68665	8, 10, 13	8.42257, 9.39128, 18.01082	5.97763, 6.35644, 10.61163	5.76336, 6	3	NA	NA
GL173989.1	23684	24438	24378, 24312, 24395	19, 23, 20	15.41096, 20.1396, 20.10979	7.95676, 9.56971, 10.49235	12.35876,	3	NA	17850
GL173989.1	26223	26576	26457, 26461, 26442	15, 9, 13	11.99233, 6.15408, 11.54949	6.93485, 4.4908, 7.12599	9.11296, 3	3	NA	15712
GL173989.1	34369	35008	34648, 34498, 34742	10, 6, 5	9.44155, 4.93312, 4.84526	6.38646, 4.04501, 3.99806	6.72156, 2	3	NA	7280
GL173989.1	64215	64733	64523, 64408, 64397	15, 20, 13	16.28407, 19.71581, 14.11158	9.52725, 10.25455, 8.64973	13.19656,	3	NA	9043
GL173989.1	76806	77437	77352, 77217, 77213	15, 11, 17	10.84562, 5.33547, 15.14737	6.23737, 3.68368, 8.34843	8.03447, 3	3	NA	21634
GL173989.1	85843	86940	86174, 85972, 86600	16, 12, 13	14.97744, 8.46754, 11.90884	8.51025, 5.42308, 7.34626	11.94792,	3	NA	30671
GL173990.1	70286	70633	70587, 70602, 70589	123, 193, 122	155.52019, 242.69926, 166.20607	33.82457, 37.55375, 39.60068	150.76471	3	NA	17253
GL173990.1	83280	83681	83473, 83421, 83490	9, 14, 11	9.24298, 14.45897, 14.68355	6.3792, 8.66788, 9.06344	6.53474, 1	3	NA	30247
GL173991.1	1855	2406	1885, 1883, 1881	8, 11, 14	7.28955, 10.59463, 16.46576	5.33177, 6.92577, 9.80275	4.72176, 7	3	NA	2202
GL173991.1	41094	41502	41122, 41113, 41195	12, 29, 7	13.8483, 35.38702, 8.21885	8.63436, 16.503, 5.84717	10.86694,	3	znf37a	14021
GL173991.1	52905	53307	53112, 53167, 53175	6, 13, 8	4.86712, 9.40471, 7.72242	4.00414, 5.79902, 5.58595	2.55558, 6	3	znf37a	2216
GL173991.1	71297	72285	71754, 71377, 71714	4, 12, 6	3.74195, 11.86108, 7.29403	3.32091, 7.51216, 5.30581	1.6222, 9.1	3	NA	0
GL173992.1	26	552	309, 264, 312	13, 17, 8	15.29169, 18.56115, 10.1465	9.29854, 10.40145, 6.82176	12.24396,	3	idh3b	33674
GL173992.1	18471	18896	18842, 18843, 18817	24, 38, 13	24.20923, 40.45844, 14.77384	11.6616, 15.81947, 9.01902	20.85028,	3	idh3b	15330
GL173993.1	45175	46306	45187, 46022, 45209	5, 7, 6	4.82177, 5.97725, 7.12188	3.98509, 4.62287, 5.22884	2.51418, 3	3	NA	0
GL173993.1	58399	58814	58600, 58756, 58768	16, 20, 13	16.75964, 18.53364, 14.5443	9.5829, 9.54017, 8.89247	13.65293,	3	NA	0
GL173993.1	74077	75206	74455, 74313, 74305, 74879	10, 21, 12, 4	10.67246, 19.54437, 15.36829, 4.30163	7.09497, 9.8421, 9.40047, 3.61556	7.87096, 1	3	ino80b	182
GL173993.1	81456	82163	81867, 81745, 81567	11, 12, 4	10.9046, 10.93692, 4.38152	7.10903, 6.95571, 3.65448	8.08987, 8	3	ino80b	7561
GL173993.1	82390	82978	82752, 82740, 82780	16, 16, 11	17.59404, 15.94397, 13.93476	10.07113, 9.09593, 8.7238	14.45148,	3	mogs	7039
GL173994.1	46	1022	170, 401, 343	10, 19, 13	11.06011, 20.29855, 16.93213	7.306, 10.89984, 10.12358	8.22712, 1	3	pfn1	17455
GL173994.1	10508	11425	10749, 11113, 10958	7, 14, 13	5.86458, 12.43922, 14.39632	4.55375, 7.44862, 8.81006	3.46146, 9	3	pfn1	7052
GL173994.1	16691	17786	16843, 16993, 17726	8, 9, 8	7.28955, 7.75488, 8.80246	5.33177, 5.501, 6.179	4.72176, 5	3	pfn1	691
GL173994.1	55158	55734	55635, 55562, 55540	14, 28, 10	16.7649, 29.22753, 13.18184	9.96273, 13.22571, 8.33771	13.6543, 2	3	XB-GENE-5720006	14076
GL173994.1	62841	63414	63189, 63037, 63007	18, 18, 7	21.75314, 19.97713, 8.69489	11.98894, 10.97931, 6.06379	18.46685,	3	XB-GENE-5720006	6396
GL173994.1	72479	73036	72580, 72580, 72570	25, 14, 7	26.50188, 12.49074, 6.75301	12.68794, 7.48027, 5.08141	23.07912,	3	XB-GENE-5720006	0
GL173994.1	84206	84600	84398, 84355, 84356	53, 40, 36	67.19766, 47.14112, 45.76396	24.31131, 18.84485, 19.79761	63.13057,	3	XB-GENE-5720006	11166
GL173995.1	8	1131	720, 657, 169	6, 6, 5	5.96715, 4.93312, 5.9506	4.64927, 4.04501, 4.54784	3.52158, 2	3	NA	NA
GL173995.1	6527	7266	6924, 6838, 7117	8, 6, 8	7.77807, 4.93312, 9.60885	5.618, 4.04501, 6.57807	5.17956, 2	3	NA	NA
GL173995.1	79654	81057	80327, 80915, 80782	7, 10, 8	6.89193, 9.39128, 9.1831	5.15998, 6.35644, 6.37229	4.37772, 6	3	NA	NA
GL173996.1	11400	12865	12577, 12505, 12107	9, 17, 13	8.50607, 16.76699, 13.45746	5.95453, 9.33319, 8.27333	5.84194, 1	3	NA	23922
GL173996.1	37258	37856	37755, 37581, 37409	6, 11, 7	5.03089, 9.3497, 6.63036	4.10511, 6.16764, 5.0111	2.70624, 6	3	NA	0
GL173996.1	52319	53039	52940, 52939, 52954	18, 28, 12	17.79149, 27.46787, 14.49372	9.67812, 12.18424, 8.97057	14.64268,	3	NA	0
GL173998.1	38682	38961	38935, 38911, 38781	9, 18, 7	7.17299, 17.19487, 6.92462	5.13877, 9.31668, 5.17829	4.61317, 1	3	helz	0
GL173998.1	86383	87225	86425, 86451, 86639	33, 40, 24	36.86175, 43.34221, 29.81695	15.7964, 16.63072, 14.96922	33.21271,	3	cacng4	0
GL173998.1	89330	90515	89785, 90363, 89875	12, 10, 5	8.87521, 5.79188, 3.83562	5.67689, 4.10708, 3.39785	6.19102, 3	3	cacng4	0
GL174000.1	153	698	178, 186, 455	6, 16, 8	5.96715, 17.16856, 10.1465	4.64927, 9.82359, 6.82176	3.52158, 1	3	NA	1413
GL174000.1	51710	52516	52307, 52322, 51800, 52417	6, 12, 4, 3	5.90927, 11.04188, 4.67325, 3.47371	4.61743, 7.01971, 3.78987, 3.03189	3.50164, 8	3	NA	13529
GL174000.1	59934	60586	60197, 60138, 60014	5, 23, 12	4.82177, 23.5013, 14.58408	3.98509, 11.57503, 9.01638	2.51418, 2	3	NA	21753
GL174001.1	41708	42402	41729, 41724, 41789	19, 19, 13	21.81444, 17.17455, 15.70541	11.78833, 9.01609, 9.51556	18.52575,	3	mrpl48	0
GL174002.1	8608	9245	8734, 8772, 8893	9, 10, 6	9.43487, 7.55261, 5.91225	6.48595, 5.21944, 4.61907	6.71665, 5	3	USH1C	12539
GL174002.1	12195	12991	12386, 12433, 12615	9, 13, 7	9.06007, 9.50531, 6.55126	6.27591, 5.86107, 4.96529	6.36394, 6	3	USH1C	8793
GL174002.1	16628	17762	17567, 17584, 17574	15, 22, 10	15.49092, 20.88093, 10.71812	9.06314, 10.32883, 7.12015	12.43497,	3	USH1C	4022
GL174002.1	22147	22638	22377, 22229, 22467	10, 7, 7	10.01299, 5.58561, 7.29952	6.7221, 4.38027, 5.38357	7.24763, 3	3	USH1C	0
GL174002.1	39074	39341	39181, 39307, 39300	5, 6, 6	4.82177, 4.93312, 7.29403	3.98509, 4.04501, 5.30581	2.51418, 2	3	USH1C	0
GL174002.1	40350	40855	40535, 40652, 40585	4, 7, 4	3.6687, 5.97725, 4.67325	3.27956, 4.62287, 3.78987	1.6222, 3.6	3	USH1C	0
GL174002.1	60551	61355	61071, 61003, 61217	15, 26, 14	13.41505, 25.54817, 16.64329	7.80796, 11.76506, 9.89801	10.45899,	3	USH1C	0
GL174002.1	86298	87701	87065, 86552, 86789	14, 15, 19	15.15267, 15.80067, 23.01756	9.07115, 9.24574, 12.47022	12.11465,	3	USH1C	0
GL174003.1	44309	44568	44521, 44543, 44543	13, 26, 8	12.43975, 26.7954, 8.4586	7.66875, 12.50798, 5.99709	9.5289, 23	3	ATG7	0
GL174003.1	48691	49136	48885, 48708, 48748	9, 15, 12	7.97705, 13.4765, 14.14699	5.637, 7.84563, 8.79187	5.36577, 1	3	ATG7	0
GL174003.1	60175	61173	60595, 60788, 60632	14, 20, 8	12.0338, 15.8975, 7.19087	7.19911, 7.96752, 5.27268	9.15254, 1	3	ATG7	3157
GL174003.1	103673	104273	103990, 104123, 103941	8, 8, 5	8.34715, 7.07159, 5.9506	5.9367, 5.20073, 4.54784	5.71011, 4	3	vgll4	13114
GL174005.1	52039	52838	52352, 52572, 52402	15, 14, 12	13.57755, 11.80872, 13.15613	7.90752, 7.06033, 8.25729	10.61316,	3	she	0
GL174006.1	889	1757	1473, 1390, 1508	5, 14, 7	4.82177, 14.45897, 8.69489	3.98509, 8.66788, 6.06379	2.51418, 1	3	TLN1	15532
GL174008.1	26403	26632	26582, 26593, 26616	13, 15, 12	13.49269, 14.39783, 15.26371	8.29387, 8.40758, 9.35067	10.53271,	3	NA	6012
GL174009.1	49506	51121	49964, 50892, 49657	14, 8, 7	10.98164, 5.23755, 6.08239	6.55025, 4.04172, 4.68683	8.16315, 3	3	NA	20234
GL174010.1	11969	13980	12591, 12782, 12294, 13843	36, 46, 18, 12	27.8035, 32.75655, 15.3061, 9.02948	9.74974, 9.3764, 8.17043, 5.7731	24.34845,	3	NA	NA
GL174010.1	15986	16974	16049, 16535, 16074	21, 36, 16	13.32334, 24.69521, 13.82283	6.28109, 8.31403, 7.80466	10.3719, 2	3	NA	NA
GL174010.1	24788	25587	25410, 25308, 25316	20, 25, 10	16.13183, 19.12602, 8.36243	8.10503, 8.39863, 5.7278	13.0512, 1	3	NA	NA
GL174011.1	6236	6778	6694, 6402, 6506	5, 11, 6	4.82177, 9.8562, 6.168	3.98509, 6.47973, 4.75778	2.51418, 7	3	NA	NA
GL174011.1	16525	18111	17194, 17411, 17036	8, 18, 8	7.99469, 19.97713, 10.1465	5.74128, 10.97931, 6.82176	5.38155, 1	3	NA	NA
GL174011.1	37727	39473	38282, 38701, 38337, 39269	18, 14, 14, 8	17.86195, 12.80997, 15.49745, 8.24665	9.72071, 7.67593, 9.26757, 5.88165	14.71061,	3	NA	NA
GL174011.1	47551	48330	47997, 47884, 47792	9, 16, 7	9.72082, 17.16856, 8.69489	6.64182, 9.82359, 6.06379	6.97155, 1	3	NA	NA
GL174011.1	63550	65223	64181, 64418, 63812, 64947	25, 25, 12, 6	24.48676, 23.54439, 12.7555, 4.62428	11.48315, 10.9238, 8.03189, 3.85173	21.11974,	3	NA	NA
GL174011.1	69261	70422	69473, 69790, 69387, 70300	10, 12, 12, 7	7.41295, 10.08092, 11.66047, 5.95183	5.1311, 6.42828, 7.39284, 4.60732	4.8384, 7.4	3	NA	NA
GL174012.1	1009	2907	2445, 1184, 2404	55, 66, 30	39.86192, 49.81899, 23.1724	10.06008, 11.14931, 9.13595	36.16146,	3	NA	NA
GL174012.1	18500	19576	19193, 19234, 19035	33, 35, 18	38.68132, 35.70133, 20.93229	16.87521, 14.30597, 11.52911	34.99999,	3	NA	NA
GL174016.1	78	335	284, 299, 297	6, 18, 5	5.96715, 19.97713, 5.9506	4.64927, 10.97931, 4.54784	3.52158, 1	3	NA	NA
GL174016.1	23914	25264	23934, 24899, 24565	5, 12, 5	4.20732, 10.73343, 5.23257	3.62846, 6.83116, 4.20435	2.04024, 8	3	NA	NA
GL174016.1	24062	25264	24735, 24899, 24565	6, 12, 5	5.24248, 10.73343, 5.23257	4.2332, 6.83116, 4.20435	2.89962, 8	3	NA	NA
GL174016.1	24062	25479	25270, 24899, 24565	5, 12, 5	4.20732, 10.73343, 5.23257	3.62846, 6.83116, 4.20435	2.04024, 8	3	NA	NA
GL174017.1	6989	8749	7046, 7572, 8342	9, 26, 13	8.40425, 22.95126, 13.84064	5.89416, 10.24395, 8.49513	5.76336, 1	3	XB-GENE-5798855	1501
GL174017.1	7000	8749	8395, 7572, 8342	11, 26, 13	10.1122, 22.95126, 13.84064	6.63573, 10.24395, 8.49513	7.34229, 1	3	XB-GENE-5798855	1501
GL174017.1	41455	42093	41906, 41952, 41589	12, 6, 7	10.88704, 4.08833, 6.36213	6.92524, 3.50569, 4.85436	8.0744, 2.0	3	NA	0
GL174018.1	71301	71651	71337, 71383, 71559	5, 6, 3	4.82177, 4.93312, 3.47371	3.98509, 4.04501, 3.03189	2.51418, 2	3	NA	4371
GL174019.1	1210	1784	1366, 1490, 1658	6, 13, 8	5.27951, 11.87216, 9.92329	4.25534, 7.32385, 6.7228	2.9335, 9.1	3	senp3	1249
GL174019.1	8926	9516	9027, 9446, 9084	9, 16, 3	7.32038, 14.41261, 3.47371	5.23124, 8.16543, 3.03189	4.75116, 1	3	eif4a1	0
GL174019.1	46027	46598	46225, 46319, 46117	13, 7, 6	14.97989, 5.97725, 7.29403	9.13126, 4.62287, 5.30581	11.94968,	3	mpdu1	0
GL174019.1	68575	69007	68792, 68624, 68903	8, 11, 5	7.02691, 9.13706, 5.9506	5.17362, 6.0355, 4.54784	4.50222, 6	3	mpdu1	10474
GL174019.1	71526	72047	71880, 71737, 71868	9, 9, 4	8.45482, 7.75488, 4.67325	5.92419, 5.501, 3.78987	5.79307, 5	3	mpdu1	13425
GL174023.1	77	512	163, 209, 162	12, 18, 9	13.8483, 19.97713, 11.64359	8.63436, 10.97931, 7.57973	10.86694,	3	NA	NA
GL174023.1	77255	77902	77563, 77711, 77835	10, 12, 12	11.06011, 11.86108, 16.36823	7.306, 7.51216, 9.85365	8.22712, 9	3	NA	NA
GL174024.1	36364	36894	36666, 36667, 36740	10, 9, 5	10.74428, 8.21099, 5.9506	7.13454, 5.77858, 4.54784	7.93927, 5	3	slc30a3	10148
GL174025.1	6365	7343	6668, 6683, 6490, 7115	8, 18, 4, 5	7.57423, 17.19487, 4.11547, 5.31857	5.4999, 9.31668, 3.5218, 4.24819	4.98865, 1	3	NA	NA
GL174025.1	24483	24787	24669, 24780, 24750	10, 26, 11	8.84451, 24.23472, 13.46208	6.02544, 10.98992, 8.49639	6.16137, 2	3	NA	NA
GL174027.1	65844	66242	66076, 65877, 66205	5, 9, 5	3.35103, 6.43335, 4.39541	3.08326, 4.66944, 3.74107	1.42597, 4	3	znf484	21132
GL174029.1	17526	18336	17566, 18048, 18183	10, 11, 8	7.10058, 6.67455, 7.11599	4.9332, 4.49475, 5.22758	4.57191, 4	3	pnliprp2	0
GL174029.1	19840	20337	19850, 19896, 19850	9, 19, 9	6.48491, 14.46084, 8.05901	4.70237, 7.39684, 5.6868	4.002, 11.6	3	pnliprp2	0
GL174029.1	32984	33796	33763, 33522, 33412	7, 16, 7	6.25077, 14.69174, 8.21885	4.78814, 8.33598, 5.84717	3.78441, 1	3	pnliprp2	3877
GL174029.1	40871	41350	41239, 40911, 41041	19, 28, 19	18.9632, 29.05602, 22.42243	10.09902, 13.12373, 12.13591	15.76968,	3	pnlip	528
GL174029.1	63925	64364	64363, 64255, 64351	7, 13, 8	5.93788, 10.43179, 8.4586	4.59878, 6.43483, 5.99709	3.52158, 7	3	kcnk18	0
GL174029.1	65408	66190	65558, 65595, 65746	18, 25, 9	18.68415, 23.34787, 8.93877	10.2151, 10.80775, 6.20662	15.49996,	3	kcnk18	112
GL174029.1	72425	73099	72863, 72669, 72837	12, 17, 8	10.20921, 13.9447, 7.76694	6.50784, 7.61716, 5.6116	7.4341, 11	3	kcnk18	7129
GL174030.1	14	799	403, 271, 294	37, 41, 23	37.05853, 37.18641, 26.32652	14.28688, 12.85926, 13.25965	33.40617,	3	NA	NA
GL174030.1	8972	9750	9205, 9353, 9674	11, 14, 9	8.262, 9.08069, 8.38703	5.4875, 5.39466, 5.88392	5.63022, 6	3	NA	NA
GL174030.1	34228	34952	34640, 34546, 34816	11, 14, 9	9.52723, 10.76513, 8.65301	6.27749, 6.41704, 6.04096	6.80251, 8	3	NA	NA
GL174030.1	72476	73268	73036, 73035, 73037	54, 48, 34	55.55353, 40.40725, 34.53438	17.36561, 12.12456, 14.02905	51.62274,	3	NA	NA
GL174031.1	65	1429	839, 717, 730	16, 25, 14	17.2479, 25.11421, 15.35015	9.86999, 11.85753, 9.184	14.1196, 2	3	NA	32681
GL174031.1	7996	12125	11121, 9004, 11149	35, 41, 34	22.94892, 22.57032, 27.89122	7.77678, 6.5265, 10.38237	19.62605,	3	NA	21985
GL174031.1	17864	21437	18619, 19069, 18617	36, 48, 25	26.70339, 33.67845, 18.93058	9.227, 9.28614, 8.29224	23.27544,	3	NA	12673
GL174031.1	26723	30307	28607, 28588, 28487	40, 47, 39	26.26333, 26.42976, 31.69717	8.09036, 6.90153, 10.76081	22.84637,	3	NA	3803
GL174031.1	43842	44696	44020, 44245, 44125	12, 31, 8	5.34056, 14.95265, 4.3023	3.55446, 5.25796, 3.43511	2.98967, 1	3	NA	9218
GL174031.1	44975	46733	45718, 45415, 45050	24, 36, 15	15.048, 18.87273, 10.32257	6.51386, 5.97908, 5.92372	12.01496,	3	NA	10351
GL174031.1	70225	71477	71302, 70880, 70687	26, 53, 22	13.26206, 36.22367, 13.79982	5.31877, 9.16798, 6.31252	10.31316,	3	NA	35601
GL174031.1	72889	74016	73423, 73430, 73431	39, 30, 30	19.94808, 9.55628, 15.80581	5.92299, 3.53151, 5.73705	16.7205, 6	3	NA	38265
GL174031.1	75193	78038	77132, 77148, 75249, 77117	43, 64, 15, 37	27.63344, 45.77712, 4.95496, 25.95049	8.0158, 10.20865, 3.03129, 8.63242	24.1824, 4	3	NA	40569
GL174034.1	6604	7621	7078, 7516, 6666	9, 12, 5	5.85972, 7.15851, 4.25472	4.30221, 4.61876, 3.65711	3.45931, 4	3	NA	20882
GL174034.1	18571	18995	18877, 18859, 18907	37, 37, 18	31.62627, 27.77596, 16.46425	11.35082, 9.46256, 8.87284	28.08253,	3	NA	9508
GL174034.1	30429	31552	30617, 30553, 31149	16, 26, 14	13.19919, 20.06243, 12.74306	7.42362, 8.62669, 7.63499	10.2543, 1	3	NA	1858
GL174034.1	38335	38881	38576, 38570, 38561	10, 14, 8	6.64819, 8.91697, 7.15322	4.64647, 5.29727, 5.25003	4.15239, 6	3	NA	2152
GL174034.1	49331	50177	49809, 49882, 49412	17, 29, 7	10.51318, 18.91542, 4.34038	5.60716, 7.28499, 3.57607	7.72171, 1	3	NA	13148
GL174034.1	67276	67550	67450, 67443, 67457	23, 28, 13	14.83288, 16.98893, 8.42084	6.62439, 6.60491, 5.19742	11.80873,	3	NA	31093
GL174034.1	68461	69614	68876, 68339, 69111, 68301, 69348	15, 12, 16, 9, 12	6.90368, 3.85881, 5.85494, 4.48903, 6.41722	3.99863, 2.75253, 3.35723, 3.43106, 4.17554	4.38815, 1	3	NA	32278
GL174034.1	72636	73354	73215, 72890, 73159	18, 20, 10	11.50771, 11.71364, 6.59592	5.95056, 5.64873, 4.61338	8.65247, 9	3	NA	36453
GL174035.1	16199	17223	17083, 16525, 16492	8, 14, 3	8.10827, 13.82362, 3.47371	5.80497, 8.29035, 3.03189	5.48761, 1	3	NA	6026
GL174035.1	22424	22828	22680, 22656, 22635	9, 10, 6	7.71701, 6.74189, 6.4014	5.47767, 4.70583, 4.8799	5.12244, 4	3	NA	421
GL174035.1	33455	33883	33482, 33502, 33481	25, 44, 18	29.70081, 53.97484, 24.00763	14.5855, 21.01249, 13.17779	26.20033,	3	NA	3719
GL174035.1	48584	49390	48939, 49151, 48608	6, 10, 4	5.96715, 9.39128, 4.67325	4.64927, 6.35644, 3.78987	3.52158, 6	3	npbwr2	7859
GL174035.1	57991	58615	58222, 58020, 58425	10, 24, 10	10.13706, 28.6314, 12.90749	6.7935, 14.28819, 8.21675	7.36489, 2	3	oprl1	5379
GL174036.1	76428	77039	77004, 76677, 76691	8, 17, 5	5.61242, 13.17414, 3.97425	4.2841, 7.15298, 3.48505	3.23505, 1	3	NA	9439
GL174037.1	39	736	381, 418, 359	5, 20, 5	4.77263, 19.93044, 5.69847	3.9578, 10.38414, 4.4331	2.51418, 1	3	mapk7	5064
GL174037.1	1895	2377	2176, 2073, 2086	14, 6, 6	15.84929, 3.76939, 6.87953	9.46495, 3.29482, 5.11627	12.77923,	3	mapk7	3423
GL174037.1	55713	56118	55990, 56015, 56010	12, 19, 8	13.8483, 21.4153, 10.1465	8.63436, 11.55717, 6.82176	10.86694,	3	rnf112	8000
GL174038.1	28178	28733	28520, 28492, 28634	8, 21, 7	8.42257, 24.35402, 8.69489	5.97763, 12.71289, 6.06379	5.76336, 2	3	NA	9682
GL174038.1	45070	45395	45366, 45340, 45349	24, 47, 14	27.83747, 54.22843, 16.91966	13.82444, 19.74302, 10.04443	24.38123,	3	NA	9885
GL174038.1	76139	76994	76629, 76747, 76723	29, 35, 27	32.4469, 39.75801, 34.50341	14.76385, 16.6773, 16.69193	28.88534,	3	NA	15264
GL174039.1	292	1680	992, 1436, 1210	13, 16, 9	10.8459, 14.57865, 9.58224	6.69156, 8.26691, 6.56676	8.03457, 1	3	NA	NA
GL174039.1	27231	28242	27612, 27305, 27676	9, 11, 8	7.17299, 9.52738, 7.30639	5.13877, 6.27759, 5.34182	4.61317, 6	3	NA	NA
GL174039.1	54235	54880	54544, 54543, 54543	17, 23, 13	14.49297, 19.04539, 12.70856	7.94987, 8.9325, 7.83036	11.48333,	3	NA	NA
GL174039.1	58792	60114	59157, 59452, 59682, 59968	15, 13, 4, 4	11.39084, 8.6375, 3.32139, 3.39155	6.56764, 5.3289, 3.0752, 3.11756	8.54179, 6	3	NA	NA
GL174040.1	3983	4473	4150, 4202, 4195	17, 24, 7	17.19112, 23.20006, 6.75301	9.58879, 11.05777, 5.08141	14.0641, 2	3	NA	13280
GL174041.1	29	768	134, 253, 456	30, 32, 25	30.6894, 34.33575, 30.81762	13.33685, 14.70709, 15.23219	27.16666,	3	NA	NA
GL174042.1	21	996	590, 693, 662	49, 53, 31	47.64442, 49.07512, 30.52792	15.28875, 14.5253, 12.86453	43.82532,	3	NA	29096
GL174043.1	10934	11256	10974, 11192, 11150	9, 9, 5	8.35434, 6.26311, 5.69847	5.86444, 4.56059, 4.4331	5.71584, 3	3	cacna1d	20832
GL174043.1	30654	31519	31456, 31369, 31463	7, 8, 5	6.84137, 6.85668, 5.80393	5.13151, 5.06969, 4.48186	4.33107, 4	3	cacna1d	569
GL174043.1	54711	55095	55093, 55035, 55049	7, 14, 12	6.94335, 11.99537, 14.49372	5.18876, 7.17543, 8.97057	4.42528, 9	3	cacna1d	0
GL174043.1	68771	69957	69019, 69085, 68877, 69583	12, 16, 3, 4	12.74986, 14.04087, 3.14943, 4.30163	8.02869, 7.93805, 2.87713, 3.61556	9.82533, 1	3	cacna1d	2365
GL174044.1	15569	15915	15721, 15814, 15722	6, 8, 4	5.96715, 6.34359, 4.38152	4.64927, 4.75053, 3.65448	3.52158, 3	3	znf585b	0
GL174046.1	11566	12796	11627, 11636, 12003	25, 41, 19	26.77224, 44.59624, 21.95763	12.84972, 16.91942, 11.8707	23.34359,	3	NA	NA
GL174047.1	27737	28043	28012, 28011, 27989	10, 12, 7	11.06011, 11.86108, 8.69489	7.306, 7.51216, 6.06379	8.22712, 9	3	NA	NA
GL174047.1	47156	48174	47561, 47263, 47959	17, 18, 12	16.90454, 15.53678, 11.76526	9.41624, 8.30996, 7.45528	13.78827,	3	NA	NA
GL174048.1	1034	2066	1252, 1357, 1169, 1556	13, 18, 8, 9	8.909, 9.41736, 5.74302, 6.72209	5.49454, 4.83383, 4.36815, 4.8535	6.2222, 6.8	3	NA	42315
GL174048.1	3472	3786	3585, 3703, 3677	9, 20, 13	4.71777, 9.63246, 9.82499	3.57477, 4.62146, 6.05869	2.48153, 7	3	NA	40595
GL174048.1	21195	21798	21260, 21603, 21337, 21616	14, 17, 17, 9	4.7657, 4.90589, 9.75925, 3.68439	3.03192, 2.84605, 5.19207, 2.93434	2.51418, 2	3	NA	22583
GL174048.1	21329	21972	21925, 21603, 21337, 21616	16, 17, 17, 9	6.40328, 4.90589, 9.75925, 3.68439	3.61859, 2.84605, 5.19207, 2.93434	3.92779, 2	3	NA	22409
GL174048.1	27753	28191	27923, 27858, 28015	10, 14, 10	5.55039, 7.30217, 7.77215	3.95657, 4.36489, 5.358	3.1808, 4.8	3	NA	16190
GL174048.1	34526	35825	34745, 35417, 35106, 35500	18, 22, 8, 10	11.56563, 12.63794, 5.2088, 6.94959	5.98278, 5.72734, 4.02307, 4.83748	8.70729, 9	3	NA	8556
GL174048.1	34741	35825	35519, 35417, 35106, 35500	10, 22, 8, 10	4.7315, 12.63794, 5.2088, 6.94959	3.45436, 5.72734, 4.02307, 4.83748	2.49377, 9	3	NA	8556
GL174048.1	51343	51698	51472, 51504, 51416	17, 24, 15	18.64535, 23.27012, 17.07266	10.45058, 11.09959, 9.97618	15.46286,	3	NA	0
GL174052.1	33	1009	628, 666, 463	12, 14, 16	13.22036, 13.0902, 18.88025	8.29296, 7.84698, 10.798	10.27237,	3	NA	4624
GL174052.1	14685	15125	15061, 14950, 15007	11, 12, 9	10.9046, 10.63474, 10.72164	7.10903, 6.77054, 7.15493	8.08987, 7	3	NA	7191
GL174052.1	41531	42144	41884, 41978, 41746	28, 35, 12	30.19897, 35.9563, 14.76955	13.80434, 14.45276, 9.10943	26.68663,	3	znf484	4462
GL174053.1	35398	35985	35579, 35527, 35563	14, 11, 9	11.66068, 6.6514, 8.34444	6.969, 4.48046, 5.85853	8.79778, 4	3	NA	NA
GL174053.1	46255	49066	46832, 48071, 48181	18, 22, 12	16.5065, 16.5076, 8.93956	8.89851, 7.77175, 5.71702	13.41011,	3	NA	NA
GL174053.1	52390	52716	52569, 52485, 52563	12, 18, 12	10.58505, 14.44747, 11.02785	6.73997, 7.65402, 7.01117	7.78888, 1	3	NA	NA
GL174055.1	29345	30823	29783, 30533, 30120	17, 22, 8	18.21101, 20.88093, 10.0913	10.19603, 10.32883, 6.79758	15.04611,	3	NA	3559
GL174056.1	49787	50708	50389, 50361, 50099	9, 7, 5	9.72082, 5.13485, 5.91431	6.64182, 4.09383, 4.53172	6.97155, 2	3	NA	NA
GL174056.1	60166	60988	60537, 60536, 60561	9, 17, 10	9.30591, 17.87191, 12.71002	6.41439, 9.99549, 8.12734	6.59443, 1	3	NA	NA
GL174057.1	66649	67405	66944, 67168, 66801	5, 5, 6	4.47183, 3.35187, 6.168	3.78598, 3.08382, 4.75778	2.26935, 1	3	NA	NA
GL174058.1	36792	37487	36906, 37272, 37276	13, 14, 5	11.98086, 11.76301, 4.77515	7.39024, 7.03214, 3.9592	9.10256, 9	3	emx2	34395
GL174059.1	44786	45528	45028, 44884, 44874	6, 7, 9	4.83561, 4.99936, 9.70054	3.98453, 4.0065, 6.63089	2.52606, 2	3	a2ml1	0
GL174059.1	71669	72191	71924, 71868, 71835	17, 30, 8	21.34551, 38.44953, 10.1465	11.95527, 17.91361, 6.82176	18.07159,	3	a2ml1	6358
GL174062.1	36840	37645	37271, 37113, 37483	9, 7, 4	9.24298, 5.97725, 4.38152	6.3792, 4.62287, 3.65448	6.53474, 3	3	NA	NA
GL174062.1	70353	71793	71070, 70970, 71453	25, 27, 15	29.22944, 28.79687, 16.75372	14.30936, 13.33976, 9.79627	25.74065,	3	NA	NA
GL174063.1	3985	4501	4245, 4094, 4215	6, 11, 9	5.96715, 10.60895, 11.64359	4.64927, 6.9343, 7.57973	3.52158, 7	3	stat5b	8949
GL174063.1	24367	25204	24454, 24810, 24900	14, 13, 11	13.26657, 9.96603, 12.78973	7.95422, 6.14603, 8.15619	10.31735,	3	stat5b	0
GL174064.1	244	988	827, 656, 825	14, 12, 5	16.7649, 11.04188, 5.91431	9.96273, 7.01971, 4.53172	13.6543, 8	3	plec	8414
GL174064.1	32017	33108	32662, 32486, 32568	11, 13, 6	10.49148, 12.86565, 5.99481	6.86423, 7.92422, 4.6644	7.70044, 1	3	plec	0
GL174064.1	41168	41553	41480, 41480, 41480	5, 11, 11	4.40263, 9.13706, 12.2694	3.74533, 6.0355, 7.88012	2.21004, 6	3	plec	0
GL174064.1	53907	54362	54248, 54055, 54094	8, 5, 6	8.10827, 3.78182, 7.29403	5.80497, 3.36365, 5.30581	5.48761, 1	3	plec	0
GL174065.1	5236	5961	5691, 5555, 5725	11, 9, 14	10.3799, 7.17446, 15.06595	6.79736, 5.1397, 9.02131	7.59574, 4	3	NA	NA
GL174065.1	17579	17883	17767, 17791, 17743	7, 8, 4	7.15806, 7.07159, 4.67325	5.30717, 5.20073, 3.78987	4.61046, 4	3	NA	NA
GL174068.1	17980	18395	18249, 18246, 18033	9, 9, 6	8.45482, 6.49199, 5.95321	5.92419, 4.70689, 4.64162	5.79307, 4	3	NA	0
GL174068.1	42715	43221	42806, 42839, 42956	6, 8, 5	5.31708, 6.44699, 4.36653	4.2777, 4.8155, 3.72397	2.96727, 4	3	NA	520
GL174068.1	88842	89591	89058, 89427, 89262	12, 8, 8	13.8483, 7.07159, 9.76268	8.63436, 5.20073, 6.64964	10.86694,	3	GGT6	6198
GL174069.1	11936	12386	12112, 12115, 11993	26, 26, 6	31.36507, 28.49224, 7.29403	15.21991, 13.52193, 5.30581	27.82689,	3	NA	34499
GL174069.1	61686	62129	61828, 61958, 61975	8, 10, 4	7.1122, 7.14103, 4.64325	5.22529, 4.95885, 3.77643	4.57946, 4	3	NA	0
GL174070.1	58396	62329	60236, 59204, 59255	38, 56, 22	22.04434, 35.95586, 17.08167	6.82683, 8.50822, 8.09598	18.74903,	3	stx3	22203
GL174070.1	64403	65159	64772, 64724, 64753	37, 51, 34	21.74926, 28.06628, 25.89012	6.89035, 6.83225, 9.37855	18.46325,	3	stx3	28210
GL174070.1	66423	67408	66887, 66814, 66824	43, 53, 36	30.76016, 33.22948, 30.03536	9.26401, 8.14327, 10.85707	27.23566,	3	stx3	30230
GL174070.1	70049	70822	70396, 70407, 70324	28, 50, 25	22.30141, 40.17756, 23.74203	9.25696, 11.43798, 11.04074	18.99904,	3	stx3	33856
GL174071.1	41802	42521	41857, 41867, 41834	7, 12, 4	7.1696, 11.86108, 4.67325	5.31345, 7.51216, 3.78987	4.61046, 9	3	sult1a1	9805
GL174071.1	49287	49950	49540, 49456, 49455	12, 20, 6	12.3187, 20.00323, 6.99807	7.78073, 10.42807, 5.17195	9.4231, 16	3	sult1a1	2376
GL174071.1	64983	65248	65168, 65065, 65156	7, 10, 9	7.1696, 9.39128, 11.30431	5.31345, 6.35644, 7.42891	4.61046, 6	3	sult1a1	0
GL174072.1	119	1142	426, 646, 308	11, 8, 7	12.43693, 7.07159, 8.69489	7.97018, 5.20073, 6.06379	9.52692, 4	3	mrps33	3439
GL174072.1	7578	7942	7712, 7797, 7712	6, 6, 5	5.09949, 4.93312, 5.06966	4.14694, 4.04501, 4.11933	2.76939, 2	3	mrps33	995
GL174072.1	16692	17141	17091, 17084, 16933	19, 18, 15	23.41291, 15.90393, 16.15968	12.68879, 8.53251, 9.45524	20.07675,	3	braf	0
GL174072.1	37616	38589	37643, 37632, 37632	13, 20, 12	13.79066, 21.17915, 14.86478	8.46641, 11.13464, 9.15668	10.8171, 1	3	braf	0
GL174072.1	46073	46640	46450, 46600, 46338	7, 10, 6	4.7913, 6.90788, 4.64903	3.87149, 4.81103, 3.8674	2.51418, 4	3	braf	110
GL174072.1	47914	48328	47971, 47990, 47967	22, 22, 21	21.76329, 20.30721, 22.59949	10.85936, 9.98502, 11.67952	18.47656,	3	braf	1951
GL174072.1	61360	61595	61414, 61425, 61462	12, 15, 11	12.52976, 14.39783, 12.48556	7.90277, 8.40758, 7.99611	9.6155, 11	3	braf	15397
GL174073.1	1756	3154	1966, 1984, 1895, 2648	12, 20, 5, 7	12.74986, 18.84531, 5.31857, 7.84208	8.02869, 9.72843, 4.24819, 5.66426	9.82533, 1	3	NA	NA
GL174073.1	1759	3154	3099, 1984, 1895, 2648	11, 20, 5, 7	10.60582, 18.84531, 5.31857, 7.84208	6.93244, 9.72843, 4.24819, 5.66426	7.80831, 1	3	NA	NA
GL174073.1	9540	10043	9830, 9741, 9962	10, 13, 9	9.44155, 11.61168, 10.01275	6.38646, 7.16421, 6.79683	6.72156, 8	3	NA	NA
GL174073.1	36545	37545	37306, 37391, 37289	11, 16, 7	12.43693, 16.51912, 8.69489	7.97018, 9.44018, 6.06379	9.52692, 1	3	NA	NA

GL174073.1	49782	50419	50050, 50189, 49861	12, 12, 13	6.90943, 5.75901, 10.38485	4.46861, 3.7923, 6.40571	4.39383, 3	3	NA	NA
GL174073.1	53837	54645	54226, 54229, 54230	39, 40, 25	35.13447, 32.92604, 23.97791	12.49384, 11.0538, 11.18059	31.51923,	3	NA	NA
GL174073.1	55755	57006	56327, 56303, 56320	18, 15, 8	12.7397, 8.60419, 5.84174	6.64898, 4.92445, 4.43151	9.81664, 6	3	NA	NA
GL174073.1	63662	64529	64016, 64107, 63839	7, 10, 11	5.39768, 7.4213, 11.4314	4.26169, 5.13639, 7.41405	3.04019, 4	3	NA	NA
GL174074.1	12709	13330	13107, 13086, 12985	8, 10, 4	5.55847, 6.82398, 3.82084	4.24931, 4.75785, 3.36473	3.18478, 4	3	NA	0
GL174074.1	45563	46064	45852, 45771, 46013	18, 26, 11	17.38211, 27.56619, 12.06254	9.43026, 12.96862, 7.76744	14.24778,	3	NA	0
GL174074.1	56767	57280	57107, 56920, 56982	7, 12, 5	6.42049, 9.70336, 4.74091	4.8888, 6.19346, 3.94006	3.94161, 7	3	NA	4078
GL174074.1	83269	83767	83605, 83696, 83642	10, 7, 8	10.53228, 4.99936, 7.95096	7.01712, 4.0065, 5.71659	7.73927, 2	3	NA	30580
GL174074.1	95912	96695	96032, 95978, 96011	8, 8, 4	8.42257, 7.07159, 4.67325	5.97763, 5.20073, 3.78987	5.76336, 4	3	NA	43223
GL174075.1	2735	3241	3034, 2989, 2924	13, 27, 13	14.79381, 29.6442, 15.0131	9.02996, 13.84552, 9.14923	11.77132,	3	alpl	11467
GL174075.1	40867	41210	40986, 40963, 40983	14, 11, 20	14.91094, 10.60895, 25.47128	8.9318, 6.9343, 13.59302	11.88377,	3	alpl	14713
GL174077.1	36133	36389	36223, 36242, 36214	7, 7, 3	6.99564, 5.97725, 3.30894	5.21786, 4.62287, 2.9554	4.47307, 3	3	OTOG	0
GL174077.1	66077	66334	66151, 66257, 66212	6, 6, 6	5.81608, 4.73925, 7.29403	4.56563, 3.92426, 5.30581	3.4176, 2.5	3	OTOG	0
GL174078.1	13442	13801	13757, 13758, 13773	32, 35, 24	36.24469, 37.49287, 30.91999	15.83805, 15.34496, 15.58778	32.60691,	3	NA	NA
GL174079.1	7743	8409	8074, 7855, 7902	13, 20, 8	15.29169, 22.87459, 10.1465	9.29854, 12.13503, 6.82176	12.24396,	3	NA	14756
GL174079.1	51980	53041	52463, 52406, 52889	7, 9, 5	5.36706, 7.21043, 4.77515	4.24225, 5.1623, 3.9592	3.01389, 4	3	NA	18190
GL174079.1	66148	66555	66333, 66356, 66481	7, 14, 13	5.39768, 9.48413, 14.39632	4.26169, 5.63643, 8.81006	3.04019, 6	3	NA	32358
GL174079.1	67854	68441	68265, 68190, 68090	11, 21, 7	10.06049, 17.60201, 6.83761	6.60432, 8.68374, 5.12939	7.29334, 1	3	NA	34064
GL174079.1	68904	69486	69252, 69343, 69335	11, 15, 5	10.1122, 11.26316, 4.64121	6.63573, 6.49004, 3.88372	7.34229, 8	3	NA	35114
GL174081.1	58222	61776	59571, 60328, 59217, 60515	19, 24, 17, 11	10.24994, 10.02269, 12.44499, 6.55129	5.08573, 4.25675, 6.71914, 4.4187	7.47307, 7	3	NA	3916
GL174081.1	61522	64618	64386, 60328, 62392, 64372, 63010	28, 24, 32, 47, 12	16.1541, 10.02269, 16.81933, 28.40594, 7.95439	6.22991, 4.25675, 5.82158, 7.55892, 5.10531	13.07306,	3	NA	7216
GL174081.1	68310	69298	68819, 68779, 68800	41, 33, 26	40.31932, 26.34691, 31.48611	14.52291, 9.89374, 15.29034	36.61193,	3	NA	14004
GL174082.1	24715	25360	25339, 25333, 25281	18, 29, 8	17.93309, 31.17793, 8.6841	9.76366, 14.00832, 6.11715	14.77832,	3	fam46c	0
GL174083.1	2455	3034	2774, 2787, 2853	21, 14, 9	18.91592, 10.32673, 8.88966	9.46486, 6.14815, 6.17838	15.72399,	3	znf687	5368
GL174084.1	9455	10028	9760, 9760, 9548	10, 13, 8	9.89241, 10.00327, 7.85773	6.65218, 6.16911, 5.66361	7.13431, 7	3	NA	NA
GL174084.1	13210	13937	13807, 13784, 13768	5, 20, 7	4.40263, 18.59503, 6.75301	3.74533, 9.57724, 5.08141	2.21004, 1	3	NA	NA
GL174084.1	22800	23270	23259, 23257, 23055	10, 14, 8	10.39648, 13.14791, 10.0913	6.94096, 7.88211, 6.79758	7.61003, 1	3	NA	NA
GL174084.1	24757	25153	24999, 25027, 25078	8, 6, 4	8.05102, 4.62266, 4.64325	5.77295, 3.8507, 3.77643	5.43473, 2	3	NA	NA
GL174084.1	37469	38006	37649, 37843, 37510	8, 11, 6	8.42257, 10.60895, 7.29403	5.97763, 6.9343, 5.30581	5.76336, 7	3	NA	NA
GL174084.1	48833	49161	49115, 49060, 49126	6, 11, 7	5.90927, 8.41104, 7.78342	4.61743, 5.58113, 5.63488	3.50164, 5	3	NA	NA
GL174085.1	24789	25995	25620, 25162, 25522	16, 14, 8	19.29793, 14.15854, 10.1465	11.02611, 8.49024, 6.82176	16.0927, 1	3	NA	NA
GL174085.1	86319	86927	86631, 86548, 86630	9, 20, 13	9.72082, 22.87459, 18.01082	6.64182, 12.13503, 10.61163	6.97155, 1	3	NA	NA
GL174086.1	40410	41018	40942, 40988, 40971	15, 18, 11	16.90423, 19.97713, 13.28509	9.88146, 10.97931, 8.40871	13.78827,	3	gba2	3986
GL174086.1	93153	93517	93378, 93406, 93343	6, 8, 6	5.96715, 7.07159, 7.29403	4.64927, 5.20073, 5.30581	3.52158, 4	3	rgp1	3434
GL174087.1	19389	20080	19450, 19700, 19501	10, 21, 10	8.98596, 18.71315, 10.1418	6.11182, 9.34355, 6.79622	6.29482, 1	3	arpc1a	0
GL174087.1	43676	44298	44060, 43959, 44163	14, 22, 11	15.31941, 22.3038, 12.19945	9.1665, 11.18464, 7.8422	12.27033,	3	NA	1053
GL174088.1	41	326	250, 243, 223	23, 32, 15	26.78552, 35.31675, 19.17716	13.52881, 15.28753, 11.09709	23.3553, 3	3	NA	NA
GL174089.1	49	620	277, 274, 478	41, 36, 22	40.47861, 34.84241, 21.87851	14.6098, 13.43026, 10.92871	36.76861,	3	rorc	25704
GL174089.1	4135	4539	4326, 4319, 4249	28, 21, 15	23.7094, 15.8658, 12.90942	10.02773, 7.67695, 7.49764	20.365, 12	3	rorc	21785
GL174090.1	961	1771	1345, 1350, 1069	12, 31, 11	13.13892, 35.32126, 12.78973	8.24771, 15.69126, 8.15619	10.1962, 3	3	NA	NA
GL174093.1	41619	42717	42407, 42227, 41877	14, 16, 9	12.91867, 13.21822, 9.41119	7.74237, 7.43523, 6.47287	9.98624, 1	3	rpl10	18676
GL174093.1	67186	67531	67270, 67286, 67288	17, 16, 16	21.34551, 17.16856, 23.11042	11.95527, 9.82359, 12.88555	18.07159,	3	rpl10	44243
GL174094.1	3504	3976	3546, 3550, 3596	13, 19, 11	12.74759, 20.73582, 14.03502	7.85373, 11.15889, 8.77076	9.82421, 1	3	mea1	279
GL174094.1	66058	67146	66660, 66658, 66650	58, 79, 39	61.44627, 81.41973, 48.81665	18.73028, 19.75356, 20.28394	57.44242,	3	mea1	62833
GL174096.1	28339	28655	28610, 28638, 28607	7, 9, 6	6.09019, 6.43335, 7.29403	4.69155, 4.66944, 5.30581	3.63504, 4	3	micalcl	29513
GL174096.1	36432	37169	36821, 36461, 36600	10, 14, 17	11.06011, 14.45897, 22.7655	7.306, 8.66788, 12.67848	8.22712, 1	3	micalcl	20999
GL174097.1	76	953	434, 516, 614	16, 19, 6	18.96503, 18.34606, 6.6567	10.84464, 9.72578, 5.00845	15.771, 15	3	NA	NA
GL174097.1	9178	9664	9447, 9449, 9494	19, 27, 12	15.8836, 22.12514, 10.93726	8.23864, 9.46127, 6.95592	12.81126,	3	NA	NA
GL174097.1	13138	13768	13557, 13417, 13343	17, 23, 12	13.52314, 19.44693, 11.30996	7.36261, 9.16498, 7.18231	10.56195,	3	NA	NA
GL174097.1	62354	62640	62533, 62447, 62593	9, 6, 6	9.36986, 4.86158, 6.35308	6.44997, 4.00069, 4.85498	6.65425, 2	3	NA	NA
GL174098.1	37936	38663	38584, 38229, 38492	12, 11, 8	11.04522, 10.3713, 8.9249	7.02174, 6.79219, 6.24211	8.22317, 7	3	npr1	3704
GL174098.1	48533	49036	48922, 48892, 48850	14, 14, 6	13.8308, 10.91946, 7.25145	8.29466, 6.51197, 5.287	10.85567,	3	ints3	1797
GL174099.1	11	491	286, 285, 275	53, 71, 27	65.48821, 84.39739, 31.964	23.3182, 24.75657, 15.22188	61.4391, 8	3	abhd1	2929
GL174099.1	19475	20045	19613, 19701, 19897	11, 16, 8	10.66406, 16.67093, 10.1465	6.96705, 9.53036, 6.82176	7.86396, 1	3	cgref1	3616
GL174099.1	62982	64012	63026, 63607, 63596	11, 10, 4	11.15818, 9.27513, 4.67325	7.25692, 6.2868, 3.78987	8.32008, 6	3	emilin1	5769
GL174099.1	65453	66105	65659, 66056, 66027	6, 11, 5	5.24248, 10.48162, 5.9506	4.2332, 6.85833, 4.54784	2.89962, 7	3	emilin1	8240
GL174101.1	17780	18190	18172, 17968, 18153	12, 9, 8	12.67544, 7.51164, 8.56955	7.98627, 5.35055, 6.05652	9.75458, 5	3	NA	35131
GL174101.1	27545	28190	27723, 27967, 27817	6, 9, 5	5.96715, 8.21099, 5.9506	4.64927, 5.77858, 4.54784	3.52158, 5	3	NA	25131
GL174102.1	65462	65758	65626, 65672, 65629	10, 13, 7	9.13239, 12.7389, 8.49722	6.2007, 7.84853, 5.97582	6.43114, 9	3	mbd5	21650
GL174104.1	22023	22515	22199, 22184, 22062	4, 16, 5	3.74195, 17.16856, 5.9506	3.32091, 9.82359, 4.54784	1.6222, 14	3	birc7	3028
GL174105.1	21256	21944	21635, 21729, 21720	14, 13, 6	15.84929, 13.14514, 7.05929	9.46495, 8.09002, 5.20024	12.77923,	3	5S_rRNA	156
GL174105.1	29202	29694	29416, 29590, 29447	4, 12, 5	3.74195, 11.86108, 5.9506	3.32091, 7.51216, 4.54784	1.6222, 9.1	3	5S_rRNA	6980
GL174106.1	19554	19954	19842, 19848, 19642	9, 8, 6	7.6342, 5.28564, 5.49907	5.42655, 4.07288, 4.38471	5.04509, 3	3	scrib	0
GL174106.1	56171	56903	56409, 56418, 56381	12, 28, 17	12.3187, 29.94002, 19.81591	7.78073, 13.64998, 11.12108	9.4231, 26	3	scrib	961
GL174108.1	35224	35876	35801, 35501, 35437	6, 5, 5	4.89903, 3.4998, 4.51488	4.02393, 3.18133, 3.81106	2.58474, 1	3	dok1	22556
GL174108.1	36145	37191	36375, 36459, 36375	7, 10, 10	5.68903, 8.24345, 10.25108	4.44496, 5.65367, 6.85862	3.3035, 5.7	3	dok1	23477
GL174108.1	38764	40367	39788, 39499, 40175	9, 13, 5	6.73165, 10.39132, 3.76962	4.85958, 6.40973, 3.35587	4.22986, 7	3	dok1	26096
GL174108.1	44940	46105	45268, 45617, 45432	13, 10, 10	12.43975, 7.75815, 9.93124	7.66875, 5.34918, 6.67475	9.5289, 5.2	3	dok1	32272
GL174109.1	19668	20323	19747, 19763, 19736, 20202	7, 8, 9, 6	6.50909, 5.43464, 8.79325, 5.43047	4.94073, 4.16933, 6.12267, 4.34463	4.02371, 3	3	casp7	17002
GL174109.1	30375	31038	30942, 30974, 30824	9, 25, 8	9.36986, 26.60415, 8.1956	6.44997, 12.74915, 5.85348	6.65425, 2	3	casp7	6287
GL174109.1	49033	49465	49145, 49321, 49121	11, 12, 15	8.47312, 9.0625, 15.48721	5.62011, 5.7937, 9.06094	5.81094, 6	3	casp7	5212
GL174109.1	51834	52367	51973, 51972, 51973	14, 19, 14	11.31081, 16.8936, 14.34766	6.75314, 8.84622, 8.60225	8.46526, 1	3	casp7	8013
GL174111.1	48708	49381	49059, 48992, 48877	16, 20, 13	19.64758, 21.43553, 15.34872	11.21376, 11.28761, 9.3288	16.43074,	3	NA	NA
GL174112.1	5004	5725	5127, 5207, 5302	6, 7, 5	5.96715, 5.97725, 5.9506	4.64927, 4.62287, 4.54784	3.52158, 3	3	NA	39676
GL174114.1	48	817	438, 707, 707	16, 32, 25	18.44349, 33.96274, 33.54047	10.55511, 14.48708, 16.74961	15.26881,	3	ifnar2.2	5969
GL174114.1	31609	32032	31677, 31683, 31676	18, 33, 12	17.79149, 32.3411, 12.62857	9.67812, 13.15491, 7.95947	14.64268,	3	ifnar2.2	16193
GL174115.1	530	1019	978, 949, 958	7, 12, 6	6.42049, 9.99443, 7.29403	4.8888, 6.37457, 5.30581	3.94161, 7	3	PKP2	2176
GL174115.1	38645	39620	39150, 39182, 39184	16, 23, 14	14.29285, 19.34487, 14.28646	8.0922, 9.10573, 8.56608	11.29159,	3	PKP2	1939
GL174115.1	85787	86157	85980, 85980, 85980	23, 21, 9	24.14755, 20.96503, 9.24728	11.96297, 10.69835, 6.38161	20.78974,	3	stk38l	0
GL174118.1	117	593	373, 411, 429	23, 23, 7	25.82433, 20.4778, 7.29952	12.96322, 9.76882, 5.38357	22.41977,	3	tkt	12021
GL174118.1	49940	50637	50503, 50150, 50435	6, 14, 10	5.09949, 10.68957, 11.6791	4.14694, 6.3706, 7.62925	2.76939, 8	3	dcp1a	0
GL174118.1	60479	60952	60886, 60851, 60891	18, 27, 11	18.52782, 27.67569, 12.48556	10.1215, 12.67118, 7.99611	15.34982,	3	dcp1a	0
GL174119.1	12366	12939	12380, 12373, 12371	56, 79, 68	62.40553, 87.57391, 84.73742	20.12867, 22.60688, 26.4847	58.39193,	3	NA	NA
GL174119.1	45788	46319	46047, 46047, 46070	11, 13, 5	11.29029, 10.11663, 5.50079	7.3332, 6.23937, 4.33868	8.44541, 7	3	NA	NA
GL174119.1	53315	54137	53621, 53733, 53834	17, 18, 11	19.01263, 17.45593, 12.86886	10.66355, 9.47501, 8.19722	15.81687,	3	NA	NA
GL174120.1	8860	9905	9208, 8934, 9683	14, 19, 14	11.84416, 13.95947, 15.27783	7.08219, 7.10579, 9.14278	8.97236, 1	3	NA	NA
GL174124.1	5783	6579	6303, 6100, 6011	15, 23, 10	13.41505, 21.76909, 9.35556	7.80796, 10.53606, 6.33507	10.45899,	3	mvp	0
GL174124.1	28890	29810	29184, 29169, 29233	25, 31, 28	23.44347, 29.40833, 30.48566	10.86418, 12.21997, 13.97527	20.10629,	3	kif22	674
GL174124.1	50188	50749	50252, 50534, 50443	7, 9, 4	6.99564, 7.28344, 4.50795	5.21786, 5.20811, 3.71446	4.47307, 4	3	maz	10165
GL174125.1	200	920	519, 587, 720	15, 19, 11	10.28452, 9.65329, 8.20847	5.90105, 4.78733, 5.45386	7.50506, 7	3	NA	NA
GL174125.1	3243	4005	3553, 3721, 3311	16, 36, 12	9.11594, 23.00426, 8.00253	5.02635, 7.58776, 5.13501	6.41768, 1	3	NA	NA
GL174125.1	4402	4787	4601, 4566, 4702	11, 26, 10	4.71165, 13.00372, 5.71887	3.32008, 5.20937, 4.06148	2.47668, 1	3	NA	NA
GL174125.1	54859	57948	57652, 57653, 57652	138, 218, 89	128.92912, 205.96342, 86.20353	18.57212, 20.23519, 18.19313	124.32207	3	NA	NA
GL174126.1	44	1048	235, 264, 272	26, 45, 14	26.52554, 48.60518, 14.92875	12.34687, 17.45266, 8.94211	23.10248,	3	SMPD1	5410
GL174126.1	41255	41842	41804, 41790, 41813	28, 44, 24	33.57988, 54.11761, 32.9096	15.81078, 21.09641, 16.65857	29.99477,	3	NA	17815
GL174127.1	181	857	505, 637, 674	32, 31, 22	33.65023, 28.7313, 24.85419	14.30313, 11.83495, 12.70716	30.06406,	3	NA	2219
GL174127.1	13333	13981	13734, 13614, 13849	9, 14, 11	8.20841, 14.22787, 13.28509	5.77703, 8.53137, 8.40871	5.58112, 1	3	NA	9444
GL174127.1	19924	20806	20339, 20315, 20617	43, 36, 21	45.35175, 36.57807, 24.6582	16.48938, 14.41064, 12.88878	41.56791,	3	NA	16035
GL174127.1	42213	43433	42707, 42748, 42580	43, 61, 30	41.60618, 63.66975, 34.12618	14.44148, 18.5947, 15.37557	37.87802,	3	MGC146454	2041
GL174127.1	48231	49351	49122, 48734, 48776	63, 108, 54	65.42185, 119.4728, 63.57319	18.63837, 24.68167, 21.72503	61.37327,	3	MGC146454	0
GL174128.1	12436	13224	12773, 12812, 12851	14, 19, 10	16.7649, 21.4153, 13.18184	9.96273, 11.55717, 8.33771	13.6543, 1	3	NA	NA
GL174128.1	20455	21202	20979, 20960, 20974	17, 19, 16	16.97511, 16.51679, 19.55336	9.45879, 8.61887, 11.1635	13.85654,	3	NA	NA
GL174131.1	43138	43692	43481, 43439, 43181	10, 9, 6	9.89241, 7.10358, 5.11235	6.65218, 5.09508, 4.15475	7.13431, 4	3	NA	NA
GL174133.1	55698	56110	56093, 55993, 56084	12, 10, 10	13.22036, 8.66076, 13.18184	8.29296, 5.91256, 8.33771	10.27237,	3	NA	5750
GL174133.1	58271	58688	58462, 58323, 58419	10, 9, 5	11.06011, 8.21099, 5.9506	7.306, 5.77858, 4.54784	8.22712, 5	3	NA	8323
GL174135.1	8920	9582	9435, 8994, 9161	12, 14, 4	12.52976, 12.38816, 4.67325	7.90277, 7.41725, 3.78987	9.6155, 9.6	3	NA	NA
GL174135.1	20120	20907	20351, 20433, 20369	7, 7, 4	7.1696, 5.97725, 4.67325	5.31345, 4.62287, 3.78987	4.61046, 3	3	NA	NA
GL174135.1	40795	41105	40976, 41040, 41030	5, 16, 5	4.82177, 17.16856, 5.80393	3.98509, 9.82359, 4.48186	2.51418, 1	3	NA	NA
GL174135.1	65656	66127	65668, 65716, 65752	16, 27, 15	16.68158, 27.59392, 18.33148	9.53667, 12.62252, 10.66149	13.57813,	3	NA	NA
GL174135.1	74162	74493	74201, 74281, 74441	7, 10, 9	7.1696, 9.39128, 11.64359	5.31345, 6.35644, 7.57973	4.61046, 6	3	NA	NA
GL174136.1	9088	10241	9603, 9607, 9959	38, 49, 18	35.80542, 43.86554, 16.98427	13.21057, 13.4186, 9.18882	32.17625,	3	chrnb2	2
GL174136.1	39482	40111	39890, 40023, 39653	6, 12, 5	4.89903, 10.16897, 4.64121	4.02393, 6.4829, 3.88372	2.58474, 7	3	NA	6608
GL174136.1	40699	42248	41160, 41892, 41180	7, 12, 8	5.97522, 9.50574, 8.14528	4.62162, 6.07027, 5.82558	3.52869, 6	3	NA	4471
GL174136.1	47544	48142	47967, 48063, 48069	6, 17, 9	5.31708, 14.86656, 9.82249	4.2777, 8.17729, 6.69629	2.96727, 1	3	NA	0
GL174137.1	29942	30384	30330, 30331, 30328	9, 8, 8	9.72082, 7.07159, 8.4586	6.64182, 5.20073, 5.99709	6.97155, 4	3	rnf8	0
GL174138.1	43000	43695	43549, 43567, 43079	11, 12, 12	11.71035, 11.86108, 14.06375	7.57196, 7.51216, 8.74831	8.8442, 9.1	3	NA	NA
GL174138.1	58561	59430	58842, 58679, 59084	14, 28, 24	14.23954, 28.88701, 31.45068	8.53829, 13.02331, 15.87941	11.24094,	3	NA	NA
GL174139.1	68511	69461	68948, 68965, 69036	9, 17, 10	7.59348, 14.22254, 10.7802	5.40134, 7.78557, 7.15425	5.0069, 11	3	NA	NA
GL174140.1	4835	5834	5197, 5590, 4970	10, 15, 4	9.89241, 14.58692, 4.67325	6.65218, 8.52203, 3.78987	7.13431, 1	3	mfsd3	62558
GL174142.1	15407	16017	15751, 15738, 15751	13, 19, 9	11.71272, 16.67602, 9.19414	7.22621, 8.71486, 6.35176	8.84616, 1	3	5S_rRNA	24090
GL174142.1	20787	21457	21136, 21184, 21156	18, 22, 9	19.17347, 21.92663, 10.28145	10.50657, 10.95766, 6.93568	15.97256,	3	5S_rRNA	18650
GL174142.1	40117	41337	40577, 40464, 40684	123, 138, 78	65.63617, 66.66223, 40.0332	7.42362, 6.6073, 6.71445	61.58561,	3	5S_rRNA	0
GL174142.1	43851	46370	45428, 45407, 44353	213, 285, 170	143.53249, 192.72075, 123.8112	10.89834, 11.16655, 12.14673	138.8429,	3	5S_rRNA	0
GL174142.1	56469	58945	57382, 56976, 56961	260, 319, 181	241.31953, 294.67416, 171.70001	19.87136, 20.02892, 19.93541	236.17377	3	5S_rRNA	0
GL174143.1	8792	9430	9180, 9102, 9277	7, 8, 4	7.1696, 7.07159, 4.67325	5.31345, 5.20073, 3.78987	4.61046, 4	3	nap1l4	8791
GL174143.1	15521	16440	16157, 16237, 15556	10, 13, 9	6.67494, 8.72347, 9.35579	4.66341, 5.38125, 6.44216	4.17798, 6	3	nap1l4	1781
GL174143.1	20339	20849	20664, 20818, 20743	20, 31, 11	16.55469, 28.27829, 11.37266	8.3546, 11.57955, 7.38048	13.45658,	3	nap1l4	0
GL174143.1	33544	34669	33882, 33862, 34448	12, 16, 9	12.74986, 16.59459, 11.64359	8.02869, 9.48505, 7.57973	9.82533, 1	3	NA	748
GL174143.1	33709	34743	34446, 34540, 34448	6, 8, 9	5.3939, 6.77831, 11.64359	4.32314, 5.0215, 7.57973	3.0374, 4.4	3	NA	674
GL174143.1	54892	55614	55136, 55459, 55095	14, 10, 4	16.7649, 9.39128, 4.67325	9.96273, 6.35644, 3.78987	13.6543, 6	3	NA	18962
GL174145.1	436	764	611, 595, 591	18, 10, 5	22.92104, 9.39128, 5.9506	12.61945, 6.35644, 4.54784	19.59912,	3	NA	NA
GL174145.1	26215	26669	26223, 26247, 26252	11, 23, 19	9.48173, 22.59564, 22.61603	6.24938, 11.03113, 12.24534	6.76069, 1	3	NA	NA
GL174145.1	37057	37922	37285, 37482, 37493	14, 22, 12	10.67102, 17.98529, 9.18327	6.3592, 8.61494, 5.86906	7.87067, 1	3	NA	NA
GL174145.1	38190	39003	38444, 38458, 38419	12, 15, 14	8.70731, 10.6671, 11.75799	5.57226, 6.12993, 7.02904	6.03286, 8	3	NA	NA
GL174145.1	44550	45480	45283, 45184, 45302	10, 17, 9	11.06011, 18.56115, 11.64359	7.306, 10.40145, 7.57973	8.22712, 1	3	NA	NA
GL174147.1	23321	23692	23626, 23626, 23531	12, 24, 12	13.8483, 28.90463, 16.36823	8.63436, 14.44646, 9.85365	10.86694,	3	acap1	7681
GL174147.1	40896	41896	41413, 41527, 41317, 41854	11, 16, 5, 5	8.84943, 13.2638, 4.07202, 4.14842	5.85597, 7.46304, 3.54571, 3.59261	6.16564, 1	3	acap1	0
GL174148.1	54564	55342	54747, 54970, 55059	6, 10, 17	5.24248, 9.07627, 21.64669	4.2332, 6.1667, 12.11304	2.89962, 6	3	NA	1008
GL174150.1	56506	56943	56754, 56547, 56578	7, 10, 5	7.1696, 9.39128, 5.9506	5.31345, 6.35644, 4.54784	4.61046, 6	3	NA	NA
GL174151.1	62	952	500, 467, 498	24, 30, 18	27.61606, 33.41243, 22.65197	13.69419, 14.95107, 12.47685	24.16528,	3	NA	867
GL174151.1	56156	57252	56425, 56447, 56467	6, 5, 5	5.96715, 3.94561, 5.9506	4.64927, 3.46715, 4.54784	3.52158, 1	3	XB-GENE-5856185	4761
GL174153.1	28575	29352	28787, 28837, 28790	9, 14, 8	9.72082, 14.45897, 9.76268	6.64182, 8.66788, 6.64964	6.97155, 1	3	atxn2l	4654
GL174153.1	36989	38023	37467, 37382, 37513	15, 25, 7	16.72085, 27.24645, 7.84208	9.7776, 13.13319, 5.66426	13.61562,	3	atxn2l	0
GL174154.1	3200	3913	3491, 3307, 3419	14, 15, 13	14.75506, 14.71623, 15.52427	8.84125, 8.60008, 9.42125	11.73423,	3	NA	27738
GL174154.1	13881	15363	14233, 14042, 13952, 14781	9, 16, 5, 6	8.11416, 12.09762, 4.70718, 6.03707	5.72019, 6.7556, 3.9211, 4.6874	5.49285, 9	3	NA	16288
GL174154.1	27417	28136	27482, 27759, 27755	8, 9, 8	7.15567, 6.8008, 8.6841	5.25151, 4.90352, 6.11715	4.61046, 4	3	NA	3515
GL174155.1	8321	10302	8776, 8504, 9852	7, 7, 9	7.1696, 5.97725, 11.21577	5.31345, 4.62287, 7.38849	4.61046, 3	3	NA	4864
GL174158.1	1050	1871	1140, 1224, 1664	9, 18, 8	7.97705, 13.7776, 6.69878	5.637, 7.25561, 4.97237	5.36577, 1	3	NA	NA
GL174158.1	37187	37885	37545, 37583, 37572	23, 30, 18	18.95384, 21.67437, 17.20298	8.87973, 8.37699, 9.3216	15.76105,	3	NA	NA
GL174158.1	49913	50442	50230, 50223, 50078	10, 22, 10	9.08301, 18.88388, 9.53891	6.17079, 9.13992, 6.44439	6.3862, 15	3	NA	NA
GL174159.1	76	664	310, 269, 435	12, 10, 5	13.8483, 9.39128, 5.9506	8.63436, 6.35644, 4.54784	10.86694,	3	NA	NA
GL174159.1	10402	11236	10822, 10754, 10710	29, 46, 17	19.81123, 33.5699, 13.31307	7.71293, 9.70634, 7.2363	16.58766,	3	NA	NA
GL174159.1	54202	55446	54565, 54422, 54145, 55013	12, 14, 14, 12	7.32501, 8.16928, 10.8526, 9.7119	4.71975, 4.85873, 6.47083, 6.19878	4.75565, 5	3	NA	NA
GL174161.1	32167	33904	32198, 33826, 33641	5, 11, 6	4.43696, 9.05476, 6.25896	3.76555, 5.98422, 4.80589	2.24017, 6	3	NA	NA
GL174161.1	32330	33908	33607, 33826, 33641	6, 11, 6	5.63898, 9.05476, 6.25896	4.46544, 5.98422, 4.80589	3.25772, 6	3	NA	NA
GL174162.1	25145	25729	25576, 25310, 25509	10, 6, 7	11.06011, 4.93312, 8.69489	7.306, 4.04501, 6.06379	8.22712, 2	3	NA	624
GL174165.1	45357	46172	45847, 45865, 45711	20, 24, 15	22.26827, 25.29108, 17.07266	11.7806, 12.31022, 9.97618	18.96687,	3	tbc1d20.1	0
GL174165.1	60335	60644	60461, 60568, 60467	12, 10, 6	13.38718, 8.79469, 6.99807	8.38496, 5.99491, 5.17195	10.43197,	3	HCK	0
GL174167.1	2687	3310	2985, 2998, 2998	8, 8, 7	8.16648, 7.07159, 8.69489	5.83735, 5.20073, 6.06379	5.54098, 4	3	xtr-let-7e-1	11315
GL174167.1	11596	13093	12299, 11833, 11833, 12478	11, 12, 3, 6	9.21796, 8.08118, 3.37781, 7.12188	6.08584, 5.18359, 2.98791, 5.22884	6.51275, 5	3	xtr-let-7e-1	1532
GL174169.1	19398	19923	19830, 19821, 19891	12, 14, 11	9.34223, 11.15936, 11.25734	5.96826, 6.65976, 7.31423	6.62897, 8	3	NA	0
GL174169.1	26035	26574	26307, 26303, 26284	9, 12, 9	7.32038, 10.98912, 10.49552	5.23124, 6.98757, 7.0436	4.75116, 8	3	NA	0
GL174171.1	61	2778	1932, 2584, 605	79, 100, 88	56.98365, 70.64335, 68.95913	10.77566, 10.84294, 12.54031	53.03374,	3	NA	1241
GL174171.1	10874	11219	11009, 11041, 11013	16, 7, 11	15.87778, 4.32411, 10.19222	9.05608, 3.56536, 6.68421	12.8057, 2	3	NA	0
GL174171.1	26366	26692	26505, 26582, 26536	11, 13, 18	10.3799, 10.55511, 19.91385	6.79736, 6.51132, 10.94239	7.59574, 7	3	zfp1	1322
GL174171.1	53670	54149	53877, 53833, 53843	8, 12, 7	7.67464, 11.86108, 8.35502	5.55832, 7.51216, 5.9108	5.08299, 9	3	dnajc5g	2440
GL174178.1	30419	31873	31127, 31452, 30928	23, 22, 14	19.15226, 14.10074, 11.99897	8.99421, 6.46833, 7.17765	15.95279,	3	NA	NA
GL174180.1	1153	1528	1446, 1341, 1431	11, 15, 12	10.60582, 13.6391, 12.50469	6.93244, 7.9452, 7.88834	7.80831, 1	3	zbtb43	10827
GL174180.1	4859	5463	5342, 5076, 5345	7, 12, 7	4.9179, 7.94095, 5.34867	3.95376, 5.09702, 4.23056	2.60319, 5	3	zbtb43	6892
GL174180.1	6244	6705	6440, 6508, 6299	11, 17, 9	9.17548, 14.26997, 8.13839	6.05941, 7.81436, 5.73483	6.47227, 1	3	zbtb43	5650
GL174181.1	19234	19680	19618, 19678, 19653	14, 15, 6	14.52862, 14.21432, 6.08001	8.70881, 8.29617, 4.71063	11.51702,	3	znf250	2336
GL174182.1	45367	46323	45857, 46147, 46060	13, 14, 14	15.29169, 14.45897, 19.30655	9.29854, 8.66788, 11.20466	12.24396,	3	NA	NA
GL174183.1	126	1119	819, 905, 718	45, 40, 29	52.94449, 44.16141, 34.40662	19.9377, 17.10356, 15.92662	49.05064,	3	NA	NA
GL174183.1	28347	29285	29104, 28882, 28886	19, 34, 12	18.13647, 34.82499, 12.62857	9.59882, 14.19696, 7.95947	14.97405,	3	NA	NA
GL174183.1	48540	49451	49339, 49346, 49137	13, 11, 12	7.99965, 6.03365, 8.99931	4.94388, 4.10206, 5.75428	5.38625, 3	3	NA	NA
GL174185.1	28038	29183	28674, 28505, 28641	22, 45, 23	12.12429, 26.73431, 17.10885	5.47727, 7.32256, 7.83846	9.23839, 2	3	NA	NA
GL174185.1	30261	31405	31120, 30967, 30963	71, 96, 39	67.15437, 84.01357, 35.98068	16.43923, 15.39929, 12.93962	63.08788,	3	NA	NA
GL174185.1	45038	45996	45264, 45493, 45312	32, 48, 34	28.72093, 42.91866, 33.97171	11.47913, 13.3023, 13.70543	25.2439, 3	3	NA	NA
GL174187.1	16056	16639	16120, 16487, 16492	7, 6, 6	6.2098, 4.68027, 6.45058	4.76362, 3.88713, 4.90508	3.74679, 2	3	NA	NA
GL174187.1	35731	35923	35735, 35779, 35757	67, 72, 57	81.99226, 83.59547, 73.53984	25.48359, 23.84322, 25.92648	77.77785,	3	NA	NA
GL174187.1	49226	50584	50055, 50359, 49769	9, 10, 12	8.35434, 8.57382, 12.50469	5.86444, 5.8589, 7.88834	5.71584, 6	3	NA	NA
GL174187.1	51210	51841	51587, 51610, 51778	12, 26, 6	12.18267, 30.27268, 5.67889	7.70145, 14.5794, 4.48822	9.29304, 2	3	NA	NA
GL174189.1	8099	9477	8513, 8996, 8705, 9410	6, 7, 3, 8	5.81608, 5.97725, 3.47371, 10.1465	4.56563, 4.62287, 3.03189, 6.82176	3.4176, 3.6	3	NA	NA
GL174189.1	15882	16691	16103, 16187, 16161	18, 24, 16	13.47082, 19.57316, 12.6443	7.07481, 8.93633, 7.086	10.51248,	3	NA	NA
GL174189.1	41626	42430	42081, 42074, 42088	14, 12, 6	11.22666, 7.2756, 5.46454	6.70125, 4.68974, 4.36458	8.38513, 4	3	NA	NA
GL174189.1	50987	51882	51439, 51310, 51255	10, 6, 12	11.06011, 4.93312, 15.36829	7.306, 4.04501, 9.40047	8.22712, 2	3	NA	NA
GL174190.1	26617	27898	27367, 27263, 26996	11, 23, 14	11.15818, 23.18973, 16.82614	7.25692, 11.38786, 9.99514	8.32008, 2	3	NA	12686
GL174190.1	41827	42713	42371, 42351, 42313	16, 27, 9	19.7934, 33.30156, 11.64359	11.29109, 16.00277, 7.57973	16.57067,	3	NA	27896
GL174192.1	6859	7099	7078, 7034, 7054	12, 8, 6	11.98519, 6.85668, 7.29403	7.58551, 5.06969, 5.30581	9.10644, 4	3	NA	0
GL174192.1	34292	34740	34658, 34506, 34466	11, 8, 5	12.43693, 7.07159, 5.9506	7.97018, 5.20073, 4.54784	9.52692, 4	3	NA	25475
GL174193.1	47	1101	331, 527, 122	14, 13, 8	16.7649, 13.14514, 10.1465	9.96273, 8.09002, 6.82176	13.6543, 1	3	NA	NA
GL174193.1	27176	27979	27518, 27655, 27676	13, 16, 7	9.31977, 10.80113, 5.76629	5.74669, 5.98557, 4.493	6.60814, 8	3	NA	NA
GL174194.1	1206	3021	2550, 2648, 2493	34, 63, 22	34.85817, 59.56123, 22.26792	14.21617, 15.86961, 11.16306	31.2476, 5	3	NA	24321
GL174194.1	45425	46456	45973, 45840, 45918	15, 25, 7	12.6618, 22.30103, 5.45422	7.34555, 10.19404, 4.2975	9.74163, 1	3	NA	2134
GL174196.1	9520	10745	10432, 10096, 9919	37, 53, 32	34.29853, 50.89833, 35.43546	12.76032, 15.40802, 15.3579	30.69914,	3	NA	NA
GL174196.1	32098	33270	32636, 32935, 32128	30, 40, 21	33.81694, 43.74676, 24.0973	15.19169, 16.86382, 12.56358	30.22763,	3	NA	NA
GL174196.1	43607	45464	43770, 44749, 44749	13, 46, 33	7.63771, 39.10523, 34.40305	4.72848, 12.14157, 14.34816	5.04855, 3	3	NA	NA
GL174196.1	43663	45464	44750, 44749, 44749	24, 46, 33	18.87538, 39.10523, 34.40305	8.54297, 12.14157, 14.34816	15.68463,	3	NA	NA
GL174198.1	1068	2031	1696, 1722, 1693	12, 10, 10	13.8483, 9.39128, 13.11399	8.63436, 6.35644, 8.30815	10.86694,	3	NA	18287
GL174198.1	49334	50535	49640, 49645, 49666	12, 24, 13	13.55951, 27.71511, 16.70862	8.47903, 13.7525, 10.0169	10.59689,	3	mon1b	6983
GL174202.1	69292	70736	69869, 69865, 70164	86, 92, 53	92.19014, 97.39583, 59.14379	21.78938, 21.68619, 19.72369	87.86983,	3	5S_rRNA	0
GL174205.1	4651	5363	5181, 5168, 4920	17, 16, 23	13.31149, 11.18144, 21.19111	7.23535, 6.20906, 10.19146	10.36022,	3	NA	NA
GL174205.1	23996	24335	24046, 24038, 24165	7, 9, 10	7.1696, 8.19918, 13.18184	5.31345, 5.77148, 8.33771	4.61046, 5	3	NA	NA
GL174206.1	4244	4803	4384, 4498, 4770	6, 10, 5	5.90927, 9.27513, 5.9506	4.61743, 6.2868, 4.54784	3.50164, 6	3	boc	0
GL174210.1	23262	23621	23441, 23405, 23396	5, 7, 4	4.82177, 5.97725, 4.67325	3.98509, 4.62287, 3.78987	2.51418, 3	3	NA	NA
GL174210.1	28780	29350	28863, 28926, 28801	8, 7, 10	8.42257, 5.97725, 11.997	5.97763, 4.62287, 7.78835	5.76336, 3	3	NA	NA
GL174213.1	4388	4802	4691, 4655, 4748	9, 12, 5	9.72082, 11.86108, 5.91431	6.64182, 7.51216, 4.53172	6.97155, 9	3	ddi2	8705
GL174213.1	19271	19709	19519, 19535, 19535	5, 8, 4	4.82177, 6.73981, 4.64325	3.98509, 4.99774, 3.77643	2.51418, 4	3	ddi2	0
GL174213.1	39099	40060	39658, 39739, 39781	9, 6, 12	9.72082, 4.93312, 15.80834	6.64182, 4.04501, 9.60504	6.97155, 2	3	ddi2	18319
GL174215.1	3183	3825	3713, 3547, 3491	8, 12, 4	7.38183, 10.83413, 3.82084	5.38666, 6.89288, 3.36473	4.80856, 8	3	NA	25135
GL174215.1	10641	11242	11226, 11216, 11216	28, 29, 14	30.10456, 28.88285, 14.66346	13.74805, 12.64966, 8.7878	26.59488,	3	NA	17718
GL174216.1	16	1438	1110, 1029, 1088	73, 85, 56	74.47237, 81.9978, 62.67302	18.94147, 17.85478, 20.27416	70.33142,	3	nek4	4806
GL174217.1	8871	10276	9729, 9154, 9182	10, 21, 6	8.84451, 19.54437, 5.793	6.02544, 9.8421, 4.55271	6.16137, 1	3	NA	NA
GL174217.1	33208	33639	33599, 33401, 33503	7, 11, 8	7.1696, 10.60895, 10.1465	5.31345, 6.9343, 6.82176	4.61046, 7	3	NA	NA
GL174218.1	6074	6787	6365, 6394, 6312	7, 7, 5	7.04884, 5.97725, 5.54867	5.2473, 4.62287, 4.3619	4.52221, 3	3	NA	8201
GL174219.1	48028	48512	48309, 48315, 48114	12, 9, 5	13.73771, 7.83963, 5.9506	8.57523, 5.55305, 4.54784	10.76705,	3	NA	17108
GL174220.1	16444	16940	16615, 16631, 16834	18, 15, 7	17.93309, 12.58265, 5.50849	9.76366, 7.29694, 4.33177	14.77832,	3	sema4a	0
GL174220.1	48831	49666	49409, 49185, 48924	9, 9, 7	7.67537, 8.21099, 8.49722	5.45199, 5.77858, 5.97582	5.08317, 5	3	sema4a	25298
GL174224.1	5946	8094	7163, 6929, 6357	25, 27, 10	22.3777, 20.11253, 9.68182	10.2387, 8.3762, 6.52889	19.07226,	3	NA	NA
GL174224.1	10760	12101	11714, 11444, 11635	13, 29, 12	9.98831, 23.17993, 11.66047	6.15984, 9.43119, 7.39284	7.22551, 2	3	NA	NA
GL174224.1	16786	18141	17617, 17391, 17696	16, 28, 11	16.75964, 30.79727, 13.46208	9.5829, 14.16104, 8.49639	13.65293,	3	NA	NA
GL174225.1	46435	46817	46500, 46607, 46639	6, 6, 5	5.95712, 4.70958, 5.40789	4.64377, 3.90561, 4.29296	3.52158, 2	3	NA	1835

GL174225.1	59789	60689	59829, 60129, 59822	6, 16, 20	5.96715, 16.90523, 23.93369	4.64927, 9.66889, 12.7427	3.52158, 1	3	NA	9360
GL174225.1	63369	64055	63738, 63790, 63748	8, 10, 8	8.22568, 7.8291, 7.50809	5.8701, 5.39387, 5.46116	5.59681, 5	3	NA	12940
GL174226.1	13696	14164	13741, 13819, 13738	10, 15, 10	10.67246, 12.96377, 10.97191	7.09497, 7.53102, 7.25854	7.87096, 1	3	NA	NA
GL174227.1	22039	22921	22367, 22433, 22662	8, 14, 9	5.63975, 11.53986, 8.38703	4.30171, 6.89446, 5.88392	3.25835, 8	3	rtkn	0
GL174228.1	1414	2150	1485, 2091, 1719, 1961	4, 6, 4, 3	3.74195, 4.93312, 4.67325, 3.47371	3.32091, 4.04501, 3.78987, 3.03189	1.6222, 2.7	3	NA	25192
GL174229.1	36869	37976	37305, 37411, 37236	13, 17, 10	13.79066, 16.3049, 12.71002	8.46641, 9.05325, 8.12734	10.8171, 1	3	NA	9547
GL174230.1	42940	43693	43323, 43338, 43299	4, 11, 4	3.74195, 10.26356, 4.01199	3.32091, 6.72731, 3.46784	1.6222, 7.6	3	BICD1	18741
GL174232.1	16029	16723	16067, 16033, 16242	5, 12, 6	4.82177, 9.58381, 6.168	3.98509, 6.11896, 4.75778	2.51418, 6	3	NA	0
GL174232.1	46709	47134	46933, 46813, 46908	20, 42, 19	23.40409, 46.90917, 22.81433	12.44081, 17.81128, 12.35676	20.06837,	3	NA	3777
GL174233.1	187	1396	614, 614, 1173	13, 13, 6	15.07528, 13.14514, 6.99807	9.18277, 8.09002, 5.17195	12.04083,	3	slc12a9	3345
GL174233.1	10502	11072	10817, 10742, 10798	9, 20, 10	8.35434, 18.23344, 10.97191	5.86444, 9.35906, 7.25854	5.71584, 1	3	slc12a9	0
GL174233.1	12644	13449	13107, 13062, 13128	9, 16, 7	8.61068, 13.2638, 7.40077	6.01615, 7.46304, 5.43746	5.94135, 1	3	slc12a9	405
GL174233.1	40090	40694	40404, 40396, 40367	7, 16, 5	5.55587, 14.80681, 5.69847	4.36159, 8.40621, 4.4331	3.18254, 1	3	NA	63
GL174233.1	45183	45873	45404, 45375, 45405	9, 11, 6	8.50607, 9.90518, 6.87953	5.95453, 6.50968, 5.11627	5.84194, 7	3	NA	436
GL174234.1	16074	17418	16600, 16944, 16553	49, 71, 46	46.79821, 66.17319, 49.72359	14.85894, 16.02526, 17.6414	42.99307,	3	NA	8358
GL174236.1	17500	17803	17681, 17576, 17664	5, 6, 8	4.47183, 4.93312, 8.4586	3.78598, 4.04501, 5.99709	2.26935, 2	3	rsg1	711
GL174236.1	20717	21323	21019, 20828, 21280	5, 11, 5	4.33555, 10.48162, 4.39541	3.70555, 6.85833, 3.74107	2.15195, 7	3	rsg1	68
GL174236.1	42531	43301	42889, 43275, 42732	8, 8, 10	7.28955, 6.37769, 11.45478	5.33177, 4.77199, 7.51412	4.72176, 4	3	rsg1	21882
GL174237.1	219	1624	1195, 898, 555	13, 7, 8	13.79066, 5.97725, 10.1465	8.46641, 4.62287, 6.82176	10.8171, 3	3	NA	NA
GL174239.1	23515	24586	24142, 24141, 24145	18, 24, 10	16.86914, 22.52568, 10.47831	9.11888, 10.65622, 6.98694	13.75452,	3	NA	NA
GL174239.1	34338	34988	34933, 34929, 34706	8, 19, 9	6.98506, 18.21411, 10.01275	5.14817, 9.64585, 6.79683	4.46457, 1	3	NA	NA
GL174241.1	37140	37846	37268, 37285, 37300	14, 10, 9	16.7649, 9.39128, 11.64359	9.96273, 6.35644, 7.57973	13.6543, 6	3	NA	NA
GL174243.1	44760	45613	44958, 44883, 44895	47, 66, 34	58.6273, 81.8455, 44.39305	22.3008, 25.92291, 19.82081	54.65628,	3	NA	NA
GL174244.1	108	918	669, 484, 256	16, 12, 10	16.15907, 11.86108, 13.18184	9.22513, 7.51216, 8.33771	13.07749,	3	srrt	7031
GL174244.1	9956	10442	9999, 10128, 10256	5, 14, 3	4.0863, 13.03306, 3.47371	3.55452, 7.81216, 3.03189	1.93351, 1	3	srrt	0
GL174245.1	19159	19612	19298, 19439, 19346	9, 12, 5	9.12009, 11.60251, 5.10931	6.30997, 7.3582, 4.14026	6.42028, 8	3	SLCO1C1	0
GL174247.1	36988	37591	37159, 37158, 37345	20, 26, 14	25.10871, 32.02458, 18.05504	13.39632, 15.60218, 10.62048	21.72465,	3	NA	NA
GL174248.1	19588	20876	19857, 20105, 20300	11, 15, 10	12.43693, 14.91533, 12.33934	7.97018, 8.71987, 7.95424	9.52692, 1	3	NA	NA
GL174249.1	12023	12633	12426, 12462, 12425	13, 18, 9	11.35857, 15.69186, 8.79325	7.00844, 8.4039, 6.12267	8.51036, 1	3	NA	NA
GL174249.1	21865	23171	22675, 22328, 22915	12, 16, 13	9.34223, 10.64954, 12.76267	5.96826, 5.89712, 7.86275	6.62897, 8	3	NA	NA
GL174249.1	28354	29137	28675, 28701, 28478	10, 25, 16	8.70772, 23.47846, 18.88025	5.94148, 10.88484, 10.798	6.03286, 2	3	NA	NA
GL174250.1	15090	15491	15186, 15229, 15236	7, 18, 8	6.60038, 17.06771, 9.76268	4.99378, 9.23949, 6.64964	4.10761, 1	3	NA	NA
GL174252.1	41302	41948	41675, 41670, 41609	6, 6, 8	5.31708, 4.93312, 8.9249	4.2777, 4.04501, 6.24211	2.96727, 2	3	pgm2l1	3921
GL174253.1	42	434	336, 299, 239	12, 14, 4	13.8483, 14.45897, 4.67325	8.63436, 8.66788, 3.78987	10.86694,	3	NA	14988
GL174253.1	26601	28206	27034, 27658, 27255	10, 30, 13	4.7315, 18.80986, 9.67139	3.45436, 7.01956, 5.96366	2.49377, 1	3	NA	0
GL174253.1	26601	28301	27614, 27658, 27255	17, 30, 13	9.8996, 18.80986, 9.67139	5.26843, 7.01956, 5.96366	7.14133, 1	3	NA	0
GL174253.1	30327	32309	31082, 30990, 31554	30, 35, 19	23.27497, 23.83997, 16.04695	9.18906, 8.1675, 8.33647	19.94223,	3	NA	0
GL174254.1	6778	7811	7536, 7433, 7446	11, 9, 12	11.42621, 8.21099, 12.88563	7.41109, 5.77858, 8.10565	8.57514, 5	3	NA	31660
GL174254.1	42922	43591	43443, 43394, 43370	11, 13, 8	12.43693, 13.14514, 10.1465	7.97018, 8.09002, 6.82176	9.52692, 1	3	NA	101
GL174255.1	35	756	353, 296, 269	20, 28, 12	23.62787, 32.84267, 14.40488	12.56888, 15.37641, 8.92522	20.28502,	3	ash1l	16730
GL174255.1	6305	7472	6940, 7098, 6799	30, 17, 29	33.18705, 17.48427, 33.78949	14.81701, 9.76452, 15.56161	29.60997,	3	ash1l	10014
GL174256.1	18077	18929	18455, 18460, 18466	123, 150, 90	128.44249, 161.4299, 104.15293	22.661, 25.12756, 25.59638	123.83858	3	5S_rRNA	0
GL174256.1	43086	43661	43565, 43363, 43377	11, 9, 7	12.43693, 8.01512, 8.35502	7.97018, 5.66016, 5.9108	9.52692, 5	3	NA	177
GL174258.1	22985	23335	23032, 23019, 23003	11, 14, 5	10.3799, 12.1395, 4.95468	6.79736, 7.26424, 4.05779	7.59574, 9	3	NA	438
GL174258.1	35738	36534	36346, 36211, 35858	21, 27, 14	21.10247, 25.72383, 18.05504	10.7812, 11.51676, 10.62048	17.83755,	3	NA	9362
GL174258.1	42178	42601	42365, 42365, 42365	27, 36, 15	36.25318, 41.03524, 20.98137	17.66791, 17.00632, 11.95164	32.61507,	3	NA	15802
GL174261.1	14153	15989	15846, 14549, 14560	11, 12, 14	8.84943, 8.5933, 14.1066	5.85597, 5.50128, 8.45936	6.16564, 6	3	nop56	2303
GL174263.1	10893	11418	11018, 11100, 11148	16, 21, 10	15.03785, 18.60078, 10.08818	8.54705, 9.27642, 6.76544	12.00487,	3	fkbp4	0
GL174264.1	37772	38544	38003, 38108, 37828	5, 11, 3	4.50727, 9.57292, 3.47371	3.80664, 6.30569, 3.03189	2.30125, 6	3	NA	NA
GL174265.1	7793	8396	8284, 8180, 8199	5, 10, 5	4.47183, 8.70492, 4.12265	3.78598, 5.93975, 3.57684	2.26935, 6	3	NA	2893
GL174265.1	36032	37966	37000, 37002, 37000	63, 59, 44	57.51713, 50.34586, 41.03686	14.96527, 13.15484, 13.76986	53.5607, 4	3	NA	31132
GL174265.1	40900	42892	41032, 42248, 42269	27, 44, 29	13.25971, 36.36203, 26.05791	5.16571, 11.4734, 11.01687	10.31082,	3	NA	36000
GL174265.1	40900	42916	42737, 42248, 42269	40, 44, 29	34.7774, 36.36203, 26.05791	11.97019, 11.4734, 11.01687	31.16876,	3	NA	36000
GL174266.1	22336	22862	22629, 22648, 22604	7, 12, 10	6.05136, 9.54462, 10.42037	4.66801, 6.09452, 6.95441	3.59939, 6	3	tbc1d20.2	1631
GL174266.1	42507	42856	42765, 42807, 42611	9, 9, 5	9.36986, 8.21099, 5.9506	6.44997, 5.77858, 4.54784	6.65425, 5	3	dgat1	843
GL174267.1	6083	7082	6741, 6551, 6693	11, 19, 11	12.43693, 21.4153, 14.24187	7.97018, 11.55717, 8.86619	9.52692, 1	3	NA	34778
GL174267.1	30643	31358	30916, 31307, 31275	11, 23, 9	10.66406, 18.24704, 10.28145	6.96705, 8.47564, 6.93568	7.86396, 1	3	NA	10502
GL174268.1	4572	5518	5104, 4885, 4701	10, 16, 8	8.61898, 16.22539, 8.80246	5.88679, 9.26486, 6.179	5.94907, 1	3	NA	NA
GL174268.1	14786	16161	14984, 15934, 15480	8, 11, 9	6.63031, 9.4823, 9.41119	4.92991, 6.24973, 6.47287	4.13604, 6	3	NA	NA
GL174268.1	14786	16161	15857, 15934, 15480	11, 11, 9	9.81032, 9.4823, 9.41119	6.45164, 6.24973, 6.47287	7.05644, 6	3	NA	NA
GL174268.1	18432	18778	18453, 18491, 18458	6, 7, 5	4.77375, 5.27726, 4.51488	3.9459, 4.18506, 3.81106	2.51418, 3	3	NA	NA
GL174268.1	34022	34565	34219, 34408, 34536	8, 11, 8	8.16648, 10.21061, 9.76268	5.83735, 6.69533, 6.64964	5.54098, 7	3	NA	NA
GL174269.1	607	1000	683, 654, 675	5, 6, 4	4.82177, 4.93312, 4.67325	3.98509, 4.04501, 3.78987	2.51418, 2	3	NA	NA
GL174271.1	41824	42227	41981, 41940, 42011	5, 10, 5	4.82177, 9.39128, 5.9506	3.98509, 6.35644, 4.54784	2.51418, 6	3	NA	NA
GL174273.1	167	649	346, 483, 456	8, 14, 5	5.78012, 10.15403, 4.12265	4.39198, 6.04265, 3.57684	3.38637, 7	3	NA	NA
GL174273.1	3335	4226	3556, 3575, 3538	19, 20, 14	16.08128, 13.40318, 12.33709	8.35706, 6.54921, 7.38584	13.00262,	3	NA	NA
GL174274.1	7451	7897	7557, 7538, 7536	22, 30, 19	25.96118, 34.4899, 25.66388	13.35351, 15.5917, 13.87136	22.55288,	3	NA	0
GL174274.1	35625	36720	36264, 35754, 36295, 35560, 36350	6, 11, 7, 4, 6	5.96715, 10.60895, 5.82304, 4.26291, 6.87953	4.64927, 6.9343, 4.52813, 3.59641, 5.11627	3.52158, 7	3	NA	25898
GL174276.1	258	885	568, 570, 378	33, 46, 14	37.51642, 48.86988, 15.49745	16.18439, 17.16623, 9.26757	33.85472,	3	ppp1r16a	6369
GL174276.1	1452	2519	2323, 1516, 1848	6, 21, 7	3.68605, 16.23501, 6.2897	3.23926, 7.88799, 4.81137	1.6222, 13	3	ppp1r16a	4735
GL174276.1	14861	15476	15438, 15436, 15440	25, 34, 19	26.86394, 34.99558, 24.85428	12.90457, 14.29576, 13.45893	23.43188,	3	ppp1r16a	0
GL174276.1	41132	42086	41658, 41783, 41575	17, 21, 13	18.46847, 22.48702, 17.40688	10.34725, 11.61241, 10.34389	15.29249,	3	5S_rRNA	0
GL174277.1	7788	8224	8054, 8086, 8079	29, 33, 24	30.60048, 34.2076, 26.30847	13.66501, 14.23402, 12.91866	27.07943,	3	sfrpx	0
GL174277.1	21287	21583	21461, 21512, 21464	10, 12, 4	9.661, 10.35, 3.82084	6.51661, 6.59497, 3.36473	6.9283, 7.7	3	sfrpx	0
GL174277.1	38416	39022	38970, 38960, 38971	10, 19, 15	9.83337, 19.41322, 18.13606	6.61776, 10.37021, 10.55788	7.07841, 1	3	folr1	500
GL174281.1	15214	15658	15408, 15364, 15396	13, 16, 12	10.50223, 12.48972, 11.07378	6.47852, 6.99232, 7.03912	7.71092, 9	3	NA	NA
GL174281.1	21062	21746	21244, 21635, 21284	9, 12, 8	6.63709, 9.78474, 7.5499	4.79941, 6.24415, 5.48567	4.14229, 7	3	NA	NA
GL174284.1	11907	12990	12741, 12742, 12731	19, 13, 9	24.51879, 13.14514, 11.64359	13.28363, 8.09002, 7.57973	21.15031,	3	NA	14841
GL174286.1	49	814	472, 473, 440	31, 34, 19	36.10809, 37.83902, 24.60107	16.15843, 15.96206, 13.32686	32.47302,	3	NA	35658
GL174286.1	22582	23558	23057, 22738, 23055	13, 18, 13	10.14103, 13.20309, 12.09796	6.2545, 6.91801, 7.46159	7.3686, 10	3	NA	12914
GL174288.1	184	815	477, 344, 720	15, 30, 18	6.24562, 13.33341, 9.50636	3.66035, 4.7918, 4.8794	3.78132, 1	3	NA	NA
GL174288.1	10867	12051	11881, 11177, 11584	26, 37, 23	10.04553, 12.48974, 11.28838	4.04437, 3.86395, 4.92481	7.27897, 9	3	NA	NA
GL174288.1	17200	18599	18417, 17578, 18473, 17701, 18006	22, 20, 29, 10, 13	11.60686, 11.49149, 14.9535, 6.99932, 9.10008	5.23088, 5.53458, 5.55096, 4.86901, 5.61162	8.74672, 8	3	NA	NA
GL174288.1	22534	23870	22652, 22769, 22835	32, 31, 18	20.94081, 16.99801, 13.33691	7.54947, 6.05464, 6.99627	17.68127,	3	NA	NA
GL174288.1	33045	34163	33554, 33445, 33289	39, 46, 20	42.33217, 46.85904, 21.96564	16.47727, 16.06641, 11.60219	38.59222,	3	NA	NA
GL174288.1	39083	40458	39888, 39186, 39159, 40299	28, 36, 15, 11	23.60262, 28.99102, 13.67124, 9.07307	9.96844, 10.33132, 7.96487, 5.99563	20.2611, 2	3	NA	NA
GL174289.1	4401	4778	4583, 4618, 4530	7, 10, 5	5.79309, 8.36423, 5.59755	4.5096, 5.72892, 4.38538	3.39779, 5	3	aldoa	2961
GL174289.1	10953	11997	11753, 11724, 10940, 11450, 11718	5, 13, 5, 5, 6	4.655, 11.87216, 4.64121, 4.57714, 5.67889	3.89157, 7.32385, 3.88372, 3.84705, 4.48822	2.42652, 9	3	aldoa	666
GL174289.1	42298	43767	42506, 42827, 42449	11, 13, 4	10.96672, 13.1283, 4.67325	7.14544, 8.08007, 3.78987	8.14942, 1	3	ppp4c	21064
GL174289.1	42448	43767	43715, 42827, 42449	5, 13, 4	4.54329, 13.1283, 4.67325	3.82752, 8.08007, 3.78987	2.33, 10.36	3	ppp4c	21214
GL174291.1	2385	2585	2522, 2516, 2530	19, 19, 8	24.51879, 21.4153, 10.1465	13.28363, 11.55717, 6.82176	21.15031,	3	NA	19154
GL174291.1	17851	19067	18753, 18321, 18330	15, 15, 7	17.09296, 13.74993, 7.40077	9.98755, 8.013, 5.43746	13.96978,	3	NA	2672
GL174292.1	6483	6945	6586, 6715, 6648	8, 12, 8	8.42257, 11.86108, 10.1465	5.97763, 7.51216, 6.82176	5.76336, 9	3	NA	19627
GL174293.1	13938	14996	14551, 14120, 14323	12, 17, 13	10.39353, 13.80996, 13.58227	6.62187, 7.53566, 8.34597	7.60856, 1	3	stx16	864
GL174293.1	27728	28557	28230, 28401, 28353	7, 15, 10	5.4599, 11.72411, 10.30677	4.30109, 6.77081, 6.89026	3.09813, 9	3	stx16	14654
GL174293.1	33121	33624	33402, 33396, 33391	14, 23, 19	12.08222, 20.65165, 18.84829	7.22895, 9.87151, 10.02962	9.19818, 1	3	stx16	20047
GL174293.1	37562	38120	38093, 37857, 37864	8, 8, 11	7.15567, 5.46019, 9.92808	5.25151, 4.18585, 6.52366	4.61046, 3	3	stx16	24488
GL174296.1	9705	10131	9758, 9884, 9742	10, 12, 5	11.06011, 11.86108, 5.9506	7.306, 7.51216, 4.54784	8.22712, 9	3	NA	NA
GL174296.1	29796	30443	29934, 30363, 29963	4, 6, 4	3.74195, 4.93312, 4.67325	3.32091, 4.04501, 3.78987	1.6222, 2.7	3	NA	NA
GL174297.1	38136	38476	38190, 38234, 38281	10, 19, 8	10.96713, 20.47061, 10.1465	7.25596, 11.002, 6.82176	8.14942, 1	3	NA	6652
GL174298.1	48	1106	439, 333, 362	52, 50, 38	55.55954, 53.2696, 45.31404	18.21818, 17.84962, 18.66188	51.62872,	3	NA	NA
GL174298.1	15441	16533	15934, 15799, 16209	24, 33, 18	21.09216, 27.8216, 18.60267	9.81167, 10.66013, 10.16634	17.82816,	3	NA	NA
GL174298.1	28404	28947	28666, 28843, 28671	18, 22, 12	10.32533, 10.14474, 7.95439	5.3073, 4.56518, 5.10531	7.54345, 7	3	NA	NA
GL174298.1	32632	33239	33061, 33190, 32845	18, 22, 12	8.33774, 7.71642, 7.02923	4.29595, 3.5599, 4.54068	5.70219, 5	3	NA	NA
GL174298.1	36701	37570	37124, 37013, 37116	41, 55, 22	32.71644, 38.06568, 18.43797	10.65673, 9.41223, 8.87842	29.1495, 3	3	NA	NA
GL174298.1	38593	39456	38706, 38779, 38744	27, 56, 16	20.89771, 45.39192, 13.73713	8.79292, 11.96091, 7.75225	17.63994,	3	NA	NA
GL174299.1	24413	24824	24639, 24738, 24520	10, 9, 8	10.13706, 7.59096, 8.9249	6.7935, 5.39978, 6.24211	7.36489, 5	3	NA	NA
GL174301.1	1011	1507	1298, 1149, 1039	13, 19, 7	14.35383, 21.4153, 8.57075	8.78627, 11.55717, 6.00887	11.34993,	3	NA	23564
GL174302.1	3246	4122	3683, 3989, 3693	9, 9, 7	7.88835, 7.28344, 7.40077	5.58287, 5.20811, 5.43746	5.28191, 4	3	fam189b	0
GL174303.1	25154	25675	25452, 25365, 25328	8, 11, 5	8.42257, 10.60895, 5.9506	5.97763, 6.9343, 4.54784	5.76336, 7	3	rgs11	18698
GL174304.1	37492	37935	37851, 37758, 37575	39, 46, 27	44.48956, 47.74661, 32.49398	17.73374, 16.54833, 15.53278	40.71792,	3	NA	NA
GL174307.1	15393	16023	15908, 15888, 15613	10, 8, 8	10.74428, 7.07159, 10.1465	7.13454, 5.20073, 6.82176	7.93927, 4	3	NA	NA
GL174307.1	25246	26233	25483, 26054, 26050	20, 30, 24	21.79824, 30.89514, 29.58706	11.50312, 13.45807, 14.83838	18.51069,	3	NA	NA
GL174308.1	33823	34133	33865, 33886, 33843	24, 39, 20	26.38029, 43.97023, 23.73483	12.96149, 17.42972, 12.62984	22.96032,	3	NA	17137
GL174310.1	33559	34084	33622, 33699, 33800	7, 9, 6	7.10297, 8.21099, 7.29403	5.27706, 5.77858, 5.30581	4.57191, 5	3	fam131b	0
GL174311.1	33330	33687	33657, 33642, 33659	24, 27, 22	26.2838, 28.43981, 26.79743	12.90395, 13.12665, 13.83268	22.86648,	3	NA	9789
GL174313.1	13412	13726	13420, 13457, 13445	16, 23, 18	19.18518, 23.5013, 24.2813	10.96495, 11.57503, 13.31378	15.98366,	3	NA	NA
GL174314.1	2042	2642	2132, 2198, 2515	12, 5, 4	9.45689, 3.58952, 4.67325	6.0398, 3.23987, 3.78987	6.73641, 1	3	raf1	9031
GL174314.1	3253	3814	3545, 3748, 3614	11, 8, 9	8.36628, 6.21066, 11.64359	5.55301, 4.66659, 7.57973	5.72708, 3	3	raf1	7859
GL174316.1	15434	16205	16149, 15805, 15631	6, 16, 4	5.96715, 17.16856, 4.67325	4.64927, 9.82359, 3.78987	3.52158, 1	3	NA	10588
GL174316.1	23859	24378	23972, 24153, 24189	18, 36, 15	17.12119, 38.35513, 16.37605	9.27196, 15.43492, 9.5803	13.99654,	3	NA	19013
GL174316.1	36744	37789	37294, 37272, 37294	212, 315, 137	218.85008, 319.85971, 147.0562	24.32305, 24.54201, 24.53604	213.77164	3	NA	31898
GL174318.1	11216	12353	12140, 11830, 12130	12, 14, 7	10.48835, 13.8891, 8.15288	6.6804, 8.32957, 5.81587	7.69782, 1	3	NA	NA
GL174319.1	16564	16936	16677, 16781, 16846	10, 14, 9	9.03421, 9.82448, 8.26067	6.14116, 5.84215, 5.80842	6.33915, 7	3	NA	NA
GL174320.1	7060	8353	7906, 7559, 7250	10, 16, 6	7.93364, 12.44939, 5.83216	5.45963, 6.9679, 4.57461	5.325, 9.71	3	NA	9266
GL174320.1	35230	37266	36257, 35943, 36251	18, 41, 21	15.61903, 35.52044, 23.4003	8.35978, 12.01322, 12.15468	12.55806,	3	NA	5244
GL174324.1	48	1577	935, 819, 711	24, 29, 28	25.99978, 29.93329, 30.48566	12.73437, 13.26932, 13.97527	22.5903, 2	3	NA	NA
GL174324.1	7145	8911	7557, 7640, 7878	15, 29, 15	13.74446, 28.50226, 16.59998	8.00965, 12.42647, 9.70872	10.77314,	3	NA	NA
GL174324.1	16759	17713	17429, 17252, 16920	21, 30, 16	15.16632, 22.69097, 16.24901	7.2824, 8.88856, 9.279	12.1279, 1	3	NA	NA
GL174324.1	21306	24351	23590, 23591, 23613	48, 77, 41	36.48084, 62.03053, 36.08691	10.41099, 12.78049, 12.29767	32.83902,	3	NA	NA
GL174324.1	28260	29191	28469, 28747, 28787	16, 19, 12	12.26603, 15.12892, 12.20749	6.85709, 7.78953, 7.71596	9.37251, 1	3	NA	NA
GL174324.1	36150	37077	36421, 36423, 36436	56, 42, 32	71.84335, 47.80266, 41.63489	25.46475, 18.32871, 19.00521	67.72878,	3	NA	NA
GL174326.1	12400	12770	12482, 12435, 12468	17, 16, 8	17.88146, 15.41438, 9.60885	10.00115, 8.77591, 6.57807	14.72916,	3	NA	NA
GL174327.1	47492	48103	47788, 47716, 47859	8, 9, 7	8.42257, 8.21099, 7.61369	5.97763, 5.77858, 5.54854	5.76336, 5	3	NA	NA
GL174329.1	7832	8206	8035, 8021, 8136	11, 11, 5	12.43693, 10.60895, 5.9506	7.97018, 6.9343, 4.54784	9.52692, 7	3	NA	NA
GL174331.1	13606	14242	13882, 14091, 13670	6, 10, 4	5.96715, 9.39128, 4.67325	4.64927, 6.35644, 3.78987	3.52158, 6	3	NA	NA
GL174332.1	12914	13847	13491, 13788, 13645	5, 7, 3	4.82177, 5.82304, 3.47371	3.98509, 4.52813, 3.03189	2.51418, 3	3	NA	NA
GL174335.1	167	731	685, 489, 546	12, 16, 13	12.3187, 15.94397, 15.34872	7.78073, 9.09593, 9.3288	9.4231, 13	3	NA	3984
GL174336.1	182	1193	972, 879, 611	9, 9, 6	9.72082, 8.21099, 7.29403	6.64182, 5.77858, 5.30581	6.97155, 5	3	NA	20283
GL174339.1	12670	13509	12889, 13115, 12908	13, 16, 7	12.43975, 15.54288, 7.20145	7.66875, 8.85379, 5.33074	9.5289, 12	3	NA	NA
GL174339.1	28397	29188	28892, 29102, 28826	8, 9, 6	6.37855, 6.12736, 5.11235	4.77253, 4.47368, 4.15475	3.90357, 3	3	NA	NA
GL174339.1	29330	29953	29921, 29909, 29919	12, 30, 20	10.25464, 30.64445, 22.98257	6.53598, 13.31039, 12.19768	7.47735, 2	3	NA	NA
GL174343.1	106	1046	403, 635, 665	25, 27, 20	29.58099, 30.24653, 25.47128	14.51547, 14.20456, 13.59302	26.08281,	3	snx17	3365
GL174343.1	74571	75439	74988, 75037, 75241	9, 14, 6	8.94274, 11.76301, 6.03707	6.20889, 7.03214, 4.6874	6.2534, 9.0	3	fndc4	0
GL174345.1	19965	20282	20050, 20054, 20041	51, 65, 52	56.83364, 77.09502, 64.86276	19.36264, 23.80943, 23.45977	52.8867, 7	3	NA	0
GL174345.1	44887	46321	45329, 45639, 44954, 45917	8, 18, 7, 7	7.47668, 17.06771, 6.6707, 7.61369	5.44269, 9.23949, 5.03432, 5.54854	4.89713, 1	3	NA	0
GL174345.1	44887	46321	46064, 45639, 44954, 45917	7, 18, 7, 7	6.42049, 17.06771, 6.6707, 7.61369	4.8888, 9.23949, 5.03432, 5.54854	3.94161, 1	3	NA	0
GL174346.1	9996	10706	10180, 10499, 10278	10, 13, 6	11.06011, 13.14514, 7.29403	7.306, 8.09002, 5.30581	8.22712, 1	3	c2cd5	5175
GL174346.1	16309	16750	16450, 16446, 16459	11, 31, 16	10.72304, 31.03973, 18.52308	7.00201, 13.16201, 10.59969	7.91902, 2	3	c2cd5	0
GL174346.1	30593	30960	30713, 30693, 30751	6, 8, 5	5.96715, 7.07159, 5.9506	4.64927, 5.20073, 4.54784	3.52158, 4	3	c2cd5	2808
GL174349.1	6351	7291	6773, 6795, 6993	9, 9, 7	9.72082, 8.21099, 8.69489	6.64182, 5.77858, 6.06379	6.97155, 5	3	NA	NA
GL174349.1	13022	13958	13455, 13296, 13114	11, 19, 7	9.61965, 16.67602, 7.20145	6.33449, 8.71486, 5.33074	6.89006, 1	3	NA	NA
GL174349.1	15782	16929	16174, 16199, 16548	12, 20, 11	10.78431, 17.88727, 12.48556	6.86236, 9.1506, 7.99611	7.9765, 14	3	NA	NA
GL174349.1	34728	35625	35069, 34943, 35132	9, 19, 6	9.72082, 21.4153, 7.29403	6.64182, 11.55717, 5.30581	6.97155, 1	3	NA	NA
GL174351.1	989	2987	1794, 2312, 1280	14, 18, 9	10.98164, 12.81028, 7.86759	6.55025, 6.68975, 5.57018	8.16315, 1	3	NA	NA
GL174351.1	989	3160	2712, 2312, 1280	11, 18, 9	7.0723, 12.81028, 7.86759	4.74161, 6.68975, 5.57018	4.54399, 1	3	NA	NA
GL174351.1	6146	8492	6649, 7884, 7563	9, 24, 13	5.24872, 19.38345, 11.17224	3.91147, 8.82879, 6.89345	2.90538, 1	3	NA	NA
GL174351.1	6146	8492	7873, 7884, 7563	10, 24, 13	7.13071, 19.38345, 11.17224	4.9523, 8.82879, 6.89345	4.59751, 1	3	NA	NA
GL174351.1	15903	17935	16384, 16663, 16336	17, 17, 11	16.6291, 12.97503, 12.55982	9.24979, 7.03392, 8.03554	13.52753,	3	NA	NA
GL174351.1	21830	24612	22462, 22466, 22438	11, 19, 13	7.39504, 12.04257, 12.09796	4.94318, 6.0291, 7.46159	4.82154, 9	3	NA	NA
GL174351.1	21830	24612	23820, 22466, 22438	15, 19, 13	11.9085, 12.04257, 12.09796	6.88353, 6.0291, 7.46159	9.03357, 9	3	NA	NA
GL174351.1	34337	35175	34829, 34747, 35006	9, 15, 6	8.16099, 13.26621, 6.5517	5.74847, 7.71668, 4.95623	5.53692, 1	3	NA	NA
GL174352.1	2051	3173	3003, 3088, 2467	12, 15, 7	13.8483, 15.80067, 8.42533	8.63436, 9.24574, 5.94313	10.86694,	3	NA	18971
GL174352.1	19440	19893	19556, 19464, 19682	7, 7, 7	6.74267, 4.82021, 7.61369	5.07552, 3.89031, 5.54854	4.23998, 2	3	NA	2251
GL174353.1	81	1246	720, 399, 458	15, 23, 13	16.20017, 22.96257, 17.53191	9.47871, 11.25142, 10.40048	13.11636,	3	NA	NA
GL174356.1	25375	26012	25952, 25926, 25892	7, 11, 6	7.1696, 10.60895, 7.29403	5.31345, 6.9343, 5.30581	4.61046, 7	3	NA	0
GL174362.1	19730	20333	19932, 19753, 20278	9, 8, 10	6.09101, 5.23755, 8.54288	4.4504, 4.04172, 5.83976	3.63576, 3	3	NA	NA
GL174362.1	24301	24883	24672, 24509, 24418	10, 11, 8	7.8239, 8.04011, 6.97106	5.3906, 5.34803, 5.13965	5.22304, 5	3	NA	NA
GL174362.1	34309	34991	34624, 34732, 34858	19, 37, 17	19.72514, 38.31143, 19.63801	10.55748, 14.99919, 11.0208	16.50548,	3	NA	NA
GL174363.1	12877	13596	13070, 13222, 13170	10, 17, 14	8.12348, 14.81481, 15.96227	5.57869, 8.14576, 9.52765	5.50152, 1	3	rassf10	1035
GL174369.1	6666	7216	7022, 7148, 7115	12, 12, 17	10.83541, 11.25867, 22.63257	6.89366, 7.1513, 12.61306	8.02495, 8	3	actr3	0
GL174371.1	11859	12282	11966, 12044, 12090	7, 9, 8	7.10297, 8.21099, 10.1465	5.27706, 5.77858, 6.82176	4.57191, 5	3	TBC1D25	277
GL174373.1	134	843	484, 609, 499	9, 9, 8	9.72082, 8.21099, 10.1465	6.64182, 5.77858, 6.82176	6.97155, 5	3	NA	NA
GL174373.1	8326	9188	8637, 8510, 8695	9, 17, 15	6.19906, 14.03605, 14.16791	4.51959, 7.67248, 8.26794	3.7376, 11	3	NA	NA
GL174373.1	9136	9674	9622, 9614, 8695, 9635	28, 28, 15, 28	28.44258, 26.88936, 14.16791, 31.89813	12.75966, 11.84506, 8.26794, 14.81651	24.97166,	3	NA	NA
GL174373.1	18264	20129	18725, 19044, 18467	8, 16, 3	7.88469, 15.80758, 3.44998	5.67897, 9.01376, 3.02115	5.27935, 1	3	NA	NA
GL174378.1	30039	31249	30492, 30530, 30791	13, 7, 9	14.10549, 5.97725, 11.21577	8.64628, 4.62287, 7.38849	11.11201,	3	NA	NA
GL174382.1	2	345	101, 181, 79	199, 257, 261	227.83076, 287.56046, 319.70407	30.34926, 29.94346, 37.30347	222.7204,	3	NA	42
GL174383.1	3759	4730	4123, 4291, 4218	12, 18, 6	12.97977, 19.97713, 7.29403	8.15868, 10.97931, 5.30581	10.04411,	3	NA	0
GL174385.1	28244	28756	28472, 28644, 28619	14, 8, 8	16.43009, 6.51779, 10.1465	9.78349, 4.8598, 6.82176	13.33671,	3	NA	1018
GL174386.1	23246	23709	23504, 23554, 23512	10, 18, 5	10.20047, 17.19487, 5.50079	6.82978, 9.31668, 4.33868	7.42612, 1	3	NA	4397
GL174386.1	31560	32962	32090, 32097, 32023	21, 16, 15	23.61699, 17.16856, 18.74072	12.28232, 9.82359, 10.87493	20.27492,	3	NA	12711
GL174387.1	13753	14183	14060, 13869, 13848	14, 13, 7	13.57329, 11.36332, 8.69489	8.13982, 7.01137, 6.06379	10.60942,	3	NA	NA
GL174388.1	1890	2452	2014, 2260, 2044	17, 13, 4	19.10743, 13.14514, 4.67325	10.71816, 8.09002, 3.78987	15.90925,	3	klhdc3	268
GL174389.1	6255	7640	7057, 7058, 7057	32, 17, 24	37.15584, 16.63185, 28.20642	16.37888, 9.25145, 14.04062	33.5013, 1	3	NA	NA
GL174392.1	16042	16432	16329, 16276, 16286	9, 8, 7	9.72082, 7.07159, 8.69489	6.64182, 5.20073, 6.06379	6.97155, 4	3	NA	0
GL174394.1	334	1101	612, 414, 686	11, 13, 6	9.61965, 8.84052, 5.20372	6.33449, 5.45267, 4.20995	6.89006, 6	3	NA	NA
GL174394.1	22464	22704	22655, 22660, 22574	18, 18, 8	21.07666, 19.08138, 9.46129	11.6109, 10.4519, 6.50801	17.81304,	3	NA	NA
GL174394.1	22960	23828	23724, 23371, 23369	8, 9, 6	7.99469, 8.21099, 7.29403	5.74128, 5.77858, 5.30581	5.38155, 5	3	NA	NA
GL174396.1	11747	12308	12251, 12281, 11748, 12256	14, 22, 5, 11	16.7649, 25.85271, 5.9506, 14.75772	9.96273, 13.29074, 4.54784, 9.09568	13.6543, 2	3	NA	NA
GL174398.1	11202	11783	11232, 11229, 11203	22, 38, 17	24.67026, 40.84258, 20.77652	12.59865, 16.0412, 11.65116	21.29787,	3	NA	NA
GL174398.1	28580	30205	29213, 29210, 28820, 29230	46, 48, 13, 34	48.49275, 48.2645, 10.85814, 39.16676	16.95782, 16.00436, 6.69914, 16.74784	44.66208,	3	NA	NA
GL174399.1	487	1278	558, 956, 962	7, 9, 8	5.10557, 4.80498, 6.83213	4.075, 3.6298, 5.05462	2.77482, 2	3	NA	NA
GL174399.1	1174	3303	2750, 2761, 962, 1810, 2757	10, 30, 8, 9, 10	7.19168, 24.78332, 6.83213, 7.12591, 7.59326	4.99095, 9.98627, 5.05462, 5.10914, 5.24512	4.63123, 2	3	NA	NA
GL174399.1	2015	3367	3114, 2761, 1810, 2757	7, 30, 9, 10	4.17554, 24.78332, 7.12591, 7.59326	3.46743, 9.98627, 5.10914, 5.24512	2.01422, 2	3	NA	NA
GL174399.1	7682	9359	8485, 8569, 8784	12, 10, 6	11.32055, 7.38914, 5.0826	7.18871, 5.11603, 4.13667	8.47391, 4	3	NA	NA
GL174399.1	29974	31471	30530, 30833, 30679	5, 8, 6	4.82177, 6.89656, 6.99807	3.98509, 5.09414, 5.17195	2.51418, 4	3	NA	NA
GL174400.1	20219	21385	21135, 21122, 20753	9, 16, 6	6.99678, 14.69174, 5.60548	5.02768, 8.33598, 4.44624	4.47395, 1	3	NA	NA
GL174401.1	17258	17583	17547, 17573, 17545	41, 38, 32	48.65628, 42.80689, 41.91822	19.28208, 17.18536, 19.16778	44.82281,	3	NA	NA
GL174403.1	7065	7626	7397, 7261, 7415	15, 20, 7	16.72085, 21.43553, 8.21885	9.7776, 11.28761, 5.84717	13.61562,	3	NA	NA
GL174404.1	159	805	367, 460, 460	9, 10, 7	9.72082, 9.39128, 8.69489	6.64182, 6.35644, 6.06379	6.97155, 6	3	NA	NA
GL174408.1	1069	1369	1317, 1286, 1295	14, 13, 9	16.7649, 13.14514, 11.64359	9.96273, 8.09002, 7.57973	13.6543, 1	3	NA	NA
GL174408.1	7107	8020	7512, 7481, 7628	9, 10, 9	9.72082, 9.39128, 11.64359	6.64182, 6.35644, 7.57973	6.97155, 6	3	NA	NA
GL174408.1	33418	34447	33724, 33647, 33915	9, 10, 4	7.97705, 9.32635, 4.67325	5.637, 6.31756, 3.78987	5.36577, 6	3	NA	NA
GL174409.1	30957	31934	31496, 31338, 31263	10, 15, 11	11.06011, 15.80067, 14.75772	7.306, 9.24574, 9.09568	8.22712, 1	3	NA	NA
GL174410.1	49	1714	740, 854, 369	14, 19, 9	15.40455, 17.17455, 9.52439	9.21493, 9.01609, 6.53516	12.35264,	3	NA	NA
GL174410.1	30750	31939	30982, 30983, 31008, 31809	50, 74, 46, 27	37.28152, 57.2528, 38.56633, 17.69061	10.23734, 11.87454, 11.89022, 7.15014	33.62476,	3	NA	NA
GL174416.1	4508	5141	5118, 5067, 4936	12, 12, 7	10.20921, 10.12474, 7.50542	6.50784, 6.45547, 5.49244	7.4341, 7.5	3	heg1	0
GL174416.1	17628	18352	17963, 17955, 18030	5, 8, 14	4.82177, 6.62693, 16.2088	3.98509, 4.92781, 9.66324	2.51418, 4	3	heg1	12114
GL174420.1	42583	43685	43259, 43251, 43248	14, 16, 10	16.43009, 17.16856, 13.18184	9.78349, 9.82359, 8.33771	13.33671,	3	ift172	14596
GL174421.1	12256	13288	12642, 12377, 12775	14, 16, 11	5.86316, 6.59801, 6.85425	3.58683, 3.71333, 4.60603	3.46142, 4	3	NA	NA
GL174424.1	3294	3958	3580, 3682, 3604	11, 13, 9	10.66406, 11.2197, 8.26067	6.96705, 6.92276, 5.80842	7.86396, 8	3	NA	0
GL174428.1	18404	18926	18787, 18764, 18798	16, 25, 9	19.7934, 26.96634, 10.64485	11.29109, 12.9658, 7.11743	16.57067,	3	NA	NA
GL174430.1	30425	30940	30484, 30910, 30718	6, 13, 4	5.96715, 13.14514, 4.67325	4.64927, 8.09002, 3.78987	3.52158, 1	3	NA	NA
GL174434.1	4606	5237	4797, 4994, 4733	9, 9, 5	9.72082, 8.21099, 5.9506	6.64182, 5.77858, 4.54784	6.97155, 5	3	ptgfrn	8146
GL174435.1	11029	11770	11287, 11451, 11271	7, 7, 4	6.60038, 5.68499, 4.67325	4.99378, 4.44244, 3.78987	4.10761, 3	3	pafah2	0
GL174436.1	4660	5210	4938, 4783, 4802	10, 8, 6	8.66311, 6.59014, 6.99807	5.914, 4.90493, 5.17195	5.99101, 4	3	chtop	1825
GL174436.1	29269	29870	29709, 29634, 29623	6, 11, 6	5.96715, 10.60895, 7.29403	4.64927, 6.9343, 5.30581	3.52158, 7	3	chtop	15089
GL174437.1	14094	14382	14235, 14246, 14256	20, 26, 12	18.5756, 24.04147, 12.20749	9.56551, 10.87677, 7.71596	15.39539,	3	NA	NA
GL174437.1	18954	19511	19305, 19336, 19470	30, 43, 24	19.25, 30.06402, 14.01785	7.21977, 8.97624, 6.01104	16.04693,	3	NA	NA
GL174437.1	24080	25189	24388, 24380, 24390	142, 139, 123	120.35403, 109.48012, 106.40667	15.52168, 13.57697, 15.7702	115.80229	3	NA	NA
GL174441.1	89	1896	896, 378, 246	30, 37, 17	31.02926, 39.21871, 19.63801	13.53719, 15.52111, 11.0208	27.49872,	3	NA	NA

GL174441.1	9397	10235	9659, 10006, 9666	18, 14, 22	21.99239, 14.45897, 29.24652	12.12048, 8.66788, 15.17695	18.69793,	3	NA	NA
GL174441.1	15503	16670	16246, 16043, 16285	18, 31, 25	20.45461, 36.71719, 32.55444	11.25597, 16.51936, 16.21129	17.20992,	3	NA	NA
GL174441.1	22664	23471	23285, 22750, 23178	9, 9, 7	9.72082, 8.21099, 8.69489	6.64182, 5.77858, 6.06379	6.97155, 5	3	NA	NA
GL174441.1	28933	29991	29441, 29246, 29180	27, 23, 17	32.16072, 27.10783, 20.37708	15.33749, 13.71666, 11.43318	28.60555,	3	NA	NA
GL174444.1	418	4684	564, 1286, 3950	21, 38, 14	14.12513, 25.54947, 10.68804	6.70941, 8.22791, 6.36966	11.1313, 2	3	NA	1237
GL174444.1	457	4705	2259, 1286, 3950	19, 38, 14	11.71885, 25.54947, 10.68804	5.85381, 8.22791, 6.36966	8.85223, 2	3	NA	1216
GL174445.1	3681	4424	3981, 4160, 4301	25, 28, 12	27.62793, 27.61719, 12.15007	13.3608, 12.27209, 7.68238	24.17701,	3	NA	NA
GL174445.1	15495	16428	15671, 16059, 16059	23, 56, 18	20.4429, 53.69389, 18.47005	9.74823, 15.65514, 10.08686	17.19943,	3	NA	NA
GL174445.1	22386	23926	22715, 22695, 22547	24, 27, 15	25.81478, 26.14468, 16.15968	12.62377, 11.7641, 9.45524	22.41065,	3	NA	NA
GL174445.1	28986	29910	29198, 29162, 29352	27, 39, 15	27.24252, 39.54327, 16.75372	12.41362, 14.8875, 9.79627	23.80018,	3	NA	NA
GL174453.1	15276	15942	15721, 15771, 15688	13, 21, 10	8.97001, 14.64109, 9.40066	5.53188, 6.9911, 6.36205	6.27965, 1	3	NA	7947
GL174453.1	19413	19989	19610, 19651, 19733	16, 20, 6	12.26603, 14.27659, 5.1729	6.85709, 7.03519, 4.19138	9.37251, 1	3	NA	12084
GL174454.1	25378	26016	25510, 25624, 25526	5, 7, 5	4.82177, 5.52121, 4.99237	3.98509, 4.33979, 4.0781	2.51418, 3	3	dpp10	0
GL174454.1	34305	34984	34737, 34399, 34447	4, 7, 5	3.74195, 4.77112, 4.67395	3.32091, 3.85834, 3.90232	1.6222, 2.6	3	dpp10	0
GL174454.1	41077	41490	41256, 41243, 41214	10, 7, 5	9.661, 5.2482, 4.67395	6.51661, 4.16649, 3.90232	6.9283, 3.0	3	dpp10	2264
GL174454.1	44806	45670	45412, 45436, 44922	9, 7, 5	6.89504, 3.92514, 4.67395	4.9633, 3.30207, 3.90232	4.38035, 1	3	dpp10	5993
GL174456.1	19349	19967	19748, 19452, 19776	9, 13, 6	4.99103, 5.66936, 4.28038	3.74759, 3.6087, 3.63104	2.67131, 3	3	NA	NA
GL174456.1	26745	27259	27053, 27012, 26923	12, 23, 7	8.74041, 16.72294, 6.1499	5.59287, 7.62665, 4.72762	6.06392, 1	3	NA	NA
GL174457.1	28671	29388	29062, 29266, 29029	21, 20, 12	27.28066, 22.4484, 16.36823	14.34912, 11.88633, 9.85365	23.83737,	3	NA	NA
GL174459.1	19053	19743	19392, 19445, 19359	11, 7, 6	10.9046, 4.53822, 6.71074	7.10903, 3.70607, 5.03498	8.08987, 2	3	NA	NA
GL174462.1	27959	29082	28103, 28101, 28408	25, 34, 12	33.75242, 45.09532, 16.36823	16.86346, 20.22505, 9.85365	30.16411,	3	NA	NA
GL174467.1	23462	24284	23850, 23765, 24051	9, 15, 8	9.63662, 15.7813, 8.1956	6.59633, 9.23437, 5.85348	6.90556, 1	3	cfap44	0
GL174468.1	15384	15653	15602, 15613, 15599	82, 97, 49	83.95745, 106.35479, 55.37701	19.72542, 23.55399, 19.46692	79.72134,	3	hist1h4c	1931
GL174468.1	20321	20667	20606, 20606, 20607	93, 60, 42	104.67295, 58.70022, 47.5857	24.4921, 16.56, 18.20277	100.24541	3	hist1h4c	6868
GL174471.1	11341	12303	11991, 11805, 11888	9, 10, 6	9.72082, 9.39128, 7.29403	6.64182, 6.35644, 5.30581	6.97155, 6	3	NA	5239
GL174472.1	9151	10236	9530, 10149, 9613	13, 13, 7	15.29169, 12.37541, 8.69489	9.29854, 7.62989, 6.06379	12.24396,	3	NA	NA
GL174473.1	15692	16143	15975, 16037, 15965	11, 8, 12	12.43693, 7.07159, 16.36823	7.97018, 5.20073, 9.85365	9.52692, 4	3	NA	NA
GL174476.1	20032	20852	20443, 20188, 20284	13, 15, 9	15.29169, 13.69427, 11.64359	9.29854, 7.97895, 7.57973	12.24396,	3	NA	NA
GL174481.1	21830	22250	22037, 22076, 22034	13, 13, 9	9.25432, 8.12599, 6.72209	5.7064, 5.01963, 4.8535	6.54596, 5	3	NA	0
GL174481.1	26053	26507	26311, 26280, 26084	10, 10, 9	7.78798, 6.11993, 6.91833	5.36798, 4.31286, 4.97804	5.18872, 3	3	NA	3797
GL174483.1	27550	28520	28341, 28345, 28330	9, 16, 13	9.72082, 17.16856, 18.01082	6.64182, 9.82359, 10.61163	6.97155, 1	3	znf630	7723
GL174505.1	22977	23667	23425, 23245, 23102	16, 17, 8	16.30427, 14.61162, 6.97106	9.31205, 8.02205, 5.13965	13.21554,	3	NA	NA
GL174505.1	25759	26509	26165, 26345, 26306	10, 10, 9	9.54985, 8.28331, 9.30116	6.45088, 5.67853, 6.41174	6.82341, 5	3	NA	NA
GL174507.1	13763	14722	14023, 14354, 14370	13, 24, 11	12.38021, 20.79901, 10.82241	7.63279, 9.64095, 7.0607	9.48056, 1	3	NA	NA
GL174507.1	18168	20370	18276, 19834, 19677	11, 27, 16	5.51277, 21.0927, 14.22317	3.78886, 8.89776, 8.04957	3.14574, 1	3	NA	NA
GL174507.1	18168	20370	19289, 19834, 19677	23, 27, 16	17.09886, 21.0927, 14.22317	7.83295, 8.89776, 8.04957	13.97532,	3	NA	NA
GL174511.1	4343	5023	4974, 4905, 4905	15, 15, 5	13.30906, 10.12746, 4.70718	7.74297, 5.80768, 3.9211	10.35786,	3	NA	NA
GL174511.1	13285	14417	14247, 13735, 13202, 14029	9, 12, 5, 9	6.86176, 7.67303, 4.07202, 8.79325	4.9422, 4.93227, 3.54571, 6.12267	4.3506, 5.2	3	NA	NA
GL174519.1	26492	27617	26915, 27121, 26581	20, 23, 10	21.98313, 27.36982, 13.18184	11.61252, 13.8686, 8.33771	18.6897, 2	3	NA	NA
GL174521.1	37	559	405, 405, 413	35, 35, 25	37.16156, 37.7818, 31.40622	15.1516, 15.51397, 15.56799	33.50679,	3	puf60	5737
GL174522.1	719	1075	980, 1048, 1047	12, 16, 7	13.8483, 17.16856, 8.69489	8.63436, 9.82359, 6.06379	10.86694,	3	NA	NA
GL174523.1	199	1324	1119, 382, 1134	9, 6, 4	9.72082, 4.93312, 4.67325	6.64182, 4.04501, 3.78987	6.97155, 2	3	NA	NA
GL174530.1	7051	8207	7239, 7250, 7370	8, 16, 6	8.42257, 15.94397, 7.29403	5.97763, 9.09593, 5.30581	5.76336, 1	3	NA	NA
GL174531.1	996	2416	1146, 2278, 2275	12, 38, 14	8.45074, 33.53299, 13.93205	5.41264, 12.00336, 8.35526	5.79047, 3	3	NA	NA
GL174531.1	1227	2418	2255, 2278, 2275	20, 38, 14	16.8497, 33.53299, 13.93205	8.5297, 12.00336, 8.35526	13.73601,	3	NA	NA
GL174533.1	6104	6344	6135, 6154, 6130	18, 31, 9	19.34385, 31.94142, 9.64095	10.60746, 13.68968, 6.59867	16.13713,	3	NA	3448
GL174534.1	26081	27156	26756, 26493, 26768	9, 10, 7	8.77314, 8.12616, 8.08824	6.111, 5.58036, 5.7849	6.09355, 5	3	slc38a5	2121
GL174535.1	22372	23679	22559, 22662, 23454	7, 13, 3	6.64707, 11.71436, 3.47371	5.02073, 7.22722, 3.03189	4.15135, 9	3	ska1	1916
GL174535.1	23229	23890	23694, 22662, 23748, 23454	7, 13, 7, 3	6.84137, 11.71436, 5.42726, 3.47371	5.13151, 7.22722, 4.28044, 3.03189	4.33107, 9	3	ska1	2773
GL174540.1	14656	16272	15619, 14719, 16125	8, 9, 5	8.42257, 8.21099, 5.50079	5.97763, 5.77858, 4.33868	5.76336, 5	3	NA	NA
GL174540.1	21087	21751	21528, 21436, 21483	11, 12, 11	8.0281, 7.57024, 8.6676	5.34048, 4.86932, 5.74212	5.41287, 5	3	NA	NA
GL174540.1	22864	24248	23481, 23419, 23696	11, 18, 14	7.74505, 12.81028, 11.30499	5.16265, 6.68975, 6.74956	5.14926, 1	3	NA	NA
GL174541.1	4920	5251	5049, 5031, 5107	23, 20, 10	16.52056, 11.34722, 6.97437	7.51649, 5.46101, 4.85319	13.42377,	3	NA	NA
GL174541.1	26099	26759	26558, 26557, 26575	21, 27, 15	20.87917, 25.93224, 15.48721	10.64659, 11.63911, 9.06094	17.62177,	3	NA	NA
GL174544.1	2423	2883	2828, 2854, 2857	14, 23, 20	14.99044, 22.312, 25.71506	8.97777, 10.86102, 13.72384	11.95856,	3	NA	0
GL174546.1	161	861	474, 502, 436	5, 14, 6	4.82177, 14.45897, 7.12188	3.98509, 8.66788, 5.22884	2.51418, 1	3	NA	NA
GL174561.1	13214	14112	13847, 13791, 13566	18, 26, 19	18.92486, 21.60497, 20.47556	10.35878, 9.47806, 11.00494	15.73261,	3	NA	NA
GL174573.1	6511	7222	6586, 7073, 6792	10, 6, 8	8.48936, 4.51135, 9.31951	5.80661, 3.77984, 6.43944	5.82603, 2	3	arfip2	0
GL174574.1	3875	4528	4254, 4292, 4349	14, 17, 6	12.38175, 15.07756, 5.56953	7.4133, 8.30587, 4.42554	9.48175, 1	3	prkca	3606
GL174576.1	7499	7988	7840, 7903, 7890	13, 29, 16	6.06866, 17.62155, 12.29567	3.82788, 6.68988, 6.87499	3.6163, 14	3	NA	NA
GL174579.1	2158	2615	2482, 2494, 2380	12, 17, 12	13.8483, 18.56115, 16.36823	8.63436, 10.40145, 9.85365	10.86694,	3	NA	NA
GL174581.1	70	863	535, 510, 213	5, 11, 10	4.82177, 10.60895, 13.18184	3.98509, 6.9343, 8.33771	2.51418, 7	3	NA	NA
GL174582.1	10678	11279	11161, 11140, 11242	8, 9, 7	8.05102, 7.35794, 8.21885	5.77295, 5.25474, 5.84717	5.43473, 4	3	NA	NA
GL174583.1	49	1132	983, 1007, 1010	33, 35, 18	26.90793, 26.86157, 15.56767	10.18215, 9.57384, 8.32867	23.47509,	3	NA	NA
GL174583.1	1884	2545	1986, 1964, 1926	16, 23, 12	9.23292, 14.35816, 8.90997	5.09107, 6.38256, 5.69856	6.52626, 1	3	NA	NA
GL174583.1	17265	18188	17894, 17826, 17896	9, 31, 16	9.72082, 37.21764, 20.763	6.64182, 16.81517, 11.79066	6.97155, 3	3	NA	NA
GL174583.1	24536	25562	25440, 24866, 24847	14, 23, 13	14.38232, 22.17331, 13.33541	8.62271, 10.77791, 8.20195	11.37721,	3	NA	NA
GL174599.1	90	454	234, 248, 341	20, 31, 14	19.70489, 32.72958, 13.43751	10.24795, 14.15394, 8.05781	16.48628,	3	NA	4806
GL174599.1	4475	5356	4687, 4762, 4911	9, 13, 14	5.44005, 8.44264, 12.33709	4.03358, 5.21062, 7.38584	3.07958, 5	3	NA	0
GL174599.1	15535	16216	15735, 15748, 15824	45, 53, 24	34.94186, 42.78308, 19.17242	10.54425, 11.70814, 8.70968	31.32996,	3	NA	1336
GL174599.1	20126	20791	20740, 20745, 20202	91, 106, 18	91.25257, 110.79589, 12.23264	19.51152, 21.94299, 6.35837	86.94301,	3	NA	5927
GL174601.1	11	846	419, 416, 416	269, 346, 233	303.58237, 390.26941, 282.55136	30.74147, 31.63876, 35.71319	298.30191	3	5S_rRNA	0
GL174601.1	21408	22446	21747, 21752, 21538	8, 20, 6	6.94372, 18.1164, 5.91225	5.12297, 9.28853, 4.61907	4.42528, 1	3	slc39a4	0
GL174601.1	21408	22632	22465, 21752, 21538	8, 20, 6	7.06928, 18.1164, 5.91225	5.19933, 9.28853, 4.61907	4.5414, 15	3	slc39a4	0
GL174604.1	12457	13518	12771, 12928, 12598	11, 17, 6	12.09119, 16.49924, 7.29403	7.78314, 9.17114, 5.30581	9.20684, 1	3	tmem256	9669
GL174607.1	4405	5723	5018, 5451, 4997	8, 15, 12	7.47668, 14.39783, 14.06375	5.44269, 8.40758, 8.74831	4.89713, 1	3	NA	NA
GL174610.1	6324	7140	6795, 6795, 6847	16, 29, 13	15.47689, 28.50226, 14.5443	8.81381, 12.42647, 8.89247	12.4218, 2	3	NA	25604
GL174611.1	21841	24711	22144, 22401, 22520	9, 17, 10	6.11771, 12.93592, 9.01123	4.4675, 7.01058, 6.12719	3.66161, 1	3	NA	NA
GL174611.1	22010	24711	24082, 22401, 22520	17, 17, 10	16.23509, 12.93592, 9.01123	9.01086, 7.01058, 6.12719	13.14931,	3	NA	NA
GL174616.1	20051	21045	20934, 21000, 21007	8, 8, 13	6.70593, 4.63504, 13.21599	4.9768, 3.65065, 8.13179	4.20487, 2	3	NA	NA
GL174620.1	4422	4923	4668, 4849, 4544	11, 18, 4	12.43693, 19.08138, 4.67325	7.97018, 10.4519, 3.78987	9.52692, 1	3	epha8	8123
GL174626.1	12166	12776	12378, 12473, 12430	7, 17, 5	5.21879, 13.21469, 4.22765	4.14768, 7.17728, 3.64077	2.87895, 1	3	NA	NA
GL174628.1	49349	49587	49502, 49478, 49471	25, 56, 25	33.57819, 70.76226, 33.39399	16.76992, 24.83735, 16.67053	29.99315,	3	zgpat	4265
GL174631.1	22571	23430	23037, 22781, 23028	107, 116, 55	106.22699, 109.68372, 53.46325	19.89332, 18.42488, 15.91645	101.7869,	3	NA	NA
GL174632.1	6887	7419	7052, 7044, 7071	13, 21, 8	12.20544, 17.80261, 9.25066	7.52692, 8.80213, 6.40569	9.31472, 1	3	fbln1	126
GL174632.1	18540	19501	19038, 19210, 19289	13, 19, 9	14.43911, 20.29855, 10.87975	8.83395, 10.89984, 7.23113	11.43182,	3	fbln1	0
GL174633.1	3073	3481	3164, 3325, 3149	8, 9, 3	8.42257, 8.21099, 3.47371	5.97763, 5.77858, 3.03189	5.76336, 5	3	NA	NA
GL174633.1	10103	10443	10182, 10181, 10183	8, 16, 5	8.42257, 16.37065, 5.9506	5.97763, 9.3517, 4.54784	5.76336, 1	3	NA	NA
GL174634.1	1990	2679	2266, 2283, 2431	10, 13, 6	8.61898, 8.96044, 4.7248	5.88679, 5.52602, 3.91518	5.94907, 6	3	kcnd1	9427
GL174634.1	22958	24180	23612, 23616, 23626	22, 31, 20	23.01801, 29.20136, 22.4589	11.61387, 12.10186, 11.89249	19.69328,	3	kcnd1	956
GL174635.1	3851	5244	5048, 4920, 4176, 5019	9, 40, 5, 6	8.25643, 42.7533, 5.59755, 6.87953	5.80588, 16.29291, 4.38538, 5.11627	5.62516, 3	3	NA	NA
GL174635.1	23365	23934	23720, 23434, 23699	8, 7, 3	8.42257, 5.97725, 3.47371	5.97763, 4.62287, 3.03189	5.76336, 3	3	NA	NA
GL174636.1	16134	16511	16168, 16155, 16175	26, 43, 20	27.0252, 43.72249, 22.98257	12.64525, 15.58556, 12.19768	23.58893,	3	NA	NA
GL174636.1	20309	20690	20346, 20311, 20335	31, 47, 24	33.7354, 49.35003, 27.62704	14.74934, 17.00243, 13.70066	30.14729,	3	NA	NA
GL174637.1	16980	17706	17381, 17315, 17487	12, 15, 5	13.73771, 15.80067, 5.9506	8.57523, 9.24574, 4.54784	10.76705,	3	NA	NA
GL174638.1	12023	14168	13485, 13167, 13772, 13102	13, 8, 10, 10	11.16633, 5.28564, 7.17119, 9.82971	6.8898, 4.07288, 4.97797, 6.61563	8.32764, 3	3	NA	NA
GL174640.1	21770	22278	21921, 22085, 21862	13, 11, 8	12.68462, 8.74006, 8.9249	7.81602, 5.78751, 6.24211	9.76298, 6	3	NA	NA
GL174641.1	78	1551	717, 743, 825	156, 199, 128	161.44095, 200.0829, 135.64723	23.3071, 22.78396, 23.55258	156.6512,	3	5S_rRNA	0
GL174644.1	18099	19103	18494, 18841, 18488	14, 25, 16	8.11589, 15.71703, 12.90039	4.82784, 6.62908, 7.24159	5.49454, 1	3	NA	NA
GL174644.1	22966	23921	23218, 23338, 23144, 23843	10, 15, 6, 6	6.22053, 9.2091, 4.17587, 5.29847	4.37622, 5.2699, 3.56299, 4.26663	3.75715, 6	3	NA	NA
GL174647.1	43957	44593	43994, 44093, 44052	8, 8, 7	8.10827, 6.37769, 7.50542	5.80497, 4.77199, 5.49244	5.48761, 4	3	NA	NA
GL174647.1	55844	56039	55870, 55872, 55892	11, 27, 14	12.43693, 33.60851, 19.68329	7.97018, 16.18004, 11.3696	9.52692, 3	3	NA	NA
GL174648.1	10257	10850	10667, 10390, 10762	5, 7, 4	4.82177, 5.97725, 4.67325	3.98509, 4.62287, 3.78987	2.51418, 3	3	NA	NA
GL174651.1	22734	23656	23085, 23334, 23354	22, 29, 14	27.73229, 34.61861, 19.03529	14.35711, 16.05165, 11.08274	24.27873,	3	NA	20913
GL174652.1	122	1481	922, 965, 334, 877	23, 32, 10, 13	18.85624, 23.96297, 8.39789, 11.59311	8.82358, 8.97946, 5.74985, 7.1528	15.66578,	3	NA	NA
GL174654.1	14496	14884	14830, 14857, 14847	9, 16, 13	7.67537, 14.92393, 15.79818	5.45199, 8.47764, 9.56343	5.08317, 1	3	NA	2616
GL174656.1	15645	16324	15844, 15896, 15980	7, 9, 3	7.1696, 8.21099, 3.47371	5.31345, 5.77858, 3.03189	4.61046, 5	3	NA	NA
GL174657.1	22225	23642	23216, 23307, 22970	8, 14, 7	7.38183, 14.44087, 7.50542	5.38666, 8.65722, 5.49244	4.80856, 1	3	NA	NA
GL174659.1	7681	8362	7825, 8125, 7824	16, 16, 8	14.23891, 12.57119, 8.35103	8.05921, 7.04167, 5.93881	11.24094,	3	trpm4	0
GL174659.1	11857	12135	12066, 11911, 12047	9, 7, 5	6.57543, 4.40767, 4.45433	4.76012, 3.62034, 3.77574	4.08545, 2	3	trpm4	0
GL174661.1	20481	21009	20709, 20536, 20537	18, 16, 13	19.60679, 14.25068, 14.39632	10.76248, 8.0664, 8.81006	16.39143,	3	NA	NA
GL174665.1	7654	8193	8074, 7911, 7982	7, 19, 6	7.1696, 19.64509, 6.99807	5.31345, 10.50948, 5.17195	4.61046, 1	3	rfx5	0
GL174676.1	21183	21631	21419, 21244, 21263	4, 7, 5	3.28906, 5.13485, 4.57714	3.0555, 4.09383, 3.84705	1.37521, 2	3	NA	NA
GL174676.1	22483	22740	22572, 22526, 22516	7, 11, 12	6.42049, 9.30635, 13.29687	4.8888, 6.14075, 8.33527	3.94161, 6	3	NA	NA
GL174678.1	2315	2787	2417, 2443, 2422	9, 13, 5	9.72082, 13.14514, 5.9506	6.64182, 8.09002, 4.54784	6.97155, 1	3	NA	NA
GL174679.1	35	1159	516, 728, 361	9, 13, 5	9.70623, 13.14514, 5.9506	6.63396, 8.09002, 4.54784	6.97147, 1	3	NA	NA
GL174681.1	52	614	428, 444, 423	157, 254, 91	163.57906, 269.6806, 95.64993	23.66706, 26.50311, 21.32766	158.77954	3	NA	NA
GL174681.1	7045	7693	7179, 7309, 7173	17, 28, 14	11.99445, 20.27686, 10.65572	6.45443, 8.19499, 6.3498	9.11496, 1	3	NA	NA
GL174681.1	20398	20972	20526, 20647, 20811	7, 12, 5	7.1696, 11.84549, 5.9506	5.31345, 7.50292, 4.54784	4.61046, 9	3	NA	NA
GL174684.1	22359	22668	22393, 22388, 22389	10, 14, 19	11.06011, 14.45897, 25.95226	7.306, 8.66788, 14.01451	8.22712, 1	3	NA	NA
GL174693.1	3351	3868	3663, 3610, 3627	17, 26, 17	14.34561, 21.2576, 13.98836	7.8603, 9.28371, 7.64359	11.34222,	3	zbtb7b	6639
GL174694.1	137	809	295, 492, 667	9, 14, 7	7.97705, 11.53986, 6.59055	5.637, 6.89446, 4.98809	5.36577, 8	3	NA	NA
GL174694.1	3250	4313	3873, 3551, 3873	15, 18, 17	11.42908, 11.49098, 15.38528	6.59091, 5.94126, 8.49346	8.57789, 8	3	NA	NA
GL174694.1	6677	7837	7375, 7264, 7255	21, 35, 19	13.26501, 25.14591, 15.3154	6.2504, 8.76011, 7.90001	10.31605,	3	NA	NA
GL174694.1	10147	11131	10812, 10656, 10533	35, 33, 19	24.21256, 20.41869, 12.84079	8.33417, 7.10916, 6.46981	20.85357,	3	NA	NA
GL174694.1	12483	13072	12791, 12805, 12767	14, 29, 18	5.52351, 16.99076, 11.60247	3.41144, 6.4098, 6.00332	3.15454, 1	3	NA	NA
GL174694.1	14102	14810	14447, 14178, 14448	39, 22, 30	28.22733, 11.12243, 24.59827	9.15087, 5.00529, 9.8869	24.76293,	3	NA	NA
GL174694.1	16475	17607	17164, 17080, 17233	20, 23, 21	11.34406, 13.80453, 15.24463	5.45941, 6.10589, 7.32621	8.49725, 1	3	NA	NA
GL174694.1	21284	23056	22073, 22064, 22538	29, 35, 19	25.83659, 32.07569, 19.4202	10.89173, 12.27045, 10.3744	22.43173,	3	NA	NA
GL174695.1	1640	2084	1922, 1932, 1932	9, 20, 9	9.72082, 22.87459, 11.64359	6.64182, 12.13503, 7.57973	6.97155, 1	3	NA	NA
GL174698.1	74	734	481, 149, 143	6, 13, 9	5.1345, 12.61502, 10.14484	4.16817, 7.77427, 6.86555	2.80161, 9	3	cars	2275
GL174698.1	5288	5976	5358, 5379, 5545	11, 13, 8	9.85927, 11.07988, 8.04677	6.48161, 6.83637, 5.77057	7.10352, 8	3	cars	0
GL174698.1	22503	22989	22729, 22555, 22700	27, 23, 23	28.86948, 25.04419, 28.93723	13.38311, 12.49946, 14.76095	25.38913,	3	cars	5786
GL174699.1	7995	8812	8201, 8259, 8184, 8738	11, 12, 4, 6	11.15818, 11.86108, 4.64325, 7.29403	7.25692, 7.51216, 3.77643, 5.30581	8.32008, 9	3	NA	NA
GL174699.1	22367	22827	22496, 22555, 22621	7, 9, 3	7.1696, 8.21099, 3.47371	5.31345, 5.77858, 3.03189	4.61046, 5	3	NA	NA
GL174702.1	438	1208	985, 972, 966	29, 50, 25	32.65865, 55.54604, 32.02895	14.88989, 19.1027, 15.91894	29.09305,	3	NA	NA
GL174708.1	37	1275	563, 991, 546	26, 34, 31	26.68953, 33.22479, 34.68353	12.44476, 13.27919, 15.31231	23.26214,	3	U1	0
GL174708.1	6190	7173	6452, 6878, 6251, 7064	16, 20, 14, 16	9.88365, 10.71125, 8.17828, 9.18585	5.45645, 5.14204, 4.86395, 5.06499	7.12699, 8	3	U1	0
GL174708.1	6190	7173	6845, 6878, 6251, 7064	9, 20, 14, 16	3.61216, 10.71125, 8.17828, 9.18585	2.89053, 5.14204, 4.86395, 5.06499	1.6222, 8.0	3	U1	0
GL174710.1	57	753	401, 368, 455	9, 23, 10	9.72082, 26.22929, 13.18184	6.64182, 13.2024, 8.33771	6.97155, 2	3	NA	NA
GL174714.1	18465	19182	18539, 18918, 18705	5, 14, 9	3.81096, 12.28739, 11.04428	3.3822, 7.35528, 7.30896	1.68502, 9	3	NA	256
GL174715.1	34	1055	466, 592, 284	30, 38, 20	34.94212, 36.31646, 23.93369	15.8601, 13.48883, 12.7427	31.33019,	3	NA	NA
GL174715.1	11589	13486	12479, 12584, 12580	42, 51, 24	42.68002, 49.75688, 27.25833	15.41891, 15.59556, 13.48302	38.93535,	3	NA	NA
GL174715.1	18879	19651	19158, 19450, 19216	7, 17, 5	7.1696, 18.56115, 5.9506	5.31345, 10.40145, 4.54784	4.61046, 1	3	NA	NA
GL174716.1	282	1014	634, 842, 533	11, 19, 11	9.34791, 16.05817, 12.55982	6.16652, 8.3432, 8.03554	6.63394, 1	3	NA	NA
GL174719.1	11802	12607	12023, 12021, 12100, 12399	6, 8, 4, 4	5.27951, 6.41216, 4.30163, 4.15126	4.25534, 4.79364, 3.61556, 3.54016	2.9335, 4.0	3	NA	NA
GL174719.1	18280	20695	18577, 19270, 20417, 18349, 19961	12, 6, 10, 6, 4	10.58505, 3.78933, 7.97488, 6.45058, 4.59709	6.73997, 3.30809, 5.48553, 4.90508, 3.75554	7.78888, 1	3	NA	NA
GL174725.1	547	1267	911, 729, 881	7, 14, 5	7.1696, 14.45897, 5.23257	5.31345, 8.66788, 4.20435	4.61046, 1	3	arhgap30	4310
GL174726.1	921	1356	1001, 996, 1000	76, 89, 60	75.3877, 79.95008, 64.16836	18.22499, 15.87703, 19.27381	71.23694,	3	NA	2123
GL174727.1	424	1401	997, 990, 337, 1218	7, 12, 12, 5	5.33678, 10.08092, 14.58408, 5.14967	4.22299, 6.42828, 9.01638, 4.16141	2.98597, 7	3	U4	16802
GL174727.1	2848	3831	3129, 3013, 3148	15, 13, 6	12.70921, 9.96603, 5.46454	7.37467, 6.14603, 4.36458	9.7865, 7.3	3	U4	14372
GL174727.1	8832	10197	8978, 9158, 9270	12, 21, 18	11.55224, 18.7699, 18.87511	7.3281, 9.37748, 10.32913	8.69537, 1	3	U4	8006
GL174727.1	18213	18951	18555, 18568, 18624	11, 13, 8	8.43721, 8.58102, 6.35764	5.59757, 5.29456, 4.75938	5.77761, 6	3	U4	0
GL174727.1	19872	22368	20123, 21152, 20147, 20808, 22318	13, 26, 12, 12, 9	10.8905, 23.54286, 11.02785, 11.02785, 7.58036	6.71917, 10.58612, 7.01117, 7.01117, 5.39321	8.07746, 2	3	U4	869
GL174727.1	19872	22455	22115, 21152, 20147, 20808, 22318	8, 26, 12, 12, 9	5.66733, 23.54286, 11.02785, 11.02785, 7.58036	4.31947, 10.58612, 7.01117, 7.01117, 5.39321	3.28389, 2	3	U4	869
GL174728.1	14116	14767	14524, 14548, 14185	6, 7, 7	5.09949, 4.5832, 7.50542	4.14694, 3.73555, 5.49244	2.76939, 2	3	NA	193
GL174729.1	5757	6318	6050, 5771, 5787	7, 18, 4	6.46447, 18.29879, 4.59709	4.91463, 9.984, 3.75554	3.98312, 1	3	or51e1	1615
GL174731.1	9282	9983	9671, 9943, 9919	9, 11, 14	9.72082, 10.60895, 19.68329	6.64182, 6.9343, 11.3696	6.97155, 7	3	NA	NA
GL174742.1	10265	11121	10799, 10904, 11008	6, 12, 4	5.43318, 10.25865, 4.55201	4.34622, 6.53846, 3.73489	3.07284, 7	3	NA	NA
GL174744.1	2690	6005	3752, 3281, 3331, 4953	34, 40, 16, 16	21.69463, 23.58165, 9.62403, 8.332	7.45004, 7.05268, 5.30962, 4.60069	18.40989,	3	NA	NA
GL174744.1	6865	7532	7255, 7376, 7492	18, 24, 16	5.47746, 6.75632, 6.74573	3.00883, 3.0524, 3.78587	3.11243, 4	3	NA	NA
GL174744.1	8143	10014	9398, 8504, 9542, 8354	26, 24, 32, 17	13.32577, 8.04577, 15.91109, 8.03264	5.34594, 3.5044, 5.47593, 4.28997	10.37431,	3	NA	NA
GL174744.1	10484	11040	10619, 10624, 10604	27, 44, 30	16.45193, 29.56386, 23.40473	6.5605, 8.55572, 9.25647	13.35798,	3	NA	NA
GL174749.1	357	1285	869, 538, 428, 789	12, 11, 7, 5	11.2086, 10.31711, 8.08824, 5.06966	7.12098, 6.75959, 5.7849, 4.11933	8.36821, 7	3	NA	NA
GL174749.1	4529	5747	5380, 5359, 5517	6, 11, 8	4.7134, 10.21061, 9.3897	3.90801, 6.69533, 6.47354	2.47723, 7	3	NA	NA
GL174754.1	12813	13161	13002, 13045, 13014	10, 11, 16	10.26482, 9.80774, 18.61066	6.86644, 6.45006, 10.64858	7.48624, 7	3	IGSF8	336
GL174754.1	21270	21989	21730, 21785, 21743	12, 13, 5	12.60209, 12.2024, 5.9506	7.9443, 7.52507, 4.54784	9.68421, 9	3	rit1	131
GL174756.1	124	1461	1128, 1122, 1348	21, 33, 15	17.51801, 28.20425, 15.88211	8.63427, 10.86324, 9.29347	14.37772,	3	NA	11520
GL174756.1	12174	12617	12296, 12322, 12266	15, 23, 13	7.61043, 10.33701, 8.016	4.37472, 4.51189, 4.95367	5.02361, 7	3	NA	364
GL174756.1	19032	19811	19194, 19171, 19488	36, 43, 12	17.67596, 18.04573, 4.63574	5.5566, 4.87986, 3.16497	14.53095,	3	NA	0
GL174756.1	20809	21515	21290, 21237, 21294	40, 61, 31	24.38481, 37.34063, 22.9865	7.35405, 8.10983, 8.76492	21.02063,	3	NA	0
GL174761.1	14111	14482	14213, 14267, 14377	7, 6, 5	7.1696, 4.93312, 5.9506	5.31345, 4.04501, 4.54784	4.61046, 2	3	NA	NA
GL174762.1	21373	21983	21526, 21421, 21527	6, 12, 3	5.96715, 11.86108, 3.47371	4.64927, 7.51216, 3.03189	3.52158, 9	3	NA	NA
GL174765.1	20256	20989	20317, 20520, 20655	9, 6, 4	9.50098, 4.93312, 4.67325	6.52233, 4.04501, 3.78987	6.77745, 2	3	NA	NA
GL174767.1	87	1842	719, 748, 1063	24, 35, 20	23.97781, 32.81274, 20.63833	11.52292, 12.67532, 10.81054	20.62537,	3	NA	NA
GL174768.1	3053	3624	3545, 3577, 3557	16, 20, 8	14.85827, 17.23653, 8.9249	8.4376, 8.76036, 6.24211	11.83222,	3	gatad2b	39
GL174769.1	20982	21592	21374, 21437, 21381	15, 9, 6	18.26603, 8.21099, 7.29403	10.62691, 5.77858, 5.30581	15.09729,	3	NA	NA
GL174772.1	20710	21131	20802, 20752, 20767	16, 30, 13	16.75964, 34.4899, 13.45746	9.5829, 15.5917, 8.27333	13.65293,	3	NA	NA
GL174774.1	14	447	353, 420, 410	13, 13, 13	15.29169, 13.14514, 18.01082	9.29854, 8.09002, 10.61163	12.24396,	3	vax1	4669
GL174776.1	89	505	417, 323, 253	6, 8, 5	5.96715, 7.07159, 5.9506	4.64927, 5.20073, 4.54784	3.52158, 4	3	NA	NA
GL174776.1	7908	8753	8561, 8519, 8338	23, 43, 31	22.28866, 41.24942, 33.30647	10.84702, 14.25229, 14.49511	18.98693,	3	NA	NA
GL174777.1	14726	15303	15206, 15210, 14998	7, 11, 5	6.79162, 10.59463, 5.9506	5.10336, 6.92577, 4.54784	4.28406, 7	3	NA	NA
GL174786.1	2316	3174	2852, 3076, 2517	10, 26, 11	11.04401, 25.18647, 12.78973	7.29736, 11.55067, 8.15619	8.22236, 2	3	NA	NA
GL174786.1	21179	21511	21389, 21470, 21509	8, 14, 14	8.42257, 14.45897, 18.52583	5.97763, 8.66788, 10.84669	5.76336, 1	3	NA	NA
GL174794.1	13853	14195	14075, 14073, 13997	23, 56, 22	23.56695, 62.07519, 26.58401	11.61446, 19.94958, 13.71123	20.22607,	3	NA	NA
GL174795.1	14261	14836	14564, 14770, 14667	13, 7, 8	15.29169, 5.97725, 10.1465	9.29854, 4.62287, 6.82176	12.24396,	3	NA	NA
GL174796.1	195	953	431, 387, 634	5, 12, 13	4.82177, 11.86108, 18.01082	3.98509, 7.51216, 10.61163	2.51418, 9	3	NA	NA
GL174801.1	16854	17348	17107, 16971, 17062	11, 16, 13	12.43693, 15.10361, 17.28449	7.97018, 8.58708, 10.28792	9.52692, 1	3	NA	NA
GL174802.1	20960	21458	21028, 21308, 21276	7, 11, 6	6.99564, 8.77819, 6.76586	5.21786, 5.81139, 5.06179	4.47307, 6	3	NA	NA
GL174803.1	6795	7188	6853, 6943, 6846	10, 10, 7	11.06011, 9.39128, 8.69489	7.306, 6.35644, 6.06379	8.22712, 6	3	NA	31783
GL174808.1	15311	15829	15497, 15801, 15623	11, 25, 11	11.02968, 30.17196, 13.19894	7.18222, 14.85972, 8.36554	8.20839, 2	3	NA	NA
GL174810.1	150	1698	1075, 1300, 168, 1266	14, 30, 11, 17	9.70564, 21.63554, 7.74126, 14.1481	5.77015, 8.35775, 5.16027, 7.74041	6.97127, 1	3	NA	NA
GL174810.1	7557	8307	8090, 8034, 7958	21, 21, 9	20.17379, 18.88456, 9.41119	10.2212, 9.44608, 6.47287	16.93778,	3	NA	NA
GL174810.1	36890	37654	36996, 37347, 37095	23, 26, 11	16.40937, 14.3904, 7.29725	7.45624, 5.81113, 4.88201	13.317, 11	3	NA	NA
GL174810.1	38941	39451	39190, 39144, 39187	11, 30, 12	5.41054, 18.029, 8.25124	3.72812, 6.67204, 5.28884	3.05259, 1	3	NA	NA
GL174810.1	46019	46835	46331, 46270, 46354	56, 108, 56	60.56705, 116.70152, 65.13881	19.14049, 23.54611, 21.63407	56.57353,	3	NA	NA
GL174811.1	19186	19637	19469, 19205, 19326	6, 7, 5	4.5407, 4.5832, 5.91431	3.79858, 3.73555, 4.53172	2.33, 2.452	3	NA	2672
GL174825.1	5109	6466	5292, 6073, 5732	5, 19, 6	3.83692, 17.23199, 7.29403	3.39868, 9.05085, 5.30581	1.70915, 1	3	abcc9	0
GL174825.1	5109	6466	6122, 6073, 5732	5, 19, 6	3.61427, 17.23199, 7.29403	3.25593, 9.05085, 5.30581	1.6222, 14	3	abcc9	0
GL174830.1	135	860	407, 610, 382	9, 12, 15	8.0679, 11.86108, 16.59998	5.69219, 7.51216, 9.70872	5.45, 9.14	3	NA	NA
GL174830.1	16335	17338	16892, 16897, 16885	50, 92, 28	35.87727, 71.50014, 21.35929	9.68558, 12.47047, 8.75554	32.24623,	3	NA	NA
GL174830.1	20531	20900	20798, 20627, 20754	12, 19, 15	4.30232, 6.06891, 9.30985	2.98578, 3.17082, 5.32815	2.12436, 3	3	NA	NA
GL174832.1	11728	12508	12278, 12037, 12234	14, 9, 7	16.7649, 8.21099, 8.69489	9.96273, 5.77858, 6.06379	13.6543, 5	3	NA	0
GL174835.1	20209	20987	20763, 20750, 20737	30, 35, 13	35.30015, 42.30475, 16.28606	16.07224, 18.18441, 9.81016	31.6812, 3	3	NA	NA
GL174836.1	6853	8174	7009, 7573, 7115	5, 18, 7	4.82177, 16.46278, 8.69489	3.98509, 8.87194, 6.06379	2.51418, 1	3	smad4.2	865
GL174836.1	6853	8174	7581, 7573, 7115	9, 18, 7	9.72082, 16.46278, 8.69489	6.64182, 8.87194, 6.06379	6.97155, 1	3	smad4.2	865
GL174845.1	1576	2005	1835, 1731, 1886	6, 9, 7	5.77068, 8.21099, 8.42533	4.54016, 5.77858, 5.94313	3.37745, 5	3	flad1	5672
GL174845.1	6557	7434	7041, 6864, 7033	22, 17, 14	22.93435, 14.4143, 14.1066	11.56366, 7.90204, 8.45936	19.61206,	3	flad1	243
GL174847.1	478	987	485, 674, 525	7, 15, 5	6.79162, 15.80067, 4.57714	5.10336, 9.24574, 3.84705	4.28406, 1	3	nrbp1	4170
GL174850.1	5763	6254	6005, 6068, 6080	9, 8, 4	8.35434, 6.66413, 4.50795	5.86444, 4.9509, 3.71446	5.71584, 4	3	xrra1	0
GL174850.1	19120	19647	19488, 19482, 19465	31, 30, 27	34.03454, 32.42001, 32.38601	14.92684, 14.36101, 15.46959	30.44033,	3	rnf169	0
GL174851.1	8906	9215	8936, 8942, 8932	46, 99, 40	60.5877, 126.73724, 55.63919	23.94062, 32.30242, 23.81452	56.5936, 1	3	NA	NA
GL174859.1	11740	12349	11950, 11923, 12114	7, 8, 7	6.09019, 6.97779, 8.69489	4.69155, 5.14375, 6.06379	3.63504, 4	3	NA	NA
GL174861.1	13938	14316	13969, 13977, 13965	9, 12, 7	9.72082, 11.60251, 8.08824	6.64182, 7.3582, 5.7849	6.97155, 8	3	NA	NA
GL174866.1	197	543	443, 465, 392	11, 15, 7	6.84598, 6.74838, 5.27202	4.6009, 3.91775, 4.18171	4.33559, 4	3	NA	NA
GL174866.1	5649	5911	5832, 5797, 5799	11, 16, 7	5.91397, 6.61216, 4.37639	4.02958, 3.72026, 3.59976	3.50611, 4	3	NA	NA
GL174866.1	12568	13160	12719, 12784, 12837	45, 55, 40	47.25626, 53.12056, 49.17293	16.70065, 15.74899, 20.04263	43.44327,	3	NA	NA
GL174877.1	9494	9980	9737, 9919, 9738	12, 13, 5	10.39353, 11.36332, 5.9506	6.62187, 7.01137, 4.54784	7.60856, 8	3	entpd2	0
GL174879.1	208	1010	641, 597, 882	6, 11, 6	5.96715, 10.60895, 7.29403	4.64927, 6.9343, 5.30581	3.52158, 7	3	NA	5041
GL174879.1	4711	5181	5091, 4877, 4982	7, 9, 10	7.1696, 7.51164, 12.52085	5.31345, 5.35055, 8.03986	4.61046, 5	3	NA	870
GL174885.1	19909	20117	19946, 19957, 19953	7, 15, 8	7.1696, 15.80067, 10.1465	5.31345, 9.24574, 6.82176	4.61046, 1	3	NA	NA
GL174889.1	19581	20411	20171, 20219, 20175	12, 18, 11	11.09909, 16.12237, 12.9493	7.05451, 8.66512, 8.23866	8.26391, 1	3	NA	NA

GL174894.1	18930	19456	19373, 19348, 19134	9, 12, 9	9.63662, 11.84549, 11.64359	6.59633, 7.50292, 7.57973	6.90556, 9	3	NA	NA
GL174895.1	17200	17476	17234, 17261, 17264	8, 6, 4	8.42257, 4.93312, 4.67325	5.97763, 4.04501, 3.78987	5.76336, 2	3	NA	NA
GL174896.1	100	832	388, 421, 421	9, 25, 8	9.72082, 26.42765, 10.1465	6.64182, 12.64351, 6.82176	6.97155, 2	3	NA	NA
GL174899.1	8121	9115	8678, 8678, 8559	13, 11, 10	13.49269, 9.8562, 11.83521	8.29387, 6.47973, 7.70798	10.53271,	3	NA	NA
GL174901.1	140	728	317, 326, 355	11, 16, 13	12.43693, 17.16856, 18.01082	7.97018, 9.82359, 10.61163	9.52692, 1	3	NA	NA
GL174903.1	13224	15202	14523, 14004, 13693, 14252	12, 16, 6, 8	9.30462, 11.4149, 5.20372, 7.42597	5.94479, 6.34731, 4.20995, 5.41279	6.59363, 8	3	NA	NA
GL174904.1	336	1858	1262, 439, 701, 1677	8, 11, 7, 3	8.42257, 10.60895, 8.69489, 3.47371	5.97763, 6.9343, 6.06379, 3.03189	5.76336, 7	3	NA	NA
GL174904.1	8073	10016	8607, 8552, 8878	8, 11, 9	7.28955, 8.59076, 9.41119	5.33177, 5.69393, 6.47287	4.72176, 6	3	NA	NA
GL174904.1	17584	18969	18220, 18166, 18651, 18237	8, 10, 7, 6	8.42257, 9.39128, 5.97725, 7.29403	5.97763, 6.35644, 4.62287, 5.30581	5.76336, 6	3	NA	NA
GL174908.1	15476	15983	15717, 15704, 15529	6, 9, 6	5.96715, 8.21099, 7.29403	4.64927, 5.77858, 5.30581	3.52158, 5	3	NA	NA
GL174917.1	9867	10645	10455, 10211, 10373	10, 14, 5	10.67246, 14.45897, 5.9506	7.09497, 8.66788, 4.54784	7.87096, 1	3	NA	NA
GL174917.1	39914	40241	40119, 40126, 39951	10, 7, 5	9.28413, 5.97725, 5.31857	6.29221, 4.62287, 4.24819	6.574, 3.65	3	NA	NA
GL174926.1	1560	2374	1763, 2099, 2264	6, 11, 8	5.96715, 10.60895, 10.1465	4.64927, 6.9343, 6.82176	3.52158, 7	3	NA	NA
GL174929.1	1876	2415	2083, 2084, 2084	15, 19, 11	18.26603, 20.55804, 14.45778	10.62691, 11.0538, 8.96373	15.09729,	3	NA	NA
GL174930.1	7147	8114	7693, 7088, 7974, 7119, 7774	7, 7, 11, 4, 4	6.94335, 5.97725, 10.60895, 4.46488, 4.38152	5.18876, 4.62287, 6.9343, 3.69425, 3.65448	4.42528, 3	3	commd7	5229
GL174937.1	159	683	277, 314, 587	6, 11, 4	5.96715, 10.60895, 4.50795	4.64927, 6.9343, 3.71446	3.52158, 7	3	NA	NA
GL174937.1	5274	5684	5386, 5478, 5338	5, 8, 7	3.78532, 5.14361, 6.92462	3.36588, 3.9808, 5.17829	1.66163, 2	3	NA	NA
GL174937.1	11710	12609	12178, 11999, 12451	13, 35, 10	13.79066, 37.3034, 12.71002	8.46641, 15.23432, 8.12734	10.8171, 3	3	NA	NA
GL174940.1	3418	4132	3642, 3961, 4032	9, 13, 7	7.28297, 10.07856, 6.63036	5.20782, 6.21577, 5.0111	4.7171, 7.4	3	NA	NA
GL174944.1	1654	2190	1908, 1935, 1882	29, 21, 10	34.25356, 24.35402, 13.18184	15.83622, 12.71289, 8.33771	30.65537,	3	NA	724
GL174945.1	16094	16376	16246, 16311, 16131	7, 10, 7	7.1696, 8.01217, 8.69489	5.31345, 5.50894, 6.06379	4.61046, 5	3	NA	0
GL174948.1	10126	10687	10348, 10444, 10596	6, 8, 3	5.96715, 7.07159, 3.47371	4.64927, 5.20073, 3.03189	3.52158, 4	3	NA	NA
GL174951.1	1230	2580	2076, 1521, 2366	12, 7, 5	13.82915, 5.97725, 5.80393	8.62415, 4.62287, 4.48186	10.85461,	3	NA	NA
GL174955.1	40	532	424, 209, 153	11, 11, 14	11.85906, 10.60895, 17.40994	7.65504, 6.9343, 10.29833	8.98595, 7	3	dpm3	2110
GL174957.1	189	918	633, 774, 766	12, 12, 11	13.8483, 11.86108, 13.1143	8.63436, 7.51216, 8.32282	10.86694,	3	NA	NA
GL174959.1	7455	7845	7627, 7635, 7517	5, 9, 8	4.82177, 8.21099, 10.1465	3.98509, 5.77858, 6.82176	2.51418, 5	3	U4	1354
GL174959.1	18564	19017	18621, 18621, 18621	15, 27, 15	18.26603, 33.60851, 21.38372	10.62691, 16.18004, 12.12757	15.09729,	3	U4	9205
GL174960.1	15448	15805	15564, 15586, 15556	15, 31, 18	10.98605, 25.25022, 18.87511	6.32215, 9.927, 10.32913	8.16731, 2	3	NA	3852
GL174960.1	18929	19489	19370, 19351, 19412	17, 24, 12	14.5428, 18.92532, 11.81848	7.98018, 8.57092, 7.4869	11.53067,	3	NA	7333
GL174962.1	18926	19439	19239, 19222, 19278	13, 15, 14	15.29169, 15.80067, 17.94273	9.29854, 9.24574, 10.5654	12.24396,	3	NA	4910
GL174964.1	2424	2806	2656, 2686, 2605	6, 11, 6	5.96715, 10.60895, 7.29403	4.64927, 6.9343, 5.30581	3.52158, 7	3	NA	NA
GL174968.1	4351	4892	4668, 4846, 4766	13, 15, 11	11.35857, 13.01314, 13.55304	7.00844, 7.56134, 8.54092	8.51036, 1	3	NA	NA
GL174972.1	18807	19254	19219, 19164, 19227	43, 53, 57	59.64878, 72.87057, 83.13759	24.80281, 27.63336, 31.53104	55.66491,	3	NA	NA
GL174974.1	6474	7166	6532, 6567, 6519	18, 21, 7	20.65644, 24.35402, 8.69489	11.37184, 12.71289, 6.06379	17.40599,	3	NA	NA
GL174975.1	18069	19017	18643, 18339, 18815	9, 23, 17	9.72082, 27.36982, 23.46806	6.64182, 13.8686, 13.01603	6.97155, 2	3	NA	NA
GL174977.1	15171	15738	15504, 15567, 15551	9, 8, 7	8.45482, 7.07159, 8.08824	5.92419, 5.20073, 5.7849	5.79307, 4	3	NA	NA
GL174978.1	1414	2017	1526, 1538, 1698	5, 5, 6	4.82177, 3.94561, 7.29403	3.98509, 3.46715, 5.30581	2.51418, 1	3	NA	NA
GL174979.1	191	866	715, 459, 836	8, 9, 6	8.42257, 8.21099, 7.29403	5.97763, 5.77858, 5.30581	5.76336, 5	3	NA	NA
GL174979.1	5817	6295	6012, 5993, 6078	25, 23, 25	26.50188, 23.5013, 31.0492	12.68794, 11.57503, 15.36476	23.07912,	3	NA	NA
GL174981.1	7931	8448	8274, 8328, 8315	23, 30, 13	14.74157, 18.80986, 9.10008	6.57755, 7.01956, 5.61162	11.72132,	3	NA	NA
GL174981.1	9680	10365	9834, 9790, 9925	18, 16, 11	10.77246, 7.49484, 7.84667	5.54716, 4.16261, 5.22647	7.96663, 5	3	NA	NA
GL174984.1	16024	16774	16134, 16561, 16554	7, 9, 8	6.46447, 8.21099, 8.56955	4.91463, 5.77858, 6.05652	3.98312, 5	3	NA	NA
GL174985.1	4719	5732	4754, 5500, 5183, 5594	72, 58, 56, 36	19.74713, 9.17638, 16.76475, 6.35877	3.55124, 2.39853, 3.71115, 2.40899	16.52712,	3	PK-G12rRNA	3040
GL174985.1	8288	8534	8438, 8392, 8398	69, 85, 45	18.04255, 22.48742, 10.5921	3.4053, 3.50344, 2.99496	14.88412,	3	PK-G12rRNA	238
GL174985.1	8818	9118	9060, 8929, 8937	74, 85, 62	20.91287, 22.48742, 20.52443	3.64853, 3.50344, 4.10179	17.65498,	3	PK-G12rRNA	0
GL174986.1	18217	19146	18589, 18664, 18605	13, 16, 6	15.29169, 17.16856, 7.29403	9.29854, 9.82359, 5.30581	12.24396,	3	NA	NA
GL175000.1	12940	13776	13049, 13339, 13328	6, 23, 8	5.96715, 27.36982, 10.1465	4.64927, 13.8686, 6.82176	3.52158, 2	3	NA	NA
GL175004.1	20	2365	889, 1162, 1025	25, 30, 13	24.4138, 26.71531, 12.76267	11.4397, 11.04741, 7.86275	21.04856,	3	NA	NA
GL175004.1	7402	8849	7948, 8119, 8053, 8540	11, 15, 6, 7	9.48173, 12.7702, 5.75442, 6.92462	6.24938, 7.41214, 4.53101, 5.17829	6.76069, 1	3	NA	NA
GL175004.1	12274	12964	12385, 12475, 12667	15, 12, 8	15.12865, 9.70336, 8.56955	8.84764, 6.19346, 6.05652	12.09208,	3	NA	NA
GL175004.1	18168	18777	18466, 18614, 18415	17, 12, 12	21.34551, 11.86108, 16.36823	11.95527, 7.51216, 9.85365	18.07159,	3	NA	NA
GL175006.1	1577	2036	1809, 1768, 1771	8, 12, 8	7.38183, 11.20359, 9.1831	5.38666, 7.11794, 6.37229	4.80856, 8	3	NA	NA
GL175006.1	14368	15406	14549, 14586, 14600	12, 38, 17	9.04939, 32.651, 16.37174	5.78552, 11.54912, 9.09382	6.35412, 2	3	NA	NA
GL175011.1	16980	17648	17227, 17431, 17208	5, 10, 5	4.82177, 9.39128, 5.91431	3.98509, 6.35644, 4.53172	2.51418, 6	3	tmem82	3562
GL175015.1	62	1230	1002, 649, 1119	51, 71, 44	50.19343, 70.56118, 47.68367	15.81712, 17.92906, 17.37082	46.3388, 6	3	NA	NA
GL175015.1	7156	10508	7591, 7571, 8034	79, 89, 53	47.19745, 49.35991, 33.92303	8.19394, 7.53847, 8.37325	43.38586,	3	NA	NA
GL175015.1	9998	13146	11485, 7571, 10756, 11497, 8034, 10913, 11835	54, 89, 30, 62, 53, 22, 32	23.20732, 49.35991, 5.43949, 24.34493, 33.92303, 7.56791, 14.67387	5.19834, 7.53847, 2.37616, 4.82897, 8.37325, 3.5024, 5.02518	19.87674,	3	NA	NA
GL175015.1	18035	18821	18230, 18247, 18245	20, 19, 18	21.98313, 18.48026, 19.15771	11.61252, 9.80703, 10.49722	18.6897, 1	3	NA	NA
GL175017.1	538	963	905, 934, 896	18, 33, 11	21.63622, 39.56544, 13.46208	11.92423, 17.39878, 8.49639	18.35375,	3	NA	NA
GL175022.1	77	4761	1056, 1047, 4714, 1062, 3147	50, 58, 24, 48, 37	28.99257, 28.61969, 5.28799, 32.16276, 21.40021	7.26502, 6.13209, 2.57103, 8.71207, 6.7563	25.5089, 2	3	NA	NA
GL175022.1	5166	5594	5438, 5495, 5541	20, 40, 18	6.00912, 14.58879, 6.77915	3.06661, 4.20176, 3.56977	3.5617, 11	3	NA	NA
GL175022.1	9695	10582	10185, 9988, 10214	17, 33, 10	15.5079, 26.88659, 9.58601	8.56821, 10.17111, 6.47231	12.45101,	3	NA	NA
GL175022.1	16456	18531	17770, 17646, 17778	27, 36, 22	23.27209, 36.24688, 24.34205	10.10253, 14.22188, 12.40437	19.93947,	3	NA	NA
GL175028.1	392	1957	1177, 788, 1811	9, 15, 8	6.06453, 9.58015, 6.50832	4.43343, 5.48539, 4.85389	3.6122, 6.9	3	NA	NA
GL175030.1	1332	1825	1591, 1582, 1625	10, 13, 6	9.44155, 10.7674, 6.6567	6.38646, 6.64292, 5.00845	6.72156, 8	3	NA	NA
GL175030.1	2283	3332	2484, 2921, 2758	8, 15, 6	7.02691, 12.58265, 6.5517	5.17362, 7.29694, 4.95623	4.50222, 9	3	NA	NA
GL175030.1	11606	12585	11817, 11822, 11836	12, 15, 16	10.3005, 11.72411, 16.70005	6.56436, 6.77081, 9.54762	7.51999, 9	3	NA	NA
GL175030.1	14147	15119	14244, 14882, 14406, 14946	8, 13, 5, 4	6.20993, 10.68145, 4.39541, 3.41557	4.66614, 6.58965, 3.74107, 3.13194	3.74679, 8	3	NA	NA
GL175040.1	3317	3715	3446, 3406, 3371	19, 28, 14	18.26836, 27.32048, 15.20637	9.67872, 12.09764, 9.10193	15.09918,	3	NA	NA
GL175051.1	17335	18374	18006, 18046, 18068	10, 12, 10	11.06011, 11.86108, 13.18184	7.306, 7.51216, 8.33771	8.22712, 9	3	NA	NA
GL175064.1	17384	17891	17459, 17653, 17522	9, 12, 6	9.72082, 11.86108, 7.29403	6.64182, 7.51216, 5.30581	6.97155, 9	3	NA	NA
GL175076.1	14168	15179	15006, 14262, 15007	9, 9, 7	9.72082, 7.75488, 8.21885	6.64182, 5.501, 5.84717	6.97155, 5	3	NA	143
GL175081.1	16224	17075	16847, 16581, 16608	19, 13, 15	20.05161, 9.96603, 15.42372	10.75274, 6.14603, 9.02331	16.81949,	3	NA	NA
GL175084.1	15866	17931	17582, 16762, 16757	79, 148, 48	68.66935, 125.99252, 44.08269	14.59706, 15.73694, 13.86884	64.58627,	3	NA	2011
GL175092.1	4733	5490	5113, 5424, 5409	10, 12, 9	5.30334, 5.23787, 7.37866	3.80362, 3.49683, 5.26769	2.9568, 3.0	3	NA	NA
GL175092.1	11255	11886	11372, 11383, 11397	14, 25, 9	8.8895, 14.18325, 5.71106	5.28098, 5.89875, 4.20698	6.20343, 1	3	NA	NA
GL175092.1	16596	17768	16777, 16777, 16298, 17468	14, 14, 12, 8	12.69785, 9.92123, 9.40735, 6.27048	7.6073, 5.90089, 6.0089, 4.70442	9.77574, 7	3	NA	NA
GL175099.1	8422	8888	8771, 8569, 8815	273, 319, 242	284.9906, 316.76184, 266.81885	25.72986, 23.3829, 28.70049	279.74582	3	NA	NA
GL175102.1	2386	3024	2558, 2591, 2582	15, 21, 4	16.72085, 24.35402, 4.55201	9.7776, 12.71289, 3.73489	13.61562,	3	NA	0
GL175105.1	13095	13738	13255, 13566, 13654	7, 11, 5	6.37715, 9.57292, 5.14967	4.86324, 6.30569, 4.16141	3.90257, 6	3	NA	NA
GL175107.1	66	2256	768, 1614, 1579	15, 14, 13	15.49092, 9.72946, 14.0426	9.06314, 5.78457, 8.61056	12.43497,	3	NA	NA
GL175107.1	5617	7090	5952, 5941, 6064, 6643	13, 24, 7, 7	8.97001, 16.43436, 4.8734, 4.9397	5.53188, 7.22111, 3.92489, 3.96789	6.27965, 1	3	NA	NA
GL175107.1	8581	9398	8993, 8974, 8822	22, 23, 18	17.80348, 14.53177, 16.82438	8.50975, 6.47052, 9.09168	14.65363,	3	NA	NA
GL175107.1	11007	12303	11713, 11652, 11656	15, 24, 10	10.25319, 15.25746, 6.73345	5.88239, 6.61851, 4.70048	7.47622, 1	3	NA	NA
GL175107.1	15220	17717	16771, 16358, 16621	16, 25, 16	14.18542, 19.08376, 15.10611	8.02648, 8.37558, 8.5886	11.18941,	3	NA	NA
GL175111.1	5444	5882	5657, 5653, 5668	13, 21, 14	15.29169, 24.35402, 18.52583	9.29854, 12.71289, 10.84669	12.24396,	3	NA	0
GL175112.1	16644	17578	16674, 17247, 17010	10, 18, 9	11.06011, 19.97713, 11.64359	7.306, 10.97931, 7.57973	8.22712, 1	3	NA	NA
GL175114.1	80	612	378, 418, 174	8, 17, 4	8.42257, 18.56115, 4.67325	5.97763, 10.40145, 3.78987	5.76336, 1	3	NA	8973
GL175114.1	16517	17636	17150, 17422, 16953	5, 12, 9	4.82177, 11.86108, 11.64359	3.98509, 7.51216, 7.57973	2.51418, 9	3	NA	5999
GL175123.1	22	741	355, 574, 546	53, 113, 41	47.64378, 108.94801, 40.65767	13.85265, 19.02442, 14.70772	43.8248, 1	3	NA	4714
GL175123.1	15732	16352	16064, 15896, 16301	20, 31, 14	8.99757, 13.40595, 7.11989	4.32743, 4.69887, 4.26334	6.30637, 1	3	NA	5070
GL175123.1	16735	17569	17414, 16928, 17413, 17392	51, 40, 76, 42	39.08699, 20.69675, 56.90012, 36.60114	10.71555, 6.03553, 11.33501, 12.22156	35.39925,	3	NA	6073
GL175138.1	5458	6020	5659, 5869, 5730	8, 10, 5	5.13458, 7.38914, 5.40789	3.97494, 5.11603, 4.29296	2.80161, 4	3	gps2	0
GL175138.1	16743	17442	17275, 17293, 17143	33, 43, 29	38.68132, 49.08422, 35.97959	16.87521, 18.61973, 16.84875	34.99999,	3	gps2	71
GL175143.1	2511	3512	3262, 2580, 3196, 2543, 2936	12, 13, 7, 6, 4	9.23027, 10.04078, 4.28332, 5.39683, 3.34447	5.8984, 6.19235, 3.53849, 4.32487, 3.08919	6.52452, 7	3	NA	NA
GL175143.1	8533	9596	9483, 8792, 8813	7, 10, 4	5.86458, 7.35725, 3.85136	4.55375, 5.09584, 3.38149	3.46146, 4	3	NA	NA
GL175145.1	12020	12841	12452, 12418, 12720	8, 9, 10	8.42257, 8.21099, 12.33934	5.97763, 5.77858, 7.95424	5.76336, 5	3	NA	NA
GL175147.1	61	848	656, 122, 657	134, 114, 97	135.14635, 113.51971, 111.49215	21.49562, 20.28732, 25.85187	130.50302	3	NA	NA
GL175147.1	4440	5120	4857, 4927, 4698	60, 51, 30	38.41509, 32.05231, 19.04399	8.59477, 8.10188, 7.12567	34.73907,	3	NA	NA
GL175148.1	875	1624	1017, 1261, 929	13, 23, 6	15.29169, 26.44164, 7.29403	9.29854, 13.32732, 5.30581	12.24396,	3	NA	NA
GL175148.1	7397	10177	7884, 9451, 9437	17, 26, 15	12.30855, 16.09107, 11.59124	6.63868, 6.59743, 6.68971	9.41404, 1	3	NA	NA
GL175148.1	7464	10177	8937, 9451, 9437	9, 26, 15	4.26525, 16.09107, 11.59124	3.29144, 6.59743, 6.68971	2.09431, 1	3	NA	NA
GL175148.1	12826	13429	13016, 13024, 13023	13, 19, 8	6.40665, 8.35575, 3.96546	4.01678, 4.16701, 3.21804	3.93103, 5	3	NA	NA
GL175149.1	45	880	235, 235, 236	14, 15, 5	16.13274, 15.80067, 5.9506	9.62159, 9.24574, 4.54784	13.05194,	3	NA	NA
GL175149.1	11804	12234	12017, 12069, 12157	6, 9, 4	5.96715, 8.21099, 4.67325	4.64927, 5.77858, 3.78987	3.52158, 5	3	NA	NA
GL175151.1	12539	12842	12667, 12677, 12664	22, 28, 15	28.01469, 35.20764, 20.14748	14.51292, 16.75789, 11.5698	24.55471,	3	NA	NA
GL175153.1	2769	3382	2860, 3020, 2846	8, 8, 6	7.28955, 6.77831, 6.82211	5.33177, 5.0215, 5.08889	4.72176, 4	3	ppm1g	3000
GL175159.1	612	1777	1672, 948, 903	8, 15, 7	8.42257, 15.80067, 8.69489	5.97763, 9.24574, 6.06379	5.76336, 1	3	NA	NA
GL175162.1	3504	3934	3902, 3904, 3906	32, 47, 40	38.78161, 54.59318, 49.54379	17.34112, 19.95276, 20.26143	35.09861,	3	gid8	8173
GL175162.1	16118	17181	16903, 16838, 16912	12, 29, 15	13.8483, 30.80901, 18.74072	8.63436, 13.78891, 10.87493	10.86694,	3	gid8	803
GL175167.1	8	888	72, 434, 394	10, 16, 8	10.81731, 16.67093, 10.00633	7.17456, 9.53036, 6.75998	8.00764, 1	3	NA	NA
GL175183.1	14662	15576	14893, 14893, 14981	11, 19, 11	10.78278, 17.58589, 12.34038	7.03733, 9.26517, 7.91841	7.97565, 1	3	NA	NA
GL175189.1	144	956	355, 386, 399	15, 31, 15	11.6639, 26.17887, 14.87928	6.73404, 10.42282, 8.69822	8.80068, 2	3	NA	NA
GL175201.1	79	1030	534, 397, 560	16, 22, 15	12.26603, 19.31492, 14.65165	6.85709, 9.39436, 8.56113	9.37251, 1	3	NA	NA
GL175201.1	4305	5643	4757, 5388, 4904	24, 17, 11	20.54096, 13.54833, 9.59812	9.49133, 7.37778, 6.32122	17.29409,	3	NA	NA
GL175201.1	13113	14178	13373, 13343, 13371	21, 29, 15	21.0274, 29.11719, 18.13606	10.73595, 12.78747, 10.55788	17.7649, 2	3	NA	NA
GL175201.1	16296	16755	16590, 16474, 16449	14, 16, 8	12.23001, 12.90802, 8.35103	7.31997, 7.24623, 5.93881	9.33844, 1	3	NA	NA
GL175212.1	142	572	323, 271, 326	17, 27, 22	4.89802, 8.26055, 11.63136	2.84273, 3.34221, 5.24242	2.58474, 5	3	NA	9146
GL175212.1	1316	1845	1668, 1548, 1681	39, 68, 28	20.21895, 39.77109, 16.43345	6.01491, 7.77744, 6.35406	16.98207,	3	NA	7873
GL175212.1	3439	4253	3505, 3615, 4022	29, 37, 16	11.16108, 12.27016, 5.7304	4.13671, 3.80463, 3.29898	8.32297, 9	3	NA	5465
GL175212.1	15014	15805	15087, 15748, 15407	15, 17, 8	8.25865, 7.52153, 4.15685	4.73068, 4.03693, 3.3412	5.62728, 5	3	NA	0
GL175218.1	9124	9998	9425, 9817, 9452	12, 9, 9	10.3005, 7.47261, 8.30233	6.56436, 5.32627, 5.83337	7.51999, 5	3	NA	1375
GL175219.1	13960	16358	15394, 15465, 15668, 16287	37, 59, 25, 8	28.1581, 47.17086, 21.64377, 4.3023	9.6418, 11.86902, 9.81332, 3.43511	24.69479,	3	NA	NA
GL175223.1	173	2853	1924, 2294, 1820	15, 30, 12	12.80515, 22.43859, 10.30599	7.4336, 8.76017, 6.56776	9.87861, 1	3	NA	NA
GL175232.1	156	1109	803, 764, 767	9, 15, 14	9.56822, 15.80067, 17.3087	6.55912, 9.24574, 10.24653	6.84121, 1	3	NA	NA
GL175236.1	2709	3003	2945, 2958, 2842	7, 8, 3	5.93788, 5.48596, 3.24307	4.59878, 4.2025, 2.92359	3.52158, 3	3	NA	NA
GL175236.1	14749	16586	15824, 15793, 15754	16, 22, 7	15.34845, 19.31492, 7.34973	8.73591, 9.39436, 5.41038	12.2978, 1	3	NA	NA
GL175243.1	12	1023	177, 299, 521	53, 102, 51	33.38454, 73.37806, 37.8171	8.1943, 11.1907, 10.2077	29.80345,	3	NA	NA
GL175243.1	2166	2848	2513, 2455, 2514	65, 118, 58	39.20565, 81.44248, 42.02798	8.04218, 10.69237, 10.1834	35.51619,	3	NA	NA
GL175243.1	14377	15233	14555, 14543, 14484	28, 46, 29	23.08373, 40.71214, 25.05115	9.68222, 12.90862, 10.45161	19.75679,	3	NA	NA
GL175243.1	15366	15649	15506, 15526, 15440	16, 12, 11	10.26719, 5.36899, 6.16287	5.67578, 3.57047, 4.18065	7.48849, 3	3	NA	NA
GL175245.1	8874	9254	8912, 8902, 8908	12, 17, 16	13.8483, 18.56115, 23.11042	8.63436, 10.40145, 12.88555	10.86694,	3	NA	NA
GL175254.1	14593	15206	15001, 15173, 15151	22, 24, 21	29.43504, 28.90463, 31.95329	15.27618, 14.44646, 16.6163	25.94112,	3	NA	NA
GL175258.1	11	649	368, 365, 348	35, 37, 13	42.02964, 39.59725, 14.85247	18.02171, 15.7402, 9.06201	38.29497,	3	NA	NA
GL175258.1	7835	8497	8413, 8405, 8161	68, 70, 42	76.44885, 78.35633, 49.3327	22.02517, 22.08389, 19.22136	72.28686,	3	NA	NA
GL175260.1	8	910	252, 291, 317	29, 44, 24	34.49933, 52.08342, 29.93386	15.98132, 19.90399, 15.03552	30.89597,	3	NA	NA
GL175260.1	6558	8148	7181, 7155, 7180	46, 42, 33	50.71767, 40.29206, 37.27829	18.20023, 14.12324, 16.04321	46.85497,	3	NA	NA
GL175260.1	12752	16368	13420, 14470, 13251, 14457, 16181	36, 55, 24, 35, 28	24.77568, 42.34344, 16.44478, 29.2564, 21.28549	8.34957, 11.00357, 7.22656, 10.77314, 8.71678	21.40038,	3	NA	NA
GL175260.1	12752	16368	14449, 14470, 13251, 14457, 16181	41, 55, 24, 35, 28	30.933, 42.34344, 16.44478, 29.2564, 21.28549	9.84075, 11.00357, 7.22656, 10.77314, 8.71678	27.40448,	3	NA	NA
GL175264.1	10165	10467	10336, 10332, 10213	7, 8, 5	6.94335, 7.07159, 4.99237	5.18876, 5.20073, 4.0781	4.42528, 4	3	NA	622
GL175264.1	14901	16301	15140, 15976, 16142	8, 10, 4	8.34715, 9.39128, 4.67325	5.9367, 6.35644, 3.78987	5.71011, 6	3	NA	3577
GL175271.1	8741	9462	8919, 9291, 9018	5, 6, 4	4.82177, 4.93312, 4.67325	3.98509, 4.04501, 3.78987	2.51418, 2	3	NA	NA
GL175272.1	9431	9837	9801, 9802, 9803	24, 29, 13	31.25967, 36.82147, 18.01082	15.77492, 17.33575, 10.61163	27.72325,	3	NA	NA
GL175287.1	15377	15727	15506, 15513, 15658	5, 7, 4	4.82177, 5.97725, 4.67325	3.98509, 4.62287, 3.78987	2.51418, 3	3	NA	NA
GL175288.1	7842	9022	8491, 8527, 8135	12, 20, 13	11.98519, 17.08177, 13.33541	7.58551, 8.66794, 8.20195	9.10644, 1	3	pde9a	0
GL175289.1	30	938	254, 259, 477	11, 18, 5	12.43693, 19.97713, 5.75061	7.97018, 10.97931, 4.45735	9.52692, 1	3	NA	NA
GL175291.1	1433	2853	2376, 1769, 2371, 2392	40, 36, 71, 35	18.28176, 10.57389, 33.45543, 19.3632	5.2605, 3.42142, 5.98805, 6.31799	15.11236,	3	NA	NA
GL175291.1	5366	5746	5482, 5526, 5531	28, 41, 20	7.5366, 9.45177, 5.86018	3.05092, 2.91305, 3.0089	4.9546, 6.8	3	NA	NA
GL175291.1	8043	8672	8201, 8224, 8226	31, 46, 25	12.80775, 17.10086, 11.57701	4.4924, 4.38064, 4.75983	9.88066, 1	3	NA	NA
GL175291.1	15358	16159	15614, 15612, 15568	27, 27, 22	28.0693, 26.14468, 24.50966	12.90567, 11.7641, 12.50369	24.60796,	3	NA	NA
GL175308.1	6045	6820	6585, 6589, 6604	120, 190, 89	78.93437, 122.60008, 63.00871	9.94846, 10.0927, 10.68557	74.74826,	3	NA	NA
GL175308.1	8596	9360	8807, 8931, 8950	149, 227, 109	111.91348, 164.68529, 86.0781	12.6183, 12.37083, 13.17533	107.42775	3	NA	NA
GL175308.1	14093	14687	14356, 14400, 14439	101, 152, 42	73.58216, 106.33933, 27.36343	11.40133, 11.24294, 8.11263	69.44962,	3	NA	NA
GL175308.1	15373	16068	15649, 15642, 15625	77, 149, 53	49.45974, 103.09504, 38.98628	9.0312, 11.02249, 10.18795	45.61457,	3	NA	NA
GL175315.1	1877	2542	2218, 2220, 2218	26, 30, 19	31.36507, 35.18344, 23.88486	15.21991, 16.00313, 12.94573	27.82689,	3	NA	NA
GL175315.1	7320	8164	7469, 7405, 7730	10, 13, 13	10.67246, 11.71436, 15.52427	7.09497, 7.22722, 9.42125	7.87096, 9	3	NA	NA
GL175315.1	14018	15913	14546, 14791, 14791	31, 43, 26	28.87347, 44.24422, 26.60724	11.91547, 15.87295, 12.39564	25.39301,	3	NA	NA
GL175321.1	101	1517	1285, 941, 1220	21, 43, 22	15.31612, 34.16316, 18.8809	7.36628, 10.75138, 9.13817	12.26775,	3	NA	NA
GL175323.1	7156	7763	7490, 7486, 7245	6, 7, 3	5.96715, 5.97725, 3.47371	4.64927, 4.62287, 3.03189	3.52158, 3	3	NA	NA
GL175327.1	8504	9357	9275, 9142, 8951	10, 11, 8	11.06011, 10.60895, 10.1465	7.306, 6.9343, 6.82176	8.22712, 7	3	NA	NA
GL175328.1	12011	12580	12196, 12280, 12426	5, 6, 3	4.82177, 4.93312, 3.47371	3.98509, 4.04501, 3.03189	2.51418, 2	3	agap3	677
GL175330.1	7678	8376	8084, 8087, 8261	5, 9, 10	4.82177, 8.21099, 10.71812	3.98509, 5.77858, 7.12015	2.51418, 5	3	NA	NA
GL175330.1	9051	9483	9282, 9273, 9213	14, 19, 16	16.7649, 21.4153, 19.25686	9.96273, 11.55717, 11.00387	13.6543, 1	3	NA	NA
GL175330.1	15232	15896	15493, 15736, 15558	18, 18, 11	22.92104, 19.97713, 14.75772	12.61945, 10.97931, 9.09568	19.59912,	3	NA	NA
GL175333.1	15487	15867	15635, 15779, 15629	6, 9, 5	5.96715, 8.21099, 5.9506	4.64927, 5.77858, 4.54784	3.52158, 5	3	NA	NA
GL175342.1	15040	15702	15590, 15419, 15336	7, 6, 5	7.1696, 4.93312, 5.9506	5.31345, 4.04501, 4.54784	4.61046, 2	3	NA	NA
GL175346.1	21	596	259, 283, 527	18, 29, 14	19.69649, 28.50226, 16.82614	10.81516, 12.42647, 9.99514	16.47841,	3	NA	NA
GL175346.1	6288	6815	6516, 6482, 6446	14, 19, 14	9.70564, 13.14951, 11.45195	5.77015, 6.6433, 6.84022	6.97127, 1	3	NA	NA
GL175346.1	8351	8893	8551, 8638, 8567	23, 31, 18	19.56259, 26.56002, 16.12098	9.23224, 10.62928, 8.66427	16.3493, 2	3	NA	NA
GL175346.1	14486	15642	14579, 14608, 15302	7, 14, 11	5.79309, 11.62806, 11.4314	4.5096, 6.94888, 7.41405	3.39779, 8	3	NA	NA
GL175346.1	14486	15642	15371, 14608, 15302	17, 14, 11	18.1272, 11.62806, 11.4314	10.1466, 6.94888, 7.41405	14.96605,	3	NA	NA
GL175366.1	1447	2085	1958, 2006, 1887	7, 15, 7	5.7233, 11.92739, 6.92462	4.4663, 6.8951, 5.17829	3.33547, 9	3	NA	NA
GL175367.1	15140	15612	15467, 15382, 15380	8, 14, 15	8.42257, 14.45897, 18.43142	5.97763, 8.66788, 10.71406	5.76336, 1	3	NA	NA
GL175381.1	14721	15614	15154, 15050, 15563	12, 15, 15	11.09909, 13.69427, 17.76194	7.05451, 7.97895, 10.35658	8.26391, 1	3	NA	NA
GL175383.1	82	2670	1132, 1127, 840	52, 77, 43	52.75191, 73.71252, 46.92784	16.73413, 17.16541, 17.38003	48.86194,	3	NA	NA
GL175383.1	7908	8796	8349, 8448, 8224, 8706	14, 16, 4, 4	16.7649, 17.16856, 4.67325, 4.67325	9.96273, 9.82359, 3.78987, 3.78987	13.6543, 1	3	NA	NA
GL175383.1	14552	15444	15067, 15190, 14853	10, 13, 6	11.06011, 13.14514, 7.29403	7.306, 8.09002, 5.30581	8.22712, 1	3	NA	NA
GL175387.1	11782	12480	12364, 12356, 12356	18, 30, 17	21.07666, 31.67912, 20.98465	11.6109, 13.92147, 11.76329	17.81304,	3	NA	NA
GL175388.1	5914	6475	6409, 6383, 6047	8, 10, 4	8.42257, 8.79469, 4.67325	5.97763, 5.99491, 3.78987	5.76336, 6	3	NA	NA
GL175389.1	312	1240	346, 853, 746	4, 9, 3	3.74195, 8.21099, 3.47371	3.32091, 5.77858, 3.03189	1.6222, 5.7	3	NA	NA
GL175389.1	11197	11991	11580, 11670, 11674	14, 31, 23	13.89699, 31.85677, 26.89275	8.3343, 13.63997, 13.59141	10.91278,	3	NA	NA
GL175398.1	169	888	635, 260, 704, 623	9, 9, 12, 5	6.63709, 5.24286, 6.99976, 3.88081	4.79941, 3.90773, 4.52293, 3.42643	4.14229, 3	3	NA	NA
GL175398.1	5450	5908	5747, 5734, 5739	16, 18, 8	12.84367, 11.84204, 6.57059	7.20709, 6.13746, 4.89275	9.91487, 9	3	NA	NA
GL175398.1	11782	12597	12428, 11998, 12023	36, 61, 33	25.76413, 43.75076, 27.9777	8.79326, 10.14469, 10.74278	22.36155,	3	NA	NA
GL175404.1	82	783	332, 389, 499	14, 30, 10	16.7649, 32.61231, 10.90706	9.96273, 14.47527, 7.22344	13.6543, 2	3	NA	NA
GL175405.1	974	1569	1538, 1501, 1525	15, 10, 11	18.26603, 9.39128, 14.75772	10.62691, 6.35644, 9.09568	15.09729,	3	NA	NA
GL175408.1	188	844	634, 640, 606	14, 9, 9	11.84416, 5.89579, 8.79325	7.08219, 4.32532, 6.12267	8.97236, 3	3	NA	NA
GL175408.1	2509	3701	2940, 3411, 3398	19, 22, 11	13.9973, 14.04143, 7.58795	7.12763, 6.43749, 5.06406	11.00892,	3	NA	NA
GL175408.1	6989	8254	7835, 7563, 7714	26, 28, 19	20.17933, 16.02654, 14.72025	8.69011, 6.17367, 7.54871	16.94323,	3	NA	NA
GL175408.1	10702	11835	11025, 11230, 11005	16, 35, 17	10.21083, 24.2731, 13.10236	5.64338, 8.36143, 7.11001	7.43562, 2	3	NA	NA
GL175408.1	14545	15332	14677, 14966, 15019	11, 19, 7	8.47312, 13.01755, 5.70698	5.62011, 6.56895, 4.45614	5.81094, 1	3	NA	NA
GL175417.1	7763	8924	8653, 7812, 8008	8, 7, 5	8.42257, 5.97725, 5.9506	5.97763, 4.62287, 4.54784	5.76336, 3	3	NA	NA
GL175417.1	13849	14510	14129, 14135, 14211	19, 32, 12	23.28778, 37.64516, 13.90113	12.61993, 16.66904, 8.66246	19.95483,	3	NA	NA
GL175420.1	32	1044	358, 438, 433	152, 267, 96	107.522, 197.58675, 72.14694	11.44637, 12.949, 11.89093	103.07128	3	NA	NA
GL175420.1	1709	2609	2084, 2063, 2088	203, 322, 128	166.06984, 261.71924, 110.29916	15.16427, 15.54247, 15.75681	161.25714	3	NA	NA
GL175420.1	9684	10673	9912, 9875, 10002	9, 20, 9	6.36774, 14.03125, 8.09849	4.62751, 6.89739, 5.71072	3.8942, 11	3	NA	NA
GL175421.1	5561	5863	5716, 5782, 5601	6, 9, 3	5.96715, 8.21099, 3.47371	4.64927, 5.77858, 3.03189	3.52158, 5	3	NA	7548
GL175422.1	13261	14273	13545, 13613, 13953	16, 21, 12	13.10843, 16.11006, 10.46657	7.36828, 7.81637, 6.66697	10.16616,	3	NA	NA
GL175431.1	14432	15341	14975, 14975, 14972	51, 77, 39	60.21231, 92.25741, 47.10915	21.26975, 25.90888, 19.27623	56.22173,	3	NA	NA
GL175448.1	4619	5386	4661, 4761, 4969	5, 11, 8	4.69353, 10.10652, 8.35103	3.9134, 6.63228, 5.93881	2.45909, 7	3	NA	NA
GL175457.1	12863	13646	13438, 13466, 13457	13, 17, 6	12.56081, 16.05386, 6.76586	7.74168, 8.90069, 5.06179	9.64511, 1	3	NA	NA
GL175461.1	109	910	384, 382, 113	10, 12, 3	11.06011, 11.86108, 3.47371	7.306, 7.51216, 3.03189	8.22712, 9	3	NA	NA
GL175463.1	89	884	299, 314, 704	10, 10, 6	11.06011, 9.39128, 7.25145	7.306, 6.35644, 5.287	8.22712, 6	3	NA	NA
GL175463.1	14603	15084	14750, 14936, 14739	5, 15, 3	4.82177, 15.80067, 3.37781	3.98509, 9.24574, 2.98791	2.51418, 1	3	NA	NA
GL175468.1	4841	5508	5236, 5029, 5246	15, 20, 20	8.08868, 9.74394, 13.31187	4.63637, 4.67404, 6.49915	5.46988, 7	3	NA	NA
GL175468.1	14523	15127	14867, 14750, 14662	5, 10, 10	4.82177, 9.39128, 13.18184	3.98509, 6.35644, 8.33771	2.51418, 6	3	NA	NA
GL175476.1	13700	14220	13883, 13962, 14054	6, 8, 4	5.96715, 7.07159, 4.67325	4.64927, 5.20073, 3.78987	3.52158, 4	3	NA	NA
GL175488.1	13378	13876	13471, 13509, 13587	5, 8, 6	4.82177, 7.07159, 7.29403	3.98509, 5.20073, 5.30581	2.51418, 4	3	NA	NA
GL175490.1	78	367	341, 278, 341	6, 16, 8	5.96715, 17.16856, 10.1465	4.64927, 9.82359, 6.82176	3.52158, 1	3	NA	NA
GL175491.1	13543	13790	13737, 13739, 13715	6, 7, 6	5.96715, 5.97725, 7.25145	4.64927, 4.62287, 5.287	3.52158, 3	3	NA	NA
GL175500.1	9181	9511	9322, 9379, 9297	16, 19, 10	19.41253, 21.19824, 12.90749	11.08796, 11.43055, 8.21675	16.20324,	3	NA	NA
GL175500.1	14548	14975	14750, 14721, 14741	9, 10, 8	9.72082, 9.39128, 10.1465	6.64182, 6.35644, 6.82176	6.97155, 6	3	NA	NA
GL175504.1	3950	4750	4428, 4402, 4239	17, 33, 11	15.62395, 27.87558, 10.66921	8.63894, 10.68868, 6.9701	12.56245,	3	umps	751
GL175504.1	6629	7246	7048, 6740, 6952	17, 23, 10	15.5079, 17.59721, 9.77985	8.56821, 8.10966, 6.58646	12.45101,	3	umps	3430
GL175504.1	14292	14902	14438, 14732, 14544	6, 12, 4	5.90927, 11.86108, 4.67325	4.61743, 7.51216, 3.78987	3.50164, 9	3	umps	11093
GL175508.1	14683	14958	14718, 14740, 14896	7, 6, 6	7.1696, 4.93312, 7.29403	5.31345, 4.04501, 5.30581	4.61046, 2	3	hars2	4381
GL175512.1	70	1084	453, 450, 444	15, 35, 21	17.09296, 40.21036, 25.89737	9.98755, 16.9448, 13.59243	13.96978,	3	NA	NA
GL175512.1	6891	8494	8074, 8004, 8084	108, 127, 71	66.42783, 71.13802, 42.87505	8.89891, 7.94379, 8.17673	62.36824,	3	NA	NA
GL175512.1	9706	10997	10351, 10490, 10813	100, 146, 69	57.1296, 86.92802, 39.6092	7.95533, 8.76163, 7.60612	53.17805,	3	NA	NA
GL175512.1	12191	13218	12417, 12517, 12536	38, 63, 36	11.43935, 23.05594, 15.54475	3.52288, 4.51361, 4.851	8.5874, 19	3	NA	NA
GL175517.1	14181	14902	14560, 14641, 14484	35, 53, 14	37.54288, 58.2693, 14.92875	15.37419, 19.24387, 8.94211	33.88042,	3	NA	NA
GL175520.1	7514	7763	7648, 7649, 7732	6, 16, 12	5.96715, 17.16856, 16.36823	4.64927, 9.82359, 9.85365	3.52158, 1	3	NA	NA

GL175531.1	132	1282	899, 614, 764	12, 12, 4	12.74986, 11.37069, 4.38152	8.02869, 7.21896, 3.65448	9.82533, 8	3	NA	NA
GL175533.1	8634	9096	8953, 8967, 8945	6, 13, 6	5.96715, 13.14514, 7.29403	4.64927, 8.09002, 5.30581	3.52158, 1	3	insig2	0
GL175536.1	5970	7566	6230, 7367, 6671, 7371	22, 27, 15, 13	20.79166, 22.94491, 14.11641, 11.86258	10.27525, 9.91808, 8.2366, 7.31799	17.5367, 1	3	NA	NA
GL175536.1	13653	14385	14180, 13814, 13929	9, 10, 3	9.72082, 9.39128, 3.47371	6.64182, 6.35644, 3.03189	6.97155, 6	3	NA	NA
GL175540.1	1369	2487	2259, 1926, 1781	10, 9, 6	10.01299, 8.21099, 7.29403	6.7221, 5.77858, 5.30581	7.24763, 5	3	NA	NA
GL175544.1	14227	14774	14619, 14651, 14684	28, 29, 25	33.96473, 31.27195, 28.93252	16.03634, 14.06427, 14.1346	30.3722, 2	3	NA	NA
GL175546.1	6027	6462	6065, 6246, 6221	5, 10, 4	4.82177, 9.39128, 4.67325	3.98509, 6.35644, 3.78987	2.51418, 6	3	NA	NA
GL175546.1	14508	14777	14716, 14598, 14657	11, 11, 6	12.43693, 10.60895, 7.29403	7.97018, 6.9343, 5.30581	9.52692, 7	3	NA	NA
GL175549.1	13036	13248	13076, 13106, 13090	12, 14, 12	13.8483, 14.45897, 16.28773	8.63436, 8.66788, 9.81872	10.86694,	3	NA	NA
GL175550.1	14204	14739	14459, 14645, 14496	12, 12, 8	13.8483, 11.86108, 10.1465	8.63436, 7.51216, 6.82176	10.86694,	3	NA	NA
GL175554.1	159	945	855, 814, 859	13, 13, 10	10.14103, 11.12608, 10.03522	6.2545, 6.86492, 6.73493	7.3686, 8.4	3	NA	NA
GL175554.1	1287	1775	1591, 1600, 1632	19, 17, 17	17.0405, 15.93163, 19.63801	8.935, 8.82633, 11.0208	13.92003,	3	NA	NA
GL175554.1	7035	8325	7229, 7303, 7253, 8219	11, 14, 6, 7	11.02968, 14.45897, 6.35308, 7.61369	7.18222, 8.66788, 4.85498, 5.54854	8.20839, 1	3	NA	NA
GL175554.1	14282	14721	14472, 14390, 14409	11, 21, 10	12.43693, 23.90685, 13.18184	7.97018, 12.45235, 8.33771	9.52692, 2	3	NA	NA
GL175556.1	13	765	127, 134, 194	24, 42, 13	26.57606, 45.44769, 16.70862	13.07811, 16.97276, 10.0169	23.15164,	3	NA	NA
GL175561.1	1251	1854	1590, 1690, 1755	5, 11, 5	4.43696, 8.81665, 5.59755	3.76555, 5.83547, 4.38538	2.24017, 6	3	NA	NA
GL175563.1	7171	7702	7463, 7605, 7507	6, 13, 4	5.96715, 13.14514, 4.67325	4.64927, 8.09002, 3.78987	3.52158, 1	3	NA	NA
GL175571.1	5549	6171	5704, 5801, 5991	6, 6, 5	5.96715, 4.93312, 5.9506	4.64927, 4.04501, 4.54784	3.52158, 2	3	NA	NA
GL175572.1	129	854	772, 669, 708	12, 9, 7	13.8483, 8.21099, 8.69489	8.63436, 5.77858, 6.06379	10.86694,	3	NA	NA
GL175575.1	100	878	589, 591, 337	18, 16, 12	18.60558, 15.80758, 14.96175	10.16809, 9.01376, 9.20442	15.42473,	3	NA	NA
GL175575.1	4757	6098	5331, 5644, 5652	14, 22, 12	11.18507, 20.55767, 13.66628	6.6756, 10.13496, 8.53679	8.34587, 1	3	NA	NA
GL175579.1	13451	14563	13547, 13818, 13968, 14328	9, 9, 9, 5	8.35434, 8.21099, 10.72164, 5.40789	5.86444, 5.77858, 7.15493, 4.29296	5.71584, 5	3	NA	NA
GL175581.1	158	819	282, 663, 269	8, 8, 5	8.42257, 7.07159, 5.9506	5.97763, 5.20073, 4.54784	5.76336, 4	3	NA	NA
GL175581.1	13976	14449	14109, 13851, 14296, 14262	8, 8, 9, 7	7.47668, 5.14361, 6.12736, 7.72582	5.44269, 3.9808, 4.47368, 5.6058	4.89713, 2	3	NA	NA
GL175588.1	237	677	459, 539, 451	5, 9, 6	4.82177, 8.21099, 7.29403	3.98509, 5.77858, 5.30581	2.51418, 5	3	NA	NA
GL175591.1	8137	9396	8277, 9167, 8851	17, 16, 14	12.13217, 9.97172, 12.65033	6.53504, 5.50656, 7.57818	9.24555, 7	3	NA	NA
GL175601.1	7898	8360	8286, 8300, 8279	14, 26, 10	16.7649, 30.7371, 13.18184	9.96273, 14.8527, 8.33771	13.6543, 2	3	NA	NA
GL175603.1	59	331	134, 113, 262	16, 16, 9	19.7934, 17.16856, 11.64359	11.29109, 9.82359, 7.57973	16.57067,	3	NA	NA
GL175609.1	14085	14456	14117, 14266, 14125	12, 8, 9	13.8483, 7.07159, 11.64359	8.63436, 5.20073, 7.57973	10.86694,	3	NA	NA
GL175616.1	11546	12064	11639, 11641, 11646	47, 68, 40	56.33447, 76.84001, 48.22197	20.96081, 22.2275, 19.48161	52.3938, 7	3	NA	NA
GL175619.1	10111	10723	10571, 10549, 10335	20, 22, 10	13.16924, 12.15947, 7.50676	6.42128, 5.49423, 5.19045	10.22507,	3	NA	NA
GL175619.1	13696	14396	13932, 14072, 14336	20, 30, 10	13.29429, 19.78235, 7.50676	6.48954, 7.46604, 5.19045	10.34385,	3	NA	NA
GL175623.1	1572	2356	1960, 1961, 1937	46, 92, 34	34.11527, 74.99285, 28.20358	9.93161, 13.50405, 10.54359	30.51999,	3	NA	NA
GL175628.1	11608	12463	12131, 12021, 11893	8, 15, 9	5.40205, 10.15781, 7.83046	4.14825, 5.8257, 5.54742	3.04418, 7	3	NA	NA
GL175628.1	13603	14390	14051, 14038, 14072	21, 29, 14	21.0274, 27.29274, 14.79463	10.73595, 11.7232, 8.86428	17.7649, 2	3	NA	NA
GL175631.1	50	1222	188, 186, 192, 911	41, 50, 29, 15	41.81072, 49.03595, 30.62645, 12.54173	15.34424, 15.61481, 13.68043, 7.27181	38.07965,	3	U6	0
GL175631.1	12040	13645	12467, 12883, 12450	34, 29, 32	13.20289, 8.69842, 17.27784	4.3191, 3.3398, 6.00088	10.25795,	3	U6	0
GL175632.1	13431	14388	13917, 14189, 13903	11, 16, 9	12.43693, 17.16856, 11.64359	7.97018, 9.82359, 7.57973	9.52692, 1	3	NA	NA
GL175638.1	1314	1858	1369, 1423, 1636	7, 11, 5	7.1696, 10.15827, 5.9506	5.31345, 6.66366, 4.54784	4.61046, 7	3	NA	608
GL175642.1	13565	14332	14094, 14078, 14112	187, 276, 149	202.34657, 309.71588, 174.19759	26.45674, 30.43341, 30.00919	197.34647	3	NA	NA
GL175646.1	317	969	535, 469, 525	13, 10, 8	15.29169, 9.39128, 8.6841	9.29854, 6.35644, 6.11715	12.24396,	3	slc37a1	99
GL175647.1	13537	14283	13787, 13748, 13964	7, 10, 6	7.1696, 9.39128, 7.29403	5.31345, 6.35644, 5.30581	4.61046, 6	3	NA	NA
GL175648.1	78	720	469, 498, 460	14, 21, 13	14.10012, 19.11862, 16.0859	8.4555, 9.58634, 9.70995	11.10747,	3	spcs2	8767
GL175648.1	12707	13423	13406, 13355, 13372	10, 19, 10	10.26482, 19.11425, 12.71002	6.86644, 10.19015, 8.12734	7.48624, 1	3	spcs2	2483
GL175650.1	9233	9798	9426, 9416, 9270	8, 10, 5	7.28955, 7.8291, 5.80393	5.33177, 5.39387, 4.48186	4.72176, 5	3	NA	0
GL175652.1	7272	7821	7561, 7561, 7561	25, 38, 24	30.06855, 45.04887, 31.72657	14.7997, 18.50528, 16.02936	26.55945,	3	NA	NA
GL175660.1	35	991	620, 637, 653	36, 43, 21	40.44396, 46.9058, 26.58709	16.65775, 17.36748, 13.97388	36.73464,	3	NA	NA
GL175669.1	6991	7493	7128, 7132, 7184	6, 9, 8	5.96715, 8.21099, 10.1465	4.64927, 5.77858, 6.82176	3.52158, 5	3	NA	NA
GL175672.1	1992	2581	2281, 2412, 2443	5, 6, 3	4.82177, 4.93312, 3.47371	3.98509, 4.04501, 3.03189	2.51418, 2	3	hm13	0
GL175675.1	122	770	416, 505, 512	13, 10, 10	15.29169, 9.39128, 13.18184	9.29854, 6.35644, 8.33771	12.24396,	3	NA	NA
GL175676.1	5953	7238	6289, 6943, 6526	8, 6, 12	8.42257, 4.93312, 16.28773	5.97763, 4.04501, 9.81872	5.76336, 2	3	NA	NA
GL175690.1	7888	8937	8800, 8370, 8194	6, 13, 12	5.96715, 12.43433, 14.76955	4.64927, 7.66548, 9.10943	3.52158, 9	3	NA	NA
GL175702.1	2575	3630	2725, 3264, 2870, 3310	10, 20, 6, 6	6.8116, 12.48105, 4.07578, 4.07578	4.75, 6.05084, 3.49745, 3.49745	4.30355, 9	3	NA	NA
GL175702.1	2575	3630	3485, 3264, 2870, 3310	10, 20, 6, 6	6.75634, 12.48105, 4.07578, 4.07578	4.71499, 6.05084, 3.49745, 3.49745	4.25291, 9	3	NA	NA
GL175705.1	12916	13953	13497, 13377, 13498	10, 10, 3	11.06011, 9.39128, 3.47371	7.306, 6.35644, 3.03189	8.22712, 6	3	NA	NA
GL175708.1	11613	12105	11845, 11845, 11846	13, 21, 22	14.79381, 24.35402, 31.08818	9.02996, 12.71289, 16.11669	11.77132,	3	NA	NA
GL175709.1	2316	2674	2463, 2565, 2340	5, 6, 4	4.82177, 4.93312, 4.67325	3.98509, 4.04501, 3.78987	2.51418, 2	3	NA	NA
GL175718.1	1856	2384	1945, 2280, 2132	9, 19, 6	6.28237, 11.87408, 3.96106	4.57292, 5.93761, 3.42191	3.81457, 9	3	NA	NA
GL175718.1	6913	7470	7217, 7202, 7219	10, 20, 10	8.04647, 14.94655, 8.43366	5.53044, 7.41618, 5.77207	5.43084, 1	3	NA	NA
GL175718.1	13327	13827	13525, 13440, 13396	6, 14, 4	5.63898, 11.71767, 3.54079	4.46544, 7.00416, 3.20587	3.25772, 9	3	NA	NA
GL175720.1	5411	6110	5683, 5705, 5470	8, 7, 4	8.42257, 5.97725, 4.67325	5.97763, 4.62287, 3.78987	5.76336, 3	3	NA	NA
GL175723.1	2883	3220	2958, 3050, 2916	6, 11, 6	5.96715, 10.60895, 7.29403	4.64927, 6.9343, 5.30581	3.52158, 7	3	NA	NA
GL175726.1	13018	13693	13065, 13079, 13470	14, 9, 5	16.7649, 8.21099, 5.9506	9.96273, 5.77858, 4.54784	13.6543, 5	3	NA	1038
GL175727.1	12703	13543	12881, 12854, 12870	184, 271, 173	219.82912, 331.54373, 215.15442	32.97944, 37.41304, 35.70486	214.74582	3	NA	NA
GL175730.1	883	1615	1487, 1405, 1171	12, 10, 11	9.49573, 7.68845, 11.49087	6.06403, 5.30522, 7.44794	6.77365, 5	3	NA	NA
GL175730.1	5806	6300	5939, 6209, 6222	13, 7, 11	11.35857, 5.55323, 12.78973	7.00844, 4.35994, 8.15619	8.51036, 3	3	NA	NA
GL175730.1	11868	12453	12098, 12291, 11992	13, 23, 19	14.97989, 26.02147, 24.11676	9.13126, 13.07981, 13.07033	11.94968,	3	NA	NA
GL175732.1	229	1751	886, 742, 689	12, 14, 8	13.8483, 14.45897, 10.1465	8.63436, 8.66788, 6.82176	10.86694,	3	NA	NA
GL175735.1	8013	8518	8039, 8020, 8020	29, 36, 21	32.34238, 36.32899, 25.89737	14.70163, 14.2686, 13.59243	28.7832, 3	3	NA	NA
GL175737.1	62	1203	761, 263, 759	19, 31, 15	15.83498, 28.99774, 13.21168	8.20955, 11.98601, 7.68323	12.76571,	3	NA	NA
GL175737.1	6629	8767	7421, 7951, 7368	42, 49, 33	24.43468, 24.52679, 22.3554	7.01978, 6.05442, 7.96353	21.06922,	3	NA	NA
GL175737.1	12991	13823	13708, 12911, 13615, 13034, 13643	22, 14, 21, 14, 11	16.06988, 4.67461, 12.36475, 9.14822, 7.51335	7.52771, 2.98739, 5.78514, 5.43495, 5.01728	12.99155,	3	NA	NA
GL175739.1	8412	9330	9078, 9147, 8772	23, 28, 18	24.94963, 30.12516, 21.79524	12.44303, 13.76034, 12.01217	21.56948,	3	NA	NA
GL175741.1	7119	7537	7508, 7496, 7507	53, 93, 35	55.0617, 94.06258, 35.55172	17.52575, 19.93888, 14.22002	51.13792,	3	5S_rRNA	221
GL175741.1	7758	8680	8615, 8608, 8166	29, 44, 23	13.08072, 21.39883, 14.35437	4.82304, 5.73908, 6.38065	10.14032,	3	5S_rRNA	0
GL175741.1	12840	13739	12899, 12900, 12897	107, 112, 68	108.56062, 111.51984, 69.52004	20.74785, 20.20782, 18.59925	104.1002,	3	5S_rRNA	3659
GL175742.1	167	1851	1465, 605, 714	18, 11, 24	14.64385, 7.30109, 26.99032	7.77164, 4.88441, 13.32427	11.62766,	3	NA	NA
GL175747.1	69	751	363, 373, 359	18, 27, 8	22.92104, 33.57398, 10.1465	12.61945, 16.16014, 6.82176	19.59912,	3	NA	NA
GL175764.1	11244	12258	11713, 11523, 11533	15, 21, 10	10.70853, 16.62178, 6.99932	6.15483, 8.11097, 4.86901	7.90562, 1	3	hdac6	262
GL175764.1	12718	13752	13138, 13522, 13249	21, 21, 32	17.41956, 17.40612, 35.43546	8.57638, 8.56849, 15.3579	14.28349,	3	hdac6	1736
GL175766.1	22	422	317, 314, 313	21, 21, 10	17.13103, 15.906, 9.0947	8.40727, 7.69984, 6.17787	14.00622,	3	NA	NA
GL175766.1	900	1821	1532, 1466, 1199	26, 18, 11	21.91354, 12.05578, 10.28376	9.65188, 6.25801, 6.7395	18.62283,	3	NA	NA
GL175766.1	6321	7276	6376, 6372, 7102, 6643, 7205	10, 10, 11, 4, 4	6.86768, 7.32562, 10.60895, 4.67325, 4.67325	4.78555, 5.07581, 6.9343, 3.78987, 3.78987	4.35559, 4	3	NA	NA
GL175766.1	12975	13696	13467, 13178, 13557	19, 12, 10	21.50921, 11.86108, 13.11399	11.61175, 7.51216, 8.30815	18.23049,	3	NA	NA
GL175770.1	2715	3129	2839, 2823, 2883	13, 12, 13	10.0641, 6.99976, 11.9555	6.20681, 4.52293, 7.37476	7.29669, 4	3	NA	NA
GL175770.1	7225	7968	7392, 7619, 7517	6, 12, 5	4.04056, 7.96865, 3.97425	3.47431, 5.1141, 3.48505	1.89399, 5	3	NA	NA
GL175770.1	12288	13596	12691, 12621, 13054	8, 7, 6	8.4095, 5.97725, 7.29403	5.97056, 4.62287, 5.30581	5.76336, 3	3	NA	NA
GL175784.1	53	1443	850, 923, 413	24, 20, 10	30.08076, 21.69995, 11.6791	15.11859, 11.44483, 7.62925	26.57127,	3	NA	NA
GL175784.1	7129	9793	7732, 8124, 7346, 9264	12, 26, 8, 11	7.18196, 15.13603, 5.46433, 7.20472	4.63296, 6.1494, 4.18852, 4.8242	4.62187, 1	3	NA	NA
GL175784.1	7129	9793	9360, 8124, 7346, 9264	18, 26, 8, 11	11.77245, 15.13603, 5.46433, 7.20472	6.09838, 6.1494, 4.18852, 4.8242	8.90383, 1	3	NA	NA
GL175784.1	9995	11363	10511, 10906, 10518, 10944	19, 22, 9, 10	12.75045, 11.04228, 5.41905, 6.29342	6.41935, 4.96845, 4.02017, 4.42218	9.82585, 8	3	NA	NA
GL175784.1	11812	13382	12300, 12526, 12579	10, 26, 16	4.98839, 15.11015, 12.6443	3.61037, 6.13749, 7.086	2.66874, 1	3	NA	NA
GL175784.1	11812	13382	13003, 12526, 12579	12, 26, 16	6.97595, 15.11015, 12.6443	4.50859, 6.13749, 7.086	4.45626, 1	3	NA	NA
GL175785.1	11675	12353	11908, 11919, 12008	5, 11, 4	4.82177, 10.60895, 4.67325	3.98509, 6.9343, 3.78987	2.51418, 7	3	NA	NA
GL175787.1	8614	9579	9148, 9312, 9093	15, 16, 9	7.7845, 6.01417, 5.61083	4.46931, 3.4323, 4.14281	5.18583, 3	3	NA	NA
GL175787.1	12282	13602	12782, 13117, 12764	36, 75, 23	31.38783, 72.42667, 21.51828	11.5568, 17.30941, 10.38632	27.84896,	3	NA	NA
GL175792.1	5420	5714	5473, 5471, 5482	16, 27, 19	16.08763, 28.00888, 23.88486	9.18228, 12.86965, 12.94573	13.00852,	3	fam219a	2194
GL175793.1	2531	3309	2741, 2747, 2759	11, 12, 4	11.02968, 10.08092, 4.67325	7.18222, 6.42828, 3.78987	8.20839, 7	3	NA	NA
GL175794.1	12126	12796	12366, 12272, 12506	4, 8, 4	3.74195, 7.07159, 4.67325	3.32091, 5.20073, 3.78987	1.6222, 4.6	3	NA	NA
GL175796.1	7313	7761	7489, 7681, 7582	7, 12, 5	5.30682, 6.55643, 3.39492	4.20391, 4.2579, 3.11232	2.95991, 4	3	NA	NA
GL175799.1	11009	11475	11318, 11317, 11285	16, 23, 12	15.41236, 22.10472, 13.22602	8.77469, 10.73683, 8.2961	12.35995,	3	NA	NA
GL175804.1	4417	5490	4931, 4458, 4577	14, 13, 9	12.38175, 10.94367, 10.01275	7.4133, 6.7521, 6.79683	9.48175, 8	3	NA	NA
GL175804.1	10727	11084	10823, 10989, 11044	9, 15, 16	8.45482, 15.70444, 19.65507	5.92419, 9.18918, 11.21774	5.79307, 1	3	NA	NA
GL175807.1	2639	3688	3298, 3452, 3306	28, 38, 26	20.32407, 25.30672, 21.13827	8.21908, 8.12568, 9.21728	17.08378,	3	NA	NA
GL175807.1	5368	7023	5823, 5724, 5704	36, 53, 38	29.92788, 42.88671, 36.36931	10.80236, 11.75155, 13.51774	26.42215,	3	NA	NA
GL175807.1	12662	13465	13011, 13069, 13074	12, 22, 6	13.8483, 25.38455, 7.29403	8.63436, 13.01836, 5.30581	10.86694,	3	NA	NA
GL175810.1	10227	10535	10351, 10356, 10401	7, 16, 4	6.25077, 14.63495, 4.67325	4.78814, 8.3013, 3.78987	3.78441, 1	3	NA	NA
GL175812.1	562	1253	682, 816, 708	11, 11, 9	12.43693, 10.60895, 11.30431	7.97018, 6.9343, 7.42891	9.52692, 7	3	NA	NA
GL175812.1	5640	6698	6001, 6041, 6461	12, 26, 17	13.55951, 27.56619, 22.00127	8.47903, 12.96862, 12.29583	10.59689,	3	NA	NA
GL175812.1	12147	13488	12495, 12492, 12621	30, 39, 17	32.20277, 42.09095, 21.76302	14.23201, 16.33801, 12.17337	28.647, 38	3	NA	NA
GL175821.1	3749	5114	4451, 4490, 4573	26, 36, 28	23.43853, 30.83754, 29.91746	10.52556, 11.2696, 13.63653	20.10161,	3	NA	NA
GL175824.1	2724	3289	3145, 3123, 3088	12, 15, 18	9.86028, 11.68429, 21.11592	6.29117, 6.74649, 11.63307	7.1041, 8.9	3	NA	NA
GL175824.1	11475	12113	11830, 11803, 11816	13, 9, 11	12.43975, 6.02249, 10.98082	7.66875, 4.4065, 7.15369	9.5289, 3.6	3	NA	NA
GL175828.1	11733	12344	11898, 11983, 11873	28, 26, 14	29.64365, 23.91458, 15.20637	13.4734, 10.80262, 9.10193	26.14402,	3	NA	10757
GL175833.1	86	1008	568, 559, 574	21, 41, 17	15.12931, 29.45045, 12.96552	7.26173, 9.19122, 7.02824	12.09267,	3	NA	NA
GL175833.1	3630	4140	4036, 3746, 3704	23, 20, 20	13.15017, 6.79667, 12.46926	5.78652, 3.37957, 6.04457	10.20667,	3	NA	NA
GL175833.1	12525	13260	12929, 12707, 12998	18, 17, 9	14.68958, 12.5571, 8.88966	7.79908, 6.78543, 6.17838	11.67146,	3	NA	NA
GL175839.1	106	1178	840, 642, 658	16, 24, 25	13.43192, 21.24533, 24.59259	7.56568, 9.90118, 11.5462	10.47512,	3	NA	NA
GL175839.1	4556	5699	5582, 4935, 4724	12, 20, 13	9.65435, 16.16593, 9.95131	6.16292, 8.12509, 6.13691	6.92291, 1	3	NA	NA
GL175839.1	11812	13139	12796, 12723, 12254	17, 19, 12	18.1272, 21.4153, 14.96175	10.1466, 11.55717, 9.20442	14.96605,	3	NA	NA
GL175849.1	11571	12206	11628, 11687, 11636	25, 30, 17	32.58788, 33.83243, 20.98465	16.22977, 15.2009, 11.76329	29.02357,	3	NA	NA
GL175852.1	1317	1963	1688, 1489, 1441	8, 10, 6	7.52511, 9.39128, 7.29403	5.47115, 6.35644, 5.30581	4.94319, 6	3	NA	NA
GL175854.1	12298	12801	12398, 12408, 12398	18, 18, 16	22.92104, 19.97713, 23.00461	12.61945, 10.97931, 12.83987	19.59912,	3	NA	NA
GL175856.1	47	407	183, 313, 170	21, 21, 13	15.98597, 11.84745, 10.11375	7.74544, 5.52521, 6.23758	12.91088,	3	NA	NA
GL175856.1	598	1272	793, 797, 783	23, 30, 14	14.71135, 17.38014, 9.27106	6.56209, 6.39081, 5.50843	11.69266,	3	NA	NA
GL175856.1	5003	5635	5163, 5162, 5120	23, 33, 15	10.83982, 15.81905, 7.52775	4.7278, 5.30442, 4.33006	8.02935, 1	3	NA	NA
GL175856.1	6169	7384	6606, 7193, 6616	21, 26, 17	12.51874, 13.63657, 12.26002	5.86358, 5.47958, 6.61012	9.60511, 1	3	NA	NA
GL175856.1	11975	13159	12603, 12582, 12596	31, 45, 22	34.54899, 49.63757, 25.21195	15.23237, 18.03561, 12.91737	30.94432,	3	NA	NA
GL175865.1	6848	7502	7132, 7273, 7217	5, 15, 6	4.23867, 12.22425, 6.2131	3.64743, 7.07697, 4.78171	2.0685, 9.4	3	cfp	3068
GL175869.1	9214	10087	9683, 9605, 9351	7, 11, 11	6.84137, 9.95469, 13.1143	5.13151, 6.5399, 8.32282	4.33107, 7	3	NA	NA
GL175873.1	8907	9448	9180, 9141, 9258	16, 17, 8	15.74146, 14.91871, 8.56955	8.97386, 8.20906, 6.05652	12.67506,	3	fgf3	0
GL175887.1	10071	10427	10398, 10381, 10398	20, 18, 14	21.61731, 16.88096, 16.46576	11.39575, 9.12607, 9.80275	18.33537,	3	NA	NA
GL175887.1	12277	12578	12310, 12333, 12316	14, 16, 10	14.10012, 15.54288, 11.24131	8.4555, 8.85379, 7.40241	11.10747,	3	NA	NA
GL175888.1	4002	5185	4767, 4396, 4509	24, 25, 16	22.1716, 20.68479, 15.94441	10.44632, 9.26557, 9.0962	18.87289,	3	NA	NA
GL175889.1	6898	7542	6925, 7152, 7146	11, 7, 4	12.43693, 5.97725, 4.67325	7.97018, 4.62287, 3.78987	9.52692, 3	3	NA	NA
GL175890.1	117	709	320, 244, 323	8, 16, 8	5.15794, 9.26992, 6.38723	3.99009, 5.11161, 4.77798	2.82436, 6	3	NA	NA
GL175890.1	12312	12729	12526, 12515, 12383	23, 26, 7	19.45826, 19.6381, 5.98393	9.17156, 8.3981, 4.62695	16.24768,	3	NA	NA
GL175896.1	681	1367	1247, 970, 823	4, 5, 3	3.74195, 3.94561, 3.47371	3.32091, 3.46715, 3.03189	1.6222, 1.8	3	trim45	2594
GL175900.1	4596	5391	5239, 5245, 5245	14, 23, 26	13.96392, 22.45275, 30.74385	8.37431, 10.94541, 14.85666	10.9769, 1	3	lix1l	0
GL175903.1	1589	1910	1765, 1744, 1804	19, 29, 9	16.233, 26.25787, 7.06537	8.44813, 11.13033, 5.07099	13.1477, 2	3	lrrc24	1978
GL175903.1	4218	4723	4664, 4601, 4650	12, 14, 8	7.32501, 7.45952, 5.37675	4.71975, 4.45321, 4.13188	4.75565, 5	3	lrrc24	0
GL175908.1	774	1545	1076, 838, 889	15, 41, 19	12.43022, 41.88918, 20.8607	7.20335, 15.38792, 11.2325	9.52692, 3	3	NA	NA
GL175912.1	11652	12405	11766, 12153, 12163	7, 10, 6	7.10297, 9.39128, 7.29403	5.27706, 6.35644, 5.30581	4.57191, 6	3	NA	828
GL175916.1	2026	2645	2584, 2484, 2090	7, 8, 4	6.2098, 6.14619, 4.30163	4.76362, 4.62573, 3.61556	3.74679, 3	3	cbs	2575
GL175922.1	11961	12523	12230, 12174, 12187	23, 23, 11	27.97737, 27.36982, 14.75772	14.21807, 13.8686, 9.09568	24.51834,	3	NA	NA
GL175926.1	73	654	380, 447, 258	8, 10, 3	8.42257, 8.20397, 3.47371	5.97763, 5.62902, 3.03189	5.76336, 5	3	NA	NA
GL175928.1	3554	3764	3624, 3644, 3727	8, 10, 12	7.99469, 8.88645, 13.7434	5.74128, 6.0511, 8.57827	5.38155, 6	3	NANOS2	740
GL175928.1	9206	10136	9460, 9625, 9739	6, 7, 8	5.96715, 5.97725, 9.05167	4.64927, 4.62287, 6.30653	3.52158, 3	3	NANOS2	6392
GL175933.1	61	796	510, 513, 466	34, 56, 25	24.02668, 42.68841, 18.85911	8.4915, 10.89016, 8.25347	20.673, 39	3	NA	NA
GL175933.1	3584	4426	4063, 4060, 4243	49, 60, 37	28.06348, 31.83695, 23.72862	7.12257, 6.72713, 7.68154	24.60268,	3	NA	NA
GL175933.1	7717	8470	7981, 7923, 7976	28, 32, 24	10.9433, 10.09636, 12.62833	4.16168, 3.54923, 5.36511	8.12738, 7	3	NA	NA
GL175937.1	304	1070	1025, 988, 850	9, 7, 6	9.72082, 5.97725, 7.29403	6.64182, 4.62287, 5.30581	6.97155, 3	3	NA	NA
GL175948.1	858	1360	1200, 1200, 1200	13, 11, 10	15.29169, 10.60895, 13.18184	9.29854, 6.9343, 8.33771	12.24396,	3	NA	NA
GL175950.1	2044	2647	2450, 2476, 2428	20, 20, 5	24.33651, 22.87459, 5.59755	12.96942, 12.13503, 4.38538	20.9737, 1	3	NA	NA
GL175954.1	2940	3690	3090, 3260, 3091	14, 25, 9	12.0338, 22.53775, 10.14484	7.19911, 10.33209, 6.86555	9.15254, 1	3	NA	NA
GL175962.1	5153	5867	5411, 5517, 5445	11, 11, 7	12.43693, 10.60895, 8.69489	7.97018, 6.9343, 6.06379	9.52692, 7	3	NA	NA
GL175968.1	2058	2775	2396, 2396, 2395	28, 32, 21	32.26664, 34.05502, 27.4641	15.03533, 14.54146, 14.44688	28.70906,	3	NA	NA
GL175969.1	45	1028	782, 820, 658	18, 24, 9	17.58399, 22.6569, 9.30116	9.55259, 10.73418, 6.41174	14.44229,	3	NA	NA
GL175970.1	585	954	760, 843, 791	12, 23, 15	6.25895, 13.03901, 10.51545	4.08243, 5.73311, 6.03897	3.79237, 1	3	NA	NA
GL175975.1	2704	3309	3060, 3060, 2907	22, 26, 13	23.7162, 29.93677, 16.0859	12.03186, 14.38093, 9.70995	20.37115,	3	NA	NA
GL175991.1	6	763	333, 261, 255	55, 60, 36	58.10873, 64.58636, 38.17169	18.27688, 19.48993, 15.32838	54.14406,	3	NA	NA
GL175991.1	5848	12083	11744, 8058, 6434, 9179, 11743	111, 142, 58, 74, 56	64.03857, 67.58179, 29.14861, 39.52466, 27.9503	8.15858, 6.50251, 6.26549, 7.00587, 6.17872	60.0055, 6	3	NA	NA
GL175993.1	8399	8858	8515, 8578, 8586	8, 8, 5	8.10827, 7.07159, 5.14967	5.80497, 5.20073, 4.16141	5.48761, 4	3	NA	NA
GL175995.1	632	1053	668, 659, 656	20, 35, 19	22.97335, 41.28206, 24.72671	12.19234, 17.57923, 13.39257	19.64963,	3	NA	NA
GL175997.1	107	1247	573, 898, 364	10, 14, 9	10.96713, 14.45897, 11.64359	7.25596, 8.66788, 7.57973	8.14942, 1	3	NA	NA
GL175998.1	43	1167	616, 752, 591	51, 67, 37	35.59111, 42.83221, 29.20522	9.35596, 8.75802, 10.14241	31.96634,	3	NA	NA
GL175998.1	3709	4369	4076, 4050, 4079	37, 52, 22	19.07605, 25.70369, 11.63136	5.90507, 6.0319, 5.24242	15.87864,	3	NA	NA
GL175998.1	6125	6338	6155, 6194, 6229	16, 18, 11	6.72902, 5.29417, 5.31563	3.77764, 2.93305, 3.67196	4.22745, 3	3	NA	NA
GL175998.1	11187	12097	11733, 11690, 11845	12, 20, 11	12.90199, 19.50661, 11.4314	8.11489, 10.12815, 7.41405	9.97033, 1	3	NA	NA
GL176007.1	8856	9131	8892, 8962, 8890	18, 27, 5	18.68415, 27.84104, 5.59755	10.2151, 12.76965, 4.38538	15.49996,	3	arhgef15	0
GL176008.1	3234	3820	3701, 3707, 3705	530, 606, 453	563.84351, 620.33459, 505.99008	28.47639, 26.20087, 31.68571	558.06012	3	NA	NA
GL176011.1	331	1879	1081, 1079, 1066	21, 36, 18	22.48839, 40.05243, 22.76598	11.61323, 16.4274, 12.53748	19.18044,	3	NA	NA
GL176011.1	6323	6808	6664, 6513, 6476	12, 25, 11	12.3187, 25.58747, 13.55304	7.78073, 12.14055, 8.54092	9.4231, 22	3	NA	NA
GL176015.1	72	1491	391, 562, 1049	11, 13, 10	12.33515, 13.14514, 13.11399	7.91559, 8.09002, 8.30815	9.43699, 1	3	NA	NA
GL176015.1	7576	8205	8010, 8016, 8014	20, 21, 13	18.89193, 20.12483, 13.97446	9.7566, 10.19169, 8.57174	15.7007, 1	3	NA	NA
GL176018.1	11213	11923	11697, 11541, 11545	5, 14, 11	4.23867, 12.97647, 12.34038	3.64743, 7.77766, 7.91841	2.0685, 10	3	NA	7277
GL176020.1	6308	7077	6424, 6655, 6875	26, 25, 23	12.45526, 7.5719, 11.95407	4.98122, 3.25925, 5.22481	9.54432, 5	3	NA	NA
GL176020.1	7764	8522	8188, 8211, 8176	26, 28, 19	12.26511, 10.18088, 9.15284	4.90342, 3.89755, 4.54338	9.37175, 7	3	NA	NA
GL176020.1	10182	11747	10350, 10325, 11370	19, 36, 22	7.1632, 15.92543, 11.83963	3.63217, 4.97273, 5.34103	4.61046, 1	3	NA	NA
GL176020.1	10182	11747	11362, 10325, 11370	22, 36, 22	9.64177, 15.92543, 11.83963	4.34671, 4.97273, 5.34103	6.91063, 1	3	NA	NA
GL176027.1	3270	4442	3476, 3800, 4350, 3721, 4259	14, 10, 14, 5, 7	12.75226, 6.50583, 10.22245, 4.74091, 6.92462	7.64062, 4.55637, 6.08441, 3.94006, 5.17829	9.82689, 4	3	NA	NA
GL176027.1	10000	11928	10913, 9931, 11262, 11302, 11752	23, 22, 26, 22, 7	21.67593, 16.27156, 21.35565, 23.85667, 5.37469	10.48041, 7.6398, 9.33842, 12.11568, 4.2471	18.39184,	3	NA	NA
GL176038.1	4766	5573	5259, 5034, 5296	8, 6, 15	7.77807, 4.73925, 17.23823	5.618, 3.92426, 10.06864	5.17956, 2	3	NA	NA
GL176038.1	11022	11825	11340, 11528, 11683	9, 10, 8	9.72082, 9.39128, 9.76268	6.64182, 6.35644, 6.64964	6.97155, 6	3	NA	NA
GL176041.1	25	794	453, 370, 668	11, 16, 7	12.43693, 17.16856, 8.15288	7.97018, 9.82359, 5.81587	9.52692, 1	3	hpx	41
GL176046.1	6430	7196	6823, 6915, 6601	5, 14, 6	4.82177, 14.45897, 6.93817	3.98509, 8.66788, 5.14396	2.51418, 1	3	NA	NA
GL176046.1	7565	7769	7670, 7662, 7627	12, 10, 8	13.8483, 9.39128, 9.76268	8.63436, 6.35644, 6.64964	10.86694,	3	NA	NA
GL176048.1	330	766	658, 735, 735	10, 11, 12	11.06011, 10.60895, 15.5838	7.306, 6.9343, 9.50165	8.22712, 7	3	NA	NA
GL176049.1	4411	5732	4873, 5288, 4424, 5283	15, 35, 5, 19	10.37959, 32.40594, 3.28985, 19.09738	5.95774, 12.45123, 3.04259, 10.17998	7.59574, 2	3	NA	NA
GL176049.1	4786	5732	5305, 5288, 5283	27, 35, 19	24.25827, 32.40594, 19.09738	10.6651, 12.45123, 10.17998	20.89803,	3	NA	NA
GL176049.1	10829	11797	11614, 10905, 11084, 11734	10, 18, 10, 7	9.08301, 16.12237, 10.71812, 7.05993	6.17079, 8.66512, 7.12015, 5.25341	6.3862, 13	3	NA	NA
GL176062.1	2050	2951	2234, 2257, 2244	6, 11, 4	5.96715, 10.60895, 4.67325	4.64927, 6.9343, 3.78987	3.52158, 7	3	NA	NA
GL176072.1	37	992	241, 241, 165	64, 119, 47	61.79903, 119.44893, 49.47888	16.52863, 20.79435, 17.07298	57.79148,	3	NA	NA
GL176072.1	7395	7862	7575, 7681, 7698	10, 8, 5	10.96713, 7.06103, 5.9506	7.25596, 5.19433, 4.54784	8.14942, 4	3	NA	NA
GL176086.1	120	623	574, 571, 556	17, 38, 14	9.87535, 25.86794, 9.5258	5.2552, 8.36319, 5.66153	7.11892, 2	3	NA	NA
GL176086.1	2681	3043	2754, 2814, 2811	18, 24, 12	6.40126, 7.14302, 4.77446	3.40273, 3.18492, 3.24049	3.92577, 4	3	NA	NA
GL176086.1	11130	11664	11483, 11464, 11287	39, 84, 37	21.66474, 55.1208, 26.72173	6.52087, 9.44486, 8.97783	18.38135,	3	NA	NA
GL176089.1	10510	11600	11324, 10843, 10753, 11318	12, 21, 11, 9	9.23027, 16.40465, 11.20072, 8.69919	5.8984, 7.98556, 7.28154, 6.06795	6.52452, 1	3	NA	NA
GL176090.1	10601	11335	11020, 10836, 11125	4, 9, 11	3.74195, 7.92635, 12.48556	3.32091, 5.60609, 7.99611	1.6222, 5.4	3	NA	NA
GL176091.1	67	928	70, 567, 538	6, 7, 6	5.96715, 5.97725, 7.29403	4.64927, 4.62287, 5.30581	3.52158, 3	3	NA	NA
GL176092.1	10669	11591	11064, 11123, 10980	14, 12, 4	16.03678, 11.86108, 4.67325	9.5688, 7.51216, 3.78987	12.95916,	3	NA	NA
GL176094.1	10033	10551	10188, 10087, 10301	9, 7, 5	9.72082, 5.97725, 5.9506	6.64182, 4.62287, 4.54784	6.97155, 3	3	NA	NA
GL176096.1	51	1144	255, 312, 167, 876	11, 7, 8, 6	11.56615, 5.97725, 8.80246, 6.25896	7.49066, 4.62287, 6.179, 4.80589	8.70729, 3	3	NA	NA
GL176096.1	10396	11548	10762, 10908, 10918	13, 17, 7	14.79381, 18.56115, 8.69489	9.02996, 10.40145, 6.06379	11.77132,	3	NA	NA
GL176100.1	9734	10320	9830, 9744, 9764	9, 13, 10	9.72082, 13.14514, 10.97191	6.64182, 8.09002, 7.25854	6.97155, 1	3	NA	NA
GL176101.1	10053	11302	10900, 10109, 11100	6, 10, 5	5.96715, 9.39128, 5.9506	4.64927, 6.35644, 4.54784	3.52158, 6	3	NA	NA
GL176103.1	5135	5823	5475, 5452, 5437	80, 75, 49	38.91275, 32.21656, 23.12909	6.31161, 5.43502, 5.66407	35.2277, 2	3	5S_rRNA	0
GL176103.1	7205	8913	8163, 7805, 7776	164, 183, 129	102.80405, 111.17293, 88.00001	9.54406, 9.18918, 10.61079	98.39004,	3	5S_rRNA	0
GL176103.1	9769	11537	10876, 10583, 10581	166, 191, 130	112.33213, 124.7904, 93.11607	10.75071, 10.29383, 11.47893	107.84285	3	5S_rRNA	0
GL176114.1	4450	5217	4909, 5010, 5093	9, 13, 5	9.72082, 13.14514, 5.9506	6.64182, 8.09002, 4.54784	6.97155, 1	3	NA	NA
GL176118.1	94	735	421, 456, 447	160, 170, 192	173.25252, 181.66646, 225.61617	25.77671, 25.3314, 32.00992	168.40599	3	NA	NA
GL176118.1	5383	6163	5493, 5485, 5482	71, 95, 36	47.43694, 68.66449, 19.32597	9.42515, 11.15278, 6.14412	43.62118,	3	NA	NA
GL176118.1	6938	7353	7062, 7080, 7050	43, 46, 36	23.90893, 24.3162, 24.77526	6.67684, 6.37511, 8.34938	20.55807,	3	NA	NA
GL176118.1	10849	11357	10949, 11003, 10963	37, 24, 20	27.41924, 14.43488, 14.54507	9.29693, 6.21219, 7.18707	23.97272,	3	NA	NA
GL176136.1	54	412	212, 240, 149	11, 12, 7	10.00935, 9.74388, 7.10639	6.57321, 6.2187, 5.27894	7.24529, 7	3	NA	1058
GL176136.1	2423	2788	2554, 2557, 2574	7, 20, 8	5.52352, 19.03817, 8.24665	4.34124, 9.84499, 5.88165	3.15454, 1	3	NA	0
GL176137.1	5681	6569	5820, 6052, 6480	6, 15, 3	4.20394, 13.58442, 3.47371	3.58131, 7.91173, 3.03189	2.04024, 1	3	NA	NA
GL176140.1	16	516	146, 134, 157	55, 100, 35	61.24626, 110.06155, 40.68607	19.95736, 23.93201, 17.22635	57.24426,	3	NA	NA
GL176143.1	1421	1788	1500, 1493, 1462	8, 10, 22	8.42257, 9.39128, 28.97125	5.97763, 6.35644, 15.03088	5.76336, 6	3	NA	NA
GL176144.1	1346	1616	1420, 1432, 1508	36, 50, 18	13.29333, 16.24549, 7.08666	4.16783, 3.92197, 3.70829	10.34304,	3	NA	NA
GL176144.1	2765	3967	3167, 3335, 3343	131, 209, 73	92.61958, 150.91963, 52.90117	11.25061, 12.19462, 10.69305	88.29523,	3	NA	NA
GL176144.1	7571	7953	7697, 7741, 7739	93, 131, 60	57.86833, 77.78284, 41.57828	8.87721, 8.6397, 9.61233	53.90695,	3	NA	NA

GL176147.1	6671	7169	6678, 6678, 6678	56, 87, 62	62.51835, 91.74436, 74.07279	20.18998, 21.19392, 23.64698	58.50275,	3	NA	NA
GL176147.1	10036	10954	10324, 10234, 10127, 10521	11, 21, 7, 13	7.0209, 12.21319, 3.76643, 9.12714	4.7096, 5.70842, 3.1972, 5.62823	4.49748, 9	3	NA	NA
GL176148.1	10666	11175	10770, 11024, 11042	7, 16, 22	7.1696, 17.16856, 27.94332	5.31345, 9.82359, 14.47367	4.61046, 1	3	NA	NA
GL176151.1	5455	6124	5789, 5786, 5744	13, 15, 13	12.38021, 13.01314, 16.49347	7.63279, 7.56134, 9.91245	9.48056, 1	3	NA	1731
GL176155.1	8767	9671	9474, 8891, 9410	8, 7, 8	7.72597, 5.68499, 9.92329	5.588, 4.44244, 6.7228	5.13061, 3	3	NA	NA
GL176165.1	48	644	411, 226, 310	7, 6, 11	7.1696, 4.93312, 12.9493	5.31345, 4.04501, 8.23866	4.61046, 2	3	NA	NA
GL176176.1	9327	10055	9656, 9608, 9641	7, 17, 7	7.1696, 18.56115, 8.69489	5.31345, 10.40145, 6.06379	4.61046, 1	3	NA	NA
GL176180.1	4033	4741	4453, 4335, 4236	5, 10, 4	4.82177, 9.39128, 4.67325	3.98509, 6.35644, 3.78987	2.51418, 6	3	med21	0
GL176182.1	10289	11071	10750, 10631, 10515	6, 8, 6	5.96715, 7.07159, 7.29403	4.64927, 5.20073, 5.30581	3.52158, 4	3	NA	NA
GL176187.1	3482	4462	4152, 4366, 3734	20, 13, 12	17.15608, 9.96603, 10.50755	8.7123, 6.14603, 6.69224	14.0307, 7	3	NA	NA
GL176192.1	10308	10895	10650, 10801, 10482	4, 16, 9	3.74195, 17.16856, 11.64359	3.32091, 9.82359, 7.57973	1.6222, 14	3	NA	NA
GL176200.1	10390	10892	10543, 10521, 10622	8, 10, 5	8.42257, 9.39128, 5.9506	5.97763, 6.35644, 4.54784	5.76336, 6	3	NA	NA
GL176202.1	10132	10963	10259, 10380, 10581	7, 10, 6	7.1696, 9.39128, 7.29403	5.31345, 6.35644, 5.30581	4.61046, 6	3	NA	NA
GL176214.1	2148	2729	2416, 2323, 2406	5, 11, 9	4.82177, 10.60895, 11.04428	3.98509, 6.9343, 7.30896	2.51418, 7	3	NA	NA
GL176215.1	2065	2719	2444, 2696, 2259	5, 6, 5	4.82177, 4.62266, 5.59755	3.98509, 3.8507, 4.38538	2.51418, 2	3	NA	NA
GL176218.1	5857	6727	6028, 6030, 5965	21, 34, 14	17.71848, 27.50511, 13.38506	8.75244, 10.18474, 8.02606	14.5719, 2	3	NA	NA
GL176219.1	60	1212	646, 834, 607	100, 110, 80	106.42235, 109.17214, 90.77878	22.40081, 20.00253, 23.71694	101.98064	3	NA	NA
GL176219.1	10410	10950	10738, 10731, 10724	35, 41, 27	39.83972, 44.79989, 34.50341	16.7256, 17.03635, 16.69193	36.13961,	3	NA	NA
GL176221.1	9748	10914	10470, 10485, 10558	11, 8, 9	12.43693, 7.07159, 11.64359	7.97018, 5.20073, 7.57973	9.52692, 4	3	NA	4250
GL176223.1	2290	4649	2830, 3363, 3346	28, 42, 21	24.25926, 35.2086, 21.05831	10.33506, 11.54, 10.75458	20.89883,	3	NA	NA
GL176229.1	74	1331	875, 553, 317	9, 8, 9	9.72082, 7.07159, 11.64359	6.64182, 5.20073, 7.57973	6.97155, 4	3	NA	NA
GL176235.1	10204	10896	10600, 10394, 10568	10, 7, 8	11.06011, 5.97725, 10.1465	7.306, 4.62287, 6.82176	8.22712, 3	3	NA	NA
GL176250.1	10092	10817	10335, 10354, 10600	11, 7, 4	12.43693, 5.97725, 4.67325	7.97018, 4.62287, 3.78987	9.52692, 3	3	NA	5249
GL176254.1	2806	3724	3291, 3304, 3305	16, 25, 16	12.07202, 19.55909, 15.26551	6.74019, 8.63629, 8.68554	9.18858, 1	3	NA	NA
GL176254.1	9932	10654	10297, 10409, 10270	9, 10, 8	6.69985, 6.21268, 7.59222	4.83936, 4.37127, 5.5104	4.20005, 3	3	NA	NA
GL176257.1	82	644	276, 218, 204	17, 18, 10	16.4953, 18.29879, 11.997	9.16875, 9.984, 7.78835	13.39926,	3	NA	NA
GL176257.1	2263	3157	2517, 2891, 2651	14, 15, 12	12.75226, 14.27489, 14.76955	7.64062, 8.33298, 9.10943	9.82689, 1	3	NA	NA
GL176257.1	9972	10691	10419, 10582, 10342	12, 16, 14	13.05874, 17.16856, 17.61756	8.20296, 9.82359, 10.40352	10.11961,	3	NA	NA
GL176264.1	10356	10716	10545, 10555, 10683	6, 9, 6	5.96715, 8.21099, 7.29403	4.64927, 5.77858, 5.30581	3.52158, 5	3	NA	NA
GL176268.1	10217	10482	10293, 10323, 10296	8, 16, 8	6.31005, 11.76475, 7.30639	4.7294, 6.55585, 5.34182	3.84065, 9	3	NA	NA
GL176271.1	8979	10619	9510, 9489, 9162	13, 16, 9	14.97989, 17.16856, 11.64359	9.13126, 9.82359, 7.57973	11.94968,	3	NA	NA
GL176272.1	9794	10603	10186, 10317, 10382	10, 13, 6	10.39648, 13.14514, 7.29403	6.94096, 8.09002, 5.30581	7.61003, 1	3	NA	NA
GL176277.1	31	788	91, 394, 240	8, 20, 6	8.42257, 22.87459, 7.12188	5.97763, 12.13503, 5.22884	5.76336, 1	3	NA	NA
GL176281.1	9192	10041	9478, 9693, 9529	11, 25, 12	12.09119, 28.99223, 15.16114	7.78314, 14.16979, 9.30141	9.20684, 2	3	NA	NA
GL176283.1	6046	7338	6564, 6844, 7001	10, 21, 6	9.95227, 23.31142, 6.5517	6.68696, 12.1022, 4.95623	7.19087, 2	3	arhgef3.1	3524
GL176292.1	9510	10138	9606, 9554, 9513	5, 11, 4	4.82177, 10.53778, 4.67325	3.98509, 6.89189, 3.78987	2.51418, 7	3	NA	5169
GL176294.1	120	659	319, 333, 333	12, 11, 7	13.55951, 10.59463, 8.69489	8.47903, 6.92577, 6.06379	10.59689,	3	NA	NA
GL176296.1	8081	8728	8242, 8278, 8623	7, 6, 7	7.1696, 4.93312, 8.69489	5.31345, 4.04501, 6.06379	4.61046, 2	3	NA	NA
GL176303.1	51	650	259, 411, 288	29, 43, 13	21.23798, 29.31948, 8.016	8.4204, 8.67471, 4.95367	17.96904,	3	NA	NA
GL176303.1	2828	3546	3016, 3229, 2941	20, 36, 18	12.18495, 22.29446, 12.86746	5.89428, 7.29464, 6.72284	9.29525, 1	3	NA	NA
GL176303.1	8403	9102	8549, 8580, 9054	16, 30, 11	11.18432, 18.7799, 8.60351	6.21076, 7.00604, 5.70193	8.34523, 1	3	NA	NA
GL176303.1	9437	10345	9590, 9564, 10032	17, 40, 10	12.41673, 29.38421, 7.56422	6.70245, 9.41101, 5.22677	9.51585, 2	3	NA	NA
GL176303.1	9493	10504	10231, 9564, 10032, 10368	12, 40, 10, 7	7.52338, 29.38421, 7.56422, 4.68368	4.84066, 9.41101, 5.22677, 3.80129	4.9421, 26	3	NA	NA
GL176306.1	897	2131	1195, 1666, 1885	6, 12, 3	5.96715, 11.86108, 3.47371	4.64927, 7.51216, 3.03189	3.52158, 9	3	NA	NA
GL176318.1	28	434	363, 389, 176	13, 7, 6	14.61367, 5.97725, 6.76586	8.93088, 4.62287, 5.06179	11.59864,	3	NA	NA
GL176322.1	76	1078	457, 734, 245	7, 9, 6	7.1696, 8.21099, 7.29403	5.31345, 5.77858, 5.30581	4.61046, 5	3	NA	NA
GL176322.1	8827	10385	10027, 9787, 9653	16, 30, 16	19.7934, 34.4899, 19.65507	11.29109, 15.5917, 11.21774	16.57067,	3	NA	NA
GL176323.1	2991	4658	3610, 3453, 3718, 4601	10, 15, 6, 6	8.66311, 11.1534, 5.43047, 5.23492	5.914, 6.42345, 4.34463, 4.22867	5.99101, 8	3	NA	NA
GL176323.1	7605	9070	8702, 8098, 8704	9, 14, 6	7.59348, 9.69817, 5.8719	5.40134, 5.76563, 4.59674	5.0069, 7.1	3	NA	NA
GL176328.1	7971	8540	8026, 8023, 8014	10, 25, 8	11.06011, 30.45643, 10.1465	7.306, 15.02432, 6.82176	8.22712, 2	3	NA	NA
GL176335.1	443	895	724, 824, 823	8, 9, 9	8.34715, 8.21099, 11.64359	5.9367, 5.77858, 7.57973	5.71011, 5	3	NA	NA
GL176338.1	6057	6554	6360, 6534, 6501	10, 8, 14	9.71769, 6.70176, 17.01463	6.54998, 4.97421, 10.0942	6.97155, 4	3	NA	NA
GL176342.1	5936	6356	5994, 5979, 5973	7, 8, 13	7.1696, 7.07159, 16.49347	5.31345, 5.20073, 9.91245	4.61046, 4	3	NA	NA
GL176348.1	24	673	178, 205, 246	14, 11, 8	16.6367, 10.60895, 9.25066	9.89449, 6.9343, 6.40569	13.53421,	3	NA	NA
GL176352.1	100	524	409, 238, 497	8, 10, 19	8.42257, 9.39128, 25.38515	5.97763, 6.35644, 13.7311	5.76336, 6	3	prss36	330
GL176358.1	233	1771	472, 1574, 1542	5, 9, 8	4.82177, 8.21099, 10.1465	3.98509, 5.77858, 6.82176	2.51418, 5	3	NA	NA
GL176358.1	414	1772	1558, 1574, 1542	13, 9, 8	15.29169, 8.21099, 10.1465	9.29854, 5.77858, 6.82176	12.24396,	3	NA	NA
GL176365.1	76	1413	525, 755, 196	7, 11, 4	7.1696, 10.60895, 4.67325	5.31345, 6.9343, 3.78987	4.61046, 7	3	NA	NA
GL176370.1	531	3322	780, 790, 1013	11, 16, 21	10.43535, 13.5443, 23.07125	6.83063, 7.63435, 11.96005	7.6474, 10	3	NA	NA
GL176370.1	531	3322	2318, 2420, 1013	10, 17, 21	9.23294, 14.71246, 23.07125	6.26141, 8.08343, 11.96005	6.52626, 1	3	NA	NA
GL176371.1	7808	8157	8060, 8090, 8016	12, 14, 8	13.8483, 14.45897, 10.1465	8.63436, 8.66788, 6.82176	10.86694,	3	NA	NA
GL176376.1	7706	8328	8302, 8296, 8223	15, 16, 16	18.26603, 17.16856, 23.11042	10.62691, 9.82359, 12.88555	15.09729,	3	NA	NA
GL176378.1	1716	2175	1936, 1959, 1958	8, 12, 7	8.42257, 11.86108, 8.69489	5.97763, 7.51216, 6.06379	5.76336, 9	3	NA	NA
GL176387.1	3198	3780	3514, 3571, 3546	13, 16, 12	15.29169, 16.67093, 14.58408	9.29854, 9.53036, 9.01638	12.24396,	3	NA	NA
GL176391.1	6550	6905	6799, 6799, 6799	23, 21, 19	28.23409, 21.73308, 25.11547	14.36443, 11.16072, 13.59366	24.76954,	3	NA	NA
GL176398.1	6315	7292	6397, 6359, 6359	47, 59, 55	55.80047, 72.44267, 74.8949	20.65061, 24.25383, 27.77522	51.86697,	3	NA	NA
GL176400.1	6923	7922	7548, 7094, 7823	8, 5, 8	7.57423, 3.41392, 8.24665	5.4999, 3.12487, 5.88165	4.98865, 1	3	NA	NA
GL176418.1	8547	9012	8760, 8819, 8831	5, 10, 7	4.82177, 9.39128, 7.61369	3.98509, 6.35644, 5.54854	2.51418, 6	3	NA	NA
GL176425.1	46	1227	538, 591, 435	36, 40, 23	37.03078, 40.17038, 26.23526	14.66982, 14.8363, 13.20592	33.3789, 3	3	slc52a3	5913
GL176434.1	5077	5571	5104, 5220, 5240	8, 11, 4	8.42257, 10.60895, 4.67325	5.97763, 6.9343, 3.78987	5.76336, 7	3	NA	NA
GL176436.1	528	988	769, 578, 697	4, 10, 4	3.74195, 9.39128, 4.67325	3.32091, 6.35644, 3.78987	1.6222, 6.8	3	NA	NA
GL176436.1	6412	7674	6849, 6801, 6476	5, 12, 4	4.82177, 11.37069, 4.67325	3.98509, 7.21896, 3.78987	2.51418, 8	3	NA	NA
GL176440.1	6321	7026	6403, 6977, 6395	39, 52, 23	34.5052, 47.43667, 21.19111	12.16701, 14.09482, 10.19146	30.9016, 4	3	NA	NA
GL176440.1	9052	10070	9580, 9588, 9656	20, 25, 21	13.1075, 16.29309, 18.71085	6.38768, 6.91444, 9.34217	10.16548,	3	NA	NA
GL176455.1	9657	10049	9901, 9900, 9799	7, 9, 8	7.1696, 8.21099, 10.1465	5.31345, 5.77858, 6.82176	4.61046, 5	3	NA	NA
GL176460.1	3235	3973	3906, 3906, 3896	31, 17, 18	36.71331, 14.12865, 21.20939	16.51706, 7.72861, 11.68575	33.06685,	3	NA	NA
GL176474.1	1016	1330	1290, 1288, 1296	27, 32, 24	37.35484, 41.74651, 34.60589	18.26252, 19.06933, 17.51814	33.69625,	3	NA	NA
GL176477.1	231	823	342, 423, 384	8, 8, 7	8.22568, 6.77831, 8.35502	5.8701, 5.0215, 5.9108	5.59681, 4	3	NA	NA
GL176477.1	4390	5008	4592, 4718, 4811	6, 12, 5	5.09949, 9.42893, 4.80993	4.14694, 6.02236, 3.97854	2.76939, 6	3	NA	NA
GL176479.1	2880	6153	3686, 4247, 4238, 5663	13, 22, 13, 11	9.28694, 16.11782, 12.05005, 9.00286	5.72647, 7.55429, 7.43242, 5.95183	6.57667, 1	3	NA	NA
GL176479.1	2880	6153	5726, 4247, 4238, 5663	23, 22, 13, 11	20.55909, 16.11782, 12.05005, 9.00286	9.81681, 7.55429, 7.43242, 5.95183	17.31147,	3	NA	NA
GL176482.1	9127	9822	9566, 9226, 9545	11, 12, 6	8.50933, 7.94095, 5.46454	5.64284, 5.09702, 4.36458	5.845, 5.47	3	NA	NA
GL176483.1	1065	1816	1357, 1127, 1566	5, 5, 5	4.82177, 3.94561, 5.06966	3.98509, 3.46715, 4.11933	2.51418, 1	3	NA	733
GL176487.1	7223	7743	7610, 7691, 7695	5, 14, 8	4.82177, 14.45897, 10.1465	3.98509, 8.66788, 6.82176	2.51418, 1	3	NA	NA
GL176496.1	157	1339	667, 754, 853	11, 21, 7	10.5483, 20.39593, 7.29952	6.89817, 10.35516, 5.38357	7.75329, 1	3	c1qb	2086
GL176496.1	5384	5818	5657, 5529, 5699	6, 6, 7	4.37941, 3.22653, 7.05993	3.69512, 2.93053, 5.25341	2.19155, 1	3	c1qb	0
GL176498.1	149	817	395, 380, 334	6, 9, 5	5.96715, 8.21099, 5.9506	4.64927, 5.77858, 4.54784	3.52158, 5	3	NA	NA
GL176499.1	2203	2855	2516, 2515, 2266	12, 33, 5	13.8483, 38.22158, 5.9506	8.63436, 16.6026, 4.54784	10.86694,	3	NA	NA
GL176502.1	62	2272	1242, 1206, 582	32, 64, 27	25.07594, 55.70713, 25.27885	9.53874, 13.88791, 11.25646	21.69379,	3	NA	NA
GL176502.1	9182	9967	9397, 9427, 9386	26, 31, 18	27.63895, 33.66599, 21.89732	13.01211, 14.70817, 12.06835	24.18761,	3	NA	NA
GL176503.1	8185	8726	8375, 8375, 8371	12, 23, 14	13.8483, 25.23193, 18.28596	8.63436, 12.61137, 10.7324	10.86694,	3	NA	NA
GL176504.1	4495	5105	4843, 4873, 4726	12, 8, 5	13.73771, 7.07159, 5.9506	8.57523, 5.20073, 4.54784	10.76705,	3	slc25a34	1817
GL176509.1	76	835	390, 378, 529	17, 19, 5	20.94161, 21.4153, 5.9506	11.74019, 11.55717, 4.54784	17.68164,	3	NA	NA
GL176514.1	56	445	110, 131, 135	13, 20, 8	15.29169, 22.87459, 10.1465	9.29854, 12.13503, 6.82176	12.24396,	3	NA	NA
GL176516.1	28	986	500, 502, 490	59, 81, 39	53.25097, 69.25912, 37.69656	14.40656, 14.27268, 13.86435	49.35215,	3	NA	4450
GL176517.1	6675	7464	7088, 7074, 6988	12, 16, 5	7.20548, 10.61968, 4.12265	4.6472, 5.87975, 3.57684	4.64326, 7	3	NA	NA
GL176518.1	9250	9837	9517, 9614, 9640	15, 20, 7	16.54256, 22.87459, 8.69489	9.6759, 12.13503, 6.06379	13.44484,	3	NA	NA
GL176526.1	728	1234	1079, 1073, 1020	14, 19, 18	7.0981, 8.38781, 13.50079	4.25125, 4.18186, 7.09242	4.5696, 5.8	3	NA	NA
GL176526.1	1546	2356	1630, 2158, 2023	12, 21, 9	5.57022, 9.65739, 4.90373	3.68442, 4.48812, 3.69226	3.19636, 7	3	NA	NA
GL176528.1	3722	4352	4075, 3770, 3849	10, 15, 10	9.77513, 14.21432, 11.5283	6.5837, 8.29617, 7.55211	7.02302, 1	3	NA	NA
GL176531.1	3349	4288	3748, 3855, 3684	10, 22, 11	6.98236, 13.75132, 7.95476	4.85825, 6.28756, 5.29439	4.46255, 1	3	NA	NA
GL176531.1	9434	9803	9543, 9586, 9674	7, 16, 10	5.30682, 11.18144, 8.39789	4.20391, 6.20906, 5.74985	2.95991, 8	3	NA	NA
GL176537.1	2927	3396	3367, 3369, 3370	104, 132, 86	131.95967, 166.9969, 116.8727	32.27613, 34.58641, 34.36506	127.33482	3	NA	NA
GL176538.1	9504	9845	9649, 9666, 9631	10, 14, 6	11.06011, 14.45897, 7.29403	7.306, 8.66788, 5.30581	8.22712, 1	3	mapre3	78
GL176540.1	78	802	364, 285, 310	7, 11, 5	6.99564, 9.22091, 5.69847	5.21786, 6.08767, 4.4331	4.47307, 6	3	NA	NA
GL176544.1	400	1747	1028, 1028, 1031	23, 22, 11	25.13729, 22.22711, 12.78973	12.55499, 11.1385, 8.15619	21.75229,	3	NA	NA
GL176550.1	6	607	466, 464, 455	68, 100, 47	79.15158, 114.82114, 59.22097	23.44084, 26.03416, 22.64924	74.96326,	3	NA	NA
GL176555.1	1960	2243	2083, 2035, 2208	7, 15, 7	7.1696, 15.80067, 8.69489	5.31345, 9.24574, 6.06379	4.61046, 1	3	NA	NA
GL176556.1	82	2950	1019, 278, 2900, 295, 2326, 2918	34, 42, 20, 25, 9, 13	27.47105, 32.39912, 9.74394, 22.12874, 5.20287, 8.25928	10.16739, 10.23018, 4.67404, 10.09386, 3.88225, 5.09983	24.0232, 2	3	NA	NA
GL176556.1	7541	8213	7612, 7681, 7807	9, 11, 7	8.11416, 8.48203, 7.15355	5.72019, 5.62571, 5.30471	5.49285, 5	3	NA	NA
GL176560.1	70	723	175, 557, 580	19, 18, 18	13.9973, 11.54821, 16.41422	7.12763, 5.97308, 8.84243	11.00892,	3	NA	NA
GL176560.1	2765	3462	3269, 3057, 3325	18, 17, 9	12.95096, 10.60534, 6.40904	6.77125, 5.65871, 4.65391	10.01686,	3	NA	NA
GL176560.1	8505	9780	9001, 8952, 8708, 9032	17, 17, 11, 14	16.36399, 12.93592, 8.79866, 12.16551	9.08912, 7.01058, 5.8242, 7.28026	13.27324,	3	NA	NA
GL176561.1	2355	3237	2775, 2793, 2507	11, 9, 8	11.49566, 7.63127, 10.1465	7.45067, 5.42473, 6.82176	8.64106, 5	3	NA	NA
GL176565.1	25	2231	1070, 1392, 1080	200, 299, 148	162.09402, 234.27263, 135.0542	14.88417, 14.44757, 17.94153	157.30238	3	NA	NA
GL176565.1	9061	9725	9339, 9270, 9482	11, 10, 12	12.01255, 9.39128, 13.22602	7.73996, 6.35644, 8.2961	9.13199, 6	3	NA	NA
GL176570.1	7710	8521	7781, 8395, 8313	7, 12, 3	7.1696, 11.86108, 3.47371	5.31345, 7.51216, 3.03189	4.61046, 9	3	NA	NA
GL176577.1	8687	9544	9076, 8953, 9321	7, 12, 4	7.1696, 11.86108, 4.67325	5.31345, 7.51216, 3.78987	4.61046, 9	3	NA	NA
GL176582.1	148	2091	876, 709, 677	15, 15, 17	10.77666, 9.83268, 16.65618	6.19582, 5.63348, 9.26618	7.97074, 7	3	NA	NA
GL176582.1	4191	5630	4742, 4974, 4689	26, 24, 14	20.96151, 16.81669, 10.8526	9.11922, 7.42201, 6.47083	17.70112,	3	NA	NA
GL176582.1	8661	9485	9235, 8958, 8950	12, 13, 12	9.49573, 8.66599, 11.60888	6.06403, 5.34624, 7.36202	6.77365, 6	3	NA	NA
GL176592.1	186	1128	268, 403, 217, 731	4, 5, 5, 7	3.74195, 3.94561, 5.9506, 8.69489	3.32091, 3.46715, 4.54784, 6.06379	1.6222, 1.8	3	NA	NA
GL176595.1	9019	9581	9251, 9252, 9393	13, 12, 8	15.29169, 11.86108, 10.1465	9.29854, 7.51216, 6.82176	12.24396,	3	NA	NA
GL176604.1	9332	9595	9366, 9379, 9377	10, 24, 11	11.06011, 28.90463, 14.75772	7.306, 14.44646, 9.09568	8.22712, 2	3	NA	NA
GL176614.1	6086	6402	6188, 6187, 6302	10, 12, 7	11.06011, 11.86108, 8.69489	7.306, 7.51216, 6.06379	8.22712, 9	3	NA	NA
GL176632.1	674	1082	923, 958, 928	17, 21, 10	21.34551, 24.35402, 13.18184	11.95527, 12.71289, 8.33771	18.07159,	3	NA	NA
GL176645.1	8630	9548	8851, 8874, 9180	20, 29, 16	24.58631, 32.99296, 21.79539	13.10866, 15.08875, 12.29292	21.21573,	3	NA	NA
GL176649.1	37	1762	643, 659, 524, 1546	52, 56, 44, 10	49.65823, 53.09222, 45.38907, 5.51269	15.16897, 15.36748, 16.0902, 3.93316	45.81035,	3	NA	NA
GL176649.1	6715	7396	7036, 6816, 7043	14, 13, 13	10.82413, 8.38839, 12.98465	6.45331, 5.17779, 7.995	8.01418, 5	3	NA	NA
GL176649.1	8754	9504	9038, 9115, 9107	17, 23, 8	14.15399, 18.94772, 6.97106	7.74398, 8.87621, 5.13965	11.15907,	3	NA	NA
GL176665.1	8763	9263	9057, 8890, 8920	8, 7, 3	8.42257, 5.97725, 3.47371	5.97763, 4.62287, 3.03189	5.76336, 3	3	NA	NA
GL176667.1	7833	8562	8299, 8299, 8333	232, 324, 142	219.29973, 304.44449, 129.66919	20.39727, 20.7852, 17.85222	214.21928	3	NA	NA
GL176668.1	23	576	295, 281, 366	970, 1166, 924	1106.25879, 1352.70642, 1083.02942	34.97468, 36.84943, 37.49978	1099.3314	3	NA	NA
GL176669.1	26	282	165, 185, 188	14, 16, 7	14.38232, 15.54288, 8.08824	8.62271, 8.85379, 5.7849	11.37721,	3	NA	NA
GL176669.1	8685	9226	9090, 8741, 8781	6, 9, 7	5.96715, 8.21099, 8.69489	4.64927, 5.77858, 6.06379	3.52158, 5	3	NA	NA
GL176678.1	7924	9354	8358, 8840, 8303	13, 14, 6	9.55588, 10.15403, 4.80295	5.89229, 6.04265, 3.96416	6.82923, 7	3	NA	NA
GL176681.1	3226	4461	4118, 4101, 3947	11, 12, 5	12.01255, 10.35, 5.40789	7.73996, 6.59497, 4.29296	9.13199, 7	3	NA	NA
GL176695.1	52	2458	629, 650, 632	114, 129, 63	90.48234, 100.30979, 46.69617	13.38186, 13.15043, 10.73266	86.1818, 9	3	NA	NA
GL176695.1	8882	9411	9175, 9076, 9206	59, 57, 31	56.4845, 50.79722, 30.33233	15.88237, 13.96872, 12.75129	52.54191,	3	NA	NA
GL176707.1	7254	7781	7576, 7504, 7680	8, 15, 8	8.34715, 15.80067, 10.1465	5.9367, 9.24574, 6.82176	5.71011, 1	3	NA	NA
GL176707.1	8999	9366	9143, 9154, 9156	13, 8, 5	15.1723, 7.07159, 5.9506	9.23486, 5.20073, 4.54784	12.13305,	3	NA	NA
GL176708.1	7852	9344	8554, 8205, 8584	9, 12, 7	9.72082, 11.86108, 8.69489	6.64182, 7.51216, 6.06379	6.97155, 9	3	NA	NA
GL176716.1	6091	6438	6205, 6131, 6201	14, 8, 4	16.7649, 7.07159, 4.67325	9.96273, 5.20073, 3.78987	13.6543, 4	3	NA	NA
GL176719.1	7387	8273	7641, 7860, 7749	12, 27, 6	11.55224, 28.43981, 7.12188	7.3281, 13.12665, 5.22884	8.69537, 2	3	bhlhe23	4862
GL176729.1	1485	4642	4044, 3907, 4154	71, 64, 40	56.02604, 47.30713, 30.73037	12.14626, 10.72527, 10.01774	52.08938,	3	NA	NA
GL176733.1	2183	2531	2454, 2445, 2505	8, 12, 6	8.42257, 11.86108, 7.29403	5.97763, 7.51216, 5.30581	5.76336, 9	3	NA	NA
GL176737.1	6308	7584	6553, 6441, 6370	5, 16, 6	4.82177, 17.16856, 7.29403	3.98509, 9.82359, 5.30581	2.51418, 1	3	NA	NA
GL176738.1	8694	9317	8969, 9020, 9121	6, 11, 4	5.96715, 10.60895, 4.67325	4.64927, 6.9343, 3.78987	3.52158, 7	3	NA	NA
GL176748.1	3052	3551	3283, 3118, 3285	13, 7, 10	12.81131, 5.97725, 11.10454	7.8918, 4.62287, 7.32977	9.88323, 3	3	NA	NA
GL176750.1	7971	8702	8219, 8326, 8058	8, 16, 9	7.52511, 13.2638, 10.01275	5.47115, 7.46304, 6.79683	4.94319, 1	3	NA	NA
GL176754.1	20	856	548, 151, 172	47, 38, 33	46.37293, 36.02841, 35.35057	15.40644, 13.3317, 14.90393	42.57386,	3	NA	3376
GL176754.1	7005	7190	7057, 7054, 7047	300, 307, 235	373.0466, 372.05057, 307.73135	39.5806, 37.21844, 43.20462	367.65582	3	NA	1689
GL176756.1	6968	7394	7122, 7130, 7161	5, 8, 6	4.82177, 7.07159, 7.29403	3.98509, 5.20073, 5.30581	2.51418, 4	3	stmn3	5347
GL176759.1	309	1075	629, 711, 589	12, 20, 7	10.1196, 17.88727, 7.84208	6.45229, 9.1506, 5.66426	7.349, 14.9	3	NA	NA
GL176759.1	4263	5126	4608, 4874, 4833	16, 22, 11	10.09971, 13.47147, 8.35618	5.57966, 6.14446, 5.54667	7.33101, 1	3	NA	NA
GL176759.1	7349	9175	8281, 8313, 8907	17, 25, 13	12.63865, 18.63084, 11.25379	6.83375, 8.13015, 6.94381	9.71961, 1	3	NA	NA
GL176784.1	98	433	285, 278, 271	17, 16, 10	21.34551, 17.16856, 13.18184	11.95527, 9.82359, 8.33771	18.07159,	3	NA	NA
GL176790.1	6458	6912	6750, 6713, 6807	7, 10, 6	7.10297, 9.39128, 7.12188	5.27706, 6.35644, 5.22884	4.57191, 6	3	NA	NA
GL176790.1	7931	8352	8050, 8189, 8083	7, 7, 4	7.10297, 5.97725, 4.55201	5.27706, 4.62287, 3.73489	4.57191, 3	3	NA	NA
GL176797.1	333	2029	944, 1099, 1096	11, 17, 16	11.42621, 16.43386, 18.43661	7.41109, 9.1315, 10.55125	8.57514, 1	3	NA	NA
GL176797.1	5751	8993	6012, 7789, 6813	13, 16, 10	10.98084, 13.17297, 10.7802	6.7751, 7.40763, 7.15425	8.16259, 1	3	NA	NA
GL176805.1	89	752	461, 405, 380	17, 21, 9	20.46701, 23.50551, 9.8849	11.48256, 12.21671, 6.72947	17.22179,	3	NA	NA
GL176805.1	7825	8505	8034, 8051, 7933	10, 13, 7	10.39648, 13.14514, 7.20145	6.94096, 8.09002, 5.33074	7.61003, 1	3	NA	NA
GL176805.1	8547	9060	8688, 8825, 8783	15, 5, 8	17.28737, 3.94561, 8.4586	10.09595, 3.46715, 5.99709	14.15669,	3	NA	NA
GL176807.1	8771	9039	8912, 8902, 8913	19, 12, 13	24.51879, 11.86108, 17.16464	13.28363, 7.51216, 10.23255	21.15031,	3	NA	NA
GL176813.1	21	588	289, 128, 127	14, 18, 12	16.7649, 19.97713, 16.36823	9.96273, 10.97931, 9.85365	13.6543, 1	3	NA	NA
GL176822.1	6970	8471	7892, 7082, 8263, 7121	8, 7, 14, 12	8.42257, 5.96795, 14.45897, 14.3175	5.97763, 4.61718, 8.66788, 8.88032	5.76336, 3	3	NA	NA
GL176826.1	7337	7925	7435, 7425, 7584	5, 10, 4	4.82177, 9.39128, 4.67325	3.98509, 6.35644, 3.78987	2.51418, 6	3	NA	NA
GL176827.1	1518	2167	1687, 1686, 2018	61, 93, 42	66.86806, 104.33247, 49.99311	20.23512, 24.3367, 19.60862	62.80425,	3	NA	NA
GL176833.1	1182	1879	1316, 1328, 1412	28, 45, 28	37.24342, 59.43621, 41.41987	17.91014, 23.74658, 20.11347	33.58719,	3	NA	NA
GL176837.1	6824	9012	8539, 8569, 8497	18, 38, 9	20.06618, 39.71725, 11.21577	11.03116, 15.3939, 7.38849	16.83343,	3	NA	NA
GL176846.1	80	1136	183, 422, 926	13, 16, 9	11.30985, 13.5443, 9.35579	6.9784, 7.63435, 6.44216	8.46461, 1	3	NA	NA
GL176846.1	7714	8144	7946, 7877, 7782	9, 15, 9	7.55321, 11.96891, 8.43009	5.37637, 6.92051, 5.90952	4.96981, 9	3	NA	NA
GL176848.1	6772	7255	7100, 6938, 7001	6, 10, 7	5.63898, 8.16488, 8.646	4.46544, 5.60458, 6.04229	3.25772, 5	3	NA	NA
GL176851.1	8345	9014	8672, 8745, 8489	9, 21, 8	9.72082, 23.50551, 8.86315	6.64182, 12.21671, 6.21039	6.97155, 2	3	NA	NA
GL176867.1	307	672	351, 350, 377	12, 25, 7	13.8483, 30.45643, 8.69489	8.63436, 15.02432, 6.06379	10.86694,	3	NA	NA
GL176868.1	1796	2531	2353, 2220, 2150	10, 11, 7	8.66311, 9.39347, 6.75301	5.914, 6.19476, 5.08141	5.99101, 6	3	NA	NA
GL176868.1	8287	8860	8351, 8590, 8723	11, 14, 7	12.43693, 14.45897, 8.69489	7.97018, 8.66788, 6.06379	9.52692, 1	3	NA	NA
GL176872.1	112	933	523, 138, 509	9, 13, 11	9.72082, 13.14514, 14.75772	6.64182, 8.09002, 9.09568	6.97155, 1	3	NA	NA
GL176876.1	73	1041	415, 304, 577	18, 13, 11	22.49411, 13.14514, 13.64572	12.39242, 8.09002, 8.58592	19.18565,	3	NA	NA
GL176878.1	7637	7970	7941, 7919, 7934	15, 16, 12	18.26603, 17.16856, 16.36823	10.62691, 9.82359, 9.85365	15.09729,	3	NA	NA
GL176885.1	6842	7718	7394, 7162, 7311	7, 12, 8	7.1696, 11.86108, 10.1465	5.31345, 7.51216, 6.82176	4.61046, 9	3	NA	NA
GL176891.1	547	1091	839, 717, 744	7, 19, 8	7.1696, 19.48976, 10.1465	5.31345, 10.41622, 6.82176	4.61046, 1	3	dera	5617
GL176892.1	66	709	470, 248, 237	7, 14, 9	7.1696, 14.45897, 11.64359	5.31345, 8.66788, 7.57973	4.61046, 1	3	NA	NA
GL176896.1	34	1060	321, 309, 89	53, 89, 23	27.82491, 49.25295, 12.56334	6.49825, 7.51702, 5.50733	24.36923,	3	NA	NA
GL176898.1	200	963	509, 588, 524	14, 15, 13	15.84929, 15.80067, 18.01082	9.46495, 9.24574, 10.61163	12.77923,	3	NA	6158
GL176903.1	8091	8803	8445, 8448, 8230	10, 9, 4	11.06011, 8.21099, 4.67325	7.306, 5.77858, 3.78987	8.22712, 5	3	NA	NA
GL176904.1	3794	4777	4158, 4039, 4646	19, 15, 11	17.50859, 12.40077, 10.5698	9.21834, 7.18527, 6.91098	14.36919,	3	NA	NA
GL176905.1	8320	8828	8352, 8354, 8357	36, 52, 38	48.29868, 70.71751, 54.7079	21.26935, 26.8872, 24.09568	44.47071,	3	NA	NA
GL176906.1	51	797	528, 471, 402	10, 7, 9	11.06011, 5.97725, 11.39481	7.306, 4.62287, 7.46977	8.22712, 3	3	NA	NA
GL176906.1	2006	2607	2142, 2285, 2343	8, 11, 6	8.42257, 10.60895, 7.12188	5.97763, 6.9343, 5.22884	5.76336, 7	3	NA	NA
GL176907.1	1222	1746	1283, 1283, 1273	23, 36, 16	29.0469, 48.0849, 22.07892	14.82219, 21.14652, 12.42524	25.56226,	3	NA	NA
GL176918.1	27	937	882, 262, 623	11, 9, 9	12.43693, 8.21099, 11.64359	7.97018, 5.77858, 7.57973	9.52692, 5	3	NA	NA
GL176918.1	7945	8755	8229, 8243, 8178	9, 13, 11	9.72082, 13.14514, 14.75772	6.64182, 8.09002, 9.09568	6.97155, 1	3	NA	NA
GL176922.1	24	464	250, 249, 259	29, 29, 20	34.62408, 35.12553, 27.04832	16.05487, 16.34976, 14.41766	31.018, 31	3	NA	NA
GL176928.1	6631	7459	7137, 7059, 6785	10, 7, 5	11.06011, 5.97725, 5.9506	7.306, 4.62287, 4.54784	8.22712, 3	3	NA	NA
GL176931.1	6781	7720	7298, 7263, 7335	12, 21, 11	13.05874, 24.1145, 12.19945	8.20296, 12.57361, 7.8422	10.11961,	3	NA	NA
GL176934.1	2367	2774	2391, 2457, 2401	11, 10, 6	11.42621, 9.39128, 6.99807	7.41109, 6.35644, 5.17195	8.57514, 6	3	NA	NA
GL176935.1	5886	6494	6055, 6217, 6055	13, 18, 6	11.87199, 15.38496, 5.39683	7.32374, 8.2181, 4.32487	8.9979, 12	3	NA	NA
GL176944.1	84	1015	372, 188, 409	31, 44, 22	15.24339, 24.02953, 11.74424	5.36719, 6.56671, 5.29575	12.20169,	3	NA	NA
GL176944.1	2907	4495	3783, 3771, 3772	83, 93, 63	38.74158, 42.7903, 34.11286	6.03948, 6.015, 6.95416	35.0593, 3	3	NA	NA
GL176944.1	5377	8587	6936, 6937, 6932	134, 127, 102	92.32113, 78.53402, 77.27361	10.824, 9.13147, 12.16523	87.99968,	3	NA	NA
GL176949.1	374	1645	773, 1289, 1539	10, 12, 6	9.77513, 10.35, 6.2131	6.5837, 6.59497, 4.78171	7.02302, 7	3	NA	NA
GL176949.1	1773	2372	1842, 1986, 1951	10, 15, 6	9.44155, 13.92002, 6.168	6.38646, 8.11689, 4.75778	6.72156, 1	3	NA	NA
GL176968.1	8	1466	631, 700, 680	24, 37, 11	25.99978, 37.6226, 13.28509	12.73437, 14.60626, 8.40871	22.5903, 3	3	NA	NA
GL176968.1	5042	6622	6202, 5710, 6007	17, 25, 7	15.00845, 20.10572, 6.51247	8.26375, 8.93987, 4.9427	11.97619,	3	NA	NA
GL176968.1	7971	8702	8558, 8369, 8504	11, 9, 8	9.26086, 6.10084, 8.35103	6.1125, 4.45669, 5.93881	6.5517, 3.7	3	NA	NA
GL176973.1	7789	8501	7881, 8118, 8473	6, 8, 4	5.90927, 7.07159, 4.67325	4.61743, 5.20073, 3.78987	3.50164, 4	3	NA	NA
GL176975.1	98	1057	722, 627, 621	14, 20, 9	16.7649, 22.87459, 11.64359	9.96273, 12.13503, 7.57973	13.6543, 1	3	NA	NA
GL176975.1	7762	8642	7922, 8096, 8149	8, 19, 13	8.42257, 21.4153, 18.01082	5.97763, 11.55717, 10.61163	5.76336, 1	3	NA	NA
GL176991.1	28	685	304, 306, 377	27, 33, 31	29.93154, 34.47113, 35.62479	14.01689, 14.38797, 15.87156	26.42561,	3	NA	NA
GL176991.1	7022	7944	7507, 7160, 7370	20, 28, 19	11.13127, 13.41737, 12.67738	5.35172, 5.08536, 6.37865	8.2952, 10	3	NA	NA
GL176991.1	8302	8693	8504, 8439, 8502	59, 115, 49	55.21032, 114.56343, 48.91734	15.29063, 20.34144, 15.94661	51.2844, 1	3	NA	NA
GL177000.1	190	743	409, 295, 446	8, 9, 4	8.42257, 8.21099, 4.67325	5.97763, 5.77858, 3.78987	5.76336, 5	3	NA	NA
GL177002.1	7486	7930	7866, 7898, 7904	25, 46, 30	34.51774, 63.45107, 45.09827	17.26872, 25.61462, 21.50061	30.91362,	3	NA	NA
GL177004.1	19	869	427, 642, 405	17, 13, 10	18.46847, 13.14514, 12.52085	10.34725, 8.09002, 8.03986	15.29249,	3	NA	NA
GL177008.1	74	323	165, 227, 275	11, 14, 6	11.29029, 13.63145, 6.87953	7.3332, 8.17488, 5.11627	8.44541, 1	3	NA	NA
GL177008.1	2134	2971	2415, 2631, 2348	12, 16, 14	12.60209, 16.22539, 18.77534	7.9443, 9.26486, 10.96344	9.68421, 1	3	NA	NA
GL177008.1	8120	8705	8395, 8250, 8548	12, 8, 11	13.8483, 7.07159, 14.75772	8.63436, 5.20073, 9.09568	10.86694,	3	NA	NA
GL177009.1	84	790	387, 474, 298	10, 11, 8	11.06011, 10.60895, 10.1465	7.306, 6.9343, 6.82176	8.22712, 7	3	NA	4995
GL177016.1	5378	5877	5482, 5630, 5451	25, 16, 9	25.0899, 13.03911, 8.43009	11.84301, 7.32604, 5.90952	21.70687,	3	NA	3963
GL177021.1	929	1165	1017, 957, 976	9, 11, 5	9.72082, 10.60895, 5.9506	6.64182, 6.9343, 4.54784	6.97155, 7	3	NA	NA

GL177023.1	1896	2502	2447, 2476, 2448	27, 29, 26	37.35484, 36.82147, 37.80034	18.26252, 17.33575, 18.72634	33.69625,	3	NA	NA	
GL177026.1	51	676	252, 246, 211	11, 11, 8	12.17114, 10.60895, 10.1465	7.82679, 6.9343, 6.82176	9.28263, 7	3	NA	NA	
GL177026.1	8226	8673	8499, 8458, 8512	13, 17, 9	15.29169, 18.56115, 11.64359	9.29854, 10.40145, 7.57973	12.24396,	3	NA	NA	
GL177032.1	134	940	458, 682, 429	6, 12, 8	5.96715, 11.86108, 10.1465	4.64927, 7.51216, 6.82176	3.52158, 9	3	NA	NA	
GL177036.1	136	813	752, 197, 440	18, 16, 8	22.92104, 17.16856, 10.1465	12.61945, 9.82359, 6.82176	19.59912,	3	NA	NA	
GL177036.1	7482	8652	8368, 8178, 8068	18, 17, 9	20.86369, 18.56115, 11.64359	11.49013, 10.40145, 7.57973	17.60698,	3	NA	NA	
GL177037.1	45	794	572, 274, 136	9, 7, 6	9.72082, 5.97725, 7.29403	6.64182, 4.62287, 5.30581	6.97155, 3	3	NA	NA	
GL177038.1	8	223	132, 149, 147	37, 39, 11	43.97989, 45.35557, 14.45778	18.31334, 18.24235, 8.96373	40.21576,	3	NA	NA	
GL177062.1	263	701	585, 607, 618	19, 17, 15	24.51879, 18.56115, 20.414	13.28363, 10.40145, 11.69434	21.15031,	3	NA	NA	
GL177066.1	474	1053	969, 1011, 862	16, 23, 6	19.7934, 27.36982, 7.29403	11.29109, 13.8686, 5.30581	16.57067,	3	NA	NA	
GL177069.1	8004	8623	8519, 8071, 8195	7, 13, 7	7.1696, 13.14514, 8.69489	5.31345, 8.09002, 6.06379	4.61046, 1	3	NA	NA	
GL177073.1	7882	8445	8236, 8234, 8237	40, 26, 23	52.22935, 31.2219, 32.49096	21.84114, 15.13645, 16.64563	48.34681,	3	U2		0
GL177077.1	815	1092	945, 919, 930	5, 14, 9	4.82177, 14.45897, 11.58206	3.98509, 8.66788, 7.55286	2.51418, 1	3	NA	NA	
GL177085.1	103	1092	939, 675, 601	7, 19, 12	7.1696, 21.4153, 16.36823	5.31345, 11.55717, 9.85365	4.61046, 1	3	U5		0
GL177087.1	8064	8472	8311, 8284, 8364	5, 9, 8	4.82177, 8.21099, 10.1465	3.98509, 5.77858, 6.82176	2.51418, 5	3	NA	NA	
GL177098.1	7059	8098	7950, 7888, 7173, 7879	26, 23, 8, 11	35.26919, 27.36982, 10.0913, 14.68355	17.41492, 13.8686, 6.79758, 9.06344	31.65079,	3	NA	NA	
GL177100.1	7978	8547	8140, 8182, 8295	6, 8, 6	5.96715, 7.07159, 7.29403	4.64927, 5.20073, 5.30581	3.52158, 4	3	NA	NA	
GL177118.1	156	681	191, 186, 185	9, 13, 9	9.72082, 13.14514, 11.64359	6.64182, 8.09002, 7.57973	6.97155, 1	3	NA	NA	
GL177119.1	176	562	536, 534, 356	7, 16, 3	7.1696, 17.16856, 3.47371	5.31345, 9.82359, 3.03189	4.61046, 1	3	NA	NA	
GL177122.1	7416	7952	7559, 7754, 7614	8, 12, 5	8.42257, 11.86108, 5.9506	5.97763, 7.51216, 4.54784	5.76336, 9	3	NA	NA	
GL177132.1	1184	2263	1598, 1496, 1707, 2185	15, 20, 15, 7	14.09249, 18.84531, 16.01939, 6.08239	8.22203, 9.72843, 9.37366, 4.68683	11.1007, 1	3	NA	NA	
GL177132.1	7676	8517	7992, 7991, 7991	115, 115, 68	117.18484, 109.58521, 75.42794	21.26584, 18.66812, 21.50137	112.65778	3	NA	NA	
GL177134.1	762	1587	1346, 1133, 1059	9, 14, 10	9.72082, 13.26509, 11.38247	6.64182, 7.95332, 7.47651	6.97155, 1	3	xtr-mir-427-2		0
GL177142.1	6666	7313	6743, 7017, 7114	6, 5, 11	5.81608, 3.94561, 12.48556	4.56563, 3.46715, 7.99611	3.4176, 1.8	3	NA	NA	
GL177146.1	117	718	412, 358, 349	22, 20, 6	28.30573, 22.4484, 7.29403	14.67216, 11.88633, 5.30581	24.83844,	3	NA	NA	
GL177146.1	5689	6336	6179, 5944, 5776	9, 8, 5	8.45482, 7.07159, 5.59755	5.92419, 5.20073, 4.38538	5.79307, 4	3	NA	NA	
GL177146.1	7705	8143	7894, 7991, 7980	10, 8, 5	9.661, 7.07159, 5.59755	6.51661, 5.20073, 4.38538	6.9283, 4.6	3	NA	NA	
GL177169.1	165	2173	1247, 1298, 1044	77, 54, 42	78.28041, 49.47479, 39.64183	19.12341, 14.36631, 13.77881	74.10052,	3	NA	NA	
GL177169.1	7752	8445	8032, 8081, 8208	15, 23, 22	18.26603, 27.36982, 31.08818	10.62691, 13.8686, 16.11669	15.09729,	3	NA	NA	
GL177170.1	1747	2046	1869, 1869, 1910	16, 19, 15	19.7934, 21.4153, 21.38372	11.29109, 11.55717, 12.12757	16.57067,	3	NA	NA	
GL177172.1	179	519	334, 257, 308	8, 10, 10	8.42257, 9.39128, 13.18184	5.97763, 6.35644, 8.33771	5.76336, 6	3	NA	NA	
GL177176.1	7570	8415	7611, 8211, 7695	7, 11, 8	7.1696, 10.26356, 9.92329	5.31345, 6.72731, 6.7228	4.61046, 7	3	atraid		150
GL177183.1	71	1158	861, 685, 925	8, 17, 11	8.42257, 18.56115, 14.75772	5.97763, 10.40145, 9.09568	5.76336, 1	3	NA	NA	
GL177183.1	7310	8394	7906, 7955, 7975	12, 24, 14	13.8483, 28.90463, 19.68329	8.63436, 14.44646, 11.3696	10.86694,	3	NA	NA	
GL177192.1	227	629	320, 592, 546	5, 6, 4	4.82177, 4.93312, 4.67325	3.98509, 4.04501, 3.78987	2.51418, 2	3	NA	NA	
GL177192.1	7563	8428	7881, 8238, 8243	7, 11, 8	7.1696, 10.60895, 10.1465	5.31345, 6.9343, 6.82176	4.61046, 7	3	NA	NA	
GL177205.1	7514	8257	7965, 8016, 7572	5, 6, 7	4.82177, 4.93312, 8.69489	3.98509, 4.04501, 6.06379	2.51418, 2	3	NA	NA	
GL177209.1	65	729	378, 409, 175	21, 20, 10	23.41865, 22.255, 11.10454	12.1655, 11.77279, 7.32977	20.08217,	3	NA	NA	
GL177209.1	7517	8391	7857, 7827, 7890	38, 46, 34	45.40854, 50.47916, 41.78159	18.7177, 18.06563, 18.29497	41.62352,	3	NA	NA	
GL177212.1	104	712	445, 220, 445	13, 11, 10	8.84874, 6.53768, 8.25787	5.4577, 4.41031, 5.66266	6.16541, 4	3	NA	NA	
GL177212.1	1905	3411	2469, 2500, 2731	26, 27, 24	23.43853, 23.60388, 25.82569	10.52556, 10.29073, 12.6303	20.10161,	3	NA	NA	
GL177212.1	7590	8390	8002, 8102, 7942	23, 22, 15	25.62302, 23.02459, 16.83199	12.8439, 11.61782, 9.84064	22.22439,	3	NA	NA	
GL177220.1	14	915	515, 512, 501	53, 59, 37	60.78531, 63.74032, 41.92189	20.63601, 19.48871, 17.09888	56.78883,	3	NA	NA	
GL177230.1	19	836	301, 167, 303	18, 30, 16	22.75759, 38.10887, 22.37427	12.53302, 17.71736, 12.56044	19.4414, 3	3	NA	NA	
GL177232.1	7008	7675	7620, 7609, 7553	20, 31, 8	26.13767, 40.09135, 10.1465	13.94781, 18.49147, 6.82176	22.72398,	3	NA	NA	
GL177241.1	1271	1735	1696, 1708, 1702	10, 14, 12	11.06011, 14.45897, 16.36823	7.306, 8.66788, 9.85365	8.22712, 1	3	NA	NA	
GL177242.1	17	754	293, 311, 304	194, 249, 110	216.13321, 272.35184, 125.39751	28.40436, 28.28778, 26.36231	211.06573	3	NA	NA	
GL177242.1	7710	8312	8168, 8119, 8175	78, 92, 45	88.31252, 99.04714, 53.33344	23.41728, 22.39812, 20.16414	84.03393,	3	NA	NA	
GL177244.1	7251	8322	7674, 7671, 7989	13, 20, 10	15.29169, 22.87459, 13.18184	9.29854, 12.13503, 8.33771	12.24396,	3	NA	NA	
GL177249.1	537	868	747, 826, 646	7, 8, 6	7.1696, 7.07159, 7.29403	5.31345, 5.20073, 5.30581	4.61046, 4	3	NA		2501
GL177252.1	14	612	260, 291, 374	37, 41, 21	38.69977, 40.16942, 22.00729	15.22201, 14.44133, 11.32533	35.01804,	3	NA	NA	
GL177252.1	7722	8298	7902, 7937, 7910	100, 92, 87	119.7965, 110.8278, 109.69756	28.34869, 27.89205, 29.89124	115.24937	3	NA	NA	
GL177257.1	28	936	428, 450, 458	18, 32, 16	19.78725, 33.59991, 20.52695	10.86837, 14.27354, 11.67144	16.56612,	3	NA	NA	
GL177257.1	5282	8192	6598, 6628, 6532	23, 29, 18	20.79595, 25.25061, 19.15771	9.95691, 10.56277, 10.49722	17.54084,	3	NA	NA	
GL177258.1	7707	8013	7850, 7829, 7835	5, 12, 6	4.82177, 11.86108, 7.29403	3.98509, 7.51216, 5.30581	2.51418, 9	3	NA	NA	
GL177259.1	7593	8032	7768, 7945, 7704	4, 7, 3	3.74195, 5.97725, 3.47371	3.32091, 4.62287, 3.03189	1.6222, 3.6	3	NA	NA	
GL177271.1	7919	8295	8185, 8162, 8175	34, 27, 24	36.49407, 27.51274, 30.41517	15.16999, 12.57423, 15.30667	32.85196,	3	NA	NA	
GL177276.1	186	458	432, 423, 432	32, 30, 19	35.59971, 31.15101, 23.01756	15.45531, 13.60907, 12.47022	31.9745, 2	3	NA	NA	
GL177276.1	1435	1816	1782, 1786, 1790	28, 35, 19	29.82583, 38.07658, 23.01756	13.58194, 15.68675, 12.47022	26.32197,	3	NA	NA	
GL177279.1	33	743	415, 485, 561	36, 30, 24	40.66463, 31.15101, 27.07887	16.78775, 13.60907, 13.37677	36.95168,	3	NA	NA	
GL177279.1	7284	8101	7622, 7633, 7519	21, 24, 15	21.97388, 25.55301, 15.11547	11.30529, 12.46712, 8.83977	18.68096,	3	NA	NA	
GL177282.1	70	978	311, 312, 297	11, 17, 6	12.43693, 18.56115, 7.29403	7.97018, 10.40145, 5.30581	9.52692, 1	3	NA	NA	
GL177282.1	7380	8263	7599, 7671, 7706	12, 22, 9	13.73771, 24.56226, 11.64359	8.57523, 12.53481, 7.57973	10.76705,	3	NA	NA	
GL177291.1	816	1116	846, 1030, 819	10, 12, 8	11.06011, 11.86108, 10.1465	7.306, 7.51216, 6.82176	8.22712, 9	3	NA	NA	
GL177310.1	6210	7076	6585, 6855, 6841	53, 74, 33	53.88219, 74.03261, 36.72525	16.91211, 18.36444, 15.71558	49.97483,	3	NA	NA	
GL177314.1	38	736	274, 616, 248	9, 16, 7	9.72082, 16.98517, 8.69489	6.64182, 9.71597, 6.06379	6.97155, 1	3	NA	NA	
GL177314.1	7392	8209	7467, 7452, 7438, 8096	10, 17, 6, 8	10.01299, 17.71433, 6.5517, 9.1831	6.7221, 9.9018, 4.95623, 6.37229	7.24763, 1	3	NA	NA	
GL177318.1	7846	8150	7961, 7988, 7889	6, 7, 3	5.96715, 5.97725, 3.47371	4.64927, 4.62287, 3.03189	3.52158, 3	3	NA	NA	
GL177325.1	23	758	533, 532, 519	40, 37, 32	48.2811, 40.58661, 39.80045	19.51647, 16.31596, 17.94007	44.45338,	3	NA		6981
GL177325.1	7181	8191	7625, 7754, 7610	50, 62, 41	38.8945, 49.06158, 33.92804	10.89566, 11.82478, 11.2321	35.20983,	3	NA		0
GL177333.1	7132	8151	7794, 7774, 7497	4, 11, 6	3.74195, 10.60895, 7.29403	3.32091, 6.9343, 5.30581	1.6222, 7.9	3	NA	NA	
GL177335.1	169	664	337, 249, 523	10, 11, 13	9.13239, 8.66477, 13.97446	6.2007, 5.74034, 8.57174	6.43114, 6	3	NA	NA	
GL177335.1	2190	2876	2552, 2677, 2769	11, 13, 7	9.66657, 9.14598, 5.45422	6.36338, 5.63981, 4.2975	6.93336, 6	3	NA	NA	
GL177335.1	7532	8204	7917, 7764, 8106	44, 56, 24	49.27628, 59.87484, 28.00961	18.27793, 18.77403, 13.92544	45.4338, 5	3	NA	NA	
GL177337.1	230	721	572, 695, 271	7, 12, 4	7.1696, 11.86108, 4.67325	5.31345, 7.51216, 3.78987	4.61046, 9	3	NA	NA	
GL177339.1	118	837	652, 609, 746	31, 43, 36	28.60717, 43.38306, 37.23484	11.76478, 15.39967, 14.78706	25.13226,	3	NA	NA	
GL177339.1	7561	8206	8061, 8010, 7995	80, 86, 78	95.03339, 107.11279, 100.79032	25.85563, 29.06285, 29.98407	90.68629,	3	NA	NA	
GL177352.1	5411	6035	5763, 5756, 5680	7, 12, 5	7.10297, 11.86108, 5.9506	5.27706, 7.51216, 4.54784	4.57191, 9	3	NA	NA	
GL177355.1	1061	1285	1183, 1167, 1156	580, 881, 559	746.99518, 1144.82019, 759.03778	46.97193, 49.74094, 54.32848	740.73401	3	NA	NA	
GL177362.1	78	1061	831, 781, 789	23, 23, 17	22.21857, 19.76013, 15.48278	10.80503, 9.34744, 8.5529	18.91817,	3	NA	NA	
GL177362.1	1294	2284	1909, 1738, 1801	13, 19, 16	10.21914, 15.12892, 14.26922	6.30293, 7.78953, 8.07774	7.4427, 12	3	NA	NA	
GL177362.1	7237	8130	7613, 7545, 7714	13, 11, 13	15.29169, 10.60895, 18.01082	9.29854, 6.9343, 10.61163	12.24396,	3	NA	NA	
GL177363.1	7553	8041	7655, 7819, 7711	4, 6, 5	3.74195, 4.93312, 5.9506	3.32091, 4.04501, 4.54784	1.6222, 2.7	3	NA	NA	
GL177364.1	129	749	462, 424, 597	31, 28, 18	35.41696, 30.8965, 21.30401	15.74811, 14.2202, 11.73892	31.79549,	3	NA	NA	
GL177364.1	7397	8074	7768, 7874, 7878	19, 27, 12	19.8867, 28.17926, 13.90113	10.65421, 12.97124, 8.66246	16.66031,	3	NA	NA	
GL177373.1	64	715	354, 480, 319	22, 32, 18	16.26324, 22.46185, 15.65701	7.63516, 8.2525, 8.38278	13.1768, 1	3	NA	NA	
GL177373.1	1120	1852	1588, 1730, 1489	16, 27, 9	10.21083, 17.3848, 6.03106	5.64338, 7.00212, 4.41199	7.43562, 1	3	NA	NA	
GL177373.1	7276	8117	7854, 7839, 7420	18, 25, 10	17.51609, 22.18474, 10.71812	9.51146, 10.12639, 7.12015	14.37616,	3	NA	NA	
GL177378.1	354	643	557, 593, 581	5, 13, 5	4.82177, 13.14514, 5.9506	3.98509, 8.09002, 4.54784	2.51418, 1	3	NA	NA	
GL177381.1	25	516	179, 130, 177	11, 20, 7	12.43693, 22.87459, 8.69489	7.97018, 12.13503, 6.06379	9.52692, 1	3	NA	NA	
GL177381.1	7409	8096	7807, 7818, 7824	32, 44, 21	37.15584, 50.6032, 27.07785	16.37888, 19.0429, 14.2403	33.5013, 4	3	NA	NA	
GL177382.1	362	2967	1168, 394, 1218, 1259, 2611	33, 14, 46, 19, 17	24.42937, 4.40412, 30.31836, 14.0056, 11.12147	8.93896, 2.85653, 8.43071, 7.13242, 5.9504	21.06395,	3	NA	NA	
GL177385.1	32	791	216, 653, 497	17, 27, 12	18.1272, 22.89178, 14.3175	10.1466, 9.88824, 8.88032	14.96605,	3	NA	NA	
GL177385.1	7565	8075	7766, 7916, 7755	17, 25, 9	13.35329, 16.29309, 7.51189	7.26045, 6.91444, 5.35071	10.39951,	3	NA	NA	
GL177398.1	1845	2598	1990, 2225, 1905	12, 29, 9	13.8483, 34.86947, 10.79992	8.63436, 16.19933, 7.19283	10.86694,	3	NA	NA	
GL177407.1	7474	8031	7725, 7725, 7747	13, 9, 6	15.29169, 8.21099, 7.29403	9.29854, 5.77858, 5.30581	12.24396,	3	NA	NA	
GL177420.1	1316	1808	1708, 1727, 1676	7, 10, 4	7.1696, 9.39128, 4.67325	5.31345, 6.35644, 3.78987	4.61046, 6	3	NA	NA	
GL177425.1	7427	8002	7881, 7829, 7898	16, 10, 8	19.18518, 9.39128, 10.1465	10.96495, 6.35644, 6.82176	15.98366,	3	NA	NA	
GL177431.1	11	520	400, 314, 329	33, 29, 24	34.16129, 30.45103, 29.03384	14.207, 13.57627, 14.52105	30.56488,	3	NA	NA	
GL177433.1	506	1486	786, 1034, 1017	8, 12, 15	8.42257, 11.86108, 20.14748	5.97763, 7.51216, 11.5698	5.76336, 9	3	NA	NA	
GL177449.1	946	1485	1301, 1211, 1333	10, 8, 10	11.06011, 7.07159, 13.18184	7.306, 5.20073, 8.33771	8.22712, 4	3	NA	NA	
GL177453.1	173	950	645, 804, 463	4, 9, 5	3.74195, 8.21099, 5.9506	3.32091, 5.77858, 4.54784	1.6222, 5.7	3	mtx1		6011
GL177460.1	9	939	611, 611, 609	121, 165, 91	126.95409, 177.0614, 98.8676	22.7971, 25.42497, 22.72415	122.35854	3	NA	NA	
GL177460.1	5040	7802	5802, 5660, 7119, 5812, 7138	31, 39, 13, 19, 13	23.63651, 25.40631, 6.40204, 14.03809, 9.07316	9.09149, 7.95289, 4.01419, 7.1512, 5.59511	20.29356,	3	NA	NA	
GL177461.1	4735	5310	4942, 4762, 4880	14, 9, 8	14.23954, 6.8008, 8.9249	8.53829, 4.90352, 6.24211	11.24094,	3	NA	NA	
GL177466.1	1681	2282	1942, 1942, 2003	16, 29, 16	16.75964, 31.6555, 18.0198	9.5829, 14.2926, 10.31552	13.65293,	3	NA	NA	
GL177470.1	42	609	503, 467, 523	9, 24, 7	9.72082, 28.90463, 8.69489	6.64182, 14.44646, 6.06379	6.97155, 2	3	usf1		4689
GL177473.1	353	711	402, 667, 592	6, 8, 9	5.96715, 7.07159, 11.64359	4.64927, 5.20073, 7.57973	3.52158, 4	3	NA	NA	
GL177491.1	22	884	227, 226, 226	10, 17, 12	11.06011, 18.56115, 16.36823	7.306, 10.40145, 9.85365	8.22712, 1	3	NA	NA	
GL177495.1	2662	3329	2889, 2818, 2893	12, 24, 17	13.13892, 28.90463, 23.46806	8.24771, 14.44646, 13.01603	10.1962, 2	3	NA	NA	
GL177502.1	155	768	467, 475, 368	11, 7, 5	12.43693, 5.97725, 5.9506	7.97018, 4.62287, 4.54784	9.52692, 3	3	NA	NA	
GL177507.1	117	498	328, 207, 372	9, 11, 4	9.72082, 10.60895, 4.67325	6.64182, 6.9343, 3.78987	6.97155, 7	3	NA	NA	
GL177507.1	6666	7836	7334, 7259, 6886	9, 9, 4	9.72082, 8.21099, 4.67325	6.64182, 5.77858, 3.78987	6.97155, 5	3	NA	NA	
GL177508.1	5468	6054	5698, 5682, 5547	11, 13, 10	12.43693, 13.14514, 13.18184	7.97018, 8.09002, 8.33771	9.52692, 1	3	NA	NA	
GL177509.1	561	1708	1588, 1642, 1521	15, 18, 9	15.71867, 16.94269, 11.21577	9.19755, 9.16356, 7.38849	12.65377,	3	NA	NA	
GL177521.1	7413	7869	7703, 7737, 7687	11, 8, 7	12.43693, 7.07159, 8.69489	7.97018, 5.20073, 6.06379	9.52692, 4	3	NA	NA	
GL177533.1	690	1141	969, 925, 968	9, 13, 8	9.72082, 13.14514, 8.29845	6.64182, 8.09002, 5.91009	6.97155, 1	3	NA	NA	
GL177533.1	6321	6688	6451, 6425, 6427	6, 8, 7	4.37941, 4.92092, 6.6707	3.69512, 3.83624, 5.03432	2.19155, 2	3	NA	NA	
GL177533.1	6814	7885	7067, 7225, 7127	9, 16, 9	7.39636, 12.90802, 9.08997	5.27874, 7.24623, 6.2929	4.82212, 1	3	NA	NA	
GL177534.1	45	1415	486, 354, 422	18, 24, 16	22.92104, 26.67478, 21.52278	12.61945, 13.13685, 12.16338	19.59912,	3	NA	NA	
GL177541.1	6591	7838	6937, 7065, 7376	23, 40, 15	18.75967, 34.12326, 13.30087	8.76814, 11.64208, 7.73795	15.57324,	3	NA	NA	
GL177551.1	6286	6977	6635, 6546, 6520	8, 14, 9	8.42257, 14.45897, 10.87975	5.97763, 8.66788, 7.23113	5.76336, 1	3	NA	NA	
GL177554.1	965	1309	1187, 1190, 1062	6, 13, 3	5.96715, 13.14514, 3.47371	4.64927, 8.09002, 3.03189	3.52158, 1	3	NA	NA	
GL177560.1	5612	6301	6151, 6187, 5979	11, 17, 5	11.93519, 18.53926, 5.9506	7.69727, 10.38866, 4.54784	9.0586, 15	3	NA	NA	
GL177568.1	5984	7748	6335, 6470, 6481	11, 14, 14	12.43693, 14.45897, 18.28596	7.97018, 8.66788, 10.7324	9.52692, 1	3	NA	NA	
GL177572.1	47	651	253, 218, 141	19, 25, 14	23.53998, 30.45643, 18.77534	12.7584, 15.02432, 10.96344	20.2, 27.12	3	NA	NA	
GL177581.1	2495	2878	2633, 2672, 2758	6, 9, 7	5.96715, 8.21099, 8.69489	4.64927, 5.77858, 6.06379	3.52158, 5	3	NA	NA	
GL177582.1	1647	2194	1752, 1750, 1692	10, 20, 14	11.06011, 22.87459, 19.68329	7.306, 12.13503, 11.3696	8.22712, 1	3	NA	NA	
GL177597.1	56	564	312, 280, 281	14, 15, 8	15.84929, 15.80067, 10.1465	9.46495, 9.24574, 6.82176	12.77923,	3	NA	NA	
GL177597.1	6702	7795	7068, 6972, 7005, 7389	12, 14, 11, 5	12.18267, 12.92044, 12.9493, 5.06966	7.70145, 7.74345, 8.23866, 4.11933	9.29304, 1	3	NA	NA	
GL177599.1	4303	4882	4620, 4436, 4463	7, 10, 6	7.1696, 9.39128, 7.25145	5.31345, 6.35644, 5.287	4.61046, 6	3	NA	NA	
GL177604.1	75	463	405, 276, 428	19, 19, 19	24.51879, 21.4153, 28.30916	13.28363, 11.55717, 15.10573	21.15031,	3	NA	NA	
GL177604.1	7234	7821	7512, 7498, 7528	8, 12, 12	8.42257, 11.86108, 16.36823	5.97763, 7.51216, 9.85365	5.76336, 9	3	NA	NA	
GL177605.1	6483	7553	6817, 6859, 6860	7, 14, 6	7.1696, 14.45897, 7.29403	5.31345, 8.66788, 5.30581	4.61046, 1	3	NA	NA	
GL177608.1	10	546	367, 385, 388	18, 22, 15	22.92104, 25.85271, 20.69165	12.61945, 13.29074, 11.82159	19.59912,	3	NA	NA	
GL177621.1	363	1140	917, 930, 410	10, 18, 4	11.06011, 19.97713, 4.67325	7.306, 10.97931, 3.78987	8.22712, 1	3	NA	NA	
GL177648.1	6757	7386	7214, 6896, 6889	10, 12, 6	10.96713, 11.72254, 7.29403	7.25596, 7.42986, 5.30581	8.14942, 9	3	NA	NA	
GL177653.1	29	499	276, 263, 398	10, 15, 16	11.06011, 15.80067, 22.07892	7.306, 9.24574, 12.42524	8.22712, 1	3	NA	NA	
GL177666.1	21	1075	504, 373, 371	213, 260, 118	218.91147, 261.38342, 114.21493	24.10582, 23.54144, 19.33525	213.83267	3	NA	NA	
GL177666.1	6923	7745	7598, 7599, 7602	23, 46, 25	21.54503, 44.73041, 28.19131	10.40228, 14.93553, 13.69598	18.26496,	3	NA	NA	
GL177667.1	25	554	325, 287, 312	47, 42, 30	55.02565, 47.80266, 39.44262	20.20214, 18.32871, 18.48036	51.10247,	3	5S_rRNA		0
GL177673.1	6911	7684	6950, 6959, 7036	8, 13, 3	8.42257, 13.14514, 3.47371	5.97763, 8.09002, 3.03189	5.76336, 1	3	NA	NA	
GL177675.1	571	1002	897, 634, 816	5, 11, 3	4.82177, 10.60895, 3.47371	3.98509, 6.9343, 3.03189	2.51418, 7	3	NA	NA	
GL177676.1	6290	7732	6848, 6747, 7307	29, 40, 20	34.25356, 42.7533, 22.80441	15.83622, 16.29291, 12.09422	30.65537,	3	NA	NA	
GL177695.1	28	535	358, 137, 224	10, 18, 8	11.06011, 19.97713, 10.1465	7.306, 10.97931, 6.82176	8.22712, 1	3	NA	NA	
GL177695.1	6911	7718	7268, 7268, 7267	88, 116, 56	94.28325, 123.00185, 62.09533	21.91271, 23.06684, 19.96048	89.94315,	3	NA	NA	
GL177700.1	14	1849	533, 244, 528, 1589	96, 117, 70, 17	79.00612, 100.47689, 62.3945, 6.28759	13.83473, 15.43484, 14.79275, 3.45356	74.81918,	3	NA	NA	
GL177700.1	3876	4637	4436, 4166, 4169	24, 24, 16	7.29068, 6.62684, 5.57147	3.23619, 3.00859, 3.22524	4.72288, 4	3	NA	NA	
GL177712.1	40	834	516, 559, 506	20, 38, 18	25.95659, 47.50827, 23.74313	13.85229, 19.95743, 13.04455	22.549, 43	3	NA	NA	
GL177712.1	6850	7700	7406, 7326, 7316	37, 39, 22	41.27616, 42.89945, 26.58401	16.71956, 16.80585, 13.71123	37.55333,	3	NA	NA	
GL177716.1	7242	7621	7411, 7405, 7398	17, 17, 18	21.34551, 18.56115, 22.76598	11.95527, 10.40145, 12.53748	18.07159,	3	NA	NA	
GL177733.1	1880	2296	2104, 2172, 2244	8, 8, 5	8.42257, 7.07159, 5.9506	5.97763, 5.20073, 4.54784	5.76336, 4	3	NA	NA	
GL177739.1	88	692	532, 542, 497	11, 19, 6	10.3251, 19.33741, 6.99807	6.7644, 10.3246, 5.17195	7.54325, 1	3	NA	NA	
GL177743.1	6587	7503	7324, 6720, 7233	7, 10, 5	5.65516, 8.70492, 5.50079	4.42382, 5.93975, 4.33868	3.27228, 6	3	NA	NA	
GL177746.1	1982	2719	2376, 2590, 2273	10, 14, 10	8.0085, 9.48413, 9.93124	5.50663, 5.63643, 6.67475	5.39412, 6	3	NA	NA	
GL177746.1	7177	7578	7444, 7444, 7422	14, 23, 10	13.89699, 23.82337, 10.42037	8.3343, 11.76844, 6.95441	10.91278,	3	NA	NA	
GL177754.1	6563	7547	7075, 7363, 7369	25, 24, 18	30.32096, 27.93659, 24.85881	14.94603, 13.88262, 13.59437	26.80639,	3	cryaa		3952
GL177787.1	6943	7565	7301, 7301, 7301	8, 10, 7	8.10827, 9.39128, 7.40077	5.80497, 6.35644, 5.43746	5.48761, 6	3	NA	NA	
GL177793.1	6429	6750	6616, 6445, 6554	9, 12, 6	9.72082, 11.86108, 7.29403	6.64182, 7.51216, 5.30581	6.97155, 9	3	NA	NA	
GL177799.1	6805	7245	7080, 6957, 6979	10, 8, 7	11.06011, 7.07159, 8.69489	7.306, 5.20073, 6.06379	8.22712, 4	3	NA	NA	
GL177810.1	62	669	376, 551, 388	23, 25, 12	27.72716, 26.78367, 14.96175	14.07465, 12.85656, 9.20442	24.27442,	3	NA	NA	
GL177810.1	6660	7492	6887, 7132, 6885	19, 25, 13	17.50859, 21.95612, 13.33541	9.21834, 9.99375, 8.20195	14.36919,	3	NA	NA	
GL177812.1	125	865	837, 791, 710	21, 57, 19	27.77672, 73.40625, 25.38515	14.612, 25.84883, 13.7311	24.32202,	3	NA	NA	
GL177817.1	391	4082	3078, 2887, 2902	65, 95, 62	40.14042, 63.06807, 46.50175	8.30321, 9.78699, 10.88764	36.43562,	3	NA	NA	
GL177817.1	7097	7521	7233, 7210, 7347	45, 46, 21	28.83662, 25.49659, 11.21665	8.07648, 6.74948, 5.21584	25.357, 22	3	NA	NA	
GL177826.1	17	813	158, 254, 580	6, 9, 7	5.96715, 8.21099, 8.69489	4.64927, 5.77858, 6.06379	3.52158, 5	3	NA	NA	
GL177826.1	7084	7550	7301, 7309, 7317	18, 43, 15	22.92104, 55.07026, 20.98137	12.61945, 22.13016, 11.95164	19.59912,	3	NA	NA	
GL177844.1	6749	7388	6851, 7138, 6816	15, 12, 8	18.26603, 11.86108, 9.31951	10.62691, 7.51216, 6.43944	15.09729,	3	NA	NA	
GL177847.1	21	1005	111, 346, 339	13, 31, 13	11.55805, 28.7313, 14.69628	7.13125, 11.83495, 8.97644	8.70034, 2	3	NA	NA	
GL177847.1	6558	7186	6644, 6669, 6905	9, 27, 9	9.72082, 31.32725, 10.87975	6.64182, 14.84619, 7.23113	6.97155, 2	3	NA	NA	
GL177853.1	60	1080	301, 236, 233	16, 16, 16	17.3328, 15.80758, 19.96942	9.91952, 9.01376, 11.38369	14.20089,	3	NA	NA	
GL177853.1	4959	7385	6693, 6209, 6595	22, 30, 16	18.08944, 23.5747, 14.3623	8.67537, 9.34511, 8.13466	14.92943,	3	NA	NA	
GL177859.1	6291	6824	6560, 6549, 6553	15, 13, 6	18.26603, 13.14514, 7.29403	10.62691, 8.09002, 5.30581	15.09729,	3	NA	NA	
GL177862.1	82	4206	149, 3425, 307, 1770, 3572	10, 53, 11, 15, 30	4.04118, 39.36602, 5.76233, 8.91835, 24.17276	3.0434, 10.33381, 3.93817, 5.10292, 9.66003	1.89458, 3	3	NA	NA	
GL177862.1	216	4206	1238, 3425, 307, 1770, 3572	18, 53, 11, 15, 30	10.11313, 39.36602, 5.76233, 8.91835, 24.17276	5.19498, 10.33381, 3.93817, 5.10292, 9.66003	7.34321, 3	3	NA	NA	
GL177862.1	216	4223	3148, 3425, 307, 1770, 3572	42, 53, 11, 15, 30	34.19078, 39.36602, 5.76233, 8.91835, 24.17276	11.0552, 10.33381, 3.93817, 5.10292, 9.66003	30.59389,	3	NA	NA	
GL177863.1	6773	7439	7254, 7169, 7235	4, 17, 14	3.74195, 18.56115, 19.68329	3.32091, 10.40145, 11.3696	1.6222, 15	3	NA	NA	
GL177865.1	1024	1290	1177, 1153, 1192	12, 13, 8	12.11599, 13.14514, 9.92329	7.66241, 8.09002, 6.7228	9.23027, 1	3	acaa2		0
GL177873.1	173	725	457, 497, 505	10, 20, 6	11.06011, 22.87459, 7.29403	7.306, 12.13503, 5.30581	8.22712, 1	3	NA	NA	
GL177873.1	5888	7361	6468, 6085, 6478	13, 19, 9	14.61367, 21.01008, 11.39481	8.93088, 11.32031, 7.46977	11.59864,	3	NA	NA	
GL177876.1	7	213	101, 89, 108	79, 125, 74	99.09218, 159.71745, 98.38962	28.51051, 34.7688, 30.88262	94.70988,	3	NA	NA	
GL177877.1	920	1361	1104, 994, 1037	12, 14, 6	13.8483, 14.45897, 7.29403	8.63436, 8.66788, 5.30581	10.86694,	3	NA	NA	
GL177885.1	6950	7385	7083, 7291, 7105	5, 10, 8	4.82177, 9.39128, 10.1465	3.98509, 6.35644, 6.82176	2.51418, 6	3	NA	NA	
GL177900.1	7129	7358	7162, 7140, 7181	11, 7, 5	12.43693, 5.97725, 5.9506	7.97018, 4.62287, 4.54784	9.52692, 3	3	NA	NA	
GL177923.1	219	653	326, 302, 274	24, 39, 18	28.77451, 48.24606, 22.10559	14.37118, 19.94739, 12.18231	25.29621,	3	NA	NA	
GL177923.1	6697	7417	7195, 7258, 7084	47, 64, 35	49.34941, 70.26711, 37.55862	17.00209, 20.65552, 15.38339	45.50589,	3	NA	NA	
GL177924.1	5980	6377	6251, 6106, 6207	9, 8, 7	9.72082, 7.07159, 8.69489	6.64182, 5.20073, 6.06379	6.97155, 4	3	NA	NA	
GL177931.1	16	792	333, 315, 343	307, 405, 225	284.7626, 359.93832, 211.19112	20.13985, 18.79662, 20.03605	279.51837	3	NA	NA	
GL177935.1	55	697	138, 159, 149	8, 14, 10	8.42257, 14.45897, 12.90749	5.97763, 8.66788, 8.21675	5.76336, 1	3	NA	NA	
GL177935.1	6356	7292	6520, 7071, 6381, 7019	7, 14, 5, 10	6.99564, 14.45897, 5.80393, 12.90749	5.21786, 8.66788, 4.48186, 8.21675	4.47307, 1	3	NA	NA	
GL177935.1	6356	7292	6994, 7071, 6381, 7019	11, 14, 5, 10	12.17114, 14.45897, 5.80393, 12.90749	7.82679, 8.66788, 4.48186, 8.21675	9.28263, 1	3	NA	NA	
GL177936.1	69	1799	1171, 495, 1006	31, 36, 13	32.42437, 35.60603, 14.39632	13.97388, 13.85882, 8.81006	28.8638, 3	3	NA	NA	
GL177936.1	6451	7400	7085, 7074, 6964	21, 25, 15	25.88765, 29.92533, 18.95532	13.58701, 14.71641, 10.98488	22.48219,	3	NA	NA	
GL177938.1	6557	6833	6705, 6648, 6693	15, 21, 15	18.26603, 24.1145, 20.14748	10.62691, 12.57361, 11.5698	15.09729,	3	NA	NA	
GL177942.1	1254	1464	1439, 1438, 1407	12, 15, 6	13.8483, 15.80067, 7.29403	8.63436, 9.24574, 5.30581	10.86694,	3	NA	NA	
GL177944.1	6466	7306	7044, 7087, 7064	12, 14, 9	13.8483, 14.45897, 11.64359	8.63436, 8.66788, 7.57973	10.86694,	3	NA	NA	
GL177952.1	6630	7322	7165, 7179, 6992	6, 16, 8	4.99728, 12.73735, 8.14528	4.08451, 7.14249, 5.82558	2.67584, 9	3	NA	NA	
GL177959.1	137	793	585, 544, 533	18, 13, 13	22.49411, 13.14514, 16.0859	12.39242, 8.09002, 9.70995	19.18565,	3	NA	NA	
GL177959.1	5952	7257	6540, 6549, 6536	17, 16, 16	19.60089, 17.16856, 20.763	10.9998, 9.82359, 11.79066	16.38573,	3	NA	NA	
GL177965.1	20	935	480, 546, 871	12, 14, 10	13.55951, 14.45897, 13.18184	8.47903, 8.66788, 8.33771	10.59689,	3	NA	NA	
GL177965.1	6030	6916	6432, 6418, 6417	16, 30, 10	17.08121, 29.23872, 11.6791	9.7724, 12.48814, 7.62925	13.95894,	3	NA	NA	
GL177975.1	160	970	808, 850, 319, 891	7, 15, 3, 7	7.1696, 15.80067, 3.47371, 8.69489	5.31345, 9.24574, 3.03189, 6.06379	4.61046, 1	3	NA	NA	
GL177978.1	11	1089	330, 381, 517	51, 64, 50	54.7126, 65.33978, 55.2419	18.1972, 18.1929, 18.93347	50.7935, 6	3	NA	NA	
GL177978.1	6605	7367	7236, 6857, 7084	20, 24, 13	23.18601, 23.34068, 15.34872	12.31532, 11.14174, 9.3288	19.85599,	3	NA	NA	
GL177979.1	896	1309	1105, 1052, 1226	9, 8, 18	9.72082, 7.07159, 24.85881	6.64182, 5.20073, 13.59437	6.97155, 4	3	NA	NA	
GL177983.1	6476	7342	6877, 7023, 6925	76, 115, 56	85.03918, 127.38454, 69.66695	22.70866, 25.15082, 24.20471	80.79177,	3	NA	NA	
GL177996.1	249	1675	365, 1089, 1052	18, 21, 9	21.52103, 20.96503, 11.64359	11.86022, 10.69835, 7.57973	18.2418, 1	3	NA	NA	
GL177996.1	6222	7289	6380, 6437, 6371	22, 24, 23	24.08614, 22.33212, 26.05526	12.25235, 10.54139, 13.09977	20.7301, 1	3	NA	NA	
GL178011.1	2180	4738	3065, 2997, 3030	110, 105, 52	70.66512, 60.03855, 30.22494	9.50253, 8.00986, 7.34588	66.56214,	3	NA	NA	
GL178011.1	5725	7262	6051, 6775, 6078	64, 78, 42	30.87554, 39.14807, 23.74151	6.06462, 6.53319, 6.7759	27.34824,	3	NA	NA	
GL178013.1	13	514	226, 206, 229	100, 68, 86	98.8644, 56.29366, 90.64609	19.43519, 12.9925, 21.10527	94.48412,	3	5_8S_rRNA		5928
GL178013.1	4518	4980	4707, 4721, 4628	61, 64, 36	31.51163, 33.73855, 16.40356	6.52857, 6.74375, 5.12825	27.96998,	3	5_8S_rRNA		1462
GL178013.1	6215	7286	6534, 6524, 6603	74, 76, 43	52.99434, 52.91321, 28.31533	10.52906, 10.13968, 8.27829	49.09975,	3	5_8S_rRNA		0
GL178015.1	4571	7258	4936, 4934, 4892	47, 62, 47	36.1357, 48.96135, 43.98174	10.52547, 11.78708, 14.17675	32.49978,	3	NA	NA	
GL178019.1	220	759	548, 290, 533	7, 9, 9	6.09019, 8.21099, 9.19414	4.69155, 5.77858, 6.35176	3.63504, 5	3	NA	NA	
GL178019.1	708	1282	1006, 1000, 533, 1195	13, 13, 9, 10	13.34951, 13.14514, 9.19414, 10.25108	8.21021, 8.09002, 6.35176, 6.85862	10.39596,	3	NA	NA	
GL178019.1	6145	7273	6749, 6453, 6297, 7069	8, 13, 8, 8	8.10827, 12.61502, 8.14528, 8.24665	5.80497, 7.77427, 5.82558, 5.88165	5.48761, 9	3	NA	NA	
GL178019.1	6190	7278	7164, 6453, 6297, 7069	7, 13, 8, 8	6.89193, 12.61502, 8.14528, 8.24665	5.15998, 7.77427, 5.82558, 5.88165	4.37772, 9	3	NA	NA	
GL178031.1	44	581	410, 432, 269	19, 29, 14	23.80016, 36.49205, 19.03529	12.89994, 17.14583, 11.08274	20.45294,	3	NA	NA	
GL178031.1	6655	7263	6976, 6769, 6973	32, 47, 22	38.64939, 52.61056, 28.7037	17.26311, 18.81977, 14.88759	34.96902,	3	NA	NA	
GL178033.1	6727	7171	6897, 6920, 7028	12, 12, 9	13.8483, 11.86108, 11.64359	8.63436, 7.51216, 7.57973	10.86694,	3	NA	NA	
GL178040.1	3092	3758	3515, 3604, 3632	9, 8, 5	7.80169, 6.97779, 5.59755	5.52977, 5.14375, 4.38538	5.20141, 4	3	NA	NA	

GL178056.1	36	288	130, 140, 109	114, 143, 79	132.02591, 164.71086, 96.72131	27.49901, 28.77662, 27.24199	127.40073	3	NA	NA
GL178072.1	562	806	665, 745, 780	4, 15, 6	3.74195, 15.80067, 7.29403	3.32091, 9.24574, 5.30581	1.6222, 12	3	NA	NA
GL178073.1	529	780	736, 725, 737	26, 42, 13	35.63428, 58.95477, 18.01082	17.61029, 24.84791, 10.61163	32.00842,	3	NA	NA
GL178083.1	6179	6718	6356, 6305, 6421	8, 8, 7	8.42257, 7.07159, 8.69489	5.97763, 5.20073, 6.06379	5.76336, 4	3	NA	NA
GL178088.1	43	1172	322, 354, 427	19, 26, 8	19.10984, 21.86052, 7.42597	10.1875, 9.62194, 5.41279	15.91125,	3	NA	NA
GL178088.1	3328	4631	3712, 3917, 4035	16, 32, 11	13.01894, 25.87039, 9.92808	7.31376, 9.94802, 6.52366	10.08178,	3	NA	NA
GL178088.1	6196	7019	6568, 6839, 6843	10, 16, 5	8.75281, 12.32991, 4.64121	5.9692, 6.89567, 3.88372	6.07523, 9	3	NA	NA
GL178092.1	6349	6604	6572, 6575, 6561	11, 26, 12	12.43693, 32.02458, 16.36823	7.97018, 15.60218, 9.85365	9.52692, 2	3	NA	NA
GL178099.1	64	1390	968, 1036, 190, 859, 1267	25, 41, 16, 10, 12	18.77733, 30.31262, 13.40532, 7.02444, 9.02948	8.2092, 9.56573, 7.54944, 4.88493, 5.7731	15.58996,	3	NA	NA
GL178099.1	6392	7164	6537, 6547, 6502	25, 33, 13	25.3255, 32.04128, 14.11158	11.98384, 12.98372, 8.64973	21.93564,	3	NA	NA
GL178103.1	30	578	225, 245, 192	15, 19, 10	17.48779, 20.47061, 12.71002	10.20672, 11.002, 8.12734	14.34959,	3	NA	NA
GL178103.1	5916	7170	6045, 6094, 6237, 6700	24, 31, 15, 12	22.29931, 26.4499, 14.01468, 10.42593	10.52194, 10.56947, 8.17463, 6.64188	18.99699,	3	NA	NA
GL178108.1	63	700	371, 419, 311	15, 22, 7	18.12902, 25.60195, 8.69489	10.55412, 13.14513, 6.06379	14.96737,	3	NA	NA
GL178108.1	6091	7162	6907, 6927, 7002	16, 20, 6	19.64758, 21.69995, 7.29403	11.21376, 11.44483, 5.30581	16.43074,	3	NA	NA
GL178116.1	966	2184	1969, 1781, 1232, 2014	24, 27, 15, 12	8.86872, 9.14443, 7.0989, 4.60449	3.80857, 3.63416, 4.10122, 3.14804	6.18473, 6	3	NA	NA
GL178125.1	130	695	213, 257, 573	10, 10, 10	11.06011, 9.39128, 13.18184	7.306, 6.35644, 8.33771	8.22712, 6	3	NA	NA
GL178125.1	6366	7147	7039, 6821, 7014	20, 26, 23	19.84853, 24.56489, 25.12075	10.3347, 11.18383, 12.54512	16.62382,	3	NA	NA
GL178139.1	103	1007	707, 762, 510	15, 26, 10	13.5229, 23.97783, 9.53891	7.87405, 10.83957, 6.44439	10.56184,	3	NA	NA
GL178139.1	3446	4006	3705, 3611, 3642	16, 18, 10	13.87338, 13.16644, 8.69318	7.83559, 6.89662, 5.93253	10.89011,	3	NA	NA
GL178142.1	5088	6330	5641, 6099, 6143	19, 29, 11	20.56775, 29.27601, 13.83651	11.05955, 12.88102, 8.67735	17.31957,	3	NA	NA
GL178147.1	52	707	114, 480, 490	16, 31, 15	19.64758, 35.77021, 18.95532	11.21376, 15.95791, 10.98488	16.43074,	3	NA	NA
GL178154.1	6344	6943	6724, 6734, 6734	10, 14, 7	11.06011, 14.45897, 8.69489	7.306, 8.66788, 6.06379	8.22712, 1	3	NA	NA
GL178156.1	59	800	239, 177, 727, 301	6, 9, 7, 13	5.96715, 8.21099, 5.97725, 18.01082	4.64927, 5.77858, 4.62287, 10.61163	3.52158, 5	3	NA	NA
GL178158.1	13	931	593, 569, 294, 581	11, 16, 8, 6	11.56615, 17.16856, 9.31951, 6.6567	7.49066, 9.82359, 6.43944, 5.00845	8.70729, 1	3	NA	NA
GL178158.1	6238	7093	6938, 6917, 6872	23, 21, 18	25.92656, 21.89512, 22.10559	13.02372, 11.25804, 12.18231	22.51952,	3	NA	NA
GL178159.1	256	912	595, 363, 536	23, 20, 16	27.97737, 19.50661, 19.25686	14.21807, 10.12815, 11.00387	24.51834,	3	NA	NA
GL178159.1	6803	7100	6975, 6876, 7033	12, 16, 7	13.8483, 17.16856, 8.69489	8.63436, 9.82359, 6.06379	10.86694,	3	NA	NA
GL178160.1	96	523	277, 279, 277	242, 308, 183	273.92932, 344.70398, 214.57869	30.5049, 30.65003, 31.55785	268.70432	3	NA	NA
GL178162.1	91	579	157, 288, 463	12, 10, 9	13.8483, 9.39128, 11.64359	8.63436, 6.35644, 7.57973	10.86694,	3	NA	NA
GL178162.1	6693	7061	6863, 6850, 6835	18, 20, 26	22.92104, 22.87459, 34.77934	12.61945, 12.13503, 17.14977	19.59912,	3	NA	NA
GL178164.1	6366	7074	6871, 6622, 6774	15, 16, 5	18.26603, 17.16856, 5.9506	10.62691, 9.82359, 4.54784	15.09729,	3	NA	NA
GL178172.1	102	1304	885, 873, 612	30, 31, 15	32.68525, 33.4732, 16.91122	14.51862, 14.59388, 9.88541	29.11878,	3	NA	NA
GL178172.1	6344	7026	6525, 6523, 6513	31, 41, 22	37.61042, 46.98243, 29.82201	17.04674, 18.3012, 15.47777	33.94708,	3	NA	NA
GL178177.1	50	542	385, 389, 414	467, 534, 327	466.3609, 521.8432, 320.9585	24.37827, 23.40283, 22.84491	460.7728,	3	NA	NA
GL178177.1	6579	7083	6960, 6729, 6967	479, 580, 454	482.03357, 585.44482, 484.05811	24.84752, 25.30189, 28.32059	476.37723	3	NA	NA
GL178181.1	117	910	433, 292, 320, 800	41, 27, 16, 7	47.28765, 28.17926, 18.69936, 6.6707	18.47951, 12.97124, 10.69793, 5.03432	43.47403,	3	NA	NA
GL178181.1	6387	7036	6604, 6633, 6800	16, 31, 14	18.75167, 36.71719, 17.40994	10.72694, 16.51936, 10.29833	15.56543,	3	NA	NA
GL178189.1	6203	7041	6691, 6550, 6580	9, 6, 10	9.72082, 4.93312, 13.18184	6.64182, 4.04501, 8.33771	6.97155, 2	3	NA	NA
GL178190.1	47	1028	107, 265, 972	12, 22, 11	8.1199, 17.89095, 10.87462	5.20753, 8.56031, 7.09143	5.49838, 1	3	NA	NA
GL178190.1	47	1440	932, 836, 972	31, 24, 11	30.44853, 20.21746, 10.87462	12.81853, 9.30469, 7.09143	26.93105,	3	NA	NA
GL178190.1	6308	7053	6801, 6741, 6731	10, 17, 9	10.67246, 18.56115, 10.87975	7.09497, 10.40145, 7.23113	7.87096, 1	3	NA	NA
GL178195.1	5309	6287	5560, 6038, 6054	17, 21, 10	20.02116, 22.93552, 13.18184	11.236, 11.87951, 8.33771	16.79069,	3	NA	0
GL178198.1	68	504	234, 374, 118	7, 8, 5	7.1696, 7.07159, 5.9506	5.31345, 5.20073, 4.54784	4.61046, 4	3	NA	NA
GL178198.1	6262	6929	6365, 6620, 6762	8, 12, 5	8.42257, 11.86108, 5.9506	5.97763, 7.51216, 4.54784	5.76336, 9	3	NA	NA
GL178201.1	227	785	542, 742, 536	8, 10, 6	8.42257, 9.39128, 7.29403	5.97763, 6.35644, 5.30581	5.76336, 6	3	NA	NA
GL178202.1	6316	7032	6615, 6626, 6625	142, 207, 94	144.98384, 213.66203, 99.30711	22.28127, 24.23632, 21.72642	140.28497	3	NA	NA
GL178207.1	6235	6973	6582, 6368, 6748	17, 12, 22	21.34551, 11.86108, 32.16058	11.95527, 7.51216, 16.63159	18.07159,	3	NA	NA
GL178214.1	55	744	292, 301, 178	28, 25, 17	30.3901, 23.67755, 18.96688	13.91829, 11.00257, 10.63714	26.8741, 2	3	NA	NA
GL178214.1	6548	6944	6806, 6782, 6815	17, 9, 23	21.34551, 8.21099, 32.15533	11.95527, 5.77858, 16.47733	18.07159,	3	NA	NA
GL178217.1	5508	6084	5674, 5840, 5832	6, 8, 3	5.96715, 7.07159, 3.47371	4.64927, 5.20073, 3.03189	3.52158, 4	3	NA	NA
GL178219.1	32	860	298, 374, 374	35, 39, 31	34.74184, 38.25102, 31.6252	13.75734, 14.16869, 13.50416	31.1342, 3	3	NA	NA
GL178219.1	6272	6965	6457, 6402, 6394	57, 58, 43	60.59748, 62.0778, 46.23166	18.71946, 19.05843, 16.9848	56.60333,	3	NA	NA
GL178220.1	85	753	302, 326, 297	30, 26, 11	25.23847, 18.03156, 8.6676	10.23244, 7.55705, 5.74212	21.85131,	3	NA	NA
GL178220.1	1953	2824	2673, 2164, 2117	15, 28, 15	7.44225, 16.60338, 11.69344	4.28406, 6.43023, 6.75208	4.86624, 1	3	NA	NA
GL178220.1	6239	6894	6501, 6485, 6492	25, 32, 9	22.31838, 25.57919, 7.2501	10.20414, 9.79705, 5.1872	19.01523,	3	NA	NA
GL178224.1	1498	1959	1673, 1709, 1729	11, 12, 8	5.10477, 4.78102, 4.70295	3.54801, 3.24407, 3.69471	2.77466, 2	3	NA	NA
GL178225.1	50	729	328, 437, 468	9, 11, 10	9.72082, 10.60895, 13.18184	6.64182, 6.9343, 8.33771	6.97155, 7	3	NA	NA
GL178237.1	13	767	517, 238, 592	18, 21, 19	16.99409, 20.12483, 22.04849	9.19478, 10.19169, 11.92281	13.87471,	3	NA	NA
GL178237.1	6306	6714	6388, 6587, 6455	6, 12, 5	5.96715, 11.86108, 5.9506	4.64927, 7.51216, 4.54784	3.52158, 9	3	NA	NA
GL178238.1	34	737	211, 219, 256	12, 27, 10	13.8483, 29.07231, 11.997	8.63436, 13.5042, 7.78835	10.86694,	3	NA	NA
GL178238.1	6076	6892	6323, 6218, 6259	36, 35, 22	40.88857, 37.11637, 26.79743	16.91979, 15.12527, 13.83268	37.17175,	3	NA	NA
GL178242.1	30	682	539, 520, 239	18, 21, 12	19.00683, 23.31142, 14.76955	10.40758, 12.1022, 9.10943	15.81175,	3	NA	NA
GL178242.1	6326	6896	6759, 6755, 6739	9, 16, 13	9.72082, 17.16856, 15.70541	6.64182, 9.82359, 9.51556	6.97155, 1	3	NA	NA
GL178251.1	29	443	143, 208, 181	15, 32, 10	18.26603, 40.45961, 13.18184	10.62691, 18.32506, 8.33771	15.09729,	3	NA	NA
GL178251.1	6095	6902	6421, 6414, 6427	30, 27, 12	37.40102, 30.66659, 14.96175	17.30691, 14.45444, 9.20442	33.74138,	3	NA	NA
GL178253.1	14	402	341, 140, 283	8, 12, 5	5.72321, 9.62333, 4.57714	4.35542, 6.14359, 3.84705	3.33547, 7	3	NA	NA
GL178253.1	3156	4934	3460, 4148, 4118	15, 9, 7	12.90264, 5.99677, 6.6707	7.49348, 4.39002, 5.03432	9.97073, 3	3	NA	NA
GL178259.1	96	857	611, 593, 469	26, 27, 14	22.508, 21.64324, 12.33709	9.9897, 9.19653, 7.38584	19.19906,	3	NA	NA
GL178259.1	4203	4718	4407, 4393, 4451	23, 31, 21	15.24048, 22.78959, 16.6425	6.83532, 8.66717, 8.12297	12.19881,	3	NA	NA
GL178259.1	6040	6744	6299, 6299, 6159	32, 22, 23	31.02794, 17.00044, 22.32921	12.77889, 8.04983, 10.87133	27.49761,	3	NA	NA
GL178275.1	977	1254	1169, 1175, 1097	7, 9, 3	7.1696, 8.21099, 3.47371	5.31345, 5.77858, 3.03189	4.61046, 5	3	NA	NA
GL178289.1	5298	6510	6157, 6307, 6134	9, 13, 6	8.88537, 12.67661, 7.05929	6.17591, 7.81122, 5.20024	6.19961, 9	3	NA	NA
GL178296.1	194	1039	516, 681, 815	5, 9, 9	4.82177, 8.21099, 10.01275	3.98509, 5.77858, 6.79683	2.51418, 5	3	NA	NA
GL178297.1	1402	1965	1550, 1626, 1793	10, 23, 17	11.06011, 27.36982, 19.63801	7.306, 13.8686, 11.0208	8.22712, 2	3	NA	NA
GL178304.1	1477	1940	1715, 1724, 1722	297, 409, 195	321.67905, 435.85223, 209.24962	28.25741, 28.05565, 26.15164	316.37115	3	NA	NA
GL178306.1	56	560	341, 220, 145	10, 9, 6	10.96713, 8.21099, 7.29403	7.25596, 5.77858, 5.30581	8.14942, 5	3	NA	NA
GL178306.1	2000	2647	2069, 2508, 2417	6, 9, 7	5.47306, 8.10603, 8.21885	4.36955, 5.71528, 5.84717	3.10811, 5	3	NA	NA
GL178309.1	110	1145	770, 759, 764	20, 26, 23	25.95659, 31.72888, 32.15533	13.85229, 15.43124, 16.47733	22.549, 28	3	NA	NA
GL178309.1	6072	6520	6242, 6073, 6319	7, 7, 5	7.1696, 5.97725, 5.9506	5.31345, 4.62287, 4.54784	4.61046, 3	3	NA	NA
GL178312.1	2733	3249	3041, 3122, 2824	5, 9, 4	4.82177, 8.21099, 4.67325	3.98509, 5.77858, 3.78987	2.51418, 5	3	NA	0
GL178314.1	3282	6398	4234, 4961, 3721, 5698	17, 26, 11, 8	13.10638, 20.78105, 10.66921, 7.15322	7.11242, 9.01951, 6.9701, 5.25003	10.16442,	3	U3	0
GL178315.1	5949	6522	6204, 6146, 6199	5, 10, 7	4.82177, 9.39128, 8.69489	3.98509, 6.35644, 6.06379	2.51418, 6	3	NA	NA
GL178316.1	30	535	178, 102, 348	6, 15, 14	4.7134, 13.58442, 13.43751	3.90801, 7.91173, 8.05781	2.47723, 1	3	NA	NA
GL178316.1	4125	5004	4474, 4736, 4450	14, 16, 20	13.32664, 14.46749, 21.34975	7.99066, 8.19898, 11.23648	10.37457,	3	NA	NA
GL178324.1	57	592	402, 363, 545	48, 85, 35	9.52533, 20.26021, 7.90613	2.69106, 3.2351, 2.80357	6.80131, 1	3	NA	NA
GL178324.1	1696	2852	1947, 1942, 2036	104, 154, 79	37.48405, 55.07531, 33.24161	4.68102, 4.7736, 5.34706	33.82315,	3	NA	NA
GL178324.1	3190	3630	3419, 3373, 3362	70, 85, 48	16.5806, 15.03878, 12.60486	3.16526, 2.64858, 3.27507	13.48183,	3	NA	NA
GL178324.1	4837	5657	5134, 5124, 5146	77, 109, 60	20.88601, 27.72783, 20.224	3.54252, 3.46221, 4.15275	17.62856,	3	NA	NA
GL178324.1	5869	6302	6129, 6070, 6142	68, 85, 38	17.65863, 18.035, 8.82246	3.38212, 2.97835, 2.89445	14.5139, 1	3	NA	NA
GL178326.1	5980	6492	6368, 6368, 6396	420, 449, 378	431.77652, 449.57034, 400.69742	25.85669, 24.46134, 27.52642	426.29675	3	NA	NA
GL178336.1	3703	4383	3955, 4118, 4123	10, 18, 10	5.99039, 10.6881, 7.45013	4.23145, 5.50158, 5.15463	3.54322, 8	3	NA	NA
GL178341.1	5704	6383	6054, 6064, 5916	24, 33, 25	25.36666, 35.19981, 27.83933	12.3555, 14.81526, 13.48673	21.97541,	3	NA	NA
GL178345.1	39	1174	424, 765, 579	10, 8, 14	11.06011, 6.73981, 18.05504	7.306, 4.99774, 10.62048	8.22712, 4	3	NA	NA
GL178347.1	242	1360	926, 860, 813	24, 28, 16	24.60735, 26.47393, 17.04172	11.90029, 11.60281, 9.74922	21.23605,	3	NA	NA
GL178347.1	3139	4361	3259, 3925, 4014	10, 24, 11	6.95336, 19.33661, 9.67839	4.83987, 8.80231, 6.37065	4.43454, 1	3	NA	NA
GL178347.1	5679	6311	5994, 5875, 6184	10, 11, 6	8.16254, 7.43881, 5.99481	5.60312, 4.97059, 4.6644	5.53763, 4	3	NA	NA
GL178360.1	10	513	239, 250, 153	208, 246, 130	219.13231, 243.86433, 130.8521	25.3606, 22.69727, 21.28502	214.05222	3	NA	NA
GL178360.1	5628	6180	5946, 5918, 5945	617, 707, 634	721.37225, 839.12622, 781.13416	36.39866, 38.20278, 41.90601	715.216, 8	3	NA	NA
GL178362.1	26	500	192, 192, 190	430, 485, 343	446.94687, 500.65887, 365.66187	26.5691, 26.3694, 27.64578	441.43506	3	NA	NA
GL178362.1	5656	6255	6107, 6107, 6150	179, 200, 144	182.26476, 192.3791, 153.16174	23.04403, 20.78028, 24.31618	177.37109	3	NA	NA
GL178365.1	54	452	160, 160, 160	13, 21, 6	15.29169, 24.35402, 7.29403	9.29854, 12.71289, 5.30581	12.24396,	3	NA	NA
GL178381.1	332	1289	847, 836, 865	8, 20, 18	8.4095, 21.01248, 21.69448	5.97056, 11.03495, 11.95651	5.76336, 1	3	NA	NA
GL178385.1	4133	5005	4382, 4700, 4857	22, 26, 10	12.50649, 12.98341, 5.31805	5.66283, 5.20082, 3.8127	9.59324, 1	3	NA	NA
GL178393.1	4299	4703	4518, 4563, 4520	7, 7, 4	7.1696, 5.97725, 4.67325	5.31345, 4.62287, 3.78987	4.61046, 3	3	NA	NA
GL178398.1	5229	5910	5759, 4895, 5823, 5262, 5806	14, 15, 9, 11, 10	11.39624, 9.39146, 5.8464, 8.57182, 8.32728	6.80584, 5.37549, 4.29367, 5.68205, 5.70592	8.54691, 6	3	NA	NA
GL178401.1	182	856	739, 371, 625	4, 11, 7	3.36974, 10.10652, 8.69489	3.10444, 6.63228, 6.06379	1.44109, 7	3	NA	NA
GL178401.1	4366	5787	4808, 4797, 5442	17, 17, 5	19.39948, 16.3049, 5.9506	10.88538, 9.05325, 4.54784	16.19131,	3	NA	NA
GL178402.1	80	387	327, 168, 223	15, 16, 14	7.17415, 6.50013, 8.17828	4.14103, 3.66558, 4.86395	4.61429, 4	3	NA	NA
GL178402.1	567	1700	1609, 1603, 1619	54, 74, 47	45.39868, 60.21884, 43.618	12.53314, 12.88236, 13.99413	41.61391,	3	NA	NA
GL178402.1	4945	5453	5105, 5314, 5031	19, 29, 13	9.31316, 14.44778, 6.5838	4.62089, 5.34861, 4.11698	6.60161, 1	3	NA	NA
GL178404.1	1309	1823	1743, 1793, 1797	23, 31, 19	26.78552, 34.67556, 25.11547	13.52881, 15.30758, 13.59366	23.3553, 3	3	NA	NA
GL178406.1	2808	3771	3243, 3274, 3608	6, 13, 6	5.31708, 12.37541, 7.29403	4.2777, 7.62989, 5.30581	2.96727, 9	3	NA	NA
GL178413.1	134	2140	1166, 1156, 319	35, 41, 17	24.36092, 25.81265, 14.18863	8.40107, 7.71057, 7.765	20.99759,	3	NA	NA
GL178413.1	4775	5841	5197, 5293, 5329	36, 53, 31	25.96285, 40.35019, 28.69644	8.88405, 10.71824, 11.81523	22.55451,	3	NA	NA
GL178424.1	281	1290	782, 779, 772	80, 104, 66	69.48312, 86.66785, 60.75232	14.61894, 14.3417, 15.37462	65.39225,	3	NA	NA
GL178424.1	4249	5280	4745, 4747, 4751	42, 72, 32	26.44949, 49.81994, 20.99202	7.76067, 9.94775, 7.57258	23.02817,	3	NA	NA
GL178429.1	27	787	383, 351, 173	17, 17, 8	17.4139, 16.17826, 9.8421	9.72241, 8.97632, 6.68602	14.27855,	3	NA	NA
GL178429.1	4842	5805	5294, 5305, 5587	27, 33, 13	30.03323, 33.94907, 15.52427	14.07749, 14.08334, 9.42125	26.52489,	3	NA	NA
GL178458.1	3033	4166	3483, 3576, 3438	7, 11, 4	6.01305, 10.3713, 4.67325	4.6447, 6.79219, 3.78987	3.56347, 7	3	NA	NA
GL178459.1	4859	5288	5074, 5081, 4970	11, 10, 11	12.43693, 9.39128, 14.75772	7.97018, 6.35644, 9.09568	9.52692, 6	3	NA	NA
GL178463.1	5224	5561	5440, 5438, 5445	9, 13, 8	9.72082, 13.14514, 10.1465	6.64182, 8.09002, 6.82176	6.97155, 1	3	NA	NA
GL178467.1	4724	5015	4869, 4898, 4898	17, 19, 8	20.2407, 21.4153, 10.1465	11.35794, 11.55717, 6.82176	17.00287,	3	NA	NA
GL178471.1	164	724	312, 650, 551	6, 6, 7	5.96715, 4.4049, 6.83761	4.64927, 3.71155, 5.12939	3.52158, 2	3	NA	NA
GL178471.1	3362	4158	4014, 3643, 3734	7, 14, 12	7.1696, 13.26509, 13.29687	5.31345, 7.95332, 8.33527	4.61046, 1	3	NA	NA
GL178474.1	4476	5124	4726, 4836, 4763	8, 12, 7	7.77807, 11.86108, 8.69489	5.618, 7.51216, 6.06379	5.17956, 9	3	NA	NA
GL178475.1	2115	2867	2331, 2202, 2410	10, 16, 16	10.67246, 16.82622, 21.79539	7.09497, 9.62227, 12.29292	7.87096, 1	3	NA	NA
GL178485.1	168	5296	367, 3289, 698, 2468, 4027, 4597	30, 55, 17, 28, 27, 18	14.39215, 31.02752, 7.62787, 16.95163, 16.01907, 8.37945	5.18451, 7.14973, 4.08904, 6.5879, 6.36073, 4.31621	11.38681,	3	NA	NA
GL178485.1	833	5296	1631, 3289, 698, 2468, 4027, 4597	23, 55, 17, 28, 27, 18	9.11316, 31.02752, 7.62787, 16.95163, 16.01907, 8.37945	4.00805, 7.14973, 4.08904, 6.5879, 6.36073, 4.31621	6.41492, 2	3	NA	NA
GL178485.1	833	5296	2447, 3289, 698, 2468, 4027, 4597	40, 55, 17, 28, 27, 18	23.02114, 31.02752, 7.62787, 16.95163, 16.01907, 8.37945	6.84708, 7.14973, 4.08904, 6.5879, 6.36073, 4.31621	19.69623,	3	NA	NA
GL178492.1	105	742	489, 345, 438	26, 29, 26	14.19957, 15.83642, 15.14635	5.72621, 5.91457, 6.15415	11.2031, 1	3	NA	NA
GL178492.1	1937	3055	2313, 2364, 2349	34, 48, 35	21.63133, 34.39036, 23.92537	7.42286, 9.56409, 8.20553	18.34899,	3	NA	NA
GL178492.1	4252	5322	4866, 4901, 4674	38, 44, 46	25.6517, 30.14167, 35.94899	8.27119, 8.78335, 10.71278	22.25197,	3	NA	NA
GL178500.1	50	989	559, 601, 502	55, 69, 50	41.12154, 52.38647, 43.41349	10.53194, 11.35868, 12.87816	37.401, 48	3	NA	NA
GL178500.1	4952	5330	5010, 5003, 5103	51, 61, 27	36.79726, 43.54548, 17.91417	9.8114, 10.07448, 7.25934	33.14948,	3	NA	NA
GL178507.1	464	1384	717, 1238, 828	10, 6, 5	11.06011, 4.93312, 5.9506	7.306, 4.04501, 4.54784	8.22712, 2	3	NA	NA
GL178511.1	14	1072	447, 565, 491	48, 61, 37	51.56842, 60.47685, 42.11572	17.78983, 17.02839, 17.21295	47.69477,	3	NA	NA
GL178529.1	138	3041	1196, 1047, 1579, 2366	24, 37, 15, 8	17.77616, 27.69018, 12.34677, 5.2088	7.93656, 9.42258, 7.15213, 4.02307	14.62794,	3	NA	NA
GL178529.1	4234	5100	4744, 4728, 4851	18, 18, 17	11.65347, 9.17626, 14.65121	6.03179, 4.71129, 8.04615	8.79147, 6	3	NA	NA
GL178530.1	3198	5192	3964, 3756, 3720	12, 17, 15	13.55951, 18.56115, 18.13606	8.47903, 10.40145, 10.55788	10.59689,	3	NA	NA
GL178531.1	83	1175	1036, 1035, 1070	88, 111, 50	73.23243, 88.26308, 41.53198	13.86736, 13.36883, 12.02827	69.10483,	3	NA	NA
GL178543.1	3875	5149	4644, 4540, 4929	13, 12, 14	13.20994, 11.86108, 16.29325	8.12823, 7.51216, 9.7093	10.26285,	3	NA	NA
GL178547.1	128	1149	511, 449, 383	21, 27, 14	23.03475, 28.35229, 16.12551	11.93841, 13.07444, 9.61761	19.70924,	3	NA	NA
GL178556.1	1899	2569	2213, 2111, 2258	9, 5, 11	9.72082, 3.94561, 14.68355	6.64182, 3.46715, 9.06344	6.97155, 1	3	NA	NA
GL178561.1	4248	5126	4418, 4405, 4416	18, 28, 13	20.06618, 29.05602, 15.89249	11.03116, 13.12373, 9.61177	16.83343,	3	NA	NA
GL178568.1	200	796	554, 604, 580	22, 36, 21	11.62862, 21.20172, 13.78461	5.24113, 6.85699, 6.52606	8.76732, 1	3	NA	NA
GL178568.1	3488	4739	3976, 4031, 4013	65, 80, 40	59.20552, 70.56116, 35.15605	15.03976, 15.01126, 12.16221	55.2276, 6	3	NA	NA
GL178569.1	2931	3243	3094, 3094, 3098	603, 775, 459	780.71906, 994.29846, 624.20117	47.83368, 47.55581, 53.31013	774.38251	3	5S_rRNA	0
GL178575.1	36	1502	452, 436, 456	127, 200, 104	88.11578, 150.50856, 78.73884	10.87779, 13.03956, 12.18942	83.83924,	3	NA	NA
GL178575.1	3375	4246	3642, 3657, 3993	46, 49, 43	15.47053, 12.66008, 18.86017	3.99419, 3.24367, 5.10795	12.4163, 9	3	NA	NA
GL178583.1	710	1247	1118, 967, 837	7, 10, 6	5.7233, 6.8797, 6.35308	4.4663, 4.79317, 4.85498	3.33547, 4	3	NA	NA
GL178588.1	3296	3963	3530, 3531, 3849	16, 17, 8	16.15907, 15.81151, 9.92329	9.22513, 8.7532, 6.7228	13.07749,	3	NA	NA
GL178589.1	249	2354	538, 551, 1584	29, 43, 16	18.48986, 28.67809, 11.90154	7.08622, 8.42014, 6.63778	15.31331,	3	NA	NA
GL178589.1	3831	4962	4547, 4644, 4668	46, 55, 22	36.83169, 41.60406, 18.61246	11.10174, 10.71655, 8.98053	33.18337,	3	NA	NA
GL178598.1	33	2797	1498, 542, 530	35, 48, 28	26.54797, 35.13172, 22.78194	9.42218, 9.85918, 9.51725	23.12473,	3	NA	NA
GL178607.1	34	954	359, 706, 370	43, 72, 31	27.56765, 49.4331, 19.56832	7.99077, 9.83358, 7.1498	24.11804,	3	NA	NA
GL178607.1	4093	4936	4651, 4652, 4654	108, 160, 115	106.83146, 159.60066, 129.26678	19.79531, 21.68777, 25.91803	102.38645	3	NA	NA
GL178608.1	46	443	270, 248, 171	22, 36, 10	23.01801, 37.44002, 9.73055	11.61387, 14.9052, 6.55755	19.69328,	3	NA	NA
GL178616.1	4579	4915	4676, 4706, 4688	12, 16, 7	12.45843, 15.67391, 7.50542	7.86167, 8.93306, 5.49244	9.54648, 1	3	NA	NA
GL178626.1	242	886	717, 493, 501	8, 7, 7	8.42257, 5.97725, 8.69489	5.97763, 4.62287, 6.06379	5.76336, 3	3	hist1h2aj	870
GL178627.1	2105	3218	2962, 2604, 3080	22, 39, 19	22.85143, 36.49102, 21.34857	11.51387, 13.21183, 11.51831	19.53157,	3	NA	NA
GL178632.1	711	1067	746, 797, 855	11, 9, 5	12.17114, 8.21099, 5.69847	7.82679, 5.77858, 4.4331	9.28263, 5	3	NA	NA
GL178632.1	2180	2744	2444, 2444, 2444	13, 15, 13	14.97989, 15.80067, 17.40688	9.13126, 9.24574, 10.34389	11.94968,	3	NA	NA
GL178643.1	2167	2838	2553, 2514, 2438	21, 18, 8	27.77672, 19.97713, 10.1465	14.612, 10.97931, 6.82176	24.32202,	3	NA	NA
GL178652.1	349	1128	569, 580, 766	10, 13, 8	6.4661, 7.16117, 7.59222	4.53125, 4.44888, 5.5104	3.98462, 4	3	NA	NA
GL178654.1	83	346	240, 239, 254	24, 26, 20	31.90354, 32.02458, 28.28388	16.12493, 15.60218, 15.02535	28.35432,	3	NA	NA
GL178660.1	95	973	471, 414, 384	24, 31, 12	13.11573, 16.7837, 6.30172	5.58738, 5.96806, 4.10753	10.17342,	3	NA	NA
GL178660.1	4012	4751	4408, 4469, 4386	67, 91, 50	61.10138, 85.11834, 50.0049	15.19768, 17.15818, 16.11417	57.10041,	3	NA	NA
GL178662.1	2082	2777	2272, 2308, 2543	12, 25, 14	10.3005, 25.26956, 16.46576	6.56436, 11.95039, 9.80275	7.51999, 2	3	NA	NA
GL178662.1	3955	4635	4357, 4541, 4442	14, 11, 5	12.64394, 8.30667, 4.70718	7.57426, 5.51556, 3.9211	9.72408, 5	3	NA	NA
GL178664.1	909	1854	1111, 1414, 1544	8, 16, 6	7.67464, 15.94397, 7.29403	5.55832, 9.09593, 5.30581	5.08299, 1	3	NA	NA
GL178664.1	3099	4071	4040, 3679, 3655	8, 16, 4	7.67464, 15.94397, 4.67325	5.55832, 9.09593, 3.78987	5.08299, 1	3	NA	NA
GL178674.1	29	792	475, 412, 474	136, 146, 91	102.91058, 94.1227, 74.23882	12.64035, 9.85221, 13.49509	98.49529,	3	NA	NA
GL178674.1	2144	2929	2729, 2613, 2606	59, 101, 44	26.54154, 51.31274, 24.13326	5.53592, 6.83623, 6.60086	23.11842,	3	NA	NA
GL178674.1	3914	4615	4295, 4312, 4172	57, 76, 19	24.97556, 31.06095, 5.47994	5.35139, 5.16068, 2.93372	21.59506,	3	NA	NA
GL178689.1	4077	4575	4353, 4501, 4534	6, 11, 7	4.83561, 8.81665, 8.69489	3.98453, 5.83547, 6.06379	2.52606, 6	3	NA	NA
GL178699.1	4237	4644	4459, 4444, 4471	19, 25, 19	22.13159, 28.34259, 25.95226	11.97036, 13.78575, 14.01451	18.83405,	3	NA	NA
GL178701.1	3488	4026	3808, 3794, 3941	6, 8, 7	4.89903, 5.14361, 7.61369	4.02393, 3.9808, 5.54854	2.58474, 2	3	NA	NA
GL178707.1	2876	4554	3143, 3093, 4297	6, 26, 11	5.96715, 27.30311, 13.46208	4.64927, 12.81135, 8.49639	3.52158, 2	3	NA	NA
GL178707.1	2876	4554	4220, 3093, 4297	12, 26, 11	13.8483, 27.30311, 13.46208	8.63436, 12.81135, 8.49639	10.86694,	3	NA	NA
GL178723.1	3135	3787	3575, 3618, 3645	12, 17, 14	13.8483, 18.56115, 19.68329	8.63436, 10.40145, 11.3696	10.86694,	3	NA	NA
GL178738.1	56	312	91, 207, 188	7, 8, 7	7.1696, 7.07159, 8.69489	5.31345, 5.20073, 6.06379	4.61046, 4	3	NA	NA
GL178741.1	2745	3195	3003, 2856, 2823	5, 6, 3	4.82177, 4.93312, 3.47371	3.98509, 4.04501, 3.03189	2.51418, 2	3	NA	NA
GL178747.1	813	3999	1719, 1720, 1927, 3553	91, 83, 51, 18	74.59704, 62.05552, 42.07449, 8.49951	13.60556, 11.50012, 11.97301, 4.37475	70.45488,	3	NA	NA
GL178747.1	813	3999	3502, 1720, 1927, 3553	26, 83, 51, 18	9.90491, 62.05552, 42.07449, 8.49951	3.99293, 11.50012, 11.97301, 4.37475	7.14635, 5	3	NA	NA
GL178748.1	92	1849	598, 607, 860	30, 40, 19	31.37869, 39.60346, 20.70442	13.74365, 14.52291, 11.14035	27.83999,	3	NA	NA
GL178753.1	3507	4446	3764, 3942, 3774	66, 79, 28	69.4265, 83.34398, 31.5288	19.36654, 20.62112, 14.59685	65.3363, 7	3	NA	NA
GL178756.1	71	1758	907, 589, 907	67, 100, 40	59.48542, 90.33113, 37.5137	14.52754, 16.4344, 13.39071	55.50385,	3	NA	NA
GL178788.1	97	487	240, 233, 235	12, 16, 12	12.05017, 14.25068, 13.7434	7.62377, 8.0664, 8.57827	9.16715, 1	3	NA	NA
GL178788.1	3150	4025	3875, 3461, 3868	8, 11, 8	7.19971, 8.59076, 8.35103	5.27799, 5.69393, 5.93881	4.63758, 6	3	NA	NA
GL178798.1	247	981	703, 717, 730	14, 16, 11	16.7649, 17.16856, 14.75772	9.96273, 9.82359, 9.09568	13.6543, 1	3	NA	0
GL178801.1	1095	1632	1581, 1571, 1586	29, 26, 9	36.08673, 29.61081, 11.58206	16.91101, 14.1878, 7.55286	32.45203,	3	NA	NA
GL178810.1	3051	3468	3295, 3103, 3127	16, 25, 9	18.34375, 27.53425, 11.21577	10.49905, 13.30495, 7.38849	15.17198,	3	NA	NA
GL178833.1	77	830	419, 430, 416	12, 16, 5	13.8483, 17.16856, 5.9506	8.63436, 9.82359, 4.54784	10.86694,	3	NA	NA
GL178838.1	2925	3411	3061, 3061, 3061	10, 10, 8	11.06011, 9.39128, 10.1465	7.306, 6.35644, 6.82176	8.22712, 6	3	NA	NA
GL178839.1	2834	3576	2923, 3214, 3441	7, 6, 8	5.52352, 4.45754, 7.59222	4.34124, 3.74538, 5.5104	3.15454, 2	3	NA	1139
GL178840.1	200	3183	2625, 1591, 798, 1441	12, 17, 13, 10	9.01403, 12.63113, 12.34408, 8.77043	5.76346, 6.8293, 7.61094, 5.98002	6.32034, 9	3	NA	NA
GL178844.1	1907	2326	2020, 2016, 2023	25, 31, 20	28.88915, 36.00061, 24.55945	14.10903, 16.09466, 13.09374	25.40799,	3	NA	NA
GL178852.1	2130	4104	2841, 2554, 4057, 2601	29, 23, 8, 15	28.84313, 23.98856, 5.87115, 16.75372	12.62633, 11.86759, 4.45035, 9.79627	25.36306,	3	NA	NA
GL178879.1	1201	1549	1258, 1250, 1232	25, 31, 10	30.32096, 39.4343, 11.997	14.94603, 18.1125, 7.78835	26.80639,	3	NA	NA
GL178883.1	69	625	314, 274, 234	8, 17, 8	7.1122, 15.81151, 8.56955	5.22529, 8.7532, 6.05652	4.57946, 1	3	NA	NA
GL178886.1	2333	3947	3178, 3175, 2702	9, 9, 5	9.72082, 8.21099, 5.9506	6.64182, 5.77858, 4.54784	6.97155, 5	3	NA	NA
GL178892.1	110	1154	508, 520, 857	16, 26, 23	12.26603, 20.59654, 21.08456	6.85709, 8.91799, 10.12812	9.37251, 1	3	NA	NA
GL178892.1	2315	2791	2678, 2561, 2419	13, 13, 11	9.15772, 7.46083, 7.58795	5.64702, 4.62414, 5.06406	6.45542, 5	3	NA	NA
GL178910.1	88	956	525, 594, 637	16, 20, 13	13.57573, 14.75447, 12.76267	7.65356, 7.30627, 7.86275	10.61158,	3	NA	NA
GL178910.1	3045	3967	3318, 3683, 3670	18, 36, 15	15.9393, 33.83622, 15.36085	8.55398, 12.87296, 8.986	12.86597,	3	NA	NA
GL178924.1	1305	2376	1629, 1640, 1912	8, 15, 10	7.77807, 15.80067, 11.83521	5.618, 9.24574, 7.70798	5.17956, 1	3	NA	NA
GL178938.1	22	607	421, 324, 492	248, 377, 157	239.61116, 372.2106, 151.6268	21.5065, 23.40951, 20.24597	234.46884	3	NA	NA
GL178939.1	536	1123	1054, 1026, 1008	5, 7, 4	4.82177, 5.97725, 4.67325	3.98509, 4.62287, 3.78987	2.51418, 3	3	NA	NA
GL178953.1	65	924	673, 453, 332	16, 23, 14	19.7934, 26.6587, 19.30655	11.29109, 13.45462, 11.20466	16.57067,	3	NA	NA
GL178957.1	97	499	161, 294, 276	5, 9, 6	4.82177, 8.21099, 7.29403	3.98509, 5.77858, 5.30581	2.51418, 5	3	NA	NA
GL178959.1	3152	3894	3504, 3250, 3423	10, 11, 7	11.06011, 10.60895, 8.69489	7.306, 6.9343, 6.06379	8.22712, 7	3	NA	NA
GL178980.1	2565	3809	3547, 2710, 3213	7, 15, 9	7.1696, 15.80067, 11.39481	5.31345, 9.24574, 7.46977	4.61046, 1	3	NA	NA
GL178992.1	104	661	257, 281, 230	22, 31, 19	6.17425, 10.0015, 6.03978	2.98394, 3.59313, 3.15891	3.71356, 7	3	5_8S_rRNA	1826
GL178992.1	1030	1733	1312, 1325, 1496	50, 56, 45	26.88802, 28.79455, 26.56948	6.61656, 6.40026, 7.2655	23.45547,	3	5_8S_rRNA	754
GL178992.1	2510	2941	2566, 2628, 2769	31, 52, 23	11.78467, 25.40448, 8.56109	4.15157, 5.95111, 3.7907	8.91569, 2	3	5_8S_rRNA	0
GL178992.1	3208	3784	3673, 3691, 3637	54, 64, 50	30.49924, 35.9277, 31.39066	7.1355, 7.29854, 8.05523	26.98061,	3	5_8S_rRNA	566
GL178995.1	2276	3741	3144, 2842, 2547, 3620	10, 13, 3, 6	10.01299, 13.14514, 3.47371, 7.29403	6.7221, 8.09002, 3.03189, 5.30581	7.24763, 1	3	NA	NA
GL179009.1	149	1945	1491, 772, 835	50, 91, 51	43.30526, 79.86648, 51.68264	12.82835, 15.30951, 16.58448	39.55118,	3	NA	NA
GL179022.1	957	1216	1065, 987, 1013	10, 24, 7	11.06011, 28.90463, 8.69489	7.306, 14.44646, 6.06379	8.22712, 2	3	srp19	0
GL179023.1	169	2010	1278, 1299, 1316	157, 169, 103	150.40778, 153.50185, 97.75034	19.92049, 18.11792, 18.11153	145.68048	3	NA	NA
GL179023.1	2788	3722	3430, 3281, 2980	36, 48, 26	14.95146, 21.09891, 11.76506	4.66492, 5.22223, 4.70203	11.92311,	3	NA	NA
GL179024.1	607	3699	808, 1841, 858, 1510, 2451	9, 24, 9, 8, 14	4.80621, 18.19479, 6.26241, 5.33388, 11.45195	3.63058, 8.16549, 4.56014, 4.10413, 6.84022	2.51418, 1	3	NA	NA

GL179024.1	607	3699	2542, 1841, 858, 1510, 2451	21, 24, 9, 8, 14	16.40761, 18.19479, 6.26241, 5.33388, 11.45195	7.98727, 8.16549, 4.56014, 4.10413, 6.84022	13.31526,	3	NA	NA
GL179046.1	32	1315	635, 1000, 971	22, 25, 16	22.52709, 26.08339, 17.55092	11.31895, 12.43741, 10.04619	19.2175, 2	3	NA	NA
GL179050.1	3175	3659	3246, 3235, 3227	12, 12, 11	13.8483, 11.86108, 14.75772	8.63436, 7.51216, 9.09568	10.86694,	3	NA	NA
GL179053.1	197	847	673, 541, 308	7, 9, 4	7.1696, 8.21099, 4.67325	5.31345, 5.77858, 3.78987	4.61046, 5	3	NA	NA
GL179071.1	622	1073	686, 702, 659	15, 20, 10	16.03559, 22.87459, 12.90749	9.3831, 12.13503, 8.21675	12.95858,	3	NA	NA
GL179071.1	3109	3604	3421, 3265, 3409	15, 9, 6	16.03559, 8.21099, 7.12188	9.3831, 5.77858, 5.22884	12.95858,	3	NA	NA
GL179076.1	37	574	178, 188, 178	79, 127, 68	92.91256, 147.82773, 84.20516	25.25907, 28.59781, 26.18861	88.58554,	3	NA	NA
GL179085.1	891	1466	1159, 1320, 945	14, 15, 10	12.0338, 9.60764, 7.86471	7.19911, 5.50146, 5.41628	9.15254, 7	3	NA	NA
GL179103.1	1546	2449	1832, 1661, 1937	9, 10, 3	9.72082, 9.39128, 3.47371	6.64182, 6.35644, 3.03189	6.97155, 6	3	NA	NA
GL179106.1	2341	3333	2903, 3028, 2882	19, 24, 14	17.87921, 19.06042, 14.16595	9.44292, 8.64669, 8.49463	14.72728,	3	NA	NA
GL179113.1	841	1589	1190, 1246, 936	9, 8, 3	9.72082, 7.07159, 3.47371	6.64182, 5.20073, 3.03189	6.97155, 4	3	NA	NA
GL179115.1	111	1261	984, 955, 964	13, 27, 10	10.379, 26.2886, 9.73055	6.40208, 11.84892, 6.55755	7.59527, 2	3	NA	NA
GL179115.1	2602	3125	2809, 2825, 2841	10, 8, 6	7.25363, 4.87833, 5.14244	5.03021, 3.80858, 4.17298	4.68848, 2	3	NA	NA
GL179117.1	71	493	371, 286, 356	10, 14, 7	11.06011, 14.45897, 8.69489	7.306, 8.66788, 6.06379	8.22712, 1	3	NA	NA
GL179134.1	114	1608	1005, 892, 1353	27, 52, 23	21.53678, 44.78341, 21.2989	9.13844, 12.87015, 10.25559	18.25711,	3	NA	NA
GL179150.1	54	1738	1210, 1257, 884	24, 27, 19	23.82647, 22.83892, 18.90983	11.43229, 9.85858, 10.06679	20.47802,	3	U5	0
GL179159.1	1359	1869	1782, 1840, 1647	5, 8, 4	4.82177, 5.99047, 4.46488	3.98509, 4.52662, 3.69425	2.51418, 3	3	NA	NA
GL179165.1	159	1212	552, 906, 715	9, 18, 5	9.72082, 19.97713, 5.9506	6.64182, 10.97931, 4.54784	6.97155, 1	3	NA	NA
GL179179.1	2774	3445	2879, 2854, 2922	10, 20, 10	11.06011, 22.87459, 13.18184	7.306, 12.13503, 8.33771	8.22712, 1	3	NA	NA
GL179205.1	263	691	466, 561, 336	6, 11, 13	3.66682, 6.44901, 12.05005	3.22642, 4.35576, 7.43242	1.6222, 4.	3	NA	NA
GL179205.1	2237	2810	2536, 2553, 2646	8, 12, 5	5.40205, 7.32335, 3.5059	4.14825, 4.71874, 3.18532	3.04418, 4	3	NA	NA
GL179208.1	61	855	341, 315, 603	7, 25, 8	7.1696, 30.45643, 10.1465	5.31345, 15.02432, 6.82176	4.61046, 2	3	NA	NA
GL179237.1	82	823	622, 425, 594	21, 36, 15	23.61699, 41.26263, 20.69165	12.28232, 17.14056, 11.82159	20.27492,	3	NA	NA
GL179251.1	103	790	439, 273, 531	33, 37, 23	24.26377, 22.89968, 16.97324	8.85884, 7.34378, 7.76377	20.90313,	3	U1	0
GL179251.1	1879	3201	2733, 2735, 2114, 2804	27, 38, 14, 24	17.98787, 23.87144, 8.15827, 18.05228	7.29552, 7.53703, 4.85236, 8.08727	14.83119,	3	U1	0
GL179259.1	1805	3210	2645, 2302, 2709	17, 25, 13	16.4953, 23.28319, 14.25214	9.16875, 10.76961, 8.72916	13.39926,	3	U5	0
GL179279.1	2371	3267	2491, 2482, 2977, 2453, 3211	14, 21, 13, 9, 8	11.93818, 19.11862, 9.89237, 7.94299, 6.83213	7.14017, 9.58634, 6.1004, 5.61625, 5.05462	9.06106, 1	3	NA	NA
GL179281.1	186	3282	2576, 2473, 968, 2447	32, 42, 16, 18	24.61956, 30.40978, 11.90154, 14.05276	9.30737, 9.35772, 6.63778, 7.4187	21.24809,	3	NA	NA
GL179286.1	110	1106	384, 772, 468	46, 59, 31	41.81871, 49.65196, 30.2678	13.45172, 12.86645, 12.71398	38.08757,	3	U5	0
GL179286.1	2489	3318	2989, 2933, 2986	29, 34, 19	21.56369, 21.82322, 15.39325	8.58621, 7.50543, 7.94624	18.28302,	3	U5	0
GL179303.1	2353	3052	2722, 2678, 2742	8, 10, 6	8.42257, 9.39128, 7.29403	5.97763, 6.35644, 5.30581	5.76336, 6	3	NA	NA
GL179324.1	1681	2642	2047, 2242, 2149	8, 11, 8	8.42257, 10.60895, 10.1465	5.97763, 6.9343, 6.82176	5.76336, 7	3	NA	NA
GL179329.1	2193	3174	2366, 2639, 2610	12, 22, 12	13.8483, 24.36754, 14.58408	8.63436, 12.41949, 9.01638	10.86694,	3	NA	NA
GL179332.1	2282	3087	2642, 2556, 2286	6, 15, 4	5.96715, 15.80067, 4.67325	4.64927, 9.24574, 3.78987	3.52158, 1	3	NA	NA
GL179333.1	2285	2587	2540, 2550, 2559	9, 23, 14	9.72082, 27.36982, 19.68329	6.64182, 13.8686, 11.3696	6.97155, 2	3	NA	NA
GL179336.1	95	909	736, 460, 773	24, 29, 11	20.97936, 25.71373, 10.10247	9.74589, 10.82247, 6.62982	17.71833,	3	NA	NA
GL179336.1	2317	3183	2924, 2975, 2778	29, 28, 16	27.24398, 24.4842, 16.43863	11.69506, 10.46172, 9.39225	23.80142,	3	NA	NA
GL179342.1	2565	3171	2892, 3077, 2928	6, 9, 8	5.96715, 8.21099, 10.1465	4.64927, 5.77858, 6.82176	3.52158, 5	3	NA	NA
GL179347.1	78	671	328, 163, 246	6, 14, 5	4.32795, 9.88878, 4.07202	3.66187, 5.88118, 3.54571	2.1485, 7.	3	NA	224
GL179354.1	149	1157	874, 860, 897	11, 11, 4	12.43693, 10.60895, 4.67325	7.97018, 6.9343, 3.78987	9.52692, 7	3	NA	NA
GL179355.1	61	1215	605, 1055, 341	6, 9, 5	5.96715, 8.21099, 5.9506	4.64927, 5.77858, 4.54784	3.52158, 5	3	NA	NA
GL179356.1	2531	3169	2805, 2767, 2802	8, 8, 8	8.42257, 7.07159, 10.1465	5.97763, 5.20073, 6.82176	5.76336, 4	3	NA	NA
GL179370.1	102	802	664, 767, 659	10, 12, 5	11.06011, 11.86108, 5.9506	7.306, 7.51216, 4.54784	8.22712, 9	3	NA	NA
GL179373.1	877	1287	1045, 1096, 990	12, 13, 10	8.29737, 7.50529, 8.92941	5.31744, 4.65031, 6.07734	5.6641, 5.	3	NA	NA
GL179379.1	115	1233	879, 688, 733	32, 60, 37	34.2057, 60.62514, 42.11572	14.63033, 17.49039, 17.21295	30.60847,	3	NA	NA
GL179395.1	66	1360	908, 1001, 1002	14, 23, 8	16.6367, 23.66098, 10.1465	9.89449, 11.67093, 6.82176	13.53421,	3	NA	NA
GL179403.1	735	1124	1017, 987, 1011	14, 29, 6	16.7649, 34.13175, 7.29403	9.96273, 15.76422, 5.30581	13.6543, 3	3	NA	NA
GL179408.1	2922	3168	2989, 3118, 3027	6, 15, 5	5.96715, 15.80067, 5.9506	4.64927, 9.24574, 4.54784	3.52158, 1	3	rpp25l	2071
GL179423.1	380	934	531, 517, 515	189, 285, 181	56.54366, 116.42168, 71.60212	4.08206, 5.64845, 5.34538	52.60072,	3	NA	NA
GL179423.1	2124	2939	2502, 2503, 2595	1363, 1386, 977	1443.12695, 1427.16687, 1030.31824	29.30487, 27.39302, 28.72406	1433.9541	3	NA	NA
GL179429.1	76	1030	801, 785, 773	17, 20, 7	20.02116, 21.01248, 8.08824	11.236, 11.03495, 5.7849	16.79069,	3	NA	NA
GL179461.1	70	838	300, 334, 488	36, 57, 30	26.287, 39.84269, 26.47279	9.03327, 9.62436, 10.91198	22.86962,	3	NA	NA
GL179461.1	2028	2945	2304, 2635, 2607	34, 51, 23	24.14196, 33.58112, 18.19828	8.54498, 8.62873, 8.44798	20.78447,	3	NA	NA
GL179465.1	10	630	494, 80, 552	47, 57, 43	31.26799, 37.9039, 32.54828	8.58315, 8.96531, 10.02898	27.73151,	3	NA	NA
GL179465.1	2416	3017	2546, 2585, 2582	93, 103, 79	85.77796, 91.61932, 77.91912	16.80866, 16.07573, 18.23451	81.52176,	3	NA	NA
GL179475.1	1993	3103	2586, 2367, 2372	43, 68, 42	39.44706, 64.26581, 42.02309	13.31406, 16.21863, 15.05791	35.75402,	3	NA	NA
GL179478.1	23	445	202, 280, 208	21, 48, 17	16.23632, 40.35134, 14.56474	7.88875, 12.09906, 7.99353	13.15022,	3	NA	NA
GL179478.1	1939	2707	2128, 2129, 2115	39, 52, 34	38.56685, 45.2631, 37.12854	14.34318, 13.08673, 15.54297	34.88777,	3	NA	NA
GL179480.1	965	1536	1500, 1515, 1483	24, 39, 17	29.02281, 50.06581, 24.74975	14.51469, 21.01896, 13.59515	25.53849,	3	NA	NA
GL179494.1	31	619	313, 364, 346	98, 154, 64	59.28798, 95.05122, 39.26	8.58906, 9.27613, 8.20516	55.30935,	3	NA	NA
GL179494.1	2220	3066	2550, 2883, 2585	160, 223, 120	126.83616, 169.5983, 102.22211	13.96806, 13.4055, 15.27423	122.2417,	3	NA	NA
GL179505.1	2340	3074	2641, 2645, 2741	93, 111, 53	103.72465, 121.15841, 57.47651	24.06079, 24.19417, 18.81291	99.30309,	3	5S_rRNA	0
GL179519.1	640	1506	966, 1114, 675, 1467	13, 16, 5, 5	14.26982, 15.94397, 5.06966, 5.06966	8.73911, 9.09593, 4.11933, 4.11933	11.26966,	3	NA	NA
GL179519.1	2580	3057	2918, 2801, 2729	7, 11, 6	6.60038, 9.7598, 6.25896	4.99378, 6.42066, 4.80589	4.10761, 7	3	NA	NA
GL179524.1	581	1445	928, 1284, 821	9, 18, 11	9.72082, 19.59288, 13.64572	6.64182, 10.7543, 8.58592	6.97155, 1	3	NA	NA
GL179527.1	30	933	209, 206, 208	331, 434, 211	181.88359, 245.48077, 109.00339	8.22034, 8.64646, 7.40141	176.99088	3	NA	NA
GL179527.1	2577	3019	2707, 2689, 2688	605, 733, 495	475.07532, 565.25421, 420.24872	15.0046, 14.58966, 17.31651	469.44614	3	NA	NA
GL179529.1	2014	2988	2312, 2493, 2709	8, 13, 9	8.42257, 13.14514, 11.64359	5.97763, 8.09002, 7.57973	5.76336, 1	3	NA	NA
GL179532.1	62	789	233, 407, 227	52, 51, 27	42.23243, 40.04766, 21.62959	11.75488, 11.11033, 9.18907	38.49406,	3	NA	NA
GL179532.1	2248	3015	2910, 2499, 2548, 2884	30, 19, 15, 15	18.48883, 8.58391, 9.17616, 9.17616	6.87549, 4.2732, 5.2509, 5.2509	15.31231,	3	NA	NA
GL179537.1	85	538	477, 246, 347	6, 11, 6	5.55471, 9.22091, 6.45058	4.41698, 6.08767, 4.90508	3.18192, 6	3	NA	NA
GL179537.1	2110	2731	2158, 2172, 2221	8, 10, 6	7.88469, 8.12616, 6.45058	5.67897, 5.58036, 4.90508	5.27935, 5	3	NA	NA
GL179538.1	2038	2886	2733, 2817, 2731	21, 23, 18	17.71848, 17.10959, 16.41422	8.75244, 7.83887, 8.84243	14.5719, 1	3	NA	NA
GL179549.1	10	782	139, 105, 237	20, 33, 10	17.58375, 33.28279, 9.0947	8.96828, 13.69667, 6.17787	14.44221,	3	NA	NA
GL179557.1	369	766	655, 591, 676	12, 10, 7	11.09909, 7.8291, 8.21885	7.05451, 5.39387, 5.84717	8.26391, 5	3	NA	NA
GL179557.1	2253	2778	2511, 2516, 2583	12, 13, 7	11.09909, 11.12608, 8.21885	7.05451, 6.86492, 5.84717	8.26391, 8	3	NA	NA
GL179560.1	1822	2970	2350, 2767, 1754, 2793	18, 23, 10, 15	13.8676, 15.45851, 7.33926, 12.74323	7.30886, 6.94936, 5.08445, 7.39557	10.88502,	3	NA	NA
GL179566.1	10	682	249, 166, 166	24, 28, 13	27.61606, 29.4016, 15.52427	13.69419, 13.32928, 9.42125	24.16528,	3	NA	NA
GL179575.1	62	1389	338, 271, 258, 850	103, 130, 65, 28	68.96348, 80.80075, 48.54749, 12.69235	10.02515, 9.22218, 10.93305, 4.80391	64.87737,	3	NA	NA
GL179575.1	2369	2957	2699, 2704, 2672	100, 123, 52	65.83952, 73.89312, 34.56197	9.73596, 8.72939, 8.77957	61.78664,	3	NA	NA
GL179582.1	216	461	399, 374, 411	6, 11, 7	5.96715, 10.60895, 8.69489	4.64927, 6.9343, 6.06379	3.52158, 7	3	NA	NA
GL179603.1	335	668	617, 554, 633	10, 12, 6	11.06011, 11.86108, 7.29403	7.306, 7.51216, 5.30581	8.22712, 9	3	NA	NA
GL179604.1	95	770	283, 283, 283	906, 950, 690	791.9469, 805.9129, 615.22772	18.77865, 17.68135, 19.40509	785.58356	3	NA	NA
GL179604.1	2410	2932	2716, 2700, 2727	451, 503, 422	269.00681, 299.62042, 292.86325	9.35827, 9.37056, 11.87895	263.79105	3	NA	NA
GL179605.1	46	880	137, 526, 583	18, 25, 13	12.14277, 13.29315, 8.28203	6.3073, 5.49515, 5.11355	9.25603, 1	3	NA	NA
GL179605.1	1578	2913	2022, 2660, 2578	18, 29, 14	12.14277, 16.83244, 9.22157	6.3073, 6.34056, 5.4788	9.25603, 1	3	NA	NA
GL179606.1	970	1417	1308, 1209, 1099	9, 17, 13	9.72082, 18.56115, 17.16464	6.64182, 10.40145, 10.23255	6.97155, 1	3	NA	NA
GL179607.1	476	786	620, 601, 511	4, 9, 3	3.74195, 8.21099, 3.47371	3.32091, 5.77858, 3.03189	1.6222, 5.	3	NA	NA
GL179633.1	112	766	521, 543, 569	29, 37, 27	31.83288, 42.08796, 30.78397	14.39823, 17.19661, 14.52417	28.28486,	3	NA	NA
GL179634.1	259	735	589, 573, 638	12, 21, 15	9.45689, 16.6659, 15.61614	6.0398, 8.13653, 9.13715	6.73641, 1	3	NA	NA
GL179634.1	2298	2804	2472, 2503, 2478	28, 39, 10	29.64365, 39.37589, 9.26679	13.4734, 14.79368, 6.28179	26.14402,	3	NA	NA
GL179639.1	64	1147	413, 641, 633	38, 44, 43	44.15136, 48.16496, 56.54957	17.97576, 17.64353, 22.99939	40.38437,	3	NA	NA
GL179645.1	109	1553	850, 580, 1008	19, 16, 10	22.13159, 17.16856, 12.71002	11.97036, 9.82359, 8.12734	18.83405,	3	NA	NA
GL179646.1	41	912	473, 480, 471	44, 81, 28	42.1809, 79.30283, 26.56957	14.36424, 18.11361, 11.65848	38.44387,	3	NA	NA
GL179651.1	2033	2491	2073, 2164, 2259	7, 15, 4	7.1696, 14.52325, 4.67325	5.31345, 8.48354, 3.78987	4.61046, 1	3	NA	NA
GL179657.1	6	686	324, 326, 312	140, 181, 116	137.99873, 170.46361, 121.34694	20.69408, 19.64657, 22.44813	133.34036	3	NA	NA
GL179662.1	2143	2870	2684, 2652, 2233	12, 20, 11	11.2086, 18.84531, 11.20072	7.12098, 9.72843, 7.28154	8.36821, 1	3	NA	NA
GL179676.1	412	826	651, 648, 601	28, 32, 20	35.94532, 38.26429, 24.34577	17.18016, 17.03559, 12.9746	32.31287,	3	NA	NA
GL179680.1	82	829	650, 246, 727	7, 6, 5	7.1696, 4.93312, 5.9506	5.31345, 4.04501, 4.54784	4.61046, 2	3	NA	NA
GL179685.1	355	742	375, 661, 471	4, 6, 3	3.74195, 4.93312, 3.47371	3.32091, 4.04501, 3.03189	1.6222, 2.	3	NA	NA
GL179686.1	12	704	305, 342, 347	798, 966, 651	735.68585, 902.98059, 627.34644	20.94275, 21.71397, 22.90415	729.47583	3	NA	NA
GL179686.1	2167	2862	2347, 2310, 2280	212, 229, 153	86.65021, 86.77593, 61.75813	5.58298, 5.16465, 5.40988	82.38739,	3	NA	NA
GL179689.1	22	545	182, 187, 180	49, 68, 25	38.13269, 54.26218, 16.64747	10.84137, 12.24601, 7.09288	34.46122,	3	NA	NA
GL179689.1	2129	2752	2503, 2580, 2605	49, 49, 37	38.13269, 33.15442, 29.66502	10.84137, 8.87392, 10.36652	34.46122,	3	NA	NA
GL179693.1	12	720	137, 116, 281	95, 85, 70	91.03156, 69.26876, 68.4394	18.11335, 13.29339, 17.3478	86.72398,	3	NA	NA
GL179693.1	2110	2800	2417, 2401, 2494	28, 32, 23	14.37672, 14.88633, 13.3106	5.47174, 5.10095, 5.86404	11.37252,	3	NA	NA
GL179696.1	2213	2846	2672, 2548, 2487	10, 11, 7	11.06011, 10.60895, 8.69489	7.306, 6.9343, 6.06379	8.22712, 7	3	NA	NA
GL179705.1	455	2387	1958, 732, 1797, 1831	12, 10, 18, 23	11.32055, 7.55261, 16.69883, 27.70251	7.18871, 5.21944, 9.01539, 14.06048	8.47391, 5	3	NA	NA
GL179715.1	61	1127	288, 436, 424	16, 25, 13	17.95866, 26.78367, 17.16464	10.28064, 12.85656, 10.23255	14.80292,	3	NA	NA
GL179716.1	329	1449	866, 481, 1337	10, 14, 7	7.31658, 8.73234, 5.27202	5.07009, 5.18801, 4.18171	4.74811, 6	3	NA	NA
GL179720.1	68	665	331, 111, 392	4, 7, 8	3.51298, 5.48955, 10.1465	3.1896, 4.31982, 6.82176	1.55088, 3	3	NA	NA
GL179720.1	2229	2718	2511, 2382, 2519	10, 13, 6	10.53228, 12.25946, 7.29403	7.01712, 7.55969, 5.30581	7.73927, 9	3	NA	NA
GL179724.1	203	630	545, 297, 267	15, 22, 7	10.31603, 14.10074, 4.37639	5.91982, 6.46833, 3.59976	7.53526, 1	3	NA	NA
GL179724.1	2132	2737	2479, 2479, 2486	34, 49, 30	32.64215, 45.19452, 31.72573	12.94961, 14.06159, 13.94909	29.07687,	3	NA	NA
GL179735.1	112	785	406, 461, 666	29, 34, 18	22.69378, 22.47545, 14.4212	9.17325, 7.79006, 7.63831	19.3788, 1	3	NA	NA
GL179735.1	2105	2718	2446, 2322, 2368	26, 46, 15	19.37173, 35.36707, 11.16794	8.25593, 10.46094, 6.43227	16.16464,	3	NA	NA
GL179736.1	30	743	258, 194, 258	85, 80, 72	68.93913, 57.49173, 59.65558	13.18787, 10.7387, 13.18638	64.85336,	3	NA	NA
GL179736.1	2079	2805	2427, 2332, 2657	75, 98, 70	57.24672, 78.02147, 57.24749	11.65439, 13.12508, 12.82511	53.29384,	3	NA	NA
GL179744.1	29	1107	333, 528, 548	60, 70, 48	49.61878, 58.95184, 43.73351	12.57016, 13.45543, 13.69755	45.7714, 5	3	NA	NA
GL179744.1	1802	2659	2436, 2334, 2432	48, 51, 27	35.71973, 36.90995, 19.05062	10.09734, 9.85468, 7.82717	32.09206,	3	NA	NA
GL179748.1	70	511	207, 264, 288	68, 84, 53	50.99237, 61.47598, 45.99309	11.10834, 11.14315, 13.09982	47.12663,	3	NA	NA
GL179748.1	2212	2762	2461, 2564, 2564	77, 105, 41	61.36432, 85.84348, 31.70365	12.55725, 13.89617, 10.18875	57.36119,	3	NA	NA
GL179749.1	14	639	219, 236, 231	12, 20, 18	13.55951, 22.06591, 22.99911	8.47903, 11.6614, 12.66051	10.59689,	3	NA	NA
GL179751.1	61	694	623, 617, 393	11, 14, 12	12.43693, 14.02228, 13.59027	7.97018, 8.40914, 8.49571	9.52692, 1	3	NA	NA
GL179751.1	2082	2641	2268, 2444, 2158	6, 14, 7	5.96715, 14.02228, 7.01416	4.64927, 8.40914, 5.22813	3.52158, 1	3	NA	NA
GL179755.1	23	473	339, 353, 358	32, 50, 27	40.03331, 59.64252, 36.16593	18.07633, 21.42746, 17.62012	36.33001,	3	NA	758
GL179758.1	128	639	429, 501, 466	35, 27, 30	29.82121, 17.76388, 25.68707	11.0661, 7.18583, 10.47745	26.31792,	3	NA	NA
GL179758.1	1768	2710	2543, 2551, 2045, 2610	30, 35, 17, 16	23.92527, 26.16542, 11.35448, 10.38845	9.52914, 9.23893, 6.08368, 5.7457	20.57401,	3	NA	NA
GL179760.1	33	722	631, 526, 505	86, 104, 47	61.71738, 72.36315, 34.88139	10.85122, 10.64903, 10.00073	57.71116,	3	NA	NA
GL179760.1	2311	2762	2491, 2458, 2476	139, 173, 75	125.68873, 154.77921, 68.84311	17.46173, 17.64696, 15.83449	121.1007,	3	NA	NA
GL179770.1	179	2703	2386, 2162, 1481	21, 19, 16	22.66679, 18.75575, 17.40151	11.71964, 9.97369, 9.95951	19.35329,	3	NA	NA
GL179778.1	15	340	173, 193, 211	30, 41, 14	34.14409, 49.21646, 17.61756	15.38622, 19.61165, 10.40352	30.54789,	3	NA	NA
GL179785.1	70	926	793, 793, 790	19, 26, 14	18.13647, 23.54286, 13.81849	9.59882, 10.58612, 8.28728	14.97405,	3	NA	NA
GL179794.1	37	749	173, 200, 190	90, 83, 50	76.75781, 61.78284, 38.48125	14.52943, 11.42021, 10.7245	72.5932, 5	3	NA	NA
GL179794.1	2105	2676	2341, 2299, 2317	44, 67, 42	25.85489, 44.49901, 29.66291	7.18488, 9.24493, 9.04222	22.44983,	3	NA	NA
GL179795.1	64	635	293, 367, 492	39, 37, 31	29.50134, 25.62724, 25.07803	9.72486, 8.48997, 9.83623	26.00559,	3	NA	1585
GL179795.1	2134	2713	2457, 2471, 2293	43, 50, 26	34.04462, 40.07597, 19.45323	10.69735, 11.39444, 8.29932	30.45029,	3	NA	0
GL179799.1	1933	2683	2231, 2294, 1976	15, 26, 9	14.46121, 24.69991, 10.28145	8.44598, 11.26332, 6.93568	11.45219,	3	NA	NA
GL179803.1	1957	2714	2144, 2267, 2240	27, 54, 29	31.44513, 62.78402, 35.74224	14.9159, 21.28, 16.71053	27.90458,	3	NA	NA
GL179804.1	2070	2697	2576, 2595, 2490	14, 6, 9	16.7649, 4.93312, 11.64359	9.96273, 4.04501, 7.57973	13.6543, 2	3	NA	NA
GL179809.1	23	951	419, 396, 322	86, 103, 93	76.43069, 93.11365, 91.2483	15.46184, 16.55536, 18.83351	72.26913,	3	NA	NA
GL179809.1	2124	2654	2500, 2484, 2359	34, 35, 24	18.67944, 17.77727, 11.82477	6.22028, 5.7307, 5.00891	15.49572,	3	NA	NA
GL179826.1	1622	2153	1787, 1825, 2010	6, 6, 7	5.96715, 4.93312, 8.69489	4.64927, 4.04501, 6.06379	3.52158, 2	3	NA	NA
GL179832.1	48	2518	197, 1813, 1809	37, 83, 17	31.01431, 77.77656, 14.39501	11.03889, 16.84371, 7.89032	27.48421,	3	NA	NA
GL179837.1	27	684	330, 249, 230	16, 21, 26	13.4795, 19.48223, 28.94963	7.59475, 9.80473, 13.79468	10.52056,	3	NA	NA
GL179837.1	2252	2673	2510, 2549, 2525	32, 24, 18	34.39614, 23.34068, 17.83988	14.74275, 11.14174, 9.70737	30.79481,	3	NA	NA
GL179839.1	118	600	220, 226, 245	24, 40, 17	14.48064, 30.96058, 10.29033	6.23446, 10.1237, 5.48323	11.47121,	3	NA	NA
GL179839.1	2130	2607	2349, 2389, 2276	76, 70, 58	77.60275, 68.85111, 59.96768	19.20214, 17.53128, 17.97282	73.43098,	3	NA	NA
GL179840.1	9	711	275, 207, 281	18, 15, 15	17.65252, 13.58442, 16.30311	9.59407, 7.91173, 9.53825	14.50784,	3	NA	NA
GL179840.1	1908	2629	2392, 2217, 2130	15, 19, 7	13.8583, 18.6168, 6.21898	8.07921, 9.88966, 4.76912	10.87599,	3	NA	NA
GL179842.1	531	1214	705, 1069, 904	9, 10, 6	9.00096, 9.39128, 6.6567	6.24222, 6.35644, 5.00845	6.30808, 6	3	NA	NA
GL179847.1	2027	2404	2157, 2147, 2174	8, 6, 10	8.42257, 4.93312, 13.18184	5.97763, 4.04501, 8.33771	5.76336, 2	3	NA	NA
GL179848.1	1	561	95, 168, 219	17, 13, 9	21.34551, 13.14514, 11.64359	11.95527, 8.09002, 7.57973	18.07159,	3	NA	NA
GL179859.1	520	810	636, 621, 644	6, 12, 5	5.96715, 11.86108, 5.9506	4.64927, 7.51216, 4.54784	3.52158, 9	3	NA	NA
GL179863.1	67	536	232, 178, 260	22, 38, 19	6.92994, 17.90396, 6.75397	3.2604, 5.37697, 3.45639	4.4137, 14	3	NA	NA
GL179863.1	1797	2607	2167, 2162, 2163	134, 124, 121	127.46036, 112.456, 122.09512	19.13715, 17.23388, 21.08399	122.86205	3	NA	NA
GL179866.1	9	666	321, 248, 514	4, 13, 6	3.74195, 13.14514, 7.29403	3.32091, 8.09002, 5.30581	1.6222, 10	3	NA	NA
GL179867.1	101	1238	972, 617, 962	50, 57, 18	56.69952, 65.92119, 22.10559	19.74919, 21.58687, 12.18231	52.75434,	3	NA	NA
GL179869.1	1797	2599	2331, 2322, 2379	45, 71, 28	48.62545, 73.03563, 29.52789	17.46404, 19.0613, 13.40446	44.79249,	3	NA	NA
GL179871.1	46	954	194, 453, 196	102, 118, 64	68.1427, 65.81306, 43.18017	9.97839, 7.84128, 9.3726	64.06575,	3	NA	NA
GL179871.1	2001	2574	2228, 2183, 2222	95, 135, 53	60.92691, 82.06085, 32.12626	9.30024, 8.96147, 7.78646	56.92856,	3	NA	NA
GL179896.1	456	1246	666, 1095, 1025	7, 12, 5	7.1696, 11.86108, 5.9506	5.31345, 7.51216, 4.54784	4.61046, 9	3	NA	NA
GL179901.1	597	1039	626, 623, 608	20, 36, 16	26.13767, 47.04663, 23.11042	13.94781, 20.54628, 12.88555	22.72398,	3	NA	NA
GL179903.1	1715	2481	2018, 2069, 2069	26, 32, 12	26.36401, 30.15594, 13.44151	12.25052, 12.28219, 8.41472	22.94448,	3	NA	NA
GL179911.1	1483	2386	2126, 2096, 2261	13, 9, 11	15.29169, 8.21099, 13.64572	9.29854, 5.77858, 8.58592	12.24396,	3	NA	NA
GL179917.1	84	711	416, 211, 558	55, 47, 32	39.90409, 30.95724, 22.24687	10.07565, 8.46775, 8.15105	36.20319,	3	NA	NA
GL179917.1	1701	2468	2198, 2081, 1992	49, 51, 28	33.48348, 35.09666, 18.18542	8.99611, 9.1734, 7.16305	29.90057,	3	NA	NA
GL179928.1	537	949	776, 779, 763	32, 49, 27	38.64939, 54.93675, 34.76451	17.26311, 19.21876, 16.83978	34.96902,	3	NA	120
GL179929.1	55	2466	1822, 1832, 1792	159, 196, 116	124.91308, 162.11739, 100.10778	13.7378, 15.42238, 15.54982	120.33065	3	NA	NA
GL179931.1	83	2349	1709, 2029, 1425	69, 82, 58	61.2417, 67.53054, 53.71929	14.63222, 13.43102, 14.95388	57.23973,	3	NA	NA
GL179935.1	33	935	737, 779, 723	20, 27, 11	15.55865, 20.07195, 8.54035	7.76977, 8.35491, 5.66231	12.50042,	3	NA	NA
GL179935.1	1725	2400	2178, 2039, 2166	14, 15, 10	9.3401, 8.33674, 7.50676	5.54983, 4.77424, 5.19045	6.62699, 5	3	NA	NA
GL179942.1	25	732	448, 423, 452	103, 107, 81	79.50742, 76.61721, 70.44902	12.56717, 11.18523, 14.69482	75.31521,	3	NA	NA
GL179942.1	1760	2450	2072, 1992, 1928	72, 109, 39	45.84903, 78.82211, 23.41415	8.82119, 11.39236, 7.16821	42.05748,	3	NA	NA
GL179944.1	38	759	611, 611, 614	28, 40, 24	26.22613, 37.51501, 23.90188	11.45889, 13.3914, 11.47744	22.80972,	3	NA	NA
GL179944.1	1444	2291	1594, 1988, 1545	11, 15, 13	7.0723, 9.13279, 10.41976	4.74161, 5.22591, 6.42737	4.54399, 6	3	NA	NA
GL179946.1	96	782	274, 271, 327	10, 9, 6	11.06011, 8.21099, 7.29403	7.306, 5.77858, 5.30581	8.22712, 5	3	NA	NA
GL179947.1	187	746	618, 632, 318	10, 16, 9	11.06011, 17.16856, 11.04428	7.306, 9.82359, 7.30896	8.22712, 1	3	NA	NA
GL179948.1	225	718	549, 606, 691	6, 8, 4	5.96715, 7.07159, 4.67325	4.64927, 5.20073, 3.78987	3.52158, 4	3	NA	NA
GL179953.1	308	951	390, 560, 673	5, 8, 8	4.82177, 7.07159, 9.46129	3.98509, 5.20073, 6.50801	2.51418, 4	3	NA	NA
GL179963.1	27	665	131, 354, 581	10, 16, 6	7.64746, 10.92524, 5.91225	5.27935, 6.05826, 4.61907	5.05779, 8	3	NA	NA
GL179963.1	1654	2377	2210, 2098, 1939	11, 15, 7	8.69501, 9.94872, 7.10639	5.75929, 5.70189, 5.27894	6.02078, 7	3	NA	NA
GL179964.1	112	594	179, 209, 184	17, 14, 15	12.41673, 9.82448, 14.43201	6.70245, 5.84215, 8.4283	9.51585, 7	3	NA	NA
GL179964.1	1377	2380	1872, 1861, 1851	34, 30, 17	32.78921, 28.52218, 17.01335	13.03255, 12.07378, 9.48184	29.22015,	3	NA	NA
GL179986.1	170	2189	1620, 1577, 1572	13, 17, 14	11.98086, 15.46309, 14.53512	7.39024, 8.5409, 8.71263	9.10256, 1	3	NA	NA
GL179988.1	125	802	299, 279, 730	14, 18, 14	8.13906, 9.54163, 9.47385	4.84124, 4.89752, 5.63023	5.51652, 6	3	NA	NA
GL179988.1	1518	2332	2177, 2003, 1564, 2197	27, 37, 12, 20	21.35017, 28.48169, 7.58695, 15.75181	9.03698, 9.79503, 4.87954, 7.88233	18.07615,	3	NA	NA
GL179989.1	61	777	627, 269, 408	10, 12, 8	9.89241, 10.16897, 10.1465	6.65218, 6.4829, 6.82176	7.13431, 7	3	NA	NA
GL179989.1	1353	2345	2179, 2081, 1696	13, 22, 7	13.79066, 22.77797, 8.69489	8.46641, 11.46975, 6.06379	10.8171, 1	3	NA	NA
GL179996.1	1665	2294	1818, 1928, 1925	12, 18, 16	13.8483, 19.97713, 22.07892	8.63436, 10.97931, 12.42524	10.86694,	3	NA	NA
GL180007.1	19	244	107, 131, 127	15, 19, 13	18.26603, 21.4153, 18.01082	10.62691, 11.55717, 10.61163	15.09729,	3	NA	NA
GL180013.1	54	924	731, 202, 653	10, 10, 6	11.06011, 9.39128, 7.29403	7.306, 6.35644, 5.30581	8.22712, 6	3	NA	NA
GL180039.1	162	2170	967, 1571, 986	24, 34, 12	23.82647, 30.86867, 13.01912	11.43229, 11.96435, 8.18077	20.47802,	3	NA	NA
GL180040.1	13	585	329, 335, 139	704, 862, 547	508.83075, 622.40552, 405.34451	12.95825, 12.99816, 13.44331	503.12399	3	NA	NA
GL180040.1	1682	2203	1949, 2026, 2026	693, 805, 524	496.53748, 559.35706, 379.34259	12.75607, 12.13965, 12.87908	490.84988	3	NA	NA
GL180041.1	236	2194	1768, 1432, 1483	32, 38, 25	31.48135, 36.02841, 27.66765	13.03943, 13.3317, 13.38448	27.94013,	3	NA	NA
GL180048.1	87	652	590, 295, 344	11, 17, 7	10.5483, 16.17826, 7.50542	6.89817, 8.97632, 5.49244	7.75329, 1	3	NA	NA
GL180048.1	1552	2038	1647, 1961, 1707	10, 10, 7	9.33595, 7.97488, 7.50542	6.32332, 5.48553, 5.49244	6.62299, 5	3	NA	NA
GL180050.1	72	2096	1458, 1161, 914	15, 14, 17	17.48779, 14.45897, 19.81591	10.20672, 8.66788, 11.12108	14.34959,	3	NA	NA
GL180055.1	1030	2167	1982, 1690, 1236, 2042	8, 16, 10, 11	8.42257, 17.16856, 11.38247, 12.78973	5.97763, 9.82359, 7.47651, 8.15619	5.76336, 1	3	NA	NA
GL180056.1	98	1614	1393, 622, 743	26, 29, 18	26.20487, 29.43697, 20.75291	12.15566, 12.97595, 11.42699	22.78872,	3	NA	NA
GL180064.1	8	569	348, 106, 148	7, 14, 11	7.1696, 14.45897, 14.75772	5.31345, 8.66788, 9.09568	4.61046, 1	3	U2	1484
GL180066.1	1266	2066	1652, 1808, 1677	11, 15, 11	12.43693, 15.80067, 14.45778	7.97018, 9.24574, 8.96373	9.52692, 1	3	NA	NA
GL180079.1	54	2037	1046, 1395, 1316	31, 39, 16	30.75727, 36.6304, 16.31163	12.99764, 13.28661, 9.31645	27.23282,	3	NA	NA
GL180093.1	41	562	535, 525, 522	41, 35, 24	56.14899, 46.74359, 35.36946	23.67541, 20.77732, 17.88733	52.21095,	3	NA	NA
GL180097.1	96	1978	1406, 876, 1396	63, 97, 50	58.57842, 94.12665, 50.14982	15.43072, 18.63245, 16.1895	54.60809,	3	NA	NA
GL180101.1	1322	1849	1656, 1351, 1653	6, 6, 7	5.96715, 4.93312, 8.69489	4.64927, 4.04501, 6.06379	3.52158, 2	3	NA	NA
GL180111.1	45	1432	813, 829, 762	13, 12, 11	14.97989, 11.86108, 13.64572	9.13126, 7.51216, 8.58592	11.94968,	3	NA	NA
GL180112.1	56	2047	1196, 936, 1943	39, 39, 27	36.47481, 32.47705, 24.8312	13.20315, 11.14249, 10.99605	32.83307,	3	NA	NA
GL180117.1	222	1933	1670, 1681, 556, 1699	114, 148, 21, 64	114.85107, 152.21513, 12.7876, 64.37895	20.75529, 22.79147, 6.00167, 17.73221	110.34105	3	NA	NA
GL180118.1	195	1999	960, 573, 562	74, 99, 54	74.43669, 98.91235, 55.57751	18.54429, 19.78169, 17.37803	70.296, 94	3	NA	NA
GL180133.1	120	641	194, 282, 456	11, 17, 7	12.43693, 17.71433, 8.69489	7.97018, 9.9018, 6.06379	9.52692, 1	3	NA	NA
GL180137.1	2161	2761	2443, 2503, 2452	12, 16, 7	13.8483, 17.16856, 8.69489	8.63436, 9.82359, 6.06379	10.86694,	3	NA	NA
GL180137.1	10058	10656	10302, 10444, 10551	10, 12, 8	10.81731, 11.84549, 10.1465	7.17456, 7.50292, 6.82176	8.00764, 9	3	NA	NA
GL180137.1	202236	202970	202635, 202276, 202665	15, 13, 6	13.10246, 8.02497, 5.20372	7.61619, 4.95904, 4.20995	10.16066,	3	NA	NA
GL180137.1	203773	204833	204355, 204334, 204271	25, 50, 20	25.98133, 53.66526, 23.73483	12.3763, 18.06508, 12.62984	22.57216,	3	NA	NA
GL180137.1	216745	217390	216935, 216913, 216876	12, 10, 11	13.8483, 9.39128, 13.83651	8.63436, 6.35644, 8.67735	10.86694,	3	NA	NA
GL180137.1	306761	308071	307820, 307692, 307433	21, 37, 23	17.92389, 33.65004, 24.49362	8.87388, 12.41154, 12.17036	14.76984,	3	NA	NA
GL180137.1	345477	346027	345866, 345681, 345690	18, 15, 11	20.25794, 15.80067, 14.75772	11.14243, 9.24574, 9.09568	17.01944,	3	NA	NA
GL180138.1	146566	147307	146838, 146966, 146977	9, 18, 8	9.72082, 19.97713, 10.1465	6.64182, 10.97931, 6.82176	6.97155, 1	3	NA	NA
GL180138.1	152493	153375	152667, 152841, 152691	8, 14, 5	6.78332, 11.45303, 5.31857	5.02459, 6.84088, 4.24819	4.27811, 8	3	NA	NA
GL180138.1	171199	171717	171359, 171491, 171490	14, 25, 19	12.43323, 22.59781, 19.68826	7.44494, 10.36719, 10.53537	9.52692, 1	3	NA	NA

GL180138.1	200507	201229	201066, 201048, 200706, 201046	15, 18, 7, 8	17.6946, 19.95398, 8.69489, 10.1465	10.31995, 10.96581, 6.06379, 6.82176	14.54864,	3	NA	NA
GL180138.1	207301	207651	207474, 207382, 207479	6, 9, 5	5.96715, 8.21099, 5.9506	4.64927, 5.77858, 4.54784	3.52158, 5	3	NA	NA
GL180139.1	12844	13547	13443, 13259, 13286	15, 16, 7	14.98937, 14.46749, 7.66926	8.76428, 8.19898, 5.57703	11.95813,	3	NA	NA
GL180139.1	56239	57236	56901, 56526, 56840	27, 17, 13	27.08428, 11.82689, 13.33541	12.31967, 6.35673, 8.20195	23.64654,	3	NA	NA
GL180139.1	62447	63524	63340, 63405, 62664, 63421	10, 23, 5, 9	10.26482, 24.86003, 5.50079, 10.56949	6.86644, 12.38952, 4.33868, 7.08032	7.48624, 2	3	NA	NA
GL180141.1	13090	14767	13452, 13453, 13464	43, 63, 24	45.54248, 61.25417, 26.55881	16.59636, 16.64309, 13.06784	41.75543,	3	NA	NA
GL180141.1	29315	30506	30174, 30172, 30230	69, 82, 40	77.26053, 84.75232, 44.1376	21.98231, 20.07208, 17.08977	73.09189,	3	NA	NA
GL180141.1	38475	38976	38710, 38851, 38830	21, 24, 8	23.41865, 24.70533, 9.68495	12.1655, 11.95904, 6.61366	20.08217,	3	NA	NA
GL180141.1	44335	44712	44655, 44672, 44540	8, 8, 7	5.30204, 4.12105, 5.82684	4.08351, 3.31812, 4.53047	2.95557, 2	3	NA	NA
GL180141.1	50584	51260	50852, 50841, 50920	15, 33, 15	9.15146, 19.61597, 10.34972	5.23666, 6.77142, 5.93991	6.44964, 1	3	NA	NA
GL180141.1	90694	91356	90811, 91213, 90977	6, 11, 3	5.31708, 10.60895, 3.47371	4.2777, 6.9343, 3.03189	2.96727, 7	3	NA	NA
GL180141.1	98122	98796	98548, 98456, 98211	7, 14, 5	7.1696, 14.45897, 5.9506	5.31345, 8.66788, 4.54784	4.61046, 1	3	NA	NA
GL180141.1	125301	125696	125422, 125389, 125479	6, 9, 4	5.96715, 8.21099, 4.67325	4.64927, 5.77858, 3.78987	3.52158, 5	3	NA	NA
GL180142.1	119521	120059	119675, 119769, 119589	8, 10, 4	8.42257, 9.39128, 4.67325	5.97763, 6.35644, 3.78987	5.76336, 6	3	NA	NA
GL180143.1	30088	30418	30132, 30132, 30259	9, 14, 11	9.72082, 14.45897, 14.75772	6.64182, 8.66788, 9.09568	6.97155, 1	3	NA	1071
GL180144.1	17557	18076	17606, 17613, 17883	7, 11, 6	5.10557, 7.63964, 6.35308	4.075, 5.09648, 4.85498	2.77482, 5	3	NA	NA
GL180144.1	134913	136099	135584, 135577, 135679	6, 12, 6	5.96715, 11.04188, 7.29403	4.64927, 7.01971, 5.30581	3.52158, 8	3	NA	NA
GL180145.1	75783	76964	75929, 76727, 76220, 76823	8, 8, 7, 4	7.88469, 5.75617, 7.61369, 4.01199	5.67897, 4.3766, 5.54854, 3.46784	5.27935, 3	3	NA	NA
GL180153.1	71469	72408	71770, 71813, 71490, 72298	7, 11, 11, 6	7.1696, 10.60895, 14.24187, 6.99807	5.31345, 6.9343, 8.86619, 5.17195	4.61046, 7	3	xtr-mir-427-3	0
GL180153.1	115433	116303	116122, 115979, 115681	15, 21, 11	5.99608, 8.69164, 5.63779	3.53516, 4.0643, 3.86348	3.54878, 6	3	xtr-mir-427-3	0
GL180160.1	23014	23577	23228, 23172, 23455	35, 43, 27	38.43927, 44.42162, 28.73781	15.89974, 15.97112, 13.30451	34.76283,	3	NA	17362
GL180160.1	31769	32377	32342, 32200, 31951	44, 68, 36	23.54988, 41.37903, 22.32084	6.41031, 8.20279, 7.30541	20.20969,	3	NA	8562
GL180160.1	33947	35999	34647, 34694, 34717	86, 98, 64	67.58973, 74.5273, 52.92333	12.54698, 12.16342, 12.77809	63.51862,	3	NA	4940
GL180160.1	73199	74435	74159, 73580, 74157	20, 32, 23	16.65181, 29.81645, 25.12075	8.41216, 12.09051, 12.54512	13.54908,	3	U4	20328
GL180160.1	87504	88242	87728, 87749, 87741	36, 43, 32	40.01211, 47.53194, 39.5574	16.40371, 17.7251, 17.79758	36.30939,	3	U4	34633
GL180160.1	99583	100322	99971, 99982, 100086	34, 35, 30	36.59077, 34.56795, 34.90543	15.22674, 13.6586, 15.83834	32.94682,	3	U4	46712
GL180160.1	112054	113302	112517, 112903, 112877	118, 117, 90	120.69195, 119.73625, 94.88988	21.54409, 21.52836, 21.38792	116.13721	3	U4	59183
GL180172.1	118777	119456	118967, 119222, 119216	12, 11, 5	13.8483, 10.60895, 5.9506	8.63436, 6.9343, 4.54784	10.86694,	3	NA	NA
GL180174.1	83838	85211	84286, 84203, 84648	25, 34, 16	31.11602, 35.88116, 20.763	15.4029, 14.81115, 11.79066	27.58298,	3	NA	NA
GL180175.1	29113	29688	29507, 29452, 29444	13, 13, 8	15.29169, 13.14514, 10.1465	9.29854, 8.09002, 6.82176	12.24396,	3	NA	NA
GL180178.1	6435	9838	6505, 7268, 9155	11, 35, 20	3.87898, 20.11583, 13.33898	2.85187, 6.60906, 6.514	1.74823, 1	3	NA	0
GL180178.1	6435	9842	7215, 7268, 9155	21, 35, 20	11.33146, 20.11583, 13.33898	5.27152, 6.60906, 6.514	8.48479, 1	3	NA	0
GL180178.1	26349	27170	26781, 26837, 26784	21, 25, 16	25.88765, 28.13462, 22.07892	13.58701, 13.66232, 12.42524	22.48219,	3	NA	0
GL180178.1	32129	34533	33538, 33927, 33230	22, 37, 29	16.82955, 28.84821, 27.16018	7.95302, 9.97019, 11.64676	13.71684,	3	NA	0
GL180178.1	43092	44444	43840, 43389, 43869	25, 23, 18	27.43165, 23.82337, 21.89732	13.24375, 11.76844, 12.06835	23.98467,	3	NA	0
GL180178.1	74017	74433	74270, 74310, 74258	12, 17, 8	9.6142, 14.12865, 8.14528	6.1379, 7.72861, 5.82558	6.88539, 1	3	NA	0
GL180178.1	105613	106442	105832, 106113, 106363	8, 15, 6	8.42257, 15.80067, 7.29403	5.97763, 9.24574, 5.30581	5.76336, 1	3	NA	23942
GL180179.1	19	1191	325, 335, 626	15, 26, 14	17.09296, 27.47779, 16.82614	9.98755, 12.91577, 9.99514	13.96978,	3	NA	NA
GL180179.1	37756	38590	38331, 38363, 38435	14, 19, 7	11.61573, 14.1866, 6.43637	6.94127, 7.23722, 4.89813	8.75458, 1	3	NA	NA
GL180179.1	41836	42649	41966, 42234, 41997, 42525	8, 15, 7, 7	5.25324, 9.0327, 5.53602, 5.53602	4.05189, 5.16839, 4.34911, 4.34911	2.90961, 6	3	NA	NA
GL180179.1	41843	42649	42302, 42234, 41997, 42525	6, 15, 7, 7	3.55483, 9.0327, 5.53602, 5.53602	3.15147, 5.16839, 4.34911, 4.34911	1.58706, 6	3	NA	NA
GL180179.1	43005	44834	43539, 43415, 44419	8, 21, 9	6.66791, 16.71027, 8.88966	4.95324, 8.16224, 6.17838	4.17132, 1	3	NA	NA
GL180179.1	49904	50912	50782, 50033, 50007	8, 14, 9	8.42257, 14.45897, 10.28145	5.97763, 8.66788, 6.93568	5.76336, 1	3	NA	NA
GL180179.1	60185	60828	60712, 60622, 60331	8, 17, 5	7.57423, 15.93163, 5.64747	5.4999, 8.82633, 4.40911	4.98865, 1	3	NA	NA
GL180179.1	64763	66431	65930, 65615, 64817, 66072	13, 24, 9, 9	8.81889, 16.10046, 7.31381, 6.72209	5.43946, 7.04768, 5.22713, 4.8535	6.13764, 1	3	NA	NA
GL180179.1	70515	72851	71609, 71690, 71676, 72563	18, 18, 10, 9	9.20939, 7.43792, 5.30232, 4.82545	4.72805, 3.86938, 3.80299, 3.64274	6.50465, 4	3	NA	NA
GL180179.1	72916	75698	74087, 74349, 74097	14, 17, 19	6.60242, 7.79693, 14.37123	3.97965, 4.17246, 7.34458	4.10964, 5	3	NA	NA
GL180179.1	80463	81183	81023, 81082, 81093	8, 14, 6	8.42257, 14.45897, 7.29403	5.97763, 8.66788, 5.30581	5.76336, 1	3	NA	NA
GL180180.1	88985	89499	89087, 89353, 89047	7, 11, 7	7.1696, 10.60895, 8.69489	5.31345, 6.9343, 6.06379	4.61046, 7	3	NA	81197
GL180181.1	108	652	442, 463, 466	87, 130, 56	100.66965, 149.6395, 67.17546	25.31081, 28.09175, 22.78007	96.27339,	3	NA	NA
GL180181.1	34761	35299	34983, 34988, 34903	50, 59, 33	54.27855, 58.39424, 33.51711	18.40104, 16.79344, 13.83236	50.36514,	3	NA	NA
GL180181.1	35700	36123	35975, 35873, 35995	19, 34, 26	14.15017, 26.73512, 24.22551	7.21609, 9.79617, 10.98452	11.15572,	3	NA	NA
GL180181.1	41892	44967	44725, 43904, 44010	17, 32, 19	10.27574, 20.86632, 13.47445	5.47516, 7.51593, 6.82762	7.49638, 1	3	NA	NA
GL180181.1	47146	48729	48253, 47818, 48374, 47503	22, 13, 14, 15	13.44324, 4.27924, 5.45609, 8.1944	6.13011, 2.88111, 3.37702, 4.69495	10.48603,	3	NA	NA
GL180181.1	81583	82150	81849, 81678, 82026	6, 7, 6	5.96715, 5.97725, 7.29403	4.64927, 4.62287, 5.30581	3.52158, 3	3	NA	NA
GL180182.1	61	918	449, 294, 436	11, 11, 8	12.43693, 10.60895, 10.1465	7.97018, 6.9343, 6.82176	9.52692, 7	3	NA	NA
GL180182.1	49981	50749	50497, 50537, 50536	5, 11, 5	4.82177, 10.60895, 5.9506	3.98509, 6.9343, 4.54784	2.51418, 7	3	NA	NA
GL180182.1	70828	71412	71200, 71290, 71052	10, 7, 3	11.06011, 5.97725, 3.47371	7.306, 4.62287, 3.03189	8.22712, 3	3	NA	NA
GL180183.1	22581	23752	23154, 23101, 23036	18, 20, 13	13.99117, 14.90774, 12.00257	7.38212, 7.39394, 7.40348	11.0029, 1	3	NA	NA
GL180183.1	35422	35943	35610, 35597, 35613	16, 18, 17	9.04691, 9.25556, 10.70376	4.9883, 4.75144, 5.71394	6.3518, 6.7	3	NA	NA
GL180183.1	36910	39017	38353, 37565, 37954	28, 37, 31	19.53073, 28.03649, 25.33466	7.81887, 9.58456, 9.97165	16.31799,	3	NA	NA
GL180183.1	45666	46154	46025, 45818, 45743	15, 15, 13	8.02637, 6.71698, 8.53966	4.60196, 3.90147, 5.26945	5.41127, 4	3	NA	NA
GL180183.1	47823	48570	48141, 48101, 47996	15, 15, 15	7.27963, 5.91511, 9.58751	4.19708, 3.4949, 5.48969	4.71414, 3	3	NA	NA
GL180183.1	51220	51923	51625, 51649, 51571	10, 17, 9	5.34021, 9.72543, 5.85663	3.82638, 5.17374, 4.30023	2.98934, 7	3	NA	NA
GL180183.1	75581	76132	75805, 75892, 76015	5, 6, 3	4.82177, 4.93312, 3.47371	3.98509, 4.04501, 3.03189	2.51418, 2	3	NA	NA
GL180184.1	52069	52630	52438, 52220, 52193	9, 8, 9	7.43495, 5.30997, 9.24728	5.30281, 4.08864, 6.38161	4.85899, 3	3	NA	45163
GL180185.1	42918	43512	43339, 43358, 43283	11, 18, 7	8.12678, 12.63894, 6.47418	5.40251, 6.5909, 4.92032	5.50441, 9	3	NA	NA
GL180185.1	43882	44410	43966, 43995, 44090	18, 21, 8	15.9393, 16.06884, 7.76694	8.55398, 7.79278, 5.6116	12.86597,	3	NA	NA
GL180185.1	51205	52021	51524, 51859, 51707	19, 23, 12	19.72514, 23.03769, 14.58408	10.55748, 11.29653, 9.01638	16.50548,	3	NA	NA
GL180185.1	77281	77940	77433, 77683, 77664	11, 15, 5	12.43693, 15.80067, 5.9506	7.97018, 9.24574, 4.54784	9.52692, 1	3	NA	NA
GL180186.1	65558	66013	65828, 65824, 65674	8, 13, 3	7.19971, 13.14514, 3.47371	5.27799, 8.09002, 3.03189	4.63758, 1	3	NA	NA
GL180187.1	54	815	702, 498, 733	21, 39, 18	25.38534, 46.60689, 24.2813	13.30436, 18.97977, 13.31378	21.99339,	3	NA	NA
GL180187.1	59452	60257	59855, 59638, 60112	17, 21, 9	15.00845, 17.80261, 7.98127	8.26375, 8.80213, 5.63957	11.97619,	3	NA	NA
GL180188.1	146	956	887, 724, 745	9, 16, 5	5.37501, 10.92524, 3.44964	3.99204, 6.05826, 3.1484	3.02176, 8	3	NA	NA
GL180188.1	75140	75929	75480, 75513, 75471	44, 51, 30	53.79531, 60.94899, 38.88202	20.907, 21.69231, 18.16146	49.88943,	3	NA	NA
GL180189.1	35395	36436	35987, 36034, 35818	13, 24, 13	12.81131, 24.86921, 14.85247	7.8918, 12.05732, 9.06201	9.88323, 2	3	NA	NA
GL180189.1	74467	75674	75292, 75289, 75274	24, 32, 14	26.97936, 35.11469, 18.28596	13.31777, 15.1678, 10.7324	23.5443, 3	3	NA	NA
GL180190.1	36699	38095	37129, 37279, 37125	96, 92, 59	59.37067, 54.80274, 35.52002	8.83754, 8.33708, 7.8817	55.39072,	3	NA	NA
GL180190.1	38560	38898	38699, 38698, 38695	225, 224, 195	214.00391, 210.9875, 207.77019	20.59056, 20.17035, 25.74689	208.94681	3	NA	NA
GL180190.1	46944	48138	47572, 47482, 47425	48, 67, 42	37.66109, 54.87099, 37.99657	10.90934, 12.72256, 12.92488	33.99674,	3	NA	NA
GL180190.1	49304	49954	49813, 49825, 49806	33, 34, 20	21.47835, 19.51647, 12.96822	7.56974, 6.54838, 6.31215	18.20137,	3	NA	NA
GL180193.1	61831	63532	62891, 62770, 62780	12, 11, 6	12.74986, 10.05535, 7.29403	8.02869, 6.6012, 5.30581	9.82533, 7	3	NA	NA
GL180194.1	6825	7883	7186, 7200, 7215	35, 40, 20	34.4272, 36.96476, 19.37516	13.57884, 13.09979, 10.0487	30.82509,	3	NA	NA
GL180194.1	36413	37146	36780, 36773, 36865	27, 35, 10	22.57471, 28.12496, 7.42212	9.71082, 10.19764, 5.1369	19.26378,	3	NA	NA
GL180194.1	47724	48402	47896, 47866, 47767	11, 23, 8	11.15818, 25.61868, 9.60885	7.25692, 12.84132, 6.57807	8.32008, 2	3	NA	NA
GL180194.1	69774	70957	70142, 70341, 70845	24, 31, 14	28.77451, 35.10245, 16.29325	14.37118, 15.56124, 9.7093	25.29621,	3	NA	NA
GL180195.1	126	921	631, 658, 663	57, 64, 44	65.69072, 73.00496, 54.35103	21.45936, 22.09084, 21.23366	61.63968,	3	NA	NA
GL180195.1	25536	26790	26014, 25966, 25988	71, 67, 54	74.75706, 70.44812, 60.06356	19.87317, 19.44997, 19.76991	70.61295,	3	NA	NA
GL180195.1	32518	32850	32549, 32561, 32557	45, 56, 27	57.55005, 69.52158, 37.09544	22.63791, 24.12101, 18.12402	53.59303,	3	NA	NA
GL180196.1	133	973	718, 681, 647	21, 14, 13	27.58681, 14.45897, 18.01082	14.51192, 8.66788, 10.61163	24.13677,	3	NA	NA
GL180198.1	39	458	262, 309, 288	14, 10, 16	16.7649, 9.39128, 23.00461	9.96273, 6.35644, 12.83987	13.6543, 6	3	NA	NA
GL180198.1	33120	35822	34349, 34784, 33993	36, 37, 30	25.88301, 25.92527, 22.90714	8.84751, 8.62125, 8.99924	22.47766,	3	NA	NA
GL180198.1	37250	38409	37792, 37593, 38213	42, 34, 19	32.51459, 22.81492, 11.91323	10.28224, 7.94063, 5.95882	28.95171,	3	NA	NA
GL180200.1	64673	65849	64731, 64770, 64779, 65686	14, 18, 8, 6	15.66744, 19.24809, 10.1465, 7.29403	9.36333, 10.55079, 6.82176, 5.30581	12.60415,	3	NA	NA
GL180202.1	4	827	375, 459, 472	18, 36, 14	22.23921, 42.45387, 18.05504	12.25494, 17.84483, 10.62048	18.93827,	3	NA	NA
GL180202.1	11761	13392	12907, 12852, 12643	48, 79, 36	44.96335, 80.86417, 39.34474	14.30583, 19.50735, 16.01224	41.18489,	3	NA	NA
GL180202.1	26574	29206	27249, 27276, 27308	47, 81, 29	38.63619, 69.44047, 25.14797	11.62124, 14.33646, 10.50552	34.95625,	3	NA	NA
GL180202.1	32884	33864	33547, 33552, 33425	39, 53, 31	38.80831, 51.05116, 32.90459	14.47711, 15.48329, 14.25734	35.12484,	3	NA	NA
GL180202.1	39254	40085	39777, 39450, 39779	27, 24, 20	32.92392, 27.49835, 26.49033	15.78361, 13.6248, 14.13188	29.35243,	3	NA	NA
GL180202.1	46016	47416	46352, 46347, 46458	36, 35, 31	37.29457, 33.51791, 37.51135	14.82143, 13.06728, 16.98839	33.63731,	3	NA	NA
GL180202.1	57964	59898	58890, 58775, 58777	49, 72, 37	50.15407, 73.53416, 40.80741	16.59817, 18.89978, 16.44501	46.29984,	3	NA	NA
GL180204.1	48	1097	620, 539, 629	53, 57, 36	59.15217, 60.23628, 43.08532	19.72831, 18.53141, 18.21841	55.17464,	3	NA	NA
GL180204.1	36260	36609	36453, 36453, 36484	23, 36, 14	24.67479, 37.44002, 15.72539	12.27878, 14.9052, 9.39581	21.30234,	3	NA	NA
GL180204.1	64852	65699	65628, 65369, 64864, 65447	8, 16, 5, 3	8.42257, 17.16856, 5.9506, 3.47371	5.97763, 9.82359, 4.54784, 3.03189	5.76336, 1	3	NA	NA
GL180206.1	92	872	290, 616, 208, 742	11, 12, 13, 5	12.43693, 11.86108, 17.924, 5.91431	7.97018, 7.51216, 10.57401, 4.53172	9.52692, 9	3	NA	NA
GL180206.1	6697	8257	7102, 7142, 7413	20, 24, 12	20.44929, 24.07552, 12.7555	10.69687, 11.58146, 8.03189	17.20509,	3	NA	NA
GL180206.1	45909	46640	46262, 46230, 46421	10, 12, 8	11.06011, 11.86108, 10.1465	7.306, 7.51216, 6.82176	8.22712, 9	3	NA	NA
GL180206.1	51272	52358	51978, 52053, 51871	15, 20, 16	18.26603, 22.87459, 20.07743	10.62691, 12.13503, 11.44009	15.09729,	3	NA	NA
GL180207.1	311	831	482, 445, 645	6, 14, 7	5.96715, 14.45897, 8.69489	4.64927, 8.66788, 6.06379	3.52158, 1	3	NA	NA
GL180210.1	34676	35267	34898, 34990, 34938	15, 26, 16	18.26603, 31.2219, 22.07892	10.62691, 15.13645, 12.42524	15.09729,	3	NA	3496
GL180210.1	44740	45540	44926, 45350, 44801	10, 11, 8	11.06011, 10.60895, 10.1465	7.306, 6.9343, 6.82176	8.22712, 7	3	NA	5019
GL180210.1	48390	48659	48447, 48426, 48490	6, 11, 5	5.96715, 10.60895, 5.9506	4.64927, 6.9343, 4.54784	3.52158, 7	3	NA	8669
GL180214.1	36293	36847	36652, 36559, 36536	8, 33, 13	8.42257, 37.42942, 15.89249	5.97763, 16.13281, 9.61177	5.76336, 3	3	NA	NA
GL180215.1	502	2512	1400, 1361, 1757, 1502	12, 8, 17, 10	7.94914, 3.58144, 11.11257, 7.80277	5.10207, 2.97266, 5.94532, 5.37729	5.33969, 1	3	NA	NA
GL180215.1	2855	4353	3302, 3284, 3204, 4122	16, 19, 10, 10	11.95846, 13.08322, 7.80277, 7.80277	6.67194, 6.60592, 5.37729, 5.37729	9.08097, 1	3	NA	NA
GL180215.1	9692	10685	9846, 10259, 10183	17, 16, 10	20.02116, 17.16856, 13.18184	11.236, 9.82359, 8.33771	16.79069,	3	NA	NA
GL180215.1	29196	30940	30596, 30364, 29455, 29807	14, 25, 8, 11	7.21163, 14.35779, 5.06913, 7.76737	4.31434, 5.97965, 3.93247, 5.17666	4.64884, 1	3	NA	NA
GL180215.1	33810	34227	34064, 34150, 34056	12, 19, 13	4.86726, 8.19805, 8.42084	3.29133, 4.09434, 5.19742	2.55571, 5	3	NA	NA
GL180215.1	34722	36077	35401, 35075, 35603	19, 19, 10	11.34604, 9.51627, 7.07516	5.65455, 4.71996, 4.91709	8.49923, 6	3	NA	NA
GL180215.1	41880	42328	42013, 42042, 42057	13, 11, 5	11.50747, 8.89461, 4.88115	7.10015, 5.88422, 4.01777	8.65224, 6	3	NA	NA
GL180215.1	46279	48387	47214, 46953, 47579	21, 20, 14	18.08131, 15.51138, 13.59776	8.9672, 7.74229, 8.15458	14.92149,	3	NA	NA
GL180218.1	145	565	418, 281, 415	13, 12, 5	15.29169, 11.86108, 5.9506	9.29854, 7.51216, 4.54784	12.24396,	3	NA	NA
GL180218.1	9958	11573	10939, 10571, 10806	17, 19, 17	15.06219, 15.57918, 17.26186	8.2965, 8.05686, 9.63127	12.02822,	3	NA	NA
GL180218.1	13196	13949	13483, 13547, 13222, 13910	12, 16, 7, 6	9.38014, 12.25152, 5.42746, 4.45771	5.99192, 6.84834, 4.28057, 3.74549	6.66397, 9	3	NA	NA
GL180218.1	33885	34817	34646, 34592, 34423	7, 13, 6	7.1696, 13.14514, 7.29403	5.31345, 8.09002, 5.30581	4.61046, 1	3	NA	NA
GL180218.1	40562	43286	42198, 42009, 40993, 42007	18, 32, 7, 16	11.47894, 21.88208, 4.28728, 11.96551	5.93457, 7.98047, 3.5411, 6.67617	8.62533, 1	3	NA	NA
GL180218.1	45291	46910	46001, 46299, 45697	18, 22, 17	11.14326, 12.13632, 12.16948	5.74925, 5.48307, 6.55693	8.30703, 9	3	NA	NA
GL180218.1	48108	48718	48614, 48463, 48621	11, 14, 10	8.47312, 10.15403, 8.54288	5.62011, 6.04265, 5.83976	5.81094, 7	3	NA	NA
GL180224.1	39512	40404	40100, 40115, 40115	64, 93, 64	65.26447, 93.52658, 72.7755	18.15655, 19.72501, 21.96885	61.21818,	3	NA	NA
GL180226.1	15582	16171	15953, 15744, 15968	8, 13, 6	8.42257, 13.14514, 7.29403	5.97763, 8.09002, 5.30581	5.76336, 1	3	NA	NA
GL180226.1	39232	40183	39741, 39509, 39259	6, 11, 6	3.51872, 6.03365, 4.82956	3.12725, 4.10206, 3.98076	1.55647, 3	3	NA	NA
GL180226.1	44045	44738	44232, 44171, 44170	15, 25, 13	10.7425, 20.10572, 12.05005	6.17526, 8.93987, 7.43242	7.93785, 1	3	NA	NA
GL180226.1	52004	52556	52145, 52219, 52217	5, 8, 8	4.82177, 7.07159, 10.1465	3.98509, 5.20073, 6.82176	2.51418, 4	3	NA	NA
GL180227.1	50732	51604	50902, 51363, 51439	14, 27, 7	16.6367, 33.30156, 8.69489	9.89449, 16.00277, 6.06379	13.53421,	3	NA	NA
GL180230.1	8997	9622	9299, 9342, 9074	23, 16, 6	25.4258, 15.22617, 6.50067	12.72675, 8.66163, 4.93052	22.03293,	3	NA	NA
GL180230.1	14813	15525	15060, 15227, 15136	8, 13, 10	8.42257, 13.14514, 12.90749	5.97763, 8.09002, 8.21675	5.76336, 1	3	NA	NA
GL180231.1	39	751	466, 327, 295	32, 51, 20	32.35574, 52.53532, 23.16452	13.54576, 17.03161, 12.30292	28.79637,	3	NA	NA
GL180231.1	15552	15976	15698, 15743, 15913	8, 10, 9	5.20521, 6.30783, 7.65011	4.02075, 4.43128, 5.43639	2.86702, 3	3	NA	NA
GL180231.1	48233	49473	49093, 49056, 49113	16, 37, 21	17.2479, 41.42659, 25.2565	9.86999, 16.80781, 13.23126	14.1196, 3	3	NA	NA
GL180232.1	50	814	137, 714, 739	16, 22, 17	9.57404, 11.41013, 12.83146	5.28151, 5.13867, 6.94833	6.84664, 8	3	NA	NA
GL180232.1	1926	4238	2489, 2682, 2480, 3666	34, 72, 33, 17	27.57123, 66.0566, 30.9242, 12.13958	10.21845, 15.65418, 12.35218, 6.53939	24.12154,	3	NA	NA
GL180232.1	38580	40068	39394, 39426, 39350	52, 49, 39	33.85103, 31.09971, 29.22946	8.53249, 8.13584, 9.60067	30.26096,	3	NA	NA
GL180232.1	40886	42058	41559, 41521, 41542	68, 82, 40	50.99237, 67.75386, 30.34254	11.10834, 13.5055, 9.84069	47.12663,	3	NA	NA
GL180232.1	46685	47621	47283, 46810, 47260	90, 127, 49	106.98876, 130.87772, 60.1388	26.97434, 22.21343, 22.20424	102.54166	3	NA	NA
GL180233.1	14539	15609	15427, 15033, 15082	18, 38, 19	19.69649, 40.08348, 22.04849	10.81516, 15.60378, 11.92281	16.47841,	3	NA	NA
GL180233.1	48225	49254	48999, 48877, 49001	20, 33, 11	23.40409, 36.57634, 13.28509	12.44081, 15.62743, 8.40871	20.06837,	3	NA	NA
GL180235.1	47992	49219	48080, 48080, 49109, 48095, 48876	9, 20, 18, 7, 6	7.75911, 15.8975, 14.36017, 7.50542, 6.45058	5.5036, 7.96752, 7.60184, 5.49244, 4.90508	5.16246, 1	3	NA	NA
GL180236.1	907	2299	1339, 1339, 1347	277, 346, 145	255.19275, 311.67697, 139.48711	19.68354, 19.12816, 19.83102	250.00705	3	NA	NA
GL180236.1	20018	20256	20100, 20117, 20128	31, 33, 14	25.02928, 25.92169, 11.48931	9.8106, 9.67782, 6.86326	21.64765,	3	NA	NA
GL180236.1	21136	21456	21300, 21298, 21284	27, 31, 10	22.67908, 26.28656, 8.39789	9.76909, 10.48099, 5.74985	19.36533,	3	NA	NA
GL180236.1	27890	28793	28207, 28467, 28591	6, 6, 4	5.96715, 4.93312, 4.67325	4.64927, 4.04501, 3.78987	3.52158, 2	3	NA	NA
GL180236.1	48936	49639	49162, 49151, 49215	38, 40, 22	45.14908, 47.14112, 27.01579	18.56445, 18.84485, 13.95629	41.36814,	3	NA	NA
GL180237.1	37741	38459	38261, 38108, 38235	9, 17, 5	9.70623, 17.26139, 5.91431	6.63396, 9.63099, 4.53172	6.97147, 1	3	NA	NA
GL180238.1	26	387	234, 156, 155	21, 25, 10	27.77672, 30.45643, 13.18184	14.612, 15.02432, 8.33771	24.32202,	3	NA	NA
GL180238.1	6579	7871	7269, 6908, 7336	90, 82, 66	97.87457, 84.19127, 77.81968	22.69343, 19.827, 23.70684	93.50248,	3	NA	NA
GL180238.1	28699	31127	29761, 30089, 30124	94, 112, 56	65.06221, 72.56068, 38.89916	10.40877, 9.6439, 9.50012	61.01827,	3	NA	NA
GL180238.1	33308	35751	34675, 34675, 34247	86, 119, 53	56.00115, 86.02676, 35.07055	9.37326, 11.52316, 8.76349	52.06488,	3	NA	NA
GL180238.1	39745	44186	43663, 43419, 43719	109, 121, 73	73.98073, 77.34366, 52.10293	10.31285, 9.53819, 10.44846	69.84364,	3	NA	NA
GL180240.1	50	792	385, 368, 353	11, 16, 14	12.43693, 17.16856, 18.77534	7.97018, 9.82359, 10.96344	9.52692, 1	3	NA	NA
GL180240.1	34837	35983	35216, 35114, 35143	16, 25, 17	19.41253, 28.13462, 21.19882	11.08796, 13.66232, 11.87761	16.20324,	3	NA	NA
GL180240.1	41976	42607	42229, 42030, 42205	13, 15, 9	15.1723, 15.80067, 11.64359	9.23486, 9.24574, 7.57973	12.13305,	3	NA	NA
GL180241.1	20	944	739, 332, 345	27, 41, 17	29.3399, 46.29793, 19.81591	13.66394, 17.90246, 11.12108	25.84829,	3	NA	NA
GL180241.1	31780	32751	32547, 32497, 32537	42, 57, 39	36.65154, 49.42227, 34.12227	12.24661, 13.36541, 11.97015	33.00615,	3	NA	NA
GL180241.1	35936	36614	36102, 36107, 36108	39, 47, 29	21.84421, 25.09776, 15.45512	6.5855, 6.48079, 5.75593	18.5551, 2	3	NA	NA
GL180241.1	37854	38703	38079, 38071, 38194	34, 38, 39	21.50571, 22.40556, 32.09809	7.3691, 6.96377, 10.95615	18.22764,	3	NA	NA
GL180241.1	40282	40920	40711, 40717, 40708	59, 70, 34	49.08221, 56.84037, 26.3135	12.63274, 12.67764, 9.58663	45.24234,	3	NA	NA
GL180241.1	48024	48666	48136, 48126, 48122	48, 42, 43	58.19263, 49.23438, 55.95539	21.55943, 19.16379, 22.65065	54.22716,	3	NA	NA
GL180242.1	10968	12122	11431, 11419, 11214	39, 43, 26	42.94985, 44.78186, 32.16442	16.83511, 16.17115, 15.68273	39.20106,	3	NA	NA
GL180242.1	44841	46082	45218, 45353, 45174	17, 34, 17	20.70049, 40.86838, 21.19882	11.60995, 17.7557, 11.87761	17.44812,	3	NA	NA
GL180246.1	139	834	661, 631, 414	9, 13, 6	9.72082, 13.14514, 7.29403	6.64182, 8.09002, 5.30581	6.97155, 1	3	NA	NA
GL180246.1	34035	34703	34153, 34468, 34123, 34502	8, 14, 4, 5	8.42257, 14.45897, 4.67325, 5.9506	5.97763, 8.66788, 3.78987, 4.54784	5.76336, 1	3	NA	NA
GL180246.1	40726	41465	40987, 41001, 41419	4, 14, 8	3.74195, 14.45897, 10.1465	3.32091, 8.66788, 6.82176	1.6222, 11	3	NA	NA
GL180246.1	46944	47685	47060, 47432, 47470	8, 8, 4	8.42257, 7.07159, 4.67325	5.97763, 5.20073, 3.78987	5.76336, 4	3	NA	NA
GL180247.1	45	1037	569, 887, 881	28, 53, 24	32.04276, 60.99978, 30.6645	14.90243, 20.75626, 15.44595	28.49042,	3	NA	NA
GL180249.1	39340	40200	39989, 39752, 39733	17, 26, 7	19.60089, 30.50245, 8.69489	10.9998, 14.71478, 6.06379	16.38573,	3	NA	NA
GL180249.1	47178	47947	47723, 47526, 47493	13, 15, 5	15.29169, 15.80067, 5.9506	9.29854, 9.24574, 4.54784	12.24396,	3	NA	NA
GL180250.1	5102	6055	5782, 5718, 5726	41, 43, 35	18.3559, 17.66711, 18.7581	5.17195, 4.77607, 6.08976	15.1838, 1	3	NA	NA
GL180250.1	6993	9600	7172, 8965, 8848	54, 85, 48	22.29081, 40.28505, 23.77443	4.98364, 6.16518, 5.95835	18.98903,	3	NA	NA
GL180250.1	6993	9600	8877, 8965, 8848	66, 85, 48	31.30466, 40.28505, 23.77443	5.97324, 6.16518, 5.95835	27.76769,	3	NA	NA
GL180250.1	9867	12203	11153, 11294, 11281	90, 104, 68	52.93704, 56.92089, 43.77108	8.16927, 7.54569, 8.86669	49.04359,	3	NA	NA
GL180250.1	13471	14179	14125, 14124, 14009	60, 97, 30	36.42161, 64.19526, 16.08158	7.9942, 9.77026, 5.84864	32.78083,	3	NA	NA
GL180250.1	40054	42223	41592, 41590, 41643	132, 140, 93	115.85535, 118.35831, 87.41145	16.37783, 15.41922, 17.39623	111.33752	3	NA	NA
GL180251.1	30056	30305	30169, 30116, 30154	10, 11, 4	9.28413, 8.62761, 4.04585	6.29221, 5.71704, 3.48564	6.574, 6.1(	3	NA	1441
GL180252.1	41	597	316, 377, 388	21, 30, 11	25.38534, 35.18344, 14.45778	13.30436, 16.00313, 8.96373	21.99339,	3	NA	NA
GL180256.1	583	1065	892, 724, 745	7, 12, 5	7.1696, 11.86108, 5.9506	5.31345, 7.51216, 4.54784	4.61046, 9	3	NA	NA
GL180258.1	23	1387	738, 926, 214	39, 74, 23	38.72741, 70.45686, 24.19472	14.43219, 16.82056, 11.99126	35.04519,	3	NA	NA
GL180258.1	25270	30612	27586, 27540, 25323, 27566	115, 155, 27, 86	59.81574, 72.29903, 6.03342, 48.07701	7.13572, 6.38611, 2.65757, 7.58426	55.82951,	3	NA	NA
GL180260.1	55	1314	207, 733, 1246	9, 12, 8	9.36986, 10.83413, 9.05167	6.44997, 6.89288, 6.30653	6.65425, 8	3	NA	NA
GL180260.1	237	1314	628, 733, 1246	7, 12, 8	6.89193, 10.83413, 9.05167	5.15998, 6.89288, 6.30653	4.37772, 8	3	NA	NA
GL180265.1	45409	46222	45777, 45895, 46152	9, 12, 4	9.00096, 11.86108, 4.67325	6.24222, 7.51216, 3.78987	6.30808, 9	3	NA	NA
GL180266.1	125	661	213, 497, 425	6, 10, 7	5.96715, 9.39128, 8.69489	4.64927, 6.35644, 6.06379	3.52158, 6	3	NA	NA
GL180267.1	25764	26992	26398, 26706, 26386	23, 33, 16	15.24048, 19.10127, 11.09761	6.83532, 6.55995, 6.15961	12.19881,	3	NA	NA
GL180268.1	54	1069	473, 708, 528	18, 12, 12	20.25794, 11.86108, 14.06375	11.14243, 7.51216, 8.74831	17.01944,	3	NA	NA
GL180268.1	6717	7304	6964, 6939, 6806	16, 14, 13	19.7934, 14.45897, 16.93213	11.29109, 8.66788, 10.12358	16.57067,	3	NA	NA
GL180270.1	202	772	448, 664, 636	9, 13, 7	9.72082, 13.14514, 8.69489	6.64182, 8.09002, 6.06379	6.97155, 1	3	NA	NA
GL180270.1	33775	34218	33938, 33933, 33821	10, 21, 6	6.03527, 15.13786, 3.68051	4.25963, 7.26651, 3.23556	3.58483, 1	3	NA	NA
GL180270.1	39417	40331	40202, 39854, 39829, 40252	14, 34, 20, 10	8.55049, 21.98307, 15.00532, 6.19112	5.08099, 7.57462, 7.44991, 4.35768	5.88478, 1	3	NA	NA
GL180270.1	41587	42145	41928, 41895, 41985	23, 41, 29	15.9086, 28.71005, 25.34408	7.1874, 8.87678, 10.615	12.83586,	3	NA	NA
GL180270.1	45600	46140	45824, 45684, 46077	16, 19, 10	13.38469, 13.28396, 8.12251	7.53684, 6.71936, 5.57808	10.43027,	3	NA	NA
GL180275.1	44246	44618	44435, 44357, 44529	7, 13, 4	7.1696, 13.14514, 4.67325	5.31345, 8.09002, 3.78987	4.61046, 1	3	NA	NA
GL180276.1	7362	8082	7551, 7465, 7467	24, 29, 17	28.77451, 34.86947, 22.63257	14.37118, 16.19933, 12.61306	25.29621,	3	NA	NA
GL180279.1	81	1090	495, 451, 517	29, 28, 19	26.08017, 19.67522, 17.60381	11.02948, 7.89104, 9.27603	22.66837,	3	NA	NA
GL180279.1	4221	6190	5589, 4725, 4587, 6076	29, 29, 25, 9	22.24543, 16.47243, 21.88331, 6.12174	8.93821, 6.18465, 9.95161, 4.47008	18.94445,	3	NA	NA
GL180279.1	9007	9694	9103, 9326, 9089	20, 15, 14	14.05739, 8.25057, 11.41485	6.91203, 4.72618, 6.81732	11.06737,	3	NA	NA
GL180279.1	33356	34907	33848, 34165, 34131	24, 35, 14	14.9026, 20.19518, 7.18467	6.44169, 6.64022, 4.29933	11.87572,	3	NA	NA
GL180279.1	37532	38684	38332, 38091, 38073	25, 31, 23	12.68492, 13.78319, 12.84562	5.22806, 4.83185, 5.64077	9.76328, 1	3	NA	NA
GL180279.1	39366	40025	39451, 39557, 39412	18, 21, 15	8.63819, 8.85816, 7.54378	4.44281, 4.1359, 4.3387	5.96784, 6	3	NA	NA
GL180282.1	287	696	556, 516, 573	15, 13, 8	18.26603, 13.14514, 10.1465	10.62691, 8.09002, 6.82176	15.09729,	3	NA	NA
GL180284.1	112	1455	1029, 1013, 1131	18, 22, 13	20.06618, 24.36754, 15.70541	11.03116, 12.41949, 9.51556	16.83343,	3	NA	NA
GL180284.1	31893	32705	32109, 32206, 32083	12, 19, 8	6.73702, 10.56678, 6.04892	4.36537, 5.24747, 4.56389	4.23515, 7	3	NA	NA
GL180284.1	33351	35385	33561, 34915, 33930, 34499	10, 23, 9, 12	4.52232, 11.55794, 6.33495, 8.70794	3.32851, 5.04519, 4.60655, 5.57265	2.31531, 8	3	NA	NA
GL180284.1	33515	35385	33863, 34915, 33930, 34499	14, 23, 9, 12	7.40719, 11.55794, 6.33495, 8.70794	4.42377, 5.04519, 4.60655, 5.57265	4.8328, 8.8	3	NA	NA
GL180284.1	33515	35385	34480, 34915, 33930, 34499	21, 23, 9, 12	13.06431, 11.55794, 6.33495, 8.70794	6.14529, 5.04519, 4.60655, 5.57265	10.12502,	3	NA	NA
GL180284.1	39355	39974	39626, 39623, 39695	20, 19, 13	14.94592, 12.48272, 12.54936	7.41582, 6.27066, 7.73479	11.91764,	3	NA	NA
GL180285.1	71	2340	1148, 1150, 1142	106, 159, 61	95.34219, 158.73459, 53.33092	16.49517, 21.7025, 13.80731	90.99159,	3	NA	NA
GL180285.1	36985	38186	37662, 37626, 37551	23, 31, 8	27.48315, 32.9115, 10.1465	13.9341, 14.26142, 6.82176	24.03497,	3	NA	NA
GL180290.1	4512	4989	4711, 4637, 4820	8, 9, 8	8.22568, 7.35794, 9.92329	5.8701, 5.25474, 6.7228	5.59681, 4	3	NA	467
GL180292.1	70	796	397, 311, 280	15, 19, 15	10.34772, 12.76079, 12.08408	5.93872, 6.42512, 6.99105	7.56472, 1	3	NA	NA
GL180292.1	3875	6102	5341, 5590, 5330	25, 38, 25	18.49305, 29.70761, 21.18133	8.05607, 10.09714, 9.54795	15.31644,	3	NA	NA
GL180292.1	11645	12370	12057, 11671, 11862	5, 8, 8	4.77263, 6.37769, 8.35103	3.9578, 4.77199, 5.93881	2.51418, 4	3	NA	NA
GL180292.1	44003	44873	44698, 44690, 44366	18, 19, 19	20.45461, 19.8035, 23.22595	11.25597, 10.60442, 12.5858	17.20992,	3	NA	NA
GL180294.1	452	898	782, 789, 618	5, 12, 5	4.69353, 11.86108, 5.23257	3.9134, 7.51216, 4.20435	2.45909, 9	3	NA	NA

GL180294.1	2807	3304	3215, 2927, 3176	12, 13, 7	13.55951, 13.14514, 7.72582	8.47903, 8.09002, 5.6058	10.59689,	3	NA	NA	
GL180296.1	37717	39468	38244, 39137, 38264	44, 56, 61	39.2766, 52.79732, 67.27011	12.87931, 15.22758, 20.44605	35.58593,	3	NA	NA	
GL180298.1	16	706	511, 390, 202	11, 15, 9	11.56615, 15.80067, 11.64359	7.49066, 9.24574, 7.57973	8.70729, 1	3	NA	NA	
GL180298.1	43204	43612	43427, 43427, 43365	60, 74, 47	73.37167, 89.44833, 62.72824	24.26812, 25.94006, 24.7098	69.24306,	3	NA	NA	
GL180303.1	33285	33635	33506, 33500, 33456	18, 18, 12	6.97146, 4.57885, 4.9645	3.65612, 2.6444, 3.34486	4.45181, 2	3	NA	NA	
GL180303.1	36734	37034	36835, 36859, 36902	21, 30, 16	8.27098, 10.96015, 7.30533	3.88613, 3.97518, 4.06591	5.63907, 8	3	NA	NA	
GL180303.1	37515	38057	37655, 37896, 37640	23, 36, 22	10.08772, 17.35543, 12.48068	4.40678, 5.44669, 5.65021	7.31934, 1	3	NA	NA	
GL180304.1	89	696	278, 303, 496	10, 17, 5	11.06011, 18.56115, 5.9506	7.306, 10.40145, 4.54784	8.22712, 1	3	NA	NA	
GL180310.1	6665	7459	7389, 7003, 7346	8, 16, 12	8.42257, 16.37065, 15.16114	5.97763, 9.3517, 9.30141	5.76336, 1	3	NA	NA	
GL180310.1	39639	40257	39976, 39986, 39889	13, 15, 9	14.61367, 15.80067, 9.76105	8.93088, 9.24574, 6.66343	11.59864,	3	NA	NA	
GL180310.1	42531	43410	43111, 43228, 43176	10, 11, 14	10.53228, 10.60895, 16.82614	7.01712, 6.9343, 9.99514	7.73927, 7	3	NA	NA	
GL180311.1	7203	7440	7403, 7392, 7391	11, 11, 7	12.43693, 10.60895, 8.69489	7.97018, 6.9343, 6.06379	9.52692, 7	3	NA	NA	
GL180312.1	41756	42469	41933, 42078, 42359	10, 22, 10	11.06011, 25.85271, 13.18184	7.306, 13.29074, 8.33771	8.22712, 2	3	NA	NA	
GL180313.1	109	569	317, 184, 427	10, 11, 6	11.06011, 10.60895, 7.29403	7.306, 6.9343, 5.30581	8.22712, 7	3	NA		33487
GL180314.1	49	1179	955, 509, 1081, 228	8, 5, 7, 7	8.42257, 3.94561, 5.97725, 8.69489	5.97763, 3.46715, 4.62287, 6.06379	5.76336, 1	3	NA	NA	
GL180314.1	34997	37006	35882, 36356, 35368	16, 18, 14	8.93373, 7.7207, 8.46716	4.92614, 4.00128, 5.03216	6.24601, 5	3	NA	NA	
GL180314.1	34997	37315	37082, 36356, 35368	9, 18, 14	3.40681, 7.7207, 8.46716	2.76673, 4.00128, 5.03216	1.47026, 5	3	NA	NA	
GL180314.1	38060	41042	40298, 40190, 38556, 40188	21, 29, 13, 16	16.11018, 19.92261, 7.93055, 11.99775	7.81643, 7.76703, 4.90257, 6.69554	13.03003,	3	NA	NA	
GL180317.1	70	820	611, 720, 446	6, 14, 6	5.96715, 14.45897, 7.29403	4.64927, 8.66788, 5.30581	3.52158, 1	3	NA	NA	
GL180317.1	41689	42864	42396, 41976, 42350	13, 21, 13	14.43911, 22.22847, 16.0859	8.83395, 11.45784, 9.70995	11.43182,	3	NA	NA	
GL180319.1	6713	8132	7078, 7217, 7129	9, 11, 7	9.72082, 10.60895, 8.69489	6.64182, 6.9343, 6.06379	6.97155, 7	3	NA	NA	
GL180320.1	174	1095	888, 441, 791, 865	9, 9, 9, 6	9.72082, 8.21099, 8.21099, 7.29403	6.64182, 5.77858, 5.77858, 5.30581	6.97155, 5	3	NA	NA	
GL180320.1	6502	7303	7014, 6978, 7106	12, 23, 5	13.8483, 27.36982, 5.9506	8.63436, 13.8686, 4.54784	10.86694,	3	NA	NA	
GL180321.1	51	773	248, 257, 630	8, 14, 7	8.42257, 14.45897, 8.69489	5.97763, 8.66788, 6.06379	5.76336, 1	3	NA	NA	
GL180321.1	6183	7355	7109, 6763, 6949	9, 21, 13	9.72082, 24.1145, 16.70862	6.64182, 12.57361, 10.0169	6.97155, 2	3	NA	NA	
GL180321.1	41831	42742	42365, 42552, 41894, 42433	12, 16, 6, 6	13.8483, 17.16856, 7.29403, 7.29403	8.63436, 9.82359, 5.30581, 5.30581	10.86694,	3	NA	NA	
GL180324.1	6877	7664	7176, 7423, 7166	10, 23, 10	11.06011, 27.36982, 13.18184	7.306, 13.8686, 8.33771	8.22712, 2	3	NA	NA	
GL180324.1	35849	36982	36499, 36031, 36002	28, 27, 27	30.19897, 28.00888, 31.35877	13.80434, 12.86965, 14.86484	26.68663,	3	NA	NA	
GL180324.1	42222	42608	42332, 42545, 42280	7, 7, 6	7.1696, 5.97725, 7.29403	5.31345, 4.62287, 5.30581	4.61046, 3	3	NA	NA	
GL180325.1	81	1502	757, 759, 738	20, 31, 10	23.62787, 35.77021, 13.18184	12.56888, 15.95791, 8.33771	20.28502,	3	NA	NA	
GL180326.1	35	735	234, 266, 477	13, 37, 8	15.29169, 43.50184, 10.1465	9.29854, 18.03078, 6.82176	12.24396,	3	NA	NA	
GL180326.1	35346	36349	36080, 35815, 36105	17, 27, 14	21.19088, 30.66659, 19.03529	11.87339, 14.45444, 11.08274	17.92307,	3	NA	NA	
GL180326.1	41531	42482	42029, 42007, 41650	17, 29, 12	20.94161, 34.13175, 16.0427	11.74019, 15.76422, 9.71071	17.68164,	3	NA	NA	
GL180334.1	232	1241	897, 881, 947	6, 10, 6	5.27951, 8.61706, 5.2665	4.25534, 5.8856, 4.24757	2.9335, 6.(	3	NA	NA	
GL180334.1	4974	5894	5378, 5308, 5288	16, 16, 17	16.60441, 14.52284, 18.96688	9.49088, 8.2328, 10.63714	13.50404,	3	NA	NA	
GL180335.1	35	795	480, 474, 436	10, 6, 12	11.06011, 4.93312, 16.36823	7.306, 4.04501, 9.85365	8.22712, 2	3	NA		14798
GL180337.1	94	785	656, 650, 657	161, 234, 142	192.85889, 269.15189, 171.97397	32.24034, 31.51141, 32.05616	187.90961	3	NA	NA	
GL180337.1	35674	36186	35855, 36007, 35877	15, 24, 7	17.90819, 28.90463, 8.69489	10.43573, 14.44646, 6.06379	14.75478,	3	NA	NA	
GL180337.1	41290	42656	41663, 41641, 41646	42, 41, 24	43.38329, 41.1304, 24.03231	15.80892, 14.96746, 11.55557	39.62743,	3	NA	NA	
GL180340.1	15	708	300, 415, 415	22, 45, 13	28.30573, 56.17128, 17.924	14.67216, 21.82674, 10.57401	24.83844,	3	NA	NA	
GL180343.1	51	436	180, 167, 164	32, 42, 25	15.43026, 18.6213, 13.7419	5.29801, 5.14313, 5.69674	12.37683,	3	NA	NA	
GL180343.1	2216	4502	3777, 3630, 3361	71, 102, 58	54.30092, 77.86816, 49.09886	11.5593, 12.3196, 12.92722	50.38721,	3	NA	NA	
GL180343.1	36888	38137	37685, 37707, 37734	75, 81, 59	76.19458, 81.8721, 66.80432	18.95276, 19.19743, 21.11995	72.03564,	3	NA	NA	
GL180346.1	21	1005	243, 239, 224	42, 51, 31	43.74598, 48.23838, 34.14983	16.01142, 14.83735, 14.99529	39.9847, 4	3	NA	NA	
GL180346.1	40592	41694	41483, 41185, 41160	25, 33, 20	17.18726, 20.9653, 14.44996	7.36883, 7.34466, 7.13314	14.06103,	3	NA	NA	
GL180353.1	37563	38054	37947, 37414, 37975, 37977	6, 7, 6, 4	5.96715, 5.8946, 4.86158, 4.38152	4.64927, 4.57222, 4.00069, 3.65448	3.52158, 3	3	NA	NA	
GL180355.1	94	1509	262, 561, 523	19, 30, 18	18.13647, 27.585, 22.5396	9.59882, 11.53781, 12.41681	14.97405,	3	NA	NA	
GL180355.1	7199	8301	7792, 8030, 7658	14, 24, 12	15.31941, 23.48334, 14.96175	9.1665, 11.22699, 9.20442	12.27033,	3	NA	NA	
GL180355.1	14773	16010	15823, 15635, 15825	13, 26, 13	9.4196, 17.96128, 10.14686	5.8082, 7.52118, 6.25812	6.70189, 1	3	NA	NA	
GL180355.1	21535	22184	21739, 21839, 22097	17, 22, 10	21.34551, 25.85271, 13.18184	11.95527, 13.29074, 8.33771	18.07159,	3	NA	NA	
GL180355.1	32693	33512	33208, 33261, 32957	15, 21, 19	17.09296, 24.35402, 23.01756	9.98755, 12.71289, 12.47022	13.96978,	3	NA	NA	
GL180355.1	38818	39859	39459, 39492, 39458	26, 42, 15	28.48278, 46.26778, 17.07266	13.51628, 17.44198, 9.97618	25.01069,	3	NA	NA	
GL180356.1	4889	5427	5072, 5053, 5228	5, 6, 6	4.82177, 4.93312, 7.29403	3.98509, 4.04501, 5.30581	2.51418, 2	3	NA	NA	
GL180359.1	7976	8320	8145, 8121, 8139	11, 9, 11	12.43693, 8.21099, 14.75772	7.97018, 5.77858, 9.09568	9.52692, 5	3	NA		4169
GL180360.1	25	930	596, 442, 352	29, 30, 14	35.80759, 36.96607, 19.59014	16.74862, 17.0531, 11.3293	32.17825,	3	NA	NA	
GL180360.1	38417	39196	38915, 39118, 38830	9, 14, 12	6.79611, 10.61504, 11.26182	4.90054, 6.32483, 7.15321	4.28827, 7	3	NA	NA	
GL180363.1	18663	19616	19135, 19114, 19192	19, 10, 10	19.10984, 8.98028, 11.10454	10.1875, 6.10835, 7.32977	15.91125,	3	NA	NA	
GL180364.1	61	364	224, 223, 270	20, 30, 18	8.35886, 13.29865, 10.11069	4.04098, 4.77922, 5.1937	5.7202, 10	3	NA	NA	
GL180364.1	1933	5054	4006, 4004, 2341, 4008	66, 98, 22, 46	48.22747, 77.96918, 12.86658, 37.01144	10.61008, 13.11028, 5.84038, 11.18198	44.40061,	3	NA	NA	
GL180364.1	9326	9866	9820, 9801, 9803	17, 16, 16	10.02239, 8.90489, 13.08988	5.33558, 4.91035, 7.35698	7.25686, 6	3	NA	NA	
GL180364.1	37576	39582	38553, 38545, 38574	232, 242, 174	218.50545, 223.5732, 183.10977	20.24485, 19.54495, 24.60686	213.42966	3	NA	NA	
GL180365.1	57	927	705, 705, 729	49, 54, 61	60.96447, 68.10481, 84.00763	22.68615, 24.32709, 29.87815	56.96571,	3	NA	NA	
GL180369.1	22	304	247, 217, 204	12, 20, 9	11.67221, 22.06591, 11.64359	7.39985, 11.6614, 7.57973	8.80793, 1	3	NA	NA	
GL180369.1	36445	37590	36745, 36769, 36797	128, 175, 100	133.86969, 190.06393, 110.97713	22.92618, 26.37162, 24.32769	129.23335	3	NA	NA	
GL180370.1	3029	4730	3944, 3731, 4260	58, 72, 53	50.56569, 63.63894, 55.27258	13.55086, 14.68817, 17.63654	46.70496,	3	NA	NA	
GL180371.1	39598	40438	39798, 40104, 39692	7, 9, 4	7.1696, 8.21099, 4.67325	5.31345, 5.77858, 3.78987	4.61046, 5	3	NA	NA	
GL180373.1	60	828	539, 357, 554	12, 17, 12	13.05874, 17.71433, 15.36829	8.20296, 9.9018, 9.40047	10.11961,	3	NA	NA	
GL180373.1	37695	38481	38286, 38166, 38318	15, 23, 8	12.07737, 18.11323, 6.32833	6.98694, 8.39982, 4.74092	9.19364, 1	3	NA	NA	
GL180374.1	32	2471	1628, 680, 1630	122, 135, 94	100.57289, 108.8217, 85.22945	14.44285, 14.0851, 16.33471	96.17747,	3	NA	NA	
GL180374.1	38090	38751	38196, 38206, 38473	79, 69, 45	85.0738, 67.81087, 48.96733	21.42031, 17.43103, 17.65646	80.82576,	3	NA	NA	
GL180378.1	134	1063	306, 295, 303	47, 63, 29	54.28016, 71.48018, 36.22158	19.77273, 21.75098, 16.98927	50.3667, 6	3	NA	NA	
GL180378.1	32865	33459	32947, 33069, 33093	15, 19, 8	17.6946, 21.4153, 10.1465	10.31995, 11.55717, 6.82176	14.54864,	3	NA	NA	
GL180378.1	39778	40320	39961, 40285, 39922	11, 7, 11	12.43693, 5.97725, 14.03502	7.97018, 4.62287, 8.77076	9.52692, 3	3	NA	NA	
GL180380.1	1542	2729	1794, 1941, 1962	13, 9, 9	11.45737, 7.28344, 9.30116	7.06931, 5.20811, 6.41174	8.60415, 4	3	NA		19903
GL180380.1	9204	10031	9626, 9481, 9589	69, 66, 35	61.5046, 52.93902, 33.04643	14.7382, 12.26185, 12.80473	57.50017,	3	NA		12601
GL180380.1	37705	38330	37987, 37945, 38246	9, 16, 14	6.14461, 9.22296, 12.25066	4.48473, 5.08555, 7.33267	3.6868, 6.6	3	NA		1517
GL180383.1	18	1145	659, 441, 446	14, 22, 10	16.03678, 23.98993, 12.90749	9.5688, 12.19509, 8.21675	12.95916,	3	NA	NA	
GL180383.1	34604	35703	35512, 35309, 35263	16, 24, 19	16.15907, 24.86921, 23.88486	9.22513, 12.05732, 12.94573	13.07749,	3	NA	NA	
GL180385.1	39056	40055	39264, 39353, 39448	12, 19, 15	13.8483, 20.47061, 18.74072	8.63436, 11.002, 10.87493	10.86694,	3	NA	NA	
GL180387.1	1790	2364	1836, 1839, 1901	5, 10, 10	4.82177, 9.39128, 13.18184	3.98509, 6.35644, 8.33771	2.51418, 6	3	NA	NA	
GL180387.1	8504	8914	8609, 8598, 8606	30, 29, 25	33.92505, 33.2124, 31.77551	15.25598, 15.21917, 15.77668	30.33351,	3	NA	NA	
GL180390.1	34788	35738	35359, 35307, 35008	10, 11, 7	9.77513, 9.4823, 7.61369	6.5837, 6.24973, 5.54854	7.02302, 6	3	NA	NA	
GL180396.1	39261	39899	39676, 39534, 39598	9, 12, 11	9.72082, 11.86108, 14.75772	6.64182, 7.51216, 9.09568	6.97155, 9	3	NA	NA	
GL180399.1	20	1131	398, 542, 356	46, 78, 33	46.16158, 85.19557, 38.05182	15.69188, 21.90246, 16.50191	42.36558,	3	NA	NA	
GL180399.1	37086	37851	37275, 37347, 37437	12, 16, 6	8.67445, 11.65768, 4.96699	5.55179, 6.49186, 4.06589	6.00102, 8	3	NA	NA	
GL180399.1	39365	39854	39799, 39792, 39685	14, 15, 10	10.74704, 10.6339, 9.44624	6.40591, 6.10999, 6.38926	7.94177, 7	3	NA	NA	
GL180400.1	3187	4263	3626, 3629, 3621	45, 57, 29	47.25626, 62.26389, 31.76077	16.70065, 19.59834, 14.35529	43.44327,	3	NA	NA	
GL180400.1	38369	39048	38694, 38466, 38906	10, 15, 7	11.06011, 15.80067, 8.69489	7.306, 9.24574, 6.06379	8.22712, 1	3	NA	NA	
GL180401.1	37838	39422	38610, 38635, 38578	16, 11, 16	19.7934, 10.60895, 22.68246	11.29109, 6.9343, 12.69862	16.57067,	3	NA	NA	
GL180402.1	52	2966	1956, 984, 2817	76, 101, 50	57.53322, 74.68265, 38.32073	11.53395, 11.67648, 10.65848	53.57644,	3	NA	NA	
GL180402.1	33831	35006	33937, 34391, 33933	70, 95, 36	61.09187, 79.47734, 31.23565	14.27663, 14.20321, 11.47705	57.09113,	3	NA	NA	
GL180402.1	37100	37849	37307, 37383, 37332	39, 66, 28	25.76409, 46.27906, 21.85174	8.0992, 9.97552, 9.01612	22.3615, 4	3	NA	NA	
GL180403.1	38655	39525	39233, 39444, 39220	69, 50, 38	80.97111, 54.06868, 47.45436	23.92033, 18.2858, 19.92565	76.7663, 5	3	NA	NA	
GL180404.1	47	634	248, 258, 168	9, 21, 11	9.72082, 20.81922, 11.31465	6.64182, 10.61044, 7.3472	6.97155, 1	3	NA	NA	
GL180404.1	2431	2908	2693, 2612, 2569	8, 14, 7	8.42257, 12.09105, 6.43637	5.97763, 7.23439, 4.89813	5.76336, 9	3	NA	NA	
GL180404.1	32004	32624	32261, 32486, 32392	24, 27, 15	24.60735, 26.14468, 15.36085	11.90029, 11.7641, 8.986	21.23605,	3	NA	NA	
GL180407.1	240	802	738, 562, 708	13, 10, 9	15.29169, 9.39128, 11.64359	9.29854, 6.35644, 7.57973	12.24396,	3	NA	NA	
GL180407.1	31405	31968	31564, 31586, 31445	24, 47, 37	31.25967, 58.66771, 51.43163	15.77492, 22.32451, 22.65334	27.72325,	3	NA	NA	
GL180408.1	38054	38545	38118, 38424, 38125	7, 12, 12	7.1696, 11.86108, 16.0427	5.31345, 7.51216, 9.71071	4.61046, 9	3	NA	NA	
GL180417.1	1024	1222	1129, 1139, 1127	15, 14, 12	18.26603, 14.45897, 15.16114	10.62691, 8.66788, 9.30141	15.09729,	3	NA	NA	
GL180421.1	96	1053	185, 778, 885	12, 16, 7	12.3187, 15.41438, 7.72582	7.78073, 8.77591, 5.6058	9.4231, 12	3	NA	NA	
GL180421.1	2472	3082	2866, 2741, 2855	6, 10, 9	5.16999, 8.28331, 10.42289	4.18963, 5.67853, 7.00726	2.83325, 5	3	NA	NA	
GL180421.1	37687	38585	37967, 37832, 37749, 38024	10, 14, 6, 3	11.06011, 14.45897, 7.29403, 3.47371	7.306, 8.66788, 5.30581, 3.03189	8.22712, 1	3	NA	NA	
GL180422.1	51	549	249, 205, 217	13, 24, 15	12.68462, 26.09829, 20.14748	7.81602, 12.79322, 11.5698	9.76298, 2	3	NA		36442
GL180424.1	19	1084	709, 590, 389	17, 11, 11	21.34551, 10.60895, 14.75772	11.95527, 6.9343, 9.09568	18.07159,	3	NA	NA	
GL180426.1	37848	38804	38270, 38307, 38151	21, 26, 15	25.14429, 29.19072, 18.95532	13.1674, 13.93821, 10.98488	21.75899,	3	NA	NA	
GL180427.1	36266	37307	36506, 36775, 36662	10, 9, 7	11.06011, 8.21099, 8.69489	7.306, 5.77858, 6.06379	8.22712, 5	3	NA	NA	
GL180428.1	196	806	451, 648, 639	8, 20, 8	8.42257, 21.34919, 10.1465	5.97763, 11.23615, 6.82176	5.76336, 1	3	NA	NA	
GL180428.1	37485	38409	37911, 37910, 37906	99, 120, 81	93.74068, 109.78372, 84.45515	17.88155, 17.41895, 20.32808	89.40501,	3	NA	NA	
GL180429.1	132	941	501, 641, 717	4, 9, 3	3.74195, 8.21099, 3.47371	3.32091, 5.77858, 3.03189	1.6222, 5.7	3	NA	NA	
GL180430.1	36544	37139	37037, 36677, 36770	5, 6, 4	4.82177, 4.93312, 4.67325	3.98509, 4.04501, 3.78987	2.51418, 2	3	NA	NA	
GL180432.1	183	3366	1407, 2123, 1709	19, 29, 15	15.14105, 20.91765, 12.99426	7.79671, 8.25887, 7.54975	12.1041, 1	3	NA	NA	
GL180432.1	6744	7636	7074, 6966, 6944	14, 14, 13	11.93818, 9.24978, 12.1463	7.14017, 5.49568, 7.49099	9.06106, 6	3	NA	NA	
GL180432.1	35766	36644	35825, 36049, 35818	8, 10, 7	8.42257, 9.39128, 8.69489	5.97763, 6.35644, 6.06379	5.76336, 6	3	NA	NA	
GL180434.1	35254	36600	35801, 36503, 35755	13, 7, 7	13.94585, 5.97725, 7.61369	8.5554, 4.62287, 5.54854	10.95967,	3	NA	NA	
GL180435.1	35560	36444	36250, 36309, 36326	12, 25, 18	10.03161, 20.98765, 17.25868	6.39767, 9.43748, 9.3554	7.26522, 1	3	NA	NA	
GL180435.1	37826	38246	37977, 37964, 38123	10, 17, 8	7.86015, 12.12978, 5.84174	5.41341, 6.53364, 4.43151	5.25709, 9	3	NA	NA	
GL180436.1	50	1313	736, 752, 734	33, 70, 33	36.65024, 77.90014, 41.92324	15.67117, 21.85227, 18.7845	33.00496,	3	NA	NA	
GL180436.1	37168	38253	38001, 38019, 37920	120, 172, 68	121.47155, 173.04121, 67.4108	21.18068, 22.33044, 17.61998	116.91183	3	NA	NA	
GL180437.1	34403	36831	35921, 36251, 35241	23, 22, 13	21.54503, 18.37326, 11.9555	10.40228, 8.84063, 7.37476	18.26496,	3	NA	NA	
GL180438.1	33435	33816	33622, 33784, 33747	11, 20, 7	9.13339, 19.03817, 7.50542	6.03321, 9.84499, 5.49244	6.43178, 1	3	NA	NA	
GL180439.1	15	902	585, 577, 605	119, 174, 88	114.70555, 163.85522, 94.80494	19.21245, 19.53777, 22.14425	110.19669	3	NA	NA	
GL180439.1	7444	8629	8134, 8112, 8181	29, 28, 14	23.89981, 19.38688, 11.64126	9.81875, 7.74736, 6.95702	20.54913,	3	NA	NA	
GL180439.1	37228	37459	37408, 37400, 37404	158, 238, 107	174.58148, 267.72833, 132.42383	26.86196, 29.99207, 30.95516	169.72684	3	NA	NA	
GL180441.1	652	1044	863, 866, 856	17, 23, 6	20.46701, 27.34036, 7.29403	11.48256, 13.85155, 5.30581	17.22179,	3	NA	NA	
GL180441.1	29954	30902	30656, 30615, 30126, 30684	21, 32, 11, 14	13.41168, 20.29681, 7.3919, 10.3731	6.3277, 7.26229, 4.94122, 6.17652	10.45602,	3	NA	NA	
GL180442.1	55	1207	515, 974, 648, 1061	9, 16, 4, 3	9.72082, 17.16856, 4.67325, 3.47371	6.64182, 9.82359, 3.78987, 3.03189	6.97155, 1	3	NA	NA	
GL180442.1	36296	36830	36567, 36533, 36448	7, 11, 3	5.33678, 9.39347, 3.47371	4.22299, 6.19476, 3.03189	2.98597, 6	3	NA	NA	
GL180445.1	138	1132	361, 345, 267	24, 37, 20	8.28653, 12.52681, 7.96861	3.59209, 3.87402, 3.87057	5.65362, 9	3	NA	NA	
GL180445.1	2239	2594	2376, 2374, 2428	17, 31, 18	4.19616, 8.67982, 6.40622	2.55162, 3.20663, 3.4049	2.03319, 6	3	NA	NA	
GL180445.1	27342	27708	27511, 27489, 27498	22, 23, 18	8.05473, 7.45235, 8.14666	3.69256, 3.37228, 4.2037	5.43842, 5	3	NA	NA	
GL180445.1	28875	30436	29932, 29015, 29548, 29034, 29932	34, 21, 37, 22, 27	17.0733, 6.27162, 17.41327, 11.30276, 15.65425	5.61911, 3.09126, 5.33945, 5.08869, 6.19492	13.95164,	3	NA	NA	
GL180445.1	31073	32099	31595, 31187, 31568, 31981	20, 27, 19, 18	6.77668, 10.016, 8.90543, 8.14666	3.37146, 3.93433, 4.42494, 4.2037	4.27263, 7	3	NA	NA	
GL180447.1	36920	38108	37837, 37516, 37479	28, 40, 47	30.78198, 42.7533, 58.95473	14.15193, 16.29291, 22.49293	27.25665,	3	NA	NA	
GL180450.1	58	781	192, 611, 319	12, 24, 12	9.86028, 18.4461, 12.15007	6.29117, 8.30413, 7.68238	7.1041, 15	3	NA	NA	
GL180450.1	2018	2759	2573, 2625, 2612	14, 36, 10	12.13106, 32.76568, 9.63365	7.25904, 12.29011, 6.50047	9.24462, 2	3	NA	NA	
GL180450.1	36799	37370	36918, 36899, 36911	14, 17, 8	16.23025, 18.56115, 10.1465	9.67496, 10.40145, 6.82176	13.14538,	3	NA	NA	
GL180454.1	78	685	320, 385, 348	12, 13, 4	13.8483, 13.14514, 4.67325	8.63436, 8.09002, 3.78987	10.86694,	3	NA	NA	
GL180456.1	8124	9040	8409, 8410, 8768, 8326	5, 10, 9, 6	3.27052, 6.40551, 5.51971, 5.02414	3.02971, 4.49295, 4.0845, 4.10097	1.36244, 4	3	NA	NA	
GL180459.1	26622	28150	27531, 26880, 27562	21, 23, 12	18.57212, 18.56726, 11.92663	9.25931, 8.65798, 7.55094	15.39198,	3	NA	NA	
GL180462.1	3484	4233	3989, 3583, 3543, 3992	16, 14, 4, 6	17.77392, 11.53986, 3.48964, 5.60548	10.17481, 6.89446, 3.17588, 4.44624	14.62581,	3	NA	NA	
GL180462.1	35325	35980	35812, 35411, 35720	12, 9, 8	13.8483, 8.21099, 10.1465	8.63436, 5.77858, 6.82176	10.86694,	3	NA	NA	
GL180466.1	49	732	486, 484, 456	90, 172, 70	95.13869, 189.63896, 82.2645	21.49487, 27.17072, 24.11679	90.79037,	3	NA	NA	
GL180466.1	36654	37509	37111, 36868, 36784	17, 22, 5	18.82666, 21.42357, 5.9506	10.55599, 10.65494, 4.54784	15.63723,	3	NA	NA	
GL180474.1	154	1416	868, 553, 560	15, 20, 11	17.6946, 20.84905, 12.78973	10.31995, 10.93703, 8.15619	14.54864,	3	NA	NA	
GL180474.1	34767	36026	35777, 35635, 35826	18, 17, 10	21.52103, 17.56014, 11.24131	11.86022, 9.80985, 7.40241	18.2418, 1	3	NA	NA	
GL180475.1	78	1081	667, 684, 681	138, 205, 108	125.95697, 187.49194, 103.27016	17.75601, 18.81189, 18.554	121.36761	3	NA	NA	
GL180475.1	36126	36883	36194, 36363, 36713	7, 13, 4	7.1696, 13.14514, 4.67325	5.31345, 8.09002, 3.78987	4.61046, 1	3	NA	NA	
GL180476.1	36736	37747	37494, 37141, 37101	26, 41, 16	32.99988, 48.43915, 20.29858	16.15999, 19.15447, 11.55461	29.42654,	3	NA	NA	
GL180486.1	47	1102	199, 210, 205	168, 193, 116	170.78828, 200.76074, 125.08887	22.73725, 24.39719, 23.8642	165.95541	3	NA	NA	
GL180486.1	34496	35695	35523, 35551, 35085	136, 128, 87	118.70534, 102.08369, 72.89465	16.28407, 13.72338, 13.99689	114.16666	3	NA	NA	
GL180486.1	36248	36877	36381, 36390, 36387	64, 100, 76	37.53406, 70.23283, 59.73981	7.72602, 10.74467, 12.24728	33.87185,	3	NA	NA	
GL180493.1	14	1595	736, 754, 1098	166, 226, 101	154.18282, 209.51543, 91.55566	18.88473, 19.54365, 16.57307	149.43599	3	NA	NA	
GL180493.1	33174	34508	33699, 33708, 33729	152, 136, 108	132.99309, 105.20158, 97.16382	16.62263, 13.1234, 16.56118	128.36237	3	NA	NA	
GL180493.1	35580	36252	36116, 36106, 35735	65, 102, 48	35.91554, 67.63483, 28.56552	7.17055, 9.8665, 7.44494	32.28389,	3	NA	NA	
GL180496.1	73	1486	499, 488, 488	40, 53, 26	41.11728, 56.22768, 29.12527	15.36511, 18.14227, 13.89927	37.39678,	3	NA	NA	
GL180496.1	17041	19107	18378, 18365, 17346	77, 75, 33	48.34523, 43.66187, 20.66961	8.74388, 7.85031, 7.21671	44.51619,	3	NA	NA	
GL180496.1	20444	20672	20624, 20622, 20622	33, 33, 21	8.23348, 7.44737, 6.3977	2.97643, 2.77461, 3.13893	5.60429, 5	3	NA	NA	
GL180496.1	20915	22615	21640, 21642, 22139	54, 62, 33	19.82067, 23.1883, 13.41265	4.43424, 4.60065, 4.48365	16.59695,	3	NA	NA	
GL180496.1	22889	26149	24362, 26112, 24350, 26112	56, 76, 56, 32	23.67251, 39.73032, 32.88712, 14.48521	5.13685, 6.84524, 7.55338, 4.95846	20.32878,	3	NA	NA	
GL180496.1	27684	28983	28843, 28826, 28857	54, 51, 40	34.81333, 28.0154, 27.2079	8.49008, 6.81699, 8.47726	31.20406,	3	NA	NA	
GL180498.1	2121	2914	2270, 2722, 2303	9, 17, 8	6.36774, 11.17157, 5.74302	4.62751, 5.97897, 4.36815	3.8942, 8.5	3	NA	NA	
GL180498.1	4302	5060	4471, 4456, 4458	11, 23, 7	8.33124, 16.95288, 4.48749	5.531, 7.75258, 3.67278	5.6958, 14	3	NA	NA	
GL180498.1	17576	18683	18158, 17973, 18015	36, 52, 39	35.93914, 52.26976, 44.2456	14.04716, 16.48516, 17.59082	32.30704,	3	NA	NA	
GL180498.1	36509	36901	36820, 36678, 36583	5, 8, 3	4.82177, 7.07159, 3.47371	3.98509, 5.20073, 3.03189	2.51418, 4	3	NA	NA	
GL180500.1	29	1189	416, 632, 566	51, 56, 35	50.6867, 53.84684, 35.69657	16.06933, 15.72874, 14.30323	46.82443,	3	NA	NA	
GL180502.1	100	726	217, 301, 319	107, 142, 78	112.71876, 141.69313, 84.27348	22.33453, 21.26407, 21.46522	108.22717	3	NA	NA	
GL180502.1	36276	36869	36485, 36411, 36727	25, 29, 14	27.23898, 30.99206, 16.12551	13.12872, 13.89775, 9.61761	23.79707,	3	NA	NA	
GL180503.1	35661	36454	36066, 36074, 35983	12, 18, 12	13.8483, 19.08138, 14.23156	8.63436, 10.4519, 8.83588	10.86694,	3	NA	NA	
GL180505.1	98	1303	742, 572, 699	15, 13, 9	18.12902, 13.14514, 11.64359	10.55412, 8.09002, 7.57973	14.96737,	3	NA	NA	
GL180506.1	72	1136	828, 636, 802	16, 19, 17	19.18518, 21.4153, 23.46806	10.96495, 11.55717, 13.01603	15.98366,	3	NA	NA	
GL180506.1	34962	36220	35625, 35714, 35514	21, 27, 25	24.68074, 32.02844, 33.10683	12.90176, 15.25977, 16.51459	21.30801,	3	NA	NA	
GL180507.1	141	2679	1221, 1230, 1238	67, 89, 63	57.71695, 82.91319, 60.87299	13.81675, 16.94674, 16.46702	53.75784,	3	NA	NA	
GL180507.1	7884	8632	7985, 8328, 8334	13, 15, 13	15.29169, 15.80067, 17.924	9.29854, 9.24574, 10.57401	12.24396,	3	NA	NA	
GL180507.1	35571	36361	35706, 35800, 35730	41, 49, 23	42.51865, 47.9978, 22.209	15.73986, 15.47005, 10.79929	38.77592,	3	NA	NA	
GL180511.1	105	1667	802, 991, 818	38, 44, 26	40.68875, 44.94072, 27.95127	15.95232, 15.84415, 13.1988	36.97522,	3	NA	NA	
GL180511.1	35818	36756	36064, 36056, 36189	14, 17, 7	16.7649, 18.56115, 8.69489	9.96273, 10.40145, 6.06379	13.6543, 1	3	NA	NA	
GL180513.1	7562	8547	8170, 8185, 7895	17, 19, 8	16.4953, 17.58589, 7.67847	9.16875, 9.26517, 5.56054	13.39926,	3	NA	NA	
GL180521.1	11	571	437, 100, 143	6, 16, 10	5.09949, 15.94397, 9.82971	4.14694, 9.09593, 6.61563	2.76939, 1	3	NA	NA	
GL180521.1	817	1470	917, 1287, 847	12, 12, 5	12.18267, 10.93692, 4.17451	7.70145, 6.95571, 3.60852	9.29304, 8	3	NA	NA	
GL180521.1	35478	36391	36128, 36316, 36292	19, 25, 16	19.72514, 25.11421, 17.40151	10.55748, 11.85753, 9.95951	16.50548,	3	NA	NA	
GL180522.1	35592	36763	36525, 36400, 36659	16, 26, 11	18.96503, 28.01936, 13.1143	10.84464, 13.2395, 8.32282	15.771, 24	3	NA	NA	
GL180525.1	21	579	396, 81, 83	12, 23, 11	13.55951, 26.6587, 14.68355	8.47903, 13.45462, 9.06344	10.59689,	3	NA	NA	
GL180525.1	34543	35287	34694, 34699, 34671	41, 54, 25	19.11378, 23.2522, 11.18216	5.40072, 5.20901, 4.59865	15.91507,	3	NA	NA	
GL180525.1	35555	36479	36279, 36129, 36106	98, 130, 67	76.61163, 97.6147, 52.97415	12.73028, 12.40691, 12.02724	72.44808,	3	NA	NA	
GL180526.1	2963	3600	2967, 3069, 3188	6, 13, 5	5.96715, 13.14514, 5.9506	4.64927, 8.09002, 4.54784	3.52158, 1	3	NA	NA	
GL180527.1	34200	34583	34534, 34556, 34516	27, 31, 21	36.25318, 40.09135, 31.13418	17.66791, 18.49147, 16.25469	32.61507,	3	NA	NA	
GL180539.1	154	511	274, 243, 447	13, 15, 12	10.71435, 9.60764, 10.50755	6.61004, 5.50146, 6.69224	7.91096, 7	3	NA	NA	
GL180539.1	711	1968	1080, 1539, 995, 1519	8, 24, 8, 11	5.55847, 18.97013, 6.15779, 9.36553	4.24931, 8.59603, 4.63309, 6.17745	3.18478, 1	3	NA	NA	
GL180539.1	711	2010	1823, 1539, 995, 1519	17, 24, 8, 11	15.39375, 18.97013, 6.15779, 9.36553	8.49863, 8.59603, 4.63309, 6.17745	12.34203,	3	NA	NA	
GL180539.1	30081	30607	30170, 30420, 30129	9, 12, 10	8.45482, 9.27893, 10.1418	5.92419, 5.92877, 6.79622	5.79307, 6	3	NA	NA	
GL180540.1	36005	36599	36126, 36221, 36106	32, 43, 18	35.81127, 49.31736, 22.99911	15.58082, 18.75496, 12.66051	32.18169,	3	NA	NA	
GL180541.1	11	765	173, 594, 506	32, 47, 19	33.29245, 46.56088, 22.81433	14.09301, 15.50516, 12.35676	29.71283,	3	NA	NA	
GL180542.1	23472	24920	24055, 24316, 23980	12, 9, 12	10.68367, 6.8008, 13.44151	6.80062, 4.90352, 8.41472	7.88119, 4	3	NA	NA	
GL180547.1	25287	25784	25651, 25453, 25682	6, 10, 7	5.96715, 9.39128, 8.69489	4.64927, 6.35644, 6.06379	3.52158, 6	3	c1qa		1491
GL180550.1	34236	35037	34448, 34440, 34454	18, 30, 15	22.49411, 36.43081, 19.64455	12.39242, 16.73931, 11.32852	19.18565,	3	NA	NA	
GL180551.1	35415	36469	36019, 35698, 35579	13, 17, 13	14.26982, 18.53926, 15.89249	8.73911, 10.38866, 9.61177	11.26966,	3	NA	NA	
GL180552.1	35526	36019	35953, 35576, 35766	7, 6, 4	7.1696, 4.93312, 4.67325	5.31345, 4.04501, 3.78987	4.61046, 2	3	NA	NA	
GL180553.1	21	985	86, 433, 76	13, 22, 14	13.34951, 23.62712, 16.29325	8.21021, 11.97865, 9.7093	10.39596,	3	NA	NA	
GL180553.1	35014	36431	35634, 35581, 35535	130, 176, 109	131.52902, 180.40317, 122.59958	21.50661, 23.30811, 25.58782	126.9073,	3	NA	NA	
GL180555.1	35587	36348	36097, 35985, 35686	16, 15, 7	19.7934, 15.80067, 8.69489	11.29109, 9.24574, 6.06379	16.57067,	3	NA	NA	
GL180557.1	35241	36322	35721, 35687, 35687	31, 34, 17	41.60549, 45.09532, 24.08167	19.35179, 20.22505, 13.29929	37.87739,	3	NA	NA	
GL180560.1	35510	36127	35689, 35761, 35650	7, 11, 4	7.1696, 10.60895, 4.67325	5.31345, 6.9343, 3.78987	4.61046, 7	3	NA	NA	
GL180562.1	22	537	151, 136, 130	11, 22, 9	12.43693, 25.85271, 11.64359	7.97018, 13.29074, 7.57973	9.52692, 2	3	NA	NA	
GL180562.1	34492	36371	34944, 35013, 34611, 35020, 36293	20, 31, 10, 10, 13	13.35762, 20.79031, 6.92496, 6.92496, 9.95131	6.52421, 7.70688, 4.82186, 4.82186, 6.13691	10.40375,	3	NA	NA	
GL180564.1	35263	36450	36087, 36113, 36328	24, 28, 19	26.97936, 31.93789, 22.42243	13.31777, 14.84014, 12.13591	23.5443, 2	3	NA	NA	
GL180567.1	1912	2941	2046, 2021, 2769, 2481	13, 6, 7, 9	12.56081, 4.93312, 5.97725, 9.76105	7.74168, 4.04501, 4.62287, 6.66343	9.64511, 2	3	NA	NA	
GL180567.1	35986	36624	36179, 36155, 36397	16, 22, 7	16.30427, 21.14817, 8.69489	9.31205, 10.48935, 6.06379	13.21554,	3	NA	NA	
GL180569.1	142	917	496, 443, 373	10, 15, 11	11.06011, 15.80067, 13.46208	7.306, 9.24574, 8.49639	8.22712, 1	3	NA	NA	
GL180569.1	35856	36452	35948, 36000, 36096	23, 21, 16	29.62834, 24.35402, 22.37427	15.14392, 12.71289, 12.56044	26.12932,	3	NA	NA	
GL180570.1	34523	35121	34785, 34771, 34624	5, 10, 5	4.82177, 9.39128, 5.9506	3.98509, 6.35644, 4.54784	2.51418, 6	3	NA		162
GL180580.1	113	849	715, 460, 564	10, 7, 17	11.06011, 5.97725, 22.63257	7.306, 4.62287, 12.61306	8.22712, 3	3	NA	NA	
GL180584.1	93	1049	453, 357, 541	91, 90, 62	99.13969, 99.11931, 72.47236	22.84482, 23.25228, 22.76605	94.75725,	3	NA	NA	
GL180584.1	35183	36245	35610, 35479, 35480	115, 113, 79	125.33994, 114.98963, 90.72444	24.33517, 21.12696, 24.15321	120.75429	3	NA	NA	
GL180585.1	35333	36203	36098, 35549, 35594	8, 13, 3	8.42257, 13.14514, 3.47371	5.97763, 8.09002, 3.03189	5.76336, 1	3	NA	NA	
GL180588.1	34680	36184	35865, 35234, 35887	19, 18, 11	23.28778, 19.41853, 13.83651	12.61993, 10.65157, 8.67735	19.95483,	3	NA	NA	
GL180589.1	6889	8088	7225, 7416, 7335	33, 30, 22	34.16129, 29.5375, 24.68033	14.207, 12.66196, 12.6046	30.56488,	3	NA	NA	
GL180589.1	35639	36144	35890, 35911, 35916	61, 96, 43	71.89362, 112.20706, 50.23222	22.93931, 26.6412, 19.28744	67.77869,	3	NA	NA	
GL180594.1	35324	36118	35740, 35864, 35592	8, 17, 6	8.42257, 18.56115, 7.29403	5.97763, 10.40145, 5.30581	5.76336, 1	3	NA	NA	
GL180596.1	19	2797	1531, 733, 2037	36, 45, 24	31.38783, 40.08017, 22.12811	11.5568, 12.93667, 10.42059	27.84896,	3	NA	NA	
GL180596.1	34604	35159	34867, 34721, 34720	29, 20, 22	34.75011, 22.87459, 28.97125	16.1291, 12.13503, 15.03088	31.14222,	3	NA	NA	
GL180597.1	26	679	324, 356, 497	14, 17, 9	16.7649, 18.56115, 11.64359	9.96273, 10.40145, 7.57973	13.6543, 1	3	NA	NA	

GL180597.1	22143	22909	22610, 22619, 22624	13, 14, 7	15.29169, 14.45897, 8.69489	9.29854, 8.66788, 6.06379	12.24396,	3	NA	NA
GL180598.1	118	850	421, 436, 208	21, 21, 14	23.81985, 23.50551, 17.01463	12.40141, 12.21671, 10.0942	20.47156,	3	NA	NA
GL180598.1	34945	36155	35355, 35378, 35353	65, 90, 69	63.07137, 81.10353, 72.73714	16.74629, 16.00533, 19.73039	59.05006,	3	NA	NA
GL180599.1	60	1054	791, 950, 430	13, 19, 5	11.87199, 14.30288, 4.91762	7.32374, 7.30478, 4.03768	8.9979, 11	3	NA	NA
GL180599.1	6399	7139	6963, 6916, 6646	13, 17, 8	11.07277, 13.01438, 7.90404	6.83197, 7.05741, 5.68997	8.23905, 1	3	NA	NA
GL180599.1	7441	7940	7521, 7776, 7630	10, 15, 7	7.78798, 10.90511, 6.75301	5.36798, 6.27326, 5.08141	5.18872, 8	3	NA	NA
GL180599.1	34022	35911	34415, 34388, 34664	130, 177, 116	92.03465, 125.34231, 95.37946	11.26446, 11.63752, 14.2654	87.71722,	3	NA	NA
GL180600.1	108	527	400, 303, 321	19, 25, 12	13.10886, 15.6003, 9.7119	6.62037, 6.57198, 6.19878	10.16657,	3	NA	NA
GL180600.1	8597	9281	8784, 8983, 9043	14, 22, 14	7.13563, 9.52408, 8.70643	4.27207, 4.29637, 5.17273	4.60231, 6	3	NA	NA
GL180600.1	32913	33667	33370, 33374, 33437	134, 166, 103	135.14635, 162.71886, 107.69244	21.49562, 21.0468, 21.80204	130.50302	3	NA	NA
GL180601.1	304	922	514, 75, 817, 778	8, 9, 8, 4	8.42257, 8.21099, 7.07159, 4.67325	5.97763, 5.77858, 5.20073, 3.78987	5.76336, 5	3	NA	NA
GL180601.1	6568	7288	6935, 6795, 6931	85, 108, 60	92.86337, 113.99238, 73.06524	22.50854, 22.47091, 24.09504	88.53705,	3	NA	NA
GL180601.1	35548	36192	35892, 35881, 35881	30, 36, 14	34.59393, 42.95786, 17.2091	15.65348, 18.14301, 10.19524	30.98841,	3	NA	NA
GL180602.1	34223	34816	34575, 34439, 34768	8, 10, 5	8.34715, 9.39128, 5.9506	5.9367, 6.35644, 4.54784	5.71011, 6	3	NA	NA
GL180603.1	19	766	218, 306, 249	62, 76, 60	67.06209, 84.88147, 68.73956	19.89152, 22.63083, 21.69691	62.99649,	3	NA	NA
GL180603.1	33451	34269	34219, 34234, 33694	66, 102, 48	68.65317, 109.97353, 54.56243	18.98891, 23.10023, 19.46828	64.57036,	3	NA	NA
GL180606.1	29075	29905	29691, 29762, 29692	11, 19, 8	10.1122, 18.68597, 8.35103	6.63573, 9.9315, 5.93881	7.34229, 1	3	NA	NA
GL180606.1	35991	36449	36134, 36259, 36202	18, 17, 8	22.49411, 18.56115, 9.8421	12.39242, 10.40145, 6.68602	19.18565,	3	NA	NA
GL180607.1	37	942	727, 628, 156, 472	11, 15, 12, 4	11.29029, 14.7819, 15.80834, 4.46488	7.3332, 8.63964, 9.60504, 3.69425	8.44541, 1	3	NA	0
GL180607.1	30908	31358	31304, 31027, 31013	16, 21, 15	10.21083, 12.83898, 11.4908	5.64338, 6.02823, 6.62849	7.43562, 1	3	xtr-mir-427-3	1915
GL180607.1	33299	34011	33747, 33665, 33364, 33642	16, 33, 8, 12	10.21083, 25.06817, 4.7202, 8.35467	5.64338, 9.25156, 3.70591, 5.35299	7.43562, 2	3	NA	0
GL180608.1	27	1000	663, 663, 825	21, 34, 10	26.14967, 43.10962, 13.18184	13.73288, 19.07472, 8.33771	22.7357, 3	3	NA	NA
GL180608.1	26414	27327	26634, 26709, 26594	19, 36, 26	13.17602, 24.3392, 24.56787	6.65828, 8.15786, 11.18558	10.23176,	3	NA	NA
GL180608.1	29389	30133	29811, 29983, 29737	21, 31, 10	12.20963, 15.75231, 5.1639	5.70662, 5.56158, 3.71781	9.31876, 1	3	NA	NA
GL180608.1	31908	32523	31999, 32252, 32195	14, 20, 14	7.0981, 9.5592, 10.16305	4.25125, 4.58706, 6.04815	4.5696, 6.9	3	NA	NA
GL180608.1	33306	34145	33953, 33829, 33958	17, 23, 13	11.72758, 13.72462, 10.24751	6.29903, 6.06644, 6.32053	8.86081, 1	3	NA	NA
GL180611.1	53	1325	1195, 923, 264	37, 40, 24	30.90577, 33.24358, 20.0886	10.984, 11.20826, 9.23065	27.37796,	3	U6	0
GL180611.1	35313	36032	35452, 35826, 35737	13, 27, 15	13.63968, 28.35229, 17.76194	8.37925, 13.07444, 10.35658	10.67258,	3	U6	0
GL180612.1	128	1112	1027, 748, 366	6, 14, 6	5.96715, 14.45897, 7.29403	4.64927, 8.66788, 5.30581	3.52158, 1	3	NA	NA
GL180615.1	26	1281	1035, 1099, 1087	47, 49, 28	40.08606, 34.98648, 23.38954	12.28636, 9.56835, 9.85052	36.38197,	3	NA	NA
GL180615.1	28713	29511	29145, 29020, 29381	20, 25, 20	24.58631, 26.96634, 26.49033	13.10866, 12.9658, 14.13188	21.21573,	3	NA	NA
GL180615.1	35022	36127	35719, 35546, 35543	127, 158, 78	140.84239, 173.36588, 90.70588	25.82748, 26.45882, 24.6175	136.16748	3	NA	NA
GL180617.1	573	1171	1040, 1044, 1034	12, 10, 9	13.73771, 9.39128, 10.14484	8.57523, 6.35644, 6.86555	10.76705,	3	NA	NA
GL180617.1	35143	35950	35279, 35526, 35268	23, 28, 16	27.0126, 30.69885, 19.45311	13.66126, 14.10238, 11.10978	23.57664,	3	NA	NA
GL180622.1	71	1198	1095, 321, 350, 815	12, 16, 6, 7	13.55951, 16.82622, 6.45058, 7.72582	8.47903, 9.62227, 4.90508, 5.6058	10.59689,	3	NA	NA
GL180622.1	35388	36082	35835, 35860, 35843	19, 15, 6	24.51879, 15.80067, 7.29403	13.28363, 9.24574, 5.30581	21.15031,	3	NA	NA
GL180625.1	46	961	841, 736, 730	55, 69, 23	53.52979, 64.99781, 21.62993	15.94907, 16.19426, 10.45294	49.62756,	3	NA	NA
GL180626.1	21742	22336	21926, 21865, 21851	10, 8, 9	11.06011, 7.07159, 11.64359	7.306, 5.20073, 7.57973	8.22712, 4	3	NA	NA
GL180627.1	31092	31627	31218, 31469, 31217	9, 11, 6	7.6342, 8.44639, 6.6567	5.42655, 5.60333, 5.00845	5.04509, 5	3	NA	NA
GL180627.1	32071	32701	32422, 32358, 32261	9, 16, 5	7.6342, 14.04087, 5.40789	5.42655, 7.93805, 4.29296	5.04509, 1	3	NA	NA
GL180629.1	24076	25080	24358, 24760, 24562	9, 8, 7	7.6342, 6.27644, 6.83761	5.42655, 4.70819, 5.12939	5.04509, 3	3	NA	NA
GL180629.1	33508	34721	33673, 34547, 33983	7, 16, 6	7.1696, 17.16856, 7.29403	5.31345, 9.82359, 5.30581	4.61046, 1	3	NA	NA
GL180634.1	33751	35127	34801, 34267, 34054	13, 12, 7	15.1723, 11.86108, 8.69489	9.23486, 7.51216, 6.06379	12.13305,	3	NA	NA
GL180637.1	12711	14279	13538, 13336, 13516	10, 16, 8	9.18236, 13.59227, 8.86315	6.23091, 7.66367, 6.21039	6.47811, 1	3	NA	10241
GL180637.1	19588	21093	20711, 20009, 20784	15, 15, 11	15.56593, 14.7819, 12.2694	9.10751, 8.63964, 7.88012	12.50679,	3	NA	3427
GL180641.1	20108	20991	20667, 20270, 20816	9, 13, 3	9.72082, 13.14514, 3.47371	6.64182, 8.09002, 3.03189	6.97155, 1	3	NA	NA
GL180642.1	2703	3036	2731, 2730, 2736	15, 20, 11	18.26603, 22.87459, 14.75772	10.62691, 12.13503, 9.09568	15.09729,	3	NA	NA
GL180651.1	8520	9308	8974, 8692, 8688	40, 53, 32	42.88338, 58.92083, 36.93673	16.36736, 19.60092, 16.24885	39.13538,	3	NA	NA
GL180657.1	197	1102	938, 827, 755	10, 10, 8	11.06011, 9.39128, 9.76268	7.306, 6.35644, 6.64964	8.22712, 6	3	NA	NA
GL180657.1	6975	7539	7433, 7459, 7380	19, 14, 10	18.26836, 13.8891, 8.18963	9.67872, 8.32957, 5.62006	15.09918,	3	NA	NA
GL180657.1	7699	8779	8397, 8281, 8096	17, 12, 27	13.31149, 9.09789, 29.13465	7.23535, 5.81578, 13.54141	10.36022,	3	NA	NA
GL180657.1	12888	13969	13147, 13629, 13392	21, 21, 17	12.62471, 11.63652, 13.06788	5.91784, 5.42083, 7.08939	9.70594, 8	3	NA	NA
GL180657.1	15424	16037	15700, 15682, 15723	24, 18, 16	13.32819, 6.97968, 9.43207	5.68574, 3.65984, 5.20195	10.37612,	3	NA	NA
GL180657.1	19872	21207	21055, 21044, 21039	38, 54, 33	33.5651, 45.49647, 29.2124	12.02005, 12.57535, 11.40624	29.98039,	3	NA	NA
GL180658.1	4275	4946	4566, 4631, 4651	9, 13, 9	9.72082, 13.14514, 11.64359	6.64182, 8.09002, 7.57973	6.97155, 1	3	NA	NA
GL180659.1	24580	25467	25265, 25314, 24973	11, 8, 10	12.43693, 7.07159, 13.18184	7.97018, 5.20073, 8.33771	9.52692, 4	3	NA	NA
GL180681.1	34	1602	536, 1034, 523	52, 59, 24	49.20884, 52.17746, 23.03411	14.94821, 13.93572, 10.95877	45.36741,	3	NA	NA
GL180683.1	22483	23208	22961, 22949, 22517	5, 8, 5	4.82177, 7.07159, 5.80393	3.98509, 5.20073, 4.48186	2.51418, 4	3	NA	NA
GL180684.1	7630	8576	7903, 7895, 7908	67, 82, 33	68.21305, 89.18166, 32.4193	18.37652, 22.07302, 13.19966	64.13533,	3	NA	NA
GL180686.1	12666	13419	13142, 13193, 13047	26, 35, 15	30.51196, 39.53695, 17.94632	14.72038, 16.5467, 10.45626	26.99282,	3	NA	NA
GL180687.1	16596	18472	17016, 17108, 18352, 17047, 18370	13, 29, 12, 18, 6	14.10549, 29.76556, 8.92355, 20.93229, 5.2665	8.64628, 13.17007, 5.70703, 11.52911, 4.24757	11.11201,	3	NA	NA
GL180689.1	1959	3023	2627, 2543, 2657	9, 16, 9	8.55802, 16.82622, 10.79992	5.98518, 9.62227, 7.19283	5.8909, 13	3	NA	NA
GL180692.1	10752	13740	11429, 11068, 12863	12, 21, 16	4.39212, 8.93532, 11.35397	3.03372, 4.16929, 6.31116	2.20411, 6	3	NA	NA
GL180692.1	10752	13740	11851, 11068, 12863	13, 21, 16	5.02996, 8.93532, 11.35397	3.26708, 4.16929, 6.31116	2.70624, 6	3	NA	NA
GL180692.1	11789	14681	12672, 13539, 12863	23, 23, 16	13.22526, 12.07799, 11.35397	5.82274, 5.28166, 6.31116	10.27715,	3	NA	NA
GL180692.1	11789	14681	14255, 13539, 12863	17, 23, 16	9.92396, 12.07799, 11.35397	5.28173, 5.28166, 6.31116	7.164, 9.35	3	NA	NA
GL180692.1	11789	14873	14746, 13539, 12863	10, 23, 16	4.4227, 12.07799, 11.35397	3.26897, 5.28166, 6.31116	2.2292, 9.3	3	NA	NA
GL180694.1	7130	7875	7652, 7761, 7557	9, 17, 9	5.24872, 8.60173, 5.57157	3.91147, 4.57941, 4.11768	2.90538, 6	3	NA	NA
GL180694.1	23878	24709	24525, 24507, 24440	52, 69, 17	58.10507, 75.09309, 18.80835	19.60941, 20.88728, 10.54536	54.14048,	3	NA	NA
GL180700.1	22621	23137	22950, 22950, 22651	16, 15, 4	19.7934, 15.80067, 4.67325	11.29109, 9.24574, 3.78987	16.57067,	3	NA	NA
GL180705.1	19788	20309	20083, 20061, 20061	11, 11, 15	9.00946, 6.72124, 16.75372	5.95596, 4.52362, 9.79627	6.31598, 4	3	NA	NA
GL180707.1	170	1501	1346, 604, 583	15, 16, 18	18.26603, 17.16856, 22.5396	10.62691, 9.82359, 12.41681	15.09729,	3	NA	NA
GL180707.1	6279	8081	7438, 7326, 7235	21, 18, 22	21.17819, 14.90085, 25.21195	10.82682, 7.92604, 12.91737	17.91116,	3	NA	NA
GL180708.1	7377	8321	8289, 8198, 8101	25, 32, 10	31.11602, 37.28684, 13.18184	15.4029, 16.45659, 8.33771	27.58298,	3	NA	NA
GL180713.1	8251	10019	8879, 9066, 9179	18, 26, 15	21.29566, 26.63135, 19.17716	11.73423, 12.41002, 11.09709	18.02512,	3	NA	NA
GL180715.1	20606	22337	21127, 21934, 20883, 21454, 22265	10, 17, 10, 9, 12	7.31658, 11.72902, 7.45013, 6.45932, 9.54032	5.07009, 6.29987, 5.15463, 4.68603, 6.09184	4.74811, 9	3	NA	NA
GL180715.1	21062	22337	22026, 21934, 21454, 22265	13, 17, 9, 12	10.46082, 11.72902, 6.45932, 9.54032	6.45284, 6.29987, 4.68603, 6.09184	7.67165, 9	3	NA	NA
GL180717.1	4357	5343	5130, 4677, 4931	6, 8, 8	5.96715, 7.07159, 10.1465	4.64927, 5.20073, 6.82176	3.52158, 4	3	NA	NA
GL180718.1	3889	4403	4088, 4161, 4203	15, 6, 10	14.98937, 4.93312, 12.52085	8.76428, 4.04501, 8.03986	11.95813,	3	NA	12018
GL180718.1	5919	6309	5989, 6067, 6061	8, 7, 6	6.70593, 5.97725, 6.87953	4.9768, 4.62287, 5.11627	4.20487, 3	3	NA	10112
GL180733.1	13041	13961	13658, 13473, 13772	8, 17, 7	8.42257, 18.56115, 8.69489	5.97763, 10.40145, 6.06379	5.76336, 1	3	NA	NA
GL180738.1	17000	18525	18416, 17500, 18331	10, 14, 10	11.06011, 14.45897, 12.33934	7.306, 8.66788, 7.95424	8.22712, 1	3	NA	NA
GL180740.1	11737	12530	12371, 12305, 12397	13, 11, 9	15.1723, 10.60895, 11.04428	9.23486, 6.9343, 7.30896	12.13305,	3	NA	NA
GL180741.1	9686	10276	9881, 9974, 9885	11, 21, 9	7.33946, 13.73307, 6.35947	4.9084, 6.4985, 4.62223	4.7691, 10	3	NA	NA
GL180741.1	13261	14215	13487, 13454, 13457	17, 24, 9	14.15399, 17.51797, 6.88962	7.74398, 7.79668, 4.95986	11.15907,	3	NA	NA
GL180742.1	19833	20480	20319, 20200, 20056	5, 7, 7	4.82177, 5.97725, 8.69489	3.98509, 4.62287, 6.06379	2.51418, 3	3	NA	NA
GL180744.1	28	838	335, 334, 354	21, 23, 9	27.77672, 27.36982, 11.64359	14.612, 13.8686, 7.57973	24.32202,	3	NA	NA
GL180747.1	42	954	509, 551, 646	12, 16, 19	13.22036, 16.51912, 23.65931	8.29296, 9.44018, 12.82349	10.27237,	3	NA	NA
GL180747.1	17340	18409	17599, 18122, 17646	9, 12, 10	6.22661, 9.13354, 9.40066	4.53723, 5.83804, 6.36205	3.7629, 6.5	3	NA	NA
GL180748.1	19519	20264	20065, 20066, 20026	37, 50, 23	34.02853, 40.69369, 21.2989	12.61461, 11.66078, 10.25559	30.43452,	3	NA	NA
GL180748.1	20925	21237	21077, 21094, 21093	12, 19, 12	6.6534, 9.25095, 8.67978	4.31552, 4.59074, 5.55511	4.15751, 6	3	NA	NA
GL180749.1	7028	8440	7540, 8326, 7271	11, 13, 8	9.34791, 10.04078, 7.34578	6.16652, 6.19235, 5.36526	6.63394, 7	3	NA	NA
GL180749.1	15711	16168	15805, 15766, 16102	6, 10, 7	4.48574, 7.35725, 6.75301	3.76346, 5.09584, 5.08141	2.28228, 4	3	NA	NA
GL180749.1	21904	22931	22116, 22763, 22299	13, 10, 13	12.94099, 8.04981, 13.84064	7.96907, 5.53254, 8.49513	10.00729,	3	NA	NA
GL180753.1	7393	9194	8541, 8517, 8490	37, 46, 34	34.57353, 42.68114, 35.65464	12.90945, 13.88315, 14.67894	30.96839,	3	NA	NA
GL180761.1	13125	13906	13261, 13256, 13242	10, 12, 9	10.46385, 11.86108, 10.72164	6.97883, 7.51216, 7.15493	7.67431, 9	3	NA	5618
GL180765.1	51	811	472, 544, 689	14, 26, 12	16.7649, 32.02458, 16.36823	9.96273, 15.60218, 9.85365	13.6543, 2	3	NA	NA
GL180779.1	19916	20980	20357, 20355, 20336	16, 14, 7	17.77392, 14.45897, 8.69489	10.17481, 8.66788, 6.06379	14.62581,	3	NA	NA
GL180791.1	13274	14108	13670, 13649, 13631	10, 10, 6	11.06011, 9.39128, 7.29403	7.306, 6.35644, 5.30581	8.22712, 6	3	NA	NA
GL180801.1	435	1381	469, 912, 786	7, 9, 5	7.1696, 8.21099, 5.9506	5.31345, 5.77858, 4.54784	4.61046, 5	3	NA	NA
GL180801.1	13341	14300	13821, 13800, 13798	9, 12, 10	9.72082, 11.86108, 13.18184	6.64182, 7.51216, 8.33771	6.97155, 9	3	NA	NA
GL180804.1	12961	13778	13448, 13257, 13709	39, 55, 29	46.18974, 64.3325, 35.97959	18.7337, 21.66691, 16.84875	42.3931, 6	3	NA	NA
GL180806.1	3488	3865	3778, 3736, 3681	10, 9, 9	8.0085, 8.01512, 7.86759	5.50663, 5.66016, 5.57018	5.39412, 5	3	NA	NA
GL180809.1	14983	15501	15331, 15340, 15354	21, 26, 9	21.64688, 25.11558, 10.01275	11.10892, 11.50872, 6.79683	18.3637, 2	3	NA	NA
GL180809.1	16674	17543	16980, 16930, 16769, 17434	12, 21, 5, 11	10.3005, 18.7699, 4.99237, 12.78973	6.56436, 9.37748, 4.0781, 8.15619	7.51999, 1	3	NA	NA
GL180815.1	94	646	491, 443, 320	11, 12, 11	12.33515, 11.86108, 12.06254	7.91559, 7.51216, 7.76744	9.43699, 9	3	NA	NA
GL180815.1	6442	8534	7939, 7249, 8316	24, 29, 22	19.67144, 24.37675, 21.14647	8.99221, 10.07904, 10.48833	16.45399,	3	NA	NA
GL180815.1	18541	19375	18905, 18696, 19346	7, 9, 6	7.1696, 8.21099, 7.29403	5.31345, 5.77858, 5.30581	4.61046, 5	3	NA	NA
GL180815.1	18937	19416	19291, 19283, 19346	11, 10, 6	12.43693, 9.39128, 7.29403	7.97018, 6.35644, 5.30581	9.52692, 6	3	NA	NA
GL180816.1	74	931	831, 818, 396	8, 13, 7	8.42257, 13.14514, 8.69489	5.97763, 8.09002, 6.06379	5.76336, 1	3	NA	NA
GL180823.1	65	933	252, 625, 768	11, 17, 11	12.43693, 18.56115, 14.75772	7.97018, 10.40145, 9.09568	9.52692, 1	3	NA	NA
GL180827.1	279	874	381, 417, 376	7, 11, 4	7.1696, 10.60895, 4.67325	5.31345, 6.9343, 3.78987	4.61046, 7	3	NA	NA
GL180827.1	20444	21135	20747, 20692, 20712	13, 19, 6	15.29169, 21.4153, 7.29403	9.29854, 11.55717, 5.30581	12.24396,	3	NA	NA
GL180836.1	13867	14274	13894, 13897, 13895	16, 9, 7	17.95866, 8.21099, 8.69489	10.28064, 5.77858, 6.06379	14.80292,	3	NA	NA
GL180838.1	55	1668	1296, 1280, 138, 1272	56, 98, 40, 45	22.3332, 44.06325, 16.57039, 20.35195	4.83695, 5.88872, 4.75156, 5.32193	19.03, 40.4	3	NA	NA
GL180838.1	1866	2567	2484, 2487, 2488	171, 222, 147	138.34467, 167.76312, 130.97708	14.5957, 13.25107, 17.12273	133.68488	3	NA	NA
GL180841.1	176	1758	836, 605, 243, 1391	13, 22, 11, 7	10.75783, 18.67604, 10.37715, 5.27202	6.63699, 9.01781, 6.79571, 4.18171	7.95226, 1	3	NA	NA
GL180841.1	2668	3425	3008, 3163, 2974	11, 10, 9	7.0723, 5.16247, 6.74947	4.74161, 3.71693, 4.87091	4.54399, 2	3	NA	NA
GL180841.1	8308	9584	8906, 9069, 9374	10, 8, 8	7.47854, 5.38416, 7.00671	5.1726, 4.13667, 5.16135	4.89879, 3	3	NA	NA
GL180846.1	14543	14966	14941, 14702, 14711	27, 29, 25	36.60899, 36.82147, 33.99218	17.86176, 17.33575, 16.99143	32.96455,	3	NA	NA
GL180857.1	8120	9389	8711, 8479, 8936	28, 33, 17	22.02194, 24.5574, 14.91739	9.10696, 9.00117, 8.20826	18.72698,	3	NA	NA
GL180857.1	14749	15535	15250, 15260, 15270	37, 35, 26	40.13301, 35.04275, 29.0371	16.05145, 13.92873, 13.84678	36.42825,	3	NA	NA
GL180862.1	103	1116	384, 384, 460	6, 15, 7	5.96715, 15.80067, 8.69489	4.64927, 9.24574, 6.06379	3.52158, 1	3	NA	NA
GL180865.1	828	1008	920, 931, 935	4, 6, 4	3.74195, 4.93312, 4.67325	3.32091, 4.04501, 3.78987	1.6222, 2.7	3	NA	NA
GL180868.1	17975	18780	18253, 18644, 18211, 18564	5, 14, 7, 8	4.82177, 14.45897, 8.69489, 10.1465	3.98509, 8.66788, 6.06379, 6.82176	2.51418, 1	3	NA	NA
GL180876.1	7594	8818	7902, 8087, 8090	18, 33, 16	19.69649, 34.83062, 18.69936	10.81516, 14.59849, 10.69793	16.47841,	3	NA	NA
GL180876.1	15992	17382	16823, 16521, 16823	27, 33, 16	30.77168, 33.61185, 18.1835	14.51687, 13.88731, 10.40854	27.24691,	3	NA	NA
GL180880.1	1494	2884	2039, 2036, 2045	123, 143, 111	133.70157, 152.17212, 128.03719	24.59361, 24.3073, 27.07328	129.06694	3	NA	NA
GL180880.1	13201	13963	13506, 13492, 13498	94, 113, 74	114.10654, 126.81258, 91.34003	28.52358, 25.71465, 26.95896	109.60251	3	NA	NA
GL180888.1	10717	10976	10867, 10738, 10874	5, 6, 6	4.82177, 3.97705, 7.29403	3.98509, 3.43246, 5.30581	2.51418, 1	3	NA	NA
GL180889.1	34	953	465, 338, 443	15, 25, 13	18.26603, 30.45643, 18.01082	10.62691, 15.02432, 10.61163	15.09729,	3	NA	NA
GL180893.1	16385	17721	17386, 16518, 17405, 16523, 17403	90, 32, 107, 21, 64	84.25713, 15.74665, 100.47321, 12.3787, 63.28003	17.14213, 5.41469, 17.89592, 5.79223, 17.21353	80.01775,	3	NA	6968
GL180894.1	3830	5459	4295, 4243, 4738	18, 27, 15	12.46786, 19.36779, 12.04749	6.49266, 7.98934, 6.96863	9.55566, 1	3	NA	NA
GL180896.1	114	784	402, 454, 385	52, 68, 44	35.129, 44.92204, 31.5328	8.98008, 9.19974, 9.347	31.51379,	3	NA	NA
GL180896.1	1956	2410	2288, 2279, 2267	45, 46, 27	27.69231, 23.3973, 14.43984	7.66009, 6.09345, 5.66008	24.24028,	3	NA	NA
GL180896.1	7657	7906	7775, 7769, 7784	60, 71, 30	59.78375, 66.31882, 28.33571	17.08026, 16.08627, 11.96657	55.7981, 6	3	NA	NA
GL180896.1	10951	11196	11076, 11102, 11083	19, 38, 15	12.34916, 30.08695, 11.69344	6.19698, 10.27851, 6.75208	9.45013, 2	3	NA	NA
GL180896.1	16888	18265	17365, 17072, 17413	15, 11, 13	15.12865, 9.66539, 14.46982	8.84764, 6.36265, 8.85107	12.09208,	3	NA	NA
GL180899.1	15367	16792	15950, 15797, 16597	8, 16, 5	7.83097, 16.82622, 5.9506	5.64832, 9.62227, 4.54784	5.22917, 1	3	NA	NA
GL180904.1	13008	13791	13262, 13105, 13115	17, 18, 19	20.46701, 18.60217, 25.66388	11.48256, 10.16604, 13.87136	17.22179,	3	NA	NA
GL180915.1	74	784	419, 401, 515	14, 15, 9	10.74704, 11.37503, 7.47813	6.40591, 6.55803, 5.32971	7.94177, 8	3	NA	NA
GL180915.1	4102	5444	5051, 4840, 4853	15, 19, 14	11.0217, 14.07215, 12.60452	6.34371, 7.1709, 7.55009	8.20168, 1	3	NA	NA
GL180917.1	15863	17596	16889, 16246, 16755	23, 38, 16	24.06243, 41.43693, 18.3512	11.91191, 16.3857, 10.50324	20.70741,	3	NA	NA
GL180922.1	8588	9210	9159, 9168, 9147	33, 37, 28	32.8285, 36.96367, 30.48566	13.43457, 14.23339, 13.97527	29.25864,	3	NA	NA
GL180922.1	11980	12547	12260, 12033, 12264	10, 19, 17	6.56907, 14.1101, 15.38528	4.59638, 7.19287, 8.49346	4.07951, 1	3	NA	NA
GL180932.1	16652	17703	17297, 17466, 17208	10, 21, 8	11.06011, 24.35402, 10.1465	7.306, 12.71289, 6.82176	8.22712, 2	3	NA	NA
GL180935.1	13675	14123	13844, 13855, 13975	9, 11, 11	9.72082, 10.60895, 14.75772	6.64182, 6.9343, 9.09568	6.97155, 7	3	NA	NA
GL180945.1	1	743	111, 458, 166	10, 10, 9	10.89158, 9.39128, 11.64359	7.21503, 6.35644, 7.57973	8.0782, 6.8	3	NA	NA
GL180945.1	16065	16647	16366, 16249, 16364	10, 15, 3	11.06011, 15.80067, 3.47371	7.306, 9.24574, 3.03189	8.22712, 1	3	NA	NA
GL180946.1	16460	16985	16617, 16726, 16796	11, 11, 8	12.43693, 10.60895, 10.1465	7.97018, 6.9343, 6.82176	9.52692, 7	3	NA	NA
GL180957.1	664	922	722, 720, 707	12, 26, 11	13.22036, 31.72888, 14.75772	8.29296, 15.43124, 9.09568	10.27237,	3	NA	NA
GL180957.1	9633	11191	10602, 10631, 10587	55, 85, 60	55.52768, 89.4459, 66.73173	16.94516, 20.9704, 20.61693	51.59761,	3	NA	NA
GL180959.1	9899	10208	10060, 10112, 10055	13, 18, 7	12.56081, 16.23409, 7.40077	7.74168, 8.73298, 5.43746	9.64511, 1	3	NA	NA
GL180965.1	14	514	249, 248, 249	44, 59, 27	50.42458, 64.36726, 30.97238	18.9395, 19.81775, 14.63598	46.56672,	3	NA	NA
GL180965.1	7995	8561	8111, 8189, 8127	18, 19, 10	14.7356, 13.35215, 7.50676	7.8267, 6.75805, 5.19045	11.71589,	3	NA	NA
GL180965.1	15691	16381	16115, 15935, 15976	15, 14, 5	13.10246, 9.66705, 4.28214	7.61619, 5.74681, 3.6736	10.16066,	3	NA	NA
GL180984.1	44	1599	469, 406, 200	12, 24, 6	13.8483, 26.87449, 7.29403	8.63436, 13.25553, 5.30581	10.86694,	3	NA	NA
GL180984.1	15016	15459	15210, 15348, 15198	10, 12, 11	11.06011, 11.60251, 13.46208	7.306, 7.3582, 8.49639	8.22712, 8	3	NA	NA
GL180994.1	951	1848	1604, 1675, 1231	8, 10, 8	8.42257, 9.39128, 9.31951	5.97763, 6.35644, 6.43944	5.76336, 6	3	NA	NA
GL181004.1	116	2626	1653, 1663, 1078, 2491	29, 30, 13, 11	15.96929, 15.69143, 6.96074, 4.7715	5.97043, 5.69114, 4.33277, 3.3545	12.89508,	3	NA	NA
GL181004.1	5094	6547	5654, 5808, 5832	30, 31, 20	17.56064, 18.19337, 13.20441	6.46835, 6.55094, 6.44044	14.41984,	3	NA	NA
GL181005.1	946	2263	2174, 1797, 1159, 2131	19, 23, 12, 17	8.35282, 9.51764, 7.20726, 9.30932	4.16566, 4.17119, 4.64828, 4.95024	5.71575, 6	3	NA	NA
GL181005.1	6114	6831	6706, 6711, 6293, 6708	27, 36, 15, 25	16.36707, 22.11556, 7.69044, 17.71977	6.52108, 7.22186, 4.4181, 7.64581	13.27624,	3	NA	NA
GL181013.1	591	1394	1060, 1148, 981	17, 19, 10	20.46701, 21.39089, 13.18184	11.48256, 11.54296, 8.33771	17.22179,	3	NA	12351
GL181014.1	2385	2827	2571, 2662, 2684	18, 19, 17	22.92104, 21.4153, 24.40832	12.61945, 11.55717, 13.44559	19.59912,	3	NA	NA
GL181015.1	7513	8244	7906, 8003, 8180	6, 15, 8	5.96715, 15.80067, 10.1465	4.64927, 9.24574, 6.82176	3.52158, 1	3	NA	NA
GL181018.1	16503	16842	16697, 16542, 16702	9, 7, 4	9.72082, 5.97725, 4.67325	6.64182, 4.62287, 3.78987	6.97155, 3	3	NA	NA
GL181028.1	15882	16257	16081, 16056, 16145	14, 19, 9	16.7649, 21.4153, 11.64359	9.96273, 11.55717, 7.57973	13.6543, 1	3	NA	NA
GL181032.1	3505	4563	3687, 3846, 4239	15, 19, 13	11.9085, 13.63158, 10.85814	6.88353, 6.91735, 6.69914	9.03357, 1	3	NA	NA
GL181034.1	1642	2462	1962, 1974, 1710	90, 118, 59	61.00827, 72.85262, 40.58591	10.0177, 9.03875, 9.47674	57.00882,	3	NA	NA
GL181034.1	3972	4795	4346, 4316, 4403	77, 130, 49	47.72346, 85.0172, 30.32855	8.5866, 9.95022, 7.86982	43.90342,	3	NA	NA
GL181037.1	8653	9316	8841, 8952, 8886	11, 8, 4	12.43693, 7.07159, 4.67325	7.97018, 5.20073, 3.78987	9.52692, 4	3	NA	NA
GL181045.1	407	1263	697, 605, 936	21, 27, 18	12.84129, 15.86781, 12.37176	6.02942, 6.2917, 6.43769	9.91269, 1	3	NA	NA
GL181045.1	3011	7603	3509, 3537, 3772, 4787, 6221	21, 35, 20, 16, 19	10.16123, 18.86502, 11.65025, 8.22991, 12.18374	4.71742, 6.12972, 5.61605, 4.54633, 6.10618	7.38865, 1	3	NA	NA
GL181045.1	3011	7603	6322, 3537, 3772, 4787, 6221	19, 35, 20, 16, 19	10.27191, 18.86502, 11.65025, 8.22991, 12.18374	5.09688, 6.12972, 5.61605, 4.54633, 6.10618	7.49265, 1	3	NA	NA
GL181046.1	14769	15740	15249, 14876, 15475	8, 9, 3	8.42257, 8.21099, 3.30894	5.97763, 5.77858, 2.9554	5.76336, 5	3	NA	NA
GL181050.1	12437	13216	12664, 12651, 13114	8, 9, 6	8.10827, 7.75488, 6.87953	5.80497, 5.501, 5.11627	5.48761, 5	3	NA	NA
GL181062.1	145	517	413, 472, 442	12, 18, 5	13.8483, 19.97713, 5.9506	8.63436, 10.97931, 4.54784	10.86694,	3	NA	NA
GL181077.1	9996	10779	10431, 10218, 10498	6, 10, 5	5.96715, 9.39128, 5.9506	4.64927, 6.35644, 4.54784	3.52158, 6	3	NA	NA
GL181084.1	1202	1833	1638, 1607, 1397	7, 8, 9	7.1696, 7.07159, 11.64359	5.31345, 5.20073, 7.57973	4.61046, 4	3	NA	NA
GL181084.1	8512	9253	9005, 8663, 8896	7, 19, 8	7.1696, 21.4153, 10.1465	5.31345, 11.55717, 6.82176	4.61046, 1	3	NA	NA
GL181098.1	2376	2894	2543, 2500, 2651	9, 10, 7	6.39666, 6.60892, 5.82684	4.646, 4.62161, 4.53047	3.92119, 4	3	NA	NA
GL181098.1	8588	9829	8870, 8860, 8862	25, 20, 23	27.62793, 21.17915, 28.81839	13.3608, 11.13464, 14.6944	24.17701,	3	NA	NA
GL181098.1	15370	16356	16075, 15778, 16227	56, 85, 48	64.89796, 95.71655, 58.29453	21.49983, 23.84319, 21.61876	60.8553, 9	3	NA	NA
GL181099.1	155	2805	916, 2376, 447, 2494	12, 20, 8, 12	9.38014, 16.16593, 6.97106, 11.7126	5.99192, 8.12509, 5.13965, 7.42393	6.66397, 1	3	NA	NA
GL181099.1	393	3016	2481, 2376, 447, 2494	15, 20, 8, 12	12.70921, 16.16593, 6.97106, 11.7126	7.37467, 8.12509, 5.13965, 7.42393	9.7865, 13	3	NA	NA
GL181105.1	15400	15999	15595, 15919, 15791	28, 26, 13	33.20707, 30.7371, 18.01082	15.59147, 14.8527, 10.61163	29.62933,	3	NA	7584
GL181107.1	8192	8532	8439, 8372, 8431	11, 9, 10	12.43693, 8.21099, 13.11399	7.97018, 5.77858, 8.30815	9.52692, 5	3	NA	NA
GL181108.1	1964	3614	2549, 2346, 3537, 2571, 3511	29, 29, 17, 30, 14	25.0786, 20.80248, 7.15576, 26.02447, 7.81143	10.46689, 8.20117, 3.85993, 10.66322, 4.65286	21.69609,	3	NA	NA
GL181108.1	7441	7870	7796, 7522, 7553	12, 22, 8	8.97894, 14.81631, 4.7202	5.74157, 6.84556, 3.70591	6.28831, 1	3	NA	NA
GL181108.1	13965	14644	14393, 14391, 14409	32, 46, 26	38.13301, 51.55863, 31.05589	16.95794, 18.67724, 15.03947	34.46145,	3	NA	NA
GL181113.1	27	532	234, 376, 221	9, 11, 8	9.72082, 10.48162, 10.1465	6.64182, 6.85833, 6.82176	6.97155, 7	3	NA	NA
GL181114.1	6256	7132	6395, 6518, 6382	24, 24, 15	32.24107, 28.90463, 21.38372	16.30582, 14.44646, 12.12757	28.68428,	3	NA	NA
GL181119.1	9457	11120	10094, 10637, 10101	52, 59, 40	48.6243, 53.08416, 39.57488	14.66367, 14.33277, 14.50717	44.79135,	3	NA	NA
GL181121.1	10287	11217	10585, 10525, 10778	11, 10, 7	10.84329, 8.36423, 7.05993	7.073, 5.72892, 5.25341	8.0324, 5.8	3	NA	NA
GL181125.1	9411	10802	9706, 9843, 9523	19, 35, 12	19.72514, 35.36826, 14.06375	10.55748, 14.11483, 8.74831	16.50548,	3	NA	NA
GL181138.1	10170	12658	10905, 11218, 11930	10, 13, 7	10.53228, 12.03475, 8.08824	7.01712, 7.4231, 5.7849	7.73927, 9	3	NA	NA
GL181138.1	10170	12658	11946, 11218, 11930	6, 13, 7	5.63898, 12.03475, 8.08824	4.46544, 7.4231, 5.7849	3.25772, 9	3	NA	NA
GL181153.1	6623	7442	6965, 6733, 7209	19, 22, 10	24.34652, 25.85271, 13.18184	13.19266, 13.29074, 8.33771	20.98338,	3	NA	NA
GL181153.1	12969	13945	13620, 13594, 13575	35, 37, 14	41.51413, 41.86434, 18.05504	17.71662, 17.06503, 10.62048	37.78754,	3	NA	NA
GL181164.1	15441	15882	15749, 15758, 15579	8, 22, 5	8.42257, 25.85271, 5.9506	5.97763, 13.29074, 4.54784	5.76336, 2	3	NA	NA
GL181166.1	24	445	374, 427, 289	5, 11, 5	4.82177, 10.60895, 5.9506	3.98509, 6.9343, 4.54784	2.51418, 7	3	NA	NA
GL181166.1	14920	15851	15372, 15414, 15367	7, 14, 13	7.1696, 14.45897, 18.01082	5.31345, 8.66788, 10.61163	4.61046, 1	3	NA	NA
GL181189.1	8432	8996	8792, 8599, 8742	7, 12, 6	7.1696, 11.86108, 7.29403	5.31345, 7.51216, 5.30581	4.61046, 9	3	NA	0
GL181192.1	353	941	389, 413, 395	15, 24, 11	15.64184, 25.03583, 12.41242	9.15231, 12.15722, 7.95707	12.57992,	3	NA	12514
GL181192.1	2284	2588	2348, 2322, 2326	14, 18, 12	13.20712, 15.95793, 13.01912	7.91811, 8.56528, 8.18077	10.26077,	3	NA	10867
GL181194.1	8282	9091	8672, 8621, 8607	11, 13, 14	12.43693, 13.14514, 19.68329	7.97018, 8.09002, 11.3696	9.52692, 1	3	NA	NA
GL181198.1	13351	14382	14121, 14272, 13879	21, 17, 10	20.24179, 15.13134, 10.3632	10.26221, 8.33865, 6.92219	17.00384,	3	NA	NA
GL181199.1	14725	15410	14817, 15263, 15078	6, 10, 4	5.96715, 9.39128, 4.67325	4.64927, 6.35644, 3.78987	3.52158, 6	3	NA	NA
GL181208.1	428	910	587, 553, 530	5, 8, 6	4.82177, 7.07159, 7.29403	3.98509, 5.20073, 5.30581	2.51418, 4	3	NA	NA
GL181209.1	2863	3235	2891, 2891, 2893	34, 44, 22	46.75436, 56.24067, 30.59363	21.16602, 22.34587, 15.87101	42.9496, 5	3	NA	NA
GL181224.1	2548	3038	2664, 2823, 2799	12, 20, 10	13.8483, 22.87459, 13.18184	8.63436, 12.13503, 8.33771	10.86694,	3	NA	NA
GL181225.1	74	855	407, 403, 446	5, 7, 6	4.82177, 5.97725, 7.29403	3.98509, 4.62287, 5.30581	2.51418, 3	3	NA	NA
GL181234.1	118	422	379, 373, 373	8, 13, 8	8.42257, 13.14514, 10.1465	5.97763, 8.09002, 6.82176	5.76336, 1	3	NA	8089
GL181234.1	6346	7419	6489, 6495, 6595	48, 65, 30	51.16983, 69.23492, 33.39226	17.57039, 19.69372, 14.93908	47.30198,	3	NA	1092
GL181234.1	12829	13651	13341, 12920, 12919	29, 30, 20	27.38073, 25.79298, 19.08737	11.77401, 10.53563, 9.87473	23.93478,	3	NA	2505
GL181235.1	201	2142	1579, 456, 1533, 475, 1305	16, 20, 19, 10, 9	12.58957, 14.3123, 13.28396, 9.01123, 7.86759	7.05281, 7.05533, 6.71936, 6.12719, 5.57018	9.6731, 11	3	NA	NA
GL181243.1	15	1822	537, 410, 121	15, 16, 9	15.71867, 15.41438, 10.56949	9.19755, 8.77591, 7.08032	12.65377,	3	NA	NA
GL181243.1	15	1822	1476, 410, 121	7, 16, 9	5.93788, 15.41438, 10.56949	4.59878, 8.77591, 7.08032	3.52158, 1	3	NA	NA
GL181244.1	6909	7457	6966, 6952, 6954	21, 42, 16	27.77672, 54.55851, 23.11042	14.612, 22.29595, 12.88555	24.32202,	3	NA	NA
GL181269.1	100	666	467, 520, 391	17, 19, 10	20.02116, 21.4153, 13.11399	11.236, 11.55717, 8.30815	16.79069,	3	NA	NA
GL181269.1	5618	7045	6393, 5909, 6404	18, 17, 14	19.00683, 16.3049, 15.80333	10.40758, 9.05325, 9.43935	15.81175,	3	NA	NA

GL181269.1	13246	14407	13588, 13453, 14335, 13456	25, 24, 12, 21	28.66822, 23.92411, 9.35334, 24.0973	13.97856, 11.49076, 5.9752, 12.56358	25.19216,	3	NA	NA
GL181272.1	2965	3288	3166, 3178, 3203	5, 14, 9	4.82177, 14.45897, 11.64359	3.98509, 8.66788, 7.57973	2.51418, 1	3	NA	NA
GL181274.1	14130	14956	14597, 14681, 14420	11, 9, 5	12.43693, 8.21099, 5.9506	7.97018, 5.77858, 4.54784	9.52692, 5	3	NA	NA
GL181280.1	113	952	662, 670, 191, 591	24, 33, 9, 19	24.77116, 34.47113, 8.34444, 21.69099	11.99852, 14.38797, 5.85853, 11.71707	21.39609,	3	NA	NA
GL181280.1	6591	7947	6756, 7222, 7572	7, 14, 12	5.7233, 10.29174, 12.38374	4.4663, 6.12676, 7.81847	3.33547, 7	3	NA	NA
GL181280.1	6984	7947	7124, 7222, 7644, 7572	8, 14, 17, 12	6.78332, 10.29174, 13.5057, 12.38374	5.02459, 6.12676, 7.35211, 7.81847	4.27811, 7	3	NA	NA
GL181280.1	12926	14057	13575, 13419, 13598	53, 69, 29	54.69189, 74.04757, 33.20194	17.33225, 20.36947, 15.21296	50.77323,	3	NA	NA
GL181282.1	7552	8678	7721, 7738, 7640	18, 25, 14	20.65644, 27.34151, 17.01463	11.37184, 13.18995, 10.0942	17.40599,	3	NA	NA
GL181286.1	14121	14933	14415, 14356, 14387	16, 19, 12	19.7934, 21.4153, 16.36823	11.29109, 11.55717, 9.85365	16.57067,	3	NA	NA
GL181294.1	12998	13545	13136, 13213, 13338	4, 7, 5	3.74195, 5.97725, 5.9506	3.32091, 4.62287, 4.54784	1.6222, 3.6	3	NA	0
GL181298.1	8031	8574	8419, 8492, 8348	7, 9, 8	7.1696, 8.21099, 10.1465	5.31345, 5.77858, 6.82176	4.61046, 5	3	NA	NA
GL181304.1	22	654	354, 470, 315	16, 12, 10	19.7934, 11.86108, 13.18184	11.29109, 7.51216, 8.33771	16.57067,	3	NA	NA
GL181304.1	6655	7303	6886, 6898, 7098	14, 21, 8	15.23546, 21.81372, 9.25066	9.11858, 11.20917, 6.40569	12.19387,	3	NA	NA
GL181315.1	75	802	434, 362, 506	10, 22, 13	11.06011, 25.85271, 18.01082	7.306, 13.29074, 10.61163	8.22712, 2	3	NA	NA
GL181315.1	6168	7254	6484, 6451, 6454, 7090	11, 27, 12, 9	11.56615, 28.70654, 14.96175, 10.56949	7.49066, 13.28584, 9.20442, 7.08032	8.70729, 2	3	NA	NA
GL181317.1	15381	16272	15776, 15557, 15481	8, 16, 8	8.42257, 17.16856, 10.0913	5.97763, 9.82359, 6.79758	5.76336, 1	3	NA	NA
GL181322.1	480	1467	1071, 1001, 859	14, 14, 8	8.73021, 7.70574, 6.50832	5.18676, 4.59259, 4.85389	6.05449, 5	3	NA	NA
GL181322.1	1816	2432	2026, 2024, 2205	32, 32, 11	27.9964, 25.4361, 9.36553	11.08134, 9.72327, 6.17745	24.53682,	3	NA	NA
GL181322.1	6974	7426	7071, 7102, 7255	8, 12, 8	4.86767, 7.75173, 8.24665	3.80166, 4.98057, 5.88165	2.55603, 5	3	NA	NA
GL181322.1	14286	15021	14599, 14499, 14506	192, 282, 117	209.36728, 306.91916, 134.04158	27.02952, 28.40714, 27.0777	204.33623	3	NA	NA
GL181338.1	8998	9689	9355, 9227, 9606	5, 9, 5	4.82177, 8.21099, 5.9506	3.98509, 5.77858, 4.54784	2.51418, 5	3	NA	NA
GL181386.1	6983	7929	7441, 7536, 7502	6, 8, 7	5.96715, 7.07159, 8.69489	4.64927, 5.20073, 6.06379	3.52158, 4	3	NA	NA
GL181398.1	5884	7134	6383, 6751, 6078	8, 10, 10	8.42257, 9.17452, 12.71002	5.97763, 6.22617, 8.12734	5.76336, 6	3	NA	NA
GL181398.1	5884	7153	7073, 6751, 6078	8, 10, 10	8.42257, 9.17452, 12.71002	5.97763, 6.22617, 8.12734	5.76336, 6	3	NA	NA
GL181412.1	1765	2545	2305, 2205, 2299	14, 11, 12	15.66744, 10.60895, 14.96175	9.36333, 6.9343, 9.20442	12.60415,	3	NA	NA
GL181424.1	7957	8338	7986, 7987, 8035	25, 33, 25	34.51774, 43.41463, 36.39062	17.26872, 19.64719, 18.21886	30.91362,	3	NA	NA
GL181440.1	6291	7344	6986, 6833, 6897	9, 12, 9	9.72082, 10.53798, 10.28145	6.64182, 6.71099, 6.93568	6.97155, 7	3	NA	NA
GL181452.1	7600	7951	7917, 7736, 7834	6, 8, 4	5.96715, 7.07159, 4.67325	4.64927, 5.20073, 3.78987	3.52158, 4	3	NA	NA
GL181456.1	6393	7393	7220, 6498, 6430, 7307	7, 12, 4, 4	7.1696, 11.86108, 4.67325, 4.67325	5.31345, 7.51216, 3.78987, 3.78987	4.61046, 9	3	NA	NA
GL181456.1	13926	14520	14277, 14373, 14379	10, 13, 7	10.01299, 13.14514, 8.69489	6.7221, 8.09002, 6.06379	7.24763, 1	3	NA	NA
GL181462.1	208	1062	436, 808, 414	12, 10, 6	13.8483, 9.39128, 7.29403	8.63436, 6.35644, 5.30581	10.86694,	3	NA	NA
GL181468.1	301	702	672, 667, 672	19, 25, 15	24.51879, 30.45643, 21.38372	13.28363, 15.02432, 12.12757	21.15031,	3	NA	NA
GL181474.1	4673	4972	4931, 4741, 4934	13, 8, 7	11.71272, 7.07159, 7.01416	7.22621, 5.20073, 5.22813	8.84616, 4	3	NA	NA
GL181474.1	8985	10447	9596, 9616, 9619	18, 19, 15	17.12119, 19.7239, 17.49481	9.27196, 10.55674, 10.21058	13.99654,	3	NA	NA
GL181476.1	237	1007	753, 777, 953	11, 10, 5	12.43693, 9.39128, 5.9506	7.97018, 6.35644, 4.54784	9.52692, 6	3	NA	NA
GL181477.1	343	2760	1439, 1160, 919, 2466	14, 30, 14, 12	9.22372, 22.77635, 12.16551, 9.88984	5.48009, 8.93219, 7.28026, 6.30956	6.5183, 19	3	NA	NA
GL181477.1	2704	4397	2971, 3772, 2466, 3427	13, 17, 12, 7	8.28401, 9.74886, 9.88984, 4.80868	5.11475, 5.18644, 6.30956, 3.8828	5.65156, 7	3	NA	NA
GL181477.1	2796	4451	3845, 3772, 3427	14, 17, 7	9.22372, 9.74886, 4.80868	5.48009, 5.18644, 3.8828	6.5183, 7.1	3	NA	NA
GL181488.1	6435	6896	6867, 6867, 6864	18, 27, 18	22.92104, 33.60851, 26.63681	12.61945, 16.18004, 14.40149	19.59912,	3	NA	NA
GL181489.1	51	3220	1771, 939, 1909	18, 24, 18	15.21293, 18.79215, 18.87511	8.11415, 8.49648, 10.32913	12.17279,	3	NA	NA
GL181489.1	8060	8787	8234, 8586, 8493	10, 19, 12	10.46385, 21.4153, 16.36823	6.97883, 11.55717, 9.85365	7.67431, 1	3	NA	NA
GL181495.1	13462	14166	13565, 13624, 14053	7, 10, 6	7.1696, 9.39128, 7.29403	5.31345, 6.35644, 5.30581	4.61046, 6	3	NA	NA
GL181502.1	13594	14318	14013, 13903, 13994	12, 16, 6	13.8483, 17.16856, 7.29403	8.63436, 9.82359, 5.30581	10.86694,	3	NA	NA
GL181508.1	92	829	450, 495, 432	14, 17, 11	16.7649, 18.56115, 14.75772	9.96273, 10.40145, 9.09568	13.6543, 1	3	NA	NA
GL181511.1	2143	4163	2952, 2990, 2358, 3382	16, 27, 6, 25	14.85827, 23.05196, 4.11531, 27.16884	8.4376, 9.9783, 3.52338, 13.08683	11.83222,	3	NA	NA
GL181523.1	13605	14223	13780, 13710, 13750	11, 17, 12	12.43693, 18.36654, 16.28773	7.97018, 10.2875, 9.81872	9.52692, 1	3	U1	0
GL181546.1	13431	14113	13839, 13690, 13830	6, 14, 11	5.96715, 14.45897, 14.75772	4.64927, 8.66788, 9.09568	3.52158, 1	3	NA	NA
GL181548.1	8658	9247	8816, 9031, 8807	8, 8, 6	5.37674, 5.40929, 4.85645	4.13187, 4.15294, 3.9975	3.02338, 3	3	NA	NA
GL181555.1	1009	1598	1172, 1345, 1530	9, 10, 4	9.72082, 7.97488, 4.67325	6.64182, 5.48553, 3.78987	6.97155, 5	3	NA	NA
GL181573.1	1302	3120	1427, 2234, 1556, 2539, 2891	14, 20, 10, 10, 13	8.99851, 12.94243, 7.56422, 7.56422, 10.78246	5.34572, 6.2982, 5.22677, 5.22677, 6.65226	6.30725, 1	3	NA	NA
GL181573.1	1340	3162	2913, 2234, 1556, 2539, 2891	18, 20, 10, 10, 13	12.95096, 12.94243, 7.56422, 7.56422, 10.78246	6.77125, 6.2982, 5.22677, 5.22677, 6.65226	10.01686,	3	NA	NA
GL181588.1	6584	6863	6597, 6755, 6726	5, 7, 4	4.82177, 5.97725, 4.67325	3.98509, 4.62287, 3.78987	2.51418, 3	3	NA	NA
GL181597.1	6750	7299	6837, 7235, 6821	7, 10, 7	7.1696, 9.39128, 8.69489	5.31345, 6.35644, 6.06379	4.61046, 6	3	NA	NA
GL181599.1	244	1175	375, 598, 955	5, 5, 9	4.82177, 3.94561, 11.64359	3.98509, 3.46715, 7.57973	2.51418, 1	3	NA	5904
GL181600.1	6117	7273	6629, 6363, 6820	4, 7, 5	3.74195, 5.97725, 5.9506	3.32091, 4.62287, 4.54784	1.6222, 3.6	3	NA	NA
GL181614.1	13157	13850	13472, 13291, 13559	20, 21, 17	25.24438, 24.35402, 23.32218	13.47022, 12.71289, 12.94709	21.85722,	3	NA	4379
GL181625.1	9074	10051	9614, 9765, 9576	8, 18, 15	8.42257, 19.97713, 20.414	5.97763, 10.97931, 11.69434	5.76336, 1	3	NA	NA
GL181637.1	13159	13868	13483, 13427, 13376	27, 46, 29	31.67873, 50.47916, 37.2397	15.05383, 18.06563, 17.5756	28.13373,	3	NA	NA
GL181650.1	106	641	357, 276, 413	8, 9, 10	8.42257, 8.21099, 13.18184	5.97763, 5.77858, 8.33771	5.76336, 5	3	NA	NA
GL181659.1	1718	2273	1756, 1739, 1807	13, 8, 4	15.07528, 7.07159, 4.67325	9.18277, 5.20073, 3.78987	12.04083,	3	NA	6093
GL181660.1	35	444	314, 397, 278	14, 14, 9	16.7649, 14.45897, 11.64359	9.96273, 8.66788, 7.57973	13.6543, 1	3	NA	NA
GL181661.1	12015	12642	12128, 12180, 12227	10, 9, 7	11.06011, 8.21099, 8.69489	7.306, 5.77858, 6.06379	8.22712, 5	3	NA	NA
GL181663.1	3645	5011	4387, 3938, 4559	10, 7, 6	11.06011, 5.97725, 7.29403	7.306, 4.62287, 5.30581	8.22712, 3	3	sft2d3	485
GL181664.1	13326	13762	13442, 13551, 13434	6, 11, 8	5.96715, 10.60895, 10.1465	4.64927, 6.9343, 6.82176	3.52158, 7	3	NA	NA
GL181666.1	282	1322	577, 777, 762	7, 10, 4	6.79162, 8.61706, 4.55201	5.10336, 5.8856, 3.73489	4.28406, 6	3	NA	NA
GL181666.1	1646	2523	2377, 1998, 1726	6, 8, 5	5.63898, 6.44699, 5.80393	4.46544, 4.8155, 4.48186	3.25772, 4	3	NA	NA
GL181676.1	4664	5138	4989, 4989, 4989	6, 9, 7	5.96715, 8.21099, 8.69489	4.64927, 5.77858, 6.06379	3.52158, 5	3	NA	NA
GL181679.1	298	974	772, 771, 303, 888	13, 21, 7, 9	13.63968, 23.12148, 7.96278, 10.56949	8.37925, 11.98982, 5.72394, 7.08032	10.67258,	3	NA	NA
GL181679.1	7595	8422	7944, 7778, 7923	21, 31, 15	18.13445, 28.47043, 14.99627	8.99874, 11.68765, 8.76842	14.9725, 2	3	NA	NA
GL181687.1	1738	2565	2283, 2253, 1977	19, 30, 15	15.8836, 26.83577, 13.39139	8.23864, 11.1149, 7.79346	12.81126,	3	NA	NA
GL181687.1	9949	10556	10217, 10424, 10401	28, 45, 21	19.3862, 32.64511, 15.91339	7.74702, 9.56715, 7.70404	16.17899,	3	NA	NA
GL181690.1	5976	6509	6227, 6165, 6162	9, 9, 4	9.72082, 8.21099, 4.67325	6.64182, 5.77858, 3.78987	6.97155, 5	3	NA	NA
GL181697.1	12697	13603	13400, 13087, 12994	20, 33, 16	19.42489, 30.76935, 17.40151	10.07876, 12.26546, 9.95951	16.21503,	3	NA	NA
GL181699.1	797	1770	1433, 1436, 1506	25, 16, 11	20.78629, 10.67955, 11.08949	9.32306, 5.9146, 7.21705	17.53143,	3	NA	NA
GL181703.1	9924	10538	10394, 10132, 10148	35, 44, 24	12.86383, 13.98111, 11.55777	4.12715, 3.77462, 4.8934	9.93412, 1	3	NA	NA
GL181703.1	11877	12666	12349, 12504, 12455	195, 289, 110	198.38831, 299.2182, 115.93394	23.29692, 25.41505, 22.50519	193.40663	3	NA	NA
GL181705.1	74	3336	2493, 2570, 249, 2454	159, 178, 111, 122	75.99876, 85.1638, 67.55226, 64.61939	6.58979, 6.6462, 8.79281, 7.34613	71.84212,	3	NA	NA
GL181705.1	3495	4672	3831, 3825, 3805	143, 145, 100	55.76309, 51.4036, 40.05716	5.1916, 4.71541, 5.19585	51.82994,	3	NA	NA
GL181705.1	5845	9142	6498, 6489, 6711	153, 142, 108	72.01146, 58.08099, 54.37688	6.45005, 5.46156, 6.80633	67.89549,	3	NA	NA
GL181716.1	8009	8476	8216, 8227, 8211	15, 17, 12	18.26603, 18.56115, 16.36823	10.62691, 10.40145, 9.85365	15.09729,	3	NA	NA
GL181722.1	27	1052	611, 641, 558	41, 70, 31	44.43035, 77.00881, 36.63922	16.82432, 21.40331, 16.4732	40.65937,	3	NA	NA
GL181722.1	7327	7778	7697, 7429, 7429	7, 7, 6	7.1696, 5.97725, 7.29403	5.31345, 4.62287, 5.30581	4.61046, 3	3	NA	NA
GL181748.1	8117	8632	8549, 8213, 8462	9, 8, 9	7.20928, 5.30997, 8.65301	5.16158, 4.08864, 6.04096	4.64652, 3	3	NA	NA
GL181769.1	11745	12405	12325, 12203, 12068	7, 10, 4	7.1696, 9.39128, 4.67325	5.31345, 6.35644, 3.78987	4.61046, 6	3	NA	NA
GL181782.1	12349	13197	12721, 12826, 12830	8, 19, 8	8.42257, 21.4153, 9.92329	5.97763, 11.55717, 6.7228	5.76336, 1	3	aebp2	5974
GL181784.1	1657	2245	2143, 2205, 2168	20, 46, 19	22.97335, 54.29951, 24.60107	12.19234, 20.25435, 13.32686	19.64963,	3	NA	NA
GL181785.1	3547	4155	3577, 3553, 3581	14, 23, 14	16.7649, 24.67931, 18.05504	9.96273, 12.28149, 10.62048	13.6543, 2	3	NA	NA
GL181789.1	10846	11416	11049, 11259, 11030	8, 10, 4	8.42257, 9.39128, 4.67325	5.97763, 6.35644, 3.78987	5.76336, 6	3	NA	NA
GL181805.1	7856	8287	8015, 8021, 8202	15, 26, 11	16.03559, 25.54817, 12.34038	9.3831, 11.76506, 7.91841	12.95858,	3	NA	NA
GL181818.1	12514	12876	12659, 12767, 12603	6, 9, 6	5.96715, 8.21099, 7.29403	4.64927, 5.77858, 5.30581	3.52158, 5	3	NA	NA
GL181830.1	2127	5399	3260, 3165, 2828	36, 29, 16	26.95947, 16.99076, 10.79843	9.34728, 6.4098, 5.98399	23.52526,	3	NA	NA
GL181830.1	4705	6379	5770, 3165, 6064, 2828, 6083	18, 29, 21, 16, 16	9.90793, 16.99076, 10.78828, 10.79843, 11.06979	5.08732, 6.4098, 5.01062, 5.98399, 6.14322	7.14919, 1	3	NA	NA
GL181835.1	12411	12762	12684, 12629, 12710	6, 12, 6	5.96715, 11.86108, 7.29403	4.64927, 7.51216, 5.30581	3.52158, 9	3	NA	NA
GL181838.1	146	615	347, 431, 310	9, 15, 7	9.72082, 15.80067, 8.69489	6.64182, 9.24574, 6.06379	6.97155, 1	3	NA	NA
GL181838.1	11248	11537	11369, 11421, 11338	11, 9, 6	6.87051, 4.16661, 4.03691	4.61612, 3.23024, 3.4719	4.35824, 2	3	NA	NA
GL181839.1	11553	12663	12132, 11609, 11883	10, 6, 6	11.06011, 4.93312, 7.29403	7.306, 4.04501, 5.30581	8.22712, 2	3	NA	NA
GL181854.1	928	1447	1261, 1077, 831, 1280	5, 6, 3, 4	4.82177, 4.93312, 3.47371, 4.67325	3.98509, 4.04501, 3.03189, 3.78987	2.51418, 2	3	NA	NA
GL181858.1	62	745	632, 656, 649	54, 54, 40	29.50207, 26.6687, 27.2079	6.84528, 6.06544, 8.47726	26.00632,	3	NA	1652
GL181858.1	4730	5424	5304, 5313, 5177	141, 141, 68	86.01885, 80.05032, 34.99661	9.04617, 8.1691, 6.60906	81.76038,	3	NA	912
GL181858.1	6179	7723	7096, 7071, 7091	168, 225, 148	120.55069, 177.12199, 127.17229	11.83617, 14.26715, 16.01081	115.99699	3	NA	2361
GL181873.1	6450	7132	7066, 7042, 7016	21, 35, 20	27.77672, 44.13349, 27.49151	14.612, 19.26198, 14.6397	24.32202,	3	NA	NA
GL181875.1	10826	11253	11113, 11044, 11032	11, 11, 5	9.90875, 9.4823, 5.80393	6.51186, 6.24973, 4.48186	7.14919, 6	3	NA	NA
GL181876.1	10240	10697	10591, 10563, 10460	16, 8, 7	19.7934, 7.07159, 8.69489	11.29109, 5.20073, 6.06379	16.57067,	3	NA	NA
GL181883.1	8301	9576	8866, 8862, 9092	28, 46, 23	28.77324, 47.65595, 24.80209	12.95575, 16.49884, 12.3549	25.29525,	3	NA	NA
GL181885.1	11530	12376	11702, 11723, 12202	15, 11, 14	18.26603, 10.60895, 19.68329	10.62691, 6.9343, 11.3696	15.09729,	3	NA	NA
GL181889.1	9589	10163	9868, 9867, 9818	9, 11, 5	9.72082, 10.60895, 5.9506	6.64182, 6.9343, 4.54784	6.97155, 7	3	NA	NA
GL181890.1	42	482	223, 234, 219	14, 21, 16	10.52208, 12.75782, 12.36386	6.26779, 5.98631, 6.91618	7.73027, 1	3	NA	NA
GL181890.1	1300	1633	1452, 1509, 1512	12, 14, 11	7.67828, 6.14576, 6.89643	4.93549, 3.73522, 4.63222	5.08599, 3	3	NA	NA
GL181890.1	10223	10843	10362, 10344, 10575	18, 34, 10	5.68938, 13.28853, 3.88695	3.09741, 4.34569, 2.95337	3.30385, 1	3	NA	NA
GL181890.1	11891	12289	12222, 12114, 12235	32, 39, 15	17.18707, 18.2006, 7.97976	5.96512, 5.35142, 4.57629	14.06087,	3	NA	NA
GL181933.1	11804	12232	12064, 12038, 11941	9, 18, 14	9.72082, 19.97713, 18.64934	6.64182, 10.97931, 10.90476	6.97155, 1	3	U4	6390
GL181945.1	67	1094	786, 805, 828	130, 162, 90	111.15211, 135.7415, 74.86757	15.5709, 15.46255, 13.91975	106.6713,	3	NA	563
GL181945.1	2272	2500	2397, 2368, 2375	51, 69, 64	17.09667, 23.86236, 33.5746	4.04377, 4.32369, 6.70346	13.97345,	3	NA	0
GL181945.1	6056	7769	6655, 6880, 6766	114, 140, 84	96.96442, 114.62526, 69.38995	15.10507, 14.55158, 13.5674	92.60046,	3	NA	1081
GL181955.1	104	2784	853, 786, 1937	57, 83, 59	28.36122, 45.80686, 40.85032	6.17447, 7.42256, 9.56574	24.89268,	3	NA	NA
GL181955.1	4474	5275	4535, 5050, 4739	71, 82, 45	40.85532, 45.05255, 26.76649	7.66486, 7.36747, 7.33374	37.1394, 4	3	NA	NA
GL181959.1	1342	1847	1813, 1829, 1803	35, 32, 14	44.29203, 37.52466, 17.83243	19.35493, 16.59762, 10.51088	40.52311,	3	NA	NA
GL181959.1	2367	3094	2672, 2637, 2561	6, 8, 6	4.48574, 5.99047, 6.45058	3.76346, 4.52662, 4.90508	2.28228, 3	3	NA	NA
GL181985.1	4230	5516	4619, 5072, 4769	20, 20, 7	24.21428, 20.53147, 7.96278	12.9009, 10.74631, 5.72394	20.85523,	3	NA	NA
GL181987.1	54	691	349, 404, 282	22, 46, 30	21.26442, 49.85961, 35.3138	10.55923, 17.71752, 16.08033	17.99497,	3	NA	NA
GL181987.1	9173	9838	9493, 9572, 9392	17, 34, 19	17.64386, 34.24377, 22.42243	9.85981, 13.86165, 12.13591	14.49984,	3	NA	NA
GL181989.1	3752	4744	3893, 4313, 3933, 4390	21, 44, 15, 23	9.65224, 23.88124, 8.32388, 15.60649	4.4858, 6.51809, 4.76705, 7.02724	6.92095, 2	3	NA	NA
GL181989.1	3752	4744	4386, 4313, 3933, 4390	24, 44, 15, 23	11.86625, 23.88124, 8.32388, 15.60649	5.02698, 6.51809, 4.76705, 7.02724	8.99294, 2	3	NA	NA
GL181989.1	9687	10119	9804, 9810, 9810	50, 95, 49	51.01946, 102.94359, 57.78624	16.64502, 22.88585, 20.84118	47.15327,	3	NA	NA
GL181990.1	3818	4289	3908, 3888, 4197	4, 8, 4	3.74195, 7.07159, 4.67325	3.32091, 5.20073, 3.78987	1.6222, 4.6	3	rxfp4	4373
GL182005.1	51	938	551, 607, 549	22, 34, 11	23.01801, 35.79005, 11.92945	11.61387, 14.75794, 7.69409	19.69328,	3	NA	NA
GL182008.1	2689	2894	2746, 2788, 2719	17, 25, 18	21.19088, 28.77122, 24.00763	11.87339, 14.03942, 13.17779	17.92307,	3	NA	NA
GL182020.1	861	1372	906, 909, 905	57, 76, 34	37.05813, 52.99244, 23.51138	8.68791, 10.16249, 8.25469	33.40579,	3	NA	NA
GL182020.1	1652	4630	2490, 2340, 2340, 4544	76, 72, 40, 18	54.75909, 46.28822, 28.74492, 7.84153	10.68089, 8.94091, 9.13064, 4.05824	50.8396, 4	3	NA	NA
GL182033.1	4401	5368	5081, 5141, 4810	43, 58, 24	50.35564, 67.21494, 28.82063	19.35949, 21.82068, 14.39789	46.49871,	3	NA	NA
GL182035.1	6940	7409	6989, 7198, 7312	9, 9, 5	9.72082, 8.21099, 5.9506	6.64182, 5.77858, 4.54784	6.97155, 5	3	NA	NA
GL182038.1	1200	1511	1276, 1324, 1272	35, 36, 19	44.29203, 45.45814, 25.95226	19.35493, 19.61818, 14.01451	40.52311,	3	NA	NA
GL182039.1	21	1150	391, 645, 393	35, 30, 23	25.86134, 19.23829, 19.09105	9.09465, 7.2144, 8.95885	22.45609,	3	5S_rRNA	0
GL182039.1	2387	3832	3255, 3022, 2720	45, 41, 26	36.58293, 30.39729, 22.19099	11.27791, 9.60299, 9.80908	32.93929,	3	5S_rRNA	0
GL182055.1	5381	5803	5485, 5676, 5581	6, 11, 4	5.96715, 10.60895, 4.67325	4.64927, 6.9343, 3.78987	3.52158, 7	3	GLMP	1730
GL182057.1	95	1052	269, 271, 309, 986	15, 23, 9, 8	11.42908, 18.56726, 7.37866, 6.21364	6.59091, 8.65798, 5.26769, 4.66848	8.57789, 1	3	NA	8651
GL182057.1	11580	12150	11822, 11784, 11826	47, 95, 36	35.76595, 84.83572, 30.08147	10.36904, 15.92997, 10.88058	32.13748,	3	NA	1784
GL182058.1	7567	9650	8854, 8875, 8851	211, 204, 124	199.3972, 180.57208, 120.7173	20.16837, 17.6237, 19.74321	194.41077	3	NA	NA
GL182067.1	4103	4644	4411, 4192, 4414	6, 10, 4	5.96715, 9.39128, 4.67325	4.64927, 6.35644, 3.78987	3.52158, 6	3	NA	NA
GL182067.1	9196	10308	9962, 9582, 9616	8, 13, 6	8.42257, 13.14514, 7.29403	5.97763, 8.09002, 5.30581	5.76336, 1	3	NA	NA
GL182073.1	10996	11791	11386, 11386, 11386	14, 15, 12	16.7649, 15.80067, 16.36823	9.96273, 9.24574, 9.85365	13.6543, 1	3	NA	NA
GL182095.1	35	371	172, 158, 181	9, 12, 9	9.72082, 11.86108, 11.64359	6.64182, 7.51216, 7.57973	6.97155, 9	3	NA	NA
GL182095.1	10313	10647	10482, 10494, 10504	14, 11, 7	16.7649, 10.60895, 8.69489	9.96273, 6.9343, 6.06379	13.6543, 7	3	NA	NA
GL182105.1	533	997	853, 955, 783	8, 9, 5	8.42257, 8.21099, 5.9506	5.97763, 5.77858, 4.54784	5.76336, 5	3	NA	NA
GL182121.1	530	1532	1080, 1221, 1007	5, 5, 7	4.82177, 3.85883, 8.646	3.98509, 3.41255, 6.04229	2.51418, 1	3	NA	850
GL182127.1	40	1046	482, 492, 125	17, 26, 10	13.78629, 19.07292, 8.77043	7.52135, 8.09769, 5.98002	10.81362,	3	NA	NA
GL182127.1	6815	7817	7018, 7400, 7199	16, 24, 10	7.38668, 10.30633, 5.54622	4.1073, 4.37087, 3.95398	4.81337, 7	3	NA	NA
GL182127.1	8685	9344	9090, 9115, 9073	28, 41, 19	19.71425, 27.46899, 14.93788	7.91058, 8.3647, 7.67672	16.49544,	3	NA	NA
GL182155.1	1369	1666	1540, 1602, 1555	47, 85, 32	34.14909, 66.39975, 27.82735	9.70214, 12.39561, 10.98932	30.55287,	3	NA	NA
GL182155.1	2799	3159	2983, 2971, 2959	46, 55, 20	31.76665, 32.5848, 12.7645	8.98462, 7.61347, 6.20233	28.22028,	3	NA	NA
GL182155.1	8403	9198	8559, 9013, 8452, 9127	23, 18, 10, 6	15.50146, 8.72156, 6.33516, 3.20762	6.97193, 4.48396, 4.44852, 2.91777	12.4454, 6	3	NA	NA
GL182159.1	351	1309	573, 578, 1235	12, 16, 9	6.004, 7.70223, 5.94272	3.9337, 4.26948, 4.35539	3.55662, 5	3	NA	NA
GL182159.1	351	1309	1183, 578, 1235	16, 16, 9	9.3283, 7.70223, 5.94272	5.14407, 4.26948, 4.35539	6.61657, 5	3	NA	NA
GL182159.1	3332	3772	3542, 3548, 3597	18, 17, 10	10.422, 7.983, 6.59592	5.35879, 4.26511, 4.61338	7.63516, 5	3	NA	NA
GL182159.1	9703	10273	10032, 10028, 9862	9, 22, 5	9.72082, 24.56226, 5.9506	6.64182, 12.53481, 4.54784	6.97155, 2	3	NA	NA
GL182164.1	445	3388	843, 2030, 659, 2494	11, 26, 7, 14	8.19386, 21.45465, 4.98481, 12.51398	5.44467, 9.39378, 3.99709, 7.49454	5.56766, 1	3	NA	NA
GL182164.1	445	3388	2743, 2030, 659, 2494	18, 26, 7, 14	16.04928, 21.45465, 4.98481, 12.51398	8.62074, 9.39378, 3.99709, 7.49454	12.97113,	3	NA	NA
GL182188.1	7535	7962	7677, 7788, 7893	9, 9, 8	9.72082, 8.21099, 10.1465	6.64182, 5.77858, 6.82176	6.97155, 5	3	NA	NA
GL182189.1	7549	9951	8427, 8384, 8488	72, 93, 66	60.54116, 78.66196, 63.4317	13.51062, 14.41608, 16.54911	56.54783,	3	NA	NA
GL182192.1	5608	6238	5739, 5936, 5650	10, 10, 4	9.60506, 8.98028, 4.38152	6.48358, 6.10835, 3.65448	6.87658, 6	3	NA	NA
GL182193.1	10310	11181	10861, 10746, 10664	32, 52, 18	32.19237, 52.9601, 19.23004	13.4508, 16.84219, 10.5401	28.63672,	3	NA	NA
GL182219.1	2237	3252	2453, 2747, 2658	16, 24, 9	16.75964, 25.7315, 11.21577	9.5829, 12.57396, 7.38849	13.65293,	3	NA	NA
GL182219.1	10427	10899	10726, 10721, 10706	5, 15, 11	4.82177, 15.80067, 14.75772	3.98509, 9.24574, 9.09568	2.51418, 1	3	NA	NA
GL182238.1	5640	6194	5827, 5832, 6027	7, 14, 10	6.60038, 14.45897, 12.33934	4.99378, 8.66788, 7.95424	4.10761, 1	3	NA	NA
GL182250.1	75	1374	564, 700, 1187	13, 14, 7	14.10549, 14.45897, 8.69489	8.64628, 8.66788, 6.06379	11.11201,	3	NA	NA
GL182250.1	8378	9002	8906, 8753, 8867	5, 11, 5	4.82177, 10.60895, 5.9506	3.98509, 6.9343, 4.54784	2.51418, 7	3	NA	NA
GL182254.1	49	1801	890, 1219, 700	23, 29, 16	24.06243, 28.20633, 17.70367	11.91191, 12.25351, 10.13439	20.70741,	3	NA	NA
GL182255.1	10283	10792	10639, 10645, 10376	10, 10, 4	11.06011, 9.39128, 4.67325	7.306, 6.35644, 3.78987	8.22712, 6	3	NA	NA
GL182276.1	5161	5962	5581, 5601, 5564	185, 200, 116	197.84663, 216.16866, 134.93851	25.73917, 26.67803, 27.88483	192.86885	3	NA	NA
GL182279.1	67	1289	439, 567, 837	21, 26, 11	20.44908, 24.16991, 9.92808	10.38721, 10.95194, 6.52366	17.20509,	3	NA	NA
GL182279.1	2346	2872	2615, 2556, 2702	11, 9, 13	8.54584, 5.47555, 12.34408	5.66575, 4.05627, 7.61094	5.88025, 3	3	NA	NA
GL182279.1	9617	10269	10076, 10087, 10211	19, 23, 5	23.53998, 26.88069, 5.9506	12.7584, 13.58438, 4.54784	20.2, 23.63	3	NA	NA
GL182286.1	309	1131	526, 638, 326, 758	9, 13, 8, 8	9.72082, 13.14514, 9.31951, 9.31951	6.64182, 8.09002, 6.43944, 6.43944	6.97155, 1	3	NA	NA
GL182299.1	8962	10230	9513, 9480, 9513	12, 28, 15	13.8483, 30.12516, 16.30311	8.63436, 13.76034, 9.53825	10.86694,	3	NA	NA
GL182321.1	95	2166	558, 1722, 485	22, 29, 14	15.58718, 16.70238, 9.39688	7.26198, 6.28398, 5.58393	12.5275, 1	3	NA	NA
GL182321.1	3826	4271	4004, 3993, 4016	23, 18, 23	13.98966, 7.11439, 15.10466	6.19775, 3.7209, 6.7647	11.00144,	3	NA	NA
GL182321.1	5311	7373	6358, 5570, 5985, 6768, 6040	15, 18, 17, 14, 18	9.15146, 7.93882, 8.03488, 7.24449, 11.50304	5.23666, 4.10437, 4.2911, 4.33266, 5.94796	6.44964, 5	3	NA	NA
GL182334.1	796	1340	1134, 1128, 1018	7, 10, 4	6.99564, 9.39128, 4.55201	5.21786, 6.35644, 3.73489	4.47307, 6	3	NA	NA
GL182342.1	8795	9699	9540, 9538, 9597	10, 21, 14	9.18236, 20.96503, 17.3087	6.23091, 10.69835, 10.24653	6.47811, 1	3	NA	NA
GL182344.1	117	1203	1053, 764, 869	61, 84, 52	37.45587, 53.02016, 38.327	8.14361, 8.92924, 10.16881	33.79546,	3	NA	NA
GL182344.1	3739	4585	4041, 4002, 3985	79, 105, 47	38.29309, 53.47069, 22.74341	6.27575, 6.8842, 5.77982	34.6189, 4	3	NA	NA
GL182344.1	6022	7221	6551, 6544, 6804	105, 115, 60	72.64021, 75.47213, 41.91431	10.56839, 9.86407, 9.72517	68.5186, 7	3	NA	NA
GL182346.1	9013	9610	9374, 9098, 9051	12, 16, 9	13.8483, 17.16856, 11.64359	8.63436, 9.82359, 7.57973	10.86694,	3	NA	NA
GL182358.1	2100	3043	2443, 2871, 2398, 2841	14, 34, 10, 12	12.33072, 33.53045, 9.63365, 12.15007	7.38193, 13.45313, 6.50047, 7.68238	9.43363, 3	3	NA	NA
GL182358.1	9973	10634	10478, 10440, 10331	15, 16, 12	16.90423, 15.94397, 14.76955	9.88146, 9.09593, 9.10943	13.78827,	3	NA	NA
GL182360.1	82	1172	425, 348, 547	24, 31, 15	28.29526, 36.71719, 20.69165	14.09251, 16.51936, 11.82159	24.82836,	3	NA	NA
GL182363.1	11	511	88, 280, 287	16, 31, 17	12.63122, 26.78326, 17.07467	7.07806, 10.75098, 9.51876	9.71235, 2	3	NA	NA
GL182383.1	10177	10526	10267, 10433, 10269	4, 11, 9	3.74195, 10.60895, 11.64359	3.32091, 6.9343, 7.57973	1.6222, 7.9	3	NA	NA
GL182385.1	180	1168	871, 921, 723	21, 27, 13	14.35601, 17.57251, 8.9147	6.83489, 7.09279, 5.49802	11.3521, 1	3	U1	0
GL182385.1	1950	2808	2162, 2053, 2052	15, 19, 11	8.68619, 10.21152, 7.0257	4.97083, 5.06628, 4.71259	6.01254, 7	3	U1	0
GL182385.1	1950	2844	2784, 2053, 2052	15, 19, 11	8.68619, 10.21152, 7.0257	4.97083, 5.06628, 4.71259	6.01254, 7	3	U1	0
GL182385.1	3193	3502	3312, 3323, 3276	11, 11, 8	5.41054, 4.21752, 4.45581	3.72812, 3.03977, 3.53444	3.05259, 2	3	U1	3
GL182387.1	9651	10368	9873, 9919, 9885	11, 26, 8	12.01255, 27.47779, 9.1831	7.73996, 12.91577, 6.37229	9.13199, 2	3	NA	NA
GL182395.1	21	337	123, 139, 307	10, 13, 4	10.67246, 13.14514, 4.67325	7.09497, 8.09002, 3.78987	7.87096, 1	3	NA	NA
GL182395.1	485	787	756, 614, 563	6, 7, 3	5.72604, 5.97725, 3.47371	4.51498, 4.62287, 3.03189	3.33547, 3	3	NA	NA
GL182400.1	7162	7485	7253, 7261, 7292	7, 6, 4	7.1696, 4.93312, 4.67325	5.31345, 4.04501, 3.78987	4.61046, 2	3	NA	NA
GL182415.1	3372	4291	3787, 3705, 3430	19, 24, 6	16.18209, 19.43052, 4.91111	8.41756, 8.85543, 4.03141	13.09924,	3	NA	NA
GL182415.1	9686	10384	10133, 10132, 10013	12, 17, 8	13.8483, 18.56115, 10.1465	8.63436, 10.40145, 6.82176	10.86694,	3	NA	NA
GL182422.1	9440	10336	9538, 10110, 9516, 10136	12, 23, 6, 9	11.32055, 23.03769, 6.76586, 10.87975	7.18871, 11.29653, 5.06179, 7.23113	8.47391, 1	3	NA	NA
GL182422.1	9440	10340	10139, 10110, 9516, 10136	15, 23, 6, 9	15.12865, 23.03769, 6.76586, 10.87975	8.84764, 11.29653, 5.06179, 7.23113	12.09208,	3	NA	NA
GL182429.1	326	971	870, 594, 672	13, 10, 9	15.1723, 9.39128, 11.21577	9.23486, 6.35644, 7.38849	12.13305,	3	NA	NA
GL182429.1	8135	8778	8665, 8544, 8661	13, 13, 18	13.63968, 11.81904, 19.91385	8.37925, 7.29135, 10.94239	10.67258,	3	NA	NA
GL182434.1	48	984	140, 258, 206, 846	14, 26, 10, 5	13.70063, 23.91458, 11.10454, 4.84526	8.21651, 10.80262, 7.32977, 3.99806	10.73143,	3	NA	NA
GL182434.1	81	984	737, 258, 206, 846	8, 26, 10, 5	6.63031, 23.91458, 11.10454, 4.84526	4.92991, 10.80262, 7.32977, 3.99806	4.13604, 2	3	NA	NA
GL182434.1	9966	10642	10079, 10018, 10081	9, 14, 7	8.66407, 11.80872, 7.50542	6.04743, 7.06033, 5.49244	5.99101, 9	3	NA	NA
GL182439.1	164	951	600, 332, 733	11, 21, 8	4.99847, 10.50717, 3.83671	3.48596, 4.87811, 3.13548	2.6769, 7.8	3	U1	0
GL182439.1	164	1040	873, 332, 733	9, 21, 8	3.63599, 10.50717, 3.83671	2.90497, 4.87811, 3.13548	1.6222, 7.8	3	U1	0
GL182439.1	1908	2593	2409, 2266, 2037	20, 18, 12	12.57417, 8.16583, 7.00989	6.10044, 4.21291, 4.52903	9.65797, 5	3	U1	39
GL182439.1	2942	3491	3081, 3120, 3350	14, 18, 15	7.28888, 8.16583, 9.73181	4.35745, 4.21291, 5.57419	4.72176, 5	3	U1	0
GL182440.1	8229	9788	8580, 9371, 8709, 9341	11, 9, 6, 6	12.43693, 8.21099, 7.29403, 7.29403	7.97018, 5.77858, 5.30581, 5.30581	9.52692, 5	3	NA	NA
GL182446.1	329	1395	1196, 830, 808	33, 34, 29	16.04077, 14.46362, 16.40573	5.38422, 4.72015, 6.15601	12.96312,	3	NA	NA
GL182446.1	2350	5129	4853, 4848, 4426	69, 74, 55	51.56902, 51.40518, 43.48501	11.08516, 10.05683, 11.4566	47.69535,	3	NA	NA
GL182449.1	11	662	109, 166, 151	18, 22, 10	22.23921, 25.8245, 13.18184	12.25494, 13.2744, 8.33771	18.93827,	3	NA	NA
GL182451.1	3386	4185	3928, 3747, 3829	4, 9, 5	3.74195, 8.21099, 5.9506	3.32091, 5.77858, 4.54784	1.6222, 5.7	3	NA	NA
GL182471.1	58	2717	1313, 768, 962	60, 54, 43	53.07888, 39.86707, 39.15946	14.01021, 10.29031, 13.16717	49.18325,	3	NA	NA
GL182481.1	9272	10205	9432, 9657, 9594	15, 22, 11	16.72085, 22.94165, 12.63525	9.7776, 11.56804, 8.07536	13.61562,	3	NA	NA
GL182483.1	6612	7128	6966, 6970, 6958	5, 17, 5	4.82177, 18.56115, 5.9506	3.98509, 10.40145, 4.54784	2.51418, 1	3	NA	NA
GL182493.1	4432	5120	4695, 4691, 4674	18, 18, 15	19.00683, 18.29879, 17.76194	10.40758, 9.984, 10.35658	15.81175,	3	NA	NA
GL182502.1	258	1091	375, 356, 330, 720	11, 16, 10, 7	11.42621, 16.51912, 11.6791, 7.61369	7.41109, 9.44018, 7.62925, 5.54854	8.57514, 1	3	NA	NA
GL182502.1	5852	6544	6027, 6209, 5980	19, 19, 14	10.9928, 8.96109, 8.66208	5.4684, 4.45146, 5.14659	8.17312, 6	3	NA	NA
GL182502.1	7809	8541	7856, 7950, 8204	12, 19, 14	5.47668, 8.96109, 8.84187	3.63131, 4.45146, 5.25276	3.11168, 6	3	NA	NA
GL182550.1	2569	3002	2965, 2953, 2974	25, 38, 38	28.4518, 46.646, 49.05736	13.85048, 19.4487, 20.86776	24.98054,	3	NA	NA

GL182573.1	6658	7087	6929, 6966, 6879	6, 11, 5	5.96715, 10.60895, 5.9506	4.64927, 6.9343, 4.54784	3.52158, 7	3	NA	NA
GL182583.1	8187	8509	8397, 8318, 8283	10, 8, 12	11.06011, 7.07159, 16.36823	7.306, 5.20073, 9.85365	8.22712, 4	3	NA	NA
GL182602.1	20	437	140, 287, 180	53, 44, 31	59.37819, 46.96049, 36.22397	19.85306, 16.96359, 16.22715	55.39814,	3	NA	6775
GL182604.1	3347	3619	3395, 3395, 3395	12, 8, 9	13.8483, 7.07159, 11.64359	8.63436, 5.20073, 7.57973	10.86694,	3	NA	NA
GL182605.1	318	740	459, 673, 606	9, 6, 9	7.17299, 3.85021, 8.01994	5.13877, 3.34853, 5.66309	4.61317, 1	3	NA	NA
GL182605.1	9041	9649	9285, 9331, 9222	10, 16, 12	11.06011, 17.16856, 16.36823	7.306, 9.82359, 9.85365	8.22712, 1	3	NA	NA
GL182625.1	2085	2958	2429, 2696, 2544	19, 21, 6	21.91879, 24.35402, 7.29403	11.84839, 12.71289, 5.30581	18.62754,	3	NA	NA
GL182626.1	1986	2376	2200, 2289, 2122	5, 6, 5	4.82177, 4.93312, 5.9506	3.98509, 4.04501, 4.54784	2.51418, 2	3	NA	NA
GL182661.1	53	608	321, 335, 335	69, 129, 57	57.57624, 121.25821, 53.90042	13.20765, 18.57787, 15.38977	53.61901,	3	NA	NA
GL182661.1	1678	2162	1735, 1729, 1732	50, 73, 31	35.71399, 52.51791, 22.43166	9.62272, 10.57509, 8.49091	32.08649,	3	NA	NA
GL182661.1	9463	10300	10179, 9965, 10130	6, 18, 5	5.96715, 19.97713, 5.9506	4.64927, 10.97931, 4.54784	3.52158, 1	3	NA	NA
GL182665.1	59	476	163, 226, 366	5, 11, 7	4.82177, 10.60895, 8.69489	3.98509, 6.9343, 6.06379	2.51418, 7	3	NA	NA
GL182677.1	3539	4280	3795, 3658, 3816	23, 28, 20	20.32814, 24.37082, 21.0579	9.6806, 10.39781, 11.06214	17.08765,	3	NA	NA
GL182694.1	29	719	280, 534, 263	17, 30, 20	19.60089, 33.00599, 25.23416	10.9998, 14.70932, 13.46466	16.38573,	3	NA	NA
GL182705.1	8550	9488	9048, 9030, 8818	10, 8, 6	11.06011, 7.07159, 7.29403	7.306, 5.20073, 5.30581	8.22712, 4	3	NA	NA
GL182707.1	1313	3097	2359, 2481, 2303	42, 52, 27	42.50861, 49.41551, 28.73781	15.3244, 15.04952, 13.30451	38.76614,	3	NA	NA
GL182710.1	65	777	272, 279, 421	11, 10, 6	9.90875, 8.12616, 6.168	6.51186, 5.58036, 4.75778	7.14919, 5	3	NA	NA
GL182710.1	1039	1673	1313, 1164, 1349	8, 14, 6	6.55639, 12.70158, 6.168	4.88389, 7.60958, 4.75778	4.06705, 9	3	NA	NA
GL182711.1	144	849	711, 613, 792	11, 17, 7	12.43693, 18.56115, 8.69489	7.97018, 10.40145, 6.06379	9.52692, 1	3	NA	NA
GL182743.1	41	888	748, 157, 733	20, 27, 20	25.38223, 31.55624, 27.04832	13.54494, 14.98154, 14.41766	21.99061,	3	NA	NA
GL182743.1	9487	9868	9654, 9730, 9731	7, 11, 11	7.1696, 10.60895, 14.75772	5.31345, 6.9343, 9.09568	4.61046, 7	3	NA	NA
GL182746.1	8171	9517	9086, 9150, 9051	10, 9, 11	11.06011, 8.21099, 14.75772	7.306, 5.77858, 9.09568	8.22712, 5	3	NA	0
GL182790.1	8427	8754	8604, 8556, 8539	11, 11, 6	12.43693, 10.60895, 7.29403	7.97018, 6.9343, 5.30581	9.52692, 7	3	NA	NA
GL182803.1	60	778	415, 503, 409	23, 31, 31	21.16297, 26.01909, 33.80585	10.17472, 10.33677, 14.79113	17.89661,	3	NA	NA
GL182803.1	9279	10020	9833, 9866, 9855	38, 57, 27	43.21235, 60.82638, 34.76451	17.42325, 18.83909, 16.83978	39.45964,	3	NA	NA
GL182809.1	90	829	571, 292, 461	14, 16, 11	16.7649, 17.16856, 14.75772	9.96273, 9.82359, 9.09568	13.6543, 1	3	NA	NA
GL182837.1	90	660	476, 392, 509	12, 11, 8	12.18267, 9.66539, 8.35103	7.70145, 6.36265, 5.93881	9.29304, 7	3	NA	NA
GL182837.1	7728	8301	8070, 8089, 8098	15, 15, 8	13.41505, 11.56639, 8.6841	7.80796, 6.67456, 6.11715	10.45899,	3	NA	NA
GL182847.1	2477	3270	3041, 3011, 2928	4, 11, 5	3.74195, 10.60895, 5.9506	3.32091, 6.9343, 4.54784	1.6222, 7.9	3	NA	NA
GL182849.1	85	1182	623, 649, 696	32, 33, 32	30.88024, 27.98422, 35.25908	12.69434, 10.74624, 15.25335	27.35276,	3	NA	NA
GL182849.1	2495	3371	2891, 2874, 3269	15, 27, 13	10.70853, 21.0037, 10.4903	6.15483, 8.84984, 6.47112	7.90562, 1	3	NA	NA
GL182852.1	1848	2352	2099, 2138, 2138	11, 16, 17	12.43693, 17.16856, 24.40832	7.97018, 9.82359, 13.44559	9.52692, 1	3	NA	NA
GL182858.1	6161	6831	6484, 6706, 6515	7, 7, 5	7.1696, 5.97725, 5.9506	5.31345, 4.62287, 4.54784	4.61046, 3	3	NA	NA
GL182874.1	7850	9363	8593, 8975, 8817	14, 15, 10	16.7649, 15.80067, 12.71002	9.96273, 9.24574, 8.12734	13.6543, 1	3	NA	4421
GL182877.1	3852	6092	5748, 4451, 5746	126, 154, 127	74.67851, 86.50774, 94.69481	8.58441, 8.11009, 12.22579	70.53523,	3	NA	NA
GL182877.1	7404	8120	7814, 7818, 7844	60, 55, 39	20.11742, 12.17003, 12.85772	4.13283, 2.94058, 3.82056	16.88357,	3	NA	NA
GL182884.1	3205	3797	3384, 3551, 3331	19, 22, 13	22.35011, 24.17682, 18.01082	12.09487, 12.30626, 10.61163	19.0463, 2	3	NA	NA
GL182885.1	8063	8658	8378, 8090, 8314	6, 7, 6	5.31708, 5.75319, 6.99807	4.2777, 4.48487, 5.17195	2.96727, 3	3	NA	NA
GL182904.1	193	1309	943, 379, 364	8, 13, 7	8.42257, 13.14514, 8.69489	5.97763, 8.09002, 6.06379	5.76336, 1	3	NA	NA
GL182919.1	11	420	211, 208, 302	53, 57, 26	64.18073, 69.37259, 35.07777	22.56406, 23.52639, 17.31171	60.1464, 6	3	NA	NA
GL182919.1	6383	7201	6756, 6764, 6757	94, 106, 76	92.46243, 103.31354, 76.27567	18.97131, 19.16404, 18.61288	88.14004,	3	NA	NA
GL182919.1	8534	9496	8678, 8665, 8704	24, 33, 31	11.78692, 17.91971, 20.85117	4.99245, 6.08951, 7.73522	8.91787, 1	3	NA	NA
GL182943.1	2672	3016	2699, 2699, 2700	69, 94, 49	92.49438, 123.64347, 69.79575	30.33997, 33.44876, 27.85088	88.17085,	3	NA	NA
GL182945.1	2381	2848	2741, 2590, 2715	9, 18, 17	9.72082, 19.97713, 24.74975	6.64182, 10.97931, 13.59515	6.97155, 1	3	U4	5773
GL182948.1	8689	9442	9154, 9279, 9142	9, 15, 6	9.36986, 15.7813, 6.6567	6.44997, 9.23437, 5.00845	6.65425, 1	3	NA	NA
GL182972.1	8986	9533	9289, 9347, 9315	11, 8, 11	12.43693, 7.07159, 14.75772	7.97018, 5.20073, 9.09568	9.52692, 4	3	NA	NA
GL182975.1	8754	9432	9257, 9249, 9178	28, 40, 21	31.3955, 45.24556, 27.07785	14.51749, 17.7338, 14.2403	27.85612,	3	NA	NA
GL182978.1	2572	3279	3062, 3066, 2686, 3071	8, 17, 3, 12	8.42257, 18.56115, 3.47371, 16.36823	5.97763, 10.40145, 3.03189, 9.85365	5.76336, 1	3	NA	NA
GL182984.1	8198	9290	8450, 8821, 8517	17, 31, 7	21.19088, 37.47519, 8.69489	11.87339, 16.96708, 6.06379	17.92307,	3	NA	NA
GL182986.1	1733	2709	2318, 2610, 2245	10, 18, 12	10.26482, 17.19487, 13.7434	6.86644, 9.31668, 8.57827	7.48624, 1	3	NA	NA
GL182988.1	7116	7997	7929, 7291, 7398	10, 17, 9	8.16254, 14.81481, 8.79325	5.60312, 8.14576, 6.12267	5.53763, 1	3	NA	NA
GL182988.1	8144	8566	8257, 8436, 8248	13, 16, 9	11.55805, 14.04087, 8.88966	7.13125, 7.93805, 6.17838	8.70034, 1	3	NA	NA
GL182989.1	8998	9425	9040, 9079, 9077	11, 10, 13	12.43693, 9.39128, 18.01082	7.97018, 6.35644, 10.61163	9.52692, 6	3	NA	NA
GL183001.1	46	980	130, 148, 107, 689	28, 25, 22, 8	29.46427, 24.81034, 26.79743, 7.42597	13.36659, 11.67607, 13.83268, 5.41279	25.96977,	3	NA	NA
GL183001.1	6746	7829	7370, 7361, 7324	30, 28, 18	33.28987, 27.17496, 21.69448	14.87817, 12.01226, 11.95651	29.71041,	3	NA	NA
GL183014.1	8706	9251	9069, 8881, 8932	18, 14, 8	22.92104, 14.45897, 10.1465	12.61945, 8.66788, 6.82176	19.59912,	3	NA	NA
GL183015.1	71	1652	897, 875, 668	111, 118, 92	108.24155, 113.81171, 94.10372	19.37196, 19.20358, 20.31127	103.78413	3	NA	NA
GL183015.1	7523	8397	8127, 8060, 8250	13, 28, 11	12.09198, 27.17496, 11.5511	7.45795, 12.01226, 7.48213	9.20698, 2	3	NA	NA
GL183020.1	75	1147	490, 584, 583	24, 36, 16	13.37616, 18.18613, 8.91166	5.70807, 5.73435, 4.91406	10.42206,	3	NA	NA
GL183020.1	5530	5734	5679, 5624, 5638	19, 27, 13	10.42788, 13.09942, 7.3354	5.17629, 5.1005, 4.55055	7.641, 10.3	3	NA	NA
GL183035.1	1864	2198	2034, 2066, 2034	12, 13, 6	13.8483, 13.14514, 7.29403	8.63436, 8.09002, 5.30581	10.86694,	3	NA	NA
GL183039.1	8827	9290	8855, 8854, 8853	36, 47, 20	47.23678, 64.39138, 28.97357	20.65663, 25.68264, 15.34882	43.42478,	3	NA	NA
GL183044.1	7546	8514	8269, 7555, 8053, 7540, 7883, 8373	13, 14, 22, 8, 4, 4	11.07277, 9.48413, 18.37326, 8.14528, 3.59344, 3.59344	6.83197, 5.63643, 8.84063, 5.82558, 3.23643, 3.23643	8.23905, 6	3	NA	NA
GL183044.1	8784	9064	8875, 8948, 8804	7, 13, 4	4.84123, 8.72347, 3.59344	3.90398, 5.38125, 3.23643	2.5312, 6.1	3	NA	NA
GL183050.1	758	1618	1154, 1301, 1302	118, 243, 139	113.02305, 251.00198, 148.53654	18.94799, 24.78915, 24.38091	108.52793	3	NA	NA
GL183050.1	7917	8753	8575, 8346, 8580	19, 28, 9	16.38781, 24.94961, 7.94299	8.54122, 10.72542, 5.61625	13.29598,	3	NA	NA
GL183051.1	8191	9353	9026, 9087, 9024	16, 17, 9	16.30427, 16.05386, 10.01275	9.31205, 8.90069, 6.79683	13.21554,	3	NA	NA
GL183054.1	75	1013	465, 822, 465	21, 29, 18	27.58681, 34.61861, 24.2813	14.51192, 16.05165, 13.31378	24.13677,	3	NA	NA
GL183061.1	8665	8884	8778, 8828, 8698	4, 7, 5	3.74195, 5.97725, 5.9506	3.32091, 4.62287, 4.54784	1.6222, 3.6	3	NA	NA
GL183080.1	7064	7596	7155, 7141, 7238	12, 22, 11	13.8483, 25.85271, 14.75772	8.63436, 13.29074, 9.09568	10.86694,	3	NA	NA
GL183081.1	21	1270	547, 567, 561	88, 112, 69	81.10156, 100.49596, 70.45969	16.57739, 16.59288, 18.64673	76.89569,	3	NA	NA
GL183098.1	6148	7000	6595, 6757, 6406	10, 17, 11	11.06011, 18.56115, 14.75772	7.306, 10.40145, 9.09568	8.22712, 1	3	NA	NA
GL183102.1	399	798	519, 584, 673	14, 10, 5	15.15267, 8.88645, 5.9506	9.07115, 6.0511, 4.54784	12.11465,	3	NA	NA
GL183102.1	1943	2615	1984, 2043, 2096	8, 19, 10	7.47668, 20.47061, 13.18184	5.44269, 11.002, 8.33771	4.89713, 1	3	NA	NA
GL183102.1	8480	9125	8863, 9010, 8820	8, 13, 5	8.42257, 13.14514, 5.9506	5.97763, 8.09002, 4.54784	5.76336, 1	3	NA	NA
GL183110.1	1558	2094	1743, 1856, 1757	7, 10, 11	5.36706, 7.55261, 11.49087	4.24225, 5.21944, 7.44794	3.01389, 5	3	NA	NA
GL183110.1	6620	7214	7035, 6909, 6867	11, 10, 6	9.71398, 8.24345, 5.67889	6.39253, 5.65367, 4.48822	6.97155, 5	3	NA	NA
GL183137.1	39	899	529, 520, 334	29, 59, 30	35.26753, 69.01646, 37.5789	16.43303, 22.33125, 17.41037	31.64943,	3	NA	NA
GL183137.1	7189	7959	7527, 7547, 7548	46, 67, 41	55.30064, 83.79023, 52.90765	20.83714, 26.48441, 21.78625	51.37355,	3	NA	NA
GL183149.1	249	783	369, 412, 534	18, 20, 18	13.02286, 13.43495, 18.60267	6.81301, 6.56666, 10.16634	10.08558,	3	NA	NA
GL183149.1	2065	3265	2902, 2297, 3138	14, 17, 10	9.0539, 10.55147, 8.61734	5.37869, 5.62856, 5.88578	6.35823, 7	3	NA	NA
GL183154.1	39	651	446, 169, 101	6, 8, 6	5.90927, 7.07159, 7.29403	4.61743, 5.20073, 5.30581	3.50164, 4	3	NA	6468
GL183165.1	6880	7395	7348, 7317, 7318	6, 14, 6	5.96715, 14.45897, 7.29403	4.64927, 8.66788, 5.30581	3.52158, 1	3	NA	NA
GL183166.1	1155	1517	1377, 1383, 1386	13, 13, 5	15.29169, 13.14514, 5.9506	9.29854, 8.09002, 4.54784	12.24396,	3	NA	NA
GL183175.1	93	3273	477, 2641, 531, 1914, 2666	11, 19, 6, 8, 7	8.54584, 17.11753, 5.53406, 7.85773, 6.6707	5.66575, 8.98159, 4.40503, 5.66361, 5.03432	5.88025, 1	3	NA	NA
GL183175.1	93	3273	2660, 2641, 531, 1914, 2666	13, 19, 6, 8, 7	10.71435, 17.11753, 5.53406, 7.85773, 6.6707	6.61004, 8.98159, 4.40503, 5.66361, 5.03432	7.91096, 1	3	NA	NA
GL183191.1	1498	1791	1737, 1740, 1738	8, 15, 17	8.42257, 15.80067, 24.08167	5.97763, 9.24574, 13.29929	5.76336, 1	3	NA	NA
GL183195.1	6539	6952	6811, 6819, 6842	22, 20, 10	29.43504, 22.87459, 13.18184	15.27618, 12.13503, 8.33771	25.94112,	3	NA	NA
GL183199.1	7986	9418	8570, 9199, 9188	52, 50, 32	47.43409, 42.9659, 30.10095	14.0936, 12.67287, 12.25107	43.61834,	3	NA	NA
GL183202.1	60	865	294, 230, 650	7, 7, 5	7.1696, 5.97725, 5.9506	5.31345, 4.62287, 4.54784	4.61046, 3	3	NA	NA
GL183215.1	6778	7263	7177, 7123, 7141	13, 16, 6	12.32133, 13.6406, 6.6567	7.59717, 7.69322, 5.00845	9.42482, 1	3	NA	NA
GL183229.1	30	590	542, 517, 415	25, 26, 23	34.51774, 32.02458, 30.91492	17.26872, 15.60218, 15.83681	30.91362,	3	NA	NA
GL183232.1	5032	5440	5221, 5199, 5241	11, 16, 6	12.43693, 17.16856, 7.29403	7.97018, 9.82359, 5.30581	9.52692, 1	3	NA	NA
GL183234.1	144	1097	480, 608, 859	10, 12, 7	11.06011, 11.86108, 8.69489	7.306, 7.51216, 6.06379	8.22712, 9	3	NA	NA
GL183242.1	6924	7394	7228, 7032, 7093	4, 10, 7	3.74195, 9.39128, 8.69489	3.32091, 6.35644, 6.06379	1.6222, 6.8	3	NA	NA
GL183250.1	1081	1568	1143, 1429, 1342	8, 11, 11	6.55639, 8.44639, 10.47247	4.88389, 5.60333, 6.85286	4.06705, 5	3	NA	NA
GL183254.1	10	1149	573, 454, 98, 471	8, 12, 6, 6	8.22568, 11.86108, 7.12188, 7.12188	5.8701, 7.51216, 5.22884, 5.22884	5.59681, 9	3	NA	NA
GL183261.1	123	1069	611, 669, 539	13, 17, 8	15.29169, 18.56115, 10.1465	9.29854, 10.40145, 6.82176	12.24396,	3	5S_rRNA	0
GL183266.1	3842	5806	5147, 5152, 5140	33, 44, 18	29.29314, 36.90414, 16.66788	11.4502, 11.72815, 8.99658	25.80264,	3	NA	NA
GL183267.1	3056	4102	3296, 3451, 4044	8, 14, 7	8.42257, 14.45897, 8.646	5.97763, 8.66788, 6.04229	5.76336, 1	3	NA	NA
GL183272.1	156	869	389, 598, 276	9, 7, 7	9.72082, 5.97725, 8.69489	6.64182, 4.62287, 6.06379	6.97155, 3	3	NA	NA
GL183272.1	8543	9270	9067, 9084, 9112	13, 13, 10	15.29169, 13.14514, 13.18184	9.29854, 8.09002, 8.33771	12.24396,	3	NA	NA
GL183283.1	8102	8550	8352, 8441, 8297	13, 13, 6	15.29169, 13.14514, 7.29403	9.29854, 8.09002, 5.30581	12.24396,	3	NA	NA
GL183290.1	64	919	382, 287, 373	16, 23, 18	15.09881, 21.63812, 20.40623	8.58417, 10.45783, 11.22809	12.06341,	3	NA	NA
GL183290.1	3341	4018	3808, 3959, 3690	18, 7, 4	17.65252, 4.20361, 3.12644	9.59407, 3.48594, 2.95476	14.50784,	3	NA	NA
GL183301.1	6825	7858	7499, 7439, 7422	23, 46, 23	27.72716, 54.17186, 29.81154	14.07465, 20.18027, 15.24424	24.27442,	3	NA	NA
GL183330.1	7613	8672	8141, 8174, 8642	8, 5, 5	8.42257, 3.94561, 5.9506	5.97763, 3.46715, 4.54784	5.76336, 1	3	NA	NA
GL183332.1	6831	7377	6940, 7025, 6931	7, 7, 5	7.1696, 5.97725, 5.9506	5.31345, 4.62287, 4.54784	4.61046, 3	3	NA	NA
GL183340.1	67	1089	235, 215, 206, 804	55, 62, 41, 34	26.60246, 24.88746, 21.93961, 16.06055	5.93981, 4.93862, 6.31077, 5.25897	23.17758,	3	NA	NA
GL183340.1	67	1104	781, 215, 206, 804	44, 62, 41, 34	17.99316, 24.88746, 21.93961, 16.06055	4.77306, 4.93862, 6.31077, 5.25897	14.83626,	3	NA	NA
GL183340.1	2211	3089	2758, 2524, 2721	84, 110, 57	53.37903, 66.50262, 37.14116	9.01578, 8.70137, 8.71487	49.47885,	3	NA	NA
GL183358.1	1472	1839	1494, 1500, 1494	8, 12, 6	8.42257, 11.86108, 7.29403	5.97763, 7.51216, 5.30581	5.76336, 9	3	NA	NA
GL183362.1	1243	1818	1714, 1544, 1544	18, 23, 13	22.92104, 27.36982, 18.01082	12.61945, 13.8686, 10.61163	19.59912,	3	NA	NA
GL183380.1	50	718	154, 268, 325	7, 8, 4	7.1696, 7.07159, 4.67325	5.31345, 5.20073, 3.78987	4.61046, 4	3	NA	NA
GL183381.1	21	940	404, 543, 503	40, 30, 31	40.59678, 29.3872, 33.1442	15.07362, 12.57445, 14.39906	36.88494,	3	NA	NA
GL183381.1	1404	2116	1777, 1724, 1954	10, 12, 11	5.69107, 8.22592, 8.12222	4.04414, 5.27316, 5.39965	3.30537, 5	3	NA	NA
GL183385.1	65	808	175, 195, 151	10, 15, 8	8.16254, 9.8904, 6.38723	5.60312, 5.66748, 4.77798	5.53763, 7	3	NA	NA
GL183385.1	1297	1865	1393, 1363, 1738	10, 18, 11	8.16254, 12.88004, 9.67839	5.60312, 6.73013, 6.37065	5.53763, 1	3	NA	NA
GL183385.1	7874	8894	8234, 8546, 8465	20, 22, 12	23.40409, 24.76114, 14.76955	12.44081, 12.6523, 9.10943	20.06837,	3	NA	NA
GL183389.1	935	1372	1337, 1308, 1342	14, 25, 22	16.7649, 30.45643, 32.54622	9.96273, 15.02432, 16.81062	13.6543, 2	3	NA	NA
GL183394.1	40	2355	870, 1107, 1478	136, 143, 73	135.24448, 134.3233, 70.30014	20.95331, 18.87782, 17.09649	130.60063	3	NA	NA
GL183395.1	5746	6624	5867, 5880, 6062	77, 105, 49	83.19205, 112.28031, 54.1313	21.37704, 22.89805, 18.76745	78.96431,	3	NA	NA
GL183403.1	57	632	314, 208, 354	6, 11, 3	5.96715, 10.60895, 3.47371	4.64927, 6.9343, 3.03189	3.52158, 7	3	NA	NA
GL183413.1	8661	9221	8920, 8938, 8876	10, 10, 12	11.06011, 9.39128, 15.16114	7.306, 6.35644, 9.30141	8.22712, 6	3	NA	NA
GL183414.1	8079	9115	8635, 8475, 8650	20, 33, 17	23.40409, 38.81641, 21.41937	12.44081, 16.95528, 11.99417	20.06837,	3	NA	NA
GL183419.1	4710	5192	4991, 4991, 4905	12, 23, 12	13.8483, 25.81799, 15.5838	8.63436, 12.95947, 9.50165	10.86694,	3	NA	NA
GL183430.1	37	1034	237, 604, 495	10, 10, 6	11.06011, 9.39128, 7.29403	7.306, 6.35644, 5.30581	8.22712, 6	3	NA	NA
GL183431.1	32	588	561, 560, 559	64, 77, 68	84.27993, 97.40298, 96.93069	28.38443, 28.66704, 33.48918	80.03999,	3	NA	NA
GL183433.1	8771	9342	9200, 9249, 9254	8, 10, 6	8.42257, 9.39128, 7.29403	5.97763, 6.35644, 5.30581	5.76336, 6	3	NA	NA
GL183446.1	1640	2125	1917, 1906, 1973	11, 11, 4	12.17114, 8.89461, 4.38152	7.82679, 5.88422, 3.65448	9.28263, 6	3	NA	NA
GL183446.1	7873	8769	7978, 8211, 7919	7, 11, 4	7.1696, 10.60895, 4.67325	5.31345, 6.9343, 3.78987	4.61046, 7	3	NA	NA
GL183447.1	8302	8704	8525, 8584, 8525	10, 7, 6	11.06011, 5.97725, 7.29403	7.306, 4.62287, 5.30581	8.22712, 3	3	NA	NA
GL183452.1	6061	6511	6427, 6256, 6305	9, 11, 8	9.72082, 10.60895, 10.1465	6.64182, 6.9343, 6.82176	6.97155, 7	3	NA	NA
GL183453.1	1444	2198	1860, 1904, 1928	11, 18, 6	7.80634, 12.88004, 4.80295	5.20114, 6.73013, 3.96416	5.20581, 1	3	NA	NA
GL183458.1	102	710	491, 367, 353	14, 13, 9	14.52862, 13.1283, 9.76105	8.70881, 8.08007, 6.66343	11.51702,	3	NA	NA
GL183458.1	6101	7004	6584, 6562, 6438	16, 18, 9	11.52409, 11.10386, 6.21488	6.41223, 5.72764, 4.52972	8.66865, 8	3	NA	NA
GL183458.1	8340	8889	8689, 8733, 8760	13, 24, 8	8.55813, 17.03233, 5.29161	5.28066, 7.53638, 4.07675	5.8909, 14	3	NA	NA
GL183460.1	26	631	169, 204, 571	17, 19, 9	20.2407, 21.39089, 10.56949	11.35794, 11.54296, 7.08032	17.00287,	3	NA	NA
GL183460.1	8277	8738	8466, 8518, 8652	8, 7, 8	8.42257, 5.97725, 10.1465	5.97763, 4.62287, 6.82176	5.76336, 3	3	NA	NA
GL183477.1	85	986	482, 642, 283	11, 13, 6	12.43693, 13.14514, 7.29403	7.97018, 8.09002, 5.30581	9.52692, 1	3	NA	NA
GL183484.1	4262	4835	4358, 4358, 4359	75, 105, 49	95.43065, 134.75044, 64.26083	28.66644, 33.14598, 24.61947	91.07967,	3	NA	NA
GL183495.1	9	837	102, 131, 62, 669	13, 18, 6, 6	13.79066, 18.60217, 6.25896, 6.25896	8.46641, 10.16604, 4.80589, 4.80589	10.8171, 1	3	NA	NA
GL183496.1	5157	5550	5291, 5411, 5379	8, 7, 6	8.42257, 5.97725, 7.29403	5.97763, 4.62287, 5.30581	5.76336, 3	3	NA	NA
GL183498.1	36	678	531, 503, 511	14, 26, 13	14.23954, 28.01936, 15.34872	8.53829, 13.2395, 9.3288	11.24094,	3	NA	NA
GL183503.1	6454	7727	7174, 6980, 7129	11, 30, 11	12.43693, 34.4899, 14.75772	7.97018, 15.5917, 9.09568	9.52692, 3	3	NA	NA
GL183504.1	2051	3014	2911, 2483, 2864	8, 14, 6	6.48409, 10.57816, 6.08001	4.83873, 6.30219, 4.71063	4.00135, 7	3	NA	NA
GL183506.1	7724	8855	8253, 8162, 8282	26, 34, 14	30.86956, 38.05053, 17.01463	14.93039, 16.08706, 10.0942	27.34261,	3	NA	NA
GL183508.1	97	577	358, 471, 378	10, 20, 7	11.06011, 22.6463, 8.49722	7.306, 12.00208, 5.97582	8.22712, 1	3	NA	NA
GL183508.1	2069	2597	2187, 2273, 2284	6, 9, 6	5.96715, 7.47261, 6.168	4.64927, 5.32627, 4.75778	3.52158, 5	3	NA	NA
GL183508.1	7967	8885	8330, 8655, 8543	6, 13, 5	5.96715, 13.14514, 5.9506	4.64927, 8.09002, 4.54784	3.52158, 1	3	NA	NA
GL183515.1	409	730	510, 701, 600	10, 13, 3	11.06011, 13.14514, 3.47371	7.306, 8.09002, 3.03189	8.22712, 1	3	NA	NA
GL183526.1	204	1377	794, 300, 902, 888	17, 5, 23, 14	21.34551, 3.94561, 27.36982, 19.68329	11.95527, 3.46715, 13.8686, 11.3696	18.07159,	3	NA	NA
GL183526.1	8126	8810	8312, 8287, 8311	55, 75, 40	66.51919, 90.47771, 48.93013	22.90589, 25.97973, 19.89937	62.45856,	3	NA	NA
GL183540.1	479	1000	872, 734, 775	8, 11, 7	8.42257, 10.60895, 8.69489	5.97763, 6.9343, 6.06379	5.76336, 7	3	NA	NA
GL183542.1	494	1055	721, 680, 725	8, 18, 5	8.10827, 19.7713, 5.9506	5.80497, 10.85902, 4.54784	5.48761, 1	3	NA	NA
GL183567.1	8069	8794	8485, 8459, 8493	74, 123, 35	82.71718, 133.75085, 39.13085	22.46062, 24.61226, 16.307	78.49471,	3	NA	NA
GL183577.1	7944	8651	8275, 8288, 8295	10, 15, 7	11.06011, 15.80067, 8.69489	7.306, 9.24574, 6.06379	8.22712, 1	3	NA	NA
GL183585.1	7370	8219	7837, 7828, 7690	47, 48, 43	48.24549, 48.2645, 49.19565	16.40251, 16.00436, 18.68434	44.41845,	3	NA	NA
GL183590.1	27	853	512, 326, 198	15, 25, 11	15.05862, 22.71902, 10.37715	8.80576, 10.4381, 6.79571	12.0251, 1	3	NA	NA
GL183591.1	5835	6469	6218, 6160, 6124	10, 11, 12	11.06011, 10.60895, 16.36823	7.306, 6.9343, 9.85365	8.22712, 7	3	NA	NA
GL183593.1	77	1379	1071, 254, 1093	16, 24, 17	13.97573, 21.89488, 19.12895	7.8982, 10.28282, 10.73053	10.98763,	3	NA	NA
GL183596.1	7091	7896	7421, 7434, 7180	8, 15, 6	8.42257, 15.80067, 7.29403	5.97763, 9.24574, 5.30581	5.76336, 1	3	NA	NA
GL183604.1	628	1329	839, 797, 1060	5, 11, 9	4.82177, 10.60895, 10.42289	3.98509, 6.9343, 7.00726	2.51418, 7	3	NA	NA
GL183604.1	7950	8541	8156, 8047, 8140	9, 12, 7	9.72082, 11.86108, 8.69489	6.64182, 7.51216, 6.06379	6.97155, 9	3	NA	NA
GL183620.1	12	854	523, 532, 491	47, 49, 20	56.06566, 56.32551, 25.00335	20.80455, 20.00485, 13.33871	52.1286, 5	3	NA	NA
GL183621.1	123	3302	1731, 2411, 557	26, 30, 16	21.55238, 23.48321, 14.8487	9.44855, 9.29735, 8.43176	18.2721, 2	3	NA	NA
GL183625.1	3872	4642	4208, 4150, 4487	14, 10, 10	11.75167, 6.11993, 8.69318	7.02514, 4.31286, 5.93253	8.88383, 3	3	NA	NA
GL183637.1	1881	4322	2556, 2552, 2038	77, 89, 50	67.55108, 71.04186, 45.32139	14.74102, 12.95624, 13.77461	63.48014,	3	NA	641
GL183637.1	8700	9244	8906, 8899, 8906	97, 116, 73	98.13348, 113.38033, 76.81157	20.15896, 19.63673, 20.02652	93.7592, 1	3	NA	2041
GL183644.1	8348	8934	8465, 8761, 8542	9, 11, 9	9.72082, 10.60895, 11.64359	6.64182, 6.9343, 7.57973	6.97155, 7	3	NA	NA
GL183651.1	213	907	553, 338, 644	6, 10, 4	5.96715, 9.39128, 4.67325	4.64927, 6.35644, 3.78987	3.52158, 6	3	NA	NA
GL183674.1	8	502	441, 476, 476	72, 113, 85	97.71761, 151.47206, 120.42977	31.64026, 37.23462, 36.98195	93.34699,	3	NA	NA
GL183677.1	6020	6378	6179, 6179, 6179	13, 19, 9	15.29169, 21.4153, 11.64359	9.29854, 11.55717, 7.57973	12.24396,	3	NA	NA
GL183678.1	2696	4311	3808, 3014, 4090, 3844	21, 13, 16, 8	20.03937, 9.1776, 12.17409, 7.95096	10.14017, 5.65923, 6.80165, 5.71659	16.80789,	3	NA	NA
GL183694.1	5779	7158	6650, 6332, 6547	25, 37, 14	25.16784, 36.96367, 14.92875	11.88958, 14.23339, 8.94211	21.78213,	3	NA	NA
GL183707.1	47	515	338, 239, 361	15, 22, 13	18.26603, 25.85271, 16.70862	10.62691, 13.29074, 10.0169	15.09729,	3	NA	NA
GL183725.1	1890	2432	2248, 2353, 2211	8, 12, 7	4.93217, 6.61696, 4.98481	3.84355, 4.29385, 3.99709	2.61618, 4	3	NA	NA
GL183742.1	42	1170	393, 331, 365	34, 43, 19	29.51673, 36.5297, 15.3154	11.23387, 11.86261, 7.90001	26.02047,	3	NA	NA
GL183779.1	2883	3769	3086, 3312, 3671	15, 17, 11	13.97429, 16.69909, 13.1143	8.15, 9.29214, 8.32282	10.98644,	3	NA	NA
GL183795.1	7566	8613	8257, 7649, 8186	8, 13, 8	8.42257, 13.1283, 9.92329	5.97763, 8.08007, 6.7228	5.76336, 1	3	NA	NA
GL183813.1	2549	3126	2650, 2983, 2608, 2931	9, 8, 5, 7	9.72082, 7.07159, 5.50079, 8.08824	6.64182, 5.20073, 4.33868, 5.7849	6.97155, 4	3	NA	NA
GL183813.1	8241	8949	8757, 8840, 8272, 8843	11, 29, 8, 10	12.43693, 34.61861, 9.05167, 11.83521	7.97018, 16.05165, 6.30653, 7.70798	9.52692, 3	3	NA	NA
GL183824.1	3911	4196	4027, 4029, 4031	14, 21, 10	15.31941, 24.35402, 12.33934	9.1665, 12.71289, 7.95424	12.27033,	3	NA	NA
GL183846.1	7612	8605	7985, 7985, 7985	21, 33, 13	27.28066, 39.82432, 17.16464	14.34912, 17.55182, 10.23255	23.83737,	3	NA	NA
GL183850.1	7873	8571	8074, 8068, 8022	11, 16, 5	12.17114, 17.16856, 5.9506	7.82679, 9.82359, 4.54784	9.28263, 1	3	NA	NA
GL183863.1	5903	6348	6221, 6084, 6117	4, 7, 9	3.74195, 5.97725, 10.87975	3.32091, 4.62287, 7.23113	1.6222, 3.6	3	NA	NA
GL183867.1	7683	8540	7880, 7836, 7723	8, 10, 8	8.42257, 9.39128, 10.1465	5.97763, 6.35644, 6.82176	5.76336, 6	3	NA	NA
GL183880.1	64	482	237, 103, 251	5, 6, 6	4.82177, 4.93312, 7.29403	3.98509, 4.04501, 5.30581	2.51418, 2	3	NA	NA
GL183885.1	3805	5420	5119, 5039, 5137	13, 28, 7	12.94099, 27.03127, 7.96278	7.96907, 11.92807, 5.72394	10.00729,	3	NA	NA
GL183889.1	1202	2344	2011, 1498, 1938	12, 12, 8	13.8483, 11.86108, 10.0913	8.63436, 7.51216, 6.79758	10.86694,	3	NA	NA
GL183893.1	8162	8565	8365, 8469, 8366	8, 9, 6	8.42257, 8.21099, 7.29403	5.97763, 5.77858, 5.30581	5.76336, 5	3	NA	NA
GL183895.1	7857	8558	8247, 8340, 8291	23, 40, 12	25.13729, 47.39385, 14.23156	12.55499, 18.9936, 8.83588	21.75229,	3	NA	NA
GL183919.1	1498	2982	1973, 2108, 2338	22, 36, 12	21.05876, 30.39366, 10.42593	10.43563, 11.04042, 6.64188	17.79552,	3	NA	NA
GL183921.1	5272	6579	5794, 5858, 5742, 6341	17, 20, 12, 6	17.19112, 18.78201, 11.81848, 4.99541	9.58879, 9.69018, 7.4869, 4.08336	14.0641, 1	3	NA	NA
GL183921.1	6828	7540	7154, 7288, 7336	5, 9, 6	3.6617, 6.6123, 5.39683	3.28661, 4.78362, 4.32487	1.6222, 4.2	3	NA	NA
GL183923.1	1967	2792	2224, 2172, 2654	17, 21, 17	11.378, 13.20118, 12.93175	6.09719, 6.21689, 7.00809	8.52911, 1	3	NA	NA
GL183923.1	3281	3694	3497, 3490, 3483	19, 18, 17	11.01742, 7.65993, 11.84798	5.48129, 3.97277, 6.369	8.19759, 5	3	NA	NA
GL183923.1	7242	8228	7587, 7913, 7697	18, 36, 11	14.11696, 33.09179, 9.14441	7.45687, 12.4665, 6.04007	11.12321,	3	NA	NA
GL183928.1	505	940	656, 558, 578	5, 12, 6	4.82177, 11.72254, 6.99807	3.98509, 7.42986, 5.17195	2.51418, 9	3	NA	NA
GL183928.1	7980	8586	8271, 8248, 8464	11, 11, 9	12.43693, 10.60895, 11.64359	7.97018, 6.9343, 7.57973	9.52692, 7	3	NA	NA
GL183937.1	6043	6904	6487, 6392, 6724	15, 14, 8	18.26603, 14.45897, 10.1465	10.62691, 8.66788, 6.82176	15.09729,	3	NA	NA
GL183954.1	7922	8407	8014, 8092, 8313	6, 8, 3	5.96715, 7.07159, 3.47371	4.64927, 5.20073, 3.03189	3.52158, 4	3	NA	NA
GL183964.1	282	980	718, 493, 874	11, 9, 3	12.43693, 8.21099, 3.47371	7.97018, 5.77858, 3.03189	9.52692, 5	3	NA	NA
GL183967.1	72	719	580, 215, 516	8, 13, 5	8.42257, 13.14514, 5.9506	5.97763, 8.09002, 4.54784	5.76336, 1	3	NA	NA
GL183967.1	7856	8497	8356, 8351, 8352	33, 61, 19	39.43015, 78.74725, 23.88486	17.31875, 26.81696, 12.94573	35.73727,	3	NA	NA
GL183975.1	35	1428	621, 632, 802	33, 26, 13	38.92649, 31.2219, 17.16464	17.02051, 15.13645, 10.23255	35.241, 27	3	NA	NA
GL183977.1	7865	8323	8166, 8312, 8037	5, 9, 4	4.82177, 8.21099, 4.67325	3.98509, 5.77858, 3.78987	2.51418, 5	3	NA	NA
GL183991.1	50	962	391, 787, 149	8, 16, 6	8.42257, 16.98517, 7.29403	5.97763, 9.71597, 5.30581	5.76336, 1	3	NA	NA
GL183991.1	7671	8174	7962, 8081, 7951	12, 9, 6	13.8483, 8.21099, 7.29403	8.63436, 5.77858, 5.30581	10.86694,	3	NA	NA
GL183995.1	1600	2026	1895, 1740, 1812	9, 6, 5	9.72082, 4.93312, 5.9506	6.64182, 4.04501, 4.54784	6.97155, 2	3	NA	NA
GL184010.1	2266	3012	2428, 2859, 2461	14, 18, 14	15.84929, 18.00717, 17.61756	9.46495, 9.80837, 10.40352	12.77923,	3	NA	NA
GL184046.1	7510	8375	7943, 7890, 7732	15, 15, 8	16.90423, 15.80067, 10.1465	9.88146, 9.24574, 6.82176	13.78827,	3	NA	NA
GL184053.1	2129	2467	2441, 2436, 2438	26, 25, 8	35.26919, 30.45643, 10.1465	17.41492, 15.02432, 6.82176	31.65079,	3	NA	NA
GL184055.1	7532	8631	8428, 8341, 8122	9, 9, 12	9.72082, 8.21099, 16.36823	6.64182, 5.77858, 9.85365	6.97155, 5	3	NA	NA
GL184063.1	873	1420	992, 935, 1081	6, 8, 6	5.96715, 7.07159, 7.29403	4.64927, 5.20073, 5.30581	3.52158, 4	3	NA	NA
GL184083.1	1002	1683	1278, 1255, 1115	12, 42, 11	9.69483, 40.29206, 11.6739	6.18815, 14.12324, 7.55148	6.96086, 3	3	NA	NA
GL184084.1	246	2079	819, 811, 555	14, 15, 11	14.38232, 12.86621, 11.4314	8.62271, 7.47111, 7.41405	11.37721,	3	NA	NA
GL184099.1	45	984	564, 578, 254	11, 19, 8	12.43693, 21.4153, 10.1465	7.97018, 11.55717, 6.82176	9.52692, 1	3	NA	NA
GL184099.1	6204	6661	6384, 6398, 6328	22, 33, 17	22.36913, 30.37097, 20.18522	11.22395, 12.04339, 11.32722	19.06395,	3	NA	NA
GL184106.1	7248	8306	7629, 7461, 7465	9, 9, 9	9.72082, 8.21099, 11.64359	6.64182, 5.77858, 7.57973	6.97155, 5	3	NA	NA
GL184111.1	7120	8135	7637, 7657, 7657	25, 40, 14	31.68083, 52.05507, 19.30655	15.72333, 21.73907, 11.20466	28.13576,	3	NA	NA
GL184130.1	7107	7384	7230, 7213, 7189	11, 12, 9	12.43693, 11.86108, 11.64359	7.97018, 7.51216, 7.57973	9.52692, 9	3	NA	NA

GL184137.1	19	739	551, 292, 361	10, 20, 12	11.06011, 22.87459, 16.36823	7.306, 12.13503, 9.85365	8.22712, 1	3	NA	NA	
GL184137.1	7358	8266	7671, 7638, 7472	10, 13, 7	9.661, 12.03475, 8.69489	6.51661, 7.4231, 6.06379	6.9283, 9.3	3	NA	NA	
GL184149.1	7973	8299	8167, 8109, 8250	5, 12, 6	4.82177, 11.86108, 7.29403	3.98509, 7.51216, 5.30581	2.51418, 9	3	NA	NA	
GL184157.1	232	810	449, 367, 664	17, 25, 9	14.4435, 19.82856, 7.31381	7.91979, 8.78546, 5.22713	11.43583,	3	NA	NA	
GL184157.1	7627	8393	7712, 7933, 7845	6, 9, 8	5.96715, 8.21099, 10.0913	4.64927, 5.77858, 6.79758	3.52158, 5	3	NA	NA	
GL184158.1	4988	5260	5048, 5065, 5031	8, 9, 5	8.42257, 8.21099, 5.9506	5.97763, 5.77858, 4.54784	5.76336, 5	3	NA	NA	
GL184177.1	109	704	366, 298, 366	13, 6, 8	15.29169, 4.93312, 10.1465	9.29854, 4.04501, 6.82176	12.24396,	3	NA	NA	
GL184177.1	5963	6700	6139, 6176, 6086	12, 15, 5	13.05874, 15.80067, 5.9506	8.20296, 9.24574, 4.54784	10.11961,	3	NA	NA	
GL184177.1	7037	7536	7134, 7228, 7295	9, 11, 6	9.12009, 10.60895, 7.29403	6.30997, 6.9343, 5.30581	6.42028, 7	3	NA	NA	
GL184183.1	4942	5848	5265, 5449, 5148	5, 13, 9	4.82177, 13.14514, 11.64359	3.98509, 8.09002, 7.57973	2.51418, 1	3	NA	NA	
GL184211.1	7323	8071	7487, 7998, 7937	4, 6, 6	3.74195, 4.93312, 7.29403	3.32091, 4.04501, 5.30581	1.6222, 2.7	3	NA	NA	
GL184221.1	1279	1637	1494, 1487, 1498	20, 26, 15	26.13767, 32.02458, 21.38372	13.94781, 15.60218, 12.12757	22.72398,	3	NA	NA	
GL184246.1	153	474	290, 305, 323	14, 13, 9	16.7649, 13.14514, 11.64359	9.96273, 8.09002, 7.57973	13.6543, 1	3	NA	NA	
GL184246.1	7373	8324	7785, 7714, 7639	11, 18, 8	12.43693, 19.97713, 10.1465	7.97018, 10.97931, 6.82176	9.52692, 1	3	NA	NA	
GL184248.1	77	1259	783, 771, 793	32, 35, 20	31.32845, 35.53384, 22.4589	12.95141, 14.20975, 11.89249	27.79107,	3	NA	NA	
GL184248.1	2276	2849	2315, 2442, 2345	15, 13, 8	10.91541, 8.96044, 6.90087	6.27947, 5.52602, 5.09678	8.10046, 6	3	NA	NA	
GL184266.1	1371	1675	1595, 1504, 1590	16, 23, 15	19.7934, 27.36982, 19.64455	11.29109, 13.8686, 11.32852	16.57067,	3	NA	NA	
GL184269.1	1947	2931	2318, 2397, 2377	26, 25, 16	25.89351, 21.514, 15.71034	11.97027, 9.73863, 8.95507	22.48761,	3	NA	NA	
GL184270.1	18	984	624, 602, 630	130, 157, 90	126.09222, 143.51266, 85.77673	19.77554, 18.15793, 17.70987	121.50247	3	NA	NA	
GL184270.1	7234	8299	7599, 8004, 7343	41, 63, 25	33.84911, 52.36595, 22.43141	11.19411, 12.8333, 10.27002	30.25918,	3	NA	NA	
GL184290.1	6	982	634, 238, 415	10, 23, 5	11.06011, 27.36982, 5.9506	7.306, 13.8686, 4.54784	8.22712, 2	3	NA	NA	
GL184294.1	7570	8184	7919, 7848, 7930	21, 24, 17	27.77672, 28.90463, 23.7686	14.612, 14.44646, 13.15613	24.32202,	3	NA	NA	
GL184305.1	83	747	555, 248, 100	7, 7, 3	7.1696, 5.97725, 3.47371	5.31345, 4.62287, 3.03189	4.61046, 3	3	NA	NA	
GL184316.1	7665	8263	8099, 8103, 7796	8, 7, 4	8.42257, 5.97725, 4.67325	5.97763, 4.62287, 3.78987	5.76336, 3	3	NA	NA	
GL184326.1	7652	8249	7921, 7949, 7922	27, 47, 41	33.18998, 59.43634, 55.99792	15.93814, 22.77573, 23.58824	29.61268,	3	NA	NA	
GL184334.1	6563	6917	6645, 6611, 6650	8, 16, 7	8.42257, 17.16856, 8.69489	5.97763, 9.82359, 6.06379	5.76336, 1	3	NA	NA	
GL184336.1	7357	8334	7572, 7559, 7530	28, 25, 31	26.22613, 23.41295, 33.1442	11.45889, 10.84616, 14.39906	22.80972,	3	NA	NA	
GL184337.1	7040	8182	7446, 7628, 7430	12, 9, 9	13.8483, 8.21099, 11.64359	8.63436, 5.77858, 7.57973	10.86694,	3	NA	NA	
GL184350.1	3920	4795	4311, 4640, 4433	9, 7, 4	9.72082, 5.97725, 4.67325	6.64182, 4.62287, 3.78987	6.97155, 3	3	NA	NA	
GL184355.1	2405	3779	2706, 3232, 2768	6, 13, 7	3.78472, 8.17733, 6.3257	3.30502, 5.05049, 4.83277	1.66163, 5	3	NA	NA	
GL184355.1	2405	4005	3779, 3232, 2768	14, 13, 7	11.22666, 8.17733, 6.3257	6.70125, 5.05049, 4.83277	8.38513, 5	3	NA	NA	
GL184355.1	6771	8016	7292, 7325, 7890	12, 19, 11	10.20921, 16.10778, 12.55982	6.50784, 8.37295, 8.03554	7.4341, 13	3	NA	NA	
GL184357.1	102	954	371, 434, 817	13, 16, 8	14.26982, 16.22539, 10.1465	8.73911, 9.26486, 6.82176	11.26966,	3	NA	NA	
GL184360.1	10	338	113, 74, 89	16, 12, 7	19.7934, 11.86108, 8.69489	11.29109, 7.51216, 6.06379	16.57067,	3	NA	NA	
GL184361.1	4484	5310	4800, 5028, 5245	13, 16, 7	10.46082, 11.80086, 6.6707	6.45284, 6.57745, 5.03432	7.67165, 9	3	NA	NA	
GL184365.1	619	1272	1063, 1160, 1238	15, 21, 20	18.26603, 24.35402, 29.33978	10.62691, 12.71289, 15.51584	15.09729,	3	NA	NA	
GL184379.1	16	770	250, 504, 70	16, 10, 8	19.41253, 9.39128, 9.76268	11.08796, 6.35644, 6.64964	16.20324,	3	NA	NA	
GL184407.1	6705	7107	6857, 6958, 6863	8, 11, 7	8.42257, 10.60895, 8.69489	5.97763, 6.9343, 6.06379	5.76336, 7	3	NA	NA	
GL184408.1	92	744	540, 532, 540	9, 16, 7	9.72082, 17.16856, 8.69489	6.64182, 9.82359, 6.06379	6.97155, 1	3	NA	NA	
GL184408.1	6107	7038	6139, 6645, 6617	8, 45, 19	4.88901, 41.25117, 20.55112	3.81552, 13.51972, 11.04971	2.57705, 3	3	NA	NA	
GL184408.1	6135	7038	6613, 6645, 6617	26, 45, 19	25.01044, 41.25117, 20.55112	11.44656, 13.51972, 11.04971	21.62959,	3	NA	NA	
GL184408.1	7549	8158	7682, 7818, 8073	13, 14, 10	9.62543, 7.38019, 8.92941	5.93525, 4.4086, 6.07734	6.8956, 4.9	3	NA	NA	
GL184420.1	1825	3049	2093, 2708, 2408	15, 14, 11	12.34004, 11.28333, 9.67839	7.148, 6.73619, 6.37065	9.44153, 8	3	NA	NA	
GL184420.1	4222	5187	4641, 4600, 4556	12, 14, 13	9.08501, 11.28333, 12.05005	5.80775, 6.73619, 7.43242	6.38792, 8	3	NA	NA	
GL184425.1	7475	8159	7739, 7771, 7612	9, 11, 6	9.72082, 10.60895, 7.29403	6.64182, 6.9343, 5.30581	6.97155, 7	3	NA	NA	
GL184459.1	765	1091	809, 800, 805	12, 18, 8	13.8483, 19.97713, 10.1465	8.63436, 10.97931, 6.82176	10.86694,	3	NA	NA	
GL184459.1	7300	8295	7683, 8074, 7209, 7702	19, 24, 5, 13	19.8867, 24.5441, 4.17451, 13.70995	10.65421, 11.86236, 3.60852, 8.41989	16.66031,	3	NA	NA	
GL184463.1	2117	2563	2329, 2410, 2444	6, 9, 3	5.96715, 8.21099, 3.47371	4.64927, 5.77858, 3.03189	3.52158, 5	3	NA	NA	
GL184471.1	46	553	250, 271, 360	9, 11, 11	9.72082, 10.60895, 14.75772	6.64182, 6.9343, 9.09568	6.97155, 7	3	NA	NA	
GL184472.1	5791	6303	5865, 5890, 6082	10, 18, 10	11.06011, 19.97713, 13.18184	7.306, 10.97931, 8.33771	8.22712, 1	3	NA	NA	
GL184476.1	3037	3822	3419, 3617, 3419	21, 20, 8	25.88765, 22.255, 10.0913	13.58701, 11.77279, 6.79758	22.48219,	3	NA	NA	
GL184484.1	7576	8317	7841, 8199, 7862	18, 31, 17	21.99239, 38.84431, 23.7686	12.12048, 17.76976, 13.15613	18.69793,	3	NA	NA	
GL184521.1	479	823	734, 747, 607	15, 27, 6	9.60485, 20.15327, 4.5515	5.49983, 8.39761, 3.80547	6.87658, 1	3	xtr-mir-427-3		0
GL184535.1	63	1298	584, 586, 385	11, 17, 10	12.43693, 18.11497, 12.71002	7.97018, 10.13939, 8.12734	9.52692, 1	3	NA	NA	
GL184535.1	5288	5809	5390, 5380, 5588	8, 6, 11	7.38183, 4.79966, 13.37278	5.38666, 3.96211, 8.45232	4.80856, 2	3	NA	NA	
GL184540.1	6390	7386	6913, 6930, 6740	23, 35, 19	27.48315, 44.13349, 24.11676	13.9341, 19.26198, 13.07033	24.03497,	3	NA	NA	
GL184542.1	5131	5703	5160, 5199, 5260	8, 13, 3	8.42257, 13.14514, 3.47371	5.97763, 8.09002, 3.03189	5.76336, 1	3	NA	NA	
GL184576.1	7158	8008	7901, 7541, 7480	6, 10, 3	5.96715, 9.39128, 3.47371	4.64927, 6.35644, 3.03189	3.52158, 6	3	NA	NA	
GL184586.1	2072	2349	2137, 2198, 2219	5, 6, 9	4.82177, 4.93312, 11.64359	3.98509, 4.04501, 7.57973	2.51418, 2	3	NA	NA	
GL184605.1	7336	7875	7503, 7505, 7596	8, 19, 11	8.42257, 21.4153, 14.75772	5.97763, 11.55717, 9.09568	5.76336, 1	3	NA	NA	
GL184610.1	6414	6849	6815, 6812, 6813	17, 16, 10	21.34551, 17.16856, 13.18184	11.95527, 9.82359, 8.33771	18.07159,	3	NA	NA	
GL184627.1	3086	3557	3420, 3246, 3415	10, 14, 8	6.26836, 7.58105, 5.33388	4.40637, 4.52182, 4.10413	3.80157, 5	3	NA	NA	
GL184627.1	4577	5059	4772, 4787, 4727	15, 16, 11	11.13, 9.29355, 8.23761	6.40927, 5.12473, 5.47217	8.29395, 6	3	NA	NA	
GL184635.1	128	965	762, 780, 713	87, 122, 51	56.84853, 79.13769, 34.2084	9.43728, 9.75938, 8.85145	52.90144,	3	NA	NA	
GL184635.1	1373	2364	2084, 2059, 2052	68, 85, 45	38.2883, 43.80758, 28.16442	7.39969, 6.82363, 7.83013	34.61412,	3	NA	NA	
GL184635.1	7571	8322	8088, 8115, 8105	18, 14, 22	22.92104, 14.45897, 28.44344	12.61945, 8.66788, 14.74701	19.59912,	3	NA	NA	
GL184639.1	101	453	274, 298, 282	6, 17, 9	5.96715, 18.56115, 11.64359	4.64927, 10.40145, 7.57973	3.52158, 1	3	NA	NA	
GL184666.1	62	1204	460, 418, 552, 1011	12, 17, 9, 9	10.20921, 15.0242, 9.64095, 9.64095	6.50784, 8.27335, 6.59867, 6.59867	7.4341, 12	3	NA	NA	
GL184667.1	236	1422	1124, 589, 828	12, 20, 10	13.8483, 22.87459, 13.18184	8.63436, 12.13503, 8.33771	10.86694,	3	NA	NA	
GL184683.1	6950	8049	7588, 7632, 7445	8, 10, 5	8.42257, 9.39128, 5.9506	5.97763, 6.35644, 4.54784	5.76336, 6	3	NA	NA	
GL184688.1	1465	1980	1521, 1487, 1785	7, 7, 5	7.1696, 5.97725, 5.9506	5.31345, 4.62287, 4.54784	4.61046, 3	3	NA	NA	
GL184714.1	548	973	917, 921, 873	14, 26, 7	16.7649, 32.02458, 8.69489	9.96273, 15.60218, 6.06379	13.6543, 2	3	NA	NA	
GL184718.1	7163	8233	7867, 7967, 7658	9, 17, 8	9.72082, 18.56115, 10.1465	6.64182, 10.40145, 6.82176	6.97155, 1	3	NA	NA	
GL184727.1	3208	3413	3370, 3385, 3384	6, 13, 9	5.96715, 13.14514, 11.64359	4.64927, 8.09002, 7.57973	3.52158, 1	3	NA	NA	
GL184743.1	127	676	462, 186, 403	7, 13, 7	4.94394, 10.04078, 6.18423	3.97064, 6.19235, 4.74828	2.62735, 7	3	NA	NA	
GL184763.1	710	1153	773, 814, 761	16, 10, 5	19.7934, 9.39128, 5.9506	11.29109, 6.35644, 4.54784	16.57067,	3	NA	NA	
GL184801.1	5299	6134	5574, 5692, 5565	22, 26, 19	14.50712, 15.37227, 14.50886	6.68143, 6.25869, 7.42489	11.49728,	3	NA	NA	
GL184823.1	6770	7701	7251, 6967, 7255	7, 7, 4	7.1696, 5.97725, 4.67325	5.31345, 4.62287, 3.78987	4.61046, 3	3	NA	NA	
GL184838.1	68	699	566, 543, 455	13, 9, 6	13.71465, 8.21099, 7.29403	8.42261, 5.77858, 5.30581	10.74495,	3	NA	NA	
GL184857.1	6746	7945	7567, 7567, 7567	14, 27, 8	16.7649, 33.60851, 10.1465	9.96273, 16.18004, 6.82176	13.6543, 3	3	NA	NA	
GL184891.1	13	850	272, 344, 335	17, 33, 15	17.80138, 37.21116, 17.58264	9.95359, 16.00342, 10.25879	14.65165,	3	NA	NA	
GL184891.1	6469	7077	6960, 6839, 6741	18, 33, 10	13.39411, 26.54054, 7.74178	7.02979, 9.99284, 5.33885	10.43859,	3	NA	NA	
GL184891.1	7293	7980	7875, 7686, 7690	18, 27, 14	13.39411, 19.83181, 12.16551	7.02979, 8.22939, 7.28026	10.43859,	3	NA	NA	
GL184906.1	1099	1726	1501, 1630, 1569	9, 13, 5	9.72082, 13.14514, 5.9506	6.64182, 8.09002, 4.54784	6.97155, 1	3	NA	NA	
GL184911.1	7051	7898	7125, 7277, 7831	5, 24, 8	4.82177, 23.77499, 9.60885	3.98509, 11.40147, 6.57807	2.51418, 2	3	NA	NA	
GL184913.1	75	744	182, 653, 170	12, 14, 7	13.8483, 12.86494, 8.69489	8.63436, 7.70954, 6.06379	10.86694,	3	NA	NA	
GL184913.1	7229	7914	7733, 7736, 7534	11, 13, 5	9.90875, 12.14594, 5.40789	6.51186, 7.49077, 4.29296	7.14919, 9	3	NA	NA	
GL184935.1	7739	8199	7904, 8104, 8087	11, 21, 9	12.43693, 24.35402, 11.64359	7.97018, 12.71289, 7.57973	9.52692, 2	3	NA	NA	
GL184947.1	6883	7427	7086, 7020, 6979	6, 12, 9	5.96715, 11.86108, 11.64359	4.64927, 7.51216, 7.57973	3.52158, 9	3	NA	NA	
GL184955.1	993	1278	1240, 1248, 1231	17, 19, 9	21.34551, 21.4153, 11.64359	11.95527, 11.55717, 7.57973	18.07159,	3	NA	NA	
GL184957.1	1300	1957	1583, 1581, 1470	10, 8, 3	11.06011, 7.07159, 3.47371	7.306, 5.20073, 3.03189	8.22712, 4	3	NA	NA	
GL184985.1	2616	3990	3696, 2962, 3889	39, 38, 20	28.06551, 22.5171, 14.08103	9.07942, 7.0064, 6.92527	24.60456,	3	NA	NA	
GL184989.1	433	991	470, 514, 460	20, 12, 10	26.13767, 11.86108, 13.18184	13.94781, 7.51216, 8.33771	22.72398,	3	NA	NA	
GL184998.1	6593	7815	7389, 7104, 7259	11, 31, 10	12.25244, 34.67556, 11.83521	7.87094, 15.30758, 7.70798	9.35958, 3	3	NA	NA	
GL185004.1	799	1054	1013, 1036, 1027	19, 18, 12	24.51879, 19.97713, 16.36823	13.28363, 10.97931, 9.85365	21.15031,	3	NA	NA	
GL185038.1	179	1318	412, 927, 388, 887	68, 321, 44, 165	12.76629, 184.67087, 8.78178, 100.35672	2.69666, 8.76264, 2.67116, 9.1312	9.84046, 1	3	NA	NA	
GL185038.1	179	1318	926, 927, 388, 887	282, 321, 44, 165	180.80927, 184.67087, 8.78178, 100.35672	10.2506, 8.76264, 2.67116, 9.1312	175.92249	3	NA	NA	
GL185038.1	3236	3847	3488, 3492, 3364	85, 120, 56	15.5125, 23.02007, 10.86981	2.69961, 2.85123, 2.70106	12.45558,	3	NA	NA	
GL185038.1	5556	6771	6328, 6329, 6336, 6678	66, 114, 64, 34	15.7142, 33.75848, 22.8005, 6.73793	3.15892, 3.93278, 4.40739, 2.56119	12.64994,	3	NA	NA	
GL185046.1	15	1088	776, 499, 754	15, 26, 21	18.26603, 30.50245, 26.35131	10.62691, 14.71478, 13.84438	15.09729,	3	NA	NA	
GL185058.1	5047	5923	5318, 5323, 5346	22, 34, 28	27.06102, 41.66864, 39.16069	13.98182, 18.22838, 18.95197	23.6241, 3	3	NA	NA	
GL185079.1	689	1033	974, 951, 949	71, 75, 47	95.97053, 98.54846, 67.38008	31.20683, 30.40737, 27.41144	91.61539,	3	NA	NA	
GL185082.1	7214	7708	7298, 7414, 7302	7, 9, 4	7.1696, 8.21099, 4.67325	5.31345, 5.77858, 3.78987	4.61046, 5	3	NA	NA	
GL185096.1	65	810	448, 473, 438	78, 133, 57	85.10056, 148.57401, 67.13444	21.85717, 26.50747, 22.2625	80.85211,	3	NA	NA	
GL185096.1	6941	8297	7236, 7292, 7288	243, 362, 153	265.79053, 383.15585, 173.38187	28.1953, 27.32445, 28.19284	260.58054	3	NA	NA	
GL185097.1	1197	1903	1443, 1358, 1365, 1760	6, 9, 5, 5	5.96715, 8.21099, 5.9506, 5.9506	4.64927, 5.77858, 4.54784, 4.54784	3.52158, 5	3	NA	NA	
GL185107.1	1163	1689	1367, 1341, 1579	8, 17, 10	8.42257, 18.56115, 13.18184	5.97763, 10.40145, 8.33771	5.76336, 1	3	NA	NA	
GL185115.1	7594	7857	7764, 7723, 7733	10, 16, 9	11.06011, 17.16856, 11.64359	7.306, 9.82359, 7.57973	8.22712, 1	3	NA	NA	
GL185146.1	334	1201	923, 941, 935	19, 20, 9	20.93125, 19.50661, 10.28145	11.274, 10.12815, 6.93568	17.67176,	3	NA	NA	
GL185146.1	6766	7161	6957, 6905, 6853	12, 12, 5	13.8483, 11.86108, 5.9506	8.63436, 7.51216, 4.54784	10.86694,	3	NA	NA	
GL185149.1	4998	6763	5820, 6133, 5778	20, 19, 16	19.28838, 19.41322, 16.83466	9.99624, 10.37021, 9.62725	16.08332,	3	NA		1280
GL185157.1	762	1726	1142, 1320, 999	6, 8, 4	5.96715, 7.07159, 4.67325	4.64927, 5.20073, 3.78987	3.52158, 4	3	NA	NA	
GL185176.1	1339	1886	1798, 1763, 1793	14, 43, 35	16.7649, 56.06343, 50.8823	9.96273, 22.71412, 23.08228	13.6543, 5	3	NA	NA	
GL185198.1	956	1368	1243, 1257, 1204	7, 15, 4	7.1696, 15.80067, 4.67325	5.31345, 9.24574, 3.78987	4.61046, 1	3	NA	NA	
GL185235.1	393	2122	660, 1780, 1648	10, 8, 9	11.06011, 7.07159, 11.64359	7.306, 5.20073, 7.57973	8.22712, 4	3	NA	NA	
GL185235.1	393	2122	1680, 1780, 1648	5, 8, 9	4.82177, 7.07159, 11.64359	3.98509, 5.20073, 7.57973	2.51418, 4	3	NA	NA	
GL185235.1	7342	8351	7749, 7563, 8290	12, 10, 4	13.8483, 9.39128, 4.67325	8.63436, 6.35644, 3.78987	10.86694,	3	NA	NA	
GL185269.1	58	2973	693, 286, 2902	19, 30, 12	13.70089, 21.51985, 9.47337	6.95705, 8.30056, 6.05008	10.73164,	3	NA	NA	
GL185269.1	187	3412	1690, 1991, 2902	17, 20, 12	11.66257, 11.6388, 9.47337	6.26134, 5.61016, 6.05008	8.79957, 8	3	NA	NA	
GL185269.1	187	3412	2890, 1991, 2902	17, 20, 12	11.66257, 11.6388, 9.47337	6.26134, 5.61016, 6.05008	8.79957, 8	3	NA	NA	
GL185283.1	6791	7161	6910, 6964, 6985	12, 22, 19	13.8483, 25.85271, 28.30916	8.63436, 13.29074, 15.10573	10.86694,	3	NA	NA	
GL185286.1	91	958	736, 660, 881	22, 25, 11	18.79573, 21.3001, 9.7601	9.08809, 9.6159, 6.42085	15.60788,	3	NA	NA	
GL185286.1	2172	3169	2218, 2267, 2210	24, 32, 21	21.20631, 30.01908, 22.75397	9.87836, 12.20479, 11.77155	17.93797,	3	NA	NA	
GL185330.1	6882	7775	7516, 7360, 7347	19, 31, 19	23.04311, 37.73788, 23.65931	12.48444, 17.12176, 12.82349	19.71723,	3	NA	NA	
GL185336.1	94	987	471, 465, 755	15, 26, 15	14.21298, 24.56489, 16.44983	8.29535, 11.18383, 9.62273	11.21566,	3	NA	NA	
GL185336.1	6987	7729	7440, 7430, 7600	15, 20, 7	16.90423, 21.69995, 8.646	9.88146, 11.44483, 6.04229	13.78827,	3	NA	NA	
GL185360.1	66	1294	1049, 945, 1040	18, 33, 13	21.29566, 38.10561, 17.65969	11.73423, 16.53382, 10.45768	18.02512,	3	NA	NA	
GL185370.1	6228	7137	6375, 6363, 6554	7, 13, 4	7.1696, 13.14514, 4.67325	5.31345, 8.09002, 3.78987	4.61046, 1	3	NA	NA	
GL185380.1	29	747	344, 544, 148	7, 8, 5	5.4599, 5.93025, 5.06966	4.30109, 4.48816, 4.11933	3.09813, 3	3	NA	NA	
GL185380.1	951	1441	1394, 1219, 1273	14, 17, 8	7.63205, 11.92627, 6.1856	4.55073, 6.41462, 4.65072	5.04403, 9	3	NA	NA	
GL185380.1	6140	7803	7058, 7118, 7114	268, 362, 147	262.58667, 338.69174, 146.80704	22.33794, 20.771, 21.43171	257.38153	3	NA	NA	
GL185394.1	7059	7714	7298, 7339, 7345	23, 24, 16	29.33336, 28.90463, 22.37427	14.98133, 14.44646, 12.56044	25.84217,	3	NA	NA	
GL185407.1	6787	7321	6940, 7057, 7053	6, 15, 8	5.96715, 15.80067, 10.1465	4.64927, 9.24574, 6.82176	3.52158, 1	3	NA	NA	
GL185418.1	6052	6527	6357, 6357, 6354	71, 91, 64	95.97053, 122.32743, 91.30348	31.20683, 34.45978, 32.47633	91.61539,	3	NA	NA	
GL185447.1	413	2108	1807, 1476, 1820	13, 20, 13	13.79066, 20.53147, 15.52427	8.46641, 10.74631, 9.42125	10.8171, 1	3	NA	NA	
GL185447.1	6957	7683	7108, 7416, 7111	15, 16, 7	18.26603, 17.16856, 8.69489	10.62691, 9.82359, 6.06379	15.09729,	3	NA	NA	
GL185455.1	6355	6734	6493, 6473, 6512	7, 13, 6	7.1696, 13.14514, 7.29403	5.31345, 8.09002, 5.30581	4.61046, 1	3	NA	NA	
GL185481.1	144	885	627, 664, 782	20, 23, 10	22.36548, 23.5013, 12.90749	11.8377, 11.57503, 8.21675	19.06081,	3	NA	NA	
GL185481.1	6465	7620	6719, 7310, 6574, 7573	16, 23, 8, 5	17.77392, 21.38155, 9.31951, 5.40789	10.17481, 10.30482, 6.43944, 4.29296	14.62581,	3	NA	NA	
GL185481.1	6465	7620	7297, 7310, 6574, 7573	11, 23, 8, 5	11.02968, 21.38155, 9.31951, 5.40789	7.18222, 10.30482, 6.43944, 4.29296	8.20839, 1	3	NA	NA	
GL185483.1	6672	7754	7189, 7115, 6957	13, 19, 9	15.29169, 21.4153, 11.64359	9.29854, 11.55717, 7.57973	12.24396,	3	NA	NA	
GL185486.1	257	1007	572, 907, 760	10, 14, 16	11.06011, 14.45897, 23.11042	7.306, 8.66788, 12.88555	8.22712, 1	3	NA	NA	
GL185518.1	28	591	295, 282, 245	47, 73, 27	47.20149, 74.04084, 27.98385	15.84378, 18.74523, 12.85473	43.38986,	3	NA	NA	
GL185518.1	7261	7786	7524, 7493, 7444	6, 9, 5	5.55471, 8.21099, 5.9506	4.41698, 5.77858, 4.54784	3.18192, 5	3	NA	NA	
GL185523.1	17	490	208, 182, 190	13, 21, 11	15.29169, 22.39999, 14.75772	9.29854, 11.56043, 9.09568	12.24396,	3	NA	NA	
GL185523.1	6362	7210	6923, 6904, 6818	21, 28, 24	20.73342, 25.55993, 26.06419	10.5587, 11.07435, 12.77285	17.48034,	3	NA	NA	
GL185531.1	6607	7694	7117, 7113, 7045	15, 19, 9	18.26603, 21.4153, 11.64359	10.62691, 11.55717, 7.57973	15.09729,	3	NA	NA	
GL185532.1	107	3365	1770, 2425, 1903	33, 40, 22	29.11182, 31.62041, 18.61246	11.35157, 10.43089, 8.98053	25.62591,	3	U4		0
GL185542.1	25	752	299, 367, 204	9, 14, 12	9.72082, 14.45897, 15.80834	6.64182, 8.66788, 9.60504	6.97155, 1	3	NA	NA	
GL185568.1	112	917	587, 571, 384	17, 30, 8	14.39438, 28.17911, 7.76694	7.88994, 11.87674, 5.6116	11.3889, 2	3	NA	NA	
GL185568.1	4798	5799	4857, 4855, 4910, 5488	21, 14, 15, 8	14.22324, 5.77302, 11.16794, 4.82597	6.76262, 3.53997, 6.43227, 3.77458	11.22581,	3	NA	NA	
GL185568.1	6512	6739	6637, 6568, 6565	14, 25, 9	8.78276, 17.63901, 5.9866	5.21779, 7.6035, 4.38351	6.10293, 1	3	NA	NA	
GL185568.1	7186	7610	7439, 7336, 7498	12, 23, 9	6.99835, 15.59014, 5.9866	4.52208, 7.01862, 4.38351	4.47545, 1	3	NA	NA	
GL185578.1	912	1313	967, 997, 975	6, 18, 9	5.96715, 19.97713, 11.64359	4.64927, 10.97931, 7.57973	3.52158, 1	3	NA	NA	
GL185586.1	6429	7003	6694, 6667, 6592	11, 8, 6	12.43693, 7.07159, 7.29403	7.97018, 5.20073, 5.30581	9.52692, 4	3	NA	NA	
GL185589.1	7147	7731	7298, 7275, 7230	8, 13, 7	8.42257, 13.14514, 8.69489	5.97763, 8.09002, 6.06379	5.76336, 1	3	NA	NA	
GL185595.1	6499	6938	6709, 6709, 6709	63, 68, 39	73.18973, 76.02809, 47.10915	22.66945, 21.80854, 19.27623	69.06242,	3	5S_rRNA		0
GL185607.1	140	619	260, 479, 342	6, 10, 8	5.96715, 9.39128, 10.1465	4.64927, 6.35644, 6.82176	3.52158, 6	3	NA	NA	
GL185607.1	6558	7530	7156, 7345, 6969	7, 9, 5	7.1696, 8.21099, 5.9506	5.31345, 5.77858, 4.54784	4.61046, 5	3	NA	NA	
GL185622.1	327	633	609, 627, 578	10, 11, 7	11.06011, 10.60895, 8.69489	7.306, 6.9343, 6.06379	8.22712, 7	3	NA	NA	
GL185627.1	2669	2870	2838, 2676, 2672	6, 7, 4	5.96715, 5.97725, 4.67325	4.64927, 4.62287, 3.78987	3.52158, 3	3	NA	NA	
GL185640.1	1349	1629	1501, 1452, 1544	10, 17, 8	9.71769, 16.56523, 9.05167	6.54998, 9.21112, 6.30653	6.97155, 1	3	NA	NA	
GL185647.1	6728	7568	6922, 7375, 7037	13, 11, 10	15.29169, 10.60895, 13.18184	9.29854, 6.9343, 8.33771	12.24396,	3	NA	NA	
GL185648.1	555	952	916, 845, 854	10, 16, 8	11.06011, 17.16856, 10.1465	7.306, 9.82359, 6.82176	8.22712, 1	3	NA	NA	
GL185657.1	1472	1912	1621, 1596, 1773	11, 12, 11	10.66406, 10.35, 11.6121	6.96705, 6.59497, 7.51665	7.86396, 7	3	NA	NA	
GL185662.1	72	1337	323, 303, 427	71, 112, 51	42.37609, 70.62849, 34.39523	8.048, 9.2666, 8.91852	38.63524,	3	NA	NA	
GL185662.1	2781	3344	3082, 3204, 3251	62, 72, 43	23.54938, 23.46229, 18.9527	4.67114, 4.11293, 5.13428	20.20923,	3	NA	NA	
GL185662.1	6466	7632	6870, 6734, 7495	49, 61, 36	24.1345, 30.14238, 20.39434	5.94309, 6.1917, 6.54423	20.77719,	3	NA	NA	
GL185675.1	39	1376	655, 928, 343	16, 22, 10	17.95866, 23.10829, 13.18184	10.28064, 11.66803, 8.33771	14.80292,	3	NA	NA	
GL185681.1	15	1418	1266, 1254, 1265	119, 124, 125	120.42887, 124.13776, 133.76936	21.13288, 20.86011, 23.90473	115.87605	3	NA	NA	
GL185687.1	39	881	650, 457, 495	71, 75, 51	72.38569, 75.07643, 56.92032	18.7599, 18.45531, 19.41084	68.26663,	3	NA	NA	
GL185695.1	94	1486	842, 845, 667	24, 24, 15	31.57662, 27.93659, 19.64455	15.948, 13.88262, 11.32852	28.03371,	3	NA	NA	
GL185707.1	100	688	270, 434, 411	7, 10, 4	7.1696, 9.39128, 4.67325	5.31345, 6.35644, 3.78987	4.61046, 6	3	NA	NA	
GL185740.1	7057	7615	7111, 7105, 7122	74, 83, 43	101.6535, 114.05475, 61.74873	32.75127, 34.55028, 26.0019	97.24903,	3	NA	NA	
GL185767.1	50	756	500, 568, 614	12, 10, 11	13.8483, 9.39128, 14.68355	8.63436, 6.35644, 9.06344	10.86694,	3	NA	NA	
GL185767.1	6556	7071	6716, 6805, 6801	13, 16, 7	15.29169, 17.16856, 8.69489	9.29854, 9.82359, 6.06379	12.24396,	3	NA	NA	
GL185782.1	753	1040	991, 866, 906	15, 23, 13	18.26603, 27.36982, 18.01082	10.62691, 13.8686, 10.61163	15.09729,	3	NA	NA	
GL185790.1	537	1004	960, 972, 927	17, 16, 11	21.34551, 17.16856, 14.75772	11.95527, 9.82359, 9.09568	18.07159,	3	NA	NA	
GL185798.1	6399	6851	6459, 6459, 6459	13, 16, 10	15.29169, 17.16856, 13.18184	9.29854, 9.82359, 8.33771	12.24396,	3	NA	NA	
GL185814.1	467	984	921, 939, 955	19, 27, 12	24.51879, 33.60851, 16.36823	13.28363, 16.18004, 9.85365	21.15031,	3	NA	NA	
GL185847.1	336	1361	981, 896, 1080	9, 12, 6	6.82879, 7.51974, 5.46454	4.92128, 4.83844, 4.36458	4.31954, 5	3	NA	NA	
GL185847.1	6830	7770	7153, 7124, 7289	30, 26, 21	31.55717, 23.24287, 24.4673	13.84925, 10.41222, 12.77852	28.01499,	3	NA	NA	
GL185864.1	780	1455	806, 811, 806	28, 39, 26	38.31764, 51.0564, 37.42201	18.49968, 21.59911, 18.53699	34.6431, 4	3	NA	NA	
GL185876.1	6094	7459	6882, 6911, 6329	20, 30, 17	24.33651, 32.23063, 19.99824	12.96942, 14.24854, 11.2232	20.9737, 2	3	NA	NA	
GL185888.1	6396	6789	6594, 6591, 6490	14, 11, 10	16.7649, 10.60895, 13.18184	9.96273, 6.9343, 8.33771	13.6543, 7	3	NA	NA	
GL185891.1	7103	7508	7287, 7399, 7316	10, 15, 10	11.06011, 15.80067, 13.18184	7.306, 9.24574, 8.33771	8.22712, 1	3	NA	NA	
GL185893.1	6252	7417	6883, 7181, 6885	7, 7, 12	7.1696, 5.97725, 16.36823	5.31345, 4.62287, 9.85365	4.61046, 3	3	NA	NA	
GL185916.1	688	1057	894, 850, 896	6, 10, 5	5.96715, 9.39128, 5.9506	4.64927, 6.35644, 4.54784	3.52158, 6	3	NA	NA	
GL185916.1	6814	7518	7335, 7335, 7248	37, 46, 28	45.18034, 54.42801, 32.87535	19.02292, 20.32899, 15.39573	41.39887,	3	NA	NA	
GL185922.1	102	720	299, 580, 316	13, 13, 8	15.29169, 13.14514, 10.1465	9.29854, 8.09002, 6.82176	12.24396,	3	NA	NA	
GL185923.1	7224	7854	7578, 7546, 7548	182, 191, 170	198.81514, 209.65509, 195.54851	26.89221, 27.40471, 29.77954	193.8317,	3	NA	NA	
GL185927.1	48	549	192, 382, 445	8, 7, 9	8.10827, 5.8946, 8.88966	5.80497, 4.57222, 6.17838	5.48761, 3	3	NA	NA	
GL185927.1	808	1135	889, 936, 956	7, 18, 17	6.89193, 19.7713, 19.81591	5.15998, 10.85902, 11.12108	4.37772, 1	3	NA	NA	
GL185927.1	6446	7545	7191, 7225, 7252	16, 33, 10	14.62621, 34.83062, 11.10454	8.29596, 14.59849, 7.32977	11.61068,	3	NA	NA	
GL185941.1	180	1221	1002, 1097, 162, 1002	8, 14, 4, 7	8.34715, 14.45897, 4.64325, 8.646	5.9367, 8.66788, 3.77643, 6.04229	5.71011, 1	3	NA	NA	
GL185941.1	6677	7561	7337, 7410, 6751, 7415	12, 20, 9, 5	13.22036, 21.88095, 10.72164, 5.40789	8.29296, 11.5521, 7.15493, 4.29296	10.27237,	3	NA	NA	
GL185956.1	1624	2158	1746, 1911, 1704	8, 5, 4	8.42257, 3.94561, 4.67325	5.97763, 3.46715, 3.78987	5.76336, 1	3	NA	NA	
GL185965.1	4023	5073	4877, 4523, 4702	11, 10, 6	12.43693, 9.39128, 7.29403	7.97018, 6.35644, 5.30581	9.52692, 6	3	NA	NA	
GL185982.1	56	485	214, 256, 228	9, 10, 8	9.72082, 9.39128, 10.1465	6.64182, 6.35644, 6.82176	6.97155, 6	3	NA	NA	
GL185985.1	6671	7245	7186, 7054, 7212	14, 12, 7	16.7649, 11.86108, 8.69489	9.96273, 7.51216, 6.06379	13.6543, 9	3	NA	NA	
GL185987.1	356	1137	453, 1029, 863	5, 14, 6	4.82177, 14.45897, 7.29403	3.98509, 8.66788, 5.30581	2.51418, 1	3	NA	NA	
GL185987.1	479	1137	962, 1029, 863	7, 14, 6	7.1696, 14.45897, 7.29403	5.31345, 8.66788, 5.30581	4.61046, 1	3	NA	NA	
GL185988.1	144	826	263, 554, 374	6, 10, 5	5.96715, 9.39128, 5.9506	4.64927, 6.35644, 4.54784	3.52158, 6	3	NA	NA	
GL185988.1	6506	7493	7340, 7278, 6769	17, 17, 8	19.39948, 17.11659, 10.0913	10.88538, 9.54398, 6.79758	16.19131,	3	NA	NA	
GL185991.1	1530	2502	2280, 2009, 2093	15, 23, 9	18.12902, 26.02147, 11.64359	10.55412, 13.07981, 7.57973	14.96737,	3	NA	NA	
GL185992.1	413	852	447, 480, 450	47, 56, 34	64.80984, 77.32239, 49.07372	25.92644, 28.66862, 22.4411	60.76838,	3	NA	NA	
GL186002.1	675	896	847, 843, 849	31, 35, 23	42.34241, 46.74359, 33.95797	19.76292, 20.77732, 17.3547	38.6021, 4	3	NA	NA	
GL186004.1	939	1563	1151, 1236, 1095	13, 14, 4	12.81131, 13.26509, 4.67325	7.8918, 7.95332, 3.78987	9.88323, 1	3	NA	NA	
GL186004.1	7150	7584	7475, 7501, 7496	51, 66, 42	63.60565, 80.35816, 56.08688	23.231, 25.09571, 23.19078	59.57824,	3	NA	NA	
GL186025.1	37	1091	474, 678, 434	16, 19, 12	18.5447, 17.95671, 13.90113	10.61177, 9.48989, 8.66246	15.36536,	3	NA	NA	
GL186030.1	2515	3442	3027, 3011, 3034	10, 18, 6	11.06011, 19.97713, 7.29403	7.306, 10.97931, 5.30581	8.22712, 1	3	NA	NA	
GL186031.1	6679	7414	6950, 7134, 7133	17, 25, 16	20.94161, 27.53425, 22.68246	11.74019, 13.30495, 12.69862	17.68164,	3	NA	NA	
GL186035.1	667	1470	1158, 1153, 1099	35, 62, 45	33.37763, 60.21692, 54.48461	12.98897, 16.52952, 20.83673	29.79667,	3	NA	NA	
GL186040.1	619	1148	647, 650, 645	16, 29, 13	19.7934, 36.82147, 18.01082	11.29109, 17.33575, 10.61163	16.57067,	3	NA	NA	
GL186046.1	172	1346	879, 702, 644	12, 14, 6	13.8483, 14.45897, 7.29403	8.63436, 8.66788, 5.30581	10.86694,	3	NA	NA	
GL186055.1	60	973	173, 825, 768	13, 27, 8	11.16633, 25.72383, 9.31951	6.8898, 11.51676, 6.43944	8.32764, 2	3	NA	NA	
GL186055.1	92	1015	919, 825, 768	17, 27, 8	13.35329, 25.72383, 9.31951	7.26045, 11.51676, 6.43944	10.39951,	3	NA	NA	
GL186055.1	5664	6758	6313, 6365, 6365	24, 48, 22	18.96559, 35.90485, 18.7905	8.59348, 10.17307, 9.08501	15.7715, 3	3	NA	NA	
GL186073.1	46	1079	555, 415, 831	8, 8, 9	6.78332, 6.66413, 9.41119	5.02459, 4.9509, 6.47287	4.27811, 4	3	NA	NA	
GL186073.1	269	1388	924, 985, 831	14, 12, 9	13.96392, 11.25867, 9.41119	8.37431, 7.1513, 6.47287	10.9769, 8	3	NA	NA	
GL186073.1	6853	7433	7263, 7301, 7253	20, 20, 9	23.85765, 22.06591, 11.64359	12.69961, 11.6614, 7.57973	20.50816,	3	NA	NA	
GL186077.1	1661	2123	1757, 1919, 1905	10, 8, 8	11.06011, 7.07159, 10.1465	7.306, 5.20073, 6.82176	8.22712, 4	3	NA	NA	

GL186077.1	6631	7571	7056, 7086, 7258	21, 23, 15	24.5685, 26.12482, 19.40672	12.83702, 13.14082, 11.21161	21.19879,	3	NA	NA
GL186080.1	5876	7413	6667, 6834, 6499	38, 50, 18	41.48602, 51.39797, 20.07449	16.41423, 16.84509, 11.036	37.75999,	3	NA	NA
GL186103.1	83	839	472, 193, 83, 514	7, 12, 6, 9	7.1696, 11.86108, 7.12188, 11.39481	5.31345, 7.51216, 5.22884, 7.46977	4.61046, 9	3	NA	NA
GL186103.1	6192	7369	6406, 6820, 6848	9, 13, 7	8.45482, 11.41208, 6.92462	5.92419, 7.04141, 5.17829	5.79307, 8	3	NA	NA
GL186103.1	6192	7384	7156, 6820, 6848	14, 13, 7	14.83249, 11.41208, 6.92462	8.88629, 7.04141, 5.17829	11.80835,	3	NA	NA
GL186114.1	308	862	338, 336, 338	14, 17, 16	16.7649, 18.56115, 23.11042	9.96273, 10.40145, 12.88555	13.6543, 1	3	NA	NA
GL186132.1	7116	7424	7215, 7277, 7267	7, 17, 6	7.1696, 18.56115, 7.29403	5.31345, 10.40145, 5.30581	4.61046, 1	3	NA	NA
GL186133.1	199	1141	879, 522, 578	14, 23, 9	16.7649, 27.36982, 11.64359	9.96273, 13.8686, 7.57973	13.6543, 2	3	NA	NA
GL186148.1	6065	7217	6625, 6408, 6162	18, 18, 7	22.49411, 19.08138, 8.69489	12.39242, 10.4519, 6.06379	19.18565,	3	NA	NA
GL186172.1	6424	7213	6756, 6693, 6862	9, 6, 4	9.72082, 4.93312, 4.67325	6.64182, 4.04501, 3.78987	6.97155, 2	3	NA	NA
GL186209.1	42	898	447, 447, 417	90, 135, 61	105.08496, 158.26389, 75.18291	26.04483, 29.48717, 24.77832	100.65384	3	NA	NA
GL186209.1	6852	7382	7119, 7122, 7105	87, 116, 42	98.66238, 137.52782, 52.91249	24.34723, 29.01164, 21.3274	94.28367,	3	NA	NA
GL186241.1	6366	7182	6446, 6422, 6963	10, 14, 12	11.06011, 14.45897, 16.36823	7.306, 8.66788, 9.85365	8.22712, 1	3	NA	NA
GL186254.1	25	770	273, 269, 277	21, 28, 19	25.88765, 34.88946, 26.56077	13.58701, 16.5743, 14.30986	22.48219,	3	NA	NA
GL186256.1	2279	2560	2450, 2363, 2380	4, 6, 5	3.74195, 4.32803, 5.9506	3.32091, 3.66192, 4.54784	1.6222, 2.2	3	NA	NA
GL186290.1	228	962	532, 302, 797	6, 5, 4	5.96715, 3.94561, 4.67325	4.64927, 3.46715, 3.78987	3.52158, 1	3	NA	NA
GL186295.1	6617	7312	7097, 7028, 7055	7, 7, 7	7.1696, 5.97725, 8.69489	5.31345, 4.62287, 6.06379	4.61046, 3	3	NA	NA
GL186315.1	676	1333	1276, 1306, 1287	6, 14, 6	5.96715, 14.45897, 7.29403	4.64927, 8.66788, 5.30581	3.52158, 1	3	NA	NA
GL186337.1	292	1078	473, 333, 794	6, 7, 4	5.96715, 5.97725, 4.67325	4.64927, 4.62287, 3.78987	3.52158, 3	3	NA	5083
GL186344.1	56	1300	726, 569, 678	11, 21, 10	12.43693, 24.35402, 13.18184	7.97018, 12.71289, 8.33771	9.52692, 2	3	NA	NA
GL186420.1	91	432	232, 234, 218	11, 12, 7	9.26086, 9.20568, 7.40077	6.1125, 5.88305, 5.43746	6.5517, 6.6	3	NA	NA
GL186420.1	658	1752	861, 898, 885, 1528	21, 15, 7, 7	21.80886, 12.44573, 7.40077, 7.40077	11.20625, 7.21287, 5.43746, 5.43746	18.52047,	3	NA	NA
GL186432.1	3743	5370	4545, 4559, 4283	13, 19, 12	13.79066, 18.48026, 14.58408	8.46641, 9.80703, 9.01638	10.8171, 1	3	NA	NA
GL186447.1	67	768	453, 494, 439	10, 17, 12	10.26482, 17.71433, 13.44151	6.86644, 9.9018, 8.41472	7.48624, 1	3	NA	NA
GL186447.1	6574	7274	7016, 6961, 7026	19, 26, 13	23.53998, 30.97684, 17.65969	12.7584, 14.99323, 10.45768	20.2, 27.63	3	NA	NA
GL186467.1	46	1130	432, 354, 250	80, 108, 54	95.03339, 119.81728, 65.6787	25.85563, 24.82534, 22.9253	90.68629,	3	NA	NA
GL186467.1	5890	7288	7069, 7092, 7091	39, 45, 33	42.1314, 46.69796, 38.45537	16.36135, 16.39293, 16.74124	38.39518,	3	NA	NA
GL186478.1	6034	7287	6327, 6813, 6148, 6893	11, 20, 13, 6	9.90875, 20.68875, 15.17843, 5.91225	6.51186, 10.84083, 9.23814, 4.61907	7.14919, 1	3	NA	NA
GL186478.1	6088	7287	7146, 6813, 6148, 6893	10, 20, 13, 6	8.75281, 20.68875, 15.17843, 5.91225	5.9692, 10.84083, 9.23814, 4.61907	6.07523, 1	3	NA	NA
GL186498.1	20	1035	300, 475, 252	25, 34, 19	22.31838, 30.00535, 17.24619	10.20414, 11.49566, 9.05945	19.01523,	3	NA	NA
GL186498.1	6423	7294	6489, 6826, 6869	16, 25, 11	15.47689, 23.41295, 12.9493	8.81381, 10.84616, 8.23866	12.4218, 2	3	NA	NA
GL186504.1	6713	7241	6773, 7046, 7015	11, 10, 9	12.43693, 9.39128, 11.64359	7.97018, 6.35644, 7.57973	9.52692, 6	3	NA	NA
GL186521.1	6899	7240	6933, 6954, 6954	13, 14, 15	15.29169, 14.45897, 21.38372	9.29854, 8.66788, 12.12757	12.24396,	3	NA	NA
GL186538.1	136	1287	887, 892, 1045	35, 41, 25	38.23525, 41.5477, 27.66765	15.77987, 15.19816, 13.38448	34.56211,	3	NA	NA
GL186541.1	6613	7271	6677, 6683, 6686	7, 9, 7	7.1696, 8.21099, 8.69489	5.31345, 5.77858, 6.06379	4.61046, 5	3	NA	NA
GL186545.1	176	1106	737, 946, 838	5, 7, 5	4.82177, 5.97725, 5.9506	3.98509, 4.62287, 4.54784	2.51418, 3	3	NA	NA
GL186548.1	1022	1718	1594, 1650, 1650	93, 138, 51	101.01289, 152.87027, 54.56311	22.85342, 26.25754, 18.11613	96.61317,	3	NA	NA
GL186571.1	110	1129	295, 896, 482, 963	15, 13, 5, 10	17.90819, 12.99541, 5.23257, 11.83521	10.43573, 8.00139, 4.20435, 7.70798	14.75478,	3	NA	NA
GL186571.1	110	1129	743, 896, 482, 963	6, 13, 5, 10	5.81608, 12.99541, 5.23257, 11.83521	4.56563, 8.00139, 4.20435, 7.70798	3.4176, 10	3	NA	NA
GL186572.1	6241	7150	6588, 6481, 6496	37, 49, 28	40.43646, 53.42782, 32.47473	16.22831, 18.37631, 15.15869	36.72728,	3	NA	NA
[truncated: 5,563,755 more chars]
